# Probabilistic Assessment of Glass Forming Ability Rules for Metallic Glasses Aided by Automated Analysis of Phase Diagrams

**DOI:** 10.1038/s41598-018-36224-3

**Published:** 2019-01-23

**Authors:** Aparajita Dasgupta, Scott R. Broderick, Connor Mack, Bhargava U. Kota, Ramachandran Subramanian, Srirangaraj Setlur, Venu Govindaraju, Krishna Rajan

**Affiliations:** 1 Department of Materials Design and Innovation, University at Buffalo, New York, USA; 2 Department of Computer Science and Engineering, University at Buffalo, New York, USA

## Abstract

The use of machine learning techniques to expedite the discovery and development of new materials is an essential step towards the acceleration of a new generation of domain-specific highly functional material systems. In this paper, we use the test case of bulk metallic glasses to highlight the key issues in the field of high throughput predictions and propose a new probabilistic analysis of rules for glass forming ability using rough set theory. This approach has been applied to a broad range of binary alloy compositions in order to predict new metallic glass compositions. Our data driven approach takes into account not only a broad variety of thermodynamic, structural and kinetic based criteria, but also incorporates qualitative and descriptive attributes associated with eutectic points in phase diagrams. For the latter, we demonstrate the use of automated machine learning methods that go far beyond text recognition approaches by also being able to interpret phase diagrams. When combined with structural descriptors, this approach provides the foundations to develop a hierarchical probabilistic predication tool that can rank the feasibility of glass formation.

## Introduction

While data driven computational materials design has had far reaching consequences including important breakthroughs in crystal structure, atomic energy predictions, and approximations of density functionals, the potential of using machine learning approaches to accelerate the materials discovery process has yet to reach desirable levels^1^. A chief reason for this is the absence of key informational parameters that significantly explain the formation of specialized materials and the resulting gap in machine learning algorithms that can efficiently capture these informational parameters from the many sources of literature and physical models available today.

A key source of information within the materials science domain has been various pictorial and graphical representations of data, including phase diagrams, CV curves, and micrographs. While many advancements have been made in text recognition, limited progress has been made in handling these types of representations beyond the capability of handling line plots. In the current study, we use the example of metallic glasses to address the above mentioned issues.

A long standing problem in identifying potential binary alloy chemistries that can be good candidates for metallic glass formation has been the challenge of finding a unified thermochemical, structural and kinetic criteria for glass formability^2,3^. A first order criteria in bulk metallic glasses that is based on inspecting phase diagrams has been the ‘deep eutectic’^4,5^, a parameter which has not been fully defined in a quantitative manner. Logically, a deep eutectic indicates a narrow solid region within the phase diagram bounded by liquidus regions, thus indicating a propensity of the region towards amorphous structure^6,7^.

A large amount of the work and progress in the field has been chiefly related to thermochemical parameters. These have primarily constituted three main characteristics: glass transition temperature (T_g_), crystallization temperature (T_x_) and liquidus temperature (T_L_). These parameters have been shown to have correlation with glass forming ability (GFA), particularly when accompanied with kinetic information in the form of critical cooling rate R_c_^4,8–14^. The reduced glass transition temperature ${T}_{rg}={T}_{g}/{T}_{l}$ measures directly the depth of the eutectic point^4^. Similarly, the width of the supercooled liquid (${\rm{\Delta }}{T}_{x}={T}_{x}-{T}_{g})$ has been shown to correlate well with the glass forming ability of the alloy^15^. Cao *et al*. for instance, showed recently that the glass transition temperature, T_g_ of a variety of metallic glasses has a close relationship with the eutectic and peritectic points within binary phase diagrams^16^.

In addition to these thermodynamic metrics, certain empirical rules have been proposed for glass formability. Chief among these are the rules defined by Inoue^8^. These rules dictate a multi-component system, a large negative heat of mixing, and a large radii difference between the constituent elements. This builds on other existing rules involving enthalpy of mixing, melting temperature, bulk moduli, and Pauling electronegativity^17,18^. Therefore, this problem clearly spans multiple data types and classes of problem, necessitating a machine driven approach to address this design challenge and accelerate discovery.

Ren *et al*. have recently demonstrated the applications of machine learning and high throughput experimentation to propose new metallic glass chemistries in specific ternary systems (Co-V-Zr, Co-Ti-Zr, Co-Fe-Zr and Fe-Ti-Nb)^19^. A previous model by Ward *et al*.^20^ was used as the starting machine learning model and validations and subsequent iterations were made using high throughput experiments, thus illustrating the computational predictive power of an iterative machine learning/experimental approach. One missing component of their work is the integration of thermodynamic description, which is known to relate with metallic glass formation and which is a focus of this current paper. In another recent study, Perim *et al*. performed an extensive computational study on potential glass forming systems^21^. From their work, which largely utilized formation enthalpies and additional metrics related to similarity to predict glass formation, they concluded that the number of systems which may be glass formers is far larger than previously thought, with more than 17% of binary alloys serving as potential glass formers. This motivates the current study, which is to provide a clear design rule for glass formers, while also building on the previous analyses by defining the compositional ranges which are glass formers. A summary of the various design rules (thermochemical, structural, and kinetic) for glass formability is provided in the supplementary material.

We assess (i) the thermochemical space in the form of phase diagrams, and (ii) the structural aspect through utilizing a series of elemental descriptors which we have carefully assessed in our prior works^22^. To accelerate the interpretation of and design from phase diagrams, machine reading applications are utilized. We have previously described an approach for the rapid machine reading of phase diagrams^23^. We apply that approach to the assessment of phase diagrams, particularly as relates to deep eutectics, to allow for the rapid reading and integration of phase diagrams. This represents a challenge, as the thermochemical metric must be a value which is readable from a phase diagram (as opposed to T_RG_ and ΔT_x_ which do not correspond to points graphically appearing on the phase diagram). Using our previously developed elemental databases, machine reading capabilities, and applications of machine learning approaches to a variety of design problems^22–24^, we are able to address the following objectives in this paper:

i. Develop a quantitative definition of ‘deep eutectic’
ii. Identify compositional ranges, as opposed to single composition values, for glass formability of a compound (based on a probabilistic approach)
iii. Integrate experimental and computational data (in the form of phase diagrams), and thermochemical and structural data, to develop a clear glass forming design rule.
iv. Generalize this framework for accelerated design so that all data entries are applicable to machine reading approaches.

## Results

As identified previously, a key component in predicting new metallic glasses has been to identify deep eutectic compositions indicating the importance of analyzing phase diagram information. We have previously demonstrated our automated phase diagram reading methodology^23^, which correctly classified phases and associate phase labels with 94% accuracy, and which we demonstrated with the capability to identify and characterize eutectic points. This capability is a key component of the design framework developed in this project. We summarize that prior work here.

**Figure 1:**
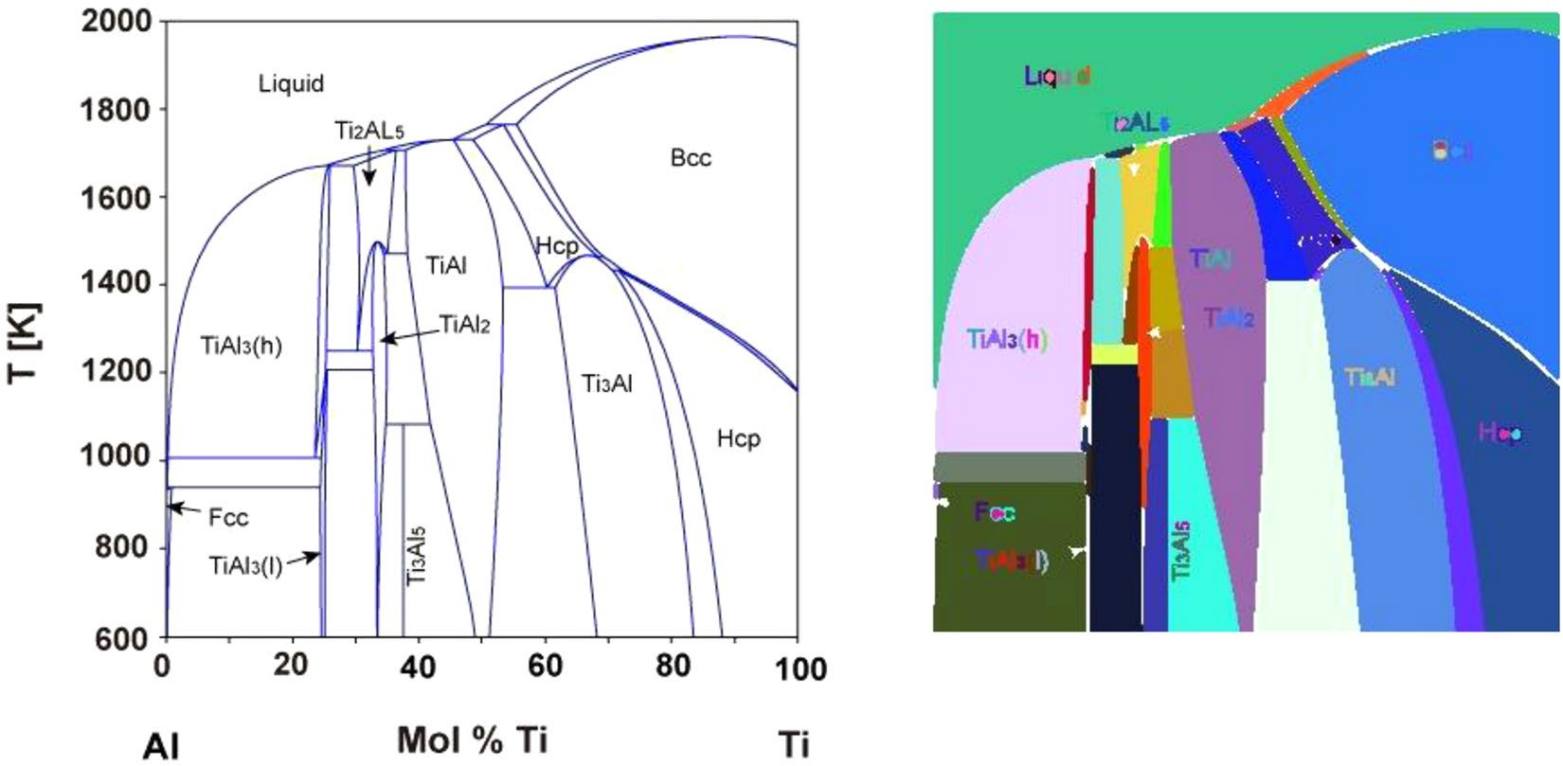
Demonstration of process for automated analysis of phase diagrams. (Left) Detection and recognition of text used to label phases. From this input figure, boundary lines are identified through Hu’s invariant moments approach, which allows us to define the shape of the curves, such as the shape of the liquidus lines at the eutectic point. (Right) Through contour analysis, we identify and separate the different regions, associated with their labels. From this, we are able to detect eutectic points by defining the eutectic point as corresponding to a single point where liquid and two separate solid + liquid phases meet.

As opposed to typical line plots, phase diagram lines represent boundaries instead of changing values. Therefore, the boundaries are not represented through simple tabular formats, and thus require a more in depth analysis than a simple digitization. An additional challenge includes the accurate identification of textual labels where the variations may arise in the form of orientation and placement (horizontal vs vertical vs labels indicated by arrows), language (Latin alpha numeric characters vs Greek characters) and the image resolution of the label where a high resolution is preferred but is not always the case in phase diagrams. Furthermore, phase diagrams have different line thicknesses, and particularly important for this application is the correct assignment of text to a phase. Ultimately we need to identify the phase boundaries between the liquid phase and two solid plus liquid phases. While these can be identified easily by eye, it is not a trivial exercise for a machine to read, although machine reading is required for scaling up and accelerating the design. A final point worth mentioning is the assignment of phase labels to regions which are unlabeled in the diagram since labeling in these regions would need to be built as a standard functionality on top of the above rules. Figure 1 depicts the current challenges in the computational analyses of phase diagrams.

This work builds on some standard challenges in diagram recognition, such as handling types of different diagrams, the complexity in representing the syntax and semantics, and particularly the challenge in handling noise^25^. In order to develop a phase diagram reading tool, a database of approximately 720 phase region contours and about 7100 text region contours was created. A gradient boosted tree-based (GBT) classifier was applied to classify between phase contours and text contours. The eutectic points can then be determined by analyzing the contour of the liquid phase for which both contour separation and accurate matching of label and region is critical. When compared to other classifiers, we found the GBT classifier to have the most robust classification to unbalanced data. In order to distinguish between text and phase contours, we applied Hu’s invariant moments^26^, which describe the shapes of the contours. The moments in this context referring to statistical moments. The 1st order moment for a group of points is their mean and thus for a shape or contour it will be a centroid. The 2nd order moments are proportional to variance and for shapes hold the orientation and eccentricity information (due to eigen decomposition).

In addition to the challenges mentioned above, a key quantity is the “depth” of the eutectic. Since there is no formal definition of this qualitative term, defining this in logical terms so that a machine learning algorithm is able to filter the appropriate systems is a challenge. By defining the eutectic angle in our study as the angle formed by the tangents of each curve at both ends of the eutectic point, we are able to surmise a rough idea of the geometrical features necessary in a given phase diagram for the system to constitute a metallic glass and hence provide a rapid screening. Furthermore, due to the nature of the assumptions and to offset the associated uncertainty, we use rough sets to provide a probabilistic framework within which we define our subsequent predictions.

### Combining Machine Learning with the Heuristics Space

**Figure 2:**
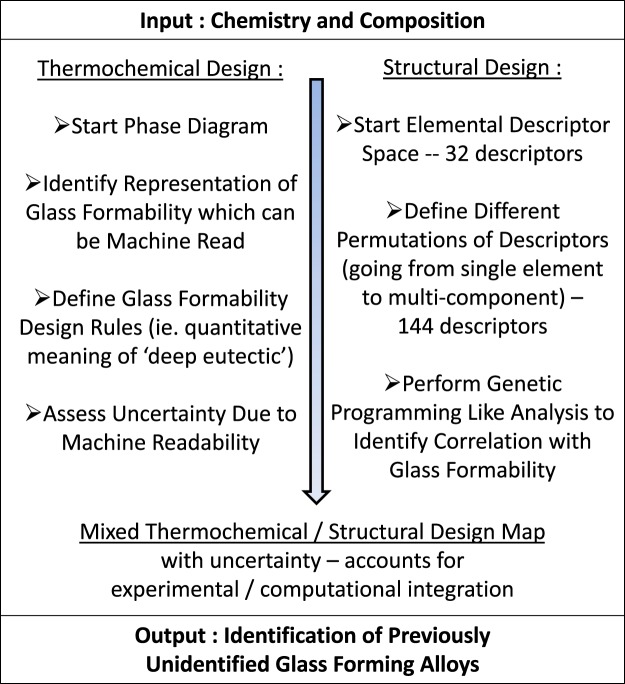
Approach developed for probabilistic design of new glass forming alloys. This approach encompasses two regimes of the defined design rules of Table S1.

A focus on the design of BMG systems has been the eutectic points within the thermodynamic phase diagram of a system. The rule of thumb has been that concentration regions lying within or near “deep eutectics” have the highest probability for possessing GFA since alloys close to such a composition tend to form stable liquids at lower temperatures, allowing for efficient glass formation^7^. An approach to identify and understand GFA has used T_0_ curves to calculate the Glass Forming Region (GFR) within an alloy system, with the T_0_ curve as the locus of the temperatures and compositions where the free energies of the two phases are equal^27^. The challenge in utilizing this value in a broader framework is that this value represents points which do not exist on the actual diagram. An objective of this work is to provide a probabilistic framework which can provide rapid search of the existing knowledge base through machine reading approaches. Therefore, to address this issue, we define an approach where a measure of “deep eutectic” is represented through an actual geometrical feature that can be extracted from the phase diagram. The logic we have developed and apply in this paper is described in Fig. 2, with the approach encompassing the thermochemical aspect of design represented by our representation of deep eutectic and the structural design represented through elemental descriptors.

Our approach predicts glass formability as a function of the alloy chemistry, as well as the composition, allowing us to identify not just the applicable chemistries, but additionally the compositional ranges through the application of uncertainty analysis. From the phase diagrams, we define a measure of glass formability which can be machine read, as opposed to the existing rules associated with T_0_. This allows us to screen a larger number of diagrams than otherwise possible. These measures are coupled with a separate informatics analysis which correlates elemental descriptors of the constituent elements with glass formability. The integration of these two separate design metrics allows us to account for the complexity of glass formability and to uncover previously unidentified chemistries. Uncertainty analysis is integrated into this framework to account for uncertainty associated with the new representation of deep eutectics, the lack of a clear quantitative definition of deep eutectic, and the compositional spread of glass forming compounds at off eutectic compositions.

Our training data for defining glass formability is based on the work of Miracle^18^, where he assessed the various contributions, largely based on radii, to define the metallic glass forming compounds. We assess 200 chemistries, with 385 eutectic compositions, for phase diagram calculations based on experimental observations^18,28–31^. The class assignment is based on Miracle’s comprehensive table. We couple the phase diagram with our extensive compositional database, to define clear design rules spanning thermochemical and structural criteria.

### Descriptor Extraction from Phase Diagrams

**Figure 3:**
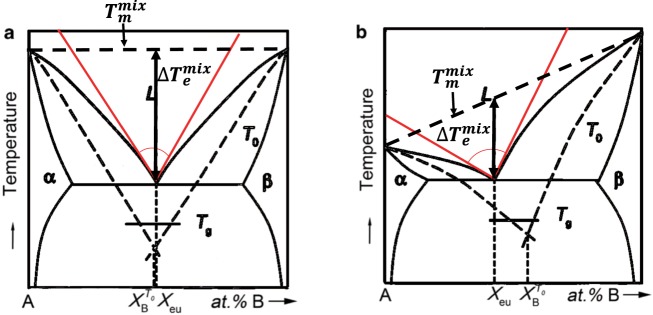
Schematic depicting the eutectic angle measurement. (**a**) Is for a symmetric eutectic system and (**b**) is depicted for an asymmetric system. In both figures for a hypothetical AB compound phase diagram, the T_0_ lines are drawn (dashed lines). The T_0_ lines intersect at ${X}_{B}^{{T}_{0}}$ and the GFR is represented by the intersection of these lines with T_g_. In the first case, the GFR is around the eutectic composition, whereas in the second case, it is slightly off-eutectic. For the current study, the eutectic angle was measured by drawing tangents to the liquidus lines at either end of the eutectic point (lines drawn in red depict the tangents). We observe that this method of drawing tangents and measuring their intersection angle is a rough estimate of the geometry between the T_0_ lines, but does not require the need to calculate the geometry, thus including important thermodynamic information within the model. Phase diagrams were redrawn from Shi *et al*.^27^.

As discussed, a key design guideline is the selection of a ‘deep eutectic’. This presents a design challenge as the term is largely ambiguous. Effectively, it implies the need for steep liquidus lines at the convergence of the eutectic point, indicating an inclination to remain liquidus (ie. amorphous). However, no clear quantitative definition or guidelines exist. A well accepted approach is summarized in Fig. 3. For instance, by narrowing the alloy search space to compositions close to the eutectic composition, liquid stability is enhanced at low temperatures^7^. As an example of a parameter used to represent the concept of deep eutectic, a line T_m_^mix^ can be defined as a common tangent line connecting the maximum temperatures of the liquidus lines (ie. maximum melting temperatures of the solid + liquid phases). The temperature difference (ΔT_e_^mix^) between T_m_^mix^ and T_eutectic_ provides a potential representation of the deepness of a eutectic^2^. An alternately proposed approach is to use T_0_ curves to calculate the GFR within an alloy system. The GFR phase is defined as the range of compositions within the alloy system where it is least likely to find nucleation of crystal phases. Between the solid and liquid phases, the curve predicts the minimum undercooling of the liquid for the partition-less formation of the crystalline solid with the same composition^27^. Hence we took the approach of identifying alternative geometrical characteristics which lend themselves to autonomous detection and can potentially serve as surrogate features that can be associated with the geometrical constructs described above. Hence we used the acuteness of angle between the tangents of the liquidus lines at the eutectic point as a rough measure of “deepness” of the eutectic. It should be emphasized that we treat this angular metric a surrogate feature that relates to one of many metrics that are used as indicators of glass forming ability, its value that lies in the fact that it lends itself to automated extraction of features in diagrams. Also the qualitative nature of this metric highlights the need for introducing the concepts of addressing uncertainty analysis in materials informatics studies, where the uncertainty is governed by heuristics.

To consider the structural component of metallic glass formability, we developed a descriptor space based on elemental components. This included size effects, electronic effects, and bonding characteristics. While radii have been identified as a key component previously, by performing a principal component analysis (PCA) we were able to quickly screen the descriptor space on the impacts on glass formability. In addition to the thermodynamic parameters discussed, certain empirical rules have been suggested to aid in the a priori discovery of bulk metallic glasses. In addition to the previously discussed rules, various other rules/suggestions have been identified involving thermodynamic descriptors such as melting temperature and enthalpy of mixing^16^, as well as structural and electronic parameters such as the bulk moduli and Pauling electronegativity^18^. Many of these rules seem to work well on certain systems while failing in others. Thus, even within the heuristics space, a unified approach is lacking.

**Figure 4:**
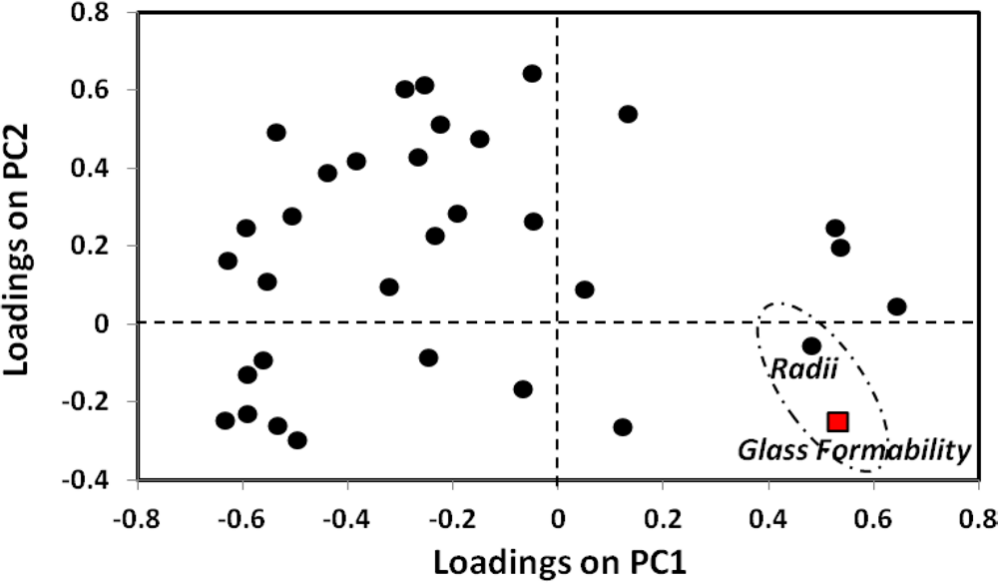
Correlation mapping of a range of elemental descriptors with glass formability. The elemental descriptors include radii, electronegativities, enthalpies, energetic contributions, electronic structure configurations, spatial misfits, thermal properties, and mechanical properties, among others, with each black circle representing a different descriptor. The axes represent the contribution onto the principal components (PCs) characterizing the different alloy systems. PC1 captured 41.4% of the variance, PC2 captured 33.1%, PC3 captured 14.5%, and all other PCs captured 11.0%. The identification of radius is consistent from two PC mapping, as well as three PC mapping. Glass formability (red square) is defined in a binary fashion to those systems which form a glass versus those that do not. From this figure, we find that radius has the largest correlation to glass formability and therefore is the key descriptor that we consider for predicting glass formability.

This motivates the application of a manifold learning approach to assess the elemental contributions to glass formability. The descriptor for glass formability was defined in a binary fashion, with zero being the compounds which have not been identified as glass forming and unity being the compounds which have been identified as glass forming. That is, the compounds labeled as “zero” are those which were not listed in the review work of Miracle^18^, which we used as the basis for training, and therefore does not mean that the compound cannot form a metallic glass, but rather that it has not been identified. This last point is important as in our models, we do not consider compounds classified as zero but which are identified as glass forming as ‘false positives’. That is, while the training data is comprehensive, it is not complete. From the result of principal component analysis (PCA) (Fig. 4), where Euclidean proximity defines correlation, we find that a function of the atomic radii is the key descriptor on glass formability, as no other descriptors sit near the glass formability point within the same quadrant. Therefore, we have recovered and expanded on existing knowledge in an unbiased manner.

**Figure 5:**
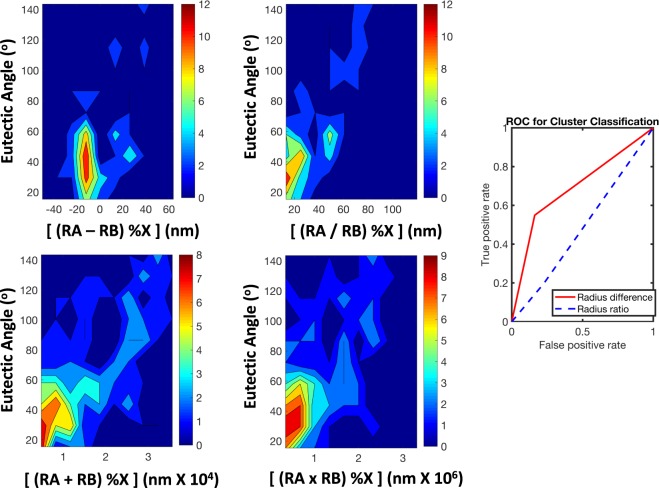
Assessment of the different combinations of elemental atomic radii to represent the multi-component contribution to glass formability. The left figure represents the denmapping of glass forming compounds with radii difference, radii ratio, radii sum, and radii product. RA is defined as the radius of the majority element and RB is the radius of the minority element. %X represents the atomic percentage of the majority element in the eutectic composition. We find the highest density representations are for the difference and ratio. To select between the two combinations, we consider the ROC (Right). From this analysis, we find that the radii difference provides the highest accuracy classification of glass formability. Therefore, in the final design map, we select this function as the representation of structural contribution to glass formability.

To assess the potential combinations of radii, we follow our previous works where we used the scaling of Villars^32–34^, Pettifor^35^, and Miedema^36,37^ to expand from elemental descriptors to the alloy contribution. However, for this particular case, no sufficient expansion exists for the radii, and therefore we need to assess the various combinations (particularly sum, difference, product, and ratio). Further, as will be discussed in the next section, a system may have multiple eutectic points, but without all of the eutectic points corresponding to glass forming compositions. Therefore, the radii contributions need to be normalized by the composition. The result of the various density mappings of metallic glass forming compounds, with the eutectic angle versus the different radii contributions, is shown in Fig. 5. The objective is to find the alloy expansion which results in the highest density and most localized representation of metallic glass forming binaries. The contouring of the figures refers to the density of glass forming compounds, and within this we clearly find the radii difference and radii ratio as being the most reasonable representations, as the sum and product representations have a large spread of data. To compare the two descriptor scalings, we visualized the performance through Receiver Operating Characteristics (ROC), which quantify the performance of class assignment^38^. In this case, the two metrics are compared in terms of the number of correctly assigned systems versus incorrectly classified systems. In this case, we find that the radii difference has a much higher classification accuracy than the radii ratio, therefore motivating our final selection of radii difference to represent the structural contribution to glass formability.

Therefore, we have identified the ‘eutectic angle’ as a measurable metric associated with a deep eutectic and radii difference scaled by composition are the optimal descriptors for discriminating glass forming compounds and non-glass forming compounds. Although we have a good binary classifier in this case, in principle we can develop more complex classifier metrics for more chemically complex systems based on the framework described in this study.

**Figure 6:**
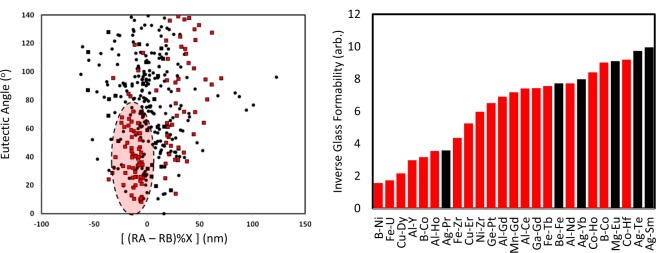
Design mapping for discovery of new glass forming alloys. (Left) From a mapping of the two identified design metrics (the metric ‘eutectic angle’ capturing thermochemical behavior and radii difference capturing structural behavior while accounting for composition), we are able to uncover a high density clustering (circled region) of glass forming chemistries. The red squares are compounds which have been identified as glass forming, the black circles have not been identified as glass forming, and the black squares correspond to glass forming compounds but with compositions which fall outside of the circled region. RA, RB and %X are defined as the same as in Fig. 5. This demonstrates the utilization of the previously unidentified descriptors for uncovering new targeted compounds. (Right) Distance from the middle of the circled region, indicating the propensity of glass formability, with the coloring corresponding with the left figure. From this, we identify six new chemistries for glass forming compounds.

The plotting of the training data for these systems is shown in Fig. 6. From this figure and the density of glass formers in Fig. 5, we find a clear cluster of glass forming compounds at radii differences just below zero, and with eutectic angles less than 75°.

Further, if we measure the Euclidean distance from the middle of the cluster (defined as the highest density grouping in Fig. 5), we can define the likelihood of glass formability. The circle drawn in Fig. 6 is mainly intended as a visual aid, but a more quantitative perspective is provided by using rough set theory. We have employed rough set theory to statistically define the region boundary while taking into account uncertainty, thus providing a thorough analysis of the region selected. This description is provided in the next section. As far as the selection of the center of the design region, we have performed an additional analysis to verify the selection of this point. We selected multiple points within the region to test for metallic glass compounds, as was done for the center point to identify compounds in Fig. 6. This analysis coupled with the rough set analysis in subsequent sections allows us to predict the composition as well as uncertainty of new metallic glass formers quantitatively (Table 1). We found through this analysis, that the point selected captured 28% more known metallic glasses than any of the other points tested. Therefore, the center point used captured the most known metallic glasses, which was the objective of this step of the work, i.e. to capture the most known systems within some distance and then explore the unknown compounds. From this, we find that Ag-Pr has a very high propensity towards glass formation, while not being previously identified as such. Further, we identify Be-Fe, Ag-Yb, Mg-Eu, Ag-Te and Ag-Sm as compounds with previously unidentified high likelihood towards glass formability. Therefore, we have developed a new approach for machine reading of phase diagrams which allows us to discover new glass forming compounds, demonstrating the utilization of the merger between machine reading and machine learning.

**Table 1:** List of previously unidentified glass forming compounds, and the composition of the pertinent eutectic point, based on the design map.

No.	System	Composition(s)
1.	Ag-Yb	Ag_20.7_Yb_79.3_
2.	Mg-Eu	Mg_30.3_Eu_69.7_
3.	Ag-Eu	Ag_21.03_Eu_78.97_
4.	Ag-Ho	Ag_19_Ho_81_
5.	Ag-Pr	Ag_22.28_Pr_77.72_
6.	Ag-Sm	Ag_18_Sm_82_
7.	Al-Th	Al_31.16_Th_68.84_, Al_42.79_Th_57.21_
8.	B-Cu	B_13.1_Cu_86.9_
9.	B-Mn	B_14.38_Mn_85.62_, B_38.58_Mn_61.42_
10.	Be-Fe	Be_35.01_Fe_64.99_
11.	N-Fe	N_8.93_Fe_91.07_, N_15.82_Fe_84.18_
12.	Na-Sb	Na_55.56_Sb_44.44_
13.	Pd-In	Pd_37.05_In_62.95_
14.	Rb-Tl	Rb_13.24_Tl_86.76_
15.	Ag-Pb	Ag_4.51_Pb_95.49_
16.	Ag-Te	Ag_69.66_Te_30.34_, Ag_88.52_Te_11.48_
17.	Al-Pd	Al_22.89_Pd_77.11_, Al_41.04_Pd_58.96_
18.	B-Re	B_41.78_Re_58.22_
19.	Be-Ag	Be_10.79_Ag_89.21_
20.	Be-Ni	Be_23.9_Ni_76.1_
21.	Be-Th	Be_34.44_Th_65.56_
22.	C-Ni	C_2.87_Ni_97.13_
23.	Cd-La	Cd_22.47_La_77.53_
24.	Mg-Pb	Mg_14.82_Pb_85.18_
25.	O-Cu	O_1.69_Cu_98.31_, O_39.27._Cu_60.73_
26.	Pr-Tl	Pr_61.68_Tl_38.32_
27.	Ru-U	Ru_18.52_U_81.48_
28.	Sb-Gd	Sb_13.04_Gd_86.96_
29.	Sb-La	Sb_3.18_La_96.82_
30.	Sb-Nd	Sb_4.47_Nd_95.53_
31.	Sb-Pr	Sb_4.86_Pr_95.14_
32.	Sb-U	Sb_46.76_U_53.24_
33.	Se-Tl	Se_41.91_Tl_58.09_
34.	Si-Ag	Si_4.1_Ag_95.9_
35.	Si-Ba	Si_13.13_Ba_86.87_
36.	Sn-La	Sn_12.41_La_87.59_
37.	Tb-Tl	Tb_73.75_Tl_26.25_, Tb_84.83_Tl_15.17_
38.	Yb-Pb	Yb_54.99_Pb_45.01_, Yb_77.31_Pb_22.69_

## Discussion

**Figure 7:**
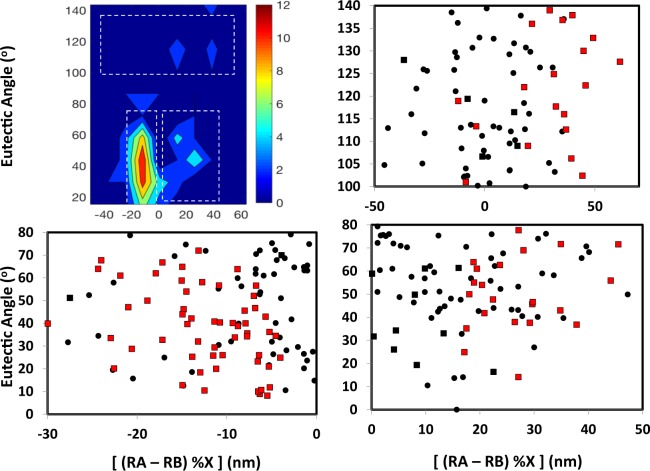
Design map comparing regions of high density metallic glass formers and low density regions. RA, RB and %X are defined as in Fig. 6. In the bottom left panel, the key design area is highlighted, where we see a very high proportion of glass forming compounds. We applied rough set theory to further assess these regions, account for uncertainty, and define compositional ranges that can be accommodated. The right panels show regions of low density which contain glass formers not following our design rules. This highlights that the approach discussed here predicts positive response and is not intended to predict negative response, in terms of glass forming ability. However, as highlighted in this figure, the majority of glass forming compounds falls within our defined design region. From this, we identify 38 previously unidentified glass forming compounds (Table 1).

Focusing on specific regions of the selection map, we consider the different regions where metallic glass forming compounds exist. An important consideration is that the selection criteria defined here is for identifying if a compound is a metallic glass former, and not for defining the negative of this criteria. This can be seen in the high density region of Fig. 7, where a very high proportion of the compounds are glass forming, while the other highlighted regions have some metallic glass formers intermixed with non-glass formers. Extracting the values around the high density glass region, we define a set of design criteria for identifying new glass forming compounds across multiple design criteria:

### Thermochemical Design

Require a ‘deep eutectic’, meaning an angle between the tangents of the liquidus lines of less than 72°.

### Structural Design

Require that the two constituent atoms have similar atomic radii, but with the minority element having a larger size than the majority element.

### Compositional Design

Require a composition close to a deep eutectic composition, and selecting composition such that the difference in radii scaled by the composition of the majority element results in a value between −25 and 0 nm.

**Figure 8:**
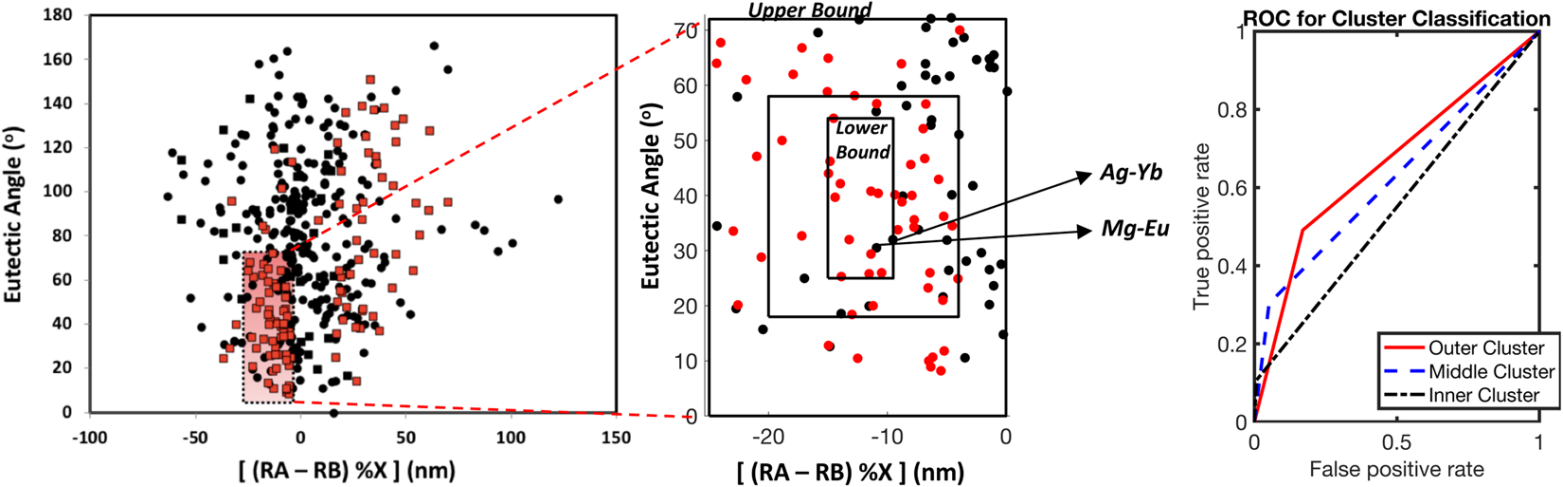
Rough set analysis of the metallic design region, to incorporate uncertainty due to the unclear quantitative definition of ‘deep eutectics’ and to expand our rule to define the compositional ranges that would be applicable, while providing confidence measures of the uncertainty. This figure highlights the different confidence intervals, which are further elucidated in Table 2. A compound falling in the lower bound is very likely a glass former, although we only capture a fraction of all glass formers. Relaxing the confidence requirements allows us to screen a larger space, but with larger uncertainty, as shown in the ROC curve.

To account for the uncertainty in our descriptor definition, the fuzziness of the definition of a ‘deep eutectic’ and to account for compositional fluctuations, we applied a rough set analysis. The main aim was to be able to classify each data point as a metallic glass system or not. In the current study, the decision system,

$$DS\,:T=(U,A{\cup }^{}\{d\})d\notin A$$

represents the model^39–41^. Here, *U* represents the 385 eutectic points studied, a non-empty finite set of objects; *A* represents the attribute set which in our case are the system identifiers, the eutectic angle and radii difference scaled with the atomic eutectic composition; and *d* is the decision attribute (whether or not the system forms a bulk metallic glass). From the analysis, three decision criteria were set: good bulk metallic glass former (“yes”), bad bulk metallic glass (“no”) and an in-between category where both the probability of a good or bad bulk metallic glass former exists (“yes/no”). Effectively this defines a ‘lower boundary’ which captures only glass formers and an ‘upper boundary’ which captures all glass formers. As discussed, some glass formers fall outside of our design range and therefore we relax the definitions of boundaries, and instead only focus on the high density region from Fig. 6. The result of our analysis is provided in Fig. 8. This therefore defines the confidence that a compound will form a bulk metallic glass. However, it should be noted that these terms are relative, as we anticipate all of the compounds within even the ‘low’ confidence region are glass forming, as no negative reports have been given on these compounds. Within the low confidence region, we do have a smaller compositional range that will form metallic glasses.

Based on these limits, we propose Ag-Yb and Mg-Eu as the potential to form metallic glasses with the highest confidence. While Ag and Yb have been used as alloying elements in multicomponent metallic glass systems to a successful degree and the scope and applications of Mg based metallic glass systems has been extensively studied^42–44^, their binary compositions predicted here have not yet been studied for GFA. The binaries Ag-Pr, Be-Fe, Ag-Yb, Mg-Eu, Ag-Te and Ag-Sm are also proposed with confidence to form bulk metallic glasses. Of these, Ag-Pr has been suggested as a potential metallic glass former^15^ and Fe, Ag and Te have been used as additions in multicomponent metallic glass systems in various combinations^45–47^, similar to Ag-Yb and Mg-Eu. Furthermore, nearly pure Ag-Te has been reported in some cases when investigating Te- based chalcogenide glasses^48^.

As an example of defining compositional limitations to glass formation, for Ag-Yb the eutectic concentration is 20.7% Ag. The radius of Ag is 144 pm and the radius of Yb is 190 pm, and therefore rA–rB is equal to −46 pm. When scaled by the composition, the value of (rA–rB) * %A is equal to −9.53. We can define the compositional ranges which would result in values between −25 and 0, as defined in Table 2 (thus highlighting the meaning of low confidence in the table), given the thermodynamic constraints of the melting temperature of the compound not reducing at a given composition and that the eutectic tie line has not terminated at that composition. Based on our compositional rule, and given the confidence definitions, the potential high confidence compositions are between 20.6% Ag and 32.6% Ag. Therefore, not only have we identified that Ag-Yb is a glass former, we have defined the compositional range for which it is a glass former.

**Table 2:** Summary of systems captured within each cluster limit. Here ‘x’ and ‘y’ represent the x and y axes within our model.

Boundary/Limits	Confidence	% BMG Captured
A $-25 < x < 0$ ${0}^{o} < y < {72}^{o}$	Low	78.57
B $-20 < x < -\,4$ ${18}^{o} < y < {58}^{o}$	Medium	51.79
C $-15 < x < -\,9.5$ ${25}^{o} < y < {54}^{o}$	High	19.64

This provides a quantitative definition of the term ‘deep eutectic’ and defines the ‘deepness’ of the eutectic, with an angle less than 54° at the eutectic point indicating a deep eutectic. From the uncertainty incorporation, we identify the trade-off between confidence of prediction and the size of the search space (% BMG captured).

Our machine learning aided approaches to describe “deep eutectics” based on geometric features of phase diagrams can be extended to multicomponent phase diagrams, even though the number of available phase diagrams becomes more limited as we increase the number of constituent elements. For instance, one can interrogate phase diagrams where crystallization pathways and/or temperature isotherms are mapped onto the compositional space of the ternary or quaternary phase diagrams. One can then use our automated feature detection strategy to map out the curvature of the liquidus surface to characterize a “deep eutectic’ for example. We are presently extending our work to multicomponent phase diagrams, the results of which will be reported at a later date.

## Conclusion

In this paper, we identified 38 new glass forming compounds, and introduced a metric for prediction confidence and defined the compositional range of glass formability. Within this, we have now quantitatively defined the meaning of a deep eutectic, with 54° between the tangents of the liquidus lines at the eutectic point defining the most rigorous definition of ‘deep’. This approach has been developed so that the pertinent information is machine read, providing an automated design framework. The theory for metallic glass formation has been expanded to include both the deep eutectic definition and a structural design aspect, which is based on atomic radii. The highest probability of metallic glass formation requires the minority element be slightly larger than the majority element. Future reports will describe experimental synthesis and characterization of predicted compounds, as well as new machine reading approaches which capture not just points on the phase diagram, but also capture points (such as T_o_ and T_mix_) which do not correspond to points on the diagram. This represents a unique merger of thermochemical design and machine learning, with implications for many graph based design applications.

## Electronic supplementary material

Supplementary File 1

Supplementary File 2

Supplementary File 3

Supplementary Data
