# Supplementary material for: Probabilistic Assessment of Glass Forming Ability Rules for Metallic Glasses Aided by Automated Analysis of Phase Diagrams
Source: Sci Rep. 2019 Jan 23;9:357. doi: 10.1038/s41598-018-36224-3 (PMC6344582; doi:10.1038/s41598-018-36224-3)
Supplement: Supplementary file 1 [file 41598_2018_36224_MOESM1_ESM.docx]

FN Clarivate Analytics Web of Science

VR 1.0

PT B

AU Liu, CT

Greer, AL

Schuh, CA

AF Liu, C. T.

Greer, A. Lindsay

Schuh, Christopher A.

BA Suryanarayana, C

Inoue, A

BF Suryanarayana, C

Inoue, A

TI BULK METALLIC GLASSES Foreword

SO BULK METALLIC GLASSES

BN 978-1-4200-8596-9

PY 2011

BP XV

EP XVIII

D2 10.1201/9781420085976-1

UT WOS:000288438000001

ER

PT J

AU Ashley, S

AF Ashley, S

TI Metallic glasses bulk up

SO MECHANICAL ENGINEERING

SN 0025-6501

PD JUN

PY 1998

VL 120

IS 6

BP 72

EP 74

UT WOS:000073847400035

ER

PT B

AU Suryanarayana, C

Inoue, A

AF Suryanarayana, C

Inoue, A

TI Bulk Metallic Glasses

SO BULK METALLIC GLASSES

BN 978-1-4200-8596-9

PY 2011

BP 1

EP 523

DI 10.1201/9781420085976-1

UT WOS:000288438000013

ER

PT J

AU Yavari, AR

Ochin, P

AF Yavari, AR

Ochin, P

TI Bulk metallic glasses - Introduction

SO ANNALES DE CHIMIE-SCIENCE DES MATERIAUX

RI yavari, alain/E-8192-2010

SN 0151-9107

PD SEP-OCT

PY 2002

VL 27

IS 5

BP 1

EP 2

DI 10.1016/S0151-9107(02)80038-1

UT WOS:000179384800001

ER

PT J

AU Liaw, PK

Choo, H

Gao, YF

Wang, GY

AF Liaw, Peter K.

Choo, Hahn

Gao, Yanfei

Wang, Gongyao

TI Bulk Metallic Glasses VI

SO METALLURGICAL AND MATERIALS TRANSACTIONS A-PHYSICAL METALLURGY AND

MATERIALS SCIENCE

SN 1073-5623

PD JUL

PY 2010

VL 41A

IS 7

BP 1627

EP 1627

DI 10.1007/s11661-010-0277-4

UT WOS:000277958700005

ER

PT J

AU Liaw, PK

Wang, GY

Choo, H

Gao, YF

AF Liaw, Peter K.

Wang, Gongyao

Choo, Hahn

Gao, Yanfei

TI Bulk Metallic Glasses IX Foreword

SO METALLURGICAL AND MATERIALS TRANSACTIONS A-PHYSICAL METALLURGY AND

MATERIALS SCIENCE

RI Choo, Hahn/A-5494-2009

OI Choo, Hahn/0000-0002-8006-8907

SN 1073-5623

PD MAY

PY 2013

VL 44A

IS 5

BP 1979

EP 1979

DI 10.1007/s11661-012-1493-x

UT WOS:000316762400002

ER

PT J

AU Baricco, M

AF Baricco, Marcello

TI Special issue "Bulk metallic glasses"

SO ADVANCED ENGINEERING MATERIALS

RI Baricco, Marcello/B-4075-2013

OI Baricco, Marcello/0000-0002-2856-9894

SN 1438-1656

PD JUN

PY 2007

VL 9

IS 6

BP 431

EP 431

DI 10.1002/adem.200790013

UT WOS:000247885800001

ER

PT J

AU Liaw, PK

Wang, GY

Schneider, J

AF Liaw, Peter K.

Wang, Gongyao

Schneider, Judy

TI Advances in bulk metallic glasses

SO JOM

SN 1047-4838

PD APR

PY 2010

VL 62

IS 4

BP 9

EP 9

DI 10.1007/s11837-010-0066-y

UT WOS:000276770100003

ER

PT J

AU DAVIS, LA

YEOW, YT

ANDERSON, PM

AF DAVIS, LA

YEOW, YT

ANDERSON, PM

TI BULK STIFFNESSES OF METALLIC GLASSES

SO JOURNAL OF APPLIED PHYSICS

SN 0021-8979

PY 1982

VL 53

IS 7

BP 4834

EP 4837

DI 10.1063/1.331313

UT WOS:A1982NX94500035

ER

PT J

AU Sealy, C

AF Sealy, Cordelia

TI Bulk metallic glasses overcome brittleness

SO MATERIALS TODAY

SN 1369-7021

PD MAY

PY 2007

VL 10

IS 5

BP 13

EP 13

UT WOS:000246484200008

ER

PT J

AU Liu, JB

AF Liu, Jianbo

TI Notes on the glass-forming ability of bulk metallic glasses

SO PHYSICS TODAY

SN 0031-9228

EI 1945-0699

PD FEB

PY 2014

VL 67

IS 2

BP 10

EP 11

DI 10.1063/PT.3.2256

UT WOS:000334252900002

ER

PT J

AU Schroers, J

AF Schroers, Jan

TI Notes on the glass-forming ability of bulk metallic glasses

SO PHYSICS TODAY

SN 0031-9228

EI 1945-0699

PD FEB

PY 2014

VL 67

IS 2

BP 11

EP 11

DI 10.1063/PT.3.2257

UT WOS:000334252900003

ER

PT J

AU Tao, Z

AF Tao, Zhang

TI Special Topic on Bulk Metallic Glasses Preface

SO SCIENCE CHINA-PHYSICS MECHANICS & ASTRONOMY

SN 1674-7348

PD MAR

PY 2010

VL 53

IS 3

SI SI

BP 389

EP 389

DI 10.1007/s11433-010-0156-4

UT WOS:000276661700001

ER

PT J

AU Liaw, PK

Wang, GY

Choo, H

Gao, YF

AF Liaw, Peter K.

Wang, Gongyao

Choo, Hahn

Gao, Yanfei

TI Symposium on Bulk Metallic Glasses-XI Foreword

SO METALLURGICAL AND MATERIALS TRANSACTIONS A-PHYSICAL METALLURGY AND

MATERIALS SCIENCE

RI Choo, Hahn/A-5494-2009

OI Choo, Hahn/0000-0002-8006-8907

SN 1073-5623

EI 1543-1940

PD JUN

PY 2015

VL 46A

IS 6

BP 2380

EP 2380

DI 10.1007/s11661-015-2809-4

UT WOS:000353236700009

ER

PT J

AU Liaw, PK

Jiang, WH

Fan, GJ

Choo, H

Gao, YF

AF Liaw, Peter K.

Jiang, Wenhui

Fan, Guojiang

Choo, Hahn

Gao, Yanfei

TI Symposium on bulk-metallic glasses IV - Foreword

SO METALLURGICAL AND MATERIALS TRANSACTIONS A-PHYSICAL METALLURGY AND

MATERIALS SCIENCE

SN 1073-5623

PD AUG

PY 2008

VL 39A

IS 8

BP 1761

EP 1761

UT WOS:000256886600001

ER

PT J

AU Xie, X

Wang, GY

Choo, H

Gao, YF

Liaw, PK

AF Xie, Xie

Wang, Gongyao

Choo, Hahn

Gao, Yanfei

Liaw, Peter K.

TI Symposium on Bulk Metallic Glasses-XII Foreword

SO METALLURGICAL AND MATERIALS TRANSACTIONS A-PHYSICAL METALLURGY AND

MATERIALS SCIENCE

SN 1073-5623

EI 1543-1940

PD APR

PY 2017

VL 48A

IS 4

BP 1544

EP 1544

DI 10.1007/s11661-017-3981-5

UT WOS:000396057300006

ER

PT J

AU Liaw, PK

Wang, GY

Choo, H

Gao, YF

AF Liaw, Peter K.

Wang, Gongyao

Choo, Hahn

Gao, Yanfei

TI Symposium on Bulk Metallic Glasses VII Foreword

SO METALLURGICAL AND MATERIALS TRANSACTIONS A-PHYSICAL METALLURGY AND

MATERIALS SCIENCE

RI Choo, Hahn/A-5494-2009

OI Choo, Hahn/0000-0002-8006-8907

SN 1073-5623

PD JUN

PY 2011

VL 42A

IS 6

BP 1449

EP 1449

DI 10.1007/s11661-011-0690-3

UT WOS:000290176100005

ER

PT J

AU Liaw, PK

Jiang, W

Choo, H

Gao, Y

AF Liaw, Peter K.

Jiang, Wenhui

Choo, Hahn

Gao, Yanfei

TI TMS Bulk-Metallic Glasses Symposium V

SO ADVANCED ENGINEERING MATERIALS

SN 1438-1656

PD NOV

PY 2008

VL 10

IS 11

BP 995

EP 995

UT WOS:000261933400001

ER

PT J

AU Sealy, C

AF Sealy, Cordelia

TI Bulk metallic glasses break the mold

SO NANO TODAY

SN 1748-0132

PD APR

PY 2009

VL 4

IS 2

BP 111

EP 111

DI 10.1016/j.nantod.2009.02.003

UT WOS:000265814900005

ER

PT B

AU Liu, CT

Greer, AL

Schuh, CA

AF Liu, C. T.

Greer, A. Lindsay

Schuh, Christopher A.

BA Suryanarayana, C

Inoue, A

BF Suryanarayana, C

Inoue, A

TI BULK METALLIC GLASSES Epilogue

SO BULK METALLIC GLASSES

BN 978-1-4200-8596-9

PY 2011

BP 517

EP 523

D2 10.1201/9781420085976-1

UT WOS:000288438000012

ER

PT J

AU Johnson, WL

AF Johnson, WL

TI Science and technology of bulk metallic glasses.

SO ABSTRACTS OF PAPERS OF THE AMERICAN CHEMICAL SOCIETY

SN 0065-7727

PD MAR 26

PY 2000

VL 219

MA 96-PHYS

BP U282

EP U282

PN 2

UT WOS:000087246201555

ER

PT J

AU RAY, R

AF RAY, R

TI BULK MICROCRYSTALLINE ALLOYS FROM METALLIC GLASSES

SO METAL PROGRESS

SN 0026-0665

PY 1982

VL 121

IS 7

BP 29

EP 31

UT WOS:A1982NT41100004

ER

PT J

AU Liaw, PK

Wang, GY

Choo, H

Gao, YF

AF Liaw, Peter K.

Wang, Gongyao

Choo, Hahn

Gao, Yanfei

TI Foreword: Symposium on Bulk Metallic Glasses VIII

SO METALLURGICAL AND MATERIALS TRANSACTIONS A-PHYSICAL METALLURGY AND

MATERIALS SCIENCE

SN 1073-5623

PD AUG

PY 2012

VL 43A

IS 8

BP 2591

EP 2591

DI 10.1007/s11661-012-1153-1

UT WOS:000305732900006

ER

PT J

AU Liaw, PK

Wang, GY

Choo, H

Gao, YF

AF Liaw, Peter K.

Wang, Gongyao

Choo, Hahn

Gao, Yanfei

TI Symposium on Bulk Metallic Glasses-X Foreword

SO METALLURGICAL AND MATERIALS TRANSACTIONS A-PHYSICAL METALLURGY AND

MATERIALS SCIENCE

RI Choo, Hahn/A-5494-2009

OI Choo, Hahn/0000-0002-8006-8907

SN 1073-5623

EI 1543-1940

PD MAY

PY 2014

VL 45A

IS 5

BP 2351

EP 2351

DI 10.1007/s11661-013-2023-1

UT WOS:000334428000004

ER

PT J

AU Ruhle, M

AF Ruehle, Manfred

TI TMS Bulk-Metallic Glasses Symposium V

SO ADVANCED ENGINEERING MATERIALS

SN 1438-1656

PD NOV

PY 2008

VL 10

IS 11

BP DGM1

EP DGM1

UT WOS:000261933400002

ER

PT J

AU Schroers, J

AF Schroers, Jan

TI BULK Metallic Glasses

SO PHYSICS TODAY

SN 0031-9228

EI 1945-0699

PD FEB

PY 2013

VL 66

IS 2

BP 32

EP 37

DI 10.1063/PT.3.1885

UT WOS:000314671800014

ER

PT J

AU Sealy, C

AF Sealy, Cordelia

TI Bulk metallic glass could keep hearts beating

SO MATERIALS TODAY

SN 1369-7021

EI 1873-4103

PD NOV

PY 2015

VL 18

IS 9

BP 478

EP 478

UT WOS:000363533300012

ER

PT J

AU XU, YF

XINMING, H

CHEN, H

WANG, WK

AF XU, YF

XINMING, H

CHEN, H

WANG, WK

TI FORMATION OF PD40NI40P20 BULK METALLIC-GLASS

SO JOURNAL OF MATERIALS SCIENCE LETTERS

SN 0261-8028

PD JUL

PY 1990

VL 9

IS 7

BP 850

EP 851

UT WOS:A1990DP61200041

ER

PT J

AU KUI, HW

GREER, AL

TURNBULL, D

AF KUI, HW

GREER, AL

TURNBULL, D

TI FORMATION OF BULK METALLIC-GLASS BY FLUXING

SO APPLIED PHYSICS LETTERS

RI Greer, Lindsay/E-9433-2017

SN 0003-6951

PY 1984

VL 45

IS 6

BP 615

EP 616

DI 10.1063/1.95330

UT WOS:A1984TK68700009

ER

PT B

AU Arentoft, M

Eriksen, RS

Hansen, HN

AF Arentoft, Mogens

Eriksen, Rasmus Solmer

Hansen, Hans Norgaard

BE Qin, Y

TI Micro-Bulk-Forming

SO MICRO-MANUFACTURING ENGINEERING AND TECHNOLOGY

SE Micro & Nano Technologies

OI Hansen, Hans Norgaard/0000-0001-7829-6962

BN 978-0-8155-1980-5

PY 2010

BP 114

EP 129

DI 10.1016/B978-0-8155-1545-6.00007-7

UT WOS:000317969200008

ER

PT J

AU Ranjan, V

AF Ranjan, V

TI Bulk metallic glass foam achieves high ductility

SO MRS BULLETIN

SN 0883-7694

PD APR

PY 2005

VL 30

IS 4

BP 267

EP 267

DI 10.1557/mrs2005.89

UT WOS:000228438400010

ER

PT J

AU MAYER, A

STAUDHAMMER, K

JOHNSON, K

AF MAYER, A

STAUDHAMMER, K

JOHNSON, K

TI ELECTROFORMED BULK NICKEL-PHOSPHORUS METALLIC-GLASS

SO PLATING AND SURFACE FINISHING

SN 0360-3164

PY 1985

VL 72

IS 11

BP 76

EP 80

UT WOS:A1985AUN3500043

ER

PT S

AU Agarwal, PC

Gupta, M

Dass, L

AF Agarwal, PC

Gupta, M

Dass, L

BE Chaturvedi, DK

Murch, GE

TI Bulk modulus and pseudopotential in metallic glasses

SO DISORDERED MATERIALS - CURRENT DEVELOPMENTS -

SE MATERIALS SCIENCE FORUM

CT International Seminar on Current Developments in Disordered Materials

(CDDM-96)

CY JAN, 1996

CL KURUKSHETRA, INDIA

SP Indian Soc Disordered Mat, Semiconductor Soc India, Kurukshetra Univ, Kurukshetra, Univ Grants Commiss, New Delhi, Dept Sci & Technol, New Delhi, Council Sci & Ind Res, New Delhi, Transtec Publicat, Switzerland

AB An expression derived for bulk modulus for metallic glass is used to calculate it for four metallic glassed using the Ashcroft's empty core model potential. The bulk modulus of these glassed is also computed using the force constant k(c) given by Bhatia and Singh. The results so obtained are found to be in good agreement with other available theoretical values.

The value of the bulk modulus is used alongwith mean atomic mass density rho and sound velocities of longitudinal [V-L (0)] and transverse [V-T(0)] waves in low momentum region to estimate remaining force constants beta and delta of the force constant model.

SN 0255-5476

BN 0-87849-737-4

PY 1996

VL 223

BP 99

EP 103

DI 10.4028/www.scientific.net/MSF.223-224.99

UT WOS:A1996BG37X00019

ER

PT J

AU RAY, R

AF RAY, R

TI BULK MICROCRYSTALLINE ALLOYS FROM METALLIC-GLASS POWDER

SO INTERNATIONAL JOURNAL OF POWDER METALLURGY

SN 0888-7462

PY 1982

VL 18

IS 3

BP 209

EP &

UT WOS:A1982NX05500001

ER

PT J

AU Loffler, JF

AF Loffler, JF

TI Bulk metallic glasses (vol 11, pg 529, 2003)

SO INTERMETALLICS

SN 0966-9795

PD OCT

PY 2003

VL 11

IS 10

BP 979

EP 979

DI 10.1016/j.intermet.2003.08.001

UT WOS:000186024000001

ER

PT J

AU Buchanan, O

AF Buchanan, O

TI Bulk metallic glasses used for manufacturing net-shape metal products

SO MRS BULLETIN

SN 0883-7694

PD NOV

PY 2002

VL 27

IS 11

BP 850

EP 851

UT WOS:000179299200016

ER

PT J

AU Telford, M

AF Telford, Mark

TI The case for bulk metallic glass

SO MATERIALS TODAY

SN 1369-7021

EI 1873-4103

PD MAR

PY 2004

VL 7

IS 3

BP 36

EP 43

DI 10.1016/S1369-7021(04)00124-5

UT WOS:000208931400021

ER

PT J

AU OELHAFEN, P

AF OELHAFEN, P

TI PHOTOELECTRON-SPECTROSCOPY OF METALLIC GLASSES - BULK, INTERFACE AND

SURFACE-PROPERTIES

SO MATERIALS SCIENCE AND ENGINEERING

SN 0025-5416

PD MAR

PY 1988

VL 99

BP 239

EP 244

DI 10.1016/0025-5416(88)90331-X

UT WOS:A1988N970700054

ER

PT J

AU Nieh, TG

AF Nieh, TG

TI Reflections on the Fourth International Conference on Bulk Metallic

Glasses

SO INTERMETALLICS

RI Nieh, Tai-Gang/G-5912-2011

OI Nieh, Tai-Gang/0000-0002-2814-3746; Liu, Chain Tsuan/0000-0001-7888-9725

SN 0966-9795

PD AUG-SEP

PY 2006

VL 14

IS 8-9

SI SI

BP 855

EP 856

DI 10.1016/j.intermet.2006.01.049

UT WOS:000237770600001

ER

PT J

AU Heilmaier, M

Eckert, J

AF Heilmaier, M

Eckert, J

TI Elevated temperature deformation behavior of Zr-based bulk metallic

glasses

SO ADVANCED ENGINEERING MATERIALS

SN 1438-1656

PD SEP

PY 2005

VL 7

IS 9

BP 833

EP 841

DI 10.1002/adem.200500080

UT WOS:000233059500009

ER

PT J

AU Inoue, A

AF Inoue, A

TI Special issue on bulk metallic glasses III - Preface

SO MATERIALS TRANSACTIONS

RI Inoue, Akihisa/E-5271-2015

SN 1345-9678

EI 1347-5320

PD APR

PY 2001

VL 42

IS 4

BP 547

EP 547

UT WOS:000168864100001

ER

PT B

AU Liu, CT

Greer, AL

Schuh, CA

AF Liu, C. T.

Greer, A. Lindsay

Schuh, Christopher A.

BA Suryanarayana, C

Inoue, A

BF Suryanarayana, C

Inoue, A

TI BULK METALLIC GLASSES Introduction

SO BULK METALLIC GLASSES

BN 978-1-4200-8596-9

PY 2011

BP 1

EP 10

D2 10.1201/9781420085976-1

UT WOS:000288438000002

ER

PT J

AU Chen, GL

Liu, CT

Chen, G

Lu, K

Huang, BY

Sass, SL

AF Chen, G. L.

Liu, C. T.

Chen, Guang

Lu, Ke

Huang, Baiyun

Sass, S. L.

TI Special issue - Advanced intermetallic alloys and bulk metallic glasses

- Preface

SO INTERMETALLICS

OI Liu, Chain Tsuan/0000-0001-7888-9725

SN 0966-9795

PD MAY-JUN

PY 2007

VL 15

IS 5-6

BP 615

EP 617

DI 10.1016/j.intermet.2006.10.001

UT WOS:000246739400001

ER

PT J

AU Risbud, A

AF Risbud, Aditi

TI Size matters in mechanical behavior of bulk metallic glasses

SO MRS BULLETIN

SN 0883-7694

EI 1938-1425

PD APR

PY 2017

VL 42

IS 4

BP 263

EP 264

DI 10.1557/mrs.2017.66

UT WOS:000399416200002

ER

PT J

AU Byrne, CJ

Eldrup, M

AF Byrne, Cormac J.

Eldrup, Morten

TI Materials science - Bulk metallic glasses

SO SCIENCE

SN 0036-8075

PD JUL 25

PY 2008

VL 321

IS 5888

BP 502

EP 503

DI 10.1126/science.1158864

UT WOS:000257888900030

PM 18653873

ER

PT J

AU Inoue, A

Saida, J

AF Inoue, A

Saida, J

TI Special issue on materials science of bulk metallic glasses - Prefacee

SO MATERIALS TRANSACTIONS

RI Saida, Junji/C-1912-2009; Inoue, Akihisa/E-5271-2015

SN 1345-9678

EI 1347-5320

PD DEC

PY 2005

VL 46

IS 12

BP 2745

EP 2745

UT WOS:000234846500036

ER

PT J

AU Thiel, PA

Heinzig, MW

Anderegg, JW

Wehner, BI

Sordelet, DJ

Glade, S

Johnson, WL

AF Thiel, PA

Heinzig, MW

Anderegg, JW

Wehner, BI

Sordelet, DJ

Glade, S

Johnson, WL

TI X-ray photoelectron spectroscopy studies of bulk metallic glasses.

SO ABSTRACTS OF PAPERS OF THE AMERICAN CHEMICAL SOCIETY

SN 0065-7727

PD MAR 26

PY 2000

VL 219

MA 98-PHYS

BP U282

EP U282

PN 2

UT WOS:000087246201557

ER

PT J

AU IVISON, PK

COWLAM, N

ZHONG, ZP

WILLIAMS, JM

AF IVISON, PK

COWLAM, N

ZHONG, ZP

WILLIAMS, JM

TI SURFACE AND BULK MAGNETISM IN FERROMAGNETIC METALLIC GLASSES

SO JOURNAL OF NON-CRYSTALLINE SOLIDS

CT 7TH INTERNATIONAL CONF ON LIQUID AND AMORPHOUS METALS

CY SEP 04-08, 1989

CL KYOTO, JAPAN

SP PHYS SOC JAPAN, INT UNION PURE & APPL PHYS, JAPAN SCI COUNCIL, JAPAN INST MET & IRON, STEEL INST JAPAN, COMMEMORAT ASSOC JAPAN WORLD EXPOSIT

SN 0022-3093

PD FEB

PY 1990

VL 117

BP 666

EP 669

DI 10.1016/0022-3093(90)90619-W

PN 2

UT WOS:A1990CW17800040

ER

PT J

AU NOVIKOV, VN

KOVAL, GM

AF NOVIKOV, VN

KOVAL, GM

TI THEORETICAL EVALUATION OF THE BULK ELASTIC NODULUS FOR METALLIC GLASSES

SO RUSSIAN METALLURGY

SN 0036-0295

PY 1988

IS 6

BP 146

EP 149

UT WOS:A1988AW15300030

ER

PT J

AU Inoue, A

Hirotsu, Y

Nieh, TG

Hono, K

AF Inoue, Akihisa

Hirotsu, Yoshihiko

Nieh, T. G.

Hono, K.

TI Special issue on Bulk Metallic Glasses - Selected papers from the Fifth

International Conference on Bulk Metallic Glasses (BMG-V)

SO MATERIALS TRANSACTIONS

RI Nieh, Tai-Gang/G-5912-2011; Inoue, Akihisa/E-5271-2015; Hono,

Kazuhiro/B-9202-2008

OI Nieh, Tai-Gang/0000-0002-2814-3746; Hono, Kazuhiro/0000-0001-7367-0193

SN 1345-9678

EI 1347-5320

PD JUL

PY 2007

VL 48

IS 7

BP 1579

EP 1579

UT WOS:000248743100001

ER

PT J

AU Nieh, TG

Liu, CT

Wang, WH

Pan, M

AF Nieh, TG

Liu, CT

Wang, WH

Pan, M

TI Reflections on the Third UEF Conference on Bulk Metallic Glasses

SO INTERMETALLICS

RI Nieh, Tai-Gang/G-5912-2011

OI Nieh, Tai-Gang/0000-0002-2814-3746

SN 0966-9795

PD OCT-NOV

PY 2004

VL 12

IS 10-11

BP 1033

EP 1033

DI 10.1016/j.internet.2004.07.035

UT WOS:000224566700001

ER

PT S

AU Aydiner, CC

Ustumdag, E

AF Aydiner, CC

Ustumdag, E

BE Denis, S

Hanabusa, T

He, BP

Mittemeijer, E

Nan, J

Noyan, IC

Scholtes, B

Tanaka, K

Xu, KW

TI Residual stresses in bulk metallic glasses due to thermal tempering

SO RESIDUAL STRESSES VII, PROCEEDINGS

SE MATERIALS SCIENCE FORUM

CT 7th International Conference onResidual Strsses (ICRS-7)

CY JUN 14-17, 2004

CL Xian, PEOPLES R CHINA

SP Chinese Mech Engn Soc, Xian Jiaotong Univ

RI Aydiner, Cahit/O-9618-2017

OI Aydiner, Cahit/0000-0001-8256-6742

SN 0255-5476

BN 0-87849-969-5

PY 2005

VL 490-491

BP 515

EP 520

DI 10.4028/www.scientific.net/MSF.490-491.515

UT WOS:000230305200089

ER

PT J

AU Cheng, YQ

Han, Z

Li, Y

Ma, E

AF Cheng, Y. Q.

Han, Z.

Li, Y.

Ma, E.

TI Cold versus hot shear banding in bulk metallic glass (vol 80, 134115,

2009)

SO PHYSICAL REVIEW B

SN 1098-0121

PD DEC

PY 2009

VL 80

IS 22

AR 229903

DI 10.1103/PhysRevB.80.229903

UT WOS:000273228500103

ER

PT J

AU Zhang, Y

Ji, YF

Zhao, DQ

Zhuang, YX

Wang, RJ

Pan, MX

Dong, YD

Wang, WH

AF Zhang, Y

Ji, YF

Zhao, DQ

Zhuang, YX

Wang, RJ

Pan, MX

Dong, YD

Wang, WH

TI Glass forming ability and properties of Zr/Nb-based bulk metallic

glasses

SO SCRIPTA MATERIALIA

RI ZHANG, Yong/B-7928-2009; Zhuang, Yanxin/F-7199-2011

OI ZHANG, Yong/0000-0002-6355-9923;

SN 1359-6462

PD APR 17

PY 2001

VL 44

IS 7

BP 1107

EP 1112

DI 10.1016/S1359-6462(00)00707-7

UT WOS:000168739400017

ER

PT J

AU Piccin, R

Tiberto, P

Ababei, G

Chiriac, H

Yavari, AR

Baricco, M

AF Piccin, R.

Tiberto, P.

Ababei, G.

Chiriac, H.

Yavari, A. R.

Baricco, M.

TI Effect of Nb and Y additions on glass formation and magnetic properties

in the Fe78B14Si8 Alloy

SO ADVANCED ENGINEERING MATERIALS

RI Ababei, Gabriel/C-4765-2011; Chiriac, Horia/C-4821-2011; Baricco,

Marcello/B-4075-2013; Tiberto, Paola Maria/M-8133-2015; yavari,

alain/E-8192-2010; Cuza, UAIC/D-2604-2009

OI Baricco, Marcello/0000-0002-2856-9894; Tiberto, Paola

Maria/0000-0002-5432-1788; Tiberto, Paola/0000-0002-7409-2281

SN 1438-1656

PD JUN

PY 2007

VL 9

IS 6

BP 480

EP 482

DI 10.1002/adem.200700042

UT WOS:000247885800011

ER

PT J

AU Ma, E

Xu, J

AF Ma, Evan

Xu, Jian

TI BIODEGRADABLE ALLOYS The glass window of opportunities

SO NATURE MATERIALS

RI Ma, En/A-3232-2010

SN 1476-1122

PD NOV

PY 2009

VL 8

IS 11

BP 855

EP 857

DI 10.1038/nmat2550

UT WOS:000271050500009

PM 19851321

ER

PT J

AU XU, YF

HUANG, XM

WANG, WK

AF XU, YF

HUANG, XM

WANG, WK

TI PREPARATION OF BULK METALLIC-GLASS PD40NI40P20 UNDER HIGH-PRESSURE

SO APPLIED PHYSICS LETTERS

SN 0003-6951

PD MAY 14

PY 1990

VL 56

IS 20

BP 1957

EP 1958

DI 10.1063/1.103227

UT WOS:A1990DC93500010

ER

PT J

AU Ding, D

Xia, L

Jo, CL

Dong, YD

AF Ding, D.

Xia, L.

Jo, C. L.

Dong, Y. D.

TI Glass forming ability of Gd55Al15Ni30 ternary alloy

SO JOURNAL OF MATERIALS SCIENCE

OI Xia, Lei/0000-0001-9198-1497

SN 0022-2461

PD AUG

PY 2006

VL 41

IS 18

BP 6112

EP 6115

DI 10.1007/s10853-006-0477-x

UT WOS:000240481800046

ER

PT J

AU [Anonymous]

AF [Anonymous]

TI Yale 'shapes up' nanomanufacturing with bulk metallic glass molds

SO AMERICAN CERAMIC SOCIETY BULLETIN

SN 0002-7812

PD MAR

PY 2009

VL 88

IS 3

BP 16

EP 17

UT WOS:000264172400004

ER

PT J

AU Watanabe, L

Conner, D

Johnson, WL

AF Watanabe, Lisa

Conner, Dale

Johnson, William L.

TI Palladium based bulk metallic glass in biomedical applications

SO ABSTRACTS OF PAPERS OF THE AMERICAN CHEMICAL SOCIETY

CT 241st National Meeting and Exposition of the American-Chemical-Society

(ACS)

CY MAR 27-31, 2011

CL Anaheim, CA

SP Amer Chem Soc

RI BAI, JIE/D-7448-2016

SN 0065-7727

PD MAR 27

PY 2011

VL 241

MA 1104-CHED

UT WOS:000291982801070

ER

PT S

AU Schroers, J

AF Schroers, Jan

BA Sarac, B

BF Sarac, B

TI Fabrication Methods of Artificial Microstructures

SO MICROSTRUCTURE-PROPERTY OPTIMIZATION IN METALLIC GLASSES

SE Springer Theses-Recognizing Outstanding PhD Research

SN 2190-5053

BN 978-3-319-13033-0; 978-3-319-13032-3

PY 2015

BP 17

EP 28

DI 10.1007/978-3-319-13033-0_2

D2 10.1007/978-3-319-13033-0

UT WOS:000372459200003

ER

PT B

AU Karmakar, B

AF Karmakar, Basudeb

BA Karmakar, B

BF Karmakar, B

TI Glasses and glass-ceramics for biomedical applications

SO FUNCTIONAL GLASSES AND GLASS-CERAMICS: PROCESSING, PROPERTIES, AND

APPLICATIONS

BN 978-0-12-805207-5; 978-0-12-805056-9

PY 2017

BP 253

EP 280

DI 10.1016/B978-0-12-805056-9.00007-6

UT WOS:000427863400009

ER

PT J

AU COMINS, JD

MACDONALD, JE

GIBBS, MRJ

SAUNDERS, GA

AF COMINS, JD

MACDONALD, JE

GIBBS, MRJ

SAUNDERS, GA

TI A SEARCH FOR RELAXATION IN A BULK PD40NI40P20 METALLIC-GLASS

SO JOURNAL OF PHYSICS F-METAL PHYSICS

OI Macdonald, John/0000-0001-5504-1692

SN 0305-4608

PD JAN

PY 1987

VL 17

IS 1

BP 19

EP 26

DI 10.1088/0305-4608/17/1/008

UT WOS:A1987F667200008

ER

PT J

AU Yannopoulos, SN

Johari, GP

AF Yannopoulos, Spyros N.

Johari, G. P.

TI Glass behaviour - Poisson's ratio and liquid's fragility

SO NATURE

RI Yannopoulos, Spyros/C-3909-2012

OI Yannopoulos, Spyros/0000-0001-6684-3172

SN 0028-0836

PD AUG 3

PY 2006

VL 442

IS 7102

BP E7

EP E8

DI 10.1038/nature04967

UT WOS:000239455900034

PM 16888850

ER

PT J

AU PAMPILLO, CA

CHEN, HS

AF PAMPILLO, CA

CHEN, HS

TI COMPREHENSIVE PLASTIC-DEFORMATION OF A BULK METALLIC GLASS

SO MATERIALS SCIENCE AND ENGINEERING

SN 0025-5416

PY 1974

VL 13

IS 2

BP 181

EP 188

DI 10.1016/0025-5416(74)90185-2

UT WOS:A1974R814900013

ER

PT J

AU Conner, RD

Rosakis, AJ

Johnson, WL

Owen, DM

AF Conner, RD

Rosakis, AJ

Johnson, WL

Owen, DM

TI Fracture toughness determination for a beryllium-bearing bulk metallic

glass

SO SCRIPTA MATERIALIA

SN 1359-6462

PD NOV 1

PY 1997

VL 37

IS 9

BP 1373

EP 1378

DI 10.1016/S1359-6462(97)00250-9

UT WOS:A1997YA84100016

ER

PT J

AU Fujita, K

Inoue, A

Zhang, A

AF Fujita, K

Inoue, A

Zhang, A

TI Fractography of fatigue crack propagation in a nanocrystalline Zr-based

bulk metallic glass

SO SCRIPTA MATERIALIA

CT 5th International Conference on Nanostructured Materials (NANO 2000)

CY AUG 20-25, 2000

CL SENDAI, JAPAN

SP Int Comm Nanostructrued Mt, Acta Materialia Inc, Inst Mat Res, Tohoku Univ, Inst Sci & Ind Res, Osaka Univ, Sendai City, Japan Soc Promot Sci, Iwatani Naoji Fdn

RI Inoue, Akihisa/E-5271-2015

SN 1359-6462

PD MAY 18

PY 2001

VL 44

IS 8-9

BP 1629

EP 1633

DI 10.1016/S1359-6462(01)00871-5

UT WOS:000169389400092

ER

PT S

AU Kawamura, Y

Ohno, Y

Chiba, A

AF Kawamura, Y

Ohno, Y

Chiba, A

BE Ma, E

Atzmon, M

Koch, CC

TI Development of welding technologies in bulk metallic glasses

SO METASTABLE, MECHANICALLY ALLOYED AND NANOCRYSTALLINE MATERIALS

SE MATERIALS SCIENCE FORUM

CT International Symposium on Metastable, Mechanically Alloyed and

Nanocrystalline Materials

CY JUN 24-29, 2001

CL UNIV MICHIGAN, ANN ARBOR, MICHIGAN

HO UNIV MICHIGAN

AB For establishment of metallurgical bonding technology of bulk metallic glasses, we have tried to weld Pd40Ni40P20 and Zr55Al10Ni5Cu30 bulk metallic glasses having a wide supercooled liquid region and high glass forming ability by three welding methods of friction, pulse-current and explosion We have succeeded in joining bulk metallic glasses to the same bulk metallic glasses or polycrystalline metallic materials. No crystallization was observed in the interface and heat-affected zones. No visible defect was recognized at the interface, showing an achievement of metallurgical bonding of bulk metallic glasses. The tensile strength of the welded bulk metallic glasses was the same as that of the parent bulk metallic glasses.

SN 0255-5476

BN 0-87849-892-3

PY 2002

VL 386-3

BP 553

EP 558

DI 10.4028/www.scientific.net/MSF.386-388.553

UT WOS:000174657900083

ER

PT B

AU Ananthanarayanan, A

Goswami, M

Kothiyal, GP

AF Ananthanarayanan, A.

Goswami, M.

Kothiyal, G. P.

BE Tyagi, AK

Banerjee, S

TI GLASSES AND GLASS-CERAMICS FOR VACUUM AND HIGH-TEMPERATURE APPLICATIONS

SO MATERIALS UNDER EXTREME CONDITIONS: RECENT TRENDS AND FUTURE PROSPECTS

BN 978-0-12-801442-4; 978-0-12-801300-7

PY 2017

BP 195

EP 233

DI 10.1016/B978-0-12-801300-7.00006-1

UT WOS:000417083300007

ER

PT J

AU Khanna, R

AF Khanna, Rohit

TI Slithering of shear bands can improve plasticity in bulk metallic

glasses

SO MRS BULLETIN

SN 0883-7694

PD MAR

PY 2008

VL 33

IS 3

BP 166

EP 167

DI 10.1557/mrs2008.40

UT WOS:000254230900006

ER

PT J

AU Kim, DH

Kim, WT

Nieh, TG

Liu, CT

AF Kim, D. H.

Kim, W. T.

Nieh, T. G.

Liu, C. T.

TI SPECIAL ISSUE The 7th International Conference on Bulk Metallic Glasses

Preface

SO INTERMETALLICS

RI bang, changwook/J-7922-2012; Kim, Do Hyang/J-6575-2012; Nieh,

Tai-Gang/G-5912-2011

OI Nieh, Tai-Gang/0000-0002-2814-3746

SN 0966-9795

PD OCT

PY 2010

VL 18

IS 10

SI SI

BP 1795

EP 1795

DI 10.1016/j.intermet.2010.07.017

UT WOS:000281420700001

ER

PT J

AU Saida, J

Inoue, A

AF Saida, Junji

Inoue, Akihisa

TI Special issue on materials science of bulk metallic Glasses-VII -

Preface

SO MATERIALS TRANSACTIONS

RI Saida, Junji/C-1912-2009

SN 1345-9678

EI 1347-5320

PD JUN

PY 2007

VL 48

IS 6

BP 1260

EP 1260

UT WOS:000247823300018

ER

PT J

AU Kovneristyi, YK

Pozdnyakov, VA

AF Kovneristyi, YK

Pozdnyakov, VA

TI Structural mechanisms of plastic strain in bulk-amorphous metallic

alloys

SO DOKLADY PHYSICS

SN 1028-3358

EI 1562-6903

PD AUG

PY 2005

VL 50

IS 8

BP 409

EP 413

DI 10.1134/1.2039981

UT WOS:000231897600006

ER

PT J

AU Chen, LS

Zhang, JZ

Wen, L

Yu, P

Xia, L

AF Chen, LanSheng

Zhang, JiaZheng

Wen, Lin

Yu, Peng

Xia, Lei

TI Outstanding magnetocaloric effect of Fe88-xZr8B4Smx (x=0, 1, 2, 3)

amorphous alloys

SO SCIENCE CHINA-PHYSICS MECHANICS & ASTRONOMY

SN 1674-7348

EI 1869-1927

PD MAY

PY 2018

VL 61

IS 5

AR 056121

DI 10.1007/s11433-017-9152-7

UT WOS:000428503800006

ER

PT B

AU Qin, FX

Dan, ZH

Wang, XM

Xie, GQ

Inoue, A

AF Qin, Fengxiang

Dan, Zhenhua

Wang, Xinmin

Xie, Guoqiang

Inoue, Akihisa

BE Laskovski, AN

TI Ti-based Bulk Metallic Glasses for Biomedical Applications

SO BIOMEDICAL ENGINEERING, TRENDS IN MATERIALS SCIENCE

RI Xie, Guoqiang/A-8619-2011

BN 978-953-307-513-6

PY 2011

BP 249

EP 268

D2 10.5772/992

UT WOS:000371133400012

ER

PT J

AU Ohnuma, M

Pryds, NH

Linderoth, S

Eldrup, M

Pedersen, AS

Pedersen, JS

AF Ohnuma, M

Pryds, NH

Linderoth, S

Eldrup, M

Pedersen, AS

Pedersen, JS

TI Bulk amorphous (Mg0.98Al0.02)(60)Cu30Y10 alloy

SO SCRIPTA MATERIALIA

RI Pedersen, Jan/A-8346-2008

OI Pedersen, Jan/0000-0002-7768-0206; Pedersen, Allan

Schroder/0000-0003-3709-3194; Linderoth, Soren/0000-0001-9863-6290;

Pryds, Nini/0000-0002-5718-7924

SN 1359-6462

PD SEP 10

PY 1999

VL 41

IS 8

BP 889

EP 893

DI 10.1016/S1359-6462(99)00225-0

UT WOS:000082838300016

ER

PT J

AU Wang, H

AF Wang, H

TI Bulk metallic glass composites

SO JOURNAL OF MATERIALS SCIENCE & TECHNOLOGY

CT Australia-China Materials Science Symposium

CY OCT 22-25, 2004

CL Univ Queensland, Sch Engn, Brisbane, AUSTRALIA

HO Univ Queensland, Sch Engn

AB Metallic glasses have attracted considerable attention due to their unique properties. The recent discovery of new glass-forming compositions makes it possible to produce metallic glasses in bulk shapes. Bulk metallic glasses offer an opportunity to revolutionize the field of structural materials with combinations of strength, elastic limit, toughness, wear resistance and corrosion resistance. The main current technical barrier is their limited ductility. Forming a composite containing ductile crystal phase in a bulk metallic glass matrix has been proven as an effective approach to increase their ductility. Three types of bulk metallic glass composites are discussed: extrinsic composites, in-situ composites and nanocrystalline composites. The paper also lists the key issues in the development of bulk metallic glass composites.

SN 1005-0302

PD JUN

PY 2005

VL 21

SU 1

BP 86

EP 90

UT WOS:000230317900022

ER

PT J

AU Chauhan, A

AF Chauhan, Aditya

TI An Assessment of Bulk Metallic Glasses for Microelectromechanical System

Devices (vol 4, pg 231, 2013)

SO INTERNATIONAL JOURNAL OF APPLIED GLASS SCIENCE

SN 2041-1286

EI 2041-1294

PD MAR

PY 2014

VL 5

IS 1

SI SI

BP 89

EP 89

DI 10.1111/ijag.12064

UT WOS:000332163300010

ER

PT J

AU Dmowski, W

Egami, T

AF Dmowski, Wojtek

Egami, Takeshi

TI Structural Anisotropy in Metallic Glasses Induced by Mechanical

Deformation

SO ADVANCED ENGINEERING MATERIALS

SN 1438-1656

EI 1527-2648

PD NOV

PY 2008

VL 10

IS 11

BP 1003

EP 1007

DI 10.1002/adem.200800121

UT WOS:000261933400003

ER

PT J

AU Tamura, T

Amiya, K

Rachmat, RS

Mizutani, Y

Miwa, K

AF Tamura, T

Amiya, K

Rachmat, RS

Mizutani, Y

Miwa, K

TI Electromagnetic vibration process for producing bulk metallic glasses

SO NATURE MATERIALS

RI Amiya, Kenji/P-6132-2014; Tamura, Takuya/D-4854-2017

OI Tamura, Takuya/0000-0001-6083-913X

SN 1476-1122

PD APR

PY 2005

VL 4

IS 4

BP 289

EP 292

DI 10.1038/nmat1341

UT WOS:000228075400014

PM 15750599

ER

PT J

AU Qiao, JC

Pelletier, JM

Casalini, R

AF Qiao, Jichao

Pelletier, Jean-Marc

Casalini, Riccardo

TI Relaxation of Bulk Metallic Glasses Studied by Mechanical Spectroscopy

(vol 117, pg 13658, 2013)

SO JOURNAL OF PHYSICAL CHEMISTRY B

SN 1520-6106

PD JAN 16

PY 2014

VL 118

IS 2

BP 648

EP 648

DI 10.1021/jp4120404

UT WOS:000330017900028

ER

PT J

AU Tao, PJ

Tu, Q

Zhang, WW

Li, DY

AF Tao, P. J.

Tu, Q.

Zhang, W. W.

Li, D. Y.

TI INVESTIGATION FOR BIOCOMPATIBILITY OF ZIRCONIUM-BASED BULK METALLIC

GLASSES WITH AND WITHOUT NI ELEMENT ADDITION

SO BASIC & CLINICAL PHARMACOLOGY & TOXICOLOGY

SN 1742-7835

EI 1742-7843

PD OCT

PY 2016

VL 119

SU 2

SI SI

MA 019

BP 10

EP 10

UT WOS:000385818700020

ER

PT J

AU Liaw, PK

Wang, GY

Schneider, J

AF Liaw, Peter K.

Wang, Gongyao

Schneider, Judy

TI Bulk Metallic Glasses: Overcoming the Challenges to Widespread

Applications

SO JOM

SN 1047-4838

PD FEB

PY 2010

VL 62

IS 2

BP 69

EP 69

DI 10.1007/s11837-010-0035-5

UT WOS:000274211000013

ER

PT B

AU Liu, CT

Greer, AL

Schuh, CA

AF Liu, C. T.

Greer, A. Lindsay

Schuh, Christopher A.

BA Suryanarayana, C

Inoue, A

BF Suryanarayana, C

Inoue, A

TI Applications

SO BULK METALLIC GLASSES

BN 978-1-4200-8596-9

PY 2011

BP 481

EP 515

D2 10.1201/9781420085976-1

UT WOS:000288438000011

ER

PT J

AU Yu, HB

Wang, WH

Zhang, JL

Shek, CH

Bai, HY

AF Yu, Hai Bin

Wang, Wei Hua

Zhang, Ji Liang

Shek, C. Hong

Bai, Hai Yang

TI Statistic Analysis of the Mechanical Behavior of Bulk Metallic Glasses

SO ADVANCED ENGINEERING MATERIALS

RI SHEK, Chan Hung/J-3857-2015; Yu, Hai Bin/E-5312-2010; zhang,

jiliang/N-5659-2015

OI SHEK, Chan Hung/0000-0002-6870-523X; Yu, Hai Bin/0000-0003-0645-0187;

SN 1438-1656

PD MAY

PY 2009

VL 11

IS 5

BP 370

EP 373

DI 10.1002/adem.200800380

UT WOS:000266474300006

ER

PT J

AU Senkov, ON

Scott, JM

Miracle, DB

AF Senkov, Oleg N.

Scott, James M.

Miracle, Daniel B.

TI Development of low density Ca-Mg-Al-based bulk metallic glasses (vol 48,

pg 1870, 2007)

SO MATERIALS TRANSACTIONS

OI Senkov, Oleg/0000-0002-9336-3702

SN 1345-9678

EI 1347-5320

PD AUG

PY 2007

VL 48

IS 8

BP 2258

EP 2258

UT WOS:000249511800052

ER

PT J

AU Chen, N

Xu, LM

Shao, Y

Georgarakis, K

AF Chen, Na

Xu, Limei

Shao, Yang

Georgarakis, Konstantinos

TI Experimental and Theoretical Advances in Amorphous Alloys

SO ADVANCES IN MATERIALS SCIENCE AND ENGINEERING

RI Xu, Limei/J-6150-2012; Georgarakis, Konstantinos/K-1939-2015; Chen,

Na/A-4120-2010; Georgarakis, Konstantinos/E-6390-2010

OI Georgarakis, Konstantinos/0000-0003-0918-7310; Shao,

Yang/0000-0001-5369-9933

SN 1687-8434

EI 1687-8442

PY 2014

AR 514104

DI 10.1155/2014/514104

UT WOS:000347821900001

ER

PT J

AU Gludovatz, B

Naleway, SE

Ritchie, RO

Kruzic, JJ

AF Gludovatz, Bernd

Naleway, Steven E.

Ritchie, Robert O.

Kruzic, Jamie J.

TI Size-dependent fracture toughness of bulk metallic glasses (vol 70, pg

198, 2014)

SO ACTA MATERIALIA

RI Ritchie, Robert/A-8066-2008; Kruzic, Jamie/M-3558-2014

OI Ritchie, Robert/0000-0002-0501-6998; Kruzic, Jamie/0000-0002-9695-1921;

Gludovatz, Bernd/0000-0002-2420-3879

SN 1359-6454

EI 1873-2453

PD NOV

PY 2014

VL 80

BP 507

EP 507

DI 10.1016/j.actamat.2014.04.001

UT WOS:000344208300047

ER

PT J

AU Floyd, M

Demetriou, MD

Johnson, WL

AF Floyd, Michael

Demetriou, Marios D.

Johnson, William L.

TI Optimizing glass formation in ferromagnetic alloys through chemical

fluxing

SO SCRIPTA MATERIALIA

SN 1359-6462

PD MAR 15

PY 2018

VL 146

BP 312

EP 315

DI 10.1016/j.scriptamat.2017.12.013

UT WOS:000424719400069

ER

PT J

AU RAY, R

AF RAY, R

TI BULK MICROCRYSTALLINE ALLOYS PREPARED FROM METALLIC GLASSES - A NOVEL

MATERIALS TECHNOLOGY

SO MATERIALS SCIENCE AND ENGINEERING

SN 0025-5416

PY 1982

VL 52

IS 1

BP 85

EP 87

DI 10.1016/0025-5416(82)90072-6

UT WOS:A1982NB85600006

ER

PT J

AU Sun, YJ

Sun, YL

Wu, RG

AF Sun Yajuan

Sun Yongli

Wu Riga

TI Influence of Thermodynamic and Kinetic Factors on the Glass Forming

Ability of Bulk Metallic Glasses

SO RARE METAL MATERIALS AND ENGINEERING

AB The forming mechanisms of the bulk metallic glasses (BMGs) were investigated from the viewpoints of thermodynamics and kinetics combined with the classical theory of nucleation and growth. The influences of the enthalpy change Delta H(f), entropy change Delta S(f), Gibbs free energy difference Delta G, melt viscosity eta and cooling rate on the glass forming ability of bulk metallic glasses and their relationships were expatiated. In addition, some criteria of BMGs' glass forming ability were summarized.

SN 1002-185X

PD DEC

PY 2010

VL 39

IS 12

BP 2157

EP 2160

UT WOS:000286546800019

ER

PT J

AU Kato, H

Hirano, T

Matsuo, A

Kawamura, Y

Inoue, A

AF Kato, H

Hirano, T

Matsuo, A

Kawamura, Y

Inoue, A

TI High strength and good ductility of Zr55Al10Ni5Cu30 bulk glass

containing ZRC particles

SO SCRIPTA MATERIALIA

RI Inoue, Akihisa/E-5271-2015; Kato, Hidemi/B-2492-2015

SN 1359-6462

PD AUG 28

PY 2000

VL 43

IS 6

BP 503

EP 507

DI 10.1016/S1359-6462(00)00452-8

UT WOS:000089373000005

ER

PT J

AU Shoji, T

Kawamura, Y

Ohno, Y

AF Shoji, T

Kawamura, Y

Ohno, Y

TI Joining of Zr41Be23Ti14Cu12Ni10 bulk metallic glasses by a friction

welding method

SO MATERIALS TRANSACTIONS

AB In order to establish metallurgical bonding technology of bulk metallic glasses, friction welding of Zr41Be23Ti14Cu12Ni10 bulk metallic glass with a wide supercooled liquid region and high glass forming ability has been tried. The Zr41Be23Ti14Cu12Ni10 bulk metallic glass has successfully welded to the same bulk metallic glass together. Moreover, the effects of friction-welding conditions such as friction time, rotational speed and upsetting pressure have been investigated under a wide range of conditions, no crystallization and no defects were observed in the inter-face.

SN 1345-9678

EI 1347-5320

PD SEP

PY 2003

VL 44

IS 9

BP 1809

EP 1816

DI 10.2320/matertrans.44.1809

UT WOS:000186013100030

ER

PT J

AU Kawamura, Y

Ohno, Y

AF Kawamura, Y

Ohno, Y

TI Metallurgical bonding of bulk metallic glasses

SO MATERIALS TRANSACTIONS

AB In order to establish metallurgical bonding technology of bulk metallic glasses, we have tried to weld Pd40Ni40P20 and Zr55Al10Ni5Cu30 bulk metallic glasses having a wide supercooled liquid region and high glass forming ability by two welding methods of friction and pulse-current. We have for the first time succeeded in joining bulk metallic glasses by both methods. No crystallization was observed in the interface and heat-affected zones. No visible defect was recognized at the interface, showing an achievement of metallurgical bonding of bulk metallic glasses. The tensile strength of the welded bulk metallic glasses was the same as that of the parent bulk metallic glasses.

SN 1345-9678

EI 1347-5320

PD APR

PY 2001

VL 42

IS 4

BP 717

EP 719

DI 10.2320/matertrans.42.717

UT WOS:000168864100033

ER

PT J

AU Wunderlich, RK

Vaillant, ML

Caron, A

Fecht, HJ

AF Wunderlich, Rainer K.

Vaillant, Marie-Laure

Caron, Arnaud

Fecht, Hans-Joerg

TI Glass-Forming Ability and Ductility of Zr-Based and Al-Rich Bulk

Metallic Glasses

SO ADVANCED ENGINEERING MATERIALS

RI Caron, Arnaud/B-6463-2010

OI Caron, Arnaud/0000-0003-0985-7441

SN 1438-1656

PD NOV

PY 2008

VL 10

IS 11

BP 1020

EP 1025

DI 10.1002/adem.200800183

UT WOS:000261933400006

ER

PT J

AU Sun, BA

Cheung, KP

Fan, JT

Lu, J

Wang, WH

AF Sun, B. A.

Cheung, K. P.

Fan, J. T.

Lu, J.

Wang, W. H.

TI Fiber metallic glass laminates

SO JOURNAL OF MATERIALS RESEARCH

AB The fabrication and properties of fiber metallic glass laminates (FMGL) composite composed of Al-based metallic glasses ribbons and fiber/epoxy layers were reported The metallic glass composite possesses structural features of low density and high specific strength compared to Al-based metallic glass and crystalline Al alloys The material shows pronounced tensile ductility compared to monolithic bulk metallic glasses

RI Sun, Baoan/C-6441-2012; CHEUNG, Ka Po/H-9184-2012

OI Sun, Baoan/0000-0001-5306-1817;

SN 0884-2914

PD DEC

PY 2010

VL 25

IS 12

BP 2287

EP 2291

DI 10.1557/JMR.2010.0291

UT WOS:000285073200005

ER

PT J

AU Orveillon, G

Senkov, ON

Soubeyroux, JL

Chevalier, B

Gorsse, S

AF Orveillon, Glenn

Senkov, Oleg N.

Soubeyroux, Jean-Louis

Chevalier, Bernard

Gorsse, Stephane

TI Composition selection and glass forming ability of Ce-based amorphous

alloys

SO ADVANCED ENGINEERING MATERIALS

RI Gorsse, Stephane/F-5170-2017; Senkov, Oleg/C-7197-2012

OI Gorsse, Stephane/0000-0003-1966-8476; Senkov, Oleg/0000-0001-5587-415X;

Senkov, Oleg/0000-0002-9336-3702

SN 1438-1656

PD JUN

PY 2007

VL 9

IS 6

BP 483

EP 486

DI 10.1002/adem.200700046

UT WOS:000247885800012

ER

PT J

AU Ortega-Hertogs, RJ

Inoue, A

Rao, KV

AF Ortega-Hertogs, RJ

Inoue, A

Rao, KV

TI Evolution from random-axis ising to Stoner-Wohlfarth type of hysteresis

loops of the cluster glass Fe3Nd phase in bulk glassy Nd60Fe30Al10 hard

magnets

SO SCRIPTA MATERIALIA

CT 5th International Conference on Nanostructured Materials (NANO 2000)

CY AUG 20-25, 2000

CL SENDAI, JAPAN

SP Int Comm Nanostructrued Mt, Acta Materialia Inc, Inst Mat Res, Tohoku Univ, Inst Sci & Ind Res, Osaka Univ, Sendai City, Japan Soc Promot Sci, Iwatani Naoji Fdn

RI Rao, K.V./F-4577-2011; Inoue, Akihisa/E-5271-2015

SN 1359-6462

PD MAY 18

PY 2001

VL 44

IS 8-9

BP 1333

EP 1336

DI 10.1016/S1359-6462(01)00706-0

UT WOS:000169389400032

ER

PT J

AU Sekol, RC

Kumar, G

Carmo, M

Gittleson, F

Hardesty-Dyck, N

Mukherjee, S

Schroers, J

Taylor, AD

AF Sekol, Ryan C.

Kumar, Golden

Carmo, Marcelo

Gittleson, Forrest

Hardesty-Dyck, Nathan

Mukherjee, Sundeep

Schroers, Jan

Taylor, Andre D.

TI Bulk Metallic Glass Micro Fuel Cell

SO SMALL

RI Mukherjee, Sundeep/N-5247-2014; kumar, golden/F-5443-2010

OI gittleson, forrest/0000-0003-0360-8348

SN 1613-6810

PD JUN 24

PY 2013

VL 9

IS 12

BP 2081

EP 2085

DI 10.1002/smll.201201647

UT WOS:000320401800008

PM 23184888

ER

PT J

AU Eckert, J

Kuhn, U

Mattern, N

Reger-Leonhard, A

Heilmaier, M

AF Eckert, J

Kuhn, U

Mattern, N

Reger-Leonhard, A

Heilmaier, M

TI Bulk nanostructured Zr-based multiphase alloys with high strength and

good ductility

SO SCRIPTA MATERIALIA

CT 5th International Conference on Nanostructured Materials (NANO 2000)

CY AUG 20-25, 2000

CL SENDAI, JAPAN

SP Int Comm Nanostructrued Mt, Acta Materialia Inc, Inst Mat Res, Tohoku Univ, Inst Sci & Ind Res, Osaka Univ, Sendai City, Japan Soc Promot Sci, Iwatani Naoji Fdn

SN 1359-6462

PD MAY 18

PY 2001

VL 44

IS 8-9

BP 1587

EP 1590

DI 10.1016/S1359-6462(01)00779-5

UT WOS:000169389400083

ER

PT S

AU Tang, YJ

Xu, H

Tan, XH

Man, H

Bai, Q

AF Tang, YongJun

Xu, Hui

Tan, XiaoHua

Man, Hua

Bai, Qin

BE Xu, B

Li, HY

TI Study on glass forming ability for the Nd60-xCo15+xAl25 alloys

SO ADVANCED COMPOSITE MATERIALS AND MANUFACTURING ENGINEERING

SE Advanced Materials Research

CT International Conference on Advanced Composite Materials and

Manufacturing Engineering (CMME 2012)

CY OCT 13-14, 2012

CL Beijing, PEOPLES R CHINA

SP Wuhan Inst Technol, Beijing Mat Res Ctr, Int Mat Res Soc

AB Bulk Nd60-xCo15+xAl25 (where x =0, 2, 5, 8, 11) sheet alloys were prepared by argon arc melting and suction casting a copper mold. Glassing forming ability (GFA) of these alloys was investigated by X-ray diffraction (XRD) and differential scanning calorimetry (DSC). Due to the dissimilarity reflected from DSC curves the thermodynamic calculation was applied. The values of Gibbs free energy (Delta G(1-x)(T-g)) for the amorphous alloys were gave out and some GFA criterions were adopted to make a comparison among the samples.

SN 1022-6680

BN 978-3-03785-522-5

PY 2012

VL 583

BP 82

EP 85

DI 10.4028/www.scientific.net/AMR.583.82

UT WOS:000317630500018

ER

PT J

AU Shoji, T

Kawamura, Y

Ohno, Y

AF Shoji, T

Kawamura, Y

Ohno, Y

TI Joining of Zr41Be23Ti14Cu12Ni10 bulk metallic glasses by a friction

welding method

SO JOURNAL OF THE JAPAN INSTITUTE OF METALS

AB In order to establish metallurgical bonding technology of bulk metallic glasses, welding of Zr41Be23Ti14Cu12Ni10 bulk metallic glasses with a wide supercooled liquid region and high glass forming ability has been tried by friction welding. The Zr41Be23Ti14 Cu12Ni10 bulk metallic glasses have successfully welded to the same bulk metallic glasses together. Under a wide range of conditions, no crystallization and no visible defects were observed in the interface. The tensile strength of the welded bulk metallic glasses was the same as that of the parent ones. Moreover, the effects of friction-welding conditions such as friction time, rotational speed and upsetting pressure have been investigated.

SN 0021-4876

EI 1880-6880

PD OCT

PY 2002

VL 66

IS 10

BP 1055

EP 1062

DI 10.2320/jinstmet1952.66.10_1055

UT WOS:000179202400012

ER

PT J

AU Reger-Leonhard, A

Heilmaier, M

Eckert, J

AF Reger-Leonhard, A

Heilmaier, M

Eckert, J

TI Newtonian flow of Zr(55)Cu(30)Al(10)Ni(5) bulk metallic glassy alloys

SO SCRIPTA MATERIALIA

SN 1359-6462

PD AUG 14

PY 2000

VL 43

IS 5

BP 459

EP 464

DI 10.1016/S1359-6462(00)00417-6

UT WOS:000089195100013

ER

PT J

AU Wang, JG

Zhao, DQ

Pan, MX

Shek, CH

Wang, WH

AF Wang, J. G.

Zhao, D. Q.

Pan, M. X.

Shek, C. H.

Wang, W. H.

TI Mechanical heterogeneity and mechanism of plasticity in metallic glasses

SO APPLIED PHYSICS LETTERS

AB The mechanical heterogeneity is quantified based on the spatial nanohardness distributions in three bulk metallic glasses with different plasticities. It is found that the metallic glass with high mechanical heterogeneity is more plastic. We propose that the appropriate mechanical heterogeneity makes the metallic glasses meliorate their plasticity by increasing inelastic strained area and promoting energy dissipation.

RI SHEK, Chan Hung/J-3857-2015

OI SHEK, Chan Hung/0000-0002-6870-523X

SN 0003-6951

PD JAN 19

PY 2009

VL 94

IS 3

AR 031904

DI 10.1063/1.3073985

UT WOS:000262724000011

ER

PT J

AU Zhang, HF

Ding, BZ

Hu, ZQ

AF Zhang, HF

Ding, BZ

Hu, ZQ

TI Investigations and progresses on bulk metal glasses

SO ACTA METALLURGICA SINICA

AB Some brief reviews about the history and background of the bulk metallic glasses are given, and several different preparation methods and forming ability on bulk metallic glasses are introdused. Furthermore, some basic researchs on the mechanical properties and other physical properties, some selected applications and future aspects of bulk metallic glasses and its composites have also been discussed.

SN 0412-1961

PD NOV 18

PY 2001

VL 37

IS 11

BP 1131

EP 1141

UT WOS:000172839900002

ER

PT J

AU Kawamura, Y

Ohno, Y

AF Kawamura, Y

Ohno, Y

TI Spark welding of Zr55Al10Ni5Cu30 bulk metallic glasses

SO SCRIPTA MATERIALIA

AB We have succeeded in welding of Zr55Al10Ni5Cu30 bulk metallic glasses using a spark welding method for the first time. No crystallization was observed in the welded and heat-affected zones and no visible interface was recognized. The tensile strength of the welded bulk metallic glasses was the same as that of the parent bulk metallic glass. (C) 2001 Acta Materialia Inc. Published by Elsevier Science Ltd. All rights reserved.

SN 1359-6462

PD JUL 31

PY 2001

VL 45

IS 2

BP 127

EP 132

DI 10.1016/S1359-6462(01)01003-X

UT WOS:000170899700001

ER

PT J

AU Yu, HB

Yu, P

Bai, HY

AF Yu, H. B.

Yu, P.

Bai, H. Y.

TI Lutetium and thulium based rare earth bulk metallic glasses

SO JOURNAL OF NON-CRYSTALLINE SOLIDS

AB We report the formation of lutetium and thulium based bulk metallic glasses based on the correlations between the thermodynamic, kinetic, elastic and other properties of metallic glasses. The two novel rare earth based bulk metallic glasses (REBMGs) exhibit excellent glass formation ability, high elastic moduli, considerable smaller Poisson's ratio, high thermal stability, and even higher mechanical strength than that of typical high strength Zr-based BMGs. The reasons for the properties of the Lu- and Tm-based and other REBMGs are discussed. (c) 2008 Elsevier B.V. All rights reserved.

RI Yu, Hai Bin/E-5312-2010

OI Yu, Hai Bin/0000-0003-0645-0187

SN 0022-3093

EI 1873-4812

PD OCT 15

PY 2008

VL 354

IS 40-41

BP 4539

EP 4542

DI 10.1016/j.jnoncrysol.2008.07.012

UT WOS:000260232600003

ER

PT J

AU Lu, ZP

Tan, H

Li, Y

Ng, SC

AF Lu, ZP

Tan, H

Li, Y

Ng, SC

TI The correlation between reduced glass transition temperature and glass

forming ability of bulk metallic glasses

SO SCRIPTA MATERIALIA

RI Lu, Zhao-Ping/A-2718-2009

OI Lu, Zhao-Ping/0000-0003-1463-8948

SN 1359-6462

PD MAR 17

PY 2000

VL 42

IS 7

BP 667

EP 673

DI 10.1016/S1359-6462(99)00417-0

UT WOS:000086283300007

ER

PT J

AU He, G

Bian, Z

Chen, GL

AF He, G

Bian, Z

Chen, GL

TI Fracture features of a Zr-base bulk glassy alloy

SO JOURNAL OF MATERIALS SCIENCE LETTERS

SN 0261-8028

PY 2001

VL 20

IS 7

BP 633

EP 636

DI 10.1023/A:1010973418202

UT WOS:000168738000014

ER

PT J

AU Wang, AP

Wang, JQ

AF Wang, A. P.

Wang, J. Q.

TI A topological approach to design Ni-based bulk metallic glasses with

high corrosion resistance

SO JOURNAL OF MATERIALS RESEARCH

AB The structural model for metallic glasses and the topological instability criterion for multicomponent alloy systems have been combined to formulate a design protocol for bulk metallic glasses (BMGs). New Ni-Nb-Zr bulk metallic glasses with high corrosion resistance have been discovered. The experimental results in literature also support the use of this approach as a practically efficient method to select bulk metallic glass-forming compositions.

SN 0884-2914

EI 2044-5326

PD JAN

PY 2007

VL 22

IS 1

BP 1

EP 4

DI 10.1557/JMR.2007.0020

UT WOS:000243463400001

ER

PT S

AU Yan, WY

Tilvawala, G

Kan, QH

AF Yan, Wenyi

Tilvawala, Gopesh

Kan, Qianhua

BE Leng, J

BarCohen, Y

Lee, I

Lu, J

TI Numerical investigation of the mechanical behaviour of shape memory bulk

metallic glass composites

SO THIRD INTERNATIONAL CONFERENCE ON SMART MATERIALS AND NANOTECHNOLOGY IN

ENGINEERING

SE Proceedings of SPIE

CT 3rd International Conference on Smart Materials and Nanotechnology in

Engineering

CY DEC 05-08, 2011

CL Shenzhen, PEOPLES R CHINA

SP Harbin Inst Technol, Natl Inst Stand & Technol, Asia Pacific Comm Smart & Nano Mat, Nanyang Technol Univ, Adv Mat Res Ctr, Natl Nat Sci Fdn China, Chinese Soc Composite Mat, Chinese Mat Res Soc, Chinese Soc Aeronaut & Astronaut, Chinese Soc Theoret & Appl Mech (CSTAM)

AB Bulk metallic glasses are a new type of advanced materials. They are characterized by their topologically disordered atomic structures. The lack of long-range translational symmetry in the atomic arrangement in bulk metallic glasses contributes to a range of unique and outstanding mechanical properties. For example, the yield strength of metallic glasses can be as large as twice that of corresponding crystalline alloys. However, the major issue to hinder metallic glasses application is the apparent brittleness. Unlike crystalline alloys, metallic glasses show abrupt failure with zero macroscopic plasticity. Various methods are being investigated in material engineering to improve the plasticity of bulk metallic glasses. The most recent progress is to develop shape memory bulk metallic glass composites, a combination of metallic glass and shape memory alloy. The large stress-induced transformation strain in shape memory alloy leads to the increase in the plasticity of the new composite material. The stress-strain behaviour of shape memory bulk metallic glass composites was investigated in this paper by using the finite element method. A unit cell model, which includes shape memory alloy phase and metallic glass phase, under uniaxial tension were numerically simulated. The effects of phase volume fraction, transformation stress and strain on the stress-strain behaviour of this new composite material were examined in this research.

RI Yan, Wenyi/H-3899-2011; kan, qianhua/J-7356-2012

OI Yan, Wenyi/0000-0001-9006-6270; kan, qianhua/0000-0002-5190-7804

SN 0277-786X

BN 978-0-8194-9087-2

PY 2012

VL 8409

AR 84090E

DI 10.1117/12.920949

UT WOS:000303519900013

ER

PT B

AU Nekouie, V

Roy, A

Silberschmidt, VV

AF Nekouie, Vahid

Roy, Anish

Silberschmidt, Vadim V.

BE Tiwari, A

Natarajan, S

TI Nanoindentation in Metallic Glasses

SO APPLIED NANOINDENTATION IN ADVANCED MATERIALS

OI Silberschmidt, Vadim V./0000-0003-3338-3311

BN 978-1-119-08452-5; 978-1-119-08449-5

PY 2017

BP 287

EP 311

D2 10.1002/9781119084501

UT WOS:000424147500014

ER

PT J

AU Kuball, A

Gross, O

Bochtler, B

Busch, R

AF Kuball, Alexander

Gross, Oliver

Bochtler, Benedikt

Busch, Ralf

TI Sulfur-bearing metallic glasses: A new family of bulk glass-forming

alloys

SO SCRIPTA MATERIALIA

AB Metallic glasses constitute a class of engineering materials having an enormous potential for many fields of application due to their superior properties. Here, we report on a new family of sulfur-bearing bulk metallic glasses. So far, sulfur was not considered as alloying element for the synthesis of bulk metallic glasses. We observe bulk glass formation in a variety of sulfur-containing systems, including titanium-based bulk glass-forming systems with an extremely high titanium content of 70 at.%. These findings allow the development of a whole new class of amorphous metals, having good processability and consisting of alloying elements suitable for industrial applications. (C) 2017 Acta Materialia Inc. Published by Elsevier Ltd. All rights reserved.

OI Bochtler, Benedikt/0000-0003-0494-735X

SN 1359-6462

PD MAR 15

PY 2018

VL 146

BP 73

EP 76

DI 10.1016/j.scriptamat.2017.11.011

UT WOS:000424719400017

ER

PT J

AU Tauseef, A

Tariq, NH

Akhter, JI

Hasan, BA

Mehmood, M

AF Tauseef, A.

Tariq, N. H.

Akhter, J. I.

Hasan, B. A.

Mehmood, M.

TI Corrosion Behavior of Zr-Cu-Ni-Al Bulk Metallic Glasses in Chloride

Medium (vol 489, pg 596, 2010)

SO JOURNAL OF ALLOYS AND COMPOUNDS

RI Mehmood, Mazhar/G-4048-2011; Tariq, Naeem ul Haq/F-3088-2013; Akhter,

Javed Iqbal/I-5791-2015

OI Mehmood, Mazhar/0000-0001-8076-0365;

SN 0925-8388

PD MAR 18

PY 2010

VL 493

IS 1-2

BP 708

EP 708

DI 10.1016/j.jallcom.2010.02.108

UT WOS:000276054200141

ER

PT J

AU Nguyen, HM

Hsiao, PY

AF Nguyen, Ha M.

Hsiao, Pai-Yi

TI Comment on "Critical and slow dynamics in a bulk metallic glass

exhibiting strong random magnetic anisotropy" [Appl. Phys. Lett. 92,

011923 (2008)]

SO APPLIED PHYSICS LETTERS

SN 0003-6951

PD MAY 4

PY 2009

VL 94

IS 18

AR 186101

DI 10.1063/1.3119632

UT WOS:000265933700074

ER

PT J

AU Du, XH

Huang, JC

Hsieh, KC

Jang, JSC

Liaw, PK

Chen, HM

Chou, HS

Lai, YH

AF Du, Xinghao

Huang, Jacob C.

Hsieh, Ker-Chang

Jang, Jason S. C.

Liaw, Peter K.

Chen, Hai-Ming

Chou, Hung-Sheng

Lai, Yan-Huei

TI Designing Ductile Zr-Based Bulk Metallic Glasses with Phase Separated

Microstructure

SO ADVANCED ENGINEERING MATERIALS

RI Huang, J./C-4276-2013

OI HUANG, Jacob Chih Ching/0000-0001-6843-3396

SN 1438-1656

PD MAY

PY 2009

VL 11

IS 5

BP 387

EP 391

DI 10.1002/adem.200800370

UT WOS:000266474300009

ER

PT J

AU Wu, JL

Pan, Y

Pi, JH

AF Wu, Jili

Pan, Ye

Pi, Jinhong

TI Nanoindentation Mechanical Properties of Indium-Alloyed Cu-Based Bulk

Metallic Glasses

SO JOURNAL OF MATERIALS ENGINEERING AND PERFORMANCE

AB In this paper, two Indium-alloyed Cu-based bulk metallic glasses, Cu54Zr37Ti8In1 and Cu50Zr37Ti8In5, have been evaluated with nanoindentation testing. Both bulk metallic glasses have homogenous nature in structure. Both hardness and Young's modulus of bulk metallic glasses do not show a loading rate-dependent. Addition of In decreases hardness and Young's modulus, but increases creep-resistance of bulk metallic glasses. Indentation creep of two bulk metallic glasses has also been investigated. The displacement-time curves of creep processes were described with generalized Kelvin model. The creep displacement, compliance spectrum, and retardation spectrum for each bulk metallic glass were discussed comparatively. The results showed that Cu50Zr37Ti8In5 has better creep-resistance at room temperature and a more relaxed state.

SN 1059-9495

EI 1544-1024

PD FEB

PY 2014

VL 23

IS 2

BP 486

EP 492

DI 10.1007/s11665-013-0765-y

UT WOS:000330594800017

ER

PT J

AU Hamill, L

Roberts, S

Davidson, M

Johnson, WL

Nutt, S

Hofmann, DC

AF Hamill, Lee

Roberts, Scott

Davidson, Marc

Johnson, William L.

Nutt, Steven

Hofmann, Douglas C.

TI Hypervelocity Impact Phenomenon in Bulk Metallic Glasses and Composites

SO ADVANCED ENGINEERING MATERIALS

SN 1438-1656

EI 1527-2648

PD JAN

PY 2014

VL 16

IS 1

BP 85

EP 93

DI 10.1002/adem.201300252

UT WOS:000330961100014

ER

PT J

AU Dragoi, D

Ustundag, E

Clausen, B

Bourke, MAM

AF Dragoi, D

Ustundag, E

Clausen, B

Bourke, MAM

TI Investigation of thermal residual stresses in tungsten-fiber/bulk

metallic glass matrix composites

SO SCRIPTA MATERIALIA

RI Clausen, Bjorn/B-3618-2015; Ustundag, Ersan/C-1258-2009

OI Clausen, Bjorn/0000-0003-3906-846X; Ustundag, Ersan/0000-0002-0812-7028

SN 1359-6462

PD JUL 31

PY 2001

VL 45

IS 2

BP 245

EP 252

DI 10.1016/S1359-6462(01)01031-4

UT WOS:000170899700017

ER

PT J

AU Tao, PJ

Zhang, WW

Tu, Q

Yang, YZ

He, YD

AF Tao, P. J.

Zhang, W. W.

Tu, Q.

Yang, Y. Z.

He, Y. D.

TI EFFECT OF METAL-HYDROGENATION ON WEAR RESISTANCE IN ZRNIAL BULK METALLIC

GLASS

SO BASIC & CLINICAL PHARMACOLOGY & TOXICOLOGY

SN 1742-7835

EI 1742-7843

PD OCT

PY 2016

VL 119

SU 2

SI SI

MA 109

BP 37

EP 37

UT WOS:000385818700110

ER

PT J

AU Zhang, EL

Ma, MZ

AF Zhang, Erlin

Ma, MingZhen

TI High-strength Mg-based bulk metallic glass composites with remarkable

plasticity (vol 100, pg 684, 2009)

SO INTERNATIONAL JOURNAL OF MATERIALS RESEARCH

SN 1862-5282

EI 2195-8556

PD OCT

PY 2009

VL 100

IS 10

BP 1470

EP 1470

UT WOS:000272031200025

ER

PT J

AU Fiore, G

Battezzati, L

AF Fiore, Gianluca

Battezzati, Livio

TI Engraving of a Pd77.5Cu6Si16.5 bulk metallic glass

SO ADVANCED ENGINEERING MATERIALS

OI Battezzati, Livio/0000-0003-1628-0409

SN 1438-1656

PD JUN

PY 2007

VL 9

IS 6

BP 509

EP 511

DI 10.1002/adem.200700049

UT WOS:000247885800018

ER

PT J

AU Egami, T

AF Egami, T

TI Nano-glass mechanism of bulk metallic glass formation

SO MATERIALS TRANSACTIONS

AB A theory of local dynamics of liquid is developed in order to explain the glass transition and fragility of multi-component alloys as it relates to formation of bulk metallic glasses. Unlike the extended hydrodynamic theories in which liquid is regarded as a continuum body, the present approach focuses on the discreteness of the atomic structure and considers the stability of local topology of the network structure. This approach has led to the prediction of the glass transition temperature, melting and glass formability. We extend this approach to describe the effects of local topology on the atomic transport and glass transition and fragility of multi-component glasses. This theory leads to a picture of a strong liquid as a nano-scale composite of glass and liquid, and suggests compositional requirements for forming bulk metallic glasses.

SN 1345-9678

EI 1347-5320

PD MAR

PY 2002

VL 43

IS 3

BP 510

EP 517

DI 10.2320/matertrans.43.510

UT WOS:000175239300049

ER

PT J

AU Wang, YM

Xu, WP

Qiang, JB

Wong, CH

Shek, CH

Dong, C

AF Wang, Y. M.

Xu, W. P.

Qiang, J. B.

Wong, C. H.

Shek, C. H.

Dong, C.

TI The e/a criterion of Zr-based bulk metallic glasses (Retraction of vol

375, pg 411, 2004)

SO MATERIALS SCIENCE AND ENGINEERING A-STRUCTURAL MATERIALS PROPERTIES

MICROSTRUCTURE AND PROCESSING

RI SHEK, Chan Hung/J-3857-2015

OI SHEK, Chan Hung/0000-0002-6870-523X

SN 0921-5093

EI 1873-4936

PD JAN 1

PY 2014

VL 589

BP 310

EP 310

DI 10.1016/j.msea.2003.10.215

UT WOS:000328521800038

ER

PT J

AU Liang, L

Hui, X

Yue, YL

Chen, GL

AF Liang, L.

Hui, X.

Yue, Y. L.

Chen, G. L.

TI Improvement of the thermal stability of Gd-Dy-Al-Co bulk metallic glass

by the addition of high bulk modulus elements

SO MATERIALS LETTERS

AB A series of new heavy rare-earth based bulk metallic glasses with high thermal stability have been obtained by a copper mold casting method. Compared with the light rare-earth based bulk metallic glasses, these alloys have much higher glass transition temperature, crystallization temperature, and larger effective activation energy for glass transition and crystallization. It was found that the addition of high bulk modulus element Ge can further enhance the thermal stability of these alloys. These results confirm that the thermal stability is closely correlated with the bulk modulus of rare-earth based bulk metallic glasses, which is useful for guiding the composition design of the BMG alloys with high thermal stability. (c) 2007 Elsevier B.V. All rights reserved.

RI Hui, Xidong/A-1741-2010

SN 0167-577X

PD MAR 15

PY 2008

VL 62

IS 6-7

BP 994

EP 997

DI 10.1016/j.matlet.2007.07.033

UT WOS:000253106100055

ER

PT J

AU Hofmann, DC

AF Hofmann, Douglas C.

TI Shape Memory Bulk Metallic Glass Composites

SO SCIENCE

SN 0036-8075

PD SEP 10

PY 2010

VL 329

IS 5997

BP 1294

EP 1295

DI 10.1126/science.1193522

UT WOS:000281657300023

PM 20829474

ER

PT J

AU Kai, W

Ren, IF

Kao, PC

Wang, RF

Chuang, CP

Freels, MW

Liaw, PK

AF Kai, Wu

Ren, I. Fei

Kao, Pei Chin

Wang, Rui Fang

Chuang, Chih-Pin

Freels, Matthew W.

Liaw, Peter K.

TI Air-Oxidation of a Cu45Zr45Al5Ag5 Bulk Metallic Glass

SO ADVANCED ENGINEERING MATERIALS

SN 1438-1656

PD MAY

PY 2009

VL 11

IS 5

BP 380

EP 386

DI 10.1002/adem.200800366

UT WOS:000266474300008

ER

PT J

AU Hammond, VH

Houtz, MD

O'Reilly, JM

AF Hammond, VH

Houtz, MD

O'Reilly, JM

TI Structural relaxation in a bulk metallic glass (vol 325, pg 179, 2003)

SO JOURNAL OF NON-CRYSTALLINE SOLIDS

SN 0022-3093

PD OCT 15

PY 2003

VL 328

IS 1-3

BP 254

EP 254

DI 10.1016/S0022-3093(03)00513-1

UT WOS:000186079300026

ER

PT J

AU Xiao, XS

Li, WH

Xia, L

Hua, Q

Fang, SS

Dong, Y

AF Xiao, XS

Li, WH

Xia, L

Hua, Q

Fang, SS

Dong, Y

TI Dynamic tensile response of Zr41.2Ti13.8Cu12.5Ni10Be22.5 bulk metallic

glass

SO JOURNAL OF MATERIALS SCIENCE LETTERS

OI Xia, Lei/0000-0001-9198-1497

SN 0261-8028

PD MAR 1

PY 2003

VL 22

IS 5

BP 407

EP 411

DI 10.1023/A:1022669832084

UT WOS:000181394200022

ER

PT S

AU Upadhyaya, GS

AF Upadhyaya, G. S.

BA Upadhyaya, GS

BF Upadhyaya, GS

TI FUTURE TRENDS

SO METAL SCIENCE: PAST, PRESENT AND FUTURE

SE Materials Science Foundations

SN 1422-3597

BN 978-3-03785-997-1

PY 2013

VL 75

BP 215

EP 237

UT WOS:000323089800007

ER

PT J

AU Torrens-Serra, J

Solivelles, F

Corro, ML

Stoica, M

Kustov, S

AF Torrens-Serra, J.

Solivelles, F.

Corro, M. L.

Stoica, M.

Kustov, S.

TI Effect of temperature and magnetic field on magnetomechanical damping of

Fe-based bulk metallic glasses (vol 49, 505003, 2016)

SO JOURNAL OF PHYSICS D-APPLIED PHYSICS

RI Stoica, Mihai/B-7069-2015; Torrens-Serra, Joan/L-9805-2018

SN 0022-3727

EI 1361-6463

PD JAN 25

PY 2017

VL 50

IS 3

AR 039601

DI 10.1088/1361-6463/aa4ebc

UT WOS:000390794500001

ER

PT J

AU Paduraru, A

Kenoufi, A

Bailey, NP

Schiotz, J

AF Paduraru, Anca

Kenoufi, Abder

Bailey, Nicholas P.

Schiotz, Jacob

TI An interatomic potential for studying CuZr bulk metallic glasses

SO ADVANCED ENGINEERING MATERIALS

RI Schiotz, Jakob/A-5692-2011

OI Schiotz, Jakob/0000-0002-0670-8013

SN 1438-1656

PD JUN

PY 2007

VL 9

IS 6

BP 505

EP 508

DI 10.1002/adem.200700047

UT WOS:000247885800017

ER

PT J

AU Miller, MK

AF Miller, MK

TI Decomposition of bulk metallic glasses

SO MATERIALS SCIENCE AND ENGINEERING A-STRUCTURAL MATERIALS PROPERTIES

MICROSTRUCTURE AND PROCESSING

CT 44th International Field Emission Symposium

CY JUL 07-11, 1997

CL NATL RES INST MET, TSUKUBA, JAPAN

HO NATL RES INST MET

AB An atom probe field ion microscopy (APFIM) characterization has been performed on the decomposition of several bulk metallic glasses. Evidence of short range order for aluminum has been detected in as-cast nickel-containing Zr60Al15Ni25 and Zr55Al10Cu5Ni30 metallic glasses. Titanium-enriched, zirconium-enriched and Be2Zr phases have been analyzed in annealed Zr41.2Ti13.8Cu12.5Ni10.0Be22.5 bulk metallic glass. (C) 1998 Elsevier Science S.A. All rights reserved.

SN 0921-5093

PD JUL 15

PY 1998

VL 250

IS 1

BP 133

EP 140

DI 10.1016/S0921-5093(98)00549-8

UT WOS:000074875600023

ER

PT J

AU Li, HQ

Yang, YS

Tong, WH

Wang, ZY

AF Li, HQ

Yang, YS

Tong, WH

Wang, ZY

TI Numerical simulation of bulk metallic glass preparation process with

suction casting

SO MATERIALS TECHNOLOGY

OI Yang, Yuansheng/0000-0003-4317-1101

SN 1066-7857

PD MAR

PY 2006

VL 21

IS 1

BP 44

EP 47

DI 10.1179/mte.2006.21.1.44

UT WOS:000236840100010

ER

PT J

AU Xing, LQ

Ochin, P

AF Xing, LQ

Ochin, P

TI Bulk glass formation in the Zr-Ti-Al-Cu-Ni system

SO JOURNAL OF MATERIALS SCIENCE LETTERS

SN 0261-8028

PD AUG 1

PY 1997

VL 16

IS 15

BP 1277

EP 1280

DI 10.1023/A:1018574808365

UT WOS:A1997XP29500014

ER

PT J

AU LAMBSON, EF

LAMBSON, WA

MACDONALD, JE

GIBBS, MRJ

SAUNDERS, GA

TURNBULL, D

AF LAMBSON, EF

LAMBSON, WA

MACDONALD, JE

GIBBS, MRJ

SAUNDERS, GA

TURNBULL, D

TI ELASTIC BEHAVIOR AND VIBRATIONAL ANHARMONICITY OF A BULK PD40NI40P20

METALLIC-GLASS

SO PHYSICAL REVIEW B

OI Macdonald, John/0000-0001-5504-1692

SN 0163-1829

PD FEB 15

PY 1986

VL 33

IS 4

BP 2380

EP 2385

DI 10.1103/PhysRevB.33.2380

UT WOS:A1986A018700034

ER

PT J

AU Flores, KM

Dauskardt, RH

AF Flores, KM

Dauskardt, RH

TI Enhanced toughness due to stable crack tip damage zones in bulk metallic

glass

SO SCRIPTA MATERIALIA

SN 1359-6462

PD OCT 8

PY 1999

VL 41

IS 9

BP 937

EP 943

DI 10.1016/S1359-6462(99)00243-2

UT WOS:000083267100005

ER

PT J

AU Park, JM

Park, JS

Kim, JH

Chang, HJ

AF Park, JM

Park, JS

Kim, JH

Chang, HJ

TI Mechanical behaviors of partially devitrified ti-based bulk metallic

glass

SO JOURNAL OF MATERIALS SCIENCE

SN 0022-2461

EI 1573-4803

PD SEP

PY 2005

VL 40

IS 18

BP 4999

EP 5001

DI 10.1007/s10853-005-1170-1

UT WOS:000232059600050

ER

PT B

AU Karmakar, B

AF Karmakar, Basudeb

BA Karmakar, B

BF Karmakar, B

TI Functional bulk metallic glasses

SO FUNCTIONAL GLASSES AND GLASS-CERAMICS: PROCESSING, PROPERTIES, AND

APPLICATIONS

BN 978-0-12-805207-5; 978-0-12-805056-9

PY 2017

BP 365

EP 390

DI 10.1016/B978-0-12-805056-9.00012-X

UT WOS:000427863400014

ER

PT J

AU Inoue, A

Zhang, T

Ishihara, S

Saida, J

Matsushita, M

AF Inoue, A

Zhang, T

Ishihara, S

Saida, J

Matsushita, M

TI Preparation and mechanical properties of nanoquasicrystalline base bulk

alloys

SO SCRIPTA MATERIALIA

CT 5th International Conference on Nanostructured Materials (NANO 2000)

CY AUG 20-25, 2000

CL SENDAI, JAPAN

SP Int Comm Nanostructrued Mt, Acta Materialia Inc, Inst Mat Res, Tohoku Univ, Inst Sci & Ind Res, Osaka Univ, Sendai City, Japan Soc Promot Sci, Iwatani Naoji Fdn

RI Saida, Junji/C-1912-2009; Zhang, Tao/O-4911-2014; Inoue,

Akihisa/E-5271-2015

SN 1359-6462

PD MAY 18

PY 2001

VL 44

IS 8-9

BP 1615

EP 1619

DI 10.1016/S1359-6462(01)00784-9

UT WOS:000169389400089

ER

PT J

AU Inoue, A

AF Inoue, A

TI Metallic glasses

SO SEN-I GAKKAISHI

RI Inoue, Akihisa/E-5271-2015

SN 0037-9875

PD SEP

PY 1996

VL 52

IS 9

BP P375

EP P381

DI 10.2115/fiber.52.9_P375

UT WOS:A1996VP62600005

ER

PT B

AU Schreiber, D

Ryan, JV

AF Schreiber, Daniel

Ryan, Joseph V.

BE Affatigato, M

TI ATOM PROBE TOMOGRAPHY OF GLASSES

SO MODERN GLASS CHARACTERIZATION

BN 978-1-119-05188-6; 978-1-118-23086-2

PY 2015

BP 391

EP 429

D2 10.1002/9781119051862

UT WOS:000386637500011

ER

PT J

AU Suh, D

Dauskardt, RH

AF Suh, D

Dauskardt, RH

TI Hydrogen effects on the mechanical and fracture behavior of a

Zr-Ti-Ni-Cu-Be bulk metallic glass

SO SCRIPTA MATERIALIA

SN 1359-6462

PD JAN 17

PY 2000

VL 42

IS 3

BP 233

EP 240

DI 10.1016/S1359-6462(99)00337-1

UT WOS:000085125300003

ER

PT J

AU Chen, JX

Zhou, SY

AF Chen, Jixiang

Zhou, Shuoyao

TI From Eutectic Clusters to Bulk Metallic Glasses of Zr-Al-Cu System

SO ADVANCED ENGINEERING MATERIALS

SN 1438-1656

EI 1527-2648

PD OCT

PY 2013

VL 15

IS 10

BP 966

EP 969

DI 10.1002/adem.201200389

UT WOS:000325367100014

ER

PT J

AU Figueroa, IA

Davies, HA

Todd, I

Yamada, K

AF Figueroa, I. A.

Davies, H. A.

Todd, I.

Yamada, K.

TI Formation and thermal stability of Cu-Hf-Ti-M glassy alloys

SO ADVANCED ENGINEERING MATERIALS

RI Figueroa, Ignacio/J-5914-2012

SN 1438-1656

PD JUN

PY 2007

VL 9

IS 6

BP 496

EP 499

DI 10.1002/adem.200700051

UT WOS:000247885800015

ER

PT J

AU Zhang, QS

Deng, YF

Zhang, HF

Ding, BZ

Hu, ZQ

AF Zhang, QS

Deng, YF

Zhang, HF

Ding, BZ

Hu, ZQ

TI Cyclic softening of Zr55Al10Ni5Cu30 bulk amorphous alloy

SO JOURNAL OF MATERIALS SCIENCE LETTERS

RI zhang, qingsheng/A-4851-2010

SN 0261-8028

PD DEC 1

PY 2003

VL 22

IS 23

BP 1731

EP 1734

DI 10.1023/B:JMSL.0000004660.67750.7c

UT WOS:000186661600022

ER

PT J

AU Grimberg, A

Buhler, F

Bochsler, P

Heber, VS

Tosatti, S

Jurewicz, AJG

Hays, CC

McNamara, K

Allton, JH

Burnett, DS

Baur, H

Wieler, R

AF Grimberg, A

Buhler, F

Bochsler, P

Heber, VS

Tosatti, S

Jurewicz, AJG

Hays, CC

McNamara, K

Allton, JH

Burnett, DS

Baur, H

Wieler, R

TI Solar wind noble gases - Preliminary results from bulk metallic glass

flown on Genesis

SO METEORITICS & PLANETARY SCIENCE

CT 68th Annual Meeting of the Meteoritical-Society

CY SEP 12-16, 2005

CL Gatlinburg, TN

SP Meteorit Soc

RI Wieler, Rainer/A-1355-2010; Hays, Charles/P-8021-2015

OI Wieler, Rainer/0000-0001-5666-7494; Hays, Charles/0000-0002-0420-1761

SN 1086-9379

PD SEP

PY 2005

VL 40

IS 9

SU S

BP A60

EP A60

UT WOS:000234871000101

ER

PT J

AU Homer, ER

Harris, MB

Zirbel, SA

Kolodziejska, JA

Kozachkov, H

Trease, BP

Borgonia, JPC

Agnes, GS

Howell, LL

Hofmann, DC

AF Homer, Eric R.

Harris, Matthew B.

Zirbel, Shannon A.

Kolodziejska, Joanna A.

Kozachkov, Henry

Trease, Brian P.

Borgonia, John-Paul C.

Agnes, Gregory S.

Howell, Larry L.

Hofmann, Douglas C.

TI New Methods for Developing and Manufacturing Compliant Mechanisms

Utilizing Bulk Metallic Glass

SO ADVANCED ENGINEERING MATERIALS

RI Homer, Eric/F-2502-2010; Howell, Larry/A-6828-2008

OI Homer, Eric/0000-0002-8617-7573; Howell, Larry/0000-0001-8132-8822

SN 1438-1656

EI 1527-2648

PD JUL

PY 2014

VL 16

IS 7

BP 850

EP 856

DI 10.1002/adem.201300566

UT WOS:000339436000002

ER

PT J

AU Zhang, AL

Chen, D

Chen, ZH

AF Zhang, Ailong

Chen, Ding

Chen, Zhenhua

TI Bulk Metallic Glass-Forming Region of Four Multicomponent Alloy Systems

(vol 50, pg 1240, 2009)

SO MATERIALS TRANSACTIONS

SN 1345-9678

EI 1347-5320

PD AUG

PY 2009

VL 50

IS 8

BP 2127

EP 2127

UT WOS:000269945300039

ER

PT J

AU Schneider, S

AF Schneider, S

TI Bulk metallic glasses

SO JOURNAL OF PHYSICS-CONDENSED MATTER

AB A recently developed large number of bulk glass-forming alloys, known as bulk metallic glasses, offer new opportunities for engineering applications and basic research on the nature of the glassy and undercooled liquid states in metals. This text gives a short review on bulk metallic glasses and their properties, focusing on the Vitreloy (TM) alloys which belong to the best-investigated group of bulk glass-forming metallic systems.

SN 0953-8984

PD AUG 27

PY 2001

VL 13

IS 34

BP 7723

EP 7736

DI 10.1088/0953-8984/13/34/316

UT WOS:000170857400017

ER

PT S

AU Xie, GQ

Zhu, SL

Qin, FX

AF Xie, Guoqiang

Zhu, Shengli

Qin, Fengxiang

BE Zhu, S

Ni, B

Ju, DY

TI Ti-based Bulk Metallic Glass Composites Produced by Spark Plasma

Sintering

SO ADVANCED MATERIALS SCIENCE AND TECHNOLOGY, (IFAMST-8)

SE Materials Science Forum

CT 8th International Forum on Advanced Materials Science and Technology

(IFAMST-8)

CY AUG 01-04, 2012

CL Fukuoka Inst Technol, Fukuoka City, JAPAN

SP Saitama Inst Technol, Chinese Mat Res Soc Japan, Chinese Acad & Profess Assoc Japan

HO Fukuoka Inst Technol

AB Using gas-atomized Ti-based metallic glassy powder, or the mixed powder blended with hydroxyapatite powder, we produced Ti-based bulk metallic glasses and the composites with high strength and satisfying large size requirements by a spark plasma sintering process. In this paper we present and review our research results on the fabrication and structure of the Ti-based bulk metallic glasses and the composites.

RI Zhu, Shengli/D-5281-2009; Xie, Guoqiang/A-8619-2011

OI Zhu, Shengli/0000-0002-0190-2626;

SN 0255-5476

PY 2013

VL 750

BP 52

EP 55

DI 10.4028/www.scientific.net/MSF.750.52

UT WOS:000319235900013

ER

PT J

AU Jiao, W

Zhao, K

Xi, XK

Zhao, DQ

Pan, MX

Wang, WH

AF Jiao, W.

Zhao, K.

Xi, X. K.

Zhao, D. Q.

Pan, M. X.

Wang, W. H.

TI Zinc-based bulk metallic glasses

SO JOURNAL OF NON-CRYSTALLINE SOLIDS

AB We report the fabrication and properties of zinc-based bulk metallic glasses (BMGs). The formation mechanism of the BMGs is also discussed. The combination properties of high corrosion resistance, low glass transition temperature T-g and high formability, low Young's modulus and density, low cost, harmless components to organisms and tunable degradation behavior make the Zn-based metallic glasses or metallic plastics (MPs) a potential candidate for micro- and nano-manufacturing materials and biomaterials. (C) 2010 Elsevier B.V. All rights reserved.

RI BAI, JIE/D-7448-2016; wei, jiao/I-7244-2013

SN 0022-3093

EI 1873-4812

PD AUG 1

PY 2010

VL 356

IS 35-36

BP 1867

EP 1870

DI 10.1016/j.jnoncrysol.2010.07.017

UT WOS:000282395500021

ER

PT J

AU DREHMAN, AJ

GREER, AL

TURNBULL, D

AF DREHMAN, AJ

GREER, AL

TURNBULL, D

TI BULK FORMATION OF A METALLIC-GLASS - PD-40 NI-40-P-20

SO APPLIED PHYSICS LETTERS

RI Greer, Lindsay/E-9433-2017

SN 0003-6951

PY 1982

VL 41

IS 8

BP 716

EP 717

DI 10.1063/1.93645

UT WOS:A1982PL39400015

ER

PT J

AU Huang, YJ

Khong, JC

Connolley, T

Mi, J

AF Huang, Yongjiang

Khong, J. C.

Connolley, Thomas

Mi, J.

TI The onset of plasticity of a Zr-based bulk metallic glass (vol 60, pg

87, 2014)

SO INTERNATIONAL JOURNAL OF PLASTICITY

RI Huang, Yongjiang/D-4809-2009

SN 0749-6419

EI 1879-2154

PD MAY

PY 2015

VL 68

BP 164

EP 164

DI 10.1016/j.ijplas.2014.12.002

UT WOS:000351962600010

ER

PT J

AU Jeong, JI

Lee, SH

Jeon, JB

Kim, SJ

AF Jeong, Jae Im

Lee, Sung Hyuk

Jeon, Je-Beom

Kim, Suk Jun

TI Excessively High Vapor Pressure of Al-Based Amorphous Alloys

SO METALS

SN 2075-4701

PD DEC

PY 2015

VL 5

IS 4

BP 1878

EP 1886

DI 10.3390/met5041878

UT WOS:000367545100009

ER

PT J

AU Kawamura, Y

Ohno, Y

AF Kawamura, Y

Ohno, Y

TI Superplastic bonding of bulk metallic glasses using friction

SO SCRIPTA MATERIALIA

AB We have succeeded in joining Pd40Ni40P20 bulk metallic glasses using a friction welding method where superplasticity of supercooled liquid was utilized. No crystallization was observed in the interface zone, and no visible interface was observed. The tensile strength of the joined bulk metallic glasses was the same as that of the parent bulk metallic glass. (C) 2001 Acta Materialia Inc. Published by Elsevier Science Ltd. All rights reserved.

SN 1359-6462

PD AUG 13

PY 2001

VL 45

IS 3

BP 279

EP 285

DI 10.1016/S1359-6462(01)01025-9

UT WOS:000172612500005

ER

PT J

AU Jiang, JZ

AF Jiang, JZ

TI Comment on "Pressure-induced amorphization of ZrTiCuNiBe bulk

glass-forming alloy" [Appl. Phys. Lett. 79, 1106 (2001)]

SO APPLIED PHYSICS LETTERS

SN 0003-6951

PD JAN 28

PY 2002

VL 80

IS 4

BP 700

EP 700

DI 10.1063/1.1445267

UT WOS:000173508900056

ER

PT J

AU BHANUMURTHY, K

DEY, GK

BANERJEE, S

AF BHANUMURTHY, K

DEY, GK

BANERJEE, S

TI METALLIC-GLASS FORMATION B SOLID-STATE REACTION IN BULK ZIRCONIUM COPPER

DIFFUSION COUPLES

SO SCRIPTA METALLURGICA

SN 0036-9748

PD SEP

PY 1988

VL 22

IS 9

BP 1395

EP 1398

DI 10.1016/S0036-9748(88)80007-3

UT WOS:A1988P835300007

ER

PT J

AU Wang, WH

Wen, P

Zhao, DQ

Pan, MX

Wang, RJ

AF Wang, WH

Wen, P

Zhao, DQ

Pan, MX

Wang, RJ

TI Relationship between glass transition temperature and Debye temperature

in bulk metallic glasses

SO JOURNAL OF MATERIALS RESEARCH

AB The Debye temperature and glass transition temperature of a variety of bulk metallic glasses (BMGs) were determined by acoustic measurement and differential scanning calorimetry, respectively. The relationship between the Debye temperature and glass transition temperature of these BMGs was analyzed, and their observed correlation was interpreted in terms of the characteristics of the glass transition in BMGs.

SN 0884-2914

PD DEC

PY 2003

VL 18

IS 12

BP 2747

EP 2751

DI 10.1557/JMR.2003.0382

UT WOS:000186955800002

ER

PT J

AU Hufnagel, TC

El-Deiry, P

Vinci, RP

AF Hufnagel, TC

El-Deiry, P

Vinci, RP

TI Development of shear band structure during deformation of a

Zr57Ti5Cu20Ni8Al10 bulk metallic glass

SO SCRIPTA MATERIALIA

RI Hufnagel, Todd/A-3309-2010

OI Hufnagel, Todd/0000-0002-6373-9377; Vinci, Richard/0000-0002-3321-860X

SN 1359-6462

PD NOV 27

PY 2000

VL 43

IS 12

BP 1071

EP 1075

DI 10.1016/S1359-6462(00)00527-3

UT WOS:000165892800004

ER

PT S

AU Chiba, A

Kawamura, Y

Nishida, M

AF Chiba, A.

Kawamura, Y.

Nishida, M.

BE Itoh, S

Hokamoto, K

TI Explosive welding of ZrTiCuNiBe bulk metallic glass to crystalline

metallic plates

SO EXPLOSION, SHOCK WAVE AND HYPERVELOCITY PHENOMENA IN MATERIALS II

SE MATERIALS SCIENCE FORUM

CT 2nd International Symposium on Explosion, Shock Wave and Hypervelocity

Phenomena (ESHP-2)

CY MAR 06-09, 2007

CL Kumamoto, JAPAN

SP Shock Wave Condensed Matter Res Ctr, 21st Cent COE Program, Kumamoto Univ, Kumamoto Univ, Fac Engn, Japan Explosives Soc Comm, Japan Soc Technol Plastic

AB Recently, a number of amorphous alloys that possess high glass-forming ability and a wide supercooled liquid region before crystallization have been discovered. Especially, bulk metallic glasses, which are made in bulk form with a thickness of similar to 10 mm at slow cooling rates of the order of 1 similar to 100 K/s, have been noted as an industrial application. Hence the welding of bulk metallic glasses to other materials is very important. Explosive welding of most popular Zr41.2Ti13.8Cu10Ni12.5Be22.5 bulk metallic glass to crystalline pure Ti and SUS304 plates is investigated in this paper. The BMGs was found to retain the amorphous structure and the original mechanical properties. The sound bonding with other materials is expected to push forward the application of bulk metallic glass for industrial usage.

RI nishida, minoru/E-4027-2012

SN 0255-5476

PY 2008

VL 566

BP 119

EP 124

DI 10.4028/www.scientific.net/MSF.566.119

UT WOS:000252748700020

ER

PT B

AU LeBourhis, E

AF LeBourhis, E

TI Glass: Mechanics and Technology, 2nd Edition

SO GLASS: MECHANICS AND TECHNOLOGY, 2ND EDITION

RI Le Bourhis, Eric/B-4568-2013

OI Le Bourhis, Eric/0000-0003-0902-5899

BN 978-3-527-67942-3; 978-3-527-33705-7

PY 2014

BP 1

EP 388

DI 10.1002/9783527679461

UT WOS:000353172000030

ER

PT J

AU Liu, YH

Wang, WH

AF Liu, Y. H.

Wang, W. H.

TI Shear bands evolution in bulk metallic glass with extended plasticity

SO JOURNAL OF NON-CRYSTALLINE SOLIDS

AB Shear band development and evolution of their spacing under bending in a Zr-based bulk metallic glass with extended plasticity has been monitored as a function of bending angle from the onset of plastic deformation to fracture. we find that sliding of existing shear bands is an important mechanism accounting for the plastic deformation of the plastic bulk metallic glass. The results are beneficial to understanding deformation in metallic glasses. (C) 2008 Elsevier B.V. All rights reserved.

RI LIU, Yanhui/B-1485-2009

SN 0022-3093

PD DEC 15

PY 2008

VL 354

IS 52-54

BP 5570

EP 5572

DI 10.1016/j.jnoncrysol.2008.09.026

UT WOS:000261710700038

ER

PT J

AU Hosokawa, S

Sato, H

Mimura, K

Happo, N

Tezuka, Y

Ichitsubo, T

Matsubara, E

Nishiyama, N

AF Hosokawa, Shinya

Sato, Hitoshi

Mimura, Kojiro

Happo, Naohisa

Tezuka, Yasuhisa

Ichitsubo, Tetsu

Matsubara, Eiichiro

Nishiyama, Nobuyuki

TI Soft X-ray emission study of Pd-Ni-Cu-P bulk metallic glass

SO JOURNAL OF ELECTRON SPECTROSCOPY AND RELATED PHENOMENA

CT 10th International Conference on Electronic Spectroscopy and Structure

CY AUG 28-SEP 01, 2006

CL Foz do Iguacu, BRAZIL

RI Ichitsubo, Tetsu/F-3201-2010; Nishiyama, Nobuyuki/C-8228-2015

SN 0368-2048

PD MAY

PY 2007

VL 156

BP XCIX

EP XCIX

UT WOS:000246726300237

ER

PT J

AU Gong, P

Deng, L

Jin, JS

Wang, SB

Wang, XY

Yao, KF

AF Gong, Pan

Deng, Lei

Jin, Junsong

Wang, Sibo

Wang, Xinyun

Yao, Kefu

TI Review on the Research and Development of Ti-Based Bulk Metallic Glasses

SO METALS

AB Ti-based bulk metallic glasses (BMGs) are very attractive for applications because of their excellent properties such as high specific strength and high corrosion resistance. In this paper, we briefly review the current status of the research and development of Ti-based bulk metallic glasses. Emphasis is laid on glass-forming ability, mechanical properties, corrosion resistance, and biocompatibility.

OI Gong, Pan/0000-0002-3833-8440

SN 2075-4701

PD NOV

PY 2016

VL 6

IS 11

AR 264

DI 10.3390/met6110264

UT WOS:000389600400011

ER

PT J

AU Hwang, J

Voyles, PM

AF Hwang, Jinwoo

Voyles, P. M.

TI Nanometer Scale Atomic Order in a Bulk Metallic Glass from Fluctuation

Microscopy

SO MICROSCOPY AND MICROANALYSIS

RI HWANG, JINWOO/D-1760-2013

OI Voyles, Paul/0000-0001-9438-4284

SN 1431-9276

PD JUL

PY 2009

VL 15

SU 2

BP 770

EP 771

DI 10.1017/S1431927609095373

UT WOS:000208119100382

ER

PT J

AU Wang, YP

Lu, K

AF Wang, YP

Lu, K

TI Electrical resistance measurement of glass tansition and crystallization

characteristics of Zr-Al-Cu-Ni metallic glasses

SO JOURNAL OF MATERIALS SCIENCE & TECHNOLOGY

AB In this paper, glass transition and thermal stability of the Zr-Al-Cu-Ni metallic glasses were investigated by using electrical resistance measurement (ERM), DSC and X-ray diffraction techniques. The experimental results show that the ERM is capable of detecting the glass transition of the amorphous alloys and can help to distinguish the crystallization products of the Zr-Al-Cu-Ni metallic glasses owing to the difference of the electrical resistivity between the precipitation phases.

SN 1005-0302

PD NOV

PY 2002

VL 18

IS 6

BP 492

EP 496

UT WOS:000179670900004

ER

PT J

AU Ji, XL

Pan, Y

Yang, SZ

Zhao, JH

Lin, PH

AF Ji Xiulin

Pan Ye

Yang Shunzhen

Zhao Jianhua

Lin Pinghua

TI Effect of Minor Additions on the Glass Forming Ability of Cu-Based Bulk

Metallic Glasses

SO RARE METAL MATERIALS AND ENGINEERING

AB The effect of minor additions and their content on the glass forming ability (GFA) of Cu(55)Zr(38)Al(7) bulk metallic glass was studied. X-ray diffraction (XRD) and differential scanning calorimetry (DSC) study show that the GFA of Cu(55)Zr(38)Al(7) bulk metallic glass is enhanced by 2 at% addition of Ag, Ti, Y or Nd. With 6 at% of Ag addition, the critical size of the metallic glass rod formed is increased from 2 to 4 mm. GFA of Cu(55)Zr(38)Al(7) bulk metallic glass can also be improved obviously by multiple additions with 2 at% Ag and Y. In conclusion, distinct effect on GFA can be obtained by substitution for chemically similar elements or by enlargement of atom dimension extension in the alloying system.

RI JI, XIULIN/D-2882-2015

OI JI, XIULIN/0000-0002-1752-6602

SN 1002-185X

PD JUN

PY 2010

VL 39

SU 1

BP 285

EP 288

UT WOS:000283344300069

ER

PT J

AU Yavari, AR

Inoue, A

Zhang, T

Botta, WJ

Kvick, A

AF Yavari, AR

Inoue, A

Zhang, T

Botta, WJ

Kvick, A

TI Metastable phases, quasicrystals and solid solutions in Zr-based bulk

glass-forming alloys

SO SCRIPTA MATERIALIA

CT 5th International Conference on Nanostructured Materials (NANO 2000)

CY AUG 20-25, 2000

CL SENDAI, JAPAN

SP Int Comm Nanostructrued Mt, Acta Materialia Inc, Inst Mat Res, Tohoku Univ, Inst Sci & Ind Res, Osaka Univ, Sendai City, Japan Soc Promot Sci, Iwatani Naoji Fdn

RI yavari, alain/E-8192-2010; Inoue, Akihisa/E-5271-2015; Zhang,

Tao/O-4911-2014; Botta, Walter/E-7763-2010

OI Botta, Walter/0000-0003-2759-573X

SN 1359-6462

PD MAY 18

PY 2001

VL 44

IS 8-9

BP 1239

EP 1244

DI 10.1016/S1359-6462(01)00689-3

UT WOS:000169389400016

ER

PT J

AU HUANG, XM

XU, YF

CHEN, H

WANG, WK

AF HUANG, XM

XU, YF

CHEN, H

WANG, WK

TI FORMATION OF BULK PD-NI-P METALLIC-GLASS BY USE OF HIGH-PRESSURE

SO CHINESE SCIENCE BULLETIN

SN 1001-6538

PD SEP

PY 1991

VL 36

IS 18

BP 1525

EP 1527

UT WOS:A1991GT14700007

ER

PT J

AU Chen, W

Liu, Z

Schroers, J

AF Chen, Wen

Liu, Ze

Schroers, Jan

TI Joining of bulk metallic glasses in air

SO ACTA MATERIALIA

AB We present a thermoplastic deforming method to join metallic glasses in air. Mechanistically during straining of the interface the oxide layer breaks and pristine alloy flows towards the interface and forms a metallic bond. To demonstrate the effectiveness of this method we chose reactive Zr35Ti30Cu7.5Be27.5 as an example bulk metallic glass system. A model is introduced which quantitatively predicts the bonding strength solely from the shear strength of the metallic glass, the initial surface roughness, and the applied strain. The ability to join even reactive metallic glasses in air on a timescale of the order of milliseconds to seconds at low pressure and temperature with predictable joint strength suggest a highly practical and economic method to join metallic glasses. Published by Elsevier Ltd. on behalf of Acta Materialia Inc.

RI Chen, Wen/C-5796-2012

SN 1359-6454

EI 1873-2453

PD JAN

PY 2014

VL 62

BP 49

EP 57

DI 10.1016/j.actamat.2013.08.053

UT WOS:000328594600002

ER

PT J

AU Tam, RCY

Shek, CH

AF Tam, RCY

Shek, CH

TI Abrasion resistance of Cu based bulk metallic glasses

SO JOURNAL OF NON-CRYSTALLINE SOLIDS

AB The abrasive wear behaviors of three copper based bulk metallic glasses have been studied using pin-on-disc measurement and compared with both annealed metallic glass and 304 stainless steel. The volume loss increases linearly with sliding distance. However, the wear resistances of metallic glasses are not directly proportional to hardness and do not follow the wear law. The wear resistance increases in the order: annealed metallic glass, metallic glass and 304 stainless steel. The wear rates of metallic glasses are strongly dependent on the applied load. Worn surface shows that the metallic glasses suffer from severe wear compared with that of 304 stainless steel. (C) 2004 Elsevier B.V. All rights reserved.

RI SHEK, Chan Hung/J-3857-2015

OI SHEK, Chan Hung/0000-0002-6870-523X

SN 0022-3093

PD NOV 1

PY 2004

VL 347

IS 1-3

BP 268

EP 272

DI 10.1016/j.jnoncrysol.2004.09.008

UT WOS:000225555600037

ER

PT J

AU Wang, JQ

Wang, WH

Bai, HY

AF Wang, J. Q.

Wang, W. H.

Bai, H. Y.

TI Soft ytterbium-based bulk metallic glasses with strong liquid

characteristic by design

SO APPLIED PHYSICS LETTERS

AB A family of Yb-based bulk metallic glasses with excellent glass-forming ability has been fabricated based on the elastic moduli correlations. The YbZnMg(Cu) glasses exhibit very strong liquid characteristic in fragility (m=26 +/- 5), while soft mechanical characteristics, such as low bulk elastic modulus (e.g., Young's modulus is about 26.5 GPa), small Poisson's ratio (0.276), low Vickers hardness (1.52 GPa) and Debye temperature, and exceptionally low glass transition temperature (T-g similar to 347 K). The soft bulk metallic glasses with exceptional values of T-g, fragility, Debye temperature, and elastic moduli confirm some found correlations in metallic glasses.

RI Wang, Jun-Qiang/G-5989-2010; Wang, Junqiang/C-2839-2015

OI Wang, Junqiang/0000-0002-8066-6237

SN 0003-6951

EI 1077-3118

PD JAN 26

PY 2009

VL 94

IS 4

AR 041910

DI 10.1063/1.3075062

UT WOS:000262971800036

ER

PT J

AU Suzuki, H

Kanazawa, I

AF Suzuki, H.

Kanazawa, I.

TI Viscosities of the Zr-based bulk metallic glass-forming liquids

SO INTERMETALLICS

CT 7th International Conference on Bulk-Metallic Glasses

CY NOV 01-05, 2009

CL Busan, SOUTH KOREA

SP Yonsei Univ, Ctr Noncrystalline Mat

AB We have discussed the glass transition and the viscosity of supercooled metallic liquids, by using the replica symmetry breaking and the gauge-invariant formula. We have analyzed the viscosity data points of the Zr-based bulk metallic glass-forming liquid with the present model. (C) 2010 Elsevier Ltd. All rights reserved.

SN 0966-9795

PD OCT

PY 2010

VL 18

IS 10

SI SI

BP 1809

EP 1812

DI 10.1016/j.intermet.2010.03.006

UT WOS:000281420700005

ER

PT J

AU Qin, WD

Li, JS

Kou, HC

Gu, XF

Xue, XY

Zhou, L

AF Qin, Weidong

Li, Jinshan

Kou, Hongchao

Gu, Xiaofeng

Xue, Xiangyi

Zhou, Lian

TI Effects of alloy addition on the improvement of glass forming ability

and plasticity of Mg-Cu-Tb bulk metallic glass

SO INTERMETALLICS

CT 6th International Conference on Bulk-Metallic Glasses (BMG-VI)

CY MAY 11-15, 2008

CL Xian, PEOPLES R CHINA

AB The effects of alloy addition on the glass forming ability, thermal stability and mechanical properties of Mg-Cu-Tb-based bulk metallic glasses were investigated. It was found that appropriate additions of Ag, Zn or Be in Mg(65)Cu(25)Tb(10) could not only improve the glass forming ability, but also enhance the strength and plasticity of these amorphous metallic alloys within a certain composition range. Crown Copyright (C) 2008 Published by Elsevier Ltd. All rights reserved.

RI Gu, Xiaofeng/E-8287-2013

OI Gu, Xiaofeng/0000-0001-8299-6451; KOU, Hongchao/0000-0003-4960-9477; Li,

Jinshan/0000-0002-6894-9760

SN 0966-9795

PD APR

PY 2009

VL 17

IS 4

BP 253

EP 255

DI 10.1016/j.intermet.2008.08.011

UT WOS:000264728400016

ER

PT J

AU Pelletier, JM

van de Moortele, B

AF Pelletier, J. M.

van de Moortele, B.

TI Mechanical properties of bulk metallic glasses: Elastic, visco-elastic

and visco-plastic components in the deformation

SO JOURNAL OF NON-CRYSTALLINE SOLIDS

CT 12th International Conference on Liquid and Amorphous Metals (LAM12)

CY JUL 11-16, 2004

CL Metz, FRANCE

AB Mechanical properties of bulk metallic glasses are strongly dependent on temperature and driving frequency, when a periodic stress is applied. By using mechanical spectroscopy, the various components of the deformation can be obtained: elastic, visco-elastic and viscoplastic. A bulk metallic glass is investigated: Vit 4, i.e., a Zr-Ti-Cu-Ni-Be alloy. The influence of the existence of nano-crystalline particles induced by annealing is also reported. (c) 2007 Elsevier B.V. All rights reserved.

SN 0022-3093

PD OCT 15

PY 2007

VL 353

IS 32-40

BP 3750

EP 3753

DI 10.1016/j.jnoncrysol.2007.05.141

UT WOS:000250235200151

ER

PT J

AU Liao, GK

Long, ZL

Zhao, MSZ

Zhong, M

Liu, W

Chai, W

AF Liao, Guangkai

Long, Zhilin

Zhao, Mingshengzi

Zhong, Min

Liu, Wei

Chai, Wei

TI Serrated flow behavior in a Pd-based bulk metallic glass under

nanoindentation

SO JOURNAL OF NON-CRYSTALLINE SOLIDS

AB This study presents the investigation on the serrated flow behavior of a Pd-based bulk metallic glass under nano indentation. Firstly, a method of curve-fitting and statistical analysis was proposed that found to successfully extract the serration events from the deformation of bulk metallic glasses during nanoindentation. Then the serrated flow at various loading rates were systematically analyzed by using the proposed method. The results showed that the lower loading rates promote the serrated flow, while the individual Serration was more likely to be triggered at higher loading rates. Combined with the intrinsic micromechanism of bulk metallic glasses, we conjectured the serrated flow features may be attributed to the evolution of liquid-like region in bulk metallic glasses. (C) 2017 Elsevier B.V. All rights reserved.

SN 0022-3093

EI 1873-4812

PD MAR 15

PY 2017

VL 460

BP 47

EP 53

DI 10.1016/j.jnoncrysol.2017.01.010

UT WOS:000397835400006

ER

PT S

AU Zhang, XF

Guo, XQ

Kim, KB

Yi, S

AF Zhang, Xin Fang

Guo, Xiaoqing

Kim, K. B.

Yi, S.

BE Tan, HH

TI TiCu-Based Bulk Metallic Glasses Exhibiting Large Compressive Plastic

Strain

SO APPLIED MECHANICS AND MECHANICAL ENGINEERING II, PTS 1 AND 2

SE Applied Mechanics and Materials

CT 2nd International Conference on Applied Mechanics and Mechanical

Engineering (ICAMME 2011)

CY OCT 08-09, 2011

CL Sanya, PEOPLES R CHINA

SP Asia Pacific Environm Sci Res Ctr, Huazhong Normal Univ, Chinese Acad Sci

AB TiCu-based bulk metallic glasses with large plasticity (up to 10%) were fabricated by copper mold suction casting. The detailed studies showed that the large plasticity resulted from the nanoscale heterogeneities formed upon solidification, which resulted in extensively shear band formation, branching and interaction.

SN 1660-9336

PY 2012

VL 138-139

BP 624

EP +

DI 10.4028/www.scientific.net/AMM.138-139.624

PN 1 + 2

UT WOS:000307193900106

ER

PT J

AU Wu, JL

Li, XZ

Cao, HB

Pan, Y

Zhu, YT

AF Wu, Jili

Li, Xingzhou

Cao, Hongbo

Pan, Ye

Zhu, Yuntian

TI Ultraviolet light irradiation on pitting corrosion of Cu-based bulk

metallic glasses

SO JOURNAL OF ALLOYS AND COMPOUNDS

AB We report the observation that UV irradiation substantially improves pitting corrosion resistance of Cu-based bulk metallic glasses. Specifically, two Cu-based bulk metallic glasses in this study showed more stable passive films under UV irradiation than under irradiation-free condition when polarized in 3.5 wt.% NaCl aqueous solution. This finding indicates that Cu-based bulk metallic glasses are promising to serve in high UV irradiation and corrosive environments. (C) 2015 Elsevier B.V. All rights reserved.

RI Zhu, Yuntian/B-3021-2008

OI Zhu, Yuntian/0000-0002-5961-7422

SN 0925-8388

EI 1873-4669

PD MAR 15

PY 2016

VL 661

BP 345

EP 348

DI 10.1016/j.jallcom.2015.11.189

UT WOS:000367521200048

ER

PT J

AU Tam, CY

Shek, CH

AF Tam, CY

Shek, CH

TI Abrasive wear of Cu60Zr30Ti10 bulk metallic glass

SO MATERIALS SCIENCE AND ENGINEERING A-STRUCTURAL MATERIALS PROPERTIES

MICROSTRUCTURE AND PROCESSING

AB The abrasive wear behaviors of Cu60Zr30Ti10 bulk metallic glass in different annealing states have been studied using pin-on-disc measurement. The volume loss increases with both sliding distance and applied load. The hardness of metallic glass increases with annealing temperature but their wear resistances are not directly proportional to hardness and do not follow the standard wear law. The wear rate increases in the order: 30% crystallized metallic glass, metallic glass and 50% crystallized metallic glass. The wear behavior of metallic glass is thought to be related to the wear mechanism involving crack initiation and propagation instead of simply on hardness. Worn surface under higher applied load shows that the metallic glass suffers from severe wear compared with that of annealed metallic glasses. (C) 2004 Elsevier B.V. All rights reserved.

RI SHEK, Chan Hung/J-3857-2015

OI SHEK, Chan Hung/0000-0002-6870-523X

SN 0921-5093

PD OCT 25

PY 2004

VL 384

IS 1-2

BP 138

EP 142

DI 10.1016/j.msea.2004.05.073

UT WOS:000224489300016

ER

PT J

AU Li, HX

Yi, S

AF Li, Hongxiang

Yi, Seonghoon

TI Fabrication of bulk metallic glasses in the alloy system

Fe-C-Si-B-P-Cr-Mo-Al using hot metal and industrial ferro-alloys

SO MATERIALS SCIENCE AND ENGINEERING A-STRUCTURAL MATERIALS PROPERTIES

MICROSTRUCTURE AND PROCESSING

CT 12th International Conference on Rapidly Quenched and Metastable

Materials

CY AUG 21-26, 2005

CL Jeju Isl, SOUTH KOREA

AB Fe-base bulk metallic glasses in the alloy system Fe-C-Si-B-P-Cr-Nlo-Al have been developed using hot metal and industrial ferro-alloys. The amorphous alloys can be cast into fully amorphous rods with diameters of up to 4 mm, demonstrating high glass-forming ability and high strength (> 3 GPa). Thermal stability and crystallization behavior of the Fe-C-Si-B-P-Cr-Nlo-Al bulk metallic glasses are also investigated. The Fe-base bulk metallic glasses can be produced cost-effectively and massively for extensive structural applications. (c) 2006 Elsevier B.V. All rights reserved.

SN 0921-5093

PD MAR 25

PY 2007

VL 449

BP 189

EP 192

DI 10.1016/j.msea.2006.02.262

UT WOS:000245477800040

ER

PT S

AU Kobelev, NP

Kolyvanov, EL

Khonik, VA

AF Kobelev, N. P.

Kolyvanov, E. L.

Khonik, V. A.

BE Darinskii, BM

Magalas, LB

TI Nonlinear elastic properties of bulk metallic glasses

Zr52.5Ti5Cu17.9Ni14.6Al10 and Pd40Cu30Ni10P20

SO MECHANICAL SPECTROSCOPY III: MECHANICAL SPECTROSCOPY AND RELAXATION

PHENOMENA IN SOLIDS

SE SOLID STATE PHENOMENA

CT 3rd International Conference on Mechanical Spectroscopy and Relaxation

Phenomena in Solids

CY 2006

CL Cracow, POLAND

SP Voronezh State Tech Univ, AGH

AB The effect of uniaxial compression on the ultrasound velocities in bulk glassy Zr52.5Ti5Cu17.9Ni14.6Al10 and Pd40Cu30Ni10P20 has been studied and the third-order elastic moduli of these glasses have been determined.

RI Khonik, Vitaly/A-5888-2009

SN 1012-0394

BN 3-908451-24-8

PY 2006

VL 115

BP 127

EP 132

DI 10.4028/www.scientific.net/SSP.115.127

UT WOS:000241414300016

ER

PT J

AU Kelton, KF

AF Kelton, KF

TI Nucleation in silicate and metallic glasses

SO INTERNATIONAL JOURNAL OF NON-EQUILIBRIUM PROCESSING

AB The formation and stability of silicate and metallic glasses are discussed. Selected examples of the time-dependent nucleation rates of crystal and quasicrystal phases in polymorphic and non-polymorphic transformations of liquids and glasses are presented. The kinetic model for the classical theory of nucleation is adequate to understand the nucleation of a phase with the same composition as the parent phase. Recent studies of nucleation in silicate glasses as a function of composition, and devitrification studies in bulk metallic and Al-rare earth metallic glasses, however, suggest that it may not be adequate when chemical partitioning occurs during nucleation. This is particularly relevant for understanding the microstructural development during the devitrification of bulk metallic glasses. Other proposed approaches are examined.

SN 1368-9290

PY 1998

VL 11

IS 2

BP 141

EP 168

UT WOS:000079604200004

ER

PT S

AU Vincent, S

Murty, BS

Bhatt, J

AF Vincent, S.

Murty, B. S.

Bhatt, J.

BE Mittal, R

Chauhan, AK

Mukhopadhyay, R

TI Thermodynamic Criteria for Bulk Metallic Glass Formation in Zr Rich

Quaternary System

SO SOLID STATE PHYSICS, PTS 1 AND 2

SE AIP Conference Proceedings

CT 56th DAE-Solid State Physics Symposium (SSPS)

CY DEC 19-23, 2011

CL SRM Univ, Kattankulathur, INDIA

SP Govt India, Dept Atom Energy (DAE), Board Res Nucl Sci (BRNS)

HO SRM Univ

AB Zr based bulk metallic glasses with unusual combination of properties are deemed to be potential materials for nuclear applications. Glass forming composition in Zr rich Zr-Cu-Al-Ni quaternary system has been predicted using thermodynamic and topological parameter P-HS. Model composition with most negative P-HS value obtained using weighing approach closely matches with available experimental composition suggesting that the present approach can be successfully used to predict bulk metallic glass forming composition.

RI Murty, BS/P-3354-2015

OI Murty, BS/0000-0002-4399-8531

SN 0094-243X

BN 978-0-7354-1044-2

PY 2012

VL 1447

BP 583

EP +

UT WOS:000306973500279

ER

PT J

AU Wei, YX

Xi, XK

Zhao, DQ

Pan, MX

Wang, WH

AF Wei, YX

Xi, XK

Zhao, DQ

Pan, MX

Wang, WH

TI Formation of MgNiPr bulk metallic glasses in air

SO MATERIALS LETTERS

AB The Mg-Ni-Pr bulk metallic glasses (BMGs) can be easily cast into glassy rods up to 3 mm. in diameter in argon and air atmosphere. The underlying mechanism for the unusual oxygen resistance during the preparation process and high glass forming ability of the alloy are investigated and discussed. (C) 2004 Elsevier B.V. All rights reserved.

SN 0167-577X

PD APR

PY 2005

VL 59

IS 8-9

BP 945

EP 947

DI 10.1016/j.matlet.2004.09.049

UT WOS:000227164000019

ER

PT J

AU Wang, K

Pan, D

Chen, MW

Zhang, W

Wang, XM

Inoue, A

AF Wang, K.

Pan, D.

Chen, M. W.

Zhang, W.

Wang, X. M.

Inoue, A.

TI Measuring elastic energy density of bulk metallic glasses by

nanoindentation

SO MATERIALS TRANSACTIONS

AB The elastic energy storing capability of bulk metallic glasses was evaluated by employing depth-sensing nanoindentation. The elastic energy densities of four glassy alloys, determined by nanoindentation measurements, are fairly close to their theoretical values estimated from elastic modulus and theoretical strength. This study provides an accurate and quick method to measure the elastic properties of bulk metallic glasses.

RI Pan, Deng/A-1090-2018; CHEN, Mingwei/A-4855-2010; Wang, Ke/M-7171-2017;

Wang, Ke/C-8021-2011; Inoue, Akihisa/E-5271-2015; Pan, Deng/C-2072-2009

OI CHEN, Mingwei/0000-0002-8274-3099; Wang, Ke/0000-0002-1174-0907; Chen,

Mingwei/0000-0002-2850-8872

SN 1345-9678

EI 1347-5320

PD AUG

PY 2006

VL 47

IS 8

BP 1981

EP 1984

DI 10.2320/matertrans.47.1981

UT WOS:000241152800027

ER

PT J

AU Caron, A

Wunderlich, R

Louzguine, DV

Egami, T

Fecht, HJ

AF Caron, A.

Wunderlich, R.

Louzguine, D. V.

Egami, T.

Fecht, H. -J.

TI On the glass transition temperature and the elastic properties in

Zr-based bulk metallic glasses

SO PHILOSOPHICAL MAGAZINE LETTERS

AB The temperature dependence of the elastic moduli was estimated from ultrasound time of flight measurements performed on bulk metallic glasses of composition Zr63-xCu24AlxNi10Co3. Using the corresponding values at the glass transition temperature, the local atomic strain was determined. The obtained values for the critical atomic strain calculated for 8 at% < x < 15 at% are close to the predicted universal criterion derived from a topological model, but may also reflect the difference in the chemical interaction that are not accounted by a topological approach.

RI Caron, Arnaud/B-6463-2010; LOUZGUINE, Dmitri/D-2492-2010

OI Caron, Arnaud/0000-0003-0985-7441; LOUZGUINE, Dmitri/0000-0001-5716-4987

SN 0950-0839

PY 2011

VL 91

IS 12

BP 751

EP 756

DI 10.1080/09500839.2011.616181

UT WOS:000296911700003

ER

PT J

AU Li, YL

Zhao, SF

Liu, YH

Gong, P

Schroers, J

AF Li, Yanglin

Zhao, Shaofan

Liu, Yanhui

Gong, Pan

Schroers, Jan

TI How Many Bulk Metallic Glasses Are There?

SO ACS COMBINATORIAL SCIENCE

AB Quantitative prediction of glass forming ability using a priori known parameters is highly desired in metallic glass development; however proven to be challenging because of the complexity of glass formation. Here, we estimate the number of potential metallic glasses (MGs) and bulk metallic glasses (BMGs) forming systems and alloys, from empirically determined alloy design rules based on a priori known parameters. Specifically, we take into account atomic size ratio, heat of mixing, and liquidus temperature, which we quantify on binary glasses and centimeter-sized BMGs. When expanding into higher order systems that can be formed among 32 practical elements, we reduce the composition space for BMG formation using developed criteria by 10(6) times and estimate similar to 3 million binary, ternary, quaternary, and quinary BMGs alloys.

SN 2156-8952

EI 2156-8944

PD NOV

PY 2017

VL 19

IS 11

BP 687

EP 693

DI 10.1021/acscombsci.7b00048

UT WOS:000415392200003

PM 28902986

ER

PT J

AU Nishiyama, N

Amiya, K

Inoue, A

AF Nishiyama, N.

Amiya, K.

Inoue, A.

TI Recent progress of bulk metallic glasses for strain-sensing devices

SO MATERIALS SCIENCE AND ENGINEERING A-STRUCTURAL MATERIALS PROPERTIES

MICROSTRUCTURE AND PROCESSING

CT 12th International Conference on Rapidly Quenched and Metastable

Materials

CY AUG 21-26, 2005

CL Jeju Isl, SOUTH KOREA

AB By use of high strength, high elastic strain limit and low Young's modulus of bulk metallic glasses, strain-sensing devices were practically prepared and the performances of the trial products using the devices were investigated. As a result, a Coriolis mass flowmeter constructed from Ti-Cu-based bulk metallic glass pipe exhibits 28.5 times higher sensitivity than conventional flowmeters made from SUS316 pipe. A pressure sensor using a Zr-based bulk metallic glass diaphragm also exhibits 3.8 times higher sensitivity than the conventional sensor using SUS630 diaphragm. These results for the industrial products made of bulk metallic glasses are promising for future developments as industrial materials with high performance. (c) 2006 Elsevier B.V. All rights reserved.

RI Nishiyama, Nobuyuki/C-8228-2015; Amiya, Kenji/P-6132-2014; Inoue,

Akihisa/E-5271-2015

SN 0921-5093

PD MAR 25

PY 2007

VL 449

BP 79

EP 83

DI 10.1016/j.msea.2006.02.384

UT WOS:000245477800016

ER

PT J

AU Wang, JQ

Qin, JY

Gu, XN

Zheng, YF

Bai, HY

AF Wang, J. Q.

Qin, J. Y.

Gu, X. N.

Zheng, Y. F.

Bai, H. Y.

TI Bulk metallic glasses based on ytterbium and calcium

SO JOURNAL OF NON-CRYSTALLINE SOLIDS

AB We report the formation of a family of bulk metallic glasses (BMGs) based on rare earth element of ytterbium and alkaline earth element of calcium. The glass-forming ability, atomic packing density and corrosion behaviors of the BMGs show an extremum around the eutectic point with the change of the concentration of Yb and Ca. (C) 2010 Elsevier B.V. All rights reserved.

RI Wang, Jun-Qiang/G-5989-2010; Zheng, Yufeng/A-4146-2010; Wang,

Junqiang/C-2839-2015; Gu, Xuenan/F-5354-2011

OI Zheng, Yufeng/0000-0002-7402-9979; Wang, Junqiang/0000-0002-8066-6237;

SN 0022-3093

EI 1873-4812

PD FEB 1

PY 2011

VL 357

IS 3

BP 1232

EP 1234

DI 10.1016/j.jnoncrysol.2010.11.046

UT WOS:000288232500076

ER

PT J

AU Zhao, DQ

Zhang, Y

Pan, MX

Wang, WH

AF Zhao, DQ

Zhang, Y

Pan, MX

Wang, WH

TI The effects of iron addition on the class-forming ability and properties

of Zr-Ti-Cu-Ni-Be-Fe bulk metallic glass

SO MATERIALS TRANSACTIONS JIM

AB Zr-Ti-Cu-Ni-Be-Fe bulk metallic glasses and metallic glassy matrix composites were produced by water quenching method. The effect of Fe addition on glass forming ability, hardness, magnetic susceptibility and thermal stability of the alloys was investigated. It was found that the glass forming ability and the properties of the alloys were sensitive to Fe content. A single amorphous phase was obtained in the alloys up to 8 at%Fe addition. These bulk metallic glasses exhibited high thermal stability, wide super-cooled liquid region, and anomalous magnetic susceptibility chi versus temperature in the supercooled liquid region. Metallic glassy composites consisting of nanocrystalline FeZr2 particles were obtained in the alloys with more than 10 at%Fe addition.

RI ZHANG, Yong/B-7928-2009

OI ZHANG, Yong/0000-0002-6355-9923

SN 0916-1821

PD NOV

PY 2000

VL 41

IS 11

BP 1427

EP 1431

DI 10.2320/matertrans1989.41.1427

UT WOS:000166559500011

ER

PT J

AU Lee, JK

Kim, KB

Lee, MH

Kim, TS

Bae, JC

AF Lee, J. K.

Kim, K. B.

Lee, M. H.

Kim, T. S.

Bae, J. C.

TI Microstructure and mechanical properties of metallic glass/metallic

glass composites

SO JOURNAL OF ALLOYS AND COMPOUNDS

CT 14th International Sympoium on Metastable and Nano-Materials

CY AUG 26-30, 2007

CL Corfu, GREECE

AB we report microstructure and mechanical properties of bulk metallic glass (BMG)/metallic glass composites fabricated by mechanical alloying with subsequent consolidation process. The microstructural investigations of a bulk composite reveal that a submicron-scale layered structure with irregular interfaces consists of three amorphous phases in tornado-like morphology. Based on these results, poor plasticity of the metallic glass composite can be understood possibly due to the irregular interfacial morphology of the submicron-scale heterogeneous amorphous phases throughout the materials. (C) 2008 Elsevier B.V. All rights reserved.

OI LEE, MIN HA/0000-0001-6006-0628

SN 0925-8388

PD AUG 26

PY 2009

VL 483

IS 1-2

BP 286

EP 288

DI 10.1016/j.jallcom.2008.07.169

UT WOS:000270619600070

ER

PT J

AU Sheng, WB

AF Sheng, WB

TI Evaluation on the reliability of criterions for glass-forming ability of

bulk metallic glasses

SO JOURNAL OF MATERIALS SCIENCE

SN 0022-2461

EI 1573-4803

PD SEP

PY 2005

VL 40

IS 18

BP 5061

EP 5066

DI 10.1007/s10853-005-1098-5

UT WOS:000232059600066

ER

PT S

AU Babilas, R

Nowosielski, R

Pilarczyk, W

Dercz, G

AF Babilas, Rafal

Nowosielski, Ryszard

Pilarczyk, Wirginia

Dercz, Grzegorz

BE Stroz, D

Dercz, G

TI Structural, Magnetic and Crystallization Study of Fe-Based Bulk Metallic

Glasses

SO APPLIED CRYSTALLOGRAPHY XXII

SE Solid State Phenomena

CT 22nd Conference on Applied Crystallography

CY SEP 02-06, 2012

CL Targanice, POLAND

SP Univ Silesia, Inst Mat Sci, Polish Acad Sci, Comm Crystallog, Int Union Crystallog, JEOL, PANalyt, NanoMEGAS, LABSOFT

AB The work presents the structural, thermal and magnetic properties analysis of Fe72B20Si4Nb4 bulk metallic glasses in as-cast state and crystallization study of bulk amorphous alloy after annealing process. The studies were performed on bulk metallic glasses in of rods form with diameter of 1.5 and 2 mm. The structure analysis of the samples in as-cast state and phase analysis of studied alloy after annealing process was carried out by the X-ray diffraction (XRD) methods. Mossbauer spectroscopy (MS) was also used to investigate the local structure for studied bulk metallic glasses. Thermal properties associated with glass transition, onset and peak crystallization temperatures was examined by differential scanning calorimetry (DSC). The soft magnetic properties examination of tested material contained initial magnetic permeability and disaccommodation of magnetic permeability.

OI Dercz, Grzegorz/0000-0003-3682-7184

SN 1012-0394

PY 2013

VL 203-204

BP 288

EP +

DI 10.4028/www.scientific.net/SSP.203-204.288

UT WOS:000329078400065

ER

PT J

AU Bakai, AS

Bakai, SA

Mikhailovskii, IM

Neklyudov, IM

Stoev, PI

Macht, MP

AF Bakai, AS

Bakai, SA

Mikhailovskii, IM

Neklyudov, IM

Stoev, PI

Macht, MP

TI On the nature of the Kaiser effect in metallic glasses

SO JETP LETTERS

AB The Kaiser effect was observed in the measurement of acoustic emission (AE) during the course of uniaxial compression of the bulk samples of metallic glasses Zr41Ti14Cu12.5Ni10Be22.5 and Zr52.5Ti5Cu17.9Ni14.6Be22.5. The field-ion microscopy study of bulk Zr41Ti14Cu12.5Ni10Be22.5 shows that this glass has a polycluster structure. This fact allows one to reveal the nature of AE appearing during the course of deformation of metallic glasses and to interpret the observed Kaiser effect. The dislocations generated at the intercluster boundaries and moving through the glass bulk are the sources of AE. (C) 2002 MAIK "Nauka / Interperiodica".

OI Stoev, Petr/0000-0001-7942-5850

SN 0021-3640

PY 2002

VL 76

IS 4

BP 218

EP 221

DI 10.1134/1.1517388

UT WOS:000178625600006

ER

PT J

AU Huang, JC

Chu, JP

Jang, JSC

AF Huang, J. C.

Chu, J. P.

Jang, J. S. C.

TI Recent progress in metallic glasses in Taiwan

SO INTERMETALLICS

AB The recent research and development on metallic glasses in Taiwan over the past decade is reviewed in this paper. The major focus was to develop tougher bulk metallic glasses (BMGs), bulk metallic glass composites (BMGCs), and thin film metallic glasses (TFMGs), mostly in Zr and Mg based systems. Due to the Taiwan industry characteristics, metallic glasses are favored in the application for micro-electromechanical systems (MEMS), including micro- or nano-imprinting for optoelectronic devices and hologram patterns. (C) 2009 Elsevier Ltd. All rights reserved.

RI Huang, J./C-4276-2013

OI HUANG, Jacob Chih Ching/0000-0001-6843-3396

SN 0966-9795

EI 1879-0216

PD DEC

PY 2009

VL 17

IS 12

BP 973

EP 987

DI 10.1016/j.intermet.2009.05.004

UT WOS:000270623400001

ER

PT J

AU Zhu, SL

Xie, GQ

Qin, FX

Wang, XM

Inoue, A

AF Zhu, Shengli

Xie, Guoqiang

Qin, Fengxiang

Wang, Xinmin

Inoue, Akihisa

TI Ni- and Be-free Zr-based bulk metallic glasses with high glass-forming

ability and unusual plasticity

SO JOURNAL OF THE MECHANICAL BEHAVIOR OF BIOMEDICAL MATERIALS

AB We developed Ni- and Be-free Zr45+xCu40-xAl7Pd5Nb3 bulk metallic glasses with large glass-forming ability and unusual plasticity. The alloys have large critical diameters (larger than 10 mm) in a wide composition range (x=0-20). The Zr50Cu35Al7Pd5Nb3 and Zr55Cu30Al7Pd5 Nb-3 alloys exhibit the largest critical diameter (between 18 and 20 mm). The Zr45+xCu40-x Al7Pd5Nb3 bulk metallic glasses also have large plastic elongation in wide composition range (x=10-17). The Zr62Cu23Al7Pd5Nb3 bulk metallic glass exhibits significant plasticity (over 20% of plastic elongation). With increasing Zr content, the compressive strength decreases except for the Zr67Cu18Al7Pd5Nb3 alloy. The fragility parameters were calculated to evaluate the glass-forming ability and plasticity. The fragility exhibits more sensitive correlation with plasticity than glass-forming ability. The ZrCuAlPdNb bulk metallic glasses have high crystallization activation energies of above 300 kJ/mol. The ZrCuAlPdNb bulk metallic glasses are favorable for application to biomaterials. (C) 2012 Elsevier Ltd. All rights reserved.

RI BAI, JIE/D-7448-2016; Xie, Guoqiang/A-8619-2011; Inoue,

Akihisa/E-5271-2015; Zhu, Shengli/D-5281-2009

OI Zhu, Shengli/0000-0002-0190-2626

SN 1751-6161

PD SEP

PY 2012

VL 13

BP 166

EP 173

DI 10.1016/j.jmbbm.2012.04.011

UT WOS:000311469300016

PM 22898203

ER

PT J

AU Qiu, SB

Yao, KF

AF Qiu, Sheng-Bao

Yao, Ke-Fu

TI Crystallization kinetics of Zr41Ti14Cu12.5Ni10Be22.5 bulk metallic glass

in pulsing current pretreatment states

SO JOURNAL OF ALLOYS AND COMPOUNDS

AB The crystallization kinetics of Zr41Ti14Cu12.5Ni10Be22.5 bulk metallic glasses in as-cast state and two pulsing current pretreatment states have been studied by both Kissinger equation and JMA equation. it has been found that the pulsing current pretreatment has apparent effects on the glass transition activation energy and structural stability of bulk metallic glasses, resulting in the decrement of free volume and decreased incubation time. After carefully comparing the crystallization process of as-cast state and pulsing current pretreatment states, no evidences of different crystallization product or phase transformation are found. Furthermore, the interior influences of high-density pulsing current on the glass transformation and crystallization behavior of bulk metallic glasses are discussed as well. (C) 2008 Elsevier B.V. All rights reserved.

SN 0925-8388

EI 1873-4669

PD MAY 5

PY 2009

VL 475

IS 1-2

BP L5

EP L8

DI 10.1016/j.jallcom.2008.07.056

UT WOS:000265911700002

ER

PT J

AU Qin, ZX

Wang, XJ

Zhang, HF

Wang, ZQ

Hu, ZQ

AF Qin Zuoxiang

Wang Xiaojing

Zhang Haifeng

Wang Zhongquang

Hu Zhuangqi

TI FRICTION WELDING OF Zr55Al10Ni5Cu30 BULK METALLIC GLASS

SO ACTA METALLURGICA SINICA

AB As a new kind of engineering material, the metallic glass can be used widely due to its excellent properties such as high strength, hardness and elastic energy and low corrosion resistance. The size of metallic glasses is generally small, which is the main limitation for their application. How to prepare a larger size metallic glass becomes one of main targets in metallic glasses research. To prepare larger size metallic glasses, one method is to optimize their chemical compositions, and another is to join small size metallic glasses together by welding. In this paper, the Zr55Al10Ni5Cu30 bulk metallic glass (BMG) has been successfully jointed by friction welding under the conditions of rotational speed from 4x 10(3) to 5x 10(3) r/min, friction pressure from 80 to 100 MPa, friction time from 0.2 to 0.4 s, upsetting pressure 200 MPa and upsetting time 2 s. The welded joint has been examined using SEM, XRD and TEM, and it is proved that the welded zone still keeps an amorphous structure. The plasticity of this metallic glass has strong temperature sensitivity near the glass transition temperature T-g. Above T-g, the metallic glass possesses a good plasticity which is necessary for friction welding of metallic glasses.

SN 0412-1961

PD MAY

PY 2009

VL 45

IS 5

BP 620

EP 624

UT WOS:000266627900018

ER

PT J

AU Fan, GJ

Zhao, JC

Liaw, PK

AF Fan, G. J.

Zhao, Ji-Cong

Liaw, P. K.

TI A four-step approach to the multicomponent bulk-metallic glass formation

SO JOURNAL OF ALLOYS AND COMPOUNDS

AB A four-step approach to fabricate the multicomponent bulk-metallic glasses is presented (1) deep eutectics, (2) mutual solubility, (3) atomic-size- and thermodynamic effects, and (4) micro-alloying Based on this alloying strategy, a detailed route to fabricate NI-based bulk-metallic glasses is outlined (C) 2010 Elsevier B V All rights reserved.

SN 0925-8388

PD MAY 14

PY 2010

VL 497

IS 1-2

BP 24

EP 27

DI 10.1016/j.jallcom.2010.01.162

UT WOS:000278744600013

ER

PT J

AU Zhang, W

Fang, CF

Li, YH

AF Zhang, Wei

Fang, Canfeng

Li, Yanhui

TI Ferromagnetic Fe-based bulk metallic glasses with high thermoplastic

formability

SO SCRIPTA MATERIALIA

AB New multi-component Fe-based bulk metallic glasses with excellent thermoplastic formability and good soft magnetic and mechanical properties were developed based on an Fe75P10C10B5 alloy. These glasses also exhibit a wide supercooled liquid region of similar to 89 K, a low glass transition temperature of similar to 705 K, low viscosity of similar to 10(7) Pa s in the supercooled liquid state and high glass-forming ability, with critical sample diameters exceeding 4 mm. (c) 2013 Acta Materialia Inc. Published by Elsevier Ltd. All rights reserved.

SN 1359-6462

PD JUL

PY 2013

VL 69

IS 1

BP 77

EP 80

DI 10.1016/j.scriptamat.2013.03.003

UT WOS:000319545100020

ER

PT J

AU Egami, T

AF Egami, T

TI Atomic transport in amorphous metals

SO ZEITSCHRIFT FUR METALLKUNDE

AB A novel mechanism of atomic transport in amorphous metallic alloys, or metallic glasses, is proposed based upon the fluctuations in the local structure. The proposed mechanism is very different from those in crystalline solids and from the free volume model, and is characterized by the bond-exchange action triggered by the local topological instability of the atomic environment. The implications of this mechanism on the liquid fragility and bulk metallic glass formation are discussed.

SN 0044-3093

PD OCT

PY 2002

VL 93

IS 10

BP 1071

EP 1076

DI 10.3139/146.021071

UT WOS:000179591100021

ER

PT J

AU Li, S

Wang, RJ

Pan, MX

Zhao, DQ

Wang, WH

AF Li, S

Wang, RJ

Pan, MX

Zhao, DQ

Wang, WH

TI Bulk metallic glasses based on heavy rare earth dysprosium

SO SCRIPTA MATERIALIA

AB DyYAlCo bulk metallic glasses (BMGs) with high thermal stability have been obtained. The origin of the high thermal stability of these new BMGs is discussed, and it is apparent that the high bulk modulus of the base component is predominantly responsible for this feature. (c) 2005 Acta Materialia Inc. Published by Elsevier Ltd. All rights reserved.

SN 1359-6462

PD DEC

PY 2005

VL 53

IS 12

BP 1489

EP 1492

DI 10.1016/j.scriptamat.2005.07.036

UT WOS:000232700500031

ER

PT J

AU Liu, LF

Cai, ZP

Li, HQ

Guo, SB

Zhang, GY

AF Liu, L. F.

Cai, Z. P.

Li, H. Q.

Guo, S. B.

Zhang, G. Y.

TI The correlation between the pressure sensitivity and the fragility/glass

transition temperature in bulk metallic glasses

SO JOURNAL OF NON-CRYSTALLINE SOLIDS

AB The correlations between the pressure sensitivity and the fragility/glass transition temperature have been addressed in various bulk metallic glasses in the present work. The results demonstrate that the pressure sensitivity of bulk metallic glasses is closely related to both the fragility index (m) and the glass transition temperature (T-g). The physical origin of the correlations has been discussed from their disordered structure, which is determined by the glass transition behavior and the glass transition temperature. Crown Copyright (C) 2011 Published by Elsevier BM. All rights reserved.

SN 0022-3093

EI 1873-4812

PD JUL 15

PY 2011

VL 357

IS 15

BP 3033

EP 3035

DI 10.1016/j.jnoncrysol.2011.03.024

UT WOS:000292947500040

ER

PT S

AU Dong, C

Wang, Q

Qiang, JB

Wang, YM

Han, G

Wu, J

Li, YH

Cheng, X

Zhu, CL

Chen, H

AF Dong, C.

Wang, Q.

Qiang, J. B.

Wang, Y. M.

Han, G.

Wu, J.

Li, Y. H.

Cheng, X.

Zhu, C. L.

Chen, H.

BE Popel, PS

TI A cluster line approach for composition rules of quasicrystals and bulk

metallic glasses

SO 13TH INTERNATIONAL CONFERENCE ON LIQUID AND AMORPHOUS METALS

SE JOURNAL OF PHYSICS CONFERENCE SERIES

CT 13th Conference on Liquid and Amorphous Metals

CY JUL 08-14, 2007

CL Ekaterinburg, RUSSIA

AB This paper analyzes the structure and composition characteristics of ternary quasicrystals and bulk metalic glasses from the viewpoint of atomic clusters. It is pointed out that quasicrystals and bulk metallic glasses satisfy a cluster line rule. The cluster line refers to a straight composition line linking a specific cluster to the third element in a ternary alloy phase diagram. A ternary quasicrystal or bulk metallic glass composition is located at the intersection point of two cluster lines. Ternary quasicrystal and bulk metallic glass compositions can be the expressed with a cluster-plus-glue-atom model which can predict new ternary bulk metallic glasses.

SN 1742-6588

PY 2008

VL 98

BP U70

EP U78

DI 10.1088/1742-6596/98/1/012015

UT WOS:000259363800015

ER

PT S

AU Hays, CC

Kim, CP

Johnson, WL

AF Hays, CC

Kim, CP

Johnson, WL

BE Eckert, J

Schlorb, H

Schultz, L

TI Enhanced plasticity of bulk metallic glasses containing ductile phase

dendrite dispersions

SO METASTABLE, MECHANICALLY ALLOYED AND NANOCRYSTALLINE MATERIALS, PTS 1

AND 2

SE MATERIALS SCIENCE FORUM

CT International Symposium on Metastable, Mechanically Alloyed and

Nanocrystalline Materials (ISMANAM-99)

CY AUG 30-SEP 03, 1999

CL DRESDEN, GERMANY

SP Deutsch Forech Gemeinsch, Inst Festkorper & Werkstofforsch Dresden, Sachsisches Minist Wissensch & Kunst, Fritsch GmbH, Stadtsparkasse Dresden, ZOZ GmbH

AB Results of x-ray diffraction, calorimetric, and mechanical property measurements are presented for a composite material based on bulk metallic glass forming compositions in the Zr-Ti-Cu-Ni-Be alloy system. On cooling from the liquid state primary dendrite growth and solute partitioning yield an as-cast microstructure consisting of ductile Ti-Zr-Nb beta-phase dendrites in a Zr-Ti-Nb-Cu-Ni-Be bulk metallic glass matrix. The bulk glass matrix comprises about 75 % of the specimen volume. The calorimetric data indicate that this glassy matrix exhibits thermophysical data similar to that of other Be-containing bulk glasses. The in-situ formed microstructure results in dramatically improved mechanical behavior under unconfined loading.

RI Hays, Charles/P-8021-2015

OI Hays, Charles/0000-0002-0420-1761

SN 0255-5476

BN 0-87849-858-3

PY 2000

VL 343-3

BP 191

EP 196

DI 10.4028/www.scientific.net/MSF.343-346.191

PN 1&2

UT WOS:000089403900031

ER

PT B

AU Murty, BS

Yeh, JW

Ranganathan, S

AF Murty, BS

Yeh, JW

Ranganathan, S

TI High-Entropy Alloys

SO HIGH-ENTROPY ALLOYS

BN 978-0-12-800526-2; 978-0-12-800251-3

PY 2014

BP 1

EP 204

UT WOS:000341158200013

ER

PT J

AU Zeng, YQ

Nishiyama, N

Yamamoto, T

Inoue, A

AF Zeng, Yuqiao

Nishiyama, Nobuyuki

Yamamoto, Tokujiro

Inoue, Akihisa

TI Ni-Rich Bulk Metallic Glasses with High Glass-Forming Ability and Good

Metallic Properties

SO MATERIALS TRANSACTIONS

AB Glass-forming ability, thermal behavior and mechanical properties of Nig(80-x)Pd(x)P(20) (10at% <= x <= 35 at%) glassy alloys have been investigated. The alloys with Pd contents higher than 25 at% exhibit high glass-forming ability, as is evidenced from the formation of a cylindrical Ni50Pd30P10 glassy rod with a diameter of 21 mm. The Ni-rich bulk metallic glasses show relatively high strength in compression and tension tests. More notably, plastic strains exceeding 2% were obtained for all the metallic glasses at room temperature in compression test. [doi:10.2320/matertrans.MRA2008453]

RI Inoue, Akihisa/E-5271-2015; Nishiyama, Nobuyuki/C-8228-2015; Yamamoto,

Tokujiro/A-8827-2011

OI Yamamoto, Tokujiro/0000-0002-4292-9446

SN 1345-9678

EI 1347-5320

PD OCT

PY 2009

VL 50

IS 10

BP 2441

EP 2445

DI 10.2320/matertrans.MRA2008453

UT WOS:000272070600016

ER

PT J

AU Fan, C

Liu, CT

Yan, HG

AF Fan, Cang

Liu, C. T.

Yan, H. G.

TI MECHANICAL PROPERTIES OF BULK METALLIC GLASSES AT CRYOGENIC TEMPERATURES

SO MODERN PHYSICS LETTERS B

AB Metallic glasses (amorphous alloys) consist of atomic clusters, interconnecting zones and free volume. The atomic clusters are connected together, resulting in the formation of a rigid skeleton through the interconnecting zones. Some metallic glasses even contain crystalline structures at the nanoscale. Even though there is supposed to be no structural change at temperatures below the glass transition temperature, metallic glasses exhibit different mechanical behaviors at cryogenic temperatures. Contrary to crystalline materials, the strength and ductility of some metallic glasses and their composites both show a significant increase with decreasing temperature in the cryogenic temperature range. This paper briefly reviews these phenomena.

OI Liu, Chain Tsuan/0000-0001-7888-9725

SN 0217-9849

EI 1793-6640

PD SEP 10

PY 2009

VL 23

IS 23

BP 2703

EP 2722

DI 10.1142/S0217984909020928

UT WOS:000269777500010

ER

PT J

AU Hu, Y

Chan, KC

Liu, L

Yang, YZ

AF Hu, Y.

Chan, K. C.

Liu, L.

Yang, Y. Z.

TI Monte Carlo simulation of dual magnetic phase behavior in bulk metallic

glasses

SO JOURNAL OF MAGNETISM AND MAGNETIC MATERIALS

AB Taking magnetic clusters as magnetic units, the Monte Carlo simulation method was employed to investigate the behavior of dual magnetic phase in bulk metallic glasses (BMGs). The simulated magnetic behavior of the two-cluster BMG system is consistent with the experimental findings of a Nd-based bulk metallic glass with dual magnetic phases.(C) 2010 Elsevier B.V. All rights reserved.

RI Chan, K.C./A-2311-2014

OI Chan, K.C./0000-0002-6173-5532

SN 0304-8853

PD SEP

PY 2010

VL 322

IS 17

BP 2567

EP 2570

DI 10.1016/j.jmmm.2010.03.021

UT WOS:000277700600023

ER

PT S

AU Helson, JA

Missirlis, Y

AF Helson, JA

Missirlis, Y

TI Biomaterials: A Tantalus Experience

SO BIOMATERIALS: A TANTALUS EXPERIENCE

SE Biological and Medical Physics Biomedical Engineering

SN 1618-7210

BN 978-3-642-12531-7

PY 2010

BP 1

EP 340

DI 10.1007/978-3-642-12532-4

UT WOS:000284507600003

ER

PT J

AU Gupta, PK

Miracle, DB

AF Gupta, Prabhat K.

Miracle, Daniel B.

TI A topological basis for bulk glass formation

SO ACTA MATERIALIA

AB The shifting of bulk metallic glass compositions from their respective nearest eutectics is rationalized in terms of the variation of the glass transition temperature with composition near the eutectic. The composition dependence of the glass transition temperature is established by considering the competition between the internal degrees of freedom and the bond constraints between unlike atoms. The average bond constraint model requires as input only information that is readily available for any system of interest and provides estimates of. (i) the minimum solute concentration needed to form bulk metallic glass; (ii) the trend in the glass transition temperature as a function of composition; and (iii) the shift of the bulk metallic glass composition from the eutectic composition. (C) 2007 Published by Elsevier Ltd on behalf of Acta Materialia Inc.

SN 1359-6454

PD AUG

PY 2007

VL 55

IS 13

BP 4507

EP 4515

DI 10.1016/j.actamat.2007.04.026

UT WOS:000248436400027

ER

PT J

AU Louzguine-Luzgin, V

Miracle, DB

Inoue, A

AF Louzguine-Luzgin, V.

Miracle, Daniel B.

Inoue, Akihisa

TI Intrinsic and Extrinsic Factors Influencing the Glass-Forming Ability of

Alloys

SO ADVANCED ENGINEERING MATERIALS

RI Inoue, Akihisa/E-5271-2015

OI Louzguine-Luzgin, Dmitri/0000-0001-5716-4987

SN 1438-1656

PD NOV

PY 2008

VL 10

IS 11

BP 1008

EP 1015

DI 10.1002/adem.200800134

UT WOS:000261933400004

ER

PT J

AU Zhang, W

Zhang, QS

Inoue, A

AF Zhang, Wie

Zhang, Qingsheng

Inoue, Akihisa

TI Synthesis and Mechanical Properties of New Cu-Zr-based Glassy Alloys

with high Glass-Forming Ability

SO ADVANCED ENGINEERING MATERIALS

RI Inoue, Akihisa/E-5271-2015; zhang, qingsheng/A-4851-2010

SN 1438-1656

PD NOV

PY 2008

VL 10

IS 11

BP 1034

EP 1038

DI 10.1002/adem.200800130

UT WOS:000261933400009

ER

PT J

AU Li, JL

Zhao, DQ

Pan, MX

Wang, WH

AF Li, J. L.

Zhao, D. Q.

Pan, M. X.

Wang, W. H.

TI Dissoluble and degradable CaLi-based metallic glasses

SO JOURNAL OF NON-CRYSTALLINE SOLIDS

AB We study the degradable and dissoluble features of a Ca-Li-Mg-Zn bulk metallic glass in pure water at room temperature. A remarkable degradable feature of the metallic glasses is that the degradation is controllable by changing the composition and components. The degradable metallic glasses with superior combined properties of polymer-like thermal plasticity at low temperature (40-70 degrees C), the ultralow elastic moduli comparable to that of human bones, and ultralow density (<2 g/cm(3)) in known metallic glasses to date, and good machinability at a lower temperature in the supercooled liquid region could have potential applications. The metallic glasses also provide a model system to study the corrosion behavior in glasses. (C) 2010 Elsevier B.V. All rights reserved.

RI BAI, JIE/D-7448-2016

SN 0022-3093

EI 1873-4812

PD JAN 1

PY 2011

VL 357

IS 1

BP 236

EP 239

DI 10.1016/j.jnoncrysol.2010.09.041

UT WOS:000286173300043

ER

PT S

AU Helsen, JA

Missirlis, Y

AF Helsen, Jozef A.

Missirlis, Yannis

BA Helson, JA

Missirlis, Y

BF Helson, JA

Missirlis, Y

TI Biomaterials A Tantalus Experience

SO BIOMATERIALS: A TANTALUS EXPERIENCE

SE Biological and Medical Physics Biomedical Engineering

SN 1618-7210

BN 978-3-642-12531-7

PY 2010

BP 1

EP 332

D2 10.1007/978-3-642-12532-4

UT WOS:000284507600001

ER

PT J

AU Zhao, ZF

Wen, P

Wang, RJ

Zhao, DQ

Pan, MX

Wang, WH

AF Zhao, ZF

Wen, P

Wang, RJ

Zhao, DQ

Pan, MX

Wang, WH

TI Formation and properties of Pr-based bulk metallic glasses

SO JOURNAL OF MATERIALS RESEARCH

AB The data on the compositional dependence of glass-forming ability, glass transition, and properties of bulk metallic glasses (BMGs) are important for understanding the nature and glass-forming ability of metallic glasses and for their application. In this paper, we report the formation of rare-earth-based Pr-(Cu,Ni)-Al pseudo-ternary BMGs with a large bulk glass-forming composition range and distinct glass transition. The compositional dependence of glass-forming ability, glass transition, and properties were systematically studied. The contrasting effects of Al and Pr on glass formation and glass transition, unique elastic properties, and phonon softening of the BMGs are discussed from the structural point of view.

RI Zhao, Zuofeng/B-1297-2010

OI Zhao, Zuofeng/0000-0002-0862-8471

SN 0884-2914

PD FEB

PY 2006

VL 21

IS 2

BP 369

EP 374

DI 10.1557/JMR.2006.0067

UT WOS:000235462000007

ER

PT J

AU Tang, MB

Bai, HY

Pan, MX

Zhao, DQ

Wang, WH

AF Tang, MB

Bai, HY

Pan, MX

Zhao, DQ

Wang, WH

TI Bulk metallic superconductive La10Cu20Ni10Al10 glass

SO JOURNAL OF NON-CRYSTALLINE SOLIDS

AB A bulk metallic superconductive La60Cu20Ni10Al10 glass with highly glass forming ability and large supercooled liquid temperature region is developed. The superconductive critical temperature determined by the electrical resistance and the specific heat measurements is 2.5 K. The obtained electron-phonon interaction parameter demonstrates that the bulk metallic glass is an intermediate coupling superconductor. The new nonmagnetic rare-earth based glass offers an ideal material for studying the superconductivity and other low temperature properties in the metallic glasses. (c) 2005 Published by Elsevier B.V.

SN 0022-3093

EI 1873-4812

PD SEP 1

PY 2005

VL 351

IS 30-32

BP 2572

EP 2575

DI 10.1016/j.jnoncrysol.2005.06.039

UT WOS:000231930800030

ER

PT J

AU Schroeder, V

Gilbert, CJ

Ritchie, RO

AF Schroeder, V

Gilbert, CJ

Ritchie, RO

TI Effect of aqueous environment on fatigue-crack propagation behavior in a

Zr-based bulk amorphous metal

SO SCRIPTA MATERIALIA

RI Ritchie, Robert/A-8066-2008

OI Ritchie, Robert/0000-0002-0501-6998

SN 1359-6462

PD APR 9

PY 1999

VL 40

IS 9

BP 1057

EP 1061

DI 10.1016/S1359-6462(99)00067-6

UT WOS:000080582500014

ER

PT J

AU Haruyama, O

AF Haruyama, Osami

TI Thermodynamic approach to free volume kinetics during isothermal

relaxation in bulk Pd-Cu-Ni-P-20 glasses

SO INTERMETALLICS

CT 6th International Workshop on Advanced Intermetallic and Metallic

Materials

CY OCT 09-15, 2005

CL Yangzhou, PEOPLES R CHINA

SP Natl Nat Sci Fdn China, Minist Sci & Technol China, Nanjing Univ Sci & Technol, Univ Sci & Technol Beijing, Oak Ridge Natl Lab, Shenyang Natl Lab Mat Sci, Cent S Univ, Harbin Inst Technol, Yangzhou City Govt

AB Isothermal relaxation behavior just under glass transition region was investigated for bulk Pd40Ni40P20 and Pd42.5Cu30Ni7.5P20 metallic glasses. The densification of the bulk sample during relaxation was examined directly by density measurement using conventional Archimedean technique. The density of as-quenched Pd40Ni40P20 increased monotonously with time. From the density data of Pd40Ni40P20 glass, the free volume relaxation was examined and the kinetics was well described by a stretched exponential function with Kohlrausch exponent less than unity. These glasses also showed a clear two-step relaxation that may be a feature peculiar to bulk metallic glasses. (C) 2006 Elsevier Ltd. All rights reserved.

SN 0966-9795

PD MAY-JUN

PY 2007

VL 15

IS 5-6

BP 659

EP 662

DI 10.1016/j.intermet.2006.10.040

UT WOS:000246739400010

ER

PT J

AU Soubeyroux, JL

Pelletier, JM

de la Bathie, RP

AF Soubeyroux, JL

Pelletier, JM

de la Bathie, RP

TI In situ crystallization of Zirconium-based bulk metallic glasses

SO PHYSICA B

CT 2nd European Conference on Neutron Scattering (ECNS 99)

CY SEP 01-04, 1999

CL BUDAPEST, HUNGARY

SP European Neutron Scattering Assoc, Budapest Neutron Ctr, Atominstitut Vienna, Forsch Zentrum Julich, HMI Berlin, ILL Grenoble, IRI Delft, ISIS Didcot, LLB Saclay, NFL Sudsvik, NPI Rez, Nuclear & Technol Inst Lisbon, PSI Villigen, Riso Natl Lab, TU Munich, Australian Nuclear Sci & Technol Org, European Phys Soc, Hungarian Acad Sci, Hungarian Minist Educ, Neutronentechn Komponenten GmbH, Ordela Inc, Oxford Instruments, Ingenieurburo Stronciwilk, Swiss Neutron, Neutron Round Table, OMFB, Natl Comm Technol Dev, Paks Nuclear Power Plant, Adv Ceram Copr, CERCA, Eurisys Mesures, Goodfellow Cambridge Ltd, KJ Lesker Co, Mirrotron Ltd

AB Zr-based bulk metallic glasses were investigated by DSC and in-situ neutron diffraction. DSC and neutron studies were performed at different heating rates and the evolution of the characteristic temperatures, glass transition were determined as a function of dT/dt. The first phase appearing at crystallisation can be correlated to a quasicrystalline phase. (C) 2000 Elsevier Science B.V. All rights reserved.

SN 0921-4526

PD MAR

PY 2000

VL 276

BP 905

EP 906

DI 10.1016/S0921-4526(99)01585-9

UT WOS:000086413000414

ER

PT J

AU Senkov, ON

Scott, JM

AF Senkov, ON

Scott, JM

TI Specific criteria for selection of alloy compositions for bulk metallic

glasses

SO SCRIPTA MATERIALIA

AB New criteria for selection of alloy compositions for bulk metallic glasses are formulated and used to produce a series of Ca-based bulk glassy alloys. (C) 2003 Acta Materialia Inc. Published by Elsevier Ltd. All rights reserved.

RI Senkov, Oleg/C-7197-2012

OI Senkov, Oleg/0000-0001-5587-415X; Senkov, Oleg/0000-0002-9336-3702

SN 1359-6462

PD FEB

PY 2004

VL 50

IS 4

BP 449

EP 452

DI 10.1016/j.scriptamat.2003.11.004

UT WOS:000187235800010

ER

PT S

AU Lu, SD

Sun, SC

Huang, XX

Zhu, XP

Li, XD

Tu, GF

Huang, SD

AF Lu, Shuaidan

Sun, Shuchen

Huang, Xiaoxiao

Zhu, Xiaoping

Li, Xiaodong

Tu, Ganfeng

Huang, Shaodong

BE Yarlagadda, P

TI Preparation of Zr52.8Cu29.1Ni7.3Al9.8Y1 bulk metallic glass by

hereditary process

SO PROCEEDINGS OF THE 3RD INTERNATIONAL CONFERENCE ON MATERIAL, MECHANICAL

AND MANUFACTURING ENGINEERING

SE AER-Advances in Engineering Research

CT 3rd International Conference on Material, Mechanical and Manufacturing

Engineering (IC3ME)

CY JUN 27-28, 2015

CL Guangzhou, PEOPLES R CHINA

AB Zr-based bulk metallic glass possesses the highest potential as a structural material among metallic glasses. However, its potential application has been restricted by a number of issues, such as fragility, small size and difficult fabrication into different shapes. In this paper, an attempt is made to evaluate the possibility of preparing a solid Zr52.8Cu29.1Ni7.3Al9.8Y1 bulk metallic glass by using binary precursors. It is found that the GFA and the stability of Zr52.8Cu29.1Ni7.3Al9.8Y1 bulk metallic glasses prepared in the hereditary process increase with the increasing quenching temperature, while the supercooled liquid region Delta T-x increase from 55 K to 83 K. The flexural strength increases to 1823 MPa at a quenching temperature of 1723 K.

SN 2352-5401

BN 978-94-6252-100-1

PY 2015

VL 27

BP 1637

EP 1642

UT WOS:000367097600316

ER

PT J

AU Yavari, AR

Nikolov, N

Nishiyama, N

Zhang, T

Inoue, A

Uriarte, JL

Heunen, G

AF Yavari, AR

Nikolov, N

Nishiyama, N

Zhang, T

Inoue, A

Uriarte, JL

Heunen, G

TI The glass transition of bulk metallic glasses studied by real-time

diffraction in transmission using high-energy synchrotron radiation

SO MATERIALS SCIENCE AND ENGINEERING A-STRUCTURAL MATERIALS PROPERTIES

MICROSTRUCTURE AND PROCESSING

CT 11th International Conference on Rapidly Quenched and Metastable

Materials

CY AUG 25-30, 2002

CL Univ Oxford, Dept Mat, Oxford, ENGLAND

HO Univ Oxford, Dept Mat

AB The "isentropic" glass transition T-g in metallic glasses can be studied by calorimetric techniques which show an endothermic event corresponding to a specific heat DeltaC(p), difference between the glassy and supercooled liquid states. In this work we show that isostructural thermal expansion coefficient alpha(th) is easily obtained by diffraction data hence an "isochoric T-g" should be detectable by diffraction. Real-time diffraction studies of phenomena occurring on a time scale of tau(t), can only be effective if acquisition time tau(a), << tau(t), The problem was that near Tg, metallic glasses are unstable and crystallize during real-time diffraction experiments. This limitation does not occur in diffraction experiments on bulk metallic glasses with large supercooled regions DeltaT = T-x - T-g using high energy, high intensity monochromatic light from synchrotron sources that allow tau(a) << tau(g). Under such conditions, the variation of alpha(th), the volume coefficient of thermal expansion has been measured for the first time by diffraction near T-g for a number of bulk metallic glasses. A clear Aceth has been detected in the same glass transition temperature range of DeltaC(p) for Pd-based metal-metalloid bulk glasses but not in Zr-based metal-metal type bulk glasses where Aceth occurs well above the calorimetric T-g. (C) 2003 Published by Elsevier B.V.

RI Zhang, Tao/O-4911-2014; yavari, alain/E-8192-2010; Inoue,

Akihisa/E-5271-2015; Nishiyama, Nobuyuki/C-8228-2015

SN 0921-5093

PD JUL 15

PY 2004

VL 375

SI SI

BP 709

EP 712

DI 10.1016/j.msca.2003.10.224

UT WOS:000223329700133

ER

PT J

AU Spaepen, F

AF Spaepen, F

TI Homogeneous flow of metallic glasses: A free volume perspective

SO SCRIPTA MATERIALIA

AB The free volume approach to the temperature-dependence of the liquid viscosity, structural relaxation, and deformation-induced softening of the glass is reviewed and assessed based on the results of recent creep data on a bulk metallic glass. (c) 2005 Acta Materialia Inc. Published by Elsevier Ltd. All rights reserved.

SN 1359-6462

PD FEB

PY 2006

VL 54

IS 3

BP 363

EP 367

DI 10.1016/j.scriptamat.2005.09.046

UT WOS:000233495600009

ER

PT J

AU Ren, HT

Pan, J

Chen, Q

Chan, KC

Liu, Y

Liu, L

AF Ren, H. T.

Pan, J.

Chen, Q.

Chan, K. C.

Liu, Y.

Liu, L.

TI Enhancement of plasticity and toughness in monolithic Zr-based bulk

metallic glass by heterogeneous microstructure

SO SCRIPTA MATERIALIA

AB A significant enhancement of plasticity and toughness was achieved by increasing Zr content but decreasing Cu content in the Zr-Cu-Ni-Al-Ta bulk metallic glass system. It was found that higher Zr content induces strong icosahedral medium-range order clusters in amorphous phase which, in turn, cause heterogeneous distribution of free volume and eventually lead to the enhancement of plasticity and toughness of the monolithic bulk metallic glasses. (C) 2010 Acta Materialia Inc. Published by Elsevier Ltd. All rights reserved.

RI Pan, Jie/L-3928-2017; Chan, K.C./A-2311-2014; Chen, Qi/A-6167-2010

OI Pan, Jie/0000-0001-5465-0736; Chan, K.C./0000-0002-6173-5532;

SN 1359-6462

PD APR

PY 2011

VL 64

IS 7

BP 609

EP 612

DI 10.1016/j.scriptamat.2010.11.047

UT WOS:000287272100004

ER

PT J

AU Senkov, ON

Scott, JM

Miracle, DB

AF Senkov, O. N.

Scott, J. M.

Miracle, D. B.

TI Development of low density Ca-Mg-Al-based bulk metallic glasses

SO MATERIALS TRANSACTIONS

CT 5th International Conference on Bulk Metallic Glasses

CY OCT 01-05, 2006

CL Osaka Univ, Awaji Isl, JAPAN

SP Minist Educ, Culture, Sports, Sci & Technol, Inst Mat Res, Tohoku Univ, Japan Soc Promot Sci, Natl Inst Mat Sci, Hyogo Int Assoc

HO Osaka Univ

AB Low density Ca-Mg-Al-based bulk metallic glasses containing additionally Cu and Zn, were produced by a copper mold casting method as wedge-shaped samples with thicknesses varying from 0.5 mm to 10 mm. The compositions of the alloys were selected using recently developed specific criteria for glass formation. A structural assessment using the efficient cluster packing model was applied and showed a good ability to represent these glasses. Thermal properties of the new metallic glasses. such as the glass transition, crystallization and melting temperatures, as well as heats of crystallization and melting are reported. The effect of the alloy composition on glass forming ability is discussed.

RI Senkov, Oleg/C-7197-2012

OI Senkov, Oleg/0000-0001-5587-415X; Senkov, Oleg/0000-0002-9336-3702

SN 1345-9678

EI 1347-5320

PD JUL

PY 2007

VL 48

IS 7

BP 1610

EP 1616

DI 10.2320/matertrans.MJ200731

UT WOS:000248743100008

ER

PT J

AU Yoshida, S

Sangleboeuf, JC

Rouxel, T

AF Yoshida, S

Sangleboeuf, JC

Rouxel, T

TI Quantitative evaluation of indentation-induced densification in glass

SO JOURNAL OF MATERIALS RESEARCH

AB To estimate the ratio of densification to Vickers indentation volume, three-dimensional images of Vickers indentations on several glasses, including silicate glasses and bulk metallic glass (BMG), were obtained before and after annealing using an atomic force microscope. Large volume recovery of Vickers indentation by annealing was observed for all glasses but BMG. Following previous studies, this recovered volume almost corresponded to the densified volume under a Vickers indenter, and the compositional dependence of densification was discussed. The ratios of densification to the total indentation volume for silica and soda-lime glasses were 92% and 61%, respectively. It was concluded that densification was a general property for silicate glasses and that the ratios of densification to the total indentation volume for all the glasses correlated well with Poisson's ratios of the glasses.

RI Sangleboeuf, Jean-Christophe/N-7588-2014

SN 0884-2914

PD DEC

PY 2005

VL 20

IS 12

BP 3404

EP 3412

DI 10.1557/JMR.2005.0418

UT WOS:000233628600029

ER

PT J

AU Zhao, LZ

Xue, RJ

Wang, WH

Bai, HY

AF Zhao, Lin-Zhi

Xue, Rong-Jie

Wang, Wei-Hua

Bai, Hai-Yang

TI LaGa-based bulk metallic glasses

SO CHINESE PHYSICS B

AB We report the formation of LaGa-based bulk metallic glasses. Ternary La-Ga-Cu glassy rods of 2-3 mm in diameter can be easily formed in a wide composition range by the conventional copper mold casting method. With minor addition of extra elements such as Co, Ni, Fe, Nb, Y, and Zr, the critical diameter of the full glassy rods of the La-Ga-Cu matrix can be markedly enhanced to at least 5 mm. The characteristics and properties of these new LaGa-based bulk metallic glasses with excellent glass formation ability and low glass transition temperature are model systems for fundamental issues investigation and could have some potential applications in micromachining field.

SN 1674-1056

EI 1741-4199

PD JAN

PY 2017

VL 26

IS 1

AR 018106

DI 10.1088/1674-1056/26/1/018106

UT WOS:000396114200002

ER

PT J

AU Li, Y

Bai, HY

Wang, WH

AF Li, Yong

Bai, H. Y.

Wang, W. H.

TI Low-temperature specific-heat anomalies associated with the boson peak

in CuZr-based bulk metallic glasses

SO PHYSICAL REVIEW B

AB We report the pronounced low-temperature specific-heat C-p anomalies associated with the boson peak in the new CuZr-based bulk metallic glasses. The origin of the C-p anomalies in the atomic glasses is interpreted with the harmonic localized mode based on the dense-packed atomic clusters structural model of metallic glass. The results might have important implications for understanding the origin of the boson peak and the structural features of metallic glasses.

SN 1098-0121

PD AUG

PY 2006

VL 74

IS 5

AR 052201

DI 10.1103/PhysRevB.74.052201

UT WOS:000240238400003

ER

PT S

AU Fukumoto, S

Minami, M

Soeda, A

Matsushima, M

Takahashi, M

Yokoyama, Y

Fujimoto, K

AF Fukumoto, S.

Minami, M.

Soeda, A.

Matsushima, M.

Takahashi, M.

Yokoyama, Y.

Fujimoto, K.

GP IOP

TI Microstructural development at weld interface between Zr-based glassy

alloy and stainless steel by resistance microwelding

SO INTERNATIONAL SYMPOSIUM ON MATERIALS SCIENCE AND INNOVATION FOR

SUSTAINABLE SOCIETY: ECO-MATERIALS AND ECO-INNOVATION FOR GLOBAL

SUSTAINABILITY (ECO-MATES 2011)

SE Journal of Physics Conference Series

CT International Symposium on

Materials-Science-and-Innovation-for-Sustainable-Society - Eco-Materials

and Eco-Innovation for Global Sustainability (ECO-MATES)/21st Iketani

Conference

CY NOV 28-30, 2011

CL Osaka, JAPAN

SP Osaka Univ, Joining & Welding Res Inst (JWRI), Osaka Univ, Ctr Environm Innovat Design Sustainabil (CEIDS), Osaka Univ, Global COE Program (Ctr Excellence Adv Struct & Funct Mat Design), Osaka Univ, Global COE Program (Ctr Elect Devices Innovat), Osaka Univ, Grad Sch Engn, Div Mat & Mfg Sci, Osaka Univ, Inst Sci & Ind Res, Osaka Univ, Renovat Ctr Instruments Sci & Technol, Osaka Univ, Photon Pioneers Ctr, Osaka Univ, Off Univ-Ind Collaborat, Innovat Div, Tohoku Univ, Inst Multidisciplinary Res Adv Mat, Iketani Sci & Technol Fdn (ISTF)

AB Zr-based bulk metallic glasses are expected to be welded to conventional structural alloys. Dissimilar welding of metallic glasses to stainless steel was carried out by resistance microwelding. The metallurgical analysis of the weld interface revealed the welding mechanism. A thin reaction layer was formed between the two liquid materials. The melting of stainless steel should be limited to obtain sound joints.

SN 1742-6588

PY 2012

VL 379

AR UNSP 012027

DI 10.1088/1742-6596/379/1/012027

UT WOS:000307653600027

ER

PT J

AU Neilson, HJ

Carter, JLW

Lewandowski, JJ

AF Neilson, Henry J.

Carter, J. L. W.

Lewandowski, John J.

TI An improved method for calculation of elastic constants of metallic

glasses

SO MATERIALS SCIENCE AND ENGINEERING A-STRUCTURAL MATERIALS PROPERTIES

MICROSTRUCTURE AND PROCESSING

AB Using a large database of metallic glasses (N=174), an equation was developed to improve estimating shear and bulk moduli of metallic glasses containing multiple constituents. This equation was applied to several different subsets of metallic glasses: those containing any element, those containing only metallic elements, those containing at least one nonmetal, and those containing at least one constituent with no more than 5 at% of the whole glass. Comparisons to other calculation methods and measured elastic constants for these systems are provided. The present equation provided an improved confidence in the estimations of bulk and shear moduli for all of the cases examined in comparison to previous equations, while the biggest improvement occurred for metallic glasses containing nonmetal constituents. (C) 2015 Elsevier B.V. All rights reserved.

RI Lewandowski, John/S-3815-2017; Carter, Jennifer/A-3241-2013

OI Lewandowski, John/0000-0002-3389-2637; Carter,

Jennifer/0000-0001-6702-729X

SN 0921-5093

EI 1873-4936

PD MAY 14

PY 2015

VL 634

BP 183

EP 187

DI 10.1016/j.msea.2015.03.025

UT WOS:000354505400025

ER

PT J

AU Wang, JQ

Wang, WH

Bai, HY

AF Wang, J. Q.

Wang, W. H.

Bai, H. Y.

TI Extended elastic model for flow in metallic glasses

SO JOURNAL OF NON-CRYSTALLINE SOLIDS

AB We report that both shear and bulk moduli, not only shear modulus, are critical parameters involved in both homogeneous and inhomogeneous flows in metallic glass. The flow activation energy (Delta F) of various glasses when scaled with average molar volume V-m, which is defined as flow activation energy density rho(E)=Delta F/V-m, can be expressed as: rho E = 10G + K/11. The extended elastic model is suggestive for understanding the glass transition and deformation in metallic glasses. (C) 2010 Elsevier B.V. All rights reserved.

RI Wang, Jun-Qiang/G-5989-2010; Wang, Junqiang/C-2839-2015

OI Wang, Junqiang/0000-0002-8066-6237

SN 0022-3093

EI 1873-4812

PD JAN 1

PY 2011

VL 357

IS 1

BP 223

EP 226

DI 10.1016/j.jnoncrysol.2010.09.015

UT WOS:000286173300040

ER

PT J

AU Men, H

Fu, JY

Pang, SJ

Ma, CL

Zhang, T

AF Men, Hua

Fu, Junying

Pang, Shujie

Ma, Chaoli

Zhang, Tao

TI Formation and thermal stability of Cu46.25Zr44.25Al7.5Er2 bulk metallic

glass with a diameter of 12 mm

SO MATERIALS TRANSACTIONS

AB Effect of substituting a small amount of Er for Zr in Cu46.25Zr46.25Al7.5 alloy on glass-forming ability was investigated. The addition of 2 at% Er effectively improves glass formation and glassy rod with a diameter of at least 12 mm was formed by copper mold casting.

RI Zhang, Tao/O-4911-2014; Pang, Shujie/D-8305-2016

SN 1345-9678

EI 1347-5320

PD NOV

PY 2006

VL 47

IS 11

BP 2882

EP 2884

DI 10.2320/matertrans.47.2882

UT WOS:000243368600047

ER

PT J

AU Luo, Q

Zhao, DQ

Pan, MX

Wang, WH

AF Luo, Q.

Zhao, D. Q.

Pan, M. X.

Wang, W. H.

TI Response to "Comment on 'Critical and slow dynamics in a bulk metallic

glass exhibiting strong random magnetic anisotropy' " [Appl. Phys. Lett.

94, 186101 (2009)]

SO APPLIED PHYSICS LETTERS

SN 0003-6951

PD MAY 4

PY 2009

VL 94

IS 18

AR 186102

DI 10.1063/1.3119633

UT WOS:000265933700075

ER

PT J

AU Wang, SY

Jiang, W

Hu, HD

Liu, PF

Wu, JL

Zhang, B

AF Wang, Shouyuan

Jiang, Wei

Hu, Haidong

Liu, Pengfeng

Wu, Jili

Zhang, Bo

TI Roles of Co element in Fe-based bulk metallic glasses utilizing

industrial FeB alloy as raw material

SO PROGRESS IN NATURAL SCIENCE-MATERIALS INTERNATIONAL

AB A series of Fe-based bulk metallic glasses were fabricated by a conventional copper mold casting method using a kind of Fe-B industrial raw alloy. It is found that Fe-B-Y-Nb bulk metallic glass with 3 at% of Co addition possesses the best glass forming ability, thermal stability, hardness, magnetic property and anti-corrosion property. The hardness test result indicates a synchronically trend with glass-forming ability parameters. The excellent glass-forming ability and a combination of good mechanical and functional properties suggest that the alloys in this work might be good candidates for commercial use.

SN 1002-0071

EI 1745-5391

PD AUG

PY 2017

VL 27

IS 4

BP 503

EP 506

DI 10.1016/j.pnsc.2017.08.004

UT WOS:000412606300016

ER

PT J

AU Sun, YJ

Li, YM

Wang, HJ

AF Sun, Yajuan

Li, Yumin

Wang, Hongjing

TI Selection of the Composition with High Glass Forming Ability in

Zr-Cu-Ni-Al Bulk Metallic Glasses

SO ADVANCES IN MATERIALS SCIENCE AND ENGINEERING

AB Three new Zr-Cu-Ni-Al bulk metallic glasses were developed through appropriate mixing of three binary eutectics Zr38.2Cu61.8, Zr51Al49, and Zr-64 Ni-36. By suppressing solidification of competing crystalline phases, a new glass forming alloy Zr51Cu24.22Ni14.06Al10.72 with the critical diameter of up to 10 mm is obtained.

SN 1687-8434

EI 1687-8442

PY 2014

AR 827191

DI 10.1155/2014/827191

UT WOS:000336272100001

ER

PT J

AU Miller, MK

Russell, KF

Martin, PM

Busch, R

Johnson, WL

AF Miller, MK

Russell, KF

Martin, PM

Busch, R

Johnson, WL

TI Characterization of bulk metallic glasses with the atom probe

SO JOURNAL DE PHYSIQUE IV

CT 43rd International Field Emission Symposium (IFES96)

CY JUL 14-19, 1996

CL MOSCOW, RUSSIA

SP Assoc Ultramicroscopy Sci & Technol, Ctr Anal Subst, Moscow Govt, Moscow Phys Soc, US DOE, Oak Ridge Natl Lab, Basic Energy Sci, Russian Fund Fundamental Res

AB An atom probe field ion microscopy survey of several bulk metallic glasses including the zirconium-based glasses Zr55Al10Ni5Cu30, Zr46.25Ti8.75Ni10Be27.5, Zr41.2Ti13.8Cu12.5Ni10Be22.5, Zr57Nb5Al10Cu15.4Ni12.6, and Zr52.5Ti5Al10Cu17.9Ni14.6, together with two non-zirconium-based glasses Ti34Zr11Cu47Ni8, and Mg65Cu25Y10, is presented. Non random distribution of solute was observed in all these glasses and crystalline regions were observed in the Ti34Zr11Cu47Ni8 and Zr55Al10Ni5Cu30 materials.

SN 1155-4339

PD SEP

PY 1996

VL 6

IS C5

BP 217

EP 222

DI 10.1051/jp4:1996535

UT WOS:A1996WF80000038

ER

PT S

AU Chong, FF

To, S

Chan, KC

AF Chong, F. F.

To, S.

Chan, K. C.

BE Lee, WB

Cheung, CF

To, S

TI Cutting Characteristics of Lanthanum Base Metallic Glass in Single Point

Diamond Turning

SO PROCEEDINGS OF PRECISION ENGINEERING AND NANOTECHNOLOGY (ASPEN2011)

SE Key Engineering Materials

CT 4th International Conference of

Asian-Society-for-Precision-Engineering-and-Nanotechnology (ASPEN 2011)

CY NOV 16-18, 2011

CL Hong Kong Polytech Univ, Hong Kong, PEOPLES R CHINA

SP Asian Soc Precis Engn & Nanotechnol, Hong Kong Polytechn Univ,Partner State Key Lab Ultraprecision Machin Technol, Chinese Mech Engn Soc, Japan Soc Precis Engn, Korean Soc Precis Engn, Tianjin Univ, State Key Lab Precis Measurement Technol & Instruments, Tsinghua Univ, State Key Lab Precis Measurement Technol & Instruments

HO Hong Kong Polytech Univ

AB To investigate the maintainability of bulk metallic glasses (BMGs), two Lanthanum base metallic glass were first characterized by using X-ray diffraction and then machined by single point diamond turning (SPDT). With increasing depths of cut (DOC) from 1 um to 5 um, surface finish was improved. However, surface finish is not improved significantly when the DOC is further increased.

RI Chan, K.C./A-2311-2014; To, Sandy/M-2815-2015; Lee, Wing Bun/P-6257-2014

OI Chan, K.C./0000-0002-6173-5532; To, Sandy/0000-0002-1676-7770; Lee, Wing

Bun/0000-0002-3413-4029

SN 1013-9826

PY 2012

VL 516

BP 651

EP +

DI 10.4028/www.scientific.net/KEM.516.651

UT WOS:000308847800114

ER

PT J

AU Jiang, JZ

Hofmann, D

Jarvis, DJ

Fecht, HJ

AF Jiang, Jian-Zhong

Hofmann, Douglas

Jarvis, David John

Fecht, Hans-J.

TI Low-Density High-Strength Bulk Metallic Glasses and Their Composites: A

Review

SO ADVANCED ENGINEERING MATERIALS

AB This review gives an overview of the field of low-density (<6gcm(-3)) bulk metallic glasses/composites and their potential engineering and space applications. The review focuses on four systems, Al-, Mg-, Ca-, and Ti-based metallic glass-forming systems. In the following sections, glass forming ability, mechanical properties, thermal stability, and corrosion resistance of the four metallic glass systems and their composites are presented.

SN 1438-1656

EI 1527-2648

PD JUN

PY 2015

VL 17

IS 6

BP 761

EP 780

DI 10.1002/adem.201400252

UT WOS:000356372500004

ER

PT J

AU Li, Z

Bai, HY

Chen, ZJ

Pan, MX

Zhao, DQ

Wang, WL

Wang, WH

AF Li, Z

Bai, HY

Chen, ZJ

Pan, MX

Zhao, DQ

Wang, WL

Wang, WH

TI Investigation of magnetic properties at low temperatures on permanent

magnetic Nd60Al10Fe20Co10 bulk metallic glass

SO ACTA PHYSICA SINICA

AB The temperature dependence of magnetic properties for Nd60Al10Fe20Co10 bulk metallic glass is investigated. The results indicate that the Nd60Al10Fe20Co10 bulk metallic glass exhibits permanent magnetic behaviour at room temperature. The coercive force and the shape of hysteresis loops change markedly when temperature decreases. AC magnetic susceptibility is measured and a peak at about 18K is found. The peak moves torwards high temperatures when the frequency is increased. This indicates that there are spin glass structures in the bulk metallic glass.

SN 1000-3290

PD JUN

PY 2003

VL 52

IS 6

BP 1461

EP 1464

UT WOS:000183472500028

ER

PT S

AU Zou, JH

Zhang, ZC

Sun, SQ

AF Zou, Jia Hua

Zhang, Zhi Chen

Sun, Shu Quan

BE Tan, HH

TI Room-temperature mechanical properties and glass formation of

Zr50Cu20Ni15Al(15-x)Tix (x=3, 5, 7) bulk metallic glass alloys

SO MECHANICAL, MATERIALS AND MANUFACTURING ENGINEERING, PTS 1-3

SE Applied Mechanics and Materials

CT International Conference on Mechanical Materials and Manufacturing

Engineering (ICMMME 2011)

CY JUN 20-22, 2011

CL Nanchang, PEOPLES R CHINA

AB In the present study, the Zr-Cu-Ni-Al based bulk metallic glasses with different Ti addition was successfully prepared by suction casting. It was found that the glass forming ability was improved with increasing of Ti content from 3 at.% to 7 at.%. However, with increasing of Ti content, the room-temperature plasticity decreased from 4.33% to 0.66%.

SN 1660-9336

BN 978-3-03785-185-2

PY 2011

VL 66-68

BP 741

EP +

DI 10.4028/www.scientific.net/AMM.66-68.741

UT WOS:000309941600141

ER

PT J

AU Chen, N

Li, Y

Yao, KF

AF Chen, Na

Li, Yang

Yao, Ke-Fu

TI Thermal stability and fragility of Pd-Si binary bulk metallic glasses

SO JOURNAL OF ALLOYS AND COMPOUNDS

CT 16th International Symposium on Metastable, Amorphous and Nanostructured

Materials

CY JUL 05-09, 2009

CL Beijing, PEOPLES R CHINA

AB The thermal stability of the glassy structure and the supercooled liquids of Pd80+xSi20-x (x = 0, 1, and 2) binary bulk metallic glasses were investigated by differential scanning calorimetry. It is found that Pd-Si binary metallic glasses exhibit large activation energy for glass transition and crystallization, indicating the glassy structure and the supercooled liquids possess high thermal stability. Based on the kinetic nature of the glass transition and thus the resulted heating rate dependence, the fragility index of the Pd-Si binary glassy alloys has been obtained, and compared with other bulk metallic glasses. (C) 2010 Elsevier B.V. All rights reserved.

RI Chen, Na/A-4120-2010

SN 0925-8388

PD AUG

PY 2010

VL 504

SU 1

BP S211

EP S214

DI 10.1016/j.jallcom.2010.02.079

UT WOS:000285252600054

ER

PT J

AU Zhou, ZH

Uher, C

Xu, DH

Johnson, WL

Gannon, W

Aronson, MC

AF Zhou, Zhenhua

Uher, Ctirad

Xu, Donghua

Johnson, W. L.

Gannon, W.

Aronson, M. C.

TI On the existence of Einstein oscillators and thermal conductivity in

bulk metallic glass

SO APPLIED PHYSICS LETTERS

AB Low-temperature specific heat and thermal conductivity of bulk metallic glasses are measured to identify the primary vibrational modes associated with their unique structures. An Einstein-type localized vibrational mode with an Einstein temperature of 112 K is found in bulk metallic glass Ni59.5Nb33.6Sn6.9. This localized vibrational mode causes resonant scattering of phonons and results in the localization of phonons which leaves the phonon hopping conduction the limiting mechanism of thermal transport in bulk metallic glass Ni59.5Nb33.6Sn6.9 at high temperature. (c) 2006 American Institute of Physics.

RI Xu, Donghua/A-5263-2008

OI Xu, Donghua/0000-0001-5018-5603

SN 0003-6951

PD JUL 17

PY 2006

VL 89

IS 3

AR 031924

DI 10.1063/1.2234281

UT WOS:000239174100050

ER

PT J

AU Zhang, XF

Wang, XD

Kim, KB

Yi, S

AF Zhang, X. F.

Wang, X. D.

Kim, K. B.

Yi, S.

TI Be effect on glass-forming ability and mechanical properties of

Ti-Cu-Co-Zr-Sn bulk metallic glasses

SO MATERIALS TRANSACTIONS

AB A series of Ti44Cu44-xCo4Zr6Sn2Bex bulk metallic glasses with x = 0-5.1 has been systematically investigated in terms of glass-forming ability and mechanical properties at room temperature. With the small amount of Be addition, the glass forming ability as well as ductility can be significantly increased leading to the fully amorphous rod having the diameter larger than 4mm and ductility larger than 10%. Upon compressing the bulk metallic glass Ti44Cu38.9Co4Zr6Sn2Be5.1, a work hardening-like behavior that may be attributed to structural heteorgeniety was observed.

SN 1345-9678

EI 1347-5320

PD SEP

PY 2006

VL 47

IS 9

BP 2321

EP 2325

DI 10.2320/matertrans.47.2321

UT WOS:000241900600037

ER

PT B

AU Liu, CT

Greer, AL

Schuh, CA

AF Liu, C. T.

Greer, A. Lindsay

Schuh, Christopher A.

BA Suryanarayana, C

Inoue, A

BF Suryanarayana, C

Inoue, A

TI Glass-Forming Ability of Alloys

SO BULK METALLIC GLASSES

BN 978-1-4200-8596-9

PY 2011

BP 49

EP 144

D2 10.1201/9781420085976-1

UT WOS:000288438000004

ER

PT J

AU Liu, YH

Wang, K

Pan, D

Wang, H

Nakayama, K

Inoue, A

Wang, WH

Chen, MW

AF Liu, YanHui

Wang, Ke

Pan, Deng

Wang, Hao

Nakayama, Koji

Inoue, Akihisa

Wang, WeiHua

Chen, MingWei

TI Plastic Deformation-Assisted Synthesis of Metallic Glass Nanostructures

SO MATERIALS TRANSACTIONS

AB In this paper, we report synthesis of various metallic glass nanostructures such as rods, wires and spheres via plastically deforming bulk metallic glasses in quasi-static compression at room temperature, and the formation mechanism and factors controlling their dimensions are discussed. The nanostructures are formed via viscous flow of softened layers within the shear band. The length of these nanostructures is dominated by the fracture toughness of metallic glass, which determines the temperature rise during plastic deformation and thus the viscosity of the softened layers. [doi: 10.2320/matertrans.M2009027]

RI Wang, Ke/M-7171-2017; Nakayama, Koji/E-5053-2012; Pan, Deng/A-1090-2018;

Wang, Hao/F-8467-2013; CHEN, Mingwei/A-4855-2010; Wang, Ke/C-8021-2011;

Wang, Hao/D-9512-2014; Inoue, Akihisa/E-5271-2015; LIU,

Yanhui/B-1485-2009; Pan, Deng/C-2072-2009

OI Wang, Ke/0000-0002-1174-0907; Wang, Hao/0000-0002-1708-8075; CHEN,

Mingwei/0000-0002-8274-3099; Wang, Hao/0000-0002-1708-8075; Chen,

Mingwei/0000-0002-2850-8872

SN 1345-9678

EI 1347-5320

PD JUL

PY 2009

VL 50

IS 7

SI SI

BP 1890

EP 1893

DI 10.2320/matertrans.M2009027

UT WOS:000269029000053

ER

PT J

AU Yang, K

Fan, XH

Li, B

Li, YH

Wang, X

Xu, XX

AF Yang Ke

Fan Xinhui

Li Bing

Li Yanhong

Wang Xin

Xu Xuanxuan

TI Effect of yttrium addition on flow behavior of Cu-Zr-Al bulk metallic

glass in the supercooled liquid region

SO JOURNAL OF RARE EARTHS

AB The high temperature deformation behaviors and thermal workability of Cu43Zr48Al9 and (Cu43Zr48Al9)(98)Y-2 bulk metallic glasses in the supercooled liquid region were investigated by the uniaxial compression tests. The results showed that the high temperature deformation behaviors were highly sensitive to strain rate and temperature, and the flow stress decreased with the increase of temperature, as well as with the decrease of strain rate. Additionally, the (Cu43Zr48Al9)(98)Y-2 bulk metallic glass displayed smaller flow stress under the same condition. The flow behavior changed from Newtonian to non- Newtonian with increase of the strain rate, as well as the decrease of temperature, which could be explained by the transition state theory. We found that (Cu43Zr48Al9) Y-98(2) bulk metallic glass had better flow behavior than the Cu43Zr48Al9 bulk metallic glass in the supercooled liquid region. In addition, the processing maps of the two bulk metallic glasses were constructed considering the power dissipation efficiency. The optimum domain for thermal workability of the bulk metallic glass was located using the processing map, where the power dissipation efficiency was larger than 0.8. It was shown that the (Cu43Zr48Al9)(98)Y-2 bulk metallic glass, which had larger area of optimum domain, had excellent thermoplastic forming.

SN 1002-0721

PD OCT

PY 2017

VL 35

IS 10

BP 1035

EP 1041

DI 10.1016/S1002-0721(17)61010-X

UT WOS:000415702200013

ER

PT J

AU Plummer, JD

Figueroa, IA

Hand, RJ

Davies, HA

Todd, I

AF Plummer, J. D.

Figueroa, I. A.

Hand, R. J.

Davies, H. A.

Todd, I.

TI Elastic properties of some bulk metallic glasses

SO JOURNAL OF NON-CRYSTALLINE SOLIDS

AB The relationships between the elastic moduli, glass forming ability and response to deformation of bulk metallic glasses are investigated. Five bulk metallic glasses are prepared from high purity elements via suction casting. The results confirm that there exists a correlation between energy absorbed to failure during compression testing and the bulk to shear modulus ratio. This finding is developed such that it corresponds only to the elastic component of energy absorption, and that the bulk modulus dominates this. Plastic deformation appears to be favored by a reduced shear modulus, although it shows greater dependence on structural features that are frozen in during the glass transition, and so may well be dependent on the liquid fragility. (C) 2009 Elsevier B.V. All rights reserved.

RI Figueroa, Ignacio/J-5914-2012

OI Plummer, John/0000-0003-4824-8497; Todd, Iain/0000-0003-0217-1658

SN 0022-3093

PD MAR 1

PY 2009

VL 355

IS 6

BP 335

EP 339

DI 10.1016/j.jnoncrysol.2008.12.011

UT WOS:000264595400001

ER

PT J

AU Wang, D

Li, Y

Sun, BB

Sui, ML

Lu, K

Ma, E

AF Wang, D

Li, Y

Sun, BB

Sui, ML

Lu, K

Ma, E

TI Bulk metallic glass formation in the binary Cu-Zr system

SO APPLIED PHYSICS LETTERS

AB Using the Cu-Zr model system, we demonstrate that bulk amorphous alloys can be obtained by copper mold casting even in a binary metallic system. The narrow, off-eutectic, bulk-glass-forming range was found to require composition pinpointing to <1 at. %. A phase selection diagram is used to explain the success of our microstructure-based approach to pinpoint the best glass former in a given system. The implications of discovering simple binary bulk amorphous alloys are discussed, in terms of its impact on understanding the formation and physics of bulk metallic glasses. (C) 2004 American Institute of Physics.

RI Sui, Manling/B-6069-2013; Ma, En/A-3232-2010

OI Sui, Manling/0000-0002-0415-5881;

SN 0003-6951

PD MAY 17

PY 2004

VL 84

IS 20

BP 4029

EP 4031

DI 10.1063/1.1751219

UT WOS:000221269800025

ER

PT J

AU Branagan, DJ

Zhou, J

Meacham, BE

Sergueeva, AV

AF Branagan, D. J.

Zhou, J.

Meacham, B. E.

Sergueeva, A. V.

TI Achieving Usable Ductility in Glassy Nanomaterials

SO ADVANCED MATERIALS & PROCESSES

SN 0882-7958

PD OCT

PY 2010

VL 168

IS 10

BP 25

EP 31

UT WOS:000283208900031

ER

PT J

AU Bian, Z

Wang, RJ

Pan, MX

Zhao, DQ

Wang, WH

AF Bian, Z

Wang, RJ

Pan, MX

Zhao, DQ

Wang, WH

TI Excellent wave absorption by zirconium-based bulk metallic glass

composites containing carbon nanotubes

SO ADVANCED MATERIALS

SN 0935-9648

PD APR 17

PY 2003

VL 15

IS 7-8

BP 616

EP 621

DI 10.1002/adma.200304565

UT WOS:000182552300019

ER

PT J

AU Wang, XY

Sun, LL

Li, G

Liu, RP

Zhang, J

Wang, WK

AF Wang, XY

Sun, LL

Li, G

Liu, RP

Zhang, J

Wang, WK

TI Impurity diffusion of Mo in Zr57Nb5Cu15.4Ni12.6Al10 bulk metallic glass

SO JOURNAL OF MATERIALS SCIENCE LETTERS

SN 0261-8028

PD FEB 1

PY 2003

VL 22

IS 3

BP 171

EP 173

DI 10.1023/A:1022285522881

UT WOS:000180750200002

ER

PT J

AU Goncharova, EV

Konchakov, RA

Makarov, AS

Kobelev, NP

Khonik, VA

AF Goncharova, E. V.

Konchakov, R. A.

Makarov, A. S.

Kobelev, N. P.

Khonik, V. A.

TI On the nature of density changes upon structural relaxation and

crystallization of metallic glasses

SO JOURNAL OF NON-CRYSTALLINE SOLIDS

AB On the basis of the Interstitialcy theory, we suggest a quantitative explanation of density changes occurring upon structural relaxation and crystallization of metallic glasses.

RI Makarov, Andrey/H-4156-2013; Khonik, Vitaly/A-5888-2009

OI Makarov, Andrey/0000-0001-6741-0619;

SN 0022-3093

EI 1873-4812

PD SEP 1

PY 2017

VL 471

BP 396

EP 399

DI 10.1016/j.jnoncrysol.2017.06.024

UT WOS:000407188100052

ER

PT J

AU Pi, JH

He, XC

Wang, ZZ

AF Pi Jinhong

He Xiancong

Wang Zhangzhong

TI Preparation of High Entropy Alloy Cu29Zr32Ti15Al5Ni19 with High Glass

Forming Ability

SO RARE METAL MATERIALS AND ENGINEERING

AB High entropy alloy (HEA) Cu29Zr32Ti15Al5Ni19 consisting of two solid solution phases and bulk metallic glass (HE-BMG) with the same composition were prepared separately by casting in a copper mold. The results indicate that the alloy has high glass forming ability. The compressive strength of as cast HEA Cu29Zr32Ti15Al5Ni19 is 1127 MPa. HEA Cu29Zr32Ti15Al5Ni19 shows good tempering resistance. The hardness (HV) of Cu29Zr32Ti15Al5Ni19 remains 8260 MPa after it is treated at 750 degrees C for 2 h.

SN 1002-185X

PD JUL

PY 2017

VL 46

IS 7

BP 1810

EP 1814

UT WOS:000407092900011

ER

PT J

AU Kovacs, Z

Ezzeldien, M

Chinh, NQ

Radnoczi, G

Lendvai, J

AF Kovacs, Zsolt

Ezzeldien, Mohammed

Nguyen Quang Chinh

Radnoczi, Gyoergy

Lendvai, Janos

TI Nanoindentation measurements on a torsionally deformed

Zr44Ti11Cu10Ni10Be25 bulk metallic glass

SO JOURNAL OF ALLOYS AND COMPOUNDS

AB Nanoindentation measurements were performed on as-cast and torsionally deformed Zr44Ti11Cu10-Ni10Be25 bulk metallic glass samples. Distribution of the measured hardness values shows peak-increasing and widening as an effect of the plastic pre-deformation. Variation in the strength of the deformation affected material and the origin of strain hardening is explained by key characteristics of shear transformation zones in bulk metallic glasses. (C) 2017 Elsevier B.V. All rights reserved.

OI Radnoczi, Gyorgy/0000-0002-5056-7625

SN 0925-8388

EI 1873-4669

PD JUN 25

PY 2017

VL 708

BP 301

EP 307

DI 10.1016/j.jallcom.2017.02.294

UT WOS:000400713300039

ER

PT J

AU Hebert, RJ

Mubarok, A

AF Hebert, Rainer J.

Mubarok, Arif

TI Thermal and Thermomechanical Behavior of Cu50Hf41.5Al8.5 Bulk Metallic

Glass after Cyclic Elastic Deformation

SO ADVANCED ENGINEERING MATERIALS

SN 1438-1656

PD NOV

PY 2008

VL 10

IS 11

BP 1064

EP 1067

DI 10.1002/adem.200800144

UT WOS:000261933400015

ER

PT B

AU Kenoufi, A

AF Kenoufi, Abdelouahab

BE Kholmurodov, KT

TI Finding Low-Energy Configurations of Copper-Based Bulk Metallic Glasses

Using Minima Hopping Global Optimization Method

SO MOLECULAR SIMULATION IN MATERIAL AND BIOLOGICAL RESEARCH

CT 3rd International Workshop on Molecular Simulation Studies in Material

and Biological Sciences

CY SEP 10-12, 2008

CL Joint Inst Nucl Res, Dubna, RUSSIA

HO Joint Inst Nucl Res

AB We propose in this article an adaptation of the Minima Hopping method to optimize the geometry of clusters and periodic systems on potential energy surface (PES) such as Bulk Metallic Glasses and copper systems.

BN 978-1-60741-553-4

PY 2009

BP 129

EP +

UT WOS:000273517800009

ER

PT J

AU Schuh, CA

Nieh, TG

AF Schuh, CA

Nieh, TG

TI A survey of instrumented indentation studies on metallic glasses

SO JOURNAL OF MATERIALS RESEARCH

AB The development of instrumented nanoindentation equipment has occurred concurrently with the discovery of many new families of bulk metallic glass during the past decade. While indentation testing has long been used to assess the mechanical properties of metallic glasses, depth-sensing capabilities offer a new approach to study the fundamental physics behind glass deformation. This article is a succinct review of the research to date on the indentation of metallic glasses. In addition to standard hardness measurements, the onset of plasticity in metallic glasses is reviewed as well as the role of shear banding in indentation, structural changes beneath the indenter, and rate-dependent effects measured by nanoindentation. The article concludes with perspectives about the future directions for nanocontact studies on metallic glasses.

RI Schuh, Christopher/C-7947-2009; Nieh, Tai-Gang/G-5912-2011

OI Nieh, Tai-Gang/0000-0002-2814-3746

SN 0884-2914

PD JAN

PY 2004

VL 19

IS 1

BP 46

EP 57

DI 10.1557/jmr.2004.0005

UT WOS:000222316100005

ER

PT J

AU Yang, C

Zhan, ZJ

Fan, CZ

Liu, RP

Wang, WK

AF Yang, C.

Zhan, Z. J.

Fan, C. Z.

Liu, R. P.

Wang, W. K.

TI In situ X-ray diffraction study on crystallization of

shock-wave-quenched Zr-based bulk metallic glasses

SO MATERIALS SCIENCE AND ENGINEERING A-STRUCTURAL MATERIALS PROPERTIES

MICROSTRUCTURE AND PROCESSING

CT 12th International Conference on Rapidly Quenched and Metastable

Materials

CY AUG 21-26, 2005

CL Cheju Isl, SOUTH KOREA

AB Crystallization of Zr(41.9)Ti(14.7)Cu(13).(1)Ni(10.1)Be(20.2) (number indicate at.%) bulk metallic glasses prepared by shock-wave quenching and waterquenching are investigated by in situ X-ray diffraction under continuous heating conditions. Different phases and different precipitation sequences are found for both bulk metallic glasses. The differences in crystallization are probably attributed to different atomic configurations between the two bulk metallic glasses prepared by different ways. (c) 2006 Elsevier B.V. All rights reserved.

RI Yang, Chao/H-1905-2015; Fan, Changzeng/I-6837-2018

OI Fan, Changzeng/0000-0001-6727-5912

SN 0921-5093

PD MAR 25

PY 2007

VL 449

BP 617

EP 620

DI 10.1016/j.msea.2006.02.339

UT WOS:000245477800141

ER

PT J

AU Chen, R

Yang, FQ

Fan, GJ

Liaw, PK

AF Chen, Rong

Yang, Fuqian

Fan, Guojiang

Liaw, Peter K.

TI Hardness variation across a Zr57Ti5Cu20Ni8Al10 bulk metallic glass

SO JOURNAL OF MATERIALS SCIENCE

RI Chen, Rong/A-7074-2012

SN 0022-2461

EI 1573-4803

PD MAR

PY 2007

VL 42

IS 6

BP 2208

EP 2211

DI 10.1007/s10853-006-1476-7

UT WOS:000244889600048

ER

PT J

AU Cui, J

Li, JS

Wang, J

Kou, HC

Qiao, JC

Gravier, S

Blandin, JJ

AF Cui, J.

Li, J. S.

Wang, J.

Kou, H. C.

Qiao, J. C.

Gravier, S.

Blandin, J. J.

TI Rheological behavior of Cu-Zr-based metallic glass in the supercooled

liquid region

SO JOURNAL OF ALLOYS AND COMPOUNDS

AB The high temperature rheological behaviors of Zr46Cu38Ag8Al8 and Cu46Zr45Al7Y2 bulk metallic glasses in supercooled liquid region have been investigated by uniaxial strain rate jump compression tests. The results indicate that the high temperature rheological behaviors are sensitive to strain rate and temperature. The rheological behavior will transform from non-Newtonian flow to Newtonian flow with the decrease of strain rate as well as the increase of temperature, which can be explained in the framework of transition state theory. And the rheological behavior of Zr46Cu38Ag8Al8 bulk metallic glass in high temperature is better than that of Cu46Zr45Al7Y2 bulk metallic glass. In addition, the parameter S of both metallic glasses are in a middle level compared with typical bulk metallic glasses, which shown that the metallic glass has an excellent formability in its supercooled liquid region. The optimum domain for thermal workability of these alloys in supercooled liquid region have been roughly located by the deformation map and the power dissipation map, in which the power dissipation efficiency is larger than 0.8. The larger area of optimum domain in the power dissipation map of Zr46Cu38Ag8Al8 exhibited that this bulk metallic glass presents an excellent workability in its supercooled liquid region, but the workability of Cu46Zr45Al7Y2 is not as good as Zr46Cu38Ag8Al8. (C) 2014 Elsevier B.V. All rights reserved.

RI WANG, Jun/A-1526-2015; WANG, Jun/B-1229-2010

OI WANG, Jun/0000-0001-8101-2967; WANG, Jun/0000-0001-8101-2967; KOU,

Hongchao/0000-0003-4960-9477

SN 0925-8388

EI 1873-4669

PD APR 15

PY 2014

VL 592

BP 189

EP 195

DI 10.1016/j.jallcom.2014.01.014

UT WOS:000331069300031

ER

PT B

AU Liu, CT

Greer, AL

Schuh, CA

AF Liu, C. T.

Greer, A. Lindsay

Schuh, Christopher A.

BA Suryanarayana, C

Inoue, A

BF Suryanarayana, C

Inoue, A

TI Magnetic Properties

SO BULK METALLIC GLASSES

BN 978-1-4200-8596-9

PY 2011

BP 459

EP 479

D2 10.1201/9781420085976-1

UT WOS:000288438000010

ER

PT J

AU Lu, CL

Liu, HM

Wang, KF

Dong, S

Liu, JM

Wang, Q

Dong, C

AF Lu, C. L.

Liu, H. M.

Wang, K. F.

Dong, S.

Liu, J. -M.

Wang, Q.

Dong, C.

TI Magnetic properties of Sm-based bulk metallic glasses

SO JOURNAL OF MAGNETISM AND MAGNETIC MATERIALS

AB We perform detailed investigation on the magnetization and specific heat of Sm-based ternary bulk metallic glasses with different Co contents at low temperature. A low temperature cluster spin-glass phase below T-f similar to 25 K is evidenced for all samples. This cluster spin-glass behavior is ascribed to competition among the multi-fold magnetic interactions and the intrinsic structural in homogeneity. The magnetic relaxation behavior of the spin-glass phase can be well described using the stretched exponential dynamics. The magnetic hysteresis under field-cooling condition and spin dynamics further demonstrate the low temperature cluster spin-glass behavior. It is revealed that the Co atoms play an important role in modulating the physical properties of the present Sm-based ternary metallic glasses. (C) 2010 Elsevier B.V. All rights reserved.

RI Dong (董), Shuai (帅)/A-5513-2008; Lu, Chengliang/B-1469-2009

OI Dong (董), Shuai (帅)/0000-0002-6910-6319;

SN 0304-8853

EI 1873-4766

PD OCT

PY 2010

VL 322

IS 19

BP 2845

EP 2850

DI 10.1016/j.jmmm.2010.04.040

UT WOS:000279437700012

ER

PT J

AU Luo, Q

Zhao, DQ

Pan, MX

Wang, RJ

Wang, WH

AF Luo, Q

Zhao, DQ

Pan, MX

Wang, RJ

Wang, WH

TI Hard and fragile holmium-based bulk metallic glasses

SO APPLIED PHYSICS LETTERS

AB A family of holmium-based bulk metallic glasses (BMGs) with high glass-forming ability is obtained. The Ho-based BMGs exhibit much larger elastic moduli and high thermal stability in contrast to other known rare-earth (RE)-based BMGs. In particular, the BMGs show a large value of fragility. It is expected that the hard RE-based glasses with high glass-forming ability and fragile behaviors make them the appropriate candidate for glass transition study. (c) 2006 American Institute of Physics.

SN 0003-6951

EI 1077-3118

PD MAY 1

PY 2006

VL 88

IS 18

AR 181909

DI 10.1063/1.2201863

UT WOS:000237321600031

ER

PT J

AU Axinte, E

AF Axinte, Eugen

TI Metallic glasses from "alchemy" to pure science: Present and future of

design, processing and applications of glassy metals

SO MATERIALS & DESIGN

AB Metallic glasses, first discovered a half century ago, are currently among the most studied metallic materials. Available in sizes up to several centimeters, with many novel, applicable properties, metallic glasses have also been the focus of research advancing the understanding of liquids and of glasses in general.

Metallic glasses (MGs), called also bulk metallic glasses (BMGs) (or glassy metals, amorphous metals, liquid metals) are considered to be the materials of the future. Due to their high strength, metallic glasses have a number of interesting applications, for example as coatings. Metallic glasses can also be corrosion resistant. Metallic glasses, and the crystalline materials derived from them, can have very good resistance to sliding and abrasive wear. Combined with their strength - and now, toughness - this makes them ideal candidates for bio-implants or military applications. Prestigious Journals such as "Nature Materials", ''Nature" frequently publish new findings on these unusual glass materials. Moreover Chinese and Asian scientists have also been showing an interest in the study of metallic glasses.

This review paper is far from exhaustive, but tries to cover the areas of interest as it follows: a short history, the local structure of BMGs and the glass forming ability (GFA), BMGs' properties, the manufacturing and some applications of BMGs and finally, about the future of BMGs as valuable materials. (C) 2011 Elsevier Ltd. All rights reserved.

RI Axinte, Eugen/C-6963-2011

OI Axinte, Eugen/0000-0002-9243-1160

SN 0261-3069

PD MAR

PY 2012

VL 35

BP 518

EP 556

DI 10.1016/j.matdes.2011.09.028

UT WOS:000301578700067

ER

PT J

AU Kobelev, NP

Kolyvanov, EL

Khonik, VA

AF Kobelev, NP

Kolyvanov, EL

Khonik, VA

TI Nonlinear elastic characteristics of Zr52.5Ti5Cu17.9Ni14.6Al10 and

Pd40Cu30Ni10P20 bulk metallic glasses

SO PHYSICS OF THE SOLID STATE

AB The influence of uniaxial compression on the propagation of sound in Zr52.5Ti5Cu17.9Ni14.6Al10 and Pd40Cu30Ni10P20 bulk metallic glasses is investigated, and the third-order elastic moduli of these glasses are determined. (c) 2005 Pleiades Publishing, Inc.

RI Khonik, Vitaly/A-5888-2009

SN 1063-7834

PD MAR

PY 2005

VL 47

IS 3

BP 405

EP 410

DI 10.1134/1.1884696

UT WOS:000228013100002

ER

PT J

AU Bokeloh, J

Divinski, SV

Reglitz, G

Wilde, G

AF Bokeloh, Joachim

Divinski, Sergiy V.

Reglitz, Gerrit

Wilde, Gerhard

TI Tracer Measurements of Atomic Diffusion inside Shear Bands of a Bulk

Metallic Glass (vol 107, 235503, 2011)

SO PHYSICAL REVIEW LETTERS

RI Divinski, Sergiy/G-9948-2011

SN 0031-9007

PD DEC 20

PY 2011

VL 107

IS 26

AR 269901

DI 10.1103/PhysRevLett.107.269901

UT WOS:000298607400012

ER

PT J

AU Qiao, JW

Zhang, Y

Liaw, PK

AF Qiao, Jun-Wei

Zhang, Yong

Liaw, Peter K.

TI Tailoring Microstructures and Mechanical Properties of Zr-Based Bulk

Metallic Glass Matrix Composites by the Bridgman Solidification

SO ADVANCED ENGINEERING MATERIALS

RI ZHANG, Yong/B-7928-2009

OI ZHANG, Yong/0000-0002-6355-9923

SN 1438-1656

PD NOV

PY 2008

VL 10

IS 11

BP 1039

EP 1042

DI 10.1002/adem.200800149

UT WOS:000261933400010

ER

PT J

AU Churyumov, AY

Bazlov, AI

Tsar'kov, AA

Starodub, KF

Luzgin, DVL

AF Churyumov, A. Yu.

Bazlov, A. I.

Tsar'kov, A. A.

Starodub, K. F.

Luzgin, D. V. Louzguine

TI Investigation and simulation of crystallization of bulk zirconium-based

metallic glasses

SO RUSSIAN JOURNAL OF NON-FERROUS METALS

AB This article is devoted to the investigation and simulation of the crystallization kinetics of zirconium-based bulk metallic glasses under heating with a constant rate, as well as under isothermal holding at an elevated temperature. The investigation into crystallization kinetics with time can provide a deeper under-standing of the crystallization mechanism of bulk metallic glasses and promote the scientifically substantiated selection of thermal treatment modes to form the desired structure and properties of composite materials on their basis.

RI LOUZGUINE, Dmitri/D-2492-2010; Tsarkov, Andrey/H-1523-2015; Starodub,

Konstantin/G-7892-2015

OI LOUZGUINE, Dmitri/0000-0001-5716-4987; Tsarkov,

Andrey/0000-0002-9179-1201; Starodub, Konstantin/0000-0003-3331-5132

SN 1067-8212

EI 1934-970X

PD JAN

PY 2014

VL 55

IS 1

BP 31

EP 36

DI 10.3103/S1067821214010052

UT WOS:000333158200006

ER

PT J

AU Yang, WM

Wan, C

Liu, HS

Li, Q

Wang, QQ

Li, H

Zhou, J

Xue, L

Shen, BL

Inoue, A

AF Yang, Weiming

Wan, Chao

Liu, Haishun

Li, Qiang

Wang, Qianqian

Li, Hui

Zhou, Jing

Xue, Lin

Shen, Baolong

Inoue, Akihisa

TI Fluxing induced boron alloying in Fe-based bulk metallic glasses

SO MATERIALS & DESIGN

AB In this work, we report on advanced insights into the effects of fluxing on Fe50Ni30P13C7 bulk metallic glasses. We find that the employed oxide fluxing agent results in significant incorporation of boron in the alloy by atom probe tomography analysis. It demonstrates that the improved glass-forming ability induced by fluxing attributes to not only the reduction of heterogeneous nucleation, but also boron alloying. In addition, the more densely packed microstructure of the fluxed alloys is also related to amount of boron addition. Such an understanding is of great importance to adequately process glass-forming liquids, in order to achieve optimized bulk metallic glass parts with enhanced thicknesses and improved mechanical properties.

SN 0264-1275

EI 1873-4197

PD SEP 5

PY 2017

VL 129

BP 63

EP 68

DI 10.1016/j.matdes.2017.05.020

UT WOS:000405099900008

ER

PT J

AU Ashby, MF

Greer, AL

AF Ashby, MF

Greer, AL

TI Metallic glasses as structural materials

SO SCRIPTA MATERIALIA

AB The potential of metallic glasses as structural materials is assessed. A wide-ranging comparison with conventional engineering materials shows metallic glasses to be restricted to niche applications, but with outstanding properties awaiting wider application, for example in micro electro-mechanical systems devices. (c) 2005 Acta Materialia Inc. Published by Elsevier Ltd. All rights reserved.

RI Greer, Alan Lindsay/G-1977-2011; Greer, Lindsay/E-9433-2017

SN 1359-6462

PD FEB

PY 2006

VL 54

IS 3

BP 321

EP 326

DI 10.1016/j.scriptamat.2005.09.051

UT WOS:000233495600002

ER

PT J

AU Liu, YH

Wang, G

Wang, RJ

Zhao, DQ

Pan, MX

Wang, WH

AF Liu, Yan Hui

Wang, Gang

Wang, Ru Ju

Zhao, De Qian

Pan, Ming Xiang

Wang, Wei Hua

TI Super plastic bulk metallic glasses at room temperature

SO SCIENCE

AB In contrast to the poor plasticity that is usually observed in bulk metallic glasses, super plasticity is achieved at room temperature in ZrCuNiAl synthesized through the appropriate choice of its composition by controlling elastic moduli. Microstructures analysis indicates that the super plastic bulk metallic glasses are composed of hard regions surrounded by soft regions, which enable the glasses to undergo true strain of more than 160%. This finding is suggestive of a solution to the problem of brittleness in, and has implications for understanding the deformation mechanism of, metallic glasses.

RI LIU, Yanhui/B-1485-2009; Wang, Gang/K-2630-2012

SN 0036-8075

PD MAR 9

PY 2007

VL 315

IS 5817

BP 1385

EP 1388

DI 10.1126/science.1136726

UT WOS:000244752200025

PM 17347434

ER

PT J

AU Cai, AH

Wang, H

Li, XS

Clen, H

An, WK

AF Cai, An-Hui

Wang, Hui

Li, Xiao-Song

Clen, Hua

An, Wei-Ke

TI Progress of component design methods for bulk metallic glass

SO MATERIALS & DESIGN

AB The development and application of the component design methods for bulk metallic glasses are summarized. And then, their application scopes are analyzed, respectively. Moreover, we propose a new method titled as similitude principle and artificial neural network model (SPANNM) for the component design and glass forming ability of bulk metallic glasses. It is shown that the predicted results by the SPANNM are in better agreement with the experimental values. (c) 2006 Elsevier Ltd. All rights reserved.

RI Wang, Hui/C-6768-2009; Wang, Hui/A-5775-2017

OI Wang, Hui/0000-0002-4915-3396

SN 0261-3069

PY 2007

VL 28

IS 10

BP 2694

EP 2697

DI 10.1016/j.matdes.2006.10.002

UT WOS:000249051200019

ER

PT J

AU Griesche, A

Macht, MP

Frohberg, G

AF Griesche, A

Macht, MP

Frohberg, G

TI Chemical diffusion in bulk glass-forming Pd40Cu30Ni10P20 melts

SO SCRIPTA MATERIALIA

AB Interdiffusion and self-diffusion of bulk metallic glass-forming Pd-Cu-Ni-P alloys have been investigated above the liquidus temperature at 993 K by the long-capillary method. Good agreement between the calculated partial mixing enthalpies and observed uphill-diffusion was found. The flow direction of uphill-diffusing elements is towards regions with the highest negative beat of mixing. (c) 2005 Acta Materialia Inc. Published by Elsevier Ltd. All rights reserved.

RI Griesche, Axel/E-6122-2010

SN 1359-6462

PD DEC

PY 2005

VL 53

IS 12

BP 1395

EP 1400

DI 10.1016/j.scriptamat.2005.08.024

UT WOS:000232700500014

ER

PT J

AU Klein, DJ

March, NH

Alonso, JA

AF Klein, D. J.

March, N. H.

Alonso, J. A.

TI Fractal network dimension determining the relation between the strength

of bulk metallic glasses and the glass transition temperature

SO APPLIED PHYSICS LETTERS

AB [Ma , Nat. Mater. 8, 30 (2009)] have uncovered the fractal dimension D-f=2.31 associated with the medium-range order in a variety of bulk metallic glasses, reflected in the first sharp diffraction peak q(1) determined from neutron and x-ray measurements. Here, based on the proposal in this journal of [Yang , Appl. Phys. Lett. 88, 221911 (2006)], which related the strength sigma(y) of bulk metallic glasses to the glass transition temperature T-g, we show that the product q(1)(f)(D)sigma(y) is linear in T-g.

RI Alonso, Julio/D-5781-2016; DONOSTIA INTERNATIONAL PHYSICS CTR.,

DIPC/C-3171-2014

OI Alonso, Julio/0000-0002-8604-8608;

SN 0003-6951

PD JUL 13

PY 2009

VL 95

IS 2

AR 021909

DI 10.1063/1.3183520

UT WOS:000268089200024

ER

PT J

AU Yang, L

Wang, XL

Porter, WD

Lu, ZP

Stoica, AD

Payzant, EA

Almer, J

Shi, DL

AF Yang, Ling

Wang, Xun-Li

Porter, Wallace D.

Lu, Zhaoping

Stoica, Alexandru D.

Payzant, E. Andrew

Almer, Jonathan

Shi, Donglu

TI Consecutive Nucleation Events During Divetrification of

Zr52.5Cu17.9Ni14.6Al10Ti5 Bulk Metallic Glass

SO ADVANCED ENGINEERING MATERIALS

RI Wang, Xun-Li/C-9636-2010; Payzant, Edward/B-5449-2009; Stoica,

Alexandru/K-3614-2013; Lu, Zhao-Ping/A-2718-2009

OI Wang, Xun-Li/0000-0003-4060-8777; Payzant, Edward/0000-0002-3447-2060;

Stoica, Alexandru/0000-0001-5118-0134; Lu, Zhao-Ping/0000-0003-1463-8948

SN 1438-1656

PD NOV

PY 2008

VL 10

IS 11

BP 1043

EP 1047

DI 10.1002/adem.200800129

UT WOS:000261933400011

ER

PT J

AU Qiao, DC

Fan, C

Liaw, PK

Choo, H

AF Qiao, Dongchun

Fan, Cang

Liaw, Peter K.

Choo, Hahn

TI Crystallization kinetics of the (Zr58Ni13.6Cu18Al10.4)Nb-1 bulk metallic

glass

SO ADVANCED ENGINEERING MATERIALS

RI Choo, Hahn/A-5494-2009

OI Choo, Hahn/0000-0002-8006-8907

SN 1438-1656

PD AUG

PY 2006

VL 8

IS 8

BP 714

EP 719

DI 10.1002/adem.200500282

UT WOS:000240388600009

ER

PT S

AU Laws, KJ

Shamlaye, KF

Gun, B

Wong, K

Ferry, M

AF Laws, K. J.

Shamlaye, K. F.

Gun, B.

Wong, K.

Ferry, M.

BE Chandra, T

Wanderka, N

Reimers, W

Ionescu, M

TI The Prediction of Glass-Forming Compositions in Metallic Systems - The

Development of New Bulk Metallic Glasses

SO THERMEC 2009, PTS 1-4

SE Materials Science Forum

CT 6th International Conference on Processing and Manufacturing of Advanced

Materials

CY AUG 25-29, 2009

CL Berlin, GERMANY

SP Minerals, Met & Mat Soc

AB A novel methodology of predicting specific compositions for glass forming alloys based on elemental cluster selection, liquidus lines, atomic packing efficiency and ab initio calculations is presented and discussed The proposed composition selection model has lead to the discovery of a number of novel, soon to be reported Mg, Cu, Zn and Ag-based bulk metallic glasses The proposed model may also be used to explain high glass forming ability and physical properties of known BMG compositions and to pin-point new or superior BMG compositions in existing glass forming systems. Further, the aforementioned model shows strong correlations between proposed elemental clusters, glass forming ability and BMG ductility. This model has also shown applicable adaptation to known ceramic oxide glass forming systems.

SN 0255-5476

PY 2010

VL 638-642

BP 1637

EP 1641

DI 10.4028/www.scientific.net/MSF.638-642.1637

PN 1-4

UT WOS:000281043800271

ER

PT J

AU Qin, FX

Xie, GQ

Wada, T

Zhu, SL

Dan, ZH

AF Qin, Fengxiang

Xie, Guoqiang

Wada, Takeshi

Zhu, Shengli

Dan, Zhenhua

TI Electrochemical Properties of Porous Pd-Based Bulk Metallic Glasses

SO MATERIALS TRANSACTIONS

CT 5th International Symposium on Designing, Processing and Properties of

Advanced Engineering Materials (ISAEM)

CY NOV 05-08, 2012

CL Toyohashi, JAPAN

SP Japan Soc Promot Sci, 176th Comm Proc Created Mat Funct

AB The corrosion behavior of porous Pd42.5Cu30Ni7.5P20 bulk metallic glasses with various porosities in Hanks' solution was investigated. The results revealed that all bulk metallic glasses exhibited similar corrosion behavior. The bulk metallic glasses with the porosity of 0 and 18% were spontaneously passivated with passive current densities between 10(-2) and 10(-1) A/m(2) in anodic polarization curves. The bulk metallic glasses with 40 and 55% porosity exhibited an anodic process with the current density increasing gradually. As the anodic potential increasing, no obvious passivity breakdown occurred in the anodic polarization process. The polarization resistance decreased with the increasing of the porosity from 0 to 55%. The breakdown potential of the passive film for the bulk metallic glass with 55% porosity was about 100 mV lower than that of the 0% porosity Pd42.5Cu30Ni7.5P20 bulk metallic glass.

RI Zhu, Shengli/D-5281-2009; Wada, Takeshi/B-2431-2015; BAI,

JIE/D-7448-2016; Xie, Guoqiang/A-8619-2011

OI Zhu, Shengli/0000-0002-0190-2626; Dan, Zhenhua/0000-0002-3026-685X

SN 1345-9678

EI 1347-5320

PD AUG

PY 2013

VL 54

IS 8

BP 1347

EP 1350

DI 10.2320/matertrans.MF201314

UT WOS:000325122900020

ER

PT S

AU Fu, MW

Chan, WL

AF Fu, Ming Wang

Chan, Wai Lun

BA Fu, MW

Chan, WL

BF Fu, MW

Chan, WL

TI Micropart Fabrication Using Bulk Metallic Glasses

SO MICRO-SCALED PRODUCTS DEVELOPMENT VIA MICROFORMING: DEFORMATION

BEHAVIOURS, PROCESSES, TOOLING AND ITS REALIZATION

SE Springer Series in Advanced Manufacturing

OI Fu, M.W./0000-0002-0409-9701

SN 1860-5168

BN 978-1-4471-6326-8; 978-1-4471-6325-1

PY 2014

BP 151

EP 175

DI 10.1007/978-1-4471-6326-8_6

D2 10.1007/978-1-4471-6326-8

UT WOS:000339963700007

ER

PT J

AU Espallargas, N

Aune, RE

Torres, C

Papageorgiou, N

Munoz, AI

AF Espallargas, N.

Aune, R. E.

Torres, C.

Papageorgiou, N.

Munoz, A. I.

TI Bulk metallic glasses (BMG) for biomedical applications-A tribocorrosion

investigation of Zr55Cu30Ni5Al10 in simulated body fluid (vol 301, pg

271, 2013)

SO WEAR

RI Espallargas, Nuria/F-9346-2016

SN 0043-1648

EI 1873-2577

PD JUL 30

PY 2013

VL 305

IS 1-2

BP 248

EP 248

DI 10.1016/j.wear.2013.07.004

UT WOS:000341961800025

ER

PT J

AU Zong, HT

Geng, CC

Kang, CY

Cao, GH

Bian, LY

Li, LX

Zhang, BQ

Li, M

AF Zong, Haitao

Geng, Chenchen

Kang, Chaoyang

Cao, Guohua

Bian, Linyan

Li, Lixin

Zhang, Baoqing

Li, Ming

TI Excellent glass forming ability and plasticity in high entropy

Zr20Ti20Hf20M20Be20 (M = Cu, Ni, Co) alloys

SO RESULTS IN PHYSICS

AB We reported here the studies of a series of Zr20Ti20Hf20M20Be20 (M = Cu, Ni and Co) quinary high entropy bulk metallic glasses. Glasses with critical diameters (D-c) of 3 mm, 8 mm and 5 mm, respectively has been successfully fabricated by copper mold casting. Strikingly, a plastic strain of 11.6% is achieved in the Zr20Ti20Hf20Cu20Be20 metallic glass. The dynamic fragility the Zr20Ti20Hf20Cu20Be20 alloy is determined from calorimetric measurements. The excellent plasticity is explained to be attributed to relatively higher fragility. (C) 2017 The Authors. Published by Elsevier B.V.

SN 2211-3797

PD MAR

PY 2018

VL 8

BP 253

EP 256

DI 10.1016/j.rinp.2017.12.002

UT WOS:000428027700039

ER

PT J

AU Nakai, Y

Sakai, K

Nakagawa, K

AF Nakai, Yoshikazu

Sakai, Koji

Nakagawa, Kenichi

TI Fatigue of Zr-based Bulk Metallic Glass Under Compression-compression

Stress

SO ADVANCED ENGINEERING MATERIALS

RI Nakai, Yoshikazu/N-2185-2016; Totsukawa, Nobuhisa/D-2028-2017

OI Nakai, Yoshikazu/0000-0002-4146-6670;

SN 1438-1656

PD NOV

PY 2008

VL 10

IS 11

BP 1026

EP 1029

DI 10.1002/adem.200800141

UT WOS:000261933400007

ER

PT J

AU Georgarakis, K

Yavari, AR

Louzguine, DV

Vaughan, G

Botta, WJ

AF Georgarakis, K.

Yavari, A. R.

Louzguine, D. V.

Vaughan, G.

Botta, W. J.

TI Atomic structure of bulk metallic glasses and their supercooled liquid

states probed by high-energy synchrotron light

SO COMPTES RENDUS PHYSIQUE

AB Like non-metallic glasses, many bulk metallic glasses manifest a glass-transition temperature T-g during heating prior to crystallisation. While the exact nature of the atomic structure of a metallic glass depends on its thermo-mechanical history (quench-rate, plastic deformation, ... ), a unique and reproducible average atomic structure is attained if the glass transition temperature can be approached in a reversible manner. However, a metallic glass is always metastable and crystallises within a time t near or above its T-g in such a way that any reciprocal or real-space information on the fully glassy state at T >= T-g must be completed within acquisition times tau(a) << t and this condition is in general difficult to attain with conventional X-ray diffraction devices.

Here we report on experiments using high-energy, high-flux synchrotron light in the transmission for probing of the atomic structure of bulk metallic glasses. Examples are given of the determination of the isochoric glass transition T-g and the quenched-in free-volume. Finally, we report on the evolution of the atomic structure in the supercooled liquid region (T > T-g) and its role in the enhancement of glass formability. (C) 2011 Academie des sciences. Published by Elsevier Masson SAS. All rights reserved.

RI yavari, alain/E-8192-2010; Botta, Walter/E-7763-2010; Georgarakis,

Konstantinos/K-1939-2015; Georgarakis, Konstantinos/E-6390-2010;

LOUZGUINE, Dmitri/D-2492-2010

OI Botta, Walter/0000-0003-2759-573X; LOUZGUINE,

Dmitri/0000-0001-5716-4987; Georgarakis,

Konstantinos/0000-0003-0918-7310

SN 1631-0705

PD APR

PY 2012

VL 13

IS 3

BP 218

EP 226

DI 10.1016/j.crhy.2011.12.010

UT WOS:000301965100003

ER

PT J

AU Wei, HQ

Long, ZL

Xu, F

Zhang, P

Tang, Y

AF Wei Hong-Qing

Long Zhi-Lin

Xu Fu

Zhang Ping

Tang Yi

TI Study of Cu(45)Zr(55-x)Alx (x=3, 7, 12) bulk metallic glasses by

ab-initio molecular dynamics simulation

SO ACTA PHYSICA SINICA

AB Local structural changes from liquid to amorphous state in three Cu(45)Zr(55-x)Alx ( x = 3, 7, 12) ternary metallic glasses have been investigated by the ab initio molecular dynamics simulation. The atomic structure of the glasses has been analyzed by means of bond-type index method in Honeycutt-Andersen and Voronoi tessellation method. Alcentered icosahedral clusters are identified as the basic local structural units and these Al-centered stable clusters play a key role in the structural heterogeneity and glass-forming ability of the Cu-Zr-Al bulk metallic glasses.

SN 1000-3290

PD JUN

PY 2014

VL 63

IS 11

AR 118101

DI 10.7498/aps.63.118101

UT WOS:000338747800043

ER

PT J

AU Wu, Y

Song, WL

Zhou, J

Cao, D

Wang, H

Liu, XJ

Lu, ZP

AF Wu Yuan

Song Wen-Li

Zhou Jie

Cao Di

Wang Hui

Liu Xiong-Jun

Lu Zhao-Ping

TI Ductilization of bulk metallic glassy material and its mechanism

SO ACTA PHYSICA SINICA

AB Bulk metallic glass has aroused intensive interest due to its unique atomic structure and properties, while its structural application is restricted by the shortcomings of its mechanical properties-room temperature brittleness and strain softening. To make up for these shortcomings, various approaches have been proposed, including tailoring intrinsic parameters such as elastic modulus and structural heterogeneity, and changing stress state or defect concentration. Bulk metallic glass composites with ex-situ added or in-situ formed crystallites have been fabricated, series of bulk metallic glasses and their composites with good mechanical properties have been designed, especially TRIP (transformation-induced plasticity)-reinforced bulk metallic glass composites with large tensile ductility and work-hardening. In this paper, we review the ductilization of bulk metallic glass and its composites, as well as the related mechanism. Particularly, fabrication, properties, structure control and the ductilization mechanism of TRIP-reinforced bulk metallic glass composite are introduced in detail. A perspective of the challenges of ductilization of bulk metallic glassy materials is also mentioned briefly.

RI Lu, Zhao-Ping/A-2718-2009

OI Lu, Zhao-Ping/0000-0003-1463-8948

SN 1000-3290

PD SEP 5

PY 2017

VL 66

IS 17

AR 176111

DI 10.7498/aps.66.176111

UT WOS:000412842000011

ER

PT J

AU Yi, J

Wang, WH

Lewandowski, JJ

AF Yi, Jun

Wang, Wei Hua

Lewandowski, John J.

TI Guiding and Deflecting Cracks in Bulk Metallic Glasses to Increase

Damage Tolerance

SO ADVANCED ENGINEERING MATERIALS

RI Lewandowski, John/S-3815-2017

OI Lewandowski, John/0000-0002-3389-2637

SN 1438-1656

EI 1527-2648

PD MAY

PY 2015

VL 17

IS 5

BP 620

EP 625

DI 10.1002/adem.201400209

UT WOS:000354417300006

ER

PT J

AU Zong, HT

Ma, MZ

Li, LX

Jing, Q

Zhang, BQ

Liu, RP

AF Zong Haitao

Ma Mingzhen

Li Lixin

Jing Qin

Zhang Baoqing

Liu Riping

TI Effect of Al Addition on the Mechanical Properties and Microstructure of

Zr35Ti30Cu7.5Be27.5 Bulk Metallic Glass

SO RARE METAL MATERIALS AND ENGINEERING

AB The effect of partial substitution of Al for Zr on the mechanical properties of Zr35-xTi30Cu7.5Be27.5Alx (x=0, 1, 1.5, 2, 2.5, 5, at%) bulk metallic glasses were studied by uniaxial compression test. The results showed that the compressive plasticity can be greatly improved from 0.95% (x=0) to 15.10% (x=1.5) and 3.45% (x=2) in Zr35Ti30Cu7.5Be27.5 bulk metallic glass. The fracture morphologies of the Zr35-xTi30Cu7.5Be27.5Alx bulk metallic glasses were characterized by means of scanning electron microscope (SEM). Transmission electron microscope (TEM) was employed to investigate the microstructures of the bulk metallic glasses containing different amounts of Al. It is found that the enhanced compressive plasticity could be attributed to the nanoscale heterogeneity in the bulk metallic glasses. The effect of Al addition on the microstructure and deformation behavior of the Zr-based bulk metallic glasses was discussed in terms of different critical shear stress (CSS).

SN 1002-185X

PD MAR

PY 2013

VL 42

IS 3

BP 470

EP 473

UT WOS:000317085900007

ER

PT J

AU Yang, BJ

Yao, JH

Zhang, J

Yang, HW

Wang, JQ

Ma, E

AF Yang, B. J.

Yao, J. H.

Zhang, J.

Yang, H. W.

Wang, J. Q.

Ma, E.

TI Al-rich bulk metallic glasses with plasticity and ultrahigh specific

strength

SO SCRIPTA MATERIALIA

AB Aluminum-based amorphous metals are interesting lightweight alloys with superior mechanical and corrosion properties, but have never been achieved in bulk form. Here we report the first success of obtaining Al-rich (86 at.% Al) bulk metallic glasses (BMGs), based on an alloy composition designed from the preferable internal glass structure. The Al BMGs discovered exhibit ultrahigh specific strength, as well as obvious plasticity. (C) 2009 Acta Materialia Inc. Published by Elsevier Ltd. All rights reserved.

RI Yang, Hongwang/D-3490-2011; Ma, En/A-3232-2010

OI Yang, Hongwang/0000-0003-1894-2242;

SN 1359-6462

PD AUG

PY 2009

VL 61

IS 4

BP 423

EP 426

DI 10.1016/j.scriptamat.2009.04.035

UT WOS:000267500300022

ER

PT J

AU Cheng, M

Wert, JA

AF Cheng, Ming

Wert, John A.

TI Modeling of microimprinting of bulk metallic glasses

SO JOURNAL OF MATERIALS SCIENCE & TECHNOLOGY

AB A finite element analysis (FEA) model has been developed to analyze microimprinting of bulk metallic glasses (BMG) near the glass transition temperature (T-g). The results reveal an approximately universal imprinting response for BMG, independent of surface feature length scale. The scale-independent nature of BMG imprinting derives from the flow characteristics of BMG in the temperature range above T-g. It also shows that the lubrication condition has a mild influence on BMG imprinting in the temperature range above T-g.

SN 1005-0302

PD NOV

PY 2006

VL 22

IS 6

BP 851

EP 854

UT WOS:000242638500025

ER

PT B

AU Pelletier, JM

Van de Moortele, B

AF Pelletier, JM

Van de Moortele, B

BE Fisher, DJ

TI Mechanical spectroscopy of bulk metallic glasses

SO DEFECTS AND DIFFUSION IN METALS: AN ANNUAL RETROSPECTIVE IV

SE DEFECT AND DIFFUSION FORUM

CT Summer School on Mechanical Spectroscop Q-1

CY 2001

CL AUSSOIS, FRANCE

AB Two bulk metallic glasses have been studied by mechanical spectroscopy through the glass transition range. The alpha-relaxation peak observed can be interpreted by the onset of long range movements in the supercooled liquid region. At lower temperature, a sub-Tg relaxation process is observed in the Pd-Cu-Ni-P alloy, but not in the Zr-Ti-Cu-Ni-Be alloy. An explanation involving the possibility of relaxation of clustered units around the P atoms is proposed. At high temperature crystallisation occurs and leads to a decrease of internal friction.

BN 3-908450-69-1

PY 2002

VL 203-2

BP 265

EP 268

DI 10.4028/www.scientific.net/DDF.203-205.265

UT WOS:000175228400023

ER

PT J

AU Das, J

Tang, MB

Kim, KB

Theissmann, R

Baier, F

Wang, WH

Eckert, J

AF Das, J

Tang, MB

Kim, KB

Theissmann, R

Baier, F

Wang, WH

Eckert, J

TI "Work-hardenable" ductile bulk metallic glass

SO PHYSICAL REVIEW LETTERS

AB Usually, monolithic bulk metallic glasses undergo inhomogeneous plastic deformation and exhibit poor ductility (< 1%) at room temperature. We present a new class of bulk metallic glass, which exhibits high strength of up to 2265 MPa together with extensive "work hardening" and large ductility of 18%. Significant increase in the flow stress was observed during deformation. The "work-hardening" capability and ductility of this class of metallic glass is attributed to a unique structure correlated with atomic-scale inhomogeneity, leading to an inherent capability of extensive shear band formation, interactions, and multiplication of shear bands.

RI Das, Jayanta/G-1559-2010

OI Das, Jayanta/0000-0001-8750-5463

SN 0031-9007

EI 1079-7114

PD MAY 27

PY 2005

VL 94

IS 20

AR 205501

DI 10.1103/PhysRevLett.94.205501

UT WOS:000229398100034

PM 16090260

ER

PT J

AU Wu, JL

Pan, Y

Pi, JH

AF Wu, J. L.

Pan, Y.

Pi, J. H.

TI Evaluation of Cu-Zr-Ti-In Bulk Metallic Glasses via Nanoindentation

SO JOURNAL OF MATERIALS ENGINEERING AND PERFORMANCE

AB In this paper, the mechanical properties of two bulk metallic glasses, Cu60Zr34Ti5In1 and Cu58Zr34Ti5In3, have been evaluated by nanoindentation tests. The hardness and Young's modulus of as-cast Cu60Zr34Ti5In1 bulk metallic glass increase from the center to the edge of the rod, but Cu58Zr34Ti5In3 shows an inverse trend. The Young's modulus of Cu58Zr34Ti5In3 is lower than that of Cu60Zr34Ti5In1. For both bulk metallic glasses, serrations on the nanoindentation load-displacement curves depend not only on loading rate but also on the maximum load. Alternatively, the onset position of the appearance of serrations on the curves increases with the loading rate. In addition, the hardness of both samples decreases as the size of the indent increases due to indentation size effects.

SN 1059-9495

PD AUG

PY 2013

VL 22

IS 8

BP 2288

EP 2292

DI 10.1007/s11665-013-0519-x

UT WOS:000322013000019

ER

PT J

AU Na, JH

Demetriou, MD

Johnson, WL

AF Na, Jong Hyun

Demetriou, Marios D.

Johnson, William L.

TI Fragility of iron-based glasses

SO APPLIED PHYSICS LETTERS

AB The viscosity of various iron-based bulk-glass-forming liquids is measured around the glass transition, and the associated fragility is calculated. Fragility is found to vary broadly between compositions, from a low value of similar to 43, which indicates fairly "strong" liquid behavior, to similar to 65, well within the region of "fragile" behavior. Despite a strong covalent bonding identified in the structure of this class of metal/metalloid glasses, their liquid fragility can be remarkably high, exceeding even the very fragile palladium and platinum bulk-glass formers. An inverse correlation between glass-forming ability and fragility is identified, suggesting that iron-based glasses are effectively "kinetically" stabilized. (C) 2011 American Institute of Physics. [doi: 10.1063/1.3651763]

SN 0003-6951

EI 1077-3118

PD OCT 17

PY 2011

VL 99

IS 16

AR 161902

DI 10.1063/1.3651763

UT WOS:000296517600026

ER

PT J

AU Liu, XR

Hong, SM

AF Liu, X. R.

Hong, S. M.

TI Evidence for a pressure-induced phase transition of amorphous to

amorphous in two lanthanide-based bulk metallic glasses (vol 90, art no

251903, 2007)

SO APPLIED PHYSICS LETTERS

SN 0003-6951

PD JAN 14

PY 2008

VL 92

IS 2

AR 029906

DI 10.1063/1.2823581

UT WOS:000252470900134

ER

PT J

AU Jia, P

Zhu, ZD

Ma, E

Xu, J

AF Jia, Peng

Zhu, Zhen-dong

Ma, Evan

Xu, Jian

TI Notch toughness of Cu-based bulk metallic glasses

SO SCRIPTA MATERIALIA

AB We have measured the notch toughness for a range of Cu-based bulk metallic glasses (BMGs). Alloying additions that enhance the glass-forming ability (GFA) are found to degrade the toughness. Within this particular family of Cu-BMGs, toughness is more directly correlated with strength (and consequently with the shear modulus or glass transition temperature) than with the Poisson's ratio. The Cu49Hf42Al9 BMG exhibits the best combination of GFA and toughness. (C) 2009 Acta Materialia Inc. Published by Elsevier Ltd. All rights reserved.

RI Ma, En/A-3232-2010

SN 1359-6462

PD JUL

PY 2009

VL 61

IS 2

BP 137

EP 140

DI 10.1016/j.scriptamat.2009.03.024

UT WOS:000266840800008

ER

PT B

AU Giordano, VM

Tlili, A

AF Giordano, Valentina M.

Tlili, Ameni

BE Termentzidis, K

TI Amorphous/Nanocrystalline Composites: Recrystallization

SO NANOSTRUCTURED SEMICONDUCTORS: AMORPHIZATION AND THERMAL PROPERTIES

BN 978-1-315-36445-2; 978-981-4745-64-2

PY 2017

BP 317

EP 349

D2 10.1201/b15634

UT WOS:000413118200015

ER

PT J

AU Evenson, Z

Busch, R

AF Evenson, Zach

Busch, Ralf

TI Equilibrium viscosity, enthalpy recovery and free volume relaxation in a

Zr44Ti11Ni10Cu10Be25 bulk metallic glass (vol 59, pg 4404, 2011)

SO ACTA MATERIALIA

SN 1359-6454

PD APR

PY 2013

VL 61

IS 6

BP 2282

EP 2282

DI 10.1016/j.actamat.2013.01.009

UT WOS:000316241400043

ER

PT J

AU Jiang, WH

Qiu, KQ

Liu, FX

Choo, H

Liaw, PK

AF Jiang, Wenhiu

Qiu, Keqiang

Liu, Fengxiao

Choo, Hahn

Liaw, Peter K.

TI Compressive deformation and fracture of a hollow bulk-metallic glass

SO ADVANCED ENGINEERING MATERIALS

RI Choo, Hahn/A-5494-2009

OI Choo, Hahn/0000-0002-8006-8907

SN 1438-1656

PD MAR

PY 2007

VL 9

IS 3

BP 147

EP 150

DI 10.1002/adem.200600249

UT WOS:000245190400006

ER

PT J

AU Guo, S

Lu, ZP

Liu, CT

AF Guo, Sheng

Lu, Z. P.

Liu, C. T.

TI Identify the best glass forming ability criterion

SO INTERMETALLICS

AB Understanding glass formation and predicting glass forming ability (GFA) are vitally important and they are long-standing challenges in the metallic glasses community. Recently, a number of criteria have been developed to evaluate GFA, based mainly on fitting the experimental data of the critical cooling rate for glass forming. In this study, physically accepted boundary conditions have been imposed to evaluate the GFA criteria, and their combination with statistical analysis result in identifying the best GFA criterion useful for various glass forming systems, including oxide glasses, cryoprotectants, and metallic glasses. (C) 2009 Elsevier Ltd. All rights reserved.

RI Guo, Sheng/C-7746-2009; Lu, Zhao-Ping/A-2718-2009

OI Guo, Sheng/0000-0001-8349-3135; Lu, Zhao-Ping/0000-0003-1463-8948; Liu,

Chain Tsuan/0000-0001-7888-9725

SN 0966-9795

PD MAY

PY 2010

VL 18

IS 5

BP 883

EP 888

DI 10.1016/j.intermet.2009.12.025

UT WOS:000277494400020

ER

PT J

AU Liu, ZY

Yang, Y

Guo, S

Liu, XJ

Lu, J

Liu, YH

Liu, CT

AF Liu, Z. Y.

Yang, Y.

Guo, S.

Liu, X. J.

Lu, J.

Liu, Y. H.

Liu, C. T.

TI Cooling rate effect on Young's modulus and hardness of a Zr-based

metallic glass

SO JOURNAL OF ALLOYS AND COMPOUNDS

AB It is known that cooling rate can affect the atomic structure and thus may possibly affect the mechanical properties of metallic glasses (MGs). In spite of the considerable efforts on the cooling rate, its effect on the mechanical properties is controversial at the present time. In this study, we present a micromechanical study of the cooling-rate effect on Young's moduli and hardness of the cast bulks and melt-spun ribbons for a Zr(55)Pd(10)Cu(20)Ni(5)Al(10) metallic glass. Using the classic nanoindentation method, the Young's moduli of the ribbon samples obtained at higher cooling rates were measured which appeared to be much lower than those of the bulk samples. However, through further experiments on slice samples cut from the as-cast bulks and finite-element (FE) analyses, we have clearly demonstrated that the measured difference in elastic moduli was mainly caused by the sample thickness effect in nanoindentation tests. To overcome such a confounding effect, microcompression experiments were performed on the as-cast and as-spun MG samples, respectively. Being consistent with the findings from nanoindentation, the microcompression results showed that the cooling rate, as ranging from similar to 10(2) to similar to 10(6) K/s, essentially has no influence on the Young's modulus and hardness of the metallic glasses. (C) 2010 Elsevier B.V. All rights reserved.

RI Guo, Sheng/C-7746-2009; LIU, Yanhui/B-1485-2009; Liu,

Xiong-Jun/C-7119-2009; Yang, Yong/G-9148-2011; Liu, XJ/G-4152-2010

OI Guo, Sheng/0000-0001-8349-3135; Yang, Yong/0000-0002-0491-8295; Liu,

Chain Tsuan/0000-0001-7888-9725

SN 0925-8388

PD FEB 17

PY 2011

VL 509

IS 7

BP 3269

EP 3273

DI 10.1016/j.jallcom.2010.12.095

UT WOS:000287570100014

ER

PT J

AU Chu, ZH

Yuan, GY

Hidemi, K

Ding, WJ

AF Chu Zhenhua

Yuan Guangyin

Hidemi, Kato

Ding Wenjiang

TI Toughening Mechanism of Zr-Based and Cu-Based Bulk Metallic Glasses

Composites by TiNb Particle

SO RARE METAL MATERIALS AND ENGINEERING

AB TiNb/Zr55Cu30Al10Ni5 and TiNb/Cu46Zr42Al7Y5 bulk metallic glass composites were prepared by Spark Plasma Sintering (SPS). The compression results show that TiNb/Zr55Cu30Al10Ni5 composites get larger plasticity than TiNb/Cu46Zr42Al7Y5 composites. The reason is that Zr55Cu30Al10Ni5 bulk metallic glass is resistant to micro-crack. The fracture toughness test proves that K-C of Zr55Cu30Al10Ni5 is higher than that of Cu46Zr42Al7Y5, so there is a larger length scale of plastic process zone R-P for Zr55Cu30Al10Ni5.

RI Kato, Hidemi/B-2492-2015

OI Ding, Wenjiang/0000-0003-0948-8025

SN 1002-185X

PD JUN

PY 2013

VL 42

IS 6

BP 1154

EP 1158

UT WOS:000321474500013

ER

PT J

AU Mu, J

Fu, HM

Zhu, ZW

Wang, AM

Li, H

Hu, ZQ

Zhang, HF

AF Mu, Juan

Fu, Huameng

Zhu, Zhengwang

Wang, Aimin

Li, Hong

Hu, Zhuangqi

Zhang, Haifeng

TI Synthesis and Properties of Al-Ni-La Bulk Metallic Glass

SO ADVANCED ENGINEERING MATERIALS

RI Zhu, Zheng-Wang/D-2799-2017; wang, am/P-2147-2016

SN 1438-1656

EI 1527-2648

PD JUL

PY 2009

VL 11

IS 7

BP 530

EP 532

DI 10.1002/adem.200900100

UT WOS:000269090500002

ER

PT J

AU Hufnagel, TC

AF Hufnagel, TC

TI Preface to the viewpoint set on mechanical behavior of metallic glasses

SO SCRIPTA MATERIALIA

RI Hufnagel, Todd/A-3309-2010

OI Hufnagel, Todd/0000-0002-6373-9377

SN 1359-6462

PD FEB

PY 2006

VL 54

IS 3

BP 317

EP 319

DI 10.1016/j.scriptamat.2005.10.004

UT WOS:000233495600001

ER

PT J

AU Qiao, JC

Chen, YX

Pelletier, JM

Kato, H

Crespo, D

Yao, Y

Khonik, VA

AF Qiao, J. C.

Chen, Y. X.

Pelletier, J. M.

Kato, H.

Crespo, D.

Yao, Y.

Khonik, V. A.

TI Viscoelasticity of Cu- and La-based bulk metallic glasses:

Interpretation based on the quasi-point defects theory

SO MATERIALS SCIENCE AND ENGINEERING A-STRUCTURAL MATERIALS PROPERTIES

MICROSTRUCTURE AND PROCESSING

AB The dynamic mechanical relaxation of metallic glasses is closely associated with the physical and mechanical properties. In the current work, the dynamic mechanical relaxation behaviors of Cu46Zr45Al7Y2 and La65Al14(Cu5/6Ag1/6)(11)(Ni1/2Co1/2)(10) bulk metallic glasses are investigated by mechanical spectroscopy. In general, metallic glasses display two relaxation modes: main (alpha) relaxation and the slow secondary (beta) relaxation. The a relaxation is linked to the dynamic glass transition phenomenon and viscous flow while the slow beta relaxation is associated with many fundamental issues, such as diffusion and glass transition phenomenon. The experimental study shows La65Al14(Cu5/6Ag1/6)(11)(Ni1/2Co1/2)(10) bulk metallic glass displays a noticeable slow beta relaxation. Contrarily, the Cu46Zr45Al7Y2 bulk metallic glass relaxation process takes the form of an "excess wing". In the framework of quasi-point defects (QPD) theory, the dynamic mechanical response of the metallic glasses is discussed.

RI Kato, Hidemi/B-2492-2015; Yao, Yao/K-3129-2012

OI Yao, Yao/0000-0002-0879-4269

SN 0921-5093

EI 1873-4936

PD MAR 14

PY 2018

VL 719

BP 164

EP 170

DI 10.1016/j.msea.2018.02.046

UT WOS:000429390300018

ER

PT B

AU Liu, CT

Greer, AL

Schuh, CA

AF Liu, C. T.

Greer, A. Lindsay

Schuh, Christopher A.

BA Suryanarayana, C

Inoue, A

BF Suryanarayana, C

Inoue, A

TI Synthesis of Bulk Metallic Glasses

SO BULK METALLIC GLASSES

BN 978-1-4200-8596-9

PY 2011

BP 145

EP 186

D2 10.1201/9781420085976-1

UT WOS:000288438000005

ER

PT J

AU Huang, YJ

Fan, HB

Wang, DJ

Sun, Y

Liu, FY

Shen, J

Sun, JF

Mi, J

AF Huang, Yongjiang

Fan, Hongbo

Wang, Dongjun

Sun, Yu

Liu, Fangyu

Shen, Jun

Sun, Jianfei

Mi, J.

TI The effect of cooling rate on the wear performance of a ZrCuAlAg bulk

metallic glass (vol 58, pg 284, 2014)

SO MATERIALS & DESIGN

RI Huang, Yongjiang/D-4809-2009

SN 0264-1275

EI 1873-4197

PD APR 5

PY 2015

VL 70

BP 82

EP 82

DI 10.1016/j.matdes.2014.12.039

UT WOS:000349620400009

ER

PT J

AU de Oliveira, MF

Botta, WJ

Kiminami, CS

Inoue, A

Yavari, AR

AF de Oliveira, MF

Botta, WJ

Kiminami, CS

Inoue, A

Yavari, AR

TI Electromechanical engraving and writing on bulk metallic glasses

SO APPLIED PHYSICS LETTERS

AB Using the intrinsic materials properties of bulk metallic glasses (BMG), namely electrical resistivities two orders of magnitude higher than good conductors and a Newtonian viscous-flow regime of deformability, a new electromechanical process has been developed for engraving on BMGs. Viscous flow in the supercooled liquid region between the glass transition temperature T-g and the crystallization temperature T-x of the bulk metallic glass allows rapid electromechanical engraving at low applied stresses. Miniaturization of the process is expected to allow submicron engraving in the future. (C) 2002 American Institute of Physics.

RI Botta, Walter/E-7763-2010; de Oliveira, Marcelo/B-9881-2012; Inoue,

Akihisa/E-5271-2015; yavari, alain/E-8192-2010; KIMINAMI,

CLAUDIO/D-4402-2012

OI Botta, Walter/0000-0003-2759-573X; de Oliveira,

Marcelo/0000-0003-4589-2463; KIMINAMI, CLAUDIO/0000-0001-8231-7316

SN 0003-6951

PD AUG 26

PY 2002

VL 81

IS 9

BP 1606

EP 1608

DI 10.1063/1.1502008

UT WOS:000177549200022

ER

PT S

AU Hofmann, DC

Johnson, WL

AF Hofmann, Douglas C.

Johnson, William L.

BE Zhao, Y

Liao, X

TI Improving Ductility in Nanostructured Materials and Metallic Glasses:

"Three Laws"

SO DUCTILITY OF BULK NANOSTRUCTURED MATERIALS

SE Materials Science Forum

AB Nanostructured materials and bulk metallic glasses are relatively new classes of engineering materials that have promise for unique metals applications. However, both these materials suffer from limited room temperature ductility in unconfined loading geometries. In this work, we present three experimental rules that we have observed to be necessary to toughen bulk metallic glasses. We reason that adaptations to these rules may provide the solution for toughening nanostructured composites and other brittle materials.

SN 0255-5476

PY 2010

VL 633-634

BP 657

EP 663

DI 10.4028/www.scientific.net/MSF.633-634.657

UT WOS:000276161200053

ER

PT J

AU Laws, KJ

Cao, JD

Reddy, C

Shamlaye, KF

Gun, B

Ferry, M

AF Laws, Kevin J.

Cao, Jake D.

Reddy, Cyndy

Shamlaye, Karl F.

Gun, Bulent

Ferry, Michael

TI Ultra magnesium-rich, low-density Mg-Ni-Ca bulk metallic glasses

SO SCRIPTA MATERIALIA

AB A range of low-density Mg-rich glass-forming compositions from the Mg-Ni-Ca alloy system have been discovered. Distinct maxima in the glass-forming ability were located and shown to produce samples of bulk metallic glass dimensions. These bulk glass-forming alloys exhibit the lowest density (<2 g cm(-3)) and highest Mg content (86 at.%) of all Mg-based bulk metallic glasses reported to date. Here, the specific alloy design method and a range properties of these amorphous alloys are revealed. (C) 2014 Acta Materialia Inc. Published by Elsevier Ltd. All rights reserved.

RI Gun, Bulent/J-1437-2016

OI Gun, Bulent/0000-0003-1992-6404

SN 1359-6462

PD OCT 1

PY 2014

VL 88

BP 37

EP 40

DI 10.1016/j.scriptamat.2014.06.002

UT WOS:000340341300010

ER

PT J

AU Bossuyt, S

AF Bossuyt, S

TI Spatial localization of the nucleation rate and formation of

inhomogeneous nanocrystalline dispersions in deeply undercooled glass

forming liquids

SO SCRIPTA MATERIALIA

RI Bossuyt, Sven/H-5660-2012

OI Bossuyt, Sven/0000-0002-9223-735X

SN 1359-6462

PD JUN 8

PY 2001

VL 44

IS 12

BP 2781

EP 2787

DI 10.1016/S1359-6462(01)00974-5

UT WOS:000170308300015

ER

PT J

AU Kapaklis, V

Schweiss, P

Politis, C

AF Kapaklis, V

Schweiss, P

Politis, C

TI Bulk amorphous and nanocrystalline reinforced Pd-based alloys:

Formation, structural, thermal and elastic properties

SO ADVANCED ENGINEERING MATERIALS

RI Kapaklis, Vassilios/A-6454-2013

OI Kapaklis, Vassilios/0000-0002-6105-1659

SN 1438-1656

EI 1527-2648

PD MAR

PY 2005

VL 7

IS 3

BP 123

EP 127

DI 10.1002/adem.200400193

UT WOS:000228363300007

ER

PT J

AU Molokanov, VV

Mikhailova, TN

Kliger, IA

Petrzhik, MI

AF Molokanov, VV

Mikhailova, TN

Kliger, IA

Petrzhik, MI

TI Comparative DSC-study of Ni70Mo10P20 and Zr65Al7.5Cu17.5Ni10 metallic

glasses in T-g-T-x temperature range

SO MATERIALS SCIENCE AND ENGINEERING A-STRUCTURAL MATERIALS PROPERTIES

MICROSTRUCTURE AND PROCESSING

CT 9th International Conference on Rapidly Quenched and Metastable

Materials

CY AUG 25-30, 1996

CL BRATISLAVA, SLOVAKIA

SP Off Strategy & Dev Soc Sci & Technol, European Commiss, Directorate Gen XII, AlliedSignal Inc, US, Deut Phys Gesell, Int Union Pure & Appl Phys, JOEL Ltd, Japan, Alps Elect Co Ltd, Japan, Perkin Elmer GmbH

AB We have studied a stability of undercooled liquid in the Ni70Mo10P20 and Zr65Al7.5Cu17.5Ni10 metallic glasses by differential scanning calorimetry and report how some thermal treatments can change the mechanism of devitrification and crystallization. (C) 1997 Elsevier Science S.A.

RI Molokanov, Vyacheslav/U-3454-2017

OI Molokanov, Vyacheslav/0000-0003-4664-463X; Petrzhik,

Mikhail/0000-0002-1736-8050

SN 0921-5093

EI 1873-4936

PD JUN 15

PY 1997

VL 226

BP 474

EP 478

DI 10.1016/S0921-5093(96)10667-5

UT WOS:A1997XJ84700090

ER

PT J

AU Xi, XK

Zhao, DQ

Pan, MX

Wang, WH

AF Xi, XK

Zhao, DQ

Pan, MX

Wang, WH

TI On the criteria of bulk metallic glass formation in MgCu-based alloys

SO INTERMETALLICS

AB Mg65Cu25RE10 bulk metallic glass (BMG) forming system with 'continuous' range of atomic size and electronegativity are obtained which allow us to systematically explore the BMG-forming characteristics. Both atomic size and electronegativity differences among the main constituents are found to be crucial for BMG formation. (c) 2004 Elsevier Ltd. All rights reserved.

SN 0966-9795

PD JUN

PY 2005

VL 13

IS 6

BP 638

EP 641

DI 10.1016/j.intermet.2004.10.003

UT WOS:000227668100010

ER

PT J

AU Park, KW

Lee, CM

Lee, MR

Fleury, E

Falk, ML

Lee, JC

AF Park, Kyoung-Won

Lee, Chang-Myeon

Lee, Mi-Rim

Fleury, Eric

Falk, Michael L.

Lee, Jae-Chul

TI Paradoxical phenomena between the homogeneous and inhomogeneous

deformations of metallic glasses

SO APPLIED PHYSICS LETTERS

AB Experiments in binary alloys demonstrate that metallic glasses exhibiting more plastic strain during homogeneous deformation tend to show lower global plasticity during inhomogeneous deformation. Testing of Cu-Zr binary alloys supports the hypothesis that the formation energy of a shear transformation zone, as extracted from the experimental data, is related to the homogeneous flow rate. We also report the microstructural aspects that control the global plasticity of metallic glasses in the light of structural disordering, softening, and shear localization.

RI Lee, Jae-Chul/M-8743-2017; Falk, Michael/A-8478-2008

OI Lee, Jae-Chul/0000-0002-9294-2163; Falk, Michael/0000-0002-8383-4259

SN 0003-6951

PD JAN 12

PY 2009

VL 94

IS 2

AR 021907

DI 10.1063/1.3064920

UT WOS:000262534900030

ER

PT J

AU Guan, PF

Fujita, T

Hirata, A

Liu, YH

Chen, MW

AF Guan, P. F.

Fujita, T.

Hirata, A.

Liu, Y. H.

Chen, M. W.

TI Structural Origins of the Excellent Glass Forming Ability of Pd40Ni40P20

SO PHYSICAL REVIEW LETTERS

AB We report a hybrid atomic packing scheme comprised of a covalent-bond-mediated "stereochemical" structure and a densely packed icosahedron in a bulk metallic glass Pd40Ni40P20. The coexistence of two atomic packing models can simultaneously satisfy the criteria for both the charge saturation of the metalloid element and the densest atomic packing of the metallic elements. The hybrid packing scheme uncovers the structural origins of the excellent glass forming ability of Pd40Ni40P20 and has important implications in understanding the bulk metallic glass formation of metal-metalloid alloys.

RI Hirata, Akihiko/A-4850-2010; LIU, Yanhui/B-1485-2009; Guan,

Pengfei/B-7653-2013; Fujita, Takeshi/B-1867-2009; CHEN,

Mingwei/A-4855-2010

OI Fujita, Takeshi/0000-0002-2318-0433; CHEN, Mingwei/0000-0002-8274-3099;

Chen, Mingwei/0000-0002-2850-8872

SN 0031-9007

EI 1079-7114

PD APR 24

PY 2012

VL 108

IS 17

AR 175501

DI 10.1103/PhysRevLett.108.175501

UT WOS:000303191100023

PM 22680882

ER

PT J

AU Guo, GQ

Wu, SY

Luo, S

Yang, L

AF Guo, Gu-Qing

Wu, Shi-Yang

Luo, Sheng

Yang, Liang

TI How Can Synchrotron Radiation Techniques Be Applied for Detecting

Microstructures in Amorphous Alloys?

SO METALS

SN 2075-4701

PD DEC

PY 2015

VL 5

IS 4

BP 2048

EP 2057

DI 10.3390/met5042048

UT WOS:000367545100019

ER

PT J

AU Zhang, Y

Pan, MX

Zhao, DQ

Wang, RJ

Wang, WH

AF Zhang, Y

Pan, MX

Zhao, DQ

Wang, RJ

Wang, WH

TI Formation of Zr-based bulk metallic glasses from low purity of materials

by yttrium addition

SO MATERIALS TRANSACTIONS JIM

AB Zr55Al15Ni10Cu20 bulk metallic glass is formed using low purity materials at a low vacuum with a small amount of yttrium addition. It is found that the glass forming ability, crystallization and melting process of the Zr55Al15Ni10Cu20 alloy are modified with yttrium addition, while the mechanical and elastic properties, such as hardness and Young's Modulus, are not obviously changed. The positive effect of yttrium addition on the glassy formation of the Zr55Al15Ni20Cu20 alloy is clarified.

RI ZHANG, Yong/B-7928-2009

OI ZHANG, Yong/0000-0002-6355-9923

SN 0916-1821

PD NOV

PY 2000

VL 41

IS 11

BP 1410

EP 1414

DI 10.2320/matertrans1989.41.1410

UT WOS:000166559500008

ER

PT J

AU Yu, P

Wang, RJ

Zhao, DQ

Bai, HY

AF Yu, P.

Wang, R. J.

Zhao, D. Q.

Bai, H. Y.

TI Anomalous temperature dependent elastic moduli of Ce-based bulk metallic

glass at low temperatures

SO APPLIED PHYSICS LETTERS

AB We report the abnormal temperature dependent elastic moduli of Ce-based bulk metallic glass (BMG) at low temperatures. Unlike other BMGs with stiffness mode, the Ce-based metallic glass exhibits an anomalous softening longitudinal acoustic mode with the decrease of temperature. Particularly, the bulk modulus shows a continuous decrease upon cooling indicating the softening of the BMG, which is completely different from that of other metallic glasses and conventional alloys. The physical origin of this abnormal elastic behavior is attributed to the alterative valences and electronic configurations at low temperatures. (c) 2007 American Institute of Physics.

SN 0003-6951

PD NOV 12

PY 2007

VL 91

IS 20

AR 201911

DI 10.1063/1.2813639

UT WOS:000251003500034

ER

PT S

AU Eckert, J

Seidel, M

Schlorke, N

Kubler, A

Schultz, L

AF Eckert, J

Seidel, M

Schlorke, N

Kubler, A

Schultz, L

BE Fiorani, D

Magini, M

TI Solid state processing of bulk metallic glass forming alloys

SO SYNTHESIS AND PROPERTIES OF MECHANICALLY ALLOYED AND NANOCRYSTALLINE

MATERIALS, PTS 1 AND 2 - ISMANAM-96

SE Materials Science Forum

CT International Symposium on Metastable, Mechanically Alloyed and

Nanocrystalline Materials (ISMANAM-96)

CY MAY 20-24, 1996

CL ROME, ITALY

SP Natl Agcy Energy Environm & New Technol, CNR, CSM, INCM q, INFM, CNR, GNSM, CNR, PF MSTA, CNR, Comitato Chim, PROMEA, MIYASHITA Fdn Mat Sci, ZOZ, FRITSCH ECOSCI, SIEMENS, RIVOIRA, FBL, SAGES GETTERS, PERKIN ELMER, SEIFERT SIARS, FLUMAC, OXFORD INSTRUMENTS CRIOGENIA, KETTLEY, BNL Bank

AB Mechanical alloying of elemental powders was used to prepare Zr- and Mg-based multicomponent metallic glasses with extended supercooled liquid region before crystallization. The compositional dependence of glass formation and the thermal stability of the powders are compared with data for melt quenched samples and discussed with respect to the influence of impurities introduced during milling. Results on consolidated samples produced by hot compaction in the supercooled liquid regime are presented and compared with data for cast bulk specimens.

RI Schultz, Ludwig/B-3383-2010

SN 0255-5476

BN 0-87849-750-1

PY 1997

VL 235-2

BP 23

EP 28

DI 10.4028/www.scientific.net/MSF.235-238.23

PN 1 & 2

UT WOS:A1997BH18D00003

ER

PT J

AU Zhang, K

Hu, Z

Li, FJ

Wei, BC

AF Zhang, Kim

Hu, Zheng

Li, Fengjiang

Wei, Bingchen

TI Viscous surface flow induced on Ti-based bulk metallic glass by heavy

ion irradiation

SO APPLIED SURFACE SCIENCE

AB Ti-based bulk metallic glass was irradiated by a 20 MeV Cl4+ ion beam under liquid-nitrogen cooling, which produced remarkable surface smoothing and roughening that respectively correspond to normal and off-normal incidence angles of irradiation. Atomic force microscopy confirms two types of periodic ripples distributed evenly over the rough glass surface. In terms of mechanism, irradiation-induced viscosity agrees with the theoretical prediction for metallic glasses near glass transition temperature. Here, a model is introduced, based on relaxation of confined viscous flow with a thin liquid-like layer, that explains both surface smoothing and ripple formation. This study demonstrates that bulk metallic glass has high morphological instability and low viscosity under ion irradiation, which assets can pave new paths for metallic glass applications. (C) 2016 Published by Elsevier B.V.

RI Hire, RSwamy/L-8279-2017

SN 0169-4332

EI 1873-5584

PD DEC 30

PY 2016

VL 390

BP 941

EP 945

DI 10.1016/j.apsusc.2016.08.083

UT WOS:000385900700115

ER

PT B

AU Kong, RH

Guo, MLT

Chang, KF

Tsao, CYA

AF Kong, R. H.

Guo, M. L. Ted

Chang, K. F.

Tsao, Chi Y. A.

BE Gomes, JFS

Meguid, SA

TI Mg-Cu-Y BULK METALLIC GLASS SYNTHESIZED VIA SPRAY FORMING

SO PROCEEDINGS OF THE 7TH INTERNATIONAL CONFERENCE ON MECHANICS AND

MATERIALS IN DESIGN (M2D2017)

CT 7th International Conference on Mechanics and Materials in Design (M2D)

CY JUN 11-15, 2017

CL Albufeira, PORTUGAL

SP Univ Porto, Fac Engn, Univ Toronto, Mech & Aerosp Design Lab, Univ Algarve, Inst Super Engn, Portuguese Assoc Expt Mech, European Soc Expt Mech, Amer Soc Expt Mech, Japanese Soc Mech Engn, Int Measurement Confederat, Assoc Francaise Mecanique, European Assoc Dynam Mat, Inst Ciencia & Inovacao Enga Mecanica & Enga Ind, Lab Biomecanica Porto, Fundacao Ciencia Technologia, ABREU, Profess Congress Organizer

AB Mg-based bulk metallic glasses (BMG) exhibit maximum specific strength among bulk metallic glass systems. In this study, Mg65Cu25Y10 bulk metallic glass (BMG) was synthesized successfully via spray forming with rapid solidification rate and much higher cooling rate than conventional routes. The microstructure and constituent composition of the Mg65Cu25Y10 BMG were measured by DSC, XRD, and SEM equipped with EDS. All the four characteristic temperatures, Tg, Tx, Tm and Tl, of the BMG were obtained from continuous-heating DSC. The glass forming ability of the Mg65Cu25Y10 BMG was determined. The incubation time of the crystallization of as-injection cast BMG was obtained by isothermal DSC, and the appropriate temperature range in supercooled region for plastic deformation was determined.

BN 978-989-98832-7-7

PY 2017

BP 543

EP 544

UT WOS:000427886600091

ER

PT J

AU Man, QK

Inoue, A

Dong, YQ

Qiang, J

Zhao, CL

Shen, BL

AF Man, Qikui

Inoue, Akihisa

Dong, Yaqiang

Qiang, Jian

Zhao, Chengliang

Shen, Baolong

TI A new CoFe-based bulk metallic glasses with high thermoplastic forming

ability

SO SCRIPTA MATERIALIA

AB The (Co0.5Fe0.5)(62)Nb6Tb6M2B30 (M = Er, Tb, Y or Dy) bulk metallic glasses with a large supercooled liquid region up to 130 K are reported. Their high processing ability was demonstrated by simple microreplication experiments. In addition, this bulk metallic glass system exhibits a high fracture strength of up to 4750 MPa and a Vickers hardness of up to 1258, together with good soft-magnetic properties. Combining good mechanical properties with high thermoplastic forming ability, these bulk metallic glasses have potential for use in engineering applications. (C) 2013 Acta Materialia Inc. Published by Elsevier Ltd. All rights reserved.

RI Inoue, Akihisa/E-5271-2015; Dong, Yaqiang/C-5370-2017

OI Dong, Yaqiang/0000-0003-1663-8052; Qiang, Jian/0000-0002-2983-2754

SN 1359-6462

PD OCT

PY 2013

VL 69

IS 7

BP 553

EP 556

DI 10.1016/j.scriptamat.2013.07.002

UT WOS:000323590700015

ER

PT J

AU Nowak, S

Ochin, P

Pasko, A

Guerin, S

Champion, Y

AF Nowak, S.

Ochin, P.

Pasko, A.

Guerin, S.

Champion, Y.

TI MECHANICAL BEHAVIOR OF Zr-BASED BULK METALLIC GLASSES

SO STRENGTH OF MATERIALS

CT 5th International Conference on Materials Structure and Micromechanics

of Fracture

CY JUN 27-29, 2007

CL Brno, CZECH REPUBLIC

AB Bulk metallic glasses have a very high corrosion resistance and mechanical strength. Bulk metallic glasses show elastic-perfectly plastic behavior with an extended region of elastic strain (approximate to 2%). But at room temperature their macroscopic plasticity is weak even though a local plastic strain is observed in shear bands. A relaxation analysis allowed studying micro-mechanisms of plastic deformation and estimating the apparent activation volume (approximate to 2000 angstrom(3)).

RI Pasko, Alexandre/C-9529-2011

OI Pasko, Alexandre/0000-0002-8047-1122

SN 0039-2316

PD JAN

PY 2008

VL 40

IS 1

BP 154

EP 157

DI 10.1007/s11223-008-0040-x

UT WOS:000262960300040

ER

PT J

AU Liu, CT

Chen, GL

Inoue, A

Nieh, TG

Wu, SK

Ritchie, R

AF Liu, CT

Chen, GL

Inoue, A

Nieh, TG

Wu, SK

Ritchie, R

TI The Fifth IUMRS International Conference on Advanced Materials -

Symposium D: Intermetallic Compounds and Bulk Metallic Glasses -

Beijing, People's Republic of China, 13-18 June 1999 - Preface

SO INTERMETALLICS

RI Nieh, Tai-Gang/G-5912-2011

OI Nieh, Tai-Gang/0000-0002-2814-3746

SN 0966-9795

PD MAY-JUN

PY 2000

VL 8

IS 5-6

BP 453

EP 453

DI 10.1016/S0966-9795(00)00027-3

UT WOS:000087393800001

ER

PT J

AU Pineda, E

Hidalgo, I

Bruna, P

Pradell, T

Labrador, A

Crespo, D

AF Pineda, E.

Hidalgo, I.

Bruna, P.

Pradell, T.

Labrador, A.

Crespo, D.

TI Structural study of conventional and bulk metallic glasses during

annealing

SO JOURNAL OF ALLOYS AND COMPOUNDS

CT 14th International Sympoium on Metastable and Nano-Materials

CY AUG 26-30, 2007

CL Corfu, GREECE

AB Metallic glasses with conventional glass-forming ability (Al-Fe-Nd, Fe-Zr-B, Fe-B-Nb compositions) and bulk metallic glasses (Ca-Mg-Cu compositions) were studied by synchrotron X-ray diffraction during annealing throughout glass transition and crystallization temperatures. The analysis of the first diffraction peak position during the annealing process allowed us to follow the free volume change during relaxation and glass transition. The structure factor and the radial distribution function of the glasses were obtained from the X-ray measurements. The structural changes occurred during annealing are analyzed and discussed. (C) 2008 Elsevier B.V. All rights reserved.

RI Crespo, Daniel/M-1535-2013; Bruna, Pere/B-4967-2013; Pineda,

Eloi/H-9081-2013; Pradell, Trinitat/D-4765-2014; Bruna, Pere/I-2999-2014

OI Crespo, Daniel/0000-0003-1743-2400; Bruna, Pere/0000-0002-7411-1278;

Pineda, Eloi/0000-0002-1871-3848; Pradell, Trinitat/0000-0002-8720-5492;

Bruna, Pere/0000-0002-7411-1278

SN 0925-8388

PD AUG 26

PY 2009

VL 483

IS 1-2

BP 578

EP 581

DI 10.1016/j.jallcom.2008.07.194

UT WOS:000270619600139

ER

PT J

AU Egami, T

AF Egami, T

TI Atomistic mechanism of bulk metallic glass formation

SO JOURNAL OF NON-CRYSTALLINE SOLIDS

CT Annual Meeting of The-Minerals-Metals-and-Materials-Society

CY FEB 17-21, 2002

CL SEATTLE, WASHINGTON

SP Minerals Met & Mat Soc, Extract & Proc Div

AB The question of alloy selection for bulk metallic glasses is addressed from the atomistic point of view, in relation to the fragility of liquid. By extending the topological theory of glass formation to multi-component systems, it is suggested that increasing the number of elements involved, increasing the atomic size ratio, increasing the attractive force between small and large atoms, and introducing a repulsive potential between small atoms will help the formation of bulk metallic glasses. (C) 2003 Elsevier Science B.V. All rights reserved.

SN 0022-3093

PD MAR

PY 2003

VL 317

IS 1-2

BP 30

EP 33

DI 10.1016/S0022-3093(02)02003-3

UT WOS:000181251300006

ER

PT J

AU Yu, HB

Wang, WH

Bai, HY

AF Yu, H. B.

Wang, W. H.

Bai, H. Y.

TI An electronic structure perspective on glass-forming ability in metallic

glasses

SO APPLIED PHYSICS LETTERS

AB Through systematic study of glass-forming ability (GFA) and the electronic specific heat coefficient in typical ternary (Cu(50)Zr(50))(100-x)Al(x) bulk metallic glasses, we provide compelling experimental evidence that the density of electronic energy states at the Fermi level indeed is closely correlated with the GFA of metallic glasses, and the best GFA can be obtained when the Fermi surfaces nearly touch the quasi-Brillouin boundaries, as predicted by the nearly free electron model. Our results highlight the significance of electronic structural effects on the formation of metallic glasses.

RI Yu, Hai Bin/E-5312-2010

OI Yu, Hai Bin/0000-0003-0645-0187

SN 0003-6951

PD FEB 22

PY 2010

VL 96

IS 8

AR 081902

DI 10.1063/1.3327337

UT WOS:000275027200017

ER

PT J

AU Nakayama, KS

Yokoyama, Y

Ono, T

Chen, MW

Akiyama, K

Sakurai, T

Inoue, A

AF Nakayama, Koji S.

Yokoyama, Yoshihiko

Ono, Takahito

Chen, Ming Wei

Akiyama, Kotone

Sakurai, Toshio

Inoue, Akihisa

TI Controlled Formation and Mechanical Characterization of Metallic Glassy

Nanowires

SO ADVANCED MATERIALS

AB Extraordinary flexible very long and individual amorphous nanowires composed of metallic glasses are produced by the drawing process based on super-plastic deformation above the glass transition temperature. The outstanding mechanical properties of metallic glasses including low Young's modulus and high strength can be inherited in nanowire.

RI Yokoyama, Yoshihiko/A-8603-2011; Inoue, Akihisa/E-5271-2015; Nakayama,

Koji/E-5053-2012; CHEN, Mingwei/A-4855-2010

OI CHEN, Mingwei/0000-0002-8274-3099; Chen, Mingwei/0000-0002-2850-8872

SN 0935-9648

PD FEB 23

PY 2010

VL 22

IS 8

BP 872

EP +

DI 10.1002/adma.200902295

UT WOS:000275253400002

PM 20217808

ER

PT J

AU Li, JQ

Wang, L

Zhang, HF

Hu, ZQ

Cai, HN

AF Li, Jieqiong

Wang, Lu

Zhang, Haifeng

Hu, Zhuangqi

Cai, Hongnian

TI Synthesis and characterization of particulate reinforced Mg-based bulk

metallic glass composites

SO MATERIALS LETTERS

AB Bulk Mg65Cu20Ag5Gd 10 metallic glass matrix composites reinforced by SiC particulates were synthesized and characterized. Thermal stability and glass forming ability of the matrix are not significantly affected by the particle addition. Compressive fracture strength of the SiC particulates reinforced composite reached about 963 MPa, a factor of 1.2 higher than monolithic glass. A small quantity of Si atoms diffusion can be detected in the composite, but no reaction layer can be observed. (c) 2006 Elsevier B.V. All rights reserved.

SN 0167-577X

PD MAY

PY 2007

VL 61

IS 11-12

BP 2217

EP 2221

DI 10.1016/j.matlet.2006.08.077

UT WOS:000246158300023

ER

PT J

AU Zhao, K

Li, JF

Zhao, DQ

Pan, MX

Wang, WH

AF Zhao, K.

Li, J. F.

Zhao, D. Q.

Pan, M. X.

Wang, W. H.

TI Degradable Sr-based bulk metallic glasses

SO SCRIPTA MATERIALIA

AB We report a family of Sr-based bulk metallic glasses (BMGs) with good glass-forming ability and many unique properties, such as ultralow glass transition temperature T-g, elastic moduli and less fragility than known BMGs. The T-g of Sr60Li11Mg9Zn20 BMG is at room temperature (similar to 299 K). Remarkably, the BMGs have tunable degradation behavior in water and the corrosion rate can be controlled by simply minor alloying, so that the full degradation time can be effectively modulated from minutes to more than weeks. (C) 2009 Acta Materialia Inc. Published by Elsevier Ltd. All rights reserved.

RI BAI, JIE/D-7448-2016

SN 1359-6462

PD DEC

PY 2009

VL 61

IS 11

BP 1091

EP 1094

DI 10.1016/j.scriptamat.2009.08.042

UT WOS:000271179800024

ER

PT S

AU Ragani, J

Wang, Q

Gravier, S

Blandin, JJ

AF Ragani, J.

Wang, Q.

Gravier, S.

Blandin, J. J.

BE Sanders, DG

TI High Temperature Forming Maps of Various Bulk Metallic Glasses

SO SUPERPLASTICITY IN ADVANCED MATERIALS

SE Key Engineering Materials

CT 10th International Conference on Superplasticity in Advanced Materials

CY JUN 29-JUL 02, 2009

CL Seattle, WA

SP Univ Washington, Dept Mech Engn & Educ Outreach Program, ICSAM 2009 Comm

AB Due to their brittleness, bulk metallic glasses (BMG) are generally difficult to form at room temperature. Casting of BMG is one way to get components but an alternative route is to use the capacity to reach particularly large strains when the glasses are deformed in their supercooled liquid region (SLR). The experimental window (temperature, time) in which high temperature forming can be carried out is directly related to the glass resistance to crystallization. Such forming windows have been identified for various bulk metallic glasses (mainly zirconium and magnesium based BMG) thanks to compression tests in the supercooled liquid region. The effects of partial crystallization on the high temperature rheologies are also discussed. Finally, forming experiments were carried out in the selected windows.

SN 1013-9826

PY 2010

VL 433

BP 345

EP 351

DI 10.4028/www.scientific.net/KEM.433.345

UT WOS:000279945300042

ER

PT J

AU Li, N

Xu, EJ

Liu, Z

Wang, XY

Liu, L

AF Li, Ning

Xu, Erjiang

Liu, Ze

Wang, Xinyun

Liu, Lin

TI Tuning apparent friction coefficient by controlled patterning bulk

metallic glasses surfaces

SO SCIENTIFIC REPORTS

AB Micro-honeycomb structures with various pitches between adjacent cells were hot-embossed on Zr35Ti30Cu8.25Be26.75 bulk metallic glass surface. The effect of pitch geometry on the frictional behavior of metallic glass surface was systematically investigated. The results revealed that all textured metallic glass surfaces show a reduction in friction coefficient compared to smooth surface. More intriguingly, the friction coefficient first decreased and then increased gradually with increasing pitches. Such unique behavior can be understood fundamentally from the perspective of competing effects between contact area and local stress level with increasing pitches. This finding not only enhance the in-depth understanding of the mechanism of the significant role of surface topography on the frictional behavior of metallic glass surface, but also opens a new route towards other functional applications for bulk metallic glasses.

SN 2045-2322

PD DEC 19

PY 2016

VL 6

AR 39388

DI 10.1038/srep39388

UT WOS:000389889800001

PM 27991571

ER

PT J

AU Wang, GY

Liaw, PK

Yokoyama, Y

Freels, M

Inoue, A

AF Wang, Gongyao

Liaw, Peter K.

Yokoyama, Yoshihiko

Freels, Matthew

Inoue, Akihisa

TI Investigations of the Factors that Affected Fatigue Behavior of Zr-Based

Bulk-Metallic Glasses

SO ADVANCED ENGINEERING MATERIALS

RI Wang, Gongyao/C-4003-2011; Inoue, Akihisa/E-5271-2015; Yokoyama,

Yoshihiko/A-8603-2011

SN 1438-1656

PD NOV

PY 2008

VL 10

IS 11

BP 1030

EP 1033

DI 10.1002/adem.200800133

UT WOS:000261933400008

ER

PT J

AU Wang, JG

Zhao, DQ

Pan, MX

Wang, WH

AF Wang, Jian-Guo

Zhao, De-Qian

Pan, Ming-Xiang

Wang, Wei-Hua

TI Iron based alloy with hierarchical structure and superior mechanical

performance

SO ADVANCED ENGINEERING MATERIALS

SN 1438-1656

PD FEB

PY 2008

VL 10

IS 1-2

BP 46

EP 50

DI 10.1002/adem.200700253

UT WOS:000253741600005

ER

PT J

AU Huo, JT

Zhao, DQ

Bai, HY

Axinte, E

Wang, WH

AF Huo, J. T.

Zhao, D. Q.

Bai, H. Y.

Axinte, E.

Wang, W. H.

TI Giant magnetocaloric effect in Tm-based bulk metallic glasses

SO JOURNAL OF NON-CRYSTALLINE SOLIDS

AB We report that the maximum magnetic entropy change Delta S-m, of Tm39Ho16Co20Al25 metallic glass can reach 18.3 Jkg(-1) K-1 under 5 T, which is larger than that of any previously reported metallic glasses and comparable with that of the giant magnetocaloric effect material Gd5Si2Ge2 compound. Even under low magnetic field of 2 T, the Delta S-m of the glass can reach 10.3 Jkg(-1) K-1 which is even larger that of most of other metallic glasses under 5 T. The excellent magnetocaloric effect and refrigerant efficiency together with unique mechanical and physical properties of the metallic glasses indicate they are promising candidate for magnetic refrigerants. (C) 2012 Elsevier B.V. All rights reserved.

RI Axinte, Eugen/C-6963-2011

OI Axinte, Eugen/0000-0002-9243-1160

SN 0022-3093

PD JAN 1

PY 2013

VL 359

BP 1

EP 4

DI 10.1016/j.jnoncrysol.2012.09.020

UT WOS:000312914600001

ER

PT J

AU Huo, JT

Huo, LS

Men, H

Wang, XM

Inoue, A

Wang, JQ

Chang, CT

Li, RW

AF Huo, Juntao

Huo, Lishan

Men, He

Wang, Xinmin

Inoue, Akihisa

Wang, Junqiang

Chang, Chuntao

Li, Run-Wei

TI The magnetocaloric effect of Gd-Tb-Dy-Al-M (M = Fe, Co and Ni)

high-entropy bulk metallic glasses

SO INTERMETALLICS

AB In this article, we report the formation of the high-entropy Gd20Tb20Dy20Al20M20 (M = Fe, Co and Ni) bulk metallic glasses with good magnetocaloric properties. Compared with most of the rare earth based metallic glasses, these alloys are found to have the comparably large maximum magnetic entropy changes (Delta S-M), but much broader widths of the Delta S-M peaks, and hence larger refrigerant capacity (RC). This can be attributed to the combination of the spin glass behaviors and the complicated compositions in these alloys. Our work show that the high entropy bulk metallic glasses is a promising candidate material as the magnetic refrigerant. (C) 2014 Elsevier Ltd. All rights reserved.

RI Xia, YuQing/C-9724-2017; Wang, Junqiang/C-2839-2015; Inoue,

Akihisa/E-5271-2015

OI Wang, Junqiang/0000-0002-8066-6237; Huo, Juntao/0000-0002-2107-4979

SN 0966-9795

EI 1879-0216

PD MAR

PY 2015

VL 58

BP 31

EP 35

DI 10.1016/j.intermet.2014.11.004

UT WOS:000348626600005

ER

PT J

AU Han, G

Qiang, JB

Wang, Q

Wang, YM

Xia, JH

Zhu, CL

Quan, SG

Dong, C

AF Han Guang

Qiang Jian-Bing

Wang Qing

Wang Ying-Min

Xia Jun-Hai

Zhu Chun-Lei

Quan Shi-Guang

Dong Chuang

TI Electrochemical potential equilibrium of electrons in ideal metallic

glasses based on the cluster-resonance model

SO ACTA PHYSICA SINICA

AB Ideal metallic glasses are the metallic glasses that satisfy electronic structure stability. Previously we have developed a so-called 'cluster-plus-glue-atom model' and more recently a 'cluster-resonance model' for the ideal metallic glasses. Good metallic glass forming compositions always satisfy simple cluster formulas [cluster] (glue atoms), with x denoting the number of glue atoms matching one cluster. In this paper we present an electrochemical potential equilibrium criterion based on these models to obtain the number of glue atoms. By examples of Cu-Zr and Co-B bulk metallic glasses, it is confirmed that the experimentally determined good BMG-forming compositions well agree with the calculated composition formulas.

SN 1000-3290

PD FEB

PY 2012

VL 61

IS 3

AR 036402

UT WOS:000301008900052

ER

PT J

AU Ma, J

Yang, C

Gong, F

Wu, XY

Liang, X

AF Ma Jiang

Yang Can

Gong Feng

Wu Xiao-Yu

Liang Xiong

TI Thermoplastic forming of bulk metallic glasses

SO ACTA PHYSICA SINICA

AB The viscosities of metallic glasses gradually drop with temperature rising in their supercooled liquid region (SLR) which enables them to be thermoplastically formed and totally overturns the processing method of traditional metallic materials: their forming can be realized under temperature and stress far below those of traditional metallic materials. Based on this property, metallic glasses are considered as the ideal miniature fabrication materials due to their unique amorphous structures and no crystalline defects such as dislocation and grain boundary.

The thermoplastic micro forming of metallic glasses in their SLR is studied in the present paper. A universal equation which describes the filling kinetics of viscous metallic glasses in the non-circular channel is proposed with the help of fluidic mechanics, and the results may be theoretically useful for the micro application of metallic glasses.

In addition, some applications in the micro thermoplastic forming of metallic glasses are introduced. A metallic glass mold insert for hot embossing of polymers is fabricated by the micro thermoplastic forming of metallic glass, and it is found to have many advantages in mechanical property, fabrication efficiency, surface quality, etc. compared with the traditional material and method. A similar approach is used to fabricate gratings, which may provide a new material and technology to produce gratings. The superhydrophobic metallic glass surface with excellent abrasion and corrosion resistance is also fabricated by constructing micro-nano hierarchical structures on metallic glass surface. The bulk metallic glass micro fuel cell is also finished and found to have good performance.

SN 1000-3290

PD SEP 5

PY 2017

VL 66

IS 17

AR 176404

DI 10.7498/aps.66.176404

UT WOS:000412842000017

ER

PT J

AU Xu, YK

Xu, J

AF Xu, YK

Xu, J

TI Ceramics particulate reinforced Mg65Cu20Zn5Y10 bulk metallic glass

composites

SO SCRIPTA MATERIALIA

AB The Mg65Cu20Zn5Y10 bulk metallic glass was reinforced with 10 vol.% SiC or 15 vol.% TiB2 particles. The introduced particles have no significant adverse effect on the glass forming ability of the matrix glass. The compressive strength of the composites reached about 1 GPa. A plastic strain to failure of 0.9% was obtained for the TiB2 reinforced composite. (C) 2003 Acta Materialia Inc. Published by Elsevier Ltd. All rights reserved.

SN 1359-6462

PD NOV

PY 2003

VL 49

IS 9

BP 843

EP 848

DI 10.1016/S1359-6462(03)00447-0

UT WOS:000185038400006

ER

PT J

AU Louzguine-Luzgin, DV

Ketov, SV

Trifonov, AS

Churymov, AY

AF Louzguine-Luzgin, D. V.

Ketov, S. V.

Trifonov, A. S.

Churymov, A. Yu.

TI Surface structure and properties of metallic glasses

SO JOURNAL OF ALLOYS AND COMPOUNDS

AB Surface state and its quality determine some important properties of metallic glasses. Control over of the nanoscale tribological behavior of metallic glasses is fundamental for their applications in micro-and nano-electromechanical devices. Owing to continuous miniaturization of these devices the mechanical contact area becomes of nanoscale, whereas the nanoscale wear resistance of metallic glasses can be improved taking into the account their surface oxides. Surface oxides also determine (bio) chemical properties of metallic glasses and alter their electrical properties. In the present paper we overview recent works on the subject and present the original research results related to the nanoscale tribological properties of metallic glasses. (C) 2018 Elsevier B.V. All rights reserved.

SN 0925-8388

EI 1873-4669

PD APR 25

PY 2018

VL 742

BP 512

EP 517

DI 10.1016/j.jallcom.2018.01.290

UT WOS:000427505800062

ER

PT J

AU Eckert, J

Seidel, M

Kubler, A

Klement, U

Schultz, L

AF Eckert, J

Seidel, M

Kubler, A

Klement, U

Schultz, L

TI Oxide dispersion strengthened mechanically alloyed amorphous Zr-Al-Cu-Ni

composites

SO SCRIPTA MATERIALIA

RI Schultz, Ludwig/B-3383-2010

SN 1359-6462

PD JAN 13

PY 1998

VL 38

IS 4

BP 595

EP 602

DI 10.1016/S1359-6462(97)00517-4

UT WOS:000071740700010

ER

PT J

AU Haruyama, O

Sakagami, H

Nishiyama, N

Inoue, A

AF Haruyama, O.

Sakagami, H.

Nishiyama, N.

Inoue, A.

TI The free volume kinetics during structural relaxation in bulk Pd-P based

metallic glasses

SO MATERIALS SCIENCE AND ENGINEERING A-STRUCTURAL MATERIALS PROPERTIES

MICROSTRUCTURE AND PROCESSING

CT 12th International Conference on Rapidly Quenched and Metastable

Materials

CY AUG 21-26, 2005

CL Jeju Isl, SOUTH KOREA

AB Isothermal enthalpy relaxation and density relaxation were investigated for bulk Pd42.5Cu30N7.5P20 and Pd40Ni40P20 metallic glasses. The density was measured at room temperature after relaxing the sample for a given time. Enthalpy relaxation data of Pd42Cu30Ni7.5P20 glass and density relaxation data of Pd40Ni40P20 glass were well fitted by a non-Debye type relaxation function. A fit to a stretched exponential relaxation function of density relaxation data at 549 K of Pd40Ni40P20 glass gave the exponential index of 0.66. Isochoric Kauzmann temperature T-K was estimated from density measurements for nearly fully relaxed bulk glasses and T-K = 456 +/- 110 K was obtained. (c) 2006 Elsevier B.V. All rights reserved.

RI Nishiyama, Nobuyuki/C-8228-2015; Inoue, Akihisa/E-5271-2015

SN 0921-5093

PD MAR 25

PY 2007

VL 449

BP 497

EP 500

DI 10.1016/j.msea.2006.02.325

UT WOS:000245477800113

ER

PT J

AU Tamura, T

Kamikihara, D

Miwa, K

AF Tamura, T.

Kamikihara, D.

Miwa, K.

TI Production of Fe based bulk metallic glasses using electromagnetic

vibrations

SO INTERNATIONAL JOURNAL OF CAST METALS RESEARCH

CT 10th Asian Foundry Congress 2008

CY MAY 21-24, 2008

CL Nagoya, PEOPLES R CHINA

AB It is known that cooling rate from the liquid state is an important factor for producing the bulk metallic glasses. However, little work has been conducted on the influence of other factors such as electric and/or magnetic fields. The authors have previously reported that the glass forming ability of Mg-Cu-Y and Fe-Co-B-Si-Nb alloys is enhanced with increasing electromagnetic vibration force. The present study aims to investigate effect of the electromagnetic vibrations on the crystal particles in Fe-Co-B-Si-Nb bulk metallic glasses. As a result, the electromagnetic vibrations were found to act mainly on decreasing the number of crystal nuclei.

RI Tamura, Takuya/D-4854-2017

OI Tamura, Takuya/0000-0001-6083-913X

SN 1364-0461

PD AUG

PY 2008

VL 21

IS 1-4

BP 86

EP 89

DI 10.1179/136404608X361729

UT WOS:000261010000018

ER

PT J

AU Hu, Y

Pan, MX

Liu, L

Zhao, YH

Zhao, DQ

Wang, WH

AF Hu, Y

Pan, MX

Liu, L

Zhao, YH

Zhao, DQ

Wang, WH

TI Synthesis of Fe-based bulk metallic glasses with low purity materials by

multi-metalloids addition

SO MATERIALS LETTERS

AB Bulk metallic glasses (BMGs) Fe61Co7Zr10Mo5W2 M(15+X) (X=0, 0.15, 0.30, 0.45, 0.60, 0.75 and 1.2) (M contains B, Al, Si, C and P) were prepared with low purity of raw materials by copper mould cast. X-ray diffraction and differential thermal analyzer results show that the glass forming ability (GFA) as well as the thermal stability of the Fe-based alloy made from low purity raw materials can be much improved by adding small amount of multi-metalloids. The positive effect of metalloids addition on the formation of the bulk metallic glasses is discussed. (C) 2002 Elsevier Science B.V All rights reserved.

SN 0167-577X

PD MAY

PY 2003

VL 57

IS 18

BP 2698

EP 2701

DI 10.1016/S0167-577X(02)01360-5

UT WOS:000182776500017

ER

PT J

AU Wang, Q

Pelletier, JM

Blandin, JJ

AF Wang, Q.

Pelletier, J. M.

Blandin, J. J.

TI Thermal stability of cerium-based bulk metallic glasses. Influence of

iron addition

SO JOURNAL OF ALLOYS AND COMPOUNDS

AB Cerium-based bulk metallic glasses are attractive due to their low glass transition temperature, comparable to that of polymers. Their mechanical properties, especially their elastic and viscoelastic ones are reported in a large temperature range. Like in other bulk metallic glasses, the shear modulus, close to 11.5 GPa at room temperature, decreases drastically in the glass temperature range (at about 373 K), while the viscoelastic component becomes very important. Heating to high temperature induces crystallization. As shown by X-ray diffraction experiments performed in situ, the increase of elastic modulus due to the formation of crystalline particles is strongly connected to the volume fraction of these particles. Addition of iron shifts the phenomena towards higher temperatures and affects the crystallization process. (C) 2010 Elsevier B.V. All rights reserved.

SN 0925-8388

PD AUG 20

PY 2010

VL 504

IS 2

BP 357

EP 361

DI 10.1016/j.jallcom.2010.05.070

UT WOS:000281019900020

ER

PT J

AU Demetriou, MD

Floyd, M

Crewdson, C

Schramm, JP

Garrett, G

Johnson, WL

AF Demetriou, Marios D.

Floyd, Michael

Crewdson, Chase

Schramm, Joseph P.

Garrett, Glenn

Johnson, William L.

TI Liquid-like platinum-rich glasses

SO SCRIPTA MATERIALIA

AB Bulk platinum-rich glasses exhibiting high bulk moduli and Poisson ratios are introduced. A bulk modulus of 217 GPa and a Poisson's ratio of 0.43 are measured, the highest values reported to date for a metallic glass. The present glasses demonstrate an unusual capacity for "liquid-like" deformation characterized by low resistance to shear flow and high resistance to cavitation, enabling extensive bending ductility in the absence of fracture. An indirect estimate of the fracture toughness yields a value of similar to 125 MPa m(1/2). (C) 2011 Acta Materialia Inc. Published by Elsevier Ltd. All rights reserved.

SN 1359-6462

PD NOV

PY 2011

VL 65

IS 9

BP 799

EP 802

DI 10.1016/j.scriptamat.2011.07.035

UT WOS:000295765300014

ER

PT J

AU Gubicza, J

Labar, JL

Agocs, E

Fatay, D

Lendvai, J

AF Gubicza, J.

Labar, J. L.

Agocs, E.

Fatay, D.

Lendvai, J.

TI Effect of nano-quasicrystals on viscosity of a Zr-based bulk metallic

glass (vol 58, pg 291, 2008)

SO SCRIPTA MATERIALIA

RI Labar, Janos Laszlo/B-8148-2008; Lendvai, Janos/J-4445-2013

OI Labar, Janos Laszlo/0000-0002-3944-8350; Gubicza,

Jeno/0000-0002-8938-7293

SN 1359-6462

PD JUN

PY 2008

VL 58

IS 11

BP 1038

EP 1038

DI 10.1016/j.scriptamat.2008.01.040

UT WOS:000255601600028

ER

PT J

AU MAO, ZL

CHEN, H

WANG, WK

AF MAO, ZL

CHEN, H

WANG, WK

TI FORMATION OF BULK METALLIC-GLASS ZR60NI20AL20 BY HIGH-PRESSURE QUENCHING

SO JOURNAL OF MATERIALS SCIENCE LETTERS

SN 0261-8028

PD NOV 1

PY 1993

VL 12

IS 21

BP 1729

EP 1730

DI 10.1007/BF00418846

UT WOS:A1993MG13300027

ER

PT J

AU Li, ZJ

Zhao, LR

Wang, YT

Chen, ZM

Tu, WK

Zhang, YQ

Bo, H

Liu, YD

Wang, LM

AF Li, Zi-Jing

Zhao, Lin-Ran

Wang, Yu-Ting

Chen, Ze-Ming

Tu, Wen-Kang

Zhang, Ya-Qi

Bo, Hong

Liu, Ying-Dan

Wang, Li-Min

TI An Attempt to Prepare Metallic Glasses from Quasicrystals

SO CHINESE PHYSICS LETTERS

AB Icosahedrons in supercooled liquids and glasses are considered to be of significance for the glass formation in alloy systems. Starting from the similarity of the local structure of quasicrystals to the icosahedrons in metallic glasses, a scheme is put forward to prepare metallic glasses based on a well-known quasicrystal Zr40Ti40Ni20. A series of (Zr40Ti40Ni20)(100-x)Co-x. metallic glasses are fabricated, and the optimized glass forming composition is determined at (Zr40Ti40Ni20)(92)Co-8. The results show that the glass-forming ability of the alloys is closely related to the quasicrystalline phases. The mechanism of the enhanced glass-forming ability is discussed.

RI Liu, Ying Dan/C-5526-2011

OI Liu, Ying Dan/0000-0002-5826-0505

SN 0256-307X

EI 1741-3540

PD APR

PY 2016

VL 33

IS 4

AR 046401

DI 10.1088/0256-307X/33/4/046401

UT WOS:000374656400023

ER

PT J

AU Guo, SF

Chan, KC

Zhu, ZQ

Wu, ZR

Chen, W

Song, M

AF Guo, S. F.

Chan, K. C.

Zhu, Z. Q.

Wu, Z. R.

Chen, W.

Song, M.

TI Microstructure and tensile behavior of small scale resistance spot

welded sandwich bulk metallic glasses

SO JOURNAL OF NON-CRYSTALLINE SOLIDS

AB In this work, a small-scale resistance spot welding method was utilized to join two dissimilar Zr-based bulk metallic glasses and to fabricate the sandwich-laminated metallic glass plates. The laminates exhibit an almost fully amorphous structure without undesirable crystallization. Elemental line scanning across the joint interface shows a uniform distribution of the main elements, demonstrating favorable metallurgical bond in the laminate. The resultant tensile strength of the welded laminate is comparable to that of the parent metallic glasses. The fractured surface of the laminate exhibits extensive multiple failure planes, suggesting that the fracture instability was mediated by a crack branching mechanism over across the joint interface. Such a crack branching mechanism results in a stepwise fracture behavior which is contrastingly different from the conventional single primary shear band dominated catastrophic fracture in monolithic metallic glasses under tension. The unique stepwise fracture behavior endows the sandwiched metallic glass laminates with an excessive strain energy absorption through the joint interface than monolithic metallic glasses. Our results demonstrate that small-scale resistance spot welding is a promising approach to scaling up metallic glasses and to fabricating metallic glass laminates with desirable mechanical performance for structural applications. (C) 2016 Elsevier B.V. All rights reserved.

OI Chan, K.C./0000-0002-6173-5532

SN 0022-3093

EI 1873-4812

PD SEP 1

PY 2016

VL 447

BP 300

EP 306

DI 10.1016/j.jnoncrysol.2016.06.026

UT WOS:000381841200044

ER

PT J

AU Li, S

Xie, GQ

Louzguine-Luzgin, DV

Cao, ZP

Yoshikawa, N

Sato, M

Inoue, A

AF Li, Song

Xie, Guoqiang

Louzguine-Luzgin, Dmitri V.

Cao, Ziping

Yoshikawa, Noboru

Sato, Motoyasu

Inoue, Akihisa

TI Microwave Sintering of Ni-Based Bulk Metallic Glass Matrix Composite in

a Single-Mode Applicator

SO MATERIALS TRANSACTIONS

AB Microwave (MW) heating and sintering of Ni-based bulk metallic glass matrix composite, consisting of gas-atomized Ni52.5Zr15Nb10Ti15Pt7.5 glassy alloy and tin powders, was performed without external pressure by using a single-mode applicator. These powders could be heated well in the magnetic field maximum, but not heated enough in the electric field maximum. A bulk sintered body with the retention of the amorphous phase was obtained below 783 K in an inert atmosphere. The addition of Sn particles promoted the densification of the sintered Ni52.5Zr15Nb10Ti15Pt7.5 glassy specimen. The results suggest that the MW sintering of metallic glasses offers a flexibility of fabricating bulk metallic glasses and metallic glass matrix composites. [doi: 10.2320/matertrans.MRA2008603]

RI Inoue, Akihisa/E-5271-2015; LOUZGUINE, Dmitri/D-2492-2010; Xie,

Guoqiang/A-8619-2011

OI LOUZGUINE, Dmitri/0000-0001-5716-4987;

SN 1345-9678

EI 1347-5320

PD DEC

PY 2008

VL 49

IS 12

BP 2850

EP 2853

DI 10.2320/matertrans.MRA2008603

UT WOS:000262932900016

ER

PT J

AU Yang, BJ

Yao, JH

Chao, YS

Wang, JQ

Ma, E

AF Yang, B. J.

Yao, J. H.

Chao, Y. S.

Wang, J. Q.

Ma, E.

TI Developing aluminum-based bulk metallic glasses

SO PHILOSOPHICAL MAGAZINE

AB This paper details a systematic investigation of the formation of Al-based bulk metallic glasses, expanding on an earlier brief report [Scripta Mater. 61 (2009) p.423]. We discuss an approach for designing and predicting the best glass-forming composition in the Al-TM-RE systems, based on the atomic cluster packing model for the internal structure of the glass. The effects of additional elements in quaternary and quinary systems on the glass-forming ability and thermal stability of the glasses are also discussed. Three new compositions, Al86Ni6Y4.5Co2La1.5, Al86Ni7Y5Co1La1 and Al86Ni7Y4.5Co1La1.5, are capable of forming fully glassy rods of 1 mm in diameter; their glass transition and other thermal properties are systematically characterized.

RI Ma, En/A-3232-2010

SN 1478-6435

PY 2010

VL 90

IS 23

BP 3215

EP 3231

AR PII 922840004

DI 10.1080/14786435.2010.484401

UT WOS:000279222000005

ER

PT J

AU Gu, B

Liu, F

Jiang, YH

Zhang, K

AF Gu, Bin

Liu, Feng

Jiang, Yihui

Zhang, Ke

TI Evaluation of glass-forming ability criterion from phase-transformation

kinetics

SO JOURNAL OF NON-CRYSTALLINE SOLIDS

AB Applying kinetic analysis upon crystallization of metallic glass, a quantitative relation between the critical cooling rate and the onset temperature of crystallization was obtained for glass-forming alloys. Effects of the onset temperature of crystallization, the liquidus temperature and the glass transition temperature on the critical cooling rate were analytically described. Three rules guiding the development of more reliable glass-forming ability criteria are suggested. (C) 2012 Elsevier B.V. All rights reserved.

SN 0022-3093

PD AUG 1

PY 2012

VL 358

IS 15

BP 1764

EP 1771

DI 10.1016/j.jnoncrysol.2012.05.019

UT WOS:000307028900008

ER

PT J

AU Deng, ST

Diao, H

Chen, YL

Yan, C

Zhang, HF

Wang, AM

Hu, ZQ

AF Deng, S. T.

Diao, H.

Chen, Y. L.

Yan, C.

Zhang, H. F.

Wang, A. M.

Hu, Z. Q.

TI Metallic glass fiber-reinforced Zr-based bulk metallic glass

SO SCRIPTA MATERIALIA

AB A novel Zr-based bulk metallic glass composite was fabricated using stainless steel capillaries as the reinforcement Large plasticity (14%) was achieved in the composite with a reinforcement volume fraction of 38% The high plasticity observed can be attributed to the formation of small glass fibers encapsulated by the steel capillaries, which promotes multiple shear bands in both metallic glass matrix and the fibers themselves A new parameter was also proposed to approximately evaluate the reinforcement efficiency (C) 2010 Acta Materialia Inc Published by Elsevier Ltd All rights reserved

RI wang, am/P-2147-2016

SN 1359-6462

PD JAN

PY 2011

VL 64

IS 1

BP 85

EP 88

DI 10.1016/j.scriptamat.2010.09.014

UT WOS:000284453300022

ER

PT J

AU Wang, YY

Zhao, W

Li, G

Li, YC

Liu, RP

AF Wang, Y. Y.

Zhao, W.

Li, G.

Li, Y. C.

Liu, R. P.

TI Pressure-induced polyamorphic transitions in ytterbium-based bulk

metallic glasses

SO MATERIALS LETTERS

AB The structure of Yb60Ca2.5Zn20Mg17.5 bulk metallic glasses was investigated by in situ angle-dispersive X-ray diffraction with a synchrotron radiation source. The glassy nature of the samples was stable up to similar to 30 GPa at room temperature. The volume and bulk modulus as a function of hydrostatic pressure were extracted from the diffraction data. A sudden change was observed in the volume and bulk modulus during compression. Results indicated that pressure induced polyamorphic phase transition in Yb-based bulk metallic glasses compared with Pr- and Gd-based bulk metallic glasses. The critical pressure of the transition was about 10 GPa. The delocalization of 4f electrons under high pressures was responsible for the amorphous-to-amorphous phase transition in Yb-based bulk metallic glasses. (C) 2013 Elsevier B.V. All rights reserved.

SN 0167-577X

EI 1873-4979

PD NOV 1

PY 2013

VL 110

BP 184

EP 187

DI 10.1016/j.matlet.2013.07.091

UT WOS:000325908300050

ER

PT J

AU Nowak, S

Ochin, P

Champion, Y

AF Nowak, S.

Ochin, P.

Champion, Y.

TI Metallic Glass Zr57Cu20Al10Ni8Ti5-Tungsten Composites

SO MATERIALS AND MANUFACTURING PROCESSES

AB Composite is prepared by mixing Zr57Cu20Al10Ni8Ti5 and W powders and compaction in a WC system at a temperature between the glass transition temperature (T-g) and crystallization temperature (T-x), using the viscous flow behaviour of the metallic glass to form the matrix. Compaction and sintering (by hot pressing and Spark Plasma Sintering) of the metallic glasses (MG) powder is reported in detail, emphasizing detrimental evolution of the amorphous phase during the milling process for viscous flow. This drawback is overcome using an atomized powder.

SN 1042-6914

PY 2009

VL 24

IS 10-11

BP 1162

EP 1167

DI 10.1080/10426910902979603

UT WOS:000274699700021

ER

PT J

AU Wang, WH

Wang, RJ

Dai, DY

Zhao, DQ

Pan, MX

Yao, YS

AF Wang, WH

Wang, RJ

Dai, DY

Zhao, DQ

Pan, MX

Yao, YS

TI Response to "Comment on 'Pressure-induced amorphization of ZrTiCuNiBe

bulk glass-forming alloy'" [Appl. Phys. Lett. 80, 700 (2002)]

SO APPLIED PHYSICS LETTERS

SN 0003-6951

PD JAN 28

PY 2002

VL 80

IS 4

BP 701

EP 701

DI 10.1063/1.1445268

UT WOS:000173508900057

ER

PT J

AU Jing, Q

Liu, RP

Shao, GJ

Wang, WK

AF Jing, Q

Liu, RP

Shao, GJ

Wang, WK

TI Preparation and super-plastic deformation of the Zr-based bulk metallic

glass

SO MATERIALS SCIENCE AND ENGINEERING A-STRUCTURAL MATERIALS PROPERTIES

MICROSTRUCTURE AND PROCESSING

AB Zr41Ti14Cu12.5Ni10Be22.5 bulk metallic glass rods with an aspect ratio of 25:1 were prepared by suction casting and water quenching methods. A suitable temperature region for super-plastic deformation of the bulk metallic glass was determined by thermal expansion experiments, and the bulk metallic glass was found to exhibit excellent super-plastic deformation ability in this temperature region. (C) 2003 Elsevier B.V. All rights reserved.

SN 0921-5093

EI 1873-4936

PD OCT 25

PY 2003

VL 359

IS 1-2

BP 402

EP 404

DI 10.1016/S0921-5093(03)00370-8

UT WOS:000185613700049

ER

PT J

AU He, L

Sun, J

AF He, L

Sun, J

TI Effect of microalloying on glass-forming ability and crystallization

kinetics of Zr52.5Cu17.9Ni14.6Al10Ti5 alloy

SO SCRIPTA MATERIALIA

AB Zr52.5Cu17.9Ni14.6Al10Ti5 bulk metallic glass prepared using sponge zirconium was microalloyed with 0.1at.%B+0.2at.%-Si + 0.1 at.%Pb. The effect of microalloying on the glass-forming ability and thermal stability of the alloy is studied. The crystallization kinetic parameters are determined by means of a Kissinger plot. The interrelation between these parameters is discussed. (c) 2005 Acta Materialia Inc. Published by Elsevier Ltd. All rights reserved.

SN 1359-6462

PD MAR

PY 2006

VL 54

IS 6

BP 1081

EP 1085

DI 10.1016/j.scriptamat.2005.12.004

UT WOS:000234941500022

ER

PT J

AU Yang, GL

Lin, X

Hu, Q

Zhang, Y

Wang, ZT

Li, P

Huang, WD

AF Yang Gaolin

Lin Xin

Hu Qiao

Zhang Ying

Wang Zhitai

Li Peng

Huang Weidong

TI CRYSTALLIZATION BEHAVIOR OF ANNEALED Zr55Cu30Al10Ni5 BULK METALLIC GLASS

DURING PULSED LASER REMELTING

SO ACTA METALLURGICA SINICA

AB The crystallization behavior of annealed metallic glasses during pulsed laser remelting was investigated in this work. The as-casted Zr55Cu30Al10Ni5 bulk metallic glasses were annealed at 390, 430, 530, 792 and 902 degrees C separately. And then these annealed alloys were remelted by pulsed laser. The experiment results show that the alloys annealed at 390 and 430 degrees C were still metallic glasses, and their crystallization behavior during remelting is similar to the remelting of metallic glass without annealing treatment. The specimens annealed at 530, 792 and 902 degrees C were completely crystallized. After remelting, the molten pools of these specimens were amorphous. For the specimens annealed at 530 and 792 degrees C, there was no obvious epitaxial growth at the bottom of molten pools. For the specimens annealed at 902 degrees C, there was little primary phase epitaxial growth at bottom of molten pool after one time laser remelting while without epitaxial growth after 11 times laser remelting. The epitaxial growth was caused by the area reserved the composition distribution of CuZr2 primary phase during laser remelting. So it is hard to obtain epitaxial growth during laser remelting for Zr55Cu30Al10Ni5 bulk metallic glasses even it has a crystallization substrate because of the slow diffusion. So it is easy to keep molten pool as amorphous state during laser treating Zr55Cu30Al10Ni5 bulk metallic glasses.

RI Lin, Xin/L-9950-2013

OI Lin, Xin/0000-0002-7491-8928

SN 0412-1961

PD JUN

PY 2013

VL 49

IS 6

BP 649

EP 657

DI 10.3724/SP.J.1037.2012.00680

UT WOS:000320216500002

ER

PT J

AU Dong, DD

Zhang, S

Wang, ZJ

Dong, C

Haussler, P

AF Dong, Dandan

Zhang, Shuang

Wang, Zijian

Dong, Chuang

Haussler, Peter

TI Composition interpretation of binary bulk metallic glasses via principal

cluster definition

SO MATERIALS & DESIGN

AB It has been pointed out that a bulk metallic glass composition could be formulated as [cluster](glue atoms)(x), where the cluster is derived from a devitrification phase. However, the selection rule of the so-called principal cluster should be specified because an alloy phase usually contains multiple clusters. In this paper, two important properties of the principal clusters are emphasized, i.e., spherical periodicity and cluster isolation, both being structural features of metallic glasses. According to these two criteria, the principal clusters are rigorously identified in devitrification phases and are used to construct cluster formulas to explain binary bulk-metallic glasses of Cu-(Zr,Hf), Ni-(Nb,Ta), Al-Ca, and Pd-Si. (C) 2016 Elsevier Ltd. All rights reserved.

SN 0261-3069

EI 1873-4197

PD APR 15

PY 2016

VL 96

BP 115

EP 121

DI 10.1016/j.matdes.2016.02.020

UT WOS:000371296000015

ER

PT J

AU Biner, SB

AF Biner, SB

TI Ductility of bulk metallic glasses and their composites with ductile

reinforcements: A numerical study

SO ACTA MATERIALIA

AB Yield behavior of bulk metallic glasses containing voids is elucidated by using unit-cell analyses and the relevant material parameters; and the results are compared with the available constitutive models. These analyses clearly show that caution is needed when transferring the constitutive models associated with void growth and ductile damage in usual metallic materials to bulk metallic glasses, largely because of the different deformation modes in bulk metallic glasses resulting from the pressure dependency of the yield surface and intrinsic softening behavior. With the constitutive model calibrated from the unit-cell analysis, the influences of a wide range of parameters (mechanical properties, volume fraction and morphology of ductile reinforcements) on the ductility of metallic glass composites are explored. The results indicate that, even though the ductile reinforcements may alter the evolution and morphology of the shear bands, the overall failure behavior is still controlled by the metallic glass matrix, leading to very little or no improvement in the composite ductility in comparison to the ductility of metallic glass matrix. The results are compared with experimental studies available in the literature. (c) 2005 Acta Materialia Inc. Published by Elsevier Ltd. All rights reserved.

SN 1359-6454

PD JAN

PY 2006

VL 54

IS 1

BP 139

EP 150

DI 10.1016/j.actamat.2005.08.043

UT WOS:000233784500015

ER

PT J

AU Qu, DD

Liss, KD

Yan, K

Reid, M

Almer, JD

Wang, YB

Liao, XZ

Shen, J

AF Qu, Dongdong

Liss, Klaus-Dieter

Yan, Kun

Reid, Mark

Almer, Jonathan D.

Wang, Yanbo

Liao, Xiaozhou

Shen, Jun

TI On the Atomic Anisotropy of Thermal Expansion in Bulk Metallic Glass

SO ADVANCED ENGINEERING MATERIALS

AB Glass transition temperature and plastic yield strength are known to be correlated in metallic glasses. We have observed by in situ synchrotron high energy X-ray diffraction anisotropy of the thermal expansion behavior in the nearest neighbor and second nearest neighbor atomic distances in the building blocks of Zr-Cu-Ni-Al based bulk metallic glass, leading inevitably to shear. Mechanical yielding of the latter on the atomic scale leads to the glass transition and the increase of the free volume. These experimental results uncover the mechanism, how glass transition and yield strength are linked.

RI Qu, Dongdong/G-4521-2011; Liss, Klaus-Dieter/E-8548-2011; Liao,

Xiaozhou/B-3168-2009; Wang, Yanbo/B-3175-2009

OI Qu, Dongdong/0000-0001-5302-4493; Liss,

Klaus-Dieter/0000-0003-4323-0343; Liao, Xiaozhou/0000-0001-8565-1758;

Yan, Kun/0000-0001-5779-6394

SN 1438-1656

PD SEP

PY 2011

VL 13

IS 9

BP 861

EP 864

DI 10.1002/adem.201000349

UT WOS:000295183300009

ER

PT J

AU Chen, JH

Chen, Y

Jiang, MQ

Chen, XW

Fu, HM

Zhang, HF

Dai, LH

AF Chen, J. H.

Chen, Y.

Jiang, M. Q.

Chen, X. W.

Fu, H. M.

Zhang, H. F.

Dai, L. H.

TI Dynamic shear punch behavior of tungsten fiber reinforced Zr-based bulk

metallic glass matrix composites

SO INTERNATIONAL JOURNAL OF IMPACT ENGINEERING

CT Recent Development of Experimental Techniques Under Impact Loading-

IUTAM Symposium

CY 2013

CL Xian, PEOPLES R CHINA

AB Dynamic shear punch tests were carried out on the tungsten fiber reinforced Zr-based bulk metallic glass composites. The experimental results show that with the increasing fiber volume fraction, the failure mode of the composites switches from shear to tensile fracture. A new failure criterion, based on the Tsai-Hill criterion and the unified failure criterion for bulk metallic glasses, is proposed to characterize fracture behavior of this bulk metallic glass composite. It is found that the shear-to-normal strength ratio a controls the transition of failure mode of this metallic glass composite. The underlying mechanism of the transition of failure mode is discussed as well. (C) 2014 Elsevier Ltd. All rights reserved.

OI Dai, LanHong/0000-0001-8991-0358

SN 0734-743X

EI 1879-3509

PD MAY

PY 2015

VL 79

BP 22

EP 31

DI 10.1016/j.ijimpeng.2014.07.006

UT WOS:000351968800005

ER

PT J

AU Qiao, JC

Pelletier, JM

Wang, Q

Jiao, W

Wang, WH

AF Qiao, J. C.

Pelletier, J. M.

Wang, Q.

Jiao, W.

Wang, W. H.

TI On calorimetric study of the fragility in bulk metallic glasses with low

glass transition temperature: (Ce0.72Cu0.28)(90-x) Al10Fex (x=0, 5 or

10) and Zn38Mg12Ca32Yb18

SO INTERMETALLICS

AB Compared with conventional bulk metallic glasses, Ce-based and Zn-based bulk metallic glasses have received considerable attention because of their possible application as structural and functional materials. Kinetic fragility parameter m in amorphous material presents degree of deviations from the Arrhenius law above the glass transition temperature (T-g) of the material. Kinetic fragility parameter (m) and Kauzmann temperature (T-K) in (Ce0.72Cu0.28)(90-x) Al10Fex (x = 0, 5 or 10) and Zn38Mg12Ca32Yb18 bulk metallic glasses have been determined by differential scanning calorimetry (DSC). Results show that Zn38Mg12Ca32Yb18 presents a higher m than (Ce0.72Cu0.28)(90-x) Al10Fex (x = 0, 5 or 10). The activation energies E-g for glass transition are 1.51 eV (x = 0), 1.59 eV (x = 5) and 1.83 eV (x = 10) in (Ce0.72Cu0.28)(90-x) Al10Fex (x = 0, 5 or 10), and 3.59 eV in Zn38Mg12Ca32Yb18, respectively. The values of E-g increase with increasing the Fe content in (Ce0.72Cu0.28)(90-x) Al10Fex (x = 0, 5 or 10) bulk metallic glasses. Kinetic fragility parameter in of bulk metallic glasses increases with the glass transition temperature T-g of bulk metallic glasses, in agreement with previous investigations. (C) 2011 Elsevier Ltd. All rights reserved.

RI wei, jiao/I-7244-2013

SN 0966-9795

EI 1879-0216

PD OCT

PY 2011

VL 19

IS 10

BP 1367

EP 1373

DI 10.1016/j.intermet.2011.04.008

UT WOS:000294522400004

ER

PT J

AU Wang, WH

AF Wang, WH

TI Elastic moduli and behaviors of metallic glasses

SO JOURNAL OF NON-CRYSTALLINE SOLIDS

AB We report apparent correlations among the elastic moduli, fracture strength, Vicker's hardness and glass transition temperature for various and available metallic glasses with marked different elastic and mechanical properties. In particular, an attempt is made to link the observed correlations with glass transition, relaxation and glass-forming ability. The clear correlations imply that the physical properties of glasses would be better controlled by selection of elements with suitable elastic moduli as constituents. (c) 2005 Published by Elsevier B.V.

SN 0022-3093

PD JUN 1

PY 2005

VL 351

IS 16-17

BP 1481

EP 1485

DI 10.1016/j.jnoncrysol.2005.03.024

UT WOS:000229814400021

ER

PT J

AU Yu, JS

Zeng, YQ

Fujita, T

Hashizume, T

Inoue, A

Sakurai, T

Chen, MW

AF Yu, J. S.

Zeng, Y. Q.

Fujita, T.

Hashizume, T.

Inoue, A.

Sakurai, T.

Chen, M. W.

TI On the effect of impurities in metallic glass formation

SO APPLIED PHYSICS LETTERS

AB We report atomic-scale characterization of impurity elements in a Pd40Ni40P20 metallic glass by state-of-the-art atom probe tomography combining with transmission electron microscopy. The significant partitioning of the impurities in heterogeneous nanocrystals of the primarily crystallized glass provides compelling evidence that minor impurities dramatically influence the stability of supercooled liquids by manipulating heterogeneous crystallization of metallic glasses.

RI Inoue, Akihisa/E-5271-2015; Fujita, Takeshi/B-1867-2009; CHEN,

Mingwei/A-4855-2010

OI Fujita, Takeshi/0000-0002-2318-0433; CHEN, Mingwei/0000-0002-8274-3099;

Chen, Mingwei/0000-0002-2850-8872

SN 0003-6951

EI 1077-3118

PD APR 5

PY 2010

VL 96

IS 14

AR 141901

DI 10.1063/1.3373528

UT WOS:000276554600021

ER

PT J

AU Kirimli, HE

Sarlar, K

Konuk, AO

Duman, N

Akdeniz, MV

Kucuk, I

AF Kirimli, Handan Engin

Sarlar, Kagan

Konuk, A. Oguz

Duman, Nagehan

Akdeniz, M. Vedat

Kucuk, Ilker

TI Glass Forming Ability and Magnetic Properties of

Fe36Ni36B19.2Si4.8Nb4-xMx (M = Cu, Zr, Ti, Y, Pt) Bulk Glassy Alloys

Fabricated by Suction Casting

SO JOURNAL OF SUPERCONDUCTIVITY AND NOVEL MAGNETISM

AB In this study, the effects of Cu, Zr, Ti, Y, Pt substitution for Nb additions on the stability and magnetic properties of Fe-Ni-based bulk metallic glass (BMG) alloys fabricated by the suction casting method are investigated. The saturation magnetization (J(s)) and coercivity (H-c) for as-cast Fe36Ni36B19.2Si4.8Nb4-xMx (M = Cu, Ti) BMG alloys were in the range of 0.51 T-0.55 T and 76-779 A/m, respectively. Differential scanning calorimetry curves show that the Fe36Ni36B19.2Si4.8Nb4-xMx (M = Cu, Ti) bulk metallic glasses have a supercooled liquid region for Cu at 44 K and for Ti at 39 K.

RI Akdeniz, M. Vedat/A-7414-2016

OI Akdeniz, M. Vedat/0000-0001-5127-7796

SN 1557-1939

PD MAY

PY 2013

VL 26

IS 5

SI SI

BP 1683

EP 1685

DI 10.1007/s10948-012-1998-y

UT WOS:000323906600051

ER

PT J

AU Xia, L

Li, WH

Fang, SS

Wei, BC

Dong, YD

AF Xia, L

Li, WH

Fang, SS

Wei, BC

Dong, YD

TI Binary Ni-Nb bulk metallic glasses

SO JOURNAL OF APPLIED PHYSICS

AB We studied the glass forming ability of Ni-Nb binary alloys and found that some of the alloys can be prepared into bulk metallic glasses by a conventional Cu-mold casting. The best glass former within the compositional range studied is off-eutectic Ni62Nb38 alloy, which is markedly different from those predicted by the multicomponent and deep eutectic rules. The glass formation mechanism for binary Ni-Nb alloys was studied from the thermodynamic point of view and a parameter gamma* was proposed to approach the ability of glass formation against crystallization. (c) 2006 American Institute of Physics.

OI Xia, Lei/0000-0001-9198-1497

SN 0021-8979

PD JAN 15

PY 2006

VL 99

IS 2

AR 026103

DI 10.1063/1.2158130

UT WOS:000235014700106

ER

PT J

AU Ma, D

Tan, H

Wang, D

Li, Y

Ma, E

AF Ma, D

Tan, H

Wang, D

Li, Y

Ma, E

TI Strategy for pinpointing the best glass-forming alloys

SO APPLIED PHYSICS LETTERS

AB We present a model for the glass forming ability (GFA), based on phase selection of the glass over all the competing crystalline phases. Our analysis indicates that the best glass-forming zone can be either symmetric, or asymmetric, about the eutectic composition. Based on the model predictions we outline a microstructure-based strategy to pinpoint the off-eutectic composition with the optimum GFA. The practical implementation and significance of the strategy is demonstrated by discovering bulk metallic glasses in Zr-Cu and Zr-Cu-Al systems. (c) 2005 American Institute of Physics.

RI Ma, Dong/G-5198-2011; Ma, En/A-3232-2010

OI Ma, Dong/0000-0003-3154-2454;

SN 0003-6951

EI 1077-3118

PD MAY 9

PY 2005

VL 86

IS 19

AR 191906

DI 10.1063/1.1922570

UT WOS:000229397900022

ER

PT J

AU Wang, H

Song, XP

Yao, XD

Zhang, HF

Hu, ZQ

AF Wang, H

Song, XP

Yao, XD

Zhang, HF

Hu, ZQ

TI Crystallization behavior of (Cu60Zr30Ti10)(99)Sn-1 bulk metallic glass

SO JOURNAL OF MATERIALS SCIENCE & TECHNOLOGY

CT Australia-China Materials Science Symposium

CY OCT 22-25, 2004

CL Univ Queensland, Sch Engn, Brisbane, AUSTRALIA

HO Univ Queensland, Sch Engn

AB The crystallization behavior and crystallization kinetics Of (CU60Zr30Ti10)(99)Sn-1 bulk metallic glass was studied by X-ray diffractometry and differential scanning calorimetry. It was found that a two-stage crystallization took place during continuous heating of the bulk metallic glass. Both the glass transition temperature T-g and the crystallization peak temperatures T-p displayed a strong dependence on the heating rate. The activation energy was determined by the Kissinger analysis method. In the first-stage of the crystallization, the transformation of the bulk metallic glass to the phase one occurred with an activation energy of 386 kJ/mol; in the second-stage, the formation of the phase two took place at an activation energy of 381 kJ/mol.

RI Yao, Xiangdong/E-1259-2013

OI Yao, Xiangdong/0000-0002-1235-5090

SN 1005-0302

PD JUN

PY 2005

VL 21

SU 1

BP 51

EP 53

UT WOS:000230317900014

ER

PT J

AU Rezaei, R

Shariati, M

Tavakoli-Anbaran, H

Deng, C

AF Rezaei, Reza

Shariati, Mahmoud

Tavakoli-Anbaran, Hossein

Deng, Chuang

TI Mechanical characteristics of CNT-reinforced metallic glass

nanocomposites by molecular dynamics simulations

SO COMPUTATIONAL MATERIALS SCIENCE

AB Carbon nanotubes (CNTs) have been widely used to strengthen different types of materials including polymers, ceramics, and metals due to their extraordinarily high strength. In this work, we investigated the influence of CNTs on the mechanical characteristics of amorphous metallic glasses by using molecular dynamics simulations. We constructed long (continuous) and short (discontinuous) CNT-reinforced metallic glass nanocomposites, determined their stress-strain responses and elastic modulus, and compared their tensile and compressive behaviors with monolithic metallic glasses. It was found that long CNTs dramatically increased the stiffness and yield strength of the metallic glass, and improved their elastic region of deformation and ability to absorb energy. In contrast, metallic glasses reinforced with short CNTs showed almost no improvement of mechanical properties. (C) 2016 Elsevier B.V. All rights reserved.

RI Tavakoli-Anbaran, Hossein/K-4761-2018

OI Tavakoli-Anbaran, Hossein/0000-0002-4705-3885

SN 0927-0256

EI 1879-0801

PD JUN 15

PY 2016

VL 119

BP 19

EP 26

DI 10.1016/j.commatsci.2016.03.036

UT WOS:000375070800003

ER

PT J

AU Nishikawa, H

WongPiromsarn, K

Abe, H

Takernoto, T

Fukuhara, M

Wada, T

Inoue, A

AF Nishikawa, Hiroshi

WongPiromsarn, Krit

Abe, Hiroya

Takernoto, Tadashi

Fukuhara, Mikio

Wada, Takeshi

Inoue, Akihisa

TI Wetting characteristics of Sn-Ag-Cu solder on Pd-based metallic glass

SO MATERIALS SCIENCE AND ENGINEERING B-ADVANCED FUNCTIONAL SOLID-STATE

MATERIALS

CT 1st International Conference on Science Technology for Advanced Ceramics

(STAC)/2nd International Conference on Joining Technology for New

Metallic Glasses and Inorganic Materials (JTMC)

CY MAY 23-25, 2007

CL Kanagawa, JAPAN

SP Tokyo Inst Technol, 21st Century Ctr Excellence Program, Mat Sci Dept Grp

AB A feasibility study has been conducted to determine whether soldering process can be used for the joining of metallic glasses. The mechanical properties of metallic glasses are extremely attractive compared with conventional crystalline materials. In order to adopt bulk metallic glasses in a broader range of engineering applications, it is very important to establish appropriate joining processes of metallic glasses. During the joint of metallic glass, the most serious issue is the reformation of glassy phase at the high temperature area. Therefore, to avoid the recrystallization of glassy phase, the soldering process has been investigated to join metallic glasses. In this study, the spread test was mainly performed at 523 K for 60 s. Results showed that the Sn-3.0 mass%Ag-0.5 mass%Cu solder deposited on Pd-based metallic glass had better wetting characteristics than the Sn-57 mass%Bi and Sn-51 mass%In solders. The microstructure at the interface between Sn-3.OAg-0.5Cu solder and Pd-based metallic glass was analyzed by scanning electron microscopy. It was clear that the intermetallic compound, PdSn4 phase, was formed at the interface. (C) 2007 Elsevier B.V. All rights reserved.

RI Inoue, Akihisa/E-5271-2015; Wada, Takeshi/B-2431-2015

SN 0921-5107

PD FEB 25

PY 2008

VL 148

IS 1-3

BP 124

EP 127

DI 10.1016/j.mseb.2007.09.069

UT WOS:000256276200029

ER

PT S

AU Mehrer, H

AF Mehrer, Helmut

BE Murch, G

Ochsner, A

Belova, I

TI Diffusion in Glassy Metals

SO DIFFUSION IN ADVANCED MATERIALS

SE Diffusion Foundations

AB Firstly, this paper reminds the reader of some basic facts about the glassy state, then of the various ways to produce amorphous metals with particular emphasis on the route of vitrification from the melt. Vitrification of an undercooled melt is the most important route from the viewpoint of the application of metallic glasses. We compare diffusion in some metallic glasses with related crystalline metals. Glassy metals, also called metallic glasses, comprise conventional [1] and bulk metallic glasses [2,3]. We remind the reader of the major experimental techniques for diffusion studies in metallic glasses. The paper then reviews our current understanding of diffusion in glassy metals (see also [4,5,6]), including conventional as well as bulk metallic glasses and undercooled melts. We cover the temperature dependence of diffusion in metallic glasses and discuss the spectrum of activation parameters of glassy metals and its difference to the corresponding one of crystalline metals. We mention the pressure dependence and the isotope effect and we discuss tracer diffusion and viscosity diffusion for a bulk metallic glass and its undercooled melt. Finally we mention computer simulations of atomic jump processes. The diffusion mechanism in metallic glasses differs from that in crystalline metals and involves thermally activated, highly collective (chain-like or caterpillar-like) diffusion jumps. Finally, we mention diffusion along shearbands in a plastically deformed glassy metal.

SN 2296-3650

BN 978-3-03835-081-1

PY 2014

VL 1

BP 125

EP 151

DI 10.4028/www.scientific.net/DF.1.125

UT WOS:000337494300008

ER

PT S

AU Xu, HW

Du, YL

Cheng, JL

Chen, G

AF Xu, H. W.

Du, Y. L.

Cheng, J. L.

Chen, G.

BE Tan, Y

Ju, DY

TI Crystallization of Cu45Zr48Al7 bulk metallic glass

SO ADVANCED MATERIAL SCIENCE AND TECHNOLOGY, PTS 1 AND 2

SE Materials Science Forum

CT 7th International Forum on Advanced Material Science and Technology

CY JUN 26-28, 2011

CL Dalian, PEOPLES R CHINA

SP Natl Nat Sci Fdn China, KC Wong Educ Fdn, Dalian Univ Technol, Changchun Res Inst Mech Sci Co Ltd

AB The crystallization behavior of Cu45Zr48Al7 bulk metallic glass was studied by differential scanning calorimetry (DSC) and x-ray diffraction (XRD). Cu45Zr48Al7 bulk metallic glass exhibits two-stage crystallization in heating process. The crystallization peak temperature To and T-p2 shifted to higher temperature with increasing heating rate. It was found that the crystalline phases homogeneously nucleared at a constant rate and grew linearly at a constant rate in the supercooled liquid. Different bulk nanostructured alloys can be derived from Cu45Zr48Al7 bulk metallic glass by changing the heating rate and/or annealing temperature.

SN 0255-5476

PY 2011

VL 675-677

BP 205

EP 208

DI 10.4028/www.scientific.net/MSF.675-677.205

UT WOS:000297036200048

ER

PT J

AU Stoica, M

Kolesar, V

Bednarcik, J

Roth, S

Franz, H

Eckert, J

AF Stoica, M.

Kolesar, V.

Bednarcik, J.

Roth, S.

Franz, H.

Eckert, J.

TI Thermal stability and magnetic properties of partially Co-substituted

(Fe(71.2)B(24)Y(4.8))(96)Nb(4) bulk metallic glasses (vol 109, 054901,

2011)

SO JOURNAL OF APPLIED PHYSICS

RI Stoica, Mihai/B-7069-2015

SN 0021-8979

PD MAY 1

PY 2011

VL 109

IS 9

AR 099902

DI 10.1063/1.3587573

UT WOS:000290588500137

ER

PT J

AU Togo, H

Zhang, Y

Kawamura, Y

Mashimo, T

AF Togo, H.

Zhang, Y.

Kawamura, Y.

Mashimo, T.

TI Properties of Zr-based bulk metallic glass under shock compression

SO MATERIALS SCIENCE AND ENGINEERING A-STRUCTURAL MATERIALS PROPERTIES

MICROSTRUCTURE AND PROCESSING

CT 12th International Conference on Rapidly Quenched and Metastable

Materials

CY AUG 21-26, 2005

CL Jeju Isl, SOUTH KOREA

AB Many kinds of bulk metallic glasses have been discovered since the 1990s. However, there have been very few investigations of the dynamic compression properties of bulk metallic glasses with amorphous structured single-phase. In this study, Hugoniot-measurement experiments have been performed on Zr55Al10Ni5Cu30 (at.%) bulk metallic glass by the inclined-mirror method combined with a powder gun in the pressure range up to 45 GPa in order to investigate the elasto-plastic transition and phase transition of the bulk metallic glass. The Hugoniot-elastic limit stress of Zr55Al10Ni5Cu30 bulk metallic glass was > 5 GPa which is much larger than that of the other metals. A kink was observed on the shock velocity versus particle velocity relationship of bulk metallic glass, which may be caused by phase transition. Ductile dimple fracture with a vein pattern was observed on the fracture surfaces of the recovered bulk metallic glass under low pressures. (c) 2006 Elsevier B.V. All rights reserved.

SN 0921-5093

PD MAR 25

PY 2007

VL 449

BP 264

EP 268

DI 10.1016/j.msea.2006.02.431

UT WOS:000245477800057

ER

PT J

AU Xia, L

Dong, YD

AF Xia, L

Dong, YD

TI Glass forming ability and kinetic characters of paramagnetic

Nd60Co40-(x)A1(x) (x = 5, 10, 15) bulk metallic glasses

SO MODERN PHYSICS LETTERS B

AB Paramagnetic Nd60Co40-xAlx (x = 5, 10, 15) bulk metallic glasses (BMGs) were prepared in the shape of rods 2 mm in diameter by suction casting. The ternary alloys have shown distinct glass transitions in Differential Scanning Calorimetry (DSC) measurements and excellent glass-forming ability. The glass transition and crystallization behaviors as well as their kinetics have been studied. The reduced glass transition temperature and the supercooled liquid region of the alloys were found to increase with the increasing content of Al. The role of Al was discussed. The parameter gamma defined by Liu et al. was employed to discuss the glass-forming ability of the alloys and the critical cooling rates as well as the critical section thickness of the alloys were predicted accordingly.

OI Xia, Lei/0000-0001-9198-1497

SN 0217-9849

EI 1793-6640

PD JUN 10

PY 2004

VL 18

IS 14

BP 679

EP 685

DI 10.1142/S0217984904007220

UT WOS:000223115700003

ER

PT J

AU Sun, BA

Pan, MX

Zhao, DQ

Wang, WH

Xi, XK

Sandor, MT

Wu, Y

AF Sun, B. A.

Pan, M. X.

Zhao, D. Q.

Wang, W. H.

Xi, X. K.

Sandor, M. T.

Wu, Y.

TI Aluminum-rich bulk metallic glasses

SO SCRIPTA MATERIALIA

AB The formation and properties of a class of Al-rich bulk metallic glasses (BMGs) are reported. The Al contents for these alloys can reach up to 40 at.%, which is the highest in known BMGs. The Al-rich BMGs deviate greatly from eutectic composition and show high thermal stability and fragility, and very high mechanical strength. These Al-rich BMGs might have implications for Al-based BMGs in general and for understanding the role of Al in glass formation. (C) 2008 Acta Materialia Inc. Published by Elsevier Ltd. All rights reserved.

RI Sun, Baoan/C-6441-2012

OI Sun, Baoan/0000-0001-5306-1817

SN 1359-6462

PD NOV

PY 2008

VL 59

IS 10

BP 1159

EP 1162

DI 10.1016/j.scriptamat.2008.08.003

UT WOS:000259885800034

ER

PT J

AU Wu, SS

Shen, BL

Inoue, A

AF Wu, SS

Shen, BL

Inoue, A

TI Preparation and properties study of bulk Fe75.5Ga3P10.5C4B4Si3 metallic

glass ring by copper mold casting

SO INTERMETALLICS

CT 3rd International Conference on Bulk Metallic Glasses

CY OCT 12-16, 2003

CL Beijing, PEOPLES R CHINA

AB The bulk Fe75.5Ga3P10.5C4B4Si3 metallic glass ring with the outer diameter of 10 mm, the inner diameter of 6 mm and the thickness of 1 mm was successfully prepared by copper mold casting. The Curie temperature is 634 K, the glass transition temperature (T-g), the supercooled liquid region (DeltaT(x)( = T-x-T-g)) and the saturation magnetization of the bulk metallic glass ring in as-made state were 634, 734, 40 K and 1.33 T. The associated activation energy of the bulk metallic glass ring was determined using differential scanning calorimetry and Kissinger's peak shift method. We hopefully can develop the bulk amorphous alloy into the application on a new type of magnetic cores. (C) 2004 Elsevier Ltd. All rights reserved.

RI Inoue, Akihisa/E-5271-2015

SN 0966-9795

PD OCT-NOV

PY 2004

VL 12

IS 10-11

BP 1261

EP 1264

DI 10.1016/j.intermet.2004.07.003

UT WOS:000224566700037

ER

PT J

AU Feng, XL

Yuan, ZZ

Zhang, XY

Cui, LZ

AF Feng Xuelei

Yuan Zizhou

Zhang Xiangyun

Cui Lizhi

TI Effect of Overheating Treatment Time on Mechanical Properties of

Zr-Cu-Based Bulk Metallic Glasses

SO RARE METAL MATERIALS AND ENGINEERING

AB Zr48Cu36Ag8Al8 bulk metallic glasses with 3 mm diameter were prepared with suction-casting into a water-cooling-copper mold for different casting treatment time from the same overheating temperature. The effect of treatment time in metallic liquid on the mechanical properties of Zr-Cu-based bulk metallic glasses were studied by X-ray diffractometer (XRD), differential scanning calorimeter (DSC), compression tests and scanning electron microscope (SEM). The results show that in a certain range of treatment time, longer treatment time of overheating treatment time would increase the density of the free volume and the deformation ability, thus decreasing the local deformation degree and increasing the fracture strength as well as the plasticity of the Zr48Cu36Ag8Al8 bulk metallic glasses.

SN 1002-185X

PD DEC

PY 2015

VL 44

IS 12

BP 3109

EP 3112

UT WOS:000367768800036

ER

PT J

AU Guo, YF

Yavari, AR

Zhang, T

AF Guo, Yaofeng

Yavari, Alain R.

Zhang, Tao

TI On the liquid-state fragility of PdNiCuP metallic glasses

SO JOURNAL OF ALLOYS AND COMPOUNDS

AB It has been widely known that Pd-Ni-Cu-P alloy system possesses the largest glass forming ability among the bulk metallic glasses. But its fragility exhibits relatively low index, lower than other good metallic glass formers. Intensive research has been done on glass forming ability, and it has been suggested that the sluggish diffusive motion and small driving force for nucleation are responsible for this unusual phenomena. However, this apparent contradiction between glass forming ability and fragility is not unique among vitreous materials. Considering the same details of vitrification of water and thermodynamic fragility, as well as unusual expansion during solidification of PdNiCuP metallic glass, it is suggested tentatively that there might be a fragile-to-strong transition during the vitrification of PdNiCuP metallic glass. (c) 2011 Elsevier B.V. All rights reserved.

RI Zhang, Tao/O-4911-2014; yavari, alain/E-8192-2010

SN 0925-8388

PD SEP 25

PY 2012

VL 536

SU 1

BP S91

EP S93

DI 10.1016/j.jallcom.2011.11.085

UT WOS:000310837500021

ER

PT J

AU Liu, MC

Huang, JC

Chou, HS

Lai, YH

Lee, CJ

Nieh, TG

AF Liu, M. C.

Huang, J. C.

Chou, H. S.

Lai, Y. H.

Lee, C. J.

Nieh, T. G.

TI A nanoscaled underlayer confinement approach for achieving

extraordinarily plastic amorphous thin film

SO SCRIPTA MATERIALIA

AB This study demonstrates that the brittle problem of a metallic glass coating can be alleviated by percolating with a nanocrystalline metallic underlayer. The brittle thin film metallic glass can become highly ductile and exhibit a plastic strain of over 50% at room temperature. The present study has an important implication for microelectromechanical systems applications as the lifespan of a brittle amorphous layer can be significantly improved by using an appropriate metallic underlayer. (C) 2009 Acta Materialia Inc. Published by Elsevier Ltd. All rights reserved.

RI Nieh, Tai-Gang/G-5912-2011; Huang, J./C-4276-2013

OI Nieh, Tai-Gang/0000-0002-2814-3746; HUANG, Jacob Chih

Ching/0000-0001-6843-3396

SN 1359-6462

PD OCT

PY 2009

VL 61

IS 8

BP 840

EP 843

DI 10.1016/j.scriptamat.2009.07.010

UT WOS:000269716000018

ER

PT J

AU Zhang, XL

Chen, G

AF Zhang, X. L.

Chen, G.

TI The effects of microalloying on thermal stability, mechanical property

and corrosion resistance of Mg-based bulk metallic glasses

SO JOURNAL OF NON-CRYSTALLINE SOLIDS

AB (Mg65Cu20Y10Zn5)(98) M-2 (M=Ti, Cr) bulk metallic glasses with diameter of 3 mm were prepared by copper mold casting. The effects of Ti or Cr on thermal stability, mechanical property and corrosion resistance were systematically investigated. It's shown that the Glass forming ability of the Mg65Cu20Y10Zn5 bulk metallic glasses slightly decreases with the addition of the elements. Whereas, the strength and corrosion resistance significantly increase. The superior corrosion resistance of the amorphous sample containing Ti or Cr is presented for the forming of homogeneous passive layer with highly protective oxides. (C) 2012 Elsevier B.V. All rights reserved.

RI BAI, JIE/D-7448-2016

SN 0022-3093

PD MAY 15

PY 2012

VL 358

IS 10

BP 1319

EP 1323

DI 10.1016/j.jnoncrysol.2012.03.002

UT WOS:000304226900011

ER

PT J

AU Tang, MB

Zhao, JT

AF Tang, M. B.

Zhao, J. T.

TI Thermodynamic behavior of glass-forming metallic supercooled liquids

SO PHYSICA B-CONDENSED MATTER

AB We analyze the enthalpy of crystallization H-x, and fusion H-m, and calculate the average difference of the heat capacity between supercooled liquid and equilibrium crystalline solid Delta C-ave,. in a series of glass-forming metallic supercooled liquids. It is found that Delta C-ave is close to a constant 13.2 J/mol/K which is an important feature for glass-forming metallic supercooled liquids, and there is no obvious correlation between the parameter phi(=1-H-x/H-m) and the glass-forming ability. The ratio of Delta C-ave to the entropy of fusion is an important parameter in determining the glass-forming ability in metallic glasses. (C) 2013 Elsevier BY. All rights reserved.

SN 0921-4526

PD OCT 1

PY 2013

VL 426

BP 1

EP 5

DI 10.1016/j.physb.2013.05.032

UT WOS:000322630400001

ER

PT J

AU Li, FS

Zhang, T

Guan, SK

Shen, NF

AF Li, FS

Zhang, T

Guan, SK

Shen, NF

TI A novel dual-amorphous-phased bulk metallic glass with soft magnetic

properties

SO MATERIALS LETTERS

AB A new concept of synthesizing dual-amorphous-phased bulk metallic glass (DAPBMG) with soft magnetic properties was proposed. The specific (Fe,Zr)-based bulk metallic glasses with dual-amorphous phases were successfully fabricated by hot-pressed consolidating a well-proportioned mixture of Fe-based and Zr-based glassy powders in the temperature range of their overlapped supercooled liquid region. It was found that the DA-PBMG with content of 50 mass% Fe-based glass still holds soft magnetic properties basically similar to that of the added Fe-based glass. (c) 2005 Elsevier B.V. All rights reserved.

RI Zhang, Tao/O-4911-2014

SN 0167-577X

PD MAY

PY 2005

VL 59

IS 11

BP 1453

EP 1457

DI 10.1016/j.matlet.2004.09.056

UT WOS:000227866700030

ER

PT J

AU Zhang, B

Zhao, DQ

Pan, MX

Wang, WH

Greer, AL

AF Zhang, B

Zhao, DQ

Pan, MX

Wang, WH

Greer, AL

TI Amorphous metallic plastic

SO PHYSICAL REVIEW LETTERS

AB We report cerium-based bulk metallic glasses with an exceptionally low glass transition temperature T-g, similar to or lower than that of many polymers. We demonstrate that, in near-boiling water, these materials can be repeatedly shaped, and can thus be regarded as metallic plastics. Their resistance to crystallization permits extended forming times above T-g and ensures an adequate lifetime at room temperature. Such materials, combining polymerlike thermoplastic behavior with the distinctive properties of metallic glasses, are highly unusual for metallic alloys and have great potential in applications and can also facilitate studies of the supercooled liquid state.

RI Greer, Lindsay/E-9433-2017; Zhang, Bo/B-1381-2013; Greer, Alan

Lindsay/G-1977-2011

SN 0031-9007

EI 1079-7114

PD MAY 27

PY 2005

VL 94

IS 20

AR 205502

DI 10.1103/PhysRevLett.94.205502

UT WOS:000229398100035

PM 16090261

ER

PT J

AU Cheng, X

Wang, Q

Chen, WR

Dong, C

AF Cheng Xu

Wang Qing

Chen WeiRong

Dong Chuang

TI Fe-B-Y-Nb bulk metallic glasses in relation to clusters

SO SCIENCE IN CHINA SERIES G-PHYSICS MECHANICS & ASTRONOMY

AB Bulk metallic glass formations in the Fe-B-Y-Nb quaternary alloy system were investigated by using the cluster line rule in combination with the minor alloying principle. The Fe-B-Y ternary system was selected as the basic system and the intersections of cluster lines were taken as the basic ternary compositions. The basic compositions were further alloyed with minor amounts of Nb. After 3-5 at.% Nb was added, the basic composition Fe68.6B25.7Y5.7, which was developed from the most densely packed cluster Fe8B3, formed 3 mm bulk metallic glasses. These quaternary bulk metallic glasses (Fe68.6B25.7Y5.7)(100-x) Nb (x) (x = 3-5 at.%) are expressed approximately with a unified simple composition formula: (Fe8B3)(1)(Y, Nb)(1). The (Fe68.6B25.7Y5.7)(97)Nb-3 bulk metallic glass has the largest glass forming ability with the following characteristic parameters T (g) = 907 K, T-x = 1006 K, T-g/T-l = 0.644, gamma = 0.434, and longness t = 22 mm. The combination of the cluster line rule and the minor-alloying principle is a promising new route towards the quantitative composition design of multi-component metallic glasses.

SN 1672-1799

PD APR

PY 2008

VL 51

IS 4

BP 421

EP 426

DI 10.1007/s11433-008-0046-1

UT WOS:000254406000011

ER

PT J

AU Qiao, JC

Pelletier, JM

Li, N

Yao, Y

AF Qiao, Ji-chao

Pelletier, Jean-marc

Li, Ning

Yao, Yao

TI Insight on Viscoelasticiy of Ti16.7Zr16.7Hf16.7CU16.7Ni16.7Be16.7 High

Entropy Bulk Metallic Glass

SO JOURNAL OF IRON AND STEEL RESEARCH INTERNATIONAL

AB High entropy bulk metallic glasses show promising mechanical and physical properties. Dynamic mechanical properties of Ti16.7Zr16.7Hf16.7Cu16.7Ni16.7Be16.7 high entropy bulk metallic glass were investigated by mechanical spectroscopy (or called dynamic mechanical analysis). The main (alpha) relaxation was observed in the framework of the loss modulus G '', which is related to the dynamic glass transition behaviour for the glassy materials. From physical model point of view, dynamic mechanical properties of the Ti16.7Zr16.7Hf16.7Cu16.7Ni16.7Be16.7 high entropy bulk metallic glass show good agreement compared with the quasi-point defects theory.

RI Yao, Yao/K-3129-2012

OI Yao, Yao/0000-0002-0879-4269

SN 1006-706X

EI 2210-3988

PD JAN

PY 2016

VL 23

IS 1

BP 19

EP 23

DI 10.1016/S1006-706X(16)30005-X

UT WOS:000373035000005

ER

PT J

AU Fujita, K

Takayama, T

Inoue, A

Zhang, T

Kimura, H

AF Fujita, K

Takayama, T

Inoue, A

Zhang, T

Kimura, H

TI Effect of nanocrystalline dispersion on fatigue in a Zr-based bulk

metallic glass

SO JOURNAL OF THE JAPAN INSTITUTE OF METALS

AB A nano-scale crystal (NC) dispersed bulk metallic glass has both high tensile strength and high ductility. The new alloy is therefore expected as a candidate of high-strength structural materials in machines and structures. However, fatigue life properties in the NC bulk glass has not been examined. In this report, effects of the nanocrystalline dispersion on fatigue was studied in a NC glassy alloy Zr55Al10Cu30Ni5 (at%). Fatigue ratio, fatigue limit sigma(w)/tensile strength sigma(B), in the NC bulk glass was estimated to be about 0.13. The value was 3 times larger than that of single phase bulk metallic glasses with the same composition reported in the literature. It is considered that the inhibition of the slip initiation and growth was induced by the nanocrystal dispersion and it increased the sigma(w).

RI Kimura, Hisamichi/D-5449-2012; Inoue, Akihisa/E-5271-2015

SN 0021-4876

EI 1880-6880

PD FEB

PY 2003

VL 67

IS 2

BP 79

EP 84

DI 10.2320/jinstmet1952.67.2_79

UT WOS:000181559400003

ER

PT J

AU Chen, B

Shi, TL

Liao, GL

AF Chen Biao

Shi Tielin

Liao Guanglan

TI Laser Welding of Zr41Ti14Cu12Ni10Be23 Bulk Metallic Glass and Zirconium

Metal

SO JOURNAL OF WUHAN UNIVERSITY OF TECHNOLOGY-MATERIALS SCIENCE EDITION

AB The laser bonding technology between the Zr41Ti 14Cu12Ni10Be23 bulk metallic glass and zirconium metal was investigated under welding parameters of 1.3 kW and 7 m/min. The welded bead, microstructure, and micro-hardness of the welded joint were examined by Keyence, transmission electron microscopy, scanning electron microscopy, and Vickers hardness, respectively. The experimental results showed that the Zr41Ti 14Cu12Ni10Be2 bulk metallic glass and zirconium metal were successfully bonded together. The Zr41Ti 14Cu12Ni10Be2 in the base material zone maintained amorphous structure, and the welding fusion zone kept the hardness as high as as-received BMG. Therefore, the laser welding technology can be used to achieve successful bonding of bulk metallic glasses and crystallization metal.

SN 1000-2413

EI 1993-0437

PD AUG

PY 2014

VL 29

IS 4

BP 786

EP 788

DI 10.1007/s11595-014-0997-1

UT WOS:000346400300027

ER

PT S

AU Chiba, A

Kawamura, Y

Nishida, M

Yamamuro, T

AF Chiba, A.

Kawamura, Y.

Nishida, M.

Yamamuro, T.

BE Itoh, S

Yoh, JJ

Hokamoto, K

TI Explosive Welding of ZrTiCuNiBe Bulk Metallic Glass to Crystalline Cu

Plate

SO EXPLOSION, SHOCK WAVE AND HIGH-ENERGY REACTION PHENOMENA

SE Materials Science Forum

CT 3rd International Symposium on Explosion, Shock Wave and High-Energy

Reaction Phenomena

CY SEP 01-03, 2010

CL Seoul Natl Univ, Seoul, SOUTH KOREA

SP Seoul Natl Univ, Korean Fed Sci & Technol Soc, Korean Soc Combust, Korea Inst Mil Sci & Technol, Seoul Convent Bur, Korea Tourism Org, Natl Space Lab, Korea, Kumamoto Univ, Shock Wave & Condensed Matter Res Ctr, Kumamoto Univ Global COE Program Global Initiat Ctr Pulsed Power Engn, Kumamoto Univ Discretionary Fund, Japan Explos Soc, Tech Sect Explos Impuls Proc, Japan Soc Tech Plastic, Comm High-Energy-Rate-Form, Inst Adv Aerosp Technol

HO Seoul Natl Univ

AB Metallic glass or amorphous alloys have been gaining popularity due to their low density, high strength, high fracture toughness, good corrosion and good wear resistance. Especially, bulk metallic glasses (BMGs), which are made in bulk form with a thickness of similar to 10mm at slow cooling rates of the order of 1 similar to 100K/s, have been noted as an industrial application. The welding to other materials becomes very important. Explosive welding of most popular Zr41.2Ti13.8Cu10Ni12.5Be22.5 bulk metallic glass to crystalline pure Cu plate is investigated in this paper and the BMG was found to retain the amorphous structure. The sound bonding with other materials is expected to push forward the application of BMGs for industrial usage.

RI nishida, minoru/E-4027-2012

SN 0255-5476

PY 2011

VL 673

BP 119

EP +

DI 10.4028/www.scientific.net/MSF.673.119

UT WOS:000296071600018

ER

PT J

AU Czeppe, T

Ochin, P

Sypien, A

Anastassova, S

AF Czeppe, Tomasz

Ochin, Patrick

Sypien, Anna

Anastassova, Solzitsa

TI The crystallization of (NiCu)ZrTiAlSi Glass/Crystalline composite

SO ADVANCED ENGINEERING MATERIALS

OI Sypien, Anna/0000-0001-7601-1943

SN 1438-1656

PD JUN

PY 2007

VL 9

IS 6

BP 500

EP 504

DI 10.1002/adem.200700048

UT WOS:000247885800016

ER

PT J

AU Qiao, JC

Pelletier, JM

AF Qiao, J. C.

Pelletier, J. M.

TI Isochronal and isothermal crystallization in Zr55Cu30Ni5 Al-10 bulk

metallic glass

SO TRANSACTIONS OF NONFERROUS METALS SOCIETY OF CHINA

AB Non-isothermal crystallization transformation kinetics and isothermal crystallization kinetics in super-cooled liquid region (SLR) in Zr55Cu30Ni5Al10 bulk metallic glasses were studied by differential scanning calorimetry (DSC) and X-ray diffraction (XRD). In isochronal mode, the average values of activation energy in Zr55Cu30Ni5Al10 bulk metallic glass determined by different models (Kissinger method, Flynn-Wall-Ozawa method and Augis-Bennett method) are in good agreement with each other. In addition, the isothermal transformation kinetics in Zr55Cu30Ni5Al10 bulk metallic glasses was described by the Johnson-Mehl-Avrami (JMA) model. For Zr55Cu30Ni5Al10 bulk metallic glass, the Avrami exponent n ranges from 2.2 to 2.9, indicating that crystallization mechanism in the bulk metallic glass was mainly diffusion-controlled; crystal growth is controlled by long range ordering diffusion in three-dimensional growth during isothermal crystallization process. The average value of activation energy in Zr55Cu30Ni5Al10 bulk metallic glass is 469 kJ/mol in isothermal transformation process.

SN 1003-6326

EI 2210-3384

PD MAR

PY 2012

VL 22

IS 3

BP 577

EP 584

DI 10.1016/S1003-6326(11)61216-8

UT WOS:000302864800014

ER

PT J

AU Li, S

Wang, RJ

Wang, WH

AF Li, S.

Wang, R. J.

Wang, W. H.

TI Bulk metallic glasses based on rare-earth elements in lanthanum series

SO JOURNAL OF NON-CRYSTALLINE SOLIDS

AB A series of bulk metallic glasses (BMGs) based on the rare-earth (RE) elements in lanthanum family have been obtained by a copper mold casting method. These chemical comparable RE elements with 'continuous' range of atomic size, electron structure and elastic constants may provide a good system for systematically exploring the BMG-forming characteristics. The glass-forming ability, elastic properties, thermal stability and their correlations in the RE-based BMGs have been investigated. (c) 2006 Published by Elsevier B.V.

SN 0022-3093

EI 1873-4812

PD OCT 1

PY 2006

VL 352

IS 36-37

BP 3942

EP 3946

DI 10.1016/j.jnoncrysol.2006.05.039

UT WOS:000241098800028

ER

PT J

AU Wu, J

Wang, Q

Chen, WR

Zhang, QY

Qiang, JB

Dong, C

AF Wu, Jiang

Wang, Qing

Chen, Weirong

Zhang, Qingyu

Qiang, Jianbing

Dong, Chuang

TI Glass formation of ternary Sm-based Sm-Al-Co bulk metallic glasses

SO JOURNAL OF UNIVERSITY OF SCIENCE AND TECHNOLOGY BEIJING

AB Ternary Sm-based Sm-Al-Co alloys at specific compositions designed using an e/a- and cluster-related criteria exhibit high glass forming abilities and form bulk glassy rods of 3 mm in diameter by a copper mold suction-casting method. Four compositions of bulk metallic glasses (BMGs) are Sm50Al25Co25, Sm52Al24Co24, Sm54Al23Co23 and Sm56Al22Co22, which all satisfy a constant conduction electron concentration of 1.5. Among them, the BMG exhibiting the largest reduced glass transition temperature (T-rg) is Sm50Al25Co25, which reaches 0.648. The glass transition temperature T-g and the onset crystallization temperature T-x of this alloy are respectively 579 and 640 K at a heating rate of 20 K/min.

RI Zhang, Qingyu/O-8472-2015

SN 1005-8850

PD JUN

PY 2007

VL 14

SU 1

BP 50

EP 53

DI 10.1016/S1005-8850(07)60108-X

UT WOS:000250593400013

ER

PT J

AU Tamura, T

Kamikihara, D

Mizutani, Y

Miwa, K

AF Tamura, Takuya

Kamikihara, Daisuke

Mizutani, Yoshiki

Miwa, Kenji

TI Effects of electromagnetic vibrations on glass-forming ability in

Fe-Co-B-Si-Nb bulk metallic glasses

SO MATERIALS TRANSACTIONS

AB It is known that cooling rate from the liquid state is an important factor for producing the bulk metallic glasses. However, almost no other factors such as electric and/or magnetic fields were investigated. The present authors have reported that a new method for producing Mg-Cu-Y bulk metallic glasses by using electromagnetic vibrations is effective in forming the metallic glass phase. However, effects of the electromagnetic vibrations on glass-forming ability in other alloy systems are not investigated. Thus, this study aims to investigate effects of the electromagnetic vibrations on glass-forming ability in Fe-Co-B-Si-Nb bulk metallic glasses. As a result, it was found that glass-forming ability of Fe-Co-B-Si-Nb alloys also enhances with increasing the electromagnetic vibration force. Moreover, the electromagnetic vibrations were found to affect the increase of the cooling rate and the decrease in the number of crystal nuclei directly, but not to affect the crystal growing rate.

RI Tamura, Takuya/D-4854-2017

OI Tamura, Takuya/0000-0001-6083-913X

SN 1345-9678

EI 1347-5320

PD MAY

PY 2006

VL 47

IS 5

BP 1360

EP 1364

DI 10.2320/matertrans.47.1360

UT WOS:000238966400015

ER

PT J

AU Sun, YL

Sun, YJ

Yang, P

Li, HM

AF Sun, Yong Li

Sun, Ya Juan

Yang, Ping

Li, Hua Ming

TI Cluster distribution entropy in Ti50Cu50 and Ti50Cu45Ni5 metallic

glasses

SO JOURNAL OF NON-CRYSTALLINE SOLIDS

AB It remains unclear how a cluster packing pattern affects the glass forming ability of bulk metallic glasses. The topological structures of Ti50Cu50 and Ti50Cu45Ni5 metallic glasses obtained by molecular dynamics simulations are compared to investigate the relation between the atomic structure and glass forming ability. The structural analysis shows that the addition of Ni in Ti50Cu50 alloy can significantly change the topological structure. Cluster distribution entropy is proposed to quantitatively descript the disordered degree of cluster distribution. It is shown that the cluster distribution entropy provides a statistical approach for assessing the microstructures of metallic glasses. (C) 2014 Elsevier B.V. All rights reserved.

SN 0022-3093

EI 1873-4812

PD SEP 1

PY 2014

VL 398

BP 16

EP 18

DI 10.1016/j.jnoncrysol.2014.04.020

UT WOS:000338819700003

ER

PT J

AU Louzguine-Luzgin, DV

Louzguina-Luzgina, LV

Churyumov, AY

AF Louzguine-Luzgin, Dmitri V.

Louzguina-Luzgina, Larissa V.

Churyumov, Alexander Yu.

TI Mechanical Properties and Deformation Behavior of Bulk Metallic Glasses

SO METALS

AB Metallic glasses demonstrate unique properties, including large elastic limit and high strength, which make them attractive for practical applications. Unlike crystalline alloys, metallic glasses, in general, do not exhibit a strain hardening effect, while plastic deformation at room temperature is localized in narrow shear bands. Room-temperature mechanical properties and deformation behavior of bulk metallic glassy samples and the crystal-glassy composites are reviewed in the present paper.

RI LOUZGUINE, Dmitri/D-2492-2010

OI LOUZGUINE, Dmitri/0000-0001-5716-4987

SN 2075-4701

PD MAR

PY 2013

VL 3

IS 1

BP 1

EP 22

DI 10.3390/met3010001

UT WOS:000343292200001

ER

PT J

AU Xu, H

Tan, XH

Dong, YD

AF Xu, H

Tan, XH

Dong, YD

TI Crystalline behaviour and magnetic properties of Nd55Fe30Al10Dy5 bulk

metallic glasses alloy

SO RARE METAL MATERIALS AND ENGINEERING

CT 5th International Workshop on Ordered Intermetallics and Advanced

Metallic Materials

CY OCT 06-09, 2003

CL Changdu, PEOPLES R CHINA

AB Crystalline behavior and magnetic properties of Nd55Fe30Al10Dy5 bulk metallic glasses alloy were investigated by differential scanning calorimeter (DSC), x-ray diffraction (XRD) and the vibrating sample magnetometer (VSM). Neither glass transition nor supercooled liquid region before crystallization was observed for the as-cast Nd55Fe30Al10Dy5 bulk metallic glasses alloy. The as-cast Nd55Fe30Al10Dy5 alloy shows the intrinsic coercivity with 452kA/m, which is higher than Nd60Fe30Al10 alloy. With increasing of annealed temperature, the intrinsic coercivity of the alloy decreases significantly, while the saturation magnetization and remanence decrease monotonously. The Nd55Fe30Al10Dy5 alloy shows soft magnetic behavior after annealing at 773K for 30 min.

SN 1002-185X

PD JUN

PY 2004

VL 33

IS 6

SU 1

BP 183

EP 186

UT WOS:000222496500043

ER

PT S

AU Louzguine, DV

AF Louzguine (Luzgin), D. V.

BA Louzguine, DV

BF Louzguine, DV

TI Formation of metallic glasses and their general physical properties

SO METALLIC GLASSES AND THEIR COMPOSITES

SE Materials Research Foundations

AB Metallic glasses and bulk metallic glasses (BMGs), in particular, are usually produced by solidification of a melt while amorphous alloys can be produced by various other techniques including physical and chemical vapor deposition, electrodeposition, mechanical attrition, ion implantation, sintering and others. The formation mechanisms of bulk metallic glasses and their general physical properties are discussed in the present chapter.

SN 2471-8890

EI 2471-8904

BN 978-1-945291-42-5

PY 2018

VL 19

BP 1

EP 18

UT WOS:000417007800001

ER

PT J

AU Satta, M

Palumbo, M

Rizzi, P

Baricco, M

AF Satta, Marta

Palumbo, Mauro

Rizzi, Pada

Baricco, Marcello

TI Ternary compounds and glass formation in the Cu-Mg-Y system

SO ADVANCED ENGINEERING MATERIALS

RI Baricco, Marcello/B-4075-2013; Rizzi, Paola/I-8810-2012

OI Baricco, Marcello/0000-0002-2856-9894; Rizzi, Paola/0000-0002-3977-2839

SN 1438-1656

EI 1527-2648

PD JUN

PY 2007

VL 9

IS 6

BP 475

EP 479

DI 10.1002/adem.200700040

UT WOS:000247885800010

ER

PT J

AU Wang, JG

Chan, KC

Fan, JC

Xia, L

Wang, G

Wang, WH

AF Wang, J. G.

Chan, K. C.

Fan, J. C.

Xia, L.

Wang, G.

Wang, W. H.

TI Buckling of metallic glass bars

SO JOURNAL OF NON-CRYSTALLINE SOLIDS

AB Uniaxial compression tests of slender metallic glass bars of composition Zr-52 5Cu17.9Ni14.6Al10Ti5 (at.%) have been conducted. It was found that the Zr-based metallic glass bars have a tendency to buckle elastically or plastically rather than to yield or fracture if its slenderness ratio is over a critical value. The elastic buckling undermines the intrinsic strength of the metallic glass, but the plastic buckling imparts the metallic glass a benign failure mode and avoids the catastrophic brittle fracture. The phenomena are understood by the unique stress state across the bar. The result has implication for the measurement of mechanical properties of bulk metallic glasses and is of significance in the application of metallic glass members in engineering structures. (C) 2013 Elsevier B.V. All rights reserved.

OI Chan, K.C./0000-0002-6173-5532; Xia, Lei/0000-0001-9198-1497

SN 0022-3093

EI 1873-4812

PD MAR 1

PY 2014

VL 387

BP 1

EP 5

DI 10.1016/j.jnoncrysol.2013.12.008

UT WOS:000333778500001

ER

PT J

AU Yamada, R

Yodoshi, N

Kawasaki, A

Watanabe, R

AF Yamada, Rui

Yodoshi, Noriharu

Kawasaki, Akira

Watanabe, Ryuzo

TI Consolidation of Fe-Co Based Metallic Glassy Powder by SPS Method

SO JOURNAL OF THE JAPAN INSTITUTE OF METALS

AB Metallic glasses have been reported to exhibit excellent properties such as high strength, high corrosion resistance, high wear resistance, resulting from their random Structure. In addition, metallic glasses usually exhibit a drastic reduction in viscosity in the Supercooled liquid region. Therefore, metallic glasses have excellent workability in this temperature range and it has already been reported that large size Of bulk metallic glasses are successfully fabricated in several Zr-, Pd-based metallic glasses.

In recent years, Fe-based metallic glasses have been intensively studied because of their excellent mechanical performance, excellent magnetic properties and rich resources. However, due to their poor glass forming ability, the size of bulk metallic glasses is limited using a copper mold casting technique.

In the present study, [(Fe0.5Co0.5)(0.75)Si0.05B0.2](96)Nb-4 bulk metallic glasses are fabricated by spark plasma sintering (SPS) of amorphous powders which have been prepared by a gas atomization. To find optimum conditions in the SPS process, Time-Temperature-Transformation diagram (TTT diagram) is also constructed by isothermal differential scanning calorimetry. After consolidation of metallic glassy powders, mechanical properties of consolidated glassy specimens are measured by compressive tests.

As a result, the TTT diagram call be constructed and maximum incubation time call be predicted at any holding temperature. Using SPS method, large size and nearly 100% relative dense glassy compacts are obtained with a loading pressure of 75 MPa and 400 MPa, comprising full amorphous in the case within incubation time. Compressive tests indicate that mechanical properties of consolidated specimens are still low, and one of the reasons may be the formation of approximately 50 nanometer crystalline phases between each particle observed by Transmission Electron Microscope (TEM).

RI Yodoshi, Noriharu/K-8072-2012

OI Yodoshi, Noriharu/0000-0003-2894-1788

SN 0021-4876

EI 1880-6880

PD APR

PY 2009

VL 73

IS 4

BP 299

EP 305

DI 10.2320/jinstmet.73.299

UT WOS:000266310400009

ER

PT J

AU Bakai, AS

Bakai, SA

Eckert, J

Neklyiadov, IM

Savchenko, VI

AF Bakai, A. S.

Bakai, S. A.

Eckert, J.

Neklyiadov, I. M.

Savchenko, V. I.

TI Mixed viscous flow and softening of bulk metallic glasses

SO JOURNAL OF NON-CRYSTALLINE SOLIDS

CT 12th International Conference on Liquid and Amorphous Metals (LAM12)

CY JUL 11-16, 2004

CL Metz, FRANCE

AB Plastic deformation of Zr52.5Ti5Cu17.9Ni14.6Al10 bulk metallic glass is investigated in a wide range of the strain rates,, and temperatures, T. Based on these results, a map of the plastic deformation modes as a function of strain rate and temperature is depicted. The region of the mixed flow contiguous to the inhomogeneous plastic deformation region is identified. In this region, at large values of F, the glass softens significantly. This effect is interpreted as a result of the cluster fragmentation. (c) 2007 Elsevier B.V. All rights reserved.

SN 0022-3093

EI 1873-4812

PD OCT 15

PY 2007

VL 353

IS 32-40

BP 3754

EP 3757

DI 10.1016/j.jnoncrysol.2007.05.142

UT WOS:000250235200152

ER

PT J

AU Hu, Y

Yan, HH

Song, Y

Li, D

Liu, B

Yan, ZJ

AF Hu Yong

Yan Honghong

Song Yu

Li Dan

Liu Bang

Yan Zhijie

TI Effect of Atomic Order Degree on Isothermal Crystallization Process of

Zr55Al10Ni5Cu30 Bulk Metallic Glass

SO RARE METAL MATERIALS AND ENGINEERING

AB The Zr55Al10Ni5Cu30 bulk metallic glasses with different atomic order degrees were obtained by changing the melting time of master alloy ingots and melt cooling rate, and the effect of atomic order degree on the crystallization process of Zr55Al10Ni5Cu30 bulk metallic glass was investigated by differential scanning calorimeter. The results show that the increase of atomic order degree of Zr55Al10Ni5Cu30 bulk metallic glass results in an obvious decrease of the incubation period but has no obvious effect on the mechanism of nucleation and growth of crystallization phase, which is diffusion-controlled three-dimensional growth and nucleation rate increasing with the time increasing. In addition, the increase of atomic order degree causes a decrease of Avrami exponent to a certain extent.

SN 1002-185X

PD JUN

PY 2014

VL 43

IS 6

BP 1357

EP 1360

UT WOS:000338483700017

ER

PT J

AU Tao, PJ

Yang, YZ

Chen, X

Gao, J

Chen, XC

AF Tao, Pingjun

Yang, Yuanzheng

Chen, Xin

Gao, Jian

Chen, Xianchao

TI Enhanced wear resistance in Zr-based bulk metallic glasses by hydrogen

SO INTERNATIONAL JOURNAL OF HYDROGEN ENERGY

AB Plate-like Zr60Ni25Al15 bulk metallic glasses (BMGs) were prepared under the gaseous mixture of hydrogen and argon. The effects of hydrogen on micro-hardness and wear resistance of the BMGs were researched. The results showed that BMGs fabricated under the gaseous mixture of hydrogen and argon exhibit higher micro-hardness and superior wear resistance, i.e., hydrogen addition can increase the wear resistance of bulk metallic glass dramatically. The enhanced wear resistance of the BMGs is attributed to the increment in micro-hardness and free volume introduced by the addition of hydrogen into the BMG matrix. The findings in present paper demonstrate that rapid solidification methods combining with hydrogenation could be an attractive means to improve the mechanical properties of bulk metallic gasses. Copyright (C) 2013, Hydrogen Energy Publications, LLC. Published by Elsevier Ltd. All rights reserved.

SN 0360-3199

PD JUL 17

PY 2013

VL 38

IS 21

BP 9052

EP 9056

DI 10.1016/j.ijhydene.2013.05.001

UT WOS:000322052800046

ER

PT J

AU Shek, CH

Lin, GM

AF Shek, CH

Lin, GM

TI Dilatometric measurements and calculation of effective pair potentials

for Zr41Ti14Cu12.5Ni10Be22.5 bulk metallic glass

SO MATERIALS LETTERS

AB Dilatation measurement was conducted on a Zr-Ti-Cu-Ni-Be bulk metallic glass to obtained information about the average pair potential in the five-component alloy. By assuming a Lannard-Jones type potential, the average nearest-neighbor separation and the effective depth of the pair potential are found to be 0.32 nm and 0.23 eV, respectively, which agrees very well with the published results measured with other distinctively different techniques. (C) 2002 Elsevier Science B.V. All rights reserved.

RI SHEK, Chan Hung/J-3857-2015

OI SHEK, Chan Hung/0000-0002-6870-523X

SN 0167-577X

PD JAN

PY 2003

VL 57

IS 5-6

BP 1229

EP 1232

AR PII S0167-577X(02)00963-1

DI 10.1016/S0167-577X(02)00963-1

UT WOS:000180262500037

ER

PT J

AU Madge, SV

AF Madge, Shantanu V.

TI Toughness of Bulk Metallic Glasses

SO METALS

AB Bulk metallic glasses (BMGs) have desirable properties like high strength and low modulus, but their toughness can show much variation, depending on the kind of test as well as alloy chemistry. This article reviews the type of toughness tests commonly performed and the factors influencing the data obtained. It appears that even the less-tough metallic glasses are tougher than oxide glasses. The current theories describing the links between toughness and material parameters, including elastic constants and alloy chemistry (ordering in the glass), are discussed. Based on the current literature, a few important issues for further work are identified.

OI Madge, Shantanu/0000-0001-7996-8652

SN 2075-4701

PD SEP

PY 2015

VL 5

IS 3

BP 1279

EP 1305

DI 10.3390/met5031279

UT WOS:000362644900010

ER

PT J

AU Granata, D

Fischer, E

Wessels, V

Loffler, JF

AF Granata, D.

Fischer, E.

Wessels, V.

Loeffler, J. F.

TI The detrimental effect of flux-induced boron alloying in Pd-Si-Cu bulk

metallic glasses

SO APPLIED PHYSICS LETTERS

AB We report on advanced insights into the fluxing of Pd-Si-Cu bulk metallic glasses. Flux-induced boron alloying and trapping of oxides are found to be associated with the employed boron oxide fluxing agent, and both influence the attainable glass-forming ability (GFA) in opposite ways. Incorporated boron strongly deteriorates the GFA due to a rising liquidus temperature, while the oxygen reduction improves it. Thus, proper fine-tuning of the fluxing time and overheating characteristics leads to an enhancement of GFA. In the current case, the critical diameter of Pd77.5Si16.5Cu6 bulk metallic glasses can be increased to 15 mm, as compared to 3mm in the unfluxed case. Based on these results, we illustrate that the development of further fluxing agents is crucial for enhancement of the key properties of bulk metallic glasses. (C) 2015 AIP Publishing LLC.

SN 0003-6951

EI 1077-3118

PD JAN 5

PY 2015

VL 106

IS 1

AR 011902

DI 10.1063/1.4905174

UT WOS:000347976900014

ER

PT S

AU Louzguine, DV

AF Louzguine (Luzgin), D. V.

BA Louzguine, DV

BF Louzguine, DV

TI Applications related to structural, functional, magnetic, chemical and

biological properties

SO METALLIC GLASSES AND THEIR COMPOSITES

SE Materials Research Foundations

AB Bulk metallic glasses are suitable for different applications owing to their excellent structural, functional, magnetic, chemical and biological properties. This chapter is devoted to this subject.

SN 2471-8890

EI 2471-8904

BN 978-1-945291-42-5

PY 2018

VL 19

BP 294

EP 320

UT WOS:000417007800007

ER

PT S

AU Lee, JK

Kim, HJ

Yamasaki, M

Kawamura, Y

Bae, JC

AF Lee, JK

Kim, HJ

Yamasaki, M

Kawamura, Y

Bae, JC

BE Zhong, ZY

Saka, H

Kim, TH

Holm, EA

Han, YF

Xie, XS

TI Synthesis of Cu-based bulk metallic glass matrix composites by warm

processing of gas atomized powders

SO PRICM 5: THE FIFTH PACIFIC RIM INTERNATIONAL CONFERENCE ON ADVANCED

MATERIALS AND PROCESSING, PTS 1-5

SE Materials Science Forum

CT 5th Pacific Rim International Conference on Advanced Materials and

Processing

CY NOV 02-05, 2004

CL Beijing, PEOPLES R CHINA

SP Chinese Soc Met, Japan Inst Met, Korea Inst Met & Mat, Minerals, Met & Mat Soc

AB The bulk metallic glass matrix composite comprising Cu54Ni6Zr22Ti18 metallic glass powder and ductile brass powder was fabricated by the warm process. The warm process was carried out by spark plasma sintering, which led to the homogeneous distribution of both phases of brass and metallic glass without pores. The metallic glass matrix composite material exhibits the same crystallization behavior of the metallic glass powder. A compressive strength of 1.0 GPa with a plastic strain of 3% was obtained in the present metallic glass composite. The composite with enhanced strength and ductility was successfully achieved by introducing a ductile phase in the hard bulk metallic glass.

RI Yamasaki, Michiaki/B-1123-2018

SN 0255-5476

BN 0-87849-960-1

PY 2005

VL 475-479

BP 3419

EP 3422

DI 10.4028/www.scientific.net/MSF.475-479.3419

PN 1-5

UT WOS:000227494704018

ER

PT J

AU Zhang, XL

Sun, JL

Luo, J

Wang, BB

Cheng, JL

AF Zhang, Xuliang

Sun, Jingli

Luo, Jian

Wang, BaoBing

Cheng, Jialin

TI Mechanical and corrosion behaviour of in situ intermetallic phases

reinforced Mg-based glass composite

SO MATERIALS SCIENCE AND TECHNOLOGY

AB A (Mg65Cu10Ni10Y10Zn5)(91)Zr-9 bulk metallic glass matrix composite, reinforced by in situ formed intermetallic phase, has been fabricated. In contrast to the monolithic Mg-based bulk metallic glasses (BMGs), the composite showed much higher fracture strength of 1039MPa and a significant plastic strain of more than 5%. Moreover, the effect of in situ formed intermetallic phase on the corrosion behaviour of the composite was also studied. The results indicated that the corrosion resistance of the composite was only slightly lower than that of the monolithic Mg-based BMGs, but still much higher than that of the AZ31 magnesium alloy. This finding gives us a new clue to enhance the mechanical properties and corrosion resistance of Mg-based alloy by designing appropriate metallic glass composites.

SN 0267-0836

EI 1743-2847

PY 2017

VL 33

IS 10

BP 1186

EP 1191

DI 10.1080/02670836.2016.1271934

UT WOS:000401762700006

ER

PT J

AU Proffen, T

Page, KL

McLain, SE

Clausen, B

Darling, TW

TenCate, JA

Lee, SY

Ustundag, E

AF Proffen, T

Page, KL

McLain, SE

Clausen, B

Darling, TW

TenCate, JA

Lee, SY

Ustundag, E

TI Atomic pair distribution function analysis of materials containing

crystalline and amorphous phases

SO ZEITSCHRIFT FUR KRISTALLOGRAPHIE

AB The atomic pair distribution function (PDF) approach has been used to study the local structure of liquids, glasses and disordered crystalline materials. In this paper, we demonstrate the use of the PDF method to investigate systems containing a crystalline and an amorphous structural phase. We present two examples: Bulk metallic glass with crystalline reinforcements and Fontainebleau sandstone, where an unexpected glassy phase was discovered. In this paper we also discuss the refinement methods used in detail.

RI Lujan Center, LANL/G-4896-2012; Page, Katharine/C-9726-2009; Proffen,

Thomas/B-3585-2009; Ustundag, Ersan/C-1258-2009; Clausen,

Bjorn/B-3618-2015

OI Page, Katharine/0000-0002-9071-3383; Proffen,

Thomas/0000-0002-1408-6031; Ustundag, Ersan/0000-0002-0812-7028;

Clausen, Bjorn/0000-0003-3906-846X; McLain, Sylvia/0000-0002-3347-7759

SN 0044-2968

PY 2005

VL 220

IS 12

BP 1002

EP 1008

DI 10.1524/zkri.2005.220.12.1002

UT WOS:000233375500004

ER

PT J

AU Chen, HY

Cao, J

Song, XG

Qi, JL

Feng, JC

AF Chen, Haiyan

Cao, Jian

Song, Xiaoguo

Qi, Junlei

Feng, Jicai

TI Effect of ion beam irradiation surface treatment on solid-state bonding

of Zr-based bulk metallic glass to pure copper

SO INDIAN JOURNAL OF PURE & APPLIED PHYSICS

AB The surface of Zr-based bulk metallic glass and pure copper was irradiated by 1 keV argon ion beam before solid-state bonding. In addition, the sound solid-state joints without macroscopic deformation were obtained at 440 degrees C for 15 min. Argon ion beam irradiation has a positive effect on the removal of oxide film on the bulk metallic glass and pure copper. The maximum tensile strength of joints was 131.06 MPa, which is much higher than the joints without surface treatment. The bulk metallic glass after bonding retained the unique amorphous structure, indicating that thermal cycle, microscopic deformation and atomic diffusion caused no crystallization of bulk metallic glass. Argon ion beam surface treatment provides a feasible route for the sound solid-state bonding of bulk metallic glass.

RI L, Qi/K-2628-2013

OI junlei, qi/0000-0003-3367-1241

SN 0019-5596

EI 0975-1041

PD MAR

PY 2014

VL 52

IS 3

BP 162

EP 165

UT WOS:000333501300003

ER

PT J

AU Sun, YL

Shen, J

AF Sun, Y. L.

Shen, J.

TI Icosahedral ordering in Cu60Zr40 metallic glass: Molecular dynamics

simulations

SO JOURNAL OF NON-CRYSTALLINE SOLIDS

AB The atomic structure of bulk metallic glasses has long been mysterious for the lack of long range order. Although the solute-centered cluster packing model has recently been proposed to disclose the nanoscale medium-range order (MRO), the atomic packing scheme in systems with large Solute concentration remains obscure. In this work, the atomic structure of Cu60Zr40 metallic glass is investigated via molecular dynamics Simulations with the Finnis-Sinclair potential. It is found that the fragments of icosahedra are dominant in the model metallic glass. The icosahedra are completely centered by solvent atoms. Extended clusters composed of two icosahedra interpenetrate and form MRO with 3-11 icosahedra. It is suggested that the structure of Cu60Zr40 metallic glass can be described by the aggregation of icosahedra. (C) 2009 Elsevier B.V. All rights reserved.

SN 0022-3093

PD SEP 1

PY 2009

VL 355

IS 31-33

BP 1557

EP 1560

DI 10.1016/j.jnoncrysol.2009.06.010

UT WOS:000269270200001

ER

PT J

AU Xia, MX

Zhang, SG

Ma, CL

Li, JG

AF Xia, Mingxu

Zhang, Shuguang

Ma, Chaoli

Li, Jianguo

TI Evaluation of glass-forming ability for metallic glasses based on

order-disorder competition

SO APPLIED PHYSICS LETTERS

AB The authors propose a calculable parameter epsilon, defined as a negative ratio of mixing entropy to mixing enthalpy, to evaluate the glass-forming ability (GFA) of metallic glasses from the viewpoint of a competition of disordering against ordering. It is found that the GFAs of most typical metallic glasses linearly distribute with epsilon and the likely formation area of bulk metallic glass could be conservatively restricted within 0.25 <epsilon < 0.6 K-1. The prediction of Tb-Fe-Al glassy alloys shows promise for rapidly locating glass formers with less cost. (c) 2006 American Institute of Physics.

OI xia, mingxu/0000-0002-3113-4795

SN 0003-6951

PD AUG 28

PY 2006

VL 89

IS 9

AR 091917

DI 10.1063/1.2345259

UT WOS:000240236600046

ER

PT S

AU Bhatt, J

Kumar, S

Murty, BS

AF Bhatt, Jatin

Kumar, S.

Murty, B. S.

BE Tan, Y

Ju, DY

TI Thermodynamic model and synthesis of Bulk Metallic Glass in Cu-Zr-Ti

system by Mechanical Alloying

SO ADVANCED MATERIAL SCIENCE AND TECHNOLOGY, PTS 1 AND 2

SE Materials Science Forum

CT 7th International Forum on Advanced Material Science and Technology

CY JUN 26-28, 2011

CL Dalian, PEOPLES R CHINA

SP Natl Nat Sci Fdn China, KC Wong Educ Fdn, Dalian Univ Technol, Changchun Res Inst Mech Sci Co Ltd

AB Based on the thermodynamic and topological approach, Cu60Zr30Ti10 has been identified as the best bulk metallic glass forming composition in Cu-Zr-Ti system. Bulk metallic glass has been successfully produced using mechanical alloying of elemental blends and consolidation of the resulting glassy powders into pellets of 8 mm diameter. Dry sliding wear of glassy pellets at different annealed states showed that the relaxed metallic glass has excellent wear resistance.

RI Sundaram, Kumar/A-4242-2013; Murty, BS/P-3354-2015

OI Sundaram, Kumar/0000-0003-1737-8758; Murty, BS/0000-0002-4399-8531

SN 0255-5476

PY 2011

VL 675-677

BP 189

EP +

DI 10.4028/www.scientific.net/MSF.675-677.189

UT WOS:000297036200044

ER

PT J

AU Vinogradov, A

Seleznev, M

Yasnikov, IS

AF Vinogradov, Alexei

Seleznev, Mikhail

Yasnikov, Igor S.

TI Dislocation characteristics of shear bands in metallic glasses

SO SCRIPTA MATERIALIA

AB Using a digital image correlation technique, we measured the spatial displacement distribution around the shear band tip terminated in a deformed bulk metallic glass. The excellent agreement is found between the experimentally observed and theoretically predicted displacement fields for dislocations, which provides a direct evidence for the dislocation-like behaviour of the shear bands in metallic glasses. (C) 2016 Acta Materialia Inc. Published by Elsevier Ltd. All rights reserved.

RI Yasnikov, Igor/F-1437-2014

OI Yasnikov, Igor/0000-0002-6120-7836

SN 1359-6462

PD MAR 15

PY 2017

VL 130

BP 138

EP 142

DI 10.1016/j.scriptamat.2016.11.017

UT WOS:000394194200030

ER

PT S

AU Li, JY

Zheng, ZZ

Wu, X

Li, JJ

AF Li, Jinyang

Zheng, Zhizhen

Wu, Xiao

Li, Jianjun

BE Qin, Y

Dean, TA

Lin, J

Yuan, SJ

Vollertsen, F

TI Micro-lateral extrusion of Zr55Cu30Al10Ni5 bulk metallic glass under

low-frequency vibration loading

SO 4TH INTERNATIONAL CONFERENCE ON NEW FORMING TECHNOLOGY (ICNFT 2015)

SE MATEC Web of Conferences

CT 4th International Conference on New Forming Technology (ICNFT)

CY AUG 06-09, 2015

CL Glasgow, ENGLAND

SP Univ Strathclyde, Univ Birmingham, Imperial Coll, Harbin Inst Technol, BIAS GmbH, Inst Mech Engineers, Engn & Phys Sci Res Council, Consortium UK Univ Mfg Engn Dept Heads, European Technol Platform Adv Engn Mat & Technol, European Nanotechnol Technol & Innovat Platform, China Soc Technol of Plastic, Arbeitsgemeinschaft Umformtechnik, AGU, Natl Key Lab Precis Hot Proc Metals, RITAI, Auto Standard Component Co Ltd, AP&T Grp, PAB Coventry, ESI Grp

AB The effect of vibration on the micro-forming ability of Zr55Cu30Al10Ni5 bulk metallic glass in its supercooled liquid region was studied. The experiment of microextrusion was carried out under different amplitude (38 similar to 760 N) and different frequency (0.1 similar to 2.0 Hz) at a fixed temperature of 723 K. The extrusion length was taken as a measure to characterize the micro-forming ability. Results reveal that the extrusion length of bulk metallic glass is effectively improved under vibration loading, and increases with increasing loading frequency and amplitude, whereas the frequency dependence is stronger. The viscosity of bulk metallic glass declines under vibration loading because of a larger free volume concentration and surface effect caused by vibration. This research indicates that the vibration forming is an effective method to enhance the micro-forming ability of bulk metallic glasses.

SN 2261-236X

BN 978-2-7598-1823-5

PY 2015

VL 21

AR 09007

DI 10.1051/matecconf/20152109007

UT WOS:000372800100093

ER

PT J

AU Zhou, BW

Zhang, XG

Zhang, W

Kimura, H

Makino, A

Inoue, A

AF Zhou, B. W.

Zhang, X. G.

Zhang, W.

Kimura, H.

Makino, A.

Inoue, A.

TI High glass forming ability and good mechanical properties of Cu-Zr-Al

bulk metallic glasses

SO MATERIALS RESEARCH INNOVATIONS

AB For developing bulk metallic glasses with high glass forming ability (GFA) and good mechanical properties, Cu92-xZrxAl8 (x=35-65 at-%) alloys have been studied by means of tilt mould casting method. The largest glassy rod with a critical diameter of 18 mm in this alloy series was obtained for Cu42Zr50Al8 alloy. The Hruby factor is appropriate for evaluating GFA in the cast Zr-Cu-Al bulk glassy alloy. The bulk metallic glasses with high GFA exhibit good mechanical properties, i.e. compressive fracture strength of 1783-2240 MPa, Young's modulus of 90-123 GPa and plastic strain of 0-0.73%.

RI Inoue, Akihisa/E-5271-2015; Kimura, Hisamichi/D-5449-2012; MAKINO,

AKIHIRO/B-2549-2009

SN 1432-8917

PD OCT

PY 2011

VL 15

IS 5

BP 310

EP 313

DI 10.1179/143307511X13109310554562

UT WOS:000295977600002

ER

PT J

AU Fu, J

Men, H

Pang, S

Ma, C

Zhang, T

AF Fu, Junying

Men, Hua

Pang, Shujie

Ma, Chaoli

Zhang, Tao

TI Formation and thermal stability of Cu-Zr-AI-Er bulk metallic glasses

with high glass-forming ability

SO JOURNAL OF UNIVERSITY OF SCIENCE AND TECHNOLOGY BEIJING

AB The formation and thermal stabilities of Cu46.25Zr46.25-xAl7.5Erx (x=0 to 8) bulk metallic glasses (BMGs) were investigated. The addition of a small amount of Er (2at%) for replacing Zr effectively improves the glass-forming ability of Cu46.25Zr46.25Al7.5 alloy, and the glassy rod with a diameter of at least 12 mm can be formed. The glass transition temperature (T-g), temperature interval of supercooled liquid region Delta T-x (=T-x-T-g), and reduced glass transition temperature T-rg (=T-g/T-l) of Cu46.25Zr44.25Al7.5Er2 glassy alloy are 699 K, 62 K and 0.607, respectively.

RI Pang, Shujie/D-8305-2016; Zhang, Tao/O-4911-2014

SN 1005-8850

PD JUN

PY 2007

VL 14

SU 1

BP 36

EP 38

DI 10.1016/S1005-8850(07)60104-2

UT WOS:000250593400009

ER

PT J

AU Kim, TS

Lee, JK

Kim, HJ

Bae, JC

AF Kim, TS

Lee, JK

Kim, HJ

Bae, JC

TI Consolidation of CU54Ni6Zr22Ti18 bulk amorphous alloy powders

SO MATERIALS SCIENCE AND ENGINEERING A-STRUCTURAL MATERIALS PROPERTIES

MICROSTRUCTURE AND PROCESSING

AB Spherical Cu54Ni6Zr22Ti18 metallic glass powders were prepared using a high-pressure gas atomizer, followed by consolidation using spark plasma sintering (SPS) process. The glass forming ability of both powders as atomized and bulks as SPSed was investigated by X-ray diffractometer (XRD) and differential scanning calorimeter (DSC) as a function of powder size distribution. The bulk maintains the amorphous structure even after the thermal consolidation. The compressive strength was increased as the powder size decreased due to an increased density. The compression tested BMG presents a fracture pattern occurred along the maximum shear plane declined similar to 45 degrees to the loading direction. (c) 2005 Elsevier B.V All rights reserved.

SN 0921-5093

PD AUG 15

PY 2005

VL 402

IS 1-2

BP 228

EP 233

DI 10.1016/j.msea.2005.04.044

UT WOS:000231552800030

ER

PT J

AU Qiao, JC

Yao, Y

Pelletier, JM

Keer, LM

AF Qiao, J. C.

Yao, Y.

Pelletier, J. M.

Keer, L. M.

TI Understanding of micro-alloying on plasticity in Cu46Zr47-xAl7Dyx (0 <=

x <= 8) bulk metallic glasses under compression: Based on mechanical

relaxations and theoretical analysis

SO INTERNATIONAL JOURNAL OF PLASTICITY

AB Lacking of plasticity at ambient temperature severely hinders the wide applications of bulk metallic glasses, and a significant challenge is to improve the plasticity. Based on the metallurgical physics, micro-alloying can be applied to adjust metallic glasses plasticity. In the current work, dynamic mechanical relaxation of Cu46Zr47-xAl7Dyx (0 <= x <= 8) bulk metallic glasses has been investigated experimentally by dynamic mechanical analysis. Compressive tests have been performed to investigate mechanical properties of the Cu based bulk metallic glasses at both ambient as well as cryogenic temperatures. The results indicated that by modifying the chemical composition, plastic deformation and dynamic mechanical relaxation processes are changed. The influence of Dysprosium (Dy) on plastic deformation is possibly related to the Johari-Goldstein (JG) relaxation in the metallic glasses. A kinetic model which may be predict the mechanical relaxation behavior and atomic mobility of the metallic glasses. In addition, experimental analyses show that thermal properties can be affected by the Dy addition of the Cu-based bulk metallic glasses. Our investigations demonstrated that micro-alloying of Dy could play an important role to influence the Cu46Zr47-xAl7Dyx bulk metallic glasses plasticity. In order to explain this behavior, the quasi-point defects theory was used to describe the micro structural heterogeneity. We postulate that the compressive plasticity is directly associated with local heterogeneity and relaxation modes for metallic glasses. (C) 2016 Elsevier Ltd. All rights reserved.

RI Yao, Yao/K-3129-2012

OI Yao, Yao/0000-0002-0879-4269

SN 0749-6419

EI 1879-2154

PD JUL

PY 2016

VL 82

BP 62

EP 75

DI 10.1016/j.ijplas.2016.02.002

UT WOS:000378190600004

ER

PT J

AU Wang, RJ

Li, FY

Wang, JF

Wang, WH

AF Wang, RJ

Li, FY

Wang, JF

Wang, WH

TI Responses of glassy structure and properties to pressure and

devitrification

SO APPLIED PHYSICS LETTERS

AB The pressure (up to 2 GPa) and devitrification-induced properties and structural changes in glasses studied by an ultrasonic method are summarized. The pressure has a larger effect on the longitudinal mode for a crystallized state and on the shear mode for a glassy state in oxide glasses. The crystallization significantly affects the longitudinal mode for oxide glasses and the shear mode for metallic glasses. The different effects are contributed to their different microstructures in short-range order for different glasses. (C) 2003 American Institute of Physics.

SN 0003-6951

PD OCT 6

PY 2003

VL 83

IS 14

BP 2814

EP 2816

DI 10.1063/1.1616200

UT WOS:000185664000028

ER

PT J

AU Wei, YX

Zhang, B

Wang, RJ

Pan, MX

Zhao, DQ

Wang, WH

AF Wei, YX

Zhang, B

Wang, RJ

Pan, MX

Zhao, DQ

Wang, WH

TI Erbium- and cerium-based bulk metallic glasses

SO SCRIPTA MATERIALIA

AB We report that the elastic constants of available bulk metallic glasses (BMGs) show a correlation with a weighted average of the elastic constants for the constituent elements. Based on the correlation, we report the formation of two families of erbium- and cerium-based BMGs with controllable elastic properties. (c) 2005 Published by Elsevier Ltd. on behalf of Acta Materialia Inc.

SN 1359-6462

PD FEB

PY 2006

VL 54

IS 4

BP 599

EP 602

DI 10.1016/j.scriptamat.2005.10.044

UT WOS:000234028400019

ER

PT J

AU Li, XL

Song, KK

Wu, YQ

Ji, H

Wang, L

AF Li, Xuelian

Song, Kaikai

Wu, Yuqin

Ji, Hong

Wang, Li

TI The mismatch entropy for bulk metallic glasses: A thermodynamic approach

SO MATERIALS LETTERS

AB Based on the thermodynamic fragility model, a new parameter (S-sigma/k(B))/M is proposed to evaluate the glass-forming ability (GFA) of metallic glasses, where M and S-sigma/k(B) are the fragility of the superheated melts and the mismatch entropy normalized by the Boltzmann constant, respectively. A positive relationship between (S-sigma/k(B))/M and glass forming ability is observed for both bulk metallic glasses and marginal metallic glasses, and an eutectic alloy with (S-sigma/k(B))/M larger than 0.17 can be selected as a bulk glassy forming candidate. As most alloys can obtain the temperature dependence of viscosity above liquidus temperature, (S-sigma/k(B))/M can be used as a prediction parameter for high GFA. Although both high and low M values can work in determining GFA, the high GFA of bulk metallic glasses is primarily due to the large mismatch entropy (S-sigma/k(B)>0.17). (C) 2013 Elsevier B.V. All rights reserved.

OI Song, Kaikai/0000-0002-5832-7546

SN 0167-577X

PD SEP 15

PY 2013

VL 107

BP 17

EP 19

DI 10.1016/j.matlet.2013.05.106

UT WOS:000323240300006

ER

PT J

AU Kiminami, CS

Lisboa, RDS

de Oliveira, MF

Bolfarini, C

Botta, WJ

AF Kiminami, C. S.

Lisboa, R. D. Sa

de Oliveira, M. F.

Bolfarini, C.

Botta, W. J.

TI Topological instability as a criterion for design and selection of easy

glass-former compositions in Cu-Zr based systems

SO MATERIALS TRANSACTIONS

CT 5th International Conference on Bulk Metallic Glasses

CY OCT 01-05, 2006

CL Osaka Univ, Awaji Isl, JAPAN

SP Minist Educ, Culture, Sports, Sci & Technol, Inst Mat Res, Tohoku Univ, Japan Soc Promot Sci, Natl Inst Mat Sci, Hyogo Int Assoc

HO Osaka Univ

AB In the present work we propose a new approach for predicting the best glass-former composition(s) in multi-component metallic glasses. By applying the lambda criterion. a topological instability criterion proposed to predict the crystallisation behaviour of Al-based systems, we show that it is also successfully possible to reproduce compositional ranges where binary and ternary bulk metallic glasses (BMGs) have recently been obtained. Our results indicate that the good glass-former composition(s) lie(s) within fields of mutual and simultaneous topological instability of all the crystalline phases competing with glassy phase.

RI de Oliveira, Marcelo/B-9881-2012; Botta, Walter/E-7763-2010; KIMINAMI,

CLAUDIO/D-4402-2012; Bolfarini, Claudemiro/E-4366-2012

OI de Oliveira, Marcelo/0000-0003-4589-2463; Botta,

Walter/0000-0003-2759-573X; KIMINAMI, CLAUDIO/0000-0001-8231-7316;

Bolfarini, Claudemiro/0000-0002-3099-3694

SN 1345-9678

EI 1347-5320

PD JUL

PY 2007

VL 48

IS 7

BP 1739

EP 1742

DI 10.2320/matertrans.MJ200745

UT WOS:000248743100033

ER

PT S

AU Kawamura, Y

Inoue, A

AF Kawamura, Y

Inoue, A

BE Sakuma, T

Aizawa, T

Higashi, K

TI Superplastic forming of Zr65Al10Ni10Cu15 metallic glass

SO TOWARDS INNOVATION IN SUPERPLASTICITY II

SE MATERIALS SCIENCE FORUM

CT 2nd International Conference on Towards Innovation in Superplasticity

CY SEP 21-24, 1998

CL KOBE, JAPAN

AB We have investigated the powder consolidation and bulk working by using the high-strain-rate superplasticity of a Zr65Al10Ni10Cu15 (at.%) metallic glass that has a wide supercooled liquid region of 105 K. The superplastic powder-consolidation process for producing bulk metallic glasses with full tensile strength has been established. The metallic glass, moreover, had an excellent workability and a wide working condition range. The successful extrusion conditions of temperature, ram-speed and pressure, which enable to work the metallic glass with retaining the glassy phase, have been established. The superplasticity inherent in the supercooled liquid seems to affect greatly the future development of bulk amorphous and nanocrystalline materials with novel properties.

RI Inoue, Akihisa/E-5271-2015

SN 0255-5476

BN 0-87849-828-1

PY 1999

VL 304-3

BP 373

EP 378

DI 10.4028/www.scientific.net/MSF.304-306.373

UT WOS:000082112300056

ER

PT J

AU Pan, DG

Zhang, HF

Wang, AM

Wang, ZG

Hu, ZQ

AF Pan, D. G.

Zhang, H. F.

Wang, A. M.

Wang, Z. G.

Hu, Z. Q.

TI Fracture instability in brittle Mg-based bulk metallic glasses

SO JOURNAL OF ALLOYS AND COMPOUNDS

AB A densely packed striation structure is observed in the apparently featureless mirror area of the fracture surface of brittle Mg-based bulk metallic glass. Taylor instability analysis based on grease model combined with a competitive microvoid formation mechanism ahead of a blunted crack tip suggests that the fracture of metallic glass is controlled by both viscous flow and ductile fracture mechanism. Spacing size of the striation is correlated with macroscopic fracture toughness. (C) 2006 Elsevier B.V. All rights reserved.

RI wang, am/P-2147-2016

SN 0925-8388

PD JUL 12

PY 2007

VL 438

IS 1-2

BP 145

EP 149

DI 10.1016/j.jallcom.2006.08.014

UT WOS:000247194900029

ER

PT J

AU Ishida, M

Uehara, T

Arai, T

Takeda, H

Yamaguchi, T

Taniguchi, T

Katsumi, T

Kobayashi, M

Ofune, H

AF Ishida, M

Uehara, T

Arai, T

Takeda, H

Yamaguchi, T

Taniguchi, T

Katsumi, T

Kobayashi, M

Ofune, H

TI Precision die-casting of optical MU/SC conversion sleeve

SO INTERMETALLICS

CT 2nd International Conference on Bulk Metallic Glasses

CY MAR 24-28, 2002

CL CHILUNG, TAIWAN

AB The performance of MU/SC conversion sleeve produced by bulk metallic glass (Zr55Al10Ni5Cu30) was examined. A precision die-casting method was applied to improve size accuracy. The size accuracy of the conversion sleeve produced by the precision die-casting method was +/-1 mum, and optical insertion loss (Li) was less than 0.3 dB for a standard value. The wear resistance of metallic glass is improved by surface oxidation treatment in air at 673 K. The MU/SC conversion sleeve produced from bulk metallic glass has superior characteristics for optical parts. (C) 2002 Elsevier Science Ltd. All rights reserved.

SN 0966-9795

PD NOV-DEC

PY 2002

VL 10

IS 11-12

BP 1259

EP 1263

AR PII S0966-9795(02)00162-0

DI 10.1016/S0966-9795(02)00162-0

UT WOS:000180002100030

ER

PT J

AU Wang, WH

Wang, RJ

Fan, GJ

Eckert, J

AF Wang, WH

Wang, RJ

Fan, GJ

Eckert, J

TI Formation and properties of Zr-(Ti, Nb)-Cu-Ni-Al bulk metallic glasses

SO MATERIALS TRANSACTIONS

CT Bulk Metallic Glasses Conference

CY SEP 24-28, 2000

CL SINGAPORE, SINGAPORE

AB The effect of composition variations and of B or Si addition on the glass forming ability and thermal stability of Zr-M-Cu-Ni-Al (M = Ti, Nb) bulk metallic glass-forming alloys have been investigated. The structural, acoustic, thermal and elastic properties of the bulk metallic glasses (BMGs) are studied by X-ray diffractmetery (XRD), differential scanning calorimetry (DSC) and ultrasonic method. A small amount of boron and silicon addition can significantly affects the GFA and crystallization behaviors and microstructural nature of the glass-forming alloys. The small changes of the density and acoustic velocities in the BMGs relative to its corresponding crystallized state indicate a similar electronic state and similar atomic interactions between the glassy and crystalline states.

SN 1345-9678

EI 1347-5320

PD APR

PY 2001

VL 42

IS 4

BP 587

EP 591

DI 10.2320/matertrans.42.587

UT WOS:000168864100009

ER

PT S

AU Jiang, W

Chen, QJ

Shen, J

Zhang, FB

Zhou, XL

Hua, XZ

AF Jiang, Wei

Chen, Qing-jun

Shen, Jun

Zhang, Fa-bi

Zhou, Xian-liang

Hua, Xiao-zhen

BE Zhang, KL

TI Salt Spray Corrosion Performance Associated with the Glass Forming

Ability of the FeCo-based Bulk Metallic Glasses

SO MATERIALS, MACHINES AND DEVELOPMENT OF TECHNOLOGIES FOR INDUSTRIAL

PRODUCTION

SE Applied Mechanics and Materials

CT International Conference on Advanced Nano-Technology and Biomedical

Material (ANTBM)

CY JUN 29-30, 2014

CL Guangzhou, PEOPLES R CHINA

AB For the bulk amorphous Fe24+xCo24-xCr15Mo14C15B6Y2(X= 0, 2, 4, 6 and 17) alloy, the corresponding corrosion properties associated with glass forming ability (GFA) have been carried out. Neutral salt spray corrosion test results show that the Fe28Co20Cr15Mo14C15B6Y2 alloy has the minimum corrosion rate, followed by Fe26Co22Cr15Mo14C15B6Y2, Fe24Co24Cr15Mo14C15B6Y2, Fe30Co18Cr15Mo14C15B6Y2, Fe41Co7Cr15Mo14C15B6Y2 and Ti6Al4V alloys. Specifically, the Fe28Co20Cr15Mo14C15B6Y2 alloy with the highest GFA also has the best corrosion resistance. With the increasing of Co addition, the corrosion resistance of the FeCo-based bulk metallic glasses is first increases and then decreases, which has the same trend of GFA with the change of Co elements. Furthermore, corrosion morphology are different for FeCo-based BMGs with different Co content.

OI Zhang, Fabi/0000-0002-4896-6854

SN 1660-9336

BN 978-3-03835-204-4

PY 2014

VL 618

BP 109

EP +

DI 10.4028/www.scientific.net/AMM.618.109

UT WOS:000348134100022

ER

PT J

AU Wei, BC

Zhang, Y

Zhuang, YX

Zhao, DQ

Pan, MX

Wang, WH

Hu, WR

AF Wei, BC

Zhang, Y

Zhuang, YX

Zhao, DQ

Pan, MX

Wang, WH

Hu, WR

TI Nd65Al10Fe25-xCox (x=0,5,10) bulk metallic glasses with wide supercooled

liquid regions

SO JOURNAL OF APPLIED PHYSICS

AB Bulk metallic glasses of Nd65Al10Fe25-xCox (x=0,5,10) have been prepared in the form of 3 mm diam rods. Results of differential scanning calrimetry, dynamic mechanical thermal analysis (DMTA), and x-ray diffraction are presented for these alloys. It is shown that the glass transition and crystallization have been observed by DMTA. The reduced glass transition temperature of these glasses, defined as the ratio between the glass transition temperature T-g and the melting temperature T-l is in the range from 0.55 to 0.62. All these glasses have a large supercooled liquid region (SLR), ranging from 80 to 130 K. The high value of reduced glass transition temperature and wide SLR agree with their good glass formation ability. (C) 2001 American Institute of Physics.

RI ZHANG, Yong/B-7928-2009; Zhuang, Yanxin/F-7199-2011

OI ZHANG, Yong/0000-0002-6355-9923;

SN 0021-8979

PD MAR 15

PY 2001

VL 89

IS 6

BP 3529

EP 3531

DI 10.1063/1.1347951

UT WOS:000167248100075

ER

PT J

AU Yang, GN

Li, Z

Guo, M

Luo, Y

Han, ZD

Lu, ZC

Wei, JQ

Shao, Y

Yao, KF

AF Yang, G. N.

Li, Z.

Guo, M.

Luo, Y.

Han, Z. D.

Lu, Z. C.

Wei, J. Q.

Shao, Y.

Yao, K. F.

TI Size effect in Pd77.5Cu6Si16.5 metallic glass micro-wires: More

scattered strength with decreasing diameter

SO APPLIED PHYSICS LETTERS

AB A size effect of more scattered strength with decreasing diameter is revealed by tension experiments on melt-spinning fabricated Pd77.5Cu6Si16.5 metallic glass microwires, and is explained from a perspective of structural inhomogeneity and a higher structure sensitivity of metallic glasses under tension condition and in a large aspect ratio. Such a result differs from the understanding of "the smaller the stronger" in the compression behaviors of metallic glasses, but indicates that the shear bands in metallic glasses actually could nucleate in a wide stress range. This finding could provide experimental evidence for the inhomogeneous structure and size effect in metallic glasses at the micro-scale, and could help the further study of their mechanical behaviors and substantial deformation mechanism. Published by AIP Publishing.

RI Wei, Jinquan/D-8925-2014

OI Wei, Jinquan/0000-0002-5827-2231; Shao, Yang/0000-0001-5369-9933

SN 0003-6951

EI 1077-3118

PD JUL 3

PY 2017

VL 111

IS 1

AR 011905

DI 10.1063/1.4991849

UT WOS:000405083600017

ER

PT J

AU Lin, JG

Wang, XF

Wen, C

AF Lin, J. G.

Wang, X. F.

Wen, C.

TI Theoretical study on behaviour of superplastic forming/diffusion bonding

of bulk metallic glasses

SO MATERIALS SCIENCE AND TECHNOLOGY

AB Based on the behaviour of the superplastic deformation of metallic glasses and Pilling's model, a diffusion bonding model suitable for metallic glasses is proposed in the present study. In the current model, the diffusion bonding processes consists of two stages: one is the plastic deformation stage and the other is the void shrinkage stage, in which, the atom diffusion and superplastic deformation are responsible for the void shrinkage. Applying this model to the diffusion bonding of a Zr based metallic glass, the predicted bonding time is in good agreement with the experimental result. A map for determining the bonding temperature and time to achieve high quality bonding in a Zr based metallic glass is suggested.

RI WANG, XF/B-6708-2009

OI Wen, Cuie/0000-0001-8008-3536

SN 0267-0836

PD MAR

PY 2010

VL 26

IS 3

BP 361

EP 366

DI 10.1179/174328408X393431

UT WOS:000275033000016

ER

PT J

AU Wang, YM

Shek, CH

Qiang, JB

Wong, CH

Wang, Q

Zhang, XF

Dong, C

AF Wang, YM

Shek, CH

Qiang, JB

Wong, CH

Wang, Q

Zhang, XF

Dong, C

TI The e/a criterion for the largest glass-forming abilities of the

Zr-Al-Ni(Co) alloys

SO MATERIALS TRANSACTIONS

CT International Symposium on Bulk Glassy Alloys

CY OCT, 2003

CL Yokohama, JAPAN

AB Composition optimization for the largest glass-forming ability has been performed in the Zr-Al-Ni(Co) systems in this investigation. Two guiding criteria, termed respectively the e/a-constant criterion and the e/a-variant criterion, are applied. They are incarnated into the e/a 1.5 composition line and the Zr9Ni(Co)(4)-Al composition line. Bulk metallic glasses are obtained by suction casting alloy melts of compositions within an e/a span of 1.3similar to1.5. with their thermal stabilities and glass forming abilities being increased with increasing e/a. The intersecting point of the two lines gives the composition Zr53Al23.5Ni(Co)(23.5) with the largest glass-forming ability.

RI SHEK, Chan Hung/J-3857-2015

OI SHEK, Chan Hung/0000-0002-6870-523X

SN 1345-9678

EI 1347-5320

PD APR

PY 2004

VL 45

IS 4

BP 1180

EP 1183

DI 10.2320/matertrans.45.1180

UT WOS:000221314900036

ER

PT J

AU Dun, CC

Liu, HS

Hou, L

Xue, L

Dou, LT

Yang, WM

Zhao, YC

Shen, BL

AF Dun, Chaochao

Liu, Haishun

Hou, Long

Xue, Lin

Dou, Lintao

Yang, Weiming

Zhao, Yucheng

Shen, Baolong

TI Ductile Co-Nb-B bulk metallic glass with ultrahigh strength

SO JOURNAL OF NON-CRYSTALLINE SOLIDS

AB A ternary Co61Nb8B31 bulk metallic glass with plasticity of 5% and yield strength of 5200 MPa was fabricated. It is shown that the primary crystallization product of the Co61Nb8B31 glass is the vertex- and edge-sharing Co2Nb type phase. The enhanced plastic strain is consistent with the observed multiple shear bands that can redistribute the internal shear stress. The appreciable plastic strain favors the formation of metallic bonding network-like structure and holds high Poisson's ratio. (c) 2013 Elsevier B.V. All rights reserved.

SN 0022-3093

EI 1873-4812

PD FEB 15

PY 2014

VL 386

BP 121

EP 123

DI 10.1016/j.jnoncrysol.2013.12.004

UT WOS:000332595300020

ER

PT J

AU Yoo, BG

Choi, IC

Kim, YJ

Ramamurty, U

Jang, JI

AF Yoo, Byung-Gil

Choi, In-Chul

Kim, Yong-Jae

Ramamurty, Upadrasta

Jang, Jae-il

TI Room-temperature anelasticity and viscoplasticity of Cu-Zr bulk metallic

glasses evaluated using nanoindentation

SO MATERIALS SCIENCE AND ENGINEERING A-STRUCTURAL MATERIALS PROPERTIES

MICROSTRUCTURE AND PROCESSING

AB Anelastic and viscoplastic characteristics of Cu50Zr50 and Cu65Zr35 binary bulk metallic glasses at room temperature were examined through nanoindentation creep experiments. Results show that both the deformations are relatively more pronounced in Cu50Zr50 than in Cu65Zr35, and their amount increases with the loading rate. The results are analyzed in terms of the influences of structural defects and loading rate on the room temperature indentation creep. (C) 2013 Elsevier B.V. All rights reserved.

RI Jang, Jae-il/A-3486-2011; Ramamurty, Upadrasta/E-5623-2011; Choi,

In-Chul/E-1499-2014

OI Jang, Jae-il/0000-0003-4526-5355;

SN 0921-5093

PD AUG 10

PY 2013

VL 577

BP 101

EP 104

DI 10.1016/j.msea.2013.04.031

UT WOS:000320837500014

ER

PT J

AU Xi, XK

Li, S

Wang, RJ

Zhao, DQ

Pan, MX

Wang, WH

AF Xi, XK

Li, S

Wang, RJ

Zhao, DQ

Pan, MX

Wang, WH

TI Bulk scandium-based metallic glasses

SO JOURNAL OF MATERIALS RESEARCH

AB The novel rare-earth scandium-based bulk metallic glasses (BMGs) are obtained by the copper mold casting method. Compared with other rare-earth BMGs reported so far, the Sc-based BMGs exhibit the highest elastic moduli (e.g., Young's modulus, E = 85 GPa; bulk modulus, B = 77.5 GPa), glass transition temperature (T-g = 62 K), and crystallization temperature (T-x = 760 K) combined with a large region of supercooled liquid (Delta Tau = 98 K). A good correlation between glass transition temperature and elastic moduli is found in a variety of rare-earth-based BMGs.

SN 0884-2914

PD SEP

PY 2005

VL 20

IS 9

BP 2243

EP 2247

DI 10.1557/JMR.2005.0281

UT WOS:000231648900001

ER

PT J

AU Li, YS

Fu, QQ

Wu, ZP

Dong, DQ

AF Li Ye-sheng

Fu Qun-qiang

Wu Zi-ping

Dong Ding-quin

TI Effect of addition of Zn and Al elements on glass-forming ability and

thermal stability of Mg-Cu-Y bulk metallic glasses

SO TRANSACTIONS OF NONFERROUS METALS SOCIETY OF CHINA

CT International Conference of Nonferrous Materials (ICNFM)

CY NOV 25-30, 2007

CL Changsha, PEOPLES R CHINA

SP China Nonferrous Met Ind Assoc, Cent S Univ

AB After substituting partial Cu and Mg with Zn or Al elements for Mg65Cu25Y10 alloy, respectively, the metallic glass plate samples with thickness of 2-3 min were prepared by water-quenching, their respective glass-forming ability and thermal stability were studied by using differential thermal analysis (DTA) and X-ray diffraction (XRD). Using Kissinger equation, the activation energies of crystallization of these metallic glasses heated with a constant rate were calculated. The results show that Al element is greatly harmful to the glass-forming ability of Mg-Cu-Y alloys and cannot acquire bulk amorphous alloys; nevertheless, the effect of Zn element addition is indeterminate for various components. The magnitudes of thermal stability are also revealed.

SN 1003-6326

PD NOV

PY 2007

VL 17

SI 1

BP S1094

EP S1098

PN B

UT WOS:000251737400112

ER

PT J

AU Wang, L

Chao, YS

AF Wang, Li

Chao, Yuesheng

TI Corrosion behavior of Fe41Co7Cr15Mo14C15B6Y2 bulk metallic glass in NaCl

solution

SO MATERIALS LETTERS

AB The electrochemical corrosion behaviors of Fe41Co7Cr15Mo14C15B6Y2 bulk metallic glasses (BMGs) were investigated by immersion tests and potentiodynamic polarization experiments. The results of immersion test in 1 N HCl, 1 N HNO3, 1 N NaOH and 3.5% NaCl solutions show that the corrosion rate of Fe41Co7Cr15Mo14C15B6Y2 metallic glasses is very low in all kinds of solutions, particularly in 3.5% NaCl solution. It indicates that the corrosion resistance in NaCl solution is superior to the other three solutions. The corrosion rate in 3.5% NaCl solution is much lower than those of other Fe-based amorphous alloys. Moreover, Fe41Co7Cr15Mo14C15B6Y2 metallic glass has excellent corrosion resistance in different concentrations of NaCl solutions through the further polarization experiments. (C) 2011 Elsevier B.V. All rights reserved.

SN 0167-577X

PD FEB 15

PY 2012

VL 69

BP 76

EP 78

DI 10.1016/j.matlet.2011.10.116

UT WOS:000300190300023

ER

PT J

AU Wang, WH

Dong, C

Shek, CH

AF Wang, WH

Dong, C

Shek, CH

TI Bulk metallic glasses

SO MATERIALS SCIENCE & ENGINEERING R-REPORTS

AB Amorphous alloys were first developed over 40 years ago and found applications as magnetic core or reinforcement added to other materials. The scope of applications is limited due to the small thickness in the region of only tens of microns. The research effort in the past two decades, mainly pioneered by a Japanese- and a US-group of scientists, has substantially relaxed this size constrain. Some bulk metallic glasses can have tensile strength up to 3000 MPa with good corrosion resistance, reasonable toughness, low internal friction and good processability. Bulk metallic glasses are now being used in consumer electronic industries, sporting goods industries, etc. In this paper, the authors reviewed the recent development of new alloy systems of bulk metallic glasses. The properties and processing technologies relevant to the industrial applications of these alloys are also discussed here. The behaviors of bulk metallic glasses under extreme conditions such as high pressure and low temperature are especially addressed in this review. In order that the scope of applications can be broadened, the understanding of the glass-forming criteria is important for the design of new alloy systems and also the processing techniques. (C) 2004 Elsevier B.V. All rights reserved.

RI SHEK, Chan Hung/J-3857-2015

OI SHEK, Chan Hung/0000-0002-6870-523X

SN 0927-796X

PD JUN 1

PY 2004

VL 44

IS 2-3

BP 45

EP 89

DI 10.1016/j.mser.2004.03.001

UT WOS:000223068800001

ER

PT J

AU Mattern, N

Kuhn, U

Hermann, H

Roth, S

Vinzelberg, H

Eckert, J

AF Mattern, N

Kuhn, U

Hermann, H

Roth, S

Vinzelberg, H

Eckert, J

TI Thermal behavior and glass transition of Zr-based bulk metallic glasses

SO MATERIALS SCIENCE AND ENGINEERING A-STRUCTURAL MATERIALS PROPERTIES

MICROSTRUCTURE AND PROCESSING

CT 11th International Conference on Rapidly Quenched and Metastable

Materials

CY AUG 25-30, 2002

CL Univ Oxford, Dept Mat, Oxford, ENGLAND

HO Univ Oxford, Dept Mat

AB The thermal behaviour of Zr-based bulk metallic glasses has been investigated in situ through the glass transition by means of differential scanning calorimetry, high temperature X-ray synchrotron diffraction, electrical resistivity, and dilatometry. The temperature dependence of the X-ray structure factor can be well described by the Debye theory. The Debye temperature of the glassy and the supercooled liquid state Of Zr52Ti5Cu18Ni15Al10 is theta = 412 and theta = 162 K, respectively. The temperature coefficient of the electrical resistivity and the thermal expansion coefficient change also at the calorimetric glass transition temperature. The results point to a significant changes in the dynamics of molecular motion at T-g. (C) 2003 Elsevier B.V. All rights reserved.

SN 0921-5093

EI 1873-4936

PD JUL 15

PY 2004

VL 375

SI SI

BP 351

EP 354

DI 10.1016/j.msea.2003.10.125

UT WOS:000223329700058

ER

PT J

AU Liu, XR

Hong, SM

Lu, SJ

Shen, R

AF Liu, X. R.

Hong, S. M.

Lue, S. J.

Shen, R.

TI Preparation of La68Al10Cu20Co2 bulk metallic glass by rapid compression

SO APPLIED PHYSICS LETTERS

AB Melt of La68Al10Cu20Co2 alloy was solidified by rapid compression within 20 ms to 5.5 GPa at 733 K. The structural analysis demonstrates that the recovered alloy is a fully glassy structure indicating that the method is an effective way for preparing bulk metallic glass. Differential scanning calorimetry analysis indicates the glass-forming ability of the alloy is enhanced according to the reduced glass transition temperature. It is found that exist markedly differences in thermodynamic and mechanical properties between the metallic glasses prepared by melt quenching and rapid compression.

SN 0003-6951

PD AUG 20

PY 2007

VL 91

IS 8

AR 081910

DI 10.1063/1.2773751

UT WOS:000248984800036

ER

PT J

AU Das, J

Kim, KB

Xu, W

Wei, BC

Zhang, ZF

Wang, WH

Yi, S

Eckert, J

AF Das, J.

Kim, K. B.

Xu, W.

Wei, B. C.

Zhang, Z. F.

Wang, W. H.

Yi, S.

Eckert, J.

TI Ductile metallic glasses in supercooled martensitic alloys

SO MATERIALS TRANSACTIONS

AB We report ductile bulk metallic glasses based on martensitic alloys. The slowly cooled specimens contain a mixture of parent 'austenite' and martensite phase. The slightly faster cooled bulk metallic glasses with 2-5 nm sized 'austenite'-like crystalline cluster reveal high strength and large ductility (16%). Shear bands propagate in a slither mode in this spatially inhomogeneous glassy structure and undergo considerable 'thickening' from 5-25 nm. A 'stress induced displacive transformation' is proposed to be responsible for both plasticity and work-hardening-like behavior of these 'M-Glasses'.

RI Zhang, Zhefeng/A-9732-2010; Zhang, BMG/C-6151-2014; Das,

Jayanta/G-1559-2010; Xu, Wei/G-6302-2012

OI Das, Jayanta/0000-0001-8750-5463;

SN 1345-9678

EI 1347-5320

PD OCT

PY 2006

VL 47

IS 10

BP 2606

EP 2609

DI 10.2320/matertrans.47.2606

UT WOS:000242429300027

ER

PT J

AU Qiao, JC

Pelletier, JM

AF Qiao, J. C.

Pelletier, J. M.

TI Enthalpy relaxation in Cu46Zr45Al7Y2 and Zr55Cu30Ni5Al10 bulk metallic

glasses by differential scanning calorimetry (DSC)

SO INTERMETALLICS

AB Structural relaxation process in Cu46Zr45Al7Y2 and Zr55Cu30Ni5Al10 bulk metallic glasses during annealing below the glass transition temperature T-g was investigated by differential scanning calorimetry (DSC) The features of enthalpy relaxation are sensitive to both annealing temperature and annealing time For a given annealing time t(a) the results indicated that the relaxation time t(a) decreases with increasing the annealing temperature T-a in good agreement with results relative to other bulk metallic glasses Additionally the enthalpy relaxation behaviour of the bulk metallic glasses appears independent on the cooling rate used before the physical aging experiments i e on the initial as-cast state The recovered enthalpy evolution of the bulk metallic glasses is well described by the Kohlrausch-Williams-Watts (KWW) exponential relaxation function as Delta H(T-a) = Delta H-eq{1 - exp[-(t(a)/iota)(beta)]} Kohlrausch exponent beta and enthalpy relaxation time iota are sensitive to the composition of the bulk metallic glasses Finally the influence of different heating treatment processes on the enthalpy relaxation in the bulk metallic glasses is presented and shows that this phenomenon is mainly reversible The structural relaxation behaviour is Interpreted by free volume model and quasi-point defects model Kinetic fragility parameters m in Cu46Zr45Al7Y2 and Zr55Cu30Ni5Al10 bulk metallic glasses are 72 and 69 respectively indicating therefore that these alloys are intermediate glasses

Crystallization process was also investigated by DSC experiments According to the Kissinger model corresponding activation energy is 3 18 eV in Cu46Zr45Al7Y2 and 3 19 eV in Zr55Cu30Ni5Al10 respectively (C) 2010 Elsevier Ltd All rights reserved

SN 0966-9795

EI 1879-0216

PD JAN

PY 2011

VL 19

IS 1

BP 9

EP 18

DI 10.1016/j.intermet.2010.08.042

UT WOS:000285122300002

ER

PT J

AU Duan, G

Wiest, A

Lind, ML

Kahl, A

Johnson, WL

AF Duan, Gang

Wiest, Aaron

Lind, Mary Laura

Kahl, Annelen

Johnson, William L.

TI Lightweight Ti-based bulk metallic glasses excluding late transition

metals

SO SCRIPTA MATERIALIA

AB Lightweight Ti-based bulk amorphous structural metals with more than double the specific strength of conventional titanium alloys have been discovered. Thermal, elastic and mechanical properties of these metallic glasses were studied and are presented. These amorphous alloys exhibit good glass-forming ability, exceptional thermal stability and high strength. The research results have important implications for designing and developing low-density bulk metallic glasses. The technological potential of this class of lightweight Ti-based glassy alloys as structural metals is very promising. (C) 2007 Acta Materialia Inc. Published by Elsevier Ltd. All rights reserved.

RI Duan, Gang/B-3188-2009

OI Lind, Mary/0000-0001-8585-8054

SN 1359-6462

PD MAR

PY 2008

VL 58

IS 6

BP 465

EP 468

DI 10.1016/j.scriptamat.2007.10.040

UT WOS:000253649800011

ER

PT J

AU Shen, TD

Xin, SW

Sun, BR

AF Shen, T. D.

Xin, S. W.

Sun, B. R.

TI Low power loss in Fe65.5Cr4Mo4Ga4P12B5.5C5 bulk metallic glasses

SO JOURNAL OF ALLOYS AND COMPOUNDS

AB Power loss - one of the important application-oriented magnetic properties of ferromagnetic metallic glasses - has been well studied in glassy ribbons rather than bulk metallic glasses. This paper studies the influence of frequency, induction, annealing, and specimen thickness on the total power loss - which is divided into hysteresis loss, classical eddy current loss, and excess eddy current loss - of bulk ferromagnetic Fe65.5Cr4Mo4Ga4P12B5.5C5 glasses. The total power loss of bulk glasses increases with frequency and peak induction and follows a power relation, similar to what has been observed in glassy ribbons. Annealing decreases both the hysteresis loss and the classical eddy current loss, resulting in a lower total power loss. The ratio of excess eddy current loss to classical eddy current loss deceases with increasing specimen thickness. The excess eddy current loss is negligible for our thickest glass and comparable to the classical eddy current loss for our thinner glasses. In contrast, the excess eddy current loss is often at least one to two orders of magnitude greater than the classical eddy current loss in glassy ribbons. The low total power loss achieved in bulk ferromagnetic glasses should be beneficial to their practical applications in energy conversion devices. (C) 2015 Elsevier B.V. All rights reserved.

OI Shen, Tongde/0000-0002-0391-3361

SN 0925-8388

EI 1873-4669

PD FEB 15

PY 2016

VL 658

BP 703

EP 708

DI 10.1016/j.jallcom.2015.10.260

UT WOS:000366940100097

ER

PT J

AU Yan, M

Kohara, S

Wang, JQ

Nogita, K

Schaffer, GB

Qian, M

AF Yan, M.

Kohara, S.

Wang, J. Q.

Nogita, K.

Schaffer, G. B.

Qian, M.

TI The influence of topological structure on bulk glass formation in

Al-based metallic glasses

SO SCRIPTA MATERIALIA

AB Synchrotron high-energy X-ray diffraction has been used to determine the atomic structural details in three selected melt-spun Al-based metallic glasses with different glass-forming abilities (GFAs), Al86Ni7Y4.5Co1La1.5, Al86Ni6Y6Co2 and Al84Ni9Y7. The atomic pair species, pair fractions and their corresponding bond distances have been established for each metallic glass. These results address the GFA of Al-based metallic glasses from the topological perspective, and explain the structural origins that are responsible for the excellent GFA of Al86Ni7Y4.5Co1La1.5. (C) 2011 Acta Materialia Inc. Published by Elsevier Ltd. All rights reserved.

SN 1359-6462

PD NOV

PY 2011

VL 65

IS 9

BP 755

EP 758

DI 10.1016/j.scriptamat.2011.07.009

UT WOS:000295765300003

ER

PT S

AU Okai, D

Inoue, M

Mori, T

Fukami, T

Kobayashi, E

Yamasaki, T

Kimura, HM

Inoue, A

AF Okai, D.

Inoue, M.

Mori, T.

Fukami, T.

Kobayashi, E.

Yamasaki, T.

Kimura, H. M.

Inoue, A.

BE Schultz, L

Eckert, J

Battezzati, L

Stoica, M

TI Static Mechanical Properties for Ca48Mg27Cu25 Bulk Metallic Glass by

Ultrasonic Velocity Measurement

SO 13TH INTERNATIONAL CONFERENCE ON RAPIDLY QUENCHED AND METASTABLE

MATERIALS

SE Journal of Physics Conference Series

CT 13th International Conference on Rapidly Quenched and Metastable

Materials

CY AUG 24-29, 2008

CL Dresden, GERMANY

AB The static mechanical properties of a Ca48Mg27Cu25 bulk metallic glass were investigated using a technique of ultrasonic measurement and compressive test. The Young's modulus (E), Poisson's ratio (v), shear modulus (G) and bulk modulus (B) for the Ca48Mg27Cu25 alloy at room temperature are significantly smaller than those for Zr- and Pd-based bulk metallic glasses. The values of E, v, G and B for the Ca48Mg27Cu25 alloy are 29.8GPa, 0.230, 12.1GPa and 18AGPa, respectively. The results of compression test for the Ca48Mg27Cu25 alloy have been also described.

SN 1742-6588

PY 2009

VL 144

AR 012029

DI 10.1088/1742-6596/144/1/012029

UT WOS:000271827100029

ER

PT S

AU Aniya, M

AF Aniya, Masaru

BE Chandra, T

Ionescu, M

Mantovani, D

TI Elastic Constants, Equation of State and Mechanical Relaxations of Some

Metallic Glasses at High Pressure

SO THERMEC 2011, PTS 1-4

SE Materials Science Forum

CT 7th International Conference on Processing and Manufacturing of Advanced

Materials

CY AUG 01-05, 2011

CL Quebec City, CANADA

SP Minerals, Metals & Mat Soc

AB One of the fundamental physical quantities necessary to describe the mechanical properties of the materials is the bulk modulus. In the present report, a simple method to estimate the values of the bulk modulus and its pressure derivative of metallic glasses is presented. The method which is based on a jellium model of metals provides a good agreement with measured data. The estimated values of the elastic constants have been used to determine the equation of state of bulk metallic glasses. It is found that the usual Mumaghan equation of state deviates considerably from the experimental results at high pressures. The deviation has been interpreted to arise from the structural relaxations. The effect of pressure on the fragility of bulk metallic glasses is discussed briefly.

SN 0255-5476

PY 2012

VL 706-709

BP 1305

EP 1310

DI 10.4028/www.scientific.net/MSF.706-709.1305

UT WOS:000308517300214

ER

PT J

AU Wang, YF

Wu, J

Wang, Q

Qiang, JB

Dong, C

AF Wang, Yanfang

Wu, Jiang

Wang, Qing

Qiang, Jianbing

Dong, Chuang

TI The bulk metallic glass formation in Zr-Al-Ni-Ti quaternary system

SO MATERIALS LETTERS

AB The optimum metallic glass compositions are located close to the intersecting line of the e/a-constant and atomic size-constant planes in a quaternary composition chart. According to these criteria, a series of Zr-Al-Ni-Ti quaternary alloys have been designed and prepared by suction casting. The electron concentration and average atomic size of two ternary glass forming alloys, Zr60Al20Ni20 and Zr53Al23.5Ni23.5, have been used. Pure glass state is reached only within a small composition range of Ti. Ti addition deteriorates the thermal stabilities, but maintains the same glass forming abilities of bulk metallic glasses. (c) 2006 Elsevier B.V. All rights reserved.

SN 0167-577X

PD APR

PY 2007

VL 61

IS 10

BP 2066

EP 2070

DI 10.1016/j.matlet.2006.08.017

UT WOS:000245760900029

ER

PT S

AU Zhang, QS

Zhang, W

Louzguine-Luzgin, DV

Inoue, A

AF Zhang, Q. S.

Zhang, W.

Louzguine-Luzgin, D. V.

Inoue, A.

BE Nie, JF

Morton, A

TI High Glass-Forming Ability and Unusual Deformation Behavior of New

Zr-Cu-Fe-Al Bulk Metallic Glasses

SO PRICM 7, PTS 1-3

SE Materials Science Forum

CT 7th Pacific Rim International Conference on Advanced Materials and

Processing

CY AUG 02-06, 2010

CL Cairns, AUSTRALIA

SP Chinese Soc Metals, Japan Inst Metals, Korean Inst Metals & Mat, Mat Australia, Minerals, Met & Mat Soc

AB A new series of bulk metallic glasses were developed by addition of Fe into the ternary Zr60Cu30Al10 alloy. Although Fe-Cu element pair shows distinct immiscibility with a large positive heat of mixing, substitution of Fe for Cu significantly improves the glass-forming ability of the ternary Zr60Cu30Al10 alloy. The critical diameter for glass-formation increases from 8 mm for Zr60Cu30Al10 alloy to 20 mm for Zr60Cu25Fe5Al10 and Zr62.5Cu22.5Fe5Al10 alloys. As compared with the ternary Zr60Cu30Al10 alloy, the new quaternary Zr-Cu-Fe-Al alloys show lower liquidus temperatures. The Zr60Cu25Fe5Al10 and Zr62.5Cu22.5Fe5Al10 alloys, the best BMG-formers in this alloy system, are found to locate very near a Zr-Cu-Fe-Al eutectic point. The new Zr-Fe-Cu-Al bulk metallic glasses exhibit high strength of about 1700 MPa. The plastic strain increases from 7.8% to 11.3% with increasing the content of Fe from 0 to 12.5%. The finding of a Ni-free Zr-based bulk glassy alloy with the extremely high glass-forming ability is expected to extend the future application of bulk metallic glasses.

RI Inoue, Akihisa/E-5271-2015

OI Louzguine-Luzgin, Dmitri/0000-0001-5716-4987

SN 0255-5476

PY 2010

VL 654-656

BP 1042

EP +

DI 10.4028/www.scientific.net/MSF.654-656.1042

PN 1-3

UT WOS:000285374600255

ER

PT J

AU Yan, M

Shen, J

Zhang, T

Zou, J

AF Yan, M.

Shen, J.

Zhang, T.

Zou, J.

TI Enhanced glass-forming ability of a Zr-based bulk metallic glass with

yttrium doping

SO JOURNAL OF NON-CRYSTALLINE SOLIDS

AB A significant enhancement in glass formation in a newly developed Zr51Cu20.7Ni12Al16.3 alloy has been achieved by yttrium doping. With just 0.5 at.% yttrium doping, the critical diameter of the as-cast alloys for glass formation has been increased from 3 mm to at least 10 mm. In the undoped, large-sized alloys, massive oxygen stabilized crystalline phases are observed but disappear in yttrium doped alloys. Very small amounts of stable alpha-Y2O3 phases found in the yttrium doped alloys, and their negligible effect on the metallic glasses' properties, provide a superior solution to achieve metallic glasses with a high glass formability. (c) 2006 Elsevier B.V. All rights reserved.

RI Zhang, Tao/O-4911-2014; Zou, Jin/B-3183-2009

OI Zou, Jin/0000-0001-9435-8043

SN 0022-3093

PD AUG 15

PY 2006

VL 352

IS 28-29

BP 3109

EP 3112

DI 10.1016/j.jnonerysol.2006.02.098

UT WOS:000239375000025

ER

PT J

AU Zhang, G

Liu, J

Zeng, Q

Wang, L

Liu, J

Jiang, J

AF Guo-qing, Zhang

Liu Jin-fang

Zeng Qiao-shi

Wang Li-na

Liu Jin-qiang

Jiang Jian-zhong

TI Effect of Nb in (La0.5Ce0.5)(64-x)Al16Ni5Cu15Nbx (x=0-5) bulk metallic

glasses

SO TRANSACTIONS OF NONFERROUS METALS SOCIETY OF CHINA

CT 6th International Workshop on Advanced Intermetallic and Metallic

Materials

CY OCT 09-15, 2005

CL Yangzhou, PEOPLES R CHINA

SP Natl Nat Sci Fdn China, Minist Sci & Technol China, Nanjing Univ Sci & Technol, Univ Sci & Technol Beijing, Oak Ridge Natl Lab, Shenyang Natl Lab Mat Sci, Cent S Univ, Harbin Inst Technol, Yangzhou City Govt

AB The effect of Nb in (La0.5Ce0.5)(64-x)Al16Ni5Cu15Nbx (x=0-5, mole fraction) bulk metallic glasses was investigated by X-ray diffractometry, differential scanning calorimetry, and scanning electron microscopy. Fully amorphous rods up to 5 mm in diameter were obtained using copper mold. Their lower glass transition temperatures are of about 401-407 K and wide supercooled liquid regions are up to 75 K. The oxidation resistance of the LaCe-based glassy alloys can be largely enhanced by adding tiny Nb, which makes the developed LaCe-based bulk metallic glasses more attractive for potential industrial applications.

RI Zeng, Qiaoshi/I-8688-2012

OI Zeng, Qiaoshi/0000-0001-5960-1378

SN 1003-6326

EI 2210-3384

PD SEP

PY 2006

VL 16

SI 2

BP S131

EP S135

UT WOS:000241774000029

ER

PT S

AU Haruyama, O

Wada, R

Kohda, M

Yokoyama, Y

Nishiyama, N

Egami, T

AF Haruyama, O.

Wada, R.

Kohda, M.

Yokoyama, Y.

Nishiyama, N.

Egami, T.

BE Chandra, T

Wanderka, N

Reimers, W

Ionescu, M

TI Inhomogeneous Amorphous Structure Of Bulk Metallic Glasses Examined From

Structural Relaxation Kinetics

SO THERMEC 2009, PTS 1-4

SE Materials Science Forum

CT 6th International Conference on Processing and Manufacturing of Advanced

Materials (THERMEC)/2nd Symposium Session on Multiscale Mechanical

Modelling of Complex Materials and Engineering Applications

CY AUG 25-29, 2009

CL Berlin, GERMANY

SP Minerals, Met & Mat Soc (TMS)

AB The kinetics of structural relaxation in fragile glass former, Pd46Cu35 5P18 5 BMG, and strong glass former, Zr50Cu40Al10 BMG, was investigated by volume relaxation The former exhibited a relaxation phenomenon that is well understood by the local topological instability model, while the latter showed monotonous relaxation behavior over a wide range down to T-g-60 K. The discrepancy may be closely related to the difference in the fragility of both glasses

SN 0255-5476

PY 2010

VL 638-642

BP 1632

EP +

DI 10.4028/www.scientific.net/MSF.638-642.1632

PN 1-4

UT WOS:000281043800270

ER

PT J

AU Joshi, SS

Katakam, S

Arora, HS

Mukherjee, S

Dahotre, NB

AF Joshi, Sameehan S.

Katakam, Shravana

Arora, Harpreet Singh

Mukherjee, Sundeep

Dahotre, Narendra B.

TI Amorphous Coatings and Surfaces on Structural Materials

SO CRITICAL REVIEWS IN SOLID STATE AND MATERIALS SCIENCES

AB Metallic glasses show a unique combination of high strength, excellent corrosion, and wear resistances because of their amorphous structure having a short-range order. In spite of excellent properties, the application of metallic glasses is restricted because of their inherent limitations in the bulk form, including limited tensile ductility. Using metallic glasses as the coatings for structural applications is an attractive way of taking advantage of their superior properties. Additionally, metallic glass-based composites having crystalline phases embedded in a amorphous matrix have also shown improved properties. Thus, metallic glasses can be synthesized as the coatings or subjected to surface modification to provide functionally superior surfaces. This article is a review of metallic glass-based coatings and surface modification of metallic glasses to achieve functionally superior surfaces for structural applications. Essential theoretical concepts were discussed which influence the processing. Common ways of processing along with the influence of various processing parameters were explored. Some non-conventional techniques which emerged as a result of continued efforts were also taken into account. Corrosion and wear properties of these materials along with the underlying mechanisms were discussed in detail. Focus was given to the recent product level applications explored in the open literature. Current challenges in the field were reviewed and guidelines for the future developments were provided.

OI katakam, shravana/0000-0001-7210-0121

SN 1040-8436

EI 1547-6561

PD JAN 2

PY 2016

VL 41

IS 1

BP 1

EP 46

DI 10.1080/10408436.2015.1053602

UT WOS:000368694500001

ER

PT J

AU Mehrer, H

AF Mehrer, Helmut

TI Diffusion in Ion-conducting Oxide Glasses and in Glassy Metals

SO ZEITSCHRIFT FUR PHYSIKALISCHE CHEMIE-INTERNATIONAL JOURNAL OF RESEARCH

IN PHYSICAL CHEMISTRY & CHEMICAL PHYSICS

AB Studies of ionic conduction and of tracer diffusion in oxide glasses and of tracer diffusion in metallic glasses mainly from our laboratory and of viscosity data are reviewed. Common aspects of the motion process of ions or atoms in oxide glasses or glassy metals are pointed out.

SN 0942-9352

PY 2009

VL 223

IS 10-11

BP 1143

EP 1160

DI 10.1524/zpch.2009.6070

UT WOS:000273163300002

ER

PT J

AU Lu, XY

Wang, MS

Du, YL

Liao, WH

AF Lu, Xiaoyang

Wang, Maosong

Du, Yulei

Liao, Wenhe

TI Anisotropy in a bulk metallic glass induced by uni-directional heat flow

SO JOURNAL OF NON-CRYSTALLINE SOLIDS

AB In this work, a controlled uni-directional heat flow was realized during the bulk metallic glasses preparation by using a vertical Bridgman solidification apparatus. Fully amorphous Zr41.2Ti13.8Cu12.5Ni10Be22.5 bulk metallic glass specimens were prepared. The obvious difference in hardness between the cross and longitudinal sections of the as-prepared Zr41.2Ti13.8Cu12.5Ni10Be22.5 bulk metallic glass specimens was identified, indicating strong mechanical anisotropy. The observed anisotropy can be ascribed to the uni-directional heat flow during the process of Bridgman solidification. The mechanical anisotropy may be explained by the free volume theory.

SN 0022-3093

EI 1873-4812

PD APR 15

PY 2018

VL 486

BP 47

EP 51

DI 10.1016/j.jnoncrysol.2018.02.009

UT WOS:000430032600007

ER

PT J

AU Hua, M

Xiang, KW

Jun, YF

Chao, LM

Tao, Z

AF Hua, M

Xiang, KW

Jun, YF

Chao, LM

Tao, Z

TI Formation and mechanical properties of Cu-Zr-Al-Sn bulk metallic glasses

SO MATERIALS TRANSACTIONS

AB Formation and mechanical properties of Cu-Zr-Al-Sn bulk metallic glasses were investigated. The glass-forming ability of Cu50Zr50 alloy is significantly improved with addition of Al, and the critical diameter for glass formation increases from 2 to 7 mm for Cu46.2Zr46.25Al7.5 alloy. Furthermore, the critical diameter is slightly increased to 8 mm by substituting 1 at% So for Zr Of Cu46.25Zr46.25Al7.5 alloy. The bulk glassy Cu46.25Zr46.25Al7.5 alloy exhibits a limited plasticity of about 1.2% to failure under compression and the plasticity is effectively enhanced due to the addition of Sri. about 4.1% for bulk glassy Cu46.25Zr45.25Al7.5Sn1 alloy.

SN 1345-9678

EI 1347-5320

PD JAN

PY 2006

VL 47

IS 1

BP 194

EP 197

UT WOS:000235568400033

ER

PT J

AU Zeng, QS

Fang, YZ

Lou, HB

Gong, Y

Wang, XD

Yang, K

Li, AG

Yan, S

Lathe, C

Wu, FM

Yu, XH

Jiang, JZ

AF Zeng, Q. S.

Fang, Y. Z.

Lou, H. B.

Gong, Y.

Wang, X. D.

Yang, K.

Li, A. G.

Yan, S.

Lathe, C.

Wu, F. M.

Yu, X. H.

Jiang, J. Z.

TI Low-density to high-density transition in Ce75Al23Si2 metallic glass

SO JOURNAL OF PHYSICS-CONDENSED MATTER

AB Using in situ high-pressure x-ray diffraction (XRD), we observed a pressure-induced polyamorphic transition from the low-density amorphous (LDA) state to the high-density amorphous (HDA) state in Ce75Al23Si2 metallic glass at about 2 GPa and 300 K. The thermal stabilities of both LDA and HDA metallic glasses were further investigated using in situ high-temperature and high-pressure XRD, which revealed different pressure dependences of the onset crystallization temperature (T-x) between them with a turning point at about 2 GPa. Compared with Ce75Al25 metallic glass, minor Si doping shifts the onset polyamorphic transition pressure from 1.5 to 2 GPa and obviously stabilizes both LDA and HDA metallic glasses with higher T-x and changes their slopes dT(x)/dP. The results obtained in this work reveal another polyamorphous metallic glass system by minor alloying (e.g. Si), which could modify the transition pressure and also properties of LDA and HDA metallic glasses. The minor alloying effect reported here is valuable for the development of more polyamorphous metallic glasses, even multicomponent bulk metallic glasses with modified properties, which will trigger more investigations in this field and improve our understanding of polyamorphism and metallic glasses.

OI Lou, Hongbo/0000-0002-5056-2576

SN 0953-8984

PD SEP 22

PY 2010

VL 22

IS 37

AR 375404

DI 10.1088/0953-8984/22/37/375404

UT WOS:000281422700009

PM 21403196

ER

PT J

AU Miskuf, J

Csach, K

Jurikova, A

Hurakova, M

Tabachnikova, ED

AF Miskuf, J.

Csach, K.

Jurikova, A.

Hurakova, M.

Tabachnikova, E. D.

TI Fragmentation of Co-Fe-Ta-B Soft Magnetic Amorphous Alloy

SO ACTA PHYSICA POLONICA A

CT 14th European Conference on Physics of Magnetism (PM)

CY JUN 23-27, 2014

CL Poznan, POLAND

SP Polish Acad Sci, Inst Mol Phys, Adam Mickiewicz Univ, Fac Phys

AB The main limitation of high-strength Co-based bulk metallic glasses for their application as structural materials is the large brittleness. Spontaneously emerging cracks in the alloy degrade the magnetic properties. We analyzed the failure characteristics of Co43Fe20Ta5.5B31.5 bulk soft magnetic metallic glass deformed in the compression at room temperature and the low strain rate. Under loading the amorphous structure stores high elastic energy. During the failure this energy is released and the alloy breaks into small particles or powder exhibiting a fragmentation mode.

SN 0587-4246

EI 1898-794X

PD FEB

PY 2015

VL 127

IS 2

BP 558

EP 560

DI 10.12693/APhysPolA.127.558

UT WOS:000352139600132

ER

PT J

AU Wang, AK

Wang, SG

Xue, RJ

Liu, GC

Zhao, K

AF Wang Ai-Kun

Wang Shi-Guang

Xue Rong-Jie

Liu Guo-Cai

Zhao Kun

TI Correlation between Atomic Size Ratio and Poisson's Ratio in Metallic

Glasses

SO CHINESE PHYSICS LETTERS

AB We report the correlation between atomic size ratio and Poisson's ratio in various metallic glasses. It is found that atomic size ratio has an influence on the atomic packing density of metallic glasses, which would significantly impact the shear modulus rather than bulk modulus. The findings may be helpful for understanding the structural origin of Poisson's ratio in metallic glasses, and are instructive for designing tough metallic glasses with large Poisson's ratio.

SN 0256-307X

EI 1741-3540

PD JUN

PY 2014

VL 31

IS 6

AR 066102

DI 10.1088/0256-307X/31/6/066102

UT WOS:000337500700036

ER

PT J

AU Scudino, S

Jerliu, B

Pauly, S

Surreddi, KB

Kuhn, U

Eckert, J

AF Scudino, S.

Jerliu, B.

Pauly, S.

Surreddi, K. B.

Kuehn, U.

Eckert, J.

TI Ductile bulk metallic glasses produced through designed heterogeneities

SO SCRIPTA MATERIALIA

AB Ductile bulk metallic glasses are produced from intrinsically brittle precursors by proper control of shear band formation and propagation. This ductilization strategy is based on the creation of alternating soft and hard regions via imprinting at room temperature. The stress fields resulting from these regions interfere with the process of irreversible deformation and effectively limit shear bands from propagating catastrophically. (C) 2011 Acta Materialia Inc. Published by Elsevier Ltd. All rights reserved.
RI Surreddi, Kumar Babu/C-2454-2008; Scudino, Sergio/D-8049-2015
OI Surreddi, Kumar Babu/0000-0001-7938-9909;
SN 1359-6462
PD NOV
PY 2011
VL 65
IS 9
BP 815
EP 818
DI 10.1016/j.scriptamat.2011.07.039
UT WOS:000295765300018
ER

PT J
AU Zheng, Q
   Cheng, S
   Strader, JH
   Ma, E
   Xu, J
AF Zheng, Q.
   Cheng, S.
   Strader, J. H.
   Ma, E.
   Xu, J.
TI Critical size and strength of the best bulk metallic glass former in the
   Mg-Cu-Gd ternary system
SO SCRIPTA MATERIALIA
AB We have developed a Mg61Cu28Gd11 bulk metallic glass (BMG) that has the highest glass-forming ability among all the known ternary Mg-based BMGs. We also used a microcompression test to determine the intrinsic strength of such brittle Mg-based BMGs. The strength and plasticity of BMGs at small sample sizes are discussed in comparison with their behavior in conventional compression tests. (c) 2006 Acta Materialia Inc. Published by Elsevier Ltd. All rights reserved.
RI Cheng, Sheng/D-9153-2013; Zheng, Qiang/A-4566-2010; Ma, En/A-3232-2010
OI Cheng, Sheng/0000-0003-1137-1926; Zheng, Qiang/0000-0002-6057-8141;
SN 1359-6462
PD JAN
PY 2007
VL 56
IS 2
BP 161
EP 164
DI 10.1016/j.scriptamat.2006.09.023
UT WOS:000242513800021
ER

PT J
AU Zhang, LL
   Li, R
   Wang, JF
   Zhang, HY
   Hua, NB
   Zhang, T
AF Zhang, Leilei
   Li, Ran
   Wang, Jianfeng
   Zhang, Huiyan
   Hua, Nengbin
   Zhang, Tao
TI The influence of Ag substitution for Cu on glass-forming ability and
   thermal properties of Mg-based bulk metallic glasses
SO JOURNAL OF NON-CRYSTALLINE SOLIDS
AB A group of pseudo-ternary Mg-(Cu-Ag)-Dy bulk metallic glasses (BMGs) was developed by copper mold casting. The glass-forming ability (GFA) is significantly improved by the coexistence of similar elements of Ag and Cu. The critical diameter for glass formation increases from 10 mm for ternary Mg56.5Cu32Dy11.5 alloy to 18 Mill for pseudo-ternary Mg56.5Cu27Ag5Dy11.5 alloy. Thermal stability, crystallization and melting behaviors of the Mg-based BMGs were evaluated. The decrease of Gibbs free energy difference between undercooled liquid and crystalline phases caused by similar element substitution with optimal amount can be responsible for the increase in GFA of the resulting alloys. (C) 2012 Elsevier B.V. All rights reserved.
RI Zhang, Tao/O-4911-2014; Li, Ran/B-4618-2010
SN 0022-3093
PD JUL 1
PY 2012
VL 358
IS 12-13
BP 1425
EP 1429
DI 10.1016/j.jnoncrysol.2012.03.010
UT WOS:000306822000003
ER

PT J
AU Xu, YL
   Fang, JX
   Gleiter, H
   Hahn, H
   Li, JG
AF Xu, Yuanli
   Fang, Jixiang
   Gleiter, Herbert
   Hahn, Horst
   Li, Jiangong
TI Quantitative determination of free volume in Pd40Ni40P20 bulk metallic
   glass
SO SCRIPTA MATERIALIA
AB For a long time, the determination of free volume has been a challenging problem in research on metallic glasses. An approach to determine quantitatively the free volume of metallic glasses from enthalpy measurements and calibration with the equilibrium free volume was developed and validated for as-cast and annealed Pd40Ni40P20 bulk metallic glasses. The free volume change with annealing time is in good agreement with that calculated theoretically from the free volume annihilation kinetics and that deduced from the density measurement results. (C) 2010 Acta Materialia Inc. Published by Elsevier Ltd. All rights reserved.
RI Fang, Jixiang/C-5981-2015; Hahn, Horst/G-9018-2011
OI Hahn, Horst/0000-0001-9901-3861; Li, Jiangong/0000-0001-6837-1084
SN 1359-6462
PD MAY
PY 2010
VL 62
IS 9
BP 674
EP 677
DI 10.1016/j.scriptamat.2010.01.025
UT WOS:000275933700011
ER

PT J
AU Lee, MH
   Kim, JY
   Kuhn, U
   Eckert, J
AF Lee, Min Ha
   Kim, Jin Young
   Kuehn, Uta
   Eckert, Juergen
TI Influence of viscous flow on the deformation behavior of bulk metallic
   glassy alloys in supercooled liquid region
SO INTERMETALLICS
AB We present the investigation of the effect of viscosity on the deformation behavior of metallic glasses at supercooled liquid region during hot extrusion. Also the change of the specific surface area in porous Hf-based metallic glass and Ni-based metallic glass were compared. The homogeneously distributed ruggedness-shape surface of ligament was observed in the Hf-based metallic glass which has lower viscosity comparing to that of Ni-based metallic glass. For lower viscosity, it is very desirable to fabricate large surface area porous metallic glass due to a change of the surface morphology of the ligaments from smooth-like to rugged-like appearance, thus suggesting an optimum regime for increasing the specific surface area by controlling viscosity of metallic glass at a given processing condition. (C) 2012 Elsevier Ltd. All rights reserved.
OI LEE, MIN HA/0000-0001-6006-0628
SN 0966-9795
PD NOV
PY 2012
VL 30
SI SI
BP 72
EP 75
DI 10.1016/j.intermet.2012.03.035
UT WOS:000308847400012
ER

PT J
AU Dong, FY
   Su, YQ
   Luo, LS
   Wang, L
   Wang, SJ
   Guo, LJ
   Fu, HZ
AF Dong, Fuyu
   Su, Yanqing
   Luo, Liangshun
   Wang, Liang
   Wang, Shujie
   Guo, Lingjie
   Fu, Hengzhi
TI Enhanced plasticity in Zr-based bulk metallic glasses by hydrogen
SO INTERNATIONAL JOURNAL OF HYDROGEN ENERGY
AB The amorphous alloys of Zr57Al10Cu15.4Ni12.6Nb5 and Zr55Cu30Ni5Al10 were melted and cast under the gaseous mixture of hydrogen and argon. The effects of hydrogen on glass plasticity of the two bulk metallic glasses were studied. Surprisingly, it was found. that hydrogen addition can increase the glass plasticity significantly. The large plasticity of glass is attributed to the large amount of free volume induced by hydrogen addition, which could make this melt hydrogenation method to be a promising new way to improve plasticity of amorphous glass. Copyright (C) 2012, Hydrogen Energy Publications, LLC. Published by Elsevier Ltd. All rights reserved.
RI Luo, LiangShun/C-7116-2008
OI Luo, LiangShun/0000-0003-3919-938X
SN 0360-3199
PD OCT
PY 2012
VL 37
IS 19
BP 14697
EP 14701
DI 10.1016/j.ijhydene.2012.07.034
UT WOS:000309493600086
ER

PT S
AU Wang, SX
   Wu, ZY
AF Wang, Sanxin
   Wu, Zeyu
BE Xu, B
   Li, HY
TI Magnetic properties and glass forming ability of Fe-B-Y-Nb-Zr bulk
   metallic glasses
SO ENERGY MATERIAL, CHEMICAL ENGINEERING AND MINING ENGINEERING
SE Advanced Materials Research
CT International Conference on Energy Material, Chemical Engineering and
   Mining Engineering (EMCEM 2012)
CY SEP 15-16, 2012
CL Wuhan, PEOPLES R CHINA
SP Wuhan Inst Technol, Beijing Mat Res Ctr, Int Mat Res Soc
AB Bulk metallic glasses (BMGs) alloys (Fe71.2B24Y4.8)96Nb4-xZrx (x = 1-4 at.%) with a maximum diameter of 5 mm were synthesized with low purity materials by copper mold casting technique. The effect of Zr substitution for Nb on the structure, thermal stability and the magnetic properties has been studied. It was found that the substitution of an appropriate amount of Zr for Nb can improve the glass forming ability. The wide supercooled liquid region Delta Tx (exceeding 123 K) and a high Tg (exceeding 868 K) demonstrated a high thermal stability of the present Fe-based BMGs. In addition, these BMGs also exhibit good soft magnetic properties with relatively high saturation magnetization of 88 emu/g, and low coercivity of 20 Oe.
SN 1022-6680
BN 978-3-03785-499-0
PY 2012
VL 577
BP 27
EP +
DI 10.4028/www.scientific.net/AMR.577.27
UT WOS:000317632300006
ER

PT J
AU Calin, M
   Eckert, J
   Schultz, L
AF Calin, M
   Eckert, J
   Schultz, L
TI Improved mechanical behavior of Cu-Ti-based bulk metallic glass by in
   situ formation of nanoscale precipitates
SO SCRIPTA MATERIALIA
AB The mechanical properties of Cu47Ti34Zr11Ni8, Cu47Ti33Zr11Ni8Fe1, and Cu47Ti33Zr11Ni8Si1 bulk metallic glasses prepared by copper mold casting are investigated. Room temperature compression tests reveal fracture strengths above 2000 MPa, Young's moduli around 100 GPa and elastic strains slightly exceeding 2.0%. Only Cu47Ti33Zr11Ni8Si1 exhibits distinct plastic strain due to a unique composite microstructure with in situ formed nanoscale precipitates in the glassy matrix. (C) 2003 Acta Materialia Inc. Published by Elsevier Science Ltd. All rights reserved.
RI Schultz, Ludwig/B-3383-2010; Calin, Mariana/J-6398-2015
SN 1359-6462
PD MAR 17
PY 2003
VL 48
IS 6
BP 653
EP 658
AR PII S1359-6462(02)00560-2
DI 10.1016/S1359-6462(02)00560-2
UT WOS:000181413100001
ER

PT J
AU BAKKE, E
   BUSCH, R
   JOHNSON, WL
AF BAKKE, E
   BUSCH, R
   JOHNSON, WL
TI THE VISCOSITY OF THE ZR46.75TI8.25CU7.5NI10BE27.5 BULK METALLIC-GLASS
   FORMING ALLOY IN THE SUPERCOOLED LIQUID
SO APPLIED PHYSICS LETTERS
AB The viscosity of the Zr46.75Ti8.25Cu7.5Ni10Be27.5 bulk metallic glass forming alloy in the supercooled liquid was measured using parallel plate rheometry. The measurements were carried out with different heating rates between 0.0167 and 1.167 K/s as well as isothermally. Because of the high thermal stability above the glass transition of this bulk metallic glass former with respect to crystallization, it was possible to measure viscosities in the range from 10(10) to 10(6) poise. This region of viscosities has not been previously measured for supercooled metallic melts. Our measurements suggest that the viscosity of the supercooled liquid of this bulk glass former exhibits a small Vogel-Fulcher temperature relative to the glass transition temperature, similar to silicate glasses. (C) 1995 American Institute of Physics.
SN 0003-6951
PD NOV 27
PY 1995
VL 67
IS 22
BP 3260
EP 3262
DI 10.1063/1.114891
UT WOS:A1995TF76500012
ER

PT J
AU Lu, ZB
   Li, JG
   Shao, H
   Gleiter, H
   Ni, X
AF Lu, Zhibin
   Li, Jiangong
   Shao, Hang
   Gleiter, H.
   Ni, Xia
TI The correlation between shear elastic modulus and glass transition
   temperature of bulk metallic glasses
SO APPLIED PHYSICS LETTERS
AB Based on Varshni equation, the shear elastic modulus at the glass transition temperature [G(T(g))] and the shear elastic modulus at 0 K [G(0)] were calculated from the elastic modulus measured at room temperature for various bulk metallic glasses (BMGs). The G(T(g))/G(0) ratios for various BMGs are almost the same and have a value around 0.85. This unique correlation implies that the glass transition occurs when the shear modulus of a BMG decreases to about 85% of G(0). This correlation between shear modulus and glass transition is of significance in understanding the glass transition of BMGs.
OI Li, Jiangong/0000-0001-6837-1084
SN 0003-6951
PD MAR 2
PY 2009
VL 94
IS 9
AR 091907
DI 10.1063/1.3093879
UT WOS:000264523100029
ER

PT J
AU Cheng, JL
   Chen, G
   Zeng, QQ
   Yun, L
   Xu, F
AF Cheng, Jia-lin
   Chen, Guang
   Zeng, Qiang-qiang
   Yun, Lu
   Xu, Feng
TI Effect of Oxygen on Microstructure Evolution and Glass Formation of
   Zr-based Metallic Glasses
SO JOURNAL OF IRON AND STEEL RESEARCH INTERNATIONAL
AB The effect of oxygen on the microstructure evolution and glass formation of Zr-based bulk metallic glasses (BMGs) was studied in detail. It was found that oxygen did not form oxides or dissolve in glass matrix, but induced the precipitation of alpha-Zr which has the high affinity and solubility of oxygen in the Zr-based bulk metallic glass (Zr-BMG). With the precipitation of alpha-Zr, the remaining melts contain much lower oxygen content and have strong glass formation, resulting in the formation of alpha-Zr/BMG composite. The findings provide an important insight into the mechanism of the oxygen on glass formation, and give us a useful guideline to avoid the oxygen detrimental for designing new BMGs.
```

RI 徐, 锋/N-8661-2017

OI 徐, 锋/0000-0001-5802-7925

```
SN 1006-706X
EI 2210-3988
PD JAN
PY 2016
VL 23
IS 1
BP 78
EP 82
DI 10.1016/S1006-706X(16)30016-4
UT WOS:000373035000016
ER

PT J
AU Jiang, MQ
   Dai, LH
AF Jiang, Minqiang
   Dai, Lanhong
TI Intrinsic correlation between fragility and bulk modulus in metallic
   glasses
SO PHYSICAL REVIEW B
AB A systematic study on the available data of 26 metallic glasses shows that there is an intrinsic correlation between fragility of a liquid and bulk modulus of its glass. The underlying physics can be rationalized within the formalism of potential energy landscape thermodynamics. It is surprising to find that the linear correlation between the fragility and the bulk-shear modulus ratio exists strictly at either absolute zero temperature or very high frequency. Further analyses indicate that a real flow event in bulk metallic glasses is shear dominant, and fragility is in inverse proportion to shear-induced bulk dilatation. Finally, extension of these findings to nonmetallic glasses is discussed.
OI Dai, LanHong/0000-0001-8991-0358
SN 2469-9950
EI 2469-9969
PD AUG
PY 2007
VL 76
IS 5
AR 054204
DI 10.1103/PhysRevB.76.054204
UT WOS:000249155100056
ER

PT J
AU Peng, H
   Li, SS
   Qi, YP
   Huang, TY
AF Peng, Hao
   Li, Shuang Shou
   Qi, Yun Peng
   Huang, Tian You
TI Mg-Ni-Gd-Ag bulk metallic glass with improved glass-forming ability and
   mechanical properties
SO INTERMETALLICS
AB The improvement of glass-forming ability (GFA) and mechanical properties by using Ag to substitute Mg in the Mg-Ni-Gd bulk metallic glass (BMG) were studied. The Mg69Ni15Gd10Ag6 bulk metallic glass (BMG) could be cast into glassy rod up to 7 mm. The activation energies of Mg69Ni15Gd10Ag6 metallic glass were calculated by the Kissinger's method to be E-g = 2.24 eV, E-x = 1.65 eV, E-p1 =1.36 eV, E-p2 = 1.59 eV, E-93 = 1.26 eV and E-p4 = 1.99 eV. The compressive fracture strength and the plastic strain of Mg69Ni15Gd10Ag6 BMG reached 846 MPa and 0.37% respectively. (C) 2010 Elsevier Ltd. All rights reserved.
SN 0966-9795
EI 1879-0216
PD JUL
PY 2011
VL 19
IS 7
BP 829
EP 832
DI 10.1016/j.intermet.2010.11.029
UT WOS:000291190900001
ER

PT J
AU Saida, J
   Kato, H
   Setyawan, ADH
   Yoshimi, K
   Inoue, A
AF Saida, Junji
   Kato, Hidemi
   Setyawan, Albertus Deny Heri
   Yoshimi, Kyosuke
   Inoue, Akihisa
TI Deformation-induced nanoscale dynamic transformation studies in
   Zr-Al-Ni-pd and Zr-Al-Ni-Cu bulk metallic glasses
SO MATERIALS TRANSACTIONS
AB Nanoscale dynamic transformations during tensile and compressive deformation of Zr65Al7.5Ni10Cu17.5 and Zr65Al7.5Ni10Pd17.5 bulk metallic glasses have been investigated. Although no apparent differences are observed in the stress-strain curves in the tensile deformation between the two alloys, fine striations and depression zones with viscous flow appear at the fracture surface near the edge in the Zr65Al7.5Ni10Pd17.5 alloy. Unlike the Zr65Al7.5Ni10Cu17.5 alloy and other bulk metallic glasses, the Zr65Al7.5Ni10Pd17.5 bulk metallic glass exhibits a large plastic strain of approximately 7% during compressive deformation. By detailed examination of the inicrostructure, we provide direct evidence for nanoscale multistep shear band formation in the Zr65Al7.5Ni10Pd17.5 metallic glass. A novel nanoscale structure where fcc Zr2Ni nanocrystalline particles are arranged in "bandlike" areas in the glassy matrix is observed near the compressive fracture tip. The suppression of the propagation of the shear bands due to dynamic nanocrystallization causes this structure. Furthermore, the results are recognized as a novel phenomenon, a nanoscale dynamic structural change by shear band propagation. and provide a new method for improving the mechanical properties of bulk metallic glasses.
RI Inoue, Akihisa/E-5271-2015; Setyawan, Albertus D.H./C-8602-2011; Saida,
   Junji/C-1912-2009; Kato, Hidemi/B-2492-2015
OI Setyawan, Albertus D.H./0000-0001-8720-6455;
SN 1345-9678
EI 1347-5320
PD JUN
PY 2007
VL 48
IS 6
BP 1327
EP 1335
DI 10.2320/matertrans.MF200615
UT WOS:000247823300030
ER

PT J
AU Lu, ZP
   Liu, CT
AF Lu, Z. P.
   Liu, C. T.
TI A scheme to design multi-component bulk metallic glasses in Ideal
   glass-forming liquids
SO MATERIALS TRANSACTIONS
AB In this study, we have proposed to use binary eutectics, rather than individual constituent elements, as basic units for designing complex multi-component bulk metallic glasses (BMGs), based on a novel physical concept of "ideal" glass-forming liquids. An innovative approach to designing multi-component BMGs in these ideal liquids was thus established and the reliability and usefulness of the current approach of this strategy has been confirmed in the Zr-Fe-Cu-Al metallic systems. As a result, several new BMGs with superior GFA in this system were successfully developed.
RI Lu, Zhao-Ping/A-2718-2009
OI Lu, Zhao-Ping/0000-0003-1463-8948; Liu, Chain Tsuan/0000-0001-7888-9725
SN 1345-9678
EI 1347-5320
PD SEP
PY 2007
VL 48
IS 9
BP 2476
EP 2482
DI 10.2320/matertrans.MJ200762
UT WOS:000250195500044
ER

PT J
AU Liu, CT
   Lu, ZP
AF Liu, CT
   Lu, ZP
TI Effect of minor alloying additions on glass formation in bulk metallic
   glasses
SO INTERMETALLICS
CT 5th International Workshop on Ordered Intermetallics and Advanced
   Metallic Materials
CY OCT 06-09, 2003
CL Changdu, PEOPLES R CHINA
AB This paper provides a comprehensive review of recent work on the effect of minor alloying additions on glass formation in bulk metallic Glasses. Recently, minor alloying addition or microalloying technology has shown to have dramatic effects on the glass formation and thermal stability of many bulk metallic glasses. This technology basically involves adding small amounts of alloying additions (usually, less 2 at%) to existing bulk metallic glasses for the purpose of further improving their glass forming ability. Experimental evidences indicate that alloying additions of small atoms with atomic radius < 0.12 nm (such as B and Si) or large atoms with radius > 0.16 nm (such as Y and Sc) are most effective in enhancing glass forming ability. The beneficial effects of these elements are discussed in terms of physical metallurgy principles. (c) 2004 Elsevier Ltd. All rights reserved.
RI Lu, Zhao-Ping/A-2718-2009
OI Lu, Zhao-Ping/0000-0003-1463-8948; Liu, Chain Tsuan/0000-0001-7888-9725
SN 0966-9795
PD MAR-APR
PY 2005
VL 13
IS 3-4
SI SI
BP 415
EP 418
DI 10.1016/j.intermet.2004.07.034
UT WOS:000227668000030
ER

PT J
AU Madge, SV
   Alexander, DTL
   Greer, AL
AF Madge, SV
   Alexander, DTL
   Greer, AL
TI An EFTEM study of compositional variations in Mg-Ni-Nd bulk metallic
   glasses
SO JOURNAL OF NON-CRYSTALLINE SOLIDS
CT Annual Meeting of The-Minerals-Metals-and-Materials-Society
CY FEB 17-21, 2002
CL SEATTLE, WASHINGTON
SP Minerals Met & Mat Soc, Extract & Proc Div
AB A range of Mg-Ni-Nd bulk metallic glasses has been cast. Around the composition Mg65Ni20Nd15 (at.%), but not at neighbouring compositions, energy-filtered transmission electron microscopy provides evidence for strong compositional inhomogeneities in the as-cast glass. In this fully glassy material, an apparent two-phase cellular microstructure is seen; one phase is rich in Ni, the other in Mg and Nd. Annealing at a temperature just above the glass transition, or even holding at room temperature for several months can eliminate the inhomogeneities. The diffusivity values derived from the homogenization kinetics are in good agreement with those known for other bulk metallic glasses. The present results suggest the existence of a closed-loop miscibility gap in the glass-forming liquid. (C) 2003 Elsevier Science B.V. All rights reserved.
RI Greer, Alan Lindsay/G-1977-2011; Greer, Lindsay/E-9433-2017; Alexander,
   Duncan/E-9703-2015
OI Alexander, Duncan/0000-0003-4350-8587; Madge,
   Shantanu/0000-0001-7996-8652
SN 0022-3093
PD MAR
PY 2003
VL 317
IS 1-2
BP 23
EP 29
DI 10.1016/S0022-3093(02)01979-8
UT WOS:000181251300005
ER

PT J
AU Liu, LF
   Dai, LH
   Bai, Y
   Wei, BC
   Eckert, J
AF Liu, LF
   Dai, LH
   Bai, Y
   Wei, BC
   Eckert, J
TI Behavior of multiple shear bands in Zr-based bulk metallic glass
SO MATERIALS CHEMISTRY AND PHYSICS
AB The deformation behavior of Zr41.2Ti13.8Cu12.5Ni10Be22.5 bulk metallic glass was studied by in situ scanning electron microscopy (SEM) quasi-static uniaxial compression tests at room temperature. Multiple shear bands were observed with a large plasticity. Microscopic examination demonstrates that slipping, branching and intersecting of multiple shear bands are the main mechanisms for enhancing the plasticity of this metallic glass. Additionally, nano/micro-scale voids and cracks at the intersecting sites of shear bands and preferential etching of shear bands were observed as well. These observations demonstrated that the formation of shear bands in bulk metallic glasses is resulted mainly from local free volume coalescence. (c) 2005 Elsevier B.V. All rights reserved.
OI Dai, LanHong/0000-0001-8991-0358
SN 0254-0584
PD SEP 15
PY 2005
VL 93
IS 1
BP 174
EP 177
DI 10.1016/j.matchemphys.2005.03.011
UT WOS:000230375200031
ER

PT J
AU Bae, DH
   Lee, SW
   Kwon, JW
   Wang, XD
   Yi, S
AF Bae, D. H.
   Lee, S. W.
   Kwon, J. W.
   Wang, X. D.
   Yi, S.
TI Ductile Zr-base bulk metallic glass
SO MATERIALS SCIENCE AND ENGINEERING A-STRUCTURAL MATERIALS PROPERTIES
   MICROSTRUCTURE AND PROCESSING
CT 12th International Conference on Rapidly Quenched and Metastable
   Materials
CY AUG 21-26, 2005
CL Cheju Isl, SOUTH KOREA
AB Bulk metallic glasses have a frozen-liquid structure comprising multi-component elements. Plastic deformation of bulk metallic glasses is highly localized into shear bands. As the shear band propagates, excess free volume is generated and spontaneously coalesced, leading to the formation of nanometer-scale voids. The voids evolve to be cracks that immediately cross the whole sample area causing a catastrophic fracture even under compression. Therefore, to prohibit the catastrophic failure, atomic clustering kinetics should be retarded. Here, we report a new monolithic zirconium-base metallic glass that is highly deformable without global failure under compression. Due to high thermal activation energy for the atomic movement, the crack formation attributed to void coalescence within shear bands can be prevented exhibiting extraordinarily high ductility under compression. (c) 2006 Elsevier B.V. All rights reserved.
RI Juhyun, Oh/H-9185-2012
SN 0921-5093
EI 1873-4936
PD MAR 25
PY 2007
VL 449
BP 111
EP 113
DI 10.1016/j.msea.2006.02.235
UT WOS:000245477800022
ER

PT J
AU Qiao, JC
   Pelletier, JM
AF Qiao, J. C.
   Pelletier, J. M.
TI Dynamic Mechanical Relaxation in Bulk Metallic Glasses: A Review
SO JOURNAL OF MATERIALS SCIENCE & TECHNOLOGY
AB Metallic glasses have aroused considerable interest in the past decades because they exhibit fascinating properties. First, this article briefly outlines the mechanical, thermal properties and application of the metallic glasses. In addition, we focus on the dynamic mechanical relaxation behaviors, i.e. main (alpha) and secondary (beta) relaxations, in metallic glasses. The mechanical relaxation behaviors are connected to the mechanical properties and physical properties in glassy materials. The main relaxation in glassy materials is related to the glass transition phenomenon and viscous flow. On the other hand, the beta relaxation is linked to many fundamental issues in metallic glasses. In these materials relaxation processes are directly related to the plastic deformation mechanism. The mechanical relaxations, particularly, the beta relaxation provides an excellent opportunity to design metallic glasses with desired physical and mechanical properties. We demonstrate the universal characteristics of main relaxation in metallic glasses. The phenomenological models and the physical theories are introduced to describe the main relaxation in metallic glasses. In parallel, we show the dependence of the alpha and beta relaxations on the thermal treatments in metallic glasses. Finally, we analyze the correlation between the atomic mobility and the thernno-mechanical treatments in metallic glasses. On the one hand, the atomic mobility in metallic glasses is reduced by physical aging or crystallization. On the other hand, the atomic mobility in metallic glass is enhanced by deformation (i.e. compression and cold rolling). Importantly, to analyze the atomic mobility in amorphous materials, a physical theory is introduced. This model invokes the concept of quasi-point defects, which correspond to the density fluctuations in the glassy materials.
SN 1005-0302
PD JUN
PY 2014
VL 30
IS 6
BP 523
EP 545
DI 10.1016/j.jmst.2014.04.018
UT WOS:000340350300002
ER

PT J
AU Madge, SV
   Louzguine-Luzgin, DV
   Inoue, A
   Greer, AL
AF Madge, Shantanu V.
   Louzguine-Luzgin, Dmitri V.
   Inoue, Akihisa
   Greer, Alan Lindsay
TI Large Compressive Plasticity in a La-Based Glass-Crystal Composite
SO METALS
AB La55Al25Cu10Ni10 metallic glass has been reinforced with 325-mesh Ta particles to obtain ex situ glass-crystal composites. The composites show a high compressive plasticity (40%) with a minor reduction (similar to 8%) in yield strength-a combination unprecedented for La-based systems and even surpassing some Zr-based glassy composites that utilize a tougher matrix. However, it is also found that the plastic strain is apparently sensitive to defects, like oxides, in the glassy matrix.
RI Sahu, Anjani/E-7590-2015; Inoue, Akihisa/E-5271-2015; Greer,
   Lindsay/E-9433-2017; LOUZGUINE, Dmitri/D-2492-2010
OI LOUZGUINE, Dmitri/0000-0001-5716-4987; Madge,
   Shantanu/0000-0001-7996-8652
SN 2075-4701
PD MAR
PY 2013
VL 3
IS 1
BP 41
EP 48
DI 10.3390/met3010041
UT WOS:000343292200003
ER

PT J
AU Ma, MZ
   Zong, HT
   Wang, HY
   Qi, YP
   Liang, SX
   Song, AJ
   Zhang, WG
   Wang, Q
   Zhang, XY
   Jing, Q
   Li, G
   Liu, RP
AF Ma MingZhen
   Zong HaiTao
   Wang HaiYan
   Qi YanPeng
   Liang SunXing
   Song Aijun
   Zhang WeiGuo
   Wang Qiang
   Zhang Xinyu
   Jing Qin
   Li Gong
   Liu RiPing
TI The fluidity and molding ability of glass-forming Zr-based alloy melt
SO SCIENCE IN CHINA SERIES G-PHYSICS MECHANICS & ASTRONOMY
AB The fluidity and filling ability of glass-forming Zr-based alloy melt in copper mould were investigated both theoretically and experimentally. The major factors which affected the flowing behavior of the metallic melt in the mold were determined, which provides the foundation for overcoming the contradiction between the filling and formation of amorphous alloy during the rapid cooling process of the metallic melts. The casting factors to prepare a metallic ring were discussed and selected. As a result, a Zr-based bulk metallic glass ring was prepared successfully.
SN 1672-1799
PD APR
PY 2008
VL 51
IS 4
BP 438
EP 444
DI 10.1007/s11433-008-0041-6
UT WOS:000254406000013
ER

PT J
AU Loffler, JF
AF Loffler, JF
TI Bulk metallic glasses
SO INTERMETALLICS
AB In the last decade metallic glasses have regained considerable interest due to the fact that new glass-forming compositions have been found that have a critical cooling rate of less than 100 K/s and can be made glassy with dimensions of I cm or more. The development of such alloys with a very high resistance to crystallization of the undercooled melt has opened new opportunities for the fundamental study of both the liquid state and the glass transition. The availability of bulk specimens has enabled measurements of various physical, particularly mechanical, properties that were previously impossible. Furthermore, these alloys are progressively being used for engineering applications. In order to recognize this significant advancement in materials science, this new class of materials is commonly referred to as "bulk metallic glasses". This article will first review the history of metallic glasses. The origins of glass formation are then discussed and thermophysical properties (crystallization, phase separation, viscosity and diffusion) are presented. Furthermore, the paper outlines magnetic and mechanical properties along with resulting applications and presents a new processing technique to discover bulk metallic glass compositions in Section 5. (C) 2003 Elsevier Science Ltd. All rights reserved.
SN 0966-9795
PD JUN
PY 2003
VL 11
IS 6
BP 529
EP 540
DI 10.1016/S0966-9795(03)00046-3
UT WOS:000183676400002
ER

PT J
AU Zhao, CL
   Dun, CC
   Man, QK
   Shen, BL
AF Zhao, Chengliang
   Dun, Chaochao
   Man, Qikui
   Shen, Baolong
TI Enhancement of plastic deformation in FeCoNbB bulk metallic glass with
   superhigh strength
SO INTERMETALLICS
AB The effect of B on the glass-forming ability of FeCoNbB alloys was investigated. Bulk metallic glasses with critical diameters up to 3 mm and superhigh yield strength of 4860 MPa were synthesized in (Fe0.5Co0.5)(71-x)Nb6B23+x (x = 0, 2, 3, 4 and 5) alloy system. Besides, Cu was added to the Fe33.5Co33.5Nb6B27 alloy with the aim at enhancing mechanical properties, and it was found that proper amount of Cu addition could effectively improve the compressive plasticity from 1.4% to 3.7% without obvious strength decreasing. The enhancement of plasticity is ascribed to the formation of clusters in Cu-contained FeCoNbB bulk metallic glasses. (C) 2012 Elsevier Ltd. All rights reserved.
SN 0966-9795
EI 1879-0216
PD JAN
PY 2013
VL 32
BP 408
EP 412
DI 10.1016/j.intermet.2012.09.018
UT WOS:000313145500055
ER

PT J
AU Ngai, KL
   Wang, Z
   Gao, XQ
   Yu, HB
   Wang, WH
AF Ngai, K. L.
   Wang, Z.
   Gao, X. Q.
   Yu, H. B.
   Wang, W. H.
TI A connection between the structural alpha-relaxation and the
   beta-relaxation found in bulk metallic glass-formers
SO JOURNAL OF CHEMICAL PHYSICS
AB New metallic glasses containing La or Ce have been introduced having dynamic properties bordering on two extremes of conventional metallic glasses. This provides opportunity to test if the trends or correlations established before in molecular and polymeric glass-formers also exist in the broader family of metallic glasses. Due to the drastically different chemical and physical structures of metallic glass-formers than soft matter, there is no guarantee that any correlation found in the latter will hold in the former. If the trends and correlations are found, the result brings metallic glasses closer to the much wider classes of glass-formers in the similarity of properties. In non-metallic glass-formers, a general and fundamental connection has been established between the non-exponentiality parameter of the structural alpha-relaxation and the separation between its relaxation time tau(alpha) and the beta-relaxation time tau(beta). In this paper, we explore the experimental data of metallic glass-formers and show the correlation applies. An explanation of this correlation is given by the Coupling Model. The establishment of the correlation may facilitate the understanding of the roles played by the beta-relaxation in macroscopic properties of metallic glasses including the relations to shear transformation zone, ductile-brittle transition in deformation, crystallization, and diffusion. (C) 2013 AIP Publishing LLC.
RI Yu, Hai Bin/E-5312-2010; Wang, Zheng/O-1993-2013
OI Yu, Hai Bin/0000-0003-0645-0187; Wang, Zheng/0000-0002-7909-4796
SN 0021-9606
PD JUL 7
PY 2013
VL 139
IS 1
AR 014502
DI 10.1063/1.4812281
UT WOS:000321716400025
PM 23822309
ER

PT J
AU Jiang, F
   Zhang, ZB
   Zhang, J
   He, L
   Sun, J
AF Jiang, F
   Zhang, ZB
   Zhang, J
   He, L
   Sun, J
TI Bending ductility and shear band spacing of copper-based metallic glass
   plate
SO ACTA METALLURGICA SINICA
AB A Copper-based bulk metallic glass with compression plastic stain near to 2% was prepared and bending tests were undertaken to investigate the dependences of ductility and shear banding spacing on metallic plat thickness. The results show that the relationship between bending ductility and the plate thickness is an exponential decay relation, which is agreement well with the relation between the bending fracture plastic stain and the plate thickness. The results also show that the shear band spacing in metallic glass plates under bending varies linearly with plate thickness and the ratio of shear band spacing to plat thickness is a constant. However, for different metallic glasses, the ratios are not a constant and dependent on their plastic deformation ability.
RI Zhang, Jian/A-5047-2009; jiang, Feng/D-1427-2012
OI jiang, Feng/0000-0003-3651-8007
SN 0412-1961
PD OCT
PY 2005
VL 41
IS 10
BP 1031
EP 1036
UT WOS:000233255700005
ER

PT J
AU Zhang, Y
   Zhou, YJ
   Hui, XD
   Wang, ML
   Chen, GL
AF Zhang Yong
   Zhou YunJun
   Hui XiDong
   Wang MeiLing
   Chen GuoLiang
TI Minor alloying behavior in bulk metallic glasses and high-entropy alloys
SO SCIENCE IN CHINA SERIES G-PHYSICS MECHANICS & ASTRONOMY
AB The effect of minor alloying on several bulk metallic glasses and high-entropy alloys was studied. It was found that minor Nb addition can optimize the interface structure between the W fiber and the Zr-based bulk metallic glass in the composites, and improve the mechanical properties. Minor Y addition can destabilize the crystalline phases by inducing lattice distortion as a result to improve the glass-forming ability, and the lattice distortion energy is closely related to the efficiency of space filling of the competing crystalline phases. A long-period ordered structure can precipitate in the Mg-based bulk metallic glass by yttrium alloying. For the high-entropy alloys, solid solution can be formed by alloying, and its mechanical properties can be comparable to most of the bulk metallic glasses.
RI ZHANG, Yong/B-7928-2009; Hui, Xidong/A-1741-2010
OI ZHANG, Yong/0000-0002-6355-9923;
SN 1672-1799
PD APR
PY 2008
VL 51
IS 4
BP 427
EP 437
DI 10.1007/s11433-008-0050-5
UT WOS:000254406000012
ER

PT J
AU Li, J
   Xiao, XS
   Dong, Y
AF Li, Jun
   Xiao, Xueshan
   Dong, Yuanda
TI Thermal stability and glass-forming ability of Y-Ce-Co-Al bulk metallic
   glasses
SO JOURNAL OF UNIVERSITY OF SCIENCE AND TECHNOLOGY BEIJING
AB The thermal stability and glass-forming ability of Y56-xCexCo20Al24 (x=15, 20, 25, 28, 38, 41, 44) bulk metallic glasses with a diameter of 5 mm were investigated by differential scanning calorimetry and X-ray diffraction. The results show that the thermal stability of the alloys decreases with the addition of Ce. It has the best glass-forming ability when x=25, whose calculated values can reach about 30 mm in diameter. The effect of Ce element could be explained on the view of Miedema's theory and electronegativity difference of amorphous alloys.
SN 1005-8850
PD JUN
PY 2007
VL 14
SU 1
BP 23
EP 25
DI 10.1016/S1005-8850(07)60101-7
UT WOS:000250593400006
ER

PT S
AU Patel, AT
   Lad, KN
   Pratap, A
AF Patel, Ashmi T.
   Lad, Kirit N.
   Pratap, Arun
BE Singhal, RK
TI Glass forming ability of Ca-based bulk metallic glasses
SO LATEST TRENDS IN CONDENSED MATTER PHYSICS: EXPERIMENTAL AND THEORETICAL
   ASPECTS
SE Solid State Phenomena
AB Knowledge of glass forming ability (GFA) of amorphous metallic alloys is very important from both theoretical and practical point of view. Thermodynamically, the Gibbs free energy difference, Delta G between the undercooled liquid and the corresponding crystalline state is driving force for crystallization. As a consequence, it is a good indicator for glass forming ability of metallic glasses. A novel expression for Delta G has been used to estimate the GFA of recently developed Ca-based bulk metallic glasses viz. Ca(53)Mg(23)Cu(24),Ca(65)Mg(15)Cu(20),Ca(40)Mg(25)Cu(35), Ca(50)Mg(22.5)Cu(27.5) and Ca(55)Mg(15)Cu(30). Different GFA criteria are also evaluated for systems taken up in the study and effect of addition of variation in composition of Ca-Mg-Cu system is also investigated. Present work suggests that among different GFA criteria, Delta G is the best criterion for the prediction of GFA for Ca-based bulk metallic glasses.
OI Lad, Kirit/0000-0003-2367-2208
SN 1012-0394
PY 2011
VL 171
BP 121
EP 126
DI 10.4028/www.scientific.net/SSP.171.121
UT WOS:000294789200009
ER

PT J
AU Luo, JH
   Wu, FF
   Huang, JY
   Wang, JQ
   Mao, SX
AF Luo, J. H.
   Wu, F. F.
   Huang, J. Y.
   Wang, J. Q.
   Mao, S. X.
TI Superelongation and Atomic Chain Formation in Nanosized Metallic Glass
SO PHYSICAL REVIEW LETTERS
AB Bulk metallic glasses are brittle and fail with no plastic strain at room temperature once shear bands propagate. How do metallic glasses deform when the size is less than that of shear bands? Here we show that Al(90)Fe(5)Ce(5) metallic glass with a size <20 nm can be extremely elongated to similar to 200%. Remarkably, even an atomic chain was formed after sample necking, which was never observed in metallic glasses. The unexpected ductility may originate from the fast surface diffusion and the absence of shear band formation, and may guide the development of ductile metallic glasses for engineering applications.
RI Huang, Jianyu/C-5183-2008
SN 0031-9007
PD MAY 28
PY 2010
VL 104
IS 21
AR 215503
DI 10.1103/PhysRevLett.104.215503
UT WOS:000278150100038
PM 20867114
ER

PT J
AU Sandor, MT
   Kecskes, LJ
   He, Q
   Xu, J
   Wu, Y
AF Sandor, Magdalena T.
   Kecskes, Laszlo J.
   He Qiang
   Xu Jian
   Wu Yue
TI Correlation of mechanical properties in bulk metallic glasses with Al-27
   NMR characteristics
SO CHINESE SCIENCE BULLETIN
AB We report Al-27 NMR and magnetic susceptibility measurements of Zr and ZrHf-based bulk metallic glasses (BMGs). Al-27 NMR Knight shift shows that there exists a clear correlation between the local electronic properties at Al sites and mechanical properties. In addition, magnetic susceptibility measurements also provide clues on the influence of the electronic states, especially the strong influence of d-orbital characteristics on the mechanical properties of toughness and hardness.
RI Kecskes, Laszlo/F-6880-2014
OI Kecskes, Laszlo/0000-0002-1342-3729
SN 1001-6538
PD DEC
PY 2011
VL 56
IS 36
BP 3937
EP 3941
DI 10.1007/s11434-011-4834-z
UT WOS:000297866900009
ER

PT J
AU Louzguine, DV
   Inoue, A
AF Louzguine, DV
   Inoue, A
TI Nanocrystallization of Cu-(Zr or Hf)-Ti metallic glasses
SO JOURNAL OF MATERIALS RESEARCH
AB Crystallization of the Cu60Zr30Ti10 and Cu60Hf25Ti15 metallic glasses was studied by x-ray diffractometry, transmission electron microscopy, differential scanning and isothermal calorimetries. Metastable Cu-Zr-Ti and Cu-Hf-Ti cubic phases primarily precipitated in the Cu60Zr30Ti10 and Cu60Hf25Ti15 metallic glasses. The Cu60Zr30Ti10 metallic glass crystallizes with low energy barrier for nucleation while crystallization of the Cu60Hf25Ti15 metallic glass takes place by nucleation and diffusion-controlled growth of cubic Cu-Hf-Ti phase particles with constant nucleation rate. The Cu60Hf25Ti15 metallic glass is characterized by a low activation energy for nucleation.
RI LOUZGUINE, Dmitri/D-2492-2010; Inoue, Akihisa/E-5271-2015
OI LOUZGUINE, Dmitri/0000-0001-5716-4987;
SN 0884-2914
PD AUG
PY 2002
VL 17
IS 8
BP 2112
EP 2120
DI 10.1557/JMR.2002.0312
UT WOS:000177208800037
ER

PT J
AU Gao, XQ
   Zhao, K
   Ke, HB
   Ding, DW
   Wang, WH
   Bai, HY
AF Gao, X. Q.
   Zhao, K.
   Ke, H. B.
   Ding, D. W.
   Wang, W. H.
   Bai, H. Y.
TI High mixing entropy bulk metallic glasses
SO JOURNAL OF NON-CRYSTALLINE SOLIDS
AB Bulk metallic glasses (BMGs) are usually based on a single principal element such as Zr, Cu, Mg and Fe. In this work, we report the formation of a series of high mixing entropy BMGs based on multiple major elements, which have unique characteristics of excellent glass-forming ability and mechanical properties compared with conventional BMGs. The high mixing entropy BMGs based on multiple major elements might be of significance in scientific studies, potential applications, and providing a novel approach in search for new metallic glass-forming systems. (C) 2011 Elsevier B.V. All rights reserved.
SN 0022-3093
EI 1873-4812
PD NOV 1
PY 2011
VL 357
IS 21
BP 3557
EP 3560
DI 10.1016/j.jnoncrysol.2011.07.016
UT WOS:000295564400001
ER

PT J
AU Li, YH
   Zhang, W
   Dong, C
   Qiang, JB
   Makino, A
AF Li, Yan-hui
   Zhang, Wei
   Dong, Chuang
   Qiang, Jian-bing
   Makino, Akihiro
TI Correlation between the glass-forming ability and activation energy of
   crystallization for Zr75-x Ni25Al (x) metallic glasses
SO INTERNATIONAL JOURNAL OF MINERALS METALLURGY AND MATERIALS
AB The thermal stability and the kinetics of glass transition and crystallization for Zr75-x Ni25Al (x) (x = 8-15) metallic glasses were investigated using differential scanning calorimetry (DSC) under continuous heating conditions. The apparent activation energy of glass transition rises monotonously with the Al content increasing; the activation energy of crystallization increases with Al changing from 8at% to 15at%, and then decreases with Al further up to 24at%, which exhibits a good correlation to the thermal stability and the glass-forming ability (GFA). The Zr60Ni25Al15 metallic glass with the largest supercooled liquid region and GFA possesses the highest activation energy of crystallization. The relation between the thermal stability, GFA and activation energy of crystallization was discussed in terms of the primary precipitated phases.
RI MAKINO, AKIHIRO/B-2549-2009
SN 1674-4799
PD MAY
PY 2013
VL 20
IS 5
BP 445
EP 449
DI 10.1007/s12613-013-0749-9
UT WOS:000319386600006
ER

EF
```
