# Supplementary material for: Probabilistic Assessment of Glass Forming Ability Rules for Metallic Glasses Aided by Automated Analysis of Phase Diagrams
Source: Sci Rep. 2019 Jan 23;9:357. doi: 10.1038/s41598-018-36224-3 (PMC6344582; doi:10.1038/s41598-018-36224-3)
Supplement: Supplementary file 2 [file 41598_2018_36224_MOESM2_ESM.docx]

FN Clarivate Analytics Web of Science

VR 1.0

PT J

AU Tarigan, HJ

Kahler, N

Ramos, NS

Kumar, G

Bernussi, AA

AF Tarigan, Hendra J.

Kahler, Niloofar

Ramos, Nelson Sepulveda

Kumar, Golden

Bernussi, Ayrton A.

TI Low reflectance of nano-patterned Pt-Cu-Ni-P bulk metallic glass

SO APPLIED PHYSICS LETTERS

AB Materials that exhibit very high absorption and low reflectance at optical frequencies are important in a variety of light harvesting applications. We explore nano-scale topography of bulk metallic glasses to realize metallic surfaces with controllable reflectance, Diffuse reflectance as low as 1.6% in the visible range is achieved with nano-patterned Pt-based model bulk metallic glass without further optimization. Effect of pattern dimensions (size, spacing, and aspect-ratio) on the reflectance of bulk metallic glasses is investigated using a combination of simulation and experiments. (C) 2015 AIP Publishing LLC.

RI kumar, golden/F-5443-2010

OI Bernussi, Ayrton/0000-0003-1797-0145; Sepulveda-Ramos,

Nelson/0000-0002-2123-7125

SN 0003-6951

EI 1077-3118

PD JUL 13

PY 2015

VL 107

IS 2

AR 021903

DI 10.1063/1.4926873

UT WOS:000358530300011

ER

PT J

AU Yu, GS

Lin, JG

Li, W

Wen, CE

AF Yu, G. S.

Lin, J. G.

Li, W.

Wen, C. E.

TI Extrusion properties of a Zr-based bulk metallic glass

SO MATERIALS LETTERS

AB The extrusion behavior of Zr(41.2)Ti(13.8)Cu(12.5)Ni(10)Be(22.5) metallic glasses in the supercooled liquid region was investigated. Good extrusion formability was observed under low strain rates at temperatures higher than 395 degrees C. The metallic glasses were fully extruded without crystallization and failure within the range of T = 395-415 degrees C under strain rates from 5 x 10(-3) s(-1) to 5 x 10(-2) s(-1), and the deformation behavior of the metallic glasses during the extrusion was found to be in a Newtonian viscous flow mode by a strain rate sensitivity of 1.0. (C) 2009 Elsevier B.V. All rights reserved.

RI Wen, Cuie/I-2872-2012

OI Wen, Cuie/0000-0001-8008-3536

SN 0167-577X

PD JUN 15

PY 2009

VL 63

IS 15

BP 1317

EP 1319

DI 10.1016/j.matlet.2009.02.058

UT WOS:000265906700027

ER

PT J

AU Hua, NB

Li, R

Wang, JF

Zhang, T

AF Hua NengBin

Li Ran

Wang JianFeng

Zhang Tao

TI Biocompatible Zr-Al-Fe bulk metallic glasses with large plasticity

SO SCIENCE CHINA-PHYSICS MECHANICS & ASTRONOMY

AB In the present study, high-zirconium ternary Zr-Al-Fe bulk metallic glasses (BMGs) with low Young's modulus and good plasticity were developed. Zr75Al7.5Fe17.5 BMG exhibits a low Young's modulus of 70 GPa and high Poisson's ratio of 0.403. Pronounced plasticity was demonstrated under both compression and bending conditions for the BMGs. Furthermore, the alloys show high corrosion resistance in phosphate buffered solution. The combination of desirable mechanical and chemical properties implies potential for biomedical applications.

RI Zhang, Tao/O-4911-2014; Li, Ran/B-4618-2010; BAI, JIE/D-7448-2016

SN 1674-7348

PD SEP

PY 2012

VL 55

IS 9

BP 1664

EP 1669

DI 10.1007/s11433-012-4831-5

UT WOS:000307719500022

ER

PT S

AU Li, YH

Wang, Q

Wu, J

Dong, C

AF Li, Y. H.

Wang, Q.

Wu, J.

Dong, C.

BE Popel, PS

TI Formation of Cu-Zr-M ternary bulk metallic glasses based on atomic

clusters

SO 13TH INTERNATIONAL CONFERENCE ON LIQUID AND AMORPHOUS METALS

SE JOURNAL OF PHYSICS CONFERENCE SERIES

CT 13th Conference on Liquid and Amorphous Metals

CY JUL 08-14, 2007

CL Ekaterinburg, RUSSIA

AB Ternary Cu-Zr-M (M = Al, Ti and Ag) bulk metallic glasses are investigated using a cluster line approach. New bulk metallic glass rods with compositions lying along the cluster line Cu5Zr6-M were fabricated by copper mould suction, where binary cluster Cu5Zr6 is an Archimedean octahedral antiprism, M being about 4 similar to 13.2 at.% for Al, 8.3 at.% for Ti and 9 at.% for Ag. The relevant mechanism was discussed in the light of the cluster-plus-glue-atom model.

SN 1742-6588

PY 2008

VL 98

BP U33

EP U36

DI 10.1088/1742-6596/98/1/012006

UT WOS:000259363800006

ER

PT J

AU Liang, L

Hui, X

Zhang, CM

Lu, ZP

Chen, GL

AF Liang, L.

Hui, X.

Zhang, C. M.

Lu, Z. P.

Chen, G. L.

TI A novel Ho36Dy20Al24Co20 bulk metallic glass with large magnetocaloric

effect

SO SOLID STATE COMMUNICATIONS

AB A new heavy rare-earth-based Ho36Dy20Al24Co20 bulk metallic glass (BMG) has been prepared by a copper mold casting. A maximum inagnetic entropy change of 11.77 J/kg K has been measured in Ho36Dy20Al24Co20 bulk metallic glass under a magnetic field of 5 Tesla, which is larger than that of the early reported Ho-based bulk metallic glass Ho30Y26Al24Co20. The half-maximum temperature range of the entropy change peak is as large as40 K, leading to a superior refrigerant capacity to those of the Gd5Si2Ge2 and Gd(5)Si(2)Ge(1.9)Fe(0.1)crystalline compounds. The excellent magnetocaloric effect together with tile unique properties of metallic glass makes this alloy a promising candidate for a magnetic refrigerant in the temperature range below 50 K. (c) 2008 Elsevier Ltd. All rights reserved.

RI Lu, Zhao-Ping/A-2718-2009; Hui, Xidong/A-1741-2010

OI Lu, Zhao-Ping/0000-0003-1463-8948;

SN 0038-1098

PD APR

PY 2008

VL 146

IS 1-2

BP 49

EP 52

DI 10.1016/j.ssc.2008.01.031

UT WOS:000254806500011

ER

PT J

AU Tamura, T

Kamikihara, D

Omura, N

Miwa, K

AF Tamura, Takuya

Kamikihara, Daisuke

Omura, Naoki

Miwa, Kenji

TI Effect of frequency of electromagnetic vibrations on glass-forming

ability in Fe-Co-B-Si-Nb bulk metallic glasses

SO REVIEWS ON ADVANCED MATERIALS SCIENCE

CT 13th International Symposium on Metastable and Nano-Materials

(ISMANAM-2006)

CY AUG 27-31, 2006

CL Warsaw Univ Technol, Fac Mat Sci & Engn, Warsaw, POLAND

HO Warsaw Univ Technol, Fac Mat Sci & Engn

AB It is known that cooling rate from the liquid state is an important factor for producing the bulk metallic glasses. However, almost no other factors such as electric and/or magnetic fields were investigated. The present authors have reported that the glass-forming ability of Mg-Cu-Y and Fe-Co-B-Si-Nb alloys is enhanced with increasing the electromagnetic vibration force. This study aims to investigate effect of the frequency of the electromagnetic vibrations on glass forming ability in Fe-Co-B-Si-Nb bulk metallic glasses. As a result, it was found that the electromagnetic vibrations affect the increase in the cooling rate and the decrease in the number of crystal nuclei directly.

RI Tamura, Takuya/D-4854-2017; Omura, Naoki/M-1949-2018

OI Tamura, Takuya/0000-0001-6083-913X;

SN 1606-5131

PD MAY

PY 2008

VL 18

IS 1

BP 10

EP 13

UT WOS:000256370800004

ER

PT J

AU Ponnambalam, V

Poon, SJ

Shiflet, GJ

AF Ponnambalam, V

Poon, SJ

Shiflet, GJ

TI Fe-based bulk metallic glasses with diameter thickness larger than one

centimeter

SO JOURNAL OF MATERIALS RESEARCH

AB Fe-Cr-Mo-(Y,Ln)-C-B bulk metallic glasses (Ln are lanthanides) with maximum diameter thicknesses reaching 12 mm have been obtained by casting. The high glass formability is attained despite a low reduced glass transition temperature of 0.58. The inclusion of Y/Ln is motivated by the idea that elements with large atomic sizes can destabilize the competing crystalline phase, enabling the amorphous phase to be formed. It is found that the role of Y/Ln as a fluxing agent is relatively small in terms of glass formability enhancement. The obtained bulk metallic glasses are non-ferromagnetic and exhibit high elastic moduli of approximately 180-200 GPa and microhardness of approximately 13 GPa.

SN 0884-2914

PD MAY

PY 2004

VL 19

IS 5

BP 1320

EP 1323

DI 10.1557/jmr.2004.0176

UT WOS:000222316500005

ER

PT J

AU Yang, YJ

Kang, FW

Xing, DW

Sun, JF

Shen, QK

Shen, J

AF Yang Ying-jun

Kang Fu-wei

Xing Da-wei

Sun Jian-fei

Shen Qing-ke

Shen Jun

TI Formation and mechanical properties of bulk Cu-Ti-Zr-Ni metallic glasses

with high glass forming ability

SO TRANSACTIONS OF NONFERROUS METALS SOCIETY OF CHINA

AB Bulk amorphous CU52.5Ti30Zr11.5Ni6 and CU53.1Ti31.4Zr9.5Ni6 alloys with a high glass forming ability can be quenched into single amorphous rods with a diameter of 5 mm, and exhibit a high fracture strength of 2 212 MPa and 2 184 MPa under compressive condition, respectively. The stress-strain curves show nearly 2% elastic strain limit, yet display no appreciable macroscopic plastic deformation prior to the catastrophic fracture due to highly localized shear bands. The present work shows clearly evidence of molten droplets besides well-developed vein patterns typical of bulk metallic glasses on the fracture surface, suggesting that localized melting induced by adiabatic heating may occur during the final failure event.

SN 1003-6326

PD JAN

PY 2007

VL 17

IS 1

BP 16

EP 20

DI 10.1016/S1003-6326(07)60041-7

UT WOS:000245096000003

ER

PT J

AU Yavari, AR

de Oliveira, MF

Kiminami, CS

Inoue, A

Botta, WJF

AF Yavari, AR

de Oliveira, MF

Kiminami, CS

Inoue, A

Botta, WJF

TI Electromechanical shaping, assembly and engraving of bulk metallic

glasses

SO MATERIALS SCIENCE AND ENGINEERING A-STRUCTURAL MATERIALS PROPERTIES

MICROSTRUCTURE AND PROCESSING

CT 11th International Conference on Rapidly Quenched and Metastable

Materials

CY AUG 25-30, 2002

CL Univ Oxford, Dept Mat, Oxford, ENGLAND

HO Univ Oxford, Dept Mat

AB Using the intrinsic materials properties of bulk metallic glasses (BMGs), namely electrical resistivities two orders of magnitude higher than good conductors and a Newtonian viscous-flow regime of deformability, a new electromechanical process has been developed for shaping, joining and engraving of BMGs. Viscous flow in the supercooled liquid region between the glass transition temperature T, and the crystallisation temperature T-x of the bulk metallic glass allows rapid electromechanical shaping, joining and engraving at low applied stresses. The process parameters are adaptable for the full maintenance of the glassy state. Miniaturisation of the process is expected to allow sub-millimetre range manipulation and submicron-engraving. (C) 2003 Published by Elsevier B.V.

RI Inoue, Akihisa/E-5271-2015; KIMINAMI, CLAUDIO/D-4402-2012; yavari,

alain/E-8192-2010; Botta, Walter/E-7763-2010; de Oliveira,

Marcelo/B-9881-2012

OI KIMINAMI, CLAUDIO/0000-0001-8231-7316; Botta,

Walter/0000-0003-2759-573X; de Oliveira, Marcelo/0000-0003-4589-2463

SN 0921-5093

PD JUL 15

PY 2004

VL 375

SI SI

BP 227

EP 234

DI 10.1016/j.msea.2003.10.267

UT WOS:000223329700033

ER

PT J

AU Zhang, Y

Zhao, DQ

Wang, RJ

Pan, MX

Wang, WH

AF Zhang, Y

Zhao, DQ

Wang, RJ

Pan, MX

Wang, WH

TI Glass forming ability and properties of Zr/Nb-based bulk metallic

glasses

SO MATERIALS TRANSACTIONS JIM

AB Zr/Nb-based bulk metallic glasses (BMGs) with excellent glass forming ability (GFA) and high thermal stability were obtained by water quenching method. The GFA of the alloys is sensitive to the Fe addition, and the highest GFA is achieved at 8 at% of Fe addition. It is found that dr defined by the difference between the onset temperature of the first crystallization event T-x and the glass transition temperature T-g (DeltaT = T-x - T-g) is more effective than T-rg (T-rg = T-g/T-m T-m, melting temperature) to reflect the GFA of the Zr/Nb-based BMGs. The elastic properties of the alloys are investigated by ultrasonic measurements.

RI ZHANG, Yong/B-7928-2009

OI ZHANG, Yong/0000-0002-6355-9923

SN 0916-1821

PD NOV

PY 2000

VL 41

IS 11

BP 1423

EP 1426

DI 10.2320/matertrans1989.41.1423

UT WOS:000166559500010

ER

PT J

AU Bian, Z

Zhang, T

Zhang, W

Inoue, A

AF Bian, Z

Zhang, T

Zhang, W

Inoue, A

TI A new soft magnetic bulk metallic glass with dual glass phases

SO MATERIALS TRANSACTIONS

AB Bulk metallic glass composites with dual amorphous phases and soft magnetic properties were produced successfully by using hot-pressed technique. Zr-based amorphous powder was mixed homogeneously with Fe-based amorphous powder, and then hot-pressed in their liquid supercooled region. The compacts still display dual amorphous phase structure, and continuous viscosity connection among different amorphous phases. The magnetization curve shows that the structures still exhibit soft magnetic properties originating from the addition of the soft magnetic phase. The successful processing of new bulk metallic glasses (BMGs) with dual amorphous phases gives us a new design concept for new materials, especially for new glass function materials making use of unique softening characteristics of BMG alloys in their liquid supercooled regions.

RI Inoue, Akihisa/E-5271-2015; Zhang, Tao/O-4911-2014

SN 1345-9678

EI 1347-5320

PD NOV

PY 2003

VL 44

IS 11

BP 2410

EP 2413

DI 10.2320/matertrans.44.2410

UT WOS:000186992700028

ER

PT J

AU Yuan, CC

Xiang, JF

Xi, XK

Wang, WH

AF Yuan, C. C.

Xiang, J. F.

Xi, X. K.

Wang, W. H.

TI NMR Signature of Evolution of Ductile-to-Brittle Transition in Bulk

Metallic Glasses

SO PHYSICAL REVIEW LETTERS

AB The mechanical properties of monolithic metallic glasses depend on the structures at atomic or subnanometer scales, while a clear correlation between mechanical behavior and structures has not been well established in such amorphous materials. In this work, we find a clear correlation of Al-27 NMR isotropic shifts with a microalloying induced ductile-to-brittle transition at ambient temperature in bulk metallic glasses, which indicates that the Al-27 NMR isotropic shift can be regarded as a structural signature to characterize plasticity for this metallic glass system. The study provides a compelling approach for investigating and understanding the mechanical properties of metallic glasses from the point of view of electronic structure.

SN 0031-9007

EI 1079-7114

PD NOV 30

PY 2011

VL 107

IS 23

AR 236403

DI 10.1103/PhysRevLett.107.236403

UT WOS:000297501900021

PM 22182108

ER

PT J

AU Chen, N

Martin, L

Luzguine-Luzgin, DV

Inoue, A

AF Chen, Na

Martin, Laura

Luzguine-Luzgin, Dmitri V.

Inoue, Akihisa

TI Role of Alloying Additions in Glass Formation and Properties of Bulk

Metallic Glasses

SO MATERIALS

AB Alloying addition, as a means of improving mechanical properties and saving on costs of materials, has been applied to a broad range of uses and products in the metallurgical fields. In the field of bulk metallic glasses (BMGs), alloying additions have also proven to play effective and important roles in promoting glass formation, enhancing thermal stability and improving plasticity of the materials. Here, we review the work on the role of alloying additions in glass formation and performance improvement of BMGs, with focus on our recent results of alloying additions in Pd-based BMGs.

RI Inoue, Akihisa/E-5271-2015; Chen, Na/A-4120-2010

SN 1996-1944

PD DEC

PY 2010

VL 3

IS 12

BP 5320

EP 5339

DI 10.3390/ma3125320

UT WOS:000298243300020

PM 28883386

ER

PT J

AU Zhang, ZY

Keppens, V

Senkov, ON

Miracle, DB

AF Zhang, Zhiying

Keppens, Veerle

Senkov, Oleg N.

Miracle, Daniel B.

TI Elastic properties of Ca-based bulk metallic glasses studied by resonant

ultrasound spectroscopy

SO MATERIALS SCIENCE AND ENGINEERING A-STRUCTURAL MATERIALS PROPERTIES

MICROSTRUCTURE AND PROCESSING

AB We report measurements of the elastic properties and internal friction of Ca-based bulk metallic glasses (BMGs), Ca50Mg20Cu30, Ca55Mg18Zn11Cu16 and Ca65Mg15Zn2O (numbers indicate at.%), as a function of temperature between 5 and 400K. Below the glass transition temperature, both Young's modulus and shear modulus decrease with increasing temperature, and the Poisson ratio increases with increasing temperature. Above the glass transition temperature, these trends reverse due to crystallization. At temperatures close to the glass transition temperature, we observe the onset of a peak in the internal friction Q(-1), attributed to crystallization. (C) 2007 Elsevier B.V. All rights reserved.

RI Senkov, Oleg/C-7197-2012

OI Senkov, Oleg/0000-0001-5587-415X; Senkov, Oleg/0000-0002-9336-3702

SN 0921-5093

PD DEC 15

PY 2007

VL 471

IS 1-2

BP 151

EP 154

DI 10.1016/j.msea.2007.01.157

UT WOS:000250168000023

ER

PT J

AU Zhao, YC

Kou, SZ

Wang, RJ

Suo, HL

Ding, YT

Li, N

Xue, SW

Liu, GQ

AF Zhao Yanchun

Kou Shengzhong

Wang Renjun

Suo Hongli

Ding Yutian

Li Na

Xue Shuwei

Liu Guangqiao

TI Effect of Overheated Level on Thermal Stability and Mechanical

Properties of Cu36Zr48Ag8Al8 Bulk Metallic Glass

SO RARE METAL MATERIALS AND ENGINEERING

AB Overheating has a pronounced effect on the thermal stability and mechanical properties of Cu36Zr48Ag8Al8 bulk metallic glass. Higher overheated temperature can enhance the thermal stability of bulk amorphous alloys, corresponding to higher specific-heat capacity and smaller initial defect concentration. Bulk amorphous alloys exhibit good compressive plasticity at small overheated levels, whereas the compressive fracture strength and microhardness show a significant increase followed by a slight decrease. The mechanical properties of bulk metallic glasses fabricated by different casting process are influenced by the interaction of the free volume and residual stresses.

SN 1002-185X

PD JAN

PY 2011

VL 40

IS 1

BP 173

EP 176

UT WOS:000291966300031

ER

PT J

AU Li, HG

Lu, YZ

Qin, ZX

Lu, X

AF Li, Hongge

Lu, Yunzhuo

Qin, Zuoxiang

Lu, Xing

TI Vibrational properties of FeCoCrMoCSY bulk metallic glasses and their

correlation with glass-forming ability

SO VACUUM

AB Here, the authors focus on the relationships between the vibrational properties and glass-forming ability (GFA) of an Fe-Co-Cr-Mo-C-B-Y alloy system. Among the alloy systems studied, the best glass former Fe-41 Co7Cr15Mo14C15B36Y2 bulk metallic glass (BMG) (Co7 alloy) showed the maximum vibrational wave number of acoustic and optical modes. The origin of the excellent GFA of the Co7 alloy was discussed in terms of the atomic cluster size and bonding energy. (C) 2016 Elsevier Ltd. All rights reserved.

SN 0042-207X

PD NOV

PY 2016

VL 133

BP 105

EP 107

DI 10.1016/j.vacuum.2016.08.012

UT WOS:000385327000016

ER

PT J

AU Zeng, YQ

Inoue, A

Nishiyama, N

Chen, MW

AF Zeng, Y. Q.

Inoue, A.

Nishiyama, N.

Chen, M. W.

TI Ni-rich Ni-Pd-P bulk metallic glasses with significantly improved

glass-forming ability and mechanical properties by Si addition

SO INTERMETALLICS

AB We report the significant effect of a small amount of Si on glass formation and mechanical properties of Ni-based bulk metallic glasses. It was found that only 2 at.% silicon dramatically enhances the glass-forming ability of Ni-based Ni-Pd-P alloys and the critical sample diameters can be improved from smaller than 7 mm of Ni(60)Pd(20)P(20) to 15 mm of Ni(60)Pd(20)P(18)Si(2). The new Ni-based bulk metallic glass also exhibits excellent mechanical properties with both high strength and good compression plasticity. (C) 2010 Elsevier Ltd. All rights reserved.

RI Inoue, Akihisa/E-5271-2015; Nishiyama, Nobuyuki/C-8228-2015; CHEN,

Mingwei/A-4855-2010

OI CHEN, Mingwei/0000-0002-8274-3099; Chen, Mingwei/0000-0002-2850-8872

SN 0966-9795

PD SEP

PY 2010

VL 18

IS 9

BP 1790

EP 1793

DI 10.1016/j.intermet.2010.05.003

UT WOS:000280857400013

ER

PT J

AU Reisgen, U

Balashov, B

Holk, J

Geffers, C

Kuhn, U

AF Reisgen, U.

Balashov, B.

Holk, J.

Geffers, C.

Kuehn, U.

TI Electron-Beam-Welding of bulk metallic glasses

SO MATERIALWISSENSCHAFT UND WERKSTOFFTECHNIK

AB Because of their excellent mechano-technological properties bulk metallic glasses form a promising, relatively new class of materials. Due to their low thermal stability the weldability of bulk metallic glasses is subject to narrow limits, in case the joining zone shall be prevented from crystallization. The paper at hand describes the status-quo of the research work on electron beam welding of beryllium-free, zirconium-based bulk metallic glasses (Zr(52,5)Ti(5)Cu(17,9)Ni(14,6)Al(10)) carried out at the Welding and Joining Institute at RWTH Aachen University. So far, high quality joints free from defects could be produced, however, it has not been accomplished to avoid the crystallization of the joining zone completely. Further research is in progress.

SN 0933-5137

PD DEC

PY 2009

VL 40

IS 12

BP 882

EP 887

DI 10.1002/mawe.200900533

UT WOS:000273394300003

ER

PT J

AU Lo, YC

Chou, HS

Cheng, YT

Huang, JC

Morris, JR

Liaw, PK

AF Lo, Y. C.

Chou, H. S.

Cheng, Y. T.

Huang, J. C.

Morris, J. R.

Liaw, P. K.

TI Structural relaxation and self-repair behavior in nano-scaled Zr-Cu

metallic glass under cyclic loading: Molecular dynamics simulations

SO INTERMETALLICS

AB Bulk metallic glasses are generally regarded as highly brittle materials at room temperature, with deformation localized within a few principal shear bands. In this simulation work, it is demonstrated that when the Zr-Cu metallic glass is in a small size-scale, it can deform under cyclic loading in a semi-homogeneous manner without the occurrence of pronounced mature shear bands. Instead, the plastic deformation in simulated samples proceeds via the network-like shear-transition zones (STZs) by the reversible and irreversible structure-relaxations during cyclic loading. Dynamic recovery and reversible/ irreversible structure rearrangements occur in the current model, along with annihilation/creation of excessive free volumes. This behavior would in-turn retard the damage growth of metallic glass. Current studies can help to understand the structural relaxation mechanism in metallic glass under loading. The results also imply that the brittle bulk metallic glasses can become ductile with the sample size being reduced. The application of metallic glasses in the form of thin film or nano pieces in micro-electro-mechanical systems (MEMS) could be promising. (C) 2010 Elsevier Ltd. All rights reserved.

RI Huang, J./C-4276-2013; Cheng, Yu-Ting/K-7432-2013; Morris, J/I-4452-2012

OI Morris, J/0000-0002-8464-9047; HUANG, Jacob Chih

Ching/0000-0001-6843-3396

SN 0966-9795

PD MAY

PY 2010

VL 18

IS 5

BP 954

EP 960

DI 10.1016/j.intermet.2010.01.012

UT WOS:000277494400031

ER

PT S

AU Louzguine, DV

AF Louzguine (Luzgin), D. V.

BA Louzguine, DV

BF Louzguine, DV

TI Glass-transition process

SO METALLIC GLASSES AND THEIR COMPOSITES

SE Materials Research Foundations

AB Although, the glass-transition phenomenon in metallic glasses has been studied extensively there are still considerable uncertainties in this field of materials science. There are some gaps in obtaining a clear picture of the glass transition. Two general mechanisms of glass formation by limiting crystal nucleation and by limiting their growth will be discussed in the present Chapter.

SN 2471-8890

EI 2471-8904

BN 978-1-945291-42-5

PY 2018

VL 19

BP 19

EP 67

UT WOS:000417007800002

ER

PT J

AU Babilas, R

Cesarz-Andraczke, K

Nowosielski, R

Burian, A

AF Babilas, Rafa

Cesarz-Andraczke, Katarzyna

Nowosielski, Ryszard

Burian, Andrzej

TI Structure, Properties, and Crystallization of Mg-Cu-Y-Zn Bulk Metallic

Glasses

SO JOURNAL OF MATERIALS ENGINEERING AND PERFORMANCE

AB The Mg60Cu30Y10 and Mg65Cu20Y10Zn5 bulk metallic glasses in the form of a rod 2 mm in diameter were successfully prepared by the conventional Cu-mold casting method. The addition of Zn caused the decrease in the crystallization and melting temperatures in comparison with the Mg60Cu30Y10 alloy. The crystallization and melting temperatures are crucial factors that influence the casting process. An increase in annealing temperature leads to structural changes by the formation of the crystalline phases and lowers the compressive strength. These results obtained for the Mg-based bulk metallic glasses (Mg-BMGs) are important for some practical reasons, in particular, for developing the fabrication process. It has been shown that minor addition of an alloying element can change glass-forming ability and strength of the Mg-BMGs.

SN 1059-9495

EI 1544-1024

PD JUN

PY 2014

VL 23

IS 6

BP 2241

EP 2246

DI 10.1007/s11665-014-0972-1

UT WOS:000336393300036

ER

PT S

AU Kasyap, S

Patel, AT

Pratap, A

AF Kasyap, Supriya

Patel, Ashmi T.

Pratap, Arun

BE Bhardwaj, S

Shekhawat, MS

Suthar, B

TI Effect of Micro Alloying on Glass Forming Ability of Cu-50 Zr-50

Metallic Glass

SO PROCEEDING OF INTERNATIONAL CONFERENCE ON RECENT TRENDS IN APPLIED

PHYSICS & MATERIAL SCIENCE (RAM 2013)

SE AIP Conference Proceedings

CT International Conference on Recent Trends in Applied Physics and

Material Science (RAM)

CY FEB 01-02, 2013

CL Govt Coll Engn & Technol Bikaner, Bikaner, INDIA

SP Dept Sci & Technol (DST), New Delhi, Dept Sci & Technol (DST), Jaipur, Council Sci & Ind Res (CSIR)

HO Govt Coll Engn & Technol Bikaner

AB The sensitivity of glass forming ability (GFA) of Cu50Zr50 metallic glass towards addition of Nb and Al has been studied. It has been found that the GFA of Cu50Zr50 ribbons increases with addition of Nb and Al, and highest GFA is achieved at 4% Al. Moreover Al addition favors the formation of Bulk Metallic Glasses (BMG). Among various GFA parameters (Delta G, T-rg, gamma, gamma(m), eta, Delta T-x,T- Q), it is the Gibbs free energy difference (Delta G), between the under cooled liquid and the corresponding crystalline phase, that best explains this variation in GFA.

SN 0094-243X

BN 978-0-7354-1160-9

PY 2013

VL 1536

BP 651

EP 652

DI 10.1063/1.4810395

UT WOS:000320861200320

ER

PT J

AU Fu, HM

Zhang, HF

Wang, H

Zhang, QS

Hu, ZQ

AF Fu, HM

Zhang, HF

Wang, H

Zhang, QS

Hu, ZQ

TI Synthesis and mechanical properties of Cu-based bulk metallic glass

composites containing in-situ TiC particles

SO SCRIPTA MATERIALIA

AB Cu-based bulk metallic glass matrix composites (BMGMCs) containing in-situ TiC particles were fabricated successfully. The yield and fracture strength increased from 1930 MPa, 2250 MPa to 2210 MPa, 2500 MPa, respectively. The ductility was improved and the hardness was also enhanced by 25%. The fracture mechanism was investigated in detail. (C) 2004 Acta Materialia Inc. Published by Elsevier Ltd. All rights reserved.

RI zhang, qingsheng/A-4851-2010

SN 1359-6462

PD APR

PY 2005

VL 52

IS 7

BP 669

EP 673

DI 10.1016/j.scriptamat.2004.10.031

UT WOS:000226874200023

ER

PT J

AU Wang, JQ

Wang, WH

Yu, HB

Bai, HY

AF Wang, J. Q.

Wang, W. H.

Yu, H. B.

Bai, H. Y.

TI Correlations between elastic moduli and molar volume in metallic glasses

SO APPLIED PHYSICS LETTERS

AB We report clear correlations between bulk modulus (K) and average molar volume V-m, and between Poisson's ratio nu and V-m for various bulk metallic glasses. The origin for the correlations between elastic moduli and V-m are discussed. The established correlation, associated with Poisson's ratio nu, and since the nu correlates with plasticity of metallic glasses, indicates that the average molar volume is important factor to be considered for plastic metallic glasses searching. The found correlations also suggest a close relation between the mechanical properties and the short-range atomic bonding, and assist in understanding deformation behavior in metallic glasses.

RI Wang, Jun-Qiang/G-5989-2010; Yu, Hai Bin/E-5312-2010; Wang,

Junqiang/C-2839-2015

OI Yu, Hai Bin/0000-0003-0645-0187; Wang, Junqiang/0000-0002-8066-6237

SN 0003-6951

EI 1077-3118

PD MAR 23

PY 2009

VL 94

IS 12

AR 121904

DI 10.1063/1.3106110

UT WOS:000264633500011

ER

PT S

AU Sun, YJ

Wu, RG

Wang, HJ

AF Sun, Yajuan

Wu, Riga

Wang, Hongjing

BE Jian, L

TI Compressive Plasticity of Zr-Based Bulk Metallic Glass at Low

Temperature

SO MANUFACTURING SCIENCE AND MATERIALS ENGINEERING, PTS 1 AND 2

SE Advanced Materials Research

CT International Conference on Manufacturing Science and Materials

Engineering (ICMSME 2011)

CY OCT 14-15, 2011

CL Shanghai, PEOPLES R CHINA

AB The mechanical properties of a new Zr-based bulk metallic glass at low temperatures were investigated. The results indicate that the fracture strength increases significantly (4.9%) and the global plasticity increases somewhat when testing temperature is lowered to 123K. The stress-strain curve of the sample deformed exhibits more serrations and smaller stress drop due to formation of more shear bands at low temperature than at room temperature.

SN 1022-6680

BN 978-3-03785-289-7

PY 2012

VL 443-444

BP 583

EP +

DI 10.4028/www.scientific.net/AMR.443-444.583

UT WOS:000308895800101

ER

PT S

AU Hays, CC

Schroers, J

Geyer, U

Bossuyt, S

Stein, N

Johnson, WL

AF Hays, CC

Schroers, J

Geyer, U

Bossuyt, S

Stein, N

Johnson, WL

BE Eckert, J

Schlorb, H

Schultz, L

TI Glass forming ability in the Zr-Nb-Ni-Cu-Al bulk metallic glasses

SO METASTABLE, MECHANICALLY ALLOYED AND NANOCRYSTALLINE MATERIALS, PTS 1

AND 2

SE MATERIALS SCIENCE FORUM

CT International Symposium on Metastable, Mechanically Alloyed and

Nanocrystalline Materials (ISMANAM-99)

CY AUG 30-SEP 03, 1999

CL DRESDEN, GERMANY

SP Deutsch Forech Gemeinsch, Inst Festkorper & Werkstofforsch Dresden, Sachsisches Minist Wissensch & Kunst, Fritsch GmbH, Stadtsparkasse Dresden, ZOZ GmbH

AB A Zr58.5Nb2.8Cu15.6Ni12.8Al10.3 (at.%) alloy was found to have an improved bulk glass forming ability compared to neighboring compositions in the Zr-Nb-Cu-Ni-Al phase diagram. This alloy is the first bulk glass forming liquid, that does not contain beryllium, to be vitrified by purely radiative cooling in the containerless electrostatic levitation process. Thus, the critical cooling rate for this alloy is less than approximate to 10 Ws. This composition is a prime example of how the glass forming ability can vary with the topology of the composition manifold for small changes in the Zr-Nb and Cu-Ni-Al ratios.

RI Hays, Charles/P-8021-2015; Bossuyt, Sven/H-5660-2012

OI Hays, Charles/0000-0002-0420-1761; Bossuyt, Sven/0000-0002-9223-735X

SN 0255-5476

BN 0-87849-858-3

PY 2000

VL 343-3

BP 103

EP 108

DI 10.4028/www.scientific.net/MSF.343-346.103

PN 1&2

UT WOS:000089403900017

ER

PT J

AU Li, Y

AF Li, Y

TI Formation, structure and properties of bulk metallic glasses

SO JOURNAL OF MATERIALS SCIENCE & TECHNOLOGY

AB Bulk metallic glasses with up to 72 mm critical section thickness have been obtained by conventional casting techniques and the properties of these materials, particularly the mechanical and magnetic properties have been studied. These materials have been demonstrated to have novel properties which are fundamentally different from their crystalline counterparts. The recent status of research and development in formation, structure and properties of bulk metallic glasses is reviewed. The techniques to produce such bulk glasses are summarized and the glass forming ability and the critical cooling rate of these materials are discussed. Further consideration of the development and application of this new class of materials will be proposed.

SN 1005-0302

PD MAR

PY 1999

VL 15

IS 2

BP 97

EP 110

UT WOS:000079552000001

ER

PT J

AU Wang, JQ

Wang, WH

Liu, YH

Bai, HY

AF Wang, J. Q.

Wang, W. H.

Liu, Y. H.

Bai, H. Y.

TI Characterization of activation energy for flow in metallic glasses

SO PHYSICAL REVIEW B

AB The molar volume (V-m) scaled flow activation energy (Delta E), namely as the activation energy density rho(E) = Delta E/V-m, is proposed to describe the flow of metallic glasses. Based on the energy landscape, both the shear and bulk moduli are critical parameters accounting for the rho(E) of both homogeneous and inhomogeneous flows in metallic glasses. The expression of rho(E) is determined experimentally to be a simple expression of rho(E) = 10/11G + 11/1K. The energy density perspective depicts a realistic picture for the flow in metallic glasses and is suggestive for understanding the glass transition and deformation in metallic glasses.

RI LIU, Yanhui/B-1485-2009; Wang, Jun-Qiang/G-5989-2010; Wang,

Junqiang/C-2839-2015

OI Wang, Junqiang/0000-0002-8066-6237

SN 2469-9950

EI 2469-9969

PD JAN 10

PY 2011

VL 83

IS 1

AR 012201

DI 10.1103/PhysRevB.83.012201

UT WOS:000286737900001

ER

PT J

AU Jiang, QK

Zhang, GQ

Chen, LY

Zeng, QS

Jiang, JZ

AF Jiang, Q. K.

Zhang, G. Q.

Chen, L. Y.

Zeng, Q. S.

Jiang, J. Z.

TI Centimeter-sized (La0.5Ce0.5)-based bulk metallic glasses

SO JOURNAL OF ALLOYS AND COMPOUNDS

AB Glass forming ability, thermal and mechanical properties of (La0.5Ce0.5)(64)Al16Ni5Cu15-xCox (x = 0, 3, 5, 8 and 10 at.%) bulk metallic glasses were investigated. Fully amorphous 1.2cm rods of the (La0.5Ce0.5)(64)Al16Ni5Cu10Co5 alloy and 1.0cm rods of (La0.5Ce0.5)(64)Al16Ni5Cu15-xCox (x = 0, 3, 5, 8 and 10 at.%) alloys are obtained. The (La0.5Ce0.5)(64)Al16Ni5Cu10Co5 alloy exhibits higher glass forming ability, lower glass transition temperature of 413 K, wider supercooled liquid region of 54 K, hardness and fracture strength of 2 GPa and 530 MPa, respectively, which could become potential micro- and nano-imprintable materials. (c) 2006 Published by Elsevier B.V.

RI Chen, Lianyi/B-3156-2008; Zeng, Qiaoshi/I-8688-2012

OI Chen, Lianyi/0000-0003-3720-398X; Zeng, Qiaoshi/0000-0001-5960-1378

SN 0925-8388

EI 1873-4669

PD NOV 9

PY 2006

VL 424

IS 1-2

BP 179

EP 182

DI 10.1016/j.jallcom.2006.07.007

UT WOS:000241414900033

ER

PT J

AU Li, YH

Zhang, W

Dong, C

Yamaura, S

Makino, A

AF Li, Y. H.

Zhang, W.

Dong, C.

Yamaura, S.

Makino, A.

TI Glass-Forming Ability, Corrosion Resistance and Mechanical Properties of

Zr60-xAl15Ni25TMx (TM = Nb and Ta) Bulk Metallic Glasses

SO MATERIALS TRANSACTIONS

CT 5th International Symposium on Designing, Processing and Properties of

Advanced Engineering Materials (ISAEM)

CY NOV 05-08, 2012

CL Toyohashi, JAPAN

SP Japan Soc Promot Sci, 176th Comm Proc Created Mat Funct

AB The thermal stability, glass-forming ability (GFA), corrosion resistance and mechanical properties of Zr60-xAl15Ni25TMx (TM = Nb and Ta, x = 0-6) bulk metallic glasses (BMGs) were investigated. The added Nb and Ta greatly increase the glass transition temperature of the base BMG whereas reduce the supercooled liquid region. Minor addition of Nb enhances the GFA of the base alloy, and Zr56Al15Ni25Nb4 BMG exhibits the largest critical diameter of 20 mm. Additions of Nb and Ta efficiently improve the corrosion resistance of the BMGs in chloride-ion-containing solutions due to the formation of highly protective surface film. In addition, the compressive yield strength of the BMGs increases gradually with increasing Nb and Ta contents.

RI MAKINO, AKIHIRO/B-2549-2009; Yamaura, Shin-ichi/A-9774-2011

SN 1345-9678

EI 1347-5320

PD AUG

PY 2013

VL 54

IS 8

BP 1368

EP 1372

DI 10.2320/matertrans.MF201316

UT WOS:000325122900024

ER

PT J

AU Park, BJ

Chang, HJ

Kim, DH

Kim, WT

Chattopadhyay, K

Abinandanan, TA

Bhattacharyya, S

AF Park, BJ

Chang, HJ

Kim, DH

Kim, WT

Chattopadhyay, K

Abinandanan, TA

Bhattacharyya, S

TI Phase separating bulk metallic glass: A hierarchical composite

SO PHYSICAL REVIEW LETTERS

AB Phase separating systems present a unique opportunity for designing composites with hierarchical microstructure at different length scales. We report here our success in synthesizing phase separating metallic glasses exhibiting the entire spectrum of microstructural possibilities expected from a phase separating system. In particular, we report novel core shell and hierarchical structures of spherical glassy droplets, resulting from critical wetting behavior and limited diffusion. We also report synthesis of a bulk phase separating glass in a metallic glass system. The combination of unique core shell and hierarchical structures in metallic glass systems opens a new avenue for the microstructure design of metallic glasses.

RI Abinandanan, Thennathur/B-5869-2009

OI Abinandanan, Thennathur/0000-0002-3918-7760

SN 0031-9007

PD JUN 23

PY 2006

VL 96

IS 24

AR 245503

DI 10.1103/PhysrevLett.96.245503

UT WOS:000238487900034

PM 16907253

ER

PT J

AU Xie, GQ

Qin, FX

Zhu, SL

AF Xie, Guoqiang

Qin, Fengxiang

Zhu, Shengli

TI Recent Progress in Ti-Based Metallic Glasses for Application as

Biomaterials

SO MATERIALS TRANSACTIONS

CT 5th International Symposium on Designing, Processing and Properties of

Advanced Engineering Materials (ISAEM)

CY NOV 05-08, 2012

CL Toyohashi, JAPAN

SP Japan Soc Promot Sci, 176th Comm Proc Created Mat Funct

AB Ti-based bulk metallic glasses are of great interest in biomedical applications due to their high corrosion resistance, excellent mechanical properties and good bi-ocompatibility. This article reviews recent progress in the development of Ti-based metallic glasses for the application as biomaterials. Ti-based (Ti-Zr-Cu-Pd, Ti-Zr-Cu-Pd-Sn, and Ti-Zr-Cu-Pd-Nb) bulk metallic glasses without toxic and allergic elements have been developed. These glassy alloys exhibited high glass-forming ability, high strength, large plasticity, good corrosion resistance and excellent biocompatibility, which open possibilities to create Ti-based metallic glass implants. Using a spark plasma sintering process, large-size Ti-based bulk metallic glasses and the composites with hydroxyapatite, as well as porous glassy alloys having approximate Young's modulus with that of bone were developed.

RI BAI, JIE/D-7448-2016; Zhu, Shengli/D-5281-2009; Xie,

Guoqiang/A-8619-2011

OI Zhu, Shengli/0000-0002-0190-2626;

SN 1345-9678

EI 1347-5320

PD AUG

PY 2013

VL 54

IS 8

BP 1314

EP 1323

DI 10.2320/matertrans.MF201315

UT WOS:000325122900014

ER

PT J

AU Csach, K

Lyakhov, SA

Khonik, VA

AF Csach, K.

Lyakhov, S. A.

Khonik, V. A.

TI Viscous flow recovery in bulk metallic glasses by heat treatment

SO TECHNICAL PHYSICS LETTERS

AB It is commonly accepted that the ability of metallic glasses to undergo viscous flow sharply and irreversibly drops with increasing duration and temperature of annealing, which is related to an increase in the degree of "irreversible" structural relaxation. The results of isothermal creep measurements show that this ability can be recovered almost completely by means of short-term heating to a temperature above that of the glass transition.

RI Khonik, Vitaly/A-5888-2009

SN 1063-7850

PD JUN

PY 2007

VL 33

IS 6

BP 498

EP 501

DI 10.1134/S1063785007060168

UT WOS:000247981600016

ER

PT J

AU Boucharat, N

Hebert, R

Rosner, H

Valiev, R

Wilde, G

AF Boucharat, N

Hebert, R

Rosner, H

Valiev, R

Wilde, G

TI Nanocrystallization of amorphous Al88Y7Fe5 alloy induced by plastic

deformation

SO SCRIPTA MATERIALIA

AB The crystallization behavior of rapidly quenched Al88Y7Fe5 glasses subjected to plastic deformation at room temperature and thermal treatments is comparatively analyzed. Both induce nanocrystallization. Severe plastic deformation strongly enhances the development of nanocrystals and thus presents a new synthesis route for bulk nanostructured materials. (c) 2005 Acta Materialia Inc. Published by Elsevier Ltd. All rights reserved.

RI Wilde, Gerhard/C-7808-2013

OI Wilde, Gerhard/0000-0001-8001-5998

SN 1359-6462

PD OCT

PY 2005

VL 53

IS 7

BP 823

EP 828

DI 10.1016/j.scriptamat.2005.06.004

UT WOS:000231341800007

ER

PT J

AU Tsyplakov, AN

Khonik, VA

Makarov, AS

Mitrofanov, YP

Afonin, GV

Kobelev, NP

Konchakov, RA

Lysenko, AV

AF Tsyplakov, A. N.

Khonik, V. A.

Makarov, A. S.

Mitrofanov, Yu P.

Afonin, G. V.

Kobelev, N. P.

Konchakov, R. A.

Lysenko, A. V.

TI On the nature of the shear viscosity and shear modulus relaxation in

metallic glasses

SO JOURNAL OF PHYSICS-CONDENSED MATTER

AB Analysis of independent isothermal and linear heating creep and shear modulus measurements performed on bulk Pd- and Zr-based metallic glasses provides evidence that the relaxation of their viscoelastic and elastic properties is controlled by 'defects', which respond to stress and temperature similarly to dumbbell interstitials in simple crystalline metals.

RI Konchakov, Roman/K-4616-2013; Mitrofanov, Yuriy/E-7963-2010; Afonin,

Gennady/K-1014-2013; Tsyplakov, Alexander/M-6132-2013; Makarov,

Andrey/H-4156-2013; Khonik, Vitaly/A-5888-2009

OI Mitrofanov, Yuriy/0000-0002-7939-5230; Afonin,

Gennady/0000-0002-7715-5065; Tsyplakov, Alexander/0000-0002-2579-6362;

Makarov, Andrey/0000-0001-6741-0619;

SN 0953-8984

PD AUG 28

PY 2013

VL 25

IS 34

AR 345402

DI 10.1088/0953-8984/25/34/345402

UT WOS:000322956200007

PM 23899581

ER

PT J

AU Kuchemann, S

Wagner, H

Schwabe, M

Bedorf, D

Arnold, W

Samwer, K

AF Kuechemann, Stefan

Wagner, Hannes

Schwabe, Moritz

Bedorf, Dennis

Arnold, Walter

Samwer, Konrad

TI Stored Mechanical Work in Inhomogeneous Deformation Processes of a

Pd-Based Bulk Metallic Glass

SO METALLURGICAL AND MATERIALS TRANSACTIONS A-PHYSICAL METALLURGY AND

MATERIALS SCIENCE

CT 10th Annual Meeting and Exhibition on Bulk Metallic Glasses

CY MAR 03-07, 2013

CL Minerals, Met & Mat Soc, San Antonio, TX

SP Minerals, Met & Mat Soc, Struct Mat Div, Amer Soc Met, Mech Behav Mat Comm

HO Minerals, Met & Mat Soc

AB We investigated the effect of anelastic rearrangements in a Pd-based metallic glass during inhomogeneous plastic deformation producing shear bands at room temperature. Therefore, we subjected bulk metallic glasses to uniaxial stresses and characterized the influence of deformation on the global configurational energy state with ultrasonic and calorimetric methods. The results provide evidence that even in an inhomogeneous plastic deformation process at room temperature, a certain amount of energy can be stored which is thermally relaxed below the glass transition temperature.

RI Arnold, Walter/G-4717-2011

OI Arnold, Walter/0000-0003-3588-8719

SN 1073-5623

EI 1543-1940

PD MAY

PY 2014

VL 45A

IS 5

BP 2389

EP 2392

DI 10.1007/s11661-013-1898-1

UT WOS:000334428000011

ER

PT J

AU Mechler, S

Schumacher, G

Zizak, I

Macht, MP

Wanderka, N

AF Mechler, S.

Schumacher, G.

Zizak, I.

Macht, M.-P.

Wanderka, N.

TI Correlation between icosahedral short range order, glass forming

ability, and thermal stability of Zr-Ti-Ni-Cu-(Be) glasses

SO APPLIED PHYSICS LETTERS

AB The structure and the crystallization behavior of the bulk metallic glass Zr46.8Ti8.2Ni10Cu7.5Be27.5 and of its Be-free derivative Zr64.5Ti11.4Ni13.8Cu10.3 are analyzed by means of differential scanning calorimetry, transmission electron microscopy, and x-ray diffraction. These alloys reveal different glass forming abilities. Thermal stability and crystallization behavior of the glasses are also different. These differences are correlated with the different degrees of icosahedral short range order in the glasses. Results fit to the model of geometric frustration, which describes the structure of metallic glasses as "highly defective quasicrystals." In the present case Be degrades the ideal quasicrystalline structure. (C) 2007 American Institute of Physics.

RI Zizak, Ivo/A-4661-2010

OI Zizak, Ivo/0000-0002-5959-0995

SN 0003-6951

EI 1077-3118

PD JUL 9

PY 2007

VL 91

IS 2

AR 021907

DI 10.1063/1.2755924

UT WOS:000248017300022

ER

PT J

AU Murali, P

Ramamurty, U

Shenoy, VB

AF Murali, P.

Ramamurty, U.

Shenoy, Vijay B.

TI Strain accommodation in inelastic deformation of glasses

SO PHYSICAL REVIEW B

AB Motivated by recent experiments on metallic glasses, we examine the micromechanisms of strain accommodation including crystallization and void formation during inelastic deformation of glasses by employing molecular statics simulations. Our atomistic simulations with Lennard-Jones-like potentials suggests that a softer short range interaction between atoms favors crystallization. Compressive hydrostatic strain in the presence of a shear strain promotes crystallization whereas a tensile hydrostatic strain is found to induce voids. The deformation subsequent to the onset of crystallization includes partial reamorphization and recrystallization, suggesting important atomistic mechanisms of plastic dissipation in glasses.

RI Palla, Murali/C-9146-2009; Ramamurty, Upadrasta/E-5623-2011

OI Palla, Murali/0000-0001-6470-4515;

SN 1098-0121

PD JAN

PY 2007

VL 75

IS 2

AR 024203

DI 10.1103/PhysRevB.75.024203

UT WOS:000243895100038

ER

PT J

AU Zhang, B

Zhao, DQ

Pan, M

Wang, RJ

Wang, WH

AF Zhang, B.

Zhao, D. Q.

Pan, Mx

Wang, R. J.

Wang, W. H.

TI Formation of cerium-based bulk metallic glasses

SO ACTA MATERIALIA

AB We report the formation and composition range of Ce-based bulk metallic glasses. Ternary Cc Al-Cu(Co,Ni) glassy rods of 1-3 mm in diameter can be easily formed in a wide composition range by a conventional copper mold cast method. Substituting Ce with low-cost Ce-rich misch metal (MM), MM-Al-Cu bulk glasses with a similar high glass-forming ability (GFA) can be obtained. With minor addition of extra elements such as Fe, Co, Ni, Nb, Zn and Si, the critical diameter of the full glassy rods of the Ce-Al-Cu matrix can be markedly enhanced from 2 mm to at least 3-10 mm. It is found that the often-cited empirical criteria for bulk metallic glass formation cannot interpret the formation and the addition effect on GFA of the metallic glasses. The striking effect and mechanism of microalloying on the GFA of the metallic glasses are studied. These materials with extremely low glass transition temperatures (341-439 K, even below the boiling temperature of water) and excellent deformability at low temperatures could have potential applications. (c) 2006 Acta Materialia Inc. Published by Elsevier Ltd. All rights reserved.

SN 1359-6454

EI 1873-2453

PD JUN

PY 2006

VL 54

IS 11

BP 3025

EP 3032

DI 10.1016/j.actamat.2006.02.044

UT WOS:000238468100014

ER

PT J

AU Li, X

Lv, F

Geng, YX

Qi, F

Xu, YJ

Liu, F

Wang, YX

AF Li, Xiang

Lv, Fang

Geng, Yaoxiang

Qi, Fang

Xu, Yingjie

Liu, Fang

Wang, Yuxin

TI Preparation and Corrosion Property of (Cu50Zr50)((100-x))Nd-x Amorphous

Alloy

SO INTERNATIONAL JOURNAL OF ELECTROCHEMICAL SCIENCE

AB Cu-Zr bulk metallic glasses (BMGs) have unique mechanical properties and developed rapidly in recent years. In this paper, a series of (Cu50Zr50)((100-x)) Nd-x (0 <= x <= 5at.%) alloys were prepared by using copper mold suction method. Their microstructure and properties were investigated by x-ray diffraction, differential scanning calorimetry, microhardness tester and electrochemical workstation. It was found that Cu49.5Zr49.5Nd1 alloy possesses the best glass forming ability. The addition of Nd element can effectively promote the glass forming ability (GFA) and microhardness of Cu-Zr-Nd alloys. The influence of Nd element on the corrosion resistance of Cu-Zr-Nd alloys was systematically discussed.

SN 1452-3981

PD JAN

PY 2017

VL 12

IS 1

BP 726

EP 732

DI 10.20964/2017.01.54

UT WOS:000396470000061

ER

PT J

AU Roberts, S

Zachrisson, C

Kozachkov, H

Ullah, A

Shapiro, AA

Johnson, WL

Hofmann, DC

AF Roberts, Scott

Zachrisson, Carl

Kozachkov, Henry

Ullah, Adam

Shapiro, Andrew A.

Johnson, William L.

Hofmann, Douglas C.

TI Cryogenic Charpy impact testing of metallic glass matrix composites

SO SCRIPTA MATERIALIA

AB Compact Charpy impact testing was employed to investigate the effect of low temperatures on the impact toughness of bulk metallic glass matrix composites (BMGMCs). Samples were fabricated via suction casting and impacted from room temperature down to liquid nitrogen temperature. Unlike monolithic glasses, BMGMCs do exhibit a steep decrease in toughness as the temperature is reduced from the ambient, caused by embrittlement in the ductile reinforcing phase. However, at cryogenic temperatures, BMGMCs have a toughness equivalent to their glassy matrix. (C) 2011 Acta Materialia Inc. Published by Elsevier Ltd. All rights reserved.

SN 1359-6462

PD MAR

PY 2012

VL 66

IS 5

BP 284

EP 287

DI 10.1016/j.scriptamat.2011.11.011

UT WOS:000300129200022

ER

PT J

AU Lee, CJ

Lai, YH

Huang, JC

Du, XH

Wang, L

Nieh, TG

AF Lee, C. J.

Lai, Y. H.

Huang, J. C.

Du, X. H.

Wang, L.

Nieh, T. G.

TI Strength variation and cast defect distribution in metallic glasses

SO SCRIPTA MATERIALIA

AB The yield strengths of micropillars of four metallic glasses were measured and subsequently analyzed using Weibull statistics. It was found that the strength variation scaled with the occurrence frequency and volume fraction of cast defects resulting from insufficient castability. The current finding is related to the size effect in bulk and micropillar metallic glass specimens. We also offered a theoretical estimate of the critical size of casting flaws in metallic glasses, above which fracture is expected to initiate. (C) 2010 Published by Elsevier Ltd. on behalf of Acta Materialia Inc.

RI Nieh, Tai-Gang/G-5912-2011; Huang, J./C-4276-2013

OI Nieh, Tai-Gang/0000-0002-2814-3746; HUANG, Jacob Chih

Ching/0000-0001-6843-3396

SN 1359-6462

PD JUL

PY 2010

VL 63

IS 1

BP 105

EP 108

DI 10.1016/j.scriptamat.2010.03.028

UT WOS:000277898900028

ER

PT J

AU Qin, CL

Zhao, WM

Inoue, A

AF Qin, Chunling

Zhao, Weimin

Inoue, Akihisa

TI Glass Formation, Chemical Properties and Surface Analysis of Cu-Based

Bulk Metallic Glasses

SO INTERNATIONAL JOURNAL OF MOLECULAR SCIENCES

AB This paper reviews the influence of alloying elements Mo, Nb, Ta and Ni on glass formation and corrosion resistance of Cu-based bulk metallic glasses (BMGs). In order to obtain basic knowledge for application to the industry, corrosion resistance of the Cu-Hf-Ti-(Mo, Nb, Ta, Ni) and Cu-Zr-Ag-Al-(Nb) bulk glassy alloy systems in various solutions are reported in this work. Moreover, X-ray photoelectron spectroscopy (XPS) analysis is performed to clarify the surface-related chemical characteristics of the alloy before and after immersion in the solutions; this has lead to a better understanding of the correlation between the surface composition and the corrosion resistance.

RI Inoue, Akihisa/E-5271-2015

OI Zhao, Weimin/0000-0002-2148-3246; Qin, Chunling/0000-0003-3525-7802

SN 1422-0067

PD APR

PY 2011

VL 12

IS 4

BP 2275

EP 2293

DI 10.3390/ijms12042275

UT WOS:000289238100014

PM 21731441

ER

PT J

AU Senkov, ON

Scott, JM

AF Senkov, ON

Scott, JM

TI Formation and thermal stability of Ca-Mg-Zn and Ca-Mg-Zn-Cu bulk

metallic glasses

SO MATERIALS LETTERS

AB Several Ca-Mg-Zn and Ca-Mg-Zn-Cu bulk metallic glasses were produced by copper mold casting method. The alloy compositions were selected using specific criteria recently identified by the authors. The glass transition temperature, crystallization temperature, temperature interval of the supercooled region, melting temperature as well as heats of crystallization, and melting are reported for these alloys. (C) 2003 Elsevier B.V. All rights reserved.

RI Senkov, Oleg/C-7197-2012

OI Senkov, Oleg/0000-0001-5587-415X; Senkov, Oleg/0000-0002-9336-3702

SN 0167-577X

PD MAR

PY 2004

VL 58

IS 7-8

BP 1375

EP 1378

DI 10.1016/j.matlet.2003.09.030

UT WOS:000188763600050

ER

PT J

AU Tian, L

Cheng, YQ

Shan, ZW

Li, J

Wang, CC

Han, XD

Sun, J

Ma, E

AF Tian, Lin

Cheng, Yong-Qiang

Shan, Zhi-Wei

Li, Ju

Wang, Cheng-Cai

Han, Xiao-Dong

Sun, Jun

Ma, Evan

TI Approaching the ideal elastic limit of metallic glasses

SO NATURE COMMUNICATIONS

AB The ideal elastic limit is the upper bound to the stress and elastic strain a material can withstand. This intrinsic property has been widely studied for crystalline metals, both theoretically and experimentally. For metallic glasses, however, the ideal elastic limit remains poorly characterized and understood. Here we show that the elastic strain limit and the corresponding strength of submicron-sized metallic glass specimens are about twice as high as the already impressive elastic limit observed in bulk metallic glass samples, in line with model predictions of the ideal elastic limit of metallic glasses. We achieve this by employing an in situ transmission electron microscope tensile deformation technique. Furthermore, we propose an alternative mechanism for the apparent 'work hardening' behaviour observed in the tensile stress-strain curves.

RI Shan, Zhiwei/B-8799-2014; Tian, Lin/N-8704-2013; Cheng,

Yongqiang/F-6567-2010; Li, Ju/A-2993-2008; Ma, En/A-3232-2010; xjtu,

campnano/Q-1904-2015

OI Li, Ju/0000-0002-7841-8058;

SN 2041-1723

PD JAN

PY 2012

VL 3

AR 609

DI 10.1038/ncomms1619

UT WOS:000299921000007

PM 22215084

ER

PT J

AU Schnabel, V

Kohler, M

Music, D

Bednarcik, J

Clegg, WJ

Raabe, D

Schneider, JM

AF Schnabel, Volker

Koehler, Mathias

Music, Denis

Bednarcik, Jozef

Clegg, William J.

Raabe, Dierk

Schneider, Jochen M.

TI Ultra-stiff metallic glasses through bond energy density design

SO JOURNAL OF PHYSICS-CONDENSED MATTER

AB The elastic properties of crystalline metals scale with their valence electron density. Similar observations have been made for metallic glasses. However, for metallic glasses where covalent bonding predominates, such as metalloid metallic glasses, this relationship appears to break down. At present, the reasons for this are not understood. Using high energy x-ray diffraction analysis of melt spun and thin film metallic glasses combined with density functional theory based molecular dynamics simulations, we show that the physical origin of the ultrahigh stiffness in both metalloid and non-metalloid metallic glasses is best understood in terms of the bond energy density. Using the bond energy density as novel materials design criterion for ultra-stiff metallic glasses, we are able to predict a Co33.0Ta3.5B63.5 short range ordered material by density functional theory based molecular dynamics simulations with a high bond energy density of 0.94 eV angstrom(-3) and a bulk modulus of 263 GPa, which is 17% greater than the stiffest Co-B based metallic glasses reported in literature.

RI Schneider, Jochen/A-4701-2012; Music, Denis/C-2978-2012

OI Music, Denis/0000-0003-2303-3676

SN 0953-8984

EI 1361-648X

PD JUN 28

PY 2017

VL 29

IS 26

AR 265502

DI 10.1088/1361-648X/aa72cb

UT WOS:000402436400002

PM 28498109

ER

PT J

AU Sun, LL

Wang, J

Kou, HC

Tang, B

Li, JS

Zhang, PX

AF Sun, L. L.

Wang, J.

Kou, H. C.

Tang, B.

Li, J. S.

Zhang, P. X.

TI Interface characteristics of a Zr-based BMG/copper laminated composite

SO SURFACE AND INTERFACE ANALYSIS

AB Diffusion bonding of metallic glasses and crystalline metals utilizing excellent superplasticity of monolithic bulk metallic glasses (BMGs) within supercooled liquid region has been found to be an efficient method to improve the room temperature plasticity and fracture toughness of metallic glass. A Zr-based BMG/copper laminated composite was fabricated by copressing method, and the interface bonding status was characterized by scanning electron microscopy (SEM) and high-resolution transmission electron microscopy. No void or crack is detected, and the interface is a metallurgical bonding of atomistic level. Although the BMG retains amorphous state after copressing at 390 degrees C, the region of the amorphous-crystalline mixture structure with the width of 30-40nm occurred within the diffusion zone. Copyright (c) 2013 John Wiley & Sons, Ltd.

RI Tang, Bin/M-1302-2018; WANG, Jun/B-1229-2010; WANG, Jun/A-1526-2015

OI Tang, Bin/0000-0002-3355-5999; WANG, Jun/0000-0001-8101-2967; WANG,

Jun/0000-0001-8101-2967; Li, Jinshan/0000-0002-6894-9760; KOU,

Hongchao/0000-0003-4960-9477

SN 0142-2421

EI 1096-9918

PD FEB

PY 2014

VL 46

IS 2

BP 61

EP 64

DI 10.1002/sia.5346

UT WOS:000329624000001

ER

PT J

AU Chen, W

Wang, Y

Qiang, J

Dong, C

AF Chen, W

Wang, Y

Qiang, J

Dong, C

TI Bulk metallic glasses in the Zr-Al-Ni-Cu system

SO ACTA MATERIALIA

AB Electron concentration and atomic size rules are important criteria for bulk metallic glass formation. According to these rules, a series of new Zr-Al-Ni-Cu amorphous alloys with a constant e/a ratio of 1.4 and an average atomic size of 0.1496 nm was designed. All the alloys have high glass forming abilities, large supercooled liquid regions DeltaT(x), and large reduced glass transition temperatures T-rg. The best glass forming composition is located near Zr63.8Al11.4Ni17.2Cu7.6. Its glass forming ability is higher than that of the Inoue alloy Zr65Al7.5Ni10Cu17.5. (C) 2003 Acta Materialia Inc. Published by Elsevier Science Ltd. All rights reserved.

SN 1359-6454

PD APR 18

PY 2003

VL 51

IS 7

BP 1899

EP 1907

DI 10.1016/S1359-6454(02)00596-7

UT WOS:000182138200006

ER

PT J

AU Soubeyroux, JL

Puech, S

Blandin, JJ

AF Soubeyroux, Jean-Louis

Puech, Sylvain

Blandin, Jean-Jacques

TI Synthesis of new Mg-based bulk metallic glasses with high glass forming

ability

SO MATERIALS SCIENCE AND ENGINEERING A-STRUCTURAL MATERIALS PROPERTIES

MICROSTRUCTURE AND PROCESSING

CT 12th International Conference on Rapidly Quenched and Metastable

Materials

CY AUG 21-26, 2005

CL Jeju Isl, SOUTH KOREA

AB New magnesium-based bulk metallic glasses have been synthesized in the system Mg.,Cu, Gdz with cone-shaped copper mould casting. Compositions have been prepared with critical diameters up to 9 mm. X-ray diffraction patterns and DSC traces have shown that a composition Mg58.5Cu30.5Gd11 shows higher 7, and a large Delta T. The substitution of gadolinium by yttrium in this formula has been studied in the same conditions, and samples with critical diameters up to 9 mm have been prepared. The glass forming ability of these compounds has been discussed. (c) 2006 Elsevier B.V All rights reserved.

SN 0921-5093

PD MAR 25

PY 2007

VL 449

BP 253

EP 256

DI 10.1016/j.msea.2006.02.429

UT WOS:000245477800054

ER

PT J

AU Basu, J

Ranganathan, S

AF Basu, J

Ranganathan, S

TI Bulk metallic glasses: A new class of engineering materials

SO SADHANA-ACADEMY PROCEEDINGS IN ENGINEERING SCIENCES

AB Bulk glass-forming alloys have emerged over the past fifteen years with attractive properties and technological promise. A number of alloy systems based on lanthanum, magnesium, zirconium, palladium, iron, cobalt and nickel have been discovered. Glass-forming ability depends on various factors like enthalpy of mixing, atomic size and multicomponent alloying. A number of processes is available to synthesise bulk metallic glasses. The crystallisation behaviour and mechanical properties of these alloys pose interesting scientific questions. Upon crystallisation many of these glasses transform to bulk nanocrystals and nanoquasicrystals. A detailed study of the structure and the crystallisation behaviour of glasses has enabled the elucidation of the possible atomic configuration in liquid alloys. Their crystallisation behaviour can be exploited to synthesise novel nanocomposite microstructures and their mechanical properties can be enhanced. A broad overview of the present status of the science and technology of bulk metallic glasses and their potential technological uses is presented.

RI Basu, Joysurya/A-8398-2009; Ranganathan, Srinivasa/F-9187-2013

OI Basu, Joysurya/0000-0001-5155-2620

SN 0256-2499

PD JUN-AUG

PY 2003

VL 28

BP 783

EP 798

DI 10.1007/BF02706459

PN 3-4

UT WOS:000186262200022

ER

PT J

AU Yamasaki, M

Kagao, S

Kawamura, Y

AF Yamasaki, M

Kagao, S

Kawamura, Y

TI Thermal diffusivity and conductivity of Zr55Al10Ni5Cu30 bulk metallic

glass

SO SCRIPTA MATERIALIA

AB We measured the thermal diffusivity of amorphous solid and supercooled liquid in a Zr(55)A(10)Ni(5)Cu(30) (at.%) bulk metallic glass using a laser flash method. The thermal diffusivity and conductivity of the amorphous solid were weakly temperature-dependent, with small positive temperature coefficients. The thermal diffusivity of supercooled liquid remains constant with temperature. &COPY; 2005 Acta Materialia Inc. Published by Elsevier Ltd. All rights reserved.

RI Yamasaki, Michiaki/B-1123-2018

SN 1359-6462

PD JUL

PY 2005

VL 53

IS 1

BP 63

EP 67

DI 10.1016/j.scriptamat.2005.03.021

UT WOS:000229513900012

ER

PT J

AU Wu, J

Wang, Q

Chen, F

Wang, YM

Qiang, TB

Dong, C

AF Wu, J.

Wang, Q.

Chen, F.

Wang, Y. M.

Qiang, T. B.

Dong, C.

TI Ternary Sm-Al-Ni bulk metallic glasses

SO INTERMETALLICS

CT 6th International Workshop on Advanced Intermetallic and Metallic

Materials

CY OCT 09-15, 2005

CL Yangzhou, PEOPLES R CHINA

SP Natl Nat Sci Fdn China, Minist Sci & Technol China, Nanjing Univ Sci & Technol, Univ Sci & Technol Beijing, Oak Ridge Natl Lab, Shenyang Natl Lab Mat Sci, Cent S Univ, Harbin Inst Technol, Yangzhou City Govt

AB The present paper is concerned with the formation of the ternary Sm-based Sm-Al-Ni bulk metallic glasses. Composition design is carried out using our e/a- and cluster-related criteria. Three bulk metallic glasses, Sm54Al23Ni23, Sm56Al22Ni22 and Sm58Al21Ni21, are obtained by suction casting into rods with diameter of 3 mm. All of them share a constant e/a = 1.5 and fall along the e/a-constant composition line in the ternary composition diagram. The Sm54Al23Ni23 BMG exhibits the best thermal stability and glass-forming ability, which is located at the intersecting point of the e/a-constant line and the Sm7Ni3-Al cluster line. (C) 2006 Elsevier Ltd. All rights reserved.

RI Chen, Feng/A-9314-2013

SN 0966-9795

EI 1879-0216

PD MAY-JUN

PY 2007

VL 15

IS 5-6

BP 652

EP 654

DI 10.1016/j.intermet.2006.10.007

UT WOS:000246739400008

ER

PT J

AU Novikov, VN

Sokolov, AP

AF Novikov, V. N.

Sokolov, A. P.

TI Correlation of fragility and Poisson's ratio: Difference between

metallic and nonmetallic glass formers

SO PHYSICAL REVIEW B

AB It is shown that fragility of supercooled metallic liquids correlates with the Poisson's ratio of the respective metallic glasses. However, the correlation differs from that found previously for simple nonmetallic glass formers [V. N. Novikov and A. P. Sokolov, Nature 431, 961 (2004)]. The observed difference is assigned to the contribution of the free electron gas to the bulk modulus in metallic glasses.

SN 1098-0121

PD AUG

PY 2006

VL 74

IS 6

AR 064203

DI 10.1103/PhysRevB.74.064203

UT WOS:000240238500029

ER

PT J

AU Ott, RT

Fan, C

Li, J

Hufnagel, TC

AF Ott, RT

Fan, C

Li, J

Hufnagel, TC

TI Structure and properties of Zr-Ta-Cu-Ni-Al bulk metallic glasses and

metallic glass matrix composites

SO JOURNAL OF NON-CRYSTALLINE SOLIDS

CT Annual Meeting of The-Minerals-Metals-and-Materials-Society

CY FEB 17-21, 2002

CL SEATTLE, WASHINGTON

SP Minerals Met & Mat Soc, Extract & Proc Div

AB We have developed a series of alloys which, upon cooling from the melt, form two-phase microstructures consisting of crystalline Ta-rich solid solution particles embedded in a bulk metallic glass matrix. These alloys have the general composition (Zr70Ni10Cu20)(90-x)TaxAl10 where 0 less than or equal to x less than or equal to 12 (all compositions are in atomic percent). These alloys have mechanical properties consistent with metallic glasses, including high yield strength (similar to2 GPa) and large elastic elongation (similar to2%). However, they also show much larger plastic strain to failure (up to 16%) in uniaxial compression than monolithic metallic glasses. (C) 2003 Elsevier Science B.V. All rights reserved.

RI Hufnagel, Todd/A-3309-2010

OI Hufnagel, Todd/0000-0002-6373-9377; Ott, Ryan/0000-0002-7159-2328

SN 0022-3093

PD MAR

PY 2003

VL 317

IS 1-2

BP 158

EP 163

DI 10.1016/S0022-3093(02)01996-8

UT WOS:000181251300025

ER

PT J

AU Wang, D

Zhao, SJ

Liu, LM

AF Wang, Da

Zhao, Shi-Jin

Liu, Li-Min

TI Theoretical Study on the Composition Location of the Best Glass Formers

in Cu-Zr Amorphous Alloys

SO JOURNAL OF PHYSICAL CHEMISTRY A

AB This study combines the molecular dynamics (MD) simulations and first-principles approach to explain the experimental observation that the best glass formers of Cu-Zr bulk metallic glasses (BMGs) have the compositions Cu50Zr50 and Cu64Zr36. These two best glass formers are first calculated to be most abundantly composed of Cu6Zr7 and Cu8Zr5 icosahedral clusters when compared in the compositional range of CuxZr(100-x) (45 <= x <= 70), and then these two icosahedral clusters are calculated to have the lowest formation energy among the icosahedral clusters CuxZr13-x (3 <= x <= 10), as well as possessing some characteristics in electronic structure and chemical hardness. Through understanding the properties of specific icosahedral clusters in metallic glasses, the structural and energetic contribution to the glass-forming ability are systematically discussed.

RI Wang, Da/K-2766-2016; Liu, Li-Min/A-7051-2010

OI Wang, Da/0000-0002-4512-7860; Liu, Li-Min/0000-0002-7137-9017; Liu,

Limin/0000-0003-3925-5310

SN 1089-5639

PD JAN 29

PY 2015

VL 119

IS 4

BP 806

EP 814

DI 10.1021/jp5120064

UT WOS:000348752400028

PM 25547898

ER

PT J

AU Mridha, S

Arora, HS

Lefebvre, J

Bhowmick, S

Mukherjee, S

AF Mridha, Sanghita

Arora, Harpreet Singh

Lefebvre, Joseph

Bhowmick, Sanjit

Mukherjee, Sundeep

TI High Temperature In Situ Compression of Thermoplastically Formed

Nano-scale Metallic Glass

SO JOM

AB The mechanical behavior of nano-scale metallic glasses was investigated by in situ compression tests in a scanning electron microscope. Platinum-based metallic glass nano-pillars were fabricated by thermoplastic forming. The nano-pillars and corresponding bulk substrate were tested in compression over the range of room temperature to glass transition. Stress-strain curves of the nano-pillars were obtained along with in situ observation of their deformation behavior. The bulk substrate as well as nano-pillars showed an increase in elastic modulus with temperature which is explained by diffusive rearrangement of atomic-scale viscoelastic units.

SN 1047-4838

EI 1543-1851

PD JAN

PY 2017

VL 69

IS 1

BP 39

EP 44

DI 10.1007/s11837-016-1961-7

UT WOS:000391458200005

ER

PT S

AU Bhatt, J

Murty, BS

AF Bhatt, J.

Murty, B. S.

BE Roosz, A

Mertinger, V

Barkoczy, P

Hoo, C

TI Identification of Bulk Metallic Forming Compositions through

Thermodynamic and Topological Models

SO SOLIDIFICATION AND GRAVITY V

SE Materials Science Forum

CT 5th International Conference on Solidification and Gravity

CY SEP 06-09, 2008

CL Univ Miskolc, Lillafured, HUNGARY

SP Univ Miskolc, Fac Mat Sci & Eng, Inst Mat Sci, Hungarian Acad Sci, Mat Sci Working Grp, Miskolc Committee Hungarian Acad Sci, Hungarian Astronaut Assoc, Hungarian Space Off

HO Univ Miskolc

AB This paper attempts to optimize the bulk metallic glass forming compositions using enthalpy of chemical mixing (Delta H-chem) as thermodynamic, mismatch entropy (Delta S-sigma/k(B)) as topological and configurational entropy (Delta S-config/R) as statistical parameters. The product of Delta H-chem and Delta S-sigma/k(B) which is termed as P-HS in the Delta S-config/R range of 0.9 to 1.0 can be correlated strongly to glass forming ability. P-HS being an important parameter has been used to design the quaternary and quinary Bulk Metallic Glass compositions from ternary compositions. This has been demonstrated for two Zr rich quaternary systems in Zr-Ti-Cu-Ni and Zr-Cu-Ni-Al based bulk metallic glasses. By weighing approach of P-HS of four Zr rich quaternary systems and one non-Zr rich systems a new Zr rich quinary bulk metallic glass in Zr-Ti-Cu-Ni-Al system is designed. Mechanical alloying is used to prepare the bulk amorphous powder at the compositions predicted by the model in order to validate the model.

RI Murty, BS/P-3354-2015

OI Murty, BS/0000-0002-4399-8531

SN 0255-5476

PY 2010

VL 649

BP 67

EP +

DI 10.4028/www.scientific.net/MSF.649.67

UT WOS:000287945200011

ER

PT J

AU Meng, D

Yi, J

Zhao, DQ

Ding, DW

Bai, HY

Pan, MX

Wang, WH

AF Meng, D.

Yi, J.

Zhao, D. Q.

Ding, D. W.

Bai, H. Y.

Pan, M. X.

Wang, W. H.

TI Tantalum based bulk metallic glasses

SO JOURNAL OF NON-CRYSTALLINE SOLIDS

AB Ta-based bulk metallic glasses with high strength (2.7 GPa) and hardness (9.7 GPa), high elastic modulus (170 GPa) and high density (12.98 g/mm(3)) were developed. The best glass forming ability so far for a Ta-Ni-Co system reaches a critical diameter of 2 mm by the copper mold casting method. It shows an exceptionally high glass transition temperature of 983 K and a high crystallization temperature up to 1023 K. The unique mechanical and physical properties make them a promising high strength material. (C) 2011 Elsevier B.V. All rights reserved.

SN 0022-3093

EI 1873-4812

PD APR 1

PY 2011

VL 357

IS 7

BP 1787

EP 1790

DI 10.1016/j.jnoncrysol.2011.01.020

UT WOS:000290006900030

ER

PT J

AU Bakkal, M

Liu, CT

Watkins, TR

Scattergood, RO

Shih, AJ

AF Bakkal, M

Liu, CT

Watkins, TR

Scattergood, RO

Shih, AJ

TI Oxidation and crystallization of Zr-based bulk metallic glass due to

machining

SO INTERMETALLICS

AB Bulk metallic glass was subjected to extreme deformation conditions during machining operation. The flash temperature, oxidation and crystallization within chips from a Zr-based bulk metallic glass were investigated. A near-infrared spectrometer was used to capture the light emission from the tool-chip interface allowing the calculation of the temperature. Very high temperatures in the 2400-2700 K range were observed and associated with the light emission and oxidation in the chips. X-ray diffraction analysis revealed oxidation of chips machined at high cutting speeds. Optical micrographs of polished and etched chip cross-sections showed a dendritic pattern due to crystallization. Using a field emission gun SEM, amorphous and crystalline regions were analyzed. This study demonstrates the oxidation and crystallization of bulk metallic glass during machining. (C) 2003 Elsevier Ltd. All rights reserved.

RI Watkins, Thomas/D-8750-2016; Scattergood, Ronald/D-5204-2009

OI Watkins, Thomas/0000-0002-2646-1329; Liu, Chain

Tsuan/0000-0001-7888-9725

SN 0966-9795

EI 1879-0216

PD FEB

PY 2004

VL 12

IS 2

BP 195

EP 204

DI 10.1016/j.intermet.2003.09.017

UT WOS:000188860100007

ER

PT J

AU Sergueeva, AV

Mara, NA

Kuntz, JD

Branagan, DJ

Mukherjee, AK

AF Sergueeva, AV

Mara, NA

Kuntz, JD

Branagan, DJ

Mukherjee, AK

TI Shear band formation and ductility of metallic glasses

SO MATERIALS SCIENCE AND ENGINEERING A-STRUCTURAL MATERIALS PROPERTIES

MICROSTRUCTURE AND PROCESSING

AB Variations in microstructure and chemical compositions of the metallic glasses found in the literature, as well as an overall lack of experimental data on inhomogeneous behavior of metallic glass make the evaluation of the effects of shear band/fracture behavior on mechanical proper-ties of metallic glasses difficult. Investigating the effect of strain localization alone on inhomogeneous flow seems to be a first step in approaching this problem. Mechanical behavior of metallic glasses at room temperature and various strain rates in tension and compression was investigated. Formation of multiple shear bands was observed at high strain rates. An increase in strain rate leads to enhanced ductility in tension, whereas, the ductility of the material in compression decreases with increasing strain rate. Differences in deformation processes in tension and compression were compared. (C) 2004 Elsevier B.V. All rights reserved.

RI Mara, Nathan/J-4509-2014

OI Mara, Nathan/0000-0002-9135-4693

SN 0921-5093

PD OCT 15

PY 2004

VL 383

IS 2

BP 219

EP 223

DI 10.1016/j.msea.2004.05.064

UT WOS:000224336600004

ER

PT J

AU Sha, ZD

Xu, B

Shen, L

Zhang, AH

Feng, YP

Li, Y

AF Sha, Z. D.

Xu, B.

Shen, L.

Zhang, A. H.

Feng, Y. P.

Li, Y.

TI The basic polyhedral clusters, the optimum glass formers, and the

composition-structure-property (glass-forming ability) correlation in

Cu-Zr metallic glasses

SO JOURNAL OF APPLIED PHYSICS

AB Using the large-scale atomic/molecular massively parallel simulator, chemical short-range order of the Cu-Zr binary system over the three good glass-forming compositional ranges has been investigated. Based on a statistical analysis, a simple route has been developed for broad investigations of the basic polyhedral clusters, optimum glass formers, as well as the composition-structure-property (glass-forming ability) correlation. In addition, topological short-range orders of the basic polyhedral clusters in the three compositional ranges were characterized. Our findings have implications for understanding the nature, forming ability, and properties of metallic glasses.

RI SHEN, LEI/G-1077-2012; SHA, Zhendong/E-9463-2012; Feng, Yuan

Ping/A-4507-2012

OI SHEN, LEI/0000-0001-6198-5753; Feng, Yuan Ping/0000-0003-2190-2284

SN 0021-8979

PD MAR 15

PY 2010

VL 107

IS 6

AR 063508

DI 10.1063/1.3359683

UT WOS:000276210800034

ER

PT J

AU Xia, L

Fang, SS

Wang, Q

Dong, YD

Liu, CT

AF Xia, L

Fang, SS

Wang, Q

Dong, YD

Liu, CT

TI Thermodynamic modeling of glass formation in metallic glasses

SO APPLIED PHYSICS LETTERS

AB In the study of metallic glasses, it is vitally important for us to understand glass formation and glass forming ability (GFA). This letter presents a thermodynamic model for evaluating glass formation in metallic glasses. An expression has been derived from considering both the stability of amorphous phases and the resistance to the formation of crystalline intermetallic phases. This equation is very useful for identifying alloy compositions with good GFA in binary and other alloy systems. The prediction of this equation has been verified by the experimental study of glass formation and glass forming ability in the Zr-Cu system. (c) 2006 American Institute of Physics.

OI Liu, Chain Tsuan/0000-0001-7888-9725; Xia, Lei/0000-0001-9198-1497

SN 0003-6951

PD APR 24

PY 2006

VL 88

IS 17

AR 171905

DI 10.1063/1.2198830

UT WOS:000237136600020

ER

PT J

AU Flege, S

Hahn, H

Averback, RS

AF Flege, S

Hahn, H

Averback, RS

TI Thermal and radiation-enhanced diffusion in the bulk metallic glass

Ni23Zr62Al15

SO PHYSICAL REVIEW B

AB The temperature dependence of tracer diffusion in the three-component system NiZrAl was measured. For the composition Ni23Zr62Al15, it was possible to measure diffusion coefficients below and above the glass transition temperature. Similar to the binary metallic glass NiZr, the diffusion coefficient was strongly dependent on the atomic size of the tracer, varying by two orders of magnitude. The results are suggestive of a collective diffusion mechanism in bulk metallic glasses. Radiation-enhanced diffusion was also measured in this alloy and compared with measurements of radiation-induced viscous flow on similar alloys.

RI Hahn, Horst/G-9018-2011

OI Hahn, Horst/0000-0001-9901-3861

SN 1098-0121

PD JAN

PY 2004

VL 69

IS 1

AR 014303

DI 10.1103/PhysRevB.69.014303

UT WOS:000188784800036

ER

PT J

AU Ramamurty, U

Lee, ML

Basu, J

Li, Y

AF Ramamurty, U

Lee, ML

Basu, J

Li, Y

TI Embrittlement of a bulk metallic glass due to low-temperature annealing

SO SCRIPTA MATERIALIA

AB Embrittlement of a bulk La-based metallic glass due to isothermal and isochronal annealing below the T-g was investigated. Results show that the impact toughness decreases with increasing annealing time or temperature, accompanied by a change in fracture morphology. Reasons for this are discussed in terms of structural relaxation. (C) 2002 Acta Materialia Inc. Published by Elsevier Science Ltd. All rights reserved.

RI Basu, Joysurya/A-8398-2009; Ramamurty, Upadrasta/E-5623-2011

OI Basu, Joysurya/0000-0001-5155-2620; Lee, Mong Li/0000-0002-9636-388X

SN 1359-6462

PD JUL 22

PY 2002

VL 47

IS 2

BP 107

EP 111

AR PII S1359-6462(02)00102-1

DI 10.1016/S1359-6462(02)00102-1

UT WOS:000177459100007

ER

PT J

AU Zheng, Q

Ma, H

Ma, E

Xu, J

AF Zheng, Qiang

Ma, Han

Ma, En

Xu, Jian

TI Mg-Cu-(Y,Nd) pseudo-ternary bulk metallic glasses: The effects of Nd on

glass-forming ability and plasticity

SO SCRIPTA MATERIALIA

AB We report the improvement of glass-forming ability by using Nd to substitute Y in the Mg-Cu-Y bulk metallic glass (BMG). A search in the Mg-Cu-(Y,Nd) pseudo-ternary system located the best glass former at the Mg57Cu31Y6.6Nd5.4 composition, where the critical size for BMG formation (diameter D-c for copper mold casting) reached at least 14 mm. In comparison with the ternary Mg-Cu-Y glass, the Nd-containing BMG, without a significant change in Poisson's ratio, exhibits higher strength and improved compressive plasticity. (c) 2006 Acta Materialia Inc. Published by Elsevier Ltd. All rights reserved.

RI Zheng, Qiang/A-4566-2010; Ma, En/A-3232-2010

OI Zheng, Qiang/0000-0002-6057-8141;

SN 1359-6462

PD SEP

PY 2006

VL 55

IS 6

BP 541

EP 544

DI 10.1016/j.scriptamat.2006.05.029

UT WOS:000239413400011

ER

PT S

AU Park, JM

Park, JS

Kim, JH

Lee, MH

Kim, WT

Kim, DH

AF Park, JM

Park, JS

Kim, JH

Lee, MH

Kim, WT

Kim, DH

BE Zhong, ZY

Saka, H

Kim, TH

Holm, EA

Han, YF

Xie, XS

TI Ti-based bulk metallic glass with high cold workability at room

temperature

SO PRICM 5: THE FIFTH PACIFIC RIM INTERNATIONAL CONFERENCE ON ADVANCED

MATERIALS AND PROCESSING, PTS 1-5

SE Materials Science Forum

CT 5th Pacific Rim International Conference on Advanced Materials and

Processing

CY NOV 02-05, 2004

CL Beijing, PEOPLES R CHINA

SP Chinese Soc Met, Japan Inst Met, Korea Inst Met & Mat, Minerals, Met & Mat Soc

AB The cold workability of Ti-based bulk metallic glasses (BMGs) have been investigated. Ti45Zr16Be20Cu10Ni9 BMG with a large compressive plastic strain of 4.7 % shows a high cold workability, i.e. total reduction ratio of 50 % by cold rolling at room temperature. The multiple shear bands formed during rolling are effective in enhancing the plasticity. The cold rolled Ti45Zr16Be20Cu10Ni9 BMG (reduction ratio: 30 %) exhibits a large plastic strain of similar to 14 %.

RI bang, changwook/J-7922-2012

OI LEE, MIN HA/0000-0001-6006-0628

SN 0255-5476

BN 0-87849-960-1

PY 2005

VL 475-479

BP 3431

EP 3434

DI 10.4028/www.scientific.net/MSF.475-479.3431

PN 1-5

UT WOS:000227494704021

ER

PT S

AU Zhang, XF

Li, HX

Jiang, ZQ

Yi, SH

AF Zhang, Xin Fang

Li, Hongxiang

Jiang, Zhiqiang

Yi, Seonghoon

BE Zhou, M

TI Bulk metallic glass Ti42Co37.1Co4Zr8Sn2Ag2Be4.9 with high glass forming

ability and ductility

SO HIGH PERFORMANCE STRUCTURES AND MATERIALS ENGINEERING, PTS 1 AND 2

SE Advanced Materials Research

CT 1st International Conference on High Performance Structures and

Materials Engineering

CY MAY 05-06, 2011

CL Beijing, PEOPLES R CHINA

SP Int Ind Elect Ctr, Shenzhen Univ, ACM Hong Kong Chapter

AB Bulk metallic glass Ti42Cu37.1Co4Zr8Sn2Ag2Be4.9 that can be cast into a fully amorphous rod of more than 6 mm in diameter by copper molder casting has been developed through systematic alloy design. The bulk metallic glass exhibits high fracture strength (sigma(f)=2071 MPa) and good ductility (epsilon(f)=5.83%) under compression.

SN 1022-6680

BN 978-3-03785-075-6

PY 2011

VL 217-218

BP 636

EP +

DI 10.4028/www.scientific.net/AMR.217-218.636

PN 1-2

UT WOS:000292278900117

ER

PT J

AU Wenwer, F

Knorr, K

Macht, MP

Mehrer, H

AF Wenwer, F

Knorr, K

Macht, MP

Mehrer, H

TI Ni tracer diffusion in the bulk metallic glasses

Zr41Ti14Cu12.5Ni10Be22.5 and Zr65Cu17.5Ni10Al7.5

SO DEFECT AND DIFFUSION FORUM

CT International Conference on Diffusion in Materials (DIMAT 96)

CY AUG 05-09, 1996

CL NORDKIRCHEN, GERMANY

SP Nordrhein Westfalen Coll Finances, Germany

AB The bulk metallic glasses Zr41Ti14Cu12.5Ni10Be22.5 and Zr65Cu17.5Ni10Al7.5 were produced from the melt which is possible with moderate cooling rates. These materials can be heated about 100 K above their glass transition temperature for rather long times without crystallization. Self-diffusion measurements of Ni-63 in Zr41Ti14Cu12.5Ni10Be22.5 and Zr65Cu17.5Ni10Al7.5 employing the radiotracer method and sputter sectioning are reported. The measurements were performed below and above the glass transition temperature, i.e., in the glassy state and in the supercooled liquid state. The Ni results are compared to diffusion measurements of Be, Co and Al in Zr41Ti14Cu12.5Ni10Be22.5. Effects of structural changes are discussed.

SN 1012-0386

PY 1997

VL 143

BP 831

EP 835

DI 10.4028/www.scientific.net/DDF.143-147.831

PN 1

UT WOS:A1997XM07600127

ER

PT J

AU Bruck, HA

Rosakis, AJ

Johnson, WL

AF Bruck, HA

Rosakis, AJ

Johnson, WL

TI The dynamic compressive behavior of beryllium bearing bulk metallic

glasses

SO JOURNAL OF MATERIALS RESEARCH

AB In 1993, a new beryllium bearing bulk metallic glass with the nominal composition of Zr41.25Ti13.75CU12.5Ni10Be22.5 was discovered at Caltech. This metallic glass can be cast as cylindrical rods as large as 16 mm in diameter, which permitted specimens to be fabricated with geometries suitable for dynamic testing, For the first time, the dynamic compressive yield behavior of a metallic glass was characterized at strain rates of 10(2) to 10(4)/s by using the split Hopkinson pressure bar. A high-speed infrared thermal detector was also used to determine if adiabatic heating occurred during dynamic deformation of the metallic glass. From these tests it appears that the yield stress of the metallic glass is insensitive to strain rate and no adiabatic heating occurs before yielding.

RI bruck, hugh/I-1154-2018

OI bruck, hugh/0000-0002-0845-765X

SN 0884-2914

EI 2044-5326

PD FEB

PY 1996

VL 11

IS 2

BP 503

EP 511

DI 10.1557/JMR.1996.0060

UT WOS:A1996TU51600030

ER

PT J

AU Li, Y

AF Li, Y

TI A relationship between glass-forming ability and reduced glass

transition temperature near eutectic composition

SO MATERIALS TRANSACTIONS

CT Bulk Metallic Glasses Conference

CY SEP 24-28, 2000

CL SINGAPORE, SINGAPORE

AB Onset temperature (solidus), T-m and offset temperature (liquidus) T-l of melting of a series of bulk glass forming alloys based on Zr, La, Mg, Pd and rare-earth elements have been measured by studying systematically the melting behaviour of these alloys using differential thermal analysis (DTA) or differential scanning calorimetry (DSC). Bulk metallic glass formation has been found to be most effective at or near their eutectic points and less effective for off-eutectic allays. Reduced glass transition temperature T-rg given by T-g/T-l is found to show a stronger correlation with critical cooling rate or critical section thickness for glass formation than T-rg given by T-g/T-m.

SN 1345-9678

EI 1347-5320

PD APR

PY 2001

VL 42

IS 4

BP 556

EP 561

DI 10.2320/matertrans.42.556

UT WOS:000168864100004

ER

PT J

AU Granata, D

Fischer, E

Loffler, JF

AF Granata, D.

Fischer, E.

Loeffler, J. F.

TI Hydrogen microalloying as a viable strategy for enhancing the

glass-forming ability of Zr-based bulk metallic glasses

SO SCRIPTA MATERIALIA

AB The development of bulk metallic glasses (BMGs) with both high critical casting thicknest and ductility is a great challenge. In this study we show that the glass-forming ability (GFA) of ductile Zr-rich Zr-Cu-Al BMGs can be increased by processing the alloys under hydrogen-containing atmosphere. After considering various reasons for this GFA enhancement, we attribute it to hydrogen microalloying, which generates a higher packing density. We also discuss the role of applied hydrogen pressure and present implications for future alloy design. (C) 2015 Acta Materialia Inc. Published by Elsevier Ltd. All rights reserved.

SN 1359-6462

PD JUL 1

PY 2015

VL 103

BP 53

EP 56

DI 10.1016/j.scriptamat.2015.03.005

UT WOS:000354343200014

ER

PT J

AU Zhang, ZY

Keppens, V

Egami, T

AF Zhang, Zhiying

Keppens, Veerle

Egami, Takeshi

TI A simple model to predict the temperature dependence of elastic moduli

of bulk metallic glasses

SO JOURNAL OF APPLIED PHYSICS

AB We report a simple model to predict the temperature dependence of elastic moduli of bulk metallic glasses from room temperature measurements, using the Varshni equation and the basic assumption that between absolute zero and the melting point, the shear and bulk moduli change, respectively, by 45% and 22%. This model has been tested using experimental data obtained on a large variety of bulk metallic glasses, and the predicted values are found to be in very good agreement with the experimental results. (c) 2007 American Institute of Physics.

SN 0021-8979

PD DEC 15

PY 2007

VL 102

IS 12

AR 123508

DI 10.1063/1.2818046

UT WOS:000251987600029

ER

PT J

AU Fan, GJ

Choo, H

Liaw, PK

AF Fan, G. J.

Choo, H.

Liaw, P. K.

TI A new criterion for the glass-forming ability of liquids

SO JOURNAL OF NON-CRYSTALLINE SOLIDS

AB Based on theoretical calculations using the fragility concept and the nucleation theory for a model glass-forming system, we propose a dimensionless criterion, phi, expressed by T-rg(Delta T-x/T-g)(a), with T-rg, the reduced glass-transition temperature, Delta T-x, the width of the supercooled liquid region when heating a glass, T-g, the glass transition temperature, and a, the exponent. The application of this simple criterion to various glasses, including network, metallic, and molecular glasses (except pure water), indicates an excellent correlation between the critical cooling rate R-c and phi in a Log R-c-phi single master plot with a = 0.143. (c) 2006 Elsevier B.V. All rights reserved.

RI Choo, Hahn/A-5494-2009

OI Choo, Hahn/0000-0002-8006-8907

SN 0022-3093

PD JAN 1

PY 2007

VL 353

IS 1

BP 102

EP 107

DI 10.1016/j.jnoncrysol.2006.08.049

UT WOS:000243613700013

ER

PT J

AU Shirasawa, N

Takigawa, Y

Uesugi, T

Higashi, K

AF Shirasawa, Naoya

Takigawa, Yorinobu

Uesugi, Tokuteru

Higashi, Kenji

TI Calculation of alloying effect on formation enthalpy of TiCu

intermetallics from first-principles calculations for designing

Ti-Cu-system metallic glasses

SO PHILOSOPHICAL MAGAZINE LETTERS

AB The effect of alloying on the formation enthalpy of TiCu intermetallics was investigated via first-principles calculations to propose a new design method for Ti-Cu-system metallic glasses. The calculation results showed good agreement with the reported experimental results that Ni, Pd, Sn and Zr improve this system's glass-forming ability. According to the calculation results, a Ti-Zr-Cu-Ga system was designed as a potential new bulk Ti-based metallic glass, and a bulk sample with a 2-mm diameter was fabricated.

RI Uesugi, Tokuteru/B-9776-2011; Takigawa, Yorinobu/A-6555-2010

OI Takigawa, Yorinobu/0000-0003-0321-6336

SN 0950-0839

EI 1362-3036

PD JAN 2

PY 2016

VL 96

IS 1

BP 27

EP 34

DI 10.1080/09500839.2015.1134833

UT WOS:000372088900004

ER

PT J

AU Zhou, W

Zhang, CW

Sheng, MQ

Hou, JX

AF Zhou, Wei

Zhang, Caiwei

Sheng, Minqi

Hou, Jixin

TI Glass Forming Ability and Corrosion Resistance of Zr-Cu- Ni-Al-Ag Bulk

Metallic Glass

SO METALS

AB Zr70-xCu12.5Ni10Al7.5Agx (x = 0-10) bulk metallic glasses (BMGs) have been prepared by copper mold casting. The glass-forming ability (GFA) and corrosion behavior of Zr-based BMGs have been investigated. It is found that the GFA of Zr-based BMGs first increases and then decreases with the increase of the Ag content, and the best glass former is Zr65Cu12.5Ni10Al7.5Ag5 with the maximum thickness of the glass phase region of 4.3 mm. The corrosion resistance is, however, found to be worsened with the increase of the Ag content. The mechanisms for the enhancement of GFA and the deterioration of corrosion resistance in the alloys are discussed.

SN 2075-4701

PD OCT

PY 2016

VL 6

IS 10

AR 230

DI 10.3390/met6100230

UT WOS:000388888100003

ER

PT J

AU Chen, MW

Inoue, A

Zhang, W

Sakurai, T

AF Chen, Mingwei

Inoue, Akihisa

Zhang, Wei

Sakurai, Toshio

TI Extraordinary plasticity of ductile bulk metallic glasses

SO PHYSICAL REVIEW LETTERS

AB Shear bands generally initiate strain softening and result in low ductility of metallic glasses. In this Letter, we report high-resolution electron microscope observations of shear bands in a ductile metallic glass. Strain softening caused by localized shearing was found to be effectively prevented by nanocrystallization that is in situ produced by plastic flow within the shear bands, leading to large plasticity and strain hardening. These atomic-scale observations not only well explain the extraordinary plasticity that was recently observed in some bulk metallic glasses, but also reveal a novel deformation mechanism that can effectively improve the ductility of monolithic metallic glasses.

RI CHEN, Mingwei/A-4855-2010; Inoue, Akihisa/E-5271-2015

OI CHEN, Mingwei/0000-0002-8274-3099; Chen, Mingwei/0000-0002-2850-8872

SN 0031-9007

PD JUN 23

PY 2006

VL 96

IS 24

AR 245502

DI 10.1103/PhysRevLett.96.245502

UT WOS:000238487900033

PM 16907252

ER

PT J

AU Wisitsorasak, A

Wolynes, PG

AF Wisitsorasak, Apiwat

Wolynes, Peter G.

TI Dynamical theory of shear bands in structural glasses

SO PROCEEDINGS OF THE NATIONAL ACADEMY OF SCIENCES OF THE UNITED STATES OF

AMERICA

AB The heterogeneous elastoplastic deformation of structural glasses is explored using the framework of the random first-order transition theory of the glass transition along with an extended mode-coupling theory that includes activated events. The theory involves coupling the continuum elastic theory of strain transport with mobility generation and transport as described in the theory of glass aging and rejuvenation. Fluctuations that arise from the generation and transport of mobility, fictive temperature, and stress are treated explicitly. We examine the nonlinear flow of a glass under deformation at finite strain rate. The interplay among the fluctuating fields leads to the spatially heterogeneous dislocation of the particles in the glass, i.e., the appearance of shear bands of the type observed in metallic glasses deforming under mechanical stress.

SN 0027-8424

PD FEB 7

PY 2017

VL 114

IS 6

BP 1287

EP 1292

DI 10.1073/pnas.1620399114

UT WOS:000393422200036

PM 28108571

ER

PT J

AU Wang, D

Shi, TL

Pan, J

Liao, GL

Tang, ZR

Liu, L

AF Wang, Dong

Shi, Teilin

Pan, Jie

Liao, Guanglan

Tang, Zirong

Liu, Lin

TI Finite element simulation and experimental investigation of forming

micro-gear with Zr-Cu-Ni-Al bulk metallic glass

SO JOURNAL OF MATERIALS PROCESSING TECHNOLOGY

AB Bulk metallic glasses exhibit Some Unique physical properties as compared to their corresponding crystalline alloys Due to the superplasticity by behaving like a Newtonian fluid in their supercooled liquid region. the bulk metallic glasses can be used to make high strength microparts by net-shape forming In this paper. the compressive tests of Zr-Cu-Ni-Al metallic glass are performed with different strain rates at a temperature of 683 K According to the experimental results. the forming evolution Of a metallic glass micro-gear IS Simulated using a finite element Simulation software DEFORM 3D. and the forming load is predicted at different processing parameters Meanwhile, the filling stages of bulk metallic glass in the micro-gear mold cavity are investigated by finite element simulation and experiment The predicted workpiece geometry shows good agreement with experimental result The forming experiments for micro-gear Of Zr-Cu-Ni-Al metallic glass are carried out by hot embossing process. and the amorphous micro-gears are obtained Successfully It is found that the finite element simulation results are in reasonable agreement with the experimental observation (C) 2009 Elsevier B.V. All rights reserved

RI Pan, Jie/L-3928-2017

OI Pan, Jie/0000-0001-5465-0736

SN 0924-0136

PD MAR 1

PY 2010

VL 210

IS 4

BP 684

EP 688

DI 10.1016/j.jmatprotec.2009.12.005

UT WOS:000274927300013

ER

PT S

AU Li, YH

Zhang, W

Dong, C

Makino, A

AF Li, Yanhui

Zhang, Wei

Dong, Chuang

Makino, Akihiro

BE Zhu, S

Ni, B

Ju, DY

TI Glass-forming ability and mechanical properties of Zr-Ni-Al bulk

metallic glasses with high Zr content

SO ADVANCED MATERIALS SCIENCE AND TECHNOLOGY, (IFAMST-8)

SE Materials Science Forum

CT 8th International Forum on Advanced Materials Science and Technology

(IFAMST-8)

CY AUG 01-04, 2012

CL Fukuoka Inst Technol, Fukuoka City, JAPAN

SP Saitama Inst Technol, Chinese Mat Res Soc Japan, Chinese Acad & Profess Assoc Japan

HO Fukuoka Inst Technol

AB The thermal stability and glass-forming ability (GFA) of Zr70Ni30-xAlx (x = 5 - 19) metallic alloys were investigated. Alloy with x = 8 has the largest critical diameter (d(c)) of 2 mm in the alloy series, which possesses the largest supercooled liquid region, T-rg and gamma value, simultaneously. Further addition of 2 - 4 at.% Nb to Zr70Ni22Al8 alloy by replacement of Zr increases the d(c) up to 6 mm. In addition, Zr70Ni22Al8 bulk metallic glass exhibits large compressive plastic strain of 13.8% with yielding stress of 1547 MPa and Young's modulus of 79 GPa. Addition of Nb increases the strength but lowers the plasticity.

SN 0255-5476

PY 2013

VL 750

BP 306

EP +

DI 10.4028/www.scientific.net/MSF.750.306

UT WOS:000319235900073

ER

PT J

AU Bhatt, J

Kumar, S

Dong, C

Murty, BS

AF Bhatt, Jatin

Kumar, S.

Dong, C.

Murty, B. S.

TI Tribological behaviour of Cu60Zr30Ti10 bulk metallic glass

SO MATERIALS SCIENCE AND ENGINEERING A-STRUCTURAL MATERIALS PROPERTIES

MICROSTRUCTURE AND PROCESSING

AB Dry sliding wear of as cast and different annealed states Of Cu60Zr30Ti10 bulk metallic glass was studied using pin-on-disc measurement technique. The relaxed metallic glass showed high hardness and excellent wear resistance. An inverse relation between the hardness and wear rate was observed for the as cast and annealed metallic glass. Coefficient of friction measured for all the above samples was found in a narrow range of 0.3-0.4 being less for as cast sample. (c) 2007 Published by Elsevier B.V.

RI Murty, BS/P-3354-2015; Sundaram, Kumar/A-4242-2013

OI Sundaram, Kumar/0000-0003-1737-8758; Murty, BS/0000-0002-4399-8531

SN 0921-5093

PD JUN 15

PY 2007

VL 458

IS 1-2

BP 290

EP 294

DI 10.1016/j.msea.2006.12.060

UT WOS:000246407600040

ER

PT S

AU Hiki, Y

Takahashi, H

AF Hiki, Y.

Takahashi, H.

BE Tokuyama, M

Oppenheim, I

Nishiyama, H

TI Calorimetric study of kinetic glass transition in metallic glasses

SO COMPLEX SYSTEMS-BOOK 1

SE AIP Conference Proceedings

CT 5th International Workshop on Complex Systems

CY SEP 25-28, 2007

CL Sendai, JAPAN

AB Differential scanning calorimetry (DSC) experiments were carried out for a bulk metallic glass (BMG), Zr41.2Ti13.8Cu12.5Ni10.0Be22.5, below and above the glass transition temperature T-g. The T-g values were determined from the DSC curves. A wide range of heating rate, q=dT/dt=0.1-100 K/min, was adopted for the experiment, and the q dependence of the apparent T-g was investigated. As q was decreased, the value of T-g decreased rapidly, then more slowly, and seemed to approach a constant value at low q. The experimental result of this kinetic glass transition phenomenon was analyzed on the basis of the relaxation process occurring in the transition temperature range.

SN 0094-243X

BN 978-0-7354-0501-1

PY 2008

VL 982

BP 177

EP +

UT WOS:000253834300028

ER

PT J

AU Zadorozhnyy, VY

Gorshenkov, MV

Churyukanova, MN

Zadorozhnyy, MY

Stepashkin, AA

Moskovskikh, DO

Ketov, SV

Zinnurova, LK

Sharma, A

Louzguine-Luzgin, DV

Kaloshkin, SD

AF Zadorozhnyy, V. Yu.

Gorshenkov, M. V.

Churyukanova, M. N.

Zadorozhnyy, M. Yu.

Stepashkin, A. A.

Moskovskikh, D. O.

Ketov, S. V.

Zinnurova, L. Kh.

Sharma, A.

Louzguine-Luzgin, D. V.

Kaloshkin, S. D.

TI Investigation of structure and thermal properties in composite materials

based on metallic glasses with small addition of polytetrafluoroethylene

SO JOURNAL OF ALLOYS AND COMPOUNDS

AB Bulk composites based on Cu54Pd28P18 metallic glass and polytetrafluoroethylene (PTFE of about 1 mass %) were produced by mechanical alloying method with subsequent spark plasma sintering. The spark plasma sintering was done at the temperature close to the super cooled liquid region of the metallic glass. Structure and thermal properties of the obtained metallic glass/polymeric composite samples were investigated. It was found that composite sample (Cu54Pd28P18/PTFE) has a relatively high thermal conductivity but the appearance of the crystalline phases including oxide phase (Cu2O) on the border between the metallic glass particles, affected the physical properties badly.

The obtained results can be beneficial in terms of obtaining different antifriction composite materials based on metallic glass and self-lubricated polymers. (C) 2016 Elsevier B.V. All rights reserved.

RI Andrey, Stepashkin/A-8244-2014; LOUZGUINE, Dmitri/D-2492-2010;

Zadorozhnyy, Vladislav/G-9616-2011; Ketov, Sergey/G-5558-2011

OI Andrey, Stepashkin/0000-0002-0034-7587; LOUZGUINE,

Dmitri/0000-0001-5716-4987; Ketov, Sergey/0000-0002-6002-603X;

Zadorozhnyy, Mikhail/0000-0001-8776-0595

SN 0925-8388

EI 1873-4669

PD JUN 15

PY 2017

VL 707

BP 264

EP 268

DI 10.1016/j.jallcom.2016.11.359

UT WOS:000400709800047

ER

PT J

AU Ma, GZ

Chen, D

AF Ma, G. Z.

Chen, D.

TI Design of Bulk Metallic Glasses and Glass Matrix Composites Near

Intermetallic Composition by the Principle of Competitive Growth

SO METAL SCIENCE AND HEAT TREATMENT

AB A Cu49Zr51 intermetallic is used as a base for synthesizing metallic glasses and composites with glass matrixes [(Cu49Zr51)(100 - x) Al (x) , where x = 0, 2, 4, 6, 8, 10 and 12 at.%]. The introduction of aluminum raises the microhardness and the ultimate compressive strength. In addition, the suppression of formation of crystalline phase upon the introduction of 8 at.% Al provides a glass-like structure in alloy (Cu49Zr51)(92)Al-8. The formation of the glass-like structure is discussed within the concept of competitive nucleation of different intermetallics.

RI 陈(chen), 鼎(ding)/O-5087-2015

OI 陈(chen), 鼎(ding)/0000-0003-0407-7542

SN 0026-0673

EI 1573-8973

PD NOV

PY 2016

VL 58

IS 7-8

BP 483

EP 486

DI 10.1007/s11041-016-0040-6

UT WOS:000389905700020

ER

PT J

AU Bordeenithikasem, P

Stolpe, M

Elsen, A

Hofmann, DC

AF Bordeenithikasem, Punnathat

Stolpe, Moritz

Elsen, Alexander

Hofmann, Douglas C.

TI Glass forming ability, flexural strength, and wear properties of

additively manufactured Zr-based bulk metallic glasses produced through

laser powder bed fusion

SO ADDITIVE MANUFACTURING

AB An evaluation of low-cost, high-oxygen content Zr-Cu-Al-Nb bulk metallic glasses (BMGs) produced through laser powder bed fusion (PBF) was performed. Four-point bending and wear resistance tests were used to compare the mechanical properties of the printed alloy with laboratory grade cast parts. It is shown that the laser PBF parts, while not being able to be cast as a bulk glass, can be printed amorphous up to at least several millimeters thick and yet still have reasonable mechanical properties.

SN 2214-8604

EI 2214-7810

PD MAY

PY 2018

VL 21

BP 312

EP 317

DI 10.1016/j.addma.2018.03.023

UT WOS:000435751300032

ER

PT J

AU Sun, YF

Wang, YR

Wei, BC

Li, WH

AF Sun, YF

Wang, YR

Wei, BC

Li, WH

TI Preparation of Cu-based bulk metallic glass matrix composites

SO JOURNAL OF MATERIALS SCIENCE & TECHNOLOGY

AB Cu47Ti34Zr11Ni8 bulk metallic glass (BMG) matrix composites containing in situ formed TiC particles and delta-TiCu dendrite phase were developed by copper mold cast. The thermal stability and microstructure of the composites are investigated. Room temperature compression tests reveal that the composite samples exhibit higher fracture strength and distinct plastic strain of 0.2%similar to 0.5%, comparing with that of the corresponding Cu47Ti34Zr11Ni8 monolithic BMG.

RI Wang, Yuren/B-2124-2013; Sun, Yufeng/R-3800-2016

OI Sun, Yufeng/0000-0002-6076-1026

SN 1005-0302

PD JAN

PY 2006

VL 22

IS 1

BP 73

EP 77

UT WOS:000235151200012

ER

PT J

AU Chang, YC

Wu, TT

Chen, MF

Lee, CJ

Huang, JC

Pan, CT

AF Chang, Y. C.

Wu, T. T.

Chen, M. F.

Lee, C. J.

Huang, J. C.

Pan, C. T.

TI Finite element simulation of micro-imprinting in Mg-Cu-Y amorphous alloy

SO MATERIALS SCIENCE AND ENGINEERING A-STRUCTURAL MATERIALS PROPERTIES

MICROSTRUCTURE AND PROCESSING

CT 5th International Conference on Physical and Numerical Simulation of

Material Processing (ICPNS 07)

CY OCT 23-27, 2007

CL Zhengzhou, PEOPLES R CHINA

SP Chinese Mech Engn Soc, Minerals, Met & Mat Soc, Japan Inst Met, Harbin Inst Technol, Zhengzhou Univ, Henan Polytech Univ, Zhongyuan Inst Technol

AB Mg-Cu-Y-based metallic glasses have exhibited superior glass-forming ability, and can be cast into bulk metallic glasses (BMGs). At temperatures above the glass transition temperature, the BMGs become supercooled viscous materials that can be formed into complicated shapes or patterns on micro- or even nano-scales. This paper presents the simulated forming evolution, using a finite element simulation software DEFORM 3D, and the experimental observations for the micro-imprinting of the Mg(58)Cu(31)Y(11) BMGs for making hexagonal micro-lens arrays. The results demonstrate that the imprinting is feasible and promising. (c) 2008 Elsevier B.V. All rights reserved.

RI Huang, J./C-4276-2013; Pan, CT/E-4776-2013

OI HUANG, Jacob Chih Ching/0000-0001-6843-3396

SN 0921-5093

PD JAN 15

PY 2009

VL 499

IS 1-2

BP 153

EP 156

DI 10.1016/j.msea.2007.11.117

UT WOS:000261703300035

ER

PT J

AU Mattern, N

Kuehn, U

Eckert, J

AF Mattern, Norbert

Kuehn, Uta

Eckert, Juergen

TI Structural behavior of amorphous and liquid metallic alloys at elevated

temperatures

SO JOURNAL OF NON-CRYSTALLINE SOLIDS

CT 12th International Conference on Liquid and Amorphous Metals (LAM12)

CY JUL 11-16, 2004

CL Metz, FRANCE

AB The thermal behavior of the short-range order of Pd40Cu30Ni10P20 bulk metallic glasses has been investigated in situ by means of hightemperature X-ray synchrotron diffraction. The dependence of the X-ray structure factor S(q) of the glassy state on temperature follows the Debye theory up to the glass transition. Above the glass transition temperature T-g, the temperature dependence of S(q) is altered toward a continuous development of structural changes in the liquid state with temperature. The behavior of the structure factor during heating and cooling through the glass transition gives experimental evidence for melting the glass, and for freezing the liquid, respectively at the caloric glass temperature. (c) 2007 Elsevier BN. All rights reserved.

SN 0022-3093

PD OCT 15

PY 2007

VL 353

IS 32-40

BP 3327

EP 3331

DI 10.1016/j.jnoncrysol.2007.05.079

UT WOS:000250235200070

ER

PT J

AU Chen, LY

Hu, HT

Zhang, GQ

Jiang, JZ

AF Chen, L. Y.

Hu, H. T.

Zhang, G. Q.

Jiang, J. Z.

TI Catching the Ni-based ternary metallic glasses with critical diameter up

to 3 mm in Ni-Nb-Zr system

SO JOURNAL OF ALLOYS AND COMPOUNDS

AB The Ni-based ternary bulk metallic glasses (BMGs) with critical diameter up to 3 mm were discovered in Ni-Nb-Zr system using a strategy for catching the best glass former based on relative glass forming ability of alloys. The best glass former Ni62Nb33Zr5 exhibits glass transition temperature of 877 K, supercooled liquid region of 40 K, compressive strength of about 3 GPa, plastic strain of about M. (c) 2007 Elsevier B.V. All rights reserved.

RI Chen, Lianyi/B-3156-2008

OI Chen, Lianyi/0000-0003-3720-398X

SN 0925-8388

PD SEP 27

PY 2007

VL 443

IS 1-2

BP 109

EP 113

DI 10.1016/j.jallcom.2007.02.114

UT WOS:000248736800021

ER

PT J

AU Meng, Z

Ning, L

Qi, C

Lin, L

AF Meng, Zhang

Ning, Li

Qi, Chen

Lin, Liu

TI Microformability of Bulk Amorphous Alloys in the Supercooled Liquid

Region

SO RARE METAL MATERIALS AND ENGINEERING

AB The microformability in the supercooled liquid region of 4 bulk metallic glasses (Pd40Cu30Ni10P20, Zr65Cu15Al10Ni10, Cu46Zr42Al7Y5, Zr58.5Cu15.6Al10.3Ni12.8Nb2.8) with different fragility values was investigated with microimprinting experiment on a micro-V shaped silicon mould. Results show that the bulk metallic glasses (BMGs) show different apparent viscosity. But under the condition of the same deformation, the 4 BMGs exhibit almost the same area of flowed into the groove, namely they show similar microformability. This is attributed to the adopted experimental conditions (T =1.07T(g), (epsilon) over dot = 2x10(-3) s(-1)) under which the BMGs show a Newtonian flow behavior. The FEM study also verifies this conclusion.

RI Li, Ning/B-2283-2009

SN 1002-185X

PD DEC

PY 2011

VL 40

IS 12

BP 2162

EP 2166

UT WOS:000299663200021

ER

PT J

AU Widom, M

Mihalkovic, M

AF Widom, M

Mihalkovic, M

TI Stability of Fe-based alloys with structure type C6Cr23

SO JOURNAL OF MATERIALS RESEARCH

AB Bulk metallic glass forms when liquid metal alloys solidify without crystallization. in the search for iron-based bulk glass-forming alloys of the metal-metalloid type (Fe-B- and Fe-C-based), crystals based on the structural prototype C6Cr23 often preempt the amorphous phase. Destabilizing this competing crystal structure could enhance glass formability. We carried out first-principles total energy calculations of enthalpy of formation to identify third elements that can effectively destabilize C6Cr23. Yttrium appears optimal among transition metals, and rare earths also are suitable. Atomic size is the dominant factor.

RI Widom, Michael/P-2531-2014

OI Widom, Michael/0000-0001-5972-5696

SN 0884-2914

PD JAN

PY 2005

VL 20

IS 1

BP 237

EP 242

DI 10.1557/JMR.2005.0028

UT WOS:000229292600031

ER

PT J

AU Wang, LM

Wang, RJ

Sun, LL

Wang, WH

Wang, WK

AF Wang, LM

Wang, RJ

Sun, LL

Wang, WH

Wang, WK

TI Pressure dependence of the elastic constants and vibrational

anharmonicity of Pd39Ni10CU30P21 bulk metallic glass

SO JOURNAL OF PHYSICS-CONDENSED MATTER

AB The pressure dependence of the acoustic velocities of a Pd39Ni10Cu30P21 bulk metallic glass have been investigated up to 0.5 GPa at room temperature with the pulse echo overlap method. Two independent second-order elastic coefficients C-11 and C-44 and their pressure derivatives are yielded. The vibrational anharmonicity is shown by calculating both the acoustic mode Gruneisen parameters in the long-wavelength limit and the thermal Gruneisen parameter, and this result is compared with that for the Pd40Ni40P20 bulk glass.

SN 0953-8984

PD JAN 22

PY 2003

VL 15

IS 2

BP 101

EP 107

AR PII S0953-8984(03)53465-8

DI 10.1088/0953-8984/15/2/310

UT WOS:000180989100024

ER

PT J

AU Xiao, XS

Fang, SS

Wang, Q

Wang, GM

Hua, Q

Dong, YD

AF Xiao, XS

Fang, SS

Wang, Q

Wang, GM

Hua, Q

Dong, YD

TI Effect of hot rolling on thermal stability and microstructure of

Zr52.5Al10Ni10Cu15Be12.5 bulk metallic glass

SO MATERIALS LETTERS

AB The thermal stability and microstructure of hot rolled Zr52.5Al10Ni10Cu15Be12.5 bulk metallic glass have been investigated. The decrease of thermal stability is due to the change of microstructure when the rolled reduction ratio is over 50%. Some ordered clusters embedded in the glassy matrix were regarded as the heterogeneous nuclear sites in subsequent crystallization. (C) 2004 Elsevier B.V. All rights reserved.

SN 0167-577X

PD JUL

PY 2004

VL 58

IS 19

BP 2357

EP 2360

DI 10.1016/j.matlet.2004.02.052

UT WOS:000222105000006

ER

PT J

AU Mochizuki, C

Senga, T

Shibata, M

AF Mochizuki, Chihiro

Senga, Takashi

Shibata, Masami

TI Electrodeposition of Pd-Ni-P Metallic Glass Films

SO ELECTROCHEMISTRY

AB In the present study, the electrodeposition of Pd-Ni-P metallic glass films has been investigated. The composition of the electrodeposited Pd-Ni-P films were controlled by adjusting the current density from 0.2 A dm(-2) to 2.5 A dm(-2). The results of DSC analysis demonstrated that the electrodeposited Pd-Ni-P films were metallic glass. The composition of the electrodeposited Pd-Ni-P films at 2.5 A dm(-2) was close to bulk metallic glass. The T-g and T-x of electrodeposited films were in good agreement with bulk metallic glass. The electrodeposition is able to fabricate the Pd-Ni-P metallic glass, which is close to bulk metallic glass.

SN 1344-3542

PD APR

PY 2011

VL 79

IS 4

BP 249

EP 251

DI 10.5796/electrochemistry.79.249

UT WOS:000289604600003

ER

PT J

AU Lan, S

Wei, XY

Zhou, J

Lu, ZP

Wu, XL

Feygenson, M

Neuefeind, J

Wang, XL

AF Lan, Si

Wei, Xiaoya

Zhou, Jie

Lu, Zhaoping

Wu, Xuelian

Feygenson, Mikhail

Neuefeind, Joerg

Wang, Xun-Li

TI In-situ study of crystallization kinetics in ternary bulk metallic glass

alloys with different glass forming abilities

SO APPLIED PHYSICS LETTERS

AB In-situ transmission electron microcopy and time-resolved neutron diffraction were used to study crystallization kinetics of two ternary bulk metallic glasses during isothermal annealing in the supercooled liquid region. It is found that the crystallization of Zr56Cu36Al8, an average glass former, follows continuous nucleation and growth, while that of Zr46Cu46Al8, a better glass former, is characterized by site-saturated nucleation, followed by slow growth. Possible mechanisms for the observed differences and the relationship to the glass forming ability are discussed. (C) 2014 AIP Publishing LLC.

RI Neuefeind, Joerg/D-9990-2015; Feygenson, Mikhail/H-9972-2014; Wang,

Xun-Li/C-9636-2010; Lu, Zhao-Ping/A-2718-2009

OI Neuefeind, Joerg/0000-0002-0563-1544; Feygenson,

Mikhail/0000-0002-0316-3265; Wang, Xun-Li/0000-0003-4060-8777; Lu,

Zhao-Ping/0000-0003-1463-8948

SN 0003-6951

EI 1077-3118

PD NOV 17

PY 2014

VL 105

IS 20

AR 201906

DI 10.1063/1.4901905

UT WOS:000345513300021

ER

PT J

AU Chen, QJ

Shen, J

Fan, HB

Sun, JF

Huang, YJ

Mccartney, DC

AF Chen, QJ

Shen, J

Fan, HB

Sun, JF

Huang, YJ

Mccartney, DC

TI Glass-forming ability of an iron-based alloy enhanced by Co addition and

evaluated by a new criterion

SO CHINESE PHYSICS LETTERS

AB A new Fe-based alloy that can be cast into a fully amorphous rod with a diameter of at least 16 mm by the conventional copper-mould casting technique is obtained by partially replacing Fe with Co in a previously reported Fe-based bulk metallic glass. The preliminary thermodynamic analysis indicates that the Co-containing alloy has a significantly lower Gibbs free energy difference between the undercooled melt and the corresponding crystalline solid, compared to the Co-free alloy, reflecting the dramatic role of the Co addition in stabilizing the supercooled melt and facilitating glass formation in iron-based alloys. Here, a new criterion, derived from the classical nucleation and growth theory, is introduced to evaluate the glass-forming ability of Fe-based bulk metallic glasses.

RI Huang, Yongjiang/D-4809-2009

OI McCartney, Graham/0000-0001-8608-9066

SN 0256-307X

EI 1741-3540

PD JUL

PY 2005

VL 22

IS 7

BP 1736

EP 1738

UT WOS:000230529000048

ER

PT J

AU Gravier, S

Kapelski, G

Suery, M

Blandin, JJ

AF Gravier, Sebastien

Kapelski, Georges

Suery, Michel

Blandin, Jean-Jacques

TI Thermoplastic Forming of Bulk Metallic Glasses

SO INTERNATIONAL JOURNAL OF APPLIED GLASS SCIENCE

AB Metallic glasses display interesting mechanical properties including high mechanical resistance and large capacity to store elastic energy. Since metallic glasses can now be produced in bulk conditions, they can be used for structural applications. In this new context, thermoforming of metallic glasses can be a particularly well adapted technique for production of small size components with good surface finish. As for other glasses, metallic glasses can be preferentially thermoformed in their supercooled liquid region. However, since metallic glasses are still emerging materials, the mechanisms of high temperature deformation remain not completely understood and intensive work is currently carried out to identify the elementary mechanisms of deformation. Moreover, since thermoforming is performed in the supercooled liquid region, the thermal stability of the glass to be formed is a key point for the success of the thermoforming process. Finally, it is also shown that thermoforming conditions can be used to produce multi materials associating metallic glasses and conventional metallic alloys.

SN 2041-1286

PD JUN

PY 2012

VL 3

IS 2

SI SI

BP 180

EP 187

DI 10.1111/j.2041-1294.2012.00083.x

UT WOS:000310849000008

ER

PT J

AU Kawamura, Y

Ohno, Y

AF Kawamura, Y

Ohno, Y

TI Successful electron-beam welding of bulk metallic glass

SO MATERIALS TRANSACTIONS

AB We have tried an electron beam welding of a Zr41Ti14Cu12Ni10Be23 (at%) bulk metallic glass plate that has an excellent glass forming ability and a thermally stable supercooled liquid. We have for the first time succeeded in the electron-beam welding of the bulk metallic glass plate with a thickness of 3.5 mm. No crystallization was observed in the bead and heat-affected zone. No visible defect or crack was recognized around the bead and heat-affected zone. The tensile strength of the welded bulk metallic glass was the same as that of the parent bulk metallic glass. The successful condition of the electron-beam welding was an electron acceleration voltage of 60 kV, beam currents of 15 to 20 mA and a scanning velocity of 33 mm/s.

SN 1345-9678

EI 1347-5320

PD NOV

PY 2001

VL 42

IS 11

SI SI

BP 2476

EP 2478

DI 10.2320/matertrans.42.2476

UT WOS:000172844400056

ER

PT J

AU Tosa, DJ

Serban, VA

Codrean, C

Buzdugan, D

Stoica, M

AF Tosa, D. J.

Serban, V. A.

Codrean, C.

Buzdugan, D.

Stoica, M.

TI Dual phase bulk metallic glasses fabricated by hot pressing using two

different types of glassy alloy powder

SO JOURNAL OF OPTOELECTRONICS AND ADVANCED MATERIALS

AB A new class of bulk metallic glasses (BMGs) the dual amorphous phased bulk metallic glass was studied with interest by researchers of materials science in the recent years, in order to bring together all the favorable properties for each amorphous phase. A dual phase bulk metallic glasses (DAPBMGs) fabricated by hot pressing using two different types of glassy alloy powder with the diameter of 10 mm and 5 mm in height were successfully achieved. The samples obtained were structural investigated by X-Ray diffraction (XRD), differential scanning calorimetry (DSC) and by scanning electron microscopy (SEM).

RI Serban, Viorel - Aurel/G-4719-2016; Stoica, Mihai/B-7069-2015

SN 1454-4164

EI 1841-7132

PD JUL-AUG

PY 2015

VL 17

IS 7-8

BP 1026

EP 1031

UT WOS:000359967600019

ER

PT J

AU Wang, D

Tan, H

Li, Y

AF Wang, D

Tan, H

Li, Y

TI Multiple maxima of GFA in three adjacent eutectics in Zr-Cu-Al alloy

system - A metallographic way to pinpoint the best glass forming alloys

SO ACTA MATERIALIA

AB We demonstrate that the three best glass forming alloys up to 8 mm in diameter are obtained by copper mold casting system in three adjacent eutectics in the Zr-Cu-Al alloy system via a metallographic approach. The narrow bulk glass forming range was found to require composition pinpointing to 1 at.% and a phase selection concept is developed to explain the success of our microstructure-based approach to pinpoint the best glass former in a given system. The significance of this method in discovering new BMGs is discussed. &COPY; 2005 Acta Materialia Inc. Published by Elsevier Ltd. All rights reserved.

SN 1359-6454

PD JUN

PY 2005

VL 53

IS 10

BP 2969

EP 2979

DI 10.1016/j.actamat.2005.03.012

UT WOS:000229519400012

ER

PT S

AU Soubeyroux, JL

Claret, N

Pelletier, JM

AF Soubeyroux, JL

Claret, N

Pelletier, JM

BE Schumacher, P

Warren, P

Cantor, B

TI Crystallization behaviour of Zr65-x-yTixAlyHf5Cu20Ni10 bulk metallic

alloys by in-situ neutron diffraction

SO METASTABLE, MECHANICALLY ALLOYED AND NANOCRYSTALLINE MATERIALS,

ISMANAM-2000

SE Materials Science Forum

CT International Symposium on Metastable, Mechanically Alloyed and

Nanocrystalline Materials (ISMANAM 2000)

CY JUL 09-14, 2000

CL ST CATHERINES COLL, OXFORD, ENGLAND

SP Zoz GmbH, Fisher Sci Orme Technologies, Oxford Ctr Adv Mat & Composites, Univ Oxford, Dept Mat

HO ST CATHERINES COLL

AB Several compounds of the Zr65-x-yTixAlyHf5Cu20Ni10 system were found to be bulk metallic glasses. They have been investigated by DSC, SEM and in-situ neutron diffraction. DSC and neutron studies were performed at the same heating rates in order to follow the evolution of the characteristic temperatures. The first phase appearing at crystallization can be correlated to a quasicrystalline phase.

SN 0255-5476

BN 0-87849-865-6

PY 2001

VL 360-3

BP 37

EP 42

DI 10.4028/www.scientific.net/MSF.360-362.37

UT WOS:000168667500006

ER

PT J

AU Qiao, DC

Peker, A

AF Qiao, Dongchun

Peker, Atakan

TI Enhanced glass forming ability in Zr-based bulk metallic glasses with Hf

Addition

SO INTERMETALLICS

AB We report on the partial substitution of Hf for Zr in Zr57Nb5Cu15.4Ni12.6Al10 bulk metallic glass (BMG) and the resulting enhanced glass forming ability and processability. Critical casting thickness increased from less than 14 mm for Zr57Nb5Cu15.4Ni12.6Al10 to more than 16 mm for Zr45Hf12Nb5Cu15.4Ni12.6Al50. This improvement contrasts with earlier reports of reduced glass forming ability with partial substitution of Hf for Zr. Furthermore, the new BMG alloy exhibited one of the best bend ductility reported to date for BMG samples that are more than 4 mm thick. The improved glass forming ability with Hf substitution is presented and the underlying factors for improved glass forming ability are discussed. (C) 2012 Elsevier Ltd. All rights reserved.

SN 0966-9795

PD MAY

PY 2012

VL 24

BP 115

EP 119

DI 10.1016/j.intermet.2012.01.028

UT WOS:000302432800018

ER

PT J

AU Jia, P

Guo, H

Li, Y

Xu, J

Ma, E

AF Jia, P

Guo, H

Li, Y

Xu, J

Ma, E

TI A new Cu-Hf-Al ternary bulk metallic glass with high glass forming

ability and ductility

SO SCRIPTA MATERIALIA

AB We have discovered a new Cu-based bulk metallic glass (BMG). Although of a simple Cu49Hf42Al9 ternary composition, the as-cast alloy is a monolithic, uniform BMG with a critical diameter as large as 10 mm. The width of the supercooled liquid region AT, and the reduced glass transition temperature T-rg for this glass are 85 K and 0.62, respectively. In addition to its high glass-forming ability and high density of similar to 11 g/cc, this BMG exhibits high ductility with a compressive plastic strain of 11-13%, making it a good candidate for applications as well as for studies of deformation behavior of Cu-based BMGs. (c) 2006 Published by Elsevier Ltd. on behalf of Acta Materialia Inc.

RI Guo, Hua/D-5066-2013; Ma, En/A-3232-2010

SN 1359-6462

PD JUN

PY 2006

VL 54

IS 12

BP 2165

EP 2168

DI 10.1016/j.scriptamat.2006.02.042

UT WOS:000237163000034

ER

PT J

AU Johnson, WL

AF Johnson, WL

TI Bulk metallic glasses - A new engineering material

SO CURRENT OPINION IN SOLID STATE & MATERIALS SCIENCE

AB The development of new metallic alloys which form bulk glasses at low cooling rates has led to significant advances in the study of undercooled liquid metals and the glass transition in metallic systems. For the first time it has become possible to carry out measurements of thermophysical properties such as specific heat, viscosity, atomic diffusion, and crystal nucleation rates for liquid alloys over the full temperature range of the undercooled melt. These new materials exhibit a rich variety of phenomena, such as liquid crystal phase separation and nanocrystalline phase formation, and have interesting potential as engineering materials.

SN 1359-0286

PD JUN

PY 1996

VL 1

IS 3

BP 383

EP 386

DI 10.1016/S1359-0286(96)80029-5

UT WOS:A1996VE41500009

ER

PT J

AU Huang, HG

Ke, HB

Zhang, P

Wang, YM

Zhang, PG

Wu, M

Liu, TW

AF Huang, H. G.

Ke, H. B.

Zhang, P.

Wang, Y. M.

Zhang, P. G.

Wu, M.

Liu, T. W.

TI Effect of minor alloying on the glass formation of U-based alloys

SO JOURNAL OF ALLOYS AND COMPOUNDS

AB The effect of minor Sn alloying on the glass formation of U-based alloys is investigated. A string of U-Fe alloys designed with the eutectic rule are all formed in a partially glassy state under melt-spinning. Minor addition of Sn gives rise to the full amorphisation of the alloys, and the achievement of the reduced glass transition temperature for new U-Fe-Sn alloys comparable to some ordinary bulk metallic glasses. This role of Sn microalloying may be ascribed to improved packing efficiency of icosahedral atomic clusters underlying in the alloy liquids. It is suggested that local atomic packing is a necessary consideration for exploiting desirable U-based multicomponent alloys with high glass forming ability. (C) 2016 Elsevier B.V. All rights reserved.

SN 0925-8388

EI 1873-4669

PD DEC 15

PY 2016

VL 688

BP 599

EP 604

DI 10.1016/j.jallcom.2016.07.229

PN B

UT WOS:000384439000075

ER

PT J

AU Shamlaye, KF

Laws, KJ

Loffler, JF

AF Shamlaye, Karl F.

Laws, Kevin J.

Loffler, Jorg F.

TI Exceptionally broad bulk metallic glass formation in the Mg-Cu-Yb system

SO ACTA MATERIALIA

AB This study presents an extensive series of novel bulk metallic glasses (BMGs) which broadly span all three corners of the Mg-Cu-Yb ternary system. Over 30 alloys were synthesised within a wide composition range from (at.%): Mg: 13-55, Cu: 17.5-45.5, and Yb: 9-70. In terms of composition, this ternary system is considered to be one of the broadest for bulk glass formation known - this probably due to the three elements' thermodynamic compatibility and unique combinations of atomic radii, which generate specific, favoured structural topologies. The investigation reports the design method, critical casting size, thermophysical characteristics and mechanical properties of these new BMGs. (C) 2017 Acta Materialia Inc. Published by Elsevier Ltd. All rights reserved.

SN 1359-6454

EI 1873-2453

PD APR 15

PY 2017

VL 128

BP 188

EP 196

DI 10.1016/j.actamat.2017.02.013

UT WOS:000397692600019

ER

PT J

AU Kim, J

AF Kim, Jonghyun

TI Weldability of Cu54Zr22Ti18Ni6 bulk metallic glass by ultrasonic welding

processing

SO MATERIALS LETTERS

AB Cu54Zr22Ti18Ni6 (numbers indicate at%) bulk metallic glass with glass forming ability of 6 mm was joined using ultrasonic welding. After welding fully amorphous materials, crystallization was investigated using micro area x-ray diffraction, scanning electron microscopy, and differential scanning calorimetry. However, there was no crystallization through the joint and the weld interface could not be ascertained. In view of joint morphology and phase stability, ultrasonic welding processing shows a potent capability for joining bulk metallic glass materials. (C) 2014 Elsevier B.V. All rights reserved.

SN 0167-577X

EI 1873-4979

PD SEP 1

PY 2014

VL 130

BP 160

EP 163

DI 10.1016/j.matlet.2014.05.056

UT WOS:000338804300044

ER

PT J

AU Xia, L

Ding, D

Shan, ST

Dong, YD

AF Xia, L

Ding, D

Shan, ST

Dong, YD

TI The glass forming ability of Cu-rich Cu-Hf binary alloys

SO JOURNAL OF PHYSICS-CONDENSED MATTER

AB We studied the glass forming ability (GFA) of Cu-rich Cu-Hf binary alloys and found that some of the alloys can be prepared as bulk metallic glasses with maximum diameter up to 2 mm by a conventional Cu-mould casting. The best glass former within the compositional range studied is off-eutectic Cu65Hf35 alloy, which is markedly different from the prediction from the multicomponent and deep eutectic rules. The GFA, thermal stability, kinetics of the glass transition and crystallization for Cu65Hf35 glassy rods were studied. The glass formation mechanism for binary Cu-Hf alloys was investigated from the thermodynamic point of view. It is suggested that the better GFA of offeutectic Cu65Hf35 alloy could be due to its higher value of the parameter gamma*, which is defined as the ratio between the driving force for glass formation and the resistance of glass formation to crystallization.

OI Xia, Lei/0000-0001-9198-1497

SN 0953-8984

PD APR 19

PY 2006

VL 18

IS 15

BP 3543

EP 3548

DI 10.1088/0953-8984/18/15/002

UT WOS:000237410400007

ER

PT J

AU Wang, S

Ye, YF

Shi, SQ

Yang, Y

AF Wang, S.

Ye, Y. F.

Shi, S. Q.

Yang, Y.

TI The thermal history effect on shear band initiation in metallic glass

SO JOURNAL OF APPLIED PHYSICS

AB The effect of thermal history on shear band initiation in metallic glass is investigated with spherical nanoindentation. Our results clearly show that the indentation size effect on the metallic-glass hardness varies systematically with the thermal history, which is in excellent agreement with the softening-induced shear-band initiation model we recently developed. On a fundamental level, the outcome of our research establishes a correlation between the shear modulus and the critical length scale for initiating an autocatalytic shear-band growth in metallic glasses. Published by AIP Publishing.

RI Yang, Yong/G-9148-2011; Shi, San-Qiang/B-8836-2008

OI Yang, Yong/0000-0002-0491-8295; Shi, San-Qiang/0000-0003-4189-0786; Ye,

Yifan/0000-0002-9172-6514

SN 0021-8979

EI 1089-7550

PD JUN 28

PY 2016

VL 119

IS 24

AR 245113

DI 10.1063/1.4954873

UT WOS:000379163800047

ER

PT J

AU Meng, QG

Zhou, JK

Zheng, HX

Li, JG

AF Meng, QG

Zhou, JK

Zheng, HX

Li, JG

TI Fragility of superheated melts and glass-forming ability in Pr-based

alloys

SO SCRIPTA MATERIALIA

AB The kinetic viscosity (eta) of superheated melts, thermal properties (T-x, T-m, T-L) and X-ray diffraction analysis on the Pr-based bulk metallic glasses (BMG) are reported and discussed. A new refined concept, the superheated fragility defined as M' = E(s)delta(x)/k(B), has been developed based on common solidification theory and the Arrhenius equation. The interrelationship between this kind of fragility and the glass-forming ability (GFA) is elaborated on and evaluated in Pr-based BMG and Al-based amorphous ribbon alloys. Using viscosity data of superheated melts, it is shown, theoretically and experimentally, that the fragility parameter M' may be used as a GFA indicator for metallic alloys. (c) 2005 Acta Materialia Inc. Published by Elsevier Ltd. All rights reserved.

RI Zheng, Hongxing/D-7472-2011

SN 1359-6462

PD MAR

PY 2006

VL 54

IS 5

BP 777

EP 781

DI 10.1016/j.scriptamat.2005.11.023

UT WOS:000234775400014

ER

PT S

AU Zhang, X

Ma, J

Bai, R

Li, Q

Sun, BL

Shen, CY

AF Zhang Xiang

Ma Jiang

Bai Ran

Li Qian

Sun Bingli

Shen Changyu

BE Gao, QJ

TI Polymer Micro Hot Embossing with Bulk Metallic Glass Mold Insert

SO MACHINERY, MATERIALS SCIENCE AND ENGINEERING APPLICATIONS

SE Advanced Materials Research

CT 2nd International Conference on Machinery, Materials Science and

Engineering Applications (MMSE 2012)

CY JUN 16-17, 2012

CL Wuhan Univ Sci & Technol, Wuhan, PEOPLES R CHINA

SP Univ Iowa, Hubei Mech Engn Soc, Chinese Mech Engn Soc

HO Wuhan Univ Sci & Technol

AB Polymer microstructures are used more and more in many fields. Hot embossing is one of molding processing to achieve micro polymer components. In this paper, bulk metallic glass was selected as mold material to fabricate mold insert of micro hot embossing. Traditional UV-lithography and ICP-etching were used to achieve micro features on silicon wafer. And then, micro features were transferred from silicon wafer to bulk metallic glass mold insert above its glass transition temperature. Finally, applied bulk metallic glass mold insert to replicate polymer microstructure with hot embossing. Three commonly used thermoplastic polymers: high-density polyethylene (HDPE), polypropylene (PP) and polycarbonate (PC) were selected in this study Experiments show that microstructures can have a good replication from bulk metallic glass mold insert to the thermoplastic polymer using hot embossing.

RI Shen, Changyu/E-7519-2016

SN 1022-6680

BN 978-3-03785-409-9

PY 2012

VL 510

BP 639

EP +

DI 10.4028/www.scientific.net/AMR.510.639

UT WOS:000312435000121

ER

PT J

AU Bouchareb, A

Bendjemil, B

Piccin, R

Baricco, M

AF Bouchareb, Abderrezak

Bendjemil, Badis

Piccin, Rafael

Baricco, Marcello

TI Influence of Rare-Earth Substitution for Iron in FeCrMoCB Bulk Metallic

Glasses

SO CHINESE PHYSICS LETTERS

AB The effects of rare earth addition on the glass forming ability of Fe(50-x)Cr(15)Mo(14)C(15)B(6)M(x) (x = 0, 2 and M=Y, Gd) bulks and ribbons are studied. The thermal and structural properties of the samples are measured by a combination of differential scanning calorimetry (DSC), x-ray diffraction and scanning electron microscopy. Chemical compositions are checked by energy dispersive spectroscopy analysis. The copper mold casting technique leads to a fully amorphous structure up to 2mm only for compositions containing Y or Gd. In the case of ribbons, a fully amorphous phase is observed for all the compositions. The roles of Y and Gd are discussed on the basis of melting behavior analyzed by high-temperature DSC. Such elements act as oxygen scavengers, avoiding heterogeneous nucleation.

RI Baricco, Marcello/B-4075-2013

OI Baricco, Marcello/0000-0002-2856-9894

SN 0256-307X

PD JUL

PY 2010

VL 27

IS 7

AR 076103

DI 10.1088/0256-307X/27/7/076103

UT WOS:000279994700053

ER

PT J

AU Kabaer, M

Kucuk, I

AF Kabaer, Mehmet

Kucuk, Ilker

TI Computational Modeling of Glass Forming Ability and Critical Diameter of

Magnetic Bulk Amorphous Alloys

SO JOURNAL OF SUPERCONDUCTIVITY AND NOVEL MAGNETISM

AB This paper presents a new approach based on an artificial neural network (ANN) and a genetic algorithm to compute the glass forming ability of magnetic bulk amorphous alloys using previously reported data in the literature. The developed network has been trained using the genetic algorithm and Levenberg-Marquardt algorithm. The model can assist in predicting the relation between the chemical compositions and the glass forming ability of magnetic bulk amorphous alloys.

SN 1557-1939

EI 1557-1947

PD JAN

PY 2011

VL 24

IS 1-2

BP 693

EP 697

DI 10.1007/s10948-010-0939-x

UT WOS:000289855700114

ER

PT J

AU Zhao, YH

Pan, MX

Zhao, DQ

Wang, WH

Eckert, J

AF Zhao, YH

Pan, MX

Zhao, DQ

Wang, WH

Eckert, J

TI Magnetic transitions in Dy-microalloyed Fe-based bulk metallic glasses

SO JOURNAL OF PHYSICS D-APPLIED PHYSICS

AB The magnetic properties of Fe70-xDyxZr8Mo5W2B15 (0 <= x <= 5) bulk metallic glasses (BMGs) are studied using dc and ac susceptibility measurements. A re-entrant spin glass behaviour is observed in the BMGs and the phenomenon is ascribed to Dy-microalloying-induced site frustration. The magnetic properties of the BMGs are found to be tunable by appropriate selection of the Dy content, and the schematic magnetic phase diagram as a function of Dy content is derived.

SN 0022-3727

PD JUL 7

PY 2005

VL 38

IS 13

BP 2162

EP 2165

DI 10.1088/0022-3727/38/13/013

UT WOS:000230714600015

ER

PT J

AU Wang, HB

Ma, LX

Li, L

Zhang, B

AF Wang, H. B.

Ma, L. X.

Li, L.

Zhang, B.

TI Fabrication of Fe-based bulk metallic glasses from low-purity industrial

raw materials

SO JOURNAL OF ALLOYS AND COMPOUNDS

AB Bulk metallic glasses with nominal compositions of (Fe71.2B24Y4.8)(96)Nb4-xTix (x = 0, 1, 2, 3, 4) were prepared with industrial-purity Fe-B alloys and sponge titanium. Partially replacing Nb with Ti (sponge titanium) substantially increased the thermal stability and glass forming ability of the alloys. The best glass former was a rod of (Fe71.2B24Y4.8)(96)Nb2Ti2, which had a critical diameter of 6 mm and a supercooled liquid region (Delta Tx) of approximate to 103 K, both among the best values in Fe-based bulk metallic glasses. We also investigated the magnetic and mechanical properties of the (Fe71.2B24Y4.8)(96)Nb2Ti2 BMG, which exhibited high saturation magnetization and compressive fracture strength of approximate to 121 emu/g and 3.4 GPa, respectively. These results demonstrate that the present Fe-based bulk metallic glasses might be a potential candidate for commercial applications with low cost and good performance. (C) 2015 Elsevier B.V. All rights reserved.

SN 0925-8388

EI 1873-4669

PD APR 25

PY 2015

VL 629

BP 1

EP 4

DI 10.1016/j.jallcom.2014.11.228

UT WOS:000349699700001

ER

PT J

AU Lu, ZP

Liu, CT

AF Lu, ZP

Liu, CT

TI A new glass-forming ability criterion for bulk metallic glasses

SO ACTA MATERIALIA

AB A new indicator of glass-forming ability (GFA) for bulk metallic glasses (BMGs) is proposed based on crystallization processes during cooling and reheating of the supercooled liquid. The interrelationship between this new parameter and the critical cooling rate or critical section thickness is elaborated and discussed in comparison with two other representatives, i.e. reduced glass transition temperature T-rg (= T-g/T-l, where T-g and T-l are the glass transition temperature and liquidus temperature, respectively) and supercooled liquid range DeltaT(xg) (= T-x-T-g, where T-x is the onset crystallization temperature and T-g the glass transition temperature). Our results have shown that DeltaT(xg) alone cannot infer relative GFA for BMGs while the new parameter gamma, defined as T-x/(T-g + T-l), has a much better interrelationship with GFA than T-rg. An approximation of the critical cooling rate and critical section thickness for glass formation in bulk metallic glasses is also formulated and evaluated. (C) 2002 Published by Elsevier Science Ltd on behalf of Acta Materialia Inc.

RI Lu, Zhao-Ping/A-2718-2009

OI Lu, Zhao-Ping/0000-0003-1463-8948; Liu, Chain Tsuan/0000-0001-7888-9725

SN 1359-6454

EI 1873-2453

PD AUG 1

PY 2002

VL 50

IS 13

BP 3501

EP 3512

AR PII S1359-6454(02)00166-0

DI 10.1016/S1359-6454(02)00166-0

UT WOS:000177559400017

ER

PT J

AU Greer, AL

AF Greer, AL

TI Metallic glasses

SO CURRENT OPINION IN SOLID STATE & MATERIALS SCIENCE

AB Multicomponent metallic glasses are being made in ever greater dimensions, and subjected to a wider range of shaping processes. Compositions are under active development to improve soft-magnetic, hard-magnetic and mechanical properties. Significant advances have also been made in exploiting metallic glasses as precursors in making ultra-fine grained materials with useful properties.

RI Greer, Alan Lindsay/G-1977-2011; Greer, Lindsay/E-9433-2017

SN 1359-0286

PD AUG

PY 1997

VL 2

IS 4

BP 412

EP 416

DI 10.1016/S1359-0286(97)80081-2

UT WOS:A1997XV11700004

ER

PT J

AU Li, G

Gao, YP

Chi, ZH

Liu, J

Zhang, T

Liu, RP

AF Li, Gong

Gao, Y. P.

Chi, Z. H.

Liu, J.

Zhang, T.

Liu, R. P.

TI Difference in microstructure of Zr41Ti14Cu12.5Ni10Be22.5 glasses

prepared in a 52 m drop tube and by water quenching

SO PHILOSOPHICAL MAGAZINE LETTERS

AB A 52 m drop tube has been used to solidify bulk-glass-forming Zr41Ti14Cu12.5Ni10Be22.5 alloy. Glassy balls with different sizes solidified from the droplets whose structural features, glass-transition behaviour and crystallization kinetics have been investigated. The results indicate that the apparent activation energies of the glass transition and main crystallization reaction are significantly different from those of samples prepared by water quenching. The structural difference between the two types of glassy specimen is revealed by compression studies and in situ energy-dispersive X-ray diffraction. The results are important for understanding the structural features of bulk-forming glasses.

SN 0950-0839

EI 1362-3036

PY 2008

VL 88

IS 7

BP 543

EP 551

DI 10.1080/09500830802290557

UT WOS:000258265500008

ER

PT J

AU Andreoli, AF

Ponsoni, JB

Soares, C

de Oliveira, MF

Kiminami, CS

AF Andreoli, Angelo Fernandes

Ponsoni, Jessica Bruna

Soares, Carolina

de Oliveira, Marcelo Falcao

Kiminami, Claudio Shyinti

TI Resistance upset welding of Zr-based bulk metallic glasses

SO JOURNAL OF MATERIALS PROCESSING TECHNOLOGY

AB Zr-based bulk metallic glasses represent an emerging important class of structural materials with interesting properties due to its amorphous nature. In this study, the structural and microstructural transformations to the heat-affected zone of a Zr-based bulk metallic glass welded by the resistance upset technology were investigated. X-ray diffraction, optical and scanning electron microscopy were used to characterize the base metal and the heat-affected zone. Vickers microhardness testing was performed to assess the mechanical response of the welded joints. The amorphous phase volume fraction measured along the welded joints shows no difference between the base metal and the heat-affected zone. It was possible to achieve a sound weld with no crystallization of the base metal.

SN 0924-0136

PD MAY

PY 2018

VL 255

BP 760

EP 764

DI 10.1016/j.jmatprotec.2018.01.034

UT WOS:000431837700079

ER

PT J

AU Meng, QG

Zhang, SG

Li, JG

Bian, XF

AF Meng, Q. G.

Zhang, S. G.

Li, J. G.

Bian, X. F.

TI Strong liquid behavior of Pr55Ni25Al20 bulk metallic glass

SO JOURNAL OF ALLOYS AND COMPOUNDS

AB The dynamic viscosities of the supercooled and superheated liquids for Pr55Ni25Al20 bulk metallic glass-forming alloy were measured as a function of temperature by the three-point beam bending and oscillating vessel methods, respectively. It was found that the viscosity over the entire experimental temperature interval could be well fitted by the Cohen-Grest relation, and the supercooled liquid of Pr55Ni25Al20 alloy behaved much closer to strong glasses than the other reported metallic glasses. The differences of entropy, enthalpy, and Gibbs free energy between the supercooled liquid and the crystal for Pr55Ni25Al20 alloy were calculated based on the specific heat capacity. It is shown that the glass-forming ability is correlated more strongly with the liquid-crystal Gibbs free energy difference than with the fragility parameter m. (c) 2006 Elsevier B.V. All rights reserved.

SN 0925-8388

PD APR 4

PY 2007

VL 431

IS 1-2

BP 191

EP 196

DI 10.1016/j.jallcom.2006.05.092

UT WOS:000245498300036

ER

PT S

AU Chen, TJ

AF Chen, Tie Jun

BE Chen, WZ

Dai, P

Chen, YL

Wang, QT

Jiang, Z

TI Glass-forming ability and crystallization kinetics of Hf-Ti-Cu-Ni-Al

bulk metallic glass

SO ADVANCED MECHANICAL DESIGN, PTS 1-3

SE Advanced Materials Research

CT 3rd International Conference on Manufacturing Science and Engineering

(ICMSE 2012)

CY MAR 27-29, 2012

CL Xiamen, PEOPLES R CHINA

SP Fujian Univ Technol, Xiamen Univ, Fuzhou Univ, Huaqiao Univ, Univ Wollongong, Fujian Mech Engn Soc, Hong Kong Ind Technol Res Ctr

AB Multi-component Hf45.6Cu27.8Ni9.3Ti5Al12.4 bulk metallic glasses (BMGs) were prepared successfully by casted into the water-cooled Cu mold. Characterization of the casted Hf45.6Cu27.8Ni9.3Ti5Al12.4 rods was carried out by X-ray diffraction. The thermal stability and crystallization kinetics were followed by differential scanning calorimetry. The results show that the alloy Hf45.6Cu27.8Ni9.3Ti5Al12.4 had a critical cylindrical rod diameter for glass formation, Dc, of 7 mm and the largest cross-sectional diameter (about 12.4mm) can be obtained in the ideal condition. The critical cooling rate for glass formation is 6.48K/s. The Hf45.6Cu27.8Ni9.3Ti5Al12.4 BMG has larger glass forming ability and higher thermal stability.

SN 1022-6680

BN 978-3-03785-372-6

PY 2012

VL 479-481

BP 1786

EP 1789

DI 10.4028/www.scientific.net/AMR.479-481.1786

UT WOS:000311979400357

ER

PT J

AU Lu, ZP

Hu, X

Li, Y

Ng, SC

AF Lu, ZP

Hu, X

Li, Y

Ng, SC

TI Glass forming ability of La-Al-Ni-Cu and Pd-Si-Cu bulk metallic glasses

SO MATERIALS SCIENCE AND ENGINEERING A-STRUCTURAL MATERIALS PROPERTIES

MICROSTRUCTURE AND PROCESSING

CT 10th International Conference on Rapidly Quenched and Metastable

Materials (RQ10)

CY AUG 23-27, 1999

CL BANGALORE, INDIA

SP Indian Inst Sci

AB Onset (T-m) and offset (T-1) melting temperatures of glass forming La-Al-Cu-Ni-(Co) and Pd-Cu-Si alloys were measured by studying the melting of these alloys using differential thermal analysis, The compositional dependence of glass transition temperature T-g, and the above two temperatures for these bulk amorphous alloys are presented. The effect of T-1 and T-rg on glass forming ability for these bulk amorphous alloys will also be evaluated. The correlation between glass forming ability and T-rg given by T-g/T-1 and T-g/T-m will be discussed. (C) 2001 Elsevier Science B.V. All rights reserved.

RI Lu, Zhao-Ping/A-2718-2009

OI Lu, Zhao-Ping/0000-0003-1463-8948; Li, Yi/0000-0001-7123-681X; Hu,

Xiao/0000-0002-0941-4205

SN 0921-5093

PD MAY 31

PY 2001

VL 304

SI SI

BP 679

EP 682

DI 10.1016/S0921-5093(00)01563-X

UT WOS:000168457100129

ER

PT J

AU Heinrich, J

Busch, R

Nonnenmacher, B

AF Heinrich, Jochen

Busch, Ralf

Nonnenmacher, Bernd

TI Processing of a bulk metallic glass forming alloy based on industrial

grade Zr

SO INTERMETALLICS

AB Bulk metallic glasses feature extraordinary properties especially being flexible as polymers and stronger than many metals at a time. Since they are also suitable for polymer-like net-shape processing, they can represent a superior alternative for investment casting in terms of material properties and processing efforts. We propose a bulk metallic glass forming alloy based on industrial grade Zr free of Be and Ni with the composition Zr59.3Cu28.8Al10.4Nb1.5 (at%), an alloying and feedstock production method and a horizontal vacuum cold-chamber die casting process for an economical production of bulk amorphous castings. The developments are presented along with analyses of thermophysical data of the alloy which will be compared with those of the alloy Zr58.5Cu15.6Ni12.8Al10.3Nb2.8 (Vitreloy 106a). (c) 2012 Elsevier Ltd. All rights reserved.

SN 0966-9795

PD JUN

PY 2012

VL 25

BP 1

EP 4

DI 10.1016/j.intermet.2012.02.011

UT WOS:000303905600001

ER

PT J

AU Corteen, J

Rainforth, M

Todd, I

AF Corteen, J.

Rainforth, M.

Todd, I.

TI A mathematical approach to transformation toughening in bulk metallic

glasses

SO SCRIPTA MATERIALIA

AB A mathematical framework used to describe transformation toughening in zirconia-based ceramics is adapted to apply to transformation toughening in bulk metallic glass matrix composites. The method is applied to the Cu(47.5)Zr(47.5)Al(5) bulk metallic glass, showing that the low volume change of transformation in this alloy leads to negligible toughening via the proposed mechanism. An alternative mechanism for toughening is presented, whereby shear bands propagate more easily in the early stages of advance. (C) 2011 Acta Materialia Inc. Published by Elsevier Ltd. All rights reserved.

OI Todd, Iain/0000-0003-0217-1658; Rainforth, William/0000-0003-3898-0318

SN 1359-6462

PD SEP

PY 2011

VL 65

IS 6

BP 524

EP 527

DI 10.1016/j.scriptamat.2011.06.018

UT WOS:000293932900017

ER

PT J

AU Yu, HB

Hu, J

Xia, XX

Sun, BA

Li, XX

Wang, WH

Bai, HY

AF Yu, H. B.

Hu, J.

Xia, X. X.

Sun, B. A.

Li, X. X.

Wang, W. H.

Bai, H. Y.

TI Stress-induced structural inhomogeneity and plasticity of bulk metallic

glasses

SO SCRIPTA MATERIALIA

AB The correlation between stress-induced structural inhomogeneity and plasticity in bulk metallic glasses (BMGs) was studied. It was found that the structural inhomogeneity induced by an appropriate stress field can markedly enhance macroscopic compressive plasticity in various monolithic BMGs. The origin for the plasticization is correlated to the size of the plastic zone of BMGs. (C) 2009 Acta Materialia Inc. Published by Elsevier Ltd. All rights reserved.

RI Yu, Hai Bin/E-5312-2010; Sun, Baoan/C-6441-2012

OI Yu, Hai Bin/0000-0003-0645-0187; Sun, Baoan/0000-0001-5306-1817

SN 1359-6462

PD SEP

PY 2009

VL 61

IS 6

BP 640

EP 643

DI 10.1016/j.scriptamat.2009.06.005

UT WOS:000268371900022

ER

PT J

AU Lu, YZ

Huang, GK

Qin, ZX

Lu, X

Huang, YJ

AF Lu, Yunzhuo

Huang, Guokun

Qin, Zuoxiang

Lu, Xing

Huang, Yongjiang

TI Mossbauer study of the ultrahigh glass-forming ability in FeCoCrMoCBY

alloy system

SO VACUUM

AB The authors report the effect of cobalt addition on the glass-forming ability (GFA) in Fe-Co-Cr-Mo-C-B-Y bulk metallic glasses (BMGs) by using Mossbauer experiments. Among the studied Fe-based alloy system, the alloy with 7% Co (Co7 alloy) displays the largest electronic quadrupole splitting. The origin of the superhigh GFA for the Co7 alloy was discussed in terms of its spatially homogeneous distribution of elements that reduces the element segregation and retards the crystallization. (C) 2017 Elsevier Ltd. All rights reserved.

OI Lu, Yunzhuo/0000-0002-7322-6815

SN 0042-207X

PD JUL

PY 2017

VL 141

BP 173

EP 175

DI 10.1016/j.vacuum.2017.04.017

UT WOS:000402352000026

ER

PT J

AU Jiang, QK

Zhang, GQ

Chen, LY

Wu, JZ

Zhang, HG

Jiang, JZ

AF Jiang, Q. K.

Zhang, G. Q.

Chen, L. Y.

Wu, J. Z.

Zhang, H. G.

Jiang, J. Z.

TI Glass formability, thermal stability and mechanical properties of

La-based bulk metallic glasses

SO JOURNAL OF ALLOYS AND COMPOUNDS

AB Substitution effects on glass forming ability (GFA) in a La-based alloy system have been studied. Fully amorphous La62Al14(Cu5/6Ag1/6)(14)Ni5Co5 alloy with at least 20 mm in diameter has been prepared by copper mold suction casting. It is found that reduced glass transition temperature and gamma value could be used to correlate with the critical size for forming bulk metallic glass (BMG) in the La-based alloy system. Thermal and mechanical properties of the BMG are studied. The low glass transition temperature, high thermal stability and exceptional higher GFA demonstrate that the BMG alloys become useful for both scientific and engineering applications. (c) 2006 Elsevier B.V. All rights reserved.

RI Chen, Lianyi/B-3156-2008

OI Chen, Lianyi/0000-0003-3720-398X

SN 0925-8388

PD NOV 9

PY 2006

VL 424

IS 1-2

BP 183

EP 186

DI 10.1016/j.jallcom.2006.07.109

UT WOS:000241414900034

ER

PT J

AU Sun, BR

Xin, SW

Shen, TD

AF Sun, B. R.

Xin, S. W.

Shen, T. D.

TI Low-temperature magnetization and magnetic exchange interactions in

Fe40Ni40P14B6 bulk metallic glasses

SO JOURNAL OF MAGNETISM AND MAGNETIC MATERIALS

AB One of the fundamental magnetic properties - the low-temperature magnetization as well as the exchange interactions derived by using a spin-wave excitation theory - has been well studied in glassy ribbons rather than bulk metallic glasses. The paper studies the temperature-dependent magnetization of a bulk Fe40Ni40P14B6 alloy, derives the range and fluctuation of exchange interactions, and compares these magnetic behaviors with those of well-studied glassy ribbons. The low-temperature magnetization of our bulk glass can be well expressed by a spin-wave excitation theory, which has been widely utilized to study the temperature-dependent magnetization of ferromagnetic glassy ribbons. Moreover, both the derived spin-wave stiffness coefficient and the derived range and fluctuation of exchange interactions in our bulk metallic glass are very similar to those in glassy ribbons, indicative of the similarity in magnetic exchange interaction between bulk metallic glasses and glassy ribbons.

SN 0304-8853

EI 1873-4766

PD MAY 1

PY 2017

VL 429

BP 276

EP 280

DI 10.1016/j.jmmm.2017.01.017

UT WOS:000397201200041

ER

PT J

AU Dou, LT

Liu, HS

Hou, L

Xue, L

Yang, WM

Zhao, YC

Chang, CT

Shen, BL

AF Dou, Lintao

Liu, Haishun

Hou, Long

Xue, Lin

Yang, Weiming

Zhao, Yucheng

Chang, Chuntao

Shen, Baolong

TI Effects of Cu substitution for Fe on the glass-forming ability and soft

magnetic properties for Fe-based bulk metallic glasses

SO JOURNAL OF MAGNETISM AND MAGNETIC MATERIALS

AB The effects of Cu substitution for Fe on the glass forming ability (GFA) and soft magnetic properties for Fe72-xCuxB20Si4Nb4 (x=0.0, 0.2, 0.4, 0.6, 0.8, and 1.0) bulk metallic glasses (BMGs) are investigated. It is found that the investigated BMGs exhibit large GFA as well as excellent soft magnetic properties, and proper substitution of Fe by Cu improves the saturation magnetization, coercive force, and effective permeability without obvious deterioration of the GFA. (C) 2014 Elsevier B.V. All rights reserved.

SN 0304-8853

EI 1873-4766

PD MAY

PY 2014

VL 358

BP 23

EP 26

DI 10.1016/j.jmmm.2014.01.014

UT WOS:000332393500005

ER

PT J

AU Jo, CL

Xia, L

Ding, D

Dong, YD

AF Jo, CL

Xia, L

Ding, D

Dong, YD

TI Glass formation ability and kinetics of the Gd55Al20Ni25 bulk metallic

glass

SO CHINESE PHYSICS LETTERS

AB We report a new bulk glass-fornling alloy Gd55Al20Ni25. The bulk sample of the alloy is prepared in the shape of rods in diameter 2 mm by suction casting. The rod exhibits typical amorphous characteristics in the x-ray diffraction pattern, paramagnetic property at 300 K, distinct glass transition and multi-step crystallization behaviour in differential scanning calorimetry traces. The glass formation ability of the alloy is investigated by using the reduced glass transition temperature T-rg and the parameter gamma. Kinetics of glass transition and primary crystallization is also studied. The fragility parameter m obtained from the Vogel-Fulcher-Tammann dependence of glass transition temperature T-g on 1n phi (phi is the heating rate) classifies the bulk metallic glasses into the intermediate category according to Angell's classification.

OI Xia, Lei/0000-0001-9198-1497

SN 0256-307X

PD MAR

PY 2006

VL 23

IS 3

BP 672

EP 674

DI 10.1088/0256-307X/23/3/041

UT WOS:000235928800041

ER

PT J

AU Makhanlall, D

Wang, G

Huang, YJ

Liu, DF

Shen, J

AF Makhanlall, D.

Wang, G.

Huang, Y. J.

Liu, D. F.

Shen, J.

TI Joining of Ti-based bulk metallic glasses using resistance spot welding

technology

SO JOURNAL OF MATERIALS PROCESSING TECHNOLOGY

AB Ti40Zr25Ni3Cu12Be20 bulk metallic glass was used to study the influence of key process parameters on the spot welds microstructures and mechanical properties. Spot weld size and crystallized volume fraction were found to interact in a complex fashion in controlling the joint mechanical response. Assessment of cooling rates indicated that crystallized volume fraction was more liable to reach significant levels in the heat-affected zone than the fusion zone. Conditions for which the welds sustained a glassy environment were also determined. Resistance spot welding was found to be a feasible technique for joining bulk metallic glasses. (C) 2012 Elsevier B.V. All rights reserved.

RI Huang, Yongjiang/D-4809-2009

SN 0924-0136

PD AUG

PY 2012

VL 212

IS 8

BP 1790

EP 1795

DI 10.1016/j.jmatprotec.2012.03.020

UT WOS:000305106600019

ER

PT J

AU Yang, L

Miller, MK

Wang, XL

Liu, CT

Stoica, AD

Ma, D

Almer, J

Shi, D

AF Yang, Ling

Miller, Michael K.

Wang, Xun-Li

Liu, Chain T.

Stoica, Alexandru D.

Ma, Dong

Almer, Jonathan

Shi, Donglu

TI Nanoscale Solute Partitioning in Bulk Metallic Glasses

SO ADVANCED MATERIALS

AB Devitrification of bulk metallic glass leads to a novel microstructure, with high-density nanoscale crystalline precipitates evenly distributed in a glassy matrix. Significant chemical segregation is revealed at unprecedented detail by atom-probe tomography. This level of detail is crucial for understanding the interference peaks observed in small-angle X-ray and neutron scattering experiments, an unsolved mistery for over a decade.

RI Wang, Xun-Li/C-9636-2010; Stoica, Alexandru/K-3614-2013; Ma,

Dong/G-5198-2011

OI Wang, Xun-Li/0000-0003-4060-8777; Stoica, Alexandru/0000-0001-5118-0134;

Ma, Dong/0000-0003-3154-2454; Liu, Chain Tsuan/0000-0001-7888-9725

SN 0935-9648

EI 1521-4095

PD JAN 19

PY 2009

VL 21

IS 3

BP 305

EP 308

DI 10.1002/adma.200801183

UT WOS:000263081700004

ER

PT J

AU Shen, TD

Schwarz, RB

AF Shen, TD

Schwarz, RB

TI Bulk ferromagnetic glasses in the Fe-Ni-P-B system

SO ACTA MATERIALIA

AB The ferromagnetic metallic glass Fe40Ni40P14B6. available only as 30-50 mum thick ribbons, has been extensively studied over the last three decades. We used a flux-melting and water-quenching technique to prepare bulk glassy Fe40Ni40P14B6 alloys in the form of 2-mm diameter spheres and 1-mm diameter rods. The Curie temperature for the bulk glasses is higher than the average value of Curie temperatures reported for the rapidly quenched ribbons. The glass-transition temperature and the crystallization temperature of the bulk glasses are lower and higher, respectively. than the average values reported for rapidly quenched ribbons, making the supercooled-liquid region as wide as 42 K. The bulk glasses crystallize by a homogeneous nucleation followed by a growth at a constant rate. The nucleation rate in the bulk glasses is four orders of magnitude lower than in the rapidly quenched ribbons, suggesting that the previous thickness limitation was due to impurities in the melt (heterogeneous nucleation). Published by Elsevier Science Ltd. on behalf of Acta Materialia Inc.

SN 1359-6454

PD MAR 14

PY 2001

VL 49

IS 5

BP 837

EP 847

DI 10.1016/S1359-6454(00)00365-7

UT WOS:000167571900011

ER

PT S

AU Qin, FX

Xie, GQ

Zhu, SL

Dan, ZH

AF Qin, Fengxiang

Xie, Guoqiang

Zhu, Shengli

Dan, Zhenhua

BE Zhu, S

Ni, B

Ju, DY

TI Effect of Minor Addition Ta on the Thermal Stability and Corrosion

Resistance of Ti-Zr-Cu-Pd Bulk Metallic Glasses

SO ADVANCED MATERIALS SCIENCE AND TECHNOLOGY, (IFAMST-8)

SE Materials Science Forum

CT 8th International Forum on Advanced Materials Science and Technology

(IFAMST-8)

CY AUG 01-04, 2012

CL Fukuoka Inst Technol, Fukuoka City, JAPAN

SP Saitama Inst Technol, Chinese Mat Res Soc Japan, Chinese Acad & Profess Assoc Japan

HO Fukuoka Inst Technol

AB In this research, the effect of Ta addition on the formation, thermal stability and corrosion behavior of Ti-Zr-Cu-Pd bulk metallic glasses were investigated. The results revealed with minor addition of Ta, higher corrosion resistance and compressive strength as well as large plastic deformation were achieved. Minor addition Ta is effective for the formation of more protectively passive film during the process of anodic polarization. In addition, proper volume fraction nanoparticle with small size is responsible for the large plastic deformation of the as-cast Ti-based bulk metallic glasses with 1% Ta addition.

RI Xie, Guoqiang/A-8619-2011; Zhu, Shengli/D-5281-2009

OI Zhu, Shengli/0000-0002-0190-2626; Dan, Zhenhua/0000-0002-3026-685X

SN 0255-5476

PY 2013

VL 750

BP 23

EP 26

DI 10.4028/www.scientific.net/MSF.750.23

UT WOS:000319235900006

ER

PT J

AU Zhu, M

Li, JJ

Yao, LJ

Jian, ZY

Chang, F

Yang, GC

AF Zhu, Man

Li, Junjie

Yao, Lijuan

Jian, Zengyun

Chang, Fang'e

Yang, Gencang

TI Non-isothermal crystallization kinetics and fragility of

(Cu46Zr47Al7)(97)Ti-3 bulk metallic glass investigated by differential

scanning calorimetry

SO THERMOCHIMICA ACTA

AB In this paper, bulk metallic glasses with the composition of (Cu46Zr47Al7)(97)Ti-3 were prepared by copper mold casting technique. X-ray diffraction (XRD) and differential scanning calorimetry (DSC) were used to investigate its structure and non-isothermal crystallization kinetics. DSC traces revealed that it undergoes two-stage crystallization. The activation energies corresponding to the characteristic temperatures have been calculated, and the results reveal that the as-cast alloys have a good thermal stability in thermodynamics. Based on Kissinger equation, the activation energies for glass transition, the first and second crystallization processes were obtained as 485 +/- 16 kJ/mol, 331 +/- 7 kJ/mol and 210 +/- 3 kJ/mol, respectively, suggesting that the nucleation process is more difficult than the grain growth process. The fitting curves using Lasocka's empirical relation show that the influence of the heating rate for crystallization is larger than glass transition. Furthermore, the kinetic fragility for (Cu46Zr47Al7)(97)Ti-3 bulk metallic glasses is evaluated. Depending on the fragility index, (Cu46Zr47Al7)(97)Ti-3 bulk metallic glasses should be considered as "intermediate glasses". (c) 2013 Elsevier B.V. All rights reserved.

SN 0040-6031

PD AUG 10

PY 2013

VL 565

BP 132

EP 136

DI 10.1016/j.tca.2013.04.017

UT WOS:000322350300017

ER

PT J

AU Zhang, B

Bai, HY

Wang, RJ

Wu, Y

Wang, WH

AF Zhang, B.

Bai, H. Y.

Wang, R. J.

Wu, Y.

Wang, W. H.

TI Shear modulus as a dominant parameter in glass transitions: Ultrasonic

measurement of the temperature dependence of elastic properties of

glasses

SO PHYSICAL REVIEW B

AB Temperature dependence of elastic properties of a Ce-based metallic glass and a polymeric glass covering glass transition is in situ studied using an ultrasonic method. We find that the transverse acoustic velocity drops sharply consistently at the calorimetric glass transition temperature T-g, while the longitudinal velocity shows sudden decrease substantially above calorimetric T-g. Only the breakdown of the shear modulus G was detected, in contrast to the weak T dependence of bulk modulus around T-g. The results demonstrate that the G can precisely control the liquid behaviors of glass forming liquids and support the models of the shear interaction and/or energy dominated glass transition.

SN 2469-9950

EI 2469-9969

PD JUL

PY 2007

VL 76

IS 1

AR 012201

DI 10.1103/PhysRevB.76.012201

UT WOS:000248487900005

ER

PT J

AU Jiao, ZB

Li, HX

Gao, JE

Wu, Y

Lu, ZP

AF Jiao, Z. B.

Li, H. X.

Gao, J. E.

Wu, Y.

Lu, Z. P.

TI Effects of alloying elements on glass formation, mechanical and

soft-magnetic properties of Fe-based metallic glasses

SO INTERMETALLICS

AB Effects of alloying additions on glass formation, mechanical and soft-magnetic properties of Fe-(Si,P,C,B)based bulk metallic glasses (BMGs) were systemically studied in detail. It was found that the glass-forming ability (GFA) and the optimum doping content strongly depend on the electronegativity of the alloying elements, which are discussed in terms of liquid phase stability and crystallization resistance of the competing crystalline phases. These BMGs exhibit high fracture strength ranging from 2800 to 3800 MPa, which closely relates to the atomic size distribution in the alloys. Furthermore, appropriate additions of Co, Ga and Cu could improve not only the GFA but also the saturation magnetization due to different coupling mechanisms. (C) 2011 Elsevier Ltd. All rights reserved.

RI Wu, Yuan/C-4025-2015; Lu, Zhao-Ping/A-2718-2009

OI Wu, Yuan/0000-0001-7857-0247; Lu, Zhao-Ping/0000-0003-1463-8948; Jiao,

Z. B./0000-0002-0556-6869

SN 0966-9795

EI 1879-0216

PD OCT

PY 2011

VL 19

IS 10

BP 1502

EP 1508

DI 10.1016/j.intermet.2011.05.020

UT WOS:000294522400022

ER

PT J

AU Gu, XJ

Poon, SJ

Shiflet, GJ

Widom, M

AF Gu, X. J.

Poon, S. Joseph

Shiflet, Gary J.

Widom, Michael

TI Mechanical properties, glass transition temperature, and bond enthalpy

trends of high metalloid Fe-based bulk metallic glasses

SO APPLIED PHYSICS LETTERS

AB Mechanical properties and glass transition temperatures (T(g)) of Fe-Cr-Mo-P-C-B bulk metallic glasses containing up to 27 at. % metalloids have been studied. The shear modulus (G) is found to decrease with increasing metalloid content and a maximum plastic strain of similar to 3% is obtained, despite the increase in the number of strong metal-metalloid bonds. Also, T(g) increases with the decrease in G, in contrast to usual behavior. By employing first-principles calculations, the results are discussed in light of atomic bonding and connectivity in the amorphous network. The findings are relevant to understanding ductility and glass transition of metallic glasses. (c) 2008 American Institute of Physics.

RI Widom, Michael/P-2531-2014

OI Widom, Michael/0000-0001-5972-5696

SN 0003-6951

PD APR 21

PY 2008

VL 92

IS 16

AR 161910

DI 10.1063/1.2917577

UT WOS:000255456100024

ER

PT J

AU Stoica, M

Hajlaoui, K

Lemoulec, A

Yavari, AR

AF Stoica, M

Hajlaoui, K

Lemoulec, A

Yavari, AR

TI New ternary Fe-based bulk metallic glass with high boron content

SO PHILOSOPHICAL MAGAZINE LETTERS

AB To satisfy thermodynamic and kinetic requirements, Fe-based alloys capable of forming bulk metallic glasses often contain five or more elements. Usually, such compositions are of the type transition-metals/metalloids, with metalloid content around 20 atomic%. Starting from known Fe-based compositions used to make melt-spun glassy ribbons, purifying the master alloy by fluxing with B2O3 and using copper mould casting, a ternary Fe66Nb4B30 bulk metallic glass was obtained. To our knowledge this is the first Fe-based fully amorphous bulk metallic glass with just three atomic constituents. The alloy is ferromagnetic with Curie temperature T-c=646 K, glass transition temperature T-g=845 K, crystallization temperature T-x=876 K, liquidus temperature T-liq=1451 K and having a mechanical strength of 4 GPa.

RI Stoica, Mihai/B-7069-2015; yavari, alain/E-8192-2010

SN 0950-0839

PD APR

PY 2006

VL 86

IS 4

BP 267

EP 275

DI 10.1080/09500830600696344

UT WOS:000237398100008

ER

PT J

AU Kawamura, Y

Kagao, S

Ohno, Y

AF Kawamura, Y

Kagao, S

Ohno, Y

TI Electron beam welding of Zr-based bulk metallic glass to crystalline Zr

metal

SO MATERIALS TRANSACTIONS

AB We have tried an electron-beam welding of a Zr-based bulk metallic glass plate to a polycrystalline Zr metal one for a Zr41Ti14Cu12Ni10Be23 (at%) bulk metallic glass having an excellent glass forming ability, We have succeeded in butt-welding the bulk metallic glass and pure Zr metal plates with a thickness of 3 mm. Invisible defect or clack was detected around the bead and heat-affected zone, The bulk metallic glass around the interface kept the amorphous state. Formation of any crystalline phases except for hcp-Zr was not detected in the interface, The interface of the successfully welded sample exhibited enough toughness and joining strength to endure the bending through 90 degrees by hammering.

SN 1345-9678

EI 1347-5320

PD DEC

PY 2001

VL 42

IS 12

BP 2649

EP 2651

DI 10.2320/matertrans.42.2649

UT WOS:000173669800030

ER

PT S

AU Zumkley, T

Naundorf, V

Macht, MP

Frohberg, G

AF Zumkley, T

Naundorf, V

Macht, MP

Frohberg, G

BE Ma, E

Atzmon, M

Koch, CC

TI Single activation enthalpy of diffusion in a ZrTiCuNiBe bulk glass

around the glass transition after structural relaxation

SO METASTABLE, MECHANICALLY ALLOYED AND NANOCRYSTALLINE MATERIALS

SE MATERIALS SCIENCE FORUM

CT International Symposium on Metastable, Mechanically Alloyed and

Nanocrystalline Materials

CY JUN 24-29, 2001

CL UNIV MICHIGAN, ANN ARBOR, MICHIGAN

HO UNIV MICHIGAN

AB B and Fe diffusion coefficients measured at and below 553 K in the Zr46.8Ti8.2Cu7.5Ni10Be27.5 bulk glass after long time relaxation at 553 K are significantly lower than before relaxation. They agree with diffusion coefficients obtained from the extrapolated high temperature (> 600 K) Arrhenius behavior of diffusion indicating that after proper relaxation the diffusion follows a uniform temperature dependence over the entire temperature range around the glass transition. Measurements of the enthalpy recovery of this bulk glass around the calorimetric glass transition indicate reversible structural changes with a relaxation time of about 106 s at 553 K.

SN 0255-5476

BN 0-87849-892-3

PY 2002

VL 386-3

BP 65

EP 70

DI 10.4028/www.scientific.net/MSF.386-388.65

UT WOS:000174657900010

ER

PT J

AU Aditya, AV

Arora, HS

Mukherjee, S

AF Aditya, Ayyagari V.

Arora, Harpreet Singh

Mukherjee, Sundeep

TI Corrosion behavior of ZrTiCuNiBe bulk metallic glass subjected to

friction stir processing

SO JOURNAL OF NON-CRYSTALLINE SOLIDS

AB The corrosion behavior of a Zirconium-based bulk metallic glass subjected to friction stir processing was evaluated. Processing was done at the tool rotational speeds of 300 rpm, 500 rpm and 900 rpm. Potentiodynamic polarization and electrochemical impedance spectroscopy studies were carried out in 0.1 M NaCl solution. A strong correlation was found between the corrosion rate and enthalpy of structural relaxation, which is a measure of free volume. Electrochemical impedance spectroscopy measurements showed lower polarization resistance for processed samples as compared to as-cast metallic glass which is consistent with the DC polarization results. Shear bands in processed specimens were found to be preferred nucleation sites for the formation of corrosion pits. In contrast, pitting was more stochastic for the as-cast metallic glass. (C) 2015 Elsevier B.V. All rights reserved.

OI Ayyagari, Venkata Aditya/0000-0002-3111-9819

SN 0022-3093

EI 1873-4812

PD OCT 1

PY 2015

VL 425

BP 124

EP 129

DI 10.1016/j.jnoncrysol.2015.05.038

UT WOS:000359958700017

ER

PT J

AU [Anonymous]

AF [Anonymous]

TI Formation and evolution of spherocrystals in a NiTiZrAlCuSi amorphous

alloy

SO INTERMETALLICS

CT 6th International Conference on Bulk-Metallic Glasses (BMG-VI)

CY MAY 11-15, 2008

CL Xian, PEOPLES R CHINA

AB The microstructure of a NiTiZrAlCuSi bulk metallic glass was investigated by transmission electron microscopy (TEM) attached with energy dispersive spectroscopy (EDS) and X-ray diffraction (XRD). TEM results showed that some spherocrystals were embedded in the amorphous matrix, but XRD patterns from the sample did not reveal distinct diffraction peaks from the minor amount of spherocrystals. EDS analyses indicated that the composition of these spherocrystals was similar to that of the metallic glasses matrix. To further study the evolution of these spherocrystals, isothermal crystallization experiment was carried out using differential scanning calorimetry (DSC). DSC curves and XRD patterns reveal the microstructure difference of the sample at different heating times. TEM images record the evolution processes of the metastable spherocrystal and amorphous matrix. (C) 2008 Elsevier Ltd. All rights reserved.

SN 0966-9795

PD APR

PY 2009

VL 17

IS 4

BP 262

EP 265

DI 10.1016/j.intermet.2008.07.014

UT WOS:000264728400018

ER

PT J

AU Li, Y

Poon, SJ

Shiflet, GJ

Xu, J

Kim, DH

Loffler, JF

AF Li, Y.

Poon, S. J.

Shiflet, G. J.

Xu, J.

Kim, D. H.

Loeffler, J. F.

TI Formation of bulk metallic glasses and their composites

SO MRS BULLETIN

AB A great expansion in the number of alloy compositions known to give bulk metallic glasses (BMGs) has occurred in recent years. This progress is reviewed, and factors contributing to glass-forming ability are discussed. Practical strategies for pinpointing compositions with optimum glass-forming ability are presented, with examples of their use. Consideration is also given to the wide range of possibilities for BMG-based composites.

RI Kim, Do Hyang/J-6575-2012

SN 0883-7694

PD AUG

PY 2007

VL 32

IS 8

BP 624

EP 628

DI 10.1557/mrs2007.123

UT WOS:000248827300013

ER

PT J

AU Ding, HY

Shao, Y

Gong, P

Li, JF

Yao, KF

AF Ding, H. Y.

Shao, Y.

Gong, P.

Li, J. F.

Yao, K. F.

TI A senary TiZrHfCuNiBe high entropy bulk metallic glass with large

glass-forming ability

SO MATERIALS LETTERS

AB Most reported bulk metallic glasses (BMGs) contain multiple constituent elements but only one of them is the principal element. Here we report that a senary Ti16.7Zr16.7Hf16.7Cu16.7Ni16.7Be16.7 high entropy bulk metallic glass (HE-BMG) with a critical diameter larger than 15 mm has been successfully prepared by copper mold casting. It shows that newly developed HE-BMG possesses large glass-forming ability, together with the supercooled liquid region Delta T (=T-x-T-g), reduced glass transition temperature T-rg (=T-g/T-1) and gamma parameter (=T-x/(T-g-T-1)) of 70 K, 0.619 and 0.422, respectively. The present result indicates that high entropy alloys, even for senary alloys or metal-metal type, could also possess large glass-forming ability. And the present HE-BMG could serve as a model material for studying the fundamental issues both of high entropy alloys and bulk metallic glasses. (C) 2014 Elsevier B.V. All rights reserved.

RI Gong, Pan/K-8870-2012; Shao, Yang/H-2722-2013

OI Gong, Pan/0000-0002-3833-8440; Shao, Yang/0000-0001-5369-9933

SN 0167-577X

EI 1873-4979

PD JUN 15

PY 2014

VL 125

BP 151

EP 153

DI 10.1016/j.matlet.2014.03.185

UT WOS:000336696400040

ER

PT J

AU Jiang, W

Wu, JL

Zhang, B

AF Jiang, Wei

Wu, Jili

Zhang, Bo

TI Linking electronic structure to beta relaxation of La-based bulk

metallic glasses

SO INTERMETALLICS

AB This work investigates the correlation of electronic structure and beta relaxation of La(70)Ni(15)A1(15), La(70)Co(15)A1(15) and La(70)Cu(15)A1(15) bulk metallic glasses. X-ray photoelectron spectroscopy measurements discovered that the activity of electrons around Fermi level of La(70)Ni(15)A1(15) and La(70)Co(15)A1(15) glasses with peak-like strong beta relaxation are more intense than La(70)Cu(15)A1(15) glass with shoulder-like weak beta relaxation and correspondingly the core levels of electronic states of Ni and Co elements are seriously split whereas that of Cu is not. This work might provide a different route to understand the origin of beta relaxation rather than the normal thinking of atomic packing structure.

SN 0966-9795

EI 1879-0216

PD JUL

PY 2018

VL 98

BP 126

EP 130

DI 10.1016/j.intermet.2018.04.020

UT WOS:000435053700015

ER

PT J

AU Jiang, W

Wu, JL

Zhang, B

AF Jiang, Wei

Wu, Jili

Zhang, Bo

TI Size effect on beta relaxation in a La-based bulk metallic glass

SO PHYSICA B-CONDENSED MATTER

AB This work studied the effect of the size of specimens on the beta relaxation. Taking La70Ni15Al15 bulk metallic glass as a model material, via dynamic mechanical analysis, we found that the thickness of specimens can affect the intensity of beta relaxation. Specifically, increasing the thickness of specimens can enhance intensity of beta relaxation. For this enhancement, we proposed that the involvedly total free volume facilitates the beta relaxed process. This finding gives a new insight on the structural relaxation of bulk metallic glasses, especially for understanding of origin of beta relaxation.

SN 0921-4526

EI 1873-2135

PD MAR 15

PY 2017

VL 509

BP 46

EP 49

DI 10.1016/j.physb.2016.12.035

UT WOS:000398055300008

ER

PT B

AU Zhou, X

Kou, HC

Wang, J

Li, JS

Blandin, JJ

Zhou, L

AF Zhou, X.

Kou, H. C.

Wang, J.

Li, J. S.

Blandin, J. J.

Zhou, L.

BE Zhou, L

Chang, H

Lu, Y

Xu, D

TI Homogeneous Flow and Superplastic Forming Process of Ti-Zr-Cu-Pd-Sn Bulk

Metallic Glass in Supercooled Liquid Region

SO TI-2011: PROCEEDINGS OF THE 12TH WORLD CONFERENCE ON TITANIUM, VOL III

CT 12th World Conference on Titanium (Ti-2011)

CY JUN 19-24, 2011

CL Nonferrous Met Soc China, Beijing, PEOPLES R CHINA

SP NW Inst Nonferrous Met Res, NW Polytechn Univ, Baoti Grp Ltd, Beijing Inst Aeronaut Mat, Chinese Acad Sci, Inst Met Res

HO Nonferrous Met Soc China

AB The Ti-Zr-Cu-Pd-Sn bulk metallic glasses have some advantages of high yield strength about 2000Mpa, good corrosion resistance and absence of toxic element, such as Al,Ni and Be. So the Ti-based metallic glass is regarded as a promising biomaterial and lightweight material. The homogenous deformation and superplastic forming process of Ti-Zr-Cu-Pd-Sn-Sn bulk metallic glass was investigated. The homogeneous deformation behavior is strongly dependent on the strain rate. The metallic glass exhibits Newtonian behavior at relative low strain rate and non-Newtonian behavior at relative high strain rate. The micro-gear was successfully extruded by superplastic forming process, indicating that the Ti-Zr-Cu-Pd-Sn bulk metallic glass processes good workability in supercooled liquid region. The deformed metallic glass was investigated by X-ray diffraction (XRD), demonstrating that no detectable crystalline phase precipitated during superplastic forming.

OI WANG, Jun/0000-0001-8101-2967; Li, Jinshan/0000-0002-6894-9760

BN 978-7-03-033894-5

PY 2012

BP 1808

EP 1811

UT WOS:000398128800026

ER

PT J

AU Pilarczyk, W

AF Pilarczyk, Wirginia

TI The investigation of the structure of bulk metallic glasses before and

after laser welding

SO CRYSTAL RESEARCH AND TECHNOLOGY

CT Symposium N on European-Materials-Research-Society (EMRS)

CY SEP 15-19, 2014

CL Warsaw, POLAND

SP European Mat Res Soc

AB Fe-based bulk metallic glasses present an interesting combination of physical, chemical and mechanical properties. During the last decade, intensive progress has been made and a number of applications have been suggested for these BMGs alloys. In order to successfully and universally use these materials, it is necessary to increase the small dimension of the elements. The main aim of this work is the examination of the structure of bulk metallic glasses and their laser welds. The studies were performed on Fe-Co-B-Si-Nb system alloy in form of plate. The samples were successfully produced by die pressure casting method and welded by the use of the TruLaser Station 5004. In this work microscopic observation and X-ray diffraction analysis were carried out. The tests results show that the initial amorphous and amorphous-crystalline structures of Fe-based bulk metallic glasses have an influence on the crystallization process in the laser welds.

SN 0232-1300

EI 1521-4079

PD SEP

PY 2015

VL 50

IS 9-10

BP 700

EP 704

DI 10.1002/crat.201400438

UT WOS:000360814000004

ER

PT J

AU Deledda, S

Eckert, J

Schultz, L

AF Deledda, S

Eckert, J

Schultz, L

TI Thermal stability of mechanically alloyed Zr-Cu-Al-Ni glass composites

containing ZrC particles as a second phase

SO SCRIPTA MATERIALIA

AB Mechanically alloyed Zr55Cu30Al10Ni5 metallic glass matrix composites powders containing up to 30 vol% of ZrC particles were investigated by differential scanning calorimetry. It is shown that the supercooled liquid behaviour is not affected by the presence of the particles, but changes in the thermal stability suggest deviations in the overall composition of the glassy matrix. (C) 2002 Acta Materialia Inc. Published by Elsevier Science Ltd. All rights reserved.

RI Schultz, Ludwig/B-3383-2010

SN 1359-6462

PD JAN 4

PY 2002

VL 46

IS 1

BP 31

EP 35

AR PII S1359-6462(01)01191-5

DI 10.1016/S1359-6462(01)01191-5

UT WOS:000175333000006

ER

PT J

AU Senkov, ON

Miracle, DB

Scott, JM

AF Senkov, ON

Miracle, DB

Scott, JM

TI Development and characterization of Ca-Mg-Zn-Cu bulk metallic glasses

SO INTERMETALLICS

CT 4th International Conference on Bulk Metallic Glasses

CY MAY 01-05, 2005

CL Gathinburg, TN

AB A number of ternary Ca-Mg-Zn and Ca-Mg-Cu and quaternary Ca-Mg-Zn-Cu bulk metallic glasses were produced using recently developed specific criteria. Their glass forming ability was correlated to the alloy chemistry, melting temperature, and driving force for crystallization of super-cooled liquid. A structural assessment using the efficient cluster packing model was also applied and showed a good ability to represent these glasses. Glass transition temperature, crystallization temperature and heat of crystallization were also determined for the produced alloys. Compression tests were conducted on a quaternary alloy at room temperature and in the temperature range of super-cooled liquid. (c) 2006 Elsevier Ltd. All rights reserved.

RI Senkov, Oleg/C-7197-2012

OI Senkov, Oleg/0000-0001-5587-415X; Senkov, Oleg/0000-0002-9336-3702

SN 0966-9795

PD AUG-SEP

PY 2006

VL 14

IS 8-9

SI SI

BP 1055

EP 1060

DI 10.1016/j.intermet.2006.01.024

UT WOS:000237770600037

ER

PT J

AU Zhang, W

Guo, H

Chen, MW

Saotome, Y

Qin, CL

Inoue, A

AF Zhang, W.

Guo, H.

Chen, M. W.

Saotome, Y.

Qin, C. L.

Inoue, A.

TI New Au-based bulk glassy alloys with ultralow glass transition

temperature

SO SCRIPTA MATERIALIA

AB New Au-based glassy alloys with high Au contents of similar to 60-70 at.%. have been found to exhibit an ultralow glass transition temperature (similar to 66-86 degrees C), a high glass-forming ability and a wide supercooled liquid region. They also have high thermodynamic and kinetic stability in the supercooled liquid state, strong oxidation resistance, good mechanical properties, very high corrosion resistance and excellent thermoplastic formability. (C) 2009 Acta Materialia Inc. Published by Elsevier Ltd. All rights reserved.

RI CHEN, Mingwei/A-4855-2010; Inoue, Akihisa/E-5271-2015; Saotome,

Yasunori/B-3267-2010; Qin, Chunling/A-4846-2010

OI CHEN, Mingwei/0000-0002-8274-3099; Saotome,

Yasunori/0000-0002-6110-5573; Chen, Mingwei/0000-0002-2850-8872

SN 1359-6462

PD OCT

PY 2009

VL 61

IS 7

BP 744

EP 747

DI 10.1016/j.scriptamat.2009.06.020

UT WOS:000269093600020

ER

PT J

AU Ji, XL

Pan, Y

Ni, FS

AF Ji, Xiulin

Pan, Ye

Ni, Fusheng

TI A thermodynamic criterion for predicting glass-forming ability in binary

metallic glasses

SO MATERIALS & DESIGN

AB A new thermodynamic model for estimation of glass forming ability (GFA) is proposed. Considering both the stability of liquid and crystal and the competition of glass and crystal, an expression of GFA has been obtained. The equation is very useful for identifying alloy compositions with good GFA of binary alloys. Finally, this thermodynamic model of GFA is not only verified in five different binary bulk metallic glasses but also showed wider application range comparing with the former model. (C) 2008 Elsevier Ltd. All rights reserved.

RI JI, XIULIN/D-2882-2015

OI JI, XIULIN/0000-0002-1752-6602

SN 0261-3069

PD MAR

PY 2009

VL 30

IS 3

BP 842

EP 845

DI 10.1016/j.matdes.2008.05.075

UT WOS:000262869100061

ER

PT J

AU Ma, J

Yi, J

Zhao, DQ

Pan, MX

Wang, WH

AF Ma, J.

Yi, J.

Zhao, D. Q.

Pan, M. X.

Wang, W. H.

TI Large size metallic glass gratings by embossing

SO JOURNAL OF APPLIED PHYSICS

AB Bulk metallic glasses have excellent thermoforming ability in their wide supercooled liquid region. We show that large-size metallic glass grating (similar to 8 x 8 mm(2)) with fine periodicity and ultra smooth surface feature can be readily fabricated by hot embossing. The method for fabrication of gratings is proved to be much cheaper, and requires low pressure and short time (less than 30 s). The metallic glass gratings exhibit comparable optical properties such as rainbow-like spectrum when shone by fluorescent lamp light. (C) 2012 American Institute of Physics. [http://dx.doi.org/10.1063/1.4752399]

SN 0021-8979

EI 1089-7550

PD SEP 15

PY 2012

VL 112

IS 6

AR 064505

DI 10.1063/1.4752399

UT WOS:000309423200150

ER

PT J

AU Zhang, M

Dai, LH

Liu, Y

Liu, L

AF Zhang, M.

Dai, L. H.

Liu, Y.

Liu, L.

TI Heterogeneous dynamics of metallic glasses

SO SCRIPTA MATERIALIA

AB Heterogeneous dynamics in the flow of supercooled metallic liquids are revealed as the oscillated mechanical response in compression and evidenced to be consistent with the range of medium length scales over which structural rearrangements occur detected by small angle X-ray scattering (SAXS) in heating from room temperature to the supercooled liquid region. This range of medium length scales is suggested to be the structural origin of the heterogeneous dynamics of metallic glasses. (C) 2015 Acta Materialia Inc. Published by Elsevier Ltd. All rights reserved.

OI Dai, LanHong/0000-0001-8991-0358

SN 1359-6462

PD OCT

PY 2015

VL 107

BP 111

EP 114

DI 10.1016/j.scriptamat.2015.05.034

UT WOS:000358806400028

ER

PT J

AU Zhang, JH

Shen, BL

Zhang, ZD

AF Zhang, Jianhua

Shen, Baolong

Zhang, Zhidong

TI Crystallization behaviors of FeSiBPMo bulk metallic glasses

SO JOURNAL OF NON-CRYSTALLINE SOLIDS

AB The effect of Mo addition on the crystallization behaviors of FeSiBPMo glassy alloys was investigated and the phase transformation behaviors were summarized. Three primary phases identified as Fe23B6, (Fe, Mo)(23)B-6 and alpha-Mn type phases were found by using X-ray diffraction transmission electron microscopy. The relationship between the glass-forming ability and the precipitated primary phase was discussed and the results demonstrate that the tendency of both the atom structured complexity and the difficulty of geometrical rearrangement for the precipitation of the primary phase coincides with the changing trend of the glass-forming ability for the FeSiBPMo bulk metallic glasses. (c) 2012 Elsevier B.V. All rights reserved.

SN 0022-3093

EI 1873-4812

PD JAN 15

PY 2013

VL 360

BP 31

EP 35

DI 10.1016/j.jnoncrysol.2012.10.010

UT WOS:000313610700007

ER

PT J

AU He, L

Zhang, SA

Sun, J

Zhang, CJ

AF He, Lin

Zhang, Shuai

Sun, Jun

Zhang, Chang-jun

TI Effect of oxygen impurity on long-term thermal stability of Zr-based

metallic glasses below glass transition temperature

SO TRANSACTIONS OF NONFERROUS METALS SOCIETY OF CHINA

AB Long-term thermal stability of a series of Zr-based metallic glasses with different oxygen contents below their glass transition temperatures was compared based on their deductive continuous-heating-transformation diagrams created by using the corollary of Kissinger analysis method. It is found that the influence of oxygen on the long-term thermal stability of Zr-based metallic glasses exhibits at lower temperature is different from that on their short-term thermal stability presented at higher temperature. For each kind of the Zr-based metallic glasses, there is a critical heating rate, phi(c), which corresponds to a critical temperature, T-c. As heating rate is smaller than phi(c) and onset devitrification temperature is below T-c, the glass with higher oxygen content will have longer incubation period for onset devitrification. The values of phi(c) and T-c are related with the glasses' reduced glass transition temperature T-rg. The improving effect of oxygen impurity on the long-term thermal stability of Zr-based metallic glasses was discovered.

SN 1003-6326

PD OCT

PY 2006

VL 16

IS 5

BP 992

EP 997

DI 10.1016/S1003-6326(06)60366-X

UT WOS:000241982300002

ER

PT J

AU Wang, ZX

Lu, JB

Xi, YJ

AF Wang Zhixin

Lu Jinbin

Xi Yanjun

TI Effect of aluminium addition on glass forming ability of

Nd55-xAl10+xFe15Co20 (x=0, 5, 10) alloys

SO JOURNAL OF RARE EARTHS

AB Nd55-xAl10+xCo20Fe15(x=0, 5, 10) bulk glass-forming alloys with distinct glass transition in differential scanning calorimetry (DSC) traces were obtained by suction casting. The glass forming ability (GFA) of the alloys was investigated. It was found that the reduced glass transition temperature (T-rg) and the parameter gamma of the alloys increased with the increasing concentration of Al. The glass formation enthalpy of the alloys was calculated based on Miedema's model, and it was suggested that the GFA of the alloys could be enhanced by the decrease of the glass formation enthalpy with Al additions.

SN 1002-0721

PD OCT

PY 2007

VL 25

IS 5

BP 619

EP 623

UT WOS:000250691900020

ER

PT J

AU Suryanarayana, C

Seki, I

Inoue, A

AF Suryanarayana, C.

Seki, I.

Inoue, A.

TI A critical analysis of the glass-forming ability of alloys

SO JOURNAL OF NON-CRYSTALLINE SOLIDS

AB Several empirical rules have been proposed during the past few years to synthesize bulk metallic glasses. But, the real reasons for the improved glass-forming ability of these alloys are still not clear and the ability to design alloy compositions to enable synthesis of larger diameter rods has not improved. The present work conducts a critical analysis of the existing data in terms of the different glass-forming criteria and concludes that the available parameters cannot satisfactorily predict the GFA and explain all the observed data. Reasons for this failure have been suggested. (C) 2009 Elsevier B.V. All rights reserved.

RI 関, 一郎/C-4211-2015; Inoue, Akihisa/E-5271-2015

SN 0022-3093

PD MAR 1

PY 2009

VL 355

IS 6

BP 355

EP 360

DI 10.1016/j.jnoncrysol.2008.12.009

UT WOS:000264595400004

ER

PT J

AU Chen, SS

Tu, JX

Wu, JJ

Hu, Q

Xie, SH

Zou, JZ

Zeng, XR

AF Chen, Shuangshuang

Tu, Jianxin

Wu, Jiajia

Hu, Qiang

Xie, Shenghui

Zou, Jizhao

Zeng, Xierong

TI Phase separation and significant plastic strain in a Zr-Cu-Ni-Al-Fe bulk

metallic glass

SO MATERIALS SCIENCE AND ENGINEERING A-STRUCTURAL MATERIALS PROPERTIES

MICROSTRUCTURE AND PROCESSING

AB In this study, we reported a phase -separated Zr62Cu18Ni10.4Al8Fe1.6 bulk metallic glass exhibiting a significant plastic strain at room-temperature. The engineering plastic strain, fracture strength and Young's modulus were determined to be about 32.6%, 2919 MPa and 90 GPa respectively. The enhanced ductility was explained from the view point of elastic energy dissipation, separation length and shear modulus difference between separated phases. This result provides a promising approach to improve the mechanical properties of bulk metallic glasses through compositional and structural design by micro alloying. (C) 2016 Elsevier B.V. All rights reserved.

SN 0921-5093

EI 1873-4936

PD FEB 22

PY 2016

VL 656

BP 84

EP 89

DI 10.1016/j.msea.2016.01.032

UT WOS:000373316300011

ER

PT J

AU Aydiner, CC

Ustundag, E

Hanan, JC

AF Aydiner, CC

Ustundag, E

Hanan, JC

TI Thermal-tempering analysis of bulk metallic glass plates using an

instant-freezing model

SO METALLURGICAL AND MATERIALS TRANSACTIONS A-PHYSICAL METALLURGY AND

MATERIALS SCIENCE

AB The viscoelastic nature of bulk metallic glasses (BMGs), their low thermal conductivity, and the fast cooling used in their processing subject them to thermal tempering. This process leads to a residual stress state in which compression on the surface is balanced by tension in the interior. For the first time, we have calculated such stresses in metallic glasses by adapting an analytical instant-freezing model previously developed for silicate glasses. This model has been demonstrated to be reasonably accurate in predicting the final residual stresses, although, due to its very nature, it neglects transient effects. For an infinite plate geometry and employing processing parameters often used for metallic glasses, we predict that significant residual Stresses can be generated in these materials during thermal tempering. Preliminary measurements conducted using the layer-removal method yield compressive residual stress values close to model predictions.

RI Ustundag, Ersan/C-1258-2009; Aydiner, Cahit/O-9618-2017

OI Ustundag, Ersan/0000-0002-0812-7028; Aydiner, Cahit/0000-0001-8256-6742

SN 1073-5623

PD NOV

PY 2001

VL 32

IS 11

BP 2709

EP 2715

DI 10.1007/s11661-001-1023-8

UT WOS:000172110200004

ER

PT S

AU Medeiros, SC

Ghosh, AK

AF Medeiros, SC

Ghosh, AK

BE Ma, E

Atzmon, M

Koch, CC

TI Microstructural and deformation characteristics of a laminated

amorphous/nanocrystalline alloy, Zr67Nb6Cu14Ni10Al3

SO METASTABLE, MECHANICALLY ALLOYED AND NANOCRYSTALLINE MATERIALS

SE MATERIALS SCIENCE FORUM

CT International Symposium on Metastable, Mechanically Alloyed and

Nanocrystalline Materials

CY JUN 24-29, 2001

CL UNIV MICHIGAN, ANN ARBOR, MICHIGAN

HO UNIV MICHIGAN

AB Considerable interest in the synthesis and properties of bulk metallic glasses has been noted in recent times. In this work, the mechanical properties of a pressure die cast alloy Zr67Ni10Nb6Cu14Al3 in the (amorphous) glassy state with a core that is in partly crystalline state have been investigated under controlled deformation conditions. Microscopy, x-ray analysis and differential scanning calorimetry were performed to characterize the die-cast material in the as-received condition. The mechanical properties of die-cast alloy Zr67Ni10Nb6Cu14Al3 were determined by tension and compression testing.

SN 0255-5476

BN 0-87849-892-3

PY 2002

VL 386-3

BP 547

EP 552

UT WOS:000174657900082

ER

PT J

AU Nollmann, N

Binkowski, I

Schmidt, V

Rosner, H

Wilde, G

AF Nollmann, Niklas

Binkowski, Isabelle

Schmidt, Vitalij

Roesner, Harald

Wilde, Gerhard

TI Impact of micro-alloying on the plasticity of Pd-based bulk metallic

glasses

SO SCRIPTA MATERIALIA

AB Micro-alloying was performed using additions of Co and Fe to monolithic Pd40Ni40P20 bulk metallic glass. Compression tests showed a plastic strain of 13% for the Co addition (1 at.%), whereas the Fe addition (0.6 at.%) led to immediate failure after reaching the elastic limit. Plasticity is not reflected by the high Poisson's ratio of 0.4 since it remained unaffected by minor alloying. Applying the fictive temperature concept to analyze the impact of minor alloying suggests that the total amount of free volume frozen-in during vitrification is less important for the mechanical properties of bulk metallic glasses than its local distribution. (c) 2015 Acta Materialia Inc. Published by Elsevier Ltd. All rights reserved.

OI Wilde, Gerhard/0000-0001-8001-5998

SN 1359-6462

PD JAN 15

PY 2016

VL 111

BP 119

EP 122

DI 10.1016/j.scriptamat.2015.08.030

UT WOS:000364253400026

ER

PT J

AU Maass, R

Klaumunzer, D

Villard, G

Derlet, PM

Loffler, JF

AF Maass, R.

Klaumuenzer, D.

Villard, G.

Derlet, P. M.

Loeffler, J. F.

TI Shear-band arrest and stress overshoots during inhomogeneous flow in a

metallic glass

SO APPLIED PHYSICS LETTERS

AB At the transition from a static to a dynamic deformation regime of a shear band in bulk metallic glasses, stress transients in terms of overshoots are observed. We interpret this phenomenon with a repeated shear-melting transition and are able to access a characteristic time for a liquidlike to solidlike transition in the shear band as a function of temperature, enabling us to understand why shear bands arrest during inhomogenous serrated flow in bulk metallic glasses. (C) 2012 American Institute of Physics. [doi:10.1063/1.3684871]

RI Maass, Robert/F-6306-2011

SN 0003-6951

PD FEB 13

PY 2012

VL 100

IS 7

AR 071904

DI 10.1063/1.3684871

UT WOS:000300436800021

ER

PT J

AU Wang, AP

Wang, JQ

Ma, E

AF Wang, A. P.

Wang, J. Q.

Ma, E.

TI Modified efficient cluster packing model for calculating alloy

compositions with high glass forming ability

SO APPLIED PHYSICS LETTERS

AB The efficient cluster packing model for bulk metallic glasses (BMGs) has been modified and extended to allow quantitative predictions of alloy compositions with high glass forming ability. The compositions calculated using this simple method compare favorably with the vast majority of the BMGs discovered in recent years. In addition, a Hf-based BMG has been obtained at the predicted composition. (c) 2007 American Institute of Physics.

RI Ma, En/A-3232-2010

SN 0003-6951

EI 1077-3118

PD MAR 19

PY 2007

VL 90

IS 12

AR 121912

DI 10.1063/1.2715172

UT WOS:000245135800050

ER

PT J

AU Zhang, QS

Zhang, W

Xie, GQ

Inoue, A

AF Zhang, Qing-sheng

Zhang, Wei

Xie, Guo-qiang

Inoue, Akihisa

TI Synthesis, structure and mechanical properties of Zr-Cu-based bulk

metallic glass composites

SO INTERNATIONAL JOURNAL OF MINERALS METALLURGY AND MATERIALS

AB The unusual glass-forming ability (GFA) of the Zr48Cu36Ag8Al8 alloy and the high ductility of the Zr48Cu36Ag8Al8 metallic glass-matrix composites containing Ta powder were reported. The bulk metallic glass rod with a diameter of 25 mm was successfully synthesized using copper mold casting for the Zr48Cu36Ag8Al8 alloy. High GFA of this alloy was found to be related to a large supercooled liquid region and a quaternary eutectic point with low melting temperature. The bulk metallic glass matrix composites were prepared by introducing extra Ta particles into the Zr48Cu36Ag8Al8 melt. The composites consist of Ta particles homogenously distributed in the Zr48Cu36Al8Ag8 metallic glass matrix. The optimum content of Ta powder is 10at% for the composite with the highest plasticity, which shows a plastic strain of 31%.

RI zhang, qingsheng/A-4851-2010; Inoue, Akihisa/E-5271-2015; Xie,

Guoqiang/A-8619-2011

SN 1674-4799

EI 1869-103X

PY 2010

VL 17

IS 2

BP 208

EP 213

DI 10.1007/s12613-010-0215-x

UT WOS:000276343200015

ER

PT J

AU Xi, XK

Zhao, DQ

Pan, MX

Wang, WH

Wu, Y

Lewandowski, JJ

AF Xi, XK

Zhao, DQ

Pan, MX

Wang, WH

Wu, Y

Lewandowski, JJ

TI Fracture of brittle metallic glasses: Brittleness or plasticity

SO PHYSICAL REVIEW LETTERS

AB We report a brittle Mg-based bulk metallic glass which approaches the ideal brittle behavior. However, a dimple structure is observed at the fracture surface by high resolution scanning electron microscopy, indicating some type of "ductile" fracture mechanism in this very brittle glass. We also show, from the available data, a clear correlation between the fracture toughness and plastic process zone size for various glasses. The results indicate that the fracture in brittle metallic glassy materials might also proceed through the local softening mechanism but at different length scales.

RI Lewandowski, John/S-3815-2017

OI Lewandowski, John/0000-0002-3389-2637

SN 0031-9007

EI 1079-7114

PD APR 1

PY 2005

VL 94

IS 12

AR 125510

DI 10.1103/PhysRevLett.94.125510

UT WOS:000228065600041

PM 15903937

ER

PT J

AU Cai, AH

Chen, H

Li, XS

Wang, H

Zhou, Y

An, WK

AF Cai, An-hui

Chen, Hua

Li, Xiao-song

Wang, Hui

Zhou, Yong

An, Wei-ke

TI An expression for the calculation of Gibbs free energy difference of

multi-component bulk metallic glasses

SO JOURNAL OF ALLOYS AND COMPOUNDS

AB We propose an expression, Delta G = Delta H-m[{alpha(T-T-m)/(1-alpha)T-m}+{T/(1-alpha)T-m}ln(T-m/T)] (O < a < 1), for the calculation of Gibbs free energy difference Delta G of multi-component metallic alloys. The results show that the theoretical Delta Gs are in better agreement with the experimental values over entire under-cooling range than those given by the TS, KN1 and KN2 expressions. The glass-forming ability of the multi-component metallic alloys has been observed to increase with the increase in the difference of the glass transition temperature and isenthalpic temperature. (c) 2006 Elsevier B.V. All rights reserved.

RI Wang, Hui/C-6768-2009; Wang, Hui/A-5775-2017

OI Wang, Hui/0000-0002-4915-3396

SN 0925-8388

PD MAR 14

PY 2007

VL 430

IS 1-2

BP 232

EP 236

DI 10.1016/j.jallcom.2006.04.072

UT WOS:000244826200041

ER

PT J

AU Lu, YM

Zeng, JF

Huang, JC

Kuan, SY

Nieh, TG

Wang, WH

Pan, MX

Liu, CT

Yang, Y

AF Lu, Y. M.

Zeng, J. F.

Huang, J. C.

Kuan, S. Y.

Nieh, T. G.

Wang, W. H.

Pan, M. X.

Liu, C. T.

Yang, Y.

TI In-situ atomic force microscopy observation revealing gel-like

plasticity on a metallic glass surface

SO JOURNAL OF APPLIED PHYSICS

AB It has been decade-long and enduring efforts to decipher the structural mechanism of plasticity in metallic glasses; however, it still remains a challenge to directly reveal the structural change, if any, that precedes; and dominant plastics flow in them. Here, by using the dynamic atomic force microscope as an "imaging"as well as a "forcing"tool, we unfold a real-time sequence of structural evolution occurring on the surface of an Au-Si thin film metallic glass. In sharp contrast to the common notion that plasticity comes along with mechanical softening in bulk metallic glasses, our experimental results directly reveal three types of nano-sized surface regions, which undergo plasticity but exhibit different characters of structural evolution following the local plasticity events, including stochastic structural rearrangement, unusual local relaxation and rejuvenation. As such, yielding on the metallic-glass surface manifests as a dynamic equilibrium between local relaxation and rejuvenation as opposed to shear instability in bulk metallic-glasses. Our finding demonstrates that plasticity on the metallic glass surface of Au-Si metallic glass bears much resemblance to that of the colloidal gels, of which nonlinear rheology rather than shear instability governs the constitutive behavior of plasticity. Published by AIP Publishing.

RI Yang, Yong/G-9148-2011

OI Yang, Yong/0000-0002-0491-8295; HUANG, Jacob Chih

Ching/0000-0001-6843-3396

SN 0021-8979

EI 1089-7550

PD MAR 7

PY 2017

VL 121

IS 9

AR 095304

DI 10.1063/1.4977856

UT WOS:000397419900041

ER

PT J

AU Qin, FX

Yoshimura, M

Wang, XM

Zhu, SL

Kawashima, A

Asami, K

Inoue, A

AF Qin, Fengxiang

Yoshimura, Masahiro

Wang, Xinming

Zhu, Shengli

Kawashima, Asahi

Asami, Katsuhiko

Inoue, Akihisa

TI Corrosion Behavior of a Ti-based bulk metallic glass and its crystalline

alloys

SO MATERIALS TRANSACTIONS

CT 5th International Conference on Bulk Metallic Glasses

CY OCT 01-05, 2006

CL Osaka Univ, Awaji Isl, JAPAN

SP Minist Educ, Culture, Sports, Sci & Technol, Inst Mat Res, Tohoku Univ, Japan Soc Promot Sci, Natl Inst Mat Sci, Hyogo Int Assoc

HO Osaka Univ

AB The structure, thermal stability and corrosion behavior of the Ti40Zr10Cu36Pd14 bulk metallic glass and its crystalline alloys have been investigated in this paper. The results of potentiodynamic polarization measurements revealed that the Ti-based bulk metallic glass and its crystalline counterparts examined were spontaneously passivated by anodic polarization with the passive current density about 10(-2) A/m(2). The higher corrosion resistance for the Ti-based bulk metallic glass and partly crystalline alloys was attributed to stable and protective passive films enriched with titanium and Zirconium. The Ti40Zr10Cu36Pd14 bulk metallic glass and its partial crystalline alloys produced recently without toxic elements are expected to be applied as biomaterials.

RI zhu, shengli/A-8818-2011; BAI, JIE/D-7448-2016; Qin,

Fengxiang/A-8359-2011; Inoue, Akihisa/E-5271-2015; Yoshimura,

Masahiro/H-5938-2011; Zhu, Shengli/D-5281-2009

OI Yoshimura, Masahiro/0000-0003-1810-0301; Zhu,

Shengli/0000-0002-0190-2626

SN 1345-9678

EI 1347-5320

PD JUL

PY 2007

VL 48

IS 7

BP 1855

EP 1858

DI 10.2320/matertrans.MJ200713

UT WOS:000248743100058

ER

PT J

AU Wang, RJ

Wang, WH

Li, FY

Wang, LM

Zhang, Y

Wen, P

Wang, JF

AF Wang, RJ

Wang, WH

Li, FY

Wang, LM

Zhang, Y

Wen, P

Wang, JF

TI The Gruneisen parameter for bulk amorphous materials

SO JOURNAL OF PHYSICS-CONDENSED MATTER

AB The longitudinal and shear velocities, and their pressure dependences, for various bulk amorphous materials, including bulk metallic glasses and nonmetallic glasses, have been measured by the ultrasonic pulse echo overlap method under hydrostatic pressure (maximum: 2 GPa) or uniaxial compression in ambient conditions. The second- or third-order elastic constants and the long-wavelength acoustic mode Gruneisen parameter are calculated. The results reveal that all metallic glasses have positive pressure dependences of both the longitudinal and shear velocities, while for most-non-metallic glasses both the longitudinal and shear velocities decrease with increasing pressure. Thus, the Gruneisen parameters evaluated for the mean mode and shear mode for metallic glasses and non-metallic glasses have opposite signs. This indicates that the influences of the short-range-order structure and bonding, correlating closely with the atomic configurations in the various amorphous materials, play an important role in the properties determined by vibrational anharmonicity. The negative and positive Gruneisen parameters exhibit mode softening and stiffness under high pressure, respectively. The results also clearly demonstrate that the Gruneisen parameter is affected by the pressure derivative of the shear modulus, especially for non-metallic glasses.

RI ZHANG, Yong/B-7928-2009

OI ZHANG, Yong/0000-0002-6355-9923

SN 0953-8984

PD JAN 29

PY 2003

VL 15

IS 3

BP 603

EP 608

AR PII S0953-8984(03)55838-6

DI 10.1088/0953-8984/15/3/324

UT WOS:000181100900028

ER

PT J

AU Choi-Yim, H

Conner, RD

Szuecs, F

Johnson, WL

AF Choi-Yim, H

Conner, RD

Szuecs, F

Johnson, WL

TI Quasistatic and dynamic deformation of tungsten reinforced

Zr57Nb5Al10Cu15.4Ni12.6 bulk metallic glass matrix composites

SO SCRIPTA MATERIALIA

AB Quasistatic and dynamic deformation behavior of composites of Zr57Nb5Al10Cu15.4Ni12.6 metallic glass reinforced with tungsten is studied. The plastic deformation of the metallic glass was increased under quasistatic compression in composites. Localized shear band fail-are of these composites results in self-sharpening behavior during ballistic impact. (C) 2001 Acta Materialia Inc. Published by Elsevier Science Ltd. All rights reserved.

SN 1359-6462

PD NOV 7

PY 2001

VL 45

IS 9

BP 1039

EP 1045

DI 10.1016/S1359-6462(01)01134-4

UT WOS:000172025200006

ER

PT J

AU Lee, DM

Sun, JH

Shin, SY

Bae, JC

Lee, CH

AF Lee, Dong-Myoung

Sun, Ju-Hyun

Shin, Seung-Yong

Bae, Jeong-Chan

Lee, Chi-Hwan

TI Improvement of glass forming ability of Cu-Ni-Zr-Ti alloys by

substitution of Hf and Nb

SO MATERIALS TRANSACTIONS

AB New bulk metallic glasses exhibiting a high glass forming ability were formed by a substitution of Hf and Nb for Zr and Ti in quaternary Cu-Ni-Zr-Ti system. An 8 mm-diameter BMG rod was obtained at the Cu50Ni8Zr15Hf3Ti23Nb1 composition by suction casting method. The glass transition temperature, crystallization temperature and reduced glass transition temperature of the BMG were 699 K, 754 K, and 0.59, respectively. Making an alloy system more complex was useful to increase the GFA.

SN 1345-9678

EI 1347-5320

PD JUN

PY 2008

VL 49

IS 6

BP 1486

EP 1489

DI 10.2320/matertrans.MRP2008066

UT WOS:000258160900046

ER

PT J

AU Qiao, JC

Cardinal, S

Pelletier, JM

Kato, H

AF Qiao, J. C.

Cardinal, S.

Pelletier, J. M.

Kato, H.

TI Insight on the process ability of bulk metallic glasses by

thermo-mechanical analysis and dynamic mechanical analysis

SO JOURNAL OF ALLOYS AND COMPOUNDS

AB Mechanical properties of glassy materials exhibit drastic changes in the supercooled liquid region (SLR). In the current research, mechanical properties in several typical bulk metallic glasses (Pd42.5Ni7.5Cu30P20, Zr56Co28Al16 and Zr58Nb3Cu16Ni13Al10) were measured using both dynamic mechanical analysis and thermo-mechanical analysis. The modifications of elastic modulus (determined by dynamic mechanical analysis) and viscosity (determined by thermo-mechanical analysis) are strongly connected. The experimental findings suggest that the elastic modulus of the bulk metallic glasses evaluated by dynamic mechanical analysis correlates with the length variations determined by thermo-mechanical analysis facilities. Evidences from the experiments demonstrate that the modifications are very important in the bulk metallic glass with a large supercooled region and a large fragility, (Pd42.5Ni7.5Cu30P20), very limited in the bulk metallic glass with a limited supercooled region and a small fragility (Zr56Co28Al16), intermediate in the third case (Zr58Nb3Cu16Ni13Al10). The onset of crystallization corresponds to the end of the deformation of the sample and to the onset of the hardness increase. Therefore, these two different techniques (dynamic mechanical analysis and thermo-mechanical analysis) can provide useful and insightful information on the process ability of these amorphous materials. (C) 2015 Elsevier B.V. All rights reserved.

RI Kato, Hidemi/B-2492-2015

SN 0925-8388

EI 1873-4669

PD APR 15

PY 2015

VL 628

BP 357

EP 363

DI 10.1016/j.jallcom.2014.12.166

UT WOS:000349084100058

ER

PT J

AU Fan, GJ

Loffler, JF

Wunderlich, RK

Fecht, HJ

AF Fan, GJ

Loffler, JF

Wunderlich, RK

Fecht, HJ

TI Thermodynamics, enthalpy relaxation and fragility of the bulk metallic

glass-forming liquid Pd43Ni10Cu27P20

SO ACTA MATERIALIA

AB Thermodynamic properties, enthalpy relaxation and fragility of a Pd43Ni10Cu27P20 bulk metallic glass-forming liquid have been investigated and the thermodynamic functions been calculated. The enthalpy relaxation from the amorphous state into the equilibrium supercooled liquid state was found to follow a stretched exponential function with the relaxation time obeying an Arrhenius law. The fragility index, calculated from the heating rate dependence of T-g, yields a value of 65. The excellent glass-forming ability of this alloy is attributed to a smaller thermodynamic driving force and larger diffusion length for crystallization than in other bulk metallic glass-forming alloys. (C) 2003 Acta Materialia Inc. Published by Elsevier Ltd. All rights reserved.

SN 1359-6454

PD FEB 9

PY 2004

VL 52

IS 3

BP 667

EP 674

DI 10.1016/j.actamat.2003.10.003

UT WOS:000188855100015

ER

PT J

AU Wang, WH

AF Wang, Wei Hua

TI The elastic properties, elastic models and elastic perspectives of

metallic glasses

SO PROGRESS IN MATERIALS SCIENCE

AB Bulk metallic glass (BMG) provides plentiful precise knowledge of fundamental parameters of elastic moduli, which offer a benchmark reference point for understanding and applications of the glassy materials. This paper comprehensively reviews the current state of the art of the study of elastic properties, the establishments of correlations between elastic moduli and properties/features, and the elastic models and elastic perspectives of metallic glasses. The goal is to show the key roles of elastic moduli in study, formation, and understanding of metallic glasses, and to present a comprehensive elastic perspectives on the major fundamental issues from processing to structure to properties in the rapidly moving field. A plentiful of data and results involving in acoustic velocities, elastic constants and their response to aging, relaxation, applied press, pressure and temperature of the metallic glasses have been compiled. The thermodynamic and kinetic parameters, stability, mechanical and physical properties of various available metallic glasses especially BMGs have also been collected. A survey based on the plentiful experimental data reveals that the linear elastic constants have striking systematic correlations with the microstructural features, glass transition temperature, melting temperature, relaxation behavior, boson peak, strength, hardness, plastic yielding of the glass, and even rheological properties of the glass forming liquids. The elastic constants of BMGs also show a correlation with a weighted average of the elastic constants of the constituent elements. We show that the elastic moduli correlations can assist in selecting alloying components with suitable elastic moduli for controlling the elastic properties and glass-forming ability of the metallic glasses, and thus the results would enable the design, control and tuning of the formation and properties of metallic glasses.

We demonstrate that the glass transition, the primary and secondary relaxations, plastic deformation and yield can be attributed to the free volume increase induced flow, and the flow can be modeled as the activated hopping between the inherent states in the potential energy landscape. We then propose an extended elastic model to understand flow in metallic glass and glass-forming supercooled liquid, and the model presents a simple and quantitative mathematic expression for flow activation energy of various glasses. The elastic perspectives, which consider all metallic glasses exhibit universal behavior based on a small number of readily measurable parameters of elastic moduli, are presented for understanding the nature and diverse properties of the metallic glasses. (C) 2011 Elsevier Ltd. All rights reserved.

SN 0079-6425

EI 1873-2208

PD APR

PY 2012

VL 57

IS 3

BP 487

EP 656

DI 10.1016/j.pmatsci.2011.07.001

UT WOS:000300654000002

ER

PT J

AU Wang, K

Chen, MW

Pan, D

Fujita, T

Zhang, W

Wang, XM

Inoue, A

AF Wang, K.

Chen, M. W.

Pan, D.

Fujita, T.

Zhang, W.

Wang, X. M.

Inoue, A.

TI Plastic deformation energy of bulk metallic glasses

SO MATERIALS SCIENCE AND ENGINEERING B-ADVANCED FUNCTIONAL SOLID-STATE

MATERIALS

CT 1st International Conference on Science Technology for Advanced Ceramics

(STAC)/2nd International Conference on Joining Technology for New

Metallic Glasses and Inorganic Materials (JTMC)

CY MAY 23-25, 2007

CL Kanagawa, JAPAN

SP Tokyo Inst Technol, 21st Century Ctr Excellence Program, Mat Sci Dept Grp

AB We develop a new method to characterize the plastic deformation energy (PDE) of bulk metallic glasses (BMGs) using depth-sensitive nanoindentation. In this work, six BMGs with different ductility were investigated by measuring their PDE of the first pop-in events during loading. The PDEs of these BMGs are found to be closely correlated to their ductility. The ductile BMGs have smaller PDE values whereas the brittle BMGs have larger values. (C) 2007 Elsevier B.V. All rights reserved.

RI CHEN, Mingwei/A-4855-2010; Pan, Deng/A-1090-2018; Pan, Deng/C-2072-2009;

Wang, Ke/C-8021-2011; Inoue, Akihisa/E-5271-2015; Wang, Ke/M-7171-2017;

Fujita, Takeshi/B-1867-2009

OI CHEN, Mingwei/0000-0002-8274-3099; Wang, Ke/0000-0002-1174-0907; Fujita,

Takeshi/0000-0002-2318-0433; Chen, Mingwei/0000-0002-2850-8872

SN 0921-5107

PD FEB 25

PY 2008

VL 148

IS 1-3

BP 101

EP 104

DI 10.1016/j.mseb.2007.09.061

UT WOS:000256276200024

ER

PT J

AU He, J

Mattern, N

Kaban, I

Dai, FP

Song, KK

Yan, ZJ

Zhao, JZ

Kim, DH

Eckert, J

AF He, Jie

Mattern, Norbert

Kaban, Ivan

Dai, Fuping

Song, Kaikai

Yan, Zhijie

Zhao, Jiuzhou

Kim, Do Hyang

Eckert, Jurgen

TI Enhancement of glass-forming ability and mechanical behavior of

zirconium-lanthanide two-phase bulk metallic glasses

SO JOURNAL OF ALLOYS AND COMPOUNDS

AB Development of the two-phase bulk metallic glasses (BMGs) is essentially retarded due to difficulties in finding of phase-separated (immiscible) alloys with high glass-forming ability (GFA) of coexistent phases. Referring to the concept of solute partitioning and minimization of free energy, in this work we present an idea that a metallic liquid system containing two liquids with individual self-assembled eutectic composition may yield two-phase BMGs upon casting. The formation of the two-glass structure is discussed and a strategy of partial substitution of chemically similar elements for overcoming the drawback of low GFA is proposed. A family of two-phase zirconium-lanthanide based metallic glasses in bulk form is developed. The mechanical behavior of the two-phase BMGs with different ratio of zirconium-rich to lanthanide-rich glassy phase is studied for the first time. This work provides a new concept for fabrication of two-phase BMGs and reveals the role of constituent phases in determining the mechanical properties of the whole glass. (C) 2014 Elsevier B.V. All rights reserved.

RI Dai, Fuping/C-1886-2015; song, kaikai/G-5908-2011

OI Song, Kaikai/0000-0002-5832-7546

SN 0925-8388

EI 1873-4669

PD JAN 5

PY 2015

VL 618

BP 795

EP 802

DI 10.1016/j.jallcom.2014.08.226

UT WOS:000344208800118

ER

PT J

AU Wang, L

Wang, X

Zhang, G

Sun, X

AF Wang, L.

Wang, X.

Zhang, G.

Sun, X.

TI PHASE TRANSITION AND PLASTICITY ENHANCEMENT OF Ti-Cu-BASED BULK METALLIC

GLASSES

SO ARCHIVES OF METALLURGY AND MATERIALS

AB In this paper, we report the complex crystallization kinetics of phase transition happening in Ti-Cu-based bulk metallic glasses (BMGs), which play significant roles in the glass formation with respect to their low reduced glass transition temperatures, T-rg. The first exothermic event just occurs when annealing the BMG samples in the supercooled liquid region, leading to the Avrami exponent deviating from conventional modes affected by the residual amorphous phase. For Ti43Cu43Ni7Zr7 BMG, the plasticity can be improved by pre-annealing at a sub-T-g temperature of 623K (approximate to 50K below T-g) for 0.5 hour, however, deteriorated by 1 hour annealing, which could be related to the change in stability of this BMG against crystallization with different pre-annealing times.

SN 1733-3490

EI 2300-1909

PY 2018

VL 63

IS 1

BP 387

EP 391

DI 10.24425/118952

UT WOS:000432340700052

ER

PT J

AU Flores, KM

Suh, D

Howell, R

Asoka-Kumar, P

Sterne, PA

Dauskardt, RH

AF Flores, KM

Suh, D

Howell, R

Asoka-Kumar, P

Sterne, PA

Dauskardt, RH

TI Flow and fracture of bulk metallic glass alloys and their composites

SO MATERIALS TRANSACTIONS

CT Bulk Metallic Glasses Conference

CY SEP 24-28, 2000

CL SINGAPORE, SINGAPORE

AB The fracture and plastic deformation mechanisms of a Zr-Ti-Ni-Cu-Be bulk metallic glass and a composite utilizing a crystalline reinforcement phase are reviewed. The relationship between stress state? free volume and shear band formation are discussed. Positron annihilation techniques were used to confirm the predicted increase in free volume after plastic straining. Strain localization and failure were examined for a wide range of stress states. Finally, methods for toughening metallic glasses are considered. Significant increases in toughness are demonstrated for a composite bulk metallic glass containing a ductile second phase which stabilizes shear band formation and distributes plastic deformation.

SN 1345-9678

EI 1347-5320

PD APR

PY 2001

VL 42

IS 4

BP 619

EP 622

DI 10.2320/matertrans.42.619

UT WOS:000168864100014

ER

PT J

AU Zhao, K

Liu, KS

Li, JF

Wang, WH

Jiang, L

AF Zhao, K.

Liu, K. S.

Li, J. F.

Wang, W. H.

Jiang, L.

TI Superamphiphobic CaLi-based bulk metallic glasses

SO SCRIPTA MATERIALIA

AB A superamphiphobic (both superhydrophobic and superoleophobic) CaLi-based bulk metallic glass (BMG) surface was constructed by etching the surface with water and modifying the etched surface with fluoroalkylsilane coating. The modified surface exhibits high corrosion resistance and stable superamphiphobicity with a high static contact angle of more than 150 degrees these properties could extend the practical applications of BMGs. The superamphiphobicity mechanism of the BMG is discussed. The facile method can be used to form other BMG surfaces with special wettability. (C) 2008 Acta Materialia Inc. Published by Elsevier Ltd. All rights reserved.

RI liu, Kesong/F-2239-2010

SN 1359-6462

PD FEB

PY 2009

VL 60

IS 4

BP 225

EP 227

DI 10.1016/j.scriptamat.2008.10.009

UT WOS:000263281100009

ER

PT J

AU Huang, YJ

Chiu, YL

Shen, J

Chen, JJJ

Sun, JF

AF Huang, Yongjiang

Chiu, Yu Lung

Shen, Jun

Chen, John J. J.

Sun, Jianfei

TI Cooling rate effect of nanomechanical response for a Ti-based bulk

metallic glass

SO JOURNAL OF NON-CRYSTALLINE SOLIDS

AB The effect of cooling rate on the mechanical behaviors of a Ti-based bulk metallic glass has been studied using nanoindentation technique. It is found that the hardness increases, while the plastic deformation capacity gradually decreases from the edge to the center of the sample. The variation of the structural uniformity within the as-cast glassy sample may account for the cooling rate dependence of mechanical performance. (C) 2010 Elsevier B.V. All rights reserved.

RI Huang, Yongjiang/D-4809-2009

SN 0022-3093

EI 1873-4812

PD MAY 1

PY 2010

VL 356

IS 20-22

BP 966

EP 970

DI 10.1016/j.jnoncrysol.2010.02.002

UT WOS:000277763600004

ER

PT J

AU Eckert, J

Das, J

Pauly, S

Duhamel, C

AF Eckert, J.

Das, J.

Pauly, S.

Duhamel, C.

TI Mechanical properties of bulk metallic glasses and composites

SO JOURNAL OF MATERIALS RESEARCH

AB The development of bulk metallic glasses and composites for improving the mechanical properties has occurred with the discovery of: many ductile metallic glasses and glass matrix composites with second phase dispersions with different length scales. This article reviews the processing, microstructure development, and resulting mechanical properties of Zr-, Ti-, Cu-, Mg-, Fe-, and Ni-based glassy alloys and also considers the superiority of composite materials containing different phases for enhancing the strength, ductility, and toughness, even leading to a "work-hardening-like" behavior. The morphology, shape, and length scale of the second phase dispersions are crucial for the delocalization of shear bands. The article concludes with some comments regarding future directions of the investigations of spatially inhoniogeneOLIS metallic glasses.

RI Das, Jayanta/G-1559-2010; Duhamel, Cecilie/B-5106-2013

OI Das, Jayanta/0000-0001-8750-5463;

SN 0884-2914

PD FEB

PY 2007

VL 22

IS 2

BP 285

EP 301

DI 10.1557/JMR.2007.0050

UT WOS:000244210800005

ER

PT J

AU Qiao, JC

Pelletier, JM

Kou, HC

Zhou, X

AF Qiao, J. C.

Pelletier, J. M.

Kou, H. C.

Zhou, X.

TI Modification of atomic mobility in a Ti-based bulk metallic glass by

plastic deformation or thermal annealing

SO INTERMETALLICS

AB Evolution of atomic mobility in a Ti40Zr25Ni8Cu9Be18 bulk metallic glass was studied by dynamic mechanical analysis (DMA) in different states: as-cast, after structural relaxation, after crystallization and after deformation (cold-rolled). Characteristics of Ti40Zr25Ni8Cu9Be18 bulk metallic glass are similar to that observed in other based bulk metallic glasses in the amorphous state: at low temperature, the material is mainly elastic and the mechanical response is independent of driving frequency, while on the other hand, the visco-elastic component becomes very large in the glass transition region. Structural relaxation and crystallization induce a decrease in the visco-elastic component, suggesting that atomic mobility is reduced. In contrast, atomic mobility is increased by a plastic deformation (i.e. cold-rolling). The higher is the cold-rolling ratio, the higher is the visco-elastic component. The experimental results were analyzed in the framework of quasi-point defects theory, which is based on the existence of defects in amorphous materials (polymer, bulk metallic glasses and other non-crystalline solids). (C) 2012 Elsevier Ltd. All rights reserved.

SN 0966-9795

PD SEP

PY 2012

VL 28

BP 128

EP 137

DI 10.1016/j.intermet.2012.04.004

UT WOS:000307038700018

ER

PT J

AU Tamura, T

Rachmat, RS

Mizutani, Y

Miwa, K

AF Tamura, T

Rachmat, RS

Mizutani, Y

Miwa, K

TI Effects of the intensity and frequency of electromagnetic vibrations on

glass-forming ability in Mg-Cu-Y bulk metallic glasses

SO MATERIALS TRANSACTIONS

AB The present authors reported that a new method for producing Mg-Cu-Y bulk metallic glasses by using electromagnetic vibrations is effective in forming the metallic glass phase, and disappearance or decrement of clusters by the electromagnetic vibrations applied to a liquid state is presumed to cause suppression of crystal nucleation [Nature Materials 4 (2005) 289]. This paper aims to investigate the effects of the intensity and frequency of electromagnetic vibrations on apparent glass-forming ability in the Mg-Cu-Y bulk metallic glasses. It was found that the apparent glass-forming ability Of Mg65Cu25Y10 alloys increases with increasing the frequency of electromagnetic vibrations up to 5000 Hz. The effects of frequency more than 5000 Hz could not be investigated because of alternating current power devices. Moreover, it was found that the apparent glass-forming ability Of Mg65Cu25Y10 alloys increases with increasing the intensity of electromagnetic vibrations by an electric current or a magnetic flux density. However, increasing excessively the electric current was found to weaken the enhancement of the apparent lass-forming ability by using the electromagnetic vibration process because the crystalline particles grow larger by the Joule heat.

RI Tamura, Takuya/D-4854-2017

OI Tamura, Takuya/0000-0001-6083-913X

SN 1345-9678

EI 1347-5320

PD AUG

PY 2005

VL 46

IS 8

BP 1918

EP 1922

DI 10.2320/matertrans.46.1918

UT WOS:000231476600036

ER

PT J

AU Zeng, QS

Liu, JF

Zhang, GQ

Wang, LN

Jiang, JZ

AF Zeng, Q. S.

Liu, J. F.

Zhang, G. Q.

Wang, L. N.

Jiang, J. Z.

TI Synthesis of LaCe-based bulk metallic glasses with low glass transition

temperature

SO INTERMETALLICS

CT 6th International Workshop on Advanced Intermetallic and Metallic

Materials

CY OCT 09-15, 2005

CL Yangzhou, PEOPLES R CHINA

SP Natl Nat Sci Fdn China, Minist Sci & Technol China, Nanjing Univ Sci & Technol, Univ Sci & Technol Beijing, Oak Ridge Natl Lab, Shenyang Natl Lab Mat Sci, Cent S Univ, Harbin Inst Technol, Yangzhou City Govt

AB LaCe-based bulk metallic glasses (BMGS) with diameter up to 10 mm have been synthesized in copper mold. The BMG alloys have low glass transition temperatures, about 403 K and large Supercooled liquid regions up to 58 K. The hardness of the BMG alloys is around 1.9 GPa. They also exhibit large plasticity and imprintability feature in the supercooled liquid region, which make the alloy a potential imprinting material. In addition, the BMG alloys might offer an ideal model system for the study of glass transition with a large experimentally accessible time and low temperature windows. (c) 2006 Elsevier Ltd. All rights reserved.

SN 0966-9795

EI 1879-0216

PD MAY-JUN

PY 2007

VL 15

IS 5-6

BP 753

EP 756

DI 10.1016/j.intermet.2006.10.012

UT WOS:000246739400028

ER

PT J

AU Cao, D

Wu, Y

Wang, H

Liu, XJ

Lu, ZP

AF Cao, Di

Wu, Yuan

Wang, Hui

Liu, Xiong-Jun

Lu, Z. P.

TI Effects of Nitrogen on the Glass Formation and Mechanical Properties of

a Ti-Based Metallic Glass

SO ACTA METALLURGICA SINICA-ENGLISH LETTERS

AB Effects of nitrogen addition on glass formation and mechanical properties of the Ti42.5Cu40Zr10Sn2.5 metallic glass were systematically investigated. It was found that a small amount of nitrogen addition facilitated the glass formation by suppressing formation of the competing eutectic structure. Unlike large atomic size elements such as Hf and Pd which usually deteriorate specific strength, nitrogen can also increase the specific strength of the current Ti-based BMGs. The results are not only helpful for understanding glass-forming ability in general, but also useful in developing cost-effective, high-performance Ti-based bulk metallic glasses with enhanced glass-forming ability.

RI Wang, Hui/A-5775-2017; Lu, Zhao-Ping/A-2718-2009; Liu,

Xiong-Jun/C-7119-2009

OI Wang, Hui/0000-0002-4915-3396; Lu, Zhao-Ping/0000-0003-1463-8948; Cao,

Di/0000-0003-3517-4740

SN 1006-7191

EI 2194-1289

PD FEB

PY 2016

VL 29

IS 2

BP 173

EP 180

DI 10.1007/s40195-016-0374-5

UT WOS:000373096300010

ER

PT J

AU Lu, BC

Yao, JH

Xu, J

Li, Y

AF Lu, Bao-Chen

Yao, Jia-Hao

Xu, Jian

Li, Yi

TI A model of atom dense packing for metallic glasses with high-solute

concentration

SO APPLIED PHYSICS LETTERS

AB Taking the chemical ordering in the metallic glasses into consideration, we have extended the efficient cluster packing model to predict the composition in high-solute concentration alloys with atomic dense packing. Its validity is supported by the good agreements between the predicted compositions with the maximum packing efficiency and the experimentally optimized bulk metallic glass formers in the Cu-Zr, Cu-Hf, and Ni-Nb binary systems. Despite its simplicity, it seems that the structure predicted by our model reflects in certain way the averaged structural configuration of metallic glasses.

SN 0003-6951

PD JUN 15

PY 2009

VL 94

IS 24

AR 241913

DI 10.1063/1.3157136

UT WOS:000267166600035

ER

PT J

AU Nascimento, M

Ragani, J

Gravier, S

Blandin, JJ

Soubeyroux, JL

AF Nascimento, M.

Ragani, J.

Gravier, S.

Blandin, J. J.

Soubeyroux, J. L.

TI Effect of temperature on the mechanical behaviour of a Ni-free

zirconium-based bulk metallic glass

SO JOURNAL OF ALLOYS AND COMPOUNDS

CT 15th International Symposium on Metastable, Amorphous and Nanostructured

Materials

CY JUL 06-10, 2008

CL Buenos Aires, ARGENTINA

AB It is well known that nickel-free zirconium-based bulk metallic glasses are of interest for their possible good biocompatibility properties. In this framework, a Zr-Nb-Cu-Al-Fe bulk metallic glass was elaborated under the form of 2 mm diameter rods. The amorphous structure was confirmed by XRD and DSC and the associated reduced index were measured The mechanical properties of the glass were investigated at both room and high temperature. At room temperature, the glass exhibits some macroscopic plasticity The rheology at high temperature was studied by both strain rate jump tests and tests at constant strain rate. From these tests, It was difficult to identify an experimental window in which the amorphous structure could be maintained and in which viscoplastic forming could be carried out under low flow stresses. In the super-cooled liquid region, this behaviour is discussed in relation with the resistance to crystallization of the investigated glass and the nature of the formed crystallites was studied by XRD The mechanical properties of this glass are also compared to results previously obtained in the case of other zirconium-based bulk metallic glasses. (C) 2009 Elsevier B V. All rights reserved.

SN 0925-8388

PD APR 16

PY 2010

VL 495

IS 2

BP 348

EP 351

DI 10.1016/j.jallcom.2009.10.186

UT WOS:000278749900016

ER

PT J

AU Zhao, K

Luo, Q

Zhao, DQ

Bai, HY

Pan, MX

Wang, WH

AF Zhao, K.

Luo, Q.

Zhao, D. Q.

Bai, H. Y.

Pan, M. X.

Wang, W. H.

TI Bulk metallic glasses based on binary rare earth elements

SO JOURNAL OF NON-CRYSTALLINE SOLIDS

AB Bulk metallic glasses (BMGs) are usually based on single element such as Zr, Cu, and Fe. In this work, we report the formation of a series of BMGs based on arbitrarily selected binary rare earth elements. Compared with single-base BMGs, the binary-base BMGs have unique characteristics of excellent glass-forming ability, tunable physical, chemical and mechanical properties. The binary-base BMGs might be of significance in scientific studies and have potential applications, and the approach for formation of metallic glasses has implications in the search for new BMG systems. (C) 2009 Elsevier B.V. All rights reserved.

SN 0022-3093

EI 1873-4812

PD JUN 15

PY 2009

VL 355

IS 16-17

BP 1001

EP 1004

DI 10.1016/j.jnoncrysol.2009.04.007

UT WOS:000267085400015

ER

PT J

AU Yang, L

Guo, GQ

AF Yang, Liang

Guo, Gu-Qing

TI Structural origin of the high glass-forming ability in Gd doped bulk

metallic glasses

SO APPLIED PHYSICS LETTERS

AB The structural origin of the high glass-forming ability (GFA) in Gd-doped CuZrAl bulk metallic glasses was investigated via synchrotron radiation techniques combined with simulations. It is found that 2 at. % Gd addition increases and stabilizes the solute-centered clusters, which leads to the relatively high atomic- and cluster-level packing efficiency and GFA in Cu(46)Zr(45)Al(7)Gd(2) alloy. However, the presence of Al-Gd solute-solute bonding in Cu(46)Zr(42)Al(7)Gd(5) decreases the packing efficiency, deteriorating the GFA of this alloy. These findings may extend our understanding on the sensitive dependence of GFA on the concentration of doping atoms in a number of multicomponent bulk metallic glasses. (C) 2010 American Institute of Physics. [doi:10.1063/1.3485117]

SN 0003-6951

PD AUG 30

PY 2010

VL 97

IS 9

AR 091901

DI 10.1063/1.3485117

UT WOS:000282187200010

ER

PT J

AU Kim, DH

Kim, WT

Park, ES

Mattern, N

Eckert, J

AF Kim, D. H.

Kim, W. T.

Park, E. S.

Mattern, N.

Eckert, J.

TI Phase separation in metallic glasses

SO PROGRESS IN MATERIALS SCIENCE

AB Phase separation phenomena in metallic glass systems are reviewed. First, a brief introduction to the enhanced glass forming ability and the phase separation in metallic glass systems is given. Nano-scale phase separation observed in frozen-in glass is discussed in marginal glass formers such as Pd-, Fe, Al-base metallic glass systems as well as in bulk glass formers in Cu-, Zr- and Mg-base metallic glass systems. Since retention of glass forming ability is essential in phase separating glasses, thermodynamic and kinetic conditions for enhanced glass forming ability is introduced. Several thermodynamic aspects for decomposition by liquid liquid phase separation which include stability conditions, decomposition in the multicomponent system, types of miscibility gap, calculation of bimodal and spinodal curves are introduced as a background for design of metallic glasses phase separating in the liquid state. The main mechanisms for phase separation are nucleation and growth mechanism and spinodal decomposition mechanism. The metallic glass systems which include an atom pair with large positive enthalpy of mixing decompose by liquid liquid phase separation separate in the liquid state, forming two typical types of microstructure: droplet or interconnected type microstructures depending on the mechanism of phase separation. By calculation of the tile line, it is possible to design phase separating metallic glasses with high glass forming ability. Eventually, bulk-type phase separating metallic glasses with mm scale can be synthesized. Moreover, if there is a group of three atoms with large positive enthalpy of mixing in the liquid state in the multicomponent system, occurrence of three phase separation in the liquid state is possible. There are several parameters affecting the microstructure evolution during phase separation, which includes glass transition and critical temperatures, shape of bimodal and spinodal curves and quenching conditions. Phase separation in the solid state of the frozen-in glasses can give a clue on the phase separation prior to crystallization or direct nanocrystallization from the amorphous matrix. The amplitude of the composition fluctuation with a fixed wavelength can grow by heating in the solid state, if the spinodal decomposition. Finally, some advantages of phase separation phenomena in metallic glass systems are highlighted. (C) 2013 Elsevier Ltd. All rights reserved.

RI Park, Eun Soo/A-4443-2008; Park, Eun Soo/A-9860-2014

SN 0079-6425

PD OCT

PY 2013

VL 58

IS 8

BP 1103

EP 1172

DI 10.1016/j.pmatsci.2013.04.002

UT WOS:000326484300001

ER

PT J

AU Rothfuss, D

Kuhn, U

Reiser, A

Fleischmann, A

Enss, C

AF Rothfuss, D.

Kuehn, U.

Reiser, A.

Fleischmann, A.

Enss, C.

TI Thermal Conductivity of Superconducting Bulk Metallic Glasses at Very

Low Temperatures

SO CHINESE JOURNAL OF PHYSICS

AB The low-temperature properties of superconducting metallic glasses are governed by atomic tunnelling states. The heat transport well below the transition into the superconducting state is limited by the resonant interaction of phonons with the tunnelling systems. So far, measurements of the thermal conductivity have been performed on thin amorphous films down to about 100 mK and on bulk metallic glasses to about 1 K. Using a novel non-contact method, we have investigated for the first time the thermal transport of a superconducting bulk metallic glass Zr52.5Ti5Cu17.9Ni14.6Al10 down to about 6 mK, testing the prediction of the tunnelling model and searching for a possible influence of nuclear moments on the heat flow. The observed temperature dependence of the thermal conductivity is in reasonable agreement with the prediction of the tunnelling model.

SN 0577-9073

PD FEB

PY 2011

VL 49

IS 1

SI SI

BP 384

EP 393

UT WOS:000286490100047

ER

PT J

AU Hechler, S

Gallino, I

Stolpe, M

Lentes, FT

Busch, R

AF Hechler, Simon

Gallino, Isabella

Stolpe, Moritz

Lentes, Frank-Thomas

Busch, Ralf

TI Analysis of thermophysical properties of lead silicates in comparison to

bulk metallic glasses

SO JOURNAL OF NON-CRYSTALLINE SOLIDS

AB The equilibrium viscosity and the specific heat capacity of three lead silicate glass forming liquids (flint glasses) with low glass transition temperatures are experimentally measured and compared to the thermophysical properties of network glass-formers like SiO2 and GeO2 and of bulk metallic glasses. The data are analyzed using the Vogel-Fulcher-Tarnmann (VFT) equation, the Mauro-Yue-Ellison-Gupta-Allan (MYEGA) equation, and the Adam-Gibbs approach. The flint glasses depart from the Arrhenius equation in terms of liquid kinetics and their corresponding kinetic fragilities are intermediate between GeO2 and the bulk metallic glass-formers. Among the lead silicates the fragility increases with increasing content of network modifiers, as lead, sodium and potassium modify the silicate network by creating non-bridging oxygen that softens the network.

SN 0022-3093

EI 1873-4812

PD APR 1

PY 2018

VL 485

BP 66

EP 73

DI 10.1016/j.jnoncrysol.2018.01.041

UT WOS:000427333900008

ER

PT J

AU Saida, J

Kato, H

Inoue, A

Ohnurna, M

AF Saida, J

Kato, H

Inoue, A

Ohnurna, M

TI Novel nanostructure and deformcifion behavior in rapidly quenched Cu-(Zr

or Hf)-Ti alloys

SO ADVANCED ENGINEERING MATERIALS

RI Inoue, Akihisa/E-5271-2015; Saida, Junji/C-1912-2009; Kato,

Hidemi/B-2492-2015

SN 1438-1656

EI 1527-2648

PD JAN

PY 2005

VL 7

IS 1-2

BP 39

EP 43

DI 10.1002/adem.200400160

UT WOS:000227152500010

ER

PT J

AU Li, S

Xi, XK

Wei, YX

Luo, Q

Wang, YT

Tang, MB

Zhang, B

Zhao, ZF

Wang, RJ

Pan, MX

Zhao, DQ

Wang, WH

AF Li, S

Xi, XK

Wei, YX

Luo, Q

Wang, YT

Tang, MB

Zhang, B

Zhao, ZF

Wang, RJ

Pan, MX

Zhao, DQ

Wang, WH

TI Formation and properties of new heavy rare-earth-based bulk metallic

glasses

SO SCIENCE AND TECHNOLOGY OF ADVANCED MATERIALS

AB A series of new families of heavy rare-earth (RE) based bulk metallic glasses (BMGs) with excellent glass-forming ability and high thermal stability have been obtained by a copper mold casting method. Compared with the light RE-based BMGs, the heavy RE-based BMGs have much higher glass transition and crystallization temperatures and higher elastic moduli. It is found that the thermal stability is closely correlated with the elastic constants in the RE-based BMGs. The correlations found are useful for guiding BMG-forming alloy design to enhance stability. (c) 2005 Elsevier Ltd. All rights reserved.

RI Zhao, Zuofeng/B-1297-2010

OI Zhao, Zuofeng/0000-0002-0862-8471

SN 1468-6996

PD OCT

PY 2005

VL 6

IS 7

BP 823

EP 827

DI 10.1016/j.stam.2005.06.019

UT WOS:000234328200020

ER

PT S

AU Yavari, AR

Inoue, A

AF Yavari, AR

Inoue, A

BE Ma, E

Atzmon, M

Koch, CC

TI Ductilisation of Fe-based metallic glasses

SO METASTABLE, MECHANICALLY ALLOYED AND NANOCRYSTALLINE MATERIALS

SE Materials Science Forum

CT International Symposium on Metastable, Mechanically Alloyed and

Nanocrystalline Materials

CY JUN 24-29, 2001

CL UNIV MICHIGAN, ANN ARBOR, MI

HO UNIV MICHIGAN

AB Bulk metallic glasses (BMGs) have potential applications due to their Newtonian viscous formability between the glass transition temperature T-g and crystallisation temperature T-x and because of their high elastic strain range of about 2% below T-g. Among various metallic glasses however, some are more brittle in nature. Fe-based metallic glasses and some others are prone to thermal embrittlement even during the quench. This occurs when the critical applied load required for propagation of cracks is below that for inducing plastic flow at the testing temperature. Higher metalloid contents are needed to obtain bulk Fe-based metallic glasses but such increase in metalloid content also leads to embrittlement of the glass. It is shown that lower metalloid contents lead to reduced ductile to brittle transition temperaturs Tdb due to contributions from structural free volume. However, the reduced metalloid contents needed for ductile behavious increase the critical cooling required for glass formation. Fe-based BMGs are of most interest for their magnetic properties and brittle mechanical behavious is not a major obstacle to applications based on magnetic properties. Nevertheless it may be possible to obtain ductile Fe-based BMG-crystalline phase composites for thermodynamically selected compositions..

RI yavari, alain/E-8192-2010; Inoue, Akihisa/E-5271-2015

SN 0255-5476

BN 0-87849-892-3

PY 2002

VL 386-3

BP 3

EP 9

DI 10.4028/www.scientific.net/MSF.386-388.3

UT WOS:000174657900001

ER

PT J

AU Miracle, DB

AF Miracle, D. B.

TI The efficient cluster packing model - An atomic structural model for

metallic glasses

SO ACTA MATERIALIA

AB A structural model is described for metallic glasses based on a new sphere packing scheme - the efficient filling of space by solute-centered clusters. This model combines random positioning of solvent atoms with atomic order of solutes. It shows that metallic glasses contain <= 4 topologically distinct species and that solutes possess specific sizes relative to solvent atoms to produce efficient atomic packing. Validation is achieved by quantitative predictions of nearest-neighbor partial coordination numbers, medium-range solute ordering, density and metallic glass topologies. Good agreement is achieved in each of these areas. This model is able to reproduce compositions for a broad range of metallic glasses, provides specific guidance for the exploration of new bulk metallic glasses and may give new insights into other metallic glass studies. The new scheme introduced here for the efficient filling of space in extended systems of unequal spheres may have relevance to other fields. (c) 2006 Acta Materialia Inc. Published by Elsevier Ltd. All rights reserved.

SN 1359-6454

PD SEP

PY 2006

VL 54

IS 16

BP 4317

EP 4336

DI 10.1016/j.actamat.2006.06.002

UT WOS:000240791900021

ER

PT J

AU Yu, P

Bai, HY

Tang, MB

Wang, WL

Wang, WH

AF Yu, P

Bai, HY

Tang, MB

Wang, WL

Wang, WH

TI CuZr-based bulk metallic glasses with good glass-forming ability

prepared by Al addition

SO ACTA PHYSICA SINICA

AB Based on the fabrication of CuZr binary bulk metallic glass (BMG), we have produced a series of CuZr-based BMGs prepared by Al addition using a copper mold casting method. CuZr-based BMGs with good glass-forming ability (GFA) have a broad glass-forming composition range. When Al content changes from 4% to 8%, CuZr-based BMGs can be produced in cylindrical rods with a diameter of at least 5mm. The physical reason of the good GFA in the alloys is analyzed. CuZr-based BMGs have a simple structure and are lower cost, so it is very possible to have potential application. At the same time, the way that we explored the CuZr-based BMGs is an effective route for designing and obtaining new multicomponent BMGs.

SN 1000-3290

PD JUL

PY 2005

VL 54

IS 7

BP 3284

EP 3289

UT WOS:000230231700054

ER

PT J

AU Wei, Y

AF Wei, Yujie Y

TI The intrinsic and extrinsic factors for brittle-to-ductile transition in

bulk metallic glasses

SO THEORETICAL AND APPLIED FRACTURE MECHANICS

AB We propose the ratio of critical strain energy density by distortional deformation over that by volumetric deformation as a material parameter to quantify the ductile-to-brittle transition in bulk metallic glasses (BMG). A BMG is regarded to be ductile (with high fracture toughness) if the ratio is low, implying shear dominated deformation precedes cavitation failure. In contrast, the BMG is brittle (with low fracture toughness) when the ratio is large, suggesting that cavitation is prone to occur before energy being dissipated via massive shear bands. The theory naturally reflects the intrinsic and extrinsic factors which could influence the brittle-to-ductile transition in bulk metallic glasses. (C) 2014 Elsevier Ltd. All rights reserved.

RI Wei, Yujie/A-3770-2009

OI Wei, Yujie/0000-0002-3213-7891

SN 0167-8442

EI 1872-7638

PD JUN

PY 2014

VL 71

SI SI

BP 76

EP 78

DI 10.1016/j.tafmec.2014.06.001

UT WOS:000341555500009

ER

PT J

AU Park, ES

Kim, DH

AF Park, ES

Kim, DH

TI Design of bulk metallic glasses with high glass forming ability and

enhancement of plasticity in metallic glass matrix composites: A review

SO METALS AND MATERIALS INTERNATIONAL

AB To overcome some of the limits of existing metallic alloys, a new alloy design concept has been introduced recently in order to control the crystallinity, i.e. to utilize crystalline, quasicrystalline, and amorphous structures. In particular, bulk metallic glasses (BMGs) receive great attention because of their unique properties due to their different atomic configuration. Recently, significant progress in enhancing glass forming ability (GFA) has led to the fabrication of BMGs having potential for application as structural and functional materials. Moreover, successful design of BMG matrix composite microstructure suggests that the plasticity of BMGs can be controlled property. In this review article, we introduce recent research results on the design of BMGs with high GFA and on the enhancement of plasticity in metallic glass matrix composites.

RI Park, Eun Soo/A-4443-2008; bang, changwook/J-7922-2012; Park, Eun

Soo/A-9860-2014

SN 1598-9623

PD FEB

PY 2005

VL 11

IS 1

BP 19

EP 27

DI 10.1007/BF03027480

UT WOS:000227448300003

ER

PT J

AU Chang, ZY

Huang, XM

Chen, LY

Ge, MY

Jiang, QK

Nie, XP

Jiang, JZ

AF Chang, Z. Y.

Huang, X. M.

Chen, L. Y.

Ge, M. Y.

Jiang, Q. K.

Nie, X. P.

Jiang, J. Z.

TI Catching Fe-based bulk metallic glass with combination of high glass

forming ability, ultrahigh strength and good plasticity in Fe-Co-Nb-B

system

SO MATERIALS SCIENCE AND ENGINEERING A-STRUCTURAL MATERIALS PROPERTIES

MICROSTRUCTURE AND PROCESSING

AB The Fe-based quaternary bulk metallic glass with critical diameter up to 2.5 mm was pinpointed in Fe-Co-Nb-B system using a strategy for catching the best glass former based on relative glass forming ability of alloys. The best glass former Fe56.04Co13.45Nb5.5B25 exhibits glass transition temperature of 821 K, supercooled region of 58 K, compressive strength of about 4.5 GPa, Vickers hardness of about 13.7 GPa, and compressive plastic strain of about 0.6%. The combination of high glass forming ability, high thermal stability, ultrahigh strength and good plasticity makes this alloy of potential applications as a structural material. (C) 2009 Elsevier B.V. All rights reserved.

RI Chen, Lianyi/B-3156-2008

OI Chen, Lianyi/0000-0003-3720-398X

SN 0921-5093

EI 1873-4936

PD AUG 20

PY 2009

VL 517

IS 1-2

BP 246

EP 248

DI 10.1016/j.msea.2009.03.082

UT WOS:000268760800035

ER

PT J

AU Jin, HJ

Gu, XJ

Zhou, F

Lu, K

AF Jin, HJ

Gu, XJ

Zhou, F

Lu, K

TI Compression stress induced flow temperature reduction in a bulk

Zr41.2Ti13.8CU12.5Ni10.0Be22.5 metallic glass

SO SCRIPTA MATERIALIA

AB For a bulk metallic glass Zr41.2Ti13.8Cu12.5Ni10.Be-0(22.5), flow behavior was investigated under uniaxial pressure up to 1.37 GPa upon continuous heating. Under compression stress, flow temperature is reduced by 45 K/1.37 GPa and the processible temperature range is expected to be possibly widened by about 70 K/1.37 GPa. (C) 2002 Acta Materialia Inc. Published by Elsevier Science Ltd. All rights reserved.

RI Jin, Hai-Jun/E-5179-2010

SN 1359-6462

PD DEC 2

PY 2002

VL 47

IS 11

BP 787

EP 791

AR PII S1359-6462(02)00302-0

DI 10.1016/S1359-6462(02)00302-0

UT WOS:000179402600012

ER

PT J

AU Fan, ZJ

Zheng, ZY

Jiao, ZB

AF Fan ZhenJun

Zheng ZhiYuan

Jiao ZengBao

TI Compressive fracture characteristics of Zr-based bulk metallic glass

SO SCIENCE CHINA-PHYSICS MECHANICS & ASTRONOMY

AB The compressive fracture characteristics of Zr-based bulk metallic glass under uniaxial compression tests are studied. The zigzag rheological behavior is observed in the compression stress-strain curves of amorphous alloys. At room temperature the uniaxial compression fracture takes place along the plane which is at a 45-degree angle to the direction of the compressive stress. The microstructure of a typical fracture pattern is the vein network. A unique, finger-like vein pattern is found to exist at the fracture surface of Zr-based bulk metallic glass.

OI Jiao, Z. B./0000-0002-0556-6869

SN 1674-7348

PD MAY

PY 2010

VL 53

IS 5

BP 823

EP 827

DI 10.1007/s11433-010-0154-6

UT WOS:000277449900009

ER

PT J

AU Kong, J

Xiong, DS

Li, JL

Yuan, QX

Tyagi, R

AF Kong, Jian

Xiong, Dangsheng

Li, Jianliang

Yuan, Qunxing

Tyagi, Rajnesh

TI Effect of Flash Temperature on Tribological Properties of Bulk Metallic

Glasses

SO TRIBOLOGY LETTERS

AB The tribological properties of Cu-based and Zr-based bulk metallic glasses (BMGs) sliding against Si(3)N(4) under dry and water lubrication were studied on a pin-on-disc tribometer. The wear mechanisms of bulk metallic glasses were investigated based on the calculated flash temperature. The friction coefficients if fully amorphous alloy are about 0.7, while those of BMGs with nanocrytalline are a little higher. The wear rates of Cu-based BMG (V101) are about one order of magnitude lower than those of Zr-based BMG (Vit1) under dry friction, even two orders of magnitude lower under water lubrication. The wear resistance of bulk metallic glasses was influenced by the flash temperature. The calculated flash temperature (3,337 K) on the friction surface of Zr-based amorphous alloy exceeds its glass transition temperature, even its melting temperature. The high flash temperature leads to glass transition accompanied with viscous flow and material transfer, which is responsible for the poor wear resistance of Zr-based BMGs.

RI xiong, dangsheng/B-1823-2015

OI xiong, dangsheng/0000-0003-3210-3728

SN 1023-8883

PD SEP

PY 2009

VL 35

IS 3

BP 151

EP 158

DI 10.1007/s11249-009-9444-4

UT WOS:000268329600001

ER

PT J

AU Melle, AK

Peres, MM

Bolfarini, C

Botta, WJ

Jorge, AM

Kiminami, CS

AF Melle, Ana Karla

Peres, Mauricio Mhirdaui

Bolfarini, Claudemiro

Botta, Walter Jose

Jorge, Alberto Moreira, Jr.

Kiminami, Claudio Shyinti

TI Consolidation of the Cu46Zr42Al7Y5 Amorphous Ribbons and Powder Alloy by

Hot Extrusion

SO MATERIALS RESEARCH-IBERO-AMERICAN JOURNAL OF MATERIALS

AB The amorphous Cu46Zr42Al7Y5 alloy presents large supercooled liquid region (Delta T-X = 100 K), with a viscosity of about 10(6) N.s/m(2) where the material can flow as a liquid, making it possible an easy deformation in this temperature region. The aim of this work was to analyze processing routes to produce bulks of metallic glasses. Two kinds of materials were used: amorphous powders and ribbons, both were consolidated by hot extrusion in temperatures inside the range between T-g and T-x, with a ram speed of 1 mm/min and extrusion ratio of 3 : 1. Analysis of X-Ray Diffratometry (XRD), Differential Scanning Calorimetry (DSC) and Scanning Electron Microscopy (SEM), revealed that the proposed consolidation routes were effective to produce large bulks of amorphous materials, even with the strong decreasing of Delta T-X observed after deformation by milling and during extrusion.

RI Jorge, Ariosto/K-2968-2014; Bolfarini, Claudemiro/E-4366-2012; KIMINAMI,

CLAUDIO/D-4402-2012; Botta, Walter/E-7763-2010; Jorge Junior,

Alberto/B-2566-2012

OI KIMINAMI, CLAUDIO/0000-0001-8231-7316; Botta,

Walter/0000-0003-2759-573X; Jorge Junior, Alberto/0000-0002-8121-9834;

Bolfarini, Claudemiro/0000-0002-3099-3694

SN 1516-1439

PD SEP-OCT

PY 2012

VL 15

IS 5

BP 728

EP 731

DI 10.1590/S1516-14392012005000095

UT WOS:000309191500006

ER

PT J

AU Zhang, DW

Wang, XD

Lou, HB

Cao, QP

Wang, LW

Zhang, DX

Jiang, JZ

AF Zhang, D. W.

Wang, X. D.

Lou, H. B.

Cao, Q. P.

Wang, L. W.

Zhang, D. X.

Jiang, J. Z.

TI Thermal behaviors of liquid La-based bulk metallic glasses

SO JOURNAL OF APPLIED PHYSICS

AB Thermal behaviors of liquid La-based bulk metallic glasses have been measured by using the dilatometer with a self-sealed sample cell. It is demonstrated that the strong glass forming liquid not only has the small thermal expansion coefficient but also shows the slow variation rate. Moreover, the strong glass former has relatively dense atomic packing and also small density change in the liquid state. The results suggest that the high glass forming ability of La-based metallic glasses would be closely related to the slow atomic rearrangements in liquid melts. (C) 2014 AIP Publishing LLC.

RI Wang, Lianwen/B-2289-2015; Zhejiang University, Dep. Optical

Eng./G-9022-2011

OI Wang, Lianwen/0000-0001-7324-2682; Lou, Hongbo/0000-0002-5056-2576

SN 0021-8979

EI 1089-7550

PD DEC 14

PY 2014

VL 116

IS 22

AR 224903

DI 10.1063/1.4903978

UT WOS:000346266300060

ER

PT J

AU Li, HX

Jiao, ZB

Gao, JE

Lu, ZP

AF Li, H. X.

Jiao, Z. B.

Gao, J. E.

Lu, Z. P.

TI Synthesis of bulk glassy Fe-C-Si-B-P-Ga alloys with high glass-forming

ability and good soft-magnetic properties

SO INTERMETALLICS

CT 7th International Conference on Bulk-Metallic Glasses

CY NOV 01-05, 2009

CL Busan, SOUTH KOREA

SP Yonsei Univ, Ctr Noncrystalline Mat

AB Effects of Ga additions on the glass-forming ability (GFA) and magnetic properties of the Fe(76)C(7.0)-Si(3.3)B(5.0)P(8.7) alloy were investigated. It was found that addition of 1-2% Ga can increase the critical diameter for glass formation from 1 to 3 mm. Moreover, the as-cast alloy containing 1% Ga exhibit good soft-magnetic properties including a high saturation magnetization value of 1.55 T and a low coercive force of 4.9 A/m. The combination of high GFA and good soft-magnetic properties make the newly developed Fe-based bulk metallic glasses promising for electric applications. (C) 2010 Elsevier Ltd. All rights reserved.

RI Lu, Zhao-Ping/A-2718-2009

OI Lu, Zhao-Ping/0000-0003-1463-8948; Jiao, Z. B./0000-0002-0556-6869

SN 0966-9795

PD OCT

PY 2010

VL 18

IS 10

SI SI

BP 1821

EP 1825

DI 10.1016/j.intermet.2010.01.021

UT WOS:000281420700008

ER

PT J

AU Madge, SV

Greer, AL

AF Madge, SV

Greer, AL

TI Effect of Ag addition on the glass-forming ability and thermal stability

of Mg-Cu-Y alloys

SO MATERIALS SCIENCE AND ENGINEERING A-STRUCTURAL MATERIALS PROPERTIES

MICROSTRUCTURE AND PROCESSING

CT 11th International Conference on Rapidly Quenched and Metastable

Materials

CY AUG 25-30, 2002

CL Univ Oxford, Dept Mat, Oxford, ENGLAND

HO Univ Oxford, Dept Mat

AB Effects of silver addition on a range Of Mg65Cu25-zAgzY10 (z = 0-15 at.%) bulk metallic glasses have been studied. No correlation could be seen between the glass-forming ability of these alloys and the thermal stability of the glasses on subsequent heating. Transmission electron microscopy and differential scanning calorimetry reveal that this discrepancy is not attributable to phase separation in the supercooled liquid. Silver addition enhances the glass-formability of these alloys because of a significant reduction in crystal growth rate, but reduces the thermal stability of these glasses, apparently by allowing growth on quenched-in nuclei. (C) 2003 Published by Elsevier B.V.

RI Greer, Lindsay/E-9433-2017; Greer, Alan Lindsay/G-1977-2011

OI Madge, Shantanu/0000-0001-7996-8652

SN 0921-5093

PD JUL 15

PY 2004

VL 375

SI SI

BP 759

EP 762

DI 10.1016/j.msea.2003.10.302

UT WOS:000223329700143

ER

PT J

AU Hsu, CF

Lin, HM

Lee, PY

AF Hsu, Chih-Feng

Lin, Hong-Ming

Lee, Pee-Yew

TI Characterization of Mechanically Alloyed Ti-based Bulk Metallic Glass

Composites Containing Carbon Nanotubes

SO ADVANCED ENGINEERING MATERIALS

SN 1438-1656

PD NOV

PY 2008

VL 10

IS 11

BP 1053

EP 1055

DI 10.1002/adem.200800115

UT WOS:000261933400013

ER

PT J

AU Fleury, E

Lee, SM

Ahn, HS

Kim, WT

Kim, DH

AF Fleury, E

Lee, SM

Ahn, HS

Kim, WT

Kim, DH

TI Tribological properties of bulk metallic glasses

SO MATERIALS SCIENCE AND ENGINEERING A-STRUCTURAL MATERIALS PROPERTIES

MICROSTRUCTURE AND PROCESSING

CT 11th International Conference on Rapidly Quenched and Metastable

Materials

CY AUG 25-30, 2002

CL Univ Oxford, Dept Mat, Oxford, ENGLAND

HO Univ Oxford, Dept Mat

AB The frictional and wear behavior of several bulk metallic glasses (BMGs) were investigated at room temperature under dry sliding condition. The influence of the type of motion on the tribological properties was of particular interest. Under identical sliding condition, the BGMs investigated in this study exhibited rather similar frictional behavior, with values ranging from 0.35 to 0.43. However, the present results indicated a significant difference in the wear performance. Surface modifications in the wear tracks were found to be dependent on the counterpart material and the nature of the sliding motion, and to contribute to the variations of the tribological properties of bulk metallic glasses. (C) 2003 Elsevier B.V. All rights reserved.

RI bang, changwook/J-7922-2012

SN 0921-5093

PD JUL 15

PY 2004

VL 375

SI SI

BP 276

EP 279

DI 10.1016/j.msea.2003.10.065

UT WOS:000223329700042

ER

PT J

AU Yang, B

Nieh, TG

AF Yang, B.

Nieh, T. G.

TI Effect of the nanoindentation rate on the shear band formation in an

Au-based bulk metallic glass

SO ACTA MATERIALIA

AB This study investigated the nanoindentation behavior of AU(49)Ag(5.5)Pd(2.3)CU(26.9)Si(16.3) bulk metallic glass samples at loading rates ranging from 0.03 to 300 mN s(-1). Notable shear band pop-in events were observed. The pop-in size was observed to increase linearly with the load and decreased exponentially with the strain rate. A free-volume mechanism was proposed for interpreting these observations quantitatively. The results and analyses also shed light on the shear band nucleation and evolution processes in bulk metallic glasses. (c) 2006 Acta Materialia Inc. Published by Elsevier Ltd. All rights reserved.

RI Nieh, Tai-Gang/G-5912-2011

OI Nieh, Tai-Gang/0000-0002-2814-3746

SN 1359-6454

EI 1873-2453

PD JAN

PY 2007

VL 55

IS 1

BP 295

EP 300

DI 10.1016/j.actamat.2006.08.028

UT WOS:000242928700027

ER

PT J

AU Egami, T

Levashov, V

Aga, R

Morris, JR

AF Egami, T.

Levashov, V.

Aga, R.

Morris, J. R.

TI Geometrical frustration and glass formation

SO METALLURGICAL AND MATERIALS TRANSACTIONS A-PHYSICAL METALLURGY AND

MATERIALS SCIENCE

CT 4th International Conference on Bulk Metallic Glasses

CY MAY 01-05, 2005

CL Gatlinburg, TN

AB The effect of geometrical frustration in the atomic structure on the formability of bulk metallic glasses is discussed from a general point of view. It is pointed out that there are two distinct and complementing pathways to easy glass formation: stabilizing the glass itself and destabilizing the corresponding crystalline state. While the discussions in the field tend to focus on the first one, the second in fact is a more effective approach. Examples of both will be discussed using soft-sphere, rather than hard-sphere, packing concepts.

RI Morris, J/I-4452-2012

OI Morris, J/0000-0002-8464-9047

SN 1073-5623

PD AUG

PY 2008

VL 39A

IS 8

BP 1786

EP 1790

DI 10.1007/s11661-008-9555-9

UT WOS:000256886600004

ER

PT J

AU Sun, BA

Pauly, S

Tan, J

Stoica, M

Wang, WH

Kuhn, U

Eckert, J

AF Sun, B. A.

Pauly, S.

Tan, J.

Stoica, M.

Wang, W. H.

Kuehn, U.

Eckert, J.

TI Serrated flow and stick-slip deformation dynamics in the presence of

shear band interaction in a Zr-based bulk metallic glass (vol 60, pg

4160, 2012)

SO ACTA MATERIALIA

RI Sun, Baoan/C-6441-2012; Stoica, Mihai/B-7069-2015; Tan, Jun/A-5291-2009

OI Sun, Baoan/0000-0001-5306-1817; Tan, Jun/0000-0003-0701-7449

SN 1359-6454

PD APR

PY 2013

VL 61

IS 6

BP 2281

EP 2281

DI 10.1016/j.actamat.2013.01.002

UT WOS:000316241400042

ER

PT J

AU Belhadi, L

Decremps, F

Pascarelli, S

Cormier, L

Le Godec, Y

Gorsse, S

Baudelet, F

Marini, C

Garbarino, G

AF Belhadi, L.

Decremps, F.

Pascarelli, S.

Cormier, L.

Le Godec, Y.

Gorsse, S.

Baudelet, F.

Marini, C.

Garbarino, G.

TI Polyamorphism in cerium based bulk metallic glasses: Electronic and

structural properties under pressure and temperature by x-ray absorption

techniques

SO APPLIED PHYSICS LETTERS

AB High pressure and high temperature x-ray absorption near edge spectroscopy experiments have been carried out on Ce60Al20Cu20 bulk metallic glass showing an electronic delocalization of the 4f-electron of cerium under pressure. In parallel, high pressure extended x-ray absorption fine structure spectroscopy reveals large structural modifications of the cerium local environment. This study provides experimental evidence that an electronic driven structural transformation occurs in cerium based bulk metallic glasses (Ce-BMGs). The effect of temperature on the hysteresis of this amorphous-amorphous phase transition is also discussed, suggesting the existence of a critical point in the phase diagram of Ce-BMGs. This work will encourage further investigations on Ce-based metallic glasses phase diagrams in order to support, or refute, the actual theoretical understanding of polyamorphism. (C) 2013 AIP Publishing LLC.

RI Garbarino, Gaston/D-1807-2013; Cormier, Laurent/D-8006-2018; Gorsse,

Stephane/F-5170-2017

OI Cormier, Laurent/0000-0002-3554-0707; Gorsse,

Stephane/0000-0003-1966-8476

SN 0003-6951

EI 1077-3118

PD SEP 9

PY 2013

VL 103

IS 11

AR 111905

DI 10.1063/1.4820434

UT WOS:000324495000026

ER

PT J

AU Yamamoto, T

Yodoshi, N

Bitoh, T

Makino, A

Inoue, A

AF Yamamoto, Tokujiro

Yodoshi, Norlharu

Bitoh, Teruo

Makino, Akihiro

Inoue, Akihisa

TI Soft magnetic Fe-based metallic glasses prepared by fluxing and

water-quenching

SO REVIEWS ON ADVANCED MATERIALS SCIENCE

CT 13th International Symposium on Metastable and Nano-Materials

(ISMANAM-2006)

CY AUG 27-31, 2006

CL Warsaw Univ Technol, Fac Mat Sci & Engn, Warsaw, POLAND

HO Warsaw Univ Technol, Fac Mat Sci & Engn

AB [(Fe(0.5)Co(0.5))(0.75)B(0.20)Si(0.05)](96)Nb(4) soft magnetic bulk metallic glasses were prepared by fluxing and water-quenching in a silica tube. Dimension of the bulk metallic glass specimens was up to 7.7 mm in diameter, which is about 1.5 times larger than those prepared by Cu mold-casting. The critical cooling rate of [(Fe(0.5)Co(0.5))(0.75)B(0.20)Si(0.05)](96)Nb(4) alloys with fluxing for forming a metallic glass phase was 150-170 K/s, which was considerably smaller than that without fluxing. Saturation magnetization was 1.13 T, and coercivity was lower than 20 A/m. Fluxing suppresses heterogeneous nucleation by isolating the nucleation sites from the molten alloys and improves their glass-forming ability.

RI MAKINO, AKIHIRO/B-2549-2009; Inoue, Akihisa/E-5271-2015; Yamamoto,

Tokujiro/A-8827-2011; Yodoshi, Noriharu/K-8072-2012

OI Yodoshi, Noriharu/0000-0003-2894-1788

SN 1606-5131

PD JUN

PY 2008

VL 18

IS 2

BP 126

EP 130

UT WOS:000257256400006

ER

PT J

AU Khonik, VA

Mitrofanov, YP

Makarov, AS

Konchakov, RA

Afonin, GV

Tsyplakov, AN

AF Khonik, V. A.

Mitrofanov, Yu. P.

Makarov, A. S.

Konchakov, R. A.

Afonin, G. V.

Tsyplakov, A. N.

TI Structural relaxation and shear softening of Pd- and Zr-based bulk

metallic glasses near the glass transition

SO JOURNAL OF ALLOYS AND COMPOUNDS

AB Precision measurements of the high-frequency shear modulus G and internal friction Q(-1) on bulk Pd- and Zr-based glasses upon thermocycling from room temperature up to the glass transition and back have been performed. Together with the pronounced shear softening and related increase of the internal friction upon approaching the glass transition temperature, a strong hysteresis of G and Q(-1) upon cooling has been found. It has been revealed that the shear softening can be quantitatively described as a result of the generation of interstitialcy defects near the glass transition. (C) 2014 Elsevier B.V. All rights reserved.

RI Tsyplakov, Alexander/M-6132-2013; Konchakov, Roman/K-4616-2013; Khonik,

Vitaly/A-5888-2009; Makarov, Andrey/H-4156-2013; Mitrofanov,

Yuriy/E-7963-2010; Afonin, Gennady/K-1014-2013

OI Tsyplakov, Alexander/0000-0002-2579-6362; Makarov,

Andrey/0000-0001-6741-0619; Mitrofanov, Yuriy/0000-0002-7939-5230;

Afonin, Gennady/0000-0002-7715-5065

SN 0925-8388

EI 1873-4669

PD APR 15

PY 2015

VL 628

BP 27

EP 31

DI 10.1016/j.jallcom.2014.12.095

UT WOS:000349084100004

ER

PT J

AU Arora, HS

Grewal, HS

Singh, H

Mukherjee, S

AF Arora, H. S.

Grewal, H. S.

Singh, H.

Mukherjee, S.

TI Zirconium based bulk metallic glass-Better resistance to slurry erosion

compared to hydroturbine steel

SO WEAR

AB The slurry erosion behavior of a zirconium based bulk metallic glass, Zr44Ti11Cu10Ni10Be25, was evaluated in this study. Slurry erosion tests were carried out using a non-circulating type test rig at impingement angles of 30 degrees, 60 degrees, and 90 degrees. Commonly used hydroturbine steel was evaluated under the same test conditions. At 30 degrees impingement angle, the metallic glass demonstrated nearly 2.6 times higher erosion resistance compared to the steel. At normal impingement, the metallic glass was marginally better. Lower erosion rates of the bulk metallic glass at oblique impingement angles was attributed to its significantly higher hardness and deformation induced partial devitrification. For normal impingement, material was removed in the form of fragments from highly strained platelets due to limited plasticity of bulk metallic glasses. The metallic glass demonstrated a brittle mode of erosion with higher erosion rate at higher angle of impingement. In contrast, hydroturbine steel showed ductile mode of erosion under the same test conditions. (C) 2013 Elsevier B.V. All rights reserved.

RI Mukherjee, Sundeep/N-5247-2014

OI Singh, Harpreet/0000-0002-6812-2667

SN 0043-1648

PD SEP 30

PY 2013

VL 307

IS 1-2

BP 28

EP 34

DI 10.1016/j.wear.2013.08.016

UT WOS:000328523800004

ER

PT J

AU Xu, BC

Xue, RJ

Zhang, B

AF Xu, B. C.

Xue, R. J.

Zhang, B.

TI Superior glass-forming ability and its correlation with density in

Ce-Ga-Cu ternary bulk metallic glasses

SO INTERMETALLICS

AB We report the formation and conventional density measurements of the ternary Ce70GaxCu30-x (at.%) glassy alloys in a wide composition range of 4 <= x <= 15. The best glassy formers with critical diameters of at least 10 mm, were found locating at the middle Ga composition range of 6 <= x <= 10, coinciding with the alloys having the highest density values. Our results provide a quantitative experimental evidence for the density dependent glass forming ability in a multicomponent metallic glass-forming system, which has been rarely reported before. The results facilitate a further precise study on the formation and structure in metallic glasses. (C) 2012 Elsevier Ltd. All rights reserved.

SN 0966-9795

PD JAN

PY 2013

VL 32

BP 1

EP 5

DI 10.1016/j.intermet.2012.09.015

UT WOS:000313145500001

ER

PT J

AU Fecht, H

Johnson, WL

AF Fecht, H

Johnson, WL

TI Thermodynamic properties and metastability of bulk metallic glasses

SO MATERIALS SCIENCE AND ENGINEERING A-STRUCTURAL MATERIALS PROPERTIES

MICROSTRUCTURE AND PROCESSING

CT 11th International Conference on Rapidly Quenched and Metastable

Materials

CY AUG 25-30, 2002

CL Univ Oxford, Dept Mat, Oxford, ENGLAND

HO Univ Oxford, Dept Mat

AB Glasses are formed if upon cooling of a melt below the glass transition temperature crystal nucleation and growth are avoided. Recently, multi-component alloys with deep eutectic temperatures have been developed which allow the formation of a metallic glass during slow cooling of the melt. These so-called bulk metallic glasses can be produced with several cubic centimeters (cm(3)) dimensions and have excellent properties making them suitable as a superior engineering material. Moreover, for these amorphous alloys crystallization can be avoided over a broad temperature-time window not accessible so far for metals. As such, the relevant thermodynamic properties of the metastable glassy and undercooled liquid states can be directly measured below and above the glass transition temperature, respectively. The obtained data gives new insight into the fundamental aspects regarding the stability of undercooled liquids and the nature of the glass transition. (C) 2003 Elsevier B.V. All rights reserved.

SN 0921-5093

PD JUL 15

PY 2004

VL 375

SI SI

BP 2

EP 8

DI 10.1016/j.msea.2003.10.254

UT WOS:000223329700002

ER

PT J

AU Wesseling, P

Nieh, TG

Wang, WH

Lewandowski, JJ

AF Wesseling, P

Nieh, TG

Wang, WH

Lewandowski, JJ

TI Preliminary assessment of flow, notch toughness, and high temperature

behavior of CU60Zr20Hf10Ti10 bulk metallic glass

SO SCRIPTA MATERIALIA

AB Microhardness, hot hardness, uniaxial compression, and notched bending experiments were conducted on Cu60Zr20Hf10Ti10 bulk metallic glass (BMG). This Cu-based BMG possesses near theoretical strength but essentially zero compressive ductility at room temperature. Notch toughness values in excess of 65 MParootm were obtained, while significant softening was obtained near T-q (C) 2004 Acta Materialia Inc. Published by Elsevier Ltd. All rights reserved.

RI Lewandowski, John/S-3815-2017; Nieh, Tai-Gang/G-5912-2011

OI Lewandowski, John/0000-0002-3389-2637; Nieh,

Tai-Gang/0000-0002-2814-3746

SN 1359-6462

PD JUL

PY 2004

VL 51

IS 2

BP 151

EP 154

DI 10.1016/j.scriptamat.2004.03.034

UT WOS:000221577300014

ER

PT J

AU Scudino, S

Surreddi, KB

Khoshkhoo, MS

Sakaliyska, M

Wang, G

Eckert, J

AF Scudino, Sergio

Surreddi, Kumar Babu

Khoshkhoo, Mohsen Samadi

Sakaliyska, Mira

Wang, Gang

Eckert, Juergen

TI Improved Room Temperature Plasticity of Zr41.2Ti13.8Cu12.5Ni10Be22.5

Bulk Metallic Glass by Channel-Die Compression

SO ADVANCED ENGINEERING MATERIALS

AB In this work, the effect of channel-die compression (CDC) on the mechanical behavior of the Zr41.2Ti13.8Cu12.5Ni10Be22.5 bulk metallic glass is analyzed. The results indicate that CDC can be successfully used as a pre-deformation process to effectively enhance the room temperature plastic strain ability of metallic glasses. The origin of the improved mechanical properties is most likely due to the creation during CDC of a heterogenous microstructure consisting of hard and soft regions able to hinder the rapid propagation of shear bands.

RI Surreddi, Kumar Babu/C-2454-2008; Scudino, Sergio/D-8049-2015

OI Surreddi, Kumar Babu/0000-0001-7938-9909;

SN 1438-1656

PD NOV

PY 2010

VL 12

IS 11

BP 1123

EP 1126

DI 10.1002/adem.201000171

UT WOS:000285210500013

ER

PT J

AU Jiang, WH

Fan, GJ

Liu, FX

Wang, GY

Choo, H

Liaw, PK

AF Jiang, W. H.

Fan, G. J.

Liu, F. X.

Wang, G. Y.

Choo, H.

Liaw, P. K.

TI Rate dependence of shear banding and serrated flows in a bulk metallic

glass

SO JOURNAL OF MATERIALS RESEARCH

AB Using an infrared camera, we observed in situ dynamic shear-banding operations during compression of a bulk metallic glass at various strain rates. We demonstrated that the shear-banding events are highly dependent on strain rates, either intermittent at the lower strain rate or successive at the higher strain rate. Serrated plastic-flow behaviors are a result of shear-banding operations. These observations provide a new insight into inhomogeneous deformation of metallic glasses.

RI Wang, Gongyao/C-4003-2011; Choo, Hahn/A-5494-2009

OI Choo, Hahn/0000-0002-8006-8907

SN 0884-2914

EI 2044-5326

PD SEP

PY 2006

VL 21

IS 9

BP 2164

EP 2167

DI 10.1557/JMR.2006.0266

UT WOS:000240578900001

ER

PT J

AU Wang, YM

Shek, CH

Wang, Q

Qiang, JB

Dong, CA

AF Wang, Yingmin

Shek, Chan Hung

Wang, Qing

Qiang, Jianbing

Dong, Chuang

TI TEM study of the cooling rate dependent crystallization behavior of

(Zr65Al10Ni10Cu15)(98)Nb-2 metallic glass

SO JOURNAL OF ALLOYS AND COMPOUNDS

CT 16th International Symposium on Metastable, Amorphous and Nanostructured

Materials

CY JUL 05-09, 2009

CL Beijing, PEOPLES R CHINA

AB The crystallization of (Zr65Al10Ni10Cu15)(98)Nb-2 metallic glasses has been studied using transmission electron microscopy (TEM), X-ray diffraction (XRD) and differential scanning calorimetry (DSC). The ribbon glass and bulk metallic glass (BMG) of this alloy exhibit different crystallization behaviors. For ribbon glass in the first stage crystallization, icosahedral quasicrystal (I-phase) precipitated together with the eta-Zr2Ni (a = 1.226 nm) phase. The BMG alloy transforms into the I-phase and two coherently coexisted phases, namely, the Al2Zr3 phase and an unknown primitive cubic phase (a = 0.76 nm) in this stage. The experimental evidence indicates that the liquid cooling rate for sample preparation has a significant effect on its crystallization behavior of this alloy glass. (C) 2010 Elsevier B.V. All rights reserved.

RI SHEK, Chan Hung/J-3857-2015

OI SHEK, Chan Hung/0000-0002-6870-523X

SN 0925-8388

PD AUG

PY 2010

VL 504

SU 1

BP S234

EP S238

DI 10.1016/j.jallcom.2010.03.162

UT WOS:000285252600060

ER

PT J

AU Hays, CC

Kim, CP

Johnson, WL

AF Hays, CC

Kim, CP

Johnson, WL

TI Large supercooled liquid region and phase separation in the

Zr-Ti-Ni-Cu-Be bulk metallic glasses

SO APPLIED PHYSICS LETTERS

AB Results of calorimetric, differential thermal analysis, and structural measurements are presented for a series of bulk metallic glass forming compositions in the Zr-Ti-Cu-Ni-Be alloy system. The calorimetric data for five alloys, prepared along the tie line between phase separating and nonphase separating compositions, show that the transition from phase separating to nonphase separating behavior is smooth. The bulk glasses near the center of the tie line exhibit large supercooled liquid regions: Delta T approximate to 135 K, the largest known for a bulk metallic glass. (C) 1999 American Institute of Physics. [S0003-6951(99)01734-9].

RI Hays, Charles/P-8021-2015

OI Hays, Charles/0000-0002-0420-1761

SN 0003-6951

PD AUG 23

PY 1999

VL 75

IS 8

BP 1089

EP 1091

DI 10.1063/1.124606

UT WOS:000082037500020

ER

PT J

AU Park, JM

Park, JS

Na, JH

Kim, DH

Kim, DH

AF Park, Jin Man

Park, Joon Sik

Na, Jong Hyun

Kim, Dong Ho

Kim, Do Hyang

TI Effect of Y addition on thermal stability and the glass forming ability

in Fe-Nb-B-Si bulk glassy alloy

SO MATERIALS SCIENCE AND ENGINEERING A-STRUCTURAL MATERIALS PROPERTIES

MICROSTRUCTURE AND PROCESSING

AB The effect of Y addition on the thermal stability and glass forming ability (GFA) has been investigated in (Fe72NbB20Si4)(100-x) Y-x (x=0-5) alloys. Addition of 3-5 at.% Y improves the GFA. With increasing the Y content, glass transition temperature (T-g) and crystallization temperature (T-x) shifted to the higher temperature region. The (FC72Nb4B20Si4)(97)Y-3 alloy shows a high thermal stability, with T-g of 859 K and the supercooled liquid regions reached maximum value of 56 K. The (Fe72Nb4B20Si4)(97)Y-3 alloy exhibits the largest gamma (=0.402) value among the alloy investigated, enabling fabrication of bulk metallic glass (BMG) sample with the diameter of 4 mm. (c) 2006 Elsevier B.V. All rights reserved.

RI bang, changwook/J-7922-2012

SN 0921-5093

PD NOV 5

PY 2006

VL 435

BP 425

EP 428

DI 10.1016/j.msea.2006.07.073

UT WOS:000241683200055

ER

PT J

AU Pauly, S

Das, J

Duhamel, C

Eckert, J

AF Pauly, Simon

Das, Jayanta

Duhamel, Cecile

Eckert, Juergen

TI Martensite formation in a ductile Cu47.5Zr47.5Al5 bulk metallic glass

composite

SO ADVANCED ENGINEERING MATERIALS

RI Duhamel, Cecilie/B-5106-2013; Das, Jayanta/G-1559-2010

OI Das, Jayanta/0000-0001-8750-5463

SN 1438-1656

PD JUN

PY 2007

VL 9

IS 6

BP 487

EP 491

DI 10.1002/adem.200700044

UT WOS:000247885800013

ER

PT J

AU Lai, YH

Chen, HM

Lee, CJ

Huang, JC

Jang, JSC

AF Lai, Y. H.

Chen, H. M.

Lee, C. J.

Huang, J. C.

Jang, J. S. C.

TI Strain burst speeds in metallic glass micropillars

SO INTERMETALLICS

CT 7th International Conference on Bulk-Metallic Glasses

CY NOV 01-05, 2009

CL Busan, SOUTH KOREA

SP Yonsei Univ, Ctr Noncrystalline Mat

AB Uniaxial microcompression and nanoscratch tests using the nanoindentation system on the Mg-, Au- and Zr-based metallic glass micropillars with diameters of 3.8 and 1 mu m were performed and compared. Strain burst phenomena were observed in all microcompression tests as indications of localized shearing. The strain burst speed of micropillars can be measured based on the raw displacement-time profile. The results indicate that strain burst speed of metallic glasses increases with increasing sample size, with decreasing wear resistance, and with decreasing ductility. The current study demonstrates that strain burst speed and wear characteristics can be regarded as promising indicators for the ductility of metallic glasses. (C) 2010 Elsevier Ltd. All rights reserved.

RI Huang, J./C-4276-2013

OI HUANG, Jacob Chih Ching/0000-0001-6843-3396

SN 0966-9795

PD OCT

PY 2010

VL 18

IS 10

SI SI

BP 1893

EP 1897

DI 10.1016/j.intermet.2010.02.037

UT WOS:000281420700025

ER

PT S

AU Bakke, E

Busch, R

Johnson, WL

AF Bakke, E

Busch, R

Johnson, WL

BE Schulz, R

TI Viscosity measurements of the Zr46.75Ti8.25Cu7.5Ni10Be27.5 bulk metallic

glass forming alloy using parallel plate rheometry and beam-bending

SO METASTABLE, MECHANICALLY ALLOYED AND NANOCRYSTALLINE MATERIALS, PTS 1

AND 2

SE MATERIALS SCIENCE FORUM

CT International Symposium on Metastable, Mechanically Alloyed and

Nanocrystalline Materials (ISMANAM-95)

CY JUL 24-28, 1995

CL QUEBEC CITY, CANADA

SP Hydro Quebec, Laval Univ, McGill Univ, Ctr Phys Mat, Nat Sci & Engn Res Council Canada, Zoz GmbH, Siemens, Xerox Canada Ltee, Precitech

AB Parallel plate rheometry and three-point beam-bending were used to measure the viscosity as a function of temperature of a bulk metallic glass, Zr46.75Ti8.25Cu7.5Ni10Be27.5 The high thermal stability above the glass transition of this bulk metallic glass former with respect to crystallization allows measuring viscosities 120 K into the supercooled liquid region. Viscosity in the range from 10(10) to 10(6) poise has been measured using parallel plate rheometry, a region of viscosities that has not been previously accessible for supercooled metallic melts. The measurements were carried out with different heating rates between 0.0167 K/s and 1.167 K/s as well as isothermally. Using three-point beam bending, viscosity in the range from 10(13) to 10(8) poise has also been measured. These two methods, which involve completely different geometries for the measurement of flow, yielded consistent values for viscosity where their applicable regions overlap. The viscosity of the supercooled liquid of this bulk glass former, above the glass transition temperature, exhibits a small Vogel-Fulcher temperature relative to the glass transition temperature similar to silicate glasses.

SN 0255-5476

BN 0-87849-738-2

PY 1996

VL 225

BP 95

EP 100

DI 10.4028/www.scientific.net/MSF.225-227.95

UT WOS:A1996BG37Y00013

ER

PT B

AU Liu, CT

Greer, AL

Schuh, CA

AF Liu, C. T.

Greer, A. Lindsay

Schuh, Christopher A.

BA Suryanarayana, C

Inoue, A

BF Suryanarayana, C

Inoue, A

TI Physical Properties

SO BULK METALLIC GLASSES

BN 978-1-4200-8596-9

PY 2011

BP 265

EP 306

D2 10.1201/9781420085976-1

UT WOS:000288438000007

ER

PT J

AU Meng, QG

Zhang, SG

Li, JG

Bian, XF

AF Meng, Q. G.

Zhang, S. G.

Li, J. G.

Bian, X. F.

TI Dilatometric measurements and glass-forming ability in Pr-based bulk

metallic glasses

SO SCRIPTA MATERIALIA

AB Dilatometric measurements were conducted to obtain the average thermal expansion coefficients (alpha(aver)) of a series of Pr-based bulk metallic glasses at various experimental conditions. A correlation was found between alpha(aver) and the weighted average of the thermal expansion coefficients for the constituent elements. By assuming Lennard-Jones type potentials, the average nearest-neighbor distances (r(1)) and the depths of effective pair potentials (V(0)) were calculated. The values of r(1) agreed well with the experimental results and V(0) correlated with the glass-forming ability. (c) 2006 Acta Materialia Inc. Published by Elsevier Ltd. All rights reserved.

SN 1359-6462

PD SEP

PY 2006

VL 55

IS 6

BP 517

EP 520

DI 10.1016/j.scriptamat.2006.05.036

UT WOS:000239413400005

ER

PT J

AU Pineda, E

Zhang, Y

Greer, AL

AF Pineda, Eloi

Zhang, Yi

Greer, A. Lindsay

TI Fragility and glass-forming ability of the Ca-Mg-Cu system

SO JOURNAL OF ALLOYS AND COMPOUNDS

CT 12th International Symposium on Metastable and Nano-Materials

(ISMANAM-2005)

CY JUL 03-07, 2005

CL Paris, FRANCE

AB Elastic moduli of the glass and fragility of the corresponding supercooled liquid were found to be correlated in recently published results. In the present work, compositions within the Ca-Mg-Cu system are used to study the relationships between liquid fragility, glass-forming ability and elastic properties. The correlation between liquid fragility and elastic properties already observed for glasses in general is found to hold in this particular system. More extensive work should be done in order to obtain conclusive results. However, if such a correlation turns out to be general in metallic systems, it will be a powerful criterion in choosing candidate compositions for new bulk metallic glasses. (C) 2006 Elsevier B.V. All rights reserved.

RI Pineda, Eloi/H-9081-2013; Greer, Alan Lindsay/G-1977-2011; Greer,

Lindsay/E-9433-2017

OI Pineda, Eloi/0000-0002-1871-3848;

SN 0925-8388

PD MAY 31

PY 2007

VL 434

SI SI

BP 145

EP 148

DI 10.1016/j.jallcom.2006.08.164

UT WOS:000246286900037

ER

PT J

AU Mattern, N

Kuhn, U

Concustell, A

Schops, A

Baro, MD

Eckert, J

AF Mattern, N.

Kuehn, U.

Concustell, A.

Schoeps, A.

Baro, M. D.

Eckert, J.

TI Phase separation and crystallization in Cu-Zr metallic glasses

SO MATERIALS TRANSACTIONS

CT 5th International Conference on Bulk Metallic Glasses

CY OCT 01-05, 2006

CL Osaka Univ, Awaji Isl, JAPAN

SP Minist Educ, Culture, Sports, Sci & Technol, Inst Mat Res, Tohoku Univ, Japan Soc Promot Sci, Natl Inst Mat Sci, Hyogo Int Assoc

HO Osaka Univ

AB The structural behavior of rapidly quenched Cu-Zr amorphous alloys was analyzed. High energy X-ray diffraction patterns and atomic pair correlation functions exhibit monotonic changes with composition. The experimental results can be well described by a solid solution-like replacement of Cu and Zr atoms in the whole composition range. No indications are observed that Would support the existence of phase separation in the supercooled liquid state of the binary Cu-Zr alloys. For Cu60Zr30Ti10 and Cu60Zr30Ti20 bulk metallic glasses the formation of ultrafine nanostructures are proven upon heating. The transformation starts below the glass transition temperature. Phase separation in Cu-Zr-Ti bulk metallic glasses is related to primary crystallization.

RI Baro, Maria Dolors/A-2096-2009

OI Baro, Maria Dolors/0000-0002-8636-1063; Concustell,

Amadeu/0000-0001-6705-3228

SN 1345-9678

EI 1347-5320

PD JUL

PY 2007

VL 48

IS 7

BP 1639

EP 1643

DI 10.2320/matertrans.MJ200708

UT WOS:000248743100014

ER

PT J

AU Deng, YF

He, LL

Zhang, QS

Zhang, HF

Ye, HQ

AF Deng, YF

He, LL

Zhang, QS

Zhang, HF

Ye, HQ

TI Deformation-induced structural changes in bulk metallic glass at room

temperature

SO ADVANCED ENGINEERING MATERIALS

RI zhang, qingsheng/A-4851-2010

SN 1438-1656

PD OCT

PY 2003

VL 5

IS 10

BP 738

EP 741

DI 10.1002/adem.200300383

UT WOS:000186505300018

ER

PT J

AU Hermann, H

Elsner, A

Lochmann, K

Stoyan, D

AF Hermann, Helmut

Elsner, Antje

Lochmann, Kristin

Stoyan, Dietrich

TI Optimisation of multi-component hard sphere liquids with respect to

dense packing

SO MATERIALS SCIENCE AND ENGINEERING A-STRUCTURAL MATERIALS PROPERTIES

MICROSTRUCTURE AND PROCESSING

CT 12th International Conference on Rapidly Quenched and Metastable

Materials

CY AUG 21-26, 2005

CL Cheju Isl, SOUTH KOREA

AB The generalised Bernal model for liquids is used as an approach to the structure of multi-component liquid and amorphous metallic alloys. It is assumed that alloys that can be described by random close packing models of hard spheres with particular high packing fraction may have a better glass forming ability than those with lower ones. We show that the optimisation of computer simulated random close packing models is a promising way to propose models with enhanced packing fraction and to specify the compositions and radii distributions required. A series of multi-component systems with enhanced packing fraction is presented and proposed as candidates for new bulk metallic glasses. (c) 2006 Elsevier B.V. All rights reserved.

SN 0921-5093

PD MAR 25

PY 2007

VL 449

BP 666

EP 670

DI 10.1016/j.msea.2005.12.106

UT WOS:000245477800152

ER

PT J

AU Zhao, K

Jiao, W

Ma, J

Gao, XQ

Wang, WH

AF Zhao, Kun

Jiao, Wei

Ma, Jiang

Gao, Xuan Qiao

Wang, Wei Hua

TI Formation and properties of strontium-based bulk metallic glasses with

ultralow glass transition temperature

SO JOURNAL OF MATERIALS RESEARCH

AB We report a family of novel Strontium (Sr)-based bulk metallic glasses (BMGs) with good glass-forming ability and ultralow glass transition temperature (T-g) by strategic composition design. The Sr-based BMGs can be easily formed with wide composition range by a conventional copper mold cast method. The glassy alloys have many unique and diversified properties such as lowest glass transition temperature, ultralow elastic modulus, small value of Poisson's ratio and fragility, homogeneous flow at room temperature and tunable water degradation behavior. The BMGs with novel physical and chemical properties could have potential applications for biomaterial and micromanufacture, and are model system for studying some fundamental issues such as crystallization, relaxation and deformation in metallic glass.

RI wei, jiao/I-7244-2013

SN 0884-2914

EI 2044-5326

PD OCT

PY 2012

VL 27

IS 20

BP 2593

EP 2600

DI 10.1557/jmr.2012.214

UT WOS:000309728300003

ER

PT S

AU Terajima, T

AF Terajima, Takeshi

BE Chandra, T

Ionescu, M

Mantovani, D

TI Development of Cu-Clad Metallic Glass for Soldering

SO THERMEC 2011, PTS 1-4

SE Materials Science Forum

CT 7th International Conference on Processing and Manufacturing of Advanced

Materials

CY AUG 01-05, 2011

CL Quebec City, CANADA

SP Minerals, Metals & Mat Soc

AB Soldering is a potential technique for joining metallic glasses. It can be performed at far below the crystallization temperature of various metallic glasses; thus, there is no possibility of crystallization. However, Cu-Zr-based metallic glass displays poor wettability to Pb-free solder, because a strong native oxide film prevents direct contact between the solder and the glass. To overcome this problem, Cu-Zr-based metallic glass clad with a thin film of Cu has been developed. This was produced by casting the melt of a Cu36Zr48Al8Ag8 pre-alloy into a Cu mold cavity, inside which a thin film of Cu with a thickness of 2 mu m was placed. Cu36Zr48Al8Ag8 metallic glass was successfully formed and welded to the Cu thin film. From microstructure analysis, it was found that a reaction layer was formed at the interface between the Cu and the Cu36Zr48Al8Ag8 metallic glass. However, no oxide layer was observed in the Cu-clad layer. It was found that the Cu cladding played an important role in preventing the formation of the surface oxide film. Consequently, solderability to the Cu-Zr-based metallic glass was drastically improved.

SN 0255-5476

PY 2012

VL 706-709

BP 1343

EP 1347

DI 10.4028/www.scientific.net/MSF.706-709.1343

UT WOS:000308517300220

ER

PT J

AU Guo, SF

Liu, L

Li, N

Li, Y

AF Guo, S. F.

Liu, L.

Li, N.

Li, Y.

TI Fe-based bulk metallic glass matrix composite with large plasticity

SO SCRIPTA MATERIALIA

AB Using alloy design, an Fe77Mo5P9C7 B-5(1) (5) bulk metallic glass matrix composite reinforced by in situ formed ductile alpha-Fe dendrites has been developed In contrast to the monolithic Fe-based bulk metallic glasses that usually Fail in brittle fracture, the resulting composite shows a significant plastic strain of more than 30% as Well as a high fracture strength of over 3 0 GPa. The in Situ formation of the ductile alpha-Fe dendrites accounts for the significant enhancement of the plasticity (C) 2009 Acta Materialia Inc Published by Elsevier Ltd All rights reserved

RI Guo, Shengfeng/E-3171-2012; Li, Ning/B-2283-2009

OI Guo, Shengfeng/0000-0002-6667-6797;

SN 1359-6462

PD MAR

PY 2010

VL 62

IS 6

BP 329

EP 332

DI 10.1016/j.scriptamat.2009.10.024

UT WOS:000275072700002

ER

PT J

AU Guo, S

Liu, CT

AF Guo, Sheng

Liu, C. T.

TI New glass forming ability criterion derived from cooling consideration

SO INTERMETALLICS

AB Currently existing expressions of glass forming ability (GFA) have been formulated based on the characteristic temperatures measured from heating of metallic glasses However, these GFA expressions are not acceptable physically as GFA is defined from cooling of the molten liquid rather than heating of the solid glasses In consideration of the relationship between the cooling and heating processes, we have derived a new GFA criterion. gamma(c)=(3T(x)-2Tg)/T(I)(T(x) onset crystallization temperature; T(g) glass transition temperature, T(I) liquidus temperature). This criterion correlates very well to the critical cooling rate and agrees exceptionally well with the physically acceptable boundary condition. (C) 2010 Elsevier Ltd All rights reserved.

RI Guo, Sheng/C-7746-2009

OI Guo, Sheng/0000-0001-8349-3135; Liu, Chain Tsuan/0000-0001-7888-9725

SN 0966-9795

PD NOV

PY 2010

VL 18

IS 11

BP 2065

EP 2068

DI 10.1016/j.intermet.2010.06.012

UT WOS:000282710400008

ER

PT J

AU Liu, JT

Guo, J

Hu, XF

Guo, SA

Meng, WJ

Xiao, QF

AF Liu, Jingtao

Guo, Jing

Hu, Xuefei

Guo, Shuai

Meng, Weijiang

Xiao, Qingfeng

TI Effect of melt superheated treatment on glass forming ability and

thermal expansion of Gd55Al25Cu10Co10 alloys

SO JOURNAL OF ALLOYS AND COMPOUNDS

AB Glass forming ability and thermal expansion behavior of nominal compositions Gd55Al25Cu10Co10 bulk metallic glasses (BMGs) at different melt superheated temperatures were investigated using differential scanning calorimetry (DSC), X-ray diffractometry (XRD), high resolution transmission electron microscopy (HRTEM) and thermal dilatometer. It was found that the glass forming ability gradually increased with increasing melt superheated temperature and reached the maximum at 1105 degrees C. In addition, the study on thermal expansion behavior revealed that the average thermal expansion coefficient decreased with increasing melt superheated temperature and at 1105 degrees C the alloy exhibited the minimal thermal expansion coefficient. (c) 2013 Elsevier B.V. All rights reserved.

SN 0925-8388

PD DEC 25

PY 2013

VL 581

BP 671

EP 674

DI 10.1016/j.jallcom.2013.07.077

UT WOS:000324823000112

ER

PT J

AU Zhao, L

Zhang, ZQ

Zhang, J

Pang, SJ

Ma, CL

Zhang, T

AF Zhao, Lei

Zhang, Zeqiang

Zhang, Jing

Pang, Shujie

Ma, Chaoli

Zhang, Tao

TI COMPOSITION DESIGN AND GLASS-FORMING ABILITY OF TI-BASED BULK METALLIC

GLASSES

SO INTERNATIONAL JOURNAL OF MODERN PHYSICS B

CT 5th International Conference on Advanced Materials Development and

Performance

CY OCT 12-15, 2008

CL Beijing, PEOPLES R CHINA

SP Tokushima Univ, Kyungpook Natl Univ, Beihang Univ, Univ Auckland

AB Ti-based bulk metallic glasses (BMGs) have great potential for use as structural materials due to their excellent mechanical properties. How to fabricate Ti-based metallic glasses with sufficient large size has always been the key issue in both the theoretic and technology aspects since finding the BMG. In this paper, we try to fabricate Ti-based metallic glasses with high glass-forming ability (GFA) through composition design based on various empirical ideas. BMG alloy with composition of Ti(43.89)Cu(43.60)Zr(6.75)Ni(5.76) was synthesized based on the deep eutectic idea, and the critical diameter of this glassy alloy reaches to 2 mm. The GFA of this alloy was further improved by small addition of Si (0.5-1 at %). BMG rods with diameters up to 3mm were synthesized for compositions of Ti(42.89)Cu(43.60)Zr(6.75)Ni(5.76)Si(1), Ti(43.89)Cu(43.60)Zr(6.25)Ni(5.76)Si(0.5) and Ti(43.89)Cu(43.60)Zr(6.75)Ni(4.76)Si(1). The amorphous structure of all the samples was confirmed by X-ray diffraction (XRD) method. The thermal stability associated with glass transition, supercooled liquid region and crystallization was investigated by differential scanning calorimetry (DSC). Possible mechanisms for the improvement of GFA were discussed.

RI Zhang, Tao/O-4911-2014; Pang, Shujie/D-8305-2016

SN 0217-9792

PD JUN 30

PY 2010

VL 24

IS 15-16

SI SI

BP 2326

EP 2331

DI 10.1142/S0217979210064873

UT WOS:000282197000017

ER

PT J

AU Han, Z

Yang, H

Wu, WF

Li, Y

AF Han, Z.

Yang, H.

Wu, W. F.

Li, Y.

TI Invariant critical stress for shear banding in a bulk metallic glass

SO APPLIED PHYSICS LETTERS

AB Through uncovering the macroscopic true stress after correction with the instant load-bearing area, we found that the critical stress for continuous shear banding of a bulk metallic glass (BMG) maintains invariant on and after yielding as the plastic deformation progresses. This finding is in strong contrast with the previous reports of either "strain hardening" or "strain softening" associated with the plastic deformation of BMGs. Our results point out that atomic cohesive energy constantly serves to be the controlling factor of the critical stress for shear banding.

SN 0003-6951

EI 1077-3118

PD DEC 8

PY 2008

VL 93

IS 23

AR 231912

DI 10.1063/1.3048869

UT WOS:000261699700032

ER

PT J

AU Rouxel, T

Ji, H

Hammouda, T

Moreac, A

AF Rouxel, T.

Ji, H.

Hammouda, T.

Moreac, A.

TI Poisson's ratio and the densification of glass under high pressure

SO PHYSICAL REVIEW LETTERS

AB Because of a relatively low atomic packing density, (C(g)) glasses experience significant densification under high hydrostatic pressure. Poisson's ratio (nu) is correlated to C(g) and typically varies from 0.15 for glasses with low C(g) such as amorphous silica to 0.38 for close-packed atomic networks such as in bulk metallic glasses. Pressure experiments were conducted up to 25 GPa at 293 K on silica, soda-lime-silica, chalcogenide, and bulk metallic glasses. We show from these high-pressure data that there is a direct correlation between nu and the maximum post-decompression density change.

RI Moreac, Alain/O-1631-2014

SN 0031-9007

PD JUN 6

PY 2008

VL 100

IS 22

AR 225501

DI 10.1103/PhysRevLett.100.225501

UT WOS:000256528400024

PM 18643428

ER

PT J

AU Wall, JJ

Weber, R

Kim, J

Liaw, PK

Choo, H

AF Wall, J. J.

Weber, R.

Kim, J.

Liaw, P. K.

Choo, H.

TI Aerodynamic levitation processing of a Zr-based bulk metallic glass

SO MATERIALS SCIENCE AND ENGINEERING A-STRUCTURAL MATERIALS PROPERTIES

MICROSTRUCTURE AND PROCESSING

AB Containerless aerodynamic levitation was used to produce the bulk metallic glass Zr57Ti5Ni8CU20Al10. Samples were processed from the liquid state at cooling rates in the range of 69-1.2 K/s. The alloy was found to vitrify at cooling rates from 69 to 11 K/s and recalesced at the slower cooling rates. X-ray diffraction confirmed that the specimens processed faster than the critical cooling rate were primarily amorphous. The processing method and its applicability to other systems are discussed. (c) 2006 Elsevier B.V. All rights reserved.

RI Choo, Hahn/A-5494-2009

OI Choo, Hahn/0000-0002-8006-8907

SN 0921-5093

PD FEB 15

PY 2007

VL 445

BP 219

EP 222

DI 10.1016/j.msea.2006.09.014

UT WOS:000244301500028

ER

PT J

AU Sergueeva, AV

Mara, NA

Kuntz, JD

Lavernia, EJ

Mukherjee, AK

AF Sergueeva, AV

Mara, NA

Kuntz, JD

Lavernia, EJ

Mukherjee, AK

TI Shear band formation and ductility in bulk metallic glass

SO PHILOSOPHICAL MAGAZINE

AB The variations in the chemical compositions of the metallic glasses reported in the literature, as well as the overall lack of experimental data concerning the inhomogeneous deformation behaviour of metallic glass, make the evaluation of the effects of shear band/fracture behaviour on the mechanical properties of metallic glasses difficult. Isolating the effect of local shear band formation on bulk inhomogeneous flow would appear to be a first step in approaching this problem. The mechanical behaviour of Vitreloy metallic glass at room temperature and at various strain rates in tension and compression was investigated. The formation of multiple shear bands was observed at high strain rates. An increase in strain rate leads to enhanced ductility in tension and compression. Some aspects of the deformation processes in tension and compression are discussed.

RI Lavernia, Enrique/I-6472-2013; Mara, Nathan/J-4509-2014

OI Lavernia, Enrique/0000-0003-2124-8964; Mara, Nathan/0000-0002-9135-4693

SN 1478-6435

PD AUG 11

PY 2005

VL 85

IS 23

BP 2671

EP 2687

DI 10.1080/14786430500157059

UT WOS:000230821600007

ER

PT J

AU Prajapati, SR

Kasyap, S

Pratap, A

AF Prajapati, Sonal R.

Kasyap, Supriya

Pratap, Arun

TI Effect of driving force of crystallization on critical cooling rate for

Pd-based metallic glasses

SO JOURNAL OF THERMAL ANALYSIS AND CALORIMETRY

AB The advent of bulk metallic glasses (BMG) has opened lot of scope of wide range of applications for this class of amorphous materials. Thermodynamics plays a very important role in glass formation in multicomponent metallic alloys. BMG's can be synthesized with relatively lower cooling rate with ease now. However, the glass formation in these systems seems to depend on quite a few parameters like enthalpy of melting, reduced glass transition temperature, under cooling. In present paper, we have studied the glass forming ability of Pd-based metallic glasses using theoretically determined Gibbs free energy difference (Delta G), between the supercooled liquid and the corresponding crystalline phase, and the critical cooling rate (R (c)). Time-temperature-transformation (TTT) diagrams were constructed to calculate R (c) using Uhlmann and Davies formulation. Different theoretical expressions of Delta G are incorporated in nucleation and growth equations to find R (c) from TTT diagram. The results obtained theoretically by Dhurandhar et al. expression of Delta G, assuming hyperbolic variation of specific heat difference (Delta C (p)), were found to be in excellent agreement with experimental data for different Pd-based systems.

SN 1388-6150

EI 1588-2926

PD MAR

PY 2017

VL 127

IS 3

BP 2083

EP 2091

DI 10.1007/s10973-016-5824-9

UT WOS:000395082300022

ER

PT J

AU Hao, GJ

Lin, JP

Zhang, Y

Chen, GL

Lu, ZP

AF Hao, G. J.

Lin, J. P.

Zhang, Y.

Chen, G. L.

Lu, Z. P.

TI Ti-Zr-Be ternary bulk metallic glasses correlated with binary eutectic

clusters

SO MATERIALS SCIENCE AND ENGINEERING A-STRUCTURAL MATERIALS PROPERTIES

MICROSTRUCTURE AND PROCESSING

AB In this paper, the reported scheme for locating good glass formers based on the concept of binary eutectic clusters is applied to predict alloy compositions with high glass-forming ability (GFA) in the Be-Ti-Zr system. Around the predicted composition, a series of novel bulk metallic glasses with high GFA, high strength and large compressive plastic strain of up to 15% was successfully developed. In particular, the specific strength of these newly developed BMGs reaches up to 450 kN m/kg and the supercooled liquid region is as high as 118 K, indicating a great potential to be used as aerospace materials. Crown Copyright (C) 2010 Published by Elsevier B.V. All rights reserved.

RI ZHANG, Yong/B-7928-2009; Lu, Zhao-Ping/A-2718-2009; Lin,

Junpin/D-1468-2013

OI ZHANG, Yong/0000-0002-6355-9923; Lu, Zhao-Ping/0000-0003-1463-8948;

SN 0921-5093

PD SEP 15

PY 2010

VL 527

IS 23

BP 6248

EP 6250

DI 10.1016/j.msea.2010.06.078

UT WOS:000281264000020

ER

PT J

AU Madge, SV

Sharma, P

Louzguine-Luzgin, DV

Greer, AL

Inoue, A

AF Madge, Shantanu V.

Sharma, Parmanand

Louzguine-Luzgin, Dmitri V.

Greer, A. Lindsay

Inoue, Akihisa

TI New La-based glass-crystal ex situ composites with enhanced toughness

SO SCRIPTA MATERIALIA

AB Ex situ composites have been synthesized by reinforcing the brittle La(55)Al(25)Cu(10)Ni(10) bulk metallic glass with varying volume fractions of spherical Ti particles. Ti is effective in significantly enhancing toughness, and optimum properties are obtained with just 20 vol.% Ti, where the compressive failure strain reaches 15% while maintaining a high strength (700 MPa). The combination of properties achieved appears to be the best among all known La-based glassy composites, which is explained in terms of the toughness of the reinforcement used. (C) 2009 Acta Materialia Inc. Published by Elsevier Ltd. All rights reserved.

RI Greer, Lindsay/E-9433-2017; LOUZGUINE, Dmitri/D-2492-2010; Sharma,

Parmanand/C-1518-2011; Inoue, Akihisa/E-5271-2015; Greer, Alan

Lindsay/G-1977-2011

OI LOUZGUINE, Dmitri/0000-0001-5716-4987; Madge,

Shantanu/0000-0001-7996-8652

SN 1359-6462

PD FEB

PY 2010

VL 62

IS 4

BP 210

EP 213

DI 10.1016/j.scriptamat.2009.10.029

UT WOS:000273067500011

ER

PT J

AU Keryvin, V

Hoang, VH

Shen, J

AF Keryvin, V.

Hoang, V. H.

Shen, J.

TI Hardness, toughness, brittleness and cracking systems in an iron-biased

bulk metallic glass by indentation

SO INTERMETALLICS

CT 6th International Conference on Bulk-Metallic Glasses (BMG-VI)

CY MAY 11-15, 2008

CL Xian, PEOPLES R CHINA

AB The mechanical behaviour of an FeCoCrMoCBY bulk metallic glass is investigated by means of indentation testing and ultrasonic echography. The elastic moduli (Young's modulus of 225 GPa, Poisson's ratio of 0.337), hardness (13.5 GPa), indentation toughness (2.2 MPa root m) and indentation brittleness (6 mu m(-1/2)) are determined. Unlike most metallic glasses, this iron-based alloy exhibits cracking features above a critical load. The cracking systems are observed by means of stepwise polishing and consist in very shallow Palmquist (or radial) cracks, which is quite uncommon in other brittle materials such as glasses and ceramics. (C) 2008 Elsevier Ltd. All rights reserved.

RI keryvin, vincent/F-1279-2010

SN 0966-9795

PD APR

PY 2009

VL 17

IS 4

BP 211

EP 217

DI 10.1016/j.intermet.2008.08.017

UT WOS:000264728400007

ER

PT J

AU Kim, JY

Jang, DC

Greer, JR

AF Kim, Ju-Young

Jang, Dongchan

Greer, Julia R.

TI Nanolaminates Utilizing Size-Dependent Homogeneous Plasticity of

Metallic Glasses

SO ADVANCED FUNCTIONAL MATERIALS

AB Homogeneous plasticity in metallic glasses is generally only observed at high temperatures or in very small structures (less than approximate to 100 nm), so their applications for structural performance have been very limited. Here, nanolaminates with alternating layers of Cu50Zr50 metallic glass and nanocrystalline Cu are synthesized and it is found that samples with an optimal composition of 112-nm-thick metallic-glass layers and 16-nm-thick Cu layers demonstrate a maximum strength of 2.513 GPa, a value 33% greater than that predicted by the rule-of-mixtures and 25% better than that of pure Cu50Zr50 metallic glass. Furthermore, approximate to 4% strain at fracture is achieved, suppressing the instantaneous catastrophic failure often associated with metallic glasses. It is postulated that this favorable combination of high strength and deformability is caused by the size-dependent deformation-mode transition in metallic glasses, from highly localized plasticity, leading to immediate failure in larger samples to homogeneous extension in the smaller ones.

RI Jang, Dongchan/C-9510-2012

OI Jang, Dongchan/0000-0002-2814-9734

SN 1616-301X

PD DEC 6

PY 2011

VL 21

IS 23

BP 4550

EP 4554

DI 10.1002/adfm.201101164

UT WOS:000297501000020

ER

PT J

AU Kozmidis-Petrovic, A

Sestak, J

AF Kozmidis-Petrovic, Ana

Sestak, Jaroslav

TI Forty years of the Hruby glass-forming coefficient via DTA when

comparing other criteria in relation to the glass stability and

vitrification ability

SO JOURNAL OF THERMAL ANALYSIS AND CALORIMETRY

CT Symposium on Applications of Thermal Analysis and Calorimetry - Workshop

and Exhibition (SATAC) 11th National Convention of Chemistry Teachers,

India

CY OCT 15-17, 2011

CL undefined, INDIA

AB The revision of the meaning of the famous Hruby glass-forming coefficient (as well as of other analogous coefficients by, e.g., Weinberg and Lu-Liu) reveals some generalized correlations between glass-forming ability (GFA) and glass stability. The relative change of the Hruby parameter is supreme in almost all cases. The Hruby parameter is more sensitive in relation to the change of both the super-cooled region and the reduced glass-transition temperature. The only exception is the restricted sensitivity respecting the reciprocal reduced glass-transition temperature in some cases of the bulk metallic glasses. The correlation of the Hruby coefficient with GFA is agreeable for oxide glasses and thus can be commonly employed as a reliable and precise glass-forming criterion. Associated problems are the experimental determination of relevant temperatures, most pertinent that for glass transition which is dependent to preparative condition of glass formation.

RI Sestak, Jaroslav/H-3025-2014

SN 1388-6150

EI 1588-2926

PD NOV

PY 2012

VL 110

IS 2

BP 997

EP 1004

DI 10.1007/s10973-011-1926-6

UT WOS:000310543000064

ER

PT B

AU Sharma, P

Inoue, A

AF Sharma, Parmanand

Inoue, Akihisa

BA Lindroos, V

Tilli, M

Lehto, A

Motooka, T

BF Lindroos, V

Tilli, M

Lehto, A

Motooka, T

TI Metallic Glass

SO HANDBOOK OF SILICON BASED MEMS MATERIALS AND TECHNOLOGIES

SE Micro & Nano Technologies

BN 978-0-81-551988-1; 978-0-81-551594-4

PY 2010

BP 447

EP 472

DI 10.1016/B978-0-8155-1594-4.00027-9

UT WOS:000311112400029

ER

PT J

AU Li, N

Liu, L

Chen, Q

Pan, J

Chan, KC

AF Li, N.

Liu, L.

Chen, Q.

Pan, J.

Chan, K. C.

TI The effect of free volume on the deformation behaviour of a Zr-based

metallic glass under nanoindentation

SO JOURNAL OF PHYSICS D-APPLIED PHYSICS

AB In this paper, the deformation behaviour of Zr65Cu17.5Ni10Al7.5 metallic glasses with different amounts of free volume was investigated with nano- and micro-indentation. It was found that the plastic flow of the metallic glasses is closely related to the amount of free volume. The metallic glasses with more free volume exhibit more conspicuous serrations with a larger pop-in size during nanoindentation and more pronounced shear bands during micro- indentation. The effect of free volume on the deformation behaviour of the Zr-based metallic glasses is discussed in terms of free volume theory.

RI Pan, Jie/L-3928-2017; Li, Ning/B-2283-2009; Chen, Qi/A-6167-2010; Chan,

K.C./A-2311-2014

OI Pan, Jie/0000-0001-5465-0736; Chan, K.C./0000-0002-6173-5532

SN 0022-3727

EI 1361-6463

PD OCT 7

PY 2007

VL 40

IS 19

BP 6055

EP 6059

DI 10.1088/0022-3727/40/19/043

UT WOS:000250604000044

ER

PT J

AU Yamamoto, T

Yokoyama, Y

Ichitsubo, T

Kimura, H

Matsubara, E

Inoue, A

AF Yamamoto, Tokujiro

Yokoyama, Yoshihiko

Ichitsubo, Tetsu

Kimura, Hisamichi

Matsubara, Eiichiro

Inoue, Akihisa

TI Precipitation of the ZrCu B2 phase in Zr50Cu50-xAlx (x=0, 4, 6) metallic

glasses by rapidly heating and cooling

SO JOURNAL OF MATERIALS RESEARCH

AB Precipitation of ZrCu with the B2 structure in Zr50Cu50-xAlx (x = 0, 4, 6) metallic glasses by rapidly heating and cooling was investigated. By rapidly heating and cooling, the ZrCu B2 phase precipitates the most in Zr50Cu46Al4 metallic glass plates prepared by tilt-casting without using a silica nozzle. The amount of the ZrCu B2 phase precipitated in Zr50Cu46Al4 metallic glass ribbons prepared by using a silica nozzle decreases by Si diffused from the silica nozzle during the preparation. This work is discussed from the viewpoint of crystallization behavior and why larger Zr-based bulk metallic glasses can be formed by suction, tilt, and cap casting without using a silica nozzle.

RI Yokoyama, Yoshihiko/A-8603-2011; Ichitsubo, Tetsu/F-3201-2010; Kimura,

Hisamichi/D-5449-2012; Yamamoto, Tokujiro/A-8827-2011; Inoue,

Akihisa/E-5271-2015

OI Ichitsubo, Tetsu/0000-0002-1127-3034; Yamamoto,

Tokujiro/0000-0002-4292-9446

SN 0884-2914

PD APR

PY 2010

VL 25

IS 4

BP 793

EP 800

DI 10.1557/JMR.2010.0105

UT WOS:000276282600025

ER

PT S

AU Uriarte, JL

Le Moulec, A

Yavari, AR

AF Uriarte, JL

Le Moulec, A

Yavari, AR

BE Schumacher, P

Warren, P

Cantor, B

TI A centrifugal force casting device for preparation of net-shape bulk

metallic glasses

SO METASTABLE, MECHANICALLY ALLOYED AND NANOCRYSTALLINE MATERIALS,

ISMANAM-2000

SE Materials Science Forum

CT International Symposium on Metastable, Mechanically Alloyed and

Nanocrystalline Materials (ISMANAM 2000)

CY JUL 09-14, 2000

CL ST CATHERINES COLL, OXFORD, ENGLAND

SP Zoz GmbH, Fisher Sci Orme Technologies, Oxford Ctr Adv Mat & Composites, Univ Oxford, Dept Mat

HO ST CATHERINES COLL

AB Recently developed Bulk Metallic Glasses (BMG) have attractive functional properties such us mechanical strength, corrosion resistance and high magnetic permeability. Using the principle of centrifugal force and taking into account the viscosity of supercooled liquid and its temperature dependence, we have developed a new experimental setup for BMG preparation. This interesting method allows the casting of the liquid metals into net-shape glassy ingots of various forms. The experimental results for a Zr based allay will be presented.

RI yavari, alain/E-8192-2010

SN 0255-5476

BN 0-87849-865-6

PY 2001

VL 360-3

BP 91

EP 94

DI 10.4028/www.scientific.net/MSF.360-362.91

UT WOS:000168667500015

ER

PT J

AU Mear, FO

Doisneau, B

Yavari, AR

Greer, AL

AF Mear, F. O.

Doisneau, B.

Yavari, A. R.

Greer, A. L.

TI Structural effects of shot-peening in bulk metallic glasses

SO JOURNAL OF ALLOYS AND COMPOUNDS

CT 14th International Symposium on Metastable and Nano-Materials

CY AUG 26-30, 2007

CL Corfu, GREECE

AB Shot-peening induces surface compressive stresses in bulk metallic glass components, improving their plasticity. Structural changes in the peened surface of fully glassy and partially crystalline Zr55Al10Cu30Ni5 are studied by X-ray diffraction, transmission electron microscopy and differential scanning calorimetry. An earlier study is extended by examining the effect of sample temperature. While fully glassy samples show no phase change under peening, partially crystalline samples show either amorphization or crystallization depending on temperature. Peening can induce very large stored energy in metallic glasses rendering them susceptible to crystallization below room temperature, a result which may be relevant for improving the plasticity of these materials. (C) 2009 Published by Elsevier B.V.

RI Greer, Lindsay/E-9433-2017; yavari, alain/E-8192-2010; Greer, Alan

Lindsay/G-1977-2011

SN 0925-8388

EI 1873-4669

PD AUG 26

PY 2009

VL 483

IS 1-2

BP 256

EP 259

DI 10.1016/j.jallcom.2007.12.105

UT WOS:000270619600063

ER

PT J

AU Cai, AH

Xiong, X

Liu, Y

Li, JH

An, WK

Luo, Y

AF Cai, An-hui

Xiong, Xiang

Liu, Yong

Li, Jiang-hong

An, Wei-ke

Luo, Yun

TI Characteristics of near-eutectic and off-eutectic Zr-Al-Ni-Cu glass

forming alloys

SO MATERIALS SCIENCE AND ENGINEERING A-STRUCTURAL MATERIALS PROPERTIES

MICROSTRUCTURE AND PROCESSING

AB The glass forming ability (GFA) and behavior of glass transition and onset crystallization for near-eutectic and off-eutectic Zr-Al-Ni-Cu metallic glasses were investigated by non-isothermal test. The results show that, as for near-eutectic alloy, the glass transition temperature T(g) is more sensitive to the heating rate than the onset crystallization temperature T(x). The apparent activation energy of glass transition E(g) is greater than that of onset crystallization E(x) and the greatest among metallic glasses studied. Its critical cooling rate R(c) is the lowest among metallic glasses studied. Moreover, the further the distance from near-eutectic composition, the larger is the R(c) for glass forming alloy. (C) 2009 Elsevier B.V. All rights reserved.

SN 0921-5093

PD AUG 15

PY 2009

VL 516

IS 1-2

BP 100

EP 102

DI 10.1016/j.msea.2009.03.025

UT WOS:000268424500016

ER

PT J

AU Tang, MB

Bai, HY

Wang, WH

AF Tang, MB

Bai, HY

Wang, WH

TI Tunneling states and localized mode in binary bulk metallic glass

SO PHYSICAL REVIEW B

AB The low-temperature specific heat of Cu50Zr50 binary bulk metallic glass is systemically measured from 1.8 K to 50.5 K. The obvious effect of the tunneling states is determined at several Kelvin by both specific heat and electrical resistivity. The density of the electron-assisted tunneling states in the bulk metallic glass at several Kelvin exceed the typical value in insulating glasses below 1 K by 2-3 orders of magnitude, and the entropy of the tunneling states is about 7.85 mJ/mol K. The specific heat in the wide temperature range was analyzed by the conventional Debye and Einstein models, and the results demonstrate the existence of the localized mode which is correlated to the boson peak in the metallic glass.

SN 2469-9950

EI 2469-9969

PD JUL

PY 2005

VL 72

IS 1

AR 012202

DI 10.1103/PhysRevB.72.012202

UT WOS:000230889800004

ER

PT J

AU Wang, WH

Wang, RJ

Li, FY

Zhao, DQ

Pan, MX

AF Wang, WH

Wang, RJ

Li, FY

Zhao, DQ

Pan, MX

TI Elastic constants and their pressure dependence of

Zr41Ti14Cu12.5Ni9Be22.5C1 bulk metallic glass

SO APPLIED PHYSICS LETTERS

AB The acoustic velocities and their pressure dependence of bulk Zr41Ti14Cu12.5Ni9Be22.5C1 metallic glass (MG) have been measured up to 0.5 GPa by using a pulse echo overlap method. The elastic constants and thermodynamic parameters as well as their pressure dependence of the MG have been determined. The obtained elastic constants were compared to that of other kinds of glasses. More information about the microstructure, elastic properties, and glass forming ability of the MG was obtained. (C) 1999 American Institute of Physics. [S0003-6951(99)00313-7].

SN 0003-6951

PD MAR 29

PY 1999

VL 74

IS 13

BP 1803

EP 1805

DI 10.1063/1.123091

UT WOS:000079408500007

ER

PT J

AU Mattern, N

AF Mattern, N.

TI Comment on "Thermal expansion measurements by X-ray scattering and

breakdown of Ehrenfest's relation in alloy liquids" [Appl. Phys. Lett.

104, 191907 (2014)]

SO APPLIED PHYSICS LETTERS

SN 0003-6951

EI 1077-3118

PD DEC 22

PY 2014

VL 105

IS 25

AR 256101

DI 10.1063/1.4904942

UT WOS:000346914000043

ER

PT S

AU Sinning, HR

AF Sinning, H. -R.

BE Igata, N

Takeuchi, S

TI Hydrogen-induced damping peak temperatures in bulk metallic glasses

SO HIGH DAMPING MATERIALS II

SE KEY ENGINEERING MATERIALS

CT 2nd International Symposium on High Damping Materials

CY SEP 09-10, 2005

CL Kyoto, JAPAN

SP Japan Soc Promot Sci, Kajima Fdn, Ogasawara Fdn, Suzuki Fdn

AB Measurements of the H-induced damping peak in Zr-based multi-component metallic glasses, with different tendency to quasicrystal formation, are added to previous reviews of the properties of this damping peak in metallic glasses, and also compared to Ti-Zr-Ni quasicrystals. The results, mainly discussed in terms of a Snoek-type relaxation mechanism for both structure types, indicate a negative correlation between the degree of icosahedral order and the temperature of the damping peak, so that improved damping properties of hydrogen-absorbing bulk metallic glasses can apparently be expected from stronger deviations from icosahedral short-range order.

SN 1013-9826

BN 0-87849-406-5

PY 2006

VL 319

BP 127

EP 132

DI 10.4028/www.scientific.net/KEM.319.127

UT WOS:000241411000020

ER

PT J

AU Lee, JK

Kim, HJ

Kim, TS

Kim, YC

Bae, JC

AF Lee, J. K.

Kim, H. J.

Kim, T. S.

Kim, Y. C.

Bae, J. C.

TI Consolidation behavior of Cu- and Ni-based bulk metallic glass

composites

SO JOURNAL OF ALLOYS AND COMPOUNDS

CT 12th International Symposium on Metastable and Nano-Materials

(ISMANAM-2005)

CY JUL 03-07, 2005

CL Paris, FRANCE

AB The Cu- and Ni-based bulk metallic glass matrix composites were fabricated by spark plasma sintering of a mixture of gas-atomized metallic glass powders and ductile brass powders. The brass powders added for the enhancement of plasticity are well distributed in the matrix after consolidation. The matrix of the composite materials remains as a fully amorphous phase after consolidation process. With increasing the brass content, the level of plasticity strain increased, although the level of strength decreased. The successful consolidation of metallic glass matrix composite with high density was attributed to viscous flow in the supercooled liquid state during spark plasma sintering. (C) 2006 Elsevier B.V. All rights reserved.

SN 0925-8388

PD MAY 31

PY 2007

VL 434

SI SI

BP 336

EP 339

DI 10.1016/j.jallcom.2006.08.304

UT WOS:000246286900088

ER

PT J

AU Wang, ZX

Lu, JB

Xi, YJ

AF Wang Zhixin

Lu Jinbin

Xi Yanjun

TI Crystallization mechanism of CeAlFeCo bulk metallic glasses

SO JOURNAL OF RARE EARTHS

AB Crystallization behaviors of Ce60Al15Fe5+xCo20-x (x=0, 5, 10) bulk metallic glasses (BMGs) were studied by means of differential scanning calorimeter (DSC) and X-ray diffraction (XRD). The crystallization processes of different samples were simulated by JMA equation. Experimental results demonstrated that incubation and crystallization time increased with decreasing isothermal temperature for the same sample. The crystallization mechanism of CeAlFeCo BMGs was discussed.

SN 1002-0721

PD FEB

PY 2010

VL 28

IS 1

BP 158

EP 160

DI 10.1016/S1002-0721(09)60071-5

UT WOS:000275969800034

ER

PT J

AU Lu, L

Xiong, DX

Wang, JB

Zhao, DS

Sun, YF

AF Lu, Lu

Xiong, Dongxia

Wang, Jianbo

Zhao, Dongshan

Sun, Yufeng

TI Varied linear phason strain and its induced domain structure in

quasicrystalline precipitates of Zr-Al-Ni-Cu-Nb bulk metallic glass

matrix composites (vol 27, pg 3041, 2012)

SO JOURNAL OF MATERIALS RESEARCH

RI Sun, Yufeng/R-3800-2016

OI Sun, Yufeng/0000-0002-6076-1026

SN 0884-2914

PD FEB

PY 2013

VL 28

IS 4

BP 658

EP 658

DI 10.1557/jmr.2012.427

UT WOS:000314988200014

ER

PT J

AU Schuh, CA

Lund, AC

AF Schuh, CA

Lund, AC

TI Atomistic basis for the plastic yield criterion of metallic glass

SO NATURE MATERIALS

RI Schuh, Christopher/C-7947-2009

OI /0000-0001-9856-2682

SN 1476-1122

PD JUL

PY 2003

VL 2

IS 7

BP 449

EP 452

DI 10.1038/nmat918

UT WOS:000183892200016

PM 12792648

ER

PT J

AU Zhang, SG

Li, JG

AF Zhang, S. G.

Li, J. G.

TI Evaluation of dynamic behaviors of metallic glass-forming liquids by

elastic constants

SO MATERIALS LETTERS

AB The dynamic behaviors of glass-forming liquids could be characterized by the reduced glass transition temperature T-rg (= T-g/T-l) and fragility index m, where T-g and T-l are the glass transition temperature and liquidus temperature, respectively. When normalized to specific volume, T-g, T-l and m of bulk metallic glasses (BMGs) reveal solid linear correlations with elastic constants. This allows formulating T-rg, and m in terms of elastic constants, and thus evaluating liquid dynamics useful for predicting and screening new glass formers efficiently. (C) 2012 Elsevier B.V. All rights reserved.

SN 0167-577X

EI 1873-4979

PD MAY 15

PY 2012

VL 75

BP 179

EP 182

DI 10.1016/j.matlet.2012.02.036

UT WOS:000303178600053

ER

PT J

AU Rafique, MMA

Qiu, D

Easton, M

AF Rafique, Muhammad Musaddique Ali

Qiu, Dong

Easton, Mark

TI Modeling and simulation of microstructural evolution in Zr based Bulk

Metallic Glass Matrix Composites during solidification (vol 2, pg 3591,

2017)

SO MRS ADVANCES

SN 2059-8521

PY 2017

VL 2

IS 58-59

BP 3673

EP 3674

DI 10.1557/adv.2017.582

UT WOS:000413253500017

ER

PT J

AU Grell, D

Gostin, PF

Eckert, J

Gebert, A

Kerscher, E

AF Grell, Daniel

Gostin, Petre Flaviu

Eckert, Juergen

Gebert, Annett

Kerscher, Eberhard

TI In Situ Electrochemical Analysis during Deformation of a Zr-Based Bulk

Metallic Glass: A Sensitive Tool Revealing Early Shear Banding

SO ADVANCED ENGINEERING MATERIALS

RI Gostin, Petre-Flaviu/D-2337-2011

OI Gostin, Petre-Flaviu/0000-0002-0528-7093; Gebert,

Annett/0000-0003-2748-3850

SN 1438-1656

EI 1527-2648

PD NOV

PY 2015

VL 17

IS 11

BP 1532

EP 1535

DI 10.1002/adem.201500273

UT WOS:000367538600002

ER

PT J

AU Jiang, JZ

AF Jiang, JZ

TI Comment on "Unusual transition phenomenon in Zr-based bulk metallic

glass upon heating at high pressure" [Appl. Phys. Lett. 80, 3087 (2002)]

SO APPLIED PHYSICS LETTERS

SN 0003-6951

PD NOV 11

PY 2002

VL 81

IS 20

BP 3894

EP 3895

DI 10.1063/1.1523639

UT WOS:000179042200064

ER

PT J

AU Caron, A

Wunderlich, R

Gu, L

Fecht, HJ

AF Caron, A.

Wunderlich, R.

Gu, L.

Fecht, H-J.

TI Structurally enhanced anelasticity in Zr-based bulk metallic glasses

SO SCRIPTA MATERIALIA

AB Atomic force acoustic microscopy was used to image bulk metallic glasses of composition Zr63-xCu24AlxNi10Co3 with 8 < x < 15. The elastic contrast was modulated on a length scale of 250-400 nm. A similar modulation was identified by transmission electron microscopy. Using the thermoelastic, model a good correlation between the observed anelasticity and the evidenced mesostructure was obtained. (C) 2011 Acta Materialia Inc. Published by Elsevier Ltd. All rights reserved.

RI Caron, Arnaud/B-6463-2010; Gu, Lin/D-9631-2011

OI Caron, Arnaud/0000-0003-0985-7441; Gu, Lin/0000-0002-7504-031X

SN 1359-6462

PD MAY

PY 2011

VL 64

IS 10

BP 946

EP 949

DI 10.1016/j.scriptamat.2011.01.043

UT WOS:000289330700006

ER

PT J

AU Makarov, S

Mitrofanov, YP

Afonin, GV

Kobelev, NP

Khonik, VA

AF Makarov, S.

Mitrofanov, Yu. P.

Afonin, G. V.

Kobelev, N. P.

Khonik, V. A.

TI Shear susceptibility - A universal integral parameter relating the shear

softening, heat effects, anharmonicity of interatomic interaction and

"defect" structure of metallic glasses

SO INTERMETALLICS

AB We studied shear modulus relaxation and heat effects occurring upon structural relaxation of La-, Zr- and Pd-based bulk metallic glasses. On this basis, we suggest a new method for the determination of the shear susceptibility, which appears to constitute a universal integral parameter relating different physical phenomena - shear softening, heat effects and anharmonicity of interatomic interaction - with the "defect" structure of metallic glasses.

RI Khonik, Vitaly/A-5888-2009; Makarov, Andrey/H-4156-2013; Konchakov,

Roman/K-4616-2013; Afonin, Gennady/K-1014-2013; Mitrofanov,

Yuriy/E-7963-2010

OI Makarov, Andrey/0000-0001-6741-0619; Afonin,

Gennady/0000-0002-7715-5065; Mitrofanov, Yuriy/0000-0002-7939-5230

SN 0966-9795

EI 1879-0216

PD AUG

PY 2017

VL 87

BP 1

EP 5

DI 10.1016/j.intermet.2017.04.001

UT WOS:000401737400001

ER

PT J

AU de Oliveira, M

Botta, WJ

Yavari, AR

AF de Oliveira, M

Botta, WJ

Yavari, AR

TI Connecting, assemblage and electromechanical shaping of bulk metallic

glasses

SO MATERIALS TRANSACTIONS JIM

AB Shaping of Bulk metallic glasses (BMG) and BMG-based composites into various complex forms has been achieved by a new electromechanical process. Bulk metallic glasses have large supercooled regions between the glass transition temperature T-g and the crystallisation temperature T-x up to some hundred degrees higher. In this range, the undercooled liquid in principal deforms in a Newtonian way, allowing thermomechanical shaping in the low viscosity range as applied to oxide glasses. Electromechanical shaping technology allows rapid shaping at low applied stresses by eliminating the thermal mass of the furnace and and the need to heat the deformation dies. Joule heating is efficiently used thanks to the high electrical resistivity of bulk metallic glasses. Here it is shown that large shape changes (high deformations) can be achieved without crystallisation. For example, crosses and other mechanically resistant complex forms are achieved from two amorphous rods or an amorphous rod and a crystalline bar.

RI de Oliveira, Marcelo/B-9881-2012; yavari, alain/E-8192-2010; Botta,

Walter/E-7763-2010

OI de Oliveira, Marcelo/0000-0003-4589-2463; Botta,

Walter/0000-0003-2759-573X

SN 0916-1821

PD NOV

PY 2000

VL 41

IS 11

BP 1501

EP 1504

DI 10.2320/matertrans1989.41.1501

UT WOS:000166559500026

ER

PT J

AU Yoshikawa, T

Tokuda, M

Inaba, T

AF Yoshikawa, Takamasa

Tokuda, Masataka

Inaba, Tadashi

TI Thermoplastic deformation and mechanical properties of Zr-based bulk

metallic glasses

SO MATERIALS TRANSACTIONS

CT 5th International Conference on Bulk Metallic Glasses

CY OCT 01-05, 2006

CL Osaka Univ, Awaji Isl, JAPAN

SP Minist Educ, Culture, Sports, Sci & Technol, Inst Mat Res, Tohoku Univ, Japan Soc Promot Sci, Natl Inst Mat Sci, Hyogo Int Assoc

HO Osaka Univ

AB The effect of heating and thermoplastic deformation on the fracture stress of Zr55CU30Al10Ni5 and Zr60Cu25Al10Ni5 bulk metallic glass at room temperature (RT) was studied. These metallic glasses were easily crystallized by the influence of heating beyond their upper limit temperature of 685 K and 713 K, respectively. The fracture stress was maintained the strength of material as cast below these temperatures while it decreased easily by crystallization. It was possible to deform these materials below these temperatures. Their strength after thermoplastic deformation depended on the amount of strain, strain rate, and temperature during the deformation. The lower temperature and higher strain rate resulted in high strength after thermoplastic deformation at RT.

SN 1345-9678

EI 1347-5320

PD JUL

PY 2007

VL 48

IS 7

BP 1838

EP 1841

DI 10.2320/matertrans.MJ200749

UT WOS:000248743100054

ER

PT J

AU Ichitsubo, T

Matsubara, E

AF Ichitsubo, T.

Matsubara, E.

TI Structural inhomogeneity of metallic glass observed by ultrasonic and

inelastic X-ray scattering measurements

SO MATERIALS SCIENCE AND ENGINEERING A-STRUCTURAL MATERIALS PROPERTIES

MICROSTRUCTURE AND PROCESSING

CT 15th International Conference on Internal Friction and Mechanical

Spectroscopy (ICIFMS-15)

CY JUL 20-25, 2008

CL Perugia, ITALY

SP Perugia Univ, Phys Dept, CNISM

AB The structural stability of metallic glasses is frequently deteriorated under ultrasonic perturbation at relatively low temperatures, e.g., near the glass transition temperature T(g), even for thermally stable Pd- and Zr-based metallic glasses. By a mechanical spectroscopy analysis, it is suggested that such an instability, i.e., crystallization, is caused by atomic motions associated with the (slow) beta relaxation, that are resonant with the ultrasonic-strain field. Furthermore, such atomic motions below T(g) are considered to occur at weakly bonded regions in a nanoscale inhomogeneous microstructure of glass, which was intuitively inferred from a partially crystallized microstructure obtained by annealing a Pd-based metallic glass just below T(g) under ultrasonic perturbation. On this basis, we proposed a structural model of metallic glasses that consists of strongly bonded regions surrounded by weakly bonded regions. To reveal the validity of the model, we have also employed the inelastic X-ray scattering technique to measure the sound velocity of nanometer wavelength of longitudinal acoustic phonons. We have found in a completely frozen Pd-based metallic glass that the velocity of nanometer wavelength exceeds ultrasound velocity of millimeter wavelength, which suggests that elastically harder nanoscale regions exist in the glass matrix. (C) 2009 Elsevier B.V. All rights reserved.

RI Ichitsubo, Tetsu/F-3201-2010

OI Ichitsubo, Tetsu/0000-0002-1127-3034

SN 0921-5093

PD SEP 25

PY 2009

VL 521-22

BP 236

EP 242

DI 10.1016/j.msea.2008.09.149

UT WOS:000270330700059

ER

PT J

AU Shao, H

Xu, YL

Shi, B

Yu, CS

Hahn, H

Gleiter, H

Li, JG

AF Shao, Hang

Xu, Yuanli

Shi, Bo

Yu, Chengshou

Hahn, Horst

Gleiter, Herbert

Li, Jiangong

TI High density of shear bands and enhanced free volume induced in

Zr70Cu20Ni10 metallic glass by high-energy ball milling

SO JOURNAL OF ALLOYS AND COMPOUNDS

AB High-energy ball milling was employed to induce plastic deformation of Zr70Cu20Ni10 metallic glass ribbons, resulting in intersected and branched shear bands with a minimal spacing of about 30 nm. Crystallization was not observed in the shear bands. The free volume content in the deformed metallic glass increases with increasing duration of ball milling. Compared with the as-spun metallic glass, 28.8% more free volume is introduced in the metallic glass after ball milling for 80 h. (c) 2012 Elsevier B.V. All rights reserved.

RI Hahn, Horst/G-9018-2011

OI Hahn, Horst/0000-0001-9901-3861; Li, Jiangong/0000-0001-6837-1084

SN 0925-8388

PD JAN 25

PY 2013

VL 548

BP 77

EP 81

DI 10.1016/j.jallcom.2012.08.132

UT WOS:000312107400014

ER

PT J

AU Soto, CEB

Vargas, IAF

Velazquez, JRF

Rodriguez, GAL

Martinez, JAV

AF Borja Soto, Carlos Ernesto

Figueroa Vargas, Ignacio Alejandro

Fonseca Velazquez, Jose Ramon

Lara Rodriguez, Gabriel Angel

Verduzco Martinez, Jorge Alejandro

TI Composition, Elastic Property and Packing Efficiency Predictions for

Bulk Metallic Glasses in Binary, Ternary and Quaternary Systems

SO MATERIALS RESEARCH-IBERO-AMERICAN JOURNAL OF MATERIALS

AB The results of database based on the efficiency packed model for metallic glasses. The database contains the atomic radii information as well as elastic properties of the most commonly used alloying elements, permitting composition, packing efficiency and elastic property predictions to be made for binary, ternary and quaternary bulk metallic glasses. Twenty different alloys per system (binary, ternary and quaternary) experimentally reported in the literature were compared with those estimated by the database. Comparison charts and diagrams showed good agreement between the composition predictions and those reported from the experimentally processed metallic glasses. The elastic properties predictions could be used to elaborate Blackman diagrams in order to know, in advance, the intrinsic toughness that the investigated alloys might present. The database is intended for designing bulk metallic glasses. Finally, some quaternary alloys were experimentally produced based on the prediction obtained with the database, showing a glassy phase. The microhardness values obtained experimentally of the Zr57.52Ag10.62Al10.62Co21.24, Zr57.19Al10.7Ni10.7Cu21.41 and Hf60.22Al9.95Cu9.95Ni19.89 alloys, are 3.8, 4.0 and 3.6 GPa, respectively. The Young's modulus calculated using microhardness values (E/H-v = 20) are closed to the values obtained by the "mixing rules".

OI Martinez-Gomez, Javier/0000-0001-8807-7595

SN 1516-1439

EI 1980-5373

PD MAR-APR

PY 2016

VL 19

IS 2

BP 285

EP 294

DI 10.1590/1980-5373-MR-2015-0537

UT WOS:000374233300003

ER

PT S

AU Schroers, J

AF Schroers, Jan

BA Sarac, B

BF Sarac, B

TI Artificial Microstructure Approach

SO MICROSTRUCTURE-PROPERTY OPTIMIZATION IN METALLIC GLASSES

SE Springer Theses-Recognizing Outstanding PhD Research

SN 2190-5053

BN 978-3-319-13033-0; 978-3-319-13032-3

PY 2015

BP 37

EP 80

DI 10.1007/978-3-319-13033-0_4

D2 10.1007/978-3-319-13033-0

UT WOS:000372459200005

ER

PT J

AU Inoue, A

Takeuchi, A

AF Inoue, A

Takeuchi, A

TI Recent progress in bulk glassy alloys

SO MATERIALS TRANSACTIONS

AB This paper deals with recent progress in stabilized supercooled liquid and the resulting bulk glassy alloys and focusing on the following factors; alloy composition, forming ability, formation mechanism, computed glass-forming ability, computed glass formation range, atomic configuration, production techniques, mechanical properties, corrosion resistance, soft magnetic properties, micro-forming ability, applications, significance to science and engineering, and future trends for bulk glassy alloys including supercooled liquid. As demonstrated in this review, the high stability of metallic supercooled liquid has already opened up new fields of investigation in basic science and yielded new engineering applications. There is every reason to expect that their importance will continue to increase.

RI Inoue, Akihisa/E-5271-2015; Takeuchi, Akira/A-3619-2010

OI Takeuchi, Akira/0000-0002-7246-8644

SN 1345-9678

EI 1347-5320

PD AUG

PY 2002

VL 43

IS 8

BP 1892

EP 1906

DI 10.2320/matertrans.43.1892

UT WOS:000177930100021

ER

PT J

AU Guo, GQ

Wu, SY

Yang, L

AF Guo, Gu-Qing

Wu, Shi-Yang

Yang, Liang

TI Structural Origin of the Enhanced Glass-Forming Ability Induced by

Microalloying Y in the ZrCuAl Alloy

SO METALS

AB In this work, the structural origin of the enhanced glass-forming ability induced by microalloying Y in a ZrCuAl multicomponent system is studied by performing synchrotron radiation experiments combined with simulations. It is revealed that the addition of Y leads to the optimization of local structures, including: (1) more Zr-centered and Y-centered icosahedral-like clusters occur in the microstructure; (2) the atomic packing efficiency inside clusters and the regularity of clusters are both enhanced. These structural optimizations help to stabilize the amorphous structure in the ZrCuAlY system, and lead to a high glass-forming ability (GFA). The present work provides an understanding of GFAs in multicomponent alloys and will shed light on the development of more metallic glasses with high GFAs.

SN 2075-4701

PD APR

PY 2016

VL 6

IS 4

AR 67

DI 10.3390/met6040067

UT WOS:000375861100001

ER

PT J

AU Dmowski, W

Fan, C

Morrison, ML

Liaw, PK

Egami, T

AF Dmowski, W.

Fan, C.

Morrison, M. L.

Liaw, P. K.

Egami, T.

TI Structural changes in bulk metallic glass after annealing below the

glass-transition temperature

SO MATERIALS SCIENCE AND ENGINEERING A-STRUCTURAL MATERIALS PROPERTIES

MICROSTRUCTURE AND PROCESSING

AB Small structural changes in bulk metallic glasses (BMGs) following thermal treatment below the glass-transition temperature were observed. Zr-based BMG ingots with nominal compositions of Zr52.5Cu17.9Ni14.6Al10.0Ti5.0 (at.%) were isothermally annealed at 630 K for 10-60 min in vacuum and then cooled down. Structural relaxation was indicated by enthalpy evolution during differential scanning calorimetry and by changes in micro-hardness. Atomic structure was studied using time-of-flight neutron diffraction followed by a pair-distribution function (PDF) analysis. Observed changes in the PDF could not be explained by a "free volume" annealing, but were consistent with the elimination of short and long inter-atomic distances. (C) 2007 Elsevier B.V. All rights reserved.

OI Morrison, Mark/0000-0003-1956-0932

SN 0921-5093

PD DEC 15

PY 2007

VL 471

IS 1-2

BP 125

EP 129

DI 10.1016/j.msea.2006.12.137

UT WOS:000250168000019

ER

PT J

AU Xu, WQ

Robin, L

Zheng, RK

Laws, KJ

Ferry, M

AF Xu, Wanqiang

Robin, Lalu

Zheng, Rongkun

Laws, Kevin J.

Ferry, Michael

TI Phase redistribution in an in situ Mg-based bulk metallic glass

composite during deformation in the supercooled liquid region (vol 63,

pg 556, 2010)

SO SCRIPTA MATERIALIA

RI Zheng, Rongkun/A-7923-2008

OI Zheng, Rongkun/0000-0002-7860-2023

SN 1359-6462

PD OCT

PY 2010

VL 63

IS 8

BP 903

EP 903

DI 10.1016/j.scriptamat.2010.06.043

UT WOS:000281184500028

ER

PT J

AU Yan, HH

Hu, Y

Yan, ZJ

Zheng, XH

Li, YT

AF Yan, Honghong

Hu, Yong

Yan, Zhijie

Zheng, Xiaohua

Li, Yongtang

TI Microstructure Evolution of Zr50Cu18Ni17Al10Ti5 Bulk Metallic Glass

during Cold-rolling

SO JOURNAL OF MATERIALS SCIENCE & TECHNOLOGY

AB Zr50Cu18Ni17Al10Ti5 bulk metallic glass has been rolled at room temperature up to 95% in thickness reduction, and the dependence of microstructure on the strain was investigated. With increasing thickness reduction, the full width at half maximum (FWHM) and crystallization enthalpy decrease gradually till 80%, and then increase evidently at 95%. It is revealed that the reversible transition between the ordered and disordered atomic configurations was found in the metallic glass as the deformation proceeds, which is further verified by the high-resolution transmission electron microscopy images. The final microstructure in metallic glass during cold-rolling is the net result of two competing processes between shear-induced disordering and diffusion controlled reordering.

SN 1005-0302

PD AUG

PY 2012

VL 28

IS 8

BP 756

EP 760

UT WOS:000309037200012

ER

PT J

AU Ji, YF

Pang, SJ

Ma, CL

Zhang, T

AF Ji, Yunfei

Pang, Shujie

Ma, Chaoli

Zhang, Tao

TI FORMATION OF La-Al-Ni-Cu-Fe BULK METALLIC GLASSES WITH HIGH

GLASS-FORMING ABILITY

SO INTERNATIONAL JOURNAL OF MODERN PHYSICS B

CT 5th International Conference on Advanced Materials Development and

Performance

CY OCT 12-15, 2008

CL Beijing, PEOPLES R CHINA

SP Tokushima Univ, Kyungpook Natl Univ, Beihang Univ, Univ Auckland

AB The effect of alloy composition on improving glass-forming ability of La-based alloys is investigated in this work. Previous composition criteria demonstrated that the alloys with high glass-forming ability should have negative heats of mixing among the main constituent elements. In this study, the addition of Fe to a La-based La-Al-Ni-Cu alloy significantly improved the glass-forming ability, although the heat of mixing between Fe and the main element La is positive. La-Al-Ni-Cu-Fe bulk metallic glasses with diameters up to 15 mm were prepared by the method of pouring the molten alloys into a copper mold. These La-Al-Ni-Cu-Fe bulk metallic glasses exhibit relatively wide supercooled liquid region of about 50 k, and high T(rg)(T(g)/T(l)) and Y(T(x)/(T(g)+T(l))) values. It is found that the addition of Fe to the La-Al-Ni-Cu alloy lowers the Gibbs free energy difference between the liquid and crystalline phases in the supercooled liquid region and enhances the glass-forming ability of the alloy.

RI Zhang, Tao/O-4911-2014; Pang, Shujie/D-8305-2016

SN 0217-9792

PD JUN 30

PY 2010

VL 24

IS 15-16

SI SI

BP 2314

EP 2319

DI 10.1142/S021797921006485X

UT WOS:000282197000015

ER

PT J

AU Lu, ZP

Li, Y

Ng, SC

AF Lu, ZP

Li, Y

Ng, SC

TI Reduced glass transition temperature and glass forming ability of bulk

glass forming alloys

SO JOURNAL OF NON-CRYSTALLINE SOLIDS

AB Onset temperature (solidus) T-m and offset temperature (liquidus) T-l of melting of a series of bulk glass forming alloys based on Zr, La, Mg, Pd and rare-earth elements have been measured by studying systematically the melting behaviour of these alloys using DTA or DSC. Bulk metallic glass formation has been found to be most effective at or near their eutectic points and less effective for off-eutectic alloys. Reduced glass transition temperature T-rg given by T-g/T-l is found to show a stronger correlation with critical cooling rate or critical section thickness for glass formation than T-rg given by T-g/T-m. (C) 2000 Elsevier Science B.V. All rights reserved.

RI Lu, Zhao-Ping/A-2718-2009

OI Lu, Zhao-Ping/0000-0003-1463-8948

SN 0022-3093

EI 1873-4812

PD MAY

PY 2000

VL 270

IS 1-3

BP 103

EP 114

DI 10.1016/S0022-3093(00)00064-8

UT WOS:000087244300012

ER

PT J

AU Chen, DZ

Jang, D

Guan, KM

An, Q

Goddard, WA

Greer, JR

AF Chen, D. Z.

Jang, D.

Guan, K. M.

An, Q.

Goddard, W. A., III

Greer, J. R.

TI Nanometallic Glasses: Size Reduction Brings Ductility, Surface State

Drives Its Extent

SO NANO LETTERS

AB We report tensile experiments on Ni80P20 metallic glass samples fabricated via a templated electroplating process and via focused ion beam milling, which differed only in their surface energy states: Ga-ion-irradiated and as-electroplated. Molecular dynamics simulations on similar Ni80Al20 systems corroborate the experimental results, which suggest that the transition from brittle to ductile behavior is driven by sample size, while the extent of ductility is driven by surface state.

RI Jang, Dongchan/C-9510-2012

OI Jang, Dongchan/0000-0002-2814-9734

SN 1530-6984

EI 1530-6992

PD SEP

PY 2013

VL 13

IS 9

BP 4462

EP 4468

DI 10.1021/nl402384r

UT WOS:000330158900079

PM 23978318

ER

PT J

AU Wei, BC

Zhang, TH

Li, WH

Xing, DM

Zhang, LC

Wang, YR

AF Wei, BC

Zhang, TH

Li, WH

Xing, DM

Zhang, LC

Wang, YR

TI Indentation creep behavior in Ce-based bulk metallic glasses at room

temperature

SO MATERIALS TRANSACTIONS

AB The room temperature creep behaviors of Cc-based bulk metallic glasses were examined by the use of nanoindentation. The creep rate and creep rate sensitivity of Ce-based BMGs were derived from indentation creep curves. The low creep rate sensitivity of Ce-based BMGs indicates that the room temperature creep is dominated by localized shear flow. The experimental creep Curves can be described by a generalized Kelvin model. Furthermore, the creep retardation spectrum is calculated for the Ce-based metallic glasses. The results showed that creep retardation spectrum consists of two relatively separated peaks with the well defined characteristic relaxation times.

RI zhang, lingchenzhng/A-1724-2009; Wang, Yuren/B-2124-2013

SN 1345-9678

EI 1347-5320

PD DEC

PY 2005

VL 46

IS 12

BP 2959

EP 2962

DI 10.2320/matertrans.46.2959

UT WOS:000234846500072

ER

PT J

AU Wang, X

Shao, Y

Yao, KF

AF Wang Xin

Shao Yang

Yao Ke-Fu

TI Chemical composition dependence of atomic oxygen erosion resistance in

Cu-rich bulk metallic glasses

SO CHINESE SCIENCE BULLETIN

AB The effect of atomic oxygen (AO) on the surface oxidation of several typical Cu-based bulk metallic glasses (BMGs) was studied in the present work. The AO source using in this study is generated by discharge plasma type ground simulation equipment. The AO erosion/oxidation resistances of the amorphous alloy samples were assessed based on the analysis of mass loss, surface color and microstructure. It is found that these Cu-based BMGs possess good AO erosion/oxidation resistance and their resistance to AO erosion/oxidation strongly depends on the chemical composition. For the samples containing more Ag and/or Cu, the AO erosion/oxidation resistance is weaker. The present result is important for designing new metallic glasses using as space materials.

RI Wang, Xin/F-8920-2014; Shao, Yang/H-2722-2013

OI Wang, Xin/0000-0002-9992-4195; Shao, Yang/0000-0001-5369-9933

SN 1001-6538

PD DEC

PY 2012

VL 57

IS 36

BP 4801

EP 4804

DI 10.1007/s11434-012-5573-5

UT WOS:000312637600019

ER

PT J

AU Qiang, JB

Zhang, W

Xie, GQ

Inoue, A

AF Qiang, J. B.

Zhang, W.

Xie, G. Q.

Inoue, A.

TI Unusual room temperature ductility of a Zr-based bulk metallic glass

containing nanoparticles

SO APPLIED PHYSICS LETTERS

AB Highly ductile Zr65Al7.5Cu27.5 bulk metallic glass (BMG) containing nanometer scaled fcc phase was obtained by copper mold casting. Room temperature compression tests revealed superior mechanical properties of the alloy: yielding stress of 1670 MPa, Young's modulus of 90 GPa, elastic strain of 2%, and especially a remarkable plasticity of over 50%. The marginal BMG is a useful model system to examine the nanocrystal coalescence model and the shear-transformation-zone model proposed for large scale plastic deformations of metallic glasses. (c) 2007 American Institute of Physics.

RI Xie, Guoqiang/A-8619-2011; Inoue, Akihisa/E-5271-2015

SN 0003-6951

PD JUN 4

PY 2007

VL 90

IS 23

AR 231907

DI 10.1063/1.2746071

UT WOS:000247145500026

ER

PT J

AU Bunz, J

Wilde, G

AF Buenz, J.

Wilde, G.

TI Direct measurement of the kinetics of volume and enthalpy relaxation of

an Au-based bulk metallic glass

SO JOURNAL OF APPLIED PHYSICS

AB Structural relaxation of glasses below their glass transition is a well-studied phenomenon that still poses several open issues. With the advent of bulk metallic glasses with exceptionally low glass transition temperatures, new options are available that are based on the experimental assessment of the time dependence of several different thermodynamic quantities by direct measurements with high accuracy. In this contribution the first direct measurement of the isothermal relaxation of the volume and the enthalpy of an Au-based bulk metallic glassformer are reported and discussed with respect of the characteristics describing the underlying processes. (C) 2013 AIP Publishing LLC.

RI Wilde, Gerhard/C-7808-2013

OI Wilde, Gerhard/0000-0001-8001-5998

SN 0021-8979

EI 1089-7550

PD DEC 14

PY 2013

VL 114

IS 22

AR 223503

DI 10.1063/1.4843716

UT WOS:000329090400012

ER

PT J

AU Wang, WH

Lewandowski, JJ

Greer, AL

AF Wang, WH

Lewandowski, JJ

Greer, AL

TI Understanding the glass-forming ability of Cu50Zr50 alloys in terms of a

metastable eutectic

SO JOURNAL OF MATERIALS RESEARCH

AB Interest in finding binary alloys that can form bulk metallic glasses has stimulated recent work on the Cu-Zr system, which is known to show glass formation over a wide composition range. This work focuses on copper mold casting of Cu50Zr50 (at.%), and it is shown that fully amorphous rods up to 2-mm diameter can be obtained. The primary intermetallic phase competing with glass formation on cooling is identified, and the glass-forming ability is interpreted in terms of a metastable eutectic involving this phase. Minor additions of aluminum increase the glass-forming ability: with addition of 4 at.% Al to Cu5oZr50, rods of at least 5-mm diameter can be cast fully amorphous. The improvement of glass-forming ability is related to suppression of the primary intermetallic phase.

RI Lewandowski, John/S-3815-2017; Greer, Alan Lindsay/G-1977-2011; Greer,

Lindsay/E-9433-2017

OI Lewandowski, John/0000-0002-3389-2637;

SN 0884-2914

PD SEP

PY 2005

VL 20

IS 9

BP 2307

EP 2313

DI 10.1557/JMR.2005.0302

UT WOS:000231648900012

ER

PT J

AU Gong, F

Chen, SH

Ran, JQ

Yang, Z

Ma, J

AF Gong, Feng

Chen, Shun Hua

Ran, Jia Qi

Yang, Zhi

Ma, Jiang

TI Tuning the performance of bulk metallic glasses by milling artificial

holes

SO MATERIALS SCIENCE AND ENGINEERING A-STRUCTURAL MATERIALS PROPERTIES

MICROSTRUCTURE AND PROCESSING

AB The mechanical performance of materials is greatly affected and could be tuned by artificial defects, especially for amorphous alloys. In present work, specially designed holes are created for bulk metallic glass and apparent mechanical performance improvement is obtained when compared with the intact ones. The fracture characterization discovers that the inner wall of the artificial hole has a blocking effect to shear bands (SBs), leading to an apparent enhancement of mechanical property. Our results demonstrate that the blocking effect of SBs induced by the designing artificial hole may provide some new sights on the plastic deformation mechanism of metallic glasses rather than the improved plasticity itself. (C) 2016 Elsevier B.V. All rights reserved.

RI Chen, S.H./H-3694-2013

OI Chen, S.H./0000-0002-7060-6249

SN 0921-5093

EI 1873-4936

PD JUN 21

PY 2016

VL 668

BP 50

EP 54

DI 10.1016/j.msea.2016.05.048

UT WOS:000378468500006

ER

PT J

AU Su, C

Chen, Y

Yu, P

Song, M

Chen, W

Guo, SF

AF Su, C.

Chen, Y.

Yu, P.

Song, M.

Chen, W.

Guo, S. F.

TI Linking the thermal characteristics and mechanical properties of

Fe-based bulk metallic glasses

SO JOURNAL OF ALLOYS AND COMPOUNDS

AB In this work, (Fe75Mo5P13C7)(100-x)B-x (x = 0.3, 0.5, 1, 1.5, 2 at.%) bulk metallic glasses (BMGs) with a diameter of 1.5 mm were successfully prepared by low purity industrial raw materials and copper mold casting. The thermal characteristics and the mechanical properties were systematically investigated. We revealed a correlation between the thermal characteristics and the mechanical properties. In particular, we found that a low glass transition temperature and a low activation energy of glass transition are generally associated with a high plasticity at room temperature. Our findings would provide a novel insight into understanding and designing high performance Fe-based BMGs from the perspective of thermal behaviors. (C) 2015 Elsevier B.V. All rights reserved.

RI Song, Min/C-3730-2013; Guo, Shengfeng/E-3171-2012

OI Song, Min/0000-0002-3197-4647; Guo, Shengfeng/0000-0002-6667-6797

SN 0925-8388

EI 1873-4669

PD APR 5

PY 2016

VL 663

BP 867

EP 871

DI 10.1016/j.jallcom.2015.12.196

UT WOS:000369060200115

ER

PT J

AU Qiao, JC

Pelletier, JM

AF Qiao, J. C.

Pelletier, J. M.

TI Kinetics of structural relaxation in bulk metallic glasses by mechanical

spectroscopy: Determination of the stretching parameter beta(KWW)

SO INTERMETALLICS

AB Structural relaxation in Cu46Zr45Al7Dy2, Ti40Zr25Ni8Cu9Be18 and Zr41.2Ti13.8Cu12.5Ni10Be22.5 (Vit 1) bulk metallic glasses has been studied by mechanical spectroscopy during annealing below the glass transition temperature T-g. Structural relaxation in bulk metallic glasses leads to an increase of the storage modulus G' and a decrease of the loss factor tan delta, which are observed after an incubation time. Kinetics of this phenomenon can be well described by a stretched exponential, similar to the Kohlrausch -Williams-Watts (KWW) equation used to fit differential scanning calorimetry (DSC) experiments. In the various investigated bulk metallic glasses the stretching exponent beta(KWW) does not depend on annealing temperature implying therefore that this exponent beta(KWW) is nearly independent of the microstructure. Decrease in the loss factor is due to a decrease in the atomic mobility. Various models have been proposed in the literature to describe this evolution: decrease in the free volume, decrease in the liquid-like regions or decrease in the defect concentration (flow defects or quasi-point defects). Present results are discussed using this concept of defect. In any case it corresponds to an increase of the short range order during annealing below the glass transition temperature T-g. (C) 2012 Elsevier Ltd. All rights reserved.

SN 0966-9795

PD SEP

PY 2012

VL 28

BP 40

EP 44

DI 10.1016/j.intermet.2012.03.058

UT WOS:000307038700006

ER

PT J

AU Qiao, JW

Zhang, Y

Jia, HL

Yang, HJ

Liaw, PK

Xu, BS

AF Qiao, J. W.

Zhang, Y.

Jia, H. L.

Yang, H. J.

Liaw, P. K.

Xu, B. S.

TI Tensile softening of metallic-glass-matrix composites in the supercooled

liquid region

SO APPLIED PHYSICS LETTERS

AB A Ti-based metallic-glass-matrix composite exhibits tensile softening (necking) in the supercooled liquid region, accompanied by a large tensile ductility and a fragmentation of dendrites. Subjected to high temperatures, concurrent crystallization does not occur, suggesting a good thermal stability of the glass matrix. The presence of high-volume-fractioned dendrites lowers the rheology of the viscous glass matrix at high temperatures, which results in an absence of super elongation as monolithic bulk metallic glasses (BMGs). A tensile strength of 970 MPa is higher than those of most BMGs under varying strain rates, ascribing to the retardation of softening by the dendrites. (C) 2012 American Institute of Physics. [http://dx.doi.org/10.1063/1.3696026]

RI Jia, Haoling/P-4853-2014; ZHANG, Yong/B-7928-2009

OI Jia, Haoling/0000-0002-4287-2929; ZHANG, Yong/0000-0002-6355-9923

SN 0003-6951

EI 1077-3118

PD MAR 19

PY 2012

VL 100

IS 12

AR 121902

DI 10.1063/1.3696026

UT WOS:000302228700025

ER

PT J

AU Wang, SL

Li, HX

Zhang, XF

Yi, S

AF Wang, S. L.

Li, H. X.

Zhang, X. F.

Yi, S.

TI Effects of Cr contents in Fe-based bulk metallic glasses on the glass

forming ability and the corrosion resistance

SO MATERIALS CHEMISTRY AND PHYSICS

AB Fe-based bulk metallicglasses of Fe(69.9-x),C(7.1)Si(3.3)B(5.5)P(8.7)Cr(x)Mo(2.5)Al(2.0)Co(1.0) (x = 0.0, 2.3-12.3) with high glass forming ability and good corrosion resistance were fabricated using industrial raw materials. Class forming ability of the Fe-based bulk metallic glasses tends to decrease with the Cr content while the corrosion, resistance increases with the Cr content. A homogeneous passive layer on the amorphous sample with 12.3 at% Cr can be formed leading to superior corrosion resistance of the amorphous sample to an austenitic stainless steel (SUS304) in the 0.5 M H(2)SO(4) and 1N HCl solutions at 298 K. Fe-based bulk metallic glasses with an optimum combination of glass forming ability and corrosion resistance can be produced in large quantities through a systematic control of the Cr content for extensive practical applications. (C) 2008 Elsevier B.V. All rights reserved.

SN 0254-0584

PD FEB 15

PY 2009

VL 113

IS 2-3

BP 878

EP 883

DI 10.1016/j.matchemphys.2008.08.057

UT WOS:000263249500067

ER

PT J

AU Perriere, L

Thai, MT

Tusseau-Nenez, S

Ochin, P

Bletry, M

Champion, Y

AF Perriere, L.

Thai, M. -T.

Tusseau-Nenez, S.

Ochin, P.

Bletry, M.

Champion, Y.

TI Spark plasma sintering for metallic glasses processing

SO REVUE DE METALLURGIE-CAHIERS D INFORMATIONS TECHNIQUES

AB Development of metallic glasses is hindered by the difficulties in manufacturing bulk parts. We report on the preparation of glassy alloys using the spark plasma sintering of gas atomized powders. The Zr57Cu20Al10Ni8Ti5 alloy processed has a high glass forming ability. Densification is obtained at the glass transition temperature (390 degrees C) under high pressure (approximate to 500 MPa). Systematic study of the effect of powder particles size and sintering time is performed through structural and thermal analyses. Local and partial devitrification of the amorphous alloy is detected, resulting from local temperature overshoots. From these analyses, an approach of the spark plasma sintering mechanism of metallic glasses is proposed.

RI Perriere, Loic/A-4415-2013

OI Perriere, Loic/0000-0002-8635-5069; Bletry, Marc/0000-0003-2509-9847

SN 0035-1563

PY 2012

VL 109

IS 1

BP 5

EP 10

DI 10.1051/meta1/2011074

UT WOS:000302737200002

ER

PT J

AU Eckert, J

AF Eckert, J

TI Mechanical alloying of highly processable glassy alloys

SO MATERIALS SCIENCE AND ENGINEERING A-STRUCTURAL MATERIALS PROPERTIES

MICROSTRUCTURE AND PROCESSING

CT 9th International Conference on Rapidly Quenched and Metastable

Materials

CY AUG 25-30, 1996

CL BRATISLAVA, SLOVAKIA

SP Off Strategy & Dev Soc Sci & Technol, European Commiss, Directorate Gen XII, AlliedSignal Inc, US, Deut Phys Gesell, Int Union Pure & Appl Phys, JOEL Ltd, Japan, Alps Elect Co Ltd, Japan, Perkin Elmer GmbH

AB Glasses are generally produced from the undercooled liquid state by rapid quenching methods or quasi-statically at slow cooling by the effective control of heterogeneous nucleation sites. For metallic systems the latter method recently has led to the development of multicomponent metallic glasses with large glass forming ability and a wide supercooled liquid region before crystallization. Large-scale bulk samples of such alloys can be produced by conventional casting techniques. Alternatively, glass formation can be achieved by solid-state processing without passing through the liquid state. This crystal-to-glass transition is observed when a sufficiently high energy level is reached and kinetic conditions prevent the establishment of equilibrium. Hence, mechanical alloying as a special form of solid-state reaction technique and subsequent consolidation of the resulting powders above the glass transition temperature can be used to prepare bulk metallic glasses via the powder metallurgy route. The glass formation and the thermal stability of mechanically alloyed glassy alloys are compared with data for melt quenched samples showing that basically the same glassy state can be reached approaching it from the liquid or the solid state. Special emphasis is given to the glass forming ranges achievable by the different techniques and to the influence of impurities. Results for consolidated bulk samples are presented and compared with data for cast bulk specimens. (C) 1997 Elsevier Science S.A.

SN 0921-5093

PD JUN 15

PY 1997

VL 226

BP 364

EP 373

DI 10.1016/S0921-5093(96)10646-8

UT WOS:A1997XJ84700069

ER

PT J

AU Bobrov, OP

Khonik, VA

Laptev, SN

AF Bobrov, OP

Khonik, VA

Laptev, SN

TI Isochronal tensile stress relaxation of a bulk metallic glass

SO SCRIPTA MATERIALIA

AB Isochronal stress relaxation of bulk Zr52.5Ti5Cu17.9Ni14.6Al10 starts at about 400 K and ends below the glass transition temperature. The kinetics of stress relaxation has been analytically described to be a result of stress-oriented irreversible structural relaxation with distributed activation energies. Satisfactory agreement between experimental and calculated data has been shown. (C) 2003 Acta Materialia Inc. Published by Elsevier Ltd. All rights reserved.

RI Khonik, Vitaly/A-5888-2009

SN 1359-6462

PD FEB

PY 2004

VL 50

IS 3

BP 337

EP 341

DI 10.1016/j.scriptamat.2003.10.017

UT WOS:000186671100005

ER

PT J

AU Bobrov, OP

Csach, K

Khonik, SV

Kitagawa, K

Lyakhov, SA

Yazvitsky, MY

Khonik, VA

AF Bobrov, O. P.

Csach, K.

Khonik, S. V.

Kitagawa, K.

Lyakhov, S. A.

Yazvitsky, M. Yu.

Khonik, V. A.

TI The recovery of structural relaxation-induced viscoelastic creep strain

in bulk and ribbon Pd40Cu30Ni10P20 glass

SO SCRIPTA MATERIALIA

AB The ability of metallic glasses to undergo homogeneous viscous flow is known to decrease strongly as a result of structural relaxation and this effect is commonly considered to be irreversible. The present paper employs creep measurements to show that this ability can be significantly recovered by short-term preannealing in the glass transition region. This technique provides a unique possibility to recover the viscoelastic properties of metallic glasses after these have deteriorated greatly via structural relaxation. (c) 2006 Acta Materialia Inc. Published by Elsevier Ltd. All rights reserved.

RI Khonik, Vitaly/A-5888-2009

SN 1359-6462

PD JAN

PY 2007

VL 56

IS 1

BP 29

EP 32

DI 10.1016/j.scriptamat.2006.08.056

UT WOS:000242060400008

ER

PT J

AU Gong, LD

Chan, KC

Yu, P

Liu, L

Wang, G

AF Gong, L. D.

Chan, K. C.

Yu, P.

Liu, L.

Wang, G.

TI Thermoplastic gas pressure forming of bulk metallic glass sheets under

biaxial tension

SO JOURNAL OF ALLOYS AND COMPOUNDS

AB The deformation behaviour and structural evolution of bulk metallic glass sheets were studied under biaxial tension in supercooled liquid state. The sheets were gas pressure formed into hemispherical and semi-ellipsoid domes using dies with aspect ratios of 1:1 and 3:2, respectively, at the optimum temperature of 676 K. The structural evolution of the metallic glass sheets was examined after deformation. The stress distributions of the bulging sheets are calculated employing a finite element model. It was found that the stress state of biaxial tension accelerates nanocrystallization, which leads to an increase in the hardness of metallic glass. The gas pressure applied, bulging time, and temperature are the principal factors involved in the formation of products. (C) 2009 Elsevier B.V. All rights reserved.

RI Chan, K.C./A-2311-2014; Wang, Gang/K-2630-2012

OI Chan, K.C./0000-0002-6173-5532;

SN 0925-8388

PD SEP 18

PY 2009

VL 484

IS 1-2

BP 159

EP 163

DI 10.1016/j.jallcom.2009.05.041

UT WOS:000271334900032

ER

PT J

AU Takeuchi, A

AF Takeuchi, Akira

TI Alloy Designs for High-Entropy Alloys, Bulk Metallic Glasses and

High-Entropy Bulk Metallic Glasses

SO JOURNAL OF THE JAPAN INSTITUTE OF METALS

AB This paper describes high-entropy alloys (HEAs), bulk metallic glasses (BMGs) and high-entropy bulk metallic glasses (HE-BMGs) for their alloy design based on the quantities corresponding to the difference in atomic size and heat of mixing. The contents started with describing the differences between HEAs and BMGs, followed by the features of HE. BMGs by focusing on the universalities underlying these three alloys. The significance of thermodynamics with ability to describe the necessary quantities for designing these alloys as a function of alloy composition was argued by referring to the early studies. Furthermore, future prospects of these alloys in terms of alloy design are described for their further progress in researches.

RI Takeuchi, Akira/A-3619-2010

OI Takeuchi, Akira/0000-0002-7246-8644

SN 0021-4876

EI 1880-6880

PY 2015

VL 79

IS 4

BP 157

EP 168

DI 10.2320/jinstmet.J2014046

UT WOS:000355175900001

ER

PT J

AU Caron, A

Zhang, QS

Minkow, A

Zadorozhnyy, VA

Fukuhara, M

Fecht, HJ

Louzguine-Luzgin, DV

Inoue, A

AF Caron, A.

Zhang, Q. S.

Minkow, A.

Zadorozhnyy, V. A.

Fukuhara, M.

Fecht, H. -J.

Louzguine-Luzgin, D. V.

Inoue, A.

TI Mesostructural effects on the mechanical properties of Zr-based bulk

metallic glasses

SO MATERIALS SCIENCE AND ENGINEERING A-STRUCTURAL MATERIALS PROPERTIES

MICROSTRUCTURE AND PROCESSING

AB Structural and elemental mapping analyses were applied to characterize the mesostructure in the bulk metallic glass-forming Zr60FexCu30-xAl10 and Zr60+xFe5Cu25-xAl10 alloys. It is found that the observed meso-/microstructure is adjustable by the addition of Fe and tunes the acoustic anelasticity of the samples, while the relaxation time derived from our ultrasound measurements inversely scales with the mechanical properties and ductility of the alloys. The measurements also give data for the thermal diffusivity and coefficient of thermal expansion. Together with mechanical compression tests the results are further discussed on the basis of the thermo-elastic effect and the related role of heat dissipation on the propagation of shear bands in metallic glasses during plastic deformation. (c) 2012 Elsevier B.V. All rights reserved.

RI Inoue, Akihisa/E-5271-2015; Zadorozhnyy, Vladislav/G-9616-2011;

LOUZGUINE, Dmitri/D-2492-2010; Caron, Arnaud/B-6463-2010

OI LOUZGUINE, Dmitri/0000-0001-5716-4987; Caron, Arnaud/0000-0003-0985-7441

SN 0921-5093

EI 1873-4936

PD OCT 15

PY 2012

VL 555

BP 57

EP 62

DI 10.1016/j.msea.2012.06.033

UT WOS:000308387200008

ER

PT J

AU Zhu, ZW

Zhang, HF

Pan, DG

Sun, WS

Hu, ZQ

AF Zhu, Zhengwang

Zhang, Haifeng

Pan, Dagang

Sun, Wensheng

Hu, Zhuangqi

TI Fabrication of binary Ni-Nb bulk metallic glass with high strength and

compressive plasticity

SO ADVANCED ENGINEERING MATERIALS

RI Zhu, Zheng-Wang/D-2799-2017

SN 1438-1656

PD OCT

PY 2006

VL 8

IS 10

BP 953

EP 957

DI 10.1002/adem.200600105

UT WOS:000241785000007

ER

PT J

AU Hasegawa, M

AF Hasegawa, M.

TI Hydrogen-induced high damping of bulk metallic glasses

SO MATERIALS SCIENCE AND ENGINEERING A-STRUCTURAL MATERIALS PROPERTIES

MICROSTRUCTURE AND PROCESSING

CT 15th International Conference on Internal Friction and Mechanical

Spectroscopy (ICIFMS-15)

CY JUL 20-25, 2008

CL Perugia, ITALY

SP Perugia Univ, Phys Dept, CNISM

AB There are two important topics concerned with the recent researches on the damping materials of hydrogenated metallic glasses (HMGs). One is the mechanism of the high hydrogen-induced internal friction of HMGs. The other is the materials processing of "bulk" HMGs for engineering. This article describes the summary of our recent studies on these topics. The first one is closely related to the local structure of the metallic glasses. Therefore, our recent results on the intermediate-range local structure of the simple two Zr-based metallic glasses are described, which has been clarified by the Voronoi analysis using the experimental data of the neutron diffraction measurements. The hydrogen-induced internal friction of HMGs is also discussed on the basis of these recent results of the local structure of the metallic glasses. In terms of the second topic, the first successful preparation of heavily hydrogenated Zr-based bulk HIVIG rods without hydrogen-induced surface embrittlement is described. They are prepared by a powder-compact-melting and liquid-casting process using Zr-Al-Ni-Cu metallic glass and ZrH(2) powders as the starting materials. It has been found that they have high damping properties. (C) 2009 Elsevier B.V. All rights reserved.

SN 0921-5093

PD SEP 25

PY 2009

VL 521-22

BP 354

EP 358

DI 10.1016/j.msea.2008.10.078

UT WOS:000270330700087

ER

PT J

AU Meng, LK

Si, Y

Suo, ZY

Wu, XF

AF Meng Likai

Si Yi

Suo Zhongyuan

Wu Xiaofeng

TI Preparation and mechanical properties of ternary Ti-Cu-Ni bulk metallic

glasses

SO RARE METAL MATERIALS AND ENGINEERING

AB The bulk metallic glass (BMG) with a diameter of 2 mm at least has been successfully fabricated by a conventional Cu-mold casting method for Ti50Cu42Ni8 alloy. The glass-forming ability (GFA), the thermal stability and mechanical properties have been investigated for the ternary Ti50Cu50-xNix (x=5, 8, 11) alloys by XRD, DSC and SEM. The results show that the Ti50Cu42Ni8 BMG exhibits a wide undercooled liquid region Delta T-x of 57 K, a high reduced glass transition temperature T-rg of 0.565 and a compressive fracture strength of 2008 MPa.

SN 1002-185X

PD JUL

PY 2008

VL 37

IS 7

BP 1163

EP 1166

UT WOS:000258345500008

ER

PT J

AU Na, YS

Lee, JH

AF Na, YS

Lee, JH

TI Interpretation of viscous deformation of Zr-based bulk metallic glass

alloys based on Nabarro-Herring creep model

SO METALS AND MATERIALS INTERNATIONAL

AB Superplastic-like viscous deformation of bulk metallic glass alloys around the glass transition temperature (Tg) was analyzed based on the Nabarro-Herring creep model, a classical creep model, where the diffusional motion of atoms or vacancies through the lattice (atomic configuration) is considered. The amorphous matrix of bulk metallic glasses that has a randomly-packed atomic configuration was assumed to behave in a manner similar to the grain boundary in polycrystalline metals so as to approximate the diffusivity of the major constituent element. In spite of rough approximation of the parameters in the Nabarro-Herring creep equation, a reasonable value of the diffusion path (d) could be obtained from the experimentally-obtained metal flow data, including the stead), state stress and the strain rate. Due to the absence of vacancy sources such as grain boundaries in homogeneous metallic glasses, the diffusion path, which, in polycrystalline materials, generally is the average distance between vacancy sources such as grain boundaries, was considered in this work as the average distance between tunneling centers in bulk metallic glass alloys. The calculated diffusion path was comparable to the density of tunneling centers around Tg, proposed by M. H. Cohen and CL S. Grest based on free volume theory. The calculated diffusion path showed monotonous decrease with temperature over Tg for Zr-based bulk metallic glass alloys. Based on this analysis, a schematic model for viscous deformation of bulk metallic glass was proposed.

SN 1598-9623

PD APR

PY 2006

VL 12

IS 2

BP 115

EP 120

DI 10.1007/BF03027466

UT WOS:000237302600003

ER

PT J

AU Xi, XK

Wang, RJ

Zhao, DQ

Pan, MX

Wang, WH

AF Xi, XK

Wang, RJ

Zhao, DQ

Pan, MX

Wang, WH

TI Glass-forming Mg-Cu-RE (RE = Gd, Pr, Nd, Tb, Y, and Dy) alloys with

strong oxygen resistance in manufacturability

SO JOURNAL OF NON-CRYSTALLINE SOLIDS

AB One of the unsolved problems for the manufacturability and the applications of bulk metallic glasses is that their glass-forming ability is very sensitive to the preparation vacuum and impurity of components because oxygen in the environments would markedly deteriorate the glass-forming ability. Here we report that the addition of rare earth elements can significantly improve the glass-forming ability and manufacturability of Mg-based alloys. The Mg-based glass-forming alloys can withstand very low vacuum in preparation process. The beneficial effects of the Gd addition on the glass-forming ability and oxygen resistance during the Mg-based glass formation are explored. (C) 2004 Elsevier B.V. All rights reserved.

SN 0022-3093

PD OCT 1

PY 2004

VL 344

IS 3

BP 105

EP 109

DI 10.1016/j.jnoncrysol.2004.07.056

UT WOS:000224628100001

ER

PT J

AU Yeh, JW

Chen, SK

Lin, SJ

Gan, JY

Chin, TS

Shun, TT

Tsau, CH

Chang, SY

AF Yeh, JW

Chen, SK

Lin, SJ

Gan, JY

Chin, TS

Shun, TT

Tsau, CH

Chang, SY

TI Nanostructured high-entropy alloys with multiple principal elements:

Novel alloy design concepts and outcomes

SO ADVANCED ENGINEERING MATERIALS

SN 1438-1656

PD MAY

PY 2004

VL 6

IS 5

BP 299

EP 303

DI 10.1002/adem.200300567

UT WOS:000221996000009

ER

PT J

AU Ohta, M

Berlev, AE

Khonik, VA

Kitagawa, K

AF Ohta, M

Berlev, AE

Khonik, VA

Kitagawa, K

TI Isothermal creep of bulk glassy Zr52.5Ti5Cu17.9Ni14.6Al10 below T-g

SO PHILOSOPHICAL MAGAZINE

AB The results of an isothermal creep investigation of Zr52.5Ti5Cu17.9Ni14.6Al10 bulk metallic glass at temperatures below the glass transition temperature are presented. The long-time (t > (2-4) x 10(3) s) creep regularities were found to be the same as those known for ribbon metallic glasses, in spite of the pronounced difference in the production quenching rates. It is argued that creep behaviour of bulk metallic glass is determined by the rate of irreversible structural relaxation. The apparent activation energy spectrum reconstructed from isothermal creep measurements agrees well with that determined from linear heating creep data.

RI Khonik, Vitaly/A-5888-2009

SN 1478-6443

PD OCT 21

PY 2003

VL 83

IS 30

BP 3463

EP 3471

DI 10.1080/14786430310001613174

UT WOS:000186684700004

ER

PT J

AU Li, G

Wang, YQ

Wang, LM

Gao, YP

Zhang, RJ

Zhan, ZJ

Sun, LL

Zhang, J

Wang, WK

AF Li, G

Wang, YQ

Wang, LM

Gao, YP

Zhang, RJ

Zhan, ZJ

Sun, LL

Zhang, J

Wang, WK

TI Wear behavior of bulk Zr41Ti14Cu12.5Ni10Be22.5 metallic glasses

SO JOURNAL OF MATERIALS RESEARCH

AB The wear behavior of bulk Zr41Ti14Cu12.5Ni10Be22.5 metallic glasses has been studied using sliding wear tests and scanning electron microscopy in both as-prepared and annealed samples. It was found that the wear resistance of differently processed samples increases in the following order: crystallized state; as-prepared state; relaxed state. The thermal stability of worn samples was also investigated by means of differential scanning calorimetry. Under the experiment conditions, no sliding wear-induced crystallization is observed in either as-prepared or relaxed samples indicating good thermal stability of the bulk metallic glasses.

SN 0884-2914

PD AUG

PY 2002

VL 17

IS 8

BP 1877

EP 1880

DI 10.1557/JMR.2002.0276

UT WOS:000177208800001

ER

PT J

AU Kokubun, R

Wang, W

Zhu, SL

Xie, GQ

Ichinose, S

Itoh, S

Takakuda, K

AF Kokubun, Ryo

Wang, Wei

Zhu, Shengli

Xie, Guoqiang

Ichinose, Shizuko

Itoh, Soichiro

Takakuda, Kazuo

TI In vivo evaluation of a Ti-based bulk metallic glass alloy bar

SO BIO-MEDICAL MATERIALS AND ENGINEERING

AB Ti-based bulk metallic glasses are reported with high strength, low Young modulus and high corrosion resistance, suggesting their potentials in biomedical applications. However a thorough in vivo evaluation of its biocompatibilities has not been conducted yet. In this study, we implanted bars of Ti-based bulk metallic glass in the femoral bone of rats, followed up local tissue reaction as well as its component ions' diffusion in local area and whole body. The Ti-based BMG (Ti40Zr10Cu34Pd14Sn2) alloy exhibited favorable features of both high strength and high elasticity. In vivo implant evaluation showed that it has a good tissue compatibility, equivalent bone integration and bonding ability with Ti sample. No component ion diffusion was detected up to 3 months post implantation. The possibility and efficacy of its use for bone implant is confirmed. Thus further long term implant study is recommended.

RI Xie, Guoqiang/A-8619-2011; Zhu, Shengli/D-5281-2009

OI Zhu, Shengli/0000-0002-0190-2626

SN 0959-2989

EI 1878-3619

PY 2015

VL 26

IS 1-2

BP 9

EP 17

DI 10.3233/BME-151546

UT WOS:000363344000002

PM 26484551

ER

PT J

AU Rezaee-Bazzaz, A

Marvi-Mashhadi, M

Haddad-Sabzevar, M

AF Rezaee-Bazzaz, A.

Marvi-Mashhadi, M.

Haddad-Sabzevar, M.

TI Study of mechanical deformation of Zr55Cu30Al10Ni5 bulk metallic glass

through instrumented indentation

SO MATERIALS SCIENCE AND ENGINEERING A-STRUCTURAL MATERIALS PROPERTIES

MICROSTRUCTURE AND PROCESSING

AB Instrumented sharp indentation experiments using both conical and Vickers diamond pyramidal indenters were carried out to study deformation characteristics of Zr55Cu30Al10Ni5 bulk metallic glass. Finite element simulations of instrumented indentation were also performed to formulate an overall constitutive response. Comparing the experimentally obtained results with the finite element predictions, it can be stated that mechanical deformation of the bulk metallic glass can be described well by both Mohr-Coulomb and Drucker-Prager constitutive criteria. Using these criteria, the extent of material pile-up observed around the indenter was also estimated very well. (C) 2011 Elsevier B.V. All rights reserved.

OI rezaee-bazzaz, abolfazl/0000-0001-9042-9051

SN 0921-5093

PD AUG 15

PY 2011

VL 528

IS 21

BP 6630

EP 6635

DI 10.1016/j.msea.2011.05.018

UT WOS:000293117100038

ER

PT J

AU Li, PY

Wang, G

Ding, D

Shen, J

AF Li, Peiyou

Wang, Gang

Ding, Ding

Shen, Jun

TI Glass forming ability and thermodynamics of new Ti-Cu-Ni-Zr bulk

metallic glasses

SO JOURNAL OF NON-CRYSTALLINE SOLIDS

AB The quaternary Ti-Cu-Ni-Zr bulk metallic glasses are developed through proportionally mixing of the binary eutectic phases of Ti27Cu73, Ti76Ni24, Zr38Cu62 and Zr76Ni24. The novel Ti-Cu-based bulk metallic glasses with the critical diameter of 4 mm are successfully developed by the modification of the proportion of these eutectic units. The different glass forming ability (GFA) is discussed in a frame of thermodynamics. The GFA of these quatemary alloys correlate well with the reduced glass transition temperature (T-rg), the gamma value, the reduced ideal glass transition temperature (T-K/T-m) (where T-K is the ideal glass transition temperature) as well as the ratio between specific heat and entropy differences Delta C-p(m)/Delta S-m at the melting temperature (T-m). (C) 2012 Elsevier B.V. All rights reserved.

SN 0022-3093

PD DEC 1

PY 2012

VL 358

IS 23

BP 3200

EP 3204

DI 10.1016/j.jnoncrysol.2012.08.005

UT WOS:000312764300024

ER

PT J

AU Bian, Z

Inoue, A

AF Bian, Z

Inoue, A

TI Ultra-low glass transition temperatures in Ce-based bulk metallic

glasses

SO MATERIALS TRANSACTIONS

AB New (Ce0.72Cu0.28)(100-x-y)Al-x(Ga,Zn)(y) bulk metallic glasses (BMGs) with ultra-low glass transition temperatures and large supercooled liquid regions (Delta T-x) were developed. The addition of Al element into Ce-Cu alloys improves significantly glass formation ability (GFA) of the alloys and the maximum value of Delta T-x is 63 K. The lowest glass transition temperature (T-g) was found in the (Ce0.72Cu0.28)(97.5)Al-2.5 BMG alloy and the value is 326 K. The addition of Ga and Zn elements into Ce-Cu-Al alloys has no obvious role in the decrease of T, However, T, has a close relation to Cc content and decreases with increasing Cc content. The activation energies of the glass transition are 136 kJ(.)mol(-1) for the (Ce0.72Cu0.28)(97.5)Al-2.5 glassy alloy and 145 kJ(.)mol(-1) for the Ce62.5Cu15Al11.5Ni10 glassy alloys, respectively. The successful fabrication of Ce-based BMGs with ultra-low T-g and large Delta T-x can further extend the potential application of bulk metallic glasses in some special industry fields.

RI Inoue, Akihisa/E-5271-2015

SN 1345-9678

EI 1347-5320

PD AUG

PY 2005

VL 46

IS 8

BP 1857

EP 1860

DI 10.2320/matertrans.46.1857

UT WOS:000231476600025

ER

PT J

AU Clausen, B

Lee, SY

Ustundag, E

Aydiner, CC

Conner, RD

Bourke, MAM

AF Clausen, B

Lee, SY

Ustundag, E

Aydiner, CC

Conner, RD

Bourke, MAM

TI Compressive yielding of tungsten fiber reinforced bulk metallic glass

composites

SO SCRIPTA MATERIALIA

AB In-situ uniaxial compression tests were conducted on four tungsten fiber reinforced bulk metallic glass matrix composites using neutron diffraction. The results were interpreted with a finite element model. Both phases were seen to approximately obey the von Mises yield criterion. The fibers were observed to yield first and then transfer load to the matrix. (C) 2003 Acta Materialia Inc. Published by Elsevier Science Ltd. All rights reserved.

RI Aydiner, Cahit/O-9618-2017; Ustundag, Ersan/C-1258-2009; Clausen,

Bjorn/B-3618-2015

OI Aydiner, Cahit/0000-0001-8256-6742; Ustundag, Ersan/0000-0002-0812-7028;

Clausen, Bjorn/0000-0003-3906-846X

SN 1359-6462

PD JUL

PY 2003

VL 49

IS 2

BP 123

EP 128

DI 10.1016/S1359-6462(03)00237-9

UT WOS:000183047500003

ER

PT J

AU Qin, CL

Zhang, W

Zhang, QS

Asami, K

Inoue, A

AF Qin, C. L.

Zhang, W.

Zhang, Q. S.

Asami, K.

Inoue, A.

TI Electrochemical properties and surface analysis of Cu-Zr-Ag-Al-Nb bulk

metallic glasses

SO JOURNAL OF ALLOYS AND COMPOUNDS

CT 14th International Symposium on Metastable and Nano-Materials

CY AUG 26-30, 2007

CL Corfu, GREECE

AB The electrochemical properties and surface characteristics of Cu-Zr-Ag-Al-Nb bulk metallic glasses (BMGs) were investigated. The alloys exhibit excellent corrosion resistance after immersion in 1 N H2SO4 and 1 N NaOH. The corrosion rates of the alloys in chloride-ion-containing solutions significantly decrease by alloying with Nb element. The formation of Zr- and Nb-enriched surface films could be responsible for the high corrosion resistance. (C) 2008 Elsevier BY. All rights reserved.

RI Inoue, Akihisa/E-5271-2015; Qin, Chunling/A-4846-2010

SN 0925-8388

EI 1873-4669

PD AUG 26

PY 2009

VL 483

IS 1-2

BP 317

EP 320

DI 10.1016/j.jallcom.2008.07.157

UT WOS:000270619600077

ER

PT J

AU Zhang, ZF

Eckert, J

AF Zhang, Zhe-Feng

Eckert, Juergen

TI The physical nature of materials strengths

SO ADVANCED ENGINEERING MATERIALS

RI Zhang, Zhefeng/A-9732-2010; Zhang, BMG/C-6151-2014

SN 1438-1656

PD MAR

PY 2007

VL 9

IS 3

BP 143

EP 146

DI 10.1002/adem.200600232

UT WOS:000245190400005

ER

PT J

AU Aboki, TA

AF Aboki, Tiburce A.

TI Development of Fe-B Based Bulk Metallic Glasses: Morphology of Residual

Phases in Fe50Ni16Mo6B18Zr10 Glass

SO METALS

AB Iron-boron based bulk metallic glasses (BMG) development has been initiated using Fe40Ni38Mo4B18 as precursor. Addition of zirconium up to 10 atomic % along with the reduction of Ni proportion improves the glass forming ability (GFA), which is optimum when Ni is suppressed in the alloy. However melting instability occurred during the materials fabrication resulting in the formation of residual crystalline phases closely related to the amorphous phase. Microstructure study shows an evolution from amorphous structure to peculiar acicular structure, particularly for Fe50Ni16Mo6B18Zr10, suggesting the amorphous structure as interconnected atomic sheets like "atomic mille feuilles" whose growth affects the alloys' GFA.

SN 2075-4701

PD JUN

PY 2013

VL 3

IS 2

BP 159

EP 177

DI 10.3390/met3020159

UT WOS:000343292700001

ER

PT J

AU Song, M

He, YH

AF Song Min

He Yue-hui

TI A unified criterion for yielding behavior of metallic glasses

SO JOURNAL OF CENTRAL SOUTH UNIVERSITY OF TECHNOLOGY

AB The yield behavior of metallic glasses was studied. Three yield criteria, including von Mises yield criterion, Mohr-Coulomb yield criterion and the unified yield criterion were used to describe the yield phenomena of the metallic glasses. Two classes of the experimental data were chosen to draw the yield loci using the unified yield criterion. It is shown that the unified yield criterion can be used to describe the yield behavior of the metallic glasses no matter whether the metallic glasses show strength-different effect or non-strength-different effect. Almost all the widely accepted yield criteria are the subsets of the unified yield criterion if the intermediate principle stress and/or the intermediate principle shear stress are not considered at all.

RI Song, Min/C-3730-2013

OI Song, Min/0000-0002-3197-4647

SN 1005-9784

PD FEB

PY 2011

VL 18

IS 1

BP 1

EP 5

DI 10.1007/s11771-011-0650-5

UT WOS:000287267900001

ER

PT S

AU Jiang, JZ

AF Jiang, JZ

BE Andersson, Y

Mittemeijer, EJ

Welzel, U

TI Phase transformations in metallic glasses

SO EUROPEAN POWDER DIFFRACTION EPDIC 8

SE MATERIALS SCIENCE FORUM

CT 8th European Powder Diffraction Conference

CY MAY 23-26, 2002

CL Uppsala, SWEDEN

AB The effects of pressure on the crystallization behaviour of metallic glasses have been discussed from both theoretical and experimental aspects. It reveals that the applied pressure strongly affects the crystallization processes of the metallic glasses. The model of competing processes of the thermodynamic potential barrier of nucleation and the diffusion activation energy under pressure is able to explain the influence of pressure on crystallization processes of the metallic glasses investigated. We also found that the densification effect induced by pressure only plays a minor role for crystallization of the metallic glasses investigated.

SN 0255-5476

BN 0-87849-935-0

PY 2004

VL 443-4

BP 211

EP 216

DI 10.4028/www.scientific.net/MSF.443-444.211

UT WOS:000189421300044

ER

PT J

AU Han, G

Qiang, JB

Li, FW

Yuan, L

Quan, SG

Wang, Q

Wang, YM

Dong, C

Haussler, P

AF Han, Guang

Qiang, Jianbing

Li, Fengwei

Yuan, Liang

Quan, Shiguan

Wang, Qing

Wang, Yingmin

Dong, Chuang

Haeussler, Peter

TI The e/a values of ideal metallic glasses in relation to cluster formulae

SO ACTA MATERIALIA

AB The electrons per atom ratio eta is an important factor in discussing the stabilization mechanism of metallic glasses. However, the eta assignment is complicated by hybridization effects, particular in transition metal systems. We have previously proposed the "cluster-plus-glue-atom" model for metallic glasses and the relevant cluster formula [cluster](glue atom)(x), x similar to, 1 or 3. In the present paper, this model is combined with Haussler's global-resonance model into a so-called cluster-resonance model. This model allows the calculation of the Fermi vector and hence the effective eta of an ideal metallic glass. By assuming an ideal dense packing of the clusters, the atomic density can be obtained and eta becomes associated with the number of atoms in the unit cluster formula Z. Experiments are carried out to verify these correlations. Alloys like Cu8Zr5Al, Ni4Zr9Al2 and [Ni7Nb5Zr]Ni-3, possessing locally the highest glass-forming abilities, are interpreted by cluster formulae, with their Zs being determined by their c/a values. Such verifications not only validate the proposed eta calculation schemes and the models behind them, but also support the ideal metallic glass feature of bulk metallic glasses with high glass-forming abilities. (C) 2011 Acta Materialia Inc. Published by Elsevier Ltd. All rights reserved.

SN 1359-6454

EI 1873-2453

PD SEP

PY 2011

VL 59

IS 15

BP 5917

EP 5923

DI 10.1016/j.actamat.2011.05.065

UT WOS:000294086900005

ER

PT J

AU Hirata, A

Hirotsu, Y

Matsubara, E

AF Hirata, Akihiko

Hirotsu, Yoshihiko

Matsubara, Eiichiro

TI Crystallization behaviours around the glass transition temperature in an

amorphous Fe-Nb-B alloy

SO INTERMETALLICS

AB Crystallization behaviours around the glass transition temperature in an amorphous Fe(70)Nb(10)B(20) alloy were investigated by means of transmission electron microscopy. Dense bcc-Fe nanocrystals initially appeared as the primary phase, followed by the dense formation of the (Fe,Nb)(23)B(6) nanocrystalline phase. The bcc-Fe nanocrystals were formed even by annealing at a temperature that is 38 K lower than the glass transition temperature. A difference of the low temperature behaviours between the present conventional amorphous alloy and a bulk metallic glass was discussed. (C) 2009 Elsevier Ltd. All rights reserved.

RI Hirata, Akihiko/A-4850-2010

SN 0966-9795

PD OCT

PY 2009

VL 17

IS 10

BP 796

EP 801

DI 10.1016/j.intermet.2009.03.007

UT WOS:000267974600005

ER

PT J

AU Wang, G

Shen, J

Sun, JF

Zhou, BD

Gerald, JDF

Llewellyn, DJ

Stachurski, ZH

AF Wang, G

Shen, J

Sun, JF

Zhou, BD

Gerald, JDF

Llewellyn, DJ

Stachurski, ZH

TI Isothermal nanocrystallization behavior of

Zr41.25Ti13.75Ni10Cu12.5Be22.5 bulk metallic glass in the supercooled

liquid region

SO SCRIPTA MATERIALIA

AB The isothermal crystallization of Zr41.25Ti13.75Ni10Cu12.5Be22.5 (at.%) bulk metallic glass in the supercooled liquid region is studied by X-ray diffraction, differential scanning calorimetry and transmission electron microscopy. The kinetics and the underlying mechanisms for the nucleation and growth of the crystalline phases precipitated in the two-stage crystallization process are discussed. (c) 2005 Acta Materialia Inc. Published by Elsevier Ltd. All rights reserved.

RI Fitz Gerald, John/A-8320-2008; Wang, Gang/K-2630-2012

OI Stachurski, Zbigniew/0000-0002-1317-0178

SN 1359-6462

PD SEP

PY 2005

VL 53

IS 6

BP 641

EP 645

DI 10.1016/j.scriptamat.2005.05.038

UT WOS:000230975500005

ER

PT J

AU Saida, J

Yamada, R

Wakeda, M

AF Saida, Junji

Yamada, Rui

Wakeda, Masato

TI Recovery of less relaxed state in Zr-Al-Ni- Cu bulk metallic glass

annealed above glass transition temperature

SO APPLIED PHYSICS LETTERS

AB The relaxation state of metallic glass is determined by the cooling rate at low temperatures in a supercooled liquid. Based on this result, we can control the relaxation state of Zr55Al10Ni5Cu30 bulk metallic glass by recovery annealing just above the glass transition temperature (T-g). We rejuvenate the relaxation state for approximately 50% in the enthalpy of relaxation at a cooling rate of 4.4K/s after annealing, as compared with that of the as-cast state. Mechanical softening also occurred upon the rejuvenation. The results suggest another method of controlling the structure of metallic glasses to improve their properties. (C) 2013 AIP Publishing LLC.

OI Wakeda, Masato/0000-0002-6377-1318

SN 0003-6951

EI 1077-3118

PD NOV 25

PY 2013

VL 103

IS 22

AR 221910

DI 10.1063/1.4835076

UT WOS:000327696300031

ER

PT J

AU Li, S

Wang, RJ

Pan, M

Zhao, DQ

Wang, WH

AF Li, S

Wang, RJ

Pan, M

Zhao, DQ

Wang, WH

TI Heavy rare earth based bulk metallic glasses with high thermal stability

SO INTERMETALLICS

AB We report a family of ternary Gd-Al-Co alloys based on the heavy gadolinium rare earth element can be readily cast into fully glassy rod by a conventional casting method. It is found that the bulk metallic glasses (BMGs) have much high thermal stability (i.e. high glass transition temperature and crystallization temperature) and high moduli compared with those of other known rare-earth based BMGs. It is confirmed that, in addition to the strong chemical interaction among the components, the high bulk modulus of the base component in the BMGs is dominantly responsible for the high thermal stability. The thermal stability is correlated with the bulk modulus of the base element in the rare earth based BMGs. (c) 2005 Published by Elsevier Ltd.

SN 0966-9795

PD JUN

PY 2006

VL 14

IS 6

BP 592

EP 595

DI 10.1016/j.intermet.2005.10.002

UT WOS:000235850900002

ER

PT J

AU Vella, PC

Dimov, SS

Brousseau, E

Whiteside, BR

Grant, CA

Tuinea-Bobe, CL

AF Vella, Pierre C.

Dimov, Stefan S.

Brousseau, Emmanuel

Whiteside, Ben R.

Grant, Colin A.

Tuinea-Bobe, Cristina-Luminita

TI A new process chain for producing bulk metallic glass replication

masters with micro- and nano-scale features (vol 76, pg 523, 2015)

SO INTERNATIONAL JOURNAL OF ADVANCED MANUFACTURING TECHNOLOGY

SN 0268-3768

EI 1433-3015

PD JUL

PY 2016

VL 85

IS 1-4

BP 941

EP 941

DI 10.1007/s00170-016-8409-7

UT WOS:000378875500079

ER

PT J

AU Khalifa, HE

Vecchio, KS

AF Khalifa, Hesham E.

Vecchio, Kenneth S.

TI Devitrification and Cooling Rate Effects on Microstructure and

Mechanical Properties in Fe57C9B11Mo12Cr8W3 Bulk Metallic Glass

SO ADVANCED ENGINEERING MATERIALS

RI Vecchio, Kenneth/F-6300-2011

OI Vecchio, Kenneth/0000-0003-0217-6803; Khalifa,

Hesham/0000-0001-5531-0493

SN 1438-1656

PD NOV

PY 2008

VL 10

IS 11

BP 1056

EP 1063

DI 10.1002/adem.200800100

UT WOS:000261933400014

ER

PT J

AU Shibata, A

Imamura, Y

Sone, M

Ishiyama, C

Higo, Y

AF Shibata, A.

Imamura, Y.

Sone, M.

Ishiyama, C.

Higo, Y.

TI Pd-Ni-P metallic glass film fabricated by electroless alloy plating

SO THIN SOLID FILMS

AB In the present study a Pd-Ni-P film has been fabricated by electroless alloy plating. The fabricated Pd-Ni-P film was found to be a metallic glass on the basis of two features, namely, an amorphous structure and a glass transition followed by crystallization during heating. The thermal stability of the supercooled liquid region, however, was lower than that of bulk Pd-Ni-P metallic glass. And unlike the conventional metallic glasses, the fabricated Pd-Ni-P film did not have a uniform microstructure. The non-uniform microstructure of this film resulted from the inhomogeneous distribution of the free Volume accompanying the electroless alloy plating reaction. (C) 2008 Elsevier B.V. All rights reserved.

RI Shibata, Akinobu/C-7884-2012; Ishiyama, Chiemi/F-3337-2015; Sone,

Masato/A-7203-2014

OI Ishiyama, Chiemi/0000-0003-2489-6318; Sone, Masato/0000-0002-7334-1952

SN 0040-6090

PD JAN 30

PY 2009

VL 517

IS 6

BP 1935

EP 1938

DI 10.1016/j.tsf.2008.09.101

UT WOS:000263019900014

ER

PT J

AU Fang, SS

Xiao, XS

Wang, Q

Xia, L

Dong, Y

AF Fang, SS

Xiao, XS

Wang, Q

Xia, L

Dong, Y

TI Relationship between supercooled liquid region and bond parameters of

Pd-based bulk metallic glasses

SO RARE METAL MATERIALS AND ENGINEERING

AB The regression analysis of the expression of supercooled liquid region DeltaT(x) for Pd-based bulk metallic glass was conducted by computer. It was obtained that the supercooled liquid region of Pd-based bulk metallic glass has close relation with their bond parameters, which can be expressed by the follow equation with a relative coefficient 97.2%: DeltaT(x) = 29.369 09+3 602.458 98(Deltax)(2)+9 992.767 58 delta(2) 213 -3.958 97n(2/3), It can be seen from the equation that the values of DeltaT(x) increase with increasing the electronegativity and atomic size parameter difference of the alloys while they decrease very little with increasing electron density.

OI Xia, Lei/0000-0001-9198-1497

SN 1002-185X

PD NOV

PY 2004

VL 33

IS 11

BP 1132

EP 1135

UT WOS:000225582400003

ER

PT J

AU Hays, CC

Kim, CP

Johnson, WL

AF Hays, CC

Kim, CP

Johnson, WL

TI Microstructure controlled shear band pattern formation and enhanced

plasticity of bulk metallic glasses containing in situ formed ductile

phase dendrite dispersions

SO PHYSICAL REVIEW LETTERS

AB Results are presented for a ductile metal reinforced bulk metallic glass matrix composite based on glass forming compositions in the Zr-Ti-Cu-Ni-Be system. Primary dendrite growth and solute partitioning in the molten state yields a microstructure consisting of a ductile crystalline Ti-Zr-Nb beta phase, with bce structure, in a Zr-Ti-Nb-Cu-Ni-Be bulk metallic glass matrix. Under unconstrained mechanical loading organized sheer band patterns develop throughout the sample. This results in a dramatic increase in the plastic strain to failure, impact resistance, and toughness of the metallic glass.

RI Hays, Charles/P-8021-2015

OI Hays, Charles/0000-0002-0420-1761

SN 0031-9007

PD MAR 27

PY 2000

VL 84

IS 13

BP 2901

EP 2904

DI 10.1103/PhysRevLett.84.2901

UT WOS:000086062000034

ER

PT J

AU Lee, MH

Bae, DH

Kim, DH

Kim, WT

Sordelet, DJ

Kim, KB

Eckert, J

AF Lee, Min Ha

Bae, Dong Hyun

Kim, Do Hyang

Kim, Won Tae

Sordelet, Daniel J.

Kim, Ki Buem

Eckert, Juergen

TI Nanocrystallization at shear bands in bulk metallic glass matrix

composites

SO SCRIPTA MATERIALIA

AB We investigated the effect of reinforcement on the formation of nanocrystals at shear bands in a Ni-based metallic glass matrix composite in comparison with monolithic Ni-based bulk metallic glass when shear bands are generated during deformation. The results suggest that the occurrence of nanocrystallization at a shear band implies a stress concentration by a geometrical effect of the reinforcement phase on the compressive loading conditions. (C) 2007 Acta Materialia Inc. Published by Elsevier Ltd. All rights reserved.

RI Juhyun, Oh/H-9185-2012; bang, changwook/J-7922-2012

OI LEE, MIN HA/0000-0001-6006-0628

SN 1359-6462

PD APR

PY 2008

VL 58

IS 8

BP 651

EP 654

DI 10.1016/j.scriptamat.2007.11.032

UT WOS:000254197600009

ER

PT J

AU Sun, GY

Chen, G

Liu, CT

Chen, GL

AF Sun, G. Y.

Chen, G.

Liu, C. T.

Chen, G. L.

TI Innovative processing and property improvement of metallic glass based

composites

SO SCRIPTA MATERIALIA

AB An innovative method was developed based on controlled solidification in the liquid-solid two-phase region to produce spherical crystalline particles dispersed in the amorphous matrix. This new processing method can greatly improve both ductility and strength of bulk metallic glass composites. (c) 2006 Acta Materialia Inc. Published by Elsevier Ltd. All rights reserved.

OI Liu, Chain Tsuan/0000-0001-7888-9725

SN 1359-6462

PD AUG

PY 2006

VL 55

IS 4

BP 375

EP 378

DI 10.1016/j.scriptamat.2006.04.017

UT WOS:000238815800024

ER

PT J

AU Zhuang, YX

Wang, WH

Zhang, Y

Pan, MX

Zhao, DQ

AF Zhuang, YX

Wang, WH

Zhang, Y

Pan, MX

Zhao, DQ

TI Crystallization kinetics and glass transition of

Zr41Ti14Cu12.5Ni10-xFexBe22.5 bulk metallic glasses

SO APPLIED PHYSICS LETTERS

AB The crystallization kinetics and glass transition behavior of Zr41Ti14Cu12.5Ni10-xFexBe22.5 (x = 0, 2, and 5) bulk metallic glasses (BMGs) have been investigated by means of differential scanning calorimeter under nonisothermal conditions. The glass transition temperature T-g, the onset crystallization temperature T-x, and the crystallization peak temperature T-pi of the BMGs are found to depend on the heating rate during the continuous heating. The effective activation energy and the frequency factor of the glass transition and crystallization of the BMGs are determined by the Kissinger method. The glass forming ability of the glass forming alloys has been discussed in view of the crystallization kinetics. (C) 1999 American Institute of Physics. [S0003-6951(99)04242-4].

RI ZHANG, Yong/B-7928-2009; Zhuang, Yanxin/F-7199-2011

OI ZHANG, Yong/0000-0002-6355-9923;

SN 0003-6951

PD OCT 18

PY 1999

VL 75

IS 16

BP 2392

EP 2394

DI 10.1063/1.125024

UT WOS:000083111100016

ER

PT J

AU Wei, S

Stolpe, M

Gross, O

Evenson, Z

Gallino, I

Hembree, W

Bednarcik, J

Kruzic, JJ

Busch, R

AF Wei, Shuai

Stolpe, Moritz

Gross, Oliver

Evenson, Zach

Gallino, Isabella

Hembree, William

Bednarcik, Jozef

Kruzic, Jamie J.

Busch, Ralf

TI Linking structure to fragility in bulk metallic glass-forming liquids

SO APPLIED PHYSICS LETTERS

AB Using in-situ synchrotron X-ray scattering, we show that the structural evolution of various bulk metallic glass-forming liquids can be quantitatively connected to their viscosity behavior in the supercooled liquid near T-g. The structural signature of fragility is identified as the temperature dependence of local dilatation on distinct key atomic length scales. A more fragile behavior results from a more pronounced thermally induced dilatation of the structure on a length scale of about 3 to 4 atomic diameters, coupled with shallower temperature dependence of structural changes in the nearest neighbor environment. These findings shed light on the structural origin of viscous slowdown during undercooling of bulk metallic glass-forming liquids and demonstrate the promise of predicting the properties of bulk metallic glasses from the atomic scale structure. (C) 2015 AIP Publishing LLC.

RI Kruzic, Jamie/M-3558-2014

OI Kruzic, Jamie/0000-0002-9695-1921

SN 0003-6951

EI 1077-3118

PD MAY 4

PY 2015

VL 106

IS 18

AR 181901

DI 10.1063/1.4919590

UT WOS:000354259200013

ER

PT J

AU Liu, Z

Chan, KC

Liu, L

Guo, SF

AF Liu, Z.

Chan, K. C.

Liu, L.

Guo, S. F.

TI Bioactive calcium titanate coatings on a Zr-based bulk metallic glass by

laser cladding

SO MATERIALS LETTERS

AB The bioactive calcium titanate coating was prepared by laser cladding on a (Zr0.62Cu0.23Fe0.05Al0.1)(97)Ag-3 bulk metallic glass. The coating, which was mainly composed with dendrite calcium titanate and a little calcium pyrophosphate, formed a strong bonding with the substrate. The cladding process generated a crystallized heat-affected zone with a thickness of 300 mu m and other parts in the substrate remained amorphous structure after laser cladding. It was demonstrated that surfaces of Zr-based bulk metallic glasses can be made bioactive and that laser cladding techniques were useful methods in the purpose of making Zr-based bulk metallic glass surface bioactive. Crown Copyright (C) 2012 Published by Elsevier B.V. All rights reserved.

RI Guo, Shengfeng/E-3171-2012; Chan, K.C./A-2311-2014

OI Guo, Shengfeng/0000-0002-6667-6797; Chan, K.C./0000-0002-6173-5532

SN 0167-577X

PD SEP 1

PY 2012

VL 82

BP 67

EP 70

DI 10.1016/j.matlet.2012.05.022

UT WOS:000306723900022

ER

PT J

AU Li, HX

Lu, ZP

Yi, S

AF Li, Hongxiang

Lu, Zhaoping

Yi, Seonghoon

TI Estimation of the Glass Forming Ability of the Fe-based Bulk Metallic

Glass Fe68.8C7.0Si3.5B5.0P9.6Cr2.1Mo2.0Al2.0 that Contains Non-metallic

Inclusions

SO METALS AND MATERIALS INTERNATIONAL

AB For the mass production of bulk metallic glasses, the use of industrial raw materials that contain certain amounts of inclusions is inevitable. The glass-forming ability of bulk metallic glasses, i.e., the critical cooling rate for glass formation upon solidification, is closely related to the nature of heterogeneous nucleation offered by inclusions during the solidification process. Significantly different effects of various types of inclusions on the glass forming ability of the alloy Fe68.8C7.0Si3.5B5.0P9.6Cr2.1Mo2.0Al2.0 are demonstrated in this study. The origins of the effects of different inclusions oil the glass forming ability are analyzed through thermodynamic, crystallographic and classical heterogeneous nucleation kinetic theories.

RI Lu, Zhao-Ping/A-2718-2009

OI Lu, Zhao-Ping/0000-0003-1463-8948

SN 1598-9623

PD FEB

PY 2009

VL 15

IS 1

BP 7

EP 14

DI 10.1007/s12540-009-0007-x

UT WOS:000263833300002

ER

PT J

AU Hou, L

Yang, WM

Liu, HS

Huo, JT

Jiao, Y

Liu, QL

AF Hou, Long

Yang, Weiming

Liu, Haishun

Huo, Juntao

Jiao, Yang

Liu, Qingling

TI Effects of Annealing on the Specific Heat and Boson Peak for FeCoBSiNb

Bulk Metallic Glass

SO JOURNAL OF LOW TEMPERATURE PHYSICS

AB Low temperature specific heats of FeCoBSiNb bulk metallic glass annealed at different temperatures are studied from 1 to 110 K, and the effects of annealing on the specific heat and boson peak are analyzed. It is found that the specific heat deviates from the Debye model, and the boson peak moves to higher temperature and the intensity decreases with isothermally annealing. By analyzing the experimental data, the origin of the boson peak is interpreted using the localized harmonic modes based on the vibrations of loose "rattler" atoms in the oversized cage structure. The results have significant implications for understanding the structure-property relationship of metallic glasses at low temperature.

SN 0022-2291

EI 1573-7357

PD JUN

PY 2015

VL 179

IS 5-6

BP 343

EP 349

DI 10.1007/s10909-015-1289-7

UT WOS:000353824600006

ER

PT J

AU Wu, J

Pan, Y

Zhang, L

Pi, J

AF Wu, J.

Pan, Y.

Zhang, L.

Pi, J.

TI Fabrication of Cu rich bulk metallic glass composites via solidification

method

SO MATERIALS SCIENCE AND TECHNOLOGY

AB Cu based bulk metallic glasses and composites with tiny crystalline phases embedded in metallic glass matrix have been successfully fabricated by solidification technique in the present work. The formation of crystalline phases and structure inhomogeneity in bulk metallic glasses was characterised. Al is used as the minor alloying element to partly substitute Cu element in 61Cu-34Zr-5Ti. The results show that quarternary 60Cu-34Zr-5Ti-1Al alloy exhibits monoamorphous feature, and 56Cu-34Zr-5Ti-5Al alloy has a few crystalline peaks superimposed on a broad diffraction peak, suggesting that a composite structure forms in certain solidification conditions. To further identify the microstructure of the as cast rod, all samples were characterised by scanning electron microscopy (SEM). Small size phases are found in 2 mm diameter 56Cu-34Zr-5Ti-5Al rod, which has larger plastic deformation. The composition of those crystalline phases is also investigated. All results indicate that the presence of certain phases in metallic matrix benefits the mechanical properties of the as cast bulk metallic glasses.

SN 0267-0836

EI 1743-2847

PD FEB

PY 2014

VL 30

IS 2

BP 166

EP 170

DI 10.1179/1743284713Y.0000000329

UT WOS:000336734700005

ER

PT J

AU Sun, WS

Liang, XB

Kulik, T

AF Sun, WS

Liang, XB

Kulik, T

TI Formation and magnetic properties of Co-Fe-based bulk metallic glasses

with supercooled liquid region

SO JOURNAL OF MAGNETISM AND MAGNETIC MATERIALS

AB A new Co-Fe-based ferromagnetic bulk metallic glass (BMG) was synthesized by copper mould casting method. The thermal stability and crystallization processes were investigated by differential scanning calorimetry (DSC) and X-ray diffraction (XRD). The soft magnetic behavior was studied by DC magnetic measurements. The high glass formation ability was interpreted in terms of the effective suppression of nucleation and growth of the intermetallic compounds which appear in the multicomponent system during solidification. The high thermal stability indicates that the new Co-Fe-based BMG could be used as high-temperatUre magnetic material. The low coercivity which was as low as 8 A/m for the as-cast sample was found in the Co-Fe-based metallic glass cylinder with a diameter of 1.5 mm. (c) 2005 Elsevier B.V. All rights reserved.

SN 0304-8853

PD APR

PY 2006

VL 299

IS 2

BP 492

EP 495

DI 10.1016/j.jmmm.2005.04.032

UT WOS:000234739900033

ER

PT J

AU Demetriou, MD

Kaltenboeck, G

Suh, JY

Garrett, G

Floyd, M

Crewdson, C

Hofmann, DC

Kozachkov, H

Wiest, A

Schramm, JP

Johnson, WL

AF Demetriou, Marios D.

Kaltenboeck, Georg

Suh, Jin-Yoo

Garrett, Glenn

Floyd, Michael

Crewdson, Chase

Hofmann, Douglas C.

Kozachkov, Henry

Wiest, Aaron

Schramm, Joseph P.

Johnson, William L.

TI Glassy steel optimized for glass-forming ability and toughness

SO APPLIED PHYSICS LETTERS

AB An alloy development strategy coupled with toughness assessments and ultrasonic measurements is implemented to design a series of iron-based glass-forming alloys that demonstrate improved glass-forming ability and toughness. The combination of good glass-forming ability and high toughness demonstrated by the present alloys is uncommon in Fe-based systems, and is attributed to the ability of these compositions to form stable glass configurations associated with low activation barriers for shear flow, which tend to promote plastic flow and give rise to a toughness higher than other known Fe-based bulk-glass-forming systems.

OI Suh, Jin-Yoo/0000-0003-3786-6652

SN 0003-6951

EI 1077-3118

PD JUL 27

PY 2009

VL 95

IS 4

AR 041907

DI 10.1063/1.3184792

UT WOS:000268611900022

ER

PT J

AU Wu, FF

Chan, KC

Jiang, SS

Chen, SH

Wang, G

AF Wu, Fu-Fa

Chan, K. C.

Jiang, Song-Shan

Chen, Shun-Hua

Wang, Gang

TI Bulk metallic glass composite with good tensile ductility, high strength

and large elastic strain limit

SO SCIENTIFIC REPORTS
AB Bulk metallic glasses exhibit high strength and large elastic strain limit but have no tensile ductility. However, bulk metallic glass composites reinforced by in-situ dendrites possess significantly improved toughness but at the expense of high strength and large elastic strain limit. Here, we report a bulk metallic glass composite with strong strain-hardening capability and large elastic strain limit. It was found that, by plastic predeformation, the bulk metallic glass composite can exhibit both a large elastic strain limit and high strength under tension. These unique elastic mechanical properties are attributed to the reversible B2 <-> B19' phase transformation and the plastic-predeformation-induced complicated stress state in the metallic glass matrix and the second phase. These findings are significant for the design and application of bulk metallic glass composites with excellent mechanical properties.
RI Chen, S.H./H-3694-2013
OI Chen, S.H./0000-0002-7060-6249; Chan, K.C./0000-0002-6173-5532
SN 2045-2322
PD JUN 16
PY 2014
VL 4
AR 5302
DI 10.1038/srep05302
UT WOS:000337341200001
PM 24931632
ER

PT J
AU Cheney, J
   Vecchio, K
AF Cheney, Justin
   Vecchio, Kenneth
TI Development of quaternary Fe-based bulk metallic glasses
SO MATERIALS SCIENCE AND ENGINEERING A-STRUCTURAL MATERIALS PROPERTIES
   MICROSTRUCTURE AND PROCESSING
AB Fe-based bulk metallic glasses have been designed using several modeling criteria. Quaternary alloys were successfully developed in the Fe-rich compositional region where the only solutes used were Cr, Nb, and B. Furthermore, the exceedingly high level of Fe, exceeding 75 wt.% in some alloys, makes this series of alloys, compositionally very close to conventional steel alloys. Therefore, their production using conventional facilities and materials is more easily attainable than previously developed bulk metallic glasses. The modeling criteria used, simultaneously analyzes the thermodynamics and kinetics of the vitrification behavior in a potential glass-forming alloy. A liquidus model, which determines and ranks the presence of deep eutectics, is used to determine the optimal compositional region. This criterion is cross-checked with an elastic strain model. Alloys compositionally located near a deep eutectic, while simultaneously containing a topology that induces significant elastic strain in a developing crystalline lattice exhibits experimentally good glass forming ability. (C) 2008 Elsevier B.V. All rights reserved.
RI Vecchio, Kenneth/F-6300-2011
OI Vecchio, Kenneth/0000-0003-0217-6803
SN 0921-5093
PD SEP 25
PY 2008
VL 492
IS 1-2
BP 230
EP 235
DI 10.1016/j.msea.2008.03.019
UT WOS:000258644500032
ER

PT J
AU Luo, L
   Tian, R
   Xiao, XS
AF Luo Lin
   Tian Rui
   Xiao Xueshan
TI Development of bulk metallic glasses based on the Dy-Al binary eutectic
   composition
SO JOURNAL OF RARE EARTHS
AB A series of dysprosium-based ternary, quadruple, and quintuple bulk metallic glasses (BMGs) based on Dy-Al binary eutectic composition were obtained with the partial substitution of Co, Gd, and Ni elements, for dysprosium. The results showed that the Dy(31)Gd(25)Co(20)Al(24) alloy, which had the best glass forming ability (GFA), could be cast into an amorphous rod with a diameter of 5 mm. The GFA of alloys was evaluated on the basis of the supercooled liquid region width, gamma parameter, the formation enthalpy, and the equivalent electronegativity, difference of amorphous alloys. It was found that the eutectic composition was closely correlated with the GFA of the Dy-based BMGs.
SN 1002-0721
PD DEC
PY 2008
VL 26
IS 6
BP 813
EP 816
DI 10.1016/S1002-0721(09)60012-0
UT WOS:000262819600012
ER

PT J
AU Li, Y
AF Li, Y
TI Bulk metallic glasses: Eutectic coupled zone and amorphous formation
SO JOM
AB Recent developments have led to renewed interest in bulk metallic glasses, which have an exceptionally low critical cooling rate for amorphous formation. They can be studied under controlled cooling conditions in which phase selection among all the crystalline and noncrystalline phases can be determined. The authors studied glass formation and glass-forming ability in relation to the eutectic coupled zone and found that for the alloys with a symmetric coupled zone, the best glass-forming region is associated with eutectic composition. For alloys with an asymmetric coupled zone, the best glass-forming region is at an off-eutectic composition. Understanding these materials could lead to the discovery of alloys of high glass-forming ability.
SN 1047-4838
EI 1543-1851
PD MAR
PY 2005
VL 57
IS 3
BP 60
EP 63
DI 10.1007/s11837-005-0236-5
UT WOS:000227635600013
ER

PT J
AU Philo, SL
   Heinrich, J
   Gallino, I
   Busch, R
   Kruzic, JJ
AF Philo, S. L.
   Heinrich, J.
   Gallino, I.
   Busch, R.
   Kruzic, J. J.
TI Fatigue crack growth behavior of a Zr58.5Cu15.6Ni12.8Al10.3Nb2.8 bulk
   metallic glass-forming alloy
SO SCRIPTA MATERIALIA
AB Fatigue crack growth behavior was studied for a Zr58.5Cu15.6Ni12.8Al10.3Nb2.8 bulk metallic glass in ambient air, demonstrating a fatigue threshold of Delta K-TH =: 1.4 MPa root m and a Paris law exponent of 1.7. A nearly stress intensity-independent crack growth regime occurred at 2.5 x 10(-8) m cycle(-1), suggesting an environmental influence of ambient air on the fatigue crack growth, as has been observed for Zr-Ti-Ni-Cu-Be bulk metallic glasses. However, this environmental fatigue effect was shifted to 25x higher growth rates due to the different chemistry. (C) 2010 Acta Materialia Inc. Published by Elsevier Ltd. All rights reserved.
RI Kruzic, Jamie/M-3558-2014
OI Kruzic, Jamie/0000-0002-9695-1921
SN 1359-6462
PD FEB
PY 2011
VL 64
IS 4
BP 359
EP 362
DI 10.1016/j.scriptamat.2010.10.042
UT WOS:000285951600014
ER

PT J
AU Zhang, YC
   Shi, TL
   Tang, ZR
   Liao, GL
AF Zhang, Yichun
   Shi, Tielin
   Tang, Zirong
   Liao, Guanglan
TI An improved method to estimate Young's modulus of Zr-based BMGs
   accounting for pile-up during nanoindentation
SO JOURNAL OF NON-CRYSTALLINE SOLIDS
AB Considerable attention has been focused on metallic glasses for their excellent mechanical properties where nanoindentation has been widely used to estimate the material parameters, while traditional analysis of nanoindentation data can overestimate the Young's modulus and hardness due to pile-up which occurs commonly in metallic glasses. In this paper, we demonstrate an improved method to estimate the Young's modulus and hardness of Zr-based bulk metallic glasses accounting for pile-up during nanoindentation. The contact boundary of the pile-up on Zr55Cu30Al10Ni5 is investigated, a model to estimate the contact area is developed, and then we achieve the Young's modulus together with hardness directly from load-depth data. Two other Zr-based bulk metallic glasses are used for verification, and four methods including two direct methods and two scanning probe microscopy-based methods are also performed for comparison. The results of the improved method agree with the referenced Young's modulus better than those methods, proving the feasibilities and potential of the method in engineering. (C) 2015 Elsevier B.V. All rights reserved.
SN 0022-3093
EI 1873-4812
PD NOV 1
PY 2015
VL 427
BP 20
EP 25
DI 10.1016/j.jnoncrysol.2015.07.031
UT WOS:000362135500004
ER

PT J
AU Bobzin, K
   Ote, M
   Linke, TF
   Konigstein, T
AF Bobzin, Kirsten
   Oete, Mehmet
   Linke, Thomas Frederik
   Koenigstein, Tim
TI Process Development for Innovative Iron Alloy Metallic Glass Coatings
SO ADVANCED ENGINEERING MATERIALS
AB Bulk metallic glasses exhibit high corrosive resistance, good tribological behavior, and low thermal conductivity. The production of metallic glass using Thermal Spray technology is a novel approach. In this study, the manufacturing of iron-based metallic glass coatings by high velocity oxygen fuel spraying is investigated. For this purpose individually designed, amorphous feedstock materials are used. By means of X-Ray-Diffraction and Differential Scanning Calorimetry amorphous structures are identified. Subsequently, the thermal diffusivities are determined by means of Laser-Flash method. The low thermal diffusivity measured proves the potential of metallic glasses as thermal barrier coatings for low temperature applications, such as combustion engines.
SN 1438-1656
EI 1527-2648
PD OCT
PY 2016
VL 18
IS 10
BP 1833
EP 1840
DI 10.1002/adem.201600177
UT WOS:000385667900018
ER

PT J
AU Deng, L
   Zhou, BW
   Yang, HS
   Jiang, X
   Jiang, BY
   Zhang, XG
AF Deng, Liang
   Zhou, Bingwen
   Yang, Hongshuo
   Jiang, Xin
   Jiang, Boyu
   Zhang, Xingguo
TI Roles of minor rare-earth elements addition in formation and properties
   of Cu-Zr-Al bulk metallic glasses
SO JOURNAL OF ALLOYS AND COMPOUNDS
AB The effect of RE addition on glass-forming ability (GFA) and mechanical properties of (Cu47Zr45Al8)(100-x)REx (RE = Ce, Nd, Gd, Y, Dy, Lu, x = 0-4 at.%) bulk metallic glasses (BMGs) has been studied in this paper. It has been found that trace amount of rare-earth addition in the Cu-rich Cu-Zr-Al alloys can decrease the temperature of glass transition (T-g) and increase gamma(gamma = T-x/(T-g + T-l)) value, and therefore improve the GFA. The (Cu47Zr45Al8)(96)Y-4 bulk metallic glass with critical diameter of 25 mm was prepared by copper-mold-casting. The (Cu47Zr45Al8)(97)Lu-3 bulk metallic glass exhibited good plasticity 4.65% at room temperature during compression test, which was applied high yield strength of 1840 MPa. With the increase of rare-earth elements addition up to 4 at.%, the fracture strength of the alloy reduced obviously and no more plastic deformation can be observed on the fracture surface. Young's modulus, fracture strength and Poisson's ratio were investigated for the mechanism of deformation (from plasticity to brittleness) in the compression tests. (C) 2015 Elsevier B.V. All rights reserved.
SN 0925-8388
EI 1873-4669
PD MAY 25
PY 2015
VL 632
BP 429
EP 434
DI 10.1016/j.jallcom.2015.01.036
UT WOS:000350388900063
ER

PT J
AU Hasegawa, M
   Soda, K
   Sato, H
   Suzuki, T
   Taketomi, T
   Takeuchi, T
   Kato, H
   Mizutani, U
AF Hasegawa, M.
   Soda, K.
   Sato, H.
   Suzuki, T.
   Taketomi, T.
   Takeuchi, T.
   Kato, H.
   Mizutani, U.
TI Stability and electronic structure of Zr-based ternary metallic glasses
   and relevant compounds
SO JOURNAL OF ALLOYS AND COMPOUNDS
CT 12th International Symposium on Metastable and Nano-Materials
   (ISMANAM-2005)
CY JUL 03-07, 2005
CL Paris, FRANCE
AB The electronic structure of the Zr-based metallic glasses has been investigated by theoretical and experimental approaches. One approach is band calculations of the Zr2Ni (Zr66.7Ni33.3) compound to investigate the electronic structure of the Zr66.7Ni33.3 metallic glass (Delta T-x = 0 K) of which the local atomic structure is similar to that of the Zr2Ni compound. The other is photoemission spectroscopy of the Zr50Cu35Al15 bulk metallic glass (BMG) (Delta T-x = 69 K). Here Delta T-x = T-x - T-x where T-x and T-g are crystallization and glass transition temperature, respectively. Both results and previous ones on the Zr55Cu30Ni5Al10 BMG indicate that there is a pseudogap at the Fermi level in the electronic structure of these Zr-based metallic glasses, independent of the value of the Delta T-x. This implies that the pseudogap at the Fermi level is one of the factors that stabilize the glass phase of Zr-based metallic glasses. (C) 2006 Elsevier B.V. All rights reserved.
RI Kato, Hidemi/B-2492-2015
SN 0925-8388
PD MAY 31
PY 2007
VL 434
SI SI
BP 149
EP 151
DI 10.1016/j.jallcom.2006.08.277
UT WOS:000246286900038
ER

PT J
AU Inoue, A
   Wang, XM
   Zhang, W
AF Inoue, A.
   Wang, X. M.
   Zhang, W.
TI Developments and applications of bulk metallic glasses
SO REVIEWS ON ADVANCED MATERIALS SCIENCE
CT 13th International Symposium on Metastable and Nano-Materials
   (ISMANAM-2006)
CY AUG 27-31, 2006
CL Warsaw Univ Technol, Fac Mat Sci & Engn, Warsaw, POLAND
HO Warsaw Univ Technol, Fac Mat Sci & Engn
AB This paper aims to review our recent data on the development researches and application examples of bulk glassy alloys. Very recently, the glass-forming ability of metallic alloys is significantly enhanced by further multiplication of alloy components based on the three component rules for bulk glass formation. The maximum diameter for glass formation exceeds 10 mm in a variety of alloy systems such as Zr, Pd, Pt, Mg, La, Fe, Co, Ni and Cu bases. By use of high glass-forming ability, good castability, good printability and unique characteristics, application examples of bulk glassy alloys have been extended to much valuable fields in which conventional crystalline alloys cannot be used. These novel advantages for bulk glassy alloys allow us to conclude that bulk glassy alloys are used in much wider application fields in the near future.
RI Inoue, Akihisa/E-5271-2015
SN 1606-5131
PD MAY
PY 2008
VL 18
IS 1
BP 1
EP 9
UT WOS:000256370800003
ER

PT J
AU Majumdar, B
   Akhtar, D
AF Majumdar, B
   Akhtar, D
TI Synthesis and crystallisation of Fe-Co-Ni-Zr(Nb)-B bulk glasses
SO MATERIALS SCIENCE AND TECHNOLOGY
AB Bulk glass formation in multicomponent Fe-Co-Ni-Zr-B and Fe-Co-Ni-Zr-Nb-B alloy systems has been investigated by means of casting in a wedge shaped copper mould. It is observed that the critical thickness for glass formation, thermal stability of the glass and the undercooled liquid region increase upon the addition of niobium. The coercivity increases abruptly above the critical thickness owing to disappearance of the amorphous phase. Amorphous ribbons of both alloy systems have been prepared with various zirconium and boron contents to obtain a spectrum of values for glass transition, crystallisation temperature and undercooled liquid region. Kinetics studies of crystallisation have revealed that the activation energy increases with increasing undercooled liquid region.
SN 0267-0836
EI 1743-2847
PD OCT
PY 2005
VL 21
IS 10
BP 1139
EP 1144
DI 10.1179/174328405X62251
UT WOS:000232884800004
ER

PT J
AU Wang, XY
   Gong, P
   Deng, L
   Jin, JS
   Wang, SB
   Zhou, P
AF Wang, Xinyun
   Gong, Pan
   Deng, Lei
   Jin, Junsong
   Wang, Sibo
   Zhou, Peng
TI Nanoindentation study on the room temperature creep characteristics of a
   senary Ti16.7Zr16.7Hf16.7Cu16.7Ni16.7Be16.7 high entropy bulk metallic
   glass
SO JOURNAL OF NON-CRYSTALLINE SOLIDS
AB The room temperature creep behavior of a newly developed Ti16.7Zr16.7Hf16.7Cu16.7Ni16.7Be16.7 high entropy bulk metallic glass was investigated by nanoindentation measurements with various loading rates in comparison with that of a typical conventional Vitreloy 1 bulk metallic glass. A generalized Kelvin model was adopted to describe the creep curves. It was found that for both the two studied alloys, the creep displacement and strain rate sensitivity increases with increasing loading rate, indicating a positive loading rate effect. Vitreloy 1 BMG exhibits a more obvious loading rate effect compared with Ti16.7Zr16.7Hf16.7Cu16.7Ni16.7Be16.7 HE-BMG. The creep compliance and retardation spectra were also derived to study the creep mechanism. Compared with other developed high entropy alloys and bulk metallic glasses (e.g. Vitreloy 1 alloy), the studied HE-BMG possesses relatively small strain rate sensitivity values, indicating a good creep resistance. Based on the experimental results, the mechanisms of the unique creep resistance of Ti16.7Zr16.7Hf16.7Cu16.7Ni16.7Be16.7 high entropy bulk metallic glass have also been explained.
RI Gong, Pan/K-8870-2012
OI Gong, Pan/0000-0002-3833-8440
SN 0022-3093
EI 1873-4812
PD AUG 15
PY 2017
VL 470
BP 27
EP 37
DI 10.1016/j.jnoncrysol.2017.04.041
UT WOS:000403732600005
ER

PT J
AU Singh, PK
   Dubey, KS
AF Singh, P. K.
   Dubey, K. S.
TI Thermodynamic behaviour of bulk metallic glasses
SO THERMOCHIMICA ACTA
AB The thermodynamic behaviour of bulk metallic glass forming melts have been studied by analyzing the temperature dependence of the Gibbs free energy difference Delta G, entropy difference Delta S and enthalpy difference Delta H between the undercooled melts and corresponding equilibrium solid phases. The study is made by calculating Delta G, Delta S and Delta H in the entire temperature range T-m to T-g for five samples Mg65Cu25Y10. Cu47Ti34Zr11Ni8, Zr46Cu46Al8,Zr-46(Cu4.5/5.5Ag1/1.5)(46)Al-8, Zr57Cu15.4Ni12.6Al10Nb5 and it is found that the calculated values of these thermodynamic parameters are very close to the experimental values, where T-m and T-g are melting temperature and glass transition temperature, respectively. (C) 2011 Elsevier B.V. All rights reserved.
SN 0040-6031
PD FEB 20
PY 2012
VL 530
BP 120
EP 127
DI 10.1016/j.tca.2011.12.012
UT WOS:000300540200016
ER

PT J
AU Xiao, XS
   Shoushi, F
   Wang, GM
   Qin, H
   Dong, Y
AF Xiao, XS
   Shoushi, F
   Wang, GM
   Qin, H
   Dong, Y
TI Influence of beryllium on thermal stability and glass-forming ability of
   Zr-Al-Ni-Cu bulk amorphous alloys
SO JOURNAL OF ALLOYS AND COMPOUNDS
AB Thermal stability and glass-forming ability of Zr65-xAl10Ni10Cu15Bex (x = 0, 3, 6, 9, 12.5, 16) bulk metallic glasses were investigated by differential scanning calorimetry, differential thermal analysis, X-ray diffraction and high-resolution transmission electron microscopy (HRTEM). The results show that the substitution of beryllium for zirconium strongly affects the supercooled liquid region (DeltaT(x) = T-x - T-g(onset)), the reduced supercooled liquid region (DeltaT(rg) = (T-g(onset))/(T-i - T-g(onset))) and the reduced glass transition temperature (T-rg = T-g/T-i). With increasing of x values, the three parameters that were used to indicate the glass forming ability and thermal stability increase first and then decrease. It is in agreement with the result that these glasses gradually change from an off-eutectic composition into a eutectic one (x = 12.5) that favours higher glass-forming ability, and then there is again a departure from the eutectic composition. Consequently, a moderate Be content will favor to increase the glass forming ability and thermal stability in Zr65-xAl10Ni10Cu15Bex bulk amorphous alloys. The effect of Be was explained in terms of Miedema's theory, electronegativity difference and atomic size parameter of the amorphous alloys. (C) 2004 Elsevier B.V. All rights reserved.
SN 0925-8388
PD AUG 11
PY 2004
VL 376
IS 1-2
BP 145
EP 148
DI 10.1016/j.jallcom.2004.01.014
UT WOS:000223025400027
ER

PT J
AU Zhang, GQ
   Jiang, QK
   Chen, LY
   Shao, M
   Liu, JF
   Jiang, JZ
AF Zhang, G. Q.
   Jiang, Q. K.
   Chen, L. Y.
   Shao, M.
   Liu, J. F.
   Jiang, J. Z.
TI Synthesis of centimeter-size Ag-doped Zr-Cu-Al metallic glasses with
   large plasticity
SO JOURNAL OF ALLOYS AND COMPOUNDS
AB Glass forming ability of Ni-free Zr48Cu45-xAl7Agx (x = 0, 1, 1.5, 2, 2.5, 3, 5 and 8 at.%) alloys has been studied. It is found that centimeter-diameter bulk metallic glasses (BMGs) can be synthesized for quaternary Zr48Cu45-xAl7Agx, (x = 2, 3, 5 and 8 at.%) alloys using copper mold casting. The maximum diameter, 12 mm, is found for the Zr48Cu43Al7Ag2 alloy while the ternary Zr48Cu45Al7 can only be cast into fully BMG with a diameter of 5 mm. The Zr48Cu43Al7Ag2 BMG alloy also exhibits a plasticity of about 3% during compression at ambient temperature together with a yielding strain of 1.9% and a yielding strength of 1900MPa. The developed Zr48Cu43Al7Ag2 BMG alloy might have biomedical applications. (c) 2006 Elsevier B.V. All rights reserved.
RI Chen, Lianyi/B-3156-2008; BAI, JIE/D-7448-2016
OI Chen, Lianyi/0000-0003-3720-398X;
SN 0925-8388
EI 1873-4669
PD NOV 9
PY 2006
VL 424
IS 1-2
BP 176
EP 178
DI 10.1016/j.jallcom.2006.06.088
UT WOS:000241414900032
ER

PT J
AU Lu, Z
   Yang, XN
   Sun, BA
   Li, YZ
   Chen, K
   Wang, WH
   Bai, HY
AF Lu, Z.
   Yang, X. N.
   Sun, B. A.
   Li, Y. Z.
   Chen, K.
   Wang, W. H.
   Bai, H. Y.
TI Divergent strain acceleration effects in metallic glasses
SO SCRIPTA MATERIALIA
AB Relaxation is a key feature in glassy physics, and the strain-induced effects on relaxations in metallic glasses are of practical significance, yet still unclear. Through a contrastive combination of dynamical mechanical spectroscopy and linear-heating stress relaxation methods, we find that strain effectively facilitates the relaxation dynamics with divergent modulation behaviors in a wide temperature range for metallic glasses. Two loading modes are coupled with linear-heating, and we experimentally confirm the "temperature-strain equivalence". Our results benefit for better understanding the unique effect of strain on relaxation and for providing guidance on applications of metallic glasses with improved performances at different temperatures. (C) 2016 Published by Elsevier Ltd on behalf of Acta Materialia Inc.
SN 1359-6462
PD MAR 15
PY 2017
VL 130
BP 229
EP 233
DI 10.1016/j.scriptamat.2016.12.017
UT WOS:000394194200050
ER

PT J
AU Concustell, A
   Sort, J
   Greer, AL
   Baro, MD
AF Concustell, A
   Sort, J
   Greer, AL
   Baro, MD
TI Anelastic deformation of a Pd40Cu30Ni10P20 bulk metallic glass during
   nanoindentation
SO APPLIED PHYSICS LETTERS
AB Time-dependent deformation processes during nanoindentation have been investigated on a Pd40Cu30Ni10P20 bulk metallic glass. Deformation under constant load has been studied as a function of prior loading rate and temperature. The constant-load displacement of the indenter into the sample shows classic relaxation kinetics and reveals the importance of anelasticity for the mechanical behavior of metallic glasses at the nanoscale. (c) 2006 American Institute of Physics.
RI Sort, Jordi/F-6582-2014; Greer, Lindsay/E-9433-2017; Greer, Alan
   Lindsay/G-1977-2011; Baro, Maria Dolors/A-2096-2009
OI Sort, Jordi/0000-0003-1213-3639; Baro, Maria Dolors/0000-0002-8636-1063;
   Concustell, Amadeu/0000-0001-6705-3228
SN 0003-6951
PD APR 24
PY 2006
VL 88
IS 17
AR 171911
DI 10.1063/1.2198517
UT WOS:000237136600026
ER

PT J
AU Bakai, SO
   Neklyudov, IM
   Savchenko, VI
   Eckert, J
   Kuhn, U
AF Bakai, SO
   Neklyudov, IM
   Savchenko, VI
   Eckert, J
   Kuhn, U
TI About influence of the thin copper shell on a plastic deformation of a
   bulk metallic glass
SO METALLOFIZIKA I NOVEISHIE TEKHNOLOGII
AB The mixed plastic deformation in the bulk metallic glass Zr52.5Ti5Cu17.9Ni14.6Al10 is investigated. The specimen is encapsulated in a thin copper cladding and subjected to uniaxial compression stress at the temperature below the glass-transition temperature by about 30 K. The results obtained agree with and provides additional support to the map of plastic deformation mechanisms of this metallic glass.
SN 1024-1809
PD MAR
PY 2004
VL 26
IS 3
BP 335
EP 342
UT WOS:000222439400006
ER

PT J
AU Kim, JS
   Lee, MH
   Kim, DH
   Kuhn, U
   Eckert, J
AF Kim, J. S.
   Lee, M. H.
   Kim, D. H.
   Kuehn, U.
   Eckert, J.
TI Synthesis of functional porous metallic material from metallic glass
   composites precursor by powder metallurgy route
SO REVUE DE METALLURGIE-CAHIERS D INFORMATIONS TECHNIQUES
AB Bulk metallic glasses foams are under consideration as highly functional material, such as energy absorbers or ultra-lightweight parts, due to their porous structure combined with desirable strengths at relatively low densities. Also, the characteristics of high surface area of foams synthesized from metallic glasses make them viable candidates as emerging materials such as high-sensitivity sensors, catalysts or hydrogen storage media. In current study, bulk metallic glass foams were fabricated by extruding powder mixtures comprised of metallic glasses blended with various kinds of fugitive phases followed by dissolution of the fugitive phases in an aqueous chemical solution, for example HNO3, to yield the final porous structure. As a result of dissolution process, porous metallic glass having uniformly distributed micro-scale porosity was fabricated using a sequential synthesis technique beginning with composite particles comprised of composites of MG and a fugitive phase followed by consolidation of the composite powders via warm extrusion.
RI Kim, Do Hyang/J-6575-2012; bang, changwook/J-7922-2012
OI LEE, MIN HA/0000-0001-6006-0628
SN 0035-1563
PY 2012
VL 109
IS 1
BP 11
EP 16
DI 10.1051/meta1/2012005
UT WOS:000302737200003
ER

PT J
AU Dong, FY
   Su, YQ
   Luo, LS
   Wang, L
   Wang, SJ
   Guo, JJ
   Fu, HZ
AF Dong, Fuyu
   Su, Yanqing
   Luo, Liangshun
   Wang, Liang
   Wang, Shujie
   Guo, Jingjie
   Fu, Hengzhi
TI Deoxidation of bulk metallic glasses by hydrogen arc melting
SO MATERIALS LETTERS
AB Hydrogen arc melting (HAM) was conducted to investigate the deoxidization effects of HAM on Zr-based alloys. The effects of the hydrogen percentage and melting duration on the oxygen content and glass-forming ability of Zr55Cu30Ni5Al10 alloys were studied. Because of the increase in the hydrogen percentage and the melting duration, the oxygen content of the alloys greatly decreased after HAM, which alleviated the detrimental effect of oxygen and improved the glass-forming ability. Thus, HAM-deoxidation may provide an effective and simple method to remove the oxygen from Zr-based metallic glasses, which can improve the prospects for widespread applications of Zr-based metallic glasses. (C) 2012 Elsevier B.V. All rights reserved.
RI Luo, LiangShun/C-7116-2008
OI Luo, LiangShun/0000-0003-3919-938X
SN 0167-577X
PD SEP 15
PY 2012
VL 83
BP 1
EP 3
DI 10.1016/j.matlet.2012.05.101
UT WOS:000308118500001
ER

PT J
AU Takeuchi, S
   Kakegawa, T
   Hashimoto, T
   Tsai, AP
   Inoue, A
AF Takeuchi, S
   Kakegawa, T
   Hashimoto, T
   Tsai, AP
   Inoue, A
TI Low temperature mechanical properties of bulk metallic glasses
SO MATERIALS TRANSACTIONS JIM
AB Compression tests have been performed for bulk metallic glasses of La55Al25Ni20 (L-glass) and Zr41Ti15Ni12CU11Be21 (Z-Glass) at temperatures from 4.2 K up to above the glass transition temperature. Shear fracture stresses are scattered between 0.75 GPa and 1.2 GPa in the L-glass, and between 1.3 GPa and 2.2 GPa in the Z-glass with a tendency of decrease towards both higher and lower temperatures from 77 K in L-glass and from 300 K in Z-glass. The normalized shear fracture stresses with respect to the Young's modulus are around 0.02 in both glasses. The micro-plastic strain measured with the strain gauge for the L-glass is about 0.2% at the shear fracture stress at room temperature, decreases with decreasing temperature and is of the order of 0.01% below 77 K. The mechanism that determines the shear fracture stress is discussed.
RI Inoue, Akihisa/E-5271-2015
SN 0916-1821
PD NOV
PY 2000
VL 41
IS 11
BP 1443
EP 1447
DI 10.2320/matertrans1989.41.1443
UT WOS:000166559500014
ER

PT J
AU Wang, WH
   Bian, Z
   Wen, P
   Zhang, Y
   Pan, MX
   Zhao, DQ
AF Wang, WH
   Bian, Z
   Wen, P
   Zhang, Y
   Pan, MX
   Zhao, DQ
TI Role of addition in formation and properties of Zr-based bulk metallic
   glasses
SO INTERMETALLICS
CT 2nd International Conference on Bulk Metallic Glasses
CY MAR 24-28, 2002
CL CHILUNG, TAIWAN
AB A various multicomponent bulk metallic glasses (BMGs) were prepared at a low cooling rates of 1-100 K/s. The effects of various additions on the glass forming ability (GFA), properties and thermal stability of the alloy systems were investigated. The structural and properties changes of the. BMGs upon addition were studies using X-ray diffraction, differential scanning calorimetry, density measurement, and acoustic measurement. It is found that the proper elemental addition can significantly improve the GFA and properties of the bulk glass-forming alloys. The addition is an effective way for improving GFA, and properties of the bulky glass-forming alloys. The roles of the additions in the glass formation, properties and crystallization are discussed. (C) 2002 Elsevier Science Ltd. All rights reserved.
RI ZHANG, Yong/B-7928-2009
OI ZHANG, Yong/0000-0002-6355-9923
SN 0966-9795
PD NOV-DEC
PY 2002
VL 10
IS 11-12
BP 1249
EP 1257
AR PII S0966-9795(02)00140-1
DI 10.1016/S0966-9795(02)00140-1
UT WOS:000180002100029
ER

PT J
AU Zhang, L
   Ma, E
   Xu, J
AF Zhang, Li
   Ma, Evan
   Xu, Jian
TI Hf-based bulk metallic glasses with critical diameter on centimeter
   scale
SO INTERMETALLICS
AB New Hf-based bulk metallic glasses (BMGs) have been discovered in the Hf-Cu-Ni-Al quaternary system. These interesting BMGs (i) are based on a refractory element and have relatively high glass transition and crystallization temperatures, (ii) exhibit excellent glass-forming ability with a critical diameter in the 8-10 mm range for copper mold casting, and (iii) have relatively high mass density (> similar to 11 g/cc). The combination of these properties is important for the BMG matrix in composites for kinetic energy penetrator applications. (C) 2008 Elsevier Ltd. All rights reserved.
RI Ma, En/A-3232-2010
SN 0966-9795
PD APR
PY 2008
VL 16
IS 4
BP 584
EP 586
DI 10.1016/j.intermet.2007.12.016
UT WOS:000255601900013
ER

PT J
AU Jiang, MQ
   Huang, BM
   Jiang, ZJ
   Lu, C
   Dai, LH
AF Jiang, M. Q.
   Huang, B. M.
   Jiang, Z. J.
   Lu, C.
   Dai, L. H.
TI Joining of bulk metallic glass to brass by thick-walled cylinder
   explosion
SO SCRIPTA MATERIALIA
AB This paper reports the development of a thick-walled cylinder explosion technique to weld a typical Zr-based bulk metallic glass (Vitreloy 1 (Vit 1)) to a commercial Cu-based crystalline alloy (brass). It is shown that a strong metallurgical bonding between the Vit 1 and the brass is achieved, which is due to significant atomic diffusion across the welding interface and shock wave propagation in the weldment. The dissimilar joining of the noncrystalline to crystalline alloy extends the application of bulk metallic glasses as structural and functional materials. (C) 2014 Acta Materialia Inc. Published by Elsevier Ltd. All rights reserved.
RI Lu, Chunsheng/B-9489-2008
OI Lu, Chunsheng/0000-0002-7368-8104; Dai, LanHong/0000-0001-8991-0358
SN 1359-6462
PD MAR 1
PY 2015
VL 97
BP 17
EP 20
DI 10.1016/j.scriptamat.2014.10.027
UT WOS:000346952900005
ER

PT J
AU Yang, XH
   Ma, XH
   Li, Q
   Guo, SF
AF Yang, Xiaohui
   Ma, Xiuhua
   Li, Qiang
   Guo, Shengfeng
TI The effect of Mo on the glass forming ability, mechanical and magnetic
   properties of FePC ternary bulk metallic glasses
SO JOURNAL OF ALLOYS AND COMPOUNDS
AB Bulk magnetic Fe80-xMoxP13C7 (x = 0, 3, 6, 9 and 12 at.%) glassy alloys were successfully prepared by the fluxing technique and J-quenching technique. The effect of Mo substitution for Fe on the glass formation ability (GFA), thermal stability, mechanical properties and magnetic properties has been studied systematically. It was found that the substitution of a small amount of Mo for Fe can dramatically enhance the GFA, which in turn, the critical maximum diameter for fully glass formation reached to 5.5 mm for x = 3 and 6.0 mm for x = 6, respectively. However, excessive substitution will promote the formation of Fe2Mo3 competition crystalline phase leading to the degradation of the GFA. Furthermore, the substitution of proper content of Mo for Fe can obviously enhance the mechanical properties of the present Fe-based alloys, which can be identified that the current FeMoPC bulk metallic glass possesses a high fracture strength (over 3000 MPa) as well as a large room temperature plasticity (above 5%). In addition, the Mo doping FePC bulk metallic glasses exhibit good soft magnetic properties with a relatively high saturation magnetization of over 1.0 T at room temperature. (C) 2012 Elsevier B. V. All rights reserved.
RI Li, Qiang/D-1865-2011; Guo, Shengfeng/E-3171-2012
OI Li, Qiang/0000-0002-1891-5490; Guo, Shengfeng/0000-0002-6667-6797
SN 0925-8388
EI 1873-4669
PD MAR 25
PY 2013
VL 554
BP 446
EP 449
DI 10.1016/j.jallcom.2012.11.170
UT WOS:000314642200070
ER

PT J
AU Tang, MB
   Zhao, DQ
   Pan, MX
   Wang, WH
AF Tang, MB
   Zhao, DQ
   Pan, MX
   Wang, WH
TI Binary Cu-Zr bulk metallic glasses
SO CHINESE PHYSICS LETTERS
AB We report that bulk metallic glasses (BMGs) can be produced up to 2mm by a copper mould casting in CuxZr1-x binary alloy with a wide glass forming composition range (45 < x < 60at.%). We find that the formation mechanism for the binary Cu-Zr binary BMG-forming alloy is obviously different from that of the intensively studied multicomponent BMGs. Our results demonstrate that the criteria for the multicomponent alloys with composition near deep eutectic and strong liquid behaviour are no longer the major concern for designing BMGs.
SN 0256-307X
PD MAY
PY 2004
VL 21
IS 5
BP 901
EP 903
UT WOS:000221175300039
ER

PT J
AU He, MK
   Chen, SH
   Yu, P
   Xia, L
AF He, M. K.
   Chen, S. H.
   Yu, P.
   Xia, L.
TI Enhanced mechanical properties of Ni62Nb38 bulk metallic glasses by Ta
   substitution
SO JOURNAL OF NON-CRYSTALLINE SOLIDS
AB By substituting Ta for Nb in a binary Ni62Nb38 bulk metallic glass (BMG), ternary Ni62Nb38-xTax (x = 5-35) BMGs were fabricated by copper mold suction casting. The present NiNbTa BMGs not only have good glass forming ability (GFA), but also have combinations of both high strength and relatively-large plastic strains, which are superior to the binary Ni62Ni38 BMG and some other Ni-Nb-based BMGs. The Ni62Nb18Ta20 BMG has fracture strength of about 3.2 GPa and a plastic strain of about 1.36%. The enhancement of the mechanical properties of the NiNbTa BMGs is related to the change of the liquid behavior and glass forming ability by Ta-substitution.
SN 0022-3093
EI 1873-4812
PD SEP 1
PY 2017
VL 471
BP 452
EP 455
DI 10.1016/j.jnoncrysol.2017.07.004
UT WOS:000407188100062
ER

PT J
AU Gonzalez, S
   Figueroa, IA
   Todd, I
AF Gonzalez, S.
   Figueroa, I. A.
   Todd, I.
TI Influence of minor alloying additions on the glass-forming ability of
   Mg-Ni-La bulk metallic glasses
SO JOURNAL OF ALLOYS AND COMPOUNDS
AB Bulk metallic glasses of Mg(60)Ni(23.6)Y(x)La((16.4-x)) and Mg(65)Ni(20)Y(x)LaMM((15-x)) with 0 <= x <= 1 at.% have been produced by injection casting. For the La-containing alloy a maximum amorphous diameter of 4 mm for x = 0.5 and 0.75 was obtained. The LaMM-containing alloy showed a maximum amorphous diameter of 2 mm for x = 0 and 0.25 but decreased to 1 mm with further Y additions. The glass-forming ability of the Mg(60)Ni(23.6)La(16.4) alloy decreased when La is partially substituted by small amounts of small atoms (Si or B) or by large atoms (Y and Si). (C) 2009 Elsevier B.V. All rights reserved.
RI Gonzalez, Sergio/A-4852-2010; Figueroa, Ignacio/J-5914-2012
OI Todd, Iain/0000-0003-0217-1658; Gonzalez Sanchez,
   Sergio/0000-0002-0211-7822
SN 0925-8388
PD SEP 18
PY 2009
VL 484
IS 1-2
BP 612
EP 618
DI 10.1016/j.jallcom.2009.05.002
UT WOS:000271334900116
ER

PT J
AU Stoica, M
   Das, J
   Bednarcik, J
   Wang, G
   Vaughan, G
   Wang, WH
   Eckert, J
AF Stoica, Mihai
   Das, Jayanta
   Bednarcik, Jozef
   Wang, Gang
   Vaughan, Gavin
   Wang, Wei Hua
   Eckert, Juergen
TI Mechanical Response of Metallic Glasses: Insights from In-situ High
   Energy X-ray Diffraction
SO JOM
AB The term "metallic glass" usually refers to a metallic alloy rapidly quenched in order to "freeze" its structure from the liquid state. A metallic glass is a metastable alloy, which lacks the symmetry typical for crystalline materials and at room temperature shows an amorphous liquid-like structure. Bulk metallic glasses (BMGs) represent a class of amorphous alloys. The most notable property of BMGs is their ultrahigh (near theoretical) strength and hardness. Because the known BMGs usually miss tensile plasticity and thus exhibit catastrophic failure upon tension it is important to understand deformation mechanisms involved and thus improve their performance. This aricle analyzes the use of synchrotron radiation for evaluating the elastic-plastic response of such materials.
RI Das, Jayanta/G-1559-2010; Stoica, Mihai/B-7069-2015; Vaughan,
   Gavin/B-1479-2010; Wang, Gang/K-2630-2012
OI Das, Jayanta/0000-0001-8750-5463;
SN 1047-4838
PD FEB
PY 2010
VL 62
IS 2
BP 76
EP 82
DI 10.1007/s11837-010-0037-3
UT WOS:000274211000015
ER

PT J
AU Wang, XY
   Tang, N
   Zheng, ZZ
   Tang, YY
   Li, JJ
AF Wang, Xinyun
   Tang, Na
   Zheng, Zhizhen
   Tang, Yingying
   Li, Jianjun
TI Constitutive Equation of Zr-Based Bulk Metallic Glasses in Supercooled
   Liquid Region
SO STEEL RESEARCH INTERNATIONAL
AB Compression experiments of Zr55Cu30Al10Ni5 bulk metallic glass were conducted in a supercooled liquid region with a Zwick/Roell mechanical testing machine. According to the overshoot phenomenon in stress-strain curves, a Maxwell-Pulse constitutive model was proposed, which combines a visco-elastic Maxwell model and pulse function. The parameters of the Maxwell-Pulse constitutive model were obtained by fitting a fictive stress model and pulse function. Compared the data of experiments with that of the fitted constitutive model, they show good agreement, especially, the phenomenon of overshoot also fits well. Finally, through numerical simulation and micro-backward extrusion experiment, it confirms that the constitutive model can describe the mechanical behaviour of Zr55Cu30Al10Ni5 bulk metallic glass in supercooled liquid region very well.
RI wang, xy/C-2888-2015
SN 1611-3683
EI 1869-344X
PD SEP
PY 2010
VL 81
IS 9
SI SI
BP 1400
EP 1403
UT WOS:000208594600346
ER

PT J
AU Park, ES
   Ohnuma, M
   Kim, DH
AF Park, E. S.
   Ohnuma, M.
   Kim, D. H.
TI Anomalous glass transition behavior in Cu-Zr-Sn alloy system
SO JOURNAL OF ALLOYS AND COMPOUNDS
CT 17th International Symposium on Metastable, Amorphous and Nanostructured
   Materials (ISMANAM 2010)
CY JUL 04-09, 2010
CL Zurich, SWITZERLAND
AB We discuss the effect of Sn addition on anomalous glass transition behavior in Cu-Zr bulk-forming metallic glasses. We found that an unusual endothermic reaction in Cu(55)Zr(40)Sn(5) ribbon can originate from the growth reaction of quenched-in nuclei from 0.4nm to 3.7nm in the supercooled liquid region. This anomalous devitrification may prove useful for the synthesis of a novel composite with uniform atomic/nanometer scale heterogeneity modulated by controlling cooling rate as well as by tailoring alloy composition. (C) 2011 Elsevier B.V. All rights reserved.
RI Park, Eun Soo/A-4443-2008; bang, changwook/J-7922-2012; Park, Eun
   Soo/A-9860-2014; Kim, Do Hyang/J-6575-2012
SN 0925-8388
PD JUN
PY 2011
VL 509
SU 1
BP S52
EP S55
DI 10.1016/j.jallcom.2011.01.094
UT WOS:000291463000012
ER

PT J
AU Ma, H
   Shi, LL
   Xu, J
   Li, Y
   Ma, E
AF Ma, H
   Shi, LL
   Xu, J
   Li, Y
   Ma, E
TI Discovering inch-diameter metallic glasses in three-dimensional
   composition space
SO APPLIED PHYSICS LETTERS
AB We have discovered low-cost bulk metallic glasses (BMGs) with diameters as large as 16 to 25 mm based on the ordinary engineering metal Mg. We also use this example to present a new strategy, to illustrate how to navigate in three-dimensional composition space for locating the best glass-forming compositions. The extraordinary glass-forming ability demonstrated by the inch-sized BMGs has implications for future development and applications of amorphous metals. (C) 2005 American Institute of Physics.
RI Ma, En/A-3232-2010
SN 0003-6951
PD OCT 31
PY 2005
VL 87
IS 18
AR 181915
DI 10.1063/1.2126794
UT WOS:000232886400025
ER

PT J
AU Zhang, J
   Park, JM
   Kim, DH
   Kim, HS
AF Zhang, Jia
   Park, Jin Man
   Kim, Do Hyang
   Kim, Hyoung Seop
TI Effect of strain rate on compressive behavior of Ti45Zr16Ni9Cu10Be20
   bulk metallic glass
SO MATERIALS SCIENCE AND ENGINEERING A-STRUCTURAL MATERIALS PROPERTIES
   MICROSTRUCTURE AND PROCESSING
CT 12th International Conference on Rapidly Quenched and Metastable
   Materials
CY AUG 21-26, 2005
CL Jeju Isl, SOUTH KOREA
AB Compressive behavior of Ti45Zr16Ni9Cu10Be20 (numbers indicate at.%) bulk metallic glasses was investigated at strain rates ranging from 10(-4) to 10(-1) s(-1). Little effect of strain rate on the mechanical properties is observed in an amorphous specimen when the strain rates are below 10(-3)s(-1). An appreciable total strain of about 5% is observed and the plastic flow is serrated. However, a positive strain rate dependence of yield strength is found when the strain rate reaches 10(-1) s(-1), which is reported for the first time in metallic glasses under a compressive loading condition. Further, the macroscopic plastic strain distinctly decreases and flow serration vanishes at a high strain rate. A comparison of the significantly different mechanical behavior of Ti45Zr16Ni9Cu10Be20 bulk metallic glass with other bulk metallic glass systems is also presented. (c) 2006 Elsevier B.V. All rights reserved.
RI Kim, Hyoung Seop/C-2166-2009; bang, changwook/J-7922-2012
OI Kim, Hyoung Seop/0000-0002-3155-583X;
SN 0921-5093
PD MAR 25
PY 2007
VL 449
BP 290
EP 294
DI 10.1016/j.msea.2006.02.405
UT WOS:000245477800063
ER

PT J
AU Jang, DC
   Maass, R
   Wang, GY
   Liaw, PK
   Greer, JR
AF Jang, Dongchan
   Maass, Robert
   Wang, Gongyao
   Liaw, Peter K.
   Greer, Julia R.
TI Fatigue deformation of microsized metallic glasses
SO SCRIPTA MATERIALIA
AB Metallic glasses typically exhibit high strength and a high elastic limit but suffer from poor fatigue resistance. This work demonstrates that 1.6 micron diameter Zr-based metallic glass samples subjected to compressive fatigue cycling did not fail after 40 x 10(6) cycles. The fatigue endurance limit was shown to increase to more than 110% of bulk yield strength under compression-compression and up to 90% under bending; those of the same material with macroscopic dimensions are typically at 50% of bulk yield strength. (c) 2012 Acta Materialia Inc. Published by Elsevier Ltd. All rights reserved.
RI Jang, Dongchan/C-9510-2012; Maass, Robert/F-6306-2011
OI Jang, Dongchan/0000-0002-2814-9734;
SN 1359-6462
PD MAY
PY 2013
VL 68
IS 10
BP 773
EP 776
DI 10.1016/j.scriptamat.2012.12.011
UT WOS:000316976500002
ER

PT J
AU Laws, KJ
   Shamlaye, KF
   Ferry, M
AF Laws, Kevin J.
   Shamlaye, Karl F.
   Ferry, Michael
TI Synthesis of Ag-based bulk metallic glass in the Ag-Mg-Ca-[Cu] alloy
   system
SO JOURNAL OF ALLOYS AND COMPOUNDS
AB A number of novel silver-based bulk metallic glasses from the Ag-Mg-Ca ternary and Ag-Mg-Ca-Cu quaternary alloy systems have been discovered. These amorphous alloys are located over a broad composition range from (at.%): 30 to 61% Ag, 7 to 38% Mg, 7 to 30% Ca and 0 to 15% Cu often far from ternary eutectic reactions. These new amorphous alloys have been developed in light of their potential applications as functional materials with high electrical and thermal conductivity combined with the superplastic formability of a metallic glass. Reported are the critical casting size and thermophysical properties of these bulk glassy alloys. (C) 2011 Elsevier B.V. All rights reserved.
SN 0925-8388
EI 1873-4669
PD FEB 5
PY 2012
VL 513
BP 10
EP 13
DI 10.1016/j.jallcom.2011.10.097
UT WOS:000298156400003
ER

PT J
AU Fu, HM
   Zhang, HF
   Wang, H
   Hu, ZQ
AF Fu, H. M.
   Zhang, H. F.
   Wang, H.
   Hu, Z. Q.
TI Cu-based bulk amorphous alloy with larger glass-forming ability and
   supercooled liquid region
SO JOURNAL OF ALLOYS AND COMPOUNDS
AB The glassy rod with a maximum sample thickness of I I mm and larger supercooled liquid region of 108 K was successfully fabricated when substituting Cu with minor amount of Ag in the Cu-Zr-Al-Gd alloy system. The value of gamma reaches a maximum of 0.418 for the Cu45.5Zr45Al7Gd2Ag0.5 bulk metallic glass (BMG) alloy. The high glass-forming ability (GFA) and larger supercooled liquid region are discussed from atomic size, negative mixing heat among constituent elements and thermodynamics. (c) 2007 Elsevier B.V. All rights reserved.
SN 0925-8388
PD JUN 30
PY 2008
VL 458
IS 1-2
BP 390
EP 393
DI 10.1016/j.jallcom.2007.03.114
UT WOS:000256641200069
ER

PT J
AU Li, YH
   Zhang, W
   Dong, C
   Qiang, JB
   Yubuta, K
   Makino, A
   Inoue, A
AF Li, Y. H.
   Zhang, W.
   Dong, C.
   Qiang, J. B.
   Yubuta, K.
   Makino, A.
   Inoue, A.
TI Unusual compressive plasticity of a centimeter-diameter Zr-based bulk
   metallic glass with high Zr content
SO JOURNAL OF ALLOYS AND COMPOUNDS
CT 16th International Symposium on Metastable, Amorphous and Nanostructured
   Materials
CY JUL 05-09, 2009
CL Beijing, PEOPLES R CHINA
AB The thermal stability, melting behavior, and glass-forming ability of Zr(70)Al(8)Cu(22-x)Ni(x) (x = 3-19 at.%) metallic glasses were investigated. Fully glassy sample with largest critical diameter of 10 mm was obtained for alloy with x = 8.5 in the alloy series, which corresponds to a deep eutectic, largest supercooled liquid region, and largest gamma value. Room temperature compression test revealed that the monolithic Zr(70)Al(8)Cu(13.5)Ni(8.5) bulk metallic glass exhibits a high yield strength of 1570 MPa, a Young's modulus of 80 GPa, and a super large plastic strain more than 70%. (C) 2010 Elsevier B.V. All rights reserved.
RI MAKINO, AKIHIRO/B-2549-2009; Yubuta, Kunio/B-8124-2011; Inoue,
   Akihisa/E-5271-2015
OI Yubuta, Kunio/0000-0002-3401-6874;
SN 0925-8388
PD AUG
PY 2010
VL 504
SU 1
BP S2
EP S5
DI 10.1016/j.jallcom.2010.02.069
UT WOS:000285252600002
ER

PT J
AU Zhang, XL
   Sun, GY
   Chen, G
AF Zhang, X. L.
   Sun, G. Y.
   Chen, G.
TI Improving the strength and the toughness of Mg-based bulk metallic glass
   by Bridgman solidification
SO MATERIALS SCIENCE AND ENGINEERING A-STRUCTURAL MATERIALS PROPERTIES
   MICROSTRUCTURE AND PROCESSING
AB The Mg-based bulk metallic glass composites with a composition of Mg75Cu13.33Y6.67Zn5 were synthesized by the copper-mould casting and the Bridgman solidification. Well-developed and homogeneously-distributed flake-shape precipitates were obtained in the in situ bulk metallic glass matrix composites by the Bridgman solidification. The glass composite obtained exhibits the ultimate strength of 1037 MPa and fracture plastic strain of 19% of the composite, which is attributed to the homogeneous dispersion of ductile phase in the glass matrix. As compared to the copper-mould casting method, the Bridgman solidification is more effective to generate the homogeneous in situ Mg-based bulk metallic glass composites. (C) 2012 Elsevier B.V. All rights reserved.
SN 0921-5093
PD MAR 1
PY 2013
VL 564
BP 158
EP 162
DI 10.1016/j.msea.2012.11.111
UT WOS:000315748100022
ER

PT J
AU Li, Y
   Yu, P
   Bai, HY
AF Li, Y
   Yu, P
   Bai, HY
TI Observation of low-temperature specific-heat anomaly in CuZrAl bulk
   metallic glasses
SO APPLIED PHYSICS LETTERS
AB Low-temperature specific heat and ultrasonic experiments on CuZrAl bulk metallic glass (BMG) are performed. The low-temperature specific-heat anomaly arising from the low-frequency vibrations responsible for the boson peak, which is correlated with the nature and physical properties of the glasses, is clearly observed in the BMG. The ratio of the excess specific heat to the Debye contribution around the maximum is 1.93 for the BMG. (c) 2005 American Institute of Physics.
SN 0003-6951
EI 1077-3118
PD JUN 6
PY 2005
VL 86
IS 23
AR 231909
DI 10.1063/1.1946898
UT WOS:000230087400018
ER

PT J
AU Li, R
   Stoica, M
   Wang, G
   Park, JM
   Li, Y
   Zhang, T
   Eckert, J
AF Li, Ran
   Stoica, Mihai
   Wang, Gang
   Park, Jin Man
   Li, Yan
   Zhang, Tao
   Eckert, Juergen
TI Glass formation, thermal properties, and elastic constants of La-Al-Co
   alloys
SO JOURNAL OF MATERIALS RESEARCH
AB The compositional dependence of glass formation and thermal and elastic properties was clarified for the ternary La-Al-Co bulk glass-forming system. The existing linear correlation between La concentration and characteristic temperatures, i.e., the glass transition temperature T-g and the onset temperature of crystallization T-x, as well as the elastic moduli in this system can give a useful guideline for the chemical design of desirable bulk metallic glasses (BMGs) with tunable physical properties in advance. The relationship between T-g and elastic constants for the La-Al-Co BMGs can be quantitatively described using a microscopic model proposed by T. Egami.
RI Li, Ran/B-4618-2010; Zhang, Tao/O-4911-2014; Stoica, Mihai/B-7069-2015;
   Wang, Gang/K-2630-2012
SN 0884-2914
EI 2044-5326
PD JUL
PY 2010
VL 25
IS 7
BP 1398
EP 1404
DI 10.1557/JMR.2010.0177
UT WOS:000279475500024
ER

PT J
AU Zhang, YQ
   Gong, HX
   Li, PF
   Tian, YJ
   Wang, LM
AF Zhang, Yaqi
   Gong, Hongxiang
   Li, Pengfei
   Tian, Yongjun
   Wang, Li-Min
TI Glass formation in narrow band-gap SnTe-based chalcogenide systems
SO MATERIALS LETTERS
AB Using specified telluride glasses to prepare the glass-ceramics containing narrow band-gap telluride crystals as the main precipitated phase would be an effective route to developing high-efficiency thermoelectric materials. Unfortunately, such telluride glasses are extremely difficult to achieve. In this paper, a strategy is proposed to vitrify these narrow band-gap tellurides by jointly focusing on the glass formation thermodynamic and kinetic factors. Novel Ga2Te3-SnTe glasses are successfully synthesized and the glass-forming region is found to be located at the SnTe-rich compositions. (C) 2017 Elsevier B.V. All rights reserved.
RI Tian, Yongjun/A-1693-2010
OI Tian, Yongjun/0000-0002-4594-4879
SN 0167-577X
EI 1873-4979
PD MAY 1
PY 2017
VL 194
BP 149
EP 151
DI 10.1016/j.matlet.2017.02.042
UT WOS:000397353700039
ER

PT J
AU Lagos, M
   Das, R
AF Lagos, Miguel
   Das, Raj
TI Brittle and Ductile Character of Amorphous Solids
SO ADVANCES IN APPLIED MATHEMATICS AND MECHANICS
AB Common silicate glasses are among the most brittle of the materials. However, on warming beyond the glass transition temperature T-g glass transforms into one of the most plastic known materials. Bulk metallic glasses exhibit similar phenomenology, indicating that it rests on the disordered structure instead on the nature of the chemical bonds. The micromechanics of a solid with bulk amorphous structure is examined in order to determine the most basic conditions the system must satisfy to be able of plastic flow. The equations for the macroscopic flow, consistent with the constrictions imposed at the atomic scale, prove that a randomly structured bulk material must be either a brittle solid or a liquid, but not a ductile solid. The theory permits to identify a single parameter determining the difference between the brittle solid and the liquid. However, the system is able of perfect ductility if the plastic flow proceeds in two dimensional plane layers that concentrate the strain. Insight is gained on the nature of the glass transition, and the phase occurring between glass transition and melting.
OI Das, Raj/0000-0001-9977-6201
SN 2070-0733
EI 2075-1354
PD JUN
PY 2016
VL 8
IS 3
BP 485
EP 498
DI 10.4208/aamm.2013.m439
UT WOS:000369433100007
ER

PT J
AU Kim, JH
   Park, JS
   Fleury, E
   Kim, WT
   Kim, DH
AF Kim, JH
   Park, JS
   Fleury, E
   Kim, WT
   Kim, DH
TI Effect of yttrium addition on thermal stability and glass forming
   ability in Fe-TM (Mn, Mo, Ni)-B ternary alloys
SO MATERIALS TRANSACTIONS
AB The effect of Y addition on the glass forming ability (GFA) of in Fe70TM(TM=Mn,Mo,Ni)(13)B-17 alloys have been examined in the present study. Substitution of 3-10 at% Fe with Y improves the GFA. Fe70-xMn13B17Yx (x = 5) and Fe70-xMo13B17Yx (x = 3) alloys exhibit high T-rg (= T-g/T-l) (>0.55) and gamma (= T-x/[T-l + T-g]) (>0.37) values enabling fabrication of 1 mm diameter bulk metallic glass. The GFA improves, when addition of Y decreases the melting temperature, and, at the same time, increases the crystallization onset temperature. Extended Kissinger analysis of the DSC data indicates that a kinetic boundary condition for the formation of the amorphous phase widens by addition of Y.
RI bang, changwook/J-7922-2012
SN 1345-9678
EI 1347-5320
PD AUG
PY 2004
VL 45
IS 8
BP 2770
EP 2775
DI 10.2320/matertrans.45.2770
UT WOS:000223707600060
ER

PT J
AU Liu, HB
   Li, JF
   Cao, QP
   Zhou, YH
AF Liu HuiBin
   Li JinFu
   Cao QingPing
   Zhou YaoHe
TI Free-volume evolution of glassy Zr65Al7.5Ni10Cu17.5 during inhomogeneous
   deformation
SO CHINESE SCIENCE BULLETIN
AB Free-volume evolutions of the as-cast and pre-annealed Zr65Al7.5Ni10Cu17.5 metallic glasses during cold-rolling were investigated using differential scanning calorimetry. As deformation degree increases, free volume in the glasses always increases first and then saturates above a critical strain, while the structure remains completely amorphous. The glass with less initial free volume possesses a higher increase rate of free-volume content during deformation, and as a result the free-volume contents of all the glasses approach each other at higher strains. Coalescence of free volume in shear bands into nanovoids is proposed to be responsible for the saturation of free-volume content at high strains.
RI Cao, Qing Ping/A-1055-2010
SN 1001-6538
PD DEC
PY 2007
VL 52
IS 24
BP 3443
EP 3447
DI 10.1007/s11434-008-0014-1
UT WOS:000252170700018
ER

PT J
AU Qiu, KQ
   Ren, YL
AF Qiu, KQ
   Ren, YL
TI Tungsten particulate rreinforced zr based bulk metallic glass composites
SO RARE METAL MATERIALS AND ENGINEERING
AB The Zr41.25Ti13.75Cu12.5Ni10Be22.5 bulk metallic glass is reinforced with up to 60 volume percent of tungsten particulate. Samples with 10mm in diameter are obtained when quenching melt contained in stainless steel tube into saturated brine. A characterization based on X-ray diffraction and scanning electron microscopy is presented. Compressive tests were performed for the metallic glass matrix composites and single phase bulk metallic glass. Compressive strain-to-failure increase significantly compared to unreinforced single phase Zr41.25Ti13.75Cu12.5Ni10Be22.5 bulk metallic glass. The results are discussed in term of particulate size and yield strength of the reinforcement.
SN 1002-185X
PD JAN
PY 2006
VL 35
IS 1
BP 66
EP 69
UT WOS:000235118100017
ER

PT J
AU Zhang, HY
   Zheng, GP
AF Zhang, H. Y.
   Zheng, G. P.
TI Three-dimensional phase-field simulation on the deformation of metallic
   glass nanowires
SO JOURNAL OF ALLOYS AND COMPOUNDS
CT International Symposium on Metastable, Amorphous and Nanostructured
   Materials (ISMANAM)
CY JUN 30-JUL 05, 2013
CL Torino, ITALY
AB It is very challenging to investigate the deformation mechanisms in micro- and nano-scale metallic glasses with diameters below several hundred nanometers using the atomistic simulation or the experimental approaches. In this work, we develop the fully three-dimensional phase-field model to bridge this gap and investigate the sample size effects on the deformation behaviors of metallic glass nanowires. The initial deformation defects on the surface are found to significantly affect the mechanical strength and deformation mode of nanowires. The improved ductility of metallic glass nanowires could be related with the multiple shear bands initiated from the nanowire surfaces. (C) 2013 Elsevier B.V. All rights reserved.
OI ZHENG, Guang Ping/0000-0003-3445-0547
SN 0925-8388
EI 1873-4669
PD DEC 5
PY 2014
VL 615
SU 1
BP S102
EP S107
DI 10.1016/j.jallcom.2013.12.114
UT WOS:000343613600022
ER

PT J
AU Lu, YZ
   Huang, YJ
   Zheng, W
   Shen, J
AF Lu, Yunzhuo
   Huang, Yongjiang
   Zheng, Wei
   Shen, Jun
TI Free volume and viscosity of Fe-Co-Cr-Mo-C-B-Y bulk metallic glasses and
   their correlation with glass-forming ability
SO JOURNAL OF NON-CRYSTALLINE SOLIDS
AB In this work, the variation of the glass-forming ability (GFA) in Fe-Co-Cr-Mo-C-B-Y alloy system with Co addition has been investigated from the viewpoints of non-isothermal viscosity and free volume. The best GFA for the alloy containing 7% Co is found to closely relate with its highest viscosity, and the minimum sizes and lowest concentrations of the intermediate and largest open volumes. The results may provide more insights into the formation mechanism of metallic glasses. (C) 2012 Elsevier B.V. All rights reserved.
RI Huang, Yongjiang/D-4809-2009
OI Lu, Yunzhuo/0000-0002-7322-6815
SN 0022-3093
PD MAY 15
PY 2012
VL 358
IS 10
BP 1274
EP 1277
DI 10.1016/j.jnoncrysol.2012.02.033
UT WOS:000304226900005
ER

PT J
AU Louzguine-Luzgin, DV
   Polkin, VI
AF Louzguine-Luzgin, D. V.
   Polkin, V. I.
TI Properties of Bulk Metallic Glasses
SO RUSSIAN JOURNAL OF NON-FERROUS METALS
AB A relatively small number of revolutionary materials have been discovered in the field of physical metallurgy and metal physics in the last several decades, and bulk metallic glasses are among them. Their strength and hardness are considerably higher and their modulus of normal elasticity is considerably lower than that of crystalline alloys, which leads to large stored elastic strain energy. These materials also have very good corrosion resistance. In this article, we present the properties of bulk metallic glasses, in particular, thermal, mechanical, magnetic, and electrical properties, corrosion resistance, as well as the application fields of these alloys.
RI LOUZGUINE, Dmitri/D-2492-2010
OI LOUZGUINE, Dmitri/0000-0001-5716-4987
SN 1067-8212
EI 1934-970X
PD JAN
PY 2017
VL 58
IS 1
BP 80
EP 92
DI 10.3103/S1067821217010084
UT WOS:000396389200012
ER

PT J
AU Shi, JL
AF Shi, Jianlin
TI Amorphous iron nanoparticles: special structural and physicochemical
   features enable chemical dynamic therapy for tumors
SO NANOMEDICINE
SN 1743-5889
EI 1748-6963
PY 2016
VL 11
IS 10
BP 1189
EP 1191
DI 10.2217/nnm-2016-0039
UT WOS:000374771100001
PM 27080928
ER

PT J
AU Yu, HB
   Yu, P
   Wang, WH
   Bai, HY
AF Yu, H. B.
   Yu, P.
   Wang, W. H.
   Bai, H. Y.
TI Thulium-based bulk metallic glass
SO APPLIED PHYSICS LETTERS
AB We report the formation and properties of a thulium-based bulk metallic glass (BMG). Compared with other known rare-earth (RE) based BMGs, Tm-based BMGs show features of excellent glass formation ability, considerable higher elastic modulus, smaller Poisson's ratio, high mechanical strength, and intrinsic brittleness. The reasons for the different properties between the Tm-based and other RE-based BMGs are discussed. It is expected that the Tm-based glasses with the unique properties are appropriate candidates for studying some important issues in BMGs. (C) 2008 American Institute of Physics.
RI Yu, Hai Bin/E-5312-2010
OI Yu, Hai Bin/0000-0003-0645-0187
SN 0003-6951
EI 1077-3118
PD APR 7
PY 2008
VL 92
IS 14
AR 141906
DI 10.1063/1.2908047
UT WOS:000254940500025
ER

PT J
AU Kobata, J
   Kimura, T
   Takigawa, Y
   Uesugi, T
   Kimura, H
   Higashi, K
AF Kobata, Junpei
   Kimura, Tsuyoshi
   Takigawa, Yorinobu
   Uesugi, Tokuteru
   Kimura, Hisamichi
   Higashi, Kenji
TI Effect of Pre-Introduced Shear Bands Direction on Deformation Behavior
   in Zr55Al10Ni5Cu30 Bulk Metallic Glass
SO MATERIALS TRANSACTIONS
AB We investigated the effect of pre-introduced shear bands direction on compressive deformation behavior in Zr55Al10Ni5Cu30 bulk metallic glass. The compressive deformation behavior varied with the difference of the direction of pre-introduced shear bands with respect to loading direction. The specimen in which shear bands were introduced at an angle of 45 degree to the loading direction showed largest plastic strain. This large plasticity was achieved clue to the activity of pre-introduced shear bands and formation of multiple shear bands. The direction of pre-introduced shear bands is an important factor to improve the plasticity in bulk metallic glasses. [doi:10.2320/matertrans.MAW200920]
RI Uesugi, Tokuteru/B-9776-2011; Higashi, Kenji/C-5540-2011; Kimura,
   Hisamichi/D-5449-2012; Takigawa, Yorinobu/A-6555-2010
OI Takigawa, Yorinobu/0000-0003-0321-6336
SN 1345-9678
EI 1347-5320
PD OCT
PY 2009
VL 50
IS 10
BP 2355
EP 2358
DI 10.2320/matertrans.MAW200920
UT WOS:000272070600003
ER

PT J
AU Abe, T
   Shimono, M
   Hashimoto, K
   Hono, K
   Onodera, H
AF Abe, T.
   Shimono, M.
   Hashimoto, K.
   Hono, K.
   Onodera, H.
TI Phase separation and glass-forming abilities of ternary alloys
SO SCRIPTA MATERIALIA
AB The liquid phase miscibility gap was calculated in ternary glass-forming systems using the sub-regular solution model. We found that liquid miscibility gaps exist at low temperature in most of the reported ternary bulk-forming metallic glass systems even if enthalpies of mixing of constituent elements are all negative. The glass-forming ranges do not overlap with the miscibility gaps except in the Cu-Ti-Zr system, suggesting that glass-forming ability is low in the phase separating compositions. (c) 2006 Acta Materialia Inc. Published by Elsevier Ltd. All rights reserved.
RI abe, taichi/H-2999-2011; SHIMONO, Masato/H-2699-2011; Hono,
   Kazuhiro/B-9202-2008
OI abe, taichi/0000-0002-5065-0939; Hono, Kazuhiro/0000-0001-7367-0193
SN 1359-6462
PD SEP
PY 2006
VL 55
IS 5
BP 421
EP 424
DI 10.1016/j.scriptamat.2006.05.020
UT WOS:000239132200001
ER

PT S
AU Sakino, Y
   Kuroda, T
   Kim, YC
AF Sakino, Yoshihiro
   Kuroda, Toshio
   Kim, You-Chul
BE Ewsuk, K
   Naito, M
   Kakeshita, T
   Kirihara, S
   Uematsu, K
   Abe, H
TI TENSILE BEHAVIOR OF Zr-BASED BULK METALLIC GLASS IN HIGH-SPEED TENSILE
   TESTS
SO CHARACTERIZATION AND CONTROL OF INTERFACES FOR HIGH QUALITY ADVANCED
   MATERIALS III
SE Ceramic Transactions
CT 3rd International Conference on Characterization and Control of
   Interfaces for High Quality Advanced Materials
CY 2009
CL Kurashiki, JAPAN
SP Amer Ceram Soc
AB High-speed tensile tests on Zr-based bulk metallic glass were carried out to determine the effect of strain rate on the tensile behavior of metallic glasses. In these tests, two strain rates, 1.7 x 10(0)/s and 2.3 x 10(1)/s, were selected, and the results were compared to those of the static test (1.8 x 10(-5)/s). Zr(48)Cu(36)Al(8)Ag(8) bulk metallic glass was chosen for this study. The diameter, gauge length, and overall length of the bulk specimen were 6 mm, 30 mm, and 60 mm, respectively.
   Regardless of the strain rate, all the specimens fractured in the elastic range. Sparking phenomena were observed at the fracture points. The tensile strength of the Zr-based bulk metallic glass becomes large; in particular, it is approximately four times the corresponding value for the crystallized specimen for all the strain rates. The tensile strength and fracture elongation of metallic glass tend to become small with an increase in the strain rate. Young's moduli were almost in the same range for all strain rates. Drastic changes in the tensile behavior of Zr-based metallic glass caused by an increase in the strain rate were not observed.
SN 1042-1122
BN 978-0-470-90917-1
PY 2010
VL 219
BP 43
EP 47
UT WOS:000287823800007
ER

PT J
AU Hosokawa, S
   Sato, H
   Happo, N
   Mimura, K
   Tezuka, Y
   Ichitsubo, T
   Matsubara, E
   Nishiyama, N
AF Hosokawa, S.
   Sato, H.
   Happo, N.
   Mimura, K.
   Tezuka, Y.
   Ichitsubo, T.
   Matsubara, E.
   Nishiyama, N.
TI Electronic structure of N42.5N7.5Cu30P20, an excellent bulk metallic
   glass former: Comparison to the Pd40Ni40P20 reference glass
SO ACTA MATERIALIA
AB In-house photoemission and inverse-photoemission spectra (PES and IPES) were measured on Pd42.5Ni7.5Cu30P20 and Pd40Ni40P20 bulk metallic glasses in order to clarify the origin of excellent glass-forming ability from the viewpoint of electronic structure. Minima are observed for both the metallic glasses at a slightly higher energy than the Fermi level. Incident photon-energy dependent PES spectra were obtained using synchrotron radiation and the Pd 4d partial density of states (DOS) was estimated from the PES data. Soft X-ray emission spectra were also measured near the Ni and Cu 2P(3/2) absorption edges to evaluate, respectively, the Ni and Cu 3d partial DOS in the valence band. The I'd 4d and the Ni and Cu 3d partials in the conduction band were obtained from X-ray absorption spectra around the Pd 3P(3/2) and Ni and Cu 2P(3/2) absorption edges, respectively. It was found that the Pd 4d partial DOS near the Fermi energy largely decreases and becomes localized by replacing the Ni atoms with the Cu atoms, which may be closely related to the excellent glass-forming ability of the Pd42.5Ni7.5Cu30P20 bulk metallic glass due to a selective formation of Pd-P covalent bonds. (C) 2007 Acta Materialia Inc. Published by Elsevier Ltd. All rights reserved.
RI Ichitsubo, Tetsu/F-3201-2010; Nishiyama, Nobuyuki/C-8228-2015
OI Ichitsubo, Tetsu/0000-0002-1127-3034; Hosokawa,
   Shinya/0000-0002-7810-6062; Mimura, Kojiro/0000-0003-1983-9095
SN 1359-6454
PD JUN
PY 2007
VL 55
IS 10
BP 3413
EP 3419
DI 10.1016/j.actamat.2007.01.041
UT WOS:000246956600011
ER

PT J
AU Wang, ML
   Chen, GL
   Hui, X
   Zhang, Y
   Bai, ZY
AF Wang, M. L.
   Chen, G. L.
   Hui, X.
   Zhang, Y.
   Bai, Z. Y.
TI Optimized interface and mechanical properties of W fiber/Zr-based bulk,
   metallic glass composites by minor Nb addition
SO INTERMETALLICS
AB W fiber/Zr-based bulk metallic glass composites were prepared by melt infiltration casting and the interface reaction in composites was studied in detail. It was found that minor Nb addition to the matrix can suppress the interface peritectic reaction and optimize the interface structure in W fiber/Zr-based bulk metallic glass composites. Taking the interface characteristics of composites and glass forming ability of the matrix into account, an optimized alloy Zr47Ti13Cu11Ni10Be16Nb3 was selected as a new metallic glass matrix. The interface in the W fiber/Zr47Ti13Cu11Ni10Be16Nb3 bulk metallic glass composite has excellent interface bonding, and the compressive strength of 70% W fiber/Zr47Ti13Cu11Ni10Be16Nb3 metallic glass composite is 2.6 GPa, 58% higher than the unreinfored matrix. Multiple shear band formation in the matrix, which results from the interaction between W fiber and the matrix, can answer for the high ultimate fraction strength and excellent plastic deformation of the composite. (c) 2007 Elsevier Ltd. All rights reserved.
RI Hui, Xidong/A-1741-2010; ZHANG, Yong/B-7928-2009
OI ZHANG, Yong/0000-0002-6355-9923
SN 0966-9795
PD OCT
PY 2007
VL 15
IS 10
BP 1309
EP 1315
DI 10.1016/j.intermet.2007.03.013
UT WOS:000249622300006
ER

PT J
AU Fu, HM
   Wang, H
   Zhang, HF
   Hu, ZQ
AF Fu, H. M.
   Wang, H.
   Zhang, H. F.
   Hu, Z. Q.
TI The effect of Gd addition on the glass-forming ability of Cu-Zr-Al alloy
SO SCRIPTA MATERIALIA
AB A new Cu-based bulk metallic glass of 10 mm diameter was successfully prepared by a conventional copper mold casting method using alloy Cu46Zr45Al7Gd2. The addition of Gd effectively alleviates the harmful effects of oxygen in the melt by the preferential formation of gadolinium oxide and significantly increases the glass-forming ability. The T-rg = T-g/T-m reaches a maximum value of 0.694 for the Cu46Zr45Al7Gd2 alloy, which is consistent with the experimental results. (c) 2006 Acta Materialia Inc. Published by Elsevier Ltd. All rights reserved.
SN 1359-6462
PD JUL
PY 2006
VL 55
IS 2
BP 147
EP 150
DI 10.1016/j.scriptamat.2006.03.052
UT WOS:000238018600009
ER

PT J
AU Liu, LF
   Zhang, HA
   Shi, C
AF Liu, L. F.
   Zhang, H. A.
   Shi, C.
TI Sliding Tribological Characteristics of a Zr-based Bulk Metallic Glass
   Near the Glass Transition Temperature
SO TRIBOLOGY LETTERS
AB Pin-on-disc apparatus was used to investigate the effect of temperature on the dry sliding friction characteristics of a Zr-based bulk metallic glass (BMG) near its glass transition temperature (T (g) = 625 K) (an analogous phenomenon in crystalline metals called the ductile-brittle transition). Unexpected variation in frictional behavior was observed in the temperature range of 603-643 K, suggesting that the glass transition and formation of protective oxide layers played roles in the tribological properties of BMG. An explanation of the behavior is given based on Scanning Electron Microscopy-Energy Dispersive Spectroscopy (SEM-EDS) analysis.
SN 1023-8883
EI 1573-2711
PD MAR
PY 2009
VL 33
IS 3
BP 205
EP 210
DI 10.1007/s11249-009-9410-1
UT WOS:000263428000007
ER

PT J
AU Chang, FY
   Cheng, CY
   Chau, SW
   Chu, JP
   Chen, YC
AF Chang, Fuh-Yu
   Cheng, Chao-Yuan
   Chau, Shiu-Wu
   Chu, Jinn P.
   Chen, Yen-Chen
TI Fabrication of Mg-based bulk metallic glass molds by thermal imprint
   process
SO INTERMETALLICS
AB Bulk metallic glasses (BMGs) have exhibited high strength, low surface energy and superior glass-forming ability. At temperatures above the glass transition temperature, BMGs become supercooled viscous materials and can be formed into complicated shapes or patterns on micro- or nano-scales. Therefore, BMGs have been considered to replicate molds for micro/nano forming applications. In this study, Mg58Cu31Y6Nd5 bulk metallic glasses were applied to study the fabrication of BMG molds from nickel master molds with micro structures by thermal imprint process. Theoretical and experimental studies were performed to understand the filling process of mold replication. A commercial computational fluid dynamics software based on the finite volume method (FVM) was used for simulating the filling behavior, where the FVM predictions reasonably well agreed with experimental measurements. With adequate operation parameters, both simulation and experimental results showed that filling rate was up to 97%. The replicated BMG molds were then employed to fabricate micro structures on PMMA substrates. It was found that the fabricated micro structures were easy to be released from the BMG molds and they gave almost the same geometrical features as that on the original master molds. Mg-based bulk metallic glasses showed good formability for replicating molds with fine structures in this study and they could be applied to micro/nano imprint applications in the future. (C) 2012 Elsevier Ltd. All rights reserved.
SN 0966-9795
PD NOV
PY 2012
VL 30
SI SI
BP 35
EP 39
DI 10.1016/j.intermet.2012.03.027
UT WOS:000308847400006
ER

PT J
AU Xie, GQ
   Louzguine-Luzgin, DV
   Song, L
   Kimura, H
   Inoue, A
AF Xie, Guoqiang
   Louzguine-Luzgin, Dmitri V.
   Song, Li
   Kimura, Hisamichi
   Inoue, Akihisa
TI Dual phase metallic glassy composites with large-size and ultra-high
   strength fabricated by spark plasma sintering
SO INTERMETALLICS
AB Two-phase bulk metallic glasses (BMGs) have attracted increasing interest since phase separation might produce metallic glasses with new physical and mechanical properties. In this study, we fabricated the dual phase glassy BMG composites by a spark plasma sintering process using a mixed powder of the gas-atomized Ni-based and Fe-based metallic glassy alloy powders. The thermal stability, microstructure, mechanical and magnetic properties of the obtained dual-phase glassy composites were investigated. The dual-phase glassy composites exhibited ultra-high strength and good soft magnetic properties which satisfy large-size requirements. (C) 2009 Elsevier Ltd. All rights reserved.
RI Inoue, Akihisa/E-5271-2015; Kimura, Hisamichi/D-5449-2012; LOUZGUINE,
   Dmitri/D-2492-2010; Xie, Guoqiang/A-8619-2011
OI LOUZGUINE, Dmitri/0000-0001-5716-4987;
SN 0966-9795
PD JUL
PY 2009
VL 17
IS 7
BP 512
EP 516
DI 10.1016/j.intermet.2009.01.003
UT WOS:000265808200006
ER

PT J
AU Laws, KJ
   Miracle, DB
   Ferry, M
AF Laws, K. J.
   Miracle, D. B.
   Ferry, M.
TI A predictive structural model for bulk metallic glasses
SO NATURE COMMUNICATIONS
AB Great progress has been made in understanding the atomic structure of metallic glasses, but there is still no clear connection between atomic structure and glass-forming ability. Here we give new insights into perhaps the most important question in the field of amorphous metals: how can glass-forming ability be predicted from atomic structure? We give a new approach to modelling metallic glass atomic structures by solving three long-standing problems: we discover a new family of structural defects that discourage glass formation; we impose efficient local packing around all atoms simultaneously; and we enforce structural self-consistency. Fewer than a dozen binary structures satisfy these constraints, but extra degrees of freedom in structures with three or more different atom sizes significantly expand the number of relatively stable, 'bulk' metallic glasses. The present work gives a new approach towards achieving the long-sought goal of a predictive capability for bulk metallic glasses.
SN 2041-1723
PD SEP
PY 2015
VL 6
AR 8123
DI 10.1038/ncomms9123
UT WOS:000362949200001
PM 26370667
ER

PT J
AU Faupel, F
   Frank, W
   Macht, MP
   Mehrer, H
   Naundorf V
   Ratzke, K
   Schober, HR
   Sharma, SK
   Teichler, H
AF Faupel, F
   Frank, W
   Macht, MP
   Mehrer, H
   Naundorf, V
   Ratzke, K
   Schober, HR
   Sharma, SK
   Teichler, H
TI Diffusion in metallic glasses and supercooled melts
SO REVIEWS OF MODERN PHYSICS
AB Amorphous metallic alloys, also called metallic glasses, are of considerable technological importance. The metastability of these systems, which gives rise to various rearrangement processes at elevated temperatures, calls for an understanding of their diffusional behavior. From the fundamental point of view, these metallic glasses are the paradigm of dense random packing. Since the recent discovery of bulk metallic glasses it has become possible to measure atomic diffusion in the supercooled liquid state and to study the dynamics of the liquid-to-glass transition in metallic systems. In the present article the authors review experimental results and computer simulations on diffusion in metallic glasses and supercooled melts. They consider in detail the experimental techniques, the temperature dependence of diffusion, effects of structural relaxation, the atom-size dependence, the pressure dependence, the isotope effect, diffusion under irradiation, and molecular-dynamics simulations. It is shown that diffusion in metallic glasses is significantly different from diffusion in crystalline metals and involves thermally activated, highly collective atomic processes. These processes appear to be closely related to low-frequency excitations. Similar thermally activated collective processes were also found to mediate diffusion in the supercooled liquid state well above the caloric glass transition temperature. This strongly supports the mode-coupling scenario of the glass transition, which predicts an arrest of liquidlike flow already at a critical temperature well above the caloric glass transition temperature.
OI Faupel, Franz/0000-0003-3367-1655; Sharma, Sanjeev
   Kumar/0000-0002-3273-0708
SN 0034-6861
PD JAN
PY 2003
VL 75
IS 1
BP 237
EP 280
DI 10.1103/RevModPhys.75.237
UT WOS:000181662300007
ER

PT J
AU Jiao, W
   Zhao, DQ
   Ding, DW
   Bai, H
   Wang, WH
AF Jiao, W.
   Zhao, D. Q.
   Ding, D. W.
   Bai, H.
   Wang, W. H.
TI Effect of free electron concentration on glass-forming ability of
   Ca-Mg-Cu system
SO JOURNAL OF NON-CRYSTALLINE SOLIDS
AB Intensive studies show that the best glass-forming ability (GFA) is presented as the Fermi surface made contact with pseudo-Brillouin boundaries. Via surveying the GFA of electronic simple Ca-Mg-Cu metallic glass system, we find that the best GFA is presented as the free electrons per atom (e/a) equals to 1.75, which is closed to the value of 1.8 proposed for the ideal amorphous state. The pseudo-gap or minimum in the density of states at the Fermi surface proposed by nearly free electron theory has not been observed in the Ca-Mg-Cu system. Our results indicate that the free electron concentration is an important but not the sole factor for influences in the glass formation. (C) 2011 Elsevier B.V. All rights reserved.
RI wei, jiao/I-7244-2013
SN 0022-3093
PD FEB 1
PY 2012
VL 358
IS 3
BP 711
EP 714
DI 10.1016/j.jnoncrysol.2011.10.033
UT WOS:000301207000038
ER

PT J
AU Li, F
   Guan, S
   Shen, B
   Makino, A
   Inoue, A
AF Li, Fushan
   Guan, Shaokang
   Shen, Baolong
   Makino, Akihiro
   Inoue, Akihisa
TI High specific strength and improved ductility of bulk
   (Mg0.65Cu0.25Gd0.1)(100-x) Ti-x metallic glass composites
SO MATERIALS TRANSACTIONS
AB The monolithic Mg65Cu25Gd10 bulk metallic glass always shows brittle fracture with a plastic strain of near zero even under a compressive load. Improved ductility and enhanced compressive strength were simultaneously achieved at room temperature in (Mg0.65Cu0.25Gd0.1)(100-x)Ti-x (x = 0, 2.5, 5 and 7.5 at%) metallic glass composites. The compressive strength (sigma(f)) reached 940MPa, and a plastic strain (epsilon(p)) of about 0.8% was undergone for the bulk metallic glass composite at x = 5. The glass composites were formed by doping Mg65Cu25Gd10 bulk metallic glass with adequate lightweight titanium with a density near that of Mg65Cu25Gd10 bulk metallic glass. The composite sustains the features of lightweight and very high specific strength which are the same as those of the Mg-based bulk metallic glass. Thus, the addition of Ti does not cause distinctly harmful influence on the glass-formation ability of the matrix glass.
RI MAKINO, AKIHIRO/B-2549-2009; Inoue, Akihisa/E-5271-2015
SN 1345-9678
EI 1347-5320
PD DEC
PY 2007
VL 48
IS 12
BP 3193
EP 3196
DI 10.2320/matertrans.MRP2007181
UT WOS:000252666300026
ER

PT J
AU Baskoutas, S
   Lemis-Petropoulos, P
   Kapaklis, V
   Koveos, Y
   Politis, C
AF Baskoutas, S
   Lemis-Petropoulos, P
   Kapaklis, V
   Koveos, Y
   Politis, C
TI Nanostructured and bulk amorphous Zr-based alloys prepared by mechanical
   alloying and arc melting
SO INTERNATIONAL JOURNAL OF MODERN PHYSICS B
AB We have produced powders of nanostructured and amorphous alloys as well as bulk amorphous alloys with composition Zr64Cu18Ni10Al8 by mechanical alloying and by quenching arc melted melts in water cooled cooper dies, respectively. The alloys were investigated by X-ray diffraction as well as by thermal analysis in order to determine the structure and thermal properties. The mechanical alloyed amorphous powders and bulk amorphous cylinders show the same thermal and X-ray characteristics. For the amorphous powders, we find that the glass transition temperature T-g is 657 K and the crystallization temperature T-x is 752 K. For bulk amorphous alloys with the same composition prepard by arc melting and liquid quenching T-g is 655 K and the T is 725 K. Moreover for the bulk amorphous alloys the supercooled liquid region DeltaT(xg) is 70 K, the reduced glass transition temperature t(g) is 0.557, the Lu-Liu parameter gamma which represents the glass forming ability for bulk metallic glasses is 0.396 and experimentally the critical cooling rate R-c takes the value 7 K s(-1).
RI Baskoutas, Sotirios/N-1325-2014; Kapaklis, Vassilios/A-6454-2013
OI Baskoutas, Sotirios/0000-0003-2782-3501; Kapaklis,
   Vassilios/0000-0002-6105-1659
SN 0217-9792
PD APR 20
PY 2003
VL 17
IS 10
BP 2035
EP 2044
DI 10.1142/S021797920301817X
UT WOS:000183599800003
ER

PT J
AU Sarlar, K
   Kucuk, I
AF Sarlar, Kagan
   Kucuk, Ilker
TI Glass forming ability and magnetic properties of
   Co(40.2-x)Fe(20.1+x)Ni6.7B22.7Si5.3Nb5 (x=0-10) bulk metallic glasses
   produced by suction casting
SO JOURNAL OF MAGNETISM AND MAGNETIC MATERIALS
AB The effect of Fe concentration on the glass forming ability (GFA) and magnetic properties in Co(40.2-x)Fe(20.1+x)Ni6.7B22.7Si5.3Nb5 (x=0-10) bulk metallic glasses were investigated. By suction casting method, the bulk metallic glasses with diameters up to 2 mm were produced. We try to find out which Fe concentration makes an influence on Co based system's magnetic properties and glass forming ability. The curves of thermal analysis, obtained using differential scanning calorimetry (DSC), show that the Co(40.2-x)Fe(20.1+x)Ni6.7B22.7Si5.3Nb5 (x=0-10) have a supercooled liquid region (Delta T-x) of about 44 K. The saturation magnetizations (J(s)) for as-cast BMG alloys were in the range of 0.62 T-0.81 T. (C) 2014 Elsevier B.V. All rights reserved.
SN 0304-8853
EI 1873-4766
PD JAN 15
PY 2015
VL 374
BP 607
EP 610
DI 10.1016/j.jmmm.2014.08.060
UT WOS:000344949000097
ER

PT J
AU Louzguine-Luzgin, DV
AF Louzguine-Luzgin, Dmitri V.
TI Early stage crystallization kinetics in metallic glass-forming alloys
SO JOURNAL OF ALLOYS AND COMPOUNDS
AB The crystallization kinetics and structural changes of a few metallic glassy alloys were monitored using X-ray diffraction, transmission electron microscopy, differential scanning and isothermal calorimetry methods. Microstructural observations were used to estimate the nucleation and growth rates. A clear comparison of the differences in the crystallization kinetics in the metallic glassy samples is observed at the early and later crystallization stages. (C) 2013 Elsevier B.V. All rights reserved.
RI LOUZGUINE, Dmitri/D-2492-2010
OI LOUZGUINE, Dmitri/0000-0001-5716-4987
SN 0925-8388
EI 1873-4669
PD FEB 15
PY 2014
VL 586
BP 216
EP 219
DI 10.1016/j.jallcom.2013.10.022
UT WOS:000329856800034
ER

PT J
AU Shin, S
   Song, MS
   Kim, TS
AF Shin, Sumin
   Song, Min-Seok
   Kim, Taek-Soo
TI Synthesis of diamond-reinforced Zr65Al10Ni10Cu15 metallic glass
   composites by pulsed current sintering
SO MATERIALS SCIENCE AND ENGINEERING A-STRUCTURAL MATERIALS PROPERTIES
   MICROSTRUCTURE AND PROCESSING
CT 5th International Conference on Physical and Numerical Simulation of
   Material Processing (ICPNS 07)
CY OCT 23-27, 2007
CL Zhengzhou, PEOPLES R CHINA
SP Chinese Mech Engn Soc, Minerals, Met & Mat Soc, Japan Inst Met, Harbin Inst Technol, Zhengzhou Univ, Henan Polytech Univ, Zhongyuan Inst Technol
AB Zr65Al10Ni10Cu15 metallic glass matrix composites (MGMC) reinforced with diamond particles were fabricated using a pulsed current sintering process. The thermal stability, microstructure and mechanical properties of the sintered bulk MGMC were investigated. The pulsed current sintered composites not only showed nearly full relative density, but also thermal stability similar to that of the initial metallic glass powders. The addition of diamond particles increased the strength which was further improved as the volume fraction increased up to similar to 10%. This corresponds to the change in fracture pattern of metallic glass after reinforcing with diamond particles. (C) 2008 Elsevier B.V. All rights reserved.
SN 0921-5093
PD JAN 15
PY 2009
VL 499
IS 1-2
SI SI
BP 525
EP 528
DI 10.1016/j.msea.2008.09.001
UT WOS:000261703300103
ER

PT J
AU Wang, Q
   Dong, C
   Qiang, JB
   Wang, YM
AF Wang, Qing
   Dong, Chuang
   Qiang, Jianbing
   Wang, Yingmin
TI Cluster line criterion and Cu-Zr-Al bulk metallic glass formation
SO MATERIALS SCIENCE AND ENGINEERING A-STRUCTURAL MATERIALS PROPERTIES
   MICROSTRUCTURE AND PROCESSING
CT 12th International Conference on Rapidly Quenched and Metastable
   Materials
CY AUG 21-26, 2005
CL Cheju Isl, SOUTH KOREA
AB The cluster line approach is presented and applied for searching the optimum glass-forming composition in the Cu-Zr-Al system. Three cluster selection rules are proposed: topologically dense packing (atomic size-related), chemical short-range order as evaluated by dissimilar atomic pairs, and composition distance to deep eutectics. Three Cu-Zr clusters Cu8Zr5, Cu6Zr5 and Cu5Zr6 satisfy all the three rules and are selected to construct cluster lines. The bulk metallic glass-forming range in the Cu-Zr-Al ternary system is thus determined. It is found that the thermal stabilities and glass-forming abilities of the bulk metallic glasses manifest monotonously increased tendencies with the increased Al content. The optimum Cu-rich and Zr-rich bulk metallic glass compositions are Cu-58.1,Zr35.9Al6 and Cu39.7Zr47.1Al13.2 (number indicate at.%) and they are, respectively, located along the Cu61.8Zr32.2Al and Cu45.7Zr54.3-Al cluster lines, where the deep eutectics exactly correspond to the dense packing clusters Cu8Zr5 and Cu5Zr6. The characteristic parameters (glass transition temperature T-g and melting temperature T) of Cu58.1Zr35.9Al6 BMG are T-g =760 K and T-g/T-l =0.648, and those of Cu39.7Zr47.1Al13.2 BMG are T-g =731 K and Tg/T-l =0.638. (c) 2006 Elsevier B.V. All rights reserved.
SN 0921-5093
PD MAR 25
PY 2007
VL 449
BP 18
EP 23
DI 10.1016/j.msea.2006.02.271
UT WOS:000245477800005
ER

PT J
AU Qiao, JC
   Cong, J
   Wang, Q
   Pelletier, JM
   Yao, Y
AF Qiao, J. C.
   Cong, J.
   Wang, Q.
   Pelletier, J. M.
   Yao, Y.
TI Effects of iron addition on the dynamic mechanical relaxation of
   Zr55Cu30Ni5Al10 bulk metallic glasses
SO JOURNAL OF ALLOYS AND COMPOUNDS
AB Dynamic mechanical relaxation behavior of (Zr55Cu30Ni5Al10)(100-x)Fe-x (x = 0, 1 and 2) bulk metallic glasses was investigated by mechanical spectroscopy. It is found that the main a relaxation of the Zr-based metallic glasses can be well described by the Kohlrausch-Williams-Watts (KWW) function. With the increasing of micro-alloying element content, i.e. iron, the value of Kohlrausch exponent beta(KWW) diminishes for the (Zr55Cu30Ni5Al10)(100-x)Fe-x (x = 0, 1 and 2) metallic glass, whereas the characteristic relaxation time increases. In the framework of the quasi-point defects theory, the physical correlation factor chi increases with higher iron content below the glass transition temperature T-g. In addition, the parameter a of the quasi-point defect theory can describe the degree of deviations from the Arrhenius law. Based on the theoretical and experimental analysis, it is implied that structural heterogeneity of the Zr55Cu30Ni5Al10 bulk metallic glass could be tailored through minor addition of the iron element. (C) 2018 Elsevier B.V. All rights reserved.
RI Yao, Yao/K-3129-2012
OI Yao, Yao/0000-0002-0879-4269
SN 0925-8388
EI 1873-4669
PD JUN 15
PY 2018
VL 749
BP 262
EP 267
DI 10.1016/j.jallcom.2018.03.285
UT WOS:000432666200030
ER

PT J
AU Liu, LF
   Zhang, HA
   Li, HQ
   Zhang, GY
AF Liu, L. F.
   Zhang, H. A.
   Li, H. Q.
   Zhang, G. Y.
TI Nanoscale morphologies on the fracture surface of bulk metallic glasses
   in the supercooled liquid region
SO SCRIPTA MATERIALIA
AB We report the observations of nanoscale corrugation, nanowave and its branching structures on the fracture surface of tough Vit. 1 bulk metallic glass (BMGs) in the supercooled liquid region. Wavelike behaviors of corrugations are observed as well. A shear band-based fracture model is proposed to interpret the formation of nanoscale morphologies in the fracture surfaces of bulk metallic glasses. The results might provide a new insight into the origin of fracture surface roughening in BMGs. Crown Copyright (c) 2009 Published by Elsevier Ltd. on behalf of Acta Materialia Inc. All rights reserved.
SN 1359-6462
PD MAY
PY 2009
VL 60
IS 9
BP 795
EP 798
DI 10.1016/j.scriptamat.2009.01.022
UT WOS:000264730000016
ER

PT J
AU Huo, JT
   Yu, HB
   Zhao, DQ
   Bai, HY
   Wang, WH
AF Huo, J. T.
   Yu, H. B.
   Zhao, D. Q.
   Bai, H. Y.
   Wang, W. H.
TI Regenerator performance below 4 K in Tm-based bulk metallic glasses
SO JOURNAL OF NON-CRYSTALLINE SOLIDS
AB The main obstacle for reaching low temperatures below 4 K using cryocoolers was the inefficiency of the regenerator materials. We report that large volumetric specific heat below 4 K in a wide temperature range has been obtained in Tm-based bulk metallic glasses, and the peak temperature of specific heat peak is tunable. We show that the amorphization of rare-earth based crystalline alloys and the lower magnetic transition temperature of spin glass behavior in the glasses result in the specific heat anomaly below 4 K. The large and tunable specific heat anomaly below 4 K indicates the potential regenerator performance of the glassy materials for sub-4 K cryocoolers. (C) 2012 Elsevier B.V. All rights reserved.
RI Yu, Hai Bin/E-5312-2010
OI Yu, Hai Bin/0000-0003-0645-0187
SN 0022-3093
EI 1873-4812
PD JUL 15
PY 2012
VL 358
IS 14
BP 1716
EP 1719
DI 10.1016/j.jnoncrysol.2012.05.008
UT WOS:000306942500012
ER

PT J
AU Perez-Bergquist, AG
   Bei, HB
   Leonard, KJ
   Zhang, YW
   Zinkle, SJ
AF Perez-Bergquist, Alejandro G.
   Bei, Hongbin
   Leonard, Keith J.
   Zhang, Yanwen
   Zinkle, Steven J.
TI Effects of ion irradiation on Zr52.5Cu17.9Ni14.6Al10Ti5 (BAM-11) bulk
   metallic glass
SO INTERMETALLICS
AB Bulk metallic glasses are intriguing candidates for nuclear applications due to their inherent amorphous structure, but their radiation response is largely unknown due to the relatively recent nature of innovations in bulk metallic glass fabrication. Here, microstructural and mechanical property evaluations have been performed on a Zr52.5Cu17.9Ni14.6Al10Ti5 bulk metallic glass (BAM-11) irradiated with 3 MeV Ni+ ions to 0.1 and 1.0 dpa at room temperature and 200 degrees C. Nanoindentation hardness and Young's modulus both decreased by 6-20% in samples irradiated at room temperature, with the sample irradiated to 1.0 dpa experiencing the greatest change in mechanical properties. However, no significant changes in properties were observed in the samples irradiated at 200 degrees C, and transmission electron microscopy showed no visible evidence of radiation damage or crystallization following ion irradiation at any of the tested conditions. These results suggest that BAM-11 bulk metallic glass may be useful for certain applications in nuclear environments. (C) 2014 Elsevier Ltd. All rights reserved.
OI Zinkle, Steven/0000-0003-2890-6915; Bei, Hongbin/0000-0003-0283-7990
SN 0966-9795
EI 1879-0216
PD OCT
PY 2014
VL 53
BP 62
EP 66
DI 10.1016/j.intermet.2014.04.016
UT WOS:000338818000009
ER

PT J
AU Wang, ZJ
   Dong, DD
   Zhang, S
   Ma, YP
   Dong, C
AF Wang, Zijian
   Dong, Dandan
   Zhang, Shuang
   Ma, Yanping
   Dong, Chuang
TI Characteristics of cluster formulas for binary bulk metallic glasses
SO JOURNAL OF ALLOYS AND COMPOUNDS
AB Compositions of typical bulk metallic glasses (BMGs) and boron-containing eutectics have been previously interpreted by cluster formulas issued from the cluster-plus-glue-atom model. In the present work, binary BMG composition formulas are established via dissociating dual-cluster formulas for their relevant eutectics. It is unveiled that BMG formulas satisfy three ubiquitous rules: cluster formula of the form [cluster](glue atom)(1 or 3) with 24 valence electrons per unit formula, the center and the glue sites occupied by the same atomic species, and the BMG composition falling within the eutectic zone. These characteristics may serve as the finger prints for good glass formers and eventually the BMG design tool. (C) 2015 Elsevier B.V. All rights reserved.
SN 0925-8388
EI 1873-4669
PD JAN 5
PY 2016
VL 654
BP 340
EP 343
DI 10.1016/j.jallcom.2015.08.244
UT WOS:000363270500048
ER

PT J
AU Pilarczyk, W
AF Pilarczyk, Wirginia
TI Preparation and characterization of Zr-based bulk metallic glasses in
   form of plate
SO JOURNAL OF ALLOYS AND COMPOUNDS
CT International Symposium on Metastable, Amorphous and Nanostructured
   Materials (ISMANAM)
CY JUN 30-JUL 05, 2013
CL Torino, ITALY
AB Zr-based bulk metallic glasses present an interesting combination of physical, chemical and mechanical properties. During the last decade, intensive progress has been made and a number of applications have been suggested for these materials. In order to successfully apply these materials, it is necessary to accurately characterize their structure, thermal stability and other properties accurately.
   The aim of the presented work is the manufacturing, examination of the structure of selected Zr-based bulk metallic alloys and confirmation of an amorphous structure using X-ray analysis, microscopic observation and thermal analysis.
   In this work, the Zr-based bulk metallic glasses in form of plate was successful produced by die pressure casting method. Designed scientific station for casting zirconium based amorphous alloys in the form of plates and rods with selected dimensions is in our university a comprehensive method for achieving amorphous materials which enables us to maintain repeatability of as-cast samples with the amorphous structure and the assumed dimensions range.
   The diffraction pattern and exothermic reaction as well as the fracture surface morphology reveal that studied as-cast Zr-based alloy is in amorphous state. The calculated GFA parameters show that the alloy exhibits satisfactory glass-forming ability in form of studied plate. These obtained values can suggest that studied alloys are suitable materials for further planned practical application at welding process. The success of Zr-based bulk metallic glasses production in form of plate with obtained sizes is important for future progress in joining research of this group of materials. (C) 2014 Elsevier B.V. All rights reserved.
SN 0925-8388
EI 1873-4669
PD DEC 5
PY 2014
VL 615
SU 1
BP S132
EP S135
DI 10.1016/j.jallcom.2014.01.037
UT WOS:000343613600028
ER

PT J
AU Wang, YM
   Wang, Q
   Zhao, JJ
   Dong, C
AF Wang, Yingmin
   Wang, Qing
   Zhao, Jijun
   Dong, Chuang
TI Ni-Ta binary bulk metallic glasses
SO SCRIPTA MATERIALIA
AB Two millimeter Ni-Ta bulk metallic glasses (BMGs) were realized over a composition range of 59-62 at.% Ni. According to the cluster-plus-glue-atom model, this composition range can be expressed by a cluster formula [MNi(6)Ta(6)]Ni(3), where M ranges from Ni to Nb. These alloys show a glass transition temperature T(g) of about 993 K, the highest among known BMG alloys. They also have a wide undercooled liquid region Delta T(x) = T(x) - T(g) exceeding 50 K. These BMGs exhibit ultrahigh strengths of about 3.5 GPa. (C) 2010 Acta Materialia Inc. Published by Elsevier Ltd. All rights reserved.
RI Zhao, Jijun/I-6030-2015
OI Zhao, Jijun/0000-0002-3263-7159
SN 1359-6462
PD JUL
PY 2010
VL 63
IS 2
BP 178
EP 180
DI 10.1016/j.scriptamat.2010.03.044
UT WOS:000278532400008
ER

PT J
AU Liu, FX
   Liaw, PK
   Wang, GY
   Chiang, CL
   Smith, DA
   Rack, PD
   Chu, JP
   Buchanan, RA
AF Liu, FX
   Liaw, PK
   Wang, GY
   Chiang, CL
   Smith, DA
   Rack, PD
   Chu, JP
   Buchanan, RA
TI Specimen-geometry effects on mechanical behavior of metallic glasses
SO INTERMETALLICS
CT 4th International Conference on Bulk Metallic Glasses
CY MAY 01-05, 2005
CL Gathinburg, TN
AB The mechanical behavior of metallic glasses under bending, compression, impact, and fatigue experiments varies, depending on the specimen geometry. The dependence of the mechanical behavior of metallic glasses on specimen geometries is reviewed and studied in this work. Different specimen geometries are found to influence the shear-band formation and deformation processes, resulting in the variations of mechanical properties. The understanding of specimen-geometry effects on the mechanical behavior of metallic glasses is critical to the improvement of mechanical properties of metallic glasses. (c) 2006 Elsevier Ltd. All rights reserved.
RI Wang, Gongyao/C-4003-2011
OI Rack, Philip/0000-0002-9964-3254
SN 0966-9795
PD AUG-SEP
PY 2006
VL 14
IS 8-9
SI SI
BP 1014
EP 1018
DI 10.1016/j.intermet.2006.01.043
UT WOS:000237770600030
ER

PT J
AU Sun, YH
AF Sun, Y. H.
TI Inverse ductile-brittle transition in metallic glasses?
SO MATERIALS SCIENCE AND TECHNOLOGY
AB There is evidence that metallic glasses can show increased plasticity as the temperature is lowered. This behaviour is the opposite to what would be expected from phenomena such as the ductile-brittle transition in conventional alloys. Data collected for the plasticity of different metallic-glass compositions tested at room temperature and below, and at strain rates from rate 10(-5) to 10(-3) s(-1), are reviewed. The analogous effects of low temperature and high strain rate, as observed in conventional alloys, are examined for metallic glasses. The relevant plastic flow in metallic glasses is inhomogeneous, sharply localised in thin shear bands. The enhanced plasticity at lower temperature is attributed principally to a transition from shear on a single dominant band to shear on multiple bands. The origins of this transition and its links to shear bands operating ` hot' or ` cold' are explored. The stress drop on a shear band after initial yielding is found to be a useful parameter for analysing mechanical behaviour. Schematic failure mode maps are proposed for metallic glasses under compression and tension. Outstanding issues are identified, and design rules are considered for metallic glasses of improved plasticity.
SN 0267-0836
EI 1743-2847
PD APR
PY 2015
VL 31
IS 6
BP 635
EP 650
DI 10.1179/1743284714Y.0000000684
UT WOS:000348343000002
ER

PT J
AU Zhao, YY
   Ma, E
   Xu, J
AF Zhao, Yuan-Yun
   Ma, Evan
   Xu, Jian
TI Reliability of compressive fracture strength of Mg-Zn-Ca bulk metallic
   glasses: Flaw sensitivity and Weibull statistics
SO SCRIPTA MATERIALIA
AB Mg96-xZnxCa4 (x = 30, 25) bulk metallic glasses (BMGs) belong to one of the brittle families of BMGs, yet their compressive fracture strength exhibit surprisingly high uniformity. This strength reliability is also supported by the Weibull modulus value derived from an analysis based on Weibull statistics. The flaw sensitivity and its obvious composition dependence are discussed. (C) 2007 Acta Materialia Inc. Published by Elsevier Ltd. All rights reserved.
RI Ma, En/A-3232-2010
SN 1359-6462
PD MAR
PY 2008
VL 58
IS 6
BP 496
EP 499
DI 10.1016/j.scriptamat.2007.10.052
UT WOS:000253649800019
ER

PT J
AU Wen, P
   Wang, WH
   Zhao, YH
   Zhao, DQ
   Pan, MX
   Li, FY
   Jin, CQ
AF Wen, P
   Wang, WH
   Zhao, YH
   Zhao, DQ
   Pan, MX
   Li, FY
   Jin, CQ
TI Glass transition in Zr46.75Ti8.25Cu7.5Ni10Be27.5 metallic glass under
   high pressure
SO PHYSICAL REVIEW B
AB An approach is developed using differential scanning calorimetry with the characteristics of relaxation to exhibit a glass transition under high pressure in metallic glass, specifically, the glass transition in Zr46.75Ti8.25Cu7.5Ni10Be27.5 bulk glass-forming alloy with a very stable supercooled liquid state. Through the approach, we obtain an averaged increase of the glass transition temperature with a pressure of 5.6 K/GPa. Based on free volume theory, the formation volume (DeltaV(f)) of 6.5+/-0.5 Angstrom(3) and migration volume (DeltaV(m)) of 6.5+/-0.5 Angstrom(3) for defect diffusion, which is related to pressure-dependent free volume change, are derived. The results contribute to the understanding of the nature of the calorimetric glass transition and diffusion in metallic glasses.
SN 2469-9950
EI 2469-9969
PD MAR
PY 2004
VL 69
IS 9
AR 092201
DI 10.1103/PhysRevB.69.092201
UT WOS:000220812800008
ER

PT J
AU Fecht, HJ
AF Fecht, HJ
TI Thermodynamic and mechanical properties of Zr-based bulk metallic
   glasses
SO PHILOSOPHICAL MAGAZINE B-PHYSICS OF CONDENSED MATTER STATISTICAL
   MECHANICS ELECTRONIC OPTICAL AND MAGNETIC PROPERTIES
CT International Workshop on Metastable Phases: Amorphous and
   Nanocrystalline Materials, Theoretical Aspects, Industrial and
   Technological Transfer
CY APR 09-11, 1996
CL BOLOGNA, ITALY
SP Univ Bologna, Italian Natl Inst Phys Matter, Inst Adv Mat, European Commiss, Joint Res Ctr, ASM Int, RoloBanca 1473 SpA
AB Glasses are generally produced from the highly undercooled liquid state by rapid quenching methods or quasistatically at slow cooling by the effective control of potent heterogeneous nucleation sites. For metallic systems the latter method recently has led to the development of bulk metallic glasses, so-called supermetals, with a complex multicomponent chemistry and advanced engineering properties. As long as crystallization can be prevented, the relevant thermodynamic properties of the metastable glassy and undercooled liquid phases can be measured below and above the glass transition temperature respectively. The data suggest that the glass transition corresponds to kinetic freezing triggered by an underlying entropic instability. Further measurements of the mechanical and wear properties of these new metastable materials point to unique engineering properties for technological applications.
SN 0141-8637
PD OCT
PY 1997
VL 76
IS 4
BP 495
EP 503
DI 10.1080/01418639708241115
UT WOS:A1997XY66500013
ER

PT J
AU Louzguine-Luzgin, DV
   Li, S
   Xie, GQ
   Inoue, A
   Yoshikawa, N
   Sato, M
AF Louzguine-Luzgin, D. V.
   Li, S.
   Xie, G. Q.
   Inoue, A.
   Yoshikawa, N.
   Sato, M.
TI MICROWAVE RADIATION PROCESSING OF METALLIC GLASSY POWDERS
SO JOURNAL OF MICROWAVE POWER AND ELECTROMAGNETIC ENERGY
AB In the present study we investigate the stability of Cu50Zr45Al5 and Ni59Ti16Zr20Sn5 glassy powders and the formation of the bulk metallic glassy samples by microwave heating in a single mode cavity (915 MHz) in the alternating magnetic field maximum. Metallic glasses exhibit low viscosity at temperatures close to the glass-transition which allows for the processing of glassy powders. Microwave heating being a volumetric heating has significant advantages over conventional heating in materials processing, such as substantial energy savings, high heating rates and process cleanness.
RI Xie, Guoqiang/A-8619-2011
SN 0832-7823
PY 2009
VL 43
IS 1
UT WOS:000209528200010
ER

PT J
AU Das, J
   Pauly, S
   Bostrom, M
   Durst, K
   Goken, M
   Eckert, J
AF Das, J.
   Pauly, S.
   Bostroem, M.
   Durst, K.
   Goeken, M.
   Eckert, J.
TI Designing bulk metallic glass and glass matrix composites in martensitic
   alloys
SO JOURNAL OF ALLOYS AND COMPOUNDS
CT 14th International Sympoium on Metastable and Nano-Materials
CY AUG 26-30, 2007
CL Corfu, GREECE
AB To circumvent the limited plasticity of bulk metallic glasses (BMGs), heterogeneous materials with glassy matrix and different type and length-scale of heterogeneities (micrometer-sized second phase particles or fibers, nanocrystals in a glassy matrix, phase separated regions, variations in short-range order by clustering) have been reported. We developed bulk metallic glasses and glass matrix composites in martensitic Zr-Cu-base alloys. Large plasticity can be obtained from microstructure consisting of either a glassy structure, or for alloys with martensitic second phase embedded in a glassy matrix. This type of glasses and glass-matrix composites are able to achieve high strength together with pronounced work-hardening. We explore the possibilities to synthesize such in situ composite microstructures based on shape memory alloys ("M-Glasses") through metal mold casting. (C) 2008 Elsevier B.V. All rights reserved.
RI Goken, Mathias/F-7940-2015; Durst, Karsten/D-1262-2011; Das,
   Jayanta/G-1559-2010
OI Goken, Mathias/0000-0002-3226-0304; Durst, Karsten/0000-0002-9246-6398;
   Das, Jayanta/0000-0001-8750-5463
SN 0925-8388
PD AUG 26
PY 2009
VL 483
IS 1-2
BP 97
EP 101
DI 10.1016/j.jallcom.2008.08.139
UT WOS:000270619600025
ER

PT J
AU Ogata, S
   Shimizu, F
   Li, J
   Wakeda, M
   Shibutani, Y
AF Ogata, Shigenobu
   Shimizu, Futoshi
   Li, Ju
   Wakeda, Masato
   Shibutani, Yoji
TI Atomistic simulation of shear localization in Cu-Zr bulk metallic glass
SO INTERMETALLICS
CT 4th International Conference on Bulk Metallic Glasses
CY MAY 01-05, 2005
CL Gatlinburg, TN
AB Shear deformations of Cu57Zr43 bulk metallic glass (BMG) model systems are performed using molecular dynamics simulation. The results suggest that both the hydrostatic stress and the stress normal to the shear plane should affect the shear response (modified Mohr-Coulomb yield criterion). We see shear localization and shear band nucleation in both a small system of 2000 atoms, and large systems of 524,288 atoms, and analyze local atomic structure evolution. (c) 2006 Elsevier Ltd. All rights reserved.
RI Li, Ju/A-2993-2008; OGATA, Shigenobu/G-5497-2011
OI Li, Ju/0000-0002-7841-8058; OGATA, Shigenobu/0000-0002-9072-4496;
   Wakeda, Masato/0000-0002-6377-1318
SN 0966-9795
EI 1879-0216
PD AUG-SEP
PY 2006
VL 14
IS 8-9
SI SI
BP 1033
EP 1037
DI 10.1016/j.intermet.2006.01.022
UT WOS:000237770600033
ER

PT J
AU Li, HX
   Wang, SL
   Yi, S
   Jiao, ZB
   Wu, Y
   Lu, ZP
AF Li, H. X.
   Wang, S. L.
   Yi, S.
   Jiao, Z. B.
   Wu, Y.
   Lu, Z. P.
TI Glass formation and magnetic properties of Fe-C-Si-B-P-(Cr-Al-Co) bulk
   metallic glasses fabricated using industrial raw materials
SO JOURNAL OF MAGNETISM AND MAGNETIC MATERIALS
AB Formation of Fe-C-Si-B-P-(Cr-Al-Co) bulk metallic glasses with enhanced glass-forming ability (GFA) and magnetic properties fabricated using industrial pig-iron and Fe-P alloys as raw materials has been studied. It was found that GFA of the pig-iron (Fe79.5C18.0Si2.3P0.2, at%) could be improved by proper additions of only metalloids C, Si, P and B, and fully glassy structure was obtained in the Fe75.5C7.0Si3.3B5.5P8.7 alloy with a critical diameter of 1 mm. With suitable additions of metallic elements Cr, Co and Al, the GFA is further increased and fully amorphous rods in 4 mm diameter can be prepared for the Fe68.2C7.0Si3.3B5.5P8.7Cr2.3Al2.0Co3.0 alloy that shows a unique combination of good soft-magnetic properties (M-s 1.03T, H-c 9.39 A/m), high GFA and high fracture strength. These low-cost Fe-based bulk metallic glasses fabricated using industrial raw materials have a potential to be utilized as engineering materials. (C) 2009 Elsevier B.V. All rights reserved.
RI Lu, Zhao-Ping/A-2718-2009; wu, yuan/E-8927-2010; Wu, Yuan/C-4025-2015
OI Lu, Zhao-Ping/0000-0003-1463-8948; Wu, Yuan/0000-0001-7857-0247; Jiao,
   Z. B./0000-0002-0556-6869
SN 0304-8853
EI 1873-4766
PD SEP
PY 2009
VL 321
IS 18
BP 2833
EP 2837
DI 10.1016/j.jmmm.2009.04.021
UT WOS:000266823200028
ER

PT J
AU Qin, CL
   Wang, LJ
   Wang, ZF
   Ding, J
   Zhao, WM
   Inoue, A
AF Qin Chunling
   Wang Lijuan
   Wang Zhifeng
   Ding Jian
   Zhao Weimin
   Inoue, Akihisa
TI Synthesis, properties and XPS analysis of Cu-, Ni-, Ti-based metallic
   glasses
SO CHINA FOUNDRY
AB Metallic glasses (MG) represent an interesting group of materials as they possess outstanding physical, chemical and mechanical properties compared to their crystalline counterparts. This paper reviews the synergistic influence of Ni and Nb elements on thermal stability of supercooled liquid and corrosion resistance of as-cast Cu-Zr(Hf)-Ti-Ni-Nb bulk metallic glasses (BMGs). Additionally, in-situ second phase reinforced Cu-based BMG composites with high corrosion resistance and excellent mechanical properties are investigated. On the other hand, this paper reports the development of ultra-high corrosion resistant Ni-based metallic glasses at high temperatures for their potential applications. Corrosion resistance and XPS analysis of the Ni-free Ti-based BMG are also introduced.
RI Wang, Zhifeng/A-8557-2010; Inoue, Akihisa/E-5271-2015
OI Wang, Zhifeng/0000-0003-3880-1957;
SN 1672-6421
PD JUL
PY 2014
VL 11
IS 4
BP 247
EP 257
UT WOS:000341768500004
ER

PT J
AU Park, ES
   Kim, WT
   Kim, DH
AF Park, ES
   Kim, WT
   Kim, DH
TI Bulk glass formation in Mg-Cu-Ag-Y-Gd alloy
SO MATERIALS TRANSACTIONS
AB The effect of partial substitution of Y for Gd on the glass forming ability of Mg65Cu15Ag10Y10 alloy has been studied using X-ray diffraction and differential thermal calorimetry. Partial substitution of Y by Gd improves the glass forming ability. The Mg65Cu15Ag10Y2Gd8 alloy shows the highest glass forming ability among the alloys investigated, enabling the fabrication of bulk metallic glass with the diameter of 9 mm by conventional Cu-mold casting method in air atmosphere. The compressive fracture strength and fracture elongation of the Mg65Cu15Ag10Y2Gd8 bulk metallic glass are 956 MPa and 1.6% respectively.
RI bang, changwook/J-7922-2012; Park, Eun Soo/A-9860-2014; Park, Eun
   Soo/A-4443-2008
SN 1345-9678
EI 1347-5320
PD JUL
PY 2004
VL 45
IS 7
BP 2474
EP 2477
DI 10.2320/matertrans.45.2474
UT WOS:000223245300087
ER

PT S
AU Zinkle, SJ
AF Zinkle, S. J.
BE Yvon, P
TI Advanced irradiation-resistant materials for Generation IV nuclear
   reactors
SO STRUCTURAL MATERIALS FOR GENERATION IV NUCLEAR REACTORS
SE Woodhead Publishing Series in Energy
SN 2044-9364
BN 978-0-08-100912-3; 978-0-08-100906-2
PY 2017
IS 106
BP 569
EP 594
DI 10.1016/B978-0-08-100906-2.00016-1
UT WOS:000402698000017
ER

PT S
AU Eckert, J
   Seidel, M
   Schultz, L
AF Eckert, J
   Seidel, M
   Schultz, L
BE Schulz, R
TI Bulk metallic glasses with significant supercooled liquid region
   prepared by mechanical alloying
SO METASTABLE, MECHANICALLY ALLOYED AND NANOCRYSTALLINE MATERIALS, PTS 1
   AND 2
SE MATERIALS SCIENCE FORUM
CT International Symposium on Metastable, Mechanically Alloyed and
   Nanocrystalline Materials (ISMANAM-95)
CY JUL 24-28, 1995
CL QUEBEC CITY, CANADA
SP Hydro Quebec, Laval Univ, McGill Univ, Ctr Phys Mat, Nat Sci & Engn Res Council Canada, Zoz GmbH, Siemens, Xerox Canada Ltee, Precitech
AB Zr-, Mg- and Al-based alloys have been prepared by mechanical alloying of elemental powders. Mechanical alloying produces Zr- and Mg-based metallic glasses with extended supercooled liquid region for several compositions. The Al-based alloys consist of mixtures of amorphous and nanocrystalline phases without significant supercooled liquid region. The phase formation and the thermal stability of the powders are compared with data for melt-spun and water quenched materials. First results on consolidated bulk samples are presented, demonstrating the potential of mechanical alloying as an alternative processing route for the preparation of bulk amorphous alloys.
RI Schultz, Ludwig/B-3383-2010
SN 0255-5476
BN 0-87849-738-2
PY 1996
VL 225
BP 113
EP 118
DI 10.4028/www.scientific.net/MSF.225-227.113
UT WOS:A1996BG37Y00016
ER

PT J
AU Lee, MH
   Lee, JY
   Bae, DH
   Kim, WT
   Sordelet, DJ
   Kim, DH
AF Lee, MH
   Lee, JY
   Bae, DH
   Kim, WT
   Sordelet, DJ
   Kim, DH
TI A development of Ni-based alloys with enhanced plasticity
SO INTERMETALLICS
CT 3rd International Conference on Bulk Metallic Glasses
CY OCT 12-16, 2003
CL Beijing, PEOPLES R CHINA
AB Enhancement of plasticity in Ni-based injection cast bulk metallic glasses (BMGs) and metallic glass matrix composites (MGMCs) has been investigated. The injection-cast Ni59Zr16Ti13Si3Sn2Nb7 BMG showed a significantly improved plastic region to failure of 6.5%, as compared to that of the injection-cast Ni59Zr20Ti16Si2Sn3 BMG of 2%. The Ni59Zr20Ti16Si2Sn3 MGMC containing brass-fibers shows macroscopic plasticity after yielding, and the level of plastic strain increased to around 2% with increasing brass content up to 40 vol.%, while monolithic warm-extruded Ni59Zr20Ti16Si2Sn3 BMG show no plastic deformation region. Present study shows that the plasticity of BMG can be improved by selecting proper BMG alloy composition or by introducing ductile reinforcing phase in the BMG matrix. (C) 2004 Elsevier Ltd. All rights reserved.
RI bang, changwook/J-7922-2012; Juhyun, Oh/H-9185-2012
OI LEE, MIN HA/0000-0001-6006-0628
SN 0966-9795
PD OCT-NOV
PY 2004
VL 12
IS 10-11
BP 1133
EP 1137
DI 10.1016/j.intermet.2004.04.027
UT WOS:000224566700017
ER

PT J
AU Ke, HB
   Wen, P
   Peng, HL
   Wang, WH
   Greer, AL
AF Ke, H. B.
   Wen, P.
   Peng, H. L.
   Wang, W. H.
   Greer, A. L.
TI Homogeneous deformation of metallic glass at room temperature reveals
   large dilatation
SO SCRIPTA MATERIALIA
AB Room-temperature uniaxial compression of Zr(46.75)Ti(8.25)Cu(7.5)Ni(10)Be(27.5) bulk metallic glass at 80% of the yield stress leads to homogeneous viscous flow. The flow in this apparently elastic regime reveals large increases in volume associated with local shearing events in the glass. The extent of this dilatation is masked in more usual tests by significant relaxation of the glass structure during and after shear. The dilatation in deformed metallic glasses can no longer be attributed only to free-volume generation at shear bands. (C) 2011 Acta Materialia Inc. Published by Elsevier Ltd. All rights reserved.
RI Greer, Lindsay/E-9433-2017; Peng, Hailong/E-5568-2018; Greer, Alan
   Lindsay/G-1977-2011
OI Peng, Hailong/0000-0001-6147-6342;
SN 1359-6462
PD MAY
PY 2011
VL 64
IS 10
BP 966
EP 969
DI 10.1016/j.scriptamat.2011.01.047
UT WOS:000289330700011
ER

PT J
AU Rouxel, T
AF Rouxel, Tanguy
TI Fracture surface energy and toughness of inorganic glasses
SO SCRIPTA MATERIALIA
AB A simple model is proposed to predict the fracture surface energy (gamma) and the fracture toughness (K-IC) of glasses from their nominal compositions. The theoretical values are compared with the experimental ones, as obtained by means of self-consistent methods such as the Single Edge Pre-crack Beam (SEPB) and Chevron Notch (CN) ones, when available. A remarkable agreement is observed for ionocovalent glasses. This corroborates previous investigations showing that crack tip is nearly atomically sharp. In the case of metallic glasses, the theoretical values are much smaller than the experimental ones, which support the occurrence of crack tip plasticity and shielding effect. (C) 2017 Acta Materialia Inc. Published by Elsevier Ltd. All rights reserved.
SN 1359-6462
PD AUG
PY 2017
VL 137
BP 109
EP 113
DI 10.1016/j.scriptamat.2017.05.005
UT WOS:000403993300024
ER

PT J
AU Fan, C
   Ott, RT
   Hufnagel, TC
AF Fan, C
   Ott, RT
   Hufnagel, TC
TI Metallic glass matrix composite with precipitated ductile reinforcement
SO APPLIED PHYSICS LETTERS
AB We report a composite material consisting of precipitated micron-scale Ta-rich solid solution particles distributed in a bulk metallic glass matrix. The reinforcing ductile particles are precipitated during melting of the master alloy of glass-forming (Zr70Ni10Cu20)(82)Ta8Al10, by using previously prepared metastable Zr-Ta solid solution binary ingots. Upon cooling from the melt, the matrix undergoes a glass transition to produce an amorphous phase while the particles of precipitated Ta solid solution are distributed in the glass matrix. The resulting material not only shows high strength (similar to2.1 GPa), but also has dramatically enhanced plastic strain to failure in uniaxial compression relative to single-phase bulk metallic glasses. The composite also displays limited tensile ductility. (C) 2002 American Institute of Physics.
RI Hufnagel, Todd/A-3309-2010
OI Hufnagel, Todd/0000-0002-6373-9377; Ott, Ryan/0000-0002-7159-2328
SN 0003-6951
EI 1077-3118
PD AUG 5
PY 2002
VL 81
IS 6
BP 1020
EP 1022
DI 10.1063/1.1498864
UT WOS:000177171400024
ER

PT J
AU Park, ES
   Kim, WT
   Kim, DH
AF Park, ES
   Kim, WT
   Kim, DH
TI The effect of in addition on the glass-forming ability in Cu-Ti-Zr-Ni-Si
   metallic glasses
SO MATERIALS TRANSACTIONS
AB The effect of In substitution for Ni on the glass forming ability has been studied in Cu47Ti33Zr11Ni8-xInxSi1 (x = 0, 2, 4, 6, 8) alloys by using thermal analysis and X-ray diffractometry. Partial substitution of Ni by In in Cu47Ti33Zr11Ni8Si1 promotes the glass forming ability. Cu47Ti33Zr11Ni6In2Si1 bulk metallic glass with diameter of 6 mm can successfully be fabricated by Cu-mold injection casting method. DeltaT(x) and K parameter show a good relationship with the measured maximum diameter of the amorphous specimen.
RI Park, Eun Soo/A-4443-2008; Park, Eun Soo/A-9860-2014; bang,
   changwook/J-7922-2012
SN 1345-9678
EI 1347-5320
PD AUG
PY 2004
VL 45
IS 8
BP 2693
EP 2696
DI 10.2320/matertrans.45.2693
UT WOS:000223707600046
ER

PT J
AU Wang, YC
   Chen, N
   Yao, KF
AF Wang Yao-Cen
   Chen Na
   Yao, Ke-Fu
TI Formation of Pd-Ni based bulk metallic glasses
SO CHINESE PHYSICS LETTERS
AB Bulk metallic glasses are prepared in Pd(40.5)Ni(40.5)Si(x)P(19-x) (x = 0-14 at.%) alloys by a combination of flux treatment and water quenching technique. It is found that the thermal stability of the Pd(40.5).Ni(40.5)Si(x)P(19-x) glassy alloys depends on the addition of Si content. Among the Pd(40.5)Ni(40.5)Si(x)P(19-x) glassy alloys studied, the Pd(40.5)Ni(40.5)Si(5)P(15) bulk metallic glass exhibits the largest supercooled liquid region (Delta T = 119 K) and the highest activation energy of crystallization (283.3 kJ/mol), showing enhanced glass formation ability and extraordinary glassy thermal stability.
RI Chen, Na/A-4120-2010; Wang, Yaocen/A-8186-2015
OI Wang, Yaocen/0000-0002-9773-737X
SN 0256-307X
PD JUN
PY 2007
VL 24
IS 6
BP 1653
EP 1655
UT WOS:000247045400061
ER

PT J
AU Guo, H
AF Guo, He
TI Ti-based alloys of bulk metallic glasses and nanostructure
SO RARE METAL MATERIALS AND ENGINEERING
AB Ti-based bulk metallic glasses (BMGs) developed in the past decade have been reviewed in terms of their compositions, glass-forming abilities and mechanical properties. The cast defects in the BMG samples, which significantly deteriorate the mechanical properties, are discussed and summarized based on the experimental results in the literature. Some results on the cast nanostructured Ti alloys with dendritic ductile beta phase are also reviewed. A special composite microstructure, dendritic beta-Ti(M) (in,micrometer size) + rod-like eutectic beta-Ti/gamma-TiCu (in nanometer size), has been obtained in the as-cast cylinders smaller than 5 mm in diameter in the Ti-Cu-Ni-Sn-Ta(Nb) systems. The Ti alloys with such composite microstructure exhibit very high strength and remarkable plasticity.
SN 1002-185X
PD MAY
PY 2007
VL 36
IS 5
BP 753
EP 758
UT WOS:000247453000001
ER

PT S
AU Louzguine, DV
AF Louzguine (Luzgin), D. V.
BA Louzguine, DV
BF Louzguine, DV
TI Structure
SO METALLIC GLASSES AND THEIR COMPOSITES
SE Materials Research Foundations
AB Crystals are characterized by a unit cell and existence of a translational symmetry. Quasicrystals do not have such a cell in three-dimensional space but exhibit a rotational symmetry. The structure of liquids and glasses is less well understood. As the atomic structure of bulk metallic glasses defines their properties, in particular mechanical, research on the structure of glassy alloys is an important topic of modern physical metallurgy.
SN 2471-8890
EI 2471-8904
BN 978-1-945291-42-5
PY 2018
VL 19
BP 68
EP 107
D2 10.21741/9781945291432
UT WOS:000417007800003
ER

PT J
AU Chen, JX
   Wang, Q
   Dong, C
AF Chen Jixiang
   Wang Qing
   Dong Chuang
TI Cluster Rule in Alloy Phase and Its Application in Zr-Al-Ni System
SO RARE METAL MATERIALS AND ENGINEERING
AB The cluster rules of alloy phase structure of some typical bulk metallic glass systems were studied. The fact that the clusters share atoms each other in different ways in the alloy phase was pointed out. After sharing there are two kinds of cluster linkages: the first is the phase composition which does not need glue atoms; the other is that glue atoms are required to form phase structure, i.e. termed cluster phases. The method provides a new idea for the structure of alloy phase. In the typical bulk metallic glass system Zr-Al-Ni, there are three cluster phases, AlNiZr-Fe2P, Al2NiZr6-InMg2 and Al5Ni3Zr2-Mn23Th6. The method to determine the clusters related with metallic glass formation based on alloy phase structure was also put forward, i.e. the clusters related with the metallic glasses are generally the isolated clusters from cluster phases, which is verified very well in Zr-Al-Ni system.
SN 1002-185X
PD JAN
PY 2011
VL 40
IS 1
BP 69
EP 73
UT WOS:000291966300009
ER

PT J
AU Yin, JM
   Cai, HN
   Cheng, XW
   Zhang, XQ
AF Yin, Jiaming
   Cai, Hongnian
   Cheng, Xingwang
   Zhang, Xinqiang
TI Al-based bulk metallic glass with large plasticity and ultrahigh
   strength
SO JOURNAL OF ALLOYS AND COMPOUNDS
AB In this study, we reported an Al86Ni7Er5Co1La1 bulk metallic glass, which exhibits ultrahigh strength of up to similar to 1525 MPa together with apparent work hardening and large ductile of similar to 11%. The high plasticity is attributed to the multiple shear bands, which restrict the catastrophic failure of the bulk metallic glass, and finally increase the flow stress of the material resulting in a work hardening behavior. (C) 2015 Elsevier B.V. All rights reserved.
SN 0925-8388
EI 1873-4669
PD NOV 5
PY 2015
VL 648
BP 276
EP 279
DI 10.1016/j.jallcom.2015.05.285
UT WOS:000361158300044
ER

PT J
AU Wang, Q
   Pelletier, JM
   Xia, L
   Xu, H
   Dong, YD
AF Wang, Q
   Pelletier, JM
   Xia, L
   Xu, H
   Dong, YD
TI The viscoelastic properties of bulk Zr55Cu25Ni5Al10Nb5 metallic glass
SO JOURNAL OF ALLOYS AND COMPOUNDS
AB In this work, we have investigated the dynamic shear modulus of the bulk Zr55Cu25Ni5Al10Nb5 metallic glasses over large temperature and frequency ranges. Like in other conventional glass forming systems. a distinct alpha relaxation with typical kinetic features is experimentally detected. Therefore, the fragility parameter, m, which is estimated from the activation energy for viscoelastic deformation, indicates that the Zr base bulk amorphous alloy is a relatively strong liquid. On the other hand. during the main mechanical relaxation, the frequency dependence of dynamic shear modulus of the Zr base bulk amorphous alloy follows the biparabolic equation derived from a physical model, which can characterize the atomic mobility and mechanical response of disordered condensed materials. (c) 2005 Elsevier B.V. All rights reserved.
OI Xia, Lei/0000-0001-9198-1497
SN 0925-8388
PD MAR 9
PY 2006
VL 413
IS 1-2
BP 181
EP 187
DI 10.1016/j.jallcom.2005.02.109
UT WOS:000236312900029
ER

PT J
AU Shi, B
   Xu, YL
   Ma, WL
   Li, C
   Estefania, CV
   Li, JG
AF Shi, Bo
   Xu, Yuanli
   Ma, Wenli
   Li, Chao
   Estefania, Carmen Verge
   Li, Jiangong
TI Effect of Ti addition on the glass forming ability, crystallization, and
   plasticity of (Zr64.13Cu15.75Ni10.12Al10)(100-x)Ti-x bulk metallic
   glasses
SO MATERIALS SCIENCE AND ENGINEERING A-STRUCTURAL MATERIALS PROPERTIES
   MICROSTRUCTURE AND PROCESSING
AB A series of (Zr(64.13)Cu(15.7)sNi(10.12)Al(10))(100-x)Ti-x (x=0-7) bulk metallic glasses were prepared by water-cooled copper mold casting method. The glass forming ability, crystallization behavior, and plasticity of the (Zr64.13Cu15.75Ni10.12Al10)(100-x)Ti-x alloys with different Ti contents were studied. The reduced glass transition temperature increases with Ti addition and a maximum value of 0.562 is achieved at x=5, indicating that the glass forming ability increases with Ti addition. Compared with that of one crystallization step for the sample without Ti addition, the Ti (x >= 2) containing alloys exhibit a two-step crystallization behavior. In addition, the initial crystallization temperature decreases from 744 to 684 K as x increases from 0 to 7. Icosahedral quasicrystal phase was found to precipitate in the amorphous matrix in the first crystallization step, implying that strong icosahedral short-range order may exist in the Ti (x >= 2) containing alloys. moreover, the (Zr64.13Cu15.75Ni10.12Al10)(100-x)Ti-x bulk metallic glasses exhibit excellent plasticity, i.e., a plastic engineering strain in compression larger than 80% for x <= 6. (C) 2015 Elsevier B.V. All rights reserved.
SN 0921-5093
EI 1873-4936
PD JUL 15
PY 2015
VL 639
BP 345
EP 349
DI 10.1016/j.msea.2015.04.100
UT WOS:000357549200045
ER

PT J
AU Li, HX
   Yi, SH
   Sohn, HS
AF Li, Hongxiang
   Yi, Seonghoon
   Sohn, Ho Sang
TI Fe-based bulk metallic glasses Fe73.8-xC7.0Si3.5BxP9.6Cr2.1Mo2Al2O (x=3
   similar to 9) prepared using hot metal and industrial raw materials
SO JOURNAL OF MATERIALS RESEARCH
AB For extensive applications of bulk metallic glasses as structural materials, Fe-based bulk metallic glasses Fe73.8-xC7.0Si3.5BxP9.6Cr2.1Mo2.0Al2.0 (at.%, x = 3, 5, 7, 9) have been developed using hot metal, commercial grade pure elements, and industrial ferro-alloys. The alloy Fe68.8C7.0Si3.5B5P9.6Cr2.1Mo2.0Al2.0 can be cast into a fully amorphous rod of 5 mm in diameter through a suction casting method. The glass forming ability of the alloys can be successfully assessed by the difference of Gibbs free energy between liquid and solid phases obtained through thermodynamic calculations.
SN 0884-2914
PD JAN
PY 2007
VL 22
IS 1
BP 164
EP 168
DI 10.1557/JMR.2007.0008
UT WOS:000243463400020
ER

PT J
AU Kumar, S
   Kim, J
   Kim, H
   Lee, C
AF Kumar, S.
   Kim, Junseoub
   Kim, Hwijun
   Lee, Changhee
TI Phase dependence of Fe-based bulk metallic glasses on properties of
   thermal spray coatings
SO JOURNAL OF ALLOYS AND COMPOUNDS
AB Thermal spray coatings of two different Fe-based bulk metallic glasses (BMGs) were prepared using atmospheric plasma spray (APS) technique. Effects of crystallinity and chemical phase composition of the initial feedstock materials and the impact of plasma jet on the properties of the Fe-based metallic glass powders were discussed by characterizing the coating properties. Also, partial and complete melting of powders in plasma and the effects on oxidation and crystallization are discussed. Powder which contains elements such as Al and Si, showed better wear property because of oxidation effect and any deviation from the optimum quantity of elements would affect coating properties. (C) 2008 Elsevier B.V. All rights reserved.
SN 0925-8388
EI 1873-4669
PD MAY 5
PY 2009
VL 475
IS 1-2
BP L9
EP L12
DI 10.1016/j.jallcom.2008.07.064
UT WOS:000265911700003
ER

PT J
AU Afonin, GV
   Khonik, SV
   Kaloyan, AA
   Khonik, VA
AF Afonin, G. V.
   Khonik, S. V.
   Kaloyan, A. A.
   Khonik, V. A.
TI Internal stresses of deformation origin in bulk metallic glasses of the
   Pd-Cu-Ni-P system
SO PHYSICS OF THE SOLID STATE
AB The effective internal shear stresses arising during unloading of bulk Pd-Cu-Ni-P metallic glasses deformed under conditions of homogeneous flow below the glass transition temperature T (g) have been determined using measurements of the torque relaxation. It has been shown that the level of effective internal stresses gradually decreases with increasing temperature and approaches zero at T -> T (g) . Possible mechanisms of the formation of internal stresses with variations in the experimental conditions have been discussed. DOI: 10.1134/S1063783412110029
RI Afonin, Gennady/K-1014-2013; Khonik, Vitaly/A-5888-2009
OI Afonin, Gennady/0000-0002-7715-5065;
SN 1063-7834
PD NOV
PY 2012
VL 54
IS 11
BP 2150
EP 2154
DI 10.1134/S1063783412110029
UT WOS:000310748800002
ER

PT J
AU Zhang, XQ
   Wang, W
   Ma, E
   Xu, J
AF Zhang, XQ
   Wang, W
   Ma, E
   Xu, J
TI Retractory Mo-Si-based glassy alloy designed for ultrahigh strength and
   thermal stability
SO JOURNAL OF MATERIALS RESEARCH
AB Mechanically alloyed Mo44Si26Ta5Zr5Fe3Co12Y5 multicomponent glassy alloy exhibits an exceptionally high glass transition temperature of 1202 K and a crystallization temperature of 1324 K, as well as an ultrahigh hardness of 18 GPa. This example is used to demonstrate metallic glasses that possess extraordinary thermal stability and ultrahigh strength and, at the same time, a wide supercooled liquid region (122 K) that is needed for processing into bulk forms through powder metallurgy routes.
RI Ma, En/A-3232-2010
SN 0884-2914
PD NOV
PY 2005
VL 20
IS 11
BP 2910
EP 2913
DI 10.1557/JMR.2005.0372
UT WOS:000233095400005
ER

PT J
AU Shen, YT
   Kim, TH
   Gangopadhyay, AK
   Kelton, KF
AF Shen, Y. T.
   Kim, T. H.
   Gangopadhyay, A. K.
   Kelton, K. F.
TI Icosahedral Order, Frustration, and the Glass Transition: Evidence from
   Time-Dependent Nucleation and Supercooled Liquid Structure Studies
SO PHYSICAL REVIEW LETTERS
AB One explanation for the glass transition is a geometrical frustration owing to the development of nonspace-filling short-range order (icosahedral, tetrahedral). However, experimental demonstrations of this are lacking. Here, the first quantitative measurements of the time-dependent nucleation rate in a Zr59Ti3Cu20Ni8Al10 bulk metallic glass are combined with the first measurements of the evolution of the supercooled liquid structure to near the glass transition temperature to provide strong support for an icosahedral-order-based frustration model for the glass transition in Zr-based glasses.
SN 0031-9007
EI 1079-7114
PD FEB 6
PY 2009
VL 102
IS 5
AR 057801
DI 10.1103/PhysRevLett.102.057801
UT WOS:000263166400070
PM 19257557
ER

PT J
AU Christie, JK
AF Christie, J. K.
TI Atomic structure of biodegradable Mg-based bulk metallic glass
SO PHYSICAL CHEMISTRY CHEMICAL PHYSICS
AB We have used highly accurate first-principles molecular dynamics simulations to elucidate the structure of Mg60Zn35Ca5 and Mg72Zn23Ca5 bulk metallic glasses, which are candidate materials for biomedical implants; these two compositions exhibit different behaviours when implanted. The environments of each species are different, and average coordination numbers are similar to 13 for Mg, similar to 11 for Zn and similar to 18-19 for Ca. A wide range of local environments were found and icosahedral motifs, often seen in bulk metallic glasses, were among the most common for both Mg and Zn. Through the computation of a chemical short-range order parameter, a moderate avoidance of Zn-Zn bonding over Zn-Mg or Zn-Ca was observed. No statistically significant difference in structure was observed between the two compositions.
OI Christie, Jamieson/0000-0001-6105-561X
SN 1463-9076
EI 1463-9084
PY 2015
VL 17
IS 19
BP 12894
EP 12898
DI 10.1039/c4cp03714f
UT WOS:000354195300052
PM 25906985
ER

PT J
AU Hui, XD
   Xu, ZY
   Wu, Y
   Chen, XH
   Liu, XJ
   Lu, ZP
AF Hui XiDong
   Xu ZhiYi
   Wu Yuan
   Chen XiaoHua
   Liu XiongJun
   Lu ZhaoPing
TI Magnetocaloric effect in Er-Al-Co bulk metallic glasses
SO CHINESE SCIENCE BULLETIN
AB A series of Er-Al-Co bulk metallic glasses (BMGs) have been prepared by the copper mold casting method. The glass forming ability and magnetocaloric effect (MCE) for these alloys have been investigated. The second-order magnetic transition from para-magnetic to ferromagnetic states takes place at about 9 K. These BMGs exhibit excellent MCE because of their large effective magneton number; Er(56)Al(24)Co(20) BMG has a maximum entropy change and refrigeration capacity of 16.06 J kg(-1) K(-1) and 546 J kg(-1), respectively, under the field of 50 kOe (10 kOe=795.775 kA/m) indicating that these BMGs are potential candidate magnetic materials for hydrogen liquefaction.
RI Lu, Zhao-Ping/A-2718-2009; Liu, Xiong-Jun/C-7119-2009; Wu,
   Yuan/C-4025-2015; Hui, Xidong/A-1741-2010
OI Lu, Zhao-Ping/0000-0003-1463-8948; Wu, Yuan/0000-0001-7857-0247;
SN 1001-6538
PD DEC
PY 2011
VL 56
IS 36
BP 3978
EP 3983
DI 10.1007/s11434-011-4855-7
UT WOS:000297866900016
ER

PT S
AU Zhang, XF
   Li, HX
   Jiang, ZQ
   Yi, S
AF Zhang, X. F.
   Li, H. X.
   Jiang, Z. Q.
   Yi, S.
BE Wang, RM
   Wu, Y
   Wu, XF
TI Effect of Minor Si and Ag Additions on Glass-Forming Ability of
   Ti-Cu-Co-Zr-Sn-Be Bulk Metallic Glass
SO NANO-SCALE AND AMOURPHOUS MATERIALS
SE Materials Science Forum
CT 11th International Conference in Asia of the
   International-Union-of-Materials-Research-Societies
CY SEP 25-28, 2010
CL Qingdao, PEOPLES R CHINA
SP Int Union Mat Res Soc (IUMRS), Chinese Mat Res Soc, Mat Res Soc Taiwan, Mat Res Soc Japan, Govt Qingdao City
AB Effect of Minor Si and Ag additions on glass-forming ability (GFA) of the base Ti44Cu38.9Co4Zr6Sn2Be5.1 (at. %) alloy are studied. (Ti44Cu38.9Co4Zr6Sn2Be5.1)(100-x)R-x (R = Si, Ag, x=0, 1, 2, 3 at. %) metallic glasses are formed by splat-quenching and copper mold suction casting. It is found that the minor Si and Ag additions enhance the glass-forming ability of Ti44Cu38.9Co4Zr6Sn2Be5.1 greatly. The maximum size of fully amorphous structure is increased from 4 mm for base alloy to be larger than 6 mm for alloys containing 1-2 at. % Si/Ag, while Si/Ag more than 2 at. % additions decrease the GFA.
SN 0255-5476
PY 2011
VL 688
BP 400
EP +
DI 10.4028/www.scientific.net/MSF.688.400
UT WOS:000302644200071
ER

PT S
AU Sun, M
   Feng, T
   Peng, G
   Rauf, A
   Wu, B
AF Sun, Ming
   Feng, Tao
   Peng, Guo
   Rauf, Abdur
   Wu, Bin
BE Adiguzel, O
   Doumanidis, CC
   Wang, C
   Wang, MC
   Zaidi, B
TI Nanostructure and Magnetic Properties of FeZr/CuZr Amorphous Multilayers
SO PROCEEDINGS OF THE 3RD ANNUAL INTERNATIONAL CONFERENCE ON ADVANCED
   MATERIAL ENGINEERING (AME 2017)
SE AER-Advances in Engineering Research
CT 3rd Annual International Conference on Advanced Material Engineering
   (AME)
CY APR 14-16, 2017
CL Shanghai, PEOPLES R CHINA
AB Fe75Zr25/Cu64Zr36 multilayers metallic glass thin films have been prepared by magnetron sputtering herein. The magnetic studies shown that the saturation magnetization of this alloys increase after annealing under glass transition temperature (Tg). The change of the magnetization might be attribute to structural transformation triggered by diffusion. This work offers an avenue to modify the nanostructure of thin films, and further to manipulate the physical properties of metallic glasses.
SN 2352-5401
BN 978-94-6252-336-4
PY 2017
VL 110
BP 22
EP 27
UT WOS:000416095400004
ER

PT S
AU Huang, YJ
   Tjahyono, NI
   Shen, J
   Chiu, YL
AF Huang, Yongjiang
   Tjahyono, Nursiani Indah
   Shen, Jun
   Chiu, Yu Lung
BE LeBourhis, E
   Morris, DJ
   Oyen, ML
   Schwaier, R
   Staedler, T
TI Reverse plasticity in nanoindentation
SO FUNDAMENTALS OF NANOINDENTATION AND NANOTRIBOLOGY IV
SE Materials Research Society Symposium Proceedings
CT Symposium on Fundamentals of Nanoindentation and Nanotribology IV held
   at the 2007 MRS Fall Meeting
CY NOV 26-29, 2007
CL Boston, MA
SP Mat Res Soc, CNRS St Gobain, FEI Electron Opt BV, Hysitron Inc, MTS Nano Instruments, Natl Inst Stand & Technol
AB This paper summarises our recent cyclic nanoindentation experiment studies on a range of materials including single crystal and nanocrystalline copper, single crystal aluminium and bulk metallic glasses with different glass transition temperatures. The unloading and reloading processes of the nanoindentation curves have been analysed. The reverse plasticity will be discussed in the context of plastic deformation mechanisms involved. The effect of loading rates on the mechanical properties of materials upon cyclic loading will also he discussed.
RI Huang, Yongjiang/D-4809-2009
SN 0272-9172
BN 978-1-55899-989-3
PY 2008
VL 1049
BP 135
EP +
UT WOS:000256390600021
ER

PT J
AU Flores, KM
AF Flores, KM
TI Structural changes and stress state effects during inhomogeneous flow of
   metallic glasses
SO SCRIPTA MATERIALIA
AB The inhomogencous flow behavior of metallic glasses is reviewed. Structural changes associated with serrated flow and related models for yielding are highlighted. A broad range of experimental results, analytical models, and computer simulations indicate that metallic glasses exhibit asymmetry in tension and compression, consistent with the Mohr-Coulomb model for yielding. (c) 2005 Acta Materialia Inc. Published by Elsevier Ltd. All rights reserved.
SN 1359-6462
PD FEB
PY 2006
VL 54
IS 3
BP 327
EP 332
DI 10.1016/j.scriptamat.2005.04.049
UT WOS:000233495600003
ER

PT J
AU Yang, L
   Xia, JH
   Wang, Q
   Dong, C
   Chen, LY
   Ou, X
   Liu, JF
   Jiang, JZ
   Klementiev, K
   Saksl, K
   Franz, H
   Schneider, JR
   Gerward, L
AF Yang, L.
   Xia, J. H.
   Wang, Q.
   Dong, C.
   Chen, L. Y.
   Ou, X.
   Liu, J. F.
   Jiang, J. Z.
   Klementiev, K.
   Saksl, K.
   Franz, H.
   Schneider, J. R.
   Gerward, L.
TI Design of Cu8Zr5-based bulk metallic glasses
SO APPLIED PHYSICS LETTERS
AB Basic polyhedral clusters have been derived from intermetallic compounds at near-eutectic composition by considering a dense packing and random arrangement of atoms at shell sites. Using such building units, bulk metallic glasses can be formed. This strategy was verified in the Cu-Zr binary system, where we have demonstrated the existence of Cu8Zr5 icosahedral clusters in Cu61.8Zr38.2, Cu64Zr36, and Cu64.5Zr35.5 amorphous alloys. Furthermore, ternary bulk metallic glasses can be developed by doping the basic Cu-Zr alloy with a minority element. This hypothesis was confirmed in systems (Cu0.618Zr0.382)(100-x)Nb-x, where x=1.5 and 2.5 at. %, and (Cu0.618Zr0.382)(98)Sn-2. The present results may open a route to prepare amorphous alloys with improved glass forming ability. (c) 2006 American Institute of Physics.
RI Chen, Lianyi/B-3156-2008; Gerward, Leif/A-7843-2011
OI Chen, Lianyi/0000-0003-3720-398X; Klementiev,
   Konstantin/0000-0002-9468-4188
SN 0003-6951
EI 1077-3118
PD JUN 12
PY 2006
VL 88
IS 24
AR 241913
DI 10.1063/1.2213020
UT WOS:000238314800031
ER

PT J
AU Ragani, J
   Volland, A
   Gravier, S
   Blandin, JJ
   Suery, M
AF Ragani, J.
   Volland, A.
   Gravier, S.
   Blandin, J. J.
   Suery, M.
TI Metallic glass/light alloy (MEGA) multimaterials elaborated by
   co-pressing at high temperature
SO JOURNAL OF ALLOYS AND COMPOUNDS
CT 15th International Symposium on Metastable, Amorphous and Nanostructured
   Materials
CY JUL 06-10, 2008
CL Buenos Aires, ARGENTINA
AB Metallic glass/light alloy (MEGA) multilayered materials are elaborated by co-pressing at high temperature They constitute a layer of Zr-based bulk metallic glass inserted between two layers of either aluminium or magnesium alloys The co-pressing tests are performed at various temperatures above the glass transition temperature of the glass under inert gas. The selected conditions for co-pressing are chosen from the knowledge of the rheological behaviour (deduced from strain rate jump tests) of both the metallic glass and the light alloys and also from information concerning the thermal stability of the glass. It is shown that the bonding between the metallic glass and the light alloys is improved by increasing the strain undergone by the glass during the process. Bonding is also improved by increasing co-pressing temperature, keeping in mind that the amorphous structure of the glass must also be preserved during the process (C) 2009 Elsevier B V All rights reserved.
SN 0925-8388
PD APR 16
PY 2010
VL 495
IS 2
BP 323
EP 326
DI 10.1016/j.jallcom.2009.10.101
UT WOS:000278749900009
ER

PT S
AU Wang, Q
   Zhu, CL
   Dong, C
   Qiang, JB
   Zhang, W
   Inoue, A
AF Wang, Q.
   Zhu, C. L.
   Dong, C.
   Qiang, J. B.
   Zhang, W.
   Inoue, A.
BE Popel, PS
TI Formation and soft magnetic properties of Co (-Fe)-Si-B-Nb bulk metallic
   glasses in relation to clusters
SO 13TH INTERNATIONAL CONFERENCE ON LIQUID AND AMORPHOUS METALS
SE Journal of Physics Conference Series
CT 13th Conference on Liquid and Amorphous Metals
CY JUL 08-14, 2007
CL Ekaterinburg, RUSSIA
AB Bulk metallic glass formations in Co-based alloy systems are investigated with the guidance of our cluster line approach and minor-alloying principle. Basic ternary alloy compositions in Co-B-Si system are first determined by cluster lines defined by linking special binary clusters to third elements. Then these basic ternary alloys are further minor-alloyed with 4-5 at. % Nb and bulk metallic glasses of 3 mm in diameter are formed in (Co8B3-Si)-Nb, Co8B3 being dense-packed cluster. The bulk metallic glasses are expressed approximately with a unified simple composition formula: (Co8B3)(1)(Si,Nb)(1). Finally a quantity of Fe substitution for Co further improves the glass-forming abilities and these Co-Fe-based quinary bulk metallic glasses have good soft magnetic properties with high saturation magnetization I-s, up to 0.98T, and low coercive force H-c, below 6 A/m.
SN 1742-6588
PY 2008
VL 98
DI 10.1088/1742-6596/98/1/012017
UT WOS:000259363800017
ER

PT J
AU Hirata, A
   Hirotsu, Y
   Amiya, K
   Inoue, A
AF Hirata, Akihiko
   Hirotsu, Yoshihiko
   Amiya, Kenji
   Inoue, Akihisa
TI Nanoscale metastable state exhibiting pseudotenfold diffraction pattern
   in Fe-based bulk metallic glass
SO PHYSICAL REVIEW B
AB We have found nanoscale metastable state with a chi-FeCrMo-like structure exhibiting pseudotenfold diffraction pattern in the course of crystallization in an Fe-based Fe(48)Cr(15)Mo(14)C(15)B(6)Tm(2) bulk metallic glass with a high glass stability. All the diffraction spots were found along the first and second halo rings of the glass structure. It was also found that the diffraction pattern with the pseudotenfold symmetry changed into the [113] zone-axis pattern of the chi-FeCrMo structure. On the basis of the results, we discussed local atomic arrangements of this metastable state in relation to the glass stability.
RI Amiya, Kenji/P-6132-2014; Inoue, Akihisa/E-5271-2015; Hirata,
   Akihiko/A-4850-2010
SN 1098-0121
PD JAN
PY 2009
VL 79
IS 2
AR 020205
DI 10.1103/PhysRevB.79.020205
UT WOS:000262978100005
ER

PT J
AU Sheng, WB
AF Sheng, WB
TI Correlations between critical section thickness and glass-forming
   ability criteria of Ti-based bulk amorphous alloys
SO JOURNAL OF NON-CRYSTALLINE SOLIDS
AB Correlations between the critical section thickness and the glass-forming ability (GFA) parameters, including the temperature interval of the supercooled liquid region Delta T-xg, the reduced glass transition temperature T-rg, the stability parameter S and parameters K-gl and gamma, have been summarized for several Ti-based bulk amorphous alloys. The results show that the indicators T-rg and gamma are preferred to be the criteria of GFA for Ti-based bulk amorphous alloys. (c) 2005 Elsevier B.V. All rights reserved.
SN 0022-3093
EI 1873-4812
PD OCT 1
PY 2005
VL 351
IS 37-39
BP 3081
EP 3086
DI 10.1016/j.jnoncrysol.2005.07.021
UT WOS:000232347200016
ER

PT J
AU Politis, C
AF Politis, Constantin
TI Bulk amorphous and nanostructured materials by high energy ball milling
SO INTERNATIONAL JOURNAL OF MODERN PHYSICS B
AB Mechanical alloying by high energy ball milling is a promising method for the production of bulk metallic glasses, amorphous alloys and nanostructured materials. Amorphous alloys reinforced by nanocrystals represent a big step in the optimization of new ultra high strength materials. Nano-scale W-Y solid solutions were synthesized by high energy ball milling. Only 1 at. % oxygen can stabilize the bulk metallic glassy state of Zr54Cu19Ni8Al8Si5Ti5O1 to higher T-x and to large supercooled liquid range Delta T-xg.
SN 0217-9792
PD JUL 30
PY 2008
VL 22
IS 18-19
BP 2905
EP 2913
DI 10.1142/S0217979208047742
UT WOS:000258876700016
ER

PT J
AU Khonik, SV
   Makarov, AS
   Podurets, KM
   Lysenko, AV
   Khonik, VA
AF Khonik, S. V.
   Makarov, A. S.
   Podurets, K. M.
   Lysenko, A. V.
   Khonik, V. A.
TI Comparative study of relaxation behavior of glassy "usual"
   Pd40Cu30Ni10P20 and "unusual" Pd40Cu40P20 by measurements of the
   electrical resistance
SO INTERMETALLICS
AB Isothermal relaxation of the electrical resistance of glassy Pd40Cu30Ni10P20 and Pd40Cu40P20 has been studied. The first glass displays usual density increase upon structural relaxation and crystallization while the second one is fully unique, its density decreases upon crystallization. It has been found that the isothermal relaxation of the electrical resistance in these glasses is quite similar indicating that the density changes occurring upon aging do not appear to be a reason for the observed relaxation. (C) 2011 Elsevier Ltd. All rights reserved.
RI Makarov, Andrey/H-4156-2013; Khonik, Vitaly/A-5888-2009; Podurets,
   Konstantin/E-5449-2014
OI Makarov, Andrey/0000-0001-6741-0619; Podurets,
   Konstantin/0000-0003-2215-3692
SN 0966-9795
PD JAN
PY 2012
VL 20
IS 1
BP 170
EP 172
DI 10.1016/j.intermet.2011.08.006
UT WOS:000297889300024
ER

PT J
AU Asami, K
   Pang, SJ
   Zhang, T
   Inoue, A
AF Asami, K
   Pang, SJ
   Zhang, T
   Inoue, A
TI Preparation and corrosion resistance of Fe-Cr-Mo-C-B-P bulk glassy
   alloys
SO JOURNAL OF THE ELECTROCHEMICAL SOCIETY
AB Fe-based bulk metallic glasses with high corrosion resistance were prepared and evaluated. By using copper mold casting, bulk glasses with a thickness up to 2.5 mm were able to be prepared in the Fe-Cr-Mo-C-B-P system. The Fe-Cr-Mo-C-B-P system metallic glasses exhibited a composition range which has a large supercooled liquid region up to 90 K and a highly reduced glass transition temperature reaching 0.6, indicating a high glass-forming ability (GFA) and high thermal stability of the supercooled liquid. They exhibit good corrosion resistance in HCl solutions. Phosphorus has a beneficial effect on corrosion resistance, but a lowered GFA. (C) 2002 The Electrochemical Society.
RI Inoue, Akihisa/E-5271-2015; Pang, Shujie/D-8305-2016; Zhang,
   Tao/O-4911-2014
SN 0013-4651
PD AUG
PY 2002
VL 149
IS 8
BP B366
EP B369
DI 10.1149/1.1490537
UT WOS:000176815100026
ER

PT J
AU Chen, CQ
   Pei, YT
   De Hosson, JTM
AF Chen, C. Q.
   Pei, Y. T.
   De Hosson, J. Th. M.
TI Strength of submicrometer diameter pillars of metallic glasses
   investigated with in situ transmission electron microscopy
SO PHILOSOPHICAL MAGAZINE LETTERS
AB We have fabricated micro-/nano-pillars of two metallic glasses (MGs), Cu-based Cu47Ti33Zr11Ni6Sn2Si1 and Zr-based Zr50Ti16.5Cu15Ni18.5, respectively, with pillar tip diameters ranging from similar to 650 to similar to 90 nm. These pillars were mechanically tested in situ in transmission electron microscopy as a function of pillar diameters. It turns out that the yield strengths of both MGs are close to those of the bulk glasses and are essentially size independent. Statistical physics description of strength for small size metallic glass is discussed.
RI De Hosson, Jeff/C-2169-2013; Chen, Changqiang/G-6310-2010
OI Pei, Yutao/0000-0002-1817-2228; De Hosson, Jeff/0000-0002-2587-3233
SN 0950-0839
PY 2009
VL 89
IS 10
BP 633
EP 640
DI 10.1080/09500830903236038
UT WOS:000271569700006
ER

PT J
AU Meduri, C
   Hasan, M
   Adam, S
   Kumar, G
AF Meduri, C.
   Hasan, M.
   Adam, S.
   Kumar, G.
TI Effect of temperature on shear bands and bending plasticity of metallic
   glasses
SO JOURNAL OF ALLOYS AND COMPOUNDS
AB We report the effect of testing temperature on the bending plasticity of Pt57.5Cu14.7Ni5.3P22.5 and Zr35Ti30Cu8.25Be26.75 metallic glasses. Bend tests ranging from liquid nitrogen to the glass transition temperature were conducted using a customized setup. The bending strain, the shear band density, and the critical shear offset at fracture decreased with increasing temperature in both the metallic glass formers. The constant strain experiments (without fracture) revealed that low temperature generates more shear bands of smaller lengths to accommodate the same applied strain. The results can be rationalized in terms of temperature dependence predicted by the nucleation and propagation models. (C) 2017 Elsevier B.V. All rights reserved.
RI Hasan, Molla Hasibul/N-6500-2018
OI Hasan, Molla Hasibul/0000-0003-2409-7751
SN 0925-8388
EI 1873-4669
PD JAN 25
PY 2018
VL 732
BP 922
EP 927
DI 10.1016/j.jallcom.2017.10.276
UT WOS:000415931900109
ER

PT J
AU Cai, AH
   Xiong, X
   Liu, Y
   An, WK
   Tan, JY
   Pan, Y
AF Cai, An-hui
   Xiong, Xiang
   Liu, Yong
   An, Wei-ke
   Tan, Jing-ying
   Pan, Ye
TI Design of new Zr-Al-Ni-Cu bulk metallic glasses
SO JOURNAL OF ALLOYS AND COMPOUNDS
AB Five new Zr-Al-Ni-Cu bulk metallic glasses were designed by the average conduction electron concentration ela and average atomic radius R,, criteria on the condition of the weak constraint condition of melting heat Delta H-m. We investigated the relationships between critical dimension Z(max) and such parameters as ela, R-a, difference in atomic radius Delta R, Delta H-m, under-cooled liquid region Delta T-x reduced glass transition ternperature T-rg, K-gl and gamma. The results show that ela, Ra, Delta R and Delta H-m are all strongly related to glass-forming ability (GFA) of alloys. Zr62.5Al12.1CU7.95Ni17.45 owns best thermal stability and GFA among live new Zr-Al-Ni-Cu bulk metallic glasses. K-gl, and gamma are better than Delta T-x, and T-rg at the point of the description of GFA of alloys. In addition, the compositions of glass-forming alloys with best GFA do not locate at but near to eutectic point. (C) 2008 Elsevier BN. All rights reserved.
SN 0925-8388
EI 1873-4669
PD JAN 22
PY 2009
VL 468
IS 1-2
BP 432
EP 437
DI 10.1016/j.jallcom.2008.01.016
UT WOS:000263080000082
ER

PT J
AU Wang, H
   Zhang, HF
   Hu, ZQ
AF Wang, H.
   Zhang, H. F.
   Hu, Z. Q.
TI Tungsten fibre reinforced Zr-based bulk metallic glass composites
SO MATERIALS AND MANUFACTURING PROCESSES
CT International Composites Conference
CY JUL 11-14, 2006
CL Sydney, AUSTRALIA
AB A Zr-based bulk metallic glass (BMG) alloy with the composition (Zr55Al10Ni5Cu30)(98.5)Si-1.5 was used as the base material to form BMG composites. Tungsten fiber reinforced BMG composites were successfully fabricated by pressure metal infiltration technique, with the volume fraction of the tungsten fiber ranging from 10% to 70%. Microstructure and mechanical properties of the BMG composites were investigated. Tungsten reinforcement significantly increased the material's ductility by changing the compressive failure mode from single shear band propagation to multiple shear bands propagation, and transferring stress from matrix to tungsten fibers.
RI Wang, Hao/A-7340-2016
OI Wang, Hao/0000-0001-8869-9669
SN 1042-6914
PY 2007
VL 22
IS 5-6
BP 687
EP 691
DI 10.1080/10426910701384922
UT WOS:000248794900021
ER

PT J
AU Mei, JN
   Li, JS
   Kou, HC
   Fu, HZ
   Zhou, L
AF Mei, JN
   Li, JS
   Kou, HC
   Fu, HZ
   Zhou, L
TI Research development of Ti-based bulk metallic glasses and its
   composites
SO RARE METAL MATERIALS AND ENGINEERING
CT Xi An International Titanium Conference (XITC05)
CY OCT 16-19, 2005
CL Xi an, PEOPLES R CHINA
SP NW Inst Nonferrous Metal Res, Nonferrous Metals Soc China, Asian Off Aerospace Res & Dev
AB The following two methods which are used to obtain Ti-based bulk metallic glasses (BMGs) with desired microstructure and mechanical properties, composition design and controllable phase transitions including liquid-solid and solid-state phase transition, have been discussed in detail in this paper. And also, the research prospect of Ti-based BMGs is proposed. As a result, these two methods have good effect for improving glass forming ability (GFA) of Ti-based BMGs and developing new kinds of glassy/nanocrystalline matrix composites with desired mechanical properties. The tendency of developing Ti-based BMGs is to obtain the application-oriented materials; namely, Ti-based glassy/nanocrystalline matrix composites by controlling the morphology, size, distribution and fraction of the precipitated phase.
SN 1002-185X
PD FEB
PY 2006
VL 35
SU 1
BP 198
EP 201
UT WOS:000237440700048
ER

PT J
AU Tong, Y
   Iwashita, T
   Dmowski, W
   Bei, H
   Yokoyama, Y
   Egami, T
AF Tong, Y.
   Iwashita, T.
   Dmowski, W.
   Bei, H.
   Yokoyama, Y.
   Egami, T.
TI Structural rejuvenation in bulk metallic glasses
SO ACTA MATERIALIA
AB Using high-energy X-ray diffraction we study structural changes in bulk metallic glasses after uniaxial compressive homogeneous deformation at temperatures slightly below the glass transition. We observe that deformation results in structural disordering corresponding to an increase in the fictive, or effective, temperature. However, the structural disordering saturates after yielding. Examination of the experimental structure and molecular dynamics simulation suggests that local changes in the atomic connectivity network are the main driving force of the structural rejuvenation. (C) 2014 Acta Materialia Inc. Published by Elsevier Ltd. All rights reserved.
RI Iwashita, Takuya/D-2724-2009
OI Bei, Hongbin/0000-0003-0283-7990
SN 1359-6454
EI 1873-2453
PD MAR
PY 2015
VL 86
BP 240
EP 246
DI 10.1016/j.actamat.2014.12.020
UT WOS:000349730900022
ER

PT J
AU Shan, SF
   Zhan, ZJ
   Fan, CZ
   Jia, YZ
   Zhang, BQ
   Liu, RP
   Wang, WK
AF Shan Sheng-Feng
   Zhan Zai-Ji
   Fan Chang-Zeng
   Jia Yuan-Zhi
   Zhang Bao-Qing
   Liu Ri-Ping
   Wang Wen-Kui
TI Improvement of Plasticity of Ti-Based Bulk Metallic Glasses by Addition
   of Nb
SO CHINESE PHYSICS LETTERS
AB Ti-based bulk metallic glasses (Ti40Zr25Cu9Ni8Be18)(100-x)Nb-x with x = 0 to 5 at.% are prepared by copper-mold casting. The glass formation ability is almost unchanged by addition of Nb. The compression plasticity is, however, apparently changed, from 3% at x = 0 to 13% at x = 3, about 330% increases at the strain rate of 1 x 10(-4)s(-1). The increment of the plasticity can be attributed to the segregation of Nb in the area of shear bands during the compression processing. An effective way to increase the plasticity of Ti-based bulk metallic glasses is thus proposed.
RI Fan, Changzeng/I-6837-2018
OI Fan, Changzeng/0000-0001-6727-5912
SN 0256-307X
PD NOV
PY 2008
VL 25
IS 11
BP 4165
EP 4167
UT WOS:000260529600089
ER

PT J
AU Li, WH
   Shin, K
   Lee, CG
   Wei, BC
   Zhang, TH
   He, YZ
AF Li, W. H.
   Shin, Keesam
   Lee, C. G.
   Wei, B. C.
   Zhang, T. H.
   He, Y. Z.
TI The characterization of creep and time-dependent properties of bulk
   metallic glasses using nanoindentation
SO MATERIALS SCIENCE AND ENGINEERING A-STRUCTURAL MATERIALS PROPERTIES
   MICROSTRUCTURE AND PROCESSING
AB Viscoelastic deformation and creep behavior of La- and Ce-based bulk metallic glasses (BMGs) with low glass transition temperature are investigated through nanoindentation at room temperature. Creep compliance and retardation spectra are derived to study the creep mechanism. The time-dependent displacement can be well described by a generalized Kelvin model. A modification is proposed to determine the elastic modulus from the generalized Kelvin model. The results are in excellent agreement with the elastic modulus determined by uniaxial compression tests. (c) 2007 Published by Elsevier B.V.
SN 0921-5093
EI 1873-4936
PD APR 15
PY 2008
VL 478
IS 1-2
BP 371
EP 375
DI 10.1016/j.msea.2007.06.015
UT WOS:000254644900049
ER

PT J
AU Li, ZG
   Hui, X
   Zhang, CM
   Wang, ML
   Chen, GL
AF Li, Z. G.
   Hui, X.
   Zhang, C. M.
   Wang, M. L.
   Chen, G. L.
TI Strengthening and toughening of Mg-Cu-(Y, Gd) bulk metallic glasses by
   minor addition of Be
SO MATERIALS LETTERS
AB Mg65Cu25Re10 and Mg65Cu24Be1Re10 (Re=Y, Gd) bulk metallic glasses (BMGs) were successfully fabricated by conventional Cu-mold casting method. By the addition of 1 at.% Be, the compressive strengths of Mg65Cu24Be1Y10 and Mg65Cu24Be1Gd10 alloys are increased from 760 MPa and 860 MPa to 930 MPa and 1025 MPa, respectively. The fracture morphology is changed from nanometer scale corrugation to micrometer scale dimple and vein pattern, indicating that the addition of minor Be obviously improves the toughness of the alloys. The fracture morphologies with different size of plastic zone in Mg-based BMGs provide probability for understanding the fracture mechanism of BMGs. (C) 2007 Elsevier B.V. All rights reserved.
RI Hui, Xidong/A-1741-2010
SN 0167-577X
PD NOV
PY 2007
VL 61
IS 28
BP 5018
EP 5021
DI 10.1016/j.matlet.2007.03.102
UT WOS:000250738700011
ER

PT J
AU Miwa, K
   Tamura, T
AF Miwa, Kenji
   Tamura, Takuya
TI Creation Process of Metallic Glasses by Electromagnetic Vibrations
SO TRANSACTIONS OF THE INDIAN INSTITUTE OF METALS
AB A new method for producing the bulk metallic glasses by using electromagnetic vibrations is proposed and also the effects of the electromagnetic vibrations on the glass forming ability of MgCuY alloys are investigated. It is found that the new method by using the electromagnetic vibrations is effective in enhancing the glass-forming ability of these alloys. Disappearance of the clusters by the electromagnetic vibrations applied to the liquid state is presumed to suppress crystalline nucleation, namely, enhancing the glass-forming ability. The effects of the intensity and frequency of electromagnetic vibrations on the apparent glass-forming ability in Mg-Cu-Y bulk metallic glasses have been clarified. The apparent glass-forming ability of these glasses increases with increase in the frequency of electromagnetic vibrations up to 5000 Hz and also with increase in the intensity of them.
   Moreover, in order to clarify the effects of the electromagnetic vibrations on glass-forming ability for other alloy systems, influence of the intensity of electromagnetic vibration force on glass-forming ability in the Fe-Co-8-Si-Nb alloys have been studied. As a result, it has been found that the glass-forming ability of Fe-Co-B-Si-Nb alloys also increases with increasing the intensity of electromagnetic vibration force.
RI Tamura, Takuya/D-4854-2017
OI Tamura, Takuya/0000-0001-6083-913X
SN 0972-2815
PD APR-JUN
PY 2007
VL 60
IS 2-3
SI SI
BP 339
EP 342
UT WOS:000208324000049
ER

PT J
AU Bakai, AS
AF Bakai, A. S.
TI Heterophase liquid states: Thermodynamics, structure, dynamics
SO CONDENSED MATTER PHYSICS
AB An overview of theoretical results and experimental data on the thermodynamics, structure and dynamics of the heterophase glass-forming liquids is presented. The theoretical approach is based on the mesoscopic heterophase fluctuations model (HPFM) developed within the framework of the bounded partition function approach. The Fischer cluster phenomenon, glass transition, liquid-liquid transformations, parametric phase diagram, cooperative dynamics and fragility of the glass-forming liquids is considered.
SN 1607-324X
EI 2224-9079
PY 2014
VL 17
IS 4
DI 10.5488/CMP.17.43701
UT WOS:000347498800009
ER

PT J
AU Zhang, QS
   Zhang, W
   Inoue, A
AF Zhang, Q. S.
   Zhang, W.
   Inoue, A.
TI Ni-free Zr-Fe-Al-Cu bulk metallic glasses with high glass-forming
   ability
SO SCRIPTA MATERIALIA
AB We report a series of Ni-free quaternary Zr-based bulk metallic glasses developed by substitution of Fe for Cu in ternary Zr60Cu30Al10 alloy. Addition of Fe lowers the liquidus temperature of the ternary Zr60Cu30Al10 alloy. The alloy compositions of Zr60Cu25Fe5Al10 and Zr62.5Cu22.5Fe5Al10 were found to be very near a quaternary eutectic point with low liquidus temperature. The as-cast glassy alloy rods with a diameter of 20 mm were easily synthesized at Zr60Cu25Fe5Al10 and Zr62.5Cu22.5Fe5Al10 to alloy compositions by copper mold casting. (C) 2009 Acta Materialia Inc. Published by Elsevier Ltd. All rights reserved.
RI Inoue, Akihisa/E-5271-2015; zhang, qingsheng/A-4851-2010
SN 1359-6462
PD AUG
PY 2009
VL 61
IS 3
BP 241
EP 244
DI 10.1016/j.scriptamat.2009.03.056
UT WOS:000266788200004
ER

PT J
AU Concustell, A
   Godard-Desmarest, S
   Carpenter, MA
   Nishiyama, N
   Greer, AL
AF Concustell, A.
   Godard-Desmarest, S.
   Carpenter, M. A.
   Nishiyama, N.
   Greer, A. L.
TI Induced elastic anisotropy in a bulk metallic glass
SO SCRIPTA MATERIALIA
AB Full evaluation of the elasticity tensor of a bulk metallic glass (Pd40Cu30Ni10P20) shows anisotropy induced by creep under uniaxial compression changing sign with increasing strain. Anisotropy due to anelastic strain is erased by zero stress annealing. At higher strain anisotropy of opposite sign suggests structural alignment by plastic flow. Induced anisotropy is most evident in the Poisson ratio, may relate to other properties, including the strength and toughness of metallic glasses, and may lead to misinterpretation of annealing effects. (C) 2011 Acta Materialia Inc. Published by Elsevier Ltd. All rights reserved.
RI Nishiyama, Nobuyuki/C-8228-2015; Greer, Lindsay/E-9433-2017; Greer, Alan
   Lindsay/G-1977-2011; Carpenter, Michael/D-4860-2015
OI Concustell, Amadeu/0000-0001-6705-3228
SN 1359-6462
PD JUN
PY 2011
VL 64
IS 12
BP 1091
EP 1094
DI 10.1016/j.scriptamat.2011.02.028
UT WOS:000290422300006
ER

PT J
AU Park, JM
   Lim, KR
   Park, ES
   Hong, S
   Park, KH
   Eckert, J
   Kim, DH
AF Park, J. M.
   Lim, K. R.
   Park, E. S.
   Hong, S.
   Park, K. H.
   Eckert, J.
   Kim, D. H.
TI Internal structural evolution and enhanced tensile plasticity of
   Ti-based bulk metallic glass and composite via cold rollingle
SO JOURNAL OF ALLOYS AND COMPOUNDS
CT International Symposium on Metastable, Amorphous and Nanostructured
   Materials (ISMANAM)
CY JUN 30-JUL 05, 2013
CL Torino, ITALY
AB The influence of cold rolling on the tensile mechanical properties and deformation behavior of Ti-based bulk metallic glass (BMG: Ti40Zr25Ni8Cu9Be18) and beta-Ti dendrite reinforced bulk metallic glass matrix composite (BMGMC: Ti40.2Zr18Ni2.85Cu7.65Be12.3Nb19) has been investigated. The cold-rolled BMG and BMGMC samples with 20% thickness reduction ratio exhibit a pronounced tensile plasticity of 0.8% and 4%, respectively. The malleable behavior of the cold-rolled samples originates from the internal structural evolution and modulation of elastic properties. (C) 2014 Elsevier B.V. All rights reserved.
SN 0925-8388
EI 1873-4669
PD DEC 5
PY 2014
VL 615
SU 1
BP S113
EP S117
DI 10.1016/j.jallcom.2014.01.075
UT WOS:000343613600024
ER

PT J
AU Bendjemil, B
   Bouchareb, A
   Belbah, A
   Bougdira, J
   Piccin, R
   Baricco, M
AF Bendjemil, Badis
   Bouchareb, Abderrezak
   Belbah, Ahmed
   Bougdira, Jamel
   Piccin, Rafael
   Baricco, Marcello
TI Crystallization Behavior of Fe50-xCr15Mo14C15B6Mx (x=0, 2 and M = Y, Gd)
   Bulk Metallic Glasses and Ribbons by in situ High Temperature X-Ray
   Diffraction
SO CHINESE PHYSICS LETTERS
AB The effect of rare earth addition on thermal stability of Fe50-xCr15Mo14C15B6Mx (x = 0, 2 and M=Y, Gd) is studied. Thermal and structural properties are measured using differential scanning calorimetry and x-ray diffraction, respectively. The microstructure is observed by using a scanning electron microscope, and chemical composition is checked by energy dispersive spectroscopy analysis. The effect of high temperature on the isothermal crystallization of Fe50-xCr15Mo14C15B6Mx (x = 0, 2 and M=Y, Gd) bulk metallic glass and ribbons is investigated by high-temperature x-ray diffraction. It is found that the crystallization behavior of Fe50-xCr15Mo14C15B6Mx (x = 0, 2 and M=Y, Gd) bulk metallic glass strongly depends on the annealing temperature. The different crystallization behavior is believed to be due to the different structures that the metallic glass possesses at different temperatures.
RI Baricco, Marcello/B-4075-2013
OI Baricco, Marcello/0000-0002-2856-9894
SN 0256-307X
PD OCT
PY 2012
VL 29
IS 10
AR 108103
DI 10.1088/0256-307X/29/10/108103
UT WOS:000309962700062
ER

PT J
AU Chen, MW
   Zhang, T
   Inoue, A
   Sakai, A
   Sakurai, T
AF Chen, MW
   Zhang, T
   Inoue, A
   Sakai, A
   Sakurai, T
TI Quasicrystals in a partially devitrified Zr65Al7.5Ni10Cu12.5Ag5 bulk
   metallic glass
SO APPLIED PHYSICS LETTERS
AB A quasicrystalline phase was discovered in a Zr65Al7.5Ni10Cu12.5Ag5 bulk metallic glass annealed in the supercooled liquid range. Through systematical transmission electron microscopy rotation analyses, the selected area electron diffraction patterns are obtained corresponding to fivefold, threefold, and twofold rotational symmetry, demonstrating that the precipitated phase is an icosahedral quasicrystalline phase with good rotational symmetry. It is concluded that the addition of Ag in the Zr-based metallic glass promotes the formation of the quasicrystalline phase. The present experimental result implies that there is a natural relationship between the Zr-based bulk metallic glasses and icosahedral quasicrystalline. (C) 1999 American Institute of Physics. [S0003-6951(99)00438-6].
RI Inoue, Akihisa/E-5271-2015; CHEN, Mingwei/A-4855-2010
OI CHEN, Mingwei/0000-0002-8274-3099; Chen, Mingwei/0000-0002-2850-8872
SN 0003-6951
PD SEP 20
PY 1999
VL 75
IS 12
BP 1697
EP 1699
DI 10.1063/1.124793
UT WOS:000082547100015
ER

PT J
AU Takahara, Y
   Narita, N
AF Takahara, Y
   Narita, N
TI Local electronic structures and chemical bonds in Zr-based metallic
   glasses
SO MATERIALS TRANSACTIONS
CT International Symposium on Bulk Glassy Alloys
CY OCT, 2003
CL Yokohama, JAPAN
AB Local electronic structures of Zr-based metallic glasses have been calculated using the discrete variational Xalpha cluster molecular orbital method. The cluster models are constructed for Zr-Cu, Zr-Ni and Zi-Pd metallic glasses on the base of local structure parameters determined by extended X-ray absorption fine structure analysis. The valence-band X-ray photoelectron spectra computed from the local electronic structures agree well with experimental spectra. Based on the feature of chemical bonds in those glasses evaluated from the electronic structures, we discuss the relationship between the chemical bond and the stability of supercooled liquid state, The strength of the Zr-Ni bond is larger than that of Zr-Cu bond, while the Cu-Cu bond strength is nearly equal to the Ni-Ni bond. Moreover, the Zr-Zr bond strength is larger in Zr-Cu glass than in Zr-Ni glass. This indicates that the formation of primary crystalline phase is more difficult in Zr-Cu glass than in Zr-Ni glass. The difficulty contributes to the relative stabilization of supercooled liquid state in Zr-Cu glass compared with that in Zr-Ni glass. It is also shown that local atomic structures of Zr-Pd glass are well described by the icosahedral atomic configuration.
SN 1345-9678
EI 1347-5320
PD APR
PY 2004
VL 45
IS 4
BP 1172
EP 1176
DI 10.2320/matertrans.45.1172
UT WOS:000221314900034
ER

PT J
AU Han, ZH
   Li, XP
   He, L
   Chen, W
   Jiang, F
   Sun, J
AF Han, Z. H.
   Li, X. P.
   He, L.
   Chen, W.
   Jiang, F.
   Sun, J.
TI Experimental characterization of plastic flow initiation in a bulk
   metallic glass via intermittent compression test
SO INTERMETALLICS
AB Intermittently plastic compression tests of the Zr52.5Cu17.9Ni14.6Al10Ti5 bulk metallic glass were performed under a geometrically constrained condition at different cross-head displacement rates. A flow stress-overshoot phenomenon was observed during the macroscopically plastic deformation. It was found that there is a close correlation between the stress-overshoot intensity and the spatial density of shear bands. The stress-overshoot intensity can be used as a parameter to characterize the ability of plastic flow initiation caused by the spatial nucleation of shear bands in the bulk metallic glass. The micromechanisms of shear band multiplication were discussed. (C) 2011 Elsevier Ltd. All rights reserved.
SN 0966-9795
EI 1879-0216
PD JUL
PY 2011
VL 19
IS 7
BP 942
EP 946
DI 10.1016/j.intermet.2011.02.013
UT WOS:000291190900017
ER

PT B
AU Liu, CT
   Greer, AL
   Schuh, CA
AF Liu, C. T.
   Greer, A. Lindsay
   Schuh, Christopher A.
BA Suryanarayana, C
   Inoue, A
BF Suryanarayana, C
   Inoue, A
TI Corrosion Behavior
SO BULK METALLIC GLASSES
BN 978-1-4200-8596-9
PY 2011
BP 307
EP 360
D2 10.1201/9781420085976-1
UT WOS:000288438000008
ER

PT J
AU Dong, C
   Wang, Q
   Chen, WR
   Zhang, QY
   Qiang, JB
   Wang, YM
AF Dong, Chuang
   Wang, Qing
   Chen, Weirong
   Zhang, Qingyu
   Qiang, Jianbing
   Wang, Yingmin
TI Cluster-based composition rules for ternary alloy systems
SO JOURNAL OF UNIVERSITY OF SCIENCE AND TECHNOLOGY BEIJING
AB The cluster-based composition rule in ternary alloy systems including quasicrystals, bulk metallic glasses, crystalline phases and Lave phases-related body-centered cubic (BCC) solid solution forming systems was summarized. The so-called cluster line in a ternary phase diagram refers to a straight composition line linking a specific binary cluster to the third element. The composition ranges of quasicrystals and bulk metallic glasses can be determined by the direct use of cluster lines, where two cluster lines intersect at the optimum phase forming composition. Furthermore, the alloys on the cluster line in Laves phase-related BCC solid solution alloy systems have larger hydrogen storage capacities.
RI Zhang, Qingyu/O-8472-2015
SN 1005-8850
PD JUN
PY 2007
VL 14
SU 1
BP 1
EP 3
DI 10.1016/S1005-8850(07)60096-6
UT WOS:000250593400001
ER

PT J
AU Koralay, H
   Yakuphanoglu, F
   Cavdar, S
   Gunen, A
   Aksu, E
AF Koralay, H
   Yakuphanoglu, F
   Cavdar, S
   Gunen, A
   Aksu, E
TI Crystallization kinetics of Bi1.7V0.3Sr2Ca2Cu3Ox glass-ceramic
SO PHYSICA B-CONDENSED MATTER
AB Crystallization kinetics of Bi1.7V0.3Sr2CaCu3Ox glass-ceramic was investigated using the differential scanning calorimetry (DSC) technique. Three characteristic phenomena were observed in the studied temperature range. The activation energies for glass transition temperature and crystallization phenomena were determined by different theoretical models. Applying the modified Johnson-Mehl-Avrami (JMA) equation reasonably suggests that crystallization process of the Bi1.7V0.3Sr2Ca2Cu3Ox glass-ceramic is carried out by a bulk growth in three dimensions. (C) 2004 Elsevier B.V. All rights reserved.
SN 0921-4526
PD JAN 31
PY 2005
VL 355
IS 1-4
BP 64
EP 71
DI 10.1016/j.physb.2004.10.023
UT WOS:000226733300009
ER

PT J
AU Li, N
   Liu, Z
   Wang, XY
   Zhang, M
AF Li, Ning
   Liu, Ze
   Wang, Xinyun
   Zhang, Meng
TI Vibration-accelerated activation of flow units in a Pd-based bulk
   metallic glass
SO MATERIALS SCIENCE AND ENGINEERING A-STRUCTURAL MATERIALS PROPERTIES
   MICROSTRUCTURE AND PROCESSING
AB Controlled activation of flow units and in-situ characterization of mechanical properties in metallic glasses are facing challenges thus far. Here, vibrational loading is introduced through nanoscale dynamic mechanical analysis technique to probe vibration-accelerated atomic level flow that plays a crucial role in the mechanical behavior of metallic glasses. The intriguing finding is that high vibrational frequency induces deep indentation depth, prominent pop-in events on load-depth curves and low storage modulus, exhibiting a vibration-facilitated activation of flow units in Pd40Cu30Ni10P20 metallic glass. Theoretical analysis revealed that vibration-moderated activation time-scale accelerate the activation of flow units and responsible for the above scenario.
SN 0921-5093
EI 1873-4936
PD APR 24
PY 2017
VL 692
BP 62
EP 66
DI 10.1016/j.msea.2017.03.062
UT WOS:000400718400008
ER

PT J
AU Sung, DS
   Kwon, OJ
   Fleury, E
   Kim, KB
   Lee, JC
   Kim, DH
   Kim, YC
AF Sung, DS
   Kwon, OJ
   Fleury, E
   Kim, KB
   Lee, JC
   Kim, DH
   Kim, YC
TI Enhancement of the glass forming ability of Cu-Zr-Al alloys by Ag
   addition
SO METALS AND MATERIALS INTERNATIONAL
AB The thermal stability and glass forming ability of Cu50-xZr43Al7Agx (x=0, 1, 3, 5, and 7) bulk metallic glass alloys have been investigated. The glass forming ability in the Cu-Zr-Al-Ag alloys increased proportionally to the Ag content and show good correlations with thermal parameters such as DeltaT(x)(=T-x-T-g), T-rg(=T-g/T-l) and gamma(=T-x/(T-g+T-l)). For the Cu43Zr43Al7Ag7 alloy, fully amorphous rods of 8 mm diameter were successfully fabricated by copper mold casting. Mechanical tests on this composition revealed also remarkable properties with compressive strength around 2000 MPa and large ductility.
RI bang, changwook/J-7922-2012
OI lee, jae-chul/0000-0002-9294-2163
SN 1598-9623
PD DEC
PY 2004
VL 10
IS 6
BP 575
EP 579
DI 10.1007/BF03027421
UT WOS:000225797600012
ER

PT J
AU Wu, JL
   Li, WH
   Pan, Y
   Qi, JY
   Wang, JG
AF Wu, Jili
   Li, Weihuo
   Pan, Ye
   Qi, Jinyan
   Wang, Jianguo
TI Microalloying and microstructures of Cu-based bulk metallic glasses &
   composites and relevant mechanical properties
SO MATERIALS & DESIGN
AB To improve the poor plasticity of bulk metallic glasses, this work reports the microalloying of a bulk metallic glass to tailor the microstructures and to enhance its plasticity. The selection of alloying elements, Hf and Mo which are soluble in Ti, is determined by binary phase diagrams. The X-ray diffraction and microstructural analysis demonstrate that the microstructural evolution of as-cast alloys is accompanied with the addition of alloying elements. The glass-forming ability of as-cast alloys increases with the addition of Hf and Mo elements, respectively. The yield strength and plasticity of both as-cast alloys are affected with the quantity of alloying elements. In addition, the intrinsic nature of alloying elements and the interaction among the alloying elements and base compositions affect the hardness of as-cast alloys. Summarily, addition of soluble alloying elements in a component of base composition can enhance the plasticity of bulk metallic glasses. (C) 2015 Elsevier Ltd. All rights reserved.
SN 0261-3069
EI 1873-4197
PD JAN 5
PY 2016
VL 89
BP 1130
EP 1136
DI 10.1016/j.matdes.2015.10.054
UT WOS:000366225200129
ER

PT J
AU Guo, S
   Liu, CT
AF Guo, Sheng
   Liu, C. T.
TI Phase stability in high entropy alloys: Formation of solid-solution
   phase or amorphous phase
SO PROGRESS IN NATURAL SCIENCE-MATERIALS INTERNATIONAL
AB The alloy design for equiatomic multi-component alloys was rationalized by statistically analyzing the atomic size difference, mixing enthalpy, mixing entropy, electronegativity, valence electron concentration among constituent elements in solid solutions forming high entropy alloys and amorphous alloys. Solid solution phases form and only form when the requirements of the atomic size difference, mixing enthalpy and mixing entropy are all met. The most significant difference between the solid solution forming high entropy alloys and bulk metallic glasses lies in the atomic size difference. These rules provide valuable guidance for the future development of high entropy alloys and bulk metallic glasses.
RI Guo, Sheng/C-7746-2009
OI Guo, Sheng/0000-0001-8349-3135; Liu, Chain Tsuan/0000-0001-7888-9725
SN 1002-0071
EI 1745-5391
PD DEC
PY 2011
VL 21
IS 6
BP 433
EP 446
DI 10.1016/S1002-0071(12)60080-X
UT WOS:000299647700001
ER

PT J
AU Henits, P
   Revesz, A
   Kovacs, Z
AF Henits, Peter
   Revesz, Adam
   Kovacs, Zsolt
TI Free volume simulation for severe plastic deformation of metallic
   glasses
SO MECHANICS OF MATERIALS
AB Evolution of internal parameters of metallic glasses subjected to high pressure torsion was simulated using a free volume based thermo-mechanical model. Steady state has been predicted in the whole, plastically sheared volume for large strains irrespectively from the applied revolution rate. Based on the simplification of the model equations in steady state, simple methods were obtained to determine the in situ temperature increase and stress distribution from external torque measurements. (C) 2012 Elsevier Ltd. All rights reserved.
RI Kovacs, Zsolt/A-8091-2010
OI Kovacs, Zsolt/0000-0001-6802-3311
SN 0167-6636
PD JUL
PY 2012
VL 50
BP 81
EP 87
DI 10.1016/j.mechmat.2012.03.008
UT WOS:000306248600008
ER

PT J
AU Lee, KS
   Jun, HJ
   Lee, YS
AF Lee, Kwang Seok
   Jun, Hyun-Joon
   Lee, Young-Seon
TI Fabrication of bimetallic rods consist of a Zr-based bulk metallic glass
   and a crystalline copper by co-extrusion
SO INTERMETALLICS
CT 7th International Conference on Bulk-Metallic Glasses
CY NOV 01-05, 2009
CL Busan, SOUTH KOREA
SP Yonsei Univ, Ctr Noncrystalline Mat
AB In this preliminary work, we demonstrated rod-type Zr(44)Ti(11)Cu(9.8)Ni(10.2)Be(25) (LM1B) bulk metallic glass/crystalline copper composites fabricated by co-extrusion. The co-extrusion tests were carried out at three different temperature-punch speed combinations within supercooled liquid region of the LM1B bulk metallic glass. The influence of process conditions on the homogeneity of the area fraction between core and sleeve along the extrusion axis, the existence and thickness of the interface layer with or without defect, and overall strength of this bimetallic compound was systematically investigated by various experiments. (C) 2010 Elsevier Ltd. All rights reserved.
SN 0966-9795
PD OCT
PY 2010
VL 18
IS 10
SI SI
BP 1958
EP 1963
DI 10.1016/j.intermet.2010.01.018
UT WOS:000281420700039
ER

PT J
AU Wang, YX
   Yao, JH
   Li, Y
AF Wang, Yinxiao
   Yao, Jiahao
   Li, Yi
TI Glass formation adjacent to the intermetallic compounds in Cu-Zr binary
   system
SO JOURNAL OF MATERIALS SCIENCE & TECHNOLOGY
AB On the contrary to the common belief that glass formation is unfeasible near terminal intermetallic compound due to fast crystallization kinetics, here we present our findings that bulk metallic glasses are readily formed near intermetallic compounds, far away from the traditional region of glass forming near eutectics. While the intermetallic compounds themselves are not possible glass formers, bulk metallic glasses can be quenched compositionally neighboring the intermetallic compounds as close as 0.5 at.%. Taking binary Cu-Zr as a model system, the phenomenon of two optimum glass forming compositions sandwiching the corresponding intermetallic compounds (Cu51Zr14, Cu10Zr7, CuZr, CuZr2 and Cu8Zr3) is observed consistently. This new scenario of "intermetallic glass" is verified by the thermodynamic principle that the alloy liquids neighboring the intermetallic compounds possess lower Gibbs free energy than that of the compounds themselves. Furthermore, the sluggish crystallization behavior of these liquids provides an additional kinetic explanation. (C) 2017 Published by Elsevier Ltd on behalf of The editorial office of Journal of Materials Science & Technology.
SN 1005-0302
PD APR
PY 2018
VL 34
IS 4
BP 605
EP 612
DI 10.1016/j.jmst.2017.09.008
UT WOS:000428188900003
ER

PT J
AU Wang, Q
   Yang, Y
   Jiang, H
   Liu, CT
   Ruan, HH
   Lu, J
AF Wang, Q.
   Yang, Y.
   Jiang, H.
   Liu, C. T.
   Ruan, H. H.
   Lu, J.
TI Superior Tensile Ductility in Bulk Metallic Glass with Gradient
   Amorphous Structure
SO SCIENTIFIC REPORTS
AB Over centuries, structural glasses have been deemed as a strong yet inherently 'brittle' material due to their lack of tensile ductility. However, here we report bulk metallic glasses exhibiting both a high strength of similar to 2 GPa and an unprecedented tensile elongation of 2-4% at room temperature. Our experiments have demonstrated that intense structural evolution can be triggered in theses glasses by the carefully controlled surface mechanical attrition treatment, leading to the formation of gradient amorphous microstructures across the sample thickness. As a result, the engineered amorphous microstructures effectively promote multiple shear banding while delay cavitation in the bulk metallic glass, thus resulting in superior tensile ductility. The outcome of our research uncovers an unusual work-hardening mechanism in monolithic bulk metallic glasses and demonstrates a promising yet low-cost strategy suitable for producing large-sized, ultra-strong and stretchable structural glasses.
RI Ruan, Haihui/F-4337-2010; Yang, Yong/G-9148-2011
OI Ruan, Haihui/0000-0002-9233-8820; Yang, Yong/0000-0002-0491-8295; LU,
   Jian/0000-0001-5362-0316; Liu, Chain Tsuan/0000-0001-7888-9725
SN 2045-2322
PD APR 23
PY 2014
VL 4
AR 4757
DI 10.1038/srep04757
UT WOS:000334836500004
PM 24755683
ER

PT J
AU Cho, H
   Choi, H
   Jo, H
   Lim, S
   Kim, B
   Lee, C
AF Cho, Hoon
   Choi, Hanshin
   Jo, Hyungho
   Lim, Sungchui
   Kim, Byoungmoon
   Lee, Changhee
TI Oxidation induced phase instability of Cu54Zr22Ti18Ni6 bulk metallic
   glass
SO MATERIALS SCIENCE AND ENGINEERING A-STRUCTURAL MATERIALS PROPERTIES
   MICROSTRUCTURE AND PROCESSING
CT 12th International Conference on Rapidly Quenched and Metastable
   Materials
CY AUG 21-26, 2005
CL Jeju Isl, SOUTH KOREA
AB Effects of isothermal oxidation on the chemical instability which triggers crystallization of Cu54Zr22Ti18Ni6 (numbers indicate at.%) bulk metallic glass were investigated. Comparing the isothermal heat flux of Cu54Zr22Ti18Ni6 bulk metallic glass within the supercooled liquid region in differential scanning calorimetry, it could be observed that the isothermal oxidation did decrease the incubation time for crystallization. What is further, this amorphous phase instability was enhanced with the decrease of particle size. Through this study, chemical instability due to preferential oxidation of alloying element and resulting deviation of chemical composition in intact metallic region needs to be considered in phase evolution of bulk metallic glass during material processing. (c) 2006 Elsevier B.V. All rights reserved.
SN 0921-5093
PD MAR 25
PY 2007
VL 449
BP 118
EP 121
DI 10.1016/j.msea.2006.03.136
UT WOS:000245477800024
ER

PT J
AU Ritter, Y
   Sopu, D
   Gleiter, H
   Albe, K
AF Ritter, Yvonne
   Sopu, Daniel
   Gleiter, Herbert
   Albe, Karsten
TI Structure, stability and mechanical properties of internal interfaces in
   Cu64Zr36 nanoglasses studied by MD simulations
SO ACTA MATERIALIA
AB Internal interfaces in a Cu64Zr36 glass were generated by joining two planar surfaces and studied with respect to their structure and stability by means of molecular dynamics simulations. In analogy to grain boundaries in crystalline materials, glass glass interfaces are characterized by a defective short-range order and an excess free volume of about 1-2%. They are stable even close to T-g and exhibit similarities to shear bands that occur in deformed metallic glasses. When deformed in uniaxial tension, pre-existing interfaces promote shear band formation and yielding occurs at lower stress than in the homogeneous bulk glass sample. The results confirm the concept of internal interfaces in nanoglasses. (C) 2011 Acta Materialia Inc. Published by Elsevier Ltd. All rights reserved.
RI Albe, Karsten/F-1139-2011; Sopu, Daniel/F-8891-2015
OI Albe, Karsten/0000-0003-4669-8056;
SN 1359-6454
PD OCT
PY 2011
VL 59
IS 17
BP 6588
EP 6593
DI 10.1016/j.actamat.2011.07.013
UT WOS:000295446000009
ER

PT J
AU Wang, Q
   Pelletier, JM
   Dong, YD
   Ji, YF
AF Wang, Q
   Pelletier, JM
   Dong, YD
   Ji, YF
TI Structural relaxation and crystallisation of bulk metallic glasses
   Zr41Ti14CU12.5Ni10-xBe22.5Fex (x=0 or 2) studied by mechanical
   spectroscopy
SO MATERIALS SCIENCE AND ENGINEERING A-STRUCTURAL MATERIALS PROPERTIES
   MICROSTRUCTURE AND PROCESSING
CT 13th International Conference on Internal Friction and Ultrasonic
   Attentuation in Solids
CY JUL 08-12, 2002
CL Bilbao, SPAIN
AB The measurements of the internal friction and dynamic shear modulus as well as differential scanning calorimetry have been performed in order to investigate the structural relaxation and crystallization of Zr41Ti14Cu12.5Be22.5Fex (x = 0 or 2) bulk metallic glasses. It is found that the glass transition is retarded and the thermal stability of supercooled liquid is increased by the Fe addition. The experimental results are well analyzed using a physical model, which can characterize atomic mobility and mechanical response of disordered condensed materials. (C) 2003 Elsevier B.V. All rights reserved.
SN 0921-5093
PD APR 15
PY 2004
VL 370
IS 1-2
SI SI
BP 316
EP 320
DI 10.1016/j.msea.2003.08.084
UT WOS:000221238500061
ER

PT S
AU Jeong, HG
   Kim, WJ
   Bae, JC
   Yoon, DJ
   Choi, S
   Na, KH
AF Jeong, HG
   Kim, WJ
   Bae, JC
   Yoon, DJ
   Choi, S
   Na, KH
BE Zhong, ZY
   Saka, H
   Kim, TH
   Holm, EA
   Han, YF
   Xie, XS
TI Hole punching onto the Zr(65)Al(10)Ni(10)CU(15)BMG sheet fabricated by
   squeeze casting
SO PRICM 5: THE FIFTH PACIFIC RIM INTERNATIONAL CONFERENCE ON ADVANCED
   MATERIALS AND PROCESSING, PTS 1-5
SE Materials Science Forum
CT 5th Pacific Rim International Conference on Advanced Materials and
   Processing
CY NOV 02-05, 2004
CL Beijing, PEOPLES R CHINA
SP Chinese Soc Met, Japan Inst Met, Korea Inst Met & Mat, Minerals, Met & Mat Soc
AB Bulk metallic glass Zr65Al10Ni10Cu15 was fabricated in a sheet form with thickness 1.5 mm by a squeeze casting method. The structure of the as-cast Zr65Al10Ni10Cu15 sheet was confirmed to be fully amorphous. The sheet was punched into a blank under high hydrostatic pressure at room temperature. A round hole was created with a possible evidence for plastic-like deformation along the edge of rim. No visible cracks were observed around the hole. This result indicates that bulk metallic glasses, which are known to be very brittle at room temperature, can be deformed in a ductile mode under hydrostatic pressure condition. Hydrostatic pressure may suppress the formation and development of micro defects leading to ductile fracture.
SN 0255-5476
BN 0-87849-960-1
PY 2005
VL 475-479
BP 3423
EP 3426
DI 10.4028/www.scientific.net/MSF.475-479.3423
PN 1-5
UT WOS:000227494704019
ER

PT S
AU Fedorov, VA
   Yakovlev, AV
   Pluzhnikova, TN
   Shlikova, AA
   Berezner, AD
AF Fedorov, V. A.
   Yakovlev, A. V.
   Pluzhnikova, T. N.
   Shlikova, A. A.
   Berezner, A. D.
GP IOP
TI Influence of laser irradiation on change properties of bulk amorphous
   Zr-Pd metallic alloys
SO XII INTERNATIONAL CONFERENCE RADIATION-THERMAL EFFECTS AND PROCESSES IN
   INORGANIC MATERIALS
SE IOP Conference Series-Materials Science and Engineering
CT 12th International Scientific Conference on Radiation-Thermal Effects
   and Processes in Inorganic Materials
CY SEP 04-12, 2016
CL Tomsk, RUSSIA
AB We study the morphological features of laser irradiation zones formed on the surface of the bulk metallic glasses. We use the nanoindentation method for estimation alloys properties caused by impulse heating during irradiation.
RI Fedorov, Victor/U-2259-2017; Berezner, Arseniy/F-5478-2017
SN 1757-8981
PY 2017
VL 168
AR UNSP 012052
DI 10.1088/1757-899X/168/1/012052
UT WOS:000398729700052
ER

PT J
AU Zhang, J
   Zhang, HF
   Quan, M
   Hu, ZQ
AF Zhang, J
   Zhang, HF
   Quan, M
   Hu, ZQ
TI Effect of pressure on thermal stability of Mg65Cu25Y10 bulk metallic
   glass
SO SCRIPTA MATERIALIA
AB The pressure effect on thermal stability of Mg65Cu2Y10 bulk metallic glass has been investigated in the pressure range up to 4 GPa. It was found that the behavior of the onset crystallization temperature versus pressure significantly differs in various pressure ranges. (C) 2003 Published by Elsevier Ltd. on behalf of Acta Materialia Inc.
SN 1359-6462
PD SEP
PY 2003
VL 49
IS 6
BP 485
EP 489
DI 10.1016/S1359-6462(03)00368-3
UT WOS:000184265600001
ER

PT J
AU Cai, AH
   Xiong, X
   Liu, Y
   An, WK
   Zhou, GJ
   Luo, Y
   Li, TL
   Li, XS
   Tan, XF
AF Cai, An-hui
   Xiong, Xiang
   Liu, Yong
   An, Wei-ke
   Zhou, Guo-jun
   Luo, Yun
   Li, Tie-lin
   Li, Xiao-song
   Tan, Xiang-fu
TI Compositional optimization of glass forming alloys based on critical
   dimension by using artificial neural network
SO TRANSACTIONS OF NONFERROUS METALS SOCIETY OF CHINA
AB An artificial neural network (ANN) model was developed for simulating and predicting critical dimension d(c) of glass forming alloys. A group of Zr-Al-Ni-Cu and Cu-Zr-Ti-Ni bulk metallic glasses were designed based on the d(c) and their d(c)values were predicted by the ANN model. Zr-Al-Ni-Cu and Cu-Zr-Ti-Ni bulk metallic glasses were prepared by injecting into copper mold. The amorphous structures and the determination of the d(c) of as-cast alloys were ascertained using X-ray diffraction. The results show that the predicted d(c) values of glass forming alloys are in agreement with the corresponding experimental values. Thus the developed ANN model is reliable and adequate for designing the composition and predicting the d(c) of glass forming alloy.
SN 1003-6326
EI 2210-3384
PD MAY
PY 2014
VL 24
IS 5
BP 1458
EP 1466
DI 10.1016/S1003-6326(14)63213-1
UT WOS:000339036700026
ER

PT J
AU Soubeyroux, JL
   Puech, S
   Donnadieu, P
   Blandin, JJ
AF Soubeyroux, J. L.
   Puech, S.
   Donnadieu, P.
   Blandin, J. J.
TI Synthesis and mechanical behavior of nanocomposite Mg-based bulk
   metallic glasses
SO JOURNAL OF ALLOYS AND COMPOUNDS
CT 12th International Symposium on Metastable and Nano-Materials
   (ISMANAM-2005)
CY JUL 03-07, 2005
CL Paris, FRANCE
AB Nanocomposites can be produced by partial crystallization of bulk metallic glasses (BMG). The introduction of such nanocrystals in an amorphous matrix can sharply change the mechanical behavior of the glass at room temperature. In the present study, the effect of crystals in Mg-based BMG is studied by partial crystallization of the Mg65Cu25Gd10 and addition of 3 at.% of iron. Various techniques (DSC, DRX, TEM) are used to get data about the nature, the volume fraction or the average size of the crystals. Mechanical properties at room temperature are investigated in compression. The fracture stress is enhanced by iron addition, but no favorable effect of partial crystallization is observed for the Mg65Cu25Gd10 BMG. These results are discussed in relation with the associated microstructures. (C) 2006 Elsevier B.V. All rights reserved.
SN 0925-8388
PD MAY 31
PY 2007
VL 434
SI SI
BP 84
EP 87
DI 10.1016/j.jallcom.2006.08.297
UT WOS:000246286900022
ER

PT J
AU Chen, ZH
   Zhang, LK
   Liu, JZ
   Chen, D
AF Chen ZhenHua
   Zhang LiKe
   Liu JiZi
   Chen Ding
TI Cryogenic treatment induced hardening for Cu-Zr-Ag-Al bulk metallic
   glasses
SO SCIENCE CHINA-TECHNOLOGICAL SCIENCES
AB Little is known about the mechanical properties of bulk metallic glassy alloys (BMGs) after cryogenic treatment. In this study, the effects of cryogenic treatment (CT) on the microstructural transition and the mechanical properties of Cu-based bulk metallic glass (BMG) were studied. The results showed that submicron AlCu2Zr and Cu5Zr phases can precipitate from the Cu-Zr-Ag-Al matrix after 192 h CT. The formation of these nanometer-sized crystalline phases substantially enhanced the mechanical properties of the bulk amorphous alloy. The micro-hardness and the compressive strength were found to increase respectively from 501 HV and 1510 MPa to 595 HV and 1910 MPa after cryogenic treatment. CT is a promising approach to strengthening metal glasses.
```

RI Liu, Jizi/N-8063-2016; 鼎, 陈/D-1695-2009; 陈(chen), 鼎(ding)/O-5087-2015

OI 鼎, 陈/0000-0001-6422-4597; 陈(chen), 鼎(ding)/0000-0003-0407-7542

```
SN 1674-7321
EI 1869-1900
PD MAR
PY 2013
VL 56
IS 3
BP 637
EP 641
DI 10.1007/s11431-012-5107-z
UT WOS:000314913900020
ER

PT J
AU Hu, Q
   Wang, JM
   Yan, YH
   Luo, FF
   Guo, S
   Liu, J
   Chen, SS
   Huang, B
   Lu, DP
   Zou, JZ
   Zeng, XR
AF Hu, Q.
   Wang, J. M.
   Yan, Y. H.
   Luo, F. F.
   Guo, S.
   Liu, J.
   Chen, S. S.
   Huang, B.
   Lu, D. P.
   Zou, J. Z.
   Zeng, X. R.
TI Anomalous thermal expansion in the deep super-cooled liquid region of a
   ZrCuAlAg bulk metallic glass
SO MATERIALS RESEARCH LETTERS
AB Bulk metallic glasses (BMGs), like other glasses, soften in the super-cooled liquid region (SCLR). Here in this work, surprisingly, a large expansion is reported occurring in the deep SCLR of a Zr47Cu37Al8Ag8 BMG. Nano-crystals (NCs) are found precipitated during the anomalous expansion of Zr47Cu37Al8Ag8, but also in the SCLR of Zr43Cu41Al8Ag8 that exhibits a conventional softening. It is found that there is a steep composition and density change at the NCs/amorphous matrix transition region in the former alloy, and this transition region exerts a thermal stress of about 0.15 MPa to the surrounding super-cooled liquid (SCL) and drives the soft SCL to expand severely.
   [GRAPHICS]
   .
RI Guo, Sheng/C-7746-2009
OI Guo, Sheng/0000-0001-8349-3135
SN 2166-3831
PY 2018
VL 6
IS 2
BP 121
EP 129
DI 10.1080/21663831.2017.1408713
UT WOS:000428141700002
ER

PT J
AU Gong, P
   Wang, SB
   Liu, Z
   Chen, W
   Li, N
   Wang, XY
   Yao, KF
AF Gong, Pan
   Wang, Sibo
   Liu, Ze
   Chen, Wen
   Li, Ning
   Wang, Xinyun
   Yao, K. F.
TI Lightweight Ti-based bulk metallic glasses with superior thermoplastic
   formability
SO INTERMETALLICS
AB The poor thermoplastic formability greatly restrains the wide application of Ti-based bulk metallic glasses. In this study, the thermoplastic formability of Ti-Zr-Be BMG has been dramatically improved by alloying method. The representative Ti41Zr25Be28Fe6 alloy exhibits a wide supercooled liquid region of similar to 123 K and low viscosity of similar to 10(6) Pa s in its supercooled liquid region, resulting in the best thermoplastic formability among all the developed Ti-based BMGs. Nanorods arrays can be successfully fabricated even in air with the help of a wetting layer. Moreover, the good glass-forming ability and excellent mechanical properties also make it applicable as engineering materials.
SN 0966-9795
EI 1879-0216
PD JUL
PY 2018
VL 98
BP 54
EP 59
DI 10.1016/j.intermet.2018.04.019
UT WOS:000435053700007
ER

PT J
AU Caris, J
   Lewandowski, JJ
AF Caris, J.
   Lewandowski, J. J.
TI Pressure effects on metallic glasses
SO ACTA MATERIALIA
AB The effects of normal stress/superimposed pressure on the flow/fracture of two different zirconium-based glasses and a hafnium-based bulk metallic glass (BMG) tested at Case Western Reserve University is presented. These results are compared with the pressure sensitivity of the bulk modulus and shear modulus of amorphous metals in the manner previously conducted by Spitzig and Richmond for crystalline metals. Although few experimental results of this nature exist, the data obtained to date reveal minimal normal stress/pressure sensitivity for the BMG examined in this study, consistent with the small pressure dependence of the shear modulus. Comparison with various yield/fracture criteria is also provided. (C) 2009 Acta Materialia Inc. Published by Elsevier Ltd. All rights reserved.
RI Lewandowski, John/S-3815-2017
OI Lewandowski, John/0000-0002-3389-2637
SN 1359-6454
PD FEB
PY 2010
VL 58
IS 3
BP 1026
EP 1036
DI 10.1016/j.actamat.2009.10.018
UT WOS:000274765600029
ER

PT J
AU Li, B
   Li, JS
   Fan, XH
   Chen, J
AF Li Bing
   Li Jinshan
   Fan Xinhui
   Chen Jian
TI Formation of Bulk Metallic Glasses from Cu-Zr Based Alloys with Yttrium
   Addition
SO RARE METAL MATERIALS AND ENGINEERING
AB Cu46Zr46Al8 bulk metallic glass (BMG) was prepared by low purity sponge zirconium instead of high purity crystal bar zirconium, and via the microalloying of adding a small amount of yttrium. The results show that the glass forming ability (GFA) and the thermal stability of the Cu46Zr46Al8 alloy are improved by yttrium addition. When the addition amount of Y is 2at% and 3at%, the enhancement of GFA is most significant. The compressive fracture strength of Y microalloyed BMG is more than 2 GPa, which is much higher than that of BMG alloy produced with high purity raw materials, but its plasticity deteriorates obviously.
OI Li, Jinshan/0000-0002-6894-9760
SN 1002-185X
PD JUL
PY 2014
VL 43
IS 7
BP 1558
EP 1561
DI 10.1016/S1875-5372(14)60124-7
UT WOS:000339783500005
ER

EF
```
