# Supplementary material for: Probabilistic Assessment of Glass Forming Ability Rules for Metallic Glasses Aided by Automated Analysis of Phase Diagrams
Source: Sci Rep. 2019 Jan 23;9:357. doi: 10.1038/s41598-018-36224-3 (PMC6344582; doi:10.1038/s41598-018-36224-3)
Supplement: Supplementary file 3 [file 41598_2018_36224_MOESM3_ESM.docx]

FN Clarivate Analytics Web of Science

VR 1.0

PT J

AU Li, FC

Xie, Y

Gu, J

Song, M

Ni, S

Guo, SF

Liao, XZ

AF Li, Fucheng

Xie, Ya

Gu, Ji

Song, Min

Ni, Song

Guo, Shengfeng

Liao, Xiaozhou

TI Inhomogeneous creep deformation in metallic glasses

SO MATERIALS SCIENCE AND ENGINEERING A-STRUCTURAL MATERIALS PROPERTIES

MICROSTRUCTURE AND PROCESSING

AB Homogeneous creep is a commonly observed phenomenon in bulk metallic glasses. Here we reported inhomogeneous creep behavior that occurs under nanoindentation when the applied stress exceeds the yield stress. Extensive investigation showed that inhomogeneous creep is associated with the local microstructure and the operation of shear bands before creep. The mechanism responsible for inhomogeneous creep is discussed. (C) 2015 Elsevier B.V. All rights reserved.

RI Guo, Shengfeng/E-3171-2012; Song, Min/C-3730-2013; Liao,

Xiaozhou/B-3168-2009

OI Guo, Shengfeng/0000-0002-6667-6797; Song, Min/0000-0002-3197-4647; Liao,

Xiaozhou/0000-0001-8565-1758

SN 0921-5093

EI 1873-4936

PD NOV 11

PY 2015

VL 648

BP 57

EP 60

DI 10.1016/j.msea.2015.09.048

UT WOS:000363820700010

ER

PT J

AU Ito, H

Yamamoto, T

Hasegawa, M

Inoue, A

AF Ito, Hirofumi

Yamamoto, Tokujiro

Hasegawa, Masashi

Inoue, Akihisa

TI Effect of nanocrystal distribution on mechanical properties of ti-based

metallic glasses

SO MATERIALS TRANSACTIONS

AB Ti45Zr5CU45Ni5 metallic glasses in which Ta and Al were substituted for Cu were evaluated in terms of mechanical properties, thermal properties and microstructures in order to determine the factors contributing to an improvement in plasticity. Samples are examined by compression testing, differential scanning calorimetry, X-ray diffractorrietry and electron microscopy. Mold-cast Ti45Zr5Cu44Ni5Ta1 bulk specimens were confirmed to consist of a metallic glass matrix and nanocrystals homogeneously dispersed at high density within the matrix. The yield stresses of both Ti45Zr5CU45-xNi5Tax, and Ti45Zr5CU45-xNi5Al, are approximately 1800MPa, and the maximum plastic strain of 3.1% was obtained for the Ti45Zr5CU44Ni5Ta1 specimen. The Ti(45)Zr(5)CU(45-x)Ni5Al(x) bulk specimens exhibited poorer plasticity due to the formation of larger crystalline grains.

RI Inoue, Akihisa/E-5271-2015; Yamamoto, Tokujiro/A-8827-2011

OI Yamamoto, Tokujiro/0000-0002-4292-9446

SN 1345-9678

EI 1347-5320

PD JUN

PY 2007

VL 48

IS 6

BP 1288

EP 1291

DI 10.2320/matertrans.MF200616

UT WOS:000247823300024

ER

PT B

AU Dai, LH

AF Dai, Lan Hong

BE Dodd, B

Bai, Y

TI Shear Banding in Bulk Metallic Glasses

SO ADIABATIC SHEAR LOCALIZATION: FRONTIERS AND ADVANCES, 2ND EDITION

SE Elsevier Insights

OI Dai, LanHong/0000-0001-8991-0358

BN 978-0-08-098200-7; 978-0-08-097781-2

PY 2012

BP 311

EP 361

DI 10.1016/B978-0-08-097781-2.00008-3

UT WOS:000315724200009

ER

PT J

AU Zhang, XC

Du, YL

Guang, C

Chen, GL

AF Zhang, XC

Du, YL

Guang, C

Chen, GL

TI Improving room temperature ductility of bulk metallic glasses by

introducing second phase in the amorphous matrix

SO RARE METAL MATERIALS AND ENGINEERING

AB Bulk metallic glasses have unique mechanical properties, including high strengths (similar to 2 GPa), large elastic limits (2%similar to 3%), and excel lent resistance to corrosion, but the poor room temperature ductility restricts its applications as structural materials. In this paper, the room temperature brittleness of bulk metallic glasses was discussed based on the deformation and fracture mechanism, and the progress in improving room temperature ductility by introducing second phase in the amorphous matrix was summarized.

SN 1002-185X

PD APR

PY 2006

VL 35

IS 4

BP 510

EP 515

UT WOS:000237365900002

ER

PT J

AU An, WK

Ding, DW

Cai, AH

Zhou, GJ

Luo, Y

Li, JH

Peng, YY

AF An WeiKe

Ding DaWei

Cai AnHui

Zhou GuoJun

Luo Yun

Li JiangHong

Peng YongYi

TI Mechanism, condition and characteristics for the formation of the

network structure in Zr-Al-Ni-Cu bulk metallic glasses

SO SCIENCE CHINA-PHYSICS MECHANICS & ASTRONOMY

AB Mechanism, condition and characteristics for the formation of the network structure in a group of Zr-Al-Ni-Cu bulk metallic glasses (BMGs) were investigated. The results show that the constituent segregation and/or the symplastic growth would be the mechanisms for the formation of the cell structure in the present Zr-Al-Ni-Cu BMGs. The cell structure can be easily obtained for the glass forming alloys whose compositions locate nearby the eutectic point. The shorter the distance is from the eutectic point, the larger the cell and the thicker the cell wall of the network structure will be. The present investigation would provide useful information for the development of the BMG with the network structure.

SN 1674-7348

EI 1869-1927

PD JUN

PY 2015

VL 58

IS 6

AR 066101

DI 10.1007/s11433-014-5602-2

UT WOS:000355241100009

ER

PT J

AU Zhu, ZD

Jia, P

Xu, JA

AF Zhu, Zhen-dong

Jia, Peng

Xu, Jian

TI Optimization for toughness in metalloid-free Ni-based bulk metallic

glasses

SO SCRIPTA MATERIALIA

AB The toughness of metalloid-free Ni-based bulk metallic glasses (BMGs) manifests a concentration dependency. In terms of measured notch toughness, Ni(40)Cu(2)Pd(2)Zr(27.6)Ti(18.4)Al(10) BMG exhibits the optimal toughness among the six investigated alloys. Its fracture energy is comparable to Fe-based BMGs with optimal toughness. In contrast to the Poisson's ratio, the shear modulus is much more sensitive to composition change in the Ni-based BMGs. Improvement in toughness of Ni-based BMGs roughly scales with reducing the shear modulus or glass transition temperature. (C) 2011 Acta Materialia Inc. Published by Elsevier Ltd. All rights reserved.

SN 1359-6462

PD APR

PY 2011

VL 64

IS 8

BP 785

EP 788

DI 10.1016/j.scriptamat.2010.12.047

UT WOS:000287908300024

ER

PT S

AU Axinte, E

Nastase, CM

Barnea, A

Axinte, L

AF Axinte, Eugen

Nastase, Corneliu Marius

Barnea, Alexandru

Axinte, Lorica

BE Nedelcu, D

Slatineanu, L

Mazuru, S

Milocevic, O

TI DEVELOPMENTS IN DESIGN AND MANUFACTURE OF BULK METALLIC GLASSES (BMGs)

-AN INTRODUCTION IN THE STATE OF THE ART

SO MODTECH 2011: NEW FACE OF T.M.C.R., VOL I AND II

SE International Conference ModTech Proceedings

CT 15th International Conference of Modern Technoliges, Quality and

Innovation

CY MAY 25-27, 2011

CL Vadul lui Voda, MOLDOVA

SP Redesign Solutions Iasi, Profess Assoc Modern Mfg Technol, Patis, Serbian Acad Sci & Arts, Inst Tech Sci, Gheorghe Asachi Tech Univ Iasi Romania, Fac Machine Mfg & Ind Management, Dept Machine Mfg Technol, Tech Univ Moldova Chisinau, Dept Machine Mfg Technol

AB This paper is an introduction in the state of the art in the relatively new domain of metallic glasses. Bulk metallic glasses (BMGs) are metallic materials with a disordered atomic-scale structure, produced directly from the liquid state during cooling. The rapid cooling, on the order of millions of degrees a second, is too fast for crystals to form and the material is "locked in" a glassy state. BMGs have been paid great attentions for its theoretical and practical reasons, since 1974, when the bulk amorphous Palladium based alloys (Pd-Cu-Si and Pd-Ni-P) were first synthesized by water quenching method. More recently, batches of amorphous steel have been produced that demonstrate strengths much greater than conventional steel alloys

SN 2069-6736

PY 2011

BP 45

EP 48

UT WOS:000392260500012

ER

PT J

AU Zhang, S

Dong, DD

Wang, ZJ

Dong, C

Haussler, P

AF Zhang, Shuang

Dong, Dandan

Wang, Zijian

Dong, Chuang

Haeussler, Peter

TI Composition formulas of Ni-(Nb, Ta) bulk metallic glasses

SO INTERMETALLICS

AB It is known that bulk metallic glass compositions can be well interpreted by cluster formulas for stable liquids [cluster](glue atom) 1 or 3, where the clusters are derived from relevant devitrification phases. In the present work, the glass forming composition formulas in Ni-Nb and Ni-Ta systems are fully examined. The procedures include choosing the appropriate eutectic crystalline phases, selecting the principal clusters via the criteria such as spherical periodicity and cluster isolation degree, and determining the glue atoms. After strictly following these procedures, the best glass forming compositions Ni62Nb38 in Ni-Nb system and the range 59-62 at.% Ni in Ni-Ta system are explained by formulas [Ni-Nb4Ni8] Nb2Ni and [M-Ni6Ta6] Ni-3 (M is a random mixture of Ni and Ta) respectively. (C) 2017 Elsevier Ltd. All rights reserved.

SN 0966-9795

EI 1879-0216

PD JUN

PY 2017

VL 85

BP 176

EP 179

DI 10.1016/j.intermet.2017.02.019

UT WOS:000399381200024

ER

PT J

AU Huo, LS

Ma, J

Ke, HB

Bai, HY

Zhao, DQ

Wang, WH

AF Huo, L. S.

Ma, J.

Ke, H. B.

Bai, H. Y.

Zhao, D. Q.

Wang, W. H.

TI The deformation units in metallic glasses revealed by stress-induced

localized glass transition

SO JOURNAL OF APPLIED PHYSICS

AB We report that even in quasi-static cyclic compressions in the apparent elastic regimes of the bulk metallic glasses, the precisely measured stress-strain curve presents a mechanical hysteresis loop, which is commonly perceived to occur only in high-frequency dynamic tests. A phenomenological viscoelastic model is established to explain the hysteresis loop and demonstrate the evolutions of the viscous zones in metallic glasses during the cyclic compression. The declining of the viscosity of the viscous zones to at least 1 x 10(12) Pa s when stress applied indicates that stress-induced localized glass to supercooled liquid transition occurs. We show that the deformation units of metallic glasses are evolved from the intrinsic heterogeneous defects in metallic glasses under stress and the evolution is a manifestation of the stress-induced localized glass transition. Our study might provide a new insight into the atomic-scale mechanisms of plastic deformation of metallic glasses. (C) 2012 American Institute of Physics. [http://dx.doi.org/10.1063/1.4728207]

SN 0021-8979

EI 1089-7550

PD JUN 1

PY 2012

VL 111

IS 11

AR 113522

DI 10.1063/1.4728207

UT WOS:000305401400070

ER

PT J

AU Lan, S

Wu, ZD

Wei, XY

Zhou, J

Lu, ZP

Neuefeind, J

Wang, XL

AF Lan, Si

Wu, Zhenduo

Wei, Xiaoya

Zhou, Jie

Lu, Zhaoping

Neuefeind, Joerg

Wang, Xun-Li

TI Structure origin of a transition of classic-to-avalanche nucleation in

Zr-Cu-Al bulk metallic glasses

SO ACTA MATERIALIA

AB Crystallization kinetics of a series of Zr-Cu-Al bulk metallic glasses with different glass-forming abilities were systematically investigated using differential scanning calorimetry, time-resolved neutron diffraction, and high-resolution transmission electron microscopy. Experimental results revealed a transition of classic-to-avalanche types of nucleation for the Zr-Cu-Al glass alloys upon isothermal annealing. The classic mode is characterized by a continuous nucleation and growth of Zr2Cu-type crystals. The avalanche mode features an abrupt formation of massive Cu10Zr7-type precipitates with a complex, low-symmetry crystalline structure. Calorimetric measurements identified an anomalous exothermic peak prior to crystallization for metallic glasses with the Cu10Zr7-type crystalline phase. The formation of a metastable amorphous/semi-crystalline phase was observed using the electron microscopy prior to crystallization for glasses with an anomalous exothermic event. Our results indicate that the formation of a metastable phase might lower the free energy barrier of the crystallization and trigger an avalanche type of nucleation. Neutron diffraction pair distribution function analysis suggests that the easy connection of short-range clusters at the medium-range length scale would favor the avalanche nucleation. (C) 2018 Acta Materialia Inc. Published by Elsevier Ltd. All rights reserved.

RI Lu, Zhao-Ping/A-2718-2009

OI Lu, Zhao-Ping/0000-0003-1463-8948; Lan, Si/0000-0002-3104-4909; Wang,

Xun-Li/0000-0003-4060-8777

SN 1359-6454

EI 1873-2453

PD MAY 1

PY 2018

VL 149

BP 108

EP 118

DI 10.1016/j.actamat.2018.02.028

UT WOS:000430895000011

ER

PT J

AU Tong, Y

Dmowski, W

Yokoyama, Y

Wang, G

Liaw, PK

Egami, T

AF Tong, Y.

Dmowski, W.

Yokoyama, Y.

Wang, G.

Liaw, P. K.

Egami, T.

TI Recovering compressive plasticity of bulk metallic glasses by

high-temperature creep

SO SCRIPTA MATERIALIA

AB Most bulk metallic glasses fail mechanically in a brittle manner, without much plasticity. Annealing at a temperature below the glass transition temperature typically results in structural relaxation and even more enhanced brittleness. However, we report here that significant plasticity can be recovered if the sample is subjected to stress during annealing, resulting in thermomechanical creep. The structural analysis indicates that the high-temperature creep alleviates the effect of the structural relaxation and thus leads to structural rejuvenation and improved plasticity. (C) 2013 Acta Materialia Inc. Published by Elsevier Ltd. All rights reserved.

RI Yokoyama, Yoshihiko/A-8603-2011

SN 1359-6462

PD OCT

PY 2013

VL 69

IS 8

BP 570

EP 573

DI 10.1016/j.scriptamat.2013.06.020

UT WOS:000324456000002

ER

PT J

AU Shahabi, HS

Scudino, S

Kuhn, U

Eckert, J

AF Shahabi, H. Shakur

Scudino, S.

Kuehn, U.

Eckert, J.

TI Metallic glass-steel composite with improved compressive plasticity

SO MATERIALS & DESIGN

AB A Zr52.5CU18Ni14.5Al10Ti5 bulk metallic glass toughened with a commercially available spring-shaped steel wire has been produced by centrifugal casting. The addition of the steel spring significantly affects shear band nucleation and propagation through the blockage, deflection and multiplication of shear bands at the glass-spring interface. As a result of the more homogeneous distribution of the plastic strain, the room temperature plasticity increases from 0.9% for the monolitic glass to about 4% for the glass-spring composite. Given the low volume fraction of the spring used in the composite (4.2 vol.%), these results demonstrate the extreme effectiveness of the steel spring for improving the plasticity of the metallic glass. (C) 2014 Elsevier Ltd. All rights reserved.

RI Scudino, Sergio/D-8049-2015; Shakur Shahabi, Hamed/B-4380-2010

OI Shakur Shahabi, Hamed/0000-0002-1872-3739

SN 0261-3069

EI 1873-4197

PD JUL

PY 2014

VL 59

BP 241

EP 245

DI 10.1016/j.matdes.2014.03.007

UT WOS:000336456500028

ER

PT J

AU Zhang, LC

Jiang, F

Zhang, DH

He, L

Sun, J

Fan, JT

Zhang, ZF

AF Zhang, Lincai

Jiang, Feng

Zhang, Dehong

He, Lin

Sun, Jun

Fan, Jitang

Zhang, Zhefeng

TI In-Situ Precipitated Nanocrystal Beneficial to Enhanced Plasticity of

Cu-Zr Based bulk Metallic Glasses

SO ADVANCED ENGINEERING MATERIALS

RI jiang, Feng/D-1427-2012; Zhang, Zhefeng/A-9732-2010; Zhang,

BMG/C-6151-2014

OI jiang, Feng/0000-0003-3651-8007;

SN 1438-1656

PD OCT

PY 2008

VL 10

IS 10

BP 943

EP 950

DI 10.1002/adem.200800123

UT WOS:000261031600006

ER

PT J

AU Liu, GB

Gao, P

Xue, Z

Tong, ZQ

Zhang, ML

AF Liu, Guangbo

Gao, Peng

Xue, Zhu

Tong, Zhongqiu

Zhang, Milin

TI Ultra-high strength Mg-Li based bulk metallic glasses: Preparation and

performance research

SO MATERIALS SCIENCE AND ENGINEERING A-STRUCTURAL MATERIALS PROPERTIES

MICROSTRUCTURE AND PROCESSING

AB In this paper, new Mg-Li based bulk metallic glasses (BMGs) are prepared by conventional copper mold injection casting method. The alloys exhibit excellent mechanical properties, such as ultra-high compressive fracture strength (maximal 729 MPa), high Vickers hardness (>2 GPa) and low elastic modulus (similar to 35 GPa). Compared with the corresponding crystal alloys, the density of the amorphous alloy samples is reduced by about 1.5% due to their free volume. Thus, it is believed that this new BMGs with these outstanding properties will broaden Mg-Li based alloys' application fields. (C) 2011 Elsevier B.V. All rights reserved.

SN 0921-5093

PD SEP 15

PY 2011

VL 528

IS 24

BP 7156

EP 7160

DI 10.1016/j.msea.2011.06.012

UT WOS:000294091900006

ER

PT J

AU Gao, Q

Jian, ZY

Xu, JF

AF Gao, Qian

Jian, Zengyun

Xu, Junfeng

TI Correlation between glass transition temperature and

Vogel-Fulcher-Tamman temperature in amorphous alloys

SO JOURNAL OF ALLOYS AND COMPOUNDS

AB The correlation between the glass transition temperature T-g and the Vogel-Fulcher-Tamman temperature T-0 in metallic glasses is investigated. The linear relationships, T-0 = 0.897T(g)-100.9, are discovered. Amorphous alloys with desired the Vogel-Fulcher-Tamman temperature T-0 can be predicted and designed by the linear relationship. (C) 2018 Elsevier B.V. All rights reserved.

SN 0925-8388

EI 1873-4669

PD APR 25

PY 2018

VL 742

BP 280

EP 283

DI 10.1016/j.jallcom.2018.01.193

UT WOS:000427505800036

ER

PT J

AU Vatamanu, LO

Lewandowski, JJ

AF Vatamanu, Luciano O.

Lewandowski, John J.

TI Pressure and temperature effects on tensile strength and plasticity of

metallic glasses

SO MECHANICS OF MATERIALS

AB The effects of superimposed hydrostatic pressure and test temperature on the strength and plasticity of two different bulk metallic glasses have been determined. The effects of superimposed hydrostatic pressure on the global tensile strength and plasticity at test temperatures well below the Tg of the metallic glass are negligible, consistent with the minor effects of superimposed pressure on the elastic constants at these temperatures. However, increasing the test temperature produces tensile strength reductions and an increase in the global plasticity at 0.1 MPa. Superposition of hydrostatic pressure produces significant increases in strength and reduced ductility at temperatures approaching Tg for both a Zr-based and La-based bulk metallic glass. Potential source(s) of the pressure-induced changes in strength and global plasticity of the bulk metallic glasses are provided, and relate to pressure-induced increases in viscosity near Tg. (C) 2012 Elsevier Ltd. All rights reserved.

RI Lewandowski, John/S-3815-2017

OI Lewandowski, John/0000-0002-3389-2637

SN 0167-6636

EI 1872-7743

PD DEC

PY 2013

VL 67

SI SI

BP 86

EP 93

DI 10.1016/j.mechmat.2012.11.011

UT WOS:000327687500014

ER

PT J

AU Hsu, CF

Kai, W

Lin, HM

Lin, CK

Lee, PY

AF Hsu, Chih-Feng

Kai, Wu

Lin, Hong-Ming

Lin, Chung-Kwei

Lee, Pee-Yew

TI Fabrication and corrosion behavior of Ti-based bulk metallic glass

composites containing carbon nanotubes

SO JOURNAL OF ALLOYS AND COMPOUNDS

CT 16th International Symposium on Metastable, Amorphous and Nanostructured

Materials

CY JUL 05-09, 2009

CL Beijing, PEOPLES R CHINA

AB This study explored the feasibility of preparing Ti50Cu28Ni15Sn7 bulk metallic glass composite with powder metallurgy. With high energy ball milling of a pure Ti, Cu, Ni, Sn and carbon nanotube (CNT) powder mixture, the CNT/Ti50Cu28Ni15Sn7 metallic glass composite powders can be formed with mechanical alloying ( MA) after 8 h milling. The bulk metallic glass composite was successfully prepared by vacuum hot pressing the as-milled CNT/Ti50Cu28Ni15Sn7 metallic glass composite powders. The corrosion behavior of the Ti50Cu28Ni15Sn7 bulk metallic glass as well as composites modified by the addition of CNT was investigated by electrochemical measurements. Electrochemical characterization was performed in Hanks' solution at 37 degrees C with physiologically relevant dissolved oxygen content. The results of potentio-dynamic polarization measurements revealed that the Ti-based bulk metallic glass composites examined showed spontaneous passivity by anodic polarization with a passive current density of about 10(-5) A/cm(2). The higher corrosion resistance of the Ti-based bulk metallic glass composites was attributed to stable and protective passive films enriched with titanium containing certain amounts of additional elements. (C) 2010 Elsevier B.V. All rights reserved.

RI BAI, JIE/D-7448-2016

SN 0925-8388

PD AUG

PY 2010

VL 504

SU 1

BP S176

EP S179

DI 10.1016/j.jallcom.2010.02.061

UT WOS:000285252600045

ER

PT J

AU Kobelev, NP

Khonik, VA

Afonin, GV

Kolyvanov, EL

AF Kobelev, N. P.

Khonik, V. A.

Afonin, G. V.

Kolyvanov, E. L.

TI On the origin of the shear modulus change and heat release upon

crystallization of metallic glasses

SO JOURNAL OF NON-CRYSTALLINE SOLIDS

AB It has been shown that the changes of the internal energy and elastic moduli occurring upon crystallization of metallic glasses can be explained under the assumption that the initial glassy structure contains "defects" similar to elastic dipoles in crystals. These "defects" define the elastic softening of glass and disappear upon crystallization so that their elastic energy is released as heat. The experimental verification of this model has been performed and characteristic parameters of elastic dipoles in a Zr-based bulk metallic glass have been determined. (C) 2014 Elsevier B.V. All rights reserved.

RI Afonin, Gennady/K-1014-2013; Khonik, Vitaly/A-5888-2009; Makarov,

Andrey/H-4156-2013

OI Afonin, Gennady/0000-0002-7715-5065; Makarov, Andrey/0000-0001-6741-0619

SN 0022-3093

EI 1873-4812

PD MAR 1

PY 2015

VL 411

BP 1

EP 4

DI 10.1016/j.jnoncrysol.2014.11.039

UT WOS:000349726100001

ER

PT J

AU Xu, HW

Du, YL

Deng, Y

AF Xu Hong-wei

Du Yu-lei

Deng Yu

TI Effect of minor-addition of Fe on structural and mechanical properties

of CuZrAl bulk metallic glass

SO TRANSACTIONS OF NONFERROUS METALS SOCIETY OF CHINA

AB The CuZrAl bulk metallic glass with minor-addition of Fe was prepared by rapid quenching method. The structures were examined by X-ray diffraction (XRD). The effect of Fe on the glass-forming ability was studied by differential scanning calorimetry (DSC). The minor-addition of Fe obviously extends the supercooled liquid region Delta T-x. The plastic strain of the Cu44Zr48Al7Fe bulk metallic glass is about 1.5%. The microstructures were examined by transmission electron microscopy (TEM). It is found that when 1%-2% Fe (mole fraction) were introduced into the CuZrAl alloy matrix, nanoscale phase separation occurs in the as-prepared Cu44Zr48Al7Fe bulk metallic glass.

SN 1003-6326

EI 2210-3384

PD MAY

PY 2012

VL 22

IS 5

BP 1123

EP 1126

DI 10.1016/S1003-6326(11)61293-4

UT WOS:000305087000019

ER

PT J

AU Wu, XF

Zhang, HF

Hu, ZQ

AF Wu, XF

Zhang, HF

Hu, ZQ

TI Deformation behaviores and mechanical properties of tungsten fiber

reinforced Zr-based bulk metallic amorphous matrix composite containing

Co

SO RARE METAL MATERIALS AND ENGINEERING

AB Tungsten fiber reinforced Zr-Ti-Cu-Be-Co bulk metallic amorphous matrix composite was fabricated by a pressure infiltrating cast method. The deformation behaviors and mechanical properties were investigated. The results indicated that Tungsten fiber reinforced bulk metallic glass matrix composite not only holds high strength of pure Zr-Ti-Cu-Be-Co bulk metallic glass, but also exhibits the strain of more than 900% higher than those of pure Zr-Ti-Cu-Be-Co bulk metallic glass. The compressive failure mode of the composite shifts from shear to the localized fiber buckling and tilting :with the increase of the volume fraction of tungsten fibers. The increase in compressive toughness comes from the fibers restricting shear band propagation, promoting the generation of multiple shear bands.

SN 1002-185X

PD JUN

PY 2005

VL 34

IS 6

BP 863

EP 866

UT WOS:000230154000006

ER

PT J

AU Zhang, J

Tan, H

Feng, YP

Li, Y

AF Zhang, J

Tan, H

Feng, YP

Li, Y

TI The effect of Y on glass forming ability

SO SCRIPTA MATERIALIA

AB We have discovered a new glass forming region (GFR) defined by melt-spinning at a speed of 30 m/s in a ternary Fe-Fe2BFe4B4Y eutectic in the Fe-B-Y alloy system. This GFR is remarkably wide, within the compositional range 19-40 at.% B, 3 to similar to 10 at.% Y and 76-50 at.% Fe, with the best glass former being Fe71.2B24Y4.8 for a I mm fully amorphous rod. The effect of Y content on the glass forming ability (GFA) is perhaps primarily due to the characteristic of the ternary triangle, and Y in the case of Fe-B-Y system should be treated as a base element. (c) 2005 Acta Materialia Inc. Published by Elsevier Ltd. All rights reserved.

RI Feng, Yuan Ping/A-4507-2012

OI Feng, Yuan Ping/0000-0003-2190-2284

SN 1359-6462

PD JUL

PY 2005

VL 53

IS 2

BP 183

EP 187

DI 10.1016/j.scriptamat.2005.03.042

UT WOS:000229689200008

ER

PT J

AU Wen, P

Wang, RJ

Pan, MX

Zhao, DQ

Wang, WH

AF Wen, P

Wang, RJ

Pan, MX

Zhao, DQ

Wang, WH

TI Characteristics of microstructure and glass transition of

(Zr0.59Ti0.06Cu0.22Ni0.13)(100-x)Al-x bulk metallic glasses

SO JOURNAL OF APPLIED PHYSICS

AB The glass transition and the microstructural characteristics of (Zr0.59Ti0.06Cu0.22Ni0.13)(100-x)Al-x (10less than or equal toxless than or equal to16) bulk metallic glasses (BMG) are investigated as function of Al content. It is found that the Al content has strong effects on the glass transition, microstructure, and properties of the alloy. The marked increase of the Debye temperature and shear modulus with increasing Al content indicates that the different Al incorporations result in microstructural change in atomic short range of the BMG. The increase of the glass transition temperature with the increase Al content is consistent with the large variation of properties with increase of Al content. The change of the microstructure of the glass-forming alloy is responsible for the change of the properties. (C) 2003 American Institute of Physics.

SN 0021-8979

PD JAN 1

PY 2003

VL 93

IS 1

BP 759

EP 761

DI 10.1063/1.1526153

UT WOS:000180002500116

ER

PT J

AU Liu, YH

Wang, K

Inoue, A

Sakurai, T

Chen, MW

AF Liu, Y. H.

Wang, K.

Inoue, A.

Sakurai, T.

Chen, M. W.

TI Energetic criterion on the intrinsic ductility of bulk metallic glasses

SO SCRIPTA MATERIALIA

AB We report the use of an energetic criterion to assess the deformability of glassy alloys based on the cooperative shearing model and energy landscape theory. The barrier energy density necessary for the activation of shear transformation zones and the initiation of shear bands shows a strong correlation with the ductility of bulk metallic glasses. This finding directly ties the ductility of metallic glasses to the forming ability of shear bands and offers an energetic insight into the mechanical behavior of metallic glasses. (C) 2009 Acta Materialia Inc. Published by Elsevier Ltd. All rights reserved.

RI Wang, Ke/M-7171-2017; LIU, Yanhui/B-1485-2009; CHEN,

Mingwei/A-4855-2010; Wang, Ke/C-8021-2011; Inoue, Akihisa/E-5271-2015

OI Wang, Ke/0000-0002-1174-0907; CHEN, Mingwei/0000-0002-8274-3099; Chen,

Mingwei/0000-0002-2850-8872

SN 1359-6462

PD APR

PY 2010

VL 62

IS 8

BP 586

EP 589

DI 10.1016/j.scriptamat.2009.12.042

UT WOS:000275972200016

ER

PT J

AU Choi-Yim, H

Johnson, WL

AF Choi-Yim, H

Johnson, WL

TI Bulk metallic glass matrix composites

SO APPLIED PHYSICS LETTERS

AB Composites with a bulk metallic glass matrix were synthesized and characterized. This was made possible by the recent development of bulk metallic glasses that exhibit high resistance to crystallization in the undercooled liquid state. In this letter, experimental methods for processing metallic glass composites are introduced. Three different bulk metallic glass forming alloys were used as the matrix materials. Both ceramics and metals were introduced as reinforcement into the metallic glass. The metallic glass matrix remained amorphous after adding up to a 30 vol% fraction of particles or short wires, X-ray diffraction patterns of the composites show only peaks from the second phase particles superimposed on the broad diffuse maxima from the amorphous phase. Optical micrographs reveal uniformly distributed particles in the matrix. The glass transition of the amorphous matrix and the crystallization behavior of the composites were studied by calorimetric methods. (C) American Institute of Physics. [S0003-6951(97)02852-0].

SN 0003-6951

PD DEC 29

PY 1997

VL 71

IS 26

BP 3808

EP 3810

DI 10.1063/1.120512

UT WOS:000071184700021

ER

PT J

AU Cao, HB

Ma, D

Hsieh, KC

Ding, L

Stratton, WG

Voyles, PM

Pan, Y

Cai, MD

Dickinson, JT

Chang, YA

AF Cao, Hongbo

Ma, Dong

Hsieh, Ker-Chang

Ding, Ling

Stratton, William G.

Voyles, Paul M.

Pan, Ye

Cai, Mingdong

Dickinson, J. Thomas

Chang, Y. Austin

TI Computational thermodynamics to identify Zr-Ti-Ni-Cu-Al alloys with high

glass-forming ability

SO ACTA MATERIALIA

AB We have used a thermodynamic computational approach to identify the compositions of Zr-Ti-Ni-Cu-Al alloys exhibiting low-lying liquidus surfaces, which tend to favor the formation of bulk metallic glasses. Guided by these calculations, we have identified several series of new Zr-based alloys with excellent glass formability, some of which can be cast into glassy rods up to 14 mm in diameter. These alloys exhibit wide undercooled liquid regions (T-x-T-g) up to 85 K and high Vickers hardness from 550 to 700 kg/mm(2). The best glass-forming alloy is Zr51Ti5Ni10Cu25Al9. The computational thermodynamic approach coupled with the reduced glass transition temperature criterion of Turnbull can rapidly identify regions of alloy composition suitable for experimental tests for glass formation. The glass-forming ability of the alloys we studied can be understood in terms of the relative liquidus temperature in a thermodynamically calculated temperature vs. composition section through a multicomponent phase diagram. It does not follow several other proposed thermodynamic or topological criteria. Our general approach may be extended into a universal too] to identify alloys with good potential to form bulk glasses. (c) 2006 Acta Materialia Inc. Published by Elsevier Ltd. All rights reserved.

RI Ma, Dong/G-5198-2011

OI Ma, Dong/0000-0003-3154-2454; Voyles, Paul/0000-0001-9438-4284

SN 1359-6454

PD JUN

PY 2006

VL 54

IS 11

BP 2975

EP 2982

DI 10.1016/j.actamat.2006.02.051

UT WOS:000238468100009

ER

PT J

AU Loffler, JF

AF Loffler, JF

TI Recent progress in the area of bulk metallic glasses

SO ZEITSCHRIFT FUR METALLKUNDE

AB Bulk metallic glasses (BMGs) form a new class of alloys which can be manufactured amorphously at a cooling rate of less than 100 K/s and in dimensions of several centimeters. Because of their particular characteristics, these glassy materials have great potential for deployment as structural and functional materials. The mechanical, magnetic, and thermophysical properties of this new materials group and the advantages they promise for the processing and manufacturing of components are presented. The possibilities for deploying bulk metallic glasses in medical devices and lightweight construction are also set out. In these two areas, we discuss some results as to the biocompatibility of metallic glasses and their potential use in vascular intervention, as well as the properties of light-metal-based bulk metallic glasses in connection with their potential as a construction material. We also present ideas concerning the development of corrosion-resistant Mg-based glasses, and the manufacture and processing of light-metal-based composites.

SN 0044-3093

PD MAR

PY 2006

VL 97

IS 3

BP 225

EP 233

UT WOS:000236671700006

ER

PT J

AU Sarac, B

Schroers, J

AF Sarac, B.

Schroers, J.

TI From brittle to ductile: Density optimization for Zr-BMG cellular

structures

SO SCRIPTA MATERIALIA

AB Two-dimensional cellular structures made from Zr35Ti30Cu7.5Be27.5 bulk metallic glass with varying relative densities realized in hexagonal structures ranging from 2.5% to 86.0% were fabricated and characterized under quasi-static compression loading. Three distinctive deformation regions were revealed: collective buckling, local failure and global failure, which originate from size effects in metallic glasses and demonstrate the ideal density of similar to 25.0% for energy absorption for this structure. Published by Elsevier Ltd. on behalf of Acta Materialia Inc.

RI Sarac, Baran/N-3706-2015

OI Sarac, Baran/0000-0002-0130-3914

SN 1359-6462

PD JUN

PY 2013

VL 68

IS 12

BP 921

EP 924

DI 10.1016/j.scriptamat.2013.02.030

UT WOS:000318584700003

ER

PT J

AU Jia-Yuang, G

Yu-Feng, S

Li-Guo, W

Shi-Jie, Z

Li-Zhao, L

Shao-Kang, G

AF Geng Jia-yuang

Sun Yu-feng

Wang Li-guo

Zhu Shi-jie

Liu Li-zhao

Guan Shao-kang

TI Effect of A1 addition on formation and mechanical properties of Mg-Cu-Gd

bulk metallic glass

SO TRANSACTIONS OF NONFERROUS METALS SOCIETY OF CHINA

AB The effect of partial substitution of Al for Cu on the glass forming ability(GFA) and mechanical proper-ties of Mg65Cu25-xAlxGd10 (x=0, 1, 3 and 5, molar fraction, %) alloys were studied by X-ray diffractometry(XRD), differential scanning calorimetry(DSC) and uniaxial compression test. The result reveals that GFA of the alloys changes slightly with increasing x from 0 to 3, and then abruptly decreases with x increasing up to 5. The plasticity can be greatly improved with appropriate substitution of Cu by Al (3%, molar fraction) in Mg65Cu25Gd10 bulk metallic glass, and the resultant fracture strength, total strain to failure, and plastic strain are 898 MPa, 2.19% and 0.2%, respectively.

SN 1003-6326

EI 2210-3384

PD OCT

PY 2007

VL 17

IS 5

BP 907

EP 912

UT WOS:000250788400005

ER

PT J

AU Zander, D

Heisterkamp, B

Gallino, I

AF Zander, Daniela

Heisterkamp, Beate

Gallino, Isabella

TI Corrosion resistance of Cu-Zr-Al-Y and Zr-Cu-Ni-Al-Nb bulk metallic

glasses

SO JOURNAL OF ALLOYS AND COMPOUNDS

CT 12th International Symposium on Metastable and Nano-Materials

(ISMANAM-2005)

CY JUL 03-07, 2005

CL Paris, FRANCE

AB Electrochemical tests of amorphous Cu46Zr42Al7Y5 in comparison to amorphous Zr58.5Cu15.6Ni12.8Al10.3Nb2.8 (Vit106a) were conducted by potentiodynamic polarization at room temperature in 0.001-0.1 MNaC1(aq) (pH 8). The influence of corrosion on the surface topography was studied by X-ray diffraction and SEM. Electrochemical measurements indicate a good corrosion resistance of both bulk metallic glasses in NaCl solutions with low molarity at pH 8 due to the formation of protective oxide films. However, at high NaCl molarity the Cu-Zr-Al-Y glass shows no passive layer and is very susceptible to pitting corrosion. The mechanisms of the formation of the oxide films as well as the nucleation and growth of pitting were clarified by associating microstructural investigations with the results of electrochemical measurements. (C) 2006 Elsevier B.V All rights reserved.

SN 0925-8388

PD MAY 31

PY 2007

VL 434

SI SI

BP 234

EP 236

DI 10.1016/j.jallcom.2006.08.112

UT WOS:000246286900061

ER

PT J

AU Wang, WH

Li, FY

Pan, MX

Zhao, DQ

Wang, RJ

AF Wang, WH

Li, FY

Pan, MX

Zhao, DQ

Wang, RJ

TI Elastic property and its response to pressure in a typical bulk metallic

glass

SO ACTA MATERIALIA

AB Acoustic velocities, elastic constants and thermodynamic parameters upon pressure up to 2 GPa of typical Zr41Ti14Cu12.5Ni10Be22.5 bulk metallic glass (BMG) have been studied by using a pulse echo overlap method. The results indicate that the glass forming ability (GFA) has a relation with the elastic constants for a glass forming system. The compression curve of the BMG is interposed among its components, and the BMG exhibits small volume changes upon pressure, confirming that the BMG has similar atomic close-packed configurations with elements which may closely related to the origin of its excellent GFA. (C) 2003 Acta Materialia Inc. Published by Elsevier Ltd. All rights reserved.

SN 1359-6454

PD FEB 9

PY 2004

VL 52

IS 3

BP 715

EP 719

DI 10.1016/i.actamat.2003.10.008

UT WOS:000188855100020

ER

PT J

AU Louzguine-Luzgin, DV

Bazlov, AI

Ketov, SV

Greer, AL

Inoue, A

AF Louzguine-Luzgin, D. V.

Bazlov, A. I.

Ketov, S. V.

Greer, A. L.

Inoue, A.

TI Crystal growth limitation as a critical factor for formation of Fe-based

bulk metallic glasses

SO ACTA MATERIALIA

AB In the present work we study the formation mechanism and crystallization behavior of Fe-based bulk metallic glasses influenced by the addition of rare-earth elements. Samples are characterized by conventional X-ray diffractometry, optical microscopy, and high-resolution fielde-mission-gun scanning and transmission electron microscopy. In the rare-earth-containing alloys, bulk metallic glasses are formed with populations of quenched-in nuclei. Consequently, crystallization on isothermal annealing occurs without any incubation period. This behavior, not typical of bulk metallic glasses in general, implies that in the rare-earth-containing alloys glass formation is possible only because of restricted crystal growth: both on cooling from the molten and on heating from the glassy state, growth of the primary chi-Fe36Cr12Mo10 crystals is too slow to be significant on a reasonable timescale. The low growth rate is connected with large inhomogeneous strain in the growing nanoparticles, while nucleation of eutectic colonies is hampered by slow diffusion of a rare-earth alloying element. (C) 2014 Acta Materialia Inc. Published by Elsevier Ltd. All rights reserved.

RI Greer, Lindsay/E-9433-2017; Inoue, Akihisa/E-5271-2015; LOUZGUINE,

Dmitri/D-2492-2010; Ketov, Sergey/G-5558-2011

OI LOUZGUINE, Dmitri/0000-0001-5716-4987; Ketov, Sergey/0000-0002-6002-603X

SN 1359-6454

EI 1873-2453

PD JAN 1

PY 2015

VL 82

BP 396

EP 402

DI 10.1016/j.actamat.2014.09.025

UT WOS:000347017800037

ER

PT J

AU Jiang, MQ

Meng, JX

Gao, JB

Wang, XL

Rouxel, T

Keryvin, V

Ling, Z

Dai, LH

AF Jiang, M. Q.

Meng, J. X.

Gao, J. B.

Wang, X. -L.

Rouxel, T.

Keryvin, V.

Ling, Z.

Dai, L. H.

TI Fractal in fracture of bulk metallic glass

SO INTERMETALLICS

AB We investigate the nanoscale periodic corrugation (NPC) structures on the dynamic fracture surface of a typical tough bulk metallic glass, submitted to high-velocity plate impact and scanned by atomic force microscopy (AFM). The detrended fluctuation analysis (DFA) of the recorded AFM profiles reveals that the valley landscapes of the NPC are nearly memoryless, characterized by Hurst parameter of 0.52 and exhibiting a self-similar fractal character with the dimension of about 1.48. Our findings confirm the existence of the "quasi-cleavage" fracture underpinned by tension transformation zones (TTZs) in metallic glasses. (C) 2010 Elsevier Ltd. All rights reserved.

RI keryvin, vincent/F-1279-2010; Wang, Xun-Li/C-9636-2010

OI Wang, Xun-Li/0000-0003-4060-8777; Dai, LanHong/0000-0001-8991-0358

SN 0966-9795

PD DEC

PY 2010

VL 18

IS 12

BP 2468

EP 2471

DI 10.1016/j.intermet.2010.08.003

UT WOS:000284447500030

ER

PT J

AU Jiang, WH

Fan, GJ

Choo, H

Liaw, PK

AF Jiang, W. H.

Fan, G. J.

Choo, H.

Liaw, P. K.

TI Ductility of a Zr-based bulk-metallic glass with different specimen's

geometries

SO MATERIALS LETTERS

AB The ductility of a Zr52.5Cu17.9Ni14.6Al10Ti5 bulk-metallic glass with different specimen's geometries in quasistatic compression experiments was investigated. The length (0 to diameter (d) ratio, l/d, of specimens significantly affects the demonstration of the ductility. The specimens with the l/d larger than 0.75 exhibit a poor ductility, while those with the l/d equal to and smaller than 0.75 show an excellent ductility. The maximum elongation before the failure was observed to be up to about 80%. This difference in ductility is a result of the geometrical constraints. The present study demonstrates that the bulk-metallic glass has the outstanding intrinsic ductility. (c) 2006 Elsevier B.V. All rights reserved.

RI Choo, Hahn/A-5494-2009

OI Choo, Hahn/0000-0002-8006-8907

SN 0167-577X

EI 1873-4979

PD DEC

PY 2006

VL 60

IS 29-30

BP 3537

EP 3540

DI 10.1016/j.matlet.2006.03.047

UT WOS:000242469000023

ER

PT J

AU Scudino, S

Eckert, J

Mickel, C

Schubert-Bischoff, P

Breitzke, H

Luders, K

Schultz, L

AF Scudino, S

Eckert, J

Mickel, C

Schubert-Bischoff, P

Breitzke, H

Luders, K

Schultz, L

TI Quasicrystalline phase formation in Zr-Ti-Nb-Cu-Ni-(Al) metallic glasses

SO JOURNAL OF ALLOYS AND COMPOUNDS

AB The crystallization behavior of (Zr0.616Ti0.087Nb0.027Cu0.15Ni0.12)(100-x)Al-x melt-spun glassy ribbons with x = 7.5 and 0 has been investigated. The glasses devitrify by the precipitation of an icosahedral quasi-crystalline phase in the first step of the crystallization process, indicating that the absence of Al does not hinder quasicrystal formation in the present alloys. (C) 2004 Elsevier B.V. All rights reserved.

RI Schultz, Ludwig/B-3383-2010; Scudino, Sergio/D-8049-2015

SN 0925-8388

EI 1873-4669

PD JAN 25

PY 2005

VL 387

IS 1-2

BP 269

EP 273

DI 10.1016/j.jallcom.2004.06.071

UT WOS:000226277400052

ER

PT J

AU Zhang, ZL

Guo, XH

Liu, JT

Yan, CJ

Xu, CB

Guo, J

AF Zhang, Zhaolei

Guo, Xiaohui

Liu, Jingtao

Yan, Chuanjin

Xu, Chunbo

Guo, Jing

TI Effect of remelting treatment on glass forming ability of RE (Gd,

Sm)(56)Al26Co18 alloys

SO JOURNAL OF ALLOYS AND COMPOUNDS

AB The bulk metallic glasses (BMGs) were prepared by a copper mold suction casting method, denoted by Gd56Al26Co18 (1) and Sm56Al26Co18 (1). Then, the obtained metallic glasses mentioned above were remelted and injected to copper mold, and the metallic glasses were prepared by remelting, denoted by Gd56Al26Co18 (2) and Sm56Al26Co18 (2). The glass forming ability and thermal expansion behavior were investigated by X-ray diffraction (XRD), differential scanning calorimetry (DSC), dilatometer and high resolution transmission electron microscopy (HRTEM). The results showed that the remelting treatment can improve the glass forming ability of the alloys. In addition, it was found that the average thermal expansion coefficients of the BMGs prepared by remelting were smaller than the BMGs prepared by first-time melting for Gd56Al26Co18 and Sm56Al26Co18 alloys, which indicated the remelting treatment can enhance the thermal stability of the Gd56Al26Co18 and Sm56Al26Co18 BMGs. (C) 2013 Elsevier B. V. All rights reserved.

SN 0925-8388

EI 1873-4669

PD MAR 5

PY 2014

VL 588

BP 530

EP 533

DI 10.1016/j.jallcom.2013.11.077

UT WOS:000330179200087

ER

PT S

AU Xie, GQ

Louzguine-Luzgin, DV

Fukuhara, M

Inoue, A

AF Xie, Guoqiang

Louzguine-Luzgin, D. V.

Fukuhara, Mikio

Inoue, Akihisa

BE Tan, Y

Ju, DY

TI Bulk metallic glassy composites with excellent electrical conductivity

and enhanced plasticity fabricated by spark plasma sintering

SO ADVANCED MATERIAL SCIENCE AND TECHNOLOGY, PTS 1 AND 2

SE Materials Science Forum

CT 7th International Forum on Advanced Material Science and Technology

CY JUN 26-28, 2011

CL Dalian, PEOPLES R CHINA

SP Natl Nat Sci Fdn China, KC Wong Educ Fdn, Dalian Univ Technol, Changchun Res Inst Mech Sci Co Ltd

AB Large-size Ni-based bulk metallic glass (BMG) composite samples exhibiting simultaneously high strength, enhanced plasticity and improved conductivity were produced by spark plasma sintering of mixed glassy powder blended with high-conductive Cu particulates. This opens new possibilities for the applications of the BMG composites as functional and structural materials.

RI Xie, Guoqiang/A-8619-2011; Inoue, Akihisa/E-5271-2015

OI Louzguine-Luzgin, Dmitri/0000-0001-5716-4987

SN 0255-5476

PY 2011

VL 675-677

BP 197

EP +

DI 10.4028/www.scientific.net/MSF.675-677.197

UT WOS:000297036200046

ER

PT S

AU Yang, XH

Nie, XP

Jiang, JZ

AF Yang, Xiao-hong

Nie, Xi-peng

Jiang, Jian-zhong

BE Chen, R

Sung, WP

TI Ti microalloying effect on corrosion resistance and thermal stability of

Cu(45)Zr(48)A(17) bulk metallic glass

SO MECHATRONICS AND INTELLIGENT MATERIALS II, PTS 1-6

SE Advanced Materials Research

CT 2nd International Conference on Mechatronics and Intelligent Materials

(MIM 2012)

CY MAY 18-19, 2012

CL Guilin, PEOPLES R CHINA

AB Bulk metallic glasses (BMGs) of Cu45Zr48-xAl7Tix with x= 0, 1.5, and 3 at.% were prepared by copper mould casting. The corrosion resistance of the BMGs with different Ti contents was examined by potentiodynamic polarization tests and weight loss measurements in 1 N NaOH, 1 N H2SO4, 1 N H2SO4 + 0.01 N NaCl and 0.5 N NaCl solutions, respectively. The newly-developed BMGs' corrosion resistance in Cl-- or both H+ and Cl--ions containing solutions can be greatly enhanced. The influence of Ti addition on glass forming ability (GFA) and thermal stability was investigated by x-ray diffraction and differential scanning calorimetry in detail. The alloy containing 1.5 at.% Ti exhibits the largest GFA, the critical size comes up to 10 mm in diameter.

SN 1022-6680

BN 978-3-03785-384-9

PY 2012

VL 490-495

BP 3868

EP +

DI 10.4028/www.scientific.net/AMR.490-495.3868

UT WOS:000312926101388

ER

PT J

AU Chu, JP

Huang, JC

Jang, JSC

Wang, YC

Liaw, PK

AF Chu, J. P.

Huang, J. C.

Jang, J. S. C.

Wang, Y. C.

Liaw, P. K.

TI Thin film metallic glasses: Preparations, properties, and applications

SO JOM

AB With unique properties such as high strength, amorphous alloys in the bulk form have been a focus for many studies in recent years. Yet, the amorphous alloys (metallic glass) in the thin film form have not received much attention. In this paper, we will review and report some important and interesting results obtained from these thin film metallic glasses in which unique physical and mechanical properties can be enhanced by changing their compositions and by the precipitation of nanoscale particles.

RI Huang, J./C-4276-2013; Wang, Yunche/A-4869-2010

OI HUANG, Jacob Chih Ching/0000-0001-6843-3396

SN 1047-4838

EI 1543-1851

PD APR

PY 2010

VL 62

IS 4

BP 19

EP 24

DI 10.1007/s11837-010-0053-3

UT WOS:000276770100005

ER

PT J

AU Pu, J

Wang, JF

Xiao, JZ

Cui, K

AF Pu, J

Wang, JF

Xiao, JZ

Cui, K

TI Formation and crystallization of bulk Pd82Si18 amorphous alloys

SO TRANSACTIONS OF NONFERROUS METALS SOCIETY OF CHINA

AB Bulk amorphous Pd82Si18 alloy with the largest diameter of 8 mm was prepared by water quenching the molten alloy with flux medium in a quartz tube. The calculation result indicates that the bulk Pd82Si18 amorphous alloys have a low critical cooling rate (R-c) of 4.589 K/s or less. The experimental results show that purifying melt may improve glass forming ability(GFA) of undercooled melt, while liquid phase separation (LPS) of undercooled melt will decrease its GFA. There are some differences in crystallization experiments between bulk metallic glass and amorphous ribbons of Pd82Si18 alloys. These include the numbers of exothermic peak, glass transition temperature T-g, crystallization temperature T-x, region of undercooling liquid (DeltaT=T-x-T-g) respectively. The links of cooling rates of melt and crystallization of Pd82Si18 amorphous alloys are explored.

SN 1003-6326

PD OCT

PY 2003

VL 13

IS 5

BP 1056

EP 1061

UT WOS:000186374500007

ER

PT J

AU Kim, JY

Gu, X

Wraith, M

Uhl, JT

Dahmen, KA

Greer, JR

AF Kim, Ju-Young

Gu, Xun

Wraith, Matt

Uhl, Jonathan T.

Dahmen, Karin A.

Greer, Julia R.

TI Suppression of Catastrophic Failure in Metallic Glass-Polyisoprene

Nanolaminate Containing Nanopillars

SO ADVANCED FUNCTIONAL MATERIALS

AB One considerable concern in metallic glass is enhancing ductility by suppressing catastrophic failure by the instantaneous propagation of shear bands. Compressed nanopillars with alternating CuZr metallic glass and polyisoprene nanolaminates exhibit >30% enhancement in plastic flow, as compared with monolithic glass, without sacrifice of strength. A suppression of stochastic strain burst signature in these metallic glass-polymer composites is reported, which is an undesirable characteristic ubiquitously present in monolithic metallic glass and in metallic glass-metal composites. The intermittent stochastic signature is quantified in each metallic glass-containing nanolaminate system by constructing histograms of burst size distributions and provide theoretical foundation for each behavior. The exceptional mechanical properties emergent in these MG-polymer nanolaminate composites are attributed to the combination of nanometer size-induced shear band suppression in metallic glasses and the damping capability of the polyisoprene layers.

SN 1616-301X

PD MAY 9

PY 2012

VL 22

IS 9

BP 1972

EP 1980

DI 10.1002/adfm.201103050

UT WOS:000303434600021

ER

PT J

AU Zhang, Y

AF Zhang, Y.

TI Inhomogeneous deformation in metallic glasses

SO MATERIALS SCIENCE AND TECHNOLOGY

AB In metallic glasses, the combination of metallic bonding with an amorphous structure gives excellent mechanical properties such as high yield stress and yield strain compared with conventional polycrystalline alloys. It is, therefore, of great interest to exploit bulk metallic glasses ( BMGs) as structural materials, particularly as many are now available in bulk form. The plastic deformation of BMGs is generally inhomogeneous, severely localised into shear bands with a characteristic thickness of only similar to 10 nm. This shear instability is associated with work softening and impedes the exploitation of the otherwise desirable mechanical properties of metallic glasses in structural applications. Recent progress in understanding work softening in metallic glasses and the consequent formation of shear bands is reviewed, considering both experimental work and molecular dynamics simulations. The basic phenomena of plastic deformation in BMGs are briefly introduced. The initiation of shear bands, their propagation, and associated structural changes within the bands are considered. Recent advances in tailoring the microstructure of metallic glasses to increase their plasticity are also presented. The mechanisms of forming shear bands in different material systems are compared, highlighting the distinct plastic deformation mechanisms in BMGs.

SN 0267-0836

EI 1743-2847

PD APR

PY 2008

VL 24

IS 4

BP 379

EP 391

DI 10.1179/174328408X276044

UT WOS:000255904100001

ER

PT J

AU Tang, JL

Yu, LS

Qiao, JC

Wang, YY

Wang, H

Duan, M

Chamas, M

AF Tang, Junlei

Yu, Liusi

Qiao, Jichao

Wang, Yingying

Wang, Hu

Duan, Ming

Chamas, Mohamad

TI Effect of atomic mobility on the electrochemical properties of a

Zr58Nb3Cu16Ni13Al10 bulk metallic glass

SO ELECTROCHIMICA ACTA

AB The corrosion behaviour of bulk metallic glasses is greatly related to their microstructures. The microstructures in bulk metallic glasses of the same chemical composition are represented to a certain extent by their atomic mobility. Evolution of the microstructure and mechanical properties of Zr58Nb3Cu16Ni13Al10 bulk metallic glass after annealing were studied with X-ray diffraction, dynamic mechanical analysis and Vickers hardness tests. Polarization curve, open circuit potential, electrochemical impedance spectroscopy and Mott-Schottky analysis were performed to investigate the effect of atomic mobility on the corrosion behaviour in 1 mol/L de-aerated H2SO4 at 333 K. Dynamic mechanical analysis indicated that the atomic mobility of Zr58Nb3Cu16Ni13Al10 bulk metallic glass was reduced by structural relaxation and crystallization. All electrochemical measurements showed that the passivation ability decreased and the corrosion resistance of Zr58Nb3Cu16Ni13Al10 bulk metallic glass deteriorated as the annealing temperature increased. Mott-Schottky analysis revealed that the defect concentration in the passive films increased and the flat-band potential shifted to a negative potential with a decrease in the atomic mobility. X-ray photoelectron spectroscopy results proved that the main chemical compositions of the passive films were ZrO2, Al2O3 and Nb2O5. The formation of a passive film was suppressed because the atomic mobility of strong passivating elements (Zr, Al and Nb) decreased. In addition, the precipitation of crystals (Zr2Cu and Cu10Zr7) accelerated the corrosion of the bulk metallic glass in H2SO4. (C) 2018 Elsevier Ltd. All rights reserved.

SN 0013-4686

EI 1873-3859

PD MAR 20

PY 2018

VL 267

BP 222

EP 233

DI 10.1016/j.electacta.2018.02.071

UT WOS:000427382500025

ER

PT J

AU Wu, FF

Zhang, ZF

Mao, SX

AF Wu, F. F.

Zhang, Z. F.

Mao, S. X.

TI Size-dependent shear fracture and global tensile plasticity of metallic

glasses

SO ACTA MATERIALIA

AB The tensile ductility or brittleness of metallic glasses is found to depend strongly oil the critical shear offset. Based oil experimental observations, the tensile shear fracture processes of metallic glasses can be divided into three stages: multiplication and coalescence of the free volume, formation of void and the final fast propagation of a shear crack. Accordingly, the size effect on the tensile shear deformation processes of metallic glass can be well understood: with decreasing specimen size smaller than the equivalent critical shear offset, the shear deformation of metallic glass is changed from unstable to stable, which leads to a transition from global brittleness on the macroscale to large global plasticity or even necking oil the microscale. These results are fundamentally useful in understanding the physical nature of tensile shear deformation of various metallic glasses and even in the design of new metallic glass materials with good plasticity. (C) 2008 Acta Materialia Inc. Published by Elsevier Ltd. All rights reserved.

RI Zhang, BMG/C-6151-2014; Zhang, Zhefeng/A-9732-2010

SN 1359-6454

PD JAN

PY 2009

VL 57

IS 1

BP 257

EP 266

DI 10.1016/j.actamat.2008.09.012

UT WOS:000263695300028

ER

PT S

AU Baricco, M

Baser, TA

Fiore, G

Piccin, R

Satta, M

Castellero, A

Rizzi, P

Battezzati, L

AF Baricco, M.

Baser, T. A.

Fiore, G.

Piccin, R.

Satta, M.

Castellero, A.

Rizzi, P.

Battezzati, L.

BE Cabibbo, M

Spigarelli, S

TI Bulk Metallic Glasses

SO RECENT DEVELOPMENTS IN THE PROCESSING AND APPLICATIONS OF STRUCTURAL

METALS AND ALLOYS

SE Materials Science Forum

CT International Conference on Recent Developments in the Processing and

Applications of Structural Metals and Alloys

CY JUN 22-25, 2008

CL Marche Polytechn Univ, Dept Mech Engn, Como, ITALY

HO Marche Polytechn Univ, Dept Mech Engn

AB Rapid quenching techniques have been Successfully applied since long time for the preparation of metallic glasses in ribbon form. Only in the recent years, the research activity addressed towards the synthesis of bulk metallic glasses (BMG), in form of ingots with a few millimetres in thickness. These materials can be obtained by casting techniques only for selected alloy compositions, characterised by a particularly high glass-forming tendency. Bulk amorphous alloys are characterised by a low modulus of elasticity and high yielding stress. The usual idea is that amorphous alloys undergo work softening and that deformation is concentrated in shear bands, which might be subjected to geometrical constraints, resulting in a substantial increase in hardness and wear resistance. The mechanical properties can be further improved by crystallisation. In fact, shear bands movement can be contrasted by incorporating a second phase in the material, which may be produced directly by controlled crystallisation. Soft magnetic properties have been obtained in Fe-based systems and they are strongly related to small variations in the microstructure, ranging from a fully amorphous phase to nanocrystalline phases with different crystal size. The high thermal stability of bulk metallic glasses makes possible the compression and shaping processes in the temperature range between glass transition and crystallisation. Aim of this paper is to present recent results on glass formation and properties of bulk metallic glasses with various compositions. Examples will be reported oil Zr, Fe, Mg and Pd-based materials, focussing on mechanical and magnetic properties,

RI Rizzi, Paola/I-8810-2012; Baricco, Marcello/B-4075-2013

OI Rizzi, Paola/0000-0002-3977-2839; Baricco, Marcello/0000-0002-2856-9894;

Castellero, Alberto/0000-0001-8290-7543; Battezzati,

Livio/0000-0003-1628-0409

SN 0255-5476

PY 2009

VL 604-605

BP 229

EP +

DI 10.4028/www.scientific.net/MSF.604-605.229

UT WOS:000263555900024

ER

PT J

AU Yang, B

Du, Y

Liu, Y

AF Yang Bing

Du Yong

Liu Yong

TI Recent progress in criterions for glass forming ability

SO TRANSACTIONS OF NONFERROUS METALS SOCIETY OF CHINA

AB The glass-forming ability(GFA) is an important factor in studying metallic glasses. So far, there are several criteria for evaluating the glass-forming ability. For predicting compositions for bulk metallic glasses, however, they show more or less accuracy and versatility for different cases. In this work, four types of criteria for the glass-forming ability are categorized and reviewed: 1) Indicators with characteristic temperatures; 2) Indicators involving structural factors; 3) Indicators based on Miedema's model; and 4) Indictors based on phase diagram. It is pointed out that a single indicator cannot be used to predict GFA of all the metallic glass systems correctly due to its limited theoretical framework, and the combination of multiple indicators shows more efficiency and accuracy. Though it is still very difficult to develop a universal indicator for GFA, recent indicators seem to be of more reliable physical meaning than those previously suggested.

OI Du, Yong/0000-0002-4187-3989

SN 1003-6326

PD FEB

PY 2009

VL 19

IS 1

BP 78

EP 84

DI 10.1016/S1003-6326(08)60232-0

UT WOS:000264905700013

ER

PT J

AU Han, JJ

Wang, CP

Wang, J

Liu, XJ

Wang, Y

Liu, ZK

AF Han, J. J.

Wang, C. P.

Wang, J.

Liu, X. J.

Wang, Y.

Liu, Z. K.

TI Compositional design of Fe-based multi-component bulk metallic glass

based on CALPHAD method

SO MATERIALS & DESIGN

AB A quantificational composition design protocol (CDP) of Fe-based bulk metallic glasses (BMGs) with excellent glass-forming ability (GFA) has been proposed on the basis of the thermodynamic calculations. The stability of liquid and the crystallization of solids were both evaluated from the perspective of atomic structure and local composition. The present protocol successfully associates the stability of liquid to the melting point of alloy and the difficulty of crystallization by considering the type and competition of potential equilibrated phases. Specifically, this protocol provided the selection criteria of phases and elements from the viewpoint of structure and composition fluctuations during nucleation for each phase, whose accuracy and effectiveness were experimentally verified.

RI Wang, Yi/D-1032-2013; Liu, Zi-Kui/A-8196-2009

OI Liu, Zi-Kui/0000-0003-3346-3696; Han, Jiajia/0000-0002-2642-0252

SN 0264-1275

EI 1873-4197

PD JUL 15

PY 2017

VL 126

BP 47

EP 56

DI 10.1016/j.matdes.2017.04.030

UT WOS:000402491400006

ER

PT J

AU Li, N

Chen, W

Liu, L

AF Li, Ning

Chen, Wen

Liu, Lin

TI Thermoplastic Micro-Forming of Bulk Metallic Glasses: A Review

SO JOM

AB Bulk metallic glasses are a fascinating class of metallic alloys with an isotropic amorphous structure that is rapidly quenched from liquid melts. The absence of a crystalline micro-structure endows them with a portfolio of properties such as high strength, high elasticity, and excellent corrosion resistance. Whereas the limited plasticity and hence poor workability at ambient temperature impede the structural application of bulk metallic glasses, the unique superplasticity within the supercooled liquid region opens an alternative window of so-called thermoplastic forming, which allows precise and versatile net-shaping of complex geometries on length scales ranging from nanometers to centimeters that were previously unachievable with conventional crystalline metal processing. Thermoplastic forming not only breaks through the bottleneck of the manufacture of bulk metallic glasses at ambient temperature but also offers an alluring prospect in micro-engineering applications. This paper comprehensively reviews some pivotal aspects of bulk metallic glasses during thermoplastic micro-forming, including an in-depth understanding of the crystallization kinetics of bulk metallic glasses and the thermoplastic processing time window, the thermoplastic forming map that clarifies the relationship between the flow characteristics and the formability, the interfacial friction in micro-forming and novel forming methods to improve the formability, and the potential applications of the hot-embossed micro-patterns/components.

SN 1047-4838

EI 1543-1851

PD APR

PY 2016

VL 68

IS 4

BP 1246

EP 1261

DI 10.1007/s11837-016-1844-y

UT WOS:000373131200027

ER

PT J

AU Zhao, ZF

Zhi, Z

Li, Z

Ping, W

Zhao, DQ

Pan, MX

Wang, WL

Wang, WH

AF Zhao, ZF

Zhi, Z

Li, Z

Ping, W

Zhao, DQ

Pan, MX

Wang, WL

Wang, WH

TI A new Pr-based bulk metallic glass and its properties

SO ACTA PHYSICA SINICA

AB A new rare-earth Pr-based bulk metallic glass (BMG) is obtained in the shape of rod up to 5 mm in diameter by die cast. Urlike other rare-earth based BMGs, it exhibits a distinct glass transition, T-g = 409K, the lowest glass transition temperature among the known BMGs, a large and stable supercooled liquid region and paramagnetic property. The glass transition as well as its kinetic nature and the fragility parameters m of the BMG have been studied. The BMG offers an ideal model to investigate the nature of lass transition as well as the relaxation and nucleation with a large experimentally accessible time and temperature window at very low temperature region.

RI Zhao, Zuofeng/B-1297-2010

OI Zhao, Zuofeng/0000-0002-0862-8471

SN 1000-3290

PD MAR

PY 2004

VL 53

IS 3

BP 850

EP 853

UT WOS:000220367400037

ER

PT J

AU Wei, HQ

Long, ZL

Zhang, ZC

Li, XA

Peng, J

Zhang, P

AF Wei Hong-Qing

Long Zhi-Lin

Zhang Zhi-Chun

Li Xiang-An

Peng Jian

Zhang Ping

TI Correlations between viscosity and glass-forming ability in bulk

amorphous alloys

SO ACTA PHYSICA SINICA

AB According to the classical crystallization theory, the influences of kinetics and thermodynamics on glass-forming ability (GFA) for bulk metallic glasses has been investigated. The theoretical analysis shows that crystallization resistance is in proportion to the viscosity of "nose" temperature (T-n) while crystallization driving force is inversely proportional to the viscosity of crystallization onset temperature (T-x) on reheating in time-temperature-transformation (TTT) curve, and therefore a GFA parameter omega(0), defined as (T-g - T-0)/(T-x - T-0) - (T-g - T-0)/(T-n - T-0), was proposed (wherein T-g and T-0 are glass transition temperature and Vogel temperature respectively). The parameter omega(0) shows an excellent correlation with the critical cooling rate for glass formation of bulk metallic glasses, with the statistical correlation factor of R-2 = 0.9626. Furthermore, the relationships between the GFA and T-rg, Delta T-x, gamma, gamma(m), Delta T-rg, alpha, beta, delta and phi, as well as the fragility of liquid could be explained reasonably based on our proposed omega(0) parameter.

SN 1000-3290

PD APR

PY 2009

VL 58

IS 4

BP 2556

EP 2564

UT WOS:000265324700066

ER

PT J

AU Zhang, Z

Chen, CL

Wang, ZL

Yu, DM

AF Zhang Zhi

Chen Chun-Ling

Wang Zhao-Long

Yu Dong-Man

TI Elastic behaviour of Nd60Al10Fe20Co10 bulk metallic glass under pressure

SO ACTA PHYSICA SINICA

AB Measurements were carried out under hydrostatic pressure up to 0.5 GPa at room temperature, by using an ultrasonic pulse-echo method to measure the transit time of longitudinal and transverse elastic waves (10MHz) in a Nd-60 Al-10 Fe-20 Co-10 bulk metallic glass. Based on the experimental data, the sound velocity, density, elastic moduli and Debye temperature were derived as functions of pressure, and the Murnaghan's equation of state was obtained. Moreover, the compression curve, the elastic constants and the Debye temperature of the bulk metallic glass are calculated on the basis of the similarity between their physical properties in the glass state and those in corresponding crystalline state. These results confirm that the elastic properties of Nd60Al10Fe20Co10 bulk metallic glass are related to its component elements.

SN 1000-3290

PD NOV

PY 2006

VL 55

IS 11

BP 5975

EP 5979

UT WOS:000241880000066

ER

PT J

AU Kokotin, V

Hermann, H

AF Kokotin, V.

Hermann, H.

TI Geometrical aspects of the glass-forming ability of dense binary

hard-sphere mixtures

SO SCRIPTA MATERIALIA

AB Non-crystalline dense binary hard-sphere mixtures are generated where the size ratio of the spheres and the fraction of small spheres are varied from 1.0 to 2.0 in steps of 0.1 and from 0 to 100% in steps of 10%, respectively. A confined region within this parameter space is defined where non-crystalline structures are stabilized. Comparison of the present results with experimental data for binary bulk metallic glasses supports the validity of geometrical arguments regarding glass-forming ability of binary metallic melts. (C) 2009 Acta Materialia Inc. Published by Elsevier Ltd. All rights reserved.

SN 1359-6462

PD AUG

PY 2009

VL 61

IS 3

BP 261

EP 264

DI 10.1016/j.scriptamat.2009.03.058

UT WOS:000266788200009

ER

PT J

AU Qiao, JC

Pelletier, JM

AF Qiao, J. C.

Pelletier, J. M.

TI Dynamic universal characteristic of the main (alpha) relaxation in bulk

metallic glasses

SO JOURNAL OF ALLOYS AND COMPOUNDS

AB The main (alpha) relaxation in typical metallic glasses was studied by dynamic mechanic analysis (DMA). Experimental results indicate that the main relaxation is a common feature in all the metallic glasses as well as other glassy-like materials. Initially, experimental results are analyzed using the Kohlrausch-Williams-Watts (KWW) phenomenological model. The Kohlrausch exponent beta(KWW) is around 0.5 near their glass transition domain. In addition, it is shown that the dynamic mechanical behavior in metallic glasses is in agreement with the predictions of the physical model called the quasi-point defects (QPDs) theory. In this model a correlation parameter (chi) is introduced, which is correlated to the disorder degree. The results on the main relaxation of the metallic glasses emphasize a striking correlation between quasi-point defects theory and KWW model: chi approximate to 0.8 beta(KWW). This relation provides new insight of the non-exponential dynamic character for the amorphous materials. (C) 2013 Elsevier B. V. All rights reserved.

SN 0925-8388

EI 1873-4669

PD MAR 15

PY 2014

VL 589

BP 263

EP 270

DI 10.1016/j.jallcom.2013.11.192

UT WOS:000330181400041

ER

PT J

AU Torrens-Serra, J

Solivelles, F

Corro, ML

Stoica, M

Kustov, S

AF Torrens-Serra, J.

Solivelles, F.

Corro, M. L.

Stoica, M.

Kustov, S.

TI Effect of temperature and magnetic field on magnetomechanical damping of

Fe-based bulk metallic glasses

SO JOURNAL OF PHYSICS D-APPLIED PHYSICS

AB Temperature and magnetic field dependence of magnetomechanical damping (MMD) of two ferromagnetic Fe-based bulk metallic glasses with different Curie temperatures, T-C, have been studied over a broad temperature interval from the para-ferromagnetic transition down to 15 K. The damping has been scanned under periodic magnetic field at certain preselected temperatures in the ferromagnetic and paramagnetic phases. The selection of bulk metallic glasses for investigations allowed us to eliminate all dislocation-related anelastic effects and facilitated separation of the MMD components. Under zero field, the non-linear MMD emerges at T-C and first increases linearly with (T-C - T), then levels off until a maximum is formed at around 36 K. At lower temperatures, the magnetic domain wall related non-linear MMD component is partially or completely supressed, depending on the alloy composition. Qualitatively similar anomaly is found in the temperature dependence of the linear macroeddy current damping. These anomalies in the temperature spectra are concomitant with the emergence of a notable hysteresis in MMD versus periodic field dependences and of a maximum of non-linear MMD close to the position of the macroeddy damping peak. The uncovered phenomena are attributed to abrupt change of magnetic properties of ferromagnetic bulk metallic glasses at low temperatures, presumably due to the re-entrant spin glass transition.

RI Torrens-Serra, Joan/L-9805-2018; Stoica, Mihai/B-7069-2015

OI Solivelles, Francesc/0000-0002-9840-8228

SN 0022-3727

EI 1361-6463

PD DEC 21

PY 2016

VL 49

IS 50

AR 505003

DI 10.1088/0022-3727/49/50/505003

UT WOS:000388744300001

ER

PT J

AU Wang, T

Si, JJ

Wu, YD

Lv, K

Liu, YH

Hui, XD

AF Wang, Tuo

Si, Jiajia

Wu, Yidong

Lv, Kuang

Liu, Yanhui

Hui, Xidong

TI Two-step work-hardening and its gigantic toughening effect in Zr-based

bulk metallic glasses

SO SCRIPTA MATERIALIA

AB In this work, a two-step work-hardening phenomenon during the compressive deformation process of Zr57.75-xCu21.15Fe4.7Al9.4Nb6COxMo1 (x = 1, 2, 3) bulk metallic glasses (BMGs) has been found for the first time. This novel work-hardening mode remarkably improves the deformability and the toughening of these BMGs. It has been evidenced that the secondary stage of work-hardening contributes as high as 60% of total strengthening and 75% of plasticitizing for the BMG with x = 3. The underlying mechanism for the two-step work-hardening has been clarified as the phase separation for first step followed by the nanocrystallization around the shear bands induced by continuous compression deformation. (C) 2018 Acta Materialia Inc. Published by Elsevier Ltd. All rights reserved.

SN 1359-6462

PD JUN

PY 2018

VL 150

BP 106

EP 109

DI 10.1016/j.scriptamat.2018.03.006

UT WOS:000432612500024

ER

PT J

AU Xia, MX

Meng, QG

Zhang, SG

Liu, J

Ma, CL

Li, JG

AF Xia, Ming-xu

Meng, Qing-ge

Zhang, Shu-guang

Liu, Jian

Ma, Chao-li

Li, Jian-guo

TI Nd60Fe30-xNixAl10 bulk metallic glasses with high hard magnetic

properties

SO MATERIALS LETTERS

AB Bulk metallic glasses (BMGs) Nd60Fe30-xNixAl10 were prepared by suction cast method. The glass forming abilities (GFAs) and the hard magnetic properties of the BMGs were examined by differential scanning calorimeter (DSC) and vibrating sample magnetometer (VSM), respectively. The results show that the largest GFA (T-x/T-m = 0.61) of the alloys was obtained when Fe was substituted by 10% Ni and the rods of Nd60Fe20Ni10Al10 have the coercivity up to 323 kA/m and the remanence up to 9.41 Am-2/kg as high as Nd-Fe-Co-Al, the Nd-Fe-based BMGs with highest hard magnetic properties up to date. The homogeneous distribution of Fe-rich nano-clusters, Nd(FeNiAl)(2), in amorphous matrix is responsible for the enhancement. (c) 2006 Elsevier B.V. All rights reserved.

RI xia, mingxu/C-4070-2011; Liu, Jian/A-2309-2010

OI xia, mingxu/0000-0002-3113-4795

SN 0167-577X

EI 1873-4979

PD JAN

PY 2007

VL 61

IS 1

BP 219

EP 222

DI 10.1016/j.matlet.2006.04.035

UT WOS:000242511300055

ER

PT J

AU Senkov, ON

Miracle, DB

AF Senkov, Oleg N.

Miracle, Daniel B.

TI Relaxation Behavior of Ca-Based Bulk Metallic Glasses

SO METALLURGICAL AND MATERIALS TRANSACTIONS A-PHYSICAL METALLURGY AND

MATERIALS SCIENCE

CT International Conference on Bulk Metallic Glasses held at the 2009 TMS

Annual Meeting

CY 2009

CL San Francisco, CA

SP Minerals, Met & Mat Soc

AB The relaxation behavior of Ca(60)Mg(20)Zn(20), Ca(60)Mg(20)Cu(20), Ca(65)Mg(15)Zn(20), Ca(50)Mg(20)Cu(30), and Ca(55)Mg(18)Zn(11)Cu(16) bulk metallic glasses was determined in the glass transition region using differential scanning calorimetry (DSC) with heating rates from 1 to 160 K/min. The activation enthalpy of structural relaxation and the fragility index m were found to be smaller in the glassy state (onset of the glass transition) than in the supercooled liquid state (end of glass transition). The Ca-based glass-forming liquids showed strong behavior of the relaxation time, with the fragility indexes m in the range of 33 to 40. The strong liquid behavior implies sluggish kinetics of crystallization in the supercooled liquid region and explains the very good glass-forming ability (GFA) of these alloys. The critical cooling rate for amorphization R (c) of the Ca-based bulk metallic glasses was estimated to be in the range of 0.3 to 10 K/s, which is similar to R (c) values for the best Pd- and Zr-based metallic glass-forming alloys discovered so far.

RI Senkov, Oleg/C-7197-2012

OI Senkov, Oleg/0000-0001-5587-415X; Senkov, Oleg/0000-0002-9336-3702

SN 1073-5623

PD JUL

PY 2010

VL 41A

IS 7

BP 1677

EP 1684

DI 10.1007/s11661-009-9981-3

UT WOS:000277958700013

ER

PT J

AU Shin, J

Kwon, J

Park, JS

Bael, D

AF Shin, Jaehyuck

Kwon, Jinwook

Park, Joon Sik

Bael, Donghyun

TI Synthesis of Ni-based bulk metallic glasses for penetrating materials

SO MATERIALS TRANSACTIONS

AB The Ni-based bulk metallic glasses (BMGs(Ni59Zr16Ti13,Nb7Si3Sn2)) modified by At, Hf and W have been synthesized for a specific application of penetrating materials. When Al was replaced with Zr of the Ni59Zr16Ti13Nb7Si3Sn2 BMG, a large lass forming ability was observed, enabling to fabricate 7 mm cylindrical amorphous specimen. Also, when Hf or W was replaced with Ni of the Ni59Zr16Ti13Nb7Si3Sn2 BMG. the 7 mm amorphous BMG specimen was obtained. For the Al modified BMG (Ni59Zr15Ti13Nb7Si3Sn2Al1), a high strength of similar to 2.6 GPa with similar to 10% plastic strain was observed. It appears that the Hf modified BMG showed an excellent candidate for the application of penetrating materials among, the examined BMGs.

RI Juhyun, Oh/H-9185-2012

SN 1345-9678

EI 1347-5320

PD AUG

PY 2008

VL 49

IS 8

BP 1796

EP 1799

DI 10.2320/matertrans.MRA2008095

UT WOS:000259799900018

ER

PT J

AU Feng, RX

Stachurski, ZH

Rodriguez, MD

Kluth, P

Araujo, LL

Bulla, D

Ridgway, MC

AF Feng, Ruixing

Stachurski, Z. H.

Rodriguez, M. D.

Kluth, P.

Araujo, L. L.

Bulla, D.

Ridgway, M. C.

TI X-ray scattering from amorphous solids

SO JOURNAL OF NON-CRYSTALLINE SOLIDS

CT 13th International Conference on the Physics of Non-Crystalline Solids

(PNCS)

CY SEP 16-20, 2012

CL Yichang, PEOPLES R CHINA

SP Hubei Sanxia New Bldg Mat Co Ltd, China Three Gorges Univ, Hubei Feilihua Quartz Glass Co Ltd, Jiansu Xiuqiang Glasswork Co Ltd, FiberHome Technologies Grp, Hubei New Huaguang Informat Mat Co Ltd, Yangtze Opt Fiber & Cable Co Ltd, AVIC Special Glass Mat Co Ltd, Hainan Univ

AB The main objective of this work is to verify the proposed models by comparing computed outcomes with experimental results. For metallic glasses the novel ideal amorphous solid model is used to simulate the structure and the atomic positions which are input into the Debye equation. Computations predict structure factor or scattered intensity which agree well with the experimentally obtained data. For covalent materials, such as amorphous silica or amorphous polyethylene with short range order the Warren approach offers a simple method to predict X-ray scattering in good agreement with experimental data. Neither of the two above methods works well for chalcogenide glasses which require calculations involving spherical harmonics. (C) 2013 Elsevier B.V. All rights reserved.

RI Araujo, Leandro/A-3720-2008; Kluth, Patrick/A-1497-2008; Rodriguez,

Matias/F-3220-2011

OI Araujo, Leandro/0000-0002-9413-8777; Kluth, Patrick/0000-0002-1806-2432;

Stachurski, Zbigniew/0000-0002-1317-0178

SN 0022-3093

EI 1873-4812

PD JAN 1

PY 2014

VL 383

SI SI

BP 21

EP 27

DI 10.1016/j.jnoncrysol.2013.04.070

UT WOS:000330819000005

ER

PT J

AU Keppens, V

Zhang, Z

Senkov, ON

Miracle, DB

AF Keppens, V.

Zhang, Z.

Senkov, O. N.

Miracle, D. B.

TI Localized Einstein modes in Ca-based bulk metallic glasses

SO PHILOSOPHICAL MAGAZINE

CT 10th International Workshop on Disordered Systems Moveno-Andalo

CY MAR 18-21, 2006

CL Trento, ITALY

SP Univ Trento, Dipartimento Fis, DYGLAGEMEM European Network, INFM-CNR, Res Ctr SOFT

AB Low-temperature specific heat and elastic moduli measurements are reported for Ca-based bulk metallic glasses. The deviation from the Debye behaviour observed in the specific heat is modelled with a simple Einstein oscillator with characteristic temperature theta(E) = 80 K. The presence of this local mode can also account for the deviation from normal 'Varshni behaviour' observed in the temperature dependence of the elastic moduli.

RI Senkov, Oleg/C-7197-2012

OI Senkov, Oleg/0000-0001-5587-415X; Senkov, Oleg/0000-0002-9336-3702

SN 1478-6435

PD JAN-FEB

PY 2007

VL 87

IS 3-5

SI SI

BP 503

EP 508

DI 10.1080/14786430600857353

UT WOS:000244150900018

ER

PT J

AU Lu, ZP

Shen, J

Xing, DW

Sun, JF

Liu, CT

AF Lu, Z. P.

Shen, J.

Xing, D. W.

Sun, J. F.

Liu, C. T.

TI Binary eutectic clusters and glass formation in ideal glass-forming

liquids

SO APPLIED PHYSICS LETTERS

AB In this letter, a physical concept of binary eutectic clusters in "ideal" glass-forming liquids is proposed based on the characteristics of most well-known bulk metallic glasses (BMGs). The authors approach also includes the treatment of binary eutectic clusters as basic units, which leads to the development of a simple but reliable method for designing BMGs more efficiently and effectively in these unique glass-forming liquids. As an example, bulk glass formers with superior glass-forming ability in the Zr-Ni-Cu-Al and Zr-Fe-Cu-Al systems were identified with the use of the strategy. (c) 2006 American Institute of Physics.

RI Lu, Zhao-Ping/A-2718-2009

OI Lu, Zhao-Ping/0000-0003-1463-8948; Liu, Chain Tsuan/0000-0001-7888-9725

SN 0003-6951

PD AUG 14

PY 2006

VL 89

IS 7

AR 071910

DI 10.1063/1.2336597

UT WOS:000239842400028

ER

PT B

AU Habazaki, H

AF Habazaki, H.

BE Richardson, TJA

TI Corrosion of Amorphous and Nanograined Alloys

SO SHREIR'S CORROSION, VOL 3: CORROSION AND DEGRADATION OF ENGINEERING

MATERIALS

BN 978-0-444-52787-5

PY 2010

BP 2192

EP 2204

UT WOS:000333463900020

ER

PT J

AU Gulzar, A

Zhao, LZ

Xue, RJ

Shahzad, K

Zhao, DQ

Wang, WH

AF Gulzar, A.

Zhao, L. Z.

Xue, R. J.

Shahzad, K.

Zhao, D. Q.

Wang, W. H.

TI Correlation between flow units and crystallization in metallic glasses

SO JOURNAL OF NON-CRYSTALLINE SOLIDS

AB The relationship between metastable and intrinsic inhomogeneous amorphous structure and the crystallization of metallic glasses (MGs) is not yet well explored. We have found a correlation between the flow unit, which can model the structural heterogeneity and explain the thermal relaxations, glass transition and plastic deformation phenomenon in MGs, and enthalpy of crystallization of MGs. The results help to understand the atomic scale evolution during nucleation and growth in the crystallization process and the nature of metallic glass. (C) 2017 Elsevier B.V. All rights reserved.

SN 0022-3093

EI 1873-4812

PD APR 1

PY 2017

VL 461

BP 61

EP 66

DI 10.1016/j.jnoncrysol.2017.01.042

UT WOS:000396955700007

ER

PT S

AU Louzguine-Luzgin, DV

AF Louzguine-Luzgin, Dmitri V.

BE Zhukov, A

TI Bulk Metallic Glasses and Glassy/Crystalline Materials

SO NOVEL FUNCTIONAL MAGNETIC MATERIALS: FUNDAMENTALS AND APPLICATIONS

SE Springer Series in Materials Science

OI Louzguine-Luzgin, Dmitri/0000-0001-5716-4987

SN 0933-033X

EI 2196-2812

BN 978-3-319-26106-5; 978-3-319-26104-1

PY 2016

VL 231

BP 397

EP 440

DI 10.1007/978-3-319-26106-5_10

D2 10.1007/978-3-319-26106-5

UT WOS:000398209700011

ER

PT S

AU Louzguine-Luzgin, DV

Inoue, A

AF Louzguine-Luzgin, Dmitri V.

Inoue, Akihisa

BE Buschow, KHJ

TI BULK METALLIC GLASSES: FORMATION, STRUCTURE, PROPERTIES, AND

APPLICATIONS

SO HANDBOOK OF MAGNETIC MATERIALS, VOL 21

SE Handbook of Magnetic Materials

RI Inoue, Akihisa/E-5271-2015

OI Louzguine-Luzgin, Dmitri/0000-0001-5716-4987

SN 1567-2719

BN 978-0-444-59595-9; 978-0-444-59593-5

PY 2013

VL 21

BP 131

EP 171

DI 10.1016/B978-0-444-59593-5.00003-9

UT WOS:000330795400003

ER

PT J

AU Suryanarayana, C

Inoue, A

AF Suryanarayana, C.

Inoue, A.

TI Iron-based bulk metallic glasses

SO INTERNATIONAL MATERIALS REVIEWS

AB The current status of research and development in Fe-based bulk metallic glasses (BMGs) is reviewed. Bulk metallic glasses are relatively new materials possessing a glassy structure and large section thickness. These materials have an exciting combination of properties such as high mechanical strength, good thermal stability, large supercooled liquid region and potential for easy forming. Ever since the first synthesis of an Fe-based BMG in an Fe-Al-Ga-P-C-B system in 1995, there has been intense activity on the synthesis and characterisation of Fe-based BMGs. These BMGs exhibit some unique characteristics which have not been obtained in conventional Fe-based crystalline alloys. This uniqueness has led to practical uses of these bulk glassy alloys as soft magnetic and structural materials. This review presents the recent results on the glass-forming ability, structure, thermal stability, mechanical properties, corrosion behaviour, soft magnetic properties and applications of Fe-based bulk glassy alloys developed during the last 15 years. This review also highlights the advanced analysis of their properties which has contributed significantly to the progress in understanding and developing of the Fe-based BMGs. The future prospects of Fe-based BMGs have also been presented.

RI Inoue, Akihisa/E-5271-2015; IQBAL, MUHAMMAD/I-8029-2015

SN 0950-6608

PD APR

PY 2013

VL 58

IS 3

BP 131

EP 166

DI 10.1179/1743280412Y.0000000007

UT WOS:000315152700001

ER

PT J

AU Li, G

Sun, LL

Wang, WK

AF Li, G

Sun, LL

Wang, WK

TI Effects of gravity field on glass forming ability in ZrTiCuNiBe alloy

SO CHINESE SCIENCE BULLETIN

AB The solidification and glass forming ability of Zr41Ti14Cu12.5Ni10Be22.5 bulk glassy forming alloy is investigated by Bridgman unidirectional solidification at different growth velocities under different gravity field orientations. Large differences in glass formation, undercooling and crystallization morphology on different solidification conditions have been found and discussed from the point of view of gravity induced convection. The results are useful for understanding the nucleation and growth in the melt and glass formation mechanism in the alloy.

SN 1001-6538

PD DEC

PY 2001

VL 46

IS 24

BP 2048

EP 2050

UT WOS:000173323300007

ER

PT J

AU Yuan, B

Li, JJ

Qiao, JW

AF Yuan, Bo

Li, Jiao-jiao

Qiao, Jun-wei

TI Statistical analysis on strain-rate effects during serrations in a

Zr-based bulk metallic glass

SO JOURNAL OF IRON AND STEEL RESEARCH INTERNATIONAL

CT 17th IUMRS International Conference in Asia

CY OCT 20-24, 2016

CL Qingdao, PEOPLES R CHINA

SP IUMRS

AB By means of statistical analysis, the deformation mechanisms taking place in elastic loading and plastic shearing stages during serrated flows on the stress-strain curves for hulk metallic glasses were studied comprehensively. Normalized serration number presented a linear increasing tendency with the decrease of applied strain rates due to the reduction of free volumes. An excellent plastic deformation was illustrated from the influences of structure arrangement with activation energy. By using mean-field theory (MFT), maximum elastic energy density at different strain rates could he predicted by MFT besides maximum stress drops during serrations. These results were helpful for understanding the serrated flow behavior or designing decent schemes to improve the plasticity of hulk metallic glasses at room temperature.

SN 1006-706X

EI 2210-3988

PD APR

PY 2017

VL 24

IS 4

BP 455

EP 461

DI 10.1016/S1006-706X(17)30069-9

UT WOS:000401088300018

ER

PT J

AU Gu, XJ

Poon, SJ

Shiflet, GJ

Lewandowski, JJ

AF Gu, X. J.

Poon, S. J.

Shiflet, G. J.

Lewandowski, J. J.

TI Ductile-to-brittle transition in a Ti-based bulk metallic glass

SO SCRIPTA MATERIALIA

AB The effects of changes in cooling rate on the density, elastic constants and fracture energy have been determined for Ti40Zr25Cu12Ni3Be20 bulk metallic glass. It is shown that changes in cooling rate directly affect the measured density and elastic constants, while the fracture energy, ranging from 148.9 to only 0.2 kJ m(-2), is also shown to correlate with these changes in cooling rate/density/elastic constants. The critical Poisson's ratio for toughness in this alloy system is 0.35, somewhat higher than that obtained in other metallic glasses, although consistent with recent theoretical predictions. Further correlations with these changes in toughness on the fracture surface appearance are provided. (C) 2009 Acta Materialia Inc. Published by Elsevier Ltd. All rights reserved.

RI Lewandowski, John/S-3815-2017

OI Lewandowski, John/0000-0002-3389-2637

SN 1359-6462

PD JUN

PY 2009

VL 60

IS 11

BP 1027

EP 1030

DI 10.1016/j.scriptamat.2009.02.037

UT WOS:000265359900027

ER

PT J

AU Fu, H

Zou, M

AF Fu, H.

Zou, M.

TI Magnetic and magnetocaloric properties of ternary Gd-Co-Al bulk metallic

glasses

SO JOURNAL OF ALLOYS AND COMPOUNDS

AB Bulk metallic glasses (BMGs) with compositions of Gd(55)Co(x)Al(45-x) (15 <= x <= 30) and Gd(60)Co(y)Al(40-y) (15 <= y <= 30) were synthesized by an injection casting technique. Temperature dependence of magnetization of the BMGs indicates that their Curie temperatures can be tailored between 96 and 143 K by varying Gd and Co concentration. The magnetic entropy changes of the BMGs are greater than 9.0 J/kg K except for the Gd(55)Co(30)Al(15) glass that exhibits a reduced magnetization due to its large Co content. The relative cooling powers of the BMGs are greater than those of any other crystalline compounds and decrease with the increasing Co content. (C) 2011 Elsevier B.V. All rights reserved.

SN 0925-8388

PD MAR 31

PY 2011

VL 509

IS 13

BP 4613

EP 4616

DI 10.1016/j.jallcom.2011.01.126

UT WOS:000288833100031

ER

PT J

AU Calin, M

Zhang, LC

Eckert, J

AF Calin, M.

Zhang, L. C.

Eckert, J.

TI Tailoring of microstructure and mechanical properties of a Ti-based bulk

metallic glass-forming alloy

SO SCRIPTA MATERIALIA

AB To achieve plasticity of Ti-based bulk metallic glasses (BMGs) without sacrificing their high strength, the microstructure of Ti50Cu20Ni24Sn3Si2B1 alloy was tailored to form in situ composites. The cast rods consist of micrometer-sized NiTi dendrites surrounded by a thin metallic glass network along with a fine CuTi3 phase. The rods show high strengths of similar to 1900-2250 MPa and plastic strains of similar to 4.5-7.8%, which are superior to the monolithic Ti-based BMGs. The microstructure-property correlations and the deformation behavior are investigated. (C) 2007 Acta Materialia Inc. Published by Elsevier Ltd. All rights reserved.

RI Zhang, Lai-Chang/B-9769-2011; Calin, Mariana/J-6398-2015

OI Zhang, Lai-Chang/0000-0003-0661-2051;

SN 1359-6462

PD DEC

PY 2007

VL 57

IS 12

BP 1101

EP 1104

DI 10.1016/j.scriptamat.2007.08.018

UT WOS:000250355500012

ER

PT S

AU Hiki, Y

Tanahashi, M

Takeuchi, S

AF Hiki, Y.

Tanahashi, M.

Takeuchi, S.

BE Igata, N

Takeuchi, S

TI Search for high damping metallic glasses

SO HIGH DAMPING MATERIALS II

SE KEY ENGINEERING MATERIALS

CT 2nd International Symposium on High Damping Materials

CY SEP 09-10, 2005

CL Kyoto, JAPAN

SP Japan Soc Promot Sci, Kajima Fdn, Ogasawara Fdn, Suzuki Fdn

AB In a hydrogen-doped metallic glass, there appear low-temperature and high-temperature internal friction peaks respectively associated with a point-defect relaxation and the crystallization. The high-temperature-side slope of low-temperature peak and also the low-temperature-side slope of high-temperature peak enhance the background internal friction near the room temperature. A hydrogen-doped Mg-base metallic glass was proposed as a high-damping material to be used near and somewhat above the room temperature. Stability of the high damping was also checked.

SN 1013-9826

BN 0-87849-406-5

PY 2006

VL 319

BP 151

EP 155

DI 10.4028/www.scientific.net/KEM.319.151

UT WOS:000241411000024

ER

PT J

AU Wang, ZR

Dong, DD

Qiang, JB

Wang, Q

Wang, YM

Dong, C

AF Wang ZengRui

Dong DanDan

Qiang JianBing

Wang Qing

Wang YingMin

Dong Chuang

TI Ti-based glassy alloys in Ti-Cu-Zr-Sn system

SO SCIENCE CHINA-PHYSICS MECHANICS & ASTRONOMY

AB Bulk amorphous formation in Ti-Cu-based multicomponent alloys, free of Ni, Pd and Be elements, were studied using the cluster-plus-glue-atom model. The basic cluster formula was revealed as [Ti9Cu6]Cu-3 to explain the best binary glass forming composition Ti50Cu50=Ti9Cu9, where the CN14 rhombi-dodecahedron Ti9Cu6 was the principal cluster in the devitrification phase CuTi. This basic cluster formula was further alloyed with Zr and Sn and a critical glass forming ability was reached at (Ti7.2Zr1.8)(Cu8.72Sn0.28) and (Ti7.2Zr1.8)(Cu8.45Sn0.55) up to 5 mm in diameter by suction casting, which was the largest in Ti-Cu-based and Ni-, Pd- and Be-free alloys.

SN 1674-7348

PD JUL

PY 2013

VL 56

IS 7

BP 1419

EP 1422

DI 10.1007/s11433-013-5104-7

UT WOS:000320502800026

ER

PT J

AU Dambatta, MS

Izman, S

Yahaya, B

Lim, JY

Kurniawan, D

AF Dambatta, M. S.

Izman, S.

Yahaya, B.

Lim, J. Y.

Kurniawan, D.

TI Mg-based bulk metallic glasses for biodegradable implant materials: A

review on glass forming ability, mechanical properties, and

biocompatibility

SO JOURNAL OF NON-CRYSTALLINE SOLIDS

AB Amorphous Mg-based bulk metallic glasses (BMGs) are relatively new materials for various engineering applications because of their superior mechanical properties and corrosion resistance. Recently, BMGs attract significant attention as a new class of biodegradable materials. Some of their properties have been observed to be superior as compared to their crystalline counterparts. The current state of the art of Mg-based BMGs development for biomedical implant applications is still focused on the glass forming ability and their formation mechanism. Some types of Mg-based BMGs demonstrate very encouraging results in terms of biodegradability and biocompatibility performances. However, there are still many Mg-based BMGs in development stage where toxic alloying elements are used. This study reviews the characteristics and role of elements with good glass forming ability toward development of Mg-based bulk metallic glasses. It discusses the glass forming ability, mechanical properties, corrosion behavior, and biocompatibility of previously reported Mg-based BMGs. It ends with the proposed strategy for future development of Mg-based BMGs for biodegradable implant utilization. (C) 2015 Elsevier B.V. All rights reserved.

RI Kurniawan, Denni/C-2703-2008

OI Kurniawan, Denni/0000-0002-4179-0454

SN 0022-3093

EI 1873-4812

PD OCT 15

PY 2015

VL 426

BP 110

EP 115

DI 10.1016/j.jnoncrysol.2015.07.018

UT WOS:000359889000019

ER

PT J

AU Fan, C

Inoue, A

AF Fan, C

Inoue, A

TI Ductility of bulk nanocrystalline composites and metallic glasses at

room temperature

SO APPLIED PHYSICS LETTERS

AB Mechanical properties of bulk Zr60Cu20Pd10Al10 nanocrystalline composite and Zr55Ni5Cu30Al10 metallic glass were measured by compression tests at room temperature. The Zr60Cu20Pd10Al10 as-quenched alloy obviously exhibits plastic strain while no distinct plastic deformation is recognized in the Zr55Ni5Cu30Al10 metallic glass. Moreover, the plastic strain increased by increasing the volume fraction of nanocrystals and achieved maximum value in the early stage of the nanocrystallization. High-resolution electron microscopy showed that, different from the microstructure of Zr55Ni5Cu30Al10 metallic glass, nanocrystals with main grain sizes of about 2 nm were embedded in the amorphous matrix of the bulk Zr60Cu20Pd10Al10 alloy which showed the maximum plastic strain. (C) 2000 American Institute of Physics. [S0003-6951(00)01027-5].

RI Inoue, Akihisa/E-5271-2015

SN 0003-6951

PD JUL 3

PY 2000

VL 77

IS 1

BP 46

EP 48

DI 10.1063/1.126872

UT WOS:000087889700016

ER

PT J

AU Wang, YC

Wang, YR

Wei, BC

Li, WH

Sun, YF

AF Wang Yinchun

Wang Yuren

Wei Bingchen

Li Weihuo

Sun Yufeng

TI Kinetics of glass transition and crystallization in carbon nanotube

reinforced Mg-Cu-Gd bulk metallic glass

SO JOURNAL OF RARE EARTHS

AB Mg65Cu25Gd10 bulk metallic glass and its carbon nanotube reinforced composite were prepared. Differential scanning calorimeter (DSc) was used to investigate the kinetics of glass transition and crystallization processes. The influence of CNTs addition to the glass matrix on the glass transition and crystallization kinetics was studied. It is shown that the kinetic effect on glass transition and crystallization are preserved for both the monothetic glass and its glass composite. Adding CNTs in to the glass matrix reduces the influence of the heating rate on the crystallization process. In addition, the CNTs increase the energetic barrier for the glass transition. This results in the decrease of GFA. The mechanism of the GFA decrease was also discussed.

RI Sun, Yufeng/R-3800-2016; Wang, Yuren/B-2124-2013

OI Sun, Yufeng/0000-0002-6076-1026;

SN 1002-0721

PD JUN

PY 2006

VL 24

IS 3

BP 327

EP 331

AR PII 1002-0721(2006)03-0327-05

DI 10.1016/S1002-0721(06)60118-X

UT WOS:000239079000014

ER

PT J

AU Bakai, SA

Volchok, OI

Stoev, PI

Kamyshanchenko, NV

Kungurtsev, ES

AF Bakai, S. A.

Volchok, O. I.

Stoev, P. I.

Kamyshanchenko, N. V.

Kungurtsev, E. S.

TI Effect of the ultrasound action on the acoustic emission and mechanical

properties of zirconium-based bulk metallic glasses

SO ACOUSTICAL PHYSICS

AB The effect of preliminary ultrasound action on the mechanical properties and features of the structure of zirconium-based bulk metallic glasses has been studied by the method of acoustic emission under uniaxial compression. Results of studies have been interpreted using the polycluster model of the structure of amorphous metallic alloys. Analysis of the obtained data has allowed us to substantiate the mechanism of the change in the structure and strength of metallic glasses as a result of alternating-sign mechanical loading with an ultrasound frequency of 20 kHz.

OI Stoev, Petr/0000-0001-7942-5850

SN 1063-7710

PD MAY

PY 2012

VL 58

IS 3

BP 277

EP 280

DI 10.1134/S1063771012020029

UT WOS:000304614500003

ER

PT J

AU Battezzati, L

AF Battezzati, Livio

TI On correlations of indexes of melt fragility in metallic glass-formers

SO REVIEWS ON ADVANCED MATERIALS SCIENCE

CT 13th International Symposium on Metastable and Nano-Materials

(ISMANAM-2006)

CY AUG 27-31, 2006

CL Warsaw Univ Technol, Fac Mat Sci & Engn, Warsaw, POLAND

HO Warsaw Univ Technol, Fac Mat Sci & Engn

AB Indexes of thermodynamic fragility show discrepancies with respect to kinetic ones for metallic glass formers (slope of viscosity at the glass transition, m, vs. reduced temperatures at which specific entropy values are obtained). On the other hand, the reduced span of the glass transition range scales well with m.

A correlation, recently reported, between melt strength/fragility and the elastic moduli of glassy materials (namely the ratio of the bulk to shear modulus) is actually not stringent. There is, however, the possibility of distinguishing among various types of glasses, e. g. inorganic, organic, metal-metal and metal-metalloid.

OI Battezzati, Livio/0000-0003-1628-0409

SN 1606-5131

PD JUN

PY 2008

VL 18

IS 2

BP 184

EP 189

UT WOS:000257256400019

ER

PT J

AU Wang, XY

Chen, Y

Zhang, NY

Zhao, LP

Pang, YT

Wang, WK

AF Wang Xiu-Ying

Chen Ying

Zhang Ning-Yu

Zhao Li-Ping

Pang Yan-Tao

Wang Wen-Kui

TI Effect of pressure on the glass transition and crystallization dynamics

of Zr46.75Ti8.25Cu7.5Ni10Be27.5 bulk amorphous alloy

SO ACTA PHYSICA SINICA

AB The glass transition and crystallization behavior of Zr-46.75 Ti-8.25 Cu-7.5 Ni-10 Be-27.5 bulk amorphous alloy are investigated by X-ray diffraction and differential scanning calorimetry under high temperature and high pressure. The results show that the free volume and enthalpy and crystallization activation energy decrease with increasing pressure.

SN 1000-3290

PD JUL

PY 2007

VL 56

IS 7

BP 4004

EP 4008

UT WOS:000248134500060

ER

PT S

AU Busch, R

Bakke, E

Johnson, WL

AF Busch, R

Bakke, E

Johnson, WL

BE Fiorani, D

Magini, M

TI On the glass forming ability of bulk metallic glasses

SO SYNTHESIS AND PROPERTIES OF MECHANICALLY ALLOYED AND NANOCRYSTALLINE

MATERIALS, PTS 1 AND 2 - ISMANAM-96

SE Materials Science Forum

CT International Symposium on Metastable, Mechanically Alloyed and

Nanocrystalline Materials (ISMANAM-96)

CY MAY 20-24, 1996

CL ROME, ITALY

SP Natl Agcy Energy Environm & New Technol, CNR, CSM, INCM q, INFM, CNR, GNSM, CNR, PF MSTA, CNR, Comitato Chim, PROMEA, MIYASHITA Fdn Mat Sci, ZOZ, FRITSCH ECOSCI, SIEMENS, RIVOIRA, FBL, SAGES GETTERS, PERKIN ELMER, SEIFERT SIARS, FLUMAC, OXFORD INSTRUMENTS CRIOGENIA, KETTLEY, BNL Bank

AB During the past few years, a number of multicomponent metallic alloys have been found to exhibit an excellent glass forming ability when cooled from the melt with rates of less than 100 K/s and as low as 1 K/s. This permits the formation of bulk amorphous metallic materials with sizes of up to several centimeters in the smallest dimension. In this article several factors are discussed that contribute to the high glass forming ability of these materials. Results based on levitation experiments, differential scanning calorimetry, viscosity, atom probe field ion microscopy-, and electron microscopy are presented. The thermodynamics as well as the kinetics of bulk metallic glass forming liquids, expressed by the Gibbs free energy and the viscosity, respectively, favor glass forming ability. In addition, phase separation in the undercooled liquid state becomes an important factor in understanding the nucleation process of crystals. Finally, the role of the different species in the multicomponent material is discussed based on solid state reactions in multicomponent systems.

SN 0255-5476

BN 0-87849-750-1

PY 1997

VL 235-2

BP 327

EP 335

DI 10.4028/www.scientific.net/MSF.235-238.327

PN 1 & 2

UT WOS:A1997BH18D00051

ER

PT J

AU Mondal, K

Kumar, G

Ohkubo, T

Oishi, K

Mukai, T

Hono, K

AF Mondal, K.

Kumar, G.

Ohkubo, T.

Oishi, K.

Mukai, T.

Hono, K.

TI Large apparent compressive strain of metallic glasses

SO PHILOSOPHICAL MAGAZINE LETTERS

AB Very high plastic strains exceeding 20% in uniaxial compression tests, which show an inflection in the stress-strain curves of Zr-based bulk metallic glasses, are shown to be a spurious effect due to mechanical interlocking of cracks with shear bands. This type of stress-strain behaviour is misinterpreted as an actual deformation in the literature. The effect of mechanical interlocking is explained by fractographic analysis.

RI Totsukawa, Nobuhisa/D-2028-2017; Hono, Kazuhiro/B-9202-2008; Mukai,

Toshiji/F-9570-2014; kumar, golden/F-5443-2010

OI Hono, Kazuhiro/0000-0001-7367-0193; Mukai, Toshiji/0000-0002-9628-5762;

SN 0950-0839

PY 2007

VL 87

IS 9

BP 625

EP 635

DI 10.1080/09500830701413904

UT WOS:000248450300002

ER

PT J

AU Kramer, L

Champion, Y

Kormout, KS

Maier-Kiener, V

Pippan, R

AF Kraemer, Lisa

Champion, Yannick

Kormout, Karoline S.

Maier-Kiener, Verena

Pippan, Reinhard

TI Bulk metallic dual phase glasses by severe plastic deformation

SO INTERMETALLICS

AB Two different metallic glass powders were consolidated and deformed via high-pressure torsion to synthesize amorphous dual phase composites. The influence of volume fraction of the two amorphous phases and the applied shear strain was investigated. By varying the applied strain, the dimensions of the phases could be systematically varied from the micro- to the nanometer regime and at the highest applied strain even a transition to a single phase state could be observed. The study illustrates the potential of producing novel bulk metallic glasses by deformation-induced mixing which are not accessible by the classical casting route.

SN 0966-9795

EI 1879-0216

PD MAR

PY 2018

VL 94

BP 172

EP 178

DI 10.1016/j.intermet.2017.12.005

UT WOS:000425579200023

ER

PT J

AU Fujita, K

Inoue, A

Zhang, T

Nishiyama, N

AF Fujita, K

Inoue, A

Zhang, T

Nishiyama, N

TI Anelastic behavior under tensile and shearing stresses in bulk metallic

glasses

SO MATERIALS TRANSACTIONS

AB It is known that an anelastic deformation occurs more remarkably for amorphous ribbon alloys than for crystalline metallic alloys by the tensile test. In addition, the result of the molecular dynamics simulation on tensile and shearing tests for Cu single component amorphous metal has indicated that the anelastic deformation occurs more remarkably under the shearing stress rather than under the tensile stress. In this report, tensile and torsional tests were actually performed for bulk metallic glasses to examine the difference in the anelastic behavior under shearing and tensile stresses. Single-phase bulk metallic glasses, La60Al20Ni10Cu5Co5, Pd40Cu30Ni10P20 and Zr55Cu30Ni5Al10 (at%), were chosen together with a steel, JIS SGD 400-D, as a representative of metallic crystals. The test specimens were a round bar shape and the diameters of a parallel gage section were 4 to 10 mm. No anelastic behavior was observed for the steel under tensile and shearing stresses. Although the metallic glasses did not exhibit distinct anelastic deformation under the tensile stress, the shearing stress mode leads to a significant anelastic deformation even at low stress level. The amount of the anelastic deformation increases with an increase in the shearing stress level.

RI Nishiyama, Nobuyuki/C-8228-2015; Inoue, Akihisa/E-5271-2015; Zhang,

Tao/O-4911-2014

SN 1345-9678

EI 1347-5320

PD AUG

PY 2002

VL 43

IS 8

BP 1957

EP 1960

DI 10.2320/matertrans.43.1957

UT WOS:000177930100030

ER

PT J

AU Li, H

Subhash, G

Gao, XL

Kecskes, LJ

Dowding, RJ

AF Li, H

Subhash, G

Gao, XL

Kecskes, LJ

Dowding, RJ

TI Negative strain rate sensitivity and compositional dependence of

fracture strength in Zr/Hf based bulk metallic glasses

SO SCRIPTA MATERIALIA

AB Quasistatic and dynamic compression tests on Zr/Hf based bulk metallic glasses revealed decreasing fracture strength with increasing strain rate and decreasing Hf content. It is believed that dynamic loading promotes crack nucleation immediately following shear band initiation whereas quasistatic loading promotes shear bands to grow to maturity. (C) 2003 Acta Materialia Inc. Published by Elsevier Ltd. All rights reserved.

RI Subhash, Ghatu/J-4851-2017; Kecskes, Laszlo/F-6880-2014; Dowding,

Robert/F-1469-2015

OI Subhash, Ghatu/0000-0002-5996-0909; Kecskes, Laszlo/0000-0002-1342-3729;

Dowding, Robert/0000-0002-4763-2131; Gao, Xin-Lin/0000-0003-2280-4942

SN 1359-6462

PD DEC

PY 2003

VL 49

IS 11

BP 1087

EP 1092

DI 10.1016/j.scriptamat.2003.08.012

UT WOS:000185451400006

ER

PT J

AU Fan, C

Yan, HG

Liu, CT

Li, HQ

Liaw, PK

Ren, Y

Egami, T

AF Fan, Cang

Yan, H. G.

Liu, C. T.

Li, H. Q.

Liaw, P. K.

Ren, Y.

Egami, T.

TI Changes in the atomic structure through glass transition observed by

X-ray scattering

SO INTERMETALLICS

AB The glass transition involves a minor change in the internal energy, and yet the physical and mechanical properties of a glass change dramatically. In order to determine the evolution of the atomic structure through the glass transition, we employed in-situ synchrotron X-ray scattering measurements as a function of temperature on a model material: Zr-Cu-Al metallic glass. We found that the thermal expansion at the atomic level is smaller than the macroscopic thermal expansion, and significantly increases above the glass transition temperature. The observed changes in the pair-distribution function (PDF) are explained in terms of the fluctuations in the local atomic volume and their change through the glass transition. (C) 2012 Elsevier Ltd. All rights reserved.

OI Liu, Chain Tsuan/0000-0001-7888-9725

SN 0966-9795

PD APR

PY 2012

VL 23

BP 111

EP 115

DI 10.1016/j.intermet.2012.01.003

UT WOS:000301275000017

ER

PT J

AU Meng, SY

Ling, HB

Li, Q

Zhang, JJ

AF Meng, Shangyong

Ling, Haibo

Li, Qiang

Zhang, Jijun

TI Development of Fe-based bulk metallic glasses with high saturation

magnetization

SO SCRIPTA MATERIALIA

AB Aimed at developing new Fe-based bulk metallic glasses (BMGs) with high saturation magnetization (J(s)), a new series of Fe-based BMG have been developed by fluxing treatment and J-quenching techniques. Among these Fe-based BMGs, (Fe90Co10)(82)P6C7B3Si2 BMG with the diameter 1.0 mm has been prepared and exhibited a very high J(s) of 1.65 T (176 emu g(-1)), which is the largest reported for Fe-based BMG so far. Additionally, the compositional dependence of glass formation ability and the magnetic properties has been discussed. (C) 2014 Acta Materialia Inc. Published by Elsevier Ltd. All rights reserved.

RI Li, Qiang/D-1865-2011

OI Li, Qiang/0000-0002-1891-5490

SN 1359-6462

PD JUN 15

PY 2014

VL 81

BP 24

EP 27

DI 10.1016/j.scriptamat.2014.02.018

UT WOS:000335622900007

ER

PT J

AU Macht, MP

Wanderka, N

Wei, Q

Sieber, I

Deyneka, N

AF Macht, MP

Wanderka, N

Wei, Q

Sieber, I

Deyneka, N

TI Tendency of primary crystal formation in ZrTiCuNiBe metallic bulk

glasses

SO MATERIALS SCIENCE AND ENGINEERING A-STRUCTURAL MATERIALS PROPERTIES

MICROSTRUCTURE AND PROCESSING

CT 10th International Conference on Rapidly Quenched and Metastable

Materials (RQ10)

CY AUG 23-27, 1999

CL BANGALORE, INDIA

SP Indian Inst Sci

AB Bull: metallic glasses can be produced from the liquid melt at low cooling rates of < 10(3) K/s. Although the bulk glasses appear to be fully amorphous when analyzed by X-ray diffraction (XRD) frequently small volume fractions of primary crystals are found. This problem is of various importance for the different bulk glass forming systems and even for glasses of the same family, but with different composition, For instance, the Zr41Ti14Cu12.5Ni10Be22.5-bulk glass is nearly free of crystals, whereas the thermally more stable Zr46.8Ti8.2Cu7.5Ni10Be27.5-bulk glass always contains a larger number of crystals, when produced under the same conditions. in the present work, the composition, structure and morphology of primary crystals in both ZrTiCuNiBe-bulk glasses are analyzed. These crystalline phases are compared with the equilibrium phases, which crystallize during very slow cooling of the liquid melt. The tendency for primary crystal formation is discussed in terms of constitutional arguments using a description of the ZrTiCuNiBe alloy by a quasi-ternary (Zr, Ti)-(Cu, Ni)-Be system, (C) 2001 Elsevier Science B.V, All rights reserved.

SN 0921-5093

PD MAY 31

PY 2001

VL 304

SI SI

BP 701

EP 705

DI 10.1016/S0921-5093(00)01573-2

UT WOS:000168457100134

ER

PT J

AU Abbasi, M

Gholamipour, R

Shahri, F

AF Abbasi, M.

Gholamipour, R.

Shahri, F.

TI Glass forming ability and mechanical properties of Nb-containing

Cu-Zr-Al based bulk metallic glasses

SO TRANSACTIONS OF NONFERROUS METALS SOCIETY OF CHINA

AB Mechanical properties of (Cu50Zr43Al7)(100-x)Nb-x (x=0,1,3,6,9) bulk metallic glasses rods with a diameter of 2.5 mm prepared by suction casting method were studied. The results of uniaxial compression tests at room temperture show that the best mechanical properties of 2.8% and 1.98 GPa for plastic strain and fracture strength, respectively, in the sample with x=3. Microstructure, fracture surface and shear bands of the samples were observed by SEM and XRD methods.

SN 1003-6326

EI 2210-3384

PD JUL

PY 2013

VL 23

IS 7

BP 2037

EP 2041

DI 10.1016/S1003-6326(13)62693-X

UT WOS:000325599600023

ER

PT J

AU Madge, SV

Wada, T

Louzguine-Luzgin, DV

Greer, AL

Inoue, A

AF Madge, S. V.

Wada, T.

Louzguine-Luzgin, D. V.

Greer, A. L.

Inoue, A.

TI Oxygen embrittlement in a Cu-Hf-Al bulk metallic glass

SO SCRIPTA MATERIALIA

AB Compression testing shows that at low-oxygen levels (240 ppm) the Cu(49)Hf(42)Al(9) glass deforms predominantly by shear and is quite tough. Specimens with higher oxygen levels (1700 ppm) have oxygen-containing dendrites in an amorphous matrix and show no plasticity. Importantly, the dominant fracture mode is not shear but local tensile failure. Although there is a slight reduction in the toughness of the glassy matrix, the severe embrittlement is primarily because of the brittle dendrites that facilitate crack initiation and propagation. (C) 2009 Acta Materialia Inc. Published by Elsevier Ltd. All rights reserved.

RI Greer, Alan Lindsay/G-1977-2011; Wada, Takeshi/B-2431-2015; Inoue,

Akihisa/E-5271-2015; LOUZGUINE, Dmitri/D-2492-2010; Greer,

Lindsay/E-9433-2017

OI LOUZGUINE, Dmitri/0000-0001-5716-4987; Madge,

Shantanu/0000-0001-7996-8652

SN 1359-6462

PD SEP

PY 2009

VL 61

IS 5

BP 540

EP 543

DI 10.1016/j.scriptamat.2009.05.018

UT WOS:000268426700024

ER

PT J

AU Jiang, F

Wang, ZJ

Zhang, ZB

Sun, J

AF Jiang, F

Wang, ZJ

Zhang, ZB

Sun, J

TI Formation of Zr-based bulk metallic glasses from low purity materials by

scandium addition

SO SCRIPTA MATERIALIA

AB Zr55Al10CU30Ni5 bulk metallic glass (BMG) is formed by using low purity sponge zirconium, instead of high purity zirconium, and other high purity raw materials with a small amount of scandium addition. The results show that glass forming ability and thermal stability of the Zr55Al10CU30Ni5 alloy are improved with scandium addition. Compressive fracture strength is similar to that of BMG alloy produced with high purity raw materials. However, plasticity of BMGs with sponge zirconium and scandium addition deteriorates. (c) 2005 Acta Materialia Inc. Published by Elsevier Ltd. All rights reserved.

RI jiang, Feng/D-1427-2012

OI jiang, Feng/0000-0003-3651-8007

SN 1359-6462

PD SEP

PY 2005

VL 53

IS 5

BP 487

EP 491

DI 10.1016/j.scriptamat.2005.05.003

UT WOS:000230617200003

ER

PT J

AU Huang, H

Zhao, HW

AF Huang, Hu

Zhao, Hongwei

TI Indenter Geometry Affecting Indentation Behaviors of the Zr-Based Bulk

Metallic Glass

SO MATERIALS TRANSACTIONS

AB Indenter dependent indentation behaviors of the Zr-based bulk metallic glass, such as adhesion, serrated flows, shear bands, residual indent morphologies, were found and analyzed in this paper. An interesting phenomenon is presented that adhesion of the Zr-based bulk metallic glass appears during the indentation test using the cube-corner indenter but it's not observed when using the Berkovich indenter. Corresponding to the adhesion behavior, "dirty" but new surfaces with the nano-scale dimple structure are formed by the cube-corner indenter while relatively smooth surfaces with few discrete shear bands are obtained by the Berkovich indenter. Indentation experiments indicate that the adhesion force between the cube-corner indenter and the Zr-based bulk metallic glass depends on the loading rate, the maximum penetration load and the cyclic loading number. These phenomena will enhance understanding of shear band formation, shear-induced softening, and adhesion of bulk metallic glasses.

RI Huang, Hu/A-3650-2012

OI Huang, Hu/0000-0002-3778-4457

SN 1345-9678

EI 1347-5320

PD SEP

PY 2014

VL 55

IS 9

BP 1400

EP 1404

DI 10.2320/matertrans.M2014136

UT WOS:000343636600006

ER

PT J

AU Qiao, JC

Pelletier, JM

Blandin, JJ

Gravier, S

AF Qiao, J. C.

Pelletier, J. M.

Blandin, J. J.

Gravier, S.

TI High temperature deformation in a lanthanum based bulk metallic glass

showing a pronounced secondary relaxation

SO MATERIALS SCIENCE AND ENGINEERING A-STRUCTURAL MATERIALS PROPERTIES

MICROSTRUCTURE AND PROCESSING

AB The deformation at high temperature of a La60Ni15Al25 bulk metallic glass has been investigated. In the experimental domain in which homogeneous deformation could be obtained, activation energies in agreement with what is usually measured for metallic glasses with similar glass transition temperatures were identified but particularly large values of activation volumes were measured. Such values of activation volumes were attributed to the pronounced secondary relaxation displayed by the investigated glass, suggesting the existence of structural heterogeneities in the microstructure. (C) 2013 Elsevier B.V. All rights reserved.

SN 0921-5093

EI 1873-4936

PD DEC 1

PY 2013

VL 586

BP 57

EP 61

DI 10.1016/j.msea.2013.07.080

UT WOS:000326903800009

ER

PT J

AU Kang, SJ

Rittgen, KT

Kwan, SG

Park, HW

Bennewitz, R

Caron, A

AF Kang, S. J.

Rittgen, K. T.

Kwan, S. G.

Park, H. W.

Bennewitz, R.

Caron, A.

TI Importance of surface oxide for the tribology of a Zr-based metallic

glass

SO FRICTION

AB Thermally grown surface oxide layers dominate the single-asperity tribological behavior of a Zr60Cu30Al10 glass. Increase in oxidation time leads to an increased contribution of shearing and a corresponding decreased contribution of ploughing to friction. This change in the dominating friction and wear mechanism results in an overall minor decrease of the friction coefficient of oxidized surfaces compared to the metallic glass sample with native surface oxide. Our results demonstrate the importance of creating a stable oxide layer for practical applications of metallic glasses in micro-devices involving sliding contact.

SN 2223-7690

EI 2223-7704

PD MAR

PY 2017

VL 5

IS 1

BP 115

EP 122

DI 10.1007/s40544-017-0149-7

UT WOS:000399646000010

ER

PT S

AU Kelton, K

Greer, L

AF Kelton, Ken

Greer, Lindsay

BA Kelton, KF

Greer, AL

BF Kelton, KF

Greer, AL

TI Transformations in the Solid Phase

SO NUCLEATION IN CONDENSED MATTER: APPLICATIONS IN MATERIALS AND BIOLOGY

SE Pergamon Materials Series

RI Greer, Lindsay/E-9433-2017

SN 1470-1804

BN 978-0-08-091264-6; 978-0-08-042147-6

PY 2010

VL 15

BP 511

EP 586

DI 10.1016/S1470-1804(09)01514-4

UT WOS:000311273400015

ER

PT J

AU Fornell, J

Rossinyol, E

Surinach, S

Baro, MD

Li, WH

Sort, J

AF Fornell, J.

Rossinyol, E.

Surinach, S.

Baro, M. D.

Li, W. H.

Sort, J.

TI Enhanced mechanical properties in a Zr-based metallic glass caused by

deformation-induced nanocrystallization

SO SCRIPTA MATERIALIA

AB Bulk metallic glass with composition Zr62Cu18Ni10Al10 exhibits high yield stress, large elasticity and large plasticity when compressed. During nanoindentation, strain hardening is observed until the maximum applied load reaches 100 mN; for higher maximum loads the typical softening found in metallic glasses is evidenced. Transmission electron microscopy observation of the nanoindented and compressed samples reveals the occurrence of deformation-induced nanocrystallization, which is likely to be related to the mechanical properties observed in this alloy. (C) 2009 Acta Materialia Inc. Published by Elsevier Ltd. All rights reserved.

RI Lv, Henry/C-9692-2011; Baro, Maria Dolors/A-2096-2009; Rossinyol,

Emma/L-9413-2014; Surinach, Santiago/A-1749-2009; Sort,

Jordi/F-6582-2014

OI Baro, Maria Dolors/0000-0002-8636-1063; Rossinyol,

Emma/0000-0002-1656-1254; Surinach, Santiago/0000-0001-8125-0594; Sort,

Jordi/0000-0003-1213-3639

SN 1359-6462

PD JAN

PY 2010

VL 62

IS 1

BP 13

EP 16

DI 10.1016/j.scriptamat.2009.09.014

UT WOS:000272014100004

ER

PT S

AU Homer, ER

Li, L

Schuh, CA

AF Homer, Eric R.

Li, Lin

Schuh, Christopher A.

BE Weinberger, CR

Tucker, GJ

TI Kinetic Monte Carlo Modeling of Nanomechanics in Amorphous Systems

SO MULTISCALE MATERIALS MODELING FOR NANOMECHANICS

SE Springer Series in Materials Science

SN 0933-033X

BN 978-3-319-33480-6; 978-3-319-33478-3

PY 2016

VL 245

BP 441

EP 468

DI 10.1007/978-3-319-33480-6_14

D2 10.1007/978-3-319-33480-6

UT WOS:000399050100015

ER

PT J

AU Shen, Y

Ma, E

Xu, J

AF Shen, Yong

Ma, Evan

Xu, Jian

TI A group of Cu(Zr)-based BMGs with critical diameter in the range of 12

to 18 mm

SO JOURNAL OF MATERIALS SCIENCE & TECHNOLOGY

AB A group of Cu(Zr)-based bulk metallic glasses (BMGs) with critical diameter (D,) in the 12 to 18 mm range have been obtained using copper mould casting. In the Cu-Zr-Y-Al quaternary system, a new record of D-c=14 mm was established for Cu-based compositions, and 16 mm for compositions based on equi-atomic CuZr. Additional partial substitution of Hf for Zr further elevated the D-c to 18 mm at Cu42Zr43Hf1.5Y3.5Al10.

RI Ma, En/A-3232-2010

SN 1005-0302

PD MAR

PY 2008

VL 24

IS 2

BP 149

EP 152

UT WOS:000254637200002

ER

PT J

AU Gu, X

Shiflet, GJ

Guo, FQ

Poon, SJ

AF Gu, X

Shiflet, GJ

Guo, FQ

Poon, SJ

TI Mg-Ca-Zn bulk metallic glasses with high strength and significant

ductility

SO JOURNAL OF MATERIALS RESEARCH

AB The development of Mg-Ca-Zn metallic glasses with improved bulk glass forming ability, high strength, and significant ductility is reported. A typical size of at least 3-4 mm amorphous samples can be prepared using conventional casting techniques. By varying the composition, the mass density of these light metal based bulk amorphous alloys ranges from 2.0 to 3.0 g/cm(3). The typical measured microhardness is 2.16 GPa, corresponding to a fracture strength of about 700 MPa and specific strength of around 250-300 MPa cm(3)/g. Unlike other Mg- or Ca-based metallic glasses, the present Mg-Ca-Zn amorphous alloys show significant ductility.

RI Gu, Xiaofeng/E-8287-2013

OI Gu, Xiaofeng/0000-0001-8299-6451

SN 0884-2914

PD AUG

PY 2005

VL 20

IS 8

BP 1935

EP 1938

DI 10.1557/JMR.2005.0245

UT WOS:000231054800002

ER

PT J

AU Qiao, JW

Wang, S

Zhang, Y

Liaw, PK

Chen, GL

AF Qiao, J. W.

Wang, S.

Zhang, Y.

Liaw, P. K.

Chen, G. L.

TI Large plasticity and tensile necking of Zr-based

bulk-metallic-glass-matrix composites synthesized by the Bridgman

solidification

SO APPLIED PHYSICS LETTERS

AB The microstructures of the in situ bulk-metallic-glass-matrix composites are usually controlled by changing the alloy compositions. In this paper, Zr-based bulk-metallic-glass-matrix composites containing dendrites with a fixed composition of Zr(37.5)Ti(32.2)Nb(7.2)Cu(6.1)Be(17.0) are synthesized by the Bridgman solidification. The sizes and volume fractions of dendrites in the composites are controlled by adjusting the withdrawal velocities. A linear relationship between the spanning lengths of individual dendrites and the withdrawal velocities is established. Large plasticity and tensile necking can be obtained by only controlling the cooling condition.

RI ZHANG, Yong/B-7928-2009

OI ZHANG, Yong/0000-0002-6355-9923

SN 0003-6951

PD APR 13

PY 2009

VL 94

IS 15

AR 151905

DI 10.1063/1.3118587

UT WOS:000265285200022

ER

PT J

AU Misra, DK

Sohn, SW

Kim, WT

Kim, DH

AF Misra, Dinesh Kumar

Sohn, Sung Woo

Kim, Won Tae

Kim, Do Hyang

TI Rate-dependent serrated flow and plastic deformation in

Ti45Zr16Be20Cu10Ni9 bulk amorphous alloy during nanoindentation

SO SCIENCE AND TECHNOLOGY OF ADVANCED MATERIALS

CT 2nd International Workshop on Superconductivity in Diamond and Related

Materials

CY JUL, 2008

CL Natl Inst Mat Sci, Tsukuba, JAPAN

HO Natl Inst Mat Sci

AB The plastic deformation of Ti45Zr16Be20Cu10Ni9 bulk metallic glass has been investigated by nanoindentation performed with loads ranging from 10 to 200 mN in a wide range of loading rates. The plastic flow in the alloy exhibited conspicuous serrations at low loading rates. The serrations, however, became less prominent as the rate of indentation increased. Atomic force microscopy showed a significant pile-up of materials around the indents, indicating that a highly localized plastic deformation occurred under nanoindentation. The possible mechanism governing the plastic deformation in bulk metallic glass specimens is tentatively discussed in terms of strain-induced free volume.

SN 1468-6996

PD DEC

PY 2008

VL 9

IS 4

AR 045004

DI 10.1088/1468-6996/9/4/045004

UT WOS:000266428000021

PM 27878032

ER

PT J

AU Hu, L

Liu, BY

Ye, F

Wei, BC

Chen, GL

AF Hu, L.

Liu, B. Y.

Ye, F.

Wei, B. C.

Chen, G. L.

TI Ca-Mg-Zn-(Ag) bulk metallic glasses prepared by unidirectional quenching

SO INTERMETALLICS

AB We fabricated ternary Ca-Mg-Zn and quaternary Ca-Mg-Zn-Ag bulk metallic glasses with diameter of 7 mm by unidirectional quenching into water-cooled Ga-In-Sn liquid alloys. It was suggested that the electromagnetic stirring caused by induction eddy current facilitated the glass formation. Glass forming ability of Ca(62.5)Mg(17.5)Zn(20-x)Ag(x) (x = 0, 1, 3, 5, 7,9) system was dependent on Ag content. It was found that in the system the in-situ formed crystalline phases enhanced the second-stage crystallization, whereas there seemed no contribution to the third-stage crystallization. (C) 2011 Elsevier Ltd. All rights reserved.

RI Ye, Feng/G-8236-2014

OI Ye, Feng/0000-0002-8808-9075

SN 0966-9795

PD MAY

PY 2011

VL 19

IS 5

BP 662

EP 665

DI 10.1016/j.intermet.2011.01.003

UT WOS:000289126800008

ER

PT J

AU Duan, G

Wiest, A

Lind, ML

Li, J

Rhim, WK

Johnson, WL

AF Duan, Gang

Wiest, Aaron

Lind, Mary L.

Li, John

Rhim, Won-Kyu

Johnson, William L.

TI Bulk metallic glass with benchmark thermoplastic processability

SO ADVANCED MATERIALS

AB Simple microreplication experiments carried out in open air using relatively low applied pressures demonstrate superior thermoplastic processability of newly designed bulk metallic glass. It is the microformed impression of a United States dime coin (see figure) made on the surface of metallic glass wafers at similar to 370 degrees C. This bulk metallic glass exhibits benchmark characteristics of thermoplastic processing and will hopefully broaden the engineering applications of amorphous metals.

RI Duan, Gang/B-3188-2009

SN 0935-9648

EI 1521-4095

PD DEC 3

PY 2007

VL 19

IS 23

BP 4272

EP +

DI 10.1002/adma.200700969

UT WOS:000251910100028

ER

PT J

AU Yang, F

Deng, Y

Yang, JL

Zhang, W

AF Yang Fei

Deng You

Yang Jianlin

Zhang Wei

TI Plastic deformation of bulk metallic glass Zr55Al10Ni5Cu30 during

nanoindentation

SO RARE METAL MATERIALS AND ENGINEERING

AB Plastic deformation of bulk metallic glass (BMG) Zr55Al10Ni5Cu30 was investigated by nanoindentation experiments at room temperature. The characters of plastic deformation were studied by means of scanning electron microscopy (SEM) and transmission electron microscopy (TEM). The results indicate that the discrete shear bands are formed around the indent and the local free volume is increased in the bulk metallic glass by deformation. The mechanism governing the plastic deformation was discussed in terms of the free volume mode.

SN 1002-185X

PD APR

PY 2007

VL 36

IS 4

BP 578

EP 582

UT WOS:000246560600004

ER

PT J

AU Gong, P

Yao, KF

Zhao, SF

AF Gong, Pan

Yao, Kefu

Zhao, Shaofan

TI Cu-alloying effect on crystallization kinetics of Ti41Zr25Be28Fe6 bulk

metallic glass

SO JOURNAL OF THERMAL ANALYSIS AND CALORIMETRY

AB Compared with Ti41Zr25Be28Fe6 bulk metallic glass, (Ti41Zr25Be28Fe6)(93)Cu-7 glassy alloy possesses a much narrower supercooled liquid region but the glass-forming ability is dramatically improved. Isochronal and isothermal differential scanning calorimetry measurements were adopted to investigate Cu-alloying effect on the crystallization transformation kinetics of Ti41Zr25Be28Fe6 glassy alloy. It is found that Cu alloying increases the activation energies of Ti41Zr25Be28Fe6 glassy alloy for glass transition and crystallization in continuous heating. Moreover, the isothermal activation energy of (Ti41Zr25Be28Fe6)(93)Cu-7 glassy alloy increases as process of crystallization transformation, while Cu-free alloy exhibits a contrary tendency. The addition of Cu also decreases the Avrami exponent of the base alloy, resulting in the suppression of crystal nucleation and growth.

RI Gong, Pan/K-8870-2012

OI Gong, Pan/0000-0002-3833-8440

SN 1388-6150

EI 1572-8943

PD AUG

PY 2015

VL 121

IS 2

BP 697

EP 704

DI 10.1007/s10973-015-4549-5

UT WOS:000357676500020

ER

PT J

AU Lowhaphandu, P

Montgomery, SL

Lewandowski, JJ

AF Lowhaphandu, P

Montgomery, SL

Lewandowski, JJ

TI Effects of superimposed hydrostatic pressure on flow and fracture of a

Zr-Ti-Ni-Cu-Be bulk amorphous alloy

SO SCRIPTA MATERIALIA

RI Lewandowski, John/S-3815-2017

OI Lewandowski, John/0000-0002-3389-2637

SN 1359-6462

PD JUN 4

PY 1999

VL 41

IS 1

BP 19

EP 24

DI 10.1016/S1359-6462(99)00128-1

UT WOS:000081707900004

ER

PT J

AU Cao, J

Chen, HY

Song, XG

Liu, JK

Feng, JC

AF Cao, J.

Chen, H. Y.

Song, X. G.

Liu, J. K.

Feng, J. C.

TI Effects of Ar ion irradiation on the diffusion bonding joints of

Zr55Cu30Ni5Al10 bulk metallic glass to aluminum alloy

SO JOURNAL OF NON-CRYSTALLINE SOLIDS

AB 1 keV low energy argon ion beam was used to irradiate the surface of Zr55Cu30Ni5Al10 bulk metallic glass. Bulk metallic glass specimens irradiated for different times were used to join with Aluminum alloy by argon ion activated diffusion bonding. Compared with the joints using the as-cast bulk metallic glass, the tensile strength of the joints using irradiated specimens had a drastic improvement. Moreover, a crystalline layer was obtained in the BMG side of the joint interface using irradiated specimens. The improved joint quality was attributed to the local heating resulting from a crystallization behavior of irradiated surface layer which increases the probability of jumping the barrier to form the atom bonds, as well as the significant enhancement of the atomic diffusivity after crystallization. (C) 2013 Elsevier B.V. All rights reserved.

SN 0022-3093

PD MAR 15

PY 2013

VL 364

BP 53

EP 56

DI 10.1016/j.jnoncrysol.2013.01.012

UT WOS:000316438400009

ER

PT J

AU Wang, DZ

Li, N

Liu, L

AF Wang, Duzhen

Li, Ning

Liu, Lin

TI Magnetic pulse welding of a Zr-based bulk metallic glass with aluminum

plate

SO INTERMETALLICS

AB Welding of bulk metallic glasses is intriguing and puzzling since, but crucial to their engineering applications. Here, magnetic pulse welding as a new strategy was introduced to weld Zr65Al10Ni10Cu15 bulk metallic glass with aluminum plate. The alluring finding is that a thin interlayer (similar to 2.5 mu m) composed of amorphous phase and Al nano-particles was formed at the welding interface, exhibiting characteristics of vortex structure. Theoretical analysis revealed that these vortex structures within the interlayer were initially triggered by local metal melting and subsequently driven by stress wave that was caused by high velocity impact.

SN 0966-9795

EI 1879-0216

PD FEB

PY 2018

VL 93

BP 180

EP 185

DI 10.1016/j.intermet.2017.12.006

UT WOS:000424717300025

ER

PT J

AU Li, WD

Bei, H

Tong, Y

Dmowski, W

Gao, YF

AF Li, Weidong

Bei, H.

Tong, Y.

Dmowski, W.

Gao, Y. F.

TI Structural heterogeneity induced plasticity in bulk metallic glasses:

From well-relaxed fragile glass to metal-like behavior

SO APPLIED PHYSICS LETTERS

AB To reveal the structural origin responsible for the sharp change of the fracture mode on the as-cast and thermally-relaxed status, we use nanomechanical testing to measure the stresses for the onset of plasticity of a metallic glass and develop a stochastic statistical model, which can be used to characterize structural heterogeneity (defect density and strength) inside the metallic glass. Our experiments and calculations found that, with increasing the structural relaxation, the defect density drops by two orders of magnitude. Correspondingly, the fracture of metallic glasses changes from a significantly plastic (metal-like) mode to an extremely brittle (fragile glass) one. (C) 2013 AIP Publishing LLC.

RI Gao, Yanfei/F-9034-2010

OI Gao, Yanfei/0000-0003-2082-857X; Bei, Hongbin/0000-0003-0283-7990

SN 0003-6951

EI 1077-3118

PD OCT 21

PY 2013

VL 103

IS 17

AR 171910

DI 10.1063/1.4827299

UT WOS:000326455100032

ER

PT S

AU Wei, S

Yang, F

Bednarcik, J

Kaban, I

Meyer, A

Busch, R

AF Wei, Shuai

Yang, Fan

Bednarcik, Jozef

Kaban, Ivan

Meyer, Andreas

Busch, Ralf

BE Tokuyama, M

Oppenheim, I

TI Polyamorphous Transformation in Bulk Metallic Glass-forming Liquid and

its Implication to Strong Liquids

SO 4TH INTERNATIONAL SYMPOSIUM ON SLOW DYNAMICS IN COMPLEX SYSTEMS: KEEP

GOING TOHOKU

SE AIP Conference Proceedings

CT 4th International Symposium on Slow Dynamics in Complex Systems

CY DEC 02-07, 2012

CL Tohoku Univ, Inst Fluid Sci, GCOE, Sendai, JAPAN

SP World Ctr Educ & Res Trans-disciplinary Flow Dynam

HO Tohoku Univ, Inst Fluid Sci, GCOE

AB Polyamorphic phase transformations are important for understanding and applications of the properties of liquids and glasses. This article reviews the recent studies of polyamorphic transitions and their implications to the properties of metallic liquids and glasses. We discuss the implications of the viscosity hysteresis in a kinetically strong Zr-based viscous liquid and propose an alternative explanation of the anomalous crystallization behavior in the framework of the polyamorphism. We also discuss the possibility of polyamorphism in other metallic glass-forming systems and their related issues.

OI Yang, Fan/0000-0001-5281-2957

SN 0094-243X

BN 978-0-7354-1141-8

PY 2013

VL 1518

BP 260

EP 265

DI 10.1063/1.4794577

UT WOS:000317036300032

ER

PT J

AU Bakai, SA

Stoev, PI

Petrusenko, YT

Gorbatenko, VM

Moscevenko, YB

AF Bakai, S. A.

Stoev, P. I.

Petrusenko, Yu. T.

Gorbatenko, V. M.

Moscevenko, Yu. B.

TI CHANGES IN MECHANICAL PROPERTIES AND STRUCTURE OF BULK METALLIC GLASSES

BASED ON ZIRCONIUM BY HIGH FREQUENCY TREATMENT

SO PROBLEMS OF ATOMIC SCIENCE AND TECHNOLOGY

AB Influence of preliminary ultrasonic treatment on mechanical properties and structure of bulk metallic glasses Zr(52.5)Ti(5)Cu(17.9)Ni(14.6)Al(10), Zr(46.25)Cu(45.25)Al(7.5)Er(1) have been investigated. Investigations were carried out by method of acoustic emission at uniaxial compression at room temperature. The results of investigations allowed to define the nature of change of the structure and metallic glasses strength under action of ultrasonic treatment.

OI Stoev, Petr/0000-0001-7942-5850

SN 1562-6016

PY 2011

IS 2

BP 174

EP 177

UT WOS:000290222500028

ER

PT J

AU Liu, S

Huang, L

Pang, SJ

Zhang, T

AF Liu, Sen

Huang, Lu

Pang, Shu-jie

Zhang, Tao

TI Effects of crystallization on corrosion behaviours of a Ni-based bulk

metallic glass

SO INTERNATIONAL JOURNAL OF MINERALS METALLURGY AND MATERIALS

AB The effects of microstructure change on the corrosion behaviours of Ni55Nb20Ti10Zr8Co7 bulk glass-forming alloy were investigated in 1 mol/L HCl and 0.5 mol/L H2SO4 solutions. Different microstructures of the Ni-based alloy were achieved by annealing the bulk glassy rod prepared by copper mould casting. The microstructure, grain size, grain distribution, and phase composition were characterized. Electrochemical behaviours of the Ni-based alloy were revealed by static immersion and anodic potentiodynamic polarization tests. It is indicated that the corrosion behaviours of the Ni-based bulk glass-forming alloy are related to its microstructures, while the fully crystallized alloy exhibits a relatively lower corrosion resistance than those of the amorphous states.

RI Zhang, Tao/O-4911-2014; Pang, Shujie/D-8305-2016; Huang, Lu/H-5325-2012;

Huang, Lu/L-4643-2013

OI Huang, Lu/0000-0001-8318-2687; Huang, Lu/0000-0001-8318-2687

SN 1674-4799

PD FEB

PY 2012

VL 19

IS 2

BP 146

EP 150

DI 10.1007/s12613-012-0530-5

UT WOS:000299419100010

ER

PT J

AU Guo, J

Bian, XF

Zhao, Y

Zhang, SJ

Li, TB

Wang, CD

AF Guo, Jing

Bian, Xiufang

Zhao, Yan

Zhang, Shijiao

Li, Taibao

Wang, Caidong

TI Correlation between the fragility of supercooled liquids and thermal

expansion in the glassy state for Gd-based glass-forming alloys

SO JOURNAL OF PHYSICS-CONDENSED MATTER

AB Dilatometric measurements were performed to obtain the average thermal expansion coefficients of a series of Gd- based bulk metallic glasses. The fragilities of these alloys were determined based on differential scanning calorimetry measurements. It was found that there is a linear correlation between the fragility parameter of supercooled liquids and the average thermal expansion coefficient in Gd- based bulk metallic glass- forming alloys.

SN 0953-8984

PD MAR 21

PY 2007

VL 19

IS 11

AR 116103

DI 10.1088/0953-8984/11/116103

UT WOS:000245650700006

ER

PT J

AU Li, HF

Xie, XH

Zhao, K

Wang, YB

Zheng, YF

Wang, WH

Qin, L

AF Li, H. F.

Xie, X. H.

Zhao, K.

Wang, Y. B.

Zheng, Y. F.

Wang, W. H.

Qin, L.

TI In vitro and in vivo studies on biodegradable CaMgZnSrYb high-entropy

bulk metallic glass

SO ACTA BIOMATERIALIA

AB In order to enhance the corrosion resistance of the Ca65Mg15Zn20 bulk metallic glass, which has too fast a degradation rate for biomedical applications, we fabricated the Ca20Mg20Zn20Sr20Yb20 high-entropy bulk metallic glass because of the unique properties of high-entropy alloys. Our results showed that the mechanical properties and corrosion behavior were enhanced. The in vitro tests showed that the Ca20Mg20Zn20Sr20Yb20 high-entropy bulk metallic glass could stimulate the proliferation and differentiation of cultured osteoblasts. The in vivo animal tests showed that the Ca20Mg20Zn20Sr20Yb20 high-entropy bulk metallic glass did not show any obvious degradation after 4 weeks of implantation, and they can promote osteogenesis and new bone formation after 2 weeks of implantation. The improved mechanical properties and corrosion behavior can be attributed to the different chemical composition as well as the formation of a unique high-entropy atomic structure with a maximum degree of disorder. (C) 2013 Acta Materialia Inc. Published by Elsevier Ltd. All rights reserved.

RI Zheng, Yufeng/A-4146-2010; Li, Huafang/D-5486-2013; Qin,

Ling/J-9047-2018; Wang, Yanbo/E-8046-2010; BAI, JIE/D-7448-2016

OI Zheng, Yufeng/0000-0002-7402-9979; Qin, Ling/0000-0001-6173-6167; Wang,

Yanbo/0000-0002-3184-4201;

SN 1742-7061

EI 1878-7568

PD NOV

PY 2013

VL 9

IS 10

BP 8561

EP 8573

DI 10.1016/j.actbio.2013.01.029

UT WOS:000326773100010

PM 23380208

ER

PT J

AU Si, JJ

Du, CX

Wang, T

Wu, YD

Wang, RS

Hui, XD

AF Si, Jiajia

Du, Chenxi

Wang, Tan

Wu, Yidong

Wang, Rongshan

Hui, Xidong

TI Glass formation and soft magnetic properties of novel Fe-rich Fe-B-Ti-Zr

bulk metallic glasses

SO JOURNAL OF ALLOYS AND COMPOUNDS

AB Composition design is an important issue concerning the development of bulk metallic glasses (BMGs), especially applicable Fe-based bulk metallic glasses (BMGs). In this work, new quaternary Fe77B18TixZr5-x (x = 1-4) BMGs were developed by promoting metastable transformation in solidification process. Their glass formation, thermal and soft magnetic properties have been investigated. Thermal analysis proves that the combined additions of Ti and Zr in the alloys have significant advantage in improving the glass forming ability (GFA). However, the alloy with best GFA does not show the optimal thermal performance, and such inconformity in MGs was usually neglected previously. We further explain the origin of the improvement in GFA and the inconformity between GFA and thermal properties by investigating the phase evolution of the crystalline alloys. Proper combined additions of Ti and Zr can promote the precipitation of metastable Fe23B6 phase, which is advantageous to GFA as the diverse crystallization pathways reflect different crystallization resistances in solidification. Owing to the high Fe content, the amorphous ribbons exhibit high saturation induction of over 1.3 T. (c) 2018 Elsevier B.V. All rights reserved.

SN 0925-8388

EI 1873-4669

PD APR 15

PY 2018

VL 741

BP 542

EP 548

DI 10.1016/j.jallcom.2018.01.074

UT WOS:000425530700066

ER

PT J

AU Men, H

Pang, SJ

Inoue, A

Zhang, T

AF Men, H

Pang, SJ

Inoue, A

Zhang, T

TI New Ti-based bulk metallic glasses with significant plasticity

SO MATERIALS TRANSACTIONS

AB Formation of Ti-based bulk metallic glasses was investigated in (Ti,Zr)-(Cu.Ni) pseudobinary system. It was found that glass-forming ability was significantly improved by the addition of Zr and Ni to the Ti-Cu binary alloys. For Ti50Zr5Cu40Ni5. Ti45Zr5Cu45Ni5, Ti42.5Zr10Cu42.3Ni5 and Ti42.5Zr7.5Cu45Ni5 alloys, glassy, alloy rods with diameters of 2 and 3 mm can be obtained by a copper mold casting method. The glassy alloys exhibit high compressive fracture strength of about 2 GPa, and the bulk glassy Ti45Zr5Cu45Ni5 alloy shows distinct plastic strain of 0.018.

RI Inoue, Akihisa/E-5271-2015; Zhang, Tao/O-4911-2014; Pang,

Shujie/D-8305-2016

SN 1345-9678

EI 1347-5320

PD OCT

PY 2005

VL 46

IS 10

BP 2218

EP 2220

DI 10.2320/matertrans.46.2218

UT WOS:000233047200017

ER

PT J

AU Liu, L

Chan, KC

AF Liu, L

Chan, KC

TI Kinetic and structural study on amorphous-to-quasicrystalline

transformation in ZrNiCuAlAg bulk metallic glass

SO INTERMETALLICS

CT 3rd International Conference on Bulk Metallic Glasses

CY OCT 12-16, 2003

CL Beijing, PEOPLES R CHINA

AB Zr65Ni10Cu7.5Al7.5Ag10 bulk metallic glass was prepared by copper-mould casting. The kinetics and structure related to the phase transformation of the bulk metallic glass were studied by X-ray diffraction, differential scanning calorimetry (DSC) and transmission electron microscopy (TEM). It was found that the Zr65Ni10Cu7.5Al7.5Ag10 bulk metallic glass exhibits a distinct glass transition followed by two exothermic reactions, with the first reaction being the amorphous-to-quasicrystalline transformation, and the second being the quasicrystalline-to-Zr2Cu intermetallic transformation. The isothermal kinetics for amorphous-to-quasicrystalline transformation was quantitatively analyzed in the framework of JMA equation. The parameter of local Avrami exponent was introduced to explain the details of nucleation and growth behaviour in the transformation. It was revealed that the local Avrami exponent for the transformation varied with an increase in the volume fraction transformed, indicating that multi-mechanisms were involved in the transformation. TEM observations demonstrated that the variation in transformation kinetics at different stages is most likely the result of the structural inhomogeneity in the as-cast bulk metallic glass caused by a temperature gradient along the radius direction during casting. (C) 2004 Elsevier Ltd. All rights reserved.

RI Chan, K.C./A-2311-2014

OI Chan, K.C./0000-0002-6173-5532

SN 0966-9795

PD OCT-NOV

PY 2004

VL 12

IS 10-11

BP 1143

EP 1148

DI 10.1016/j.intermet.2004.04.021

UT WOS:000224566700019

ER

PT J

AU Cheng, M

Zhang, SH

Wert, JA

AF Cheng, M.

Zhang, S. H.

Wert, J. A.

TI Finite element analysis of microimprinting of bulk metallic glasses in

supercooled liquid regime

SO JOURNAL OF MATERIALS SCIENCE

AB A finite element analysis (FEA) model to analyze imprint of a bulk metallic glass (BMG) in the temperature range near the glass transition temperature (T-g) has been developed. The material model includes both Newtonian and non-Newtonian flow behavior. The results reveal that the topology of the imprinted surface depends strongly on temperatures, but only mildly on surface feature scale. As a result of the flow characteristics of BMG in the temperature range above T-g, the lubrication condition has only a slight effect on BMG imprinting.

RI Zhang, Shi-Hong/C-8772-2011

SN 0022-2461

PD AUG

PY 2007

VL 42

IS 15

BP 5999

EP 6003

DI 10.1007/s10853-006-1119-z

UT WOS:000247934600015

ER

PT J

AU Meyer, C

Ellendt, N

Srivastava, VC

Uhlenwinkel, V

AF Meyer, Christoph

Ellendt, Nils

Srivastava, Vikas C.

Uhlenwinkel, Volker

TI Cooling conditions for the generation of bulk metallic glasses by

droplet deposition

SO INTERNATIONAL JOURNAL OF MATERIALS RESEARCH

CT 14th International Conference on Rapidly Quenched and Metastable

Materials (RQ)

CY AUG 28-SEP 02, 2011

CL Salvador, BRAZIL

SP Fed Univ Sao Carlos, Dept Mat Engn

AB The cooling rate during material processing until glass transition rate is the key parameter for the production of bulk metallic glasses. But in the past, little attention has been paid to advanced production techniques such as deposition of molten metal sprays or spray forming, which offer elevated cooling rates. In this work, cooling conditions during spray forming were investigated due to its utmost importance for producing amorphous structures. Spray forming is treated in this work as a three step cooling process consisting of droplet flight phase, splat phase and deposit phase. All cooling steps were simulated for different droplet sizes. The surface temperature of the deposit was found to play an important role in the production of metallic glasses via spray forming. The simulation model can be used to find suitable spray conditions for the generation of bulk metallic glasses.

RI Srivastava, vikas/C-9668-2010; Ellendt, Nils/I-6892-2013; Sahu,

Anjani/E-7590-2015

OI Ellendt, Nils/0000-0001-7242-8063;

SN 1862-5282

PD SEP

PY 2012

VL 103

IS 9

BP 1090

EP 1095

DI 10.3139/146.110802

UT WOS:000309443700003

ER

PT J

AU Jiang, MQ

Wu, XQ

Wei, YP

Wilde, G

Dai, LH

AF Jiang, M. Q.

Wu, X. Q.

Wei, Y. P.

Wilde, G.

Dai, L. H.

TI Cavitation bubble dynamics during pulsed laser ablation of a metallic

glass in water

SO EXTREME MECHANICS LETTERS

AB We report a cavitation bubble formation in water induced by nanosecond pulsed laser ablation of a Zrbased (Vitreloy 1) bulk metallic glass target. Only the first bubble occurs due to an explosive-boiling-type ablation of the target. A theoretical model is developed to quantitatively describe the bubble nucleation and its initial growth. The results demonstrate that the laser-induced plasma can induce the nucleation of the bubble. Furthermore, it is revealed that the initial bubble growth is approximately adiabatic and inertial, obeying the Rayleigh-Plesset theory, albeit the significant ablation. This work sheds insight into the mechanics of water-confined laser ablation of metallic glasses, and provides guidance for synthesizing amorphous nanoparticles. (C) 2016 Elsevier Ltd. All rights reserved.

OI Dai, LanHong/0000-0001-8991-0358; Wilde, Gerhard/0000-0001-8001-5998

SN 2352-4316

PD FEB

PY 2017

VL 11

BP 24

EP 29

DI 10.1016/j.eml.2016.11.014

UT WOS:000401153700004

ER

PT J

AU Raghavan, R

Boopathy, K

Ghisleni, R

Pouchon, MA

Ramamurty, U

Michler, J

AF Raghavan, R.

Boopathy, K.

Ghisleni, R.

Pouchon, M. A.

Ramamurty, U.

Michler, J.

TI Ion irradiation enhances the mechanical performance of metallic glasses

SO SCRIPTA MATERIALIA

AB We demonstrate that irradiation may enhance the plasticity in metallic glasses by increasing the free-volume content via micropillar compression experiments on an ion-irradiated bulk metallic glass (BMG). Results show that irradiation decreases the flow stress and enhances the shear band formation by lowering the magnitude of stress serrations in plastic flow regime. These results highlight that amorphous alloys can mitigate the deleterious affects of severe ion irradiation as compared to their crystalline counterparts. (C) 2009 Acta Materialia Inc. Published by Elsevier Ltd. All rights reserved.

RI Raghavan, Rejin/A-2177-2015; Pouchon, Manuel Alexandre/J-7213-2015;

Michler, Johann/B-4672-2010; Kombaiah, Boopathy/E-5568-2011; Ghisleni,

Rudy/E-7884-2010; Ramamurty, Upadrasta/E-5623-2011

OI Raghavan, Rejin/0000-0002-7108-7081; Michler,

Johann/0000-0001-8860-4068;

SN 1359-6462

PD APR

PY 2010

VL 62

IS 7

BP 462

EP 465

DI 10.1016/j.scriptamat.2009.12.013

UT WOS:000274888600008

ER

PT J

AU Huang, XM

Wang, XD

He, Y

Cao, QP

Jiang, JZ

AF Huang, X. M.

Wang, X. D.

He, Y.

Cao, Q. P.

Jiang, J. Z.

TI Are there two glass transitions in Fe-M-Y-B (M = Mo, W, Nb)bulk metallic

glasses?

SO SCRIPTA MATERIALIA

AB An anomalous glass transition was detected in differential scanning calorimetry (DSC) curves for Fe-M-Y-B (M = Mo, W, Nb) bulk metallic glasses, which has been investigated by DSC, X-ray diffraction and extended X-ray absorption fine structure. It is confirmed that the second inflection event in the supercooled liquid region is not a real glass transition, but rather a local atomic structure reordering, which is mainly caused by the strong affinity between B and other constituent elements as well as the repulsive interaction between M and Y. (C) 2008 Acta Materialia Inc. Published by Elsevier Ltd. All rights reserved.

RI Cao, Qing Ping/A-1055-2010

SN 1359-6462

PD FEB

PY 2009

VL 60

IS 3

BP 152

EP 155

DI 10.1016/j.scriptamat.2008.09.022

UT WOS:000263280900007

ER

PT J

AU Ma, D

Stoica, AD

Yang, L

Wang, XL

Lu, ZP

Neuefeind, J

Kramer, MJ

Richardson, JW

Proffen, T

AF Ma, D.

Stoica, A. D.

Yang, L.

Wang, X.-L.

Lu, Z. P.

Neuefeind, J.

Kramer, M. J.

Richardson, J. W.

Proffen, Th.

TI Nearest-neighbor coordination and chemical ordering in multicomponent

bulk metallic glasses

SO APPLIED PHYSICS LETTERS

AB The authors report complementary use of high-energy x-ray and neutron diffraction to probe the local atomic structure in a Zr-based bulk metallic glass. By analyzing the partial coordination numbers, the authors demonstrate the presence of multiple types of solute-centered clusters in the multicomponent glass and efficient packing of the amorphous structure at atomic scale. The authors' findings provide a basis for understanding how local structures change during phase transformation and mechanical deformation of multicomponent amorphous alloys. (c) 2007 American Institute of Physics.

RI Wang, Xun-Li/C-9636-2010; Ma, Dong/G-5198-2011; Lujan Center,

LANL/G-4896-2012; Stoica, Alexandru/K-3614-2013; Lu,

Zhao-Ping/A-2718-2009; Proffen, Thomas/B-3585-2009; Neuefeind,

Joerg/D-9990-2015

OI Wang, Xun-Li/0000-0003-4060-8777; Ma, Dong/0000-0003-3154-2454; Stoica,

Alexandru/0000-0001-5118-0134; Lu, Zhao-Ping/0000-0003-1463-8948;

Proffen, Thomas/0000-0002-1408-6031; Neuefeind,

Joerg/0000-0002-0563-1544

SN 0003-6951

PD MAY 21

PY 2007

VL 90

IS 21

AR 211908

DI 10.1063/1.2742315

UT WOS:000246775900025

ER

PT J

AU Jiang, Q

Chi, BQ

Li, JC

AF Jiang, Q

Chi, BQ

Li, JC

TI A valence electron concentration criterion for glass-formation ability

of metallic liquids

SO APPLIED PHYSICS LETTERS

AB Critical cooling rate of a metallic liquid (R-c) necessary to form glass is correlated to the valence concentration e/a of the liquid (e and a denote valence and atom number in a unit cell, respectively). R-c of liquid is minimized at e/a=3.5. The principles of the composition design to form bulk metallic glasses are as follows: e/a=3.5, the component number (n) in the alloy is larger than three, and the component percentage of the ith component (x(i)) approaches 1/n. (C) 2003 American Institute of Physics.

RI Jiang, Qing/F-4073-2012

OI Jiang, Qing/0000-0003-0660-596X

SN 0003-6951

PD MAY 5

PY 2003

VL 82

IS 18

BP 2984

EP 2986

DI 10.1063/1.1571984

UT WOS:000182570000016

ER

PT J

AU Flores, KM

Dauskardt, RH

AF Flores, KM

Dauskardt, RH

TI Local heating associated with crack tip plasticity in Zr-Ti-Ni-Cu-Be

bulk amorphous metals

SO JOURNAL OF MATERIALS RESEARCH

AB Deformation in metallic glasses is generally considered to arise from flow in localized shear bands, where adiabatic heating is thought to reduce glass viscosity, Evidence has been inferred from the veined fracture surfaces and molten droplets reported for metallic glasses. In this work, the detailed spatially resolved surface temperature increase and subsequent dissipation associated with crack tip plasticity in a Zr-Ti-Ni-Cu-Be bulk metallic glass is characterized for the first time. Maximum temperatures of up to 54.2 K were estimated from a heat conduction model and shown to be in excellent agreement with a nonhardening plasticity model for the heat generated by a propagating crack. Local cooling was also observed and shown to be consistent with thermoelastic effects.

SN 0884-2914

EI 2044-5326

PD MAR

PY 1999

VL 14

IS 3

BP 638

EP 643

DI 10.1557/JMR.1999.0642

UT WOS:000082550300003

ER

PT J

AU Liu, TK

Gao, YF

Bei, HB

AF Liu, Tingkun

Gao, Yanfei

Bei, Hongbin

TI Probing elastically or plastically induced structural heterogeneities in

bulk metallic glasses by nanoindentation pop-in tests

SO AIP ADVANCES

AB Shear banding dynamics in bulk metallic glasses (BMGs) is manifested by the spatiotemporal evolution of strain fields which in turn depend on structural heterogeneities. The spacing of these heterogeneities, as a characteristic length scale, was determined from the analysis of nanoindentation pop-in tests using a stochastic model. Furthermore, the pre-stress by elastic bending and residual stress by plastic bending of BMGplates were found to dramatically decrease such spacings, thus increasing heterogeneity density and mechanically rejuvenating the glass structure. (C) 2017 Author(s).

RI Gao, Yanfei/F-9034-2010

OI Gao, Yanfei/0000-0003-2082-857X; Bei, Hongbin/0000-0003-0283-7990

SN 2158-3226

PD AUG

PY 2017

VL 7

IS 8

AR 085216

DI 10.1063/1.4993719

UT WOS:000409090200059

ER

PT J

AU Su, C

Anand, L

AF Su, C

Anand, L

TI Plane strain indentation of a Zr-based metallic glass: Experiments and

numerical simulation

SO ACTA MATERIALIA

AB The response of a Zr-based metallic glass in instrumented plane strain indentation with a cylindrical indenter tip is studied experimentally. A recently developed constitutive model and simulation capability for metallic glasses is used to numerically calculate indentation load versus depth curves, and the evolution of corresponding shear-band patterns under the indenter. The numerical simulations are shown to compare very favorably with the corresponding experimental results. (c) 2005 Acta Materialia Tnc. Published by Elsevier Ltd. All rights reserved.

RI Anand, Lallit/B-6332-2009

OI Anand, Lallit/0000-0002-4581-7888

SN 1359-6454

PD JAN

PY 2006

VL 54

IS 1

BP 179

EP 189

DI 10.1016/j.actamat.2005.08.040

UT WOS:000233784500020

ER

PT J

AU Zhang, QS

Zhang, HF

Deng, YF

Ding, BZ

Hu, ZQ

AF Zhang, QS

Zhang, HF

Deng, YF

Ding, BZ

Hu, ZQ

TI Bulk metallic glass formation of Cu-Zr-Ti-Sn alloys

SO SCRIPTA MATERIALIA

AB Cu-Zr-Ti-Sn bulk metallic glasses were produced by copper mold casting. The effects of Sn addition on glass-forming ability (GFA), thermal stability of the Cu60Zr30Ti10 bulk metallic glass were investigated. It was found that a bulk metallic glass of 5 mm in diameter was prepared in a (Cu60Zr30Ti10)(99)Sn-1 alloy by copper mold casting. The addition of 1 at.% Sn is effective for an increase in GFA. The DeltaT(x) and T-g/T-I are 46 K and 0.63, respectively, for (Cu60Zr30Ti10)(99)Sn-1 alloy. With increasing the content of Sn, the value of TITI increases, but the alloys begin to lose bulk metallic GFA. The new parameter gamma has a better correlation with the GFA of the Cu-based alloys. (C) 2003 Published by Elsevier Science Ltd. on behalf of Acta Materialia Inc.

RI zhang, qingsheng/A-4851-2010

SN 1359-6462

PD AUG

PY 2003

VL 49

IS 4

BP 273

EP 278

DI 10.1016/S1359-6462(03)00285-9

UT WOS:000183663000002

ER

PT J

AU Keryvin, V

Nadot, Y

Yokoyama, Y

AF Keryvin, V.

Nadot, Y.

Yokoyama, Y.

TI Fatigue pre-cracking and toughness of the Zr55Cu30Al10Ni5 bulk metallic

glass for two oxygen levels

SO SCRIPTA MATERIALIA

AB Samples of a Zr-based bulk metallic glass with a small (1000 appm) or very small (less than 300 appm) oxygen content are pre-cracked by fatigue and tested for toughness evaluation. It is shown that oxygen trapped in oxide dendrites eases the initiation of a straight crack and embrittles the glass even at such low concentrations. In contrast, when oxygen is dissolved in the glass, fatigue crack initiation becomes difficult and it is not possible to get a crack passing straight through the glass. (C) 2007 Acta Materialia Inc. Published by Elsevier Ltd. All rights reserved.

RI keryvin, vincent/F-1279-2010; Yokoyama, Yoshihiko/A-8603-2011

SN 1359-6462

PD JUL

PY 2007

VL 57

IS 2

BP 145

EP 148

DI 10.1016/j.scriptamat.2007.03.042

UT WOS:000247059800019

ER

PT J

AU Koster, U

Jastrow, L

Meuris, M

AF Koester, Uwe

Jastrow, Lioba

Meuris, Monika

TI Oxidation of Cu60Zr30Ti10 metallic glasses

SO MATERIALS SCIENCE AND ENGINEERING A-STRUCTURAL MATERIALS PROPERTIES

MICROSTRUCTURE AND PROCESSING

CT 12th International Conference on Rapidly Quenched and Metastable

Materials

CY AUG 21-26, 2005

CL Jeju Isl, SOUTH KOREA

AB Cu-Zr-based bulk metallic glasses are of increasing interest due to their excellent properties. Cu-rich Cu60Zr30Ti10 and Zr-rich Zr69.5Cu12Ni11Al7.5 glasses (numbers indicate at.%) are examples which combine good glass forming ability with excellent mechanical properties making them a material of choice for a variety of applications. Any application, however, requires adequate thermal stability, i.e. resistance against crystallization and oxidation. The aim of this paper is a detailed investigation on the oxidation of Cu60Zr30Ti10 metallic glasses. Whereas oxidation in Zr-rich Zr69.5Cu12Ni11Al7.5 is assumed to be controlled by oxygen diffusion through a homogeneous scale towards the ZrO2/glass interface, in Cu-rich Cu60Zr30Ti10 metallic glasses multilayered scales were found to develop with an assembly of Cu-oxide needles at the outer surface. Due to developing stresses and the formation of voids the outer oxide layer loses contact at the interface and starts to peel off during ongoing oxidation. (c) 2006 Elsevier B.V. All rights reserved.

SN 0921-5093

PD MAR 25

PY 2007

VL 449

BP 165

EP 168

DI 10.1016/j.msea.2006.02.291

UT WOS:000245477800034

ER

PT B

AU Tosa, DI

Cazacu, A

Serban, VA

AF Tosa, Dacian Ioan

Cazacu, Andrei

Serban, Viorel Aurel

GP TANGER

TI DUAL AMORPHOUS PHASED BULK METALLIC GLASS WITH SOFT MAGNETIC PROPERTIES

SO METAL 2014: 23RD INTERNATIONAL CONFERENCE ON METALLURGY AND MATERIALS

CT 23rd International Conference on Metallurgy and Materials

CY MAY 21-23, 2014

CL Brno, CZECH REPUBLIC

SP TANGER Ltd, VSB Tech Univ Ostrava, Czech Soc New Mat & Technologies, ASM Int, Engn Acad Czech Republic, Mat Res Soc Serbia, Norwegian Co Mat & Technol, French Soc Metallurgy & Mat, Italian Assoc Metallurgy, Austrian Soc Metallurgy & Mat, Portuguese Soc Mat, Mat Informat Soc

AB In recent years, the dual amorphous phased bulk metallic glass (DAPBMGs) was studied with interest by researchers of materials science, which is a new class of bulk metallic glasses (BMGs). These materials consist of several amorphous phases and are expecting to bring together all the favorable properties for each phase.

The DAPBMGs with soft magnetic properties can be obtained by powder metallurgy process; the amorphous powders can be produced either by mechanical alloying (MA) or by high pressure Ar gas atomization. Compacting of the glassy alloy powder to obtain the dual amorphous phased bulk metallic glass can be achieved by using a hot-pressed technique (HP) or by spark plasma sintering (SPS) process, in the supercooled liquid region of the amorphous phases.

There are presented some families of dual amorphous phased bulk metallic glass (DAPBMGs) with soft magnetic properties.

This paper synthesizes the current work of the researchers that is the processing, characterization and understanding of these new classes of materials, the dual amorphous phased bulk metallic glass with soft magnetic properties.

RI Serban, Viorel - Aurel/G-4719-2016

BN 978-80-87294-54-3

PY 2014

BP 436

EP 441

UT WOS:000350641700068

ER

PT J

AU Lu, SD

Sun, SC

Huang, XX

Zhu, XP

Tu, GF

Li, KH

AF Lu, Shuaidan

Sun, Shuchen

Huang, Xiaoxiao

Zhu, Xiaoping

Tu, Ganfeng

Li, Kuanhe

TI Glass-forming ability and mechanical properties of a

Zr52.8Cu29.1Ni7.3Al9.8Y1 bulk metallic glass prepared by hereditary

process

SO GREEN PROCESSING AND SYNTHESIS

AB Zr-based bulk metallic glass possesses the highest potential as a structural material among metallic glasses. However, the production conditions have a great effect on its glass-forming ability (GFA) and mechanical characteristics. In this paper, an attempt was made to find the effect of a hereditary structure on the GFA and mechanical properties of a solid Zr52.8Cu29.1Ni7.3Al9.8Y1 bulk metallic glass in order to evaluate a novel process of using binary alloys as precursors, which have a hereditary relation to the aim metallic glass (MGs). When the quenching temperature is below the threshold overheating temperature, the hereditary process can improve the GFA and compressive strength obviously. At a quenching temperature of 1523 K, the hereditary process can improve the supercooled liquid region Delta T-x from 33 K to 55 K and the compressive strength from 1555 MPa to 1652 MPa.

SN 2191-9542

EI 2191-9550

PD JAN

PY 2016

VL 5

IS 1

SI SI

BP 65

EP 70

DI 10.1515/gps-2015-0039

UT WOS:000374765600011

ER

PT J

AU Kim, HS

Kato, H

Inoue, A

Chen, HS

Hong, SI

AF Kim, HS

Kato, H

Inoue, A

Chen, HS

Hong, SI

TI Microforming of bulk metallic glasses: Constitutive modelling and

applications

SO MATERIALS TRANSACTIONS

CT International Symposium on Bulk Glassy Alloys

CY OCT, 2003

CL Yokohama, JAPAN

AB Microforming can be a good application for bulk metallic glasses. It is important to simulate the deformation behaviour of the bulk metallic glasses in a supercooled liquid region for manufacturing micromachine parts. For these purposes, a correct constitutive model which can reproduce viscosity results is essential for good predicting capability. In this paper, we studied deformation behaviour of the bulk metallic glasses using the finite element method in conjunction with the fictive stress constitutive model which can describe non-Newtonian as well as Newtonian behaviour. A combination of kinetic equation which describes the mechanical response of the bulk metallic glasses at a given temperature and evolution equations for internal variables provide the constitutive equation of the fictive stress model. The internal variables arc associated with fictive stress and relation time. The model has a modular structure and can be adjusted to describe a particular type of microforming process. Implementation of the model into the MARC software has shown its versatility and good predictive capability.

RI Kim, Hyoung Seop/C-2166-2009; Kato, Hidemi/B-2492-2015; Inoue,

Akihisa/E-5271-2015

OI Kim, Hyoung Seop/0000-0002-3155-583X;

SN 1345-9678

EI 1347-5320

PD APR

PY 2004

VL 45

IS 4

BP 1228

EP 1232

DI 10.2320/matertrans.45.1228

UT WOS:000221314900045

ER

PT J

AU Man, QK

Sun, HJ

Dong, YQ

Shen, BL

Kimura, H

Makino, A

Inoue, A

AF Man, Qikui

Sun, Huaijun

Dong, Yaqiang

Shen, Baolong

Kimura, Hisamichi

Makino, Akihoro

Inoue, Akihisa

TI Enhancement of glass-forming ability of CoFeBSiNb bulk glassy alloys

with excellent soft-magnetic properties and superhigh strength

SO INTERMETALLICS

CT 7th International Conference on Bulk-Metallic Glasses

CY NOV 01-05, 2009

CL Busan, SOUTH KOREA

SP Yonsei Univ, Ctr Noncrystalline Mat

AB Co-based bulk glassy alloys with diameters up to 4.5 mm were formed in Co46Fe20B22+xSi6-xNb6 (x = 0-2) system. The increase of B to Si concentration ratio is effective in improving the glass-forming ability. In addition to high glass-forming ability, the glassy alloys exhibit excellent soft-magnetic properties, i.e., high saturation magnetization of 0.63-0.69 T, low coercive force of 1.17-2.35 A/m, and high effective permeability of 1.36-2.65 x 10(4) at 1 kHz under a field of 1 A/m. The bulk glassy alloys also exhibit superhigh fracture strength of 4400 MPa. (C) 2010 Elsevier Ltd. All rights reserved.

RI Inoue, Akihisa/E-5271-2015; MAKINO, AKIHIRO/B-2549-2009; Kimura,

Hisamichi/D-5449-2012; Dong, Yaqiang/C-5370-2017

OI Dong, Yaqiang/0000-0003-1663-8052

SN 0966-9795

EI 1879-0216

PD OCT

PY 2010

VL 18

IS 10

SI SI

BP 1876

EP 1879

DI 10.1016/j.intermet.2010.02.047

UT WOS:000281420700021

ER

PT J

AU Baser, TA

Baricco, M

AF Baser, Tanya Aycan

Baricco, Marcello

TI Glass forming ability of (Cu(50) Zr(50))(96)M(4) (M=none, Al,Nb) bulk

metallic glasses

SO REVIEWS ON ADVANCED MATERIALS SCIENCE

CT 13th International Symposium on Metastable and Nano-Materials

(ISMANAM-2006)

CY AUG 27-31, 2006

CL Warsaw Univ Technol, Fac Mat Sci & Engn, Warsaw, POLAND

HO Warsaw Univ Technol, Fac Mat Sci & Engn

AB The effect of minor addition of Al and Nb on bulk glass formability in Cu(50) Zr(50) alloy is investigated. Quenching rate effect was followed comparing the results obtained on ribbons and on different sections of cone-shaped ingots. The phase mixture has been clarified for each alloy, combining structural, microstructural and composition analyses. A fully amorphous phase has been found in ribbons, whereas only for (Cu(50) Zr(50)) (96)Al(4) a bulk metallic glasses has been obtained. Minor Al addition hinders the formation of the CuZr martensite phase. The effect of composition on the glass forming abilty has been evaluated with emprical parameters, but only the calculation of driving forces for nucleation of crystal phases, using the CALPHAD method, may explain the results.

RI Baricco, Marcello/B-4075-2013

OI Baricco, Marcello/0000-0002-2856-9894

SN 1606-5131

PD MAY

PY 2008

VL 18

IS 1

BP 71

EP 76

UT WOS:000256370800017

ER

PT J

AU Yang, Q

Pang, SJ

Li, R

Zhang, T

AF Yang, Qing

Pang, Shujie

Li, Ran

Zhang, Tao

TI EFFECT OF COEXISTENCE OF SIMILAR ELEMENTS La AND Ce ON FORMATION OF

(La-Ce)-Al-Cu BULK METALLIC GLASSES

SO INTERNATIONAL JOURNAL OF MODERN PHYSICS B

CT 5th International Conference on Advanced Materials and Processing

CY SEP 02-05, 2008

CL Harbin Inst Technol, Harbin, PEOPLES R CHINA

SP Natl Key Lab Preis Hot Proc Metals, State Key Lab Adv Welding Product Technol, Natl Nat Sci Fdn China, Minerals, Met & Mat Soc

HO Harbin Inst Technol

AB Bulk metallic glasses (BMGs) in pseudo-ternary (La-Ce)-Al-Cu system with high glass-forming ability (GFA) were synthesized based on the beneficial effect of the coexistence of similar elements La and Ce with similar atomic size and various valence electronic structures on GFA. With the coexistence of La and Ce in (LaxCe1-x)(65)Al10Cu25 system, bulk metallic glasses with diameters up to 12 mm can be produced by copper mold casting. Besides the high GFA, the (LaxCe1-x)(65)Al10Cu25 BMGs with x = 0.6 and 0.7 exhibit low glass transition temperature T-g around 362 K and wide upercooled liquid regions Delta T-x(Delta T-x = T-x-T-g, where T-x is the onset temperature of crystallization) of about 80 K. Compared with ternary La-Al-Cu and Ce-Al-Cu systems, significant improvement of GFA for the (La-Ce)-Al-Cu system is caused by the coexistence of similar elements La and Ce, and the mechanism is discussed from a thermodynamic viewpoint.

RI Pang, Shujie/D-8305-2016; Zhang, Tao/O-4911-2014; Li, Ran/B-4618-2010

SN 0217-9792

EI 1793-6578

PD MAR 20

PY 2009

VL 23

IS 6-7

BP 1235

EP 1240

DI 10.1142/S0217979209060749

UT WOS:000266134700076

ER

PT J

AU Jiao, W

Xi, XK

Zhao, DQ

Pan, MX

Wang, WH

AF Jiao, W.

Xi, X. K.

Zhao, D. Q.

Pan, M. X.

Wang, W. H.

TI Fabrication of bulk metallic glasses at the region of multiple

quasi-peritectic reactions

SO INTERMETALLICS

AB We report that Ca-Cu-Mg bulk metallic glasses (BMGs) can be fabricated at the region of multiple quasiperitectic reactions by a conventional copper mold casting method, demonstrating that finding a deep eutectic composition is not the sole solution for the fabrication of BMGs. Unusual relationship between the glass transition temperature and the elastic constants of these BMGs were discussed in comparison with other BMGs. These results have implications for exploring new BMGs and understanding the glass formation mechanism. (C) 2010 Elsevier Ltd. All rights reserved.

RI wei, jiao/I-7244-2013

SN 0966-9795

PD APR

PY 2011

VL 19

IS 4

BP 586

EP 588

DI 10.1016/j.intermet.2010.12.010

UT WOS:000287341400021

ER

PT J

AU Cheney, J

Vecchio, K

AF Cheney, Justin

Vecchio, Kenneth

TI Prediction of glass-forming compositions using liquidus temperature

calculations

SO MATERIALS SCIENCE AND ENGINEERING A-STRUCTURAL MATERIALS PROPERTIES

MICROSTRUCTURE AND PROCESSING

AB A predictive model for metallic glass compositional design was constructed based upon the calculated liquidus temperatures. Furthermore, a parameter, termed a, was defined in order to quantify the relative depth of a eutectic in an alloy system. Large alpha parameters signify higher glass-forming ability. Comparisons of this theory and experimental results given in literature from varied sources and alloy types, show that compositions containing relatively high alpha values correspond to successfully produced bulk metallic glasses. Specifically, an alpha parameter value in excess of I suggests a slight tendency for glass formation, while a values greater than 1.5 suggest a very strong tendency. Thus, the a parameter can serve as a tool for quantifying potential bulk metallic glass alloys in terms of limiting their requirement for rapid quenching. This technique is appropriate for both constituent element selection and composition optimization. (C) 2007 Elsevier B.V. All rights reserved.

RI Vecchio, Kenneth/F-6300-2011

OI Vecchio, Kenneth/0000-0003-0217-6803

SN 0921-5093

PD DEC 15

PY 2007

VL 471

IS 1-2

BP 135

EP 143

DI 10.1016/j.msea.2007.02.120

UT WOS:000250168000021

ER

PT B

AU Liu, YJ

Qin, MY

Quan, YM

Liu, WY

AF Liu Yajun

Qin Mengyang

Quan YanMing

Liu Wangyu

BE Chen, D

Narutaki, N

Fan, R

Ochi, A

TI Depth of cut effect on Chip Formation during Machining

Zr41.2Ti13.8Cu12.5Ni10.0Be22.5 Bulk Metallic Glass

SO PROGRESS OF MACHINING TECHNOLOGY

CT 10th International Conference on Progress of Machining Technology

(ICPMT2012)

CY SEP 25-27, 2012

CL Tsubame, JAPAN

SP Japan Soc Precis Engn, Beihang Univ, Tsubane City, Chinese Soc Adv Mfg Technol, Japan Soc Precis Engn, Tech Comm Cutt, Japan Soc Precis Engn, Tech Comm Machin Difficult Cut Mat, Chines Mech Engn Soc, Biejing Municipal Commiss Educ, Japan Soc Abras Technol, Japan Soc Mech Engn

AB Chip formation of Zr41.2Ti13.8Cu12.5Ni10.0Be22.5 bulk metallic glass(BMG) during machining with different depth of cut was investigated using SEM, X-ray diffraction. It is interesting that very good plasticity occurs during machining Zr-based BMG compare to a complete brittleness in tension. The chip morphology was unique and showed the presence of plastic shear bands.

BN 978-4-9903247-8-0

PY 2012

BP 183

EP 186

UT WOS:000327044000045

ER

PT J

AU Na, JH

Kim, WT

Kim, DH

Yi, S

AF Na, JH

Kim, WT

Kim, DH

Yi, S

TI Bulk metallic glass formation in Ni-Zr-Nb-Al alloy systems

SO MATERIALS LETTERS

AB Ni-based metallic glasses containing no metalloids are developed in Ni-Zr-Nb-Al alloy system. A partial replacement of Zr with Nb in Ni-Zr-Al alloys significantly enhances the glass-forming ability (GFA) and enlarges the undercooled liquid region during continuous heating of glassy ribbons. A quaternary Ni-Zr-Nb-Al metallic glasses exhibit large undercooled liquid region (>50 K), indicating high glass-forming ability of the alloy. The glass transition and crystallization temperatures of a quaternary Ni61Zr28Nb7Al4 alloy are 848 and 898 K, respectively, exhibiting a wide undercooled liquid region of 50 K. A complete Ni61Zr28Nb7Al4 glassy rod (phi1 mm) can be prepared by copper mold injection casting. (C) 2003 Elsevier B.V. All rights reserved.

RI bang, changwook/J-7922-2012

SN 0167-577X

PD FEB

PY 2004

VL 58

IS 5

BP 778

EP 782

DI 10.1016/j.matlet.2003.07.026

UT WOS:000188216300045

ER

PT S

AU Aniya, M

Ikeda, M

AF Aniya, M.

Ikeda, M.

BE Takemura, K

TI Pressure dependence of the fragility in metallic glass forming liquids:

Predictions from a theoretical model and materials properties

correlations

SO INTERNATIONAL CONFERENCE ON HIGH PRESSURE SCIENCE AND TECHNOLOGY, JOINT

AIRAPT-22 AND HPCJ-50

SE Journal of Physics Conference Series

CT Joint AIRAPT-22 and HPCJ-50 Conference/International Conference on High

Pressure Science and Technology

CY JUL 26-31, 2009

CL Tokyo, JAPAN

AB By studying the pressure dependence of the fragility, it is expected that new horizons will open to understand the relaxation behaviour of supercooled liquids. However, as far as the authors are informed, no study has been performed on pressure dependence of the fragility in bulk metallic glass forming systems. In the present study, such dependence is investigated based on materials properties correlations. It is suggested that the effect of pressure on the fragility of bulk metallic glasses is very small. Our analysis indicates also that the fragility of metallic glasses will increase with the application of pressure. This behaviour contrasts with the behaviour observed in molecular systems, where the fragility remains almost constant or decreases slightly with the application of pressure. The result found is discussed in terms of the bond strength-coordination number fluctuation model of the viscosity.

SN 1742-6588

PY 2010

VL 215

AR 012083

DI 10.1088/1742-6596/215/1/012083

UT WOS:000292385100083

ER

PT S

AU Tabachnikova, ED

Bengus, VZ

Molokanov, VV

AF Tabachnikova, ED

Bengus, VZ

Molokanov, VV

BE Schulz, R

TI Low temperature plasticity and failure of a bulk Cu50Zr35Ti8Hf5Ni2

metallic glass

SO METASTABLE, MECHANICALLY ALLOYED AND NANOCRYSTALLINE MATERIALS, PTS 1

AND 2

SE MATERIALS SCIENCE FORUM

CT International Symposium on Metastable, Mechanically Alloyed and

Nanocrystalline Materials (ISMANAM-95)

CY JUL 24-28, 1995

CL QUEBEC CITY, CANADA

SP Hydro Quebec, Laval Univ, McGill Univ, Ctr Phys Mat, Nat Sci & Engn Res Council Canada, Zoz GmbH, Siemens, Xerox Canada Ltee, Precitech

AB A high local plasticity and a formation of the ''vein pattern'' after the low temperature shear failure are observed under a uniaxial compression of a bulk Cu50Zr35Ti8 Hf5Ni2 metallic glass. These observations give a possibility to exclude the explanation of an existence of the low viscosity layer and a high local plasticity in the catastrophic shear band by a high hydrostatic tension without increasing of a local temperature. Local adiabatic heating is the only cause of a high local plasticity of metallic glasses during the low temperature shear failure.

OI Molokanov, Vyacheslav V./0000-0003-4664-463X

SN 0255-5476

BN 0-87849-738-2

PY 1996

VL 225

BP 107

EP 111

DI 10.4028/www.scientific.net/MSF.225-227.107

UT WOS:A1996BG37Y00015

ER

PT J

AU Trexler, MM

Thadhani, NN

AF Trexler, Morgana Martin

Thadhani, Naresh N.

TI Mechanical properties of bulk metallic glasses

SO PROGRESS IN MATERIALS SCIENCE

AB The mechanical properties of bulk metallic glasses, including their superior strength and hardness, and excellent corrosion and wear resistance, combined with their general inability to undergo homogeneous plastic deformation have been a subject of fascination for scientists and engineers. The scientific Interest stems from the unconventional deformation and failure initiation mechanisms in this class of materials in which the typical carriers of plastic flow (dislocations) are absent Metallic glasses undergo highly localized, heterogeneous deformation by formation of shear bands, a particular mode of deformation of interest for certain applications, but which also causes them to fail catastrophically due to uninhibited shear band propagation. Varying degrees of brittle and plastic failure creating intricate fracture patterns are observed in metallic glasses, quite different from those observed in crystalline solids The tension-compression anisotropy, strain-rate sensitivity, thermal stability, stress-induced crystallization and polyamorphism transformations, are some of the attributes that have sparked engineering studies on bulk metallic glasses. Understanding of the glass-forming ability and the deformation and failure mechanisms of bulk metallic glasses, has given insight into alloy compositions and intrinsically-forming or extrinsically-added reinforcement phases for creating composite structures, to attain the combination of high strength, tensile ductility, and fracture toughness needed for use in advanced structural applications. The relative ease of fabricating metallic glasses into bulk forms, combined with their unique mechanical properties, has made these materials attractive options for possible applications in aerospace, naval, sports equipment, luxury goods, armor and anti-armor systems, electronic packaging, and biomedical devices (C) 2010 Elsevier Ltd. All rights reserved.

RI Trexler, Morgana/E-9003-2013

SN 0079-6425

PD NOV

PY 2010

VL 55

IS 8

BP 759

EP 839

DI 10.1016/j.pmatsci.2010.04.002

UT WOS:000280947200001

ER

PT J

AU Xie, KF

Yao, KF

Huang, TY

AF Xie, Ke-Fei

Yao, Ke-Fu

Huang, Tian-You

TI A Ti-based bulk glassy alloy with high strength and good glass forming

ability

SO INTERMETALLICS

CT 7th International Conference on Bulk-Metallic Glasses

CY NOV 01-05, 2009

CL Busan, SOUTH KOREA

SP Yonsei Univ, Ctr Noncrystalline Mat

AB Ti-based bulk metallic glasses have attracted lots of attention of scientific researchers due to their lightweight, low cost and high specific strength. But only a few Ti-based alloys have been reported possessing large glass forming ability. Then, research on new Ti-based alloys with good glass forming ability is necessary and important. In present work, rods of Ti(43.15)Zr(9.59)Cu(36.24)Ni(9.06)Sn(1.96) glassy alloy with diameter of 3 mm have been fabricated by using copper mould casting method. It has been found that this Ti-based bulk metallic glass possesses good mechanical properties at room temperature. The compressive yield strength and the fracture strength of the glassy alloy are 2360 MPa and 2640 MPa, respectively, together with the plastic strain of 2.24%. The present results show that adding an element or increasing the amount of an element with large difference in atomic size ratio with other main constitutive elements and more negative mixing heat with other main constitutive elements in an alloy would benefit to enhance the glass forming ability of the alloy. (C) 2010 Elsevier Ltd. All rights reserved.

SN 0966-9795

PD OCT

PY 2010

VL 18

IS 10

SI SI

BP 1837

EP 1841

DI 10.1016/j.intermet.2010.02.036

UT WOS:000281420700012

ER

PT J

AU Sheng, JF

Feng, HB

Yu, RH

AF Sheng Jianfeng

Feng Haibo

Yu Ronghai

TI Fabrication and magnetic properties of Y-Fe-Co-B bulk metallic glasses

SO RARE METAL MATERIALS AND ENGINEERING

AB A new Y6Fe60.5Co11.5B22 Fe-based bulk metallic glass with a maximum diameter of 2 mm has been fabricated by suction casting method in a water-cooled copper mould. The interrelationship between cooling rates and magnetic properties were investigated and the critical cooling rate of the amorphous alloy was calculated. The bulk amorphous alloy has a coercivity of 2.53 A/m, a saturated magnetization of 1.24 T, and a high initial susceptibility. The critical cooling rate (R-c) of the Y6Fe60.5Co11.5B22 amorphous alloy was determined to be about 119 K/s.

SN 1002-185X

PD JUN

PY 2007

VL 36

IS 6

BP 1078

EP 1081

UT WOS:000248201800033

ER

PT J

AU Wu, XL

Hong, YS

AF Wu, XL

Hong, YS

TI Novel Fe70Zr10Ni6Al4Si6B4 thick metallic glass coating produced by laser

cladding

SO MATERIALS SCIENCE AND TECHNOLOGY

AB A novel multicomponent thick metallic glass coating has been synthesised by laser cladding. The maximum coating thickness was I mm. The clad cooling rate restrained the epitaxial growth of dendrites in the metallic glass coating. The metallic glass had high glass forming ability with a wide supercooled liquid region ranging from 59 to 70 K. The metallic glass coating also revealed high hardness and good corrosion resistance. (C) 2001 IoM Communications Ltd.

SN 0267-0836

PD AUG

PY 2001

VL 17

IS 8

BP 1025

EP 1028

DI 10.1179/026708301101510906

UT WOS:000170670600022

ER

PT S

AU Lee, MH

Jun, JH

Eckert, J

AF Lee, Min Ha

Jun, Joong Hwan

Eckert, Juergen

BE Nie, JF

Morton, A

TI Effect of Residual Stress on Mechanical Property of Monolithic Bulk

Metallic Glass

SO PRICM 7, PTS 1-3

SE Materials Science Forum

CT 7th Pacific Rim International Conference on Advanced Materials and

Processing

CY AUG 02-06, 2010

CL Cairns, AUSTRALIA

SP Chinese Soc Metals, Japan Inst Metals, Korean Inst Metals & Mat, Mat Australia, Minerals, Met & Mat Soc

AB Mechanical treatments such as deep rolling are known to affect the strength and toughness of metallic glass due to the residual stress. It is well known that compressive residual stress states usually enhance the mechanical properties in conventional metallic materials. We present investigations on the change of fracture behavior related with mechanical properties of "brittle" bulk metallic glass by cold rolling at room temperature. Improvement of the intrinsic plasticity is observed not only after constrained cyclic compression but also after cold rolling. Moreover, neither nanocrystallization nor phase separation occurs during deformation. By these findings we provide a unique fundamental basis by considering the introduction of structural inhomogeneity and ductility improvement in metallic glasses. The experimental evidence clearly supports that such an inhomogeneous glassy can be produced by residual stress in well known "brittle" bulk metallic glasses, and does not depend on a specific pinpointed chemical composition.

OI LEE, MIN HA/0000-0001-6006-0628

SN 0255-5476

PY 2010

VL 654-656

BP 1050

EP +

DI 10.4028/www.scientific.net/MSF.654-656.1050

PN 1-3

UT WOS:000285374600257

ER

PT J

AU Duan, G

Lind, ML

Demetriou, MD

Johnson, WL

Goddard, WA

Cagin, T

Samwer, K

AF Duan, Gang

Lind, Mary Laura

Demetriou, Marios D.

Johnson, William L.

Goddard, William A., III

Cagin, Tahir

Samwer, Konrad

TI Strong configurational dependence of elastic properties for a binary

model metallic glass

SO APPLIED PHYSICS LETTERS

AB In this work, the strong dependence of elastic properties on configurational changes in a Cu-Zr binary metallic glass assessed by molecular dynamics simulations is reported. By directly evaluating the temperature dependence and configurational potential energy dependence of elastic constants, the shear modulus dependence on the specific configurational inherent state of metallic glasses is shown to be much stronger than the dependence on Debye-Gruneisen thermal expansion. (c) 2006 American Institute of Physics.

RI Duan, Gang/B-3188-2009

SN 0003-6951

PD OCT 9

PY 2006

VL 89

IS 15

AR 151901

DI 10.1063/1.2360203

UT WOS:000241247900027

ER

PT J

AU Shao, Y

Yao, KF

Li, M

Liu, X

AF Shao, Yang

Yao, Kefu

Li, Mo

Liu, Xue

TI Two-zone heterogeneous structure within shear bands of a bulk metallic

glass

SO APPLIED PHYSICS LETTERS

AB Shear bands, the main plastic strain carrier in metallic glasses, are severely deformed regions often considered as disordered and featureless. Here we report the observations of a sandwich-like heterogeneous structure inside shear bands in Pd40.5Ni40.5P19 metallic glass sample after plastic deformation by high-resolution transmission electron microscopy. The experimental results suggest a two-step plastic deformation mechanism with corresponding microstructure evolution at atomic scale, which may intimately connected to the stability of the shear band propagation and the overall plastic deformability. (C) 2013 AIP Publishing LLC.

RI Liu, Xue/J-8111-2014; Shao, Yang/H-2722-2013

OI Liu, Xue/0000-0002-4966-3417; Shao, Yang/0000-0001-5369-9933

SN 0003-6951

EI 1077-3118

PD OCT 21

PY 2013

VL 103

IS 17

AR 171901

DI 10.1063/1.4826117

UT WOS:000326455100023

ER

PT J

AU Ding, D

Wang, P

Guan, Q

Tang, MB

Xia, L

AF Ding Ding

Wang Peng

Guan Quan

Tang Mei-Bo

Xia Lei

TI Excellent Glass Forming Ability and Refrigeration Capacity of a

Gd55Al20Ni12Co10Mn3 Bulk Metallic Glass

SO CHINESE PHYSICS LETTERS

AB We investigate an excellent refrigeration capacity R-c of Gd55Al20Ni12Co10Mn3 bulk metallic glass (BMG). The Gd55Al20Ni12Co10Mn3 glassy rod is subjected to Cu mold suction-casting to prepare bulky metallic glasses, with a diameter of 3 mm. The glass forming ability as well as the magnetic properties of the BMG is investigated. The BMG exhibits a rather high glass formation ability with critical diameter of about 5.6 mm. The peak value of magnetic entropy change of about 8 J.kg(-1)K(-1) is obtained in this alloy. This BMG alloy also exhibits excellent magnetic refrigerant capacity of about 880 J.kg(-1) under the field of 5T and 35% larger than that of other alloys reported previously, supposed to be closely related to the high effective moment (similar to 7.3 mu(B)) of the Gd55Al20Ni12Co10Mn3 BMG.

OI Xia, Lei/0000-0001-9198-1497

SN 0256-307X

PD SEP

PY 2013

VL 30

IS 9

AR 096104

DI 10.1088/0256-307X/30/9/096104

UT WOS:000324374800027

ER

PT J

AU Wang, ZT

Zeng, KY

Li, Y

AF Wang, Z. T.

Zeng, K. Y.

Li, Y.

TI The correlation between glass formation and hardness of the amorphous

phase

SO SCRIPTA MATERIALIA

AB We report a non-monotonous behavior of the hardness of amorphous Cu-Zr films as a function of composition, by applying a combinatorial deposition and nanoindentation method with unparalleled compositional resolution. Distinct peaks in hardness were identified at particular compositions to correlate well with the previously reported density peaks. Our results not only facilitate the discovery of new glass-forming alloys, but also raise the possibility of developing bulk metallic glasses with enhanced plasticity and/or ductility for engineering applications. (C) 2011 Acta Materialia Inc. Published by Elsevier Ltd. All rights reserved.

RI Zeng, Kaiyang/C-3413-2008

OI Zeng, Kaiyang/0000-0002-3348-0018

SN 1359-6462

PD NOV

PY 2011

VL 65

IS 9

BP 747

EP 750

DI 10.1016/j.scriptamat.2011.06.043

UT WOS:000295765300001

ER

PT J

AU Chen, D

Takeuchi, A

Inoue, A

AF Chen, Ding

Takeuchi, Akira

Inoue, Akihisa

TI Gd-Co-Al and Gd-Ni-Al bulk metallic glasses with high glass forming

ability and good mechanical properties

SO MATERIALS SCIENCE AND ENGINEERING A-STRUCTURAL MATERIALS PROPERTIES

MICROSTRUCTURE AND PROCESSING

AB The glass forming ability of Gd-Co-Al and Gd-Al-Ni ternary alloy systems with a composition range of 50-70 at.% Gd and 0-40 at.% Al were investigated by copper mold casting. Bulk glassy alloys with composition of Gd60Co25Al15 and Gd60Ni15Al25 with the maximum diameters of 5 and 4 mm, respectively, were produced in the Gd-Al-Co and Gd-Al-Ni alloy systems The reduce glass transformation temperature (T-g/T-1) and the distance of supercooling region Delta T-x are 0.6 and 45 K for the Gd60Co25Al15 and Gd60Ni15Al25 bulk metallic glasses, respectively. The compressive fracture strength sigma(f) and Young's modulus E of these two glassy alloys are 1250 MPa and 63 GPa for Gd60Co25Al15, 1300 MPa and 65 GPa for Gd60Ni15Al25 alloy, respectively. The Gd-Co-Al and Gd-Al-Ni bulk glassy alloys with high glass forming ability and good mechanical properties are promising for the future development as a new type function materials. (C) 2006 Elsevier B.V. All rights reserved.

RI Inoue, Akihisa/E-5271-2015; 陈(chen), 鼎(ding)/O-5087-2015; 鼎,

陈/D-1695-2009; Takeuchi, Akira/A-3619-2010

OI 陈(chen), 鼎(ding)/0000-0003-0407-7542; 鼎, 陈/0000-0001-6422-4597;

Takeuchi, Akira/0000-0002-7246-8644

SN 0921-5093

EI 1873-4936

PD MAY 25

PY 2007

VL 457

IS 1-2

BP 226

EP 230

DI 10.1016/j.msea.2006.12.028

UT WOS:000246216900034

ER

PT J

AU Nishiyama, N

Amiya, K

Inoue, A

AF Nishiyama, Nobuyuki

Amiya, Kenji

Inoue, Akihisa

TI Novel applications of bulk metallic glass for industrial products

SO JOURNAL OF NON-CRYSTALLINE SOLIDS

CT 12th International Conference on Liquid and Amorphous Metals (LAM12)

CY JUL 11-16, 2004

CL Metz, FRANCE

AB The possibility of applying metallic glass parts in industrial products is investigated. Ti-based glassy tubes have been applied to a Coriolis mass flowmeter, and the prototype of the flowmeter exhibits 28.5 times higher sensitivity than that of the conventional one made of SUS630. Zr55Al10Ni5Cu30 glassy diaphragms are applied in pressure sensors. The prototype of pressure sensor with a Zr-based glassy diaphragm exhibits 3.8 times higher sensitivity than that of the sensor with the conventional SUS630 diaphragm. The obtained results suggest that innovative products can be produced by using of metallic glass parts, and metallic glass is promising as an industrial material with outstandingly high performance for the near future. (c) 2007 Elsevier B.V. All rights reserved.

RI Amiya, Kenji/P-6132-2014; Inoue, Akihisa/E-5271-2015; Nishiyama,

Nobuyuki/C-8228-2015

SN 0022-3093

PD OCT 15

PY 2007

VL 353

IS 32-40

BP 3615

EP 3621

DI 10.1016/j.jnoncrysol.2007.05.170

UT WOS:000250235200127

ER

PT J

AU Gong, P

Yao, KF

Ding, HY

AF Gong, Pan

Yao, Kefu

Ding, Hongyu

TI CENTIMETER-SIZED Ti-BASED QUATERNARY BULK METALLIC GLASS PREPARED BY

WATER QUENCHING

SO INTERNATIONAL JOURNAL OF MODERN PHYSICS B

AB Till now, the developed centimeter-sized Ti-based bulk metallic glasses (BMGs) always consist of at least five elements. We report that Ti41Zr25Be28Fe6 quaternary glassy alloy can be made up to 10 mm in diameter by water quenching, while only phi 8 mm fully glassy rod can be obtained by copper mould suction casting. This alloy possesses fewer constituent elements, wider supercooled liquid region and higher specific strength than other developed centimeter-sized Ti-based BMGs and has wide prospect for practical application. Our results also indicate that for Ti41Zr25Be28Fe6 alloy which possesses relatively strong glass-forming ability, reducing the heterogeneous impurities in the melt is more effective to obtain fully glassy samples than increasing the cooling rate merely.

RI Gong, Pan/K-8870-2012

OI Gong, Pan/0000-0002-3833-8440

SN 0217-9792

EI 1793-6578

PD JUL 20

PY 2013

VL 27

IS 18

AR 1350087

DI 10.1142/S0217979213500872

UT WOS:000321626600006

ER

PT J

AU Liang, L

Hui, X

Zhang, CM

Chen, GL

AF Liang, L.

Hui, X.

Zhang, C. M.

Chen, G. L.

TI A Dy-based bulk metallic glass with high thermal stability and excellent

magnetocaloric properties

SO JOURNAL OF ALLOYS AND COMPOUNDS

AB A new heavy rare-earth-based Dy36Ho20Al24Co20 bulk metallic glass (BMG) with high thermal stability and excellent magnetocaloric properties has been prepared by a copper mold casting. Compared with the other known rare-earth-based BMGs, this BMG alloy possess higher glass transition temperature and crystallization temperature, larger effective activation energy for glass transition and crystallization. Under a modest magnetic field, this BMG alloy exhibits a comparable or even larger magnetocaloric effect than that the previously reported RE-based BMGs and crystalline compounds. The high thermal stability and the excellent magnetocaloric effect together with other merits of the BMGs make this BMG alloy suitable candidate for use as magnetic refrigerant in a temperature range below 50 K. (C) 2007 Elsevier B.V. All rights reserved.

RI Hui, Xidong/A-1741-2010

SN 0925-8388

PD SEP 8

PY 2008

VL 463

IS 1-2

BP 30

EP 33

DI 10.1016/j.jallcom.2007.09.041

UT WOS:000258697300009

ER

PT J

AU Wang, XY

Wang, WK

Zhan, ZJ

Xu, FY

Zhang, NY

Wang, FX

Chen, Y

Pang, YT

Zhao, LP

Wang, J

AF Wang, X. Y.

Wang, W. K.

Zhan, Z. J.

Xu, F. Y.

Zhang, N. Y.

Wang, F. X.

Chen, Y.

Pang, Y. T.

Zhao, L. P.

Wang, J.

TI Compression behaviour and micro-structure evaluation of

Zr57Nb5Cu15.4Ni12.6Al10 bulk metallic glass under high pressure

SO MATERIALS LETTERS

AB Compression behaviour and micro-structure evaluation of Zr57Nb5Cu15.4Ni12.6Al10 bulk metallic glass is investigated at room temperature up to 32.8 GPa using in-situ high pressure energy dispersive X-ray diffraction with a synchrotron radiation source. The equation of state of the bulk metallic glass is -Delta V/V=0.012P-2.49 x 10(-4)p(2)-9.5 x 10(-7) P-3 + 5.02 x 10(-8)P(4). The result shows that the nearest atom pair of the as-quenched bulk metallic glass corresponds to Zr-Zr correlations. And with pressure increasing, the nearest atom pair changes to a new one at 32.8 GPa. (c) 2006 Elsevier B.V All rights reserved.

SN 0167-577X

PD MAY

PY 2007

VL 61

IS 11-12

BP 2170

EP 2172

DI 10.1016/j.matlet.2006.08.041

UT WOS:000246158300012

ER

PT J

AU Tang, MB

Bai, HY

Pan, MX

Zhao, DQ

Wang, WH

AF Tang, MB

Bai, HY

Pan, MX

Zhao, DQ

Wang, WH

TI Einstein oscillator in highly-random-packed bulk metallic glass

SO APPLIED PHYSICS LETTERS

AB Metallic glasses have often been regarded as ideal model systems of dense random packing with strong interaction among their components. Here we report direct evidence for the presence of the Einstein oscillator with an Einstein temperature theta(E) of 74 K, which induces the boson peak at 4.9 meV in bulk metallic glass Zr46.75Ti8.25Cu7.5Ni10Be27.5. The presence of an Einstein oscillator suggests the existence of the vibrations of loose atoms in an independent localized harmonic mode in the highly random packed metallic glasses. (C) 2005 American Institute of Physics.

SN 0003-6951

EI 1077-3118

PD JAN 10

PY 2005

VL 86

IS 2

AR 021910

DI 10.1063/1.1849420

UT WOS:000226701500028

ER

PT J

AU Duan, G

De Blauwe, K

Lind, ML

Schramm, JP

Johnson, WL

AF Duan, Gang

De Blauwe, Katrien

Lind, Mary Laura

Schramm, Joseph P.

Johnson, William L.

TI Compositional dependence of thermal, elastic, and mechanical properties

in Cu-Zr-Ag bulk metallic glasses

SO SCRIPTA MATERIALIA

AB Starting from the two binary bulk glass formers in the Cu-Zr system, we systematically investigated the compositional dependence of glass formation, thermal, elastic, and mechanical properties in the Cu-Zr-Ag ternary bulk glassy alloys. Both CU(50-x)-Zr50Agx and CU(64-x)Zr36Agx series show a good combination of high glass-forming ability and high Poisson's ratio. However, compressive plastic deformation was only observed in CU(50-x)Zr50Agx series and the possible underlying mechanism is discussed. (c) 2007 Acta Materialia Inc. Published by Elsevier Ltd. All rights reserved.

RI Duan, Gang/B-3188-2009

SN 1359-6462

PD FEB

PY 2008

VL 58

IS 3

BP 159

EP 162

DI 10.1016/j.scriptamat.2007.10.001

UT WOS:000251943200001

ER

PT J

AU Dong, M

Hao, T

Yong, Z

Yi, L

AF Dong, M

Hao, T

Yong, Z

Yi, L

TI Correlation between glass formation and type of eutectic coupled zone in

eutectic alloys

SO MATERIALS TRANSACTIONS

AB Glass formation is basically to avoid nucleation upon quenching. However, the growth of each nucleus is also important as certain undercooling is needed. Thus the subsequent competition between the growth of crystalline phases and the formation of amorphous phase should be considered. In this paper, we summarized our recent studies in Pd-Ni-Cu-P system and La-Cu-Ni-Al alloy systems. It is concluded that the glass forming ability of a eutectic alloy system depends on the type of the eutectics, i.e. symmetric or asymmetric eutectic coupled zone. For the alloy systems with symmetric eutectic coupled zone, the best glass forming alloys should be at or very close to the eutectic composition. For the alloys with asymmetric eutectic coupled zone, the best glass forming alloys should be at off-eutectic compositions.

SN 1345-9678

EI 1347-5320

PD OCT

PY 2003

VL 44

IS 10

BP 2007

EP 2010

UT WOS:000186411800016

ER

PT J

AU Kato, H

Chen, HS

Inoue, A

AF Kato, H.

Chen, H. -S.

Inoue, A.

TI Relationship between thermal expansion coefficient and glass transition

temperature in metallic glasses

SO SCRIPTA MATERIALIA

AB The thermal expansion coefficients of 13 metallic glasses were measured using a thermo-mechanical analyser. A unique correlation was found between the linear thermal expansion coefficient and the glass transition temperature-their product is nearly constant similar to 8.24 x 10(-3). If one assumes the Debye expression for thermal activation, the total linear thermal expansion up to glass transition temperature (T-g) is reduced to 6 x 10(-3), nearly 25% of that at the fusion of pure metals. (C) 2008 Acta Materialia Inc. Published by Elsevier Ltd. All rights reserved.

RI Kato, Hidemi/B-2492-2015; Inoue, Akihisa/E-5271-2015

SN 1359-6462

PD JUN

PY 2008

VL 58

IS 12

BP 1106

EP 1109

DI 10.1016/j.scriptamat.2008.02.006

UT WOS:000255900200018

ER

PT J

AU Yu, ZW

Liu, Y

Li, J

Lian, LX

Sun, WZ

Liu, H

AF Yu Zhengwei

Liu Ying

Li Jun

Lian Lixian

Sun Wenze

Liu Hai

TI Fabrication of FeSiB Bulk Metallic Glasses by Spark Plasma Sintering

SO RARE METAL MATERIALS AND ENGINEERING

AB The glass transition temperature (T(g)), onset crystallization temperature (T(x)) and supercooled liquid region (Delta T(x)) of FeSiB glassy powders were tested by DSC. Bulk metallic glasses 10 mm in diameter and 7 mm in height with a relative density of 92.3% were fabricated by spark plasma sintering using glassy powders. The phase composition, microstructure, magnetic properties and compressive strength of bulk samples were discussed by XRD, SEM, VSM and universal experiment machine, respectively. The results indicate the optimal relative density of bulk metallic glasses have been obtained at the sintering temperature of 360 degrees C and under the external applied pressure of 500 MPa. The bulk amorphous alloy has a compressive strength of 1200 MPa and a saturated magnetization of 1.44 T. After crystallization at 400 degrees C, the bulk sample has a compressive strength of 2039 MPa and a saturated magnetization of 1.54 T.

SN 1002-185X

PD APR

PY 2009

VL 38

SU 1

BP 44

EP 47

UT WOS:000276258900010

ER

PT J

AU Gonzalez, S

Figueroa, IA

Zhao, H

Davies, HA

Todd, I

Adeva, P

AF Gonzalez, S.

Figueroa, I. A.

Zhao, H.

Davies, H. A.

Todd, I.

Adeva, P.

TI Effect of mischmetal substitution on the glass-forming ability of

Mg-Ni-La bulk metallic glasses

SO INTERMETALLICS

AB The effect of La substitution by LaMM (lanthanum mischmetal) on the glass-forming ability of a Mg-Ni-La metallic glass has been studied. For compositions close to the eutectic Mg(69)Ni(18)La(13), the LaMM substitution improved the glass-forming ability. for instance up to a critical amorphous diameter of 2 mm for the Mg(65)Ni(20)LaMM(15) alloy. However, of all the alloys studied, it has been observed that La-containing alloys have higher GFA for compositions further from the eutectic and a maximum critical amorphous diameter of 2.5 mm was obtained for the Mg(60)Ni(23.6)La(16.4) alloy. (C) 2009 Elsevier Ltd. All rights reserved.

RI Gonzalez, Sergio/A-4852-2010; Figueroa, Ignacio/J-5914-2012

OI Todd, Iain/0000-0003-0217-1658; Gonzalez Sanchez,

Sergio/0000-0002-0211-7822; Adeva, Paloma/0000-0002-9111-8893

SN 0966-9795

PD NOV

PY 2009

VL 17

IS 11

BP 968

EP 971

DI 10.1016/j.intermet.2009.04.012

UT WOS:000269164600016

ER

PT J

AU Liu, Y

Zhu, YT

Luo, XK

Liu, ZM

AF Liu, Yong

Zhu Yitian

Luo Xuekun

Liu, Zuming

TI Wear behavior of a Zr-based bulk metallic glass and its composites

SO JOURNAL OF ALLOYS AND COMPOUNDS

AB The wear behaviors of Zr(52.5)Cu(17.9)Al(10)Ni(14.6)Ti(5) metallic glasses with different contents of crystalline phases were studied by using a pin-on-disc test. The results indicate that the friction coefficient of the metallic glass with a steel counterpart is in the range of 0.24-0.32. Both surface softening and crystallization occur on the surface of the metallic glass during wear, and the wear curve is not as stable as the crystalline materials due to the interaction of the two processes. The wear mechanism of the metallic glass may change with wear conditions and the crystallinity. The fully amorphous material shows an abrasive wear at a low load, then adhesive wear at a high load. Increasing the crystallinity results in more abrasive wear. The wear behaviors of the metallic glass and its crystalline composites do not follow the Archard's equation. Only a good combination of the hardness and the toughness can the metallic glass be wear resistant. (C) 2010 Elsevier B.V. All rights reserved.

SN 0925-8388

PD JUL 30

PY 2010

VL 503

IS 1

BP 138

EP 144

DI 10.1016/j.jallcom.2010.04.217

UT WOS:000280623000030

ER

PT J

AU Park, ES

Chang, HJ

Kim, DH

AF Park, E. S.

Chang, H. J.

Kim, D. H.

TI Improvement of glass-forming ability and phase separation in Cu-Ti-rich

Cu-Ti-Zr-Ni-Si bulk metallic glasses

SO JOURNAL OF ALLOYS AND COMPOUNDS

CT 16th International Symposium on Metastable, Amorphous and Nanostructured

Materials

CY JUL 05-09, 2009

CL Beijing, PEOPLES R CHINA

AB Present study reports improvement of glass-forming ability (GFA) and phase separation in Cu-Ti-rich Cu-Ti-Zr-Ni-Si bulk metallic glasses (BMGs) by tailoring the constituent elements. The MA of metalloid element, Sn having relatively large negative enthalpy of mixing can lead to improve GFA (up to 8 mm in diameter) as well as thermal stability (up to Delta T-x = 48 K) by optimizing the substitution element. And the addition of elements having relatively large positive enthalpy of mixing (partial substitution of Zr or Ti with Y) can lead to the liquid state phase separation in Cu-Ti-Sn-Zr-Ni-Si BMG, although the addition lead to drastic deterioration of the GFA. (C) 2010 Elsevier B.V. All rights reserved.

RI Kim, Do Hyang/J-6575-2012; Park, Eun Soo/A-9860-2014; Park, Eun

Soo/A-4443-2008; bang, changwook/J-7922-2012

SN 0925-8388

PD AUG

PY 2010

VL 504

SU 1

BP S27

EP S30

DI 10.1016/j.jallcom.2010.04.129

UT WOS:000285252600008

ER

PT J

AU Shao, G

AF Shao, G

TI Thermodynamic and kinetic aspects of intermetallic amorphous alloys

SO INTERMETALLICS

AB The glass transition in metallic alloy systems can be modelled thermodynamically, using the Calphad approach, as a second-order transition from the supercooled liquid phase, giving good predictability for glass transition temperatures and the thermodynamic stability of the amorphous phase in intermetallic alloy systems. The resultant thermodynamic database can also be used for calculating crystallisation temperatures for the glass devitrification, and the Calphad approach is a powerful tool for designing bulk metallic glass alloys. (C) 2003 Elsevier Science Ltd. All rights reserved.

RI Shao, Guosheng/C-2143-2016

OI Shao, Guosheng/0000-0003-1498-7929

SN 0966-9795

PD APR

PY 2003

VL 11

IS 4

BP 313

EP 324

DI 10.1016/S0966-9795(02)00248-0

UT WOS:000181381300005

ER

PT J

AU Wang, JF

Liu, L

Zou, H

Pu, J

Xiao, JZ

AF Wang, JF

Liu, L

Zou, H

Pu, J

Xiao, JZ

TI Study on the structure relaxation of Pd40Ni10Cu30P20 bulk metallic glass

SO RARE METAL MATERIALS AND ENGINEERING

AB The structural relaxation of Pd40Ni10Cu30P20 bulk metallic glass below glass transition temperature Tg was investigated by differential scanning calorimetry (DSC). The results reveal that the structural relaxation in the bulk metallic glass can be divided into two stages corresponding to two different temperature regions. In temperature range from 400 K to 520 K, the low temperature structure relaxation occurs. The structure change induced by this is local or short-range and the free volume don't considerably annihilate. out So the density of the alloy only shows a slight change with the increase of the annealing temperature. However, in the temperature range from 520 K to Tg, the high temperature structure relaxation occur, in which the intermediate-range and long-range atomic diffusion would take place and the free volume in the bulk metallic glass can largely annihilat through sample surface. This causes a considerable increase in the density with the annealing temperature. It was also found that the preannealing relaxation has a heavy effect on the kinetics of the glass transition, while little affect on the subsequent crystallization processes.

SN 1002-185X

PD JAN

PY 2005

VL 34

IS 1

BP 98

EP 101

UT WOS:000226733700022

ER

PT J

AU Bakai, SO

Scheretskiy, OA

Bakai, KS

Gorbatenko, VM

Volchok, OI

AF Bakai, S. O.

Scheretskiy, O. A.

Bakai, K. S.

Gorbatenko, V. M.

Volchok, O. I.

TI EFFECT OF HIGH FREQUENCY PRE LOAD ON MECHANICAL AND THERMO PHYSICAL

PROPERTIES OF BULK METALLIC GLASS (Zr55Cu30Al10Ni5)(99)Y-1

SO PROBLEMS OF ATOMIC SCIENCE AND TECHNOLOGY

AB The effect of pre-high-frequency (ultrasonic) mechanical loading on the thermo physical and mechanical properties of samples of a new, long-term structural material - the bulk metallic glass (Zr55Cu30Al10Ni5)(99)Y-1. The dependence of the thermophysical and mechanical properties of metallic glass on the parameters of the high-frequency loading of the material.

SN 1562-6016

PY 2016

IS 2

BP 78

EP 85

UT WOS:000413416900012

ER

PT J

AU Rodrigues, BP

Zanotto, ED

AF Rodrigues, Bruno Poletto

Zanotto, Edgar Dutra

TI Evaluation of the guided random parameterization method for critical

cooling rate calculations

SO JOURNAL OF NON-CRYSTALLINE SOLIDS

AB We focus on a recently suggested approach to the calculation of critical cooling rates for glass formation. It is a "random parameterization" method that is guided by a limited number of isothermal scanning calorimetry experiments. However, several assumptions have been made in its derivation that may not mirror the actual crystallization behavior of most supercooled liquids, which may jeopardize the estimation of glass forming ability. We evaluate those assumptions and the applicability of the method is tested for lithium disilicate glass (which displays moderate internal nucleation rates) and dibarium titanium silicate glass (which displays very high internal nucleation rates, similar to those of metallic glasses). Both glasses nucleate homogeneously and exhibit polymorphic crystallization. Our calculations show that some overlooked variables, such as the sample geometry, nucleation induction-times, surface crystallization and the breakdown of the Stokes-Einstein/Eyring equation, have significant roles on the calculated time-temperature-transformation curves during heating experiments. We demonstrate that the proposed random parameterization method can only be used when a glass forming liquid that undergoes internal crystallization is cooled from above its liquidus to various test temperatures. If the sample undergoes predominant surface crystallization or if it is heated to the test temperature several corrections must be made. (C) 2012 Elsevier B.V. All rights reserved.

RI Zanotto, Edgar/A-7992-2011

OI Zanotto, Edgar/0000-0003-4931-4505

SN 0022-3093

PD SEP 15

PY 2012

VL 358

IS 18-19

BP 2626

EP 2634

DI 10.1016/j.jnoncrysol.2012.06.010

UT WOS:000311175900011

ER

PT J

AU Li, Z

Bai, HY

Zhao, DQ

Pan, MX

Wang, WL

Wang, WH

AF Li, Z

Bai, HY

Zhao, DQ

Pan, MX

Wang, WL

Wang, WH

TI Hard magnetic Pr55Al12Fe30Cu3 bulk metallic glass

SO ACTA PHYSICA SINICA

AB In this paper we report the formation of a new Pr-55 Al-12 Fe-30 Cu-3 bulk metallic glass. Cylindrical Pr-55 Al-12 Fe-30 Cu-3 specimens of 5 mm in diameter and 100 mm in length were prepared by copper mold suction casting. Differential scanning calorimetry results indicated that the Pr-based bulk metallic glass system has a wide supercooled liquid region about 64K. The melting temperature of the amorphous state is about 140K higher than that of its crystalline state. Hysteresis loops were measured, and the results indicated that the Pr-55 Al-12 Fe-30 Cu-3 bulk metallic has hard magnetic property, while the completely crystallized alloy has soft magnetic property at room temperature.

SN 1000-3290

PD MAR

PY 2003

VL 52

IS 3

BP 652

EP 655

UT WOS:000181642200026

ER

PT J

AU Ding, SY

Liu, YH

Li, YL

Liu, Z

Sohn, S

Walker, FJ

Schroers, J

AF Ding, Shiyan

Liu, Yanhui

Li, Yanglin

Liu, Ze

Sohn, Sungwoo

Walker, Fred J.

Schroers, Jan

TI Combinatorial development of bulk metallic glasses

SO NATURE MATERIALS

AB The identification of multicomponent alloys out of a vast compositional space is a daunting task, especially for bulk metallic glasses composed of three or more elements. Despite an increasing theoretical understanding of glass formation, bulk metallic glasses are predominantly developed through a sequential and time-consuming trial-and-error approach. Even for binary systems, accurate quantum mechanical approaches are still many orders of magnitude away from being able to simulate the relatively slow kinetics of glass formation. Here, we present a high-throughput strategy where similar to 3,000 alloy compositions are fabricated simultaneously and characterized for thermoplastic formability through parallel blow forming. Using this approach, we identified the composition with the highest thermoplastic formability in the glass-forming system Mg-Cu-Y. The method provides a versatile toolbox for unveiling complex correlations of material properties and glass formation, and should facilitate a drastic increase in the discovery rate of metallic glasses.

RI LIU, Yanhui/B-1485-2009

OI Walker, Frederick/0000-0002-8094-249X

SN 1476-1122

EI 1476-4660

PD MAY

PY 2014

VL 13

IS 5

BP 494

EP 500

DI 10.1038/NMAT3939

UT WOS:000334845600021

PM 24728462

ER

PT J

AU Gerold, U

Wiedenmann, A

Keiderling, U

Fecht, HJ

AF Gerold, U

Wiedenmann, A

Keiderling, U

Fecht, HJ

TI Decomposition and crystallization of the bulk amorphous Zr11Ti34Cu47Ni8

alloy studied by SANS

SO PHYSICA B

CT 1st European Conference on Neutron Scattering (ECNS 96)

CY OCT 08-11, 1996

CL INTERLAKEN, SWITZERLAND

SP European Neutron Scattering Assoc, Paul Scherrer Inst, Villigen, Swiss Soc Neutron Scattering

AB The microstructural evolution upon thermal annealing of the bulk amorphous alloy Zr11Ti34Cu47Ni8 was investigated by means of small-angle neutron scattering (SANS) and X-ray diffraction. The results are compared with those from the metallic glass Zr(41)Ti(14)Ci(12.5)Ni(10)Be(22.5). In both cases, inhomogeneities of about 2 nm extension have been detected upon annealing and interpreted as decomposition of the amorphous phase. In the Be-free metallic glass crystallization occurred simultaneously with the decomposition.

SN 0921-4526

PD JUN

PY 1997

VL 234

BP 995

EP 996

DI 10.1016/S0921-4526(96)01236-7

UT WOS:A1997XG66600368

ER

PT J

AU Lenain, A

Blandin, JJ

Kapelski, G

Volpi, F

Gravier, S

AF Lenain, A.

Blandin, J. J.

Kapelski, G.

Volpi, F.

Gravier, S.

TI Hf-rich bulk metallic glasses as potential insulating structural

material

SO MATERIALS & DESIGN

AB Tremendous properties of bulk metallic glasses (BMGs) have led to numerous potential applications in the past fewyears. An obvious interest has been given to their potential as structural material given their high mechanical properties and their ability to be produced in bulk parts of several millimeters in size. Unlike their crystalline counterparts, they also show very promising insulating properties due to the absence of a periodic lattice. In the present work, the interest of using bulk amorphous metallic alloys as materials combining good structural properties and thermal insulation is discussed. The study of Hf- rich BMGs enables to reduce the thermal losses through a higher sigma c/k performance ratio and thus shows very good potential for future insulating and structural applications. (C) 2017 Elsevier Ltd. All rights reserved.

SN 0264-1275

EI 1873-4197

PD FEB 5

PY 2018

VL 139

BP 467

EP 472

DI 10.1016/j.matdes.2017.11.030

UT WOS:000423753200048

ER

PT J

AU Kabaer, M

Ovalioglu, H

Kucuk, I

AF Kabaer, M.

Ovalioglu, H.

Kucuk, I.

TI Computational Modeling of Magnetic Properties and Glass Forming Ability

of Bulk Amorphous Materials

SO ACTA PHYSICA POLONICA A

CT 14th Czech and Slovak Conference on Magnetism

CY JUN 06-09, 2010

CL Kosice, SLOVAKIA

SP Safarik Univ, Fac Sci, Inst Phys, Slovak Acad Sci, Slovak Phys Soc

AB A model based on artificial neural network was designed for the simulation and estimation of glass forming ability parameters and saturation magnetization and coercivity of bulk glassy alloys Its performance is evaluated by the influences of different kinds of alloys and elements on the glass forming ability and magnetic properties The values of glass forming ability parameters and saturation magnetization and coercivity values estimated by artificial neural network agree well with the experimental values, indicating that the model is reliable and adequate

SN 0587-4246

EI 1898-794X

PD NOV

PY 2010

VL 118

IS 5

BP 827

EP 828

DI 10.12693/APhysPolA.118.827

UT WOS:000285797100048

ER

PT J

AU Kim, WJ

Ma, DS

Jeong, HG

AF Kim, WJ

Ma, DS

Jeong, HG

TI Superplastic flow in a Zr65Al10Ni10Cu15 metallic glass crystallized

during deformation in a supercooled liquid region

SO SCRIPTA MATERIALIA

AB The deformation behavior of a powder-metallurgy processed Zr65Al10Ni10Cu15 metallic glass was characterized in the supercooled liquid region under a condition where crystallization is controlled to occur during deformation. The alloy exhibited a tensile elongation as large as 750% at 6.3 x 10(-3) s(-1)-696 K though crystallization occurred during deformation. (C) 2003 Published by Elsevier Ltd. on behalf of Acta Materialia Inc.

SN 1359-6462

PD DEC

PY 2003

VL 49

IS 11

BP 1067

EP 1073

DI 10.1016/j.scriptamat.2003.08.014

UT WOS:000185451400003

ER

PT J

AU Ren, YL

Zuo, JH

Qiu, KQ

Zhang, HF

Hu, ZQ

AF Ren, YL

Zuo, JH

Qiu, KQ

Zhang, HF

Hu, ZQ

TI A new Nd-based bulk metallic glass

SO ACTA METALLURGICA SINICA

AB A new Nd(61)Fe(30)Zn(9) bulk metallic glass with a diameter of 2.5 mm was obtained using copper mold casting. No glass transition temperature or supercooled liquid region was observed by using conventional DSC-7 facility. The crystallization temperature for the bulk sample is 730 K, which is about 70 K lower than that of Nd(60)Fe(30)Al(10) bulk metallic glass. The melting characteristics of Nd(61)Fe(30)Zn(9) alloy show that its compositional is at a eutectic point, but the alloys exhibit non-eutectic microstructure due to high tendency of undercooled ability.

SN 0412-1961

PD MAR

PY 2004

VL 40

IS 3

BP 301

EP 304

UT WOS:000220611700015

ER

PT J

AU Men, H

Kim, WT

Kim, DH

AF Men, H

Kim, WT

Kim, DH

TI Fabrication and mechanical properties of Mg65Cu15Ag5Pd5Gd10 bulk

metallic glass

SO MATERIALS TRANSACTIONS

AB The effect of partial substitution of Cu for Ag and Pd on the glass forming ability of Mg65Cu2.5Gd10 alloy has been studied. Mg65Cu15Ag5Pd5Gd10 bulk metallic glass with diameter of at least 10 mm can successfully be fabricated by conventional Cu-mold casting method in air atmosphere. The critical cooling rate for glass formation is estimated to be similar to0.7 K/s. The compressive fracture strength and fracture elongation of the Mg65Cu15Ag5Pd5Gd10 bulk metallic glass are 817 MPa and 1.6% respectively.

RI bang, changwook/J-7922-2012

SN 1345-9678

EI 1347-5320

PD OCT

PY 2003

VL 44

IS 10

BP 2141

EP 2144

DI 10.2320/matertrans.44.2141

UT WOS:000186411800037

ER

PT J

AU Li, HX

Kim, KB

Yi, S

AF Li, H. X.

Kim, K. B.

Yi, S.

TI Enhanced glass-forming ability of Fe-based bulk metallic glasses

prepared using hot metal and commercial raw materials through the

optimization of Mo content

SO SCRIPTA MATERIALIA

AB The glass-forming ability of Fe-based metallic glass Fe71.2-xC7.0Si3.3B5.5P8.7Cr2.3Al2.0Mox (x = 0-6.5 at.%), which can be produced in large quantities and cost-effectively using hot metal and commercial raw materials, was significantly enhanced by optimizing the Mo content, leading to the formation of a fully amorphous rod of 6 mm in diameter with high compressive fracture strength (similar to 3.3 GPa). It is concluded that the enhanced glass-forming ability is attributed to the suppression of the primary alpha-(Fe, Mo) phase by the optimum amount of Mo substitution for Fe. (c) 2007 Acta Materialia Inc. Published by Elsevier Ltd. All rights reserved.

SN 1359-6462

PD JUN

PY 2007

VL 56

IS 12

BP 1035

EP 1038

DI 10.1016/j.scriptamat.2007.02.038

UT WOS:000246605300008

ER

PT J

AU Ping, W

De, QZ

Ming, XP

Wei, HW

Jia, PS

Yu, PS

AF Ping, W

De, QZ

Ming, XP

Wei, HW

Jia, PS

Yu, PS

TI Relaxation behaviors of bulk metallic glass forming

Zr46.75Ti8.25Cu7.5Ni10.0Be27.5 alloy

SO INTERMETALLICS

CT 3rd International Conference on Bulk Metallic Glasses

CY OCT 12-16, 2003

CL Beijing, PEOPLES R CHINA

AB Low frequency internal friction and differential scanning calorimetry were applied to investigate calorimetric glass transition as well as relaxation characteristics of the supercooled liquid states in a bulk metallic glass-forming Zr46.75Ti8.25Cu7.5Ni10.0Be27.5 alloy. A dramatic decrease in structural relaxation time of the supercooled liquid was exhibited by internal friction measurements. The dependence of temperature on the relaxation time fits well with the Vogel-Tammann-Fulcher form. Based on the free volume theory the calorimetric glass transition was explained as the relaxation of supercooled liquid inspired at the experimental timescale. The results exhibit that the calorimetrical glass transition is a mainly kinetic process in this glass. (C) 2004 Elsevier Ltd. All rights reserved.

SN 0966-9795

PD OCT-NOV

PY 2004

VL 12

IS 10-11

BP 1245

EP 1249

DI 10.1016/j.intermet.2004.04.031

UT WOS:000224566700034

ER

PT J

AU Aydiner, CC

Ustundag, E

AF Aydiner, CC

Ustundag, E

TI Residual stresses in a bulk metallic glass cylinder induced by thermal

tempering

SO MECHANICS OF MATERIALS

AB Bulk metallic glasses are attractive structural materials that develop residual stresses during processing due to thermal tempering. In the present study, stress generation is analyzed experimentally and theoretically in a bulk metallic glass cylinder. By using a stainless steel tube as mold, thermal contact is assured throughout casting and quenching to better define the boundary conditions of the problem. The resulting residual stresses are measured using the crack compliance method. It is shown that high stresses can be attained in metallic glasses due to thermal tempering: about -300 MPa compression on the surface balanced by +150 MPa tension in the middle. The finite element method is then employed to deduce the convection heat transfer coefficient during quenching and to model the residual stress generation. The latter analysis is performed with a previously developed viscoelastic model. This model is shown to be accurate within 15-30% of the experimental stress data. It is therefore a powerful tool for estimating processing-induced residual stresses in bulk metallic glasses. (C) 2004 Elsevier Ltd. All rights reserved.

RI Ustundag, Ersan/C-1258-2009; Aydiner, Cahit/O-9618-2017

OI Ustundag, Ersan/0000-0002-0812-7028; Aydiner, Cahit/0000-0001-8256-6742

SN 0167-6636

PD JAN

PY 2005

VL 37

IS 1

BP 201

EP 212

DI 10.1016/j.mechmat.2004.03.001

UT WOS:000224589700014

ER

PT J

AU Chen, HC

Yan, L

Liu, RD

Tang, MB

Wang, G

Huang, HF

Hai, Y

Zhou, XT

AF Chen, H. C.

Yan, L.

Liu, R. D.

Tang, M. B.

Wang, G.

Huang, H. F.

Hai, Y.

Zhou, X. T.

TI Anisotropic nanocrystallization of a Zr-based metallic glass induced by

Xe ion irradiation

SO INTERMETALLICS

AB Structural modification in a Zr-based metallic glass caused by irradiation with 7 MeV Xe26+ ions was investigated. Needle-like nanocrystalline structures, formed under ion irradiation, consist of Cu10Zr7 phase (primary) and/or minor (NixCu1-x)(10)Zr-7 phase. The formation of needle-like nanocrystals suggested an anisotropic atomic diffusion caused by ion irradiation. (C) 2014 Elsevier Ltd. All rights reserved.

SN 0966-9795

EI 1879-0216

PD SEP

PY 2014

VL 52

BP 15

EP 19

DI 10.1016/j.intermet.2014.03.006

UT WOS:000336873700003

ER

PT J

AU Packard, CE

Witmer, LM

Schuh, CA

AF Packard, C. E.

Witmer, L. M.

Schuh, C. A.

TI Hardening of a metallic glass during cyclic loading in the elastic range

SO APPLIED PHYSICS LETTERS

AB Although fatigue failure is well documented in metallic glasses, the mechanism responsible for damage accumulation during cyclic loading below the yield point remains elusive. This letter describes a high-resolution nanomechanical study of an Fe-based bulk metallic glass subjected to cyclic loading in the nominal elastic range. An increase in the yield load was observed with an increasing number of subyield loading cycles, providing a clean documentation of kinematic irreversibility in very small volumes of material that experience no shear bands either prior to or during cyclic loading. (C) 2008 American Institute of Physics.

RI Packard, Corinne/A-9606-2010; Schuh, Christopher/C-7947-2009

OI Packard, Corinne/0000-0002-5815-8586; /0000-0001-9856-2682

SN 0003-6951

PD APR 28

PY 2008

VL 92

IS 17

AR 171911

DI 10.1063/1.2919722

UT WOS:000255524000029

ER

PT J

AU Cui, J

Li, JS

Wang, J

Li, LY

Kou, HC

AF Cui, Jing

Li, Jinshan

Wang, Jun

Li, Liyuan

Kou, Hongchao

TI Deformation behavior of a Ti-based bulk metallic glass composite in the

supercooled liquid region

SO MATERIALS & DESIGN

AB Deformation behavior of the Ti50Zr20Nb12Cu5Be13 bulk metallic glass composite in the supercooled liquid region have been investigated by uniaxial compressive as well as tensile tests. The results indicated that all stress-strain curves show work-hardening and work-softening phenomenon. The high temperature deformation behavior is sensitive to strain rate and temperature. Rheological behavior of Ti50Zr20Nb12Cu5Be13 bulk metallic glass composite was analyzed by activation volume quantitatively, which implied that the Ti50Zr20Nb12Cu5Be13 has better rheological behavior than that of conventional Ti alloy, but not as good as Ti-based bulk metallic glass. In addition, dendrite morphology of the Ti50Zr20Nb12Cu5Be13 almost does not change after deformation, and tensile fracture is full of dimples. (C) 2015 Elsevier Ltd. All rights reserved.

RI WANG, Jun/A-1526-2015

OI WANG, Jun/0000-0001-8101-2967; Li, Jinshan/0000-0002-6894-9760

SN 0261-3069

EI 1873-4197

PD JAN 15

PY 2016

VL 90

BP 595

EP 600

DI 10.1016/j.matdes.2015.10.129

UT WOS:000367235100071

ER

PT J

AU Huang, YJ

Xue, P

Guo, S

Wu, Y

Cheng, X

Fan, HB

Ning, ZL

Cao, FY

Xing, DW

Sun, JF

Liaw, PK

AF Huang, Yongjiang

Xue, Peng

Guo, Shu

Wu, Yang

Cheng, Xiang

Fan, Hongbo

Ning, Zhiliang

Cao, Fuyang

Xing, Dawei

Sun, Jianfei

Liaw, Peter K.

TI Liquid-solid joining of bulk metallic glasses

SO Scientific Reports

AB Here, we successfully welded two bulk metallic glass (BMG) materials, Zr51Ti5Ni10Cu25Al9 and Zr50.7Cu28Ni9Al12.3 (at. %), using a liquid-solid joining process. An atomic-scale metallurgical bonding between two BMGs can be achieved. The interface has a transition layer of similar to 50 mu m thick. The liquid-solid joining of BMGs can shed more insights on overcoming their size limitation resulting from their limited glass-forming ability and then promoting their applications in structural components.

RI Huang, Yongjiang/D-4809-2009

SN 2045-2322

PD JUL 29

PY 2016

VL 6

AR 30674

DI 10.1038/srep30674

UT WOS:000380652600001

PM 27471073

ER

PT J

AU Bobrov, OP

Khonik, VA

Laptev, SN

Yazvitsky, MY

AF Bobrov, OP

Khonik, VA

Laptev, SN

Yazvitsky, MY

TI Comparative internal friction study of bulk and ribbon glassy

Zr52.5Ti5CU17.9Ni14.6Al10

SO SCRIPTA MATERIALIA

AB The results of comparative internal friction (IF) investigation of a Zr-based metallic glass prepared in the bulk and ribbon forms are presented. It is shown that absolute values and temperature alterations of the IF and normalized modulus during heating of bulk and ribbon samples are fairly similar. This fact indicates that change of the quenching rate by three to four orders of magnitude either does not affect the amount of the free volume frozen in or the relaxation centres responsible for the relaxation behavior are not connected with the free volume. The possible consequences of this observation are discussed. (C) 2003 Acta Materialia Inc. Published by Elsevier Science Ltd. All rights reserved.

RI Khonik, Vitaly/A-5888-2009

SN 1359-6462

PD AUG

PY 2003

VL 49

IS 3

BP 255

EP 260

DI 10.1016/S1359-6462(03)00212-4

UT WOS:000183334300010

ER

PT J

AU Alamgir, FM

Jain, H

Schwarz, RB

Jin, O

Williams, DB

AF Alamgir, FM

Jain, H

Schwarz, RB

Jin, O

Williams, DB

TI Electronic structure of Pd-based bulk metallic glasses

SO JOURNAL OF NON-CRYSTALLINE SOLIDS

CT 9th International Conference on the Physics of Non-Crystalline Solids

(PNS 1999)

CY OCT 17-21, 1999

CL TUCSON, ARIZONA

SP NASA, Div Micrograv Sci & Applicat, NSF, Div Mat Res, Guardian Ind Corp, Elsevier Ltd

AB From high-resolution X-ray photoelectron spectroscopy (XPS) of PdxNi(80-x)P20 bulk metallic glasses (BMGs) we observe bonding states in the ternary glasses that cannot be simulated by linear combinations of the valence bands of the binaries. Thus, the bonding in the ternary glasses is qualitatively different from those of the binary alloys of this composition range. Further, we observe that there are more such states in Pd40Ni40P20 than in Pd40.5Cu40.5P19, explaining the greater glass-formability of the former. Within the bulk glass formation range, 30 less than or equal to x less than or equal to 60, the valence band of the most stable glass, Pd40Ni40P20, is found to be a linear combination of those of the glasses at the extremes of this range. Comparison of XPS core level binding energies of glasses and crystals in PdxNi(80-x)P20 show that the electronic structure of glasses is closest to that of the corresponding crystals at x = 30, which agrees with a minimum in the heat of crystallization for this composition. (C) 2000 Elsevier Science B.V. All rights reserved.

OI Alamgir, Faisal/0000-0002-0894-8096

SN 0022-3093

PD SEP

PY 2000

VL 274

IS 1-3

BP 289

EP 293

DI 10.1016/S0022-3093(00)00192-7

UT WOS:000089298300040

ER

PT J

AU Shin, HS

Chang, SN

Kim, DK

AF Shin, Hyung-Seop

Chang, Soon-Nam

Kim, Do Kyung

TI Deformation behaviors of Zr-based bulk metallic glass under impact

indentation

SO INTERNATIONAL JOURNAL OF MODERN PHYSICS B

CT 6th International Symposium on Impact Engineering

CY SEP 16-19, 2007

CL Daejeon, SOUTH KOREA

AB Metallic glasses are amoiphous meta-stable solids and are now being processed in bulk form suitable for structural applications under impact loading. Bulk metallic glasses have many unique mechanical properties such as high yield strength and fracture toughness, good corrosion and wear resistance that distinguish them from crystalline metals and alloys. However, only a few studies could be found mentioning the dynamic response and damage of metallic glasses under impact or shock loading. In this study, we employed a small explosive detonator for the dynamic indentation to a Zr-based bulk amorphous metal in order to evaluate the damage behavior of bulk amorphous metal under impact or shock loading conditions. Results were compared with those of spherical indentation under quasistatic and impact loading and were discussed. The interface bonded specimen method was adopted in order to observe the subsurface damage, especially the formation of shear bands induced during indentation under different loading conditions.

SN 0217-9792

PD APR 30

PY 2008

VL 22

IS 9-11

BP 1775

EP 1782

DI 10.1142/S0217979208047407

UT WOS:000256702600115

ER

PT J

AU Luo, CY

Zhao, YH

Xi, XK

Wang, G

Zhao, DQ

Pan, MX

Wang, WH

Kou, SZ

AF Luo, CY

Zhao, YH

Xi, XK

Wang, G

Zhao, DQ

Pan, MX

Wang, WH

Kou, SZ

TI Making amorphous steel in air by rare earth microalloying

SO JOURNAL OF NON-CRYSTALLINE SOLIDS

AB Recently great advancement in the fabrication of structural amorphous steels (SASs) with large cross-section sizes has been obtained. One of the remained key problems for the manufacturability and the application of SASs is that their glass-forming ability is very sensitive to the processing environments. Here we present the discovery that the bulk amorphous alloy Fe44Mn11Cr10Mo12C15B6Ho2 has high oxygen resistance and can be prepared by conventional copper-mold casting method in air atmosphere. Rod-shaped amorphous samples of diameter up to 6 mm at least have been fabricated successfully. This amorphous steel alloy showed high glass-forming ability and good manufacturability. The beneficial effects of the addition of holmium on the glass-forming ability and oxygen resistance during the SAS formation are explored. (c) 2005 Elsevier B.V. All rights reserved.

RI Wang, Gang/K-2630-2012

SN 0022-3093

PD FEB 1

PY 2006

VL 352

IS 2

BP 185

EP 188

DI 10.1016/j.jnoncrysol.2005.11.013

UT WOS:000234963000014

ER

PT J

AU Yao, KF

Chen, N

AF Yao, Kefu

Chen Na

TI Pd-Si binary bulk metallic glass

SO SCIENCE IN CHINA SERIES G-PHYSICS MECHANICS & ASTRONOMY

AB Pd(80+x) Si(20-x) (x = 0, 1, and 2) binary metallic glasses with the diameter ranging from 7 to 8 mm were prepared by a combination of fluxing and water quenching or air cooling. Thermal analysis results show that with increasing Si content, the glass transition temperature T(g), the initial crystallization temperature T(x) and the onset crystallization temperature T(p) of Pd-Si binary glassy alloys increase. Moreover, the supercooled liquid region reaches 61 K. It indicates that Pd-Si binary alloys possess large glass forming ability, which can be greatly improved by fluxing treatment.

RI Chen, Na/A-4120-2010

SN 1672-1799

PD APR

PY 2008

VL 51

IS 4

BP 414

EP 420

DI 10.1007/s11433-008-0051-4

UT WOS:000254406000010

ER

PT J

AU Pang, SJ

Zhang, T

Asami, K

Inoue, A

AF Pang, SJ

Zhang, T

Asami, K

Inoue, A

TI Synthesis of Fe-Cr-Mo-C-B-P bulk metallic glasses with high corrosion

resistance

SO ACTA MATERIALIA

AB Bulk metallic glasses with a maximum thickness (t(max)) of 1.0-2.7 mm were synthesized in the Fe43Cr16Mo16(C, B, P)(25) system over a wide composition range by copper mold casting. They exhibit a large supercooled liquid region (DeltaT(x)) of 40-90 K and a high reduced glass transition temperature (T-g/T-l) of 0.54-0.60, indicating high glass-forming ability (GFA) and high thermal stability of the supercooled liquid. The critical cooling rate for glass formation was evaluated to be of the order of 10(2) K s(-1). The bulk metallic glasses exhibited high corrosion resistance in aggressive HCl solutions. The alloying element P has a beneficial effect on corrosion resistance. (C) 2002 Acta Materialia Inc. Published by Elsevier Science Ltd. All rights reserved.

RI Inoue, Akihisa/E-5271-2015; Zhang, Tao/O-4911-2014; Pang,

Shujie/D-8305-2016

SN 1359-6454

PD FEB 8

PY 2002

VL 50

IS 3

BP 489

EP 497

DI 10.1016/S1359-6454(01)00366-4

UT WOS:000173480200004

ER

PT J

AU Singh, D

Yadav, TP

Mandal, RK

Tiwari, RS

Srivastava, ON

AF Singh, Devinder

Yadav, T. P.

Mandal, R. K.

Tiwari, R. S.

Srivastava, O. N.

TI Effect of Ga substitution on the crystallization behaviour and glass

forming ability of Zr-Al-Cu-Ni alloys

SO MATERIALS SCIENCE AND ENGINEERING A-STRUCTURAL MATERIALS PROPERTIES

MICROSTRUCTURE AND PROCESSING

AB The crystallization behaviour of melt spun Zr(69.5)Al(7.5-x)Ga(x)Cu(12)Ni(11) (x = 0-7.5; in at.%) metallic glasses has been investigated by X-ray diffraction (XRD), transmission electron microscopy (TEM) and differential scanning calorimetry (DSC). The DSC traces showed changes in crystallization behaviour with substitution of Ga. Formation of single nano-quasicrystalline phase by controlled crystallization of glasses has been found only for 0 <= x <= 1.5. Further increase of Ga content gives rise to formation of the quasicrystals together with Zr(2)Cu type crystalline phase. In addition to this, the substitution of Ga influences the size and shape of nano-quasicrystals. The glass forming abilities (GFAs) of these metallic glasses were assessed by the recognition of glass forming ability indicators, i.e. reduced glass transition temperature (T(rg)) and supercooled liquid region (Delta T(x)). The glass transition temperature (T(g)) has been observed for all the melt spun ribbons. (C) 2009 Elsevier B.V. All rights reserved.

RI Yadav, Thakur Prasad/A-3030-2012

SN 0921-5093

PD JAN 15

PY 2010

VL 527

IS 3

BP 469

EP 473

DI 10.1016/j.msea.2009.10.020

UT WOS:000273983800008

ER

PT J

AU Inoue, A

Nishiyama, N

AF Inoue, Akihisa

Nishiyama, Nobuyuki

TI New bulk metallic glasses for applications as magnetic-sensing,

chemical, and structural materials

SO MRS BULLETIN

AB Since 1988, it has been demonstrated that metallic glasses can be made in bulk form with diameters larger than several millimeters. At present, several alloy systems with maximum diameters for glass formation exceeding 1 cm are known. As a result, Zr-, Ti-, Fe-, Co-, Ni-, and Cu-based bulk metallic glasses (BMGs) are already in use for magnetic-sensing, chemical, and structural applications. In this article, recently developed BMGs with critical diameters of more than 1 cm are summarized, and some of their industrial applications are reviewed.

SN 0883-7694

PD AUG

PY 2007

VL 32

IS 8

BP 651

EP 658

DI 10.1557/mrs2007.128

UT WOS:000248827300018

ER

PT J

AU Kobata, J

Takigawa, Y

Chung, SW

Tsuda, H

Kimura, H

Higashi, K

AF Kobata, J.

Takigawa, Y.

Chung, S. W.

Tsuda, H.

Kimura, H.

Higashi, K.

TI Nanoscale amorphous "band-like" structure induced by friction stir

processing in Zr55Cu30Al10Ni5 bulk metallic glass

SO MATERIALS LETTERS

AB Friction stir processing (FSP) is successfully applied in Zr-based bulk metallic glass in this study. In the FSP specimen obtained, large defects and cracks are not present in the FSP region. The microstructure in the friction zone (FZ) exhibited an amorphous "band-like" structure with widths of 10-45 nm and a small number of nanoscale crystalline particles were observed along the "band-like" structure. We concluded that the nanoscale amorphous "band-like" structure obtained in this study is nanoscale shear bands. From this result, FSP is expected to be a useful method of processing bulk metallic glass with nanoscale shear bands to investigate the influence of shear bands on the deformation behavior of bulk metallic glass. (c) 2006 Elsevier B.V. All rights reserved.

RI Higashi, Kenji/C-5540-2011; Takigawa, Yorinobu/A-6555-2010; Kimura,

Hisamichi/D-5449-2012

OI Takigawa, Yorinobu/0000-0003-0321-6336;

SN 0167-577X

PD JUL

PY 2007

VL 61

IS 17

BP 3771

EP 3773

DI 10.1016/j.matlet.2006.12.031

UT WOS:000247787200056

ER

PT J

AU Sarlar, K

Kucuk, I

AF Sarlar, Kagan

Kucuk, Ilker

TI Phase separation and glass forming ability of

(Fe0.72Mo0.04B0.24)(100-x)Gd-x (x=4, 8) bulk metallic glasses

SO JOURNAL OF NON-CRYSTALLINE SOLIDS

AB The effect of Gd addition on the Glass Forming Ability (GFA), magnetic and thermal properties in (Fe0.72Mo0.04B0.24)(100-x)Gd-x (x = 4, 8) bulk metallic glasses were investigated. The examined amorphous alloys'. structural, thermal and magnetic properties were investigated by using X-ray diffraction (XRD), scanning electron mi-croscopy (SEM) differential scanning calorimeter (DSC) and vibrating sample magnetometer (VSM). The results show that molten (Fe0.72Mo0.04B0.24)(92)Gd-8 presents the fully amorphous structure and solidifies into two different Fe-rich and B-rich amorphous phases. The DSC curve shows that the (Fe0.72Mo0.04B0.24)(92)Gd-8 has supercooled liquid region (Delta T-x) of about 70 K and the saturation magnetization (J(s)) and coercivity (H-c) for as-cast bulk metallic glasses (BMGs) was 0.53 T and 223 A/m respectively. (C) 2016 Elsevier B.V. All rights reserved.

SN 0022-3093

EI 1873-4812

PD SEP 1

PY 2016

VL 447

BP 198

EP 201

DI 10.1016/j.jnoncrysol.2016.06.003

UT WOS:000381841200030

ER

PT J

AU Wu, GJ

Li, R

Liu, ZQ

Chen, BQ

Zhang, T

AF Wu GuoJuan

Li Ran

Liu ZengQian

Chen BingQing

Zhang Tao

TI Effects of the laser surface treatment on the mechanical properties of

CuZr-based bulk metallic glasses

SO SCIENCE CHINA-PHYSICS MECHANICS & ASTRONOMY

AB Surfaces of three types of CuZr-based bulk metallic glasses (BMGs) were modified by laser surface treatment (LST), and the influence of the treatment on structure and mechanical properties of these alloys was investigated. The phase structure of as-cast and laser-treated samples was characterized by XRD and the morphology of the alloys after fracture was examined by SEM. The compressive plasticity of treated Cu47.5Zr47.5Al5 and Cu46.5Zr47.5Al5Co1 BMGs can be improved from 0.5% to 2.0% and from 1.2% to 5.7% respectively compared with the as-cast ones, while (Cu0.55Zr0.40Al0.05)(99)Er-1 BMG shows insignificant change of plasticity. The improvement in plasticity is attributed to induced crystallization of B2 CuZr phase in the treated surface zone of selected metallic glasses.

RI Chen, Bingqing/R-6300-2016; Li, Ran/B-4618-2010; Zhang, Tao/O-4911-2014

SN 1674-7348

PD MAY

PY 2013

VL 56

IS 5

BP 925

EP 927

DI 10.1007/s11433-013-5016-6

UT WOS:000318516200010

ER

PT J

AU Zhang, Y

Zhou, YJ

Lin, JP

Chen, GL

Liaw, PK

AF Zhang, Yong

Zhou, Yun Jun

Lin, Jun Pin

Chen, Guo Liang

Liaw, Peter K.

TI Solid-solution phase formation rules for multi-component alloys

SO ADVANCED ENGINEERING MATERIALS

RI Lin, Junpin/D-1468-2013; ZHANG, Yong/B-7928-2009

OI ZHANG, Yong/0000-0002-6355-9923

SN 1438-1656

PD JUN

PY 2008

VL 10

IS 6

BP 534

EP 538

DI 10.1002/adem.200700240

UT WOS:000257525200003

ER

PT J

AU Chi, BQ

Jiang, Q

AF Chi, BQ

Jiang, Q

TI Mixing entropy difference between liquid and crystal of Zr base

amorphous alloys

SO ADVANCED ENGINEERING MATERIALS

RI Jiang, Qing/F-4073-2012

OI Jiang, Qing/0000-0003-0660-596X

SN 1438-1656

EI 1527-2648

PD JUN

PY 2005

VL 7

IS 6

BP 512

EP 517

DI 10.1002/adem.200400158

UT WOS:000230412900012

ER

PT J

AU Jin, H

Wen, J

Lu, K

AF Jin, H

Wen, J

Lu, K

TI Shear stress induced reduction of glass transition temperature in a bulk

metallic glass

SO ACTA MATERIALIA

AB In contrast to previous observations of a slight increase in glass transition temperature (T-g) under hydrostatic pressure, we report, for the first time, a drastic reduction of T-g (40 K/500 MPa) induced by shear stress in a Zr46.75Ti8.25Cu7.5Ni10.0Be27.5 bulk metallic glass, which gives rise to the experimental observation of the drastic decrease in flow temperature while a compressive stress was applied. The significant T-g reduction is attributed to a large activation volume of relaxation &UDelta; V-t under shear stress (126 &ANGS;(3)), and can be quantitatively interpreted in terms of the free volume model. &COPY; 2005 Acta Materialia Inc. Published by Elsevier Ltd. All rights reserved.

RI Jin, Hai-Jun/E-5179-2010

SN 1359-6454

PD JUN

PY 2005

VL 53

IS 10

BP 3013

EP 3020

DI 10.1016/j.actamat.2005.03.015

UT WOS:000229519400016

ER

PT J

AU Shen, TD

Sun, BR

Xin, SW

AF Shen, T. D.

Sun, B. R.

Xin, S. W.

TI Effects of metalloids on the thermal stability and glass forming ability

of bulk ferromagnetic metallic glasses

SO JOURNAL OF ALLOYS AND COMPOUNDS

AB We prepared three types of bulk ferromagnetic glasses, Fe-Mo-P-C, Fe-Mo-Ga-P-B-C, and Fe-(Co, Sb, Cr, Mo, Ga)-P-B-C by a flux-melting and water-quenching technique. We systematically changed the content of both alloying metals and metalloids to improve the glass-forming ability (GFA) and thermal stability - the difference between crystallization temperature T-x and glass transition temperature T-g of bulk ferromagnetic glasses. One of our flux-melted ferromagnetic alloys with optimized compositions has a critical cooling rate on the order of similar to 10 K s(-1), suggesting that the flux treatments play an important role in improving the GFA. Tuning the thermal stability of supercooled liquid by modifying the content of alloying metalloids is more effective than by modifying the content of alloying metals. Adding 1 at.% alloying metalloids and metals can increase T-x-T-g by similar to 20 K and 4 K, respectively. We found that upon increasing the content of alloying metalloids, T-x-T-g increases and reaches a maximum where the GFA is highest. After T-x-T-g reaches a maximum, a further increase in the content of alloying metalloids lowers the GFA whereas T-x-T-g may either increase or decrease. Bulk ferromagnetic glasses can only be formed by modifying the content of metalloids within a narrow range, similar to 2-6 at.%, in our alloy systems. Our experimental results suggest that optimizing the content of metalloids is a powerful tool for improving the GFA and thermal stability of bulk metallic glasses. (C) 2015 Elsevier B.V. All rights reserved.

SN 0925-8388

EI 1873-4669

PD MAY 15

PY 2015

VL 631

BP 60

EP 66

DI 10.1016/j.jallcom.2015.01.070

UT WOS:000350388800010

ER

PT J

AU Hong, SH

Kim, JT

Mun, SC

Kim, YS

Park, HJ

Na, YS

Lim, KR

Park, JM

Kim, KB

AF Hong, Sung Hwan

Kim, Jeong Tae

Mun, Sang Chul

Kim, Young Seok

Park, Hae Jin

Na, Young Sang

Lim, Ka Ram

Park, Jin Man

Kim, Ki Buem

TI Influence of spherical particles and interfacial stress distribution on

viscous flow behavior of Ti-Cu-Ni-Zr-Sn bulk metallic glass composites

SO INTERMETALLICS

AB Bulk metallic glass composites containing micro-scale B2 particles are subject to investigation with regards to the influence of B2 particles and interfacial stress and strain distribution on the viscous flow behavior at a supercooled liquid state. An increased volume fraction of B2 particles leads to an increase of minimum viscosity and influences viscous flow behavior before crystallization. In high temperature deformation, the bulk metallic glass shows homogeneous deformation feature. However, the heterogeneous deformation feature is found in thermoplastically deformed bulk metallic glass composite. The strong stain accumulation and sluggish viscous flow occur around B2 particles, which are caused by the heterogeneous stress distribution linked to stress concentration and shear stress impediment around B2 particles. The sluggish viscous flow of super-cooled liquids around B2 particles during high temperature deformation induces an increase of viscosity and strongly affects the viscous flow behavior of Ti-based bulk metallic glass composites containing micro-scale spherical B2 particles.

OI Kim, Ki Buem/0000-0002-6180-2715

SN 0966-9795

EI 1879-0216

PD DEC

PY 2017

VL 91

BP 90

EP 94

DI 10.1016/j.intermet.2017.08.016

UT WOS:000413386700013

ER

PT S

AU Wang, YF

Li, L

Sun, C

Lu, QL

Shi, ZQ

AF Wang, Yanfang

Li, Li

Sun, Chuan

Lu, Qinglong

Shi, Zhiqiang

BE Wang, RM

Wu, Y

Wu, XF

TI Rare Earth Elements on Glass-forming Ability and Thermal Stability of

Cu-Zr-Al Metallic Glass

SO NANO-SCALE AND AMOURPHOUS MATERIALS

SE Materials Science Forum

CT 11th International Conference in Asia of the

International-Union-of-Materials-Research-Societies

CY SEP 25-28, 2010

CL Qingdao, PEOPLES R CHINA

SP Int Union Mat Res Soc (IUMRS), Chinese Mat Res Soc, Mat Res Soc Taiwan, Mat Res Soc Japan, Govt Qingdao City

AB The rare earth elements (RE = Y, Sm, La, Ce) were used as alloying materials in Cu50Zr45Al5 BMG, and their influences on the glass-forming ability and thermal stability were studied in this paper. All the samples remained in full metallic glass state with minor additions of Y, Sm and La. Increasing the amount of RE additions, the Cu10Zr7 and Zr2Cu phases precipitated and glass transition temperature T-g and crystallization temperature T-x significantly decreased. The reduced glass transition temperature T-rg =T-g/T-l ranged from 0.592 to 0.611 and the gamma parameter ranged from 0.393 to 0.409.

SN 0255-5476

PY 2011

VL 688

BP 426

EP 430

DI 10.4028/www.scientific.net/MSF.688.426

UT WOS:000302644200075

ER

PT J

AU Li, M

Wang, CZ

Hao, SG

Kramer, MJ

Ho, KM

AF Li, Maozhi

Wang, C. Z.

Hao, S. G.

Kramer, M. J.

Ho, K. M.

TI Structural heterogeneity and medium-range order in ZrxCu100-x metallic

glasses

SO PHYSICAL REVIEW B

AB Realistic three-dimensional atomistic structures of ZrxCu100-x (x=35,50) bulk metallic glasses are constructed using a combination of x-ray diffraction experiment and computational modeling. A cluster correlation method is developed to analyze the medium-range order in amorphous systems. We show that the glass systems consist of a stringlike backbone network formed by icosahedral clusters and a liquidlike structure filling in the remaining space. These findings are consistent with those obtained from our independent classical molecular-dynamics studies with embedded-atom method potential for ZrCu system. Such a heterogeneous structure provides a fundamental structural perspective of dynamical heterogeneity and glass formation.

RI Hao, Shaogang/E-3527-2010; 石, 源/D-5929-2012; ruc, phy/E-4170-2012

SN 2469-9950

EI 2469-9969

PD NOV

PY 2009

VL 80

IS 18

AR 184201

DI 10.1103/PhysRevB.80.184201

UT WOS:000272310900051

ER

PT J

AU Parlar, Z

Bakkal, M

Shih, AJ

AF Parlar, Zeynep

Bakkal, Mustafa

Shih, Albert J.

TI Sliding tribological characteristics of Zr-based bulk metallic glass

SO INTERMETALLICS

AB Wear and friction characteristics of Zr-based bulk metallic glass under dry sliding conditions are investigated. This study demonstrates that load and sliding speed significantly affect the wear characteristics of bulk metallic glass material and sliding distance is less effective on wear. Critical sliding speed and normal force limits, 1 m/s and 10N, respectively, are distinguished. Overall average coefficient of friction value was in the range of 0.35-0.45, better than that of conventional structural materials such as AISI 6061-T6 and AISI 304. Analysis of the worn surface revealed that the bulk metallic glass was exposed to inhomogeneous shear deformation, adhesive wear, and abrasive wear during sliding test. Three sizes of wear debris, oversize flakes, machining chips and powder-like debris, are collected. There was no change recorded in surface and cross-sectional hardness measurements after the sliding test. This study concludes that bulk metallic glass is better in general friction characteristics than conventional structural materials such as AISI 6061-T6 and AISI 304. (c) 2007 Elsevier Ltd. All rights reserved.

SN 0966-9795

PD JAN

PY 2008

VL 16

IS 1

BP 34

EP 41

DI 10.1016/j.intermet.2007.07.011

UT WOS:000253168300005

ER

PT J

AU Brothers, AH

Scheunemann, R

DeFouw, JD

Dunand, DC

AF Brothers, AH

Scheunemann, R

DeFouw, JD

Dunand, DC

TI Processing and structure of open-celled amorphous metal foams

SO SCRIPTA MATERIALIA

AB Amorphous metallic foams with an open-cell structure are processed with the salt replication method by infiltration of a sintered salt pattern with liquid Vit106, a Zr-based bulk metallic glass. After pattern removal in nitric acid, the Vit106 foams exhibit highly uniform pores, about 250 mum in size, and relative densities in the range 15-22%. Processing parameters, including pattern selection, sintering, and removal, are investigated. (C) 2004 Acta Materialia Inc. Published by Elsevier Ltd. All rights reserved.

RI Dunand, David/B-7515-2009

OI Dunand, David/0000-0001-5476-7379

SN 1359-6462

PD FEB

PY 2005

VL 52

IS 4

BP 335

EP 339

DI 10.1016/j.scriptamat.2004.10.002

UT WOS:000225485400015

ER

PT J

AU Salimon, AI

Ashby, MF

Brechet, Y

Greer, AL

AF Salimon, AI

Ashby, MF

Brechet, Y

Greer, AL

TI Bulk metallic glasses: what are they good for?

SO MATERIALS SCIENCE AND ENGINEERING A-STRUCTURAL MATERIALS PROPERTIES

MICROSTRUCTURE AND PROCESSING

CT 11th International Conference on Rapidly Quenched and Metastable

Materials

CY AUG 25-30, 2002

CL Univ Oxford, Dept Mat, Oxford, ENGLAND

HO Univ Oxford, Dept Mat

AB Bulk metallic glasses (BMGs) are not cheap materials, and they are unlikely to become so for some time. Finding applications for them relies on identifying products in which the BMGs can offer sufficiently high improvements in performance to justify the cost. The research applies the notions of material-property space and performance indices and explores three strategies for systematically finding potential applications for a "new" material. Different approaches to compare the "new" material with conventional materials identify the potential victims for substitution, in which established current applications suggest promising applications for the "new" material. The output is a ranked list of technically viable applications. They are then subjected to a market analysis. Finally, an analysis is made of current and future applications of bulk metallic glasses. (C) 2003 Elsevier B.V. All rights reserved.

RI Greer, Alan Lindsay/G-1977-2011; Greer, Lindsay/E-9433-2017

SN 0921-5093

PD JUL 15

PY 2004

VL 375

SI SI

BP 385

EP 388

DI 10.1016/j.msea.2003.10.167

UT WOS:000223329700066

ER

PT J

AU Slipenyuk, A

Eckert, J

AF Slipenyuk, A

Eckert, J

TI Correlation between enthalpy change and free volume reduction during

structural relaxation of Zr55Cu30Al10Ni5 metallic glass

SO SCRIPTA MATERIALIA

AB The correlation between enthalpy change (DeltaH) and free volume reduction (Deltanu(f)) during structural relaxation of Zr55Cu30Al10Ni5 bulk metallic glass was investigated by differential scanning calorimetry (DSC) and density measurements of thermally treated samples. A linear dependence between DeltaH and Deltanu(f) over a wide temperature range was found. (C) 2003 Acta Materialia Inc. Published by Elsevier Ltd. All rights reserved.

SN 1359-6462

PD JAN

PY 2004

VL 50

IS 1

BP 39

EP 44

DI 10.1016/j.scriptamat.2003.09.038

UT WOS:000186207800008

ER

PT J

AU Gauthier, C

Pelletier, JM

Wang, Q

Blandin, JJ

AF Gauthier, C

Pelletier, JM

Wang, Q

Blandin, JJ

TI Viscoelastic and viscoplastic properties of bulk metallic glasses -

Comparison with oxide glasses and amorphous polymers

SO JOURNAL OF NON-CRYSTALLINE SOLIDS

CT 10th International Conference on the Physics of Non-Crystalline Solids

(PNCS)

CY JUL 13-17, 2003

CL Univ Parma, Parma, ITALY

HO Univ Parma

AB Mechanical behavior of non-crystalline solids is strongly dependent upon temperature: at low temperature they behave like elastic solids whereas at high temperature their behavior is that of a viscous liquid. In an intermediate temperature range, near the glass transition, viscoelastic behavior is preponderant, characterized by strong relaxation effects. In this paper, the viscoelastic behavior of bulk metallic glasses is compared to the one of oxide glasses and amorphous polymers. Both linear and non-linear aspects are considered. A special attention is paid to similarities and differences. (C) 2004 Elsevier B.V. All rights reserved.

SN 0022-3093

PD OCT 15

PY 2004

VL 345

BP 469

EP 472

DI 10.1016/j.jnoncrysol.2004.08.067

UT WOS:000225583200089

ER

PT J

AU Kiljan, A

Nowosielski, R

Babilas, R

AF Kiljan, Anna

Nowosielski, Ryszard

Babilas, Rafal

TI PROPERTIES AND STRUCTURES OF BULK METALLIC GLASSES BASED ON MAGNESIUM

SO MATERIALI IN TEHNOLOGIJE

AB Nowadays, Mg-based amorphous alloys are very attractive materials used in industries such as the automotive, aviation and medical industries. This group of bulk metallic glasses has specific properties such as corrosion resistance, strength and stiffness that are higher than those of crystalline ones. Also, Mg-based amorphous alloys are characterized by their high glass-forming ability, low density, good ductility, light weight, low cost, and good thermal and electrical conductivities. This work presents the basic information regarding metallic glasses. The project was focused on Mg-based bulk metallic glasses. The work shows the results of a differential thermal analysis (DTA), differential scanning calorimetry (DSC) and calorimetric surveys that determined the temperature at the beginning and at the end of crystallization. X-ray studies were performed and they confirmed the formation of the alloy's amorphous structure. The results of cross-sectional SEM and EDS were presented using a scanning electron microscope. This confirmed the homogeneity of the chemical composition and the structure of amorphous samples in the form of a plate with a thickness of 1 mm and a width of 5 mm. The value of the average sample microhardness is 295 HV.

SN 1580-2949

EI 1580-3414

PD JUL-AUG

PY 2017

VL 51

IS 4

BP 563

EP 567

DI 10.17222/mit.2015.300

UT WOS:000408399700002

ER

PT J

AU Wu, JL

Pan, Y

Li, XZ

Wang, XF

AF Wu, Jili

Pan, Ye

Li, Xingzhou

Wang, Xianfei

TI Microstructure evolution and mechanical properties of Nb-alloyed

Cu-based bulk metallic glasses and composites

SO MATERIALS & DESIGN

AB This paper reports the microstructure evolution of Cu-50.2 Zr40.8Ti9 Nb-x(x) (x = 0.5, 1.0, and 2.0 at.%) bulk metallic glass and bulk metallic glass composites accompanied with the addition of Nb and the corresponding mechanical properties. The X-ray diffraction and characterization of microstructures demonstrate that the microstructures of as-cast alloys undergo a composite-amorphous evolution. DSC analysis indicates that the glass-forming ability of as-cast alloys increases with addition of Nb. The microstructure evolution can be contributed to the combination of the stabilization of Nb on precipitated crystalline phases and cooling time. 1.0 at.% Nb-alloyed sample has the best plasticity (15.1%) and the highest fracture strength (2205 MPa) among three as-cast alloys. This work suggests that the uniformly dispersed tiny crystalline phases in glassy matrix can enhance the plasticity of bulk metallic glasses. (C) 2015 Elsevier Ltd. All rights reserved.

SN 0264-1275

EI 1873-4197

PD JUN 15

PY 2015

VL 75

BP 32

EP 39

DI 10.1016/j.matdes.2015.03.013

UT WOS:000352209200005

ER

PT J

AU Zhang, XL

Wang, JX

Sun, YX

Fu, YS

Liu, JC

AF Zhang Xiaoli

Wang Jinxiang

Sun Yuxin

Fu Yanshu

Liu Jiacong

TI Study progress of tungsten-fiber reinforced bulk metallic-glass matrix

composites

SO RARE METAL MATERIALS AND ENGINEERING

AB Bulk metallic glass reinforced by tungsten-fiber can not only keep its properties of high hardness and strength, but also has higher density and exhibits excellent self-sharpening behavior. Consequently tungsten-fiber reinforced bulk metallic-glass matrix composites attracted the experts at home and abroad a attention. In this paper, present research status of the fabrications, interface characteristics, mechanical properties and deformation behavior of the tungsten-fiber reinforced bulk metallic-glass matrix composites has been summarized and the research in the future was expected.

SN 1002-185X

PD AUG

PY 2008

VL 37

IS 8

BP 1323

EP 1328

UT WOS:000259154500002

ER

PT J

AU Mechler, N

Wanderka, N

Macht, MP

AF Mechler, S.

Wanderka, N.

Macht, M. -P.

TI Interdependence between glass stability and phase formation sequence

during crystallization of Zr46.8Ti8.2Cu7.5Ni10Be27.5 bulk glass

SO INTERNATIONAL JOURNAL OF MATERIALS RESEARCH

AB Knowledge of the crystallization sequence of metallic glasses can yield a deep insight into the origin of their thermal stability. Crystallization of metallic glasses often leads to the simultaneous formation of different metastable crystalline phases that makes their identification difficult. In order to separate formation of crystalline phases from each other, long term isothermal pre-annealing below the glass transition temperature of Zr46.8Ti8.2Cu7.5Ni10Be27.5 bulk metallic glass prior to crystallization was performed. It is found that this pre-annealing strongly influences the crystallization sequence and depending on the pre-annealing time leads to preferential formation of different phases. In addition to the well known intermetallic Be2Zr phase and a quasicrystalline phase, three further previously unknown crystalline phases are identified and described in terms of their structure and composition. The formation of the crystalline phases is discussed with respect to the glass forming ability and thermal stability of the glass phase.

SN 1862-5282

PD MAY

PY 2010

VL 101

IS 5

BP 601

EP 610

DI 10.3139/146.110324

UT WOS:000278825700008

ER

PT J

AU Dmowski, W

Gierlotka, S

Wang, Z

Yokoyama, Y

Palosz, B

Egami, T

AF Dmowski, W.

Gierlotka, S.

Wang, Z.

Yokoyama, Y.

Palosz, B.

Egami, T.

TI Pressure Induced Liquid-to-Liquid Transition in Zr-based Supercooled

Melts and Pressure Quenched Glasses

SO SCIENTIFIC REPORTS

AB Through high-energy x-ray diffraction and atomic pair density function analysis we find that Zr-based metallic alloy, heated to the supercooled liquid state under hydrostatic pressure and then quenched to room temperature, exhibits a distinct glassy structure. The PDF indicates that the Zr-Zr distances in this glass are significantly reduced compared to those quenched without pressure. Annealing at the glass transition temperature at ambient pressure reverses structural changes and the initial glassy state is recovered. This result suggests that pressure causes a liquid-to-liquid phase transition in this metallic alloy supercooled melt. Such a pressure induced transition is known for covalent liquids, but has not been observed for metallic liquids. The High Pressure Quenched glasses are stable in ambient conditions after decompression.

SN 2045-2322

PD JUL 26

PY 2017

VL 7

AR 6564

DI 10.1038/s41598-017-06890-w

UT WOS:000406364600076

PM 28747789

ER

PT J

AU Tamura, T

Kamikihara, D

Omura, N

Miwa, K

AF Tamura, Takuya

Kamikihara, Daisuke

Omura, Naoki

Miwa, Kenji

TI Effect of electromagnetic vibrations on Fe-Co-B-Si-Nb bulk metallic

glasses

SO MATERIALS TRANSACTIONS

AB It is known that cooling rate from the liquid state is an important factor for producing the bulk metallic glasses. However, almost no other factors such as electric and/or magnetic fields were investigated. The present authors have reported that a new method for producing Mg-Cu-Y bulk metallic glasses by using electromagnetic vibrations is effective in forming the metallic glass phase. Moreover, the present authors have reported that the glass-forming ability of Fe-Co-B-Si-Nb alloys also is enhanced with increasing the electromagnetic vibration force. Thus, this study aims to investigate effects of the electromagnetic vibrations on Fe-Co-B-Si-Nb bulk metallic glasses in order to investigate further. Half round lines which consist of fine crystal particles in a glassy phase were observed in the boundary part ofthe molybdenum electrode for the alloy with the electromagnetic vibrations. It was considered that the reactants of the molybdenum electrode which were solid at 1573 K were moved into the sample by the electromagnetic vibrations and caused the nuclei of crystal particles which composed the half round lines. If tungsten was used as the electrodes, there was no influence of electrodes in the center of the samples. When molybdenum or tungsten was used as the electrodes, the effect of the electromagnetic vibrations was found to be the same, namely the electromagnetic vibrations act on decreasing the number of crystal nuclei.

RI Omura, Naoki/M-1949-2018; Tamura, Takuya/D-4854-2017

OI Tamura, Takuya/0000-0001-6083-913X

SN 1345-9678

EI 1347-5320

PD JAN

PY 2007

VL 48

IS 1

BP 53

EP 57

DI 10.2320/matertrans.48.53

UT WOS:000244308600010

ER

PT J

AU Guo, SF

Chan, KC

Chen, Q

Li, JJ

Liu, L

AF Guo, S. F.

Chan, K. C.

Chen, Q.

Li, J. J.

Liu, L.

TI Tensile plastic deformation of a Zr-based bulk metallic glass composite

in the supercooled liquid region

SO SCRIPTA MATERIALIA

AB The tensile deformation behavior of Zr(55.9)Cu(18.6)Ni(10)Al(7.5)Ta(8) bulk metallic glass composite was investigated in the supercooled liquid region at various strain rates and temperatures. The deformation of the bulk metallic glass composite exhibited superplastic behavior, which is closely related to the strain rate and temperature. This excellent superplasticity, with a maximum elongation over 650%, indicates that the bulk Metallic glass composite looks promising for thermoplastic forming applications. (c) 2008 Acta Materialia Inc. Published by Elsevier Ltd. All rights reserved.

RI Guo, Shengfeng/E-3171-2012; Chen, Qi/A-6167-2010; Chan, K.C./A-2311-2014

OI Guo, Shengfeng/0000-0002-6667-6797; Chan, K.C./0000-0002-6173-5532

SN 1359-6462

PD MAR

PY 2009

VL 60

IS 6

BP 369

EP 372

DI 10.1016/j.scriptamat.2008.11.006

UT WOS:000263398100004

ER

PT J

AU Bae, DH

Park, JM

Na, JH

Kim, DH

Kim, YC

Lee, JK

AF Bae, DH

Park, JM

Na, JH

Kim, DH

Kim, YC

Lee, JK

TI Deformation behavior of Ti-Zr-Ni-Cu-Be metallic glass and composite in

the supercooled liquid region

SO JOURNAL OF MATERIALS RESEARCH

AB The deformation behavior of Ti-based bulk metallic glass (BMG) and metallic glass matrix composite (MGMC), both having a multistep crystallization behavior upon heating, has been investigated in the supercooled liquid region. The BMG deforms homogeneously and exhibits moderate elongation to failure due to its multistep crystallization behavior, but shows a significant variation of the flow stress during deformation. For the MGMC containing an in situ beta-phase, a stress-overshoot characteristic, observed in the BMG, is not presented, but elongation to failure is rather limited because the crystalline beta-phase prevents the viscous flow of the amorphous phase. The different presence of the crystalline phases in the metallic glasses can differently affect the flow behavior of metallic glass in the supercooled liquid region.

RI Juhyun, Oh/H-9185-2012; bang, changwook/J-7922-2012

SN 0884-2914

PD MAR

PY 2004

VL 19

IS 3

BP 937

EP 942

DI 10.1557/jmr.2004.19.3.937

UT WOS:000222316300035

ER

PT J

AU Kim, JT

Hong, SH

Bian, XL

Gokuldoss, PK

Song, KK

Eckert, J

Park, JM

Kim, KB

AF Kim, Jeong Tae

Hong, Sung Hwan

Bian, Xilei

Gokuldoss, Prashanth Konda

Song, Kaikai

Eckert, Jurgen

Park, Jin Man

Kim, Ki Buem

TI Effect of boron addition on thermal and mechanical properties of

Co-Cr-Mo-C-(B) glass-forming alloys

SO INTERMETALLICS

AB In this work, we investigated the effect of boron addition on glass-forming ability and mechanical properties of Co-Cr-Mo-C alloys. The starting alloy was (Co0.65Cr0.13Mo0.22)(80)C-20 derived from the Co65Cr13Mo22 ternary eutectic composition. This alloy is almost fully crystalline and exhibits brittle mechanical properties. Replacing carbon with boron allows obtaining bulk metallic glasses (BMGs) and bulk metallic glass composites. The designed alloys show very high strength (similar to 4100 MPa), wide super-cooled liquid region (similar to 100 K) and large endothermic enthalpy of the supercooled liquid region (similar to 35 J/g), indicating high thermal stability. The electronegativity difference and supercooled liquid region of the present alloys are comparable to rare-earth elements containing BMGs. The physical properties of the present alloys corresponded well with the alloy design strategy based on the unified parameter using the glass transition temperature, fracture strength, and molar volume. Furthermore, we propose the optimum compositional condition for glass formation by controlling the carbon-to-boron ratio and the influence of carbon on phase formation in this alloy system is discussed.

SN 0966-9795

EI 1879-0216

PD AUG

PY 2018

VL 99

BP 1

EP 7

DI 10.1016/j.intermet.2018.05.006

UT WOS:000436221800001

ER

PT J

AU Sun, YL

Sun, YJ

Li, HM

AF Sun, Yong Li

Sun, Ya Juan

Li, Hua Ming

TI Cluster deviation degree in Lennard-Jones glass-forming liquid

SO JOURNAL OF NON-CRYSTALLINE SOLIDS

AB It remains unclear how to describe the cluster packing pattern quantitatively in glass-forming liquid. Here the atomic structures of Lennard-Jones glass-forming liquid obtained by molecular dynamics simulations are investigated. Cluster deviation degree is proposed to quantitatively descript the disordered degree of cluster distribution. The results show that the cluster mean deviation degree follows a power-law behavior with temperature. The size of the central atom has an impact on the cluster deviation degree. The proposed concept provides a statistical approach to describe the cluster evolution during the glass-forming process and the structural distinction of metallic glasses. (C) 2016 Elsevier B.V. All rights reserved.

SN 0022-3093

EI 1873-4812

PD JAN 1

PY 2017

VL 455

BP 59

EP 61

DI 10.1016/j.jnoncrysol.2016.10.029

UT WOS:000390074200009

ER

PT J

AU Guo, J

Zu, FQ

Chen, ZH

Li, XF

Xi, Y

Shen, RR

Zhang, Y

AF Guo, Jing

Zu, Fang-Qiu

Chen, Zhi-Hao

Li, Xian-Fen

Xi, Yun

Shen, Rong-Rong

Zhang, Yan

TI Attempt to depict glass forming ability of bulk metallic glasses using

the criterion of the total relaxation time at the glass transition

SO JOURNAL OF NON-CRYSTALLINE SOLIDS

AB The relationship between the total relaxation time at the glass transition tau(tot)(Tg) and Glass Forming Ability (GFA) of Bulk Metallic Glasses (BMG) has been discussed. Subsequently tau(tot)(Tg) is applied to estimate the GFA of Zr52.5Al10Ni14.6Cu17.9Ti5 and Zr57Al10Ni12.6-Cu15.4Nb5 BMGs. The result indicates that the GFA of the former is greater than that of the latter, which is also demonstrated by the widely accepted criteria of both supercooled liquid region Delta T-x and reduced glass transition temperature T-rg. That testifies the relaxation time of glass transition (tau(tot)(Tg)) is feasible for depicting GFA of BMGs. As a criterion estimating GFA, tau(tot)(Tg) could be easily obtained in practice, and then the occurrence of this criterion will promote exploring new bulk glassy composition. In addition, when tau(tot)(Tg) and Delta T-x are calculated, the characteristic temperatures are determined by a electrical resistivity method besides DSC method. The values obtained from electrical resistivity method agree well with those from DSC method, which proves that the electrical resistivity method is applicable for determining the characteristic temperatures of BMGs. (c) 2006 Elsevier B.V. All rights reserved.

RI Zu, Fang-Qiu/E-8394-2010

OI Zu, Fang-Qiu/0000-0003-4952-6225

SN 0022-3093

PD OCT 1

PY 2006

VL 352

IS 36-37

BP 3859

EP 3863

DI 10.1016/j.jnoncrysol.2006.06.035

UT WOS:000241098800016

ER

PT J

AU Kelton, KF

AF Kelton, KF

TI A new model for nucleation in bulk metallic glasses

SO PHILOSOPHICAL MAGAZINE LETTERS

AB The nanostructured microstructures frequently obtained on crystallization of recently discovered easy metallic glass formers are generally believed to indicate a high nucleation rate and a slow growth velocity. This has been taken to signal either phase separation on an extremely fine scale in the undercooled liquid or glass prior to devitrification, or an extremely high density of impurity sites, favouring heterogeneous nucleation. Here, a model for homogeneous nucleation, which takes account of the linked fluxes of interface attachment and diffusion in the glass to the cluster neighbourhood, is proposed to explain these observations.

SN 0950-0839

PD JUN

PY 1998

VL 77

IS 6

BP 337

EP 343

DI 10.1080/095008398178318

UT WOS:000073888600005

ER

PT J

AU Zhang, T

Men, H

Pang, SJ

Fu, JY

Ma, CL

Inoue, A

AF Zhang, T.

Men, H.

Pang, S. J.

Fu, J. Y.

Ma, C. L.

Inoue, A.

TI Effects of a minor addition of Si and/or Sn on formation and mechanical

properties of Cu-Zr-Ti bulk metallic glass

SO MATERIALS SCIENCE AND ENGINEERING A-STRUCTURAL MATERIALS PROPERTIES

MICROSTRUCTURE AND PROCESSING

CT 12th International Conference on Rapidly Quenched and Metastable

Materials

CY AUG 21-26, 2005

CL Cheju Isl, SOUTH KOREA

AB Effects of the addition of a small amount of Sn and/or Si on glass-forming ability and mechanical properties of Cu5OZr42.Ti-5(7.5) (at.%) bulk metallic glass were studied. The critical diameter for glass formation is 5 mm for Cu50Zr42.5Ti7.5 alloy, and increases to 6 mm for (Cu0.5Zr0.425Ti0.075)(99)Sn-1 and (Cu0.5Zr0.425Ti0.075)(99)Si-1 alloys and 7mm for (Cu0.5Zr0.425Ti0.075)(98.8)Sn0.6Si0.6. In uniaxial compression, (Cu0.5Zr0.425Ti0.075)(99)Sn-1 and (Cu0.5Zr0.425Ti0.075)(98.8)Sn0.6Si0.6 bulk metallic glasses exhibit large plasticity of 8% and 5.3%, respectively, while the limited plasticity of 0.5% and 0.6% is obtained for Cu50Zr42.5Ti7.5 and (Cr0.5Zr0.425Ti0.075)(99)Si-1 bulk metallic glasses. (c) 2006 Elsevier B.V. All rights reserved.

RI Pang, Shujie/D-8305-2016; Inoue, Akihisa/E-5271-2015; Zhang,

Tao/O-4911-2014

SN 0921-5093

PD MAR 25

PY 2007

VL 449

BP 295

EP 298

DI 10.1016/j.msea.2005.12.102

UT WOS:000245477800064

ER

PT J

AU Xiao, YH

Wu, Y

Liu, ZY

Wu, HH

Lu, ZP

AF Xiao YueHua

Wu Yuan

Liu ZhiYuan

Wu HongHui

Lue ZhaoPing

TI Effects of cooling rates on the mechanical properties of a Ti-based bulk

metallic glass

SO SCIENCE CHINA-PHYSICS MECHANICS & ASTRONOMY

AB Mechanical properties of the glassy specimens fabricated at different cooling rates with a composition of Ti40Zr25Cu12Ni3Be20 were systematically investigated. It was confirmed that faster cooling rates caused not only a larger amount of frozen-in free volume but also a higher glass transition temperature in the bulk glassy alloy. Increase in the free volume was found to favor plastic deformation and then to give rise to larger compressive plasticity, whilst the rise in the glass transition temperature seemed to be closely related to the higher yield strength. Moreover, the increase of yield strength and plasticity induced by fast cooling rates may also be associated with the residual stress generated during the fabrication process. Our results suggest that the deformation behavior of bulk metallic glasses is sensitive to various factors and influences from the other factors should be excluded as far as cooling-rate effects on bulk metallic glasses are considered.

RI Lu, Zhao-Ping/A-2718-2009; Wu, Hong-Hui/M-2215-2013; Wu,

Yuan/C-4025-2015; wu, yuan/E-8927-2010

OI Lu, Zhao-Ping/0000-0003-1463-8948; Wu, Hong-Hui/0000-0002-1381-2281; Wu,

Yuan/0000-0001-7857-0247;

SN 1674-7348

EI 1869-1927

PD MAR

PY 2010

VL 53

IS 3

SI SI

BP 394

EP 398

DI 10.1007/s11433-010-0136-8

UT WOS:000276661700003

ER

PT J

AU Sun, LL

Kikegawa, T

Wang, WK

AF Sun, LL

Kikegawa, T

Wang, WK

TI Transitions of amorphous-crystalline-amorphous in bulk metallic glass

under HP and HT

SO CHINESE SCIENCE BULLETIN

AB In-situ SR-XRD measurements revealed that the crystallization process in Zr(41.2)Ti(13.8)Cu(12.5)Ni(10)Be(22.5) bulk metallic glass Is significantly different from that in traditional glasses. Subsequent heating at 10 GPa converts the sample from amorphous phase into the metastable fcc phase and then leads to the fcc phase back to the amorphous phase, indicating that there exists 'reversible' phase transition phenomena in the material under high pressure and high temperature.

SN 1001-6538

PD JAN

PY 2002

VL 47

IS 2

BP 100

EP 101

UT WOS:000174261900003

ER

PT J

AU Chen, J

Zhang, Y

He, JP

Yao, KF

Wei, BC

Chen, GL

AF Chen, J

Zhang, Y

He, JP

Yao, KF

Wei, BC

Chen, GL

TI Metallographic analysis of Cu-Zr-Al bulk amorphous alloys with yttrium

addition

SO SCRIPTA MATERIALIA

AB Minor yttrium addition can improve the glass-forming ability of Cu-Zr-Al ternary alloys via suppression of the growth of eutectic clusters. Yttrium addition also makes the room temperature ductility of the alloys decrease, and both the compressive strength and elastic strain limits increase slightly. (c) 2006 Acta Materialia Inc. Published by Elsevier Ltd. All rights reserved.

RI ZHANG, Yong/B-7928-2009

OI ZHANG, Yong/0000-0002-6355-9923

SN 1359-6462

PD APR

PY 2006

VL 54

IS 7

BP 1351

EP 1355

DI 10.1016/j.scriptamat.2005.12.002

UT WOS:000235256200025

ER

PT J

AU Liu, YH

Wang, G

Pan, MX

Yu, P

Zhao, DQ

Wang, WH

AF Liu, Y. H.

Wang, G.

Pan, M. X.

Yu, P.

Zhao, D. Q.

Wang, W. H.

TI Deformation behaviors and mechanism of Ni-Co-Nb-Ta bulk metallic glasses

with high strength and plasticity

SO JOURNAL OF MATERIALS RESEARCH

AB A class of Ni-Co-Nb-Ta bulk metallic glasses (BMGs) with a high glass-forming ability is developed. With proper compositional modification, the BMGs exhibit the enhanced plastic strain (up to 4%) and the ultimate strength (up to 3540 MPa). It is found that the interactions of shear bands such as intersecting, arresting, and branching, which normally are related to the plastic metallic glasses, can be observed both in the plastic and brittle Ni-Co-Nb-Ta BMGs. Obvious serrated flow behavior is observed during plastic deformation. The origins of the plasticity and the serrated flow in the Ni-based BMGs are analyzed in analogy to that in crystalline materials.

RI LIU, Yanhui/B-1485-2009; Wang, Gang/K-2630-2012

SN 0884-2914

PD APR

PY 2007

VL 22

IS 4

BP 869

EP 875

DI 10.1557/JMR.2007.0104

UT WOS:000245489400008

ER

PT S

AU Seidel, M

Eckert, J

Bauer, HD

Schultz, L

AF Seidel, M

Eckert, J

Bauer, HD

Schultz, L

BE Schulz, R

TI Mechanical alloyed Zr-based metallic glasses with significant

supercooled liquid region

SO METASTABLE, MECHANICALLY ALLOYED AND NANOCRYSTALLINE MATERIALS, PTS 1

AND 2

SE MATERIALS SCIENCE FORUM

CT International Symposium on Metastable, Mechanically Alloyed and

Nanocrystalline Materials (ISMANAM-95)

CY JUL 24-28, 1995

CL QUEBEC CITY, CANADA

SP Hydro Quebec, Laval Univ, McGill Univ, Ctr Phys Mat, Nat Sci & Engn Res Council Canada, Zoz GmbH, Siemens, Xerox Canada Ltee, Precitech

AB Zr-Al-Cu-Ni and Zr-Ti-Cu-Ni amorphous alloys exhibiting a significant supercooled Liquid region have been prepared by mechanical alloying of elemental powders. The alloying process upon milling was followed by x-ray diffraction, differential scanning calorimetry (DSC), optical and scanning electron microscopy (SEM) and transmission electron microscopy (TEM). The thermal stability of the materials was investigated by constant-rate heating DSC and isothermal annealing experiments. Finally, bulk amorphous samples were produced by consolidating the as-milled powders at temperatures above the glass transition and first data on the mechanical properties of consolidated samples are given.

RI Schultz, Ludwig/B-3383-2010

SN 0255-5476

BN 0-87849-738-2

PY 1996

VL 225

BP 119

EP 124

DI 10.4028/www.scientific.net/MSF.225-227.119

UT WOS:A1996BG37Y00017

ER

PT J

AU Qiu, SB

Yao, KF

AF Qiu, Sheng-Bao

Yao, Ke-Fu

TI Novel application of the electrodeposition on bulk metallic glasses

SO APPLIED SURFACE SCIENCE

AB The electrodeposition technique was employed to prepare fine-grained copper coatings on the substrate of the Zr(41)Ti(14)Cu(12.5)Ni(10)Be(22.5) bulk metallic glass (BMG). It is found that Zr-based BMGs with copper coatings exhibited larger plastic strains than those without coatings, at the same strain rate under the uniaxial compression. Contrary to the uncoated metallic glass, the existence of copper coatings inhibited the rapid propagation of primary shear bands, and promoted the generation of shear bands, which led to that the coated samples exhibited large plastic strain eventually. It reveals that the electrodeposition technique is a promising approach to improve the plasticity of 'traditionally brittle' BMGs. (C) 2008 Elsevier B. V. All rights reserved.

SN 0169-4332

PD DEC 30

PY 2008

VL 255

IS 5

BP 3454

EP 3458

DI 10.1016/j.apsusc.2008.07.077

UT WOS:000261299200151

ER

PT J

AU Yu, GS

Ma, M

Lin, JG

AF Yu Gengsheng

Ma Mu

Lin Jianguo

TI Effect of Pressure Sensitivity Index on Shear Band and Fracture Angle

SO RARE METAL MATERIALS AND ENGINEERING

AB The included angles (2 theta) and the fracture angles (theta) of the Zr41.2Ti13.8Cu12.5Ni10Be22.5 bulk metallic glass which was as-cast and annealed respectively were studied by the Rockwell indentation and MTS. After heating, the included angle 2 theta is in the range of 88 degrees to 79 degrees, while the fracture angle theta is 40 degrees similar to 44 degrees under compressive deformation, which is half of the included angle. By Mohr-Coulomb criterion, the pressure sensitive index can be obtained on the basis of the measured 2 theta, which increases with increasing annealing temperature. The effect of the normal stress on the fracture plane should be quite remarkable and can change the critical shear fracture condition of metallic glasses.

SN 1002-185X

PD OCT

PY 2008

VL 37

IS 10

BP 1733

EP 1736

UT WOS:000260751700010

ER

PT J

AU Jiang, JZ

Saksl, K

AF Jiang, JZ

Saksl, K

TI Structural stability of Pd40CU30Ni10P20 metallic glass in supercooled

liquid region

SO MATERIALS SCIENCE AND ENGINEERING A-STRUCTURAL MATERIALS PROPERTIES

MICROSTRUCTURE AND PROCESSING

CT 11th International Conference on Rapidly Quenched and Metastable

Materials

CY AUG 25-30, 2002

CL Univ Oxford, Dept Mat, Oxford, ENGLAND

HO Univ Oxford, Dept Mat

AB Phase separation of bulk and ribbon Pd40Cu30Ni10P20 glasses, annealed in the supercooled liquid region at ambient pressure and high pressures, has been studied by means of differential scanning calorimetry (DSC) and X-ray diffraction techniques. DSC measurements show only one glass transition event in all annealed samples, indicating that no phase separation occurs in the alloy annealed in the supercooled liquid region. Phase analyses reveal at least six crystalline phases in the crystallized sample: monoclinic, tetragonal Cu3Pd-like, rhombohedral, fcc-Ni2Pd2P, fcc-(Ni, Pd) solid solution, and body-centered tetragonal (bct) Ni3P-like phases. Annealing treatments under external pressures in the vicinity of the glass transition temperature neither induce phase separation nor alter the glass transition temperature of the Pd40Cu30Ni10P20 bulk glass. (C) 2003 Published by Elsevier B.V.

SN 0921-5093

PD JUL 15

PY 2004

VL 375

SI SI

BP 733

EP 737

DI 10.1016/j.msea.2003.10.075

UT WOS:000223329700138

ER

PT J

AU Raghavan, R

Shastry, VV

Kumar, A

Jayakumar, T

Ramamurty, U

AF Raghavan, R.

Shastry, V. V.

Kumar, A.

Jayakumar, T.

Ramamurty, U.

TI Toughness of as-cast and partially crystallized composites of a bulk

metallic glass

SO INTERMETALLICS

AB The deformation and fracture response of a bulk metallic glass (BMG) post-annealing above the glass transition temperature is examined. The toughness of the glass-matrix composite exhibits a sharp transition beyond a critical volume fraction of crystallization to values as low as that of brittle silicate glass. Instrumented indentation tests supplemented by impact tests were used to study this ductile to brittle transition exhibited by the partially crystallized samples. Indentation on the anneal-embrittled specimens shows lateral cracks in addition to cracks along the corners. The applicability of the Poisson's ratio-toughness correlation with respect to partially crystallized samples is also investigated. (C) 2009 Elsevier Ltd. All rights reserved.

RI Raghavan, Rejin/A-2177-2015; Ramamurty, Upadrasta/E-5623-2011; Kumar,

Anish/A-3544-2016

OI Raghavan, Rejin/0000-0002-7108-7081; Kumar, Anish/0000-0002-8219-1095

SN 0966-9795

PD OCT

PY 2009

VL 17

IS 10

BP 835

EP 839

DI 10.1016/j.intermet.2009.03.012

UT WOS:000267974600010

ER

PT J

AU Ma, H

Ma, E

Xu, J

AF Ma, H

Ma, E

Xu, J

TI A new Mg65Cu7.5Ni7.5Zn5Ag5Y10 bulk metallic glass with strong

glass-forming ability

SO JOURNAL OF MATERIALS RESEARCH

AB We report a new Mg-based bulk metallic glass-forming alloy: Mg65Cu7.5Ni7.5Zn5 Ag5Y10. The alloy exhibits a glass-forming ability significantly stronger than all previously discovered Mg-based glass formers. Fully glassy rods 9 mm in diameter can be obtained by using copper mold casting. The critical cooling rate for glass formation was estimated to be <50 Ks(-1). The reduced glass-transition temperature (T-rg) of the glass was determined to be 0.59.

RI Ma, En/A-3232-2010

SN 0884-2914

PD OCT

PY 2003

VL 18

IS 10

BP 2288

EP 2291

DI 10.1557/JMR.2003.0319

UT WOS:000185821000003

ER

PT J

AU Wen, P

Zhao, ZF

Pan, MX

Wang, WH

AF Wen, Ping

Zhao, Zuo Feng

Pan, Ming Xiang

Wang, Wei Hua

TI Mechanical relaxation in supercooled liquids of bulk metallic glasses

SO PHYSICA STATUS SOLIDI A-APPLICATIONS AND MATERIALS SCIENCE

AB We report the mechanical relaxation behaviors in typical supercooled liquids of bulk metallic glasses (BMGs). The metallic supercooled liquids are ideal systems for studying intrinsic motions of glass-former supercooled liquids because their structure is close to the simple "dense random packing of spheres'' model. We show that the primary relaxation in the frequency domain is dissipative and can be described by the empirical Kohlrausch-Williams-Watts function, and the temperature dependence of the primary relaxation time has the Vogel-Fulcher-Tamman form. Beyond the primary relaxation, an excess wing is found on the high-frequency tail of the primary relaxation. A corresponding shoulder is exhibited at a given frequency in the temperature region below the glass-transition temperature. The experimental results confirm that the decoupling between the slow beta relaxation and the primary (a) relaxation exists in metallic supercooled liquids. Based on the current models of the glass transition, we demonstrate that the dynamically heterogeneity originates in the heterogeneous microstructure of metallic supercooled liquids, and a picture of the heterogeneous microstructure is provided. (C) 2010 WILEY-VCH Verlag GmbH & Co. KGaA, Weinheim

RI Zhao, Zuofeng/B-1297-2010

OI Zhao, Zuofeng/0000-0002-0862-8471

SN 1862-6300

PD DEC

PY 2010

VL 207

IS 12

BP 2693

EP 2703

DI 10.1002/pssa.201026475

UT WOS:000285848500007

ER

PT J

AU Mitrofanov, YP

Khonik, SV

Lyakhov, SA

Khoviv, AM

Khonik, VA

AF Mitrofanov, Yu. P.

Khonik, S. V.

Lyakhov, S. A.

Khoviv, A. M.

Khonik, V. A.

TI Recovery of the shear modulus of relaxed bulk glassy Pd40Cu30Ni10P20 by

cooling from elevated temperatures at low rates

SO INTERMETALLICS

AB We found that the room-temperature shear modulus of a relaxed bulk metallic glass decreases with the cooling rate T upon cooling from elevated temperatures even though (T) over dot is only a few K/min. The effect is observed as a result of cooling not only from the supercooled liquid state but also from a wide range of temperatures below the glass transition. A possible reason for the effect is discussed. (C) 2010 Elsevier Ltd. All rights reserved.

RI Mitrofanov, Yuriy/E-7963-2010; Khonik, Vitaly/A-5888-2009

OI Mitrofanov, Yuriy/0000-0002-7939-5230;

SN 0966-9795

PD MAR

PY 2011

VL 19

IS 3

BP 419

EP 422

DI 10.1016/j.intermet.2010.11.012

UT WOS:000286956400024

ER

PT J

AU Qiang, JB

Zhang, W

Inoue, A

AF Qiang, J. B.

Zhang, W.

Inoue, A.

TI Ni-(Zr/Hf)-(Nb/Ta)-Al bulk metallic glasses with high thermal

stabilities

SO INTERMETALLICS

CT 6th International Conference on Bulk-Metallic Glasses (BMG-VI)

CY MAY 11-15, 2008

CL Xian, PEOPLES R CHINA

AB Thermal stability is a critical consideration in the application of metallic glasses as hydrogen separation material. The development of new Ni-based bulk metallic glasses (BMGs) with enhanced thermal stability is desirable. The present work investigated the alloying effects of refractory metals Hf and Ta on the Ni(60)Zr(20)Nb(15)Al(5) bulk metallic glass. Two serial alloys, namely, Ni(60)Zr(20-x)Hf(x)Nb(15)Al(5) (x = 0 similar to 20 at.%) and Ni(60)Zr(20)Ta(y)Nb(15-y)Al(5) (y = 0 similar to 15 at.%), were investigated in the present work. The addition of Hf or Ta was revealed to be effective in improving the thermal stability of the basic alloy, while the glass-forming ability of the alloy is slightly reduced resulting from the addition of Hf or Ta. The possible mechanism of these alloying effects is discussed. (C) 2008 Elsevier Ltd. All rights reserved.

RI Inoue, Akihisa/E-5271-2015

SN 0966-9795

PD APR

PY 2009

VL 17

IS 4

BP 249

EP 252

DI 10.1016/j.intermet.2008.11.008

UT WOS:000264728400015

ER

PT J

AU Kundig, AA

Ohnuma, M

Ohkubo, T

Abe, T

Hono, K

AF Kundig, A. A.

Ohnuma, M.

Ohkubo, T.

Abe, T.

Hono, K.

TI Glass formation and phase separation in the Ag-Cu-Zr system

SO SCRIPTA MATERIALIA

AB Glass formation and phase separation in Ag-Cu-Zr alloys have been investigated by energy-filtered transmission electron microscopy to discuss the possibility of phase separation in bulk metallic glasses. The experimentally determined glass forming composition range overlaps marginally with the calculated miscibility gap. As-quenched amorphous Ag20Cu48Zr32 alloy shows a composition contrast in a fully amorphous structure. Subsequently crystallized microstructure shows a good size correlation with the prior compositional fluctuations, indicating preferred nucleation in one of the phase-separated regions. (c) 2006 Acta Materialia Inc. Published by Elsevier Ltd. All rights reserved.

RI Hono, Kazuhiro/B-9202-2008; abe, taichi/H-2999-2011

OI Hono, Kazuhiro/0000-0001-7367-0193; abe, taichi/0000-0002-5065-0939

SN 1359-6462

PD SEP

PY 2006

VL 55

IS 5

BP 449

EP 452

DI 10.1016/j.scriptamat.2006.05.012

UT WOS:000239132200008

ER

PT J

AU Choi-Yim, H

Xu, DH

Lind, ML

Loffler, JF

Johnson, WL

AF Choi-Yim, H

Xu, DH

Lind, ML

Loffler, JF

Johnson, WL

TI Structure and mechanical properties of bulk glass-forming Ni-Nb-Sn

alloys

SO SCRIPTA MATERIALIA

AB Ternary alloys of composition Ni60Nb40-xSnx, with 3 <= x < 9, were found to form bulk metallic glasses. These alloys exhibit a high elastic modulus and measured compressive yield strength between 1.8 and 2.8 GPa. The measured yield strength of these ternary alloys depends strongly on their Sn content and is generally lower than the strength estimated from modulus and hardness. The tendency to phase separation suggested by small-angle neutron scattering experiments is used to account for the obvious discrepancy between the measured and the theoretical strengths. (c) 2005 Acta Materialia Inc. Published by Elsevier Ltd. All rights reserved.

RI Xu, Donghua/A-5263-2008

OI Lind, Mary/0000-0001-8585-8054; Xu, Donghua/0000-0001-5018-5603

SN 1359-6462

PD JAN

PY 2006

VL 54

IS 2

BP 187

EP 190

DI 10.1016/j.scriptamat.2005.09.040

UT WOS:000233226000012

ER

PT J

AU Kawamura, Y

AF Kawamura, Y

TI Liquid phase and supercooled liquid phase welding of bulk metallic

glasses

SO MATERIALS SCIENCE AND ENGINEERING A-STRUCTURAL MATERIALS PROPERTIES

MICROSTRUCTURE AND PROCESSING

CT 11th International Conference on Rapidly Quenched and Metastable

Materials

CY AUG 25-30, 2002

CL Univ Oxford, Dept Mat, Oxford, ENGLAND

HO Univ Oxford, Dept Mat

AB Recent progress on welding in bulk metallic glasses (BMGs) has been reviewed. BMGs have been successfully welded to BMGs or crystalline metals by liquid phase welding using explosion, pulse-current and electron-beam methods, and by supercooled liquid phase welding using friction method. Successful welding of the liquid phase methods was due to the high glass-forming ability of the BMGs and the high concentration of welding energy in these methods. In contrast, the supercooled liquid phase welding was successful due to the thermally stable supercooled liquid state of the BMGs and the superplasticity and viscous flow of the supercooled liquid. The successful welding of BMGs to BMGs and crystalline materials is promising for the future development of BMGs as engineering materials. (C) 2003 Elsevier B.V. All rights reserved.

SN 0921-5093

PD JUL 15

PY 2004

VL 375

SI SI

BP 112

EP 119

DI 10.1016/j.msea.2003.10.097

UT WOS:000223329700017

ER

PT J

AU Yokota, Y

Aniya, M

AF Yokota, Yuko

Aniya, Masaru

TI Evaluation of the elastic properties of bulk metallic glasses

SO JOURNAL OF THERMAL ANALYSIS AND CALORIMETRY

CT 4th International Symposium on New Frontiers of Thermal Studies of

Materials

CY NOV 30-DEC 02, 2008

CL Yokohama, JAPAN

SP JSCTA, SPCTAJ

AB The development of bulk metallic glasses as a prominent class of functional and structural materials has attracted considerable interest in the last years. One of the fundamental physical quantities necessary to describe the mechanical properties of the materials is the bulk modulus. In the present article, a simple method to estimate the bulk modulus and its pressure derivative is proposed. It is shown that these quantities can be estimated from the values of the constituent elements and their compositions. Comparison with measured data shows good agreement. The physical background of the method is discussed based on the jellium model of metals.

SN 1388-6150

PD JAN

PY 2010

VL 99

IS 1

BP 105

EP 108

DI 10.1007/s10973-009-0484-7

UT WOS:000274213300017

ER

PT J

AU Maddala, DR

Hebert, RJ

AF Maddala, Dharma R.

Hebert, Rainer J.

TI Sliding wear behavior of Fe50-xCr15Mo14C15B6Erx (x=0,-1, 2 at%) bulk

metallic glass

SO WEAR

AB The sliding wear behavior was investigated for Fe50-xCr15Mo14C15B6Erx (x=0, 1, 2 at%) bulk metallic glasses (BMG). Minor alloying with 1-2 at% erbium resulted in an increase in hardness and a deterioration of the indentation fracture toughness, but the wear resistance increased with increasing Er content. The Fe48Cr15Mo14C15B6Er2 bulk metallic glass was then annealed and the wear resistance increased in the following order: crystallized, as-cast, and structurally relaxed condition. Transmission electron microscopy studies did not reveal sliding wear-induced crystallization, but instead shear band formation. The results demonstrate that micro-alloying is a useful approach for improving the wear resistance of bulk metallic glasses and that the combination of micro-alloying and annealing can substantially improve the wear resistance. (C) 2012 Elsevier B.V. All rights reserved.

SN 0043-1648

EI 1873-2577

PD JUL 30

PY 2012

VL 294

BP 246

EP 256

DI 10.1016/j.wear.2012.06.007

UT WOS:000311003200002

ER

PT J

AU Yiu, P

Jang, JSC

Chang, SY

Chen, YC

Chu, JP

Hsueh, CH

AF Yiu, P.

Jang, J. S. C.

Chang, S. Y.

Chen, Y. C.

Chu, J. P.

Hsueh, C. H.

TI Plasticity enhancement of Zr-based bulk metallic glasses by direct

current electropulsing

SO JOURNAL OF ALLOYS AND COMPOUNDS

AB Direct current electropulsing was used to improve the plasticity of (Zr53Cu30Ni9Al8)(99.5)Si-0.5 bulk metallic glasses. After the electropulsing treatment, the specimen showed reductions in both the glass transition and the crystallization temperatures while retaining its amorphous structure, and both Young's modulus and the hardness decreased while the nanoindentation loading curve became more serrated. Using the bond-interface method and Vickers indentation, the treated specimen showed more branching of semicircular shear bands and less radial shear bands compared to its as-cast counterpart. The possible plasticity enhancement mechanism of the electropulsing treatment was also discussed. (C) 2012 Elsevier B. V. All rights reserved.

SN 0925-8388

PD JUN 5

PY 2012

VL 525

BP 68

EP 72

DI 10.1016/j.jallcom.2012.02.074

UT WOS:000302574800013

ER

PT J

AU Xu, X

Chen, LY

Zhang, GQ

Wang, N

Jiang, JZ

AF Xu, X.

Chen, L. Y.

Zhang, G. Q.

Wang, L. N.

Jiang, J. Z.

TI Formation of bulk metallic glasses in Cu45Zr48-xAl7REx (RE = La, Ce, Nd,

Gd and 0 <= x <= 5 at.%)

SO INTERMETALLICS

AB We report a series of bulk metallic glass-forming alloys of compositions (Cu45Zr48-xAl7REx, RE = La, Ce, Nd, Gd and 0 <= x <= 5 at.%). By using a conventional copper mold sucking method, alloys with diameters ranging from 5 to 10 mm can be readily solidified into an amorphous structure without detectable crystallites. The best glass-forming ability is obtained for the alloys Cu45Zr46Al7RE2. Possible effects of RE addition on the glass-forming ability are discussed. In addition, the compositional effect on mechanical properties of Zr-Cu-Al-Gd alloys is presented. (C) 2007 Elsevier Ltd. All rights reserved.

RI Chen, Lianyi/B-3156-2008

OI Chen, Lianyi/0000-0003-3720-398X

SN 0966-9795

PD AUG

PY 2007

VL 15

IS 8

BP 1066

EP 1070

DI 10.1016/j.intermet.2006.12.010

UT WOS:000248065300011

ER

PT J

AU Okai, D

Inoue, M

Mori, T

Fukami, T

Yamasaki, T

Kimura, HM

Inoue, A

AF Okai, D.

Inoue, M.

Mori, T.

Fukami, T.

Yamasaki, T.

Kimura, H. M.

Inoue, A.

TI Annealing effect on mechanical constants for Ca48Mg27Cu25 bulk metallic

glass

SO JOURNAL OF ALLOYS AND COMPOUNDS

CT 16th International Symposium on Metastable, Amorphous and Nanostructured

Materials

CY JUL 05-09, 2009

CL Beijing, PEOPLES R CHINA

AB An annealing effect on elastic moduli of a Ca48Mg27Cu25 bulk metallic glass has been investigated. The elastic moduli of Ca48Mg27Cu25 bulk metallic glass were estimated using a technique of ultrasonic velocity measurements. The Young's modulus (E), Poisson's ratio (v), shear modulus (G) and bulk modulus (B) for the as-cast Ca48Mg27Cu25 alloy at room temperature were found out to be sensitive to the change of amorphous structure for the alloy. The crystallization of Ca48Mg27Cu25 bulk metallic glass led to the increases of E, G and B, and the decrease of v for the alloy. (C) 2010 Elsevier B.V. All rights reserved.

RI Kimura, Hisamichi/D-5449-2012; Inoue, Akihisa/E-5271-2015

SN 0925-8388

EI 1873-4669

PD AUG

PY 2010

VL 504

SU 1

BP S95

EP S97

DI 10.1016/j.jallcom.2010.03.103

UT WOS:000285252600025

ER

PT J

AU Du, XH

Huang, JC

Liu, CT

Lu, ZP

AF Du, X. H.

Huang, J. C.

Liu, C. T.

Lu, Z. P.

TI New criterion of glass forming ability for bulk metallic glasses

SO JOURNAL OF APPLIED PHYSICS

AB It has been confirmed that glass-forming ability (GFA) is related to not only liquid phase stability but also the crystallization resistance. In this study, it was found the liquidus temperature T(l) and supercooled liquid region T(x)-T(g) could reflect the stability of glass-forming liquids at the equilibrium and undercooled state, respectively, while the onset crystallization temperature T(x) could indicate the crystallization resistance during glass formation. Thus, a modified gamma parameter, defined as gamma(m)=(2T(x)-T(g))/T(l), has been established. This parameter shows an excellent correlation with the GFA of bulk metallic glasses, with the statistical correlation factor of R(2)=0.931. (c) 2007 American Institute of Physics.

RI Lu, Zhao-Ping/A-2718-2009; Huang, J./C-4276-2013

OI Lu, Zhao-Ping/0000-0003-1463-8948; HUANG, Jacob Chih

Ching/0000-0001-6843-3396; Liu, Chain Tsuan/0000-0001-7888-9725

SN 0021-8979

PD APR 15

PY 2007

VL 101

IS 8

AR 086108

DI 10.1063/1.2718286

UT WOS:000246072200176

ER

PT J

AU Kumar, G

Prades-Rodel, S

Blatter, A

Schroers, J

AF Kumar, Golden

Prades-Rodel, Silke

Blatter, Andreas

Schroers, Jan

TI Unusual brittle behavior of Pd-based bulk metallic glass

SO SCRIPTA MATERIALIA

AB The bending plasticity of Pd-based and Pt-based bulk metallic glasses (BMGs) is compared. Low cooling rates obtained during bulk casting render Pd-BMG (Pd(43)Cu(27)Ni(10)P(20)) brittle, whereas Pt-BMG (Pt(57.5)Cu(14.7)Ni(5.3)P(22.5)) retains high plasticity at all cooling rates. The ratio of the shear modulus (G) to the bulk modulus (B) in the two alloys is similar (similar to 0.2). A previously proposed critical G/B value of 0.41 for tough to brittle transition is not supported by our findings, which rather suggest an alloy specific critical G/B. (C) 2011 Acta Materialia Inc. Published by Elsevier Ltd. All rights reserved.

RI kumar, golden/F-5443-2010

SN 1359-6462

PD OCT

PY 2011

VL 65

IS 7

BP 585

EP 587

DI 10.1016/j.scriptamat.2011.06.029

UT WOS:000294882500006

ER

PT J

AU Mattern, N

AF Mattern, N.

TI Structure formation in liquid and amorphous metallic alloys

SO JOURNAL OF NON-CRYSTALLINE SOLIDS

CT 10th International Conference on the Structure of Non-Crystalline

Materials (NCM 10)

CY SEP 18-22, 2006

CL Prague, CZECH REPUBLIC

AB Bulk metallic glasses developed in last 15 years represent a new class of amorphous metallic alloys. These multi-component metallic alloys can be obtained at relatively low cooling rates, which allow the production of large-scale materials by conventional casting processes. Furthermore, bulk metallic glasses show a glass transition well below the crystallization temperature enabling hot deformation, but also to investigate the glass transition phenomenon in a metallic system. The thermal behavior of Zr- and Pd-based bulk metallic glasses was studied by in situ X-ray diffraction at elevated temperatures. The temperature dependence of the X-ray structure factor of the glassy state can be well described by the Debye theory. At the caloric glass transition the temperature dependence of the structure alters, pointing to a continuous development of structural changes in the liquid state. The short-range order of the glass, of the supercooled liquid, and of the equilibrium melt is found to be very similar. The existence of complex chemically ordered clusters in the melt is supposed to be related to the high glass-forming ability of the alloys. The microstructure of metallic glasses consisting of elements with negative enthalpy of mixing is homogeneous at dimensions above 1 nm. Phase separation in the liquid state appears in metallic systems with large positive enthalpy of mixing of the elements like Nb-Y. Thermodynamic calculations of the Ni-Nb-Y phase diagram show that the miscibility gap of the monotectic binary Nb-Y system extends into the ternary up to large Ni content. Experimental evidence of the phase separation in ternary Ni-Nb-Y melts is obtained by in situ X-ray diffraction at elevated temperatures and differential scanning calorimetry. The phase separated melt can be frozen into a two-phase amorphous metallic alloy by rapid quenching from the liquid. The microstructure depends on the chemical composition and consists of two amorphous regions, one Nb-enriched and the other Y-enriched, with a size distribution from several nanometers up to micrometer dimension. The experimental results confirm the close relationship between the structure of metallic glasses and the corresponding under-cooled liquids. (c) 2007 Elsevier B.V. All rights reserved.

SN 0022-3093

PD JUN 15

PY 2007

VL 353

IS 18-21

BP 1723

EP 1731

DI 10.1016/j.jnoncrysol.2007.01.042

UT WOS:000247330600007

ER

PT J

AU Bakai, SO

AF Bakai, S. O.

TI Impact of Ultrasonic Treatment on Mechanical Properties of Bulk Metallic

Glass Zr52.5Ti5Cu17.9N14.6Al10

SO METALLOFIZIKA I NOVEISHIE TEKHNOLOGII

AB Influence of preliminary ultrasonic treatment on mechanical properties and structure of bulk metallic glass Zr52.5Ti5Cu17.9Ni14.6Al10 is investigated. Investigations are carried out by the method of acoustic emission under uniaxial compression at room temperature. The results of investigations allow understanding of the nature of changes of acoustic emission and metallic-glass strength under the action of ultrasonic treatment.

SN 1024-1809

PD MAR

PY 2011

VL 33

IS 3

BP 383

EP 388

UT WOS:000294586300009

ER

PT J

AU Yu, P

Zhang, NZ

Cui, YT

Wen, L

Zeng, ZY

Xia, L

AF Yu, P.

Zhang, N. Z.

Cui, Y. T.

Wen, L.

Zeng, Z. Y.

Xia, L.

TI Achieving an enhanced magneto-caloric effect by melt spinning a

Gd55Co25Al20 bulk metallic glass into amorphous ribbons

SO JOURNAL OF ALLOYS AND COMPOUNDS

AB The magneto-caloric effect (MCE) and microstructure of the Gd55Co25Al20 metallic glasses were studied in the present work. It was found that the amorphous ribbons exhibit a peak value of magnetic entropy change (-Delta S-m(peak)) up to 39.5% higher than that of the bulk metallic glass (BMG) under a magnetic field of 1 T. The mechanism for the enhanced MCE of amorphous ribbons was investigated and the dependence of magneto-caloric behaviors on the microstructure of the glassy alloys was revealed. The larger -Delta S-m(peak) as well as refrigeration capacity under low magnetic field, together with the higher heat exchange efficiency of the ribbons, indicates that the amorphous ribbons could be more suitable candidates as the magnetic refrigerants rather than the BMG. (C) 2015 Elsevier B.V. All rights reserved.

SN 0925-8388

EI 1873-4669

PD JAN 15

PY 2016

VL 655

BP 353

EP 356

DI 10.1016/j.jallcom.2015.09.205

UT WOS:000364603100047

ER

PT J

AU Cao, WH

Zhang, JL

Shek, CH

AF Cao, W. H.

Zhang, J. L.

Shek, C. H.

TI The oxidation behavior of Cu42Zr42Al8Ag8 bulk metallic glasses

SO JOURNAL OF MATERIALS SCIENCE

AB The oxidation behavior of Cu42Zr42Al8Ag8 bulk metallic glass was studied in synthetic air over the temperature range of 330-460 degrees C. The oxidation kinetics of the metallic glass follows a single parabolic rate law at 330 and 390 degrees C and a two-stage parabolic rate law from 420 to 460 degrees C. Silver precipitates on the topmost oxide layer of the metallic glass were revealed by energy-dispersive X-ray spectroscopy together with X-ray diffraction pattern. Observation using scanning electron microscopy shows that silver metal precipitated at all temperatures and some islands were formed on the outermost copper oxide layer at high temperatures. Multilayered oxide scales were also observed with silver precipitates sandwiched between copper- and zirconium-rich oxides layers.

RI SHEK, Chan Hung/J-3857-2015; zhang, jiliang/N-5659-2015

OI SHEK, Chan Hung/0000-0002-6870-523X;

SN 0022-2461

EI 1573-4803

PD FEB

PY 2013

VL 48

IS 3

BP 1141

EP 1146

DI 10.1007/s10853-012-6851-y

UT WOS:000312904900019

ER

PT J

AU Johnson, WL

AF Johnson, WL

TI Bulk amorphous metal - An emerging engineering material

SO JOM-JOURNAL OF THE MINERALS METALS & MATERIALS SOCIETY

AB During the last two decades, researchers have developed families of metal alloys that exhibit exceptional resistance to crystallization in the undercooled liquid state. Upon cooling, these alloys readily form glass or vitrify to form bulk amorphous alloys or bulk metallic glasses. The stability of the undercooled molten alloys with respect to crystallization has enabled studies of liquid thermodynamics, rheology, atomic diffusion, and the glass transition previously not possible in metallic systems. Bulk amorphous alloys exhibit very high strength, specific strength, and elastic strain limit, along with unusual combinations of other engineering properties. These factors, taken together; suggest that bulk amorphous metals will become widely used engineering materials during the next decade.

SN 1047-4838

PD MAR

PY 2002

VL 54

IS 3

BP 40

EP 43

DI 10.1007/BF02822619

UT WOS:000174334200008

ER

PT J

AU Schmitz, S

Loser, W

Klauss, H

Buchner, B

AF Schmitz, S.

Loeser, W.

Klauss, H.

Buechner, B.

TI Effect of elements with positive enthalpy of mixing on mechanical

properties of bulk metallic glasses

SO JOURNAL OF ALLOYS AND COMPOUNDS

CT 17th International Symposium on Metastable, Amorphous and Nanostructured

Materials (ISMANAM 2010)

CY JUL 04-09, 2010

CL Zurich, SWITZERLAND

AB Samples of (Cu(46)Zr(46)Al(8))(100-x)Z(x) metallic glass forming alloys with diameters 2-6mm were prepared by injection casting. The effect of minor amounts of elements Z = Gd, Co and Re with positive enthalpy of mixing within the Gd-Zr, Cu-Co and Cu-Re terminal systems was compared. The addition of Gd up to x = 2 slightly enhances the glass forming ability, Co reduces the critical diameter of bulk metallic glass formation, whereas even for small fractions of Re bulk samples were crystalline, but only amorphous splats can be prepared. Both Gd and Co diminish the crystallization temperature T(x) with respect to the Cu(46)Zr(46)Al(8) master alloy, but in Re-bearing splats T(x) is increased. Alloying with optimum amounts of Gd and Co up to x = 2 leads to plastic deformability of rods, 2 and 3mm in diameter, in comparison with the brittle Cu(46)Zr(46)Al(8) bulk metallic glass. (C) 2010 Elsevier B. V. All rights reserved.

RI Buchner, Bernd/E-2437-2016

OI Buchner, Bernd/0000-0002-3886-2680

SN 0925-8388

PD JUN

PY 2011

VL 509

SU 1

BP S131

EP S135

DI 10.1016/j.jallcom.2010.12.054

UT WOS:000291463000030

ER

PT J

AU Bian, Z

He, G

Chen, GL

AF Bian, Z

He, G

Chen, GL

TI Investigation of shear bands under compressive testing for Zr-base bulk

metallic glasses containing nanocrystals

SO SCRIPTA MATERIALIA

AB This paper investigated the dependence of shear bands on the volume fraction of nanocrystals (V-crys) under compressive testing. For Zr-base bulk amorphous alloy, the mean size of shear bands is about 0.3 mum. For bulk metallic glasses containing V-crys less than 42%, the mean size of shear bands is less than 0.3 mum. Shear bands become much thinner and denser with the increase of V-crys. Once V-crys is larger than this value, no shear bands can be observed. (C) 2002 Published by Elsevier Science Ltd. on behalf of Acta Materialia Inc.

SN 1359-6462

PD MAR 25

PY 2002

VL 46

IS 6

BP 407

EP 412

AR PII S1359-6462(01)01233-7

DI 10.1016/S1359-6462(01)01233-7

UT WOS:000175818500001

ER

PT J

AU Ding, HY

Yao, KF

AF Ding, H. Y.

Yao, K. F.

TI High entropy Ti20Zr20Cu20Ni20Be20 bulk metallic glass

SO JOURNAL OF NON-CRYSTALLINE SOLIDS

AB In this letter, we report that a new bulk metallic glass (BMG) of Ti20Zr20Cu20Ni20Be20, with a critical diameter of 3 mm, has been successfully fabricated using copper mold casting method. Different from most reported BMGs which possess one or two main constituents, this newly developed BMG possesses five elements with equal atomic concentration, which has been defined as high entropy alloy. This high entropy bulk metallic glass (HE-BMG) exhibits high fracture strength of 2315 MPa but a brittle behavior. The glass forming ability, mechanical property and phase transformation after annealing of the HE-BMG are discussed in detail. And a way for designing high entropy bulk glassy alloy has been proposed. (C) 2013 Elsevier B.V. All rights reserved.

RI Ding, Hongyu/G-9833-2013

SN 0022-3093

EI 1873-4812

PD MAR 15

PY 2013

VL 364

BP 9

EP 12

DI 10.1016/j.jnoncrysol.2013.01.022

UT WOS:000316438400002

ER

PT J

AU Yu, LS

Tang, JL

Qiao, JC

Wang, H

Wang, YY

Apreutesei, M

Chamas, M

Duan, M

AF Yu, Liusi

Tang, Junlei

Qiao, Jichao

Wang, Hu

Wang, Yingying

Apreutesei, Mihai

Chamas, Mohammad

Duan, Ming

TI Effect of Yttrium Addition on Corrosion Resistance of Zr-based Bulk

Metallic Glasses in NaCl Solution

SO INTERNATIONAL JOURNAL OF ELECTROCHEMICAL SCIENCE

AB This study investigated the effect of yttrium addition on the electrochemical properties of (Zr58Nb3Cu16Ni13Al10)(100-x)Y-x (x = 0, 0.5, 2.5 at.%) bulk metallic glasses in 3.5 wt.% NaCl solution. Electrochemical measurements, scanning electron microscopy (SEM), energy dispersive spectroscopy (EDS) and X-ray photoelectron spectroscopy (XPS) were employed. Zr-based bulk metallic glasses exhibited good corrosion resistance. It was ascribed to the formation of a protective passive film which was composed of ZrO2, Al2O3 and a few oxides of other alloying elements, i.e. Y2O3. However, the breakdown potential decreased due to the addition of yttrium. Electrochemical impedance spectroscopy (EIS) suggested that yttrium addition could decrease the charge transfer resistance. That is, the addition of yttrium was harmful to the corrosion resistance of this Zr-based bulk metallic glass in 3.5 wt.% NaCl solution. It was attributed to the precipitation of Cu-Y richly quasi-crystal phase owing to yttrium addition, which caused inhomogeneous distribution of copper and yttrium in microstructure.

OI Chamas, Mohamad/0000-0002-1088-5290

SN 1452-3981

PD JUL

PY 2017

VL 12

IS 7

BP 6506

EP 6519

DI 10.20964/2017.07.47

UT WOS:000406090700056

ER

PT J

AU Mechler, S

Wanderka, N

Macht, MP

AF Mechler, S

Wanderka, N

Macht, MP

TI Crystallization behavior of low temperature pre-annealed

Zr46.8Ti8.2Ni10CU7.5Be27.5-bulk glass

SO MATERIALS SCIENCE AND ENGINEERING A-STRUCTURAL MATERIALS PROPERTIES

MICROSTRUCTURE AND PROCESSING

CT 11th International Conference on Rapidly Quenched and Metastable

Materials

CY AUG 25-30, 2002

CL Univ Oxford, Dept Mat, Oxford, ENGLAND

HO Univ Oxford, Dept Mat

AB During heat treatment of metallic bulk glasses at temperatures below the calorimetric glass transition temperature T, the glasses undergo microstructural alterations indicated by their thermal behavior as observed by DSC, but not discernible by X-ray diffraction (XRD). A reversible enthalpy recovery at T-g indicates a slow, reversible relaxation of the glass into a temperature dependent structural equilibrium state. After prolonged annealing times below T, the crystallization is irreversibly affected, i.e. it starts at a lower temperature, takes a different pathway and ends up in a different crystalline microstructure. This behavior was studied in Zr46.8Ti8.2Ni10Cu7.5Be27.5-bulk glass (V4) after preceding long term annealing well below T-g = 603 K by means of differential scanning calorimeter (DSC), XRD, scanning electron microscopy (SEM), transmission electron microscopy (TEM), and microhardness measurements. (C) 2003 Elsevier B.V. All rights reserved.

SN 0921-5093

EI 1873-4936

PD JUL 15

PY 2004

VL 375

SI SI

BP 355

EP 358

DI 10.1016/j.msea.2003.10.128

UT WOS:000223329700059

ER

PT J

AU Zumkley, T

Naundorf, V

Macht, MP

Frohberg, G

AF Zumkley, T

Naundorf, V

Macht, MP

Frohberg, G

TI Effect of reversible structural relaxation on diffusion in a ZrTiCuNiBe

bulk glass

SO SCRIPTA MATERIALIA

AB Diffusion coefficients of B and Fe were measured in the Zr46.8Ti8.2Cu7.5Ni10Be27.5 bulk glass which was relaxed at 553 K for up to nine months. While above 600 K the diffusion coefficients in the relaxed and as-cast glass were equal, at and below 553 K the diffusion coefficients in the relaxed glass were significantly lower than in the as-cast glass. (C) 2001 Acta Materialia Inc. Published by Elsevier Science Ltd. All rights reserved.

SN 1359-6462

PD AUG 31

PY 2001

VL 45

IS 4

BP 471

EP 477

DI 10.1016/S1359-6462(01)01047-8

UT WOS:000171306900015

ER

PT J

AU Rouxel, T

AF Rouxel, Tanguy

TI Elastic properties and short-to medium-range order in glasses

SO JOURNAL OF THE AMERICAN CERAMIC SOCIETY

AB Very different materials are named "Glass," with Young's modulus (E) and Poisson's ratio (nu) extending from 5 to 180 GPa and from 0.1 to 0.4, respectively, in the case of bulk inorganic glasses. Although glasses have in common the lack of long-range order in the atomic organization, they offer a wide range of structural features at the nanoscale and we show in this analysis that beside the essential role of elastic properties for materials selection in mechanical design, the elastic characteristics (E, nu) at the continuum scale allow to get insight into the short- and medium-range orders existing in glasses. In particular, nu, the atomic packing density (C-g) and the glass network dimensionality appear to be strongly correlated. Maximum values for nu and C-g are observed for metallic glasses (nu similar to 0.4 and C-g > 0.7), which are based on cluster-like structural units. Atomic networks consisting primarily of chains and layers units (chalcogenides, low Si-content silicate, and phosphate glasses) correspond to nu > 0.25 and C-g > 0.56. On the contrary, nu < 0.25 is associated with a highly cross-linked network, such as in a-SiO2, with a tri-dimensional organization resulting in a low packing density. Moreover, the temperature dependence of the elastic moduli brings a new light on the structural changes occurring above the glass transition temperature and on the depolymerization rate in the supercooled liquid. The softening rate depends on the level of cooperativity of atomic movements at the source of the deformation process, with an obvious correlation with the "fragility" of the liquid.

SN 0002-7820

PD OCT

PY 2007

VL 90

IS 10

BP 3019

EP 3039

DI 10.1111/j.1551-2916.2007.01945.x

UT WOS:000249663000001

ER

PT J

AU Jing, G

Bian, XF

Tao, L

Yan, Z

Li, TB

Bo, Z

Sun, B

AF Jing, Guo

Bian Xiufang

Tao, Lin

Yan, Zhao

Li, Taibao

Bo, Zhang

Sun Baoan

TI Formation and interesting thermal expansion behavior of novel Sm-based

bulk metallic glasses

SO INTERMETALLICS

AB Two kinds of Sm-based alloys, Sm55Al25Co20 and Sm55Al25Cu10Co10, have been cast into full glassy rods up to 4 and 3 mm in diameter, respectively, by copper mold casting. The substitution of 10 at% Co for 10 at% Cu leads to the increase in glass forming ability and thermal stability of the Sm55Al25Co20 bulk metallic glass (BMG). An interesting phenomenon is observed that Sm-based BMGs exhibit different thermal expansion behaviors compared to previously studied Cu-based BMGs above the glass transition temperature. The phenomenon correlates to the degree of contraction for the sample in the supercooled liquid region. (C) 2006 Elsevier Ltd. All rights reserved.

RI Sun, Baoan/C-6441-2012

OI Sun, Baoan/0000-0001-5306-1817

SN 0966-9795

EI 1879-0216

PD JUL

PY 2007

VL 15

IS 7

BP 929

EP 933

DI 10.1016/j.intermet.2006.11.003

UT WOS:000247415000012

ER

PT J

AU Li, S

Wang, RJ

Pan, MX

Zhao, DQ

Wang, WH

AF Li, S.

Wang, R. J.

Pan, M. X.

Zhao, D. Q.

Wang, W. H.

TI Formation and properties of RE55Al25Co20 (RE = Y, Ce, La, Pr, Nd, Gd,

Tb, Dy, Ho and Er) bulk metallic glasses

SO JOURNAL OF NON-CRYSTALLINE SOLIDS

AB We report that a series of ternary RE55Al25Co20 (RE = Y, Ce, La, Pr, Nd, Gd, Tb, Dy, Ho and Er) alloys can be readily cast into bulk glasses by a conventional casting method. The characteristics and properties of these new bulk metallic glasses (BMGs) are studied and compared. Due to the chemical comparability and well-regulated variety in atomic size, properties and elastic constants of these rare earth elements, the RE55Al25Co20 BMGs could be regarded as a model system to investigate the glass-forming ability, thermal stability, glass transition, crystallization behavior, liquid fragility, elastic and mechanical properties as well as their relationships. An attempt is made to highlight commonality and contrasts of the effects of various factors on the metallic glasses formation and properties. (C) 2007 Elsevier B.V. All rights reserved.

SN 0022-3093

EI 1873-4812

PD FEB 1

PY 2008

VL 354

IS 10-11

BP 1080

EP 1088

DI 10.1016/j.jnoncrysol.2007.08.022

UT WOS:000253216700039

ER

PT J

AU Zhang, GQ

Li, XJ

Shao, M

Wang, LN

Yang, JL

Gao, LP

Chen, LY

Liu, CX

AF Zhang, G. Q.

Li, X. J.

Shao, M.

Wang, L. N.

Yang, J. L.

Gao, L. P.

Chen, L. Y.

Liu, C. X.

TI Wear behavior of a series of Zr-based bulk metallic glasses

SO MATERIALS SCIENCE AND ENGINEERING A-STRUCTURAL MATERIALS PROPERTIES

MICROSTRUCTURE AND PROCESSING

AB Dry sliding wear behaviors of a series of bulk metallic glasses with nominal compositions of Zr48Cu45-xAl7Agx (x= 0, 2, 5, 8 at.%) are investigated by pin-on-disk method at room temperature. Compared with Ag-free Zr48Cu45Al7 glassy alloy, wear resistances of the Ag-doped bulk metallic glasses are significantly enhanced and increased with the content of Ag, and their microhardnesses also increase. The results of compression test show that the plastic strain decreases with the increase of Ag content for the quaternary Zr-Cu-Al-Ag alloys. SEM observations demonstrate that the cracks initiate and propagate perpendicular to the frictional direction during the debris formation. (C) 2007 Elsevier B.V. All rights reserved.

RI Chen, Lianyi/B-3156-2008

OI Chen, Lianyi/0000-0003-3720-398X

SN 0921-5093

PD FEB 25

PY 2008

VL 475

IS 1-2

BP 124

EP 127

DI 10.1016/j.msea.2007.05.039

UT WOS:000253693100025

ER

PT J

AU Golovin, YI

Ivolgin, VI

Khonik, VA

Kitagawa, K

Tyurin, AI

AF Golovin, YI

Ivolgin, VI

Khonik, VA

Kitagawa, K

Tyurin, AI

TI Serrated plastic flow during nanoindentation of a bulk metallic glass

SO SCRIPTA MATERIALIA

AB The results of nanoindentation tests of bulk glassy Pd40CU30Ni10P20 using a specially designed instrument with high time and spatial resolution are presented. Pronounced serrations of the indenter penetration depth are observed. The parameters of serrated flow (the number of serrations, their amplitude and duration) are dependent on the duration of the loading force pulse. (C) 2001 Acta Materialia Inc. Published by Elsevier Science Ltd. All rights reserved.

RI Tyurin, Alexander/F-1131-2017; Khonik, Vitaly/A-5888-2009; Golovin,

Yuri/P-7039-2014

SN 1359-6462

PD OCT 29

PY 2001

VL 45

IS 8

BP 947

EP 952

DI 10.1016/S1359-6462(01)01116-2

UT WOS:000172221900011

ER

PT J

AU Haruyama, O

Yokoyama, Y

Inoue, A

AF Haruyama, Osami

Yokoyama, Yoshihiko

Inoue, Akihisa

TI Precise measurement of density in the isothermal relaxation processes of

Pd42.5CU30Ni7.5P20 and Zr50CU40Al10 glasses

SO MATERIALS TRANSACTIONS

CT 5th International Conference on Bulk Metallic Glasses

CY OCT 01-05, 2006

CL Osaka Univ, Awaji Isl, JAPAN

SP Minist Educ, Culture, Sports, Sci & Technol, Inst Mat Res, Tohoku Univ, Japan Soc Promot Sci, Natl Inst Mat Sci, Hyogo Int Assoc

HO Osaka Univ

AB The isothermal relaxation processes of bulk Zr50Cu40Al10 and Pd42.5Cu30Ni7.5P20 glasses were examined by density reduction associated with relaxation. Density experiments were carried out by buoyancy method at room temperature. The isothermal relaxation Curves were best fitted by a stretched exponential function with a Kohlrausch exponent less than unity. Based on free volume model, the reduced free volume x(f)(as) = 0.0355 and flow defect concentration C-f(as) = 5.80 x 10(-13) in as-quenched state was obtained for Pd42.5Cu30Ni7.5P20 glass. However, the temperature at which the free volume started to deviate from equilibrium concentration on cooling of the melt was not in accordance with calorimetric glass transition temperature.

RI Inoue, Akihisa/E-5271-2015; Yokoyama, Yoshihiko/A-8603-2011

SN 1345-9678

EI 1347-5320

PD JUL

PY 2007

VL 48

IS 7

BP 1708

EP 1710

DI 10.2320/matertrans.MJ200772

UT WOS:000248743100027

ER

PT J

AU Sun, WS

Kulik, T

Liang, XB

Ferenc, J

AF Sun, W. S.

Kulik, T.

Liang, X. B.

Ferenc, J.

TI Thermal stability and magnetic properties of Co-Fe-Hf-Ti-Mo-B bulk

metallic glass

SO INTERMETALLICS

CT 4th International Conference on Bulk Metallic Glasses

CY MAY 01-05, 2005

CL Gatlinburg, TN

AB A new ferromagnetic Co-Fe-Hf-Ti-Mo-B bulk metallic glass was produced by copper mould casting method, using industrial raw materials. The magnetic properties were studied with the high-performance hysteresis loop tracer. The low coercivity, as low as 2 A/m for the as-cast sample, was found in the Co-Fe-based metallic glass rod with a diameter of 1.5 mm, the saturation magnetization is 0.6 T. The thermal stability was characterized by differential thermal analysis, X-ray diffraction was used to confirm the amorphous state of as-cast alloy and to perform the structural analysis of crystallization products. Crystallization temperature exceeding 900 K and supercooled liquid region of 35 K were found in the investigated glass. The high glass forming ability was explained by the slow nucleation rate of intermetallic compounds which are structurally complex in multicomponent system during solidification. High crystallization temperature suggests that the studied bulk metallic glass could be used as high-temperature soft magnetic material. (c) 2006 Elsevier Ltd. All rights reserved.

SN 0966-9795

PD AUG-SEP

PY 2006

VL 14

IS 8-9

SI SI

BP 1066

EP 1068

DI 10.1016/j.intermet.2006.01.026

UT WOS:000237770600039

ER

PT J

AU Sun, Y

Zhang, HF

Fu, HM

Wang, AM

Hu, ZQ

AF Sun, Y.

Zhang, H. F.

Fu, H. M.

Wang, A. M.

Hu, Z. Q.

TI Mg-Cu-Ag-Er bulk metallic glasses with high glass forming ability and

compressive strength

SO MATERIALS SCIENCE AND ENGINEERING A-STRUCTURAL MATERIALS PROPERTIES

MICROSTRUCTURE AND PROCESSING

AB We study the glass forming ability (GFA) and mechanical properties of the Mg-Cu-Ag-Er bulk metallic glass (BMG) systemically in this paper. A new best glass former Mg60.5Cu19.667Ag9.833Er10 is pinpointed using systematic strategy in 3D space. The critical glass formation diameter for this alloy has improved significantly from 6 mm to 11 mm. The Mg63Cu16.8Ag11.2Er9 BMG exhibits yielding and plastic deformation during compressive loading. The fracture strength and plastic strain of Mg63Cu16.8Ag11.2Er9 BMG is 1098 Wa and 0.5%, respectively. The superior mechanical properties compare with other Mg-based BMGs are attributed to the introduction of Er. (C) 2008 Elsevier B.V. All rights reserved.

RI wang, am/P-2147-2016; Sun, Yu/C-4915-2017

OI Sun, Yu/0000-0002-2136-4466

SN 0921-5093

EI 1873-4936

PD FEB 25

PY 2009

VL 502

IS 1-2

BP 148

EP 152

DI 10.1016/j.msea.2008.10.008

UT WOS:000263812500023

ER

PT J

AU Wang, ML

Wang, QM

Hui, XD

AF Wang Meiling

Wang Qingmei

Hui Xidong

TI Influences of Cooling Velocity on the Properties of Metallic Glass

SO RARE METAL MATERIALS AND ENGINEERING

AB The metallic glassy ribbons and bulk cylinders with the same compositions were prepared by melt spinning and suction casting, respectively. The influences of cooling velocity on the microstructure, thermal stability and mechanical properties of metallic glasses were studied by differential scanning calorimetry, XRD, HRTEM and Nano Indenter. The results show that the types of short ordered clusters in the glassy ribbons are the same as those in the glassy bulk cylinders, but more ordered clusters and less free volume can form in the later, because of the lower cooling velocity during preparation. Consequently, the glassy ribbons have higher thermal stability, yield strength, Young's modulus and hardness than the glassy bulk cylinders.

SN 1002-185X

PD DEC

PY 2016

VL 45

IS 12

BP 3255

EP 3261

UT WOS:000391243500042

ER

PT J

AU Qiao, JC

Pelletier, M

AF Qiao, J. C.

Pelletier, M.

TI Analysis of atomic mobility in a Cu38Zr46Ag8Al8 bulk metallic glass

SO JOURNAL OF ALLOYS AND COMPOUNDS

AB Atomic mobility in as-cast and annealed Cu38Zr46Ag8Al8 bulk metallic glass samples is analyzed by performing dynamic mechanical analysis. The loss factor is directly connected to the energy lost during application of the stress. Structural relaxation process and crystallization lead to a decrease of the atomic mobility in the bulk metallic glass. A physical model, based on the concept of quasi point defects is introduced, to describe the atomic mobility. Movements in amorphous materials are correlated. The correlation factor chi reflects the atomic mobility in bulk metallic glasses: structural relaxation and crystallization lead to a decrease of chi, implying the reduction of atomic mobility. The evolution of elastic, visco-elastic and viscoplastic components after structural relaxation and partial crystallization state during the mechanical response has been obtained. Compared with as-cast state, structural relaxation induced an increase of elastic component and a decrease of visco-elastic component in the metallic glass. (C) 2012 Elsevier B.V. All rights reserved.

SN 0925-8388

PD FEB 5

PY 2013

VL 549

BP 370

EP 374

DI 10.1016/j.jallcom.2012.10.113

UT WOS:000312109200060

ER

PT J

AU Yang, L

Ge, T

Guo, GQ

Huang, CL

Meng, XF

Wei, SH

Chen, D

Chen, LY

AF Yang, L.

Ge, T.

Guo, G. Q.

Huang, C. L.

Meng, X. F.

Wei, S. H.

Chen, D.

Chen, L. Y.

TI Atomic and cluster level dense packing contributes to the high

glass-forming ability in metallic glasses

SO INTERMETALLICS

AB The microstructure features of a representative Zr48Cu45Al7 bulk metallic glass (BMG) were investigated via a series of simulations and calculations coupled with the synchrotron radiation-based experiments. It was revealed that bond shortening occurs in the atomic pairs, due to the strong interaction between the Al dopant atoms and their neighbors. The bond shortening leads to the atomic and cluster level dense packing in the local structures, which should be the structural mechanism of the high glass-forming ability in Al-microalloyed BMGs. This work not only reveals the atomic and cluster level microstructures in this class of glass materials, but also has implications for developing other BMGs with relatively large critical sizes. (C) 2012 Elsevier Ltd. All rights reserved.

RI Wei, Shi-Hao/L-6266-2013; Chen, Lianyi/B-3156-2008

OI Chen, Lianyi/0000-0003-3720-398X

SN 0966-9795

EI 1879-0216

PD MAR

PY 2013

VL 34

BP 106

EP 111

DI 10.1016/j.intermet.2012.11.009

UT WOS:000314325200018

ER

PT J

AU Hu, YP

Lan, D

Dai, GL

Jiang, H

Duan, L

Wei, BC

AF Hu, Yanping

Lan, Ding

Dai, Guoliang

Jiang, Heng

Duan, Li

Wei, BingChen

TI Study on crystallization kinetics of binary alloys through model

colloidal mixtures

SO JOURNAL OF ALLOYS AND COMPOUNDS

CT 16th International Symposium on Metastable, Amorphous and Nanostructured

Materials

CY JUL 05-09, 2009

CL Beijing, PEOPLES R CHINA

AB Since the discovery of amorphous alloys, extensive attentions have been paid to understand the mechanism of glass-forming. The structural studies on its underlying mechanism have met challenge on direct structural characterization. It has been shown that the phase behavior of colloids dispersed in a solvent is thermodynamically equivalent to that of atoms and small molecules, however, colloids can be studied with optical microscopy due to their relatively large size. In this work, we use a binary colloidal model system with the particle size ratio comparable to atomic ratio of reported binary bulk metallic glasses to study the topological effect on crystallization and glass-forming ability of binary metallic alloys (Cu-Hf and Cu-Zr systems). The crystallization kinetics and structure of the colloid system were studied by real-time optical examination and light scattering technique. The results exhibit that there are two confined regions in the mixing ratio (composition) range in the colloid system with an enhanced glass-forming ability and retarded crystallization kinetics. The agreement between results of the model system and the experimental data on the binary bulk metallic glass formation suggests that purely topological factor plays an important role in determining the glass-forming ability. (C) 2010 Elsevier B.V. All rights reserved.

RI Lan, Ding/A-6880-2014

SN 0925-8388

EI 1873-4669

PD AUG

PY 2010

VL 504

SU 1

BP S243

EP S246

DI 10.1016/j.jallcom.2010.02.151

UT WOS:000285252600062

ER

PT J

AU Jiang, MQ

Jiang, SY

Dai, LH

AF Jiang Min-Qiang

Jiang Si-Yue

Dai Lan-Hong

TI Inherent Shear-Dilatation Coexistence in Metallic Glass

SO CHINESE PHYSICS LETTERS

AB Shear deformation can induce normal stress or hydrostatic stress in metallic glasses [ Nature Mater. 2 ( 2003) 449, Intermetallics 14 ( 2006) 1033]. We perform the bulk deformation of three-dimensional Cu(46)Zr(54) metallic glass (MG) and Cu single crystal model systems using molecular dynamics simulation. The results indicate that hydrostatic stress can incur shear stress in MG, but not in crystal. The resultant pronounced asymmetry between tension and compression originates from this inherent shear-dilatation coexistence in MG.

OI Dai, LanHong/0000-0001-8991-0358

SN 0256-307X

PD JAN

PY 2009

VL 26

IS 1

AR 016103

UT WOS:000262866100053

ER

PT J

AU Xu, J

Zhao, ZF

Zuo, M

Xing, Q

Sun, ZX

Wang, Y

AF Xu, Jing

Zhao, Zhengfeng

Zuo, Min

Xing, Qi

Sun, Zhenxi

Wang, Yan

TI Effects of Ca addition on the glass formation, microhardness and

corrosion resistance in different solutions of Zr66.7-xNi33.3Cax (x=0,

1, 3 and 5 at.%) metallic glasses

SO JOURNAL OF ALLOYS AND COMPOUNDS

AB In this study, the effects of the Ca addition on the glass forming ability (GFA), thermal stability, microhardness and corrosion resistance of as-spun Zr66.7-xNi33.3Cax (x = 0, 1, 3 and 5 at.%) alloys have been investigated using X-ray diffraction (XRD), differential scanning calorimetry (DSC), Vickers-type hardness tester and electrochemical measurement. The corroded sample surfaces were examined using field emission scanning electron microscopy (FESEM). The results show that the Ca addition can effectively enhance the GFA of the as-spun Zr-based alloys. Moreover, the Zr63.7Ni33.3Ca3 metallic glass shows the highest thermal stability indicated by the largest activation energy for crystallization. The Ca addition can effectively enhance the microhardness and 3 at.% Ca addition exhibits the highest microhardness value. The corrosion resistance of the Zr-based metallic glasses in 0.5 M NaCl, 1 M HCl and 1 M NaOH solutions has been tested by electrochemical workstation. 5 at.% Ca addition effectively improve the corrosion resistance of Zr-based metallic glasses in chloride-containing solutions, and all the Zr-based metallic glasses exhibit good corrosion resistance in NaOH solution. (C) 2014 Elsevier B. V. All rights reserved.

SN 0925-8388

EI 1873-4669

PD MAY 15

PY 2014

VL 595

BP 178

EP 184

DI 10.1016/j.jallcom.2014.01.146

UT WOS:000332396900027

ER

PT J

AU Zhou, Y

Zhao, Y

Qu, BY

Wang, L

Zhou, RL

Wu, YC

Zhang, B

AF Zhou, Y.

Zhao, Y.

Qu, B. Y.

Wang, L.

Zhou, R. L.

Wu, Y. C.

Zhang, B.

TI Remarkable effect of Ce base element purity upon glass forming ability

in Ce-Ga-Cu bulk metallic glasses

SO INTERMETALLICS

AB Raw Ce element materials of eleven different purities are used to prepare bulk metallic glasses with the same nominal composition of Ce70Ga8Cu22 (at.%). In the high-purity regime of Ce (98.13-99.87wt.%), three distinct peaks are observed in the curve plotting the purity vs. the glassy rod critical diameter (D-c); and with similar to 0.11 wt.% decrease in purity, the D-c can increase sharply from 1 to 10 mm. In the relatively low-purity regime of 96.15-98.13 wt.%, the low material purity is found to be beneficial for glass formation; and with a similar to 0.61 wt.% decrease in purity, the Dc increases dramatically from 1.5 mm to at least 20 mm. Such a sensitive and systematic purity-dependent glass-forming ability has rarely been reported before in metallic glasses. It is also suggested that the high stability of the competing crystalline phases results from the mixture effect via addition of multiple impurity elements into the matrix glass-forming alloys, and that this addition of impurity elements may be the dominant factor responsible for their intrinsic glass-forming ability of these alloys. The results provide systematic evidence for the strong purity and composition effects that are present in glass formation, and can be used to shed light on scientific research and industrial applications in the field of metallic glasses. (c) 2014 Elsevier Ltd. All rights reserved.

SN 0966-9795

EI 1879-0216

PD JAN

PY 2015

VL 56

BP 56

EP 62

DI 10.1016/j.intermet.2014.09.003

UT WOS:000347141200009

ER

PT J

AU Evenson, Z

Raedersdorf, S

Gallino, I

Busch, R

AF Evenson, Zach

Raedersdorf, Sven

Gallino, Isabella

Busch, Ralf

TI Equilibrium viscosity of Zr-Cu-Ni-Al-Nb bulk metallic glasses

SO SCRIPTA MATERIALIA

AB The equilibrium viscosities of the Zr(57)Cu(15.4)Ni(12.6)Al(10)Nb(5) and Zr(58.5)Cu(15.6)Ni(12.8)Al(10.3)Nb(2.8) bulk metallic glasses are determined in three-point beam-bending experiments. The experimental data are described by the Vogel-Fulcher-Tammann equation. The fragility parameters are found to be 35.2 and 21.0, respectively. Viscosity measurements close to the glass transition show an initial relaxation into the supercooled liquid region, followed by partial crystallization of the amorphous matrix. This is evidenced by a dramatic increase in the viscosity into a new, metastable composition for longer annealing times. (C) 2010 Acta Materialia Inc. Published by Elsevier Ltd. All rights reserved.

SN 1359-6462

PD SEP

PY 2010

VL 63

IS 6

BP 573

EP 576

DI 10.1016/j.scriptamat.2010.06.008

UT WOS:000280381900003

ER

PT J

AU Huo, LS

Bai, HY

Xi, XK

Ding, DW

Zhao, DQ

Wang, WH

Huang, RJ

Li, LF

AF Huo, L. S.

Bai, H. Y.

Xi, X. K.

Ding, D. W.

Zhao, D. Q.

Wang, W. H.

Huang, R. J.

Li, L. F.

TI Tensile properties of ZrCu-based bulk metallic glasses at ambient and

cryogenic temperatures

SO JOURNAL OF NON-CRYSTALLINE SOLIDS

AB The tensile behaviors of a series of (Zr47.5Cu47.5Al5)(1) (-) (x)(Zr80Nb20)(x) (x = 0, 0.05, 0.10, 0.15) bulk metallic glasses were studied at ambient and cryogenic (77 K) temperatures. It is found that the tensile strength of the alloys increases as the temperature decreases from 298 K to 77 K. The maximum enhancement is 15.7%. and the toughness of these alloys does not deteriorate at low temperatures. We demonstrate that the higher energy required to raise the temperature in the shear bands from the cryogenic temperature to glass transition temperature is the origin of the tensile strength enhancement at low temperatures. (C) 2011 Elsevier B.V. All rights reserved.

SN 0022-3093

EI 1873-4812

PD AUG

PY 2011

VL 357

IS 16-17

BP 3088

EP 3093

DI 10.1016/j.jnoncrysol.2011.04.017

UT WOS:000294038500009

ER

PT J

AU Laws, KJ

Shamlaye, KF

Gun, B

Ferry, M

AF Laws, Kevin J.

Shamlaye, Karl F.

Gun, Bulent

Ferry, Michael

TI Synthesis of copper-based bulk metallic glasses in the ternary Cu-Mg-Ca

system

SO JOURNAL OF ALLOYS AND COMPOUNDS

AB This paper reports the discovery of novel copper-based bulk metallic glasses free of group IV transition metals (Zr, Hf and Ti) in the Ca-Cu-Mg ternary system. Alloys of compositions ranging from Cu-33-55at.%, Mg-18-36at.% and Ca-18-36at.%, located far from eutectic reactions, were found to exhibit high glass-forming ability (up to 8 mm using conventional copper mold casting), high hardness (up to 328H(V)) and low densities (2.9-4.0g/cm(3)). (C) 2009 Elsevier B.V. All rights reserved.

RI Gun, Bulent/J-1437-2016

OI Gun, Bulent/0000-0003-1992-6404

SN 0925-8388

EI 1873-4669

PD NOV 3

PY 2009

VL 486

IS 1-2

BP L27

EP L29

DI 10.1016/j.jallcom.2009.06.205

UT WOS:000271872400008

ER

PT J

AU Li, LL

Luo, Q

Li, RF

Zhao, HY

Chapman, KW

Chupas, PJ

Wang, LH

Liu, HZ

AF Li, Liangliang

Luo, Qiang

Li, Renfeng

Zhao, Haiyan

Chapman, Karena W.

Chupas, Peter J.

Wang, Luhong

Liu, Haozhe

TI Polyamorphism in Yb-based metallic glass induced by pressure

SO SCIENTIFIC REPORTS

AB The Yb62.5Zn15Mg17.5Cu5 metallic glass is investigated using synchrotron x-ray total scattering method up to 38.4GPa. The polyamorphic transformation from low density to high density with a transition region between 14.1 and 25.2 GPa is observed, accompanying with a volume collapse reflected by a discontinuousness of isothermal bulk modulus. This collapse is caused by that distortional icosahedron short range order precedes to perfect icosahedron, which might link to Yb 4f electron delocalization upon compression, and match the result of in situ electrical resistance measurement under high pressure conditions. This discovery in Yb-based metallic glass, combined with the previous reports on other metallic glass systems, demonstrates that pressure induced polyamorphism is the general behavior for typical lanthanide based metallic glasses.

RI Liu, Haozhe/E-6169-2011

SN 2045-2322

PD APR 25

PY 2017

VL 7

AR 46762

DI 10.1038/srep46762

UT WOS:000400177200001

PM 28440339

ER

PT J

AU Kawamura, Y

Shoji, T

Ohno, Y

AF Kawamura, Y

Shoji, T

Ohno, Y

TI Welding technologies of bulk metallic glasses

SO JOURNAL OF NON-CRYSTALLINE SOLIDS

CT Annual Meeting of The-Minerals-Metals-and-Materials-Society

CY FEB 17-21, 2002

CL SEATTLE, WASHINGTON

SP Minerals Met & Mat Soc, Extract & Proc Div

AB We have succeeded in joining bulk metallic glasses (BMGs) to the same ones, different ones or commercial crystalline metallic materials by friction and electron-beam welding methods. No crystallization and no visible defects were observed in the interface. Metallurgical bonding of BMGs to BMGs and crystalline metals were obtained. The tensile strength of the welded BMGs was the same as that of the parent BMGs or crystalline metallic materials. The successful results of the friction and electron-beam welding are expected to push forward the application of BMGs. (C) 2003 Elsevier Science B.V. All rights reserved.

SN 0022-3093

PD MAR

PY 2003

VL 317

IS 1-2

BP 152

EP 157

DI 10.1016/S0022-3093(02)02005-7

UT WOS:000181251300024

ER

PT J

AU Chen, N

Louzguine-Luzgin, DV

Xie, GQ

Wada, T

Inoue, A

AF Chen, N.

Louzguine-Luzgin, D. V.

Xie, G. Q.

Wada, T.

Inoue, A.

TI Influence of minor Si addition on the glass-forming ability and

mechanical properties of Pd40Ni40P20 alloy

SO ACTA MATERIALIA

AB The influence of a minor Si addition oil the glass-forming ability and mechanical properties of Pd40Ni40P20 alloy was investigated. It is suggested that the minor Si addition can adjust the composition to be closer to eutectic, which favors a large undercooling. Furthermore, Si addition led to an enhancement in the ductility of Pd-Ni-P bulk metallic glasses. Simultaneously it is of great interest that there are nanoscale and microscale wavy steps observed on the fracture surface of Pd40Ni40Si4P16 metallic glass. It is Suggested that a quickly running crack dissipates excess energy in terms of stress wave, leaving Such wavy traces oil the fracture surface of the deformed Pd-based metallic glass. (C) 2009 Acta Materialia Inc. Published by Elsevier Ltd. All rights reserved.

RI Inoue, Akihisa/E-5271-2015; LOUZGUINE, Dmitri/D-2492-2010; Xie,

Guoqiang/A-8619-2011; Wada, Takeshi/B-2431-2015; Chen, Na/A-4120-2010

OI LOUZGUINE, Dmitri/0000-0001-5716-4987;

SN 1359-6454

PD MAY

PY 2009

VL 57

IS 9

BP 2775

EP 2780

DI 10.1016/j.actamat.2009.02.028

UT WOS:000266180300020

ER

PT J

AU Ragani, J

Volland, A

Valque, S

Liu, Y

Gravier, S

Blandin, JJ

Suery, M

AF Ragani, J.

Volland, A.

Valque, S.

Liu, Y.

Gravier, S.

Blandin, J. J.

Suery, M.

TI Using thermoforming capacity of metallic glasses to produce

multimaterials

SO JOURNAL OF ALLOYS AND COMPOUNDS

CT 16th International Symposium on Metastable, Amorphous and Nanostructured

Materials

CY JUL 05-09, 2009

CL Beijing, PEOPLES R CHINA

AB In addition to casting, thermoforming is a particularly interesting way to produce components in bulk metallic glasses since large strains can be achieved when the BMGs are deformed in their supercooled liquid region. The experimental window (temperature, time) in which high temperature forming can be carried out is directly related to the crystallization resistance of the glass. Such forming windows have been identified for zirconium based bulk metallic glasses thanks to thermal analysis and compression tests in the supercooled liquid region. Based on this identification, the thermoforming capacity of the studied glasses was used to produce multimaterials associating metallic glasses with conventional metallic alloys. Two processes have been preferentially investigated (co-extrusion and co-pressing) and the interface quality of the elaborated multi materials was studied. (C) 2010 Elsevier B.V. All rights reserved.

RI Liu, Yong/C-6526-2014

SN 0925-8388

PD AUG

PY 2010

VL 504

SU 1

BP S267

EP S270

DI 10.1016/j.jallcom.2010.03.210

UT WOS:000285252600068

ER

PT S

AU Buzdugan, D

Codrean, C

Serban, VA

Voda, M

AF Buzdugan, Dragos

Codrean, Cosmin

Serban, Viorel Aurel

Voda, Mircea

BE Nicoara, M

Opris, C

TI Mechanical behavior of Fe60Co14Ga2P10B5Si3Al3C3 bulk metallic glass

SO ADVANCED MATERIALS AND STRUCTURES V

SE Solid State Phenomena

CT 5th International Conference on Advanced Materials and Structures (AMS)

CY OCT 24-25, 2013

CL Timisoara, ROMANIA

SP Politechnica Fdn

AB Development of Fe-based bulk metallic glasses (BMG) with good mechanical and soft magnetic properties has become a major objective in the materials science field. Bulk metallic glasses present an interesting combination of properties. They exhibit very high strength (both in tension and compression), large elastic elongation limit, high hardness, excellent corrosion resistance, and good soft magnetic properties. These properties makes them suitable for many applications like high resistant control cables, pressure vessels, micro-components, pressure sensors, microgears for motors, magnetic cores for power supplies and hard fibers in composite materials. Multi-component Fe60Co14Ga2P10B5Si3Al3C3 bulk metallic glass was synthesized in rod form with a diameter of 1 mm by copper mould casting technique using raw industrial materials. The obtained alloy was analyzed by X-ray diffraction (XRD), differential thermal analysis (DTA) and scanning electron microscopy (SEM) techniques, in order to determine the phase constituent, the thermal stability and the fracture surfaces of as-cast samples. The mechanical behaviour was investigated by microhardness and compression tests. The values recorded for hardness and fracture strength includes this alloy in the category of high resistant materials.

RI Serban, Viorel - Aurel/G-4719-2016

SN 1012-0394

BN 978-3-03835-212-9

PY 2014

VL 216

BP 45

EP 48

DI 10.4028/www.scientific.net/SSP.216.45

UT WOS:000347924100008

ER

PT J

AU Kong, J

Xiong, DS

Yuan, QX

Ye, ZT

AF Kong, J

Xiong, DS

Yuan, QX

Ye, ZT

TI Strengthening bulk metallic glasses with minor alloying additions

SO TRANSACTIONS OF NONFERROUS METALS SOCIETY OF CHINA

CT 5th International Forum on Advanced Material Science and Technology

CY JUN 14-17, 2006

CL Zhangxiajie, PEOPLES R CHINA

SP Natl Nat Sci Fdn China, Xiangtan Univ, Key Lab Adv Mat & Rheol* Properties, Minist Educ China

AB Cu47Ti34Zr11Ni8, (Cu47Ti34Zr11Ni8)(99)Si and (Cu47Ti34Zr11Ni8)(99)Al bulk metallic glass were prepared by copper mold casting method, and the thermal stability, mechanical properties and microstructures of them were studied. With minor alloying of Si and Al additions, the glass transition temperature (T-g), crystallization temperature (T-x1) and temperature interval of supercooled liquid region Delta T-x (=T-x1-T-g) and reduced glass transition temperature (T-rg) were proved to be changed from 672 K, 734 K, 62 K, 0.575 to 691 K, 752 K, 61 K, 0.592 and to 681 K, 729 K, 48 K, 0.590, respectively. The results indicate that the glass-forming ability (GFA) are improved with minor alloying additions. And the bulk glasses also exhibits high three point-bending flexural strength. Because of the additions of Si and Al, three point-bending flexural strength and flexural modulus of the bulk glass change from 2 350 MPa, 102 GPa to 3 260 MPa, 102 GPa and 2 970 MPa, 108GPa respectively. The obvious strengthening is due to the appearance of the medium-range ordered regions with a size of 2-5 nm under the high-resolution TEM image. The reason that the mixed amorphous and nanocrystalline phases caused by minor alloying of Si and Al additions, is that Si or Al is the third kind of elements, which are different from other constituting elements, and there are a strong bonding and atoms size effects between constituting elements, which cause the glass-forming ability (GFA) and the bulk metallic glasses strength improving.

RI xiong, dangsheng/B-1823-2015

OI xiong, dangsheng/0000-0003-3210-3728

SN 1003-6326

PD JUN

PY 2006

VL 16

SI 1

BP S598

EP S602

DI 10.1016/S1003-6326(06)60263-X

PN B

UT WOS:000238442600036

ER

PT J

AU Chen, LY

Cao, QP

Jiang, JZ

Deng, JW

AF Chen, Lian-Yi

Cao, Qing-Ping

Jiang, J. Z.

Deng, Jing-Wei

TI Ion sputter erosion in metallic glass-A response to "Comment on:

Homogeneity of Zr(64.13)Cu(15.75)Ni(10.12)Al(10) bulk metallic glass" by

L-Y. Chen, Y-W. Zeng, Q-P. Cao, B-J. Park, Y-M. Chen, K. Hono, U.

Vainio, Z-L. Zhang, U. Kaiser, X-D. Wang, and J-Z Jiang [J. Mater. Res.

24, 3116 (2009)]

SO JOURNAL OF MATERIALS RESEARCH

AB The morphology of the dark and bright regions observed by transmission electron microscopy for the Zr(64.13)Cu(15.75)Ni(10.12)Al(10) bulk metallic glass strongly depends on the ion beam parameters used for ion milling. This indicates that the ion beam could introduce surface fluctuation to metallic glasses during ion milling.

RI Chen, Lianyi/B-3156-2008

OI Chen, Lianyi/0000-0003-3720-398X

SN 0884-2914

PD MAR

PY 2010

VL 25

IS 3

BP 602

EP 604

DI 10.1557/JMR.2010.0079

UT WOS:000275221100024

ER

PT J

AU Babilas, R

Nowosielski, R

Pawlyta, M

Fitch, A

Burian, A

AF Babilas, Rafal

Nowosielski, Ryszard

Pawlyta, Miroslawa

Fitch, Andy

Burian, Andrzej

TI Microstructural characterization of Mg-based bulk metallic glass and

nanocomposite

SO MATERIALS CHARACTERIZATION

AB New magnesium-based bulk metallic glasses Mg60Cu30Y10 have been prepared by pressure casting. Glassy alloys were successfully annealed to become nanocomposite containing 200 nm crystallites in an amorphous matrix. The microstructure of bulk glassy alloy and nanocomposite obtained during heat treatment was examined by X-ray diffraction and scanning and high-resolution electron microscopy. Metallic glass has been also studied to explain the structural characteristics by the reverse Monte Carlo (RMC) modeling based on the diffraction data. The HRTEM images allow to indicate some medium-range order (MRO) regions about 2-3 nm in size and formation of local atomic clusters. The RMC modeling results confirmed some kinds of short range order (SRO) structures. It was found that the structure of bulk metallic glass formed by the pressure casting is homogeneous. The composite material contained very small particles in the amorphous matrix. Homogeneous glassy alloy had better corrosion resistance than a composite containing nanocrystalline particles in a glassy matrix. (C) 2015 Elsevier Inc. All rights reserved.

SN 1044-5803

EI 1873-4189

PD APR

PY 2015

VL 102

BP 156

EP 164

DI 10.1016/j.matchar.2015.02.019

UT WOS:000355335200020

ER

PT J

AU Yamamoto, T

Ito, H

Hasegawa, M

Inoue, A

AF Yamamoto, Tokujiro

Ito, Hirofumi

Hasegawa, Masashi

Inoue, Akihisa

TI Mechanical properties and microstructures of composites of Ti-based

metallic glass and beta-Ti

SO MATERIALS TRANSACTIONS

CT 5th International Conference on Bulk Metallic Glasses

CY OCT 01-05, 2006

CL Osaka Univ, Awaji Isl, JAPAN

SP Minist Educ, Culture, Sports, Sci & Technol, Inst Mat Res, Tohoku Univ, Japan Soc Promot Sci, Natl Inst Mat Sci, Hyogo Int Assoc

HO Osaka Univ

AB Microstructures and mechanical properties of Cu mold-cast Ti50Cu25Ni15Sn5Ta5 and Ti45Zr5Cu44Ni5Ta1 alloys were investigated by means of X-ray diffraction, electron microscopy and compressive testing. Ti50Cu25Ni15Sn5Ta5 alloys form microscopic composites consisting of Ti-based metallic glass as primary phase and beta-Ti phases, while Ti45Zr5Cu44Ni5Ta1 alloys consisting of Ti-based metallic glass matrix and high density of nanocrystals dispersed in the matrix homogeneously. Ti50Cu25Ni15Sn5Ta5 bulk composite alloys showed 1.6% of plastic deformation and 2200 MPa of 0.2% of proof stress. The alloys also exhibited work hardening because of the presence of microscopic crystalline phases. Ti45Zr5Cu44Ni5Ta1 bulk alloys also deformed plastically after stress reached 2000 MPa without work hardening.

RI Yamamoto, Tokujiro/A-8827-2011; Inoue, Akihisa/E-5271-2015

OI Yamamoto, Tokujiro/0000-0002-4292-9446

SN 1345-9678

EI 1347-5320

PD JUL

PY 2007

VL 48

IS 7

BP 1812

EP 1815

DI 10.2320/matertrans.MJ200756

UT WOS:000248743100049

ER

PT S

AU Aydiner, CC

Ustundag, E

Prime, MB

Peker, A

AF Aydiner, CC

Ustundag, E

Prime, MB

Peker, A

BE Dias, AM

Pina, J

Batista, AC

Diogo, E

TI Residual stresses in bulk metallic glasses - II: Measurement

SO ECRS 6: PROCEEDINGS OF THE 6TH EUROPEAN CONFERENCE ON RESIDUAL STRESSES

SE Materials Science Forum

CT 6th European Conference on Residual Stresses (ECRS 6)

CY JUL 10-12, 2002

CL COIMBRA, PORTUGAL

SP Reitoria Univ Coimbra, Fund Ciencia Tecnol, Minist Ciencia Technol, Apoio Programa Operat Ciencia, Tech, Inovacao Quadro Commun Apoio III, Fund Calouste Gulbenkian, Camara Municipal Coimbra, Governo Civil Coimbra, Comiss Coordenac Reg Ctr, Embaicada Franca, BPI

AB New multi-component metallic alloys with exceptional glass forming ability have recently been developed at Caltech. These alloys allow the processing of large amorphous specimens. The possibility of formation of thermal-tempering-induced residual stresses during the processing of these bulk metallic glass (BMG) specimens was investigated. The crack compliance method was used to measure the stress profiles in a BMG plate that was cast in a copper mold. The measured profiles were roughly parabolic suggesting that thermal tempering was the dominant residual stress generation mechanism. However, the magnitude of the measured stresses was significantly lower than modeling predictions. Possible reasons for this discrepancy are presented in relation to the actual casting process and material properties.

RI Aydiner, Cahit/O-9618-2017; Ustundag, Ersan/C-1258-2009

OI Aydiner, Cahit/0000-0001-8256-6742; Ustundag, Ersan/0000-0002-0812-7028;

Prime, Michael/0000-0002-4098-5620

SN 0255-5476

BN 0-87849-900-8

PY 2002

VL 404-7

BP 257

EP 262

DI 10.4028/www.scientific.net/MSF.404-407.257

UT WOS:000177256900039

ER

PT J

AU Qin, FX

Zhou, Y

Ji, C

Dan, ZH

Xie, GQ

Yang, S

AF Qin, Feng-Xiang

Zhou, Yang

Ji, Chuan

Dan, Zhen-Hua

Xie, Guo-Qiang

Yang, Sen

TI Enhanced Mechanical Properties, Corrosion Behavior and Bioactivity of

Ti-based Bulk Metallic Glasses with Minor Addition Elements

SO ACTA METALLURGICA SINICA-ENGLISH LETTERS

AB In this research, corrosion behavior, mechanical properties and bioactivity of Ti-Zr-Cu-Pd-Sn bulk metallic glasses with minor addition of Au, Pt, Nb or Ta elements were investigated. The results revealed that minor additions of the elements were beneficial to enhancing mechanical properties and corrosion resistance of Ti-based bulk metallic glasses. Minor addition of the element (especially with Nb and Ta addition) results in the improvement in plastic deformation ability due to the existing of nanoparticles with a size smaller than 10 nm in glassy matrix, inhibiting the deformation of the shear bonds. Enrichments of Ti and Zr elements in oxide layer were responsible for high corrosion resistance. The bioactivity of Ti-based bulk metallic glasses was also investigated. The best combination of large plastic deformation ability, good corrosion resistance and bioactivity in Ti40Zr10Cu33Pd14Sn2Ta1 BMG was obtained.

RI Xie, Guoqiang/A-8619-2011

OI Dan, Zhenhua/0000-0002-3026-685X

SN 1006-7191

EI 2194-1289

PD NOV

PY 2016

VL 29

IS 11

BP 1011

EP 1018

DI 10.1007/s40195-016-0468-0

UT WOS:000387275200004

ER

PT J

AU Qiao, JW

Jia, HL

Liaw, PK

AF Qiao, Junwei

Jia, Haoling

Liaw, Peter K.

TI Metallic glass matrix composites

SO MATERIALS SCIENCE & ENGINEERING R-REPORTS

AB The mechanical properties of ex-situ and in-situ metallic glass matrix composites (MGMCs) have proven to be both scientifically unique and of potentially important for practical applications. However, the underlying deformation mechanisms remain to be studied. In this article, we review the development, fabrication, microstructures, and properties of MGMCs, including the room-temperature, cryogenic temperature, and high-temperature mechanical properties upon quasi-static and dynamic loadings. In parallel, the deformation mechanisms are experimentally and theoretically explored. Moreover, the fatigue, corrosion, and wear behaviors of MGMCs are discussed. Finally, the potential applications and important unresolved issues are identified and discussed. Crown Copyright (C) 2015 Published by Elsevier B.V. All rights reserved.

SN 0927-796X

EI 1879-212X

PD FEB

PY 2016

VL 100

BP 1

EP 69

DI 10.1016/j.mser.2015.12.001

UT WOS:000370994800001

ER

PT J

AU Guduru, RK

Darling, KA

Scattergood, RO

Koch, CC

Murty, KL

Bakkal, M

Shih, AJ

AF Guduru, R. K.

Darling, K. A.

Scattergood, R. O.

Koch, C. C.

Murty, K. L.

Bakkal, M.

Shih, A. J.

TI Shear punch tests for a bulk metallic glass

SO INTERMETALLICS

AB A shear punch test technique was used for characterization of the mechanical properties of Zr-5Ti-17.9Cu-14.6Ni-10Al bulk metallic glass. The ultimate shear stress values matched very closely with values derived from uniaxial compression tests reported in the literature. This is consistent with a lack of pressure sensitivity in compression reported for this particular metallic glass. Deformation response was strain rate insensitive up to a critical rate, beyond which softening occurred. The latter was attributed to thermal heating effects. (c) 2006 Elsevier Ltd. All rights reserved.

RI Scattergood, Ronald/D-5204-2009; GUDURU, RAMESH KUMAR/N-8541-2016; Koch,

Carl/B-9101-2008; GUDURU, RAMESH KUMAR/G-6631-2011

OI GUDURU, RAMESH KUMAR/0000-0002-9570-4208;

SN 0966-9795

PD DEC

PY 2006

VL 14

IS 12

BP 1411

EP 1416

DI 10.1016/j.intermet.2006.01.052

UT WOS:000240782800006

ER

PT J

AU Reyes-Retana, JA

Naumis, GG

AF Reyes-Retana, J. A.

Naumis, G. G.

TI Ab initio study of Si doping effects in Pd-Ni-P bulk metallic glass

SO JOURNAL OF NON-CRYSTALLINE SOLIDS

AB In order to understand the improved glass formation ability of bulk metallic glass due to Si doping, several structures were obtained by means of density functional theory. The results indicate that Si enters mainly as a substitute for P in the clusters, inducing a shoving effect in the P-P random network, The corresponding electronic properties indicate an enhanced cluster stabilization due to an electronic mechanism, which reduces the density of states at the Fermi level. (C) 2014 Elsevier B.V. All rights reserved.

OI Naumis, Gerardo/0000-0002-1338-1522

SN 0022-3093

EI 1873-4812

PD FEB 1

PY 2015

VL 409

BP 49

EP 53

DI 10.1016/j.jnoncrysol.2014.11.011

UT WOS:000348888000008

ER

PT J

AU Yu, P

Bai, HY

AF Yu, Peng

Bai, H. Y.

TI Poisson's ratio and plasticity in CuZrAl bulk metallic glasses

SO MATERIALS SCIENCE AND ENGINEERING A-STRUCTURAL MATERIALS PROPERTIES

MICROSTRUCTURE AND PROCESSING

AB The study of compositional dependence of Poisson's ratio and compressive plasticity in a CuZr-based bulk metallic glass (BMG) forming system demonstrates that the plasticity of the BMGs is very sensitive to the change of composition. The sensitivity can be well characterized by the Poisson's ratio. The Poisson's ratio and plasticity have a homologous evolution with regard to the adjustment of composition in the alloys. The work has implication that the correlation between the Poisson's ratio and plasticity might provide useful guideline for the development of plastic BMGs in known or unknown BMG-forming alloys. (C) 2007 Elsevier B.V. All rights reserved.

SN 0921-5093

PD JUN 25

PY 2008

VL 485

IS 1-2

BP 1

EP 4

DI 10.1016/j.msea.2007.07.062

UT WOS:000256194300001

ER

PT J

AU Volland, A

Ragani, J

Liu, Y

Gravier, S

Suery, M

Blandin, JJ

AF Volland, A.

Ragani, J.

Liu, Y.

Gravier, S.

Suery, M.

Blandin, J. J.

TI Design of multi materials combining crystalline and amorphous metallic

alloys

SO JOURNAL OF ALLOYS AND COMPOUNDS

AB Multi materials, associating zirconium based bulk metallic glasses and crystalline metallic alloys like magnesium alloys or copper are elaborated by co-deformation processing performed in the supercooled liquid regions (SLR) of the bulk metallic glasses. Two processes are investigated: co-extrusion and co-pressing. In the first case, filamentary composites with various designs can be produced whereas in the second case sandwich structures are obtained. The experimental window (temperature, time) in which processing can be carried out is directly related to the crystallisation resistance of the glass which requires getting information about the crystallisation conditions in the selected metallic glasses. Thermoforming windows are identified for the studied BMGs by thermal analysis and compression tests in their SLR. The mechanical properties of the produced multi materials are investigated thanks to specifically developed mechanical devices and the interfaces between the amorphous and the crystalline alloys are characterised. (c) 2012 Elsevier B.V. All rights reserved.

RI Liu, Yong/C-6526-2014

SN 0925-8388

PD SEP 25

PY 2012

VL 536

SU 1

BP S143

EP S147

DI 10.1016/j.jallcom.2011.12.043

UT WOS:000310837500033

ER

PT J

AU Go, YH

Cho, J

Jeong, CY

Kang, CS

Park, JS

AF Go, Y. H.

Cho, J.

Jeong, C. Y.

Kang, C. S.

Park, J. S.

TI Stress distribution of bulk metallic glass/metal laminate composites

during uni-axial fracture

SO MATERIALS SCIENCE AND ENGINEERING A-STRUCTURAL MATERIALS PROPERTIES

MICROSTRUCTURE AND PROCESSING

AB The fracture behaviors of laminated composites of bulk metallic glass (BMG (Vitreloy I))/crystalline layer have been examined via both experimental and simulation approaches. The specimen array of BMG/Zr/BMG by an electron-discharge bonding technique showed a completed bonding, and the fracture tests showed that when the number of BMG layer increases up to three layers, the fracture energy for the laminated specimen is increased. The simulation results of the laminated composite exhibited that the enhancement of fracture energy for the composites is mainly due to the shear stress distribution through the interface between BMG layers. (C) 2007 Elsevier B.V. All rights reserved.

SN 0921-5093

PD JUL 15

PY 2007

VL 460

BP 377

EP 382

DI 10.1016/j.msea.2007.01.079

UT WOS:000246726200053

ER

PT J

AU He, G

Loser, W

Eckert, J

AF He, G

Loser, W

Eckert, J

TI Devitrification and phase transformation of (Ti(0.5)Cu0.25Ni(0.15)

Sn0.05Zr0.05)(100-x) Mo-x metallic glasses

SO SCRIPTA MATERIALIA

AB The thermal stability and the crystallization behavior of the melt-spun ribbons of (Ti0.5Cu0.25Ni0.15Sn0.05Zr0.05)(100-x)Mo-x metallic glasses were investigated in terms of the thermal analysis. The crystallization during the glass devitrification and the solidification of the liquid for the alloys exhibit a strong similarity. (C) 2003 Acta Materialia Inc. Published by Elsevier Ltd. All rights reserved.

SN 1359-6462

PD JAN

PY 2004

VL 50

IS 1

BP 7

EP 11

DI 10.1016/j.scriptamat.2003.09.049

UT WOS:000186207800002

ER

PT J

AU Kanungo, BP

Glade, SC

Asoka-Kumar, P

Flores, KM

AF Kanungo, BP

Glade, SC

Asoka-Kumar, P

Flores, KM

TI Characterization of free volume changes associated with shear band

formation in Zr- and Cu-based bulk metallic glasses

SO INTERMETALLICS

CT 3rd International Conference on Bulk Metallic Glasses

CY OCT 12-16, 2003

CL Beijing, PEOPLES R CHINA

AB The free volume model for flow in metallic glasses predicts a significant increase in free volume at the onset of plastic deformation. The details of these structural changes are unclear, however, particularly during strain localization in shear bands. In this study, the free volume changes associated with inhomogeneous plastic deformation of a Cu-based bulk metallic glass were examined using positron annihilation spectroscopy (PAS). PAS results indicated that there was a distribution of free volume site sizes in both the as-quenched and rolled glasses, and that the concentration of larger sites increased with deformation. Differential scanning calorimetry was also used to observe the glass transition behaviors of Cu- and Zr-based glasses after rolling and annealing. Annealing resulted in an increase in the height of the endothermic glass transition peak, consistent with structural relaxation relative to the as-quenched material. Deformation resulted in both a lower endothermic peak height and an earlier and deeper exothermic peak associated with structural relaxation, indicating a more disordered structure with more free volume. (C) 2004 Elsevier Ltd. All rights reserved.

SN 0966-9795

PD OCT-NOV

PY 2004

VL 12

IS 10-11

BP 1073

EP 1080

DI 10.1016/j.intermet.2004.04.033

UT WOS:000224566700008

ER

PT J

AU Birol, Y

AF Birol, Y

TI Low-temperature crystallization of Fe78B13Si9 and Fe81B13.5Si3.5C2

metallic glasses

SO MATERIALS SCIENCE AND ENGINEERING A-STRUCTURAL MATERIALS PROPERTIES

MICROSTRUCTURE AND PROCESSING

AB The low-temperature crystallization behavior of Fe78B13Si9 and Fe81B13.5Si3.5C2 metallic glasses was investigated. Samples of each glass were isothermally annealed in vacuum and in air between 623 and 773 K. X-ray diffraction, optical-electron microscopy and differential scanning calorimetry were employed to follow the crystallization reaction. Crystallization in Fe78B13Si9 below 748 K always starts at the surface of the ribbons and is more prevalent on the wheel-side. Primary alpha(Fe) dendrites grow in a direction more or less parallel to the surface normal. Further growth of these dendrites is no longer evident, however, once crystallization in the bulk starts. When annealed in air, the air-side of the ribbons have also experienced substantial surface crystallization prior to any crystallization in the bulk. The response of the Fe81B13.5Si3.5C2 metallic glass to low-temperature anneals, on the other hand, is considerably different. Crystallization takes place predominantly in the interior of the ribbons, regardless of the annealing temperature and the atmosphere. Differences in the low-temperature crystallization behavior of these glasses and the underlying mechanisms are discussed. (C) 1998 Elsevier Science S.A. All rights reserved.

OI birol, yucel/0000-0002-5829-1045

SN 0921-5093

PD JUN 30

PY 1998

VL 249

IS 1-2

BP 79

EP 83

DI 10.1016/S0921-5093(98)00611-X

UT WOS:000075310200012

ER

PT J

AU Cheng, JL

Chen, G

Zhang, ZW

Wang, ZZ

Wang, ZY

Li, XQ

AF Cheng, Jia-Lin

Chen, Guang

Zhang, Zhong-Wu

Wang, Zhang-Zhong

Wang, Zai-You

Li, Xiao-Quan

TI Oxygen segregation in the Zr-based bulk metallic glasses

SO INTERMETALLICS

AB Effects of oxygen on the glass formation of Zr41.2Ti13.8Cu12.5Ni10Be22.5 and Zr39.6Ti33.9Nb7.6Ni6.4Be12.5 alloys were studied. Our results indicated that oxygen is segregated in the precipitated Zr3NiO-type and beta-Zr crystals, respectively, making the glass matrices keep good glass formation. Interestingly, as compared with the oxygen-free sample, the Zr39.6Ti33.9Nb7.6Ni6.4Be12.5 added with 2000 ppm oxygen exhibits much higher yield stress with only a little ductility loses. This founding gives us a new clue to avoid the detriment of oxygen to the to the glass formation just by designing appropriate glass composites. (C) 2014 Elsevier Ltd. All rights reserved.

RI zhang, zhongwu/G-1875-2012

OI zhang, zhongwu/0000-0002-2874-2976

SN 0966-9795

EI 1879-0216

PD JUN

PY 2014

VL 49

BP 149

EP 153

DI 10.1016/j.intermet.2014.01.002

UT WOS:000334085800022

ER

PT J

AU Matsuura, M

Konno, K

Asada, K

Sakurai, M

Zhang, W

Inoue, A

AF Matsuura, M.

Konno, K.

Asada, K.

Sakurai, M.

Zhang, W.

Inoue, A.

TI Embrittlement and local structures for the Cu-, Ni- and Zr-based bulk

metallic glasses

SO MATERIALS SCIENCE AND ENGINEERING A-STRUCTURAL MATERIALS PROPERTIES

MICROSTRUCTURE AND PROCESSING

CT 12th International Conference on Rapidly Quenched and Metastable

Materials

CY AUG 21-26, 2005

CL Cheju Isl, SOUTH KOREA

AB To study the microscopic reasons for the embrittlement of the bulk metallic glasses, the local structures around main constituent atoms, i.e. Zr, Ni and Cu in the Zr60Al15Ni25, Ni60Nb20Ti15Zr5 and Cu60Zr30Ti10 glasses (numbers indicate at.%) were investigated by X-ray absorption fine structure measurements. The fluctuations in the interatomic distance (sigma 2) of the main constituent atoms in the three metallic glasses are deduced from the results. The sigma 2 value for Zr in the Zr60Al15Ni25 was highest in terms of temperature variation as well as in the absolute value among the three metallic glasses even though Zr has the largest atomic size among the constituent atoms. A peak in the Fourier transform of the Zr K-edge for Zr60Al15Ni25 at a short distance may be associated with oxygen around Zr atoms. (c) 2006 Elsevier B.V. All rights reserved.

RI Inoue, Akihisa/E-5271-2015

SN 0921-5093

PD MAR 25

PY 2007

VL 449

BP 535

EP 537

DI 10.1016/j.msea.2006.02.378

UT WOS:000245477800121

ER

PT J

AU Mubarok, A

Hebert, RJ

AF Mubarok, A.

Hebert, R. J.

TI Thermomechanical Behavior of Cu50Hf41.5Al8.5 Bulk Metallic Glass after

Sustained Elastic Deformation

SO METALLURGICAL AND MATERIALS TRANSACTIONS A-PHYSICAL METALLURGY AND

MATERIALS SCIENCE

CT International Conference on Bulk Metallic Glasses held at the 2009 TMS

Annual Meeting

CY 2009

CL San Francisco, CA

SP Minerals, Met & Mat Soc

AB Thermomechanical analysis (TMA) was conducted in a temperature modulated mode to analyze the effect of static and dynamic elastic compression on a Cu50Hf41.5Al8.5 bulk metallic glass. The nonreversible length changes clearly demonstrate that the elastic loading affects the thermomechanical behavior of the metallic glass. A sustained static elastic compressive load increases the relative length decrease, while a dynamic elastic load to the same maximum load and for the same time reduces the length decrease. A preliminary interpretation suggests that the static compression raises the defect of free volume level, but the dynamic compression mimics annealing and reduces the free volume level. Elastic compression thus emerges as a novel tool to control the free volume level of metallic glasses.

SN 1073-5623

EI 1543-1940

PD JUL

PY 2010

VL 41A

IS 7

BP 1658

EP 1663

DI 10.1007/s11661-009-0067-z

UT WOS:000277958700010

ER

PT J

AU Louzguine, DV

Inoue, A

AF Louzguine, DV

Inoue, A

TI Structural and thermal investigations of a high-strength Cu-Zr-Ti-Co

bulk metallic glass

SO PHILOSOPHICAL MAGAZINE LETTERS

AB Cu55Zr30Ti10Co5 bulk metallic glass exhibits a high compressive fracture strength of 2.31 GPa and Young's modulus of 130 GPa, values that are higher than those of other Cu- and Zr-based metallic glasses. The addition of Co to the ternary Cu-Zr-Ti alloy stabilizes the supercooled liquid. On heating, the Cu55Zr30Ti10Co5 metallic glass devitrified and formed an intermediate intermetallic compound prior to reaching equilibrium by diffusion-controlled growth at constant nucleation rate. In the fully annealed state the structure consists of the equilibrium Cu10Zr7 phase with the slightly reduced lattice parameters a = 0.933 nm, b = 0.928 nm and c = 1.254 nm and a small fraction of an unidentified phase.

RI Inoue, Akihisa/E-5271-2015; LOUZGUINE, Dmitri/D-2492-2010

OI LOUZGUINE, Dmitri/0000-0001-5716-4987

SN 0950-0839

PY 2003

VL 83

IS 3

BP 191

EP 196

DI 10.1080/0950083031000066126

UT WOS:000180668300006

ER

PT J

AU Kobelev, NP

Kolyvanov, EL

Khonik, VA

AF Kobelev, N. P.

Kolyvanov, E. L.

Khonik, V. A.

TI Higher-order elastic moduli of the metallic glass Pd40Cu30Ni10P20

SO PHYSICS OF THE SOLID STATE

AB The effect of uniaxial loading on the parameters of ultrasonic wave propagation in the bulk metallic glass Pd40Cu30Ni10P20 has been studied. The third-order and fourth-order elastic moduli have been calculated based on the obtained results. It has been shown that the nonlinearity of the elastic properties of this glass are substantially higher than that of the previously studied Zr-based glass.

RI Khonik, Vitaly/A-5888-2009

SN 1063-7834

EI 1090-6460

PD AUG

PY 2015

VL 57

IS 8

BP 1483

EP 1487

DI 10.1134/S1063783415080119

UT WOS:000358796000001

ER

PT J

AU Matsuura, M

Fujita, T

Konno, K

Zhang, W

Chen, MW

AF Matsuura, M.

Fujita, T.

Konno, K.

Zhang, W.

Chen, M. W.

TI Direct structural evidence for dynamic heterogeneity in supercooled

liquid bulk metallic glass

SO SCRIPTA MATERIALIA

AB In situ measurements of the X-ray absorption fine structure for the supercooled liquid state of Au65Cu10.5Si17Ag7.5 bulk metallic glass, which exhibits extraordinarily low T-g, and a stable supercooled liquid state, were performed. Analyses of data combined with molecular dynamics simulation suggest that the glass transition is caused by the breakdown of Cu-Cu bonding which connects Cu-centered icosahedral clusters. Cu atoms play a key role in the enhancement of glass formability and the low T-g of the Au65Cu10.5Si17Ag7.5 bulk metallic glass. (C) 2012 Acta Materialia Inc. Published by Elsevier Ltd. All rights reserved.

RI Fujita, Takeshi/B-1867-2009; CHEN, Mingwei/A-4855-2010

OI Fujita, Takeshi/0000-0002-2318-0433; CHEN, Mingwei/0000-0002-8274-3099;

Chen, Mingwei/0000-0002-2850-8872

SN 1359-6462

PD JUN

PY 2012

VL 66

IS 11

BP 927

EP 930

DI 10.1016/j.scriptamat.2012.02.033

UT WOS:000303621900020

ER

PT J

AU Gonzalez, S

Chen, N

Zhang, QS

Louzguine-Luzgin, DV

Perepezko, JH

Inoue, A

AF Gonzalez, S.

Chen, N.

Zhang, Q. S.

Louzguine-Luzgin, D. V.

Perepezko, J. H.

Inoue, A.

TI Effect of shear bands initiated in the pre-yield region on the

deformation behaviour of Zr-based metallic glasses

SO SCRIPTA MATERIALIA

AB The effect of serrations initiated in the elastic deformation region before macroscopic yielding on the mechanical behaviour of Zr65Fe5Cu20Al10 bulk metallic glassy rods was analysed using a large number of samples. The apparent work-hardening-like behaviour and the plasticity tend to increase with the number of shear bands in the pre-yield region, which was analysed in terms of interaction with the shear bands generated during plastic deformation. (C) 2010 Acta Materialia Inc. Published by Elsevier Ltd. All rights reserved.

RI Inoue, Akihisa/E-5271-2015; LOUZGUINE, Dmitri/D-2492-2010; Chen,

Na/A-4120-2010; Gonzalez, Sergio/A-4852-2010

OI LOUZGUINE, Dmitri/0000-0001-5716-4987; Gonzalez Sanchez,

Sergio/0000-0002-0211-7822

SN 1359-6462

PD APR

PY 2011

VL 64

IS 8

BP 713

EP 716

DI 10.1016/j.scriptamat.2010.12.025

UT WOS:000287908300006

ER

PT J

AU Bhatt, J

Murty, BS

AF Bhatt, Jatin

Murty, B. S.

TI Thermodynamic modeling of Zr-Ti-Cu-Ni-Be bulk metallic glass

SO TRANSACTIONS OF THE INDIAN INSTITUTE OF METALS

AB In the present thermodynamic model, the optimization of bulk metallic glass forming compositions in Zr-Ti-Cu-Ni-Be system using enthalpy of chemical mixing (Delta H(chem)) as thermodynamic, mismatch entropy (Delta S(sigma)/k(B)) as topological and configurational entropy (Delta S(config)/R) as statistical parameters were studied. The product of Delta H(chem) and Delta S(sigma)/k(B) which is termed as Delta P(HS) in the Delta S(config)/R range of 0.9 to 1.0 can be strongly correlated to glass forming ability. Using the contributions of Delta P(HS) values of all ternary compositions possible in Zr-Ti-Cu-Ni-Be system, the final quinary composition is designed. Composition obtained from present model was found to closely match with compositions reported in literature.

RI Murty, BS/P-3354-2015

OI Murty, BS/0000-0002-4399-8531

SN 0019-493X

PD OCT

PY 2009

VL 62

IS 4-5

BP 413

EP 416

DI 10.1007/s12666-009-0056-3

UT WOS:000276133700030

ER

PT J

AU Luo, Q

Wang, WH

AF Luo, Q.

Wang, W. H.

TI Rare earth based bulk metallic glasses

SO JOURNAL OF NON-CRYSTALLINE SOLIDS

AB Recently, the rare earth based bulk metallic glasses (REBMGs) have attracted increasing interest due to their unique properties and potential applications as functional glassy materials. These REBMGs display many fascinating properties such as heavy fermion behavior, thermoplastic properties near room temperature, excellent magnetocaloric effect, hard magnetism, and polyamorphism, all of which are of interest not only for basic research but also for metallurgy and technology. These characteristics and properties are ascribed to the unique electronic, magnetic and atomic structures of the REBMGs. In this review paper, the fabrication, glass-forming ability, polyamorphism, elastic, thermal, and physical properties are summarized and discussed. Owing to the unique electronic structure of rare earth elements, the electric and magnetic properties of the REBMGs are especially addressed. The works have implications for seeking novel metallic glasses with controllable properties and for understanding the nature of glass formation. The development of REBMGs as functional materials might promote and extend the commercial applications of metallic glasses. (C) 2009 Elsevier B.V. All rights reserved

SN 0022-3093

EI 1873-4812

PD MAY 15

PY 2009

VL 355

IS 13

BP 759

EP 775

DI 10.1016/j.jnoncrysol.2009.02.006

UT WOS:000266649100001

ER

PT J

AU Kohda, M

Haruyama, O

Ohkubo, T

Egami, T

AF Kohda, M.

Haruyama, O.

Ohkubo, T.

Egami, T.

TI Kinetics of volume and enthalpy relaxation in Pt60Ni15P25 bulk metallic

glass

SO PHYSICAL REVIEW B

AB We examined the kinetics of the structural relaxation in a Pt60Ni15P25 bulk metallic glass by density and enthalpy measurements. Measurements were made slightly below the glass transition temperature, with and without preannealing at a temperature above the glass transition temperature. The results are elucidated in terms of the two-components model, which includes positive as well as negative fluctuations in local density.

SN 2469-9950

EI 2469-9969

PD MAR 1

PY 2010

VL 81

IS 9

AR 092203

DI 10.1103/PhysRevB.81.092203

UT WOS:000276207300007

ER

PT J

AU Zhang, AL

Chen, D

Chen, ZH

AF Zhang, Ailong

Chen, Ding

Chen, Zhenhua

TI Predicting the eutectic compositions of four multicomponent alloy

systems by a simple approach

SO JOURNAL OF ALLOYS AND COMPOUNDS

AB A simple approach based on the efficient cluster packing model (ECP model) was proposed to predict eutectic compositions in Ca-Mg-Zn, Mg-Cu-Y, Zr-Cu-Al ternary and Zr-Ti-Ni-Cu-Al quinary alloy systems. Predicted eutectic compositions are found to be in good agreement with experimental results. It may provide new insights into atomic packing of multicomponent eutectic alloy containing no more than four topologically different elements, and may be a new and simple way to obtain novel bulk metallic glasses in these alloy systems. (C) 2010 Elsevier B.V. All rights reserved.

RI 陈(chen), 鼎(ding)/O-5087-2015; 鼎, 陈/D-1695-2009

OI 陈(chen), 鼎(ding)/0000-0003-0407-7542; 鼎, 陈/0000-0001-6422-4597

SN 0925-8388

PD JAN 21

PY 2011

VL 509

IS 3

BP 648

EP 650

DI 10.1016/j.jallcom.2010.09.147

UT WOS:000285318400020

ER

PT J

AU Nieh, TG

Wadsworth, J

AF Nieh, TG

Wadsworth, J

TI Homogeneous deformation of bulk metallic glasses

SO SCRIPTA MATERIALIA

AB Homogeneous deformation of bulk metallic glasses (BMGs) is reviewed. Homogeneous deformation usually takes place at temperatures near and above the glass transition temperature. The deformation behavior depends upon strain rate. At low strain rates, BMGs behave like a Newtonian fluid (m = 1) but plastic flow becomes non-Newtonian at high strain rates. The non-Newtonian behavior is a result of microstructural instability, namely, the concurrent formation of nanocrystals in the amorphous matrix during deformation. Despite the difference in deformation behavior, BMGs usually show large ductility (over 300%) at temperatures in the supercooled liquid region. (c) 2005 Acta Materialia Inc. Published by Elsevier Ltd. All rights reserved.

RI Nieh, Tai-Gang/G-5912-2011

OI Nieh, Tai-Gang/0000-0002-2814-3746

SN 1359-6462

PD FEB

PY 2006

VL 54

IS 3

BP 387

EP 392

DI 10.1016/j.scriptamat.2005.04.052

UT WOS:000233495600013

ER

PT S

AU Yang, F

Gao, ZH

Wang, D

Liu, Y

AF Yang, Feng

Gao, Zhi-hua

Wang, Di

Liu, Yang

BE William, Z

Xiong, F

TI An experimental study for crystallization characteristics of Nd-based

alloy

SO COMPUTATIONAL MATERIALS SCIENCE, PTS 1-3

SE Advanced Materials Research

CT International Conference on Computational Materials Science (CMS 2011)

CY APR 17-18, 2011

CL Guangzhou, PEOPLES R CHINA

AB The bulk metallic glass has received much attention from scientist and engineer. In this paper, we fabricated metallic glass of Nd-based and carried out experimental research. This kind of metallic glass has shown a distinct glass transition and stable super-cooled liquid region. At the same time, we find the paramagnetic performance of Nd-based metallic glass is different from other hard magnetic alloys at ordinary temperature. The DSC experiment indicate that the glass transition temperature increase with the heating temperature.

SN 1022-6680

BN 978-3-03785-155-5

PY 2011

VL 268-270

BP 611

EP 615

DI 10.4028/www.scientific.net/AMR.268-270.611

PN 1-3

UT WOS:000303850200115

ER

PT J

AU Jung, HY

Stoica, M

Yi, S

Kim, DH

Eckert, J

AF Jung, H. Y.

Stoica, M.

Yi, S.

Kim, D. H.

Eckert, J.

TI Preparation of cast-iron-based nanocrystalline alloy with Cu and Nb

addition

SO INTERMETALLICS

AB The effect of minor Cu and Nb addition on glass-forming ability and nanocrystallization behavior of castiron-based bulk metallic glass was investigated. With simultaneous Cu and Nb addition, the crystallization kinetics of the amorphous alloys adequately modified to have enlarged initial nucleation rate and reduced growth rate of the primary alpha-Fe phase. However, due to the increased tendency to form secondary Fe3C and Fe2P phase, the cast-iron-based bulk metallic glass with combined Cu and Nb addition had a reduced processing window for nanocrystallization. Also, the simultaneous Cu and Nb addition decreased glass-forming ability of the alloy. Thus, it was revealed that the classical nanocrystallization strategy introducing combined Cu and Nb addition is not a suitable alternative to facilitate nanocrystallization of cast-iron-based bulk metallic glasses. (c) 2015 Elsevier Ltd. All rights reserved.

RI Stoica, Mihai/B-7069-2015

SN 0966-9795

EI 1879-0216

PD FEB

PY 2016

VL 69

BP 54

EP 61

DI 10.1016/j.intermet.2015.10.014

UT WOS:000366772700009

ER

PT J

AU Jiang, QK

Zhang, GQ

Yang, L

Wang, XD

Saksl, K

Franz, H

Wunderlich, R

Fecht, H

Jiang, JZ

AF Jiang, Q. K.

Zhang, G. Q.

Yang, L.

Wang, X. D.

Saksl, K.

Franz, H.

Wunderlich, R.

Fecht, H.

Jiang, J. Z.

TI La-based bulk metallic glasses with critical diameter up to 30 mm

SO ACTA MATERIALIA

AB We report composition optimization, thermal and physical properties of new La-based bulk metallic glasses with high glass forming ability (GFA) based on a ternary La62Al14Cu24 alloy. By refining the (Cu, Ag)/(Ni, Co) and La/(Cu, Ag) ratios in the La-Al-(Cu,Ag)-(Ni, Co) pseudo-quaternary alloy, the formation of 30 mm diameter of La65Al14(Cu5/6Ag1/6)(11)(Ni1/2Co1/2)(10) bulk metallic glass (BMG) alloy is achieved using water quenching. The origin of the high GFA was investigated from the kinetic, structural and thermodynamic points of view, and was found to be due to the smaller difference in Gibbs free-energy between the amorphous and crystalline phases in the pseudo-quaternary alloy. These alloys exhibit low glass transition temperatures, below 430 K, and relatively wide supercooled liquid regions of 40-60 K. Mechanical tests on these alloys show a fracture strength of 650 GPa, Vicker's hardness 200 kg mm(-2), Young's modulus 35 GPa, shear modulus 13 GPa and Poisson ratio 0.356. The La-based BMGs are useful for both scientific and engineering applications. (C) 2007 Acta Materialia Inc. Published by Elsevier Ltd. All rights reserved.

SN 1359-6454

EI 1873-2453

PD AUG

PY 2007

VL 55

IS 13

BP 4409

EP 4418

DI 10.1016/j.actamat.2007.04.021

UT WOS:000248436400017

ER

PT J

AU Yu, DC

Geng, Y

Li, ZK

Liu, DM

Fu, HM

Zhu, ZW

Qi, Y

Zhang, HF

AF Yu, Dechuan

Geng, Yan

Li, Zhengkun

Liu, Dingming

Fu, Huameng

Zhu, Zhengwang

Qi, Yang

Zhang, Haifeng

TI A new method locating good glass-forming compositions

SO JOURNAL OF ALLOYS AND COMPOUNDS

AB A new method was proposed to pinpoint the compositions with good glass forming ability (GFA) by combining atomic clusters and mixing entropy. The clusters were confirmed by analyzing competing crystalline phases. The method was applied to the Zr-Al-Ni-Cu-Ag alloy system. A series of glass formers with diameter up to 20 mm were quickly detected in this system. The good glass formers were located only after trying 5 compositions around the calculated composition. The method was also effective in other multi-component systems. This method might provide a new way to understand glass formation and to quickly pinpoint compositions with high GFA. (C) 2015 Elsevier B.V. All rights reserved.

RI qi, yang/H-8625-2016; Zhu, Zheng-Wang/D-2799-2017

OI qi, yang/0000-0003-1915-474X;

SN 0925-8388

EI 1873-4669

PD OCT 15

PY 2015

VL 646

BP 620

EP 625

DI 10.1016/j.jallcom.2015.05.184

UT WOS:000361153700092

ER

PT J

AU Xing, DM

Zhang, TH

Li, WH

Wei, BC

AF Xing, Dongmei

Zhang, Taihua

Li, Weihuo

Wei, Bingchen

TI The characterization of plastic flow in three different bulk metallic

glass systems

SO JOURNAL OF ALLOYS AND COMPOUNDS

AB Plastic deformation behaviors of Zr52.5Al10Ni10Cu15Be12.5, Mg65Cu25Gd10 and Pd43Ni10Cu27P20 bulk metallic glasses (BMGs) are studied by using the depth-sensing nanoindentation, macroindentation and uniaxial compression. The significant difference in plastic deformation behavior cannot be correlated to the Poisson's ratio or the ratio of shear modulus to bulk modulus of the three BMGs, but can be explained by the free volume model. It is shown that the nucleation of local shear band is easy and multiple shear bands can be activated in the Zr52.5Al10Ni10Cu15Be12.5 alloy, which exhibits a distinct plastic strain during uniaxial compression and less serrated flow during nanoindentation. (c) 2006 Elsevier B.V. All rights reserved.

SN 0925-8388

PD MAY 16

PY 2007

VL 433

IS 1-2

BP 318

EP 323

DI 10.1016/j.jallcom.2006.06.077

UT WOS:000245773100060

ER

PT J

AU Aitken, ZH

Jafary-Zadeh, M

Lewandowski, JJ

Zhang, YW

AF Aitken, Zachary H.

Jafary-Zadeh, Mehdi

Lewandowski, John J.

Zhang, Yong-Wei

TI Anharmonic model for the elastic constants of bulk metallic glass across

the glass transition

SO PHYSICAL REVIEW B

AB Here we examine the role of anharmonicity in the elastic constants of bulk metallic glasses and develop an anharmonic model to consider the effects of both pressure and temperature across the glass transition. By comparing against reported experimental data and elastic constants obtained from molecular dynamics simulations, we show that the model is able to capture reported elastic constants from cryogenic temperatures through the glass transition and under hydrostatic pressures up to 18 GPa. Microstructural indicators based on short-range order analysis also display strong correlations with the bulk and shear moduli across the range of pressures and temperatures studied. These results not only greatly expand our understanding in the physical origins of elastic properties of bulk metallic glasses but are also of practical interest for application to processing routes for bulk metallic glass materials.

OI Zhang, Yong-Wei/0000-0001-7255-1678

SN 2469-9950

EI 2469-9969

PD JAN 9

PY 2018

VL 97

IS 1

AR 014101

DI 10.1103/PhysRevB.97.014101

UT WOS:000419613800001

ER

PT J

AU Matsuura, M

Fujita, T

Kawashima, A

Zeng, YQ

Kimura, H

Guan, PF

Chen, MW

Inoue, A

Konno, K

Asada, K

AF Matsuura, Makoto

Fujita, Takeshi

Kawashima, Asahi

Zeng Yuqiao

Kimura, Hisamichi

Guan, Penfei

Chen, Mingwei

Inoue, Akihisa

Konno, Kazuya

Asada, Kaku

TI Local atomic structure of Ni60Pd20P20 and Ni60Pd20P17B3 bulk metallic

glasses and the origin of glass forming ability

SO JOURNAL OF ALLOYS AND COMPOUNDS

AB Ni60Pd20P17B3 alloy has high glass forming ability (GFA) and forms bulk metallic glass (BMG) which exhibits good mechanical properties of a high strength and a large plasticity. Furthermore the plastic strain of the Ni60Pd20P17B3 BMG increases with lowering temperature by 3.4 times from 295 K to 77K. In order to know the origin of such mechanical properties and reasons of high glass formability of this alloy from an atomic scale point of view, XAFS and X-ray diffraction measurements for Ni60Pd20P20 and Ni60Pd20P17B3 metallic glasses have been done using the large scale synchrotron radiation. XAFS results of the Ni and Pd K-edge for the Ni60Pd20P20 metallic glass can be well represented by the Pd4Se-type structure model. A result of interatomic distances agrees with those of the ab initio calculations. Temperature dependence of the Debye-Waller factor reflects strong Ni-P and Pd-P bondings and a large thermal fluctuation of metal-metal bondings. A high GFA of the Ni60Pd20P17B3 alloy compared with Ni60Pd20P20 is discussed based on the XAFS and total pair distribution results. (C) 2010 Elsevier B.V. All rights reserved.

RI Fujita, Takeshi/B-1867-2009; CHEN, Mingwei/A-4855-2010; Kimura,

Hisamichi/D-5449-2012; Inoue, Akihisa/E-5271-2015; Guan,

Pengfei/B-7653-2013

OI Fujita, Takeshi/0000-0002-2318-0433; CHEN, Mingwei/0000-0002-8274-3099;

Chen, Mingwei/0000-0002-2850-8872

SN 0925-8388

PD APR 30

PY 2010

VL 496

IS 1-2

BP 135

EP 139

DI 10.1016/j.jallcom.2010.02.038

UT WOS:000278744500034

ER

PT J

AU Petersen, AS

Cheung, AM

Neilson, HJ

Poon, SJ

Shiflet, GJ

Lewandowski, JJ

AF Petersen, Alexander. S.

Cheung, Andrew. M.

Neilson, Henry. J.

Poon, S. Joseph.

Shiflet, Gary. J.

Lewandowski, John. J.

TI Processing and Properties of Ni-Based Bulk Metallic Glass via Spark

Plasma Sintering of Pulverized Amorphous Ribbons

SO MRS ADVANCES

AB Ni-based bulk metallic glasses and composites with high absolute densities exceeding 11 g/cm(3) were synthesized via spark plasma sintering of Ni45Co10Ta25Nb20 powders produced from pulverized, melt-spun amorphous ribbons. Optimizing the synthesis via selection of sintering temperature, uniaxial load pressure, and powder mechanical screening yielded samples with relative densities of nearly 100% and hardness values in excess of 12.5 GPa without cracking. Mechanical testing included Weibull modulus determination for hardness and compression testing at 10(-3) s(-1) and 10(3) s(-1) strain rates. The capability of using spark plasma sintering to fabricate high hardness, high density, large scale metallic glasses is demonstrated. The mechanical properties of these compacted comminuted melt-spun glass ribbons are presented.

SN 2059-8521

PY 2017

VL 2

IS 61

BP 3815

EP 3820

DI 10.1557/adv.2017.605

UT WOS:000427708700008

ER

PT J

AU Venkatesh, V

Gouthama

Mondal, K

AF Venkatesh, V.

Gouthama

Mondal, K.

TI Effect of cast temperature, size and annealing condition on the serrated

flow during nano-indentation of Zr-based bulk metallic glasses

SO JOURNAL OF ALLOYS AND COMPOUNDS

AB The present work deals with mechanical properties of five multicomponent Zr-based bulk metallic glasses: Zr58Cu22Fe2Ag6Al12, Zr58Cu22Fe4Ag4Al12, Zr58Cu22Fe4Co4Al12, Zr58Cu22Fe8Al12, and Zr58Cu22Ag8Al12, using nano-indentation technique at very low loading rate (80 mu N/s). Zr-based glasses exhibit serrated flow during nano-indentation, manifested as a stepped load- displacement curve. Systematic study on the influence of free volume and nanocrystallization on shear band formation by varying the casting temperature and cross-section of the Zr58Cu22Fe2Ag6Al12, and annealing above and below glass transition temperature of the Zr58Cu22Co4Fe4Al12 alloy is carried out. Analysis of the experimental data has revealed that higher casting temperature and larger casting cross-section cause partial crystallization, which decreases the hardness by promoting multiple shear band formation. However, hardness of the annealed glasses with similar fraction of nanocrystalline phases increases due to the reduction of free volumes as confirmed through electron diffraction studies in transmission electron microscope. Moreover, both free volume and nanocrystalline phase are found to have significant influence on the mechanical properties of bulk metallic glasses investigated in the present study. (C) 2016 Elsevier B.V. All rights reserved.

SN 0925-8388

EI 1873-4669

PD JAN 25

PY 2017

VL 692

BP 745

EP 757

DI 10.1016/j.jallcom.2016.09.033

UT WOS:000386231200099

ER

PT J

AU Wu, WF

Yao, KF

AF Wu, WF

Yao, KF

TI The progress in research of nano-crystallization of amorphous alloys

SO RARE METAL MATERIALS AND ENGINEERING

AB Nanocrystalline materials are the most attractive materials in the field of materials science and engineering in the world. Nano-crystallization of metallic glasses is an effective way to prepare nanocrystalline materials. Here main nano-crystallization methods, including thermal crystallization, electrical crystallization, mechanical crystallization and crystallization under pressure etc. Their principle, problems associated as well as recent technological development are reviewed. The research progress in Tsinghua university in nano-crystallization of Bulk Metallic Glasses(BMG) treated by high density pulse current is simply reported.

SN 1002-185X

PD APR

PY 2005

VL 34

IS 4

BP 505

EP 509

UT WOS:000228948100001

ER

PT J

AU Bakai, AS

Bakai, SA

Neklyudov, IM

Stoev, PJ

Eckert, J

Macht, MP

AF Bakai, A. S.

Bakai, S. A.

Neklyudov, I. M.

Stoev, P. J.

Eckert, J.

Macht, M.-P.

TI On the Kaiser effect in bulk metallic glasses

SO JOURNAL OF NON-CRYSTALLINE SOLIDS

CT 12th International Conference on Liquid and Amorphous Metals (LAM12)

CY JUL 11-16, 2004

CL Metz, FRANCE

AB The Kaiser effect appears during the testing of samples in the repetitive loading and unloading regimes and is found in the fact that the acoustic emission (AE) originates in each subsequent loading only when the stress exceeds its maximum value achieved in the preceding loading. We observed the Kaiser effect in measurements of AE during uniaxial compression of samples of the bulk metallic glasses Zr41Ti14Cu12.5Ni10Be22.5 and Zr52.5Ti5Cu17.9Ni14.6Al10 at room temperature. It is shown that the Kaiser effect recovers after annealing below the glass transition temperature. Mechanisms of structure reconstructions providing the Kaiser effect and its recovery are considered within the framework of polycluster model. (c) 2007 Elsevier B.V. All rights reserved.

OI Stoev, Petr/0000-0001-7942-5850

SN 0022-3093

PD OCT 15

PY 2007

VL 353

IS 32-40

BP 3769

EP 3771

DI 10.1016/j.jnoncrysol.2007.05.145

UT WOS:000250235200155

ER

PT J

AU Wang, H

Fu, HM

Zhang, HF

Hu, ZQ

AF Wang, H.

Fu, H. M.

Zhang, H. F.

Hu, Z. Q.

TI A practical thermodynamic method to calculate the best glass-forming

composition for bulk metallic glasses

SO INTERNATIONAL JOURNAL OF NONLINEAR SCIENCES AND NUMERICAL SIMULATION

AB Bulk metallic glasses are regarded as a new class of engineering materials because of their extraordinary high strength, great elasticity, and high corrosion and wear resistance. The selection of good glass-forming composition is the most important issue in BMG development. In this paper, a thermodynamic method, which combines element substitution and mixing enthalpy calculation, has been successfully developed to predict the optimum glass-forming compositions in Cu-Zr-Ti, Cu-Hf-Ti, Cu-Zr-Hf-Ti and Pd-Ni-P alloy systems. Alloy compositions with the largest glass-forming ability are always accompanied with the smallest enthalpy value for the alloy systems. The prediction results have been confirmed by experiments. It is concluded that the thermodynamic method provides a practical and effective tool to find the optimum glass forming composition.

SN 1565-1339

PY 2007

VL 8

IS 2

BP 171

EP 178

UT WOS:000246429300006

ER

PT J

AU Poon, SJ

Shiflet, GJ

Guo, FQ

Ponnambalam, V

AF Poon, SJ

Shiflet, GJ

Guo, FQ

Ponnambalam, V

TI Glass formability of ferrous- and aluminum-based structural metallic

alloys

SO JOURNAL OF NON-CRYSTALLINE SOLIDS

CT Annual Meeting of The-Minerals-Metals-and-Materials-Society

CY FEB 17-21, 2002

CL SEATTLE, WASHINGTON

SP Minerals Met & Mat Soc, Extract & Proc Div

AB Synthesis of ferrous- and aluminum-based amorphous metals as prospective structural materials is presented and discussed in light of atomic size-composition interaction effects. The search of prospective bulk metallic glasses (BMGs) may benefit from noting that current BMG alloys can be broadly categorized into two atom size-composition classes, distinctly different from ordinary metallic glasses which can exist over a much wider atom size-composition range. The high formability of one class of BMGs is suggested to be due to the presence of a structure-reinforced network or backbone formed by tightly bound components in the undercooled liquid. For the ferrous-based BM, G alloys investigated, it is proposed that zirconium-boron and molybdenum-carbon atom pairs constitute the strong backbone structures. Although aluminum-based BMG has not been reported, the good formability of some current aluminum-glasses is suggested to be due to the presence of backbone. structures formed by transition metal-lanthanide and magnesium-copper pairs. The ferrous-based bulk metallic glasses obtained have a high reduced glass transition, temperature reaching 0.63 and large supercooled liquid region up to 100 K. These bulk metallic glasses are found to be nonferromagnetic above 160 K as well as having Vickers hardness and specific tensile strengths that far exceed those reported for steel alloys. Magnetization and susceptibility results are presented and compared with ab initio magnetic-structure calculations [D.M. Nicholson, M. Widom, Y. Wang, unpublished results]. Relevant factors on forming bulk metallic glasses are discussed. (C) 2003 Elsevier Science B.V. All rights reserved.

SN 0022-3093

PD MAR

PY 2003

VL 317

IS 1-2

BP 1

EP 9

DI 10.1016/S0022-3093(02)02000-8

UT WOS:000181251300002

ER

PT J

AU Zhou, X

Kou, HC

Wang, J

Li, JS

Zhou, L

AF Zhou, X.

Kou, H. C.

Wang, J.

Li, J. S.

Zhou, L.

TI Enthalpy recovery and its effect on homogeneous flow stress during

supercooled liquid region for Ti40Zr25Ni8Cu9Be18 bulk metallic glass

SO JOURNAL OF NON-CRYSTALLINE SOLIDS

AB The homogeneous flow exhibits strain softening for Ti40Zr25Ni8Cu9Be18 bulk metallic glass, which is related to increase in free volume concentration based on free volume model. When metallic glass is pre-annealed above T-g before deformation, the trend of strain softening becomes slow with pre-annealing time, indicating that the enthalpy recovery contributes to strain softening, because the enthalpy of metallic glass will recover towards equilibrium value above T-g, and leads to increase in free volume concentration. So the strain softening for Ti40Zr25Ni8Cu9Be18 bulk metallic glass is related to enthalpy recovery. (C) 2011 Elsevier B.V. All rights reserved.

RI WANG, Jun/A-1526-2015; WANG, Jun/B-1229-2010

OI WANG, Jun/0000-0001-8101-2967; WANG, Jun/0000-0001-8101-2967; Li,

Jinshan/0000-0002-6894-9760; KOU, Hongchao/0000-0003-4960-9477

SN 0022-3093

PD AUG

PY 2011

VL 357

IS 16-17

BP 3049

EP 3052

DI 10.1016/j.jnoncrysol.2011.05.016

UT WOS:000294038500001

ER

PT J

AU Park, ES

Huh, MY

Kim, HJ

Bae, JC

AF Park, E. S.

Huh, M. Y.

Kim, H. J.

Bae, J. C.

TI High strain rate response of Zr-based bulk metallic glass in supercooled

liquid region

SO INTERMETALLICS

CT 7th International Conference on Bulk-Metallic Glasses

CY NOV 01-05, 2009

CL Busan, SOUTH KOREA

SP Yonsei Univ, Ctr Noncrystalline Mat

AB The stress strain curves of Zr-based bulk metallic glass (BMG) were measured at very high strain rates in the supercooled liquid region (SLR) using a uniaxial compression test. For this purpose, the composite samples consisting of Zr-based metallic glass particles embedded in the crystalline nickel matrix were prepared in order to avoid rapid failure of the BMG alloy. The stress strain curves of the BMG alloy were calculated using the data of composite samples. The strain rate sensitivity m = similar to 0.1 of the present BMG alloy was determined at strain rates ranging from 10(0) to 10(1) s(-1). (C) 2010 Elsevier Ltd. All rights reserved.

SN 0966-9795

PD OCT

PY 2010

VL 18

IS 10

SI SI

BP 1889

EP 1892

DI 10.1016/j.intermet.2010.02.040

UT WOS:000281420700024

ER

PT J

AU Yuan, ZZ

Bao, SL

Lu, Y

Zhang, DP

Yao, L

AF Yuan, Zi-Zhou

Bao, Shi-Lei

Lu, Ye

Zhang, Da-Peng

Yao, Lin

TI A new criterion for evaluating the glass-forming ability of bulk glass

forming alloys

SO JOURNAL OF ALLOYS AND COMPOUNDS

AB Based on the classical theory of nucleation and growth, we propose a dimensionless criterion, beta, expressed by TxTg/(T-1 - T-x)(2), with T, the glass transition temperature, T-x, the onset of crystallization temperature and T-1, the liquidus temperature for evaluating the glass-forming ability (GFA) of bulk metallic glasses (BMGs). A survey of the readily available experimental data associated with the thermal analysis of various BMGs show that the new criterion, beta, is statistically better than other currently used criteria, such as gamma (=T-x/(T-t + T-g)) and T-g (=T-g/T-1), and also better than newly proposed criteria, such as delta (=T-x/(T-1 - T-g)) and phi (=T-rg((T-x - T-g)/T-g)(0.143)) in reflecting the GFA. It is also demonstrated that the beta criterion can be used as a guideline for exploring new bulk metallic glass formers in a wide range of BMGs. (c) 2007 Elsevier B.V. All rights reserved.

SN 0925-8388

PD JUL 14

PY 2008

VL 459

IS 1-2

BP 251

EP 260

DI 10.1016/j.jallcom.2007.05.037

UT WOS:000257006300052

ER

PT J

AU Xu, M

Sun, Y

Quan, MX

Wang, YD

Zuo, L

AF Xu Min

Sun Yu

Quan Mingxiu

Wang Yandong

Zuo Liang

TI Glass-forming ability and soft magnetic properties of Fe-Co-Nd-Nb-B

amorphous alloys

SO ACTA METALLURGICA SINICA

AB By applying the gamma criterion the maximum glass-forming ability (GFA) of Fe67Co10-xNd3B20 (x=0, 2, 4, 6, 10) amorphous alloys was recognized when x=6, while the supercooled liquid region was extended to 87 K and the thermo-stability was improved. The tested Fe-based amorphous alloys exhibit good soft magnetic properties with relatively high saturation magnetization and low coercive force. The coercive force is lowered and the soft magnetism is improved remarkably by annealing treatment at the temperature below the glass transition temperature. The structure relaxation and the increase of magnetic hyperfine fields may cause the enhancement of the soft magnetic properties after annealing.

RI wang, yandong/G-9404-2013; Sun, Yu/C-4915-2017

OI Sun, Yu/0000-0002-2136-4466

SN 0412-1961

PD JUL

PY 2007

VL 43

IS 7

BP 699

EP 704

UT WOS:000248548200006

ER

PT J

AU Wang, WH

AF Wang, W. H.

TI Bulk Metallic Glasses with Functional Physical Properties

SO ADVANCED MATERIALS

AB In this review, we report on the formation of a variety of novel, metallic, glassy materials that might well have applications as functional materials. The metallic glasses with excellent glass-forming ability, display many fascinating properties and features such as excellent wave-absoption ability, exceptionally low glass-transition temperatures (similar to 35-60 degrees C) approaching room temperature, ultralow elastic moduli comparable to that of human bone, high elasticity and high strength,superplasticity and poly-mer-like thermoplastic formability near room temperature an excellent magnetocaloric effect, hard magnetism and tunable magnetic properties, heavy-fermion behavior, superhydrophobicity and superoleophobicity, and polyamorphism, all of which are of interest not only for basic research but also for technological applications. A strategy based on elastic moduli correlations for fabrication of bulk metallic, glasses (BMCs) with controllable properties is presented. The work has implications in the search for novel metallic glasses with unique functional properties, for ad g our understanding of the nature and formation of glasses, and for extending the applications of the materials.

SN 0935-9648

EI 1521-4095

PD DEC 4

PY 2009

VL 21

IS 45

SI SI

BP 4524

EP 4544

DI 10.1002/adma.200901053

UT WOS:000272916800002

ER

PT J

AU Zeng, YQ

Nishiyama, N

Inoue, A

AF Zeng Yuqiao

Nishiyama, Nobuyuki

Inoue, Akihisa

TI Development of Ni-Pd-P-B Bulk Metallic Glasses with High Glass-Forming

Ability

SO MATERIALS TRANSACTIONS

CT IUMRS International Conference in Asia

CY DEC 10-12, 2008

CL Nagoya, JAPAN

SP IUMRS

AB A small amount of boron was added to Ni60Pd20P20 and Ni65Pd15P20 alloys. The alloys containing 3 at% boron showed improved thermal stability and glass-forming ability. The supercooled liquid region of as-spun ribbons was enlarged by about 10 K for both Ni60Pd20P17B3 and Ni65Pd15P17B3 alloys. The critical diameters for glass formation were increased up to 15 mm for Ni60Pd20P17B3 and 10 mm for Ni65Pd15P17B3, respectively. The reason of the minor B addition leading to the significant improvements of thermal stability and glass-forming ability was investigated. [doi:10.2320/matertrans.ME200834]

RI Inoue, Akihisa/E-5271-2015; Nishiyama, Nobuyuki/C-8228-2015

SN 1345-9678

EI 1347-5320

PD JUN

PY 2009

VL 50

IS 6

SI SI

BP 1243

EP 1246

DI 10.2320/matertrans.ME200834

UT WOS:000268615800004

ER

PT J

AU Guan, BR

Shi, XT

Dan, ZH

Xie, GQ

Niinomi, M

Qin, FX

AF Guan, Baoru

Shi, Xuetao

Dan, Zhenhua

Xie, Guoqiang

Niinomi, Mitsuo

Qin, Fengxiang

TI Corrosion behavior, mechanical properties and cell cytotoxity of

Zr-based bulk metallic glasses

SO INTERMETALLICS

AB ZrAlCoNb bulk metallic glasses with different Nb contents were fabricated by copper mold casting. Corrosion behavior, mechanical properties and cell cytotoxity were investigated. The investigated Zr-based bulk metallic glasses exhibit high corrosion resistance due to the enrichment of Zr and Al in the oxide layer. The yield strength of 1975 MPa and a plastic strain of 3.5% for Zr56Al16Co23Nb5 BMG are obtained. The cell viability is improved with increasing of Nb content. (C) 2016 Elsevier Ltd. All rights reserved.

RI Xie, Guoqiang/A-8619-2011

OI Dan, Zhenhua/0000-0002-3026-685X

SN 0966-9795

EI 1879-0216

PD MAY

PY 2016

VL 72

BP 69

EP 75

DI 10.1016/j.intermet.2016.02.001

UT WOS:000372386000010

ER

PT J

AU Qin, FX

Wang, XM

Xie, GQ

Inoue, A

AF Qin, F. X.

Wang, X. M.

Xie, G. Q.

Inoue, A.

TI Distinct plastic strain of Ni-free Ti-Zr-Cu-Pd-Nb bulk metallic glasses

with potential for biomedical applications

SO INTERMETALLICS

AB (Ti40Zr10Cu36Pd14)(100-x)Nb-x (x=1, 3, 5at.%) bulk metallic glasses containing nano-particles exhibited ultrahigh strength and distinct plastic strain. The yield strength exceeding 2050 MPa, Young's modulus of about 80 GPa and the plastic strain of over 6.5% were obtained for 1% and 3% Nb-added alloys due to the suppression of the propagation of shear bands by nano-particles in situ formed in the glassy matrix. The developed Ni-free Ti-based bulk metallic glasses with low Young's modulus and good mechanical properties are promising candidates for application as artificial dental root materials and other biomaterials. (C) 2008 Elsevier Ltd. All rights reserved.

RI BAI, JIE/D-7448-2016; Xie, Guoqiang/A-8619-2011; Qin,

Fengxiang/A-8359-2011; Inoue, Akihisa/E-5271-2015

SN 0966-9795

PD AUG

PY 2008

VL 16

IS 8

BP 1026

EP 1030

DI 10.1016/j.intermet.2008.05.004

UT WOS:000259135000012

ER

PT J

AU He, MK

Zhang, Y

Xia, L

Yu, P

AF He, MengKe

Zhang, Yi

Xia, Lei

Yu, Peng

TI Kinetics and thermal stability of the Ni62Nb38-xTax (x=5, 10, 15, 20 and

25) bulk metallic glasses

SO SCIENCE CHINA-PHYSICS MECHANICS & ASTRONOMY
AB We studied thermal stability and its relationship to the glass-forming ability (GFA) of the Ni62Nb38-xTax (x=5, 10, 15, 20, 25) bulk metallic glasses (BMG) from a kinetic point of view. By fitting the heating-rate dependence of glass transition temperature (T-g(onset)) and crystallization temperatures (T-x(onset) and T-x(Peak)) of the Ni62Nb38-xTax BMG using the Vogel-Fulcher-Tammann (VFT) equation, we obtained the ideal glass transition and crystallization temperatures (T-g(0) and T-x(0)) and the fragility parameter (m), and also constructed continuous heating transition (CHT) diagrams for crystallization of the BMG. The CHT diagrams of the BMG indicate enhanced thermal stability by Ta addition; the T-g(0) as well as the T-x(0) also illustrates this improved stability limit. The compositional dependence of m, which agrees well with that of the reduced glass-transition temperature, indicates a strong correlation between liquid fragility and glass-forming ability in the present alloy system. These results provide new evidence for understanding thermal stability, liquid fragility, and GFA in BMG.
OI Xia, Lei/0000-0001-9198-1497
SN 1674-7348
EI 1869-1927
PD JUL
PY 2017
VL 60
IS 7
AR 076111
DI 10.1007/s11433-017-9030-4
UT WOS:000402890500003
ER

PT J
AU Huang, HG
   Xu, HY
   Zhang, PG
   Wang, YM
   Ke, HB
   Zhang, P
   Liu, TW
AF Huang Huogen
   Xu Hongyang
   Zhang Pengguo
   Wang Yingmin
   Ke Haibo
   Zhang Pei
   Liu Tianwei
TI U-Cr Binary Alloys with Anomalous Glass-Forming Ability
SO ACTA METALLURGICA SINICA
AB As for actinide metallic glasses, a minor branch of metallic glasses, almost all of them are binary alloys and their glass-forming rule has been insufficiently studied. Considering that binary alloy systems are the base of ternary or more component glass systems that possess better glass-forming ability, binary glass systems U-Fe, U-Co and U-Cr are chosen for study. After earlier investigation on the first two systems, the glass formation in U-Cr system is explored in this work. According to the eutectic criterion, a series of U-Cr alloys were designed at the Cr-side of the eutectic point U81Cr19. Under the preparation of melt-spinning, these alloys can be formed into a single amorphous phase with the capacity, of crystallizing at about 700 K. The reduced crystallization temperature (T-rx) of some U-Cr alloys exceeded 0.6, higher than those of U-Fe and U-Co metallic glasses, and comparable to those of ordinary bulk amorphous al-loys. Being inconsistent with the prediction based on thermodynamics, kinetics and efficient structural packing, U-Cr alloy system shows anomalous strong glass-forming ability among reported actinide binary glasses. This abnormal behavior might be related to the existence of comparatively more mediate metastable phases in U-Cr system, which can be speculated from the multi-peak crystallization phenomenon. This system could be a potential system model for studying the glass formation of actinide amorphous al-loys further.
SN 0412-1961
PD FEB
PY 2017
VL 53
IS 2
BP 233
EP 238
DI 10.11900/0412.1961.2016.00275
UT WOS:000395966800012
ER

PT J
AU Hsueh, CH
   Bei, H
   Liu, CT
   Becher, PF
   George, EP
AF Hsueh, C. H.
   Bei, H.
   Liu, C. T.
   Becher, P. F.
   George, E. P.
TI Shear fracture of bulk metallic glasses with controlled applied normal
   stresses
SO SCRIPTA MATERIALIA
AB The Mohr-Coulomb criterion has been invoked to characterize the dependence of shear fracture of bulk metallic glasses on the stress normal to the shear plane. However, systematic verification of its applicability is lacking. We use a unique test fixture that is capable of controlling (i) the location of shear fracture and (ii) the ratio of the normal to shear stress on the fracture plane to perform tests on bulk metallic glasses to examine the applicability of the Mohr-Coulomb criterion. (C) 2008 Acta Materialia Inc. Published by Elsevier Ltd. All rights reserved.
RI Hsueh, Chun-Hway/G-1345-2011; George, Easo/L-5434-2014
OI Liu, Chain Tsuan/0000-0001-7888-9725; Bei, Hongbin/0000-0003-0283-7990
SN 1359-6462
PD JUL
PY 2008
VL 59
IS 1
BP 111
EP 114
DI 10.1016/j.scriptamat.2008.02.042
UT WOS:000256121200028
ER

PT J
AU Hojati-Talemi, P
   Gibson, MA
   East, D
   Simon, GP
AF Hojati-Talemi, Pejman
   Gibson, Mark A.
   East, Daniel
   Simon, George P.
TI High performance bulk metallic glass/carbon nanotube composite cathodes
   for electron field emission
SO APPLIED PHYSICS LETTERS
AB We report the preparation of new nanocomposites based on a combination of bulk metallic glass and carbon nanotubes for electron field emission applications. The use of bulk metallic glass as the matrix ensures high electrical and thermal conductivity, high thermal stability, and ease of processing, whilst the well dispersed carbon nanotubes act as highly efficient electron emitters. These advantages, alongside excellent electron emission properties, make these composites one of the best reported options for electron emission applications to date. VC 2011 American Institute of Physics. [doi: 10.1063/1.3659898]
RI Hojati Talemi, Pejman/D-6831-2011
OI Hojati Talemi, Pejman/0000-0001-5673-8491; Simon,
   George/0000-0001-8832-1164
SN 0003-6951
PD NOV 7
PY 2011
VL 99
IS 19
AR 194104
DI 10.1063/1.3659898
UT WOS:000297030200084
ER

PT J
AU Li, B
   Li, YH
   Yang, K
   Li, JS
   Fan, XH
AF Li Bing
   Li Yanhong
   Yang Ke
   Li Jinshan
   Fan Xinhui
TI Effect of yttrium addition on the non-isothermal crystallization
   kinetics and fragility of Cu-Zr-Al bulk metallic glass
SO THERMOCHIMICA ACTA
AB Bulk metallic glasses with the composition of Cu(43)Zr(48)A(19) and (Cu(43)Zr(48)A(19))(98)Y-2 were prepared by copper mold casting technique in this paper. X-ray diffraction (XRD) and differential scanning calorimetry (DSC) were used to investigate their structure and non-isothermal crystallization kinetics. The results show that the influence of heating rate on crystallization is larger than that on glass transition. The crystallization process of (Cu(43)Zr(48)A(19))(98)Y-2 is more sensitive to heating rate than that of Cu(43)Zr(48)A(19). Due to Yttrium addition, the glass transition and nucleation process for crystallization become easier, while the grain growth process for crystallization become more difficult based on the activation energies. The Avrami exponents of (Cu(43)Zr(48)A(19))(98)Y-2 decrease at low heating rates, which is totally different from that of Cu(43)Zr(48)A(19). These two bulk metallic glasses should be considered as "Intermediately fragile glass formers" according to their fragility indices. (C) 2016 Published by Elsevier B.V.
SN 0040-6031
EI 1872-762X
PD OCT 20
PY 2016
VL 642
BP 105
EP 110
DI 10.1016/j.tca.2016.08.001
UT WOS:000386403400014
ER

PT J
AU Li, JF
   Zhao, DQ
   Zhang, ML
   Wang, WH
AF Li, J. F.
   Zhao, D. Q.
   Zhang, M. L.
   Wang, W. H.
TI CaLi-based bulk metallic glasses with multiple superior properties
SO APPLIED PHYSICS LETTERS
AB We describe a class of metallic glasses that consists of low- cost components and exhibiting multiple superior properties such as exceptionally low glass transition temperature (similar to 35-60 degrees C) ultralow elastic moduli (similar to 23 GPa) comparable to that of human bones, high elasticity and strength, ultralow density (< 2 g/cm(3)), exceptional thermodynamic and kinetic stability in supercooled liquid state, strong liquid fragility, ultrahigh specific strength and lower electrical resistivity, and polymerlike thermoplastic formability near room temperature. Such metallic glasses could have potential applications and facilitate studies of the nature of glasses. (C) 2008 American Institute of Physics. [DOI: 10.1063/1.2998262]
SN 0003-6951
EI 1077-3118
PD OCT 27
PY 2008
VL 93
IS 17
AR 171907
DI 10.1063/1.2998262
UT WOS:000260571800022
ER

PT J
AU Rouxel, T
   Yoshida, S
AF Rouxel, Tanguy
   Yoshida, Satoshi
TI The fracture toughness of inorganic glasses
SO JOURNAL OF THE AMERICAN CERAMIC SOCIETY
AB Measuring the fracture toughness (K-Ic) of glasses still remains a difficult task, raising experimental and theoretical problems as well. The available methods to estimate K-Ic are reviewed, with emphasis on their respective advantages and drawbacks. In view of our current understanding, this analysis gives precedence to the SEPB method. The ultimate glass strength, the critical flaw size, and the indentation load for the onset of crack initiation are discussed, in the light of the fundamentals of fracture mechanics and classical background regarding the mechanics of brittle materials. Analytical expressions were further proposed to predict the fracture energy and fracture toughness of glasses from different chemical systems from their nominal compositions. The theoretical values were compared with the experimental ones, as obtained by self-consistent methods when available. The agreement observed in most cases suggests that measured K-Ic values correspond to the crack propagation regime (as opposed to the crack initiation threshold), and supports previous investigations in glasses and ceramics, which showed that a crack tip is nearly atomically sharp in these materials (but for metallic glasses). Some ideas to design tougher glasses are finally presented.
SN 0002-7820
EI 1551-2916
PD OCT
PY 2017
VL 100
IS 10
BP 4374
EP 4396
DI 10.1111/jace.15108
UT WOS:000414922900002
ER

PT J
AU Schuster, BE
   Wei, Q
   Hufnagel, TC
   Ramesh, KT
AF Schuster, B. E.
   Wei, Q.
   Hufnagel, T. C.
   Ramesh, K. T.
TI Size-independent strength and deformation mode in compression of a
   Pd-based metallic glass
SO ACTA MATERIALIA
AB We present quasi-static, room temperature compression data for Pd40Ni40P20 metallic glasses, with specimen sizes ranging from the submicron to several millimeters in diameter. We observe no change in deformation mode over this range. At all sizes, plastic flow is localized in shear bands, which are accompanied by sudden strain bursts. This metallic glass shows only a modest increase in strength in going from bulk to micrometer-sized specimens. We show that stress gradients in tapered specimens can complicate measurement of the yield strength of metallic glasses in microcompression. Estimates of yield strength based on the minimum cross-sectional area implicitly assume that yielding is controlled by a maximum effective shear stress criterion. An alternative is the shear plane yield criterion, in which the minimum shear stress on the shear band trajectory determines yield. Application of this criterion in tapered microspecimens reinforces the notion that metallic glasses possess relatively size-independent mechanical properties. Published by Elsevier Ltd on behalf of Acta Materialia Inc.
RI Hasan, Molla/M-2465-2016; Wei, Qiuming/B-7579-2008; Hufnagel,
   Todd/A-3309-2010
OI Hasan, Molla/0000-0003-2409-7751; Hufnagel, Todd/0000-0002-6373-9377
SN 1359-6454
PD OCT
PY 2008
VL 56
IS 18
BP 5091
EP 5100
DI 10.1016/j.actamat.2008.06.028
UT WOS:000260704100015
ER

PT J
AU Mitrofanov, YP
   Wang, DP
   Wang, WH
   Khonik, VA
AF Mitrofanov, Yu. P.
   Wang, D. P.
   Wang, W. H.
   Khonik, V. A.
TI Interrelationship between heat release and shear modulus change due to
   structural relaxation of bulk metallic glasses
SO JOURNAL OF ALLOYS AND COMPOUNDS
AB Within the framework of the Interstitialcy theory it is shown that the amount of heat released during structural relaxation of metallic glasses must be proportional to the room-temperature change of the shear modulus. This prediction is verified by using experimental data taken on seven bulk metallic glasses. It is found that the kinetics of exothermal heat flow upon structural relaxation is determined by shear modulus relaxation. The released heat per one kinetic relaxation unit ("defect") for all glasses practically coincides with the "defect" formation enthalpy calculated using the Interstitialcy theory providing further support for the description of structural relaxation on the basis of this approach. (C) 2016 Elsevier B.V. All rights reserved.
RI Khonik, Vitaly/A-5888-2009; Mitrofanov, Yuriy/E-7963-2010
OI Mitrofanov, Yuriy/0000-0002-7939-5230
SN 0925-8388
EI 1873-4669
PD AUG 25
PY 2016
VL 677
BP 80
EP 86
DI 10.1016/j.jallcom.2016.03.217
UT WOS:000375481100012
ER

PT J
AU Shen, YT
   Xing, LQ
   Kelton, KF
AF Shen, YT
   Xing, LQ
   Kelton, KF
TI Formation and crystallization of ZrCuTi metallic glasses
SO PHILOSOPHICAL MAGAZINE
AB We report the suction casting of Zr65Cu27Ti8 bulk metallic glasses with diameters up to 1 mm. While the substitution of Ti for Cu increases the glass forming ability significantly, the onset crystallization temperature and the width of the supercooled liquid region decrease. This decrease is due to the appearance of all icosahedral quasicrystal phase, which crystallises from the glass Lit a lower temperature than the Zr2Cu devitrification product ill the Zr65Cu35 glasses. Based on the results of recent structural studies of undercooled Ti/Zr-based alloys, this likely reflects the formation of icosahedral order in the undercooled liquid, which increases the nucleation barrier for the Zr2Cu phase during the quench, making glass formation easier.
SN 1478-6435
PD NOV 1
PY 2005
VL 85
IS 31
BP 3673
EP 3682
DI 10.1080/14786430500156724
UT WOS:000233537700003
ER

PT J
AU Li, CL
   Wei, YJ
   Shi, XH
AF Li, Congling
   Wei, Yujie
   Shi, Xinghua
TI Reformation Capability of Short-Range Order and Their Medium-Range
   Connections Regulates Deformability of Bulk Metallic Glasses
SO SCIENTIFIC REPORTS
AB Metallic glasses (MGs) typically have high yield strength while low ductility, and the latter is commonly considered as the Achilles' heel of MGs. Elucidate the mechanism for such low ductility becomes the research focus of this field. With molecular level simulations, we show the degree of short-range order (SRO) of atomic structure for brittle Fe-based glass decreases dramatically during the stretch, while mild change occurs in ductile Zr-based glass. The reformation capability for SRO and their medium-range connections is found to be the primary characteristics to differentiate the deformability between the two metallic glasses. We suspect that, in addition to the strength of networks formed by SRO structure, the reformation capability to reform SRO networks also plays the key role in regulating the ductility in metallic glasses. Our study provides important insights into the understanding about the mechanisms accounting for ductility or brittleness of bulk metallic glasses.
RI Wei, Yujie/A-3770-2009; Shi, Xinghua/F-9378-2010
OI Wei, Yujie/0000-0002-3213-7891; Shi, Xinghua/0000-0001-5012-3453
SN 2045-2322
PD JUL 16
PY 2015
VL 5
AR 12177
DI 10.1038/srep12177
UT WOS:000358008100001
PM 26178316
ER

PT J
AU Afonin, GV
   Khonik, SV
   Konchakov, RA
   Kobelev, NP
   Kaloyan, AA
   Khonik, VA
AF Afonin, G. V.
   Khonik, S. V.
   Konchakov, R. A.
   Kobelev, N. P.
   Kaloyan, A. A.
   Khonik, V. A.
TI Internal stresses induced by plastic shear deformation of Zr-(Cu,Ag)-Al
   bulk metallic glasses
SO JOURNAL OF NON-CRYSTALLINE SOLIDS
AB Effective internal shear stress sigma(i) induced by torsional deformation of Zr(46)(Cu(4/5)Ag(1/5))(46)Al(8) and Zr(46)Cu(46)Al(8) bulk metallic glasses different by the glass-forming ability of the maternal melts has been determined by measurements of stress relaxation upon stepwise unloading. It has been found that the ratio sigma(i)/sigma(0) (sigma(0) is the initially applied shear stress) decreases upon increasing the temperature from approximate to 0.8 at T = 450K (T approximate to 0.64 x T(g)) to approximate to 0.08 at T = 638K (T approximate to 0.91 x T(g)) independently of sigma(0) and glass composition. The obtained result is in good agreement with earlier data obtained on ribbon metallic glasses. The origin of deformation-induced internal stresses and their connection with deformation mechanisms of metallic glasses has been briefly discussed. (C) 2011 Elsevier B.V. All rights reserved.
RI Afonin, Gennady/K-1014-2013; Khonik, Vitaly/A-5888-2009; Konchakov,
   Roman/K-4616-2013
OI Afonin, Gennady/0000-0002-7715-5065;
SN 0022-3093
PD JAN 15
PY 2012
VL 358
IS 2
BP 220
EP 223
DI 10.1016/j.jnoncrysol.2011.09.016
UT WOS:000298909400015
ER

PT J
AU Zhao, JX
   Qu, RT
   Wu, FF
   Zhang, ZF
   Shen, BL
   Stoica, M
   Eckert, J
AF Zhao, J. X.
   Qu, R. T.
   Wu, F. F.
   Zhang, Z. F.
   Shen, B. L.
   Stoica, M.
   Eckert, J.
TI Fracture mechanism of some brittle metallic glasses
SO JOURNAL OF APPLIED PHYSICS
AB A systematic study on the fracture surface of brittle Mg-, Fe-, and Co-based metallic glasses under compressive loading is approached and a fracture mechanism is proposed. Experimentally, the metallic glass samples are compressed into many small fragments, displaying an explosion fracture feature. Therefore, an energy equilibrium model is employed to describe the fracture processes of those brittle metallic glasses. Furthermore, some regular nanoscale steps, which were scarcely discovered, are found on the mirror region on their fracture surfaces. It is suggested that such nanoscale steps are associated with the energy distribution in metallic glasses and are created by the shear waves generated by the instability of crack propagation during the explosion rupture processes. Based on the comparison of experimental observations with numerical calculations, we recommend a novel model for interpreting the development of nanoscale steps on the dynamic fracture surfaces of these brittle metallic glasses, which appropriately describes the experimental findings.
RI Qu, Rui Tao/B-9183-2008; Zhang, BMG/C-6151-2014; Zhang,
   Zhefeng/A-9732-2010; zhao, jiaxi/C-6974-2012; Stoica, Mihai/B-7069-2015
OI Qu, Rui Tao/0000-0002-3358-7659;
SN 0021-8979
PD MAY 15
PY 2009
VL 105
IS 10
AR 103519
DI 10.1063/1.3129313
UT WOS:000266500100088
ER

PT J
AU Takeuchi, A
   Chen, N
   Wada, T
   Yokoyama, Y
   Kato, H
   Inoue, A
   Yeh, JW
AF Takeuchi, A.
   Chen, N.
   Wada, T.
   Yokoyama, Y.
   Kato, H.
   Inoue, A.
   Yeh, J. W.
TI Pd20Pt20Cu20Ni20P20 high-entropy alloy as a bulk metallic glass in the
   centimeter
SO INTERMETALLICS
AB A Pd20Pt20Cu20Ni20P20 bulk metallic glass (BMG) with a high-entropy (HE) alloy composition and a maximum diameter of 10 mm was fabricated by fluxed water quenching. The system and composition of the Pd20Pt20Cu20Ni20P20 alloy were determined from a prototype ternary Pd40Ni40P20 BMG in accordance with two strategic alloy designs of (1) HE alloy defined by an equi-atomic alloy with five or more elements and (2) exchangeability of the constituent elements with a similar chemical nature in the periodic table. Pd20Pt20Cu20Ni20P20 HE-BMG had a supercooled liquid range of 65 K and a reduced glass transition temperature of 0.71. Successful formation of Pd20Pt20Cu20Ni20P20 HE-BMG is significant to develop new alloys for HE alloys and BMGs. (C) 2011 Elsevier Ltd. All rights reserved.
RI Kato, Hidemi/B-2492-2015; Takeuchi, Akira/A-3619-2010; Chen,
   Na/A-4120-2010; Inoue, Akihisa/E-5271-2015; Wada, Takeshi/B-2431-2015
OI Takeuchi, Akira/0000-0002-7246-8644;
SN 0966-9795
EI 1879-0216
PD OCT
PY 2011
VL 19
IS 10
BP 1546
EP 1554
DI 10.1016/j.intermet.2011.05.030
UT WOS:000294522400028
ER

PT J
AU Lu, YZ
   Huang, YJ
   Shen, J
   Lu, X
   Qin, ZX
   Zhang, ZH
AF Lu, Yunzhuo
   Huang, Yongjiang
   Shen, Jun
   Lu, Xing
   Qin, Zuoxiang
   Zhang, Zhihua
TI Effect of Co addition on the shear viscosity of Fe-based bulk metallic
   glasses
SO JOURNAL OF NON-CRYSTALLINE SOLIDS
AB In this work, a non-isothermal three-point bending creep test has been proposed for measuring the non-isothermal shear viscosity of Fe-Co-Cr-Mo-C-B-Y bulk metallic glasses (BMGs). The variation of the viscosity induced by Co addition has been discussed from the viewpoints of atomic packing density and metal-metalloid bonds. It has been found that the Co7 BMG alloy with highest viscosity exhibited the densest atomic structure and the strongest covalent-like metal-metalloid bonds, which could suppress the atomic viscous flow and thus dramatically enhance the viscosity. (C) 2014 Elsevier B.V. All rights reserved.
RI Huang, Yongjiang/D-4809-2009
OI Lu, Yunzhuo/0000-0002-7322-6815
SN 0022-3093
EI 1873-4812
PD NOV 1
PY 2014
VL 403
BP 62
EP 66
DI 10.1016/j.jnonaysol.2014.07.012
UT WOS:000342548700010
ER

PT S
AU de Oliveira, MF
   Botta, WJ
   Yavari, AR
AF de Oliveira, MF
   Botta, WJ
   Yavari, AR
BE Schumacher, P
   Warren, P
   Cantor, B
TI Shaping, joining and engraving of bulk metallic glasses using an
   electromechanical process
SO METASTABLE, MECHANICALLY ALLOYED AND NANOCRYSTALLINE MATERIALS,
   ISMANAM-2000
SE Materials Science Forum
CT International Symposium on Metastable, Mechanically Alloyed and
   Nanocrystalline Materials (ISMANAM 2000)
CY JUL 09-14, 2000
CL ST CATHERINES COLL, OXFORD, ENGLAND
SP Zoz GmbH, Fisher Sci Orme Technologies, Oxford Ctr Adv Mat & Composites, Univ Oxford, Dept Mat
HO ST CATHERINES COLL
AB Some intrinsic materials properties of bulk metallic glasses (BMG) such as electrical resistivities two orders of magnitude higher than good conductors and a Newtonian viscous-flow regime of deformability have allowed shaping, joining and engraving of BMGs using a new electromechanical process. The new process exploits the fact that bulk metallic glasses have large liquid supercooled regions between the glass transition temperature T, and the crystallisation temperature T,. In this range, the undercooled liquid in principle deforms in a Newtonian way, allowing thermomechanical shaping in the low viscosity range as applied to oxide glasses. Electromechanical processing technology has been used for rapid shaping at low applied stresses by eliminating the thermal mass of the furnace and the need to heat the deformation dies. The process parameters has been adapted to fully maintain the glassy state or, when desired in appropriate compositions, to induce nanocrystallisation or nano-quasicrystallisation during the joining or shaping operation.
RI de Oliveira, Marcelo/B-9881-2012; yavari, alain/E-8192-2010
OI de Oliveira, Marcelo/0000-0003-4589-2463; Botta,
   Walter/0000-0003-2759-573X
SN 0255-5476
BN 0-87849-865-6
PY 2001
VL 360-3
BP 1
EP 6
DI 10.4028/www.scientific.net/MSF.360-362.1
UT WOS:000168667500001
ER

PT J
AU Revesz, A
   Henits, P
   Kovacs, Z
AF Revesz, Adam
   Henits, Peter
   Kovacs, Zsolt
TI Structural changes in Zr-based bulk metallic glasses deformed by high
   pressure torsion
SO JOURNAL OF ALLOYS AND COMPOUNDS
CT 15th International Symposium on Metastable, Amorphous and Nanostructured
   Materials
CY JUL 06-10, 2008
CL Buenos Aires, ARGENTINA
AB Zr(44)Ti(11)Cu(10)Ni(10)Be(25) and Zr(57)Ti(5)Cu(20)Al(10)Ni(8) bulk metallic glass disks have been subjected to severe plastic deformation by high pressure torsion Structural changes occurred during torsion straining were detected by high intensity synchrotron X-ray diffraction, modulated thermal analysis and hardness measurements. The samples show no evidence of nanocrystallization, however, they posses atomic bond length change in the amorphous state. Simultaneously, a change in the heat capacity of the deformed state is detected which is preserved above the glass transition in the supercooled liquid range (C) 2009 Elsevier B V. All rights reserved
RI Kovacs, Zsolt/A-8091-2010
OI Kovacs, Zsolt/0000-0001-6802-3311
SN 0925-8388
PD APR 16
PY 2010
VL 495
IS 2
BP 338
EP 340
DI 10.1016/j.jallcom.2009.10.175
UT WOS:000278749900013
ER

PT J
AU Luo, Y
   Yang, GN
   Shao, Y
   Yao, KF
AF Luo, Yun
   Yang, Guannan
   Shao, Yang
   Yao, Kefu
TI The effect of void defects on the shear band nucleation of metallic
   glasses
SO INTERMETALLICS
AB Molecular dytaamics simulations were employed to investigate the effect of void defect on the shear band nucleation in a Cu64Zr36 metallic glass. Unlike uniform samples, the samples with void defects can form shear bands at a much lower strain. Finite element simulation revealed stress concentration near the voids, which can explain the early formation of shear bands. The shear band behaviors are also influenced by the size, shape and distribution of the defects. These findings can provide simulation support to understand the shear band heterogeneous nucleation process of metallic glasses in the real case.
SN 0966-9795
EI 1879-0216
PD MAR
PY 2018
VL 94
BP 114
EP 118
DI 10.1016/j.intermet.2017.12.026
UT WOS:000425579200016
ER

PT J
AU Qin, YS
   Shi, Y
   Han, XL
   Song, KK
   Cao, CD
   Li, XL
   Wang, SH
   He, J
   Wang, L
   Kaban, I
   Eckert, J
AF Qin, Y. S.
   Shi, Y.
   Han, X. L.
   Song, K. K.
   Cao, C. D.
   Li, X. L.
   Wang, S. H.
   He, J.
   Wang, L.
   Kaban, I.
   Eckert, J.
TI Formation and phase evolution of liquid phase-separated metallic glasses
   with double glass transition, crystallization and melting
SO MATERIALS TODAY COMMUNICATIONS
AB Zr57CO27Al16 and La57Co27Al16 alloys with excellent glass-forming ability (GFA) were adopted to synthesize new phase-separated bulk metallic glasses (BMGs) upon rapid solidification. (ZrxLa1-x)(57)Co27Al16 (x = 0.2, 0.4, 0.6, and 0.8) melts separate into ZrCo-rich and La-rich liquids upon cooling due to the existence of a miscibility gap in Zr-La liquids. The intrinsic and extrinsic mechanisms for the formation of phase-separated alloys under different casting conditions were analyzed in detail. The GFA of the studied alloys changes in the following order: Zr57Co27Al16 >La57Co27Al15 > Zr11.4La45.6 Co27Al16 > Zr22.8La34.2CO27Al16 > Zr34.2La22.8Co27Al16 >Zr45.6La11.4CO27Al16. The quaternary Zr-La-Co-Al phase-separated metallic glasses (MGs) are shown to experience double glass transition, crystallization and melting events, which could be regarded as possible model materials to study the glassy transition and structural relaxation behavior of BMGs. (C) 2016 Elsevier Ltd. All rights reserved.
RI song, kaikai/G-5908-2011
OI Song, Kaikai/0000-0002-5832-7546
SN 2352-4928
PD SEP
PY 2016
VL 8
BP 64
EP 71
DI 10.1016/j.mtcomm.2016.06.001
UT WOS:000386879900009
ER

PT J
AU Kramer, L
   Kormout, KS
   Setman, D
   Champion, Y
   Pippan, R
AF Kraemer, Lisa
   Kormout, Karoline S.
   Setman, Daria
   Champion, Yannick
   Pippan, Reinhard
TI Production of Bulk Metallic Glasses by Severe Plastic Deformation
SO METALS
AB The aim of this study was to show the possibility to produce bulk metallic glass with severe plastic deformation. High pressure torsion was used to consolidate Zr-based metallic glass powder and deform it further to weld the powder particles together. The produced samples were investigated with Scanning electron microscope (SEM), Transmission electron microscope (TEM), Differential scanning calorimetry (DSC) and X-ray diffraction (XRD) to check if the specimens are fully dense and have an amorphous structure. The results show that the specimens remain amorphous during high pressure torsion and the density depends on the applied strain. Additional Vickers hardness measurements enable a comparison with literature and show for Zr-based metallic glass powder typical values (approximately 500 HV).
SN 2075-4701
PD JUN
PY 2015
VL 5
IS 2
BP 720
EP 729
DI 10.3390/met5020720
UT WOS:000357508400016
ER

PT J
AU Zhou, X
   Kou, HC
   Wang, J
   Li, JS
   Zhou, L
AF Zhou, Xun
   Kou, Hongchao
   Wang, Jun
   Li, Jinshan
   Zhou, Lian
TI Crystallization and compressive behaviors of Ti40Zr25Ni8Cu9Be18 BMG cast
   from different liquid states
SO INTERMETALLICS
AB The initial microstructure of Ti40Zr25Ni8Cu9Be18 metallic glass is sensitive to preparation condition. The Ti40Zr25Ni8Cu9Be18 metallic glasses, prepared at different liquid temperature by copper mold casting, would possess different first crystallization product because of difference in initial microstructure. During first crystallization, Crystallization product is mainly metastable icosahedral phase for metallic glass prepared at relative low liquid temperature. However, the mixture of alpha-Ti + icosahedral + unknown phase precipitates from metallic glass prepared at relative high liquid temperature. Moreover, the difference in initial microstructure also has a significant influence on compressive properties. Ti40Zr25Ni8Cu9Be18 metallic glasses prepared at relative low temperature exhibit better plasticity. One of important reasons is that dominant icosahedral short range order (SRO) increases shear viscosity of shear band, and the possibility of catastrophic failure caused by fast and continuous slide of shear band is depressed. Crown Copyright (C) 2012 Published by Elsevier Ltd. All rights reserved.
RI WANG, Jun/A-1526-2015; WANG, Jun/B-1229-2010
OI WANG, Jun/0000-0001-8101-2967; WANG, Jun/0000-0001-8101-2967; KOU,
   Hongchao/0000-0003-4960-9477; Li, Jinshan/0000-0002-6894-9760
SN 0966-9795
PD SEP
PY 2012
VL 28
BP 45
EP 50
DI 10.1016/j.intermet.2012.03.061
UT WOS:000307038700007
ER

PT J
AU Zumkley, T
   Naundorf, V
   Macht, MP
   Fielitz, P
   Frohberg, G
AF Zumkley, T
   Naundorf, V
   Macht, MP
   Fielitz, P
   Frohberg, G
TI Relation between time and temperature dependence of diffusion and the
   structural state in ZrTiCuNiBe bulk glasses
SO MATERIALS TRANSACTIONS
AB Time and temperature dependence of impurity diffusion is measured in detail for several ZrTiCuNiBe bulk glasses in a wide temperature range. We find that neither the quenched-in free volume nor a small volume fraction of crystals have a significant effect on the diffusivity. It is shown that the composition of these glasses has a much weaker effect on the diffusion coefficients than have structural changes of the glass. By long time relaxation the reversible metastable equilibrium of the glass structure could be reached at temperatures below the calorimetric glass transition. For this metastable equilibrium state a single set of diffusion parameters, activation enthalpy and pre-factor of the diffusion coefficient, is derived in the entire temperature range above and below the glass transition. This indicates that the usually observed non-linear Arrhenius behavior of diffusion coefficients in the ZrTiCuNiBe bulk glasses is caused by insufficient structural relaxation at temperatures below the calorimetric glass transition.
SN 1345-9678
EI 1347-5320
PD AUG
PY 2002
VL 43
IS 8
BP 1921
EP 1930
DI 10.2320/matertrans.43.1921
UT WOS:000177930100025
ER

PT J
AU Pelletier, JM
AF Pelletier, J. M.
TI Influence of structural relaxation on atomic mobility in a
   Zr41.2Ti13.8CU125Ni10.0Be22.5 (Vit1) bulk metallic glass
SO JOURNAL OF NON-CRYSTALLINE SOLIDS
AB The effect of an annealing at a temperature above or below the glass transition temperature in a Zr41.2Ti13.8CU125Ni10.0Be22.5 bulk metallic glass was investigated using dynamic mechanical analysis. Structural relaxation influences both the storage modulus (elastic component) and the loss modulus (viscoelastic component). Kinetics can be captured by a stretched exponential relaxation function. Experimental results are correctly described using a physical model based on the concept of defects for the mechanical response of amorphous materials and especially for the characteristic time relative to atomic mobility. (C) 2008 Elsevier B.V. All rights reserved.
SN 0022-3093
PD AUG 1
PY 2008
VL 354
IS 31
BP 3666
EP 3670
DI 10.1016/j.jnoncrysol.2008.04.017
UT WOS:000258051500002
ER

PT J
AU Lan, S
   Lau, MT
   Kui, HW
AF Lan, S.
   Lau, M. T.
   Kui, H. W.
TI The time constant of the spinodal decomposition in Pd41.75Ni41.75P17.5
   bulk metallic glasses
SO JOURNAL OF NON-CRYSTALLINE SOLIDS
AB Experimental arrangements were made to study the spinodal mechanism occurring in undercooled molten Pd41.75Ni41.75P17.5 alloys as a function of time. It can be estimated from the experimental results that the lower bound of the duration (or completion time) of the spinodal mechanism at a temperature of approximate to 625 K is about 200 s. Simple kinetic analysis shows that the time constant tau of the spinodal decomposition at a temperature of approximate to 625 K is similar to 0.002 s(-1). Both quantities are useful indicators for other metallic glasses and bulk metallic glasses (BMG) that also undergo spinodal decomposition. (C) 2012 Elsevier B.V. All rights reserved.
SN 0022-3093
PD FEB 1
PY 2013
VL 361
BP 1
EP 8
DI 10.1016/j.jnoncrysol.2012.10.032
UT WOS:000315557400001
ER

PT J
AU Zhang, QS
   Qin, CL
   Inoue, A
AF Zhang, Qingsheng
   Qin, Chunling
   Inoue, Akihisa
TI Cast bulk glassy alloys: fabrication, alloy development and properties
SO CHINA FOUNDRY
AB Metallic glasses represent an interesting group of materials as they possess outstanding physical, chemical and mechanical properties compared to their crystalline counterparts. Currently, with well designed compositions it is possible to cast liquid alloys into the glassy state at low critical cooling rates from 100 K.s(-1) to 1 K.s(-1) and in large critical sample sizes up to several centimeters, which significantly enhances the promise for possible applications as advanced engineering materials. This paper reviews the development of (ZrCu)-based bulk metallic glasses with large sizes by copper mold casting and their unique properties. Additionally, the ex-situ and in-situ second phases reinforced BMG composites with large plasticity are also presented.
RI Inoue, Akihisa/E-5271-2015
SN 1672-6421
PD AUG
PY 2010
VL 7
IS 3
BP 278
EP 286
UT WOS:000281560700012
ER

PT J
AU Fan, C
   Wilson, TW
   Dmowski, W
   Choo, H
   Richardson, JW
   Maxey, ER
   Liaw, PK
AF Fan, Cang
   Wilson, T. W.
   Dmowski, W.
   Choo, H.
   Richardson, J. W.
   Maxey, E. R.
   Liaw, P. K.
TI Quenched-in quasicrystal medium-range order and pair distribution
   function study on Zr55Cu35Al10 bulk metallic
SO INTERMETALLICS
CT 4th International Conference on Bulk Metallic Glasses
CY MAY 01-05, 2005
CL Gatlinburg, TN
AB The quasicrystals phase was found in a crystallized Zr55Cu35Al10 bulk metallic glass by X-ray analyses. Pair-distribution-function (PDF) studies on as-cast and partially crystallized states were performed by neutron scattering measurements. The PDF study shows that a medium-range order exists in the as-cast metallic glass. The investigation on the partial crystallization confirms that the local structures of the metallic glass consist of a quenched-in icosahedral medium-range order, which contribute to the quasicrystallization by reheating. (c) 2006 Elsevier Ltd. All rights reserved.
RI Choo, Hahn/A-5494-2009
OI Choo, Hahn/0000-0002-8006-8907
SN 0966-9795
EI 1879-0216
PD AUG-SEP
PY 2006
VL 14
IS 8-9
SI SI
BP 888
EP 892
DI 10.1016/j.intermet.2006.01.005
UT WOS:000237770600007
ER

PT J
AU Yang, WM
   Liu, HS
   Dun, CC
   Li, JW
   Zhao, YC
   Shen, BL
AF Yang, Weiming
   Liu, Haishun
   Dun, Chaochao
   Li, Jiawei
   Zhao, Yucheng
   Shen, Baolong
TI Nearly free electron model to glass-forming ability of multi-component
   metallic glasses
SO JOURNAL OF NON-CRYSTALLINE SOLIDS
AB A nearly free electron model for explaining the glass-forming ability (GFA) of multi-component bulk metallic glasses (BMGs) is proposed. It can be drawn from this model that the density is closely correlated with the GFA of BMGs and the best GFA can be obtained when the densities of states (DOS) at Fermi level for glasses nearly touch one of the crystalline. Moreover, compelling experimental evidence is provided through systematic study of bulk (Fe1-xCox)(71.4)Cu0.6B20Si4Nb4 (x = 0, 0.1, 0.3, 0.5, and 0.7 at.%) BMGs. Our results highlight the significance density effects on the formation of MGs, and elucidate one process by which the glass-forming tendency may be enhanced in MGs. (C) 2012 Elsevier B.V. All rights reserved.
RI Yang, Weiming/H-3951-2013
SN 0022-3093
PD FEB 1
PY 2013
VL 361
BP 82
EP 85
DI 10.1016/j.jnoncrysol.2012.10.026
UT WOS:000315557400013
ER

PT J
AU Zhang, B
   Wang, RJ
   Wang, WH
AF Zhang, B
   Wang, RJ
   Wang, WH
TI Response of acoustic and elastic properties to pressure and
   crystallization of Ce-based bulk metallic glass
SO PHYSICAL REVIEW B
AB The density and acoustic velocities of a Ce70Al10Ni10Cu10 bulk metallic glass (BMG) under hydrostatic-pressure (up to 0.5 GPa) and in crystallized state in ambient conditions were measured in situ by a pulse echo overlap method. The pressure derivatives of velocities and Gruneisen parameters as well as the equation of state (EOS) of the BMG were determined and compared to those of various other BMGs and nonmetallic glasses. Surprisingly, the BMG, unlike other BMGs with normal mode stiffness, exhibits an anomalous soft longitudinal acoustic mode under pressure similar to that of typical oxide glasses. An unusually large softening of longitudinal acoustic phonons in the BMG, relative to its crystalline state, is also observed, analogous with that in oxide glasses. The possible origin for the anomaly is the intrinsic glassy structure containing short-range covalent bonds.
SN 2469-9950
EI 2469-9969
PD SEP
PY 2005
VL 72
IS 10
AR 104205
DI 10.1103/PhysRevB.72.104205
UT WOS:000232228800036
ER

PT J
AU Tang, XP
   Geyer, U
   Busch, R
   Johnson, WL
   Wu, Y
AF Tang, XP
   Geyer, U
   Busch, R
   Johnson, WL
   Wu, Y
TI Diffusion mechanisms in metallic supercooled liquids and glasses
SO NATURE
AB The mechanisms of atomic transport in supercooled liquids and the nature of the glass transition are long-standing problems(1-4) Collective atomic motion is thought to play an important role(4-6) in both phenomena. A metallic supercooled liquid represents an ideal system for studying intrinsic collective motions because of its structural similarity to the "dense random packing of spheres" model(7), which is conceptually simple. Unlike polymeric and network glasses, metallic supercooled liquids have only recently become experimentally accessible, following the discovery of bulk metallic glasses(8-12). Here we report a Be-9 nuclear magnetic resonance study of Zr-based bulk metallic glasses(8,9) in which we investigate microscopic transport in supercooled liquids around the glass transition regime. Combining our results with diffusion measurements, we demonstrate that two distinct processes contribute to long-range transport in the supercooled liquid state: single-atom hopping and collective motion, the latter being the dominant process. The effect of the glass transition is clearly visible in the observed diffusion behaviour of the Be atoms.
SN 0028-0836
PD NOV 11
PY 1999
VL 402
IS 6758
BP 160
EP 162
UT WOS:000083716400042
ER

PT J
AU Naz, GJ
   Dong, DD
   Geng, YX
   Wang, YM
   Dong, C
AF Naz, Gul Jabeen
   Dong, Dandan
   Geng, Yaoxiang
   Wang, Yingmin
   Dong, Chuang
TI Composition formulas of Fe-based transition metals-metalloid bulk
   metallic glasses derived from dual-cluster model of binary eutectics
SO SCIENTIFIC REPORTS
AB It is known that bulk metallic glasses follow simple composition formulas [cluster](glue atom) 1 or 3 with 24 valence electrons within the framework of the cluster-plus-glue-atom model. Though the relevant nearest-neighbor cluster can be readily identified from a devitrification phase, the glue atoms remains poorly defined. The present work is devoted to understanding the composition rule of Fe-(B, P, C) based multi-component bulk metallic glasses, by introducing a cluster-based eutectic liquid model. This model regards a eutectic liquid to be composed of two stable liquids formulated respectively by cluster formulas for ideal metallic glasses from the two eutectic phases. The dual cluster formulas are first established for binary Fe-( B, C, P) eutectics: [Fe-Fe-14] B2Fe + [B-B2Fe8] Fe approximate to Fe83.3B16.7 for eutectic Fe83B17, [P-Fe-14] P + [P-Fe-9] P2Fe approximate to Fe82.8P17.2 for Fe83P17, and [C-Fe-6] Fe-3 + [C-Fe-9] C2Fe approximate to Fe82.6C17.4 for Fe82.7C17.3. The second formulas in these dual-cluster formulas, being respectively relevant to devitrification phases Fe2B, Fe3P, and Fe3C, well explain the compositions of existing Fe-based transition metalsmetalloid bulk metallic glasses. These formulas also satisfy the 24-electron rule. The proposition of the composition formulas for good glass formers, directly from known eutectic points, constitutes a new route towards understanding and eventual designing metallic glasses of high glass forming abilities.
SN 2045-2322
PD AUG 22
PY 2017
VL 7
AR 9150
DI 10.1038/s41598-017-09100-9
UT WOS:000408284900002
PM 28831157
ER

PT S
AU Sidorov, V
   Rojkov, I
   Mikhailov, V
   Svec, P
   Janickovic, D
AF Sidorov, V.
   Rojkov, I.
   Mikhailov, V.
   Svec, P.
   Janickovic, D.
BE Wang, L
   Liu, H
TI The Influence of Ga Additions on Electric and Magnetic Properties of
   Co47Fe21B21Si5Nb6 Alloy in Crystal and Liquid States
SO PROCEEDINGS FOR THE XV LIQUID AND AMORPHOUS METALS (LAM-15)
   INTERNATIONAL CONFERENCE
SE AIP Conference Proceedings
CT 15th International Conference on Liquid and Amorphous Metals (LAM)
CY SEP 15-20, 2013
CL Beijing, PEOPLES R CHINA
SP Harbin InstTechnol, Dalian Univ Technol, Key Lab Mat Modificat Laser, Ion & Electron Beams, Surface & Technol Surface Phys & Chem Lab, Shandong Univ, Key Lab Liquid Solid Struct Evolut & Proc Mat, Univ Sci & Technol Beijing, State Key Lab Adv Met & Mat, Zhejiang Univ, Int Ctr New Struct Mat & Lab New Struct Mat
AB The influence of small additions of gallium on electric resistivity and magnetic susceptibility of the bulk glass forming Co47Fe20.9B21.2Si4.6Nb6.3 alloy was studied in a wide temperature range up to 1830 K. Gallium atoms were found to increase resistivity but decrease susceptibility of the alloy. The suppositions about clusters surrounding Ga atoms in the melt and new GFA criterion are given
SN 0094-243X
BN 978-0-7354-1320-7
PY 2015
VL 1673
AR 020017
DI 10.1063/1.4928271
UT WOS:000368442400017
ER

PT J
AU Zong, HT
   Ma, MZ
   Zhang, XY
   Qi, L
   Li, G
   Jing, Q
   Liu, RP
AF Zong Hai-Tao
   Ma Ming-Zhen
   Zhang Xin-Yu
   Qi Li
   Li Gong
   Jing Qin
   Liu Ri-Ping
TI Formation and Compression Behavior of Two-Phase Bulk Metallic Glasses
   with a Minor Addition of Aluminum
SO CHINESE PHYSICS LETTERS
AB A remarkable enhancement in room-temperature compressive deformability is realized by the minor-addition of 1.5 at.% Al in ZrTi-based bulk metallic glass. Two amorphous phases are observed by transmission electron microscopy in the Al-containing alloys and this explains the improvement of compression deformability. The studies suggest that phase separation might occur in glass forming alloys with a negative enthalpy of mixing.
SN 0256-307X
PD MAR
PY 2011
VL 28
IS 3
AR 036103
DI 10.1088/0256-307X/28/3/036103
UT WOS:000288120900040
ER

PT J
AU Arora, HS
   Aditya, AV
   Mukherjee, S
AF Arora, Harpreet Singh
   Aditya, Ayyagari V.
   Mukherjee, Sundeep
TI Structural relaxation driven increase in elastic modulus for a bulk
   metallic glass
SO JOURNAL OF APPLIED PHYSICS
AB The change in elastic modulus as a function of temperature was investigated for a zirconium-based bulk metallic glass. High temperature nano-indentation was done over a wide temperature range from room temperature to the glass-transition. At higher temperature, there was a transition from inhomogeneous to homogeneous deformation, with a decrease in serrated flow and an increase in creep displacement. Hardness was found to decrease, whereas elastic modulus was found to increase with temperature. The increase in elastic modulus for metallic glass at higher temperature was explained by diffusive rearrangement of atoms resulting in free volume annihilation. This is in contrast to elastic modulus increase with temperature for silicate glasses due to compaction of its open three dimensional coordinated structure without any atomic diffusion. (C) 2015 AIP Publishing LLC.
OI Ayyagari, Venkata Aditya/0000-0002-3111-9819
SN 0021-8979
EI 1089-7550
PD JAN 7
PY 2015
VL 117
IS 1
AR 014902
DI 10.1063/1.4905145
UT WOS:000347958600049
ER

PT J
AU Wang, XD
   Cao, QP
   Jiang, JZ
   Franz, H
   Schroers, J
   Valiev, RZ
   Ivanisenko, Y
   Gleiter, H
   Fecht, HJ
AF Wang, X. D.
   Cao, Q. P.
   Jiang, J. Z.
   Franz, H.
   Schroers, J.
   Valiev, R. Z.
   Ivanisenko, Y.
   Gleiter, H.
   Fecht, H-J
TI Atomic-level structural modifications induced by severe plastic shear
   deformation in bulk metallic glasses
SO SCRIPTA MATERIALIA
AB Structural evolution of an Au-based bulk metallic glass (BMG) after severe plastic deformation (SPD) was investigated The newly formed glass contains high-density shear bands, a reduced ordering and a concomitant excess free volume Moreover, it exhibits a less changed local structure even in the supercooled liquid region, but a reduced thermal stability reflected in an accelerated crystal nucleation and growth process These results suggest that SPD modifies the atomic structure of BMGs by localized shear band formation, thus producing so-called nanoglasses (C) 2010 Acta Materialia Inc Published by Elsevier Ltd All rights reserved
RI Ivanisenko, Yulia/F-9479-2010
SN 1359-6462
PD JAN
PY 2011
VL 64
IS 1
BP 81
EP 84
DI 10.1016/j.scriptamat.2010.09.015
UT WOS:000284453300021
ER

PT J
AU Fatay, D
   Gubicza, J
   Szommer, P
   Lendvai, J
   Bletry, M
   Guyot, P
AF Fatay, D
   Gubicza, J
   Szommer, P
   Lendvai, J
   Bletry, M
   Guyot, P
TI Thermal stability and mechanical properties of a Zr-based bulk amorphous
   alloy
SO MATERIALS SCIENCE AND ENGINEERING A-STRUCTURAL MATERIALS PROPERTIES
   MICROSTRUCTURE AND PROCESSING
CT 13th International Conference on the Strength of Materials (ICSMA 13)
CY AUG, 2003
CL Budapest, HUNGARY
SP Eotvos Univ Budapest, Minist Educ Republic Hungary, Naval Res Int Field-Office, USN Office
AB The thermal and mechanical properties of a Zr-Al-Cu-Ti-Ni bulk metallic glass were investigated. The glass transition and the crystallization were studied by calorimetry and X-ray diffraction. It was found that the crystallization occurred in two steps. The precipitating phases, and the activation energies were determined. It was established that the crystallization was controlled by the diffusion of the alloying Cu and Ni atoms. The creep behavior was investigated by indentation tests. The viscosity and the activation energy of the deformation process determined from indentation were in reasonable agreement with those obtained by compression tests. (C) 2004 Elsevier B.V. All rights reserved.
RI Szommer, Peter/I-1592-2017; Lendvai, Janos/J-4445-2013
OI Szommer, Peter/0000-0002-0737-3788; Bletry, Marc/0000-0003-2509-9847;
   Gubicza, Jeno/0000-0002-8938-7293
SN 0921-5093
EI 1873-4936
PD DEC 15
PY 2004
VL 387
BP 1001
EP 1004
DI 10.1016/j.msea.2004.01.088
UT WOS:000226042400205
ER

PT S
AU Kim, CP
   Szuecs, F
   Johnson, WL
AF Kim, CP
   Szuecs, F
   Johnson, WL
BE Schumacher, P
   Warren, P
   Cantor, B
TI Microstructure and mechanical properties of ductile phase containing
   bulk metallic glass composites
SO METASTABLE, MECHANICALLY ALLOYED AND NANOCRYSTALLINE MATERIALS,
   ISMANAM-2000
SE Materials Science Forum
CT International Symposium on Metastable, Mechanically Alloyed and
   Nanocrystalline Materials (ISMANAM 2000)
CY JUL 09-14, 2000
CL ST CATHERINES COLL, OXFORD, ENGLAND
SP Zoz GmbH, Fisher Sci Orme Technologies, Oxford Ctr Adv Mat & Composites, Univ Oxford, Dept Mat
HO ST CATHERINES COLL
AB Ductile phase containing bulk metallic glass composites are prepared and investigated by SEM, X-ray diffraction, microprobe, and demonstrate improved mechanical properties in compression, tensile, and Charpy impact tests. The remarkable glass forming ability of bulk glass forming compositions in Zr-Ti-Cu-Ni-Be system allows for the preparation of ductile metal reinforced composites with a bulk metallic glass matrix via in-situ processing. Primary dendrite growth accompanied by solute partitioning in the molten state yields an equilibrium microstructure containing of a ductile crystalline phase with bcc-structure in a metallic glass matrix with varying volume fractions of bcc-phase and varying scales of the microstructure. The incorporation of a ductile metal phase into a metallic glass matrix yields a constraint that allows for the generation of multiple shear bands and stabilizes crack growth in the matrix. These results increase global plasticity of metallic glass under unconstrained loading condition and increase toughness, impact resistance, and resistance to crack propagation. Sub-standard Charpy specimens prepared from one of these composites have demonstrated impact toughness 300% greater than that of metallic glass matrix alone. Also, the strain to failure in tension is about 5% and 12% in compression.
SN 0255-5476
BN 0-87849-865-6
PY 2001
VL 360-3
BP 49
EP 54
DI 10.4028/www.scientific.net/MSF.360-362.49
UT WOS:000168667500008
ER

PT J
AU Sokolov, AP
   Novikov, VN
   Kisliuk, A
AF Sokolov, A. P.
   Novikov, V. N.
   Kisliuk, A.
TI Fragility and mechanical moduli: do they really correlate?
SO PHILOSOPHICAL MAGAZINE
CT 10th International Workshop on Disordered Systems Moveno-Andalo
CY MAR 18-21, 2006
CL Trento, ITALY
SP Univ Trento, Dipartimento Fis, DYGLAGEMEM European Network, INFM-CNR, Res Ctr SOFT
AB The correlation between the Fragility of glass-forming systems and the ratio of instantancous bulk to shear moduli, K-infinity/G(infinity) of the corresponding glasses is analyzed for bulk metallic and polymeric materials. We emphasize the importance of the free electron contribution to the bulk modulus of metals and a specific intramolecular contribution to fragility in the case of polymers for some long molecules. We argue that these two Contributions are the main reasons for the failure of the correlation between fragility and K-infinity/G(infinity) observed for these materials.
SN 1478-6435
PD JAN-FEB
PY 2007
VL 87
IS 3-5
SI SI
BP 613
EP 621
DI 10.1080/14786430600880769
UT WOS:000244150900031
ER

PT J
AU Miracle, DB
   Louzguine-Luzgin, DV
   Louzguina-Luzgina, LV
   Inoue, A
AF Miracle, D. B.
   Louzguine-Luzgin, D. V.
   Louzguina-Luzgina, L. V.
   Inoue, A.
TI An assessment of binary metallic glasses: correlations between
   structure, glass forming ability and stability
SO INTERNATIONAL MATERIALS REVIEWS
AB This manuscript explores the influence of atomic structure on glass forming ability and thermal stability in binary metallic glasses. A critical assessment gives literature data for 628 alloys from 175 binary glass systems. The atomic structure is quantified for each alloy using the efficient cluster packing model. Comparison of atomic structure with amorphous thickness and thermal stability gives the following major results. Binary glasses show a strong preference for discrete solute to solvent atomic radius ratios R*, which give efficient local atomic packing. Of 15 possible R* values, only five are common and only four represent the most stable glasses. The most stable binary glasses are also typically solute rich, with enough solute atoms to fill all the solute sites and roughly one-third of the solvent sites. This suggests that antisite defects, where solutes occupy solvent atom sites, are important in the glass forming ability of the most stable glasses. This stabilising effect results from an increase in the number of more stable solute-solvent bonds in solute rich glasses. Solute rich glasses also enable efficient global atomic packing. Together, these structural constraints represent only a narrow range of topologies and thus give a useful predictive tool for the exploration and discovery of new binary bulk metallic glasses (BMGs).
RI LOUZGUINE, Dmitri/D-2492-2010; Inoue, Akihisa/E-5271-2015
OI LOUZGUINE, Dmitri/0000-0001-5716-4987;
SN 0950-6608
EI 1743-2804
PD JUL
PY 2010
VL 55
IS 4
BP 218
EP 256
DI 10.1179/095066010X12646898728200
UT WOS:000280308100002
ER

PT J
AU Clavaguera-Mora, MT
AF Clavaguera-Mora, MT
TI Glass formation in metallic systems
SO BERICHTE DER BUNSEN-GESELLSCHAFT-PHYSICAL CHEMISTRY CHEMICAL PHYSICS
CT Discussion Meeting of the
   Deutsche-Bunsengesellschaft-fur-Physikalische-Chemie on Thermodynamic of
   Alloys
CY MAR 22-26, 1998
CL KIEL, GERMANY
SP Deut Bunsengesell Phys Chem
AB The aim of the present paper is to analyse the formation and stability of metallic glasses. Attention is focused to the history of the progress on new glassy alloys with specific applications: from soft magnetic Fe-based ribbons obtained by melt spinning or planar flow casting, through metallic glasses acting as precursors of nanocrystalline precipitates embedded in an amorphous matrix, towards he finding of new multicomponent metallic alloys with large glass-forming ability that open a new area of development and applications of bulk metallic glasses. The glass formation process is discussed in terms of the structure of the resultant material and in terms of the thermodynamics and kinetics of the process. The development of structural criteria for glass formation is based upon the notion that the free energy of particularly stable glassy structures will be low enough to make the driving force for crystallization negligible. Both structural and energetic criteria point to an enhanced glass-forming ability in multicomponent alloys. Kinetic criteria allow to differentiate between the ease of glass formation from an undercooled melt (or from a mixture of crystalline grains, as in solid state amorphization) and the stability of the glassy state versus devitrification or crystallization. Key kinetic parameters are viscosity and, more specifically in multicomponent systems, diffusivities of the different elements in the crystalline, glassy and metastable liquid state, respectively. The formation of nanostructured materials from controlled thermal treatment and their temperature stability is discussed in terms of the concentration gradients developing at the interface between the nanocrystals and the amorphous matrix. The qualitative influence of several parameters in the glass-forming ability and glass-stability as well as their Link to the empirical criteria that have been successfully employed to predict the glass-forming ability are presented.
SN 0005-9021
PD SEP
PY 1998
VL 102
IS 9
BP 1291
EP 1297
DI 10.1002/bbpc.19981020939
UT WOS:000076015600038
ER

PT J
AU Chen, WR
   Wang, Z
   Han, G
AF Chen Weirong
   Wang Zhou
   Han Guang
TI Composition design and optimization of Zr-Al-Ni-Fe bulk amorphous alloys
SO RARE METAL MATERIALS AND ENGINEERING
AB Zr-Al-Ni-Fe alloy series with different compositions were designed according to the criteria of constant electron concentration and atomic size. XRD results indicate that only the four alloys near the Zr-Al-Ni ternary system formed the bulk metallic glass. It is revealed by DSC and DTA curves that the best composition forming glass is Zr60.7Al15.5Ni15.5Fe8.3 with the thermodynamic characteristic parameters of T-g=710 K, T-x =770 K, Delta T-x=60 K, T-g/T-m=0.607, and T-g/T-l=0.552, respectively.
SN 1002-185X
PD JUL
PY 2006
VL 35
IS 7
BP 1017
EP 1020
UT WOS:000239522200002
ER

PT J
AU Tripathi, MK
   Chattopadhyay, PP
   Ganguly, S
AF Tripathi, Manwendra K.
   Chattopadhyay, P. P.
   Ganguly, Subhas
TI Evolutionary intelligence in design and synthesis of bulk metallic
   glasses by mechanical alloying
SO MATERIALS AND MANUFACTURING PROCESSES
AB The present work demonstrates a new approach of a combinatorial design of Bulk Metallic Glasses using evolutionary intelligence of genetic programming (GP) and genetic algorithm (GA). A glass-forming potential expression, carrying similar sense as the glass-forming ability (GFA) indicator expression, has been identified by principal component analysis and GP, enabling one to map the fitness of the composition from the multidimensional attribute space of the alloy in the course of GA search. The genetically inspired alloy compositions are evolved by GA search and subsequently validated through existing empirical knowledge. Two randomly chosen compositions are subjected to experimental trial where the glass-forming potential has been evaluated by amorphization potential through mechanical alloying. A literature-reported good glass -forming composition has been studied as a reference alloy and comparison was made for the proposed designed alloys.
OI Ganguly, Subhas/0000-0003-0878-7904; K Tripathi,
   Manwendra/0000-0001-5163-8862
SN 1042-6914
EI 1532-2475
PY 2017
VL 32
IS 10
SI SI
BP 1059
EP 1066
DI 10.1080/10426914.2017.1279305
UT WOS:000405597400003
ER

PT J
AU Ji, XL
   Pan, Y
AF Ji Xiu-lin
   Pan Ye
TI A thermodynamic approach to assess glass-forming ability of bulk
   metallic glasses
SO TRANSACTIONS OF NONFERROUS METALS SOCIETY OF CHINA
AB According to the Gibbs free energy difference between liquid and crystal, a thermodynamic glass-forming ability(GFA) parameter related to characteristic temperatures, onset crystallization temperature(T(x)) and liquidus temperature(T(1)), was proposed for evaluating the GFA of bulk metallic glasses(BMGs). The new parameter defined as omega-T(1)(T(1)+T(x))/(T(x)(T(1)-T(x))) has good correlation with the critical section thickness(Z(c)) of Ca-Mg-Cu BMGs. Being verified by the glasses data, including oxide glasses, which were used to validate the former GFA parameters, omega is one of the most reliable and applicable GFA parameters among T(rg) (=T(g)/T(1)), gamma(=T(x)(T(1)+T(g))), alpha (=T(x)/T(1)), delta (=T(x)/(T(1)-T(g))), and so on. Finally, predicting GFA of Cu-Ag-Zr-Ti and Cu-Zr-Ti-Al BMGs using omega was compared with the experimental results.
RI JI, XIULIN/D-2882-2015
OI JI, XIULIN/0000-0002-1752-6602
SN 1003-6326
PD OCT
PY 2009
VL 19
IS 5
BP 1271
EP 1279
DI 10.1016/S1003-6326(08)60438-0
UT WOS:000270793600037
ER

PT J
AU Huang, YJ
   Ning, ZL
   Shen, Z
   Liang, WZ
   Sun, HC
   Sun, JF
AF Huang, Yongjiang
   Ning, Zhiliang
   Shen, Zhe
   Liang, Weizhong
   Sun, Haicao
   Sun, Jianfei
TI Bending behavior of as-cast and annealed ZrCuNiAl bulk metallic glass
SO JOURNAL OF MATERIALS SCIENCE & TECHNOLOGY
AB Here ZrCuNiAl bulk metallic glass samples were annealed below its glass transition temperature. The bending behaviors and thermal properties of the as-cast and the annealed samples were studied. The increase of annealing time leads to the transition from ductile to more brittle behavior during bending tests. Meanwhile, prolonging the annealing causes a gradual decrease in the free volume content. Based on free volume theory, the change in the microstructure and mechanical properties induced by annealing treatment has been interpreted in detail. (C) 2017 Published by Elsevier Ltd on behalf of The editorial office of Journal of Materials Science & Technology.
SN 1005-0302
PD OCT
PY 2017
VL 33
IS 10
BP 1153
EP 1158
DI 10.1016/j.jmst.2017.07.002
UT WOS:000414224600012
ER

PT J
AU Wu, L
   Li, SD
   Fang, JL
   Chen, QH
   Peng, K
AF Wu, Lin
   Li, Shandong
   Fang, Jianglin
   Chen, Qinhui
   Peng, Kun
TI Enhancement of the glass forming ability of La-Al-Cu glassy alloys by
   partial substitution of Al by Mg
SO JOURNAL OF ALLOYS AND COMPOUNDS
CT 16th International Symposium on Metastable, Amorphous and Nanostructured
   Materials
CY JUL 05-09, 2009
CL Beijing, PEOPLES R CHINA
AB Partial substitution of Al by Mg was studied to reveal the influence of the neighboring element for glass forming ability (GFA) of La(62)Al(14)Cu(24) alloy. A series of quaternary La(62)Al(14-x)Mg(x)Cu(24) (x = 2.8, 3.2, 3.5, 4.2, 5.0) alloys were cast by copper mold casting. As a result, the La-Al-Mg-Cu alloys with full glassy rods of 10 mm in diameter were achieved, suggesting that partial substitution of Al by Mg dramatically enhances the GFA. The enhancement of GFA can be mainly attributed to the large atomic size ratios among these constituent elements. (C) 2010 Elsevier B.V. All rights reserved.
SN 0925-8388
PD AUG
PY 2010
VL 504
SU 1
BP S38
EP S40
DI 10.1016/j.jallcom.2010.03.102
UT WOS:000285252600011
ER

PT J
AU Mattern, N
   Sakowski, J
   Kuhn, U
   Vinzelberg, H
   Eckert, K
AF Mattern, N
   Sakowski, J
   Kuhn, U
   Vinzelberg, H
   Eckert, K
TI Structural behavior and glass transition of bulk metallic glasses
SO JOURNAL OF NON-CRYSTALLINE SOLIDS
CT 10th International Conference on the Physics of Non-Crystalline Solids
   (PNCS)
CY JUL 13-17, 2003
CL Univ Parma, Parma, ITALY
HO Univ Parma
AB The thermal behavior of Pd40Cu30Ni10P20 and Zr60Ti2Cu20Ni8Al10 bulk metallic glasses has been investigated in situ through the glass transition by means of differential scanning calorimetry, high-temperature X-ray synchrotron diffraction, and electrical resistivity. The temperature dependence of the X-ray structure factor can be well described by the Debye theory within the glassy state with a Debye temperature of theta = 296 K for Pd40Cu30Ni10P20, and theta = 418 K for Zr60Ti2Cu20Ni8Al10, respectively. At the glass transition temperature the temperature dependence of the structure changes, pointing to a continuous development of structural changes in the liquid state with temperature. The electrical resistivity behaves similar to the structure factor in accordance with Ziman theory. (C) 2004 Elsevier B.V. All rights reserved.
SN 0022-3093
PD OCT 15
PY 2004
VL 345
BP 758
EP 761
DI 10.1016/j.jnoncrysol.2004.08.197
UT WOS:000225583200147
ER

PT J
AU Lee, KS
   Jo, YM
   Lee, YS
AF Lee, Kwang Seok
   Jo, Yu Mi
   Lee, Young-Seon
TI Crystallization and high-temperature deformation behavior of Cu49Zr45Al6
   bulk metallic glass within supercooled liquid region
SO JOURNAL OF NON-CRYSTALLINE SOLIDS
AB Crystallization and plastic deformation of a Cu49Zr45Al6 ternary bulk metallic glass were investigated within a supercooled liquid region. In isochronal annealing processes, effective activation energies for the primary crystallization of Cu49Zr45Al6 ternary bulk metallic glass were calculated to be over 367 kJ/mol, which is quite high compared with other Cu-Zr-based bulk metallic glasses. Based on isothermal transformation kinetics described by the Johnson-Mehl-Avrami model, Avrami exponent (n) was calculated to be between 2.52 and 3.50, indicating that crystallization mechanism showed a diffusion-controlled growth with increasing nucleation rate. The appropriate working temperature-strain rate combination for feasible solid-to-solid forming without in situ crystallization was deduced by constructing a process map superimposed on a time-temperature transformation curve, thereby indicating phase crystallization. Furthermore, the actual solid-to-solid formability around this processing window was confirmed by overlapping the laboratory-scale extrusion ability. (C) 2013 Elsevier B.V. All rights reserved.
SN 0022-3093
EI 1873-4812
PD SEP 15
PY 2013
VL 376
BP 145
EP 151
DI 10.1016/j.jnoncrysol.2013.05.036
UT WOS:000323874100023
ER

PT J
AU Onoki, T
   Wang, X
   Zhu, S
   Hoshikawa, Y
   Sugiyama, N
   Akao, M
   Yasuda, E
   Yoshimura, M
   Inoue, A
AF Onoki, Takamasa
   Wang, Xinmin
   Zhu, Shengli
   Hoshikawa, Yasuto
   Sugiyama, Naota
   Akao, Masaru
   Yasuda, Eiichi
   Yoshimura, Masahiro
   Inoue, Akihisa
TI Bioactivity of titanium-based bulk metallic glass surfaces via
   hydrothermal hot-pressing treatment
SO JOURNAL OF THE CERAMIC SOCIETY OF JAPAN
AB Apatite forming and hydroxyapatite ceramics bonding ability of titanium (Ti)-based bulk metallic glasses (Ti40Zr10CU36Pd14 and (Ti0.40Zr0.10Cu0.36Pd0.14)(99)Ca-1: BMGs) were investigated as a new type of biomaterials. Powder mixture of CaHPO4 center dot 2H(2)O and Ca (OH) (2) and the BMGs disks were treated with autoclave for hydrothermal hot-pressing (HHP) simultaneously (150 degrees C, 40 MPa, 2hours). Direct bonding hydroxyapatite ceramics and both type of BMGs could not be achieved. And then, the BMGs disks with the HHP treatment were soaked in simulated body fluid for 7-10 days. It was observed that apatite are deposited and covered on the surface of the BMGs with the HHP treatment. It was firstly demonstrated that surfaces of Ti based bulk metallic glasses can be made bioactive and that HHP techniques were useful methods in purpose of making Ti-based bulk metallic glass surface bioactive.
RI zhu, shengli/A-8818-2011; Inoue, Akihisa/E-5271-2015; Yoshimura,
   Masahiro/H-5938-2011
OI Yoshimura, Masahiro/0000-0003-1810-0301
SN 1882-0743
EI 1348-6535
PD JAN
PY 2008
VL 116
IS 1349
BP 115
EP 117
DI 10.2109/jcersj2.116.115
UT WOS:000252626800026
ER

PT J
AU Yousfi, MA
   Hajlaoui, K
   Tourki, Z
   Yavari, AR
AF Yousfi, M. A.
   Hajlaoui, K.
   Tourki, Z.
   Yavari, A. R.
TI Serrated Flow Model for Metallic Glasses under Compressive Loading
SO ACTA METALLURGICA SINICA-ENGLISH LETTERS
AB A theological model is proposed that incorporates the serrated flow nature of metallic glasses. It involves the process of the nucleation, propagation and the arrest of a shear bands in the samples subjected to compressive deformation at room temperature. Numerical resolution of the constitutive equations resulting from the model is compared with the stress-strain curve obtained from in-situ nano-compression test in SEM of Zr-based metallic glass. Parametric identification method was applied and enabled us to release the physical parameters of the model. The obtained results showed that the model is adequately valid to describe the experimental data and the almost adjustable model parameters are physically meaningful and comparable to literature.
RI yavari, alain/E-8192-2010
SN 1006-7191
EI 2194-1289
PD OCT
PY 2013
VL 26
IS 5
BP 503
EP 508
DI 10.1007/s40195-013-0041-z
UT WOS:000326205000002
ER

PT J
AU Kim, J
   Kawamura, Y
AF Kim, Jonyhyun
   Kawamura, Y.
TI Electron beam welding of Zr-based BMG/Ni joints: Effect of beam
   irradiation position on mechanical and microstructural properties
SO JOURNAL OF MATERIALS PROCESSING TECHNOLOGY
AB The electron beam welding of 3mm thick Zr41Be23Ti14Cu12Ni10 bulk metallic glass (BMG) plate to Ni metal was investigated. The BMG was welded to Ni with an electron acceleration voltage of 60 kV, a beam current of 13.5 mA, welding speed of 45 mm/s and dw = 0.2 mm. The flexural strength of the welded joint (415 MPa) was higher than the yield strength of the Ni (345 MPa). The electron beam irradiation position had a strong influence on the joint properties. When the electronbeam was too far from the crystalline metal, no joining was achieved, and when the electron beam was too close to the crystalline metal it led to crystallization in the weld. (C) 2007 Elsevier B.V. All rights reserved.
SN 0924-0136
PD OCT 16
PY 2008
VL 207
IS 1-3
BP 112
EP 117
DI 10.1016/j.jmatprotec.2007.12.090
UT WOS:000259786200013
ER

PT J
AU Lu, YZ
   Huang, YJ
   Shen, J
AF Lu, Yunzhuo
   Huang, Yongjiang
   Shen, Jun
TI The electronic structure origin for ultrahigh glass-forming ability of
   the FeCoCrMoCBY alloy system
SO JOURNAL OF APPLIED PHYSICS
AB The authors report the effect of the addition of cobalt on glass-forming ability (GFA) in Fe-Co-Cr-Mo-C-B-Y bulk metallic glasses using Raman scattering and x-ray photoelectron spectroscopy. Among the alloy system, the alloy with 7% Co displays a unique electronic structure including the minimum core-level binding energy and the maximum Raman peak frequency. The origin of the superhigh GFA for the 7% Co alloy is discussed in terms of its unique electronic structure that leads to the densest atomic packing and strongest directional bonds. (C) 2011 American Institute of Physics. [doi: 10.1063/1.3622510]
RI Huang, Yongjiang/D-4809-2009
OI Lu, Yunzhuo/0000-0002-7322-6815
SN 0021-8979
EI 1089-7550
PD AUG 1
PY 2011
VL 110
IS 3
AR 033720
DI 10.1063/1.3622510
UT WOS:000293956600082
ER

PT J
AU Evenson, Z
   Schmitt, T
   Nicola, M
   Gallino, I
   Busch, R
AF Evenson, Zach
   Schmitt, Tobias
   Nicola, Mathias
   Gallino, Isabella
   Busch, Ralf
TI High temperature melt viscosity and fragile to strong transition in
   Zr-Cu-Ni-Al-Nb(Ti) and Cu47Ti34Zr11Ni8 bulk metallic glasses
SO ACTA MATERIALIA
AB The molten viscous behavior of Vitreloy 106, 106a, 105 and 101 bulk metallic glass-forming alloys was investigated at temperatures above T-liq. Couette concentric cylinder viscometry was used to determine the isothermal and continuous cooling melt viscosities. Isothermal three point beam bending measurements of the viscosity in the vicinity of T-g revealed a kinetically stronger liquid at low temperatures than at high temperatures, near T-liq. This behavior is revealed to be the result of a fragile to strong transition in the undercooled liquid, possibly due to polyamorphism. (c) 2012 Acta Materialia Inc. Published by Elsevier Ltd. All rights reserved.
SN 1359-6454
PD JUL
PY 2012
VL 60
IS 12
BP 4712
EP 4719
DI 10.1016/j.actamat.2012.05.019
UT WOS:000307415200005
ER

PT J
AU Yue, XX
   Inoue, A
   Liu, CT
   Fan, C
AF Yue, Xingxing
   Inoue, Akihisa
   Liu, Chain-Tsuan
   Fan, Cang
TI The Development of Structure Model in Metallic Glasses
SO MATERIALS RESEARCH-IBERO-AMERICAN JOURNAL OF MATERIALS
AB Metallic glasses (amorphous alloys) have grown from a singular observation to an expansive class of alloys with a broad range of scientific interests. Their unique properties require a robust understanding on the structures at the atomic level while alloys in this class have a similar outlook on the microstructure. In this review, we went through the history of the majority studies on the structure models of metallic glasses, and summarized their historical contributions to the understanding of the structure metallic glasses and the relationship between their structures and properties.
SN 1516-1439
EI 1980-5373
PD MAR-APR
PY 2017
VL 20
IS 2
BP 326
EP 338
DI 10.1590/1980-5373-MR-2016-0318
UT WOS:000400160300005
ER

PT J
AU Lewandowski, JJ
   Wang, WH
   Greer, AL
AF Lewandowski, JJ
   Wang, WH
   Greer, AL
TI Intrinsic plasticity or brittleness of metallic glasses
SO PHILOSOPHICAL MAGAZINE LETTERS
AB The intrinsic plasticity or brittleness of crystalline metals correlates with the ratio of the elastic shear modulus mu to the bulk modulus B; when the ratio mu/B exceeds a critical value, the metal is brittle. Sufficient data on elastic moduli and toughness are now available to permit an assessment for metallic glasses. We find a similar correlation, with the critical value of mu/B for metallic glasses (0.41-0.43) more sharply defined than for crystalline metals. This critical value applies also for annealing-induced embrittlement of metallic glasses. The clear correlation between mechanical behaviour (plasticity or brittleness) and mu/B assists in understanding flow and fracture mechanisms, and in guiding alloy design to alleviate brittleness of metallic glasses.
RI Greer, Lindsay/E-9433-2017; Greer, Alan Lindsay/G-1977-2011;
   Lewandowski, John/S-3815-2017
OI Lewandowski, John/0000-0002-3389-2637
SN 0950-0839
EI 1362-3036
PD FEB
PY 2005
VL 85
IS 2
BP 77
EP 87
DI 10.1080/09500830500080474
UT WOS:000230131900004
ER

PT J
AU Qiao, JW
   Liaw, PK
   Zhang, Y
AF Qiao, J. W.
   Liaw, P. K.
   Zhang, Y.
TI Ductile-to-brittle transition of in situ dendrite-reinforced
   metallic-glass-matrix composites
SO SCRIPTA MATERIALIA
AB The maximum strength of monolithic bulk metallic glasses (BMGs) at 77 K is predicted according to the proposed equation, sigma(max.77) = 55 rho(0)/M Delta T-g + Delta sigma. For the dendrite-reinforced BMG-matrix composites, a distinctly increased maximum strength is seen; however, a ductile-to-brittle transition seems to be present when lowering the temperature to 77 K, which is caused by the ductile-to-brittle transition of the dendrites. The crack, initiated within the dendrites, may extend to the glass matrix at low temperatures. As a result, early failure of the composites occurs. Crown Copyright (C) 2010 Published by Elsevier Ltd. on behalf of Acta Materialia Inc. All rights reserved.
RI ZHANG, Yong/B-7928-2009
OI ZHANG, Yong/0000-0002-6355-9923
SN 1359-6462
PD MAR
PY 2011
VL 64
IS 5
BP 462
EP 465
DI 10.1016/j.scriptamat.2010.11.012
UT WOS:000286540700025
ER

PT J
AU Du, XH
   Huang, JC
   Hsieh, KC
   Lai, YH
   Chen, HM
   Jang, JSC
   Liaw, PK
AF Du, X. H.
   Huang, J. C.
   Hsieh, K. C.
   Lai, Y. H.
   Chen, H. M.
   Jang, J. S. C.
   Liaw, P. K.
TI Two-glassy-phase bulk metallic glass with remarkable plasticity
SO APPLIED PHYSICS LETTERS
AB Using the computational-thermodynamic approach, the potential compositions of Zr-Cu-Ni-Al alloy system exhibiting the two-liquid miscibility phase equilibrium in the liquid temperature region have been identified. The resulting Zr base bulk metallic glasses show a microstructure of two microscaled glassy phases. The glass possesses a remarkable macroscopic plastic strain of 30% at room temperature. The gain of mechanical properties is attributed to the unique glassy structure correlated with the chemical inhomogeneity on the micron scale, the hard phases surrounded by the soft phases, leading extensive shear-band formation, interactions, and multiplication. (C) 2007 American Institute of Physics.
RI Huang, J./C-4276-2013
OI HUANG, Jacob Chih Ching/0000-0001-6843-3396
SN 0003-6951
PD SEP 24
PY 2007
VL 91
IS 13
AR 131901
DI 10.1063/1.2790380
UT WOS:000249787000024
ER

PT J
AU Ichitsubo, T
   Matsubara, E
   Anazawa, K
   Nishiyama, N
   Naito, M
   Hirotsu, Y
AF Ichitsubo, T.
   Matsubara, E.
   Anazawa, K.
   Nishiyama, N.
   Naito, M.
   Hirotsu, Y.
TI Low-temperature crystallization caused by ultrasound in
   Pd42.5Ni7.5Cu30P20 and Pd40Ni40P20 bulk metallic glasses
SO MATERIALS SCIENCE AND ENGINEERING A-STRUCTURAL MATERIALS PROPERTIES
   MICROSTRUCTURE AND PROCESSING
CT 14th International Conference on Internal Friction and Mechanical
   Spectroscopy (ICIFMS-14)
CY SEP 05-09, 2005
CL Kyoto, JAPAN
SP ICIFUAS, Iketani Sci Tech Fdn
AB Crystallization behavior of Pd42.5Ni7.5CU30P20 and Pd40Ni40P20 (number indicate at.%) bulk metallic glasses under ultrasonic (US) vibrations of subAow-MHz frequencies has been systematically investigated. It was found that, when the glassy sample was subjected to the US vibrations for a long duration, the crystallization occurred even (near) below the glass transition temperature T-g The microstructural features of the US-accelerated crystalline phases are presented, and the mechanism of the US-accelerated crystallization is briefly discussed in terms of beta relaxation. (c) 2006 Elsevier B.V. All rights reserved.
RI Nishiyama, Nobuyuki/C-8228-2015; Ichitsubo, Tetsu/F-3201-2010
OI Ichitsubo, Tetsu/0000-0002-1127-3034
SN 0921-5093
PD DEC 20
PY 2006
VL 442
IS 1-2
SI SI
BP 273
EP 277
DI 10.1016/j.msea.2006.01.149
UT WOS:000242975700056
ER

PT J
AU Louzguine-Luzgin, DV
AF Louzguine-Luzgin, D. V.
TI Vitrification and devitrification processes in metallic glasses
SO JOURNAL OF ALLOYS AND COMPOUNDS
AB The phenomenon of vitrification on cooling, also called the glass-transition still remains not fully understood. Recent studies by different techniques were performed in order to shed light on this and on the converse phenomenon of devitrification. Devitrification may either lead to the formation of a supercooled liquid, a crystal(s) or a quasicrystal(s). The present paper is a review of recent findings on the processes of vitrification and devitrification in metallic glasses. (C) 2012 Elsevier B.V. All rights reserved.
RI LOUZGUINE, Dmitri/D-2492-2010
OI LOUZGUINE, Dmitri/0000-0001-5716-4987
SN 0925-8388
EI 1873-4669
PD FEB 15
PY 2014
VL 586
SU 1
BP S2
EP S8
DI 10.1016/j.jallcom.2012.09.057
UT WOS:000328188800002
ER

PT J
AU Liang, L
   Hui, X
   Zhang, CM
   Chen, GL
AF Liang, L.
   Hui, X.
   Zhang, C. M.
   Chen, G. L.
TI An Er-based bulk metallic glass with high thermal stability and
   excellent magnetocaloric properties
SO INTERMETALLICS
AB A new heavy rare-earth-based Er36Ho20Al24Co20 bulk metallic glass (BMG) has been prepared by a copper mold casting. It is shown that this BMG alloy possesses higher glass transition temperature and crystallization temperature, larger effective activation energy for glass transition and crystallization compared with those of other known rare-earth-based BMGs. Under low magnetic field, this BMG alloy exhibits a comparable or even larger magnetocaloric effect than that of the previously reported RE-based BMGs and crystalline compounds. The high thermal stability and the excellent magnetocaloric effect together with other merits of the BMGs make this BMG alloy a suitable candidate for use as magnetic refrigerant in a temperature range below 50 K. (C) 2007 Elsevier Ltd. All rights reserved.
RI Hui, Xidong/A-1741-2010
SN 0966-9795
PD FEB
PY 2008
VL 16
IS 2
BP 198
EP 201
DI 10.1016/j.intermet.2007.09.005
UT WOS:000253275200009
ER

PT S
AU Bobrov, OP
   Laptev, SN
   Khonik, VA
AF Bobrov, O. P.
   Laptev, S. N.
   Khonik, V. A.
BE Darinskii, BM
   Magalas, LB
TI Isothermal stress relaxation of bulk and ribbon Pd40Cu30Ni10P20 metallic
   glass
SO MECHANICAL SPECTROSCOPY III: MECHANICAL SPECTROSCOPY AND RELAXATION
   PHENOMENA IN SOLIDS
SE SOLID STATE PHENOMENA
CT 3rd International Conference on Mechanical Spectroscopy and Relaxation
   Phenomena in Solids
CY 2006
CL Cracow, POLAND
SP Voronezh State Tech Univ, AGH
AB The paper presents the results of detailed measurements of the isothermal tensile stress relaxation in Pd40Cu30Ni10P20 bulk and ribbon samples, which differ by similar to 10(4) times in their production quenching rates. The relaxation law is derived. It is shown that the relaxation kinetics is not much affected by the quenching rate.
RI Khonik, Vitaly/A-5888-2009
SN 1012-0394
BN 3-908451-24-8
PY 2006
VL 115
BP 121
EP 126
DI 10.4028/www.scientific.net/SSP.115.121
UT WOS:000241414300015
ER

PT J
AU Bendert, JC
   Blodgett, ME
   Gangopadhyay, AK
   Kelton, KF
AF Bendert, J. C.
   Blodgett, M. E.
   Gangopadhyay, A. K.
   Kelton, K. F.
TI Measurements of volume, thermal expansion, and specific heat in
   Zr57Cu15.4Ni12.6Al10Nb5 and Zr58.5Cu15.6Ni12.8Al10.3Nb2.8 liquids and
   glasses
SO APPLIED PHYSICS LETTERS
AB The temperature dependence of the specific volume, thermal expansion coefficient, and specific heat in the equilibrium, supercooled liquids, and glasses of the bulk metallic glass-forming alloys Zr57Cu15.4Ni12.6Al10Nb5 and Zr58.5Cu15.6Ni12.8Al10.3Nb2.8 are determined using the containerless electrostatic levitation technique. Such measurements have not been possible thus far because of rapid crystallization from the supercooled metastable liquids. The results show a decrease in the thermal expansion coefficient with decreasing temperature before the onset of the glass transition. The difference in the rate of decrease between the two alloys indicates larger structural contributions to the thermal expansion for the stronger liquid at high temperatures. (C) 2013 AIP Publishing LLC.
SN 0003-6951
PD MAY 27
PY 2013
VL 102
IS 21
AR 211913
DI 10.1063/1.4808030
UT WOS:000320620400029
ER

PT J
AU Chen, B
   Shi, TL
   Li, M
   Yang, F
   Yan, F
   Liao, GL
AF Chen, B.
   Shi, T. L.
   Li, M.
   Yang, F.
   Yan, F.
   Liao, G. L.
TI Laser welding of annealed Zr55Cu30Ni5Al10 bulk metallic glass
SO INTERMETALLICS
AB Laser welding is one of the promising ways for manufacturing metallic glass products with complicated shape and geometry. In this work we focus on the effect of annealing treatment and welding parameters on laser welding of annealed Zr55Cu30Ni5Al10 bulk metallic glass as intended and unintended heat treatment occurs in the process. We find that laser welding can produce well welded specimen plates with no obvious welding defects in the joints and high welding speed may lead to better joints. Although higher annealing temperature or longer annealing time leads crystallization, bulk metallic glass material still remains largely amorphous in the heat affected zone. Compared with the welded joint without annealing, the micro-hardness and bending strength are enhanced due to the presence of the nanocrystals occurred in annealed welding joint. Therefore, appropriate annealing treatment with the annealing temperature near the glass transition temperature and annealing time as long as that in hot embossing of BMG parts may play a beneficial role in laser welding of metallic glasses. (C) 2013 Elsevier Ltd. All rights reserved.
SN 0966-9795
EI 1879-0216
PD MAR
PY 2014
VL 46
BP 111
EP 117
DI 10.1016/j.intermet.2013.11.008
UT WOS:000331024700019
ER

PT J
AU Chen, QJ
   Fan, HB
   Shen, J
   Sun, JF
   Lu, ZP
AF Chen, QJ
   Fan, HB
   Shen, J
   Sun, JF
   Lu, ZP
TI Critical cooling rate and thermal stability of Fe-Co-Zr-Y-Cr-Mo-B
   amorphous alloy
SO JOURNAL OF ALLOYS AND COMPOUNDS
AB The critical cooling rate of the Fe61Co5Zr8Y2Cr2Mo7B15 bulk amorphous alloy was determined to be 37 K/s, providing an indication that this alloy has a high glass-forming ability. The non-isothermal crystallization kinetics of the Fe61Co5Zr8Y2Cr2Mo7B15 amorphous alloy prepared by using the copper-mold casting or melt-spinning were also investigated. It is found that the activation energies in the non-isothermal crystallization process show a strong dependence on the cooling rates for glass formation, revealing that the melt-spun amorphous alloy has a higher thermal stability. (c) 2005 Elsevier B.V. All rights reserved.
RI Lu, Zhao-Ping/A-2718-2009
OI Lu, Zhao-Ping/0000-0003-1463-8948
SN 0925-8388
PD JAN 5
PY 2006
VL 407
IS 1-2
BP 125
EP 128
DI 10.1016/j.jallcom.2005.06.031
UT WOS:000234613600020
ER

PT J
AU Rouxel, T
AF Rouxel, Tanguy
TI Elastic properties of glasses: a multiscale approach
SO COMPTES RENDUS MECANIQUE
AB Very different materials are named 'Glass', with Young's modulus E and Poisson's ratio v extending from 5 to 180 GPa. and from 0.1 to 0.4, respectively, in the case of bulk inorganic glasses. Glasses have in common the lack of long range order in the atomic organization. Beside the essential role of elastic properties for materials selection in mechanical design, we show in this analysis that macroscopical elastic characteristics (E, nu) provide an interesting way to get insight into the short- and medium-range orders existing in glasses. In particular, nu, the packing density (C-g) and the glass network dimensionality appear to be strongly correlated. Networks consisting primarily of chains and layers units (chalcogenides, low Si-content silicate glasses) correspond to nu > 0.25 and C-g > 0.56, with maximum values observed for metallic glasses (nu similar to 0.4 and C-g > 0.7). On the contrary, nu < 0.25 is associated to a highly cross-linked network with a tri-dimensional organization resulting in a low packing density. Moreover, the temperature dependence of the elastic moduli brings a new light on the 'fragility' of glasses (as introduced by Angell) and on the level of cooperativity of atomic movements at the source of the deformation process. (c) 2006 Academie des sciences. Published by Elsevier Masson SAS. All rights reserved.
SN 1631-0721
PD DEC
PY 2006
VL 334
IS 12
BP 743
EP 753
DI 10.1016/j.crme.2006.08.001
UT WOS:000243064000008
ER

PT J
AU Lu, ZP
   Liu, CT
   Thompson, JR
   Porter, WD
AF Lu, ZP
   Liu, CT
   Thompson, JR
   Porter, WD
TI Structural amorphous steels
SO PHYSICAL REVIEW LETTERS
AB Recent advancement in bulk metallic glasses, whose properties are usually superior to their crystalline counterparts, has stimulated great interest in fabricating bulk amorphous steels. While a great deal of effort has been devoted to this field, the fabrication of structural amorphous steels with large cross sections has remained an alchemist's dream because of the limited glass-forming ability (GFA) of these materials. Here we report the discovery of structural amorphous steels that can be cast into glasses with large cross-section sizes using conventional drop-casting methods. These new steels showed interesting physical, magnetic, and mechanical properties, along with high thermal stability. The underlying mechanisms for the superior GFA of these materials are discussed.
RI Lu, Zhao-Ping/A-2718-2009
OI Lu, Zhao-Ping/0000-0003-1463-8948; Liu, Chain Tsuan/0000-0001-7888-9725
SN 0031-9007
EI 1079-7114
PD JUN 18
PY 2004
VL 92
IS 24
AR 245503
DI 10.1103/PhysRevLett.92.245503
UT WOS:000222112900024
PM 15245095
ER

PT J
AU Xu, M
   Wang, QJ
AF Xu, Min
   Wang, Qunjiao
TI Thermal stability and magnetic properties of Fe-Co-M-Zr-Nb-Ge-B (M=Mo,
   Cr) bulk metallic glasses
SO PROGRESS IN NATURAL SCIENCE-MATERIALS INTERNATIONAL
AB Fe62Co8-xMxZr6Nb4Ge1B19 (M=Mo, Cr) bulk metallic glasses were synthesized in the diameter range up to 2 mm by copper mold casting, which exhibit high thermal stability and large glass-forming ability. The super-cooled liquid region diminishes by the dissolution of Mo. The addition of 2 at% Cr leads to the broading of the liquid region remarkably, resulting in the improvement of thermal stability. The crystallization takes place through a single exothermic reaction, accompanying the precipitation of more than three kinds of crystallized phases such as alpha-Fe, Fe2Zr and ZrB2. The Fe-based alloys show soft ferromagnetic properties. The saturation magnetization (sigma(s)) decreases with increasing Mo or Cr content while the saturated magnetostriction increases with raising Mo or Cr content. There is no evident change in the sigma(s) and coercive force (H-c) with annealing temperature below the crystallization temperature, which suggests a more relaxed atomic configuration the glasses have. The crystallization causes a substantial enhancement in both sigma(s). and H-c. Each soft magnetic property of the glasses containing Cr with higher thermal stability is superior to that of the alloys containing Mo. (C) 2014 Chinese Materials Research Society. Production and hosting by Elsevier B.V. All rights reserved.
SN 1002-0071
EI 1745-5391
PD APR
PY 2014
VL 24
IS 2
BP 116
EP 120
DI 10.1016/j.pnsc.2014.03.004
UT WOS:000337868100005
ER

PT J
AU Cai, AH
   Xiong, X
   Liu, Y
   An, WK
   Tan, JY
AF Cai, An-hui
   Xiong, Xiang
   Liu, Yong
   An, Wei-ke
   Tan, Jing-ying
TI Artificial neural network modeling of reduced glass transition
   temperature of glass forming alloys
SO APPLIED PHYSICS LETTERS
AB A model based on radial base function artificial neural network (RBFANN) was designed for the simulation and prediction of reduced glass transition temperature T(rg) of glass forming alloys. Its performance is examined by the influences of different kinds of alloys and elements, large and minor change of element content on the T(rg), and composition dependence of T(rg) for La-Al-Ni ternary alloy system. Moreover, a group of Zr-Al-Ni-Cu bulk metallic glasses is designed by RBFANN. The values of T(rg) predicted by RBFANN agree well with the experimental values, indicating that the model is reliable and adequate. (c) 2008 American Institute of Physics.
SN 0003-6951
PD MAR 17
PY 2008
VL 92
IS 11
AR 111909
DI 10.1063/1.2899633
UT WOS:000254292400029
ER

PT J
AU Yuan, T
   Wang, GY
   Feng, QM
   Liaw, PK
   Yokoyama, Y
   Inoue, A
AF Yuan, Tao
   Wang, Gongyao
   Feng, Qingming
   Liaw, Peter K.
   Yokoyama, Yoshihiko
   Inoue, Akihisa
TI Modeling size effects on fatigue life of a zirconium-based bulk metallic
   glass under bending
SO ACTA MATERIALIA
AB A size effect on the fatigue-life cycles of a Zr50Cu30Al10Ni10 (at.%) bulk metallic glass has been observed in the four-point-bending fatigue experiment. Under the same bending-stress condition, large-sized samples tend to exhibit longer fatigue lives than small-sized samples. This size effect on the fatigue life cannot be satisfactorily explained by the flaw-based Weibull theories. Based on the experimental results, this study explores possible approaches to modeling the size effects on the bending-fatigue life of bulk metallic glasses, and proposes two fatigue-life models based on the Weibull distribution. The first model assumes, empirically, log-linear effects of the sample thickness on the Weibull parameters. The second model incorporates the mechanistic knowledge of the fatigue behavior of metallic glasses, and assumes that the shear-band density, instead of the flaw density, has significant influence on the bending fatigue-life cycles. Promising predictive results provide evidence of the potential validity of the models and their assumptions. (C) 2012 Acta Materialia Inc. Published by Elsevier Ltd. All rights reserved.
RI Inoue, Akihisa/E-5271-2015; Yokoyama, Yoshihiko/A-8603-2011
SN 1359-6454
PD JAN
PY 2013
VL 61
IS 1
BP 273
EP 279
DI 10.1016/j.actamat.2012.09.057
UT WOS:000313604400025
ER

PT J
AU Bian, Z
   Inoue, A
AF Bian, Z
   Inoue, A
TI Ce-Cu-Fe-Al-Si bulk metallic glass alloys with high glass forming
   ability
SO MATERIALS TRANSACTIONS
AB New Ce-Cu-Fe-Al-Si bulk metallic glasses (BMGs) with high glass formation ability (GFA) anti large supercooled liquid regions (Delta T-x, Delta T-x = T-x-T-g) were developed. Investigation shows that the addition of Fe and Si elements is very effective to improve the glass formation ability of Ce-based BMGs. For an optimizing composition, the Delta T-x value is 95 K and the alloy can be prepared into bulk metallic glass roots with the diameters of at least 10 mm. In the Ce-Cu-Al-Fe alloys, the glass transition temperature (T-g), crystallization temperature (T-x) and Delta T-x increase with increasing Fe content. The reduced glass transition temperature (T-rg = T-g/T-1) of the present BMG system is about 0.5-0.6 and is much lower than that of other metal base BMG systems with high GFA. Compressive fracture strength (sigma(f)) and Young's modulus (E) of the Ce-Cu-Al-Fe BMGs are similar to 930 MPa and similar to 45 GPa, respectively, and increase with increasing Fe content.
RI Inoue, Akihisa/E-5271-2015
SN 1345-9678
EI 1347-5320
PD NOV
PY 2005
VL 46
IS 11
BP 2541
EP 2544
DI 10.2320/matertrans.46.2541
UT WOS:000234127100040
ER

PT S
AU Wu, JP
   Li, SD
   Liu, MM
   Cai, XL
   Hu, Y
   Qiu, J
   Lin, JH
AF Wu, Jianpeng
   Li, Shandong
   Liu, Meimei
   Cai, Xinle
   Hu, Yi
   Qiu, Jie
   Lin, Jianhua
BE Zeng, JM
   Kim, YH
   Chen, YF
TI Effects of C addition on glass-forming in FeCPSiBMoMn bulk metallic with
   good soft-magnetic properties
SO NEW MATERIALS, APPLICATIONS AND PROCESSES, PTS 1-3
SE Advanced Materials Research
CT International Conference on Chemical, Material and Metallurgical
   Engineering (ICCMME 2011)
CY DEC 23-25, 2011
CL Beihai, PEOPLES R CHINA
SP Guangxi Univ, China, Wuhan Univ Sci & Technol, Queensland Univ Technol
AB The effect of C substitution on the glass forming ability (GFA) and soft magnetic properties of Fe-based bulk metallic glasses (BMG) Fe79.4-xCxSi3.5B5.1P8.9Mo3Mn0.2 (x = 4.2, 5.2, and 7.0) alloys have been investigated. It is revealed that fully glassy alloy rods with diameters up to 4 mm can be prepared by conventional copper mold casting method even using the low-cost industrial Fe-P master alloy. Properly substituting of Fe by C gives rise to an enhancement of GFA. Moreover, all the samples exhibit good soft magnetic properties with high saturation magnetization up to 1.16 T and low coercivity of 204 A/m.
SN 1022-6680
BN 978-3-03785-309-2
PY 2012
VL 399-401
BP 1012
EP +
DI 10.4028/www.scientific.net/AMR.399-401.1012
UT WOS:000310249300202
ER

PT J
AU Rouxel, T
AF Rouxel, T.
TI Thermodynamics of viscous flow and elasticity of glass forming liquids
   in the glass transition range
SO JOURNAL OF CHEMICAL PHYSICS
AB The elastic moduli of glasses from different chemical systems, including oxide, chalcogenide, oxynitride, and metallic, were investigated through the glass transition (T(g)), typically from 0.4 to 1.3 T(g). These data were used to interpret the temperature sensitivity of the shear viscosity coefficient obtained on the same materials. The relevant Gibbs free activation energy was estimated from the apparent heat of flow by means of the temperature dependence of the shear elastic modulus. The activation entropy associated with the viscous flow was also derived and was found to correlate with the fragile versus strong character of the glass forming liquids. Finally, the physicochemistry of the flow process was described on the basis of the glass network de-structuration which shows up through the temperature dependence of Poisson's ratio, and an expression for the shear viscosity coefficient is proposed which is chiefly based on the high temperature elastic behavior. (C) 2011 American Institute of Physics. [doi: 10.1063/1.3656695]
SN 0021-9606
PD NOV 14
PY 2011
VL 135
IS 18
AR 184501
DI 10.1063/1.3656695
UT WOS:000297472800027
PM 22088069
ER

PT J
AU Zhang, T
   Yang, Q
   Ji, YF
   Li, R
   Pang, SJ
   Wang, JF
   Xu, T
AF Zhang Tao
   Yang Qin
   Ji YunFei
   Li Ran
   Pang ShuJie
   Wang JianFeng
   Xu Tao
TI Centimeter-scale-diameter Co-based bulk metallic glasses with fracture
   strength exceeding 5000 MPa
SO CHINESE SCIENCE BULLETIN
AB Co(48-x) Fe (x) Cr(15)Mo(14)C(15)B(6)Er(2) (x = 2.5 and 5) bulk metallic glasses (BMGs) with critical size (d (c)) up to 15 mm in diameter were synthesized by copper mold casting. Co(43)Fe(5)Cr(15)Mo(14)C(15)B(6)Er(2) BMG has a wide supercooled liquid region (84 K) and ultrahigh fracture strength exceeding 5000 MPa, which is the highest value for reported metallic glasses with d (c) exceeding 1 cm. Furthermore, the Poisson ratio, Young modulus, shear modulus and bulk modulus of this alloy were evaluated to be 0.31, 217 GPa, 82 GPa and 214 GPa, respectively. The ideal configurational entropy of Co(48-x) Fe (x) Cr(15)Mo(14)C(15)B(6)Er(2) (x = 0 and 5) has been calculated. The introduction of the similar element Fe in the Fe-free Co-Cr-Mo-C-B-Er alloy will be beneficial to increasing the ideal configurational entropy and depressing the critical cooling rate to achieve greater glass-forming ability. The combination of superior glass-forming ability and ultrahigh fracture strength makes the present Co(48-x) Fe (x) Cr(15)Mo(14)C(15)B(6)Er(2) (x = 2.5 and 5) BMGs promising candidates as advanced structural materials.
RI Zhang, Tao/O-4911-2014; Pang, Shujie/D-8305-2016; Li, Ran/B-4618-2010
SN 1001-6538
PD DEC
PY 2011
VL 56
IS 36
BP 3972
EP 3977
DI 10.1007/s11434-011-4765-8
UT WOS:000297866900015
ER

PT J
AU Wang, YQ
   Li, J
   Tian, R
   Fang, SS
   Xiao, XS
AF Wang, YQ
   Li, J
   Tian, R
   Fang, SS
   Xiao, XS
TI Bulk metallic glasses in Sm-Cu-Ni-Al alloy systems
SO JOURNAL OF RARE EARTHS
AB A new series of Sm-Cu-Ni-Al bulk metallic glasses were prepared by suction casting. Their glass-forming ability and thermal stability were investigated by X-ray diffraction (XRD) and differential scanning calorimetry (DSC). The results of DSC indicate that the alloy components strongly affect the widths of supercooled liquid region (Delta T-x + T-x - T-g(onset)).The widths of supercooled liquid region for Sm70-xCuxNi10Al20(x = 12.5, 15.0, 17.5), SM65-x Cu15NixAl20 (x = 8, 10, 12) and Sm75-xCu15Ni10Alx(x = 15, 20, 25) increase first and then decrease with the increase of x atomic fraction. And Sm55Cu15Ni10Al20 alloy was found to be with the largest width and it can be cast into bulk metallic glass with up to 3 mm in diameter. The glass-forming ability of the alloys was explained in terms of Miedema's theory, electronegativity difference and atomic size parameter of the amorphous alloys.
SN 1002-0721
PD DEC
PY 2005
VL 23
SU S
BP 415
EP 418
AR PII 1002-0721(2005)0415-04
UT WOS:000236093300106
ER

PT J
AU Kobelev, NP
   Khonik, VA
   Makarov, AS
   Afonin, GV
   Mitrofanov, YP
AF Kobelev, N. P.
   Khonik, V. A.
   Makarov, A. S.
   Afonin, G. V.
   Mitrofanov, Yu. P.
TI On the nature of heat effects and shear modulus softening in metallic
   glasses: A generalized approach
SO JOURNAL OF APPLIED PHYSICS
AB The paper presents a new approach to the nature of heat effects and shear modulus softening in metallic glasses. The approach is based on the assumption that the glass contains quenched-in "defects"-elastic dipoles. Using the nonlinear elastic representation of the internal energy of glass with quenched-in elastic dipoles, we derive a simple analytical law, which connects the heat flow and temperature derivative of the shear modulus. Specially performed experiments confirmed the validity of this law. The exothermal and endothermal heat processes in glass reveal through the relaxation of the shear modulus confirming it as a key parameter for the understanding the relaxation processes in glasses. (C) 2014 AIP Publishing LLC.
RI Mitrofanov, Yuriy/E-7963-2010; Afonin, Gennady/K-1014-2013; Khonik,
   Vitaly/A-5888-2009; Makarov, Andrey/H-4156-2013
OI Mitrofanov, Yuriy/0000-0002-7939-5230; Afonin,
   Gennady/0000-0002-7715-5065; Makarov, Andrey/0000-0001-6741-0619
SN 0021-8979
EI 1089-7550
PD JAN 21
PY 2014
VL 115
IS 3
AR 033513
DI 10.1063/1.4862399
UT WOS:000330615500030
ER

PT J
AU Li, BS
   Shahabi, HS
   Scudino, S
   Eckert, J
   Kruzic, JJ
AF Li, B. S.
   Shahabi, H. Shakur
   Scudino, S.
   Eckert, J.
   Kruzic, J. J.
TI Designed heterogeneities improve the fracture reliability of a Zr-based
   bulk metallic glass
SO MATERIALS SCIENCE AND ENGINEERING A-STRUCTURAL MATERIALS PROPERTIES
   MICROSTRUCTURE AND PROCESSING
AB Bulk metallic glasses (BMGs) have been reported to have large variability in model fracture toughness. In this study, alternating soft and hard regions were created in a Zr52.5Ti5Cu18Ni14.5Al10 (at%) BMG via mechanical imprinting at room temperature. While only 50% of as-cast samples demonstrated plastic deformation during mode I fracture tests, 100% of imprinted samples demonstrated measurable plasticity and the scatter in measured mode I fracture toughness was significantly reduced. Mechanical treatments show promise for improving the fracture reliability of BMGs. (C) 2015 Elsevier B.V. All rights reserved.
RI Scudino, Sergio/D-8049-2015; Kruzic, Jamie/M-3558-2014
OI Kruzic, Jamie/0000-0002-9695-1921
SN 0921-5093
EI 1873-4936
PD OCT 14
PY 2015
VL 646
BP 242
EP 248
DI 10.1016/j.msea.2015.08.055
UT WOS:000362132000026
ER

PT J
AU Zhang, LC
   Xing, DM
   Zhang, TH
   Wei, BC
   Li, WH
   Wang, YR
AF Zhang, Lingchen
   Xing, Dongmei
   Zhang, Taihua
   Wei, Bingchen
   Li, Weihuo
   Wang, Yuren
TI Deformation behavior during nanoindentation in Ce-based bulk metallic
   glasses
SO CHINESE SCIENCE BULLETIN
AB The deformation behavior and the effect of the loading rate on the plastic deformation in Ce-based bulk metallic glasses (BMGs) were investigated through nanoindentation tests. The results showed that the loading rate dependence of plastic deformation during nanoindentation measurements in the Ce-based BMGs is quite unique in contrast to that of other BMG alloys. The load-displacement (P-h) curves of Ce60Al15Cu10Ni15 BMG exhibit a homogeneous plastic deformation at low loading rates, and a prominent serrated flow at high strain rates, whereas, the P-h curves of Ce65Al10Cu10Ni10Nb5 exhibit homogenous plastic deformation at all studied loading rates. The room temperature creep behavior could clearly be observed in these two alloys. The mechanism of the unique plastic deformation feature in the Ce-based BMGs was studied.
RI zhang, lingchenzhng/A-1724-2009; Wang, Yuren/B-2124-2013
SN 1001-6538
PD JUL
PY 2006
VL 51
IS 13
BP 1639
EP 1643
DI 10.1007/s11434-006-2013-4
UT WOS:000240487600016
ER

PT J
AU Zhang, Y
   Li, Y
   Tan, H
   Chen, GL
   Davies, HA
AF Zhang, Y.
   Li, Y.
   Tan, H.
   Chen, G. L.
   Davies, H. A.
TI Glass forming ability criteria for La-Al-(Cu, Ni) alloys
SO JOURNAL OF NON-CRYSTALLINE SOLIDS
AB Criteria for evaluating the glass forming ability (GFA) of La-Al-(Cu. Ni) alloys were studied. The GFA criteria can be categorized into two types, according to whether the liquidus temperature T, is included in the formula or not. The T, inclusive criteria generally predict a maximum in GFA at the eutectic composition at which T, is a minimum. However, for La-Al-(Cu,Ni) alloys, the best GFA does not correspond to the eutectic. The paper discusses the suggestion that the solidification temperature Ts should be used instead of T, for evaluating the GFA of La-Al-(Cu,Ni) alloys. (c) 2006 Elsevier B.V. All rights reserved.
RI ZHANG, Yong/B-7928-2009
OI ZHANG, Yong/0000-0002-6355-9923
SN 0022-3093
PD DEC 15
PY 2006
VL 352
IS 52-54
BP 5482
EP 5486
DI 10.1016/j.jnoncrysol.2006.09.027
UT WOS:000242919300005
ER

PT S
AU Pang, JJ
   Tan, MJ
   Jarfors, A
   Chuang, PD
AF Pang, J. J.
   Tan, M. J.
   Jarfors, Anders
   Chuang, P. D.
BE Chandra, T
   Wanderka, N
   Reimers, W
   Ionescu, M
TI Glass Formation and Structural Study of Ti50Cu50 Alloy by Molecular
   Dynamics
SO THERMEC 2009, PTS 1-4
SE Materials Science Forum
CT 6th International Conference on Processing and Manufacturing of Advanced
   Materials (THERMEC)/2nd Symposium Session on Multiscale Mechanical
   Modelling of Complex Materials and Engineering Applications
CY AUG 25-29, 2009
CL Berlin, GERMANY
SP Minerals, Met & Mat Soc (TMS)
AB Ti-based metallic glasses (MGs) due to their relative low densities exhibit ultrahigh specific characteristics In this article the glass-forming behavior and atomic structure of Ti50Cu50 MG were investigated through molecular dynamics simulation (MDS) using the general embedded-atom method (GEAM) potential. As observed experimentally, simulated Ti50Cu50 alloy undergoes three states on quenching. (i) equilibrium liquid; (ii) supercooled liquid and (iii) glassy solid. The atomic configuration of the glass was analysed based on the radial distribution function (RDF) and Voronoi tessellation (VT) It was found that there exist a variety of polyhedral units in Ti50Cu50 MG, where distorted icosohedral and bcc clusters are dominant
RI Tan, Ming-Jen/A-3862-2011
OI Tan, Ming-Jen/0000-0002-3583-1723; Jarfors, Anders/0000-0002-0101-0062
SN 0255-5476
PY 2010
VL 638-642
BP 1665
EP +
DI 10.4028/www.scientific.net/MSF.638-642.1665
PN 1-4
UT WOS:000281043800276
ER

PT J
AU Liu, FJ
   Yao, KF
   Ding, HY
AF Liu, F. J.
   Yao, K. F.
   Ding, H. Y.
TI Fe-based glassy alloys with high iron content and high saturation
   magnetization
SO INTERMETALLICS
AB Iron-based bulk metallic glasses (BMGs) are attractive due to their excellent magnetic properties. It is known that increasing the iron content in alloys would enhance the magnetic property of glassy alloys, but would reduce their glass forming ability. Despite researchers are keen to develop Fe-based BMGs with high Fe content, BMG with Fe content higher than 79 at.% has not been reported. Here we report that Fe-based BMGs with Fe content as high as 78 similar to 81 at.% have been successfully prepared. The as-prepared Fe81Mo1P7.5C5.5B2Si3 BMG possesses a saturation magnetization of 1.64 T, which is significantly higher than the reported value in BMGs. (C) 2011 Elsevier Ltd. All rights reserved.
RI Ding, Hongyu/G-9833-2013
SN 0966-9795
EI 1879-0216
PD NOV
PY 2011
VL 19
IS 11
BP 1674
EP 1677
DI 10.1016/j.intermet.2011.07.011
UT WOS:000295431400007
ER

PT J
AU Lang, L
   Tian, ZA
   Xiao, SF
   Deng, HQ
   Ao, BY
   Chen, PH
   Hu, WY
AF Lang, Lin
   Tian, Zean
   Xiao, Shifang
   Deng, Huiqiu
   Ao, Bingyun
   Chen, Piheng
   Hu, Wangyu
TI Molecular dynamics simulations of the structure evolutions of Cu-Zr
   metallic glasses under irradiation
SO NUCLEAR INSTRUMENTS & METHODS IN PHYSICS RESEARCH SECTION B-BEAM
   INTERACTIONS WITH MATERIALS AND ATOMS
CT 13th International Conference on Computer Simulation of Radiation
   Effects in Solids (COSIRES)
CY JUN 19-24, 2016
CL Loughborough Univ, Loughborough, ENGLAND
SP Inst Phys, CCP5, IAEA, UK Engn & Phys Sci Res Council
HO Loughborough Univ
AB Molecular dynamics simulations have been performed to investigate the structural evolution of Cu64.5Zr35.5 metallic glasses under irradiation. The largest standard cluster analysis (LSCA) method was used to quantify the microstructure within the collision cascade regions. It is found that the majority of clusters within the collision cascade regions are full and defective icosahedrons. Not only the smaller structures (common neighbor subcluster) but also primary clusters greatly changed during the collision cascades; while most of these radiation damages self-recover quickly in the following quench states. These findings indicate the Cu-Zr metallic glasses have excellent irradiation-resistance properties. (C) 2016 Elsevier B.V. All rights reserved.
RI Hu, Wangyu/B-5762-2009; Deng, Huiqiu/A-9530-2009
OI Hu, Wangyu/0000-0001-7416-3994; Deng, Huiqiu/0000-0001-8986-104X
SN 0168-583X
EI 1872-9584
PD FEB 15
PY 2017
VL 393
BP 77
EP 81
DI 10.1016/j.nimb.2016.11.028
UT WOS:000394194500018
ER

PT J
AU Li, R
   Pang, SJ
   Men, H
   Ma, CL
   Zhang, T
AF Li, R
   Pang, SJ
   Men, H
   Ma, CL
   Zhang, T
TI Formation and mechanical properties of (Ce-La-Pr-Nd)-Co-Al bulk glassy
   alloys with superior glass-forming ability
SO SCRIPTA MATERIALIA
AB Bulk (Ce-La-Pr-Nd)(65)Co25Al10 metallic glass with critical diameter of 15 mm and high mechanical strength was developed. The addition of multi-lanthanide metals with diverse electron configurations and similar atomic sizes induced a sharply decreased liquidus temperature and remarkably improved glass-forming ability of (Ce-La-Pr-Nd)(65)Co25Al10 compared with single-lanthanide metal-based alloys, e.g. La65Co25Al10 and Ce65Co25Al10. (c) 2005 Acta Materialia Inc. Published by Elsevier Ltd. All rights reserved.
RI Zhang, Tao/O-4911-2014; Li, Ran/B-4618-2010; Pang, Shujie/D-8305-2016
SN 1359-6462
PD MAR
PY 2006
VL 54
IS 6
BP 1123
EP 1126
DI 10.1016/j.scriptamat.2005.11.074
UT WOS:000234941500029
ER

PT J
AU Ma, H
   Xu, J
   Ma, E
AF Ma, H
   Xu, J
   Ma, E
TI Mg-based bulk metallic glass composites with plasticity and high
   strength
SO APPLIED PHYSICS LETTERS
AB Composite alloys of (Mg0.65Cu0.075Ni0.075Zn0.05Ag0.05Y0.1)(100-x)Fe-x(x=9 and 13) have been produced through copper mold casting, based on a good bulk metallic glass former. Upon cooling the melt, an alpha-Fe solid solution precipitates uniformly with sizes in the 1-10 mum range while the remaining melt undergoes a glass transition. The in situ composite has a compressive strength as high as similar to1 GPa, a factor of 1.6 higher than the single-phase metallic glass. In contrast to all the previous Mg-based monolithic glasses that always fail in the elastic regime, a plastic strain to failure of the order of 1% was obtained for the Fe-toughened composite. (C) 2003 American Institute of Physics.
RI Ma, En/A-3232-2010
SN 0003-6951
PD OCT 6
PY 2003
VL 83
IS 14
BP 2793
EP 2795
DI 10.1063/1.1616192
UT WOS:000185664000021
ER

PT J
AU Lin, T
   Bian, XF
   Jiang, J
AF Lin, T
   Bian, XF
   Jiang, J
TI Relation between calculated Lennard-Jones potential and thermal
   stability of Cu-based bulk metallic glasses
SO PHYSICS LETTERS A
AB Two metallic bulk glasses, Cu60Zr30Ti10 and Cu47Ti33Zr11Ni8Si1, with a diameter of 3 mm were prepared by copper mold casting method. Dilatometric measurement was carried out on the two glassy alloys to obtain information about the average nearest-neighbour distance r(0) and the effective depth of pair potential V-0. By assuming a Lennard-Jones potential, r(0) and V-0 were calculated to be 0.28 nm and 0.16 eV for Cu60Zr30Ti10 and 0.27 nm and 0.13 eV for Cu47Ti33Zr11Ni8Si1, respectively. It was found that the glassy alloy Cu60Zr30Ti10 was more stable than Cu47Ti33Zr11Ni8Si1 against heating from both experiment and calculation. (c) 2006 Elsevier B.V. All rights reserved.
SN 0375-9601
PD MAY 15
PY 2006
VL 353
IS 6
BP 497
EP 499
DI 10.1016/j.physleta.2006.01.012
UT WOS:000237686100011
ER

PT J
AU Wu, JL
   Pan, Y
   Li, XZ
   Wang, XF
AF Wu, Jili
   Pan, Ye
   Li, Xingzhou
   Wang, Xianfei
TI Composition design of plastic Cu-Zr-Ti-Ni bulk metallic glass: A
   liquidus perspective
SO MATERIALS SCIENCE AND ENGINEERING A-STRUCTURAL MATERIALS PROPERTIES
   MICROSTRUCTURE AND PROCESSING
AB In this paper, newly designed plastic Cu51Zr37Ti8Ni4 bulk metallic glass has been successfully fabricated. By purposely increasing the liquidus temperature in a quaternary Cu-Zr-Ti-Ni alloy system, glass-forming ability of as-cast bulk metallic glasses shows a desired decrease trend. As-cast Cu51Zr37Ti8Ni4, which has a lower glass-forming ability and a higher liquidus temperature than Cu52Zr37Ti8Ni3, is of monolithic amorphous structure, and also displays remarkable plasticity of 10.5% together with the fracture strength of 2145 MPa. Multiple and interactive shear bands can be discovered on the fracture surface of fractured Cu51Zr37Ti8Ni4. In addition, the deformation of Cu51Zr37Ti8Ni4 shows serrated flow behavior. By a statistics analysis of serrations in the stress-time curve, the serrated flow behavior of the sample follows the self-organized critical behavior. This work suggests that, with the aid of CALPHAD, composition design of plastic bulk metallic glasses can be achieved according to the liquidus perspective. (C) 2014 Elsevier B.V. All rights reserved.
SN 0921-5093
EI 1873-4936
PD JUL 1
PY 2014
VL 608
BP 16
EP 20
DI 10.1016/j.msea.2014.04.072
UT WOS:000338404800003
ER

PT J
AU Rogers, DN
   Shen, YL
AF Rogers, D. N.
   Shen, Y. -L.
TI Deformation localization in constrained layers of metallic glasses: A
   parametric modeling analysis
SO THIN SOLID FILMS
AB Localized plastic deformation known as shear banding is a prominent feature in metallic glasses. In this study we perform parametric three-dimensional finite element analyses, using primarily a thin layer of metallic glass on top of a cylindrical base, to study how physical constraint can affect this localized form of deformation and the corresponding macroscopic stress-strain response. Random perturbation points are added to the metallic glass model to facilitate the formation of shear bands. The modeling result suggests that the mechanical behavior of metallic glasses can be significantly influenced by the geometrical confinement. Under nominally uniaxial compressive loading, a lower thickness-to-diameter ratio results in higher plastic flow stresses. Shear bands tend to concentrate in regions away from the interface with the base material. The findings provide a mechanistic rationale for experimental observations based on the micropillar compression test. The deformation pattern in a multilayered metallic glass structure is also examined. (C) 2013 Elsevier B.V. All rights reserved.
RI Shen, Yu-Lin/C-1942-2008
SN 0040-6090
PD JUN 30
PY 2014
VL 561
BP 108
EP 113
DI 10.1016/j.tsf.2013.05.170
UT WOS:000337857100015
ER

PT J
AU Wang, JQ
   Yu, P
   Bai, HY
AF Wang, J. Q.
   Yu, P.
   Bai, H. Y.
TI Minor addition induced enhancement of strength of Mg-based bulk metallic
   glass
SO JOURNAL OF NON-CRYSTALLINE SOLIDS
AB We report that the fracture strength of Mg-based bulk metallic glasses (BMGs) can be dramatically enhanced up to 1.10 GPa by minor Gd addition. The Poisson's ratio v of the BMG also decreases to 0.261 close to that of brittle oxide glasses when 1 at.% Gd was added. Such significant enhancement in strength which approaches the theoretical strength value and dramatically decrease in the Poisson's ratio are attributed to the structural change of the BMGs induced by the Gd minor addition. (C) 2008 Elsevier B.V. All rights reserved.
RI Wang, Jun-Qiang/G-5989-2010; Wang, Junqiang/C-2839-2015
OI Wang, Junqiang/0000-0002-8066-6237
SN 0022-3093
EI 1873-4812
PD DEC 15
PY 2008
VL 354
IS 52-54
BP 5440
EP 5443
DI 10.1016/j.jnoncrysol.2008.08.010
UT WOS:000261710700016
ER

PT J
AU Zheng, Q
AF Zheng, Qiang
TI DESIGN STRATEGIES TO IMPROVE THE PLASTICITY OF BULK METALLIC GLASSES
SO REVIEWS ON ADVANCED MATERIALS SCIENCE
AB Bulk metallic glasses (BMGs) have many excellent properties, such as high strength, high hardness, high specific strength, better corrosion resistance, and excellent soft magnetic property as well. However, their lack of plasticity due to the shear localization at room temperature which has been considered the Achilles' heel seriously restricts their application as structural materials. So far, a great deal of efforts has been devoted to improving the ductility and toughness of BMGs. In this paper, the-state-of-art eight successful design strategies were summarized in order to give clues and directions to improve the ductility and toughness of bulk metallic glass. Both the advantage and disadvantage of each method were briefly analyzed.
RI Zheng, Qiang/A-4566-2010
OI Zheng, Qiang/0000-0002-6057-8141
SN 1606-5131
PD FEB
PY 2015
VL 40
IS 1
BP 1
EP 14
UT WOS:000350328900001
ER

PT J
AU Li, P
   Hao, JY
   Tan, JL
   Wang, Q
AF Li, Ping
   Hao, Jingyan
   Tan, Jialong
   Wang, Qing
TI The effect of Al on the elastic properties of (Cu0.56Zr0.44)(100-x)Al-x
   bulk metallic glasses
SO MATERIALS SCIENCE AND ENGINEERING A-STRUCTURAL MATERIALS PROPERTIES
   MICROSTRUCTURE AND PROCESSING
AB (Cu0.56Zr0.44)(100-x)Al-x series bulk metallic glasses were prepared by using copper mould suction casting method. The thermal parameters were obtained by thermodynamic analysis. Longitudinal and shear ultrasonic velocities were measured by water-immersion focusing method and the elastic parameters were calculated. Results show that the elastic properties are improved with increasing Al content, which is attributed to the stronger interaction of Al-Zr than Cu-Zr. Furthermore, the more the elastic properties, the higher some of the thermodynamic parameters such as Delta T, T-x, T-g/T-1 and T-x/T-m are. (C) 2009 Elsevier B.V. All rights reserved.
SN 0921-5093
EI 1873-4936
PD AUG 25
PY 2009
VL 518
IS 1-2
BP 16
EP 18
DI 10.1016/j.msea.2009.04.017
UT WOS:000268532300003
ER

PT J
AU Li, JQ
   Law, JY
   Ma, HR
   He, AN
   Man, QK
   Men, H
   Huo, JT
   Chang, CT
   Wang, XM
   Li, RW
AF Li, Jiawei
   Law, Jia Yan
   Ma, Haoran
   He, Aina
   Man, Qikui
   Men, He
   Huo, Juntao
   Chang, Chuntao
   Wang, Xinmin
   Li, Run-Wei
TI Magnetocaloric effect in Fe-Tm-B-Nb metallic glasses near room
   temperature
SO JOURNAL OF NON-CRYSTALLINE SOLIDS
AB Magnetic properties and magnetocaloric effect in (Fe0.76-xTmxB0.24)(96)Nb-4 (x = 0, 0.01, 0.05, 0.1, 0.17 and 0.18) metallic glasses have been studied. The appropriate substitution of Fe by Tm increases the glass-forming ability of the alloys and allows tuning the Curie temperature (T-C) to near room temperature. A linear relationship between magnetic entropy change and saturation magnetization has been observed. The formation of minority nanocrystalline Tm2Fe14B1 phase has been observed at higher Tm additions, which could influence the magnetocaloric effect of the alloy. These Tm doped Fe-based alloys, with enhanced thermal stability, good magnetocaloric responses and tunable T-C, are good candidates for near room-temperature magnetic refrigerants. (C) 2015 Elsevier B.V. All rights reserved.
RI Law, JiaYan/A-1983-2015; Xia, YuQing/C-9724-2017
OI Law, JiaYan/0000-0002-1431-6773;
SN 0022-3093
EI 1873-4812
PD OCT 1
PY 2015
VL 425
BP 114
EP 117
DI 10.1016/j.jnoncrysol.2015.06.002
UT WOS:000359958700015
ER

PT J
AU Yang, C
   Zhang, C
   Xing, W
   Liu, L
AF Yang, Chong
   Zhang, Cheng
   Xing, Wei
   Liu, Lin
TI 3D printing of Zr-based bulk metallic glasses with complex geometries
   and enhanced catalytic properties
SO INTERMETALLICS
AB Bulk metallic glasses (BMGs) are difficult to manufacture by conventional processes due to the superior strength and extreme brittleness of these materials. Here, we report on the manufacturing of Zr-based BMG components with complex geometries, including cubic, hollow, and lattice structures, by using a selective laser melting (SLM) 3D-printing technique. High amorphous phase fractions between 74 and 90 wt% are achieved depending on the geometries manufactured. To take advantage of complex geometries with large specific surface areas, the lattice BMG samples were selected, as an example, to be dealloyed to obtain a milli/nano hierarchical porous structure, which exhibits excellent catalytic properties towards degradation of methyl orange, showing a promising potential for functional applications.
SN 0966-9795
EI 1879-0216
PD MAR
PY 2018
VL 94
BP 22
EP 28
DI 10.1016/j.intermet.2017.12.018
UT WOS:000425579200004
ER

PT S
AU Evenson, Z
   Schmitt, T
   Nicola, M
   Gallino, I
   Busch, R
AF Evenson, Zach
   Schmitt, Tobias
   Nicola, Mathias
   Gallino, Isabella
   Busch, Ralf
BE Tokuyama, M
   Oppenheim, I
TI High Temperature Melt Viscosity and Fragile-To-Strong Transition in
   Zr-Cu-Ni-Al-Nb(Ti) and Cu47Ti34Zr11Ni8 Bulk Metallic Glasses
SO 4TH INTERNATIONAL SYMPOSIUM ON SLOW DYNAMICS IN COMPLEX SYSTEMS: KEEP
   GOING TOHOKU
SE AIP Conference Proceedings
CT 4th International Symposium on Slow Dynamics in Complex Systems
CY DEC 02-07, 2012
CL Tohoku Univ, Inst Fluid Sci, GCOE, Sendai, JAPAN
SP World Ctr Educ & Res Trans-disciplinary Flow Dynam
HO Tohoku Univ, Inst Fluid Sci, GCOE
AB The molten viscous behavior of the Vitreloy 106, 106a, 105 and 101 bulk metallic glass-forming alloys is investigated at temperatures above T-liq. Couette concentric cylinder viscometry is used to determine the isothermal and continuous cooling melt viscosities. Isothermal three-point beam-bending measurements of the viscosity in the vicinity of T-g reveal a kinetically stronger liquid at low temperatures than at high temperatures near T-liq. This behavior is revealed to be the result of a fragile-to-strong transition in the undercooled liquid, possibly due to polyamorphism.
SN 0094-243X
BN 978-0-7354-1141-8
PY 2013
VL 1518
BP 197
EP 205
DI 10.1063/1.4794568
UT WOS:000317036300023
ER

PT J
AU Li, HX
   Yi, S
AF Li, H. X.
   Yi, S.
TI Corrosion behaviors of bulk metallic glasses
   Fe66.7C7.0S3.3B5.5P8.7Cr2.3Al2.0Mo4.5 having different crystal volume
   fractions
SO MATERIALS CHEMISTRY AND PHYSICS
AB The influence of crystallization on the corrosion resistance of bulk metallic glass Fe66.7C7.0Si3.3B5.5P8.7Cr2.3Al2.0Mo4.5 fabricated using industrial raw materials has been studied in the solutions of 0.5 M H2SO4 and 1N HCl, respectively It was found that corrosion resistance is strongly dependent on the microstructural change caused by annealing treatment. As-cast rods comprising fully amorphous phase exhibit higher corrosion resistance than crystallized rods. (C) 2008 Elsevier B.V. All rights reserved.
SN 0254-0584
PD NOV 15
PY 2008
VL 112
IS 1
BP 305
EP 309
DI 10.1016/j.matchemphys.2008.05.061
UT WOS:000259423200056
ER

PT J
AU Lee, SW
   Lee, CM
   Ahn, JP
   Kim, YC
   Lee, JC
AF Lee, Seok-Woo
   Lee, Chang-Myun
   Ahn, Jae-Pyoung
   Kim, Yu-Chan
   Lee, Jae-Chul
TI A parameter governing the plasticity of Cu-Zr containing bulk metallic
   glasses
SO MATERIALS SCIENCE AND ENGINEERING A-STRUCTURAL MATERIALS PROPERTIES
   MICROSTRUCTURE AND PROCESSING
CT 12th International Conference on Rapidly Quenched and Metastable
   Materials
CY AUG 21-26, 2005
CL Jeju Isl, SOUTH KOREA
AB Experiments were conducted on bulk metallic glasses (BMGs) to identify a crucial factor that governs the degree of the plasticity shown by these alloys. It was found that in situ formation of nanocrystallites, which is known as the deformation-induced nanocrystallization, play a key role in providing initiation sites of the local deformation, indicating that a high degree of plasticity could be achieved from BMGs in which deformation facilitates nanocrystallization. The degree of this deformation-induced nanocrystallization was found to be strongly dependent on the activation energy for crystallization. In this paper, we report that a factor governing the plasticity of BMGs lies in the activation energy for crystallization. (c) 2006 Elsevier B.V. All rights reserved.
RI Lee, Seok-Woo/D-8205-2011; Lee, Jae-Chul/M-8743-2017
OI Lee, Jae-Chul/0000-0002-9294-2163; Lee, Seok-Woo/0000-0001-6752-5694
SN 0921-5093
PD MAR 25
PY 2007
VL 449
BP 172
EP 175
DI 10.1016/j.msea.2006.02.306
UT WOS:000245477800036
ER

PT J
AU Olivetti, E
   Baricco, M
   Ferrara, E
   Tiberto, P
   Martino, L
AF Olivetti, E
   Baricco, M
   Ferrara, E
   Tiberto, P
   Martino, L
TI Effect of annealing on the magnetic properties of Nd70Fe20Al10 bulk
   metallic glasses
SO JOURNAL OF MAGNETISM AND MAGNETIC MATERIALS
CT 2nd Joint European Magnetic Symposia (JEMS 04)
CY SEP 05-10, 2004
CL Dresden, GERMANY
AB In this work, the influence of thermal treatments on the hysteresis behaviour of Nd70Fe20Al10 bulk metallic glasses is studied. Two samples obtained applying different quenching rates have been characterized: (a) master alloy ingots, prepared through arc melting, and (b) cone-shaped ingots obtained by copper mould casting. DSC measurements have been performed on both alloys. Selected samples have been submitted to subsequent annealing. Hard magnetic properties have been observed at room temperature either in the as-east master alloy or in the cone-shaped ingots. High values of coercivity are still observed after treatment at temperatures close to the crystallisation temperature (up to 500 degrees C). The different magnetic behaviour of the samples is discussed in terms of differences in the residual amorphous phase composition. (c) 2004 Elsevier B.V. All rights reserved.
RI Olivetti, Elena/D-3834-2013; Tiberto, Paola Maria/M-8133-2015; Baricco,
   Marcello/B-4075-2013
OI Olivetti, Elena/0000-0003-2210-0072; Tiberto, Paola
   Maria/0000-0002-5432-1788; Baricco, Marcello/0000-0002-2856-9894;
   Tiberto, Paola/0000-0002-7409-2281
SN 0304-8853
EI 1873-4766
PD APR
PY 2005
VL 290
SI SI
BP 1214
EP 1216
DI 10.1016/j.jmmm.2004.11.388
PN 2
UT WOS:000228837900111
ER

PT J
AU Tong, Y
   Qiao, JC
   Zhang, C
   Pelletier, JM
   Yao, Y
AF Tong, Y.
   Qiao, J. C.
   Zhang, C.
   Pelletier, J. M.
   Yao, Y.
TI Mechanical properties of Ti16.7Zr16.7Hf16.7Cu16.7Ni16.7Be16.7
   high-entropy bulk metallic glass
SO JOURNAL OF NON-CRYSTALLINE SOLIDS
AB In the current work, the plasticity at room temperature, as well as the creep and stress relaxation behaviors at high temperature of the Ti16.7Zr16.7Hf16.7Cu16.7Ni16.7Be16.7 high-entropy bulk metallic glass have been investigated. In the compressive testing, the fracture strength can reach 2539 MPa at room temperature and the plasticity of the Ti16.7Zr16.7Hf16.7Cu16.7Ni16.7Be16.7 high-entropy metallic glass is better at the lower strain-rate. The creep property was studied by stress jump tests at different temperatures. It is noted that the Newtonian behavior of the alloy would turn to non-Newtonian at high temperature with increasing of the strain rate. The apparent activation volume of Ti16.7Zr16.7Hf16.7Cu16.7Ni16.7Be16.7 high-entropy metallic glass is smaller than that of traditional metallic glasses. It is found that the heterogeneity of the alloy system will be enhanced as temperature increases near and below the glass transition temperature T-g. (C) 2016 Elsevier B.V. All rights reserved.
RI Yao, Yao/K-3129-2012
OI Yao, Yao/0000-0002-0879-4269
SN 0022-3093
EI 1873-4812
PD NOV 15
PY 2016
VL 452
BP 57
EP 61
DI 10.1016/j.jnoncrysol.2016.08.022
UT WOS:000388050800010
ER

PT S
AU Aydiner, CC
   Ustundag, E
AF Aydiner, CC
   Ustundag, E
BE Dias, AM
   Pina, J
   Batista, AC
   Diogo, E
TI Residual stresses in bulk metallic glasses - I: Modeling
SO ECRS 6: PROCEEDINGS OF THE 6TH EUROPEAN CONFERENCE ON RESIDUAL STRESSES
SE Materials Science Forum
CT 6th European Conference on Residual Stresses (ECRS 6)
CY JUL 10-12, 2002
CL COIMBRA, PORTUGAL
SP Reitoria Univ Coimbra, Fund Ciencia Tecnol, Minist Ciencia Technol, Apoio Programa Operat Ciencia, Tech, Inovacao Quadro Commun Apoio III, Fund Calouste Gulbenkian, Camara Municipal Coimbra, Governo Civil Coimbra, Comiss Coordenac Reg Ctr, Embaicada Franca, BPI
AB The recent advent of multi-component alloys with exceptional glass forming ability has allowed the processing of large metallic specimens with amorphous structure. The possibility of formation of thermal tempering stresses during the processing of these bulk metallic glass (BMG) specimens was first investigated using an "instant freezing model". It was shown that convective cooling of Zr(41.2)Ti(13.8)Cu(12.5)Ni(10)Be(22.5) plates with high heat transfer coefficients could potentially generate significant compressive stresses balanced with mid-plane tension. The predictions of the instant freezing model were then compared to calculations that consider the viscoelastic properties of BMGs.
RI Aydiner, Cahit/O-9618-2017; Ustundag, Ersan/C-1258-2009
OI Aydiner, Cahit/0000-0001-8256-6742; Ustundag, Ersan/0000-0002-0812-7028
SN 0255-5476
BN 0-87849-900-8
PY 2002
VL 404-7
BP 49
EP 54
DI 10.4028/www.scientific.net/MSF.404-407.49
UT WOS:000177256900008
ER

PT J
AU Xia, XX
   Wang, WH
   Greer, AL
AF Xia, Xing-Xiang
   Wang, Wei-Hua
   Greer, A. Lindsay
TI Plastic zone at crack tip: A nanolab for formation and study of metallic
   glassy nanostructures
SO JOURNAL OF MATERIALS RESEARCH
AB We report that various metallic glassy nanostructures including nanoridges, nanocones, nanowires, nanospheres, and nanoscale-striped patterns are spontaneously formed on the fracture surface of bulk metallic glasses at room temperature. A clear correlation between the dimensions of these nanostructures and the size of the plastic zone at the crack tip has been found, providing a way to control nanostructure sizes by controlling the plastic zone size intrinsically or extrinsically. This approach to forming metallic glassy nanostructures also has implications for understanding the deformation and fracture mechanisms of metallic glasses.
RI Greer, Lindsay/E-9433-2017; Greer, Alan Lindsay/G-1977-2011
SN 0884-2914
EI 2044-5326
PD SEP
PY 2009
VL 24
IS 9
BP 2986
EP 2992
DI 10.1557/JMR.2009.0362
UT WOS:000269585300031
ER

PT J
AU Yavari, AR
   Botta, WJ
   Rodrigues, CAD
   Greer, AL
   Uriate, JL
   Huenen, G
   Vaughan, G
   Inoue, A
AF Yavari, AR
   Botta, WJ
   Rodrigues, CAD
   Greer, AL
   Uriate, JL
   Huenen, G
   Vaughan, G
   Inoue, A
TI FeNiB-based metallic glasses with fcc crystallisation products
SO JOURNAL OF NON-CRYSTALLINE SOLIDS
CT 5th Brazillian Symposium on Glasses and Related Materials/1st
   International Symposium on Non-Crystalline Solids in Brazil
CY AUG 19-22, 2001
CL FOZ DO IGUACU, BRAZIL
AB Bulk metallic glasses (MGs) are currently subject of intense research due to their unique mechanical properties: Newtonian viscous formability between the glass transition temperature T-g and crystallisation temperature T-x and elastic strain range of well over 1% below T-g. However, some MGs are brittle. Fe-based MGs and some others are prone to thermal embrittlement even during the quench. Compositional adjustments in the design of FeB-based alloys have allowed preparation of metalloid poor Fe40Co5Ni35Zr5B15 that has gamma-Fe as a primary crystallisation product for obtaining ductility of metallic glass-matrix toughened by gamma-Fe crystallisation. Structural characterisation using diffraction of high energy synchrotron light, transmission electron microscopy, scanning electron microscopy are presented together with hardness and wear data. (C) 2002 Elsevier Science B.V. All rights reserved.
RI Vaughan, Gavin/B-1479-2010; Rodrigues, Cesar/B-5885-2014; Botta,
   Walter/E-7763-2010; Greer, Lindsay/E-9433-2017; yavari,
   alain/E-8192-2010; Greer, Alan Lindsay/G-1977-2011; Inoue,
   Akihisa/E-5271-2015
OI Botta, Walter/0000-0003-2759-573X; rodrigues, cesar augusto
   duarte/0000-0002-0352-549X
SN 0022-3093
PD JUN
PY 2002
VL 304
IS 1-3
BP 44
EP 50
AR PII S0022-3093(02)01002-5
DI 10.1016/S0022-3093(02)01002-5
UT WOS:000176121200008
ER

PT J
AU Kazimirov, VY
   Louca, D
   Widom, M
   Gu, XJ
   Poon, SJ
   Shiflet, GJ
AF Kazimirov, V. Yu.
   Louca, Despina
   Widom, M.
   Gu, X. -J.
   Poon, S. J.
   Shiflet, G. J.
TI Local organization and atomic clustering in multicomponent amorphous
   steels
SO PHYSICAL REVIEW B
AB The characteristics of atom packing in multicomponent metallic alloys with distinct short- and medium-range order (SMRO) are notoriously difficult to provide as the local structure is made up of multiple overlapping pair correlations. In the case of binary glasses, several cluster models have been proposed that can describe the SMRO well going beyond models of dense random packing of hard spheres. By combining the pair density function analysis of neutron-diffraction data and modeling using ab initio computational techniques, the tendency for local atomic clustering is investigated and properties of multicomponent bulk Fe(78-x-y)TM(x)RE(y)(C(22-z)B(z)) amorphous alloys [transition metal (TM) and rare earth (RE)] are studied going beyond simple binary systems. The results are relevant to most bulk metallic glass forming alloys with more than one kind of solute ion.
RI Widom, Michael/P-2531-2014; Kazimirov, Viacheslav/O-1834-2013
OI Widom, Michael/0000-0001-5972-5696;
SN 1098-0121
PD AUG
PY 2008
VL 78
IS 5
AR 054112
DI 10.1103/PhysRevB.78.054112
UT WOS:000259368200038
ER

PT B
AU Nishikawa, H
   Naoi, T
   Saito, M
   Mizuno, J
   Fukuhara, M
AF Nishikawa, H.
   Naoi, T.
   Saito, M.
   Mizuno, J.
   Fukuhara, M.
BE Fujii, H
TI Contact angle between Sn-Ag-Cu solder and electroplated Cu-based Bulk
   Metallic Glass
SO PROCEEDINGS OF THE 1ST INTERNATIONAL JOINT SYMPOSIUM ON JOINING AND
   WELDING
CT 1st International Joint Symposium on Joining and Welding
CY NOV 06-08, 2013
CL Osaka, JAPAN
SP Osaka Univ, Joining & Welding Res Inst, TWI, Ohio State Univ, Edison Welding Inst
AB Bulk metallic glasses (BMGs), which are non-crystalline metallic alloys, can be fabricated with low cooling rates in many alloy systems. So, it is possible to produce BMGs that are several centimeters thick. BMGs exhibit useful engineering properties of high mechanical strength and high corrosion resistance. In order to extend the engineering application of BMGs, it is necessary to establish appropriate joint process of BMG / BMG and BMG / crystalline metal. In this study, surface treatment using hydrofluoric (HF) solution and copper electroplating were performed and the spread test of the Sn-3.0Ag-0.5Cu solder was performed to clarify wetting behavior of the solder on electroplated Cu60Zr30Ti10 BMG.
BN 978-1-78242-164-1; 978-1-78242-163-4
PY 2013
BP 553
EP 556
UT WOS:000347899800090
ER

PT J
AU Kolomytsev, V
   Pasko, A
   Sezonenko, A
AF Kolomytsev, V
   Pasko, A
   Sezonenko, A
TI Selection rules for the production of bulk metallic glass precursors for
   shape memory materials
SO ARCHIVES OF METALLURGY AND MATERIALS
AB The paper is focused on the development of the following technological paths: (metallic melt) -> (bulk metallic glass precursor of shape memory material) -> (superplastic deformation on heating through the supercooled liquid region) -> (crystallization and final microcrystalline material with martensitic transformation in a predetermined temperature range). Main attention is paid to design of multicomponent (Ti,ZrHf)(A)(Co,Ni,Cu,Pd)(B) alloys with compositions varied around an effective intermetallic compound AB (A:B = 45:55; 50:50; 55:45). One of the challenging problems here is to find a chemical composition window of our multicomponent systems fitting the known empirical rules for a high amorphization ability and keeping the martensitic transformation and good shape memory properties after crystallization.
RI Pasko, Alexandre/C-9529-2011
OI Pasko, Alexandre/0000-0002-8047-1122
SN 0860-7052
PY 2004
VL 49
IS 4
BP 825
EP 840
UT WOS:000227653700009
ER

PT J
AU Jun, HJ
   Lee, KS
   Kim, CP
   Chang, YW
AF Jun, H. -J.
   Lee, K. S.
   Kim, C. P.
   Chang, Y. W.
TI Ductility enhancement of a Ti-based bulk metallic glass through
   annealing treatment below the glass transition temperature
SO INTERMETALLICS
AB The effect of annealing treatment on mechanical properties has been investigated for a Ti-based bulk metallic glass (BMG). A series of compression tests as well as Vickers hardness tests has thus been performed at room temperature for the BMG specimens annealed in an Ar atmosphere at several temperatures near the glass transition temperature (T-g). The ductility under compression was found to increase up to about 42% without reducing the maximum strength, but with higher hardness values. Thermal properties were also analyzed by using a differential scanning calorimetry (DSC), while microstructures of annealed specimens were studied through X-ray diffraction (XRD) and also a high resolution transmission electron microscopy (HRTEM). (C) 2011 Elsevier Ltd. All rights reserved.
SN 0966-9795
EI 1879-0216
PD JAN
PY 2012
VL 20
IS 1
BP 47
EP 54
DI 10.1016/j.intermet.2011.08.010
UT WOS:000297889300007
ER

PT J
AU Choi-Yim, H
   Busch, R
   Koster, U
   Johnson, WL
AF Choi-Yim, H
   Busch, R
   Koster, U
   Johnson, WL
TI Synthesis and characterization of particulate reinforced
   Zr57Nb5Al10Cu15.4Ni12.6 bulk metallic glass composites
SO ACTA MATERIALIA
AB The Zr57Nb5Al10Cu15.4Ni12.6 bulk metallic glass forming liquid is reinforced with WC, SiC, W, or Ta particles. Structure, microstructure and thermal stability of the composites are studied by X-ray diffraction, optical microscopy and differential scanning calorimetry. The metallic glass matrix remains amorphous after adding up to 20 vol.% of particles. The reactions at the interfaces between the matrix and the different reinforcing materials are investigated with scanning electron microscopy, transmission electron microscopy and electron microprobe. The mechanical properties of the composites are studied in compression and tension. The influence of the introduced particles on the thermal stability of the matrix as well as on the mechanical properties is discussed. (C) 1999 Acta Metallurgica Inc. Published by Elsevier Science Ltd. All rights reserved.
SN 1359-6454
PD JUN 22
PY 1999
VL 47
IS 8
BP 2455
EP 2462
DI 10.1016/S1359-6454(99)00103-2
UT WOS:000081439600015
ER

PT J
AU Ai, K
   Dai, LH
AF Ai, Ke
   Dai, LanHong
TI Numerical study of pile-up in bulk metallic glass during spherical
   indentation
SO SCIENCE IN CHINA SERIES G-PHYSICS MECHANICS & ASTRONOMY
AB Pile-up around indenter is usually observed during instrumented indentation tests on bulk metallic glass. Neglecting the pile-up effect may lead to errors in evaluating hardness, Young's modulus, stress-strain response, etc. Finite element analysis was employed to implement numerical simulation of spherical indentation tests on bulk metallic glass. A new model was proposed to describe the pile-up effect. By using this new model, the contact radius and hardness of Zr41.2Ti13.8Cu12.5Ni10Be22.5 bulk metallic glass were obtained under several different indenter loads with pile-up, and the results agree well with the data generated by numerical simulation.
SN 1672-1799
PD APR
PY 2008
VL 51
IS 4
BP 379
EP 386
DI 10.1007/s11433-008-0043-4
UT WOS:000254406000006
ER

PT S
AU Johnson, WL
AF Johnson, WL
BE Schulz, R
TI Fundamental aspects of bulk metallic glass formation in multicomponent
   alloys
SO METASTABLE, MECHANICALLY ALLOYED AND NANOCRYSTALLINE MATERIALS, PTS 1
   AND 2
SE MATERIALS SCIENCE FORUM
CT International Symposium on Metastable, Mechanically Alloyed and
   Nanocrystalline Materials (ISMANAM-95)
CY JUL 24-28, 1995
CL QUEBEC CITY, CANADA
SP Hydro Quebec, Laval Univ, McGill Univ, Ctr Phys Mat, Nat Sci & Engn Res Council Canada, Zoz GmbH, Siemens, Xerox Canada Ltee, Precitech
AB During the past several years, a number of multicomponent alloy families have been investigated in which the liquid alloys form metallic glass at cooling rates below 1000 K/s and as low as 1 K/s or less. These ''bulk'' metallic glass forming alloys have been cast from the melt into glass samples with the smallest dimension ranging from millimeters to centimeters. The undercooled liquid alloys show remarkable resistance to crystallization permitting studies of thermal and physical properties, the glass transition, and crystallization behavior of the melt over previously inaccessible temperatures in the deeply undercooled region. One group of these alloys, which includes the Zr-Ti-Ni-Cu-Be and Zr-Ti-Ni-Cu systems, has been extensively investigated in our laboratory. Results of atomic diffusion and viscosity measurements, crystallization behavior and TTT-diagrams, and studies of liquid phase separation in the deeply undercooled Liquid have been carried out and will be discussed. Engineering properties and potential applications of these bulk glasses will be briefly mentioned.
SN 0255-5476
BN 0-87849-738-2
PY 1996
VL 225
BP 35
EP 49
DI 10.4028/www.scientific.net/MSF.225-227.35
UT WOS:A1996BG37Y00005
ER

PT J
AU Sun, HY
   Wang, JS
   Cao, LJ
   Xue, QG
AF Sun, Haoyan
   Wang, Jingsong
   Cao, Lijun
   Xue, Qingguo
TI Effect of electropulsing treatment on the crystallization kinetics of
   zr(55)Ni(5)Al(10)Cu(30) bulk metallic glass
SO THERMOCHIMICA ACTA
AB Zr55Ni5Al10Cu30 bulk metallic glass with a diameter of 3 mm was prepared by copper-mold suction casting combined with covering flux technology under non-vacuum conditions. The non-isothermal crystallization kinetics of Zr55Ni5Al10Cu30 bulk metallic glass prepared with and without electropulsing treatment on the molten alloy have been studied using the modified JMA equation. Results show that, after electropulsing treatment, the glass transition temperature increases, while the crystallization temperatures and supercooled liquid range both decrease. The activation energies of glass transition and crystallization become reduced and the Avrami exponents of crystallization become raised, indicating that the glass transition and crystallization of Zr55Ni5Al10Cu30 bulk metallic glass are promoted by electropulsing treatment. (C) 2012 Elsevier B.V. All rights reserved.
SN 0040-6031
PD JUN 10
PY 2012
VL 537
BP 80
EP 85
DI 10.1016/j.tca.2012.03.011
UT WOS:000304026100013
ER

PT J
AU Li, B
   Li, ZY
   Xiong, JG
   Xing, L
   Wang, D
   Li, Y
AF Li, B
   Li, ZY
   Xiong, JG
   Xing, L
   Wang, D
   Li, Y
TI Laser welding of Zr45Cu48Al7 bulk glassy alloy
SO JOURNAL OF ALLOYS AND COMPOUNDS
AB Using a laser welding method, we have successfully welded two pieces of bulk metallic glasses based on Zr45CU48Al7 without crystallization. No crystallization was observed and no visible defect of crack was recognized in the bead and heated-affected zone (HAZ) under the condition of welding scanning speed of 8 m/min with an output laser power of 1200 W. Our analysis of the temperature profile the thermal cycle (temperature history) in the bead and HAZ indicates that the temperatures in the bead and HAZ rapidly decreased to below 430 degrees C resulted in glass formation from the melt in the bead and non-crystallization in the HAZ in the sample scanned at 8 m/min. (c) 2005 Elsevier B.V. All rights reserved.
SN 0925-8388
PD MAR 9
PY 2006
VL 413
IS 1-2
BP 118
EP 121
DI 10.1016/j.jallcom.2005.07.005
UT WOS:000236312900020
ER

PT J
AU de Oliveira, MF
   Pereira, FS
   Bolfarini, C
   Kiminami, CS
   Botta, WJ
AF de Oliveira, M. F.
   Pereira, F. S.
   Bolfarini, C.
   Kiminami, C. S.
   Botta, W. J.
TI Topological instability, average electronegativity difference and glass
   forming ability of amorphous alloys
SO INTERMETALLICS
CT 6th International Conference on Bulk-Metallic Glasses (BMG-VI)
CY MAY 11-15, 2008
CL Xian, PEOPLES R CHINA
AB In this paper, we report the remarkable agreement of the glass forming ability of binary alloys with a new criterion that combines the topological instability parameter (lambda) and the average electronegativity difference among the elements of an alloy, assuming both exert a synergetic effect. The best glass forming compositions for Zr-Cu and Ti-Ni systems are well predicted by this new approach. Although the new criterion needs further refinement, it is concluded that the proposed approach is a promising and simple tool to guide and reduce the tedious and labour intensive work to find good glass former compositions in metallic systems. (C) 2008 Elsevier Ltd. All rights reserved.
RI Bolfarini, Claudemiro/E-4366-2012; Botta, Walter/E-7763-2010; KIMINAMI,
   CLAUDIO/D-4402-2012; de Oliveira, Marcelo/B-9881-2012
OI Botta, Walter/0000-0003-2759-573X; KIMINAMI,
   CLAUDIO/0000-0001-8231-7316; de Oliveira, Marcelo/0000-0003-4589-2463;
   Bolfarini, Claudemiro/0000-0002-3099-3694
SN 0966-9795
EI 1879-0216
PD APR
PY 2009
VL 17
IS 4
BP 183
EP 185
DI 10.1016/j.intermet.2008.09.013
UT WOS:000264728400001
ER

PT J
AU Shen, TD
   Schwarz, RB
AF Shen, TD
   Schwarz, RB
TI Bulk ferromagnetic glasses prepared by flux melting and water quenching
SO APPLIED PHYSICS LETTERS
AB Several ferromagnetic bulk amorphous alloys of the type Fe-(Co, Cr, Mo, Ga, Sb)-P-B-C, containing between 62 and 71 at. % Fe, have been prepared in the form of 4-mm-diam rods. The glass synthesis consists of mechanically alloying the constituents, purifying the melts in B2O3 flux inside fused silica tubes, and quenching the melts at cooling rates on the order of 100 K/s. All these glasses have a large supercooled region T-x-T-g, ranging from 35 to 61 K, within which the glass can be shaped under a relatively small applied load. It is shown that the value of T-x-T-g depends strongly on the metalloid composition. These bulk metallic glasses have very low coercivity and low hysteresis losses. (C) 1999 American Institute of Physics. [S0003-6951(99)00927-4].
SN 0003-6951
PD JUL 5
PY 1999
VL 75
IS 1
BP 49
EP 51
DI 10.1063/1.124273
UT WOS:000081169300017
ER

PT J
AU Yao, JH
   Hostert, C
   Music, D
   Frisk, A
   Bjorck, M
   Schneider, JM
AF Yao, J. H.
   Hostert, C.
   Music, D.
   Frisk, A.
   Bjorck, M.
   Schneider, J. M.
TI Synthesis and mechanical properties of Fe-Nb-B thin-film metallic
   glasses
SO SCRIPTA MATERIALIA
AB Fe-Nb-B. thin-film metallic glasses (TFMGs) were synthesized via a combinatorial sputtering approach to probe the property-composition correlation. The boron content was found to dominate the mechanical properties of the TFMGs. The similar to 10% smaller strength of Fe-Nb-B TFMGs compared to existing bulk metallic glass with similar composition may be attributed to the absence of a network-like structure based on (Fe,M)(23)B-6 phase due to the extreme quenching conditions employed. (C) 2012 Acta Materialia Inc. Published by Elsevier Ltd. All rights reserved.
RI Music, Denis/C-2978-2012; Schneider, Jochen/A-4701-2012
OI Music, Denis/0000-0003-2303-3676; Bjorck, Matts/0000-0002-9358-4791;
   Frisk, Andreas/0000-0001-9234-0907
SN 1359-6462
PD JUL
PY 2012
VL 67
IS 2
BP 181
EP 184
DI 10.1016/j.scriptamat.2012.04.011
UT WOS:000305771700016
ER

PT J
AU Guo, GQ
   Yang, L
   Wu, SY
AF Guo, G. Q.
   Yang, L.
   Wu, S. Y.
TI Structural investigation upon the high glass-forming ability in Fe doped
   ZrCuAl multicomponent alloys
SO INTERMETALLICS
AB Understanding the underlying mechanism of microalloying affecting the glass formation in multicomponent alloys is scientifically required, while addressing this issue remains a challenge. In this work, the structural mechanisms of extraordinarily high glass-forming ability (GFA) caused by Fe doping in ZrCuAl bulk metallic glasses was investigated via synchrotron radiation techniques combined with simulations. It is revealed that when minor Fe (5 at.%) are doped, the local structures (clusters) have relatively high values of some structural parameters, in terms of fivefold symmetry, geometrical regularity, atomic packing, and chemical interaction between heterogeneous atoms. The combination of these structural factors leads to stabilized amorphous structure and enhanced GFA. This work may extend our understanding on the sensitive dependence of GFA on the concentration of doping atoms in multicomponent metallic glasses. (C) 2015 Elsevier Ltd. All rights reserved.
SN 0966-9795
EI 1879-0216
PD APR
PY 2016
VL 71
BP 24
EP 30
DI 10.1016/j.intermet.2015.11.008
UT WOS:000370915100005
ER

PT J
AU Ichitsubo, T
   Matsubara, E
   Saida, J
   Chen, HS
   Nishiyama, N
   Yamamoto, T
AF Ichitsubo, T.
   Matsubara, E.
   Saida, J.
   Chen, H. S.
   Nishiyama, N.
   Yamamoto, T.
TI Mechanism of ultrasound-induced structural instability in bulk metallic
   glasses
SO REVIEWS ON ADVANCED MATERIALS SCIENCE
CT 13th International Symposium on Metastable and Nano-Materials
   (ISMANAM-2006)
CY AUG 27-31, 2006
CL Warsaw Univ Technol, Fac Mat Sci & Engn, Warsaw, POLAND
HO Warsaw Univ Technol, Fac Mat Sci & Engn
AB This work devotes to consider an underlying mechanism of the ultrasound-induced instability phenomenon observed for some metallic glasses. Mechanical spectroscopy analysis strongly suggests that the instability is caused by that atomic motions associated with the. relaxation are resonant with the periodic ultrasonic-strain field. It is reasonable to consider that such atomic motions at temperatures lower than the kinetic freezing temperature T(g) originates from relatively weakly bonded regions in an inhomogeneous microstructure of glass.
RI Nishiyama, Nobuyuki/C-8228-2015; Ichitsubo, Tetsu/F-3201-2010; Saida,
   Junji/C-1912-2009; Yamamoto, Tokujiro/A-8827-2011
OI Ichitsubo, Tetsu/0000-0002-1127-3034
SN 1606-5131
PD MAY
PY 2008
VL 18
IS 1
BP 37
EP 40
UT WOS:000256370800010
ER

PT J
AU Choi-Yim, H
   Conner, RD
   Johnson, WL
AF Choi-Yim, H
   Conner, RD
   Johnson, WL
TI In situ composite formation in the Ni-(Cu)-Ti-Zr-Si system
SO SCRIPTA MATERIALIA
AB In situ composites were synthesized by arc melting Ni-(Cu)-Ti-Zr-Si alloys. The X-ray diffraction patterns of rapidly cooled cast strips show a primary Ni(Ti,Zr) B2 structure superimposed on the diffuse scattering maxima from the amorphous phase. Compression test results show that the composite starts to yield at 1200 MPa and fractures at 1900 MPa. (c) 2005 Acta Materialia Inc. Published by Elsevier Ltd. All rights reserved.
SN 1359-6462
PD DEC
PY 2005
VL 53
IS 12
BP 1467
EP 1470
DI 10.1016/j.scriptamat.2005.08.011
UT WOS:000232700500027
ER

PT J
AU Remenyi, G
   Biljakovic, K
   Staresinic, D
   Dominko, D
   Ristic, R
   Babic, E
   Figueroa, IA
   Davies, HA
AF Remenyi, G.
   Biljakovic, K.
   Staresinic, D.
   Dominko, D.
   Ristic, R.
   Babic, E.
   Figueroa, I. A.
   Davies, H. A.
TI Looking for footprint of bulk metallic glass in electronic and phonon
   heat capacities of Cu55Hf45-xTix alloys
SO APPLIED PHYSICS LETTERS
AB We report on the heat capacity investigation of Cu55Hf45-xTix metallic glasses. The most appropriate procedure to estimate low temperature electronic and phonon contributions has been determined. Both contributions exhibit monotonous Ti concentration dependence, demonstrating that there is no relation of either the electron density of states at the Fermi level or the Debye temperature to the increased glass forming ability in the Ti concentration range x = 15-30. The thermodynamic parameters (e. g., reduced glass temperature) remain better indicators in assessing the best composition for bulk metallic glass formation. (C) 2014 AIP Publishing LLC.
RI Staresinic, Damir/M-3714-2015; Dominko, Damir/R-5027-2016
OI Staresinic, Damir/0000-0002-7379-8717; Dominko,
   Damir/0000-0003-2818-0771
SN 0003-6951
EI 1077-3118
PD APR 28
PY 2014
VL 104
IS 17
AR 171906
DI 10.1063/1.4875043
UT WOS:000336142500031
ER

PT S
AU Kawaguchi, T
   Li, FW
   Zhu, SJ
   Wang, YM
   Dong, C
AF Kawaguchi, T.
   Li, F. W.
   Zhu, S. J.
   Wang, Y. M.
   Dong, C.
BE Zhu, S
   Ni, B
   Ju, DY
TI Effect of Chemical Composition on Fracture Behavior in ZrNiAl Metal
   Glass
SO ADVANCED MATERIALS SCIENCE AND TECHNOLOGY, (IFAMST-8)
SE Materials Science Forum
CT 8th International Forum on Advanced Materials Science and Technology
   (IFAMST-8)
CY AUG 01-04, 2012
CL Fukuoka Inst Technol, Fukuoka City, JAPAN
SP Saitama Inst Technol, Chinese Mat Res Soc Japan, Chinese Acad & Profess Assoc Japan
HO Fukuoka Inst Technol
AB Compressive tests of ZrNiAlbulk metallic glass (BMG) were carried out to investigate the compositional dependence of mechanical properties. The strength increased but the ductility decreased with an increase in Al content. Moreover, it was confirmed that the formation of multiple shear bands became difficult with an increase in glass forming ability.
SN 0255-5476
PY 2013
VL 750
BP 19
EP +
DI 10.4028/www.scientific.net/MSF.750.19
UT WOS:000319235900005
ER

PT J
AU Ma, D
   Chang, YA
AF Ma, D
   Chang, YA
TI Competitive formation of ternary metallic glasses
SO ACTA MATERIALIA
AB In an effort to elucidate the formation of metallic glasses and glass-matrix composites in ternary eutectic systems, we have applied the competitive growth principle with the premise that glass formation competes with the growth of all the possible crystalline structures. It is found that each crystalline structure defines a characteristic glass-forming velocity beyond which its growth will be completely suppressed by glass formation. A glass-forming composition diagram, as thus derived, indicates that the formation of a full glass is limited by the complete suppression of growth of the primary competing structure that has the greatest characteristic glass-forming velocity. Moreover, the formation of two types of glass-matrix composites, i.e., single-phase dendrites plus a glass, and single-phase dendrites plus a two-phase eutectic plus a glass, has been predicted as a result of the incomplete suppression of growth of the primary and/or secondary competing structures. A new criterion gauging the globally optimum glass-forming ability in a ternary eutectic system is also derived. (c) 2006 Acta Materialia Inc. Published by Elsevier Ltd. All rights reserved.
RI Ma, Dong/G-5198-2011
OI Ma, Dong/0000-0003-3154-2454
SN 1359-6454
EI 1873-2453
PD APR
PY 2006
VL 54
IS 7
BP 1927
EP 1934
DI 10.1016/j.actamat.2005.12.015
UT WOS:000236948700023
ER

PT J
AU Wang, WH
   Wen, P
   Wang, LM
   Zhang, Y
   Pan, MX
   Zhao, DQ
   Wang, RJ
AF Wang, WH
   Wen, P
   Wang, LM
   Zhang, Y
   Pan, MX
   Zhao, DQ
   Wang, RJ
TI Equation of state of bulk metallic glasses studied by an ultrasonic
   method
SO APPLIED PHYSICS LETTERS
AB The acoustic velocities and their pressure dependence of various Zr- and Pd-based bulk metallic glasses (BMGs) have been measured by using a pulse echo overlap method. The elastic constants as well as their pressure dependence of the BMGs have been determined, and the equation of state of these BMGs was obtained and compared to that of other glasses and crystalline solids. The structural characteristic of the BMGs is discussed. (C) 2001 American Institute of Physics.
RI ZHANG, Yong/B-7928-2009
OI ZHANG, Yong/0000-0002-6355-9923
SN 0003-6951
PD DEC 10
PY 2001
VL 79
IS 24
BP 3947
EP 3949
DI 10.1063/1.1426272
UT WOS:000172489100014
ER

PT J
AU Battezzati, L
   Mortarino, GM
AF Battezzati, Livio
   Mortarino, G. M.
TI On thermophysical and mechanical properties of glass-forming alloys
SO JOURNAL OF ALLOYS AND COMPOUNDS
CT 14th International Symposium on Metastable and Nano-Materials
CY AUG 26-30, 2007
CL Corfu, GREECE
AB This contribution reviews the relationships between thermophysical properties of glass-forming alloys: glass transition, specific heat, entropy of fusion, fragility indexes, using recent models for ranking undercooled liquids and glasses. These rely on both empirical correlations and on the description of local minima in potential energy landscapes of the material. Then, the relationships between mechanical properties and some of the above quantities are considered and discussed with the aim of getting insight into the mechanism of shear band propagation during mechanical failure when, following up a shear offset event, a local temperature rise occurs and the shear band matures to give a runaway crack. (C) 2008 Elsevier B.V. All rights reserved.
OI Battezzati, Livio/0000-0003-1628-0409
SN 0925-8388
EI 1873-4669
PD AUG 26
PY 2009
VL 483
IS 1-2
BP 222
EP 226
DI 10.1016/j.jallcom.2008.08.099
UT WOS:000270619600055
ER

PT J
AU Wang, YL
   Ma, E
   Xu, J
AF Wang, Yan-Ling
   Ma, Evan
   Xu, Jian
TI Bulk metallic glass formation near the TiCu-TiNi pseudo-binary eutectic
   composition
SO PHILOSOPHICAL MAGAZINE LETTERS
AB No bulk metallic glass (BMG) has been observed so far in the Ti-Cu binary system. It has been found that by choosing a pseudo-binary eutectic (L -> TiCu + TiNi) deeper than that in the binary, reachable via only small substitution of Cu with Ni, the glass-forming ability can be effectively elevated. BMGs of 1-1.5mm diameter have been formed over a composition range around Ti50Cu43Ni7. The new BMGs are reproducibly malleable with significant compressive plastic strains.
RI Ma, En/A-3232-2010
SN 0950-0839
PD MAY
PY 2008
VL 88
IS 5
BP 319
EP 325
DI 10.1080/09500830801968583
UT WOS:000256968500001
ER

PT J
AU Hofmann, DC
   Suh, JY
   Wiest, A
   Johnson, W
AF Hofmann, Douglas C.
   Suh, Jin-Yoo
   Wiest, Aaron
   Johnson, William
TI New processing possibilities for highly toughened metallic glass matrix
   composites with tensile ductility
SO SCRIPTA MATERIALIA
AB New processing possibilities are explored for a semi-solidly-processed bulk metallic glass matrix composite based on the ZrTiNb-CuBe system. Thermoplastic processing in the supercooled liquid region, extensive room temperature cold-rolling, and tensile ductility with work-hardening are all achieved. This research demonstrates that bulk metallic glass matrix composites have potential that far exceeds high tensile ductility and ultra-high toughness. (C) 2008 Acta Materialia Inc. Published by Elsevier Ltd. All rights reserved.
OI Suh, Jin-Yoo/0000-0003-3786-6652
SN 1359-6462
PD OCT
PY 2008
VL 59
IS 7
BP 684
EP 687
DI 10.1016/j.scriptamat.2008.05.046
UT WOS:000258968300003
ER

PT J
AU Agarwal, PC
AF Agarwal, P. C.
TI Phonon dispersion in bulk metallic glass Zr55Cu30Al10Ni15
SO JOURNAL OF NON-CRYSTALLINE SOLIDS
AB The expressions for the phonon frequencies of Zr55CU30Al10Ni15 bulk metallic glass employing a simple model given by Bhatia and Singh for a hypothetical one-component metallic glass are derived both for longitudinal and transverse modes of excitations. The model assumes a central force, effective between the nearest neighbours, and a volume dependent force. Both types of excitations of phonons are computed for the Zr55CU30Al10Ni15 bulk metallic glass for the first time both for self-consistent screening of conduction electrons with and without the inclusion of correlation effects. Phonon frequency expressions reproduce the main characteristic features of the dispersion curves. The theoretical results predicted are in a good agreement with available experimental data of different quaternary bulk metallic glasses having same constituents. These are also compared to the theoretical results of a quaternary glass to have an insight of the structural behavior of the glass under consideration. (c) 2006 Elsevier B.V. All rights reserved.
SN 0022-3093
PD SEP 1
PY 2006
VL 352
IS 30-31
BP 3196
EP 3199
DI 10.1016/j.jnoncrysol.2006.05.018
UT WOS:000240211100005
ER

PT J
AU Ren, S
   Chen, D
   Zhao, XY
AF Ren, Shuai
   Chen, Ding
   Zhao, Xiuyuan
TI Effect of ceramic rolling on the mechanical properties of
   Ti42.5Cu42.5Ni10Zr5 bulk metallic glass composite
SO MATERIALS SCIENCE AND ENGINEERING A-STRUCTURAL MATERIALS PROPERTIES
   MICROSTRUCTURE AND PROCESSING
AB Prior plastic deformation processing, ceramic rolling, was utilized to tune mechanical properties of Ti42.5Cu42.5Ni10Zr5 bulk metallic glass (BMG) composite. The metallic glass composite exhibits plastic deformation with 15% thickness reduction ratio through ceramic rolling at room temperature. The mechanical properties of the Ti-based BMG composite can be improved by cold rolling, which the compressive strength rises 140 MPa and the cold-rolled sample exhibits enhanced plasticity of 5.3%. Such improvement in mechanical properties is attributed to the hydrostatic stress during ceramic rolling and the interaction between the crystal phases and pre-introduced shear bands. (C) 2015 Elsevier B.V. All rights reserved.
```

RI 陈(chen), 鼎(ding)/O-5087-2015

OI 陈(chen), 鼎(ding)/0000-0003-0407-7542

```
SN 0921-5093
EI 1873-4936
PD OCT 14
PY 2015
VL 646
BP 90
EP 95
DI 10.1016/j.msea.2015.08.057
UT WOS:000362132000012
ER

PT J
AU Yang, DK
   Wen, CE
   Han, FS
   Yang, YJ
AF Yang, Dengke
   Wen, Cui'e
   Han, Fusheng
   Yang, Yujie
TI Phase formation and physical properties of mechanically alloyed
   amorphous 55Mg35Ni10Si
SO JOURNAL OF NON-CRYSTALLINE SOLIDS
AB Amorphous 55Mg35Ni10Si alloy powder has been synthesized by mechanical alloying technique using pure Mg, Ni and Si elemental powders. The transformation of the crystalline powders into an amorphous one has been investigated by X-ray diffraction, scanning electron microscopy, transmission electron microscopy and differential scanning calorimetry. The new material produced has a higher thermal stability than reported results, which is beneficial to the fabrication of Mg-Ni-Si bulk amorphous components through powder metallurgy. (c) 2006 Elsevier B.V. All rights reserved.
OI Han, Fusheng/0000-0002-6030-6509; Wen, Cuie/0000-0001-8008-3536
SN 0022-3093
PD SEP 1
PY 2006
VL 352
IS 30-31
BP 3244
EP 3248
DI 10.1016/j.noncrysol.2006.05.027
UT WOS:000240211100012
ER

PT J
AU Flores, KM
   Dauskardt, RH
AF Flores, KM
   Dauskardt, RH
TI Fracture and deformation of bulk metallic glasses and their composites
SO INTERMETALLICS
CT International Symposium on Intermetallic and Advanced Materials
CY MAR 02-06, 2003
CL San Diego, CA
AB Bulk metallic glasses represent an exciting new class of materials with a wide range of potential applications. Plastic deformation of metallic glasses occurs by the formation of shear bands; however, the effect of stress state and the extent of local structural rearrangement during flow are under active investigation. In this work, we review our investigations of the mode II deformation and fracture behavior of a zirconium-based bulk metallic glass. The mode II fracture toughness was found to be similar to75 MParootm, similar to4-5 times larger than the mode I toughness. This may be explained by the sensitivity of flow to normal stresses. The fracture and fatigue behavior of a BMG composite utilizing a ductile reinforcement phase has also been examined and compared with that of the monolithic alloy. The second phase blocks the propagation of shear bands and distributes the plastic deformation, resulting in extensive stable crack growth at stress intensities double the intrinsic toughness of the unreinforced glass. The composite also exhibits an improved fatigue endurance limit. Implications for future composite microstructures are discussed. (C) 2004 Elsevier Ltd. All rights reserved.
SN 0966-9795
PD JUL-SEP
PY 2004
VL 12
IS 7-9
SI SI
BP 1025
EP 1029
DI 10.1016/j.intermet.2004.05.004
UT WOS:000223396900048
ER

PT J
AU Park, JS
   Lim, HK
   Park, ES
   Shin, HS
   Lee, WH
   Kim, WT
   Kim, DH
AF Park, JS
   Lim, HK
   Park, ES
   Shin, HS
   Lee, WH
   Kim, WT
   Kim, DH
TI Fracture behavior of bulk metallic glass/metal laminate composites
SO MATERIALS SCIENCE AND ENGINEERING A-STRUCTURAL MATERIALS PROPERTIES
   MICROSTRUCTURE AND PROCESSING
AB The fracture behaviors of laminated composites of bulk metallic glass (BMG (Vitreloy 1))/crystalline layer have been examined. The composites stacked by BMG/thin Zr layer (25 mu m) have been bonded by electron-discharge bonding technique. The fracture tests have been carried out by non-standard self-designed subsize-charpy tester. When the number of BMG layer increases tip to three layers. the fracture energy for the laminated specimen is increased due to crack redirection and absorption of the fracture energy at the crystalline layer during fracture. (c) 2005 Elsevier B.V. All rights reserved.
RI Park, Eun Soo/A-4443-2008; bang, changwook/J-7922-2012; Park, Eun
   Soo/A-9860-2014
SN 0921-5093
PD FEB 15
PY 2006
VL 417
IS 1-2
BP 239
EP 242
DI 10.1016/j.msea.2005.10.048
UT WOS:000235473700030
ER

PT J
AU Louzguine-Luzgin, DV
   Georgarakis, K
   Zadorozhnyy, V
   Chen, N
   Nakayama, K
   Vaughan, G
   Yavari, AR
   Inoue, A
AF Louzguine-Luzgin, Dmitri V.
   Georgarakis, Konstantinos
   Zadorozhnyy, Vladislav
   Chen, Na
   Nakayama, Koji
   Vaughan, Gavin
   Yavari, Alain R.
   Inoue, Akihisa
TI Atomic structure changes and phase transformation behavior in Pd-Si bulk
   glass-forming alloy
SO INTERMETALLICS
AB Synchrotron-radiation studies allowed continuous acquisition of X-ray diffraction spectra for binary Pd81Si19 bulk metallic glass forming alloy in the glassy, crystalline and liquid state. Such an investigation allowed determination of structural changes in the liquid and glassy states using real-space radial RDF(R) and atomic pair PDF(R) distribution functions. Crystallization behavior of this alloy was also studied by conventional X-ray diffractometry, transmission electron microscopy and differential scanning calorimetry in isochronal and isothermal mode. (C) 2011 Elsevier Ltd. All rights reserved.
RI Nakayama, Koji/E-5053-2012; Chen, Na/A-4120-2010; yavari,
   alain/E-8192-2010; Inoue, Akihisa/E-5271-2015; Georgarakis,
   Konstantinos/K-1939-2015; Zadorozhnyy, Vladislav/G-9616-2011; LOUZGUINE,
   Dmitri/D-2492-2010; Georgarakis, Konstantinos/E-6390-2010
OI LOUZGUINE, Dmitri/0000-0001-5716-4987; Georgarakis,
   Konstantinos/0000-0003-0918-7310
SN 0966-9795
EI 1879-0216
PD JAN
PY 2012
VL 20
IS 1
BP 135
EP 140
DI 10.1016/j.intermet.2011.08.022
UT WOS:000297889300020
ER

PT J
AU Li, R
   Pang, SJ
   Men, H
   Zhang, T
AF Li, R
   Pang, SJ
   Men, H
   Zhang, T
TI Ce-rich misch metal-based bulk metallic glasses with high glass-forming
   ability
SO MATERIALS TRANSACTIONS
AB Ce-rich misch metal (Mm)-based bulk metallic glasses (Mm consists of Ce, La, Pr and Nd) with high glass-forming ability are developed in Mm-Co-Al alloy system. The critical diameter for glass-formation is 10, 5 and 3 mm and the supercooled liquid region Delta(T)(x) (Delta T-x = T-x-T-g, T-x is crystallization temperature and T. is glass transition temperature) are 27, 90 and 79 K for Mm(65)Co(25)Al(10) (Mm(65)), Mm(60)Co(20)Al(20) (Mm60) and Mm(55)Co(25)Al(20) (Mm55) alloys, respectively. Reduced glass transition temperature T-rg (T-rg = T-g/T-m, where T-m is melting temperature) is 0.62, 0.65 and 0.69 for Mm65 Mm60 and Mm55 respectively. The employment of the misch metal, which comprises multi-rare earth elements, instead of pure Cc in the Ce-Al-Co system can improve the glass-forming ability in spite of the tiny atomic size difference of the rare earth metals. The present alloy system is an exception to the criteria of Delta T-x, T-g/T-m and different atomic size distribution as well as negative heat of mixing used for evaluating the relative GFA.
RI Li, Ran/B-4618-2010; Zhang, Tao/O-4911-2014; Pang, Shujie/D-8305-2016
SN 1345-9678
EI 1347-5320
PD OCT
PY 2005
VL 46
IS 10
BP 2291
EP 2294
DI 10.2320/matertrans.46.2291
UT WOS:000233047200029
ER

PT J
AU Chen, W
   Ketkaew, J
   Liu, Z
   Mota, RMO
   O'Brien, K
   da Silva, CS
   Schroers, J
AF Chen, Wen
   Ketkaew, Jittisa
   Liu, Ze
   Mota, Rodrigo Miguel Ojeda
   O'Brien, Kevin
   da Silva, Caio Sene
   Schroers, Jan
TI Does the fracture toughness of bulk metallic glasses scatter?
SO SCRIPTA MATERIALIA
AB A method is introduced to determine notch toughness of bulk metallic glasses (BMGs). Through thermoplastic replication of Si molds, unprecedented control in fabricating BMG toughness samples can be achieved and influences such as cooling rate, thermal history, residual stress, sample geometry, and notch precision are drastically reduced. For the 20 Zr44Ti11Cu10Ni10Be25 BMG samples, we measured a notch toughness of 109 +/- 3 MPa root m. Such a much smaller scatter than the previously reported suggests reliable properties of BMGs when thermoplastically formed. (C) 2015 Published by Elsevier Ltd. on behalf of Acta Materialia Inc.
SN 1359-6462
PD OCT
PY 2015
VL 107
BP 1
EP 4
DI 10.1016/j.scriptamat.2015.05.003
UT WOS:000358806400001
ER

PT J
AU Wu, JA
   Grosdidier, T
   Allain-Bonasso, N
   Gaffet, E
   Dong, CA
AF Wu, Jiang
   Grosdidier, Thierry
   Allain-Bonasso, Nathalie
   Gaffet, Eric
   Dong, Chuang
TI On the mechanically induced crystallization of FCC phases by mechanical
   milling in ZrAlNiCu bulk metallic glasses
SO JOURNAL OF ALLOYS AND COMPOUNDS
CT 16th International Symposium on Metastable, Amorphous and Nanostructured
   Materials
CY JUL 05-09, 2009
CL Beijing, PEOPLES R CHINA
AB In the present study, amorphous-nanocrystalline phase transformation induced by mechanical milling of full monolithic bulk metallic glasses (based on Zr(65)Al(7.5)Ni(10)Cu(17.5) and Zr(58)Al(16)Ni(11)Cu(15) alloys) has been investigated using X-ray diffraction as well as transmission electron microscopy. Nanocrystals having an FCC structure and a grain size of several 10nm precipitate in the early stages of the milling process and remain stable for long milling duration. The structure changes induced by milling give a new insight on the preparation of amorphous-related alloys when using the method of mechanical milling. (C) 2010 Elsevier B.V. All rights reserved.
RI GAFFET, Eric/C-3695-2013
OI GAFFET, Eric/0000-0002-6451-3011
SN 0925-8388
PD AUG
PY 2010
VL 504
SU 1
BP S264
EP S266
DI 10.1016/j.jallcom.2010.03.110
UT WOS:000285252600067
ER

PT J
AU Shan, SF
   Zhan, ZJ
   Jia, YZ
   Fan, CZ
   Zhang, BQ
   Liu, RP
   Wang, WK
AF Shan, Shengfeng
   Zhan, Zaiji
   Jia, Yuanzhi
   Fan, Changzeng
   Zhang, Baoqing
   Liu, Riping
   Wang, Wenkui
TI IMPROVEMENT OF PLASTICITY OF TI-BASED BULK METALLIC GLASSES BY PHASE
   TRANSITION OF A COOLING MEDIUM
SO INTERNATIONAL JOURNAL OF MODERN PHYSICS B
CT 5th International Conference on Advanced Materials and Processing
CY SEP 02-05, 2008
CL Harbin Inst Technol, Harbin, PEOPLES R CHINA
SP Natl Key Lab Preis Hot Proc Metals, State Key Lab Adv Welding Product Technol, Natl Nat Sci Fdn China, Minerals, Metals & Mat Soc
HO Harbin Inst Technol
AB Ti-based bulk metallic glasses (BMGs), Ti(40)Zr(25)Cu(9)Ni(8)Be(18), were prepared by phase transition cooling (PTC) and copper mould casting(CMC) technology. Ti(40)Zr(25)Cu(9)Ni(8)Be(18) glassy rod of more than 13 mm in diameter was prepared by PTC technology which shows the high cooling rate obtained by this technology. No obviously differences in thermal property were found between the BMGs prepared by the PTC and CMC technology. The compressive plasticity of the alloy was apparently changed, from 8% using CMC to 37% by using PTC technology. PTC technology is probably a promising method for preparing BMG and an effective way to increase the plasticity of BMGs.
RI Fan, Changzeng/I-6837-2018
OI Fan, Changzeng/0000-0001-6727-5912
SN 0217-9792
PD MAR 20
PY 2009
VL 23
IS 6-7
BP 1319
EP 1323
DI 10.1142/S0217979209060889
UT WOS:000266134700090
ER

PT J
AU Lee, PY
   Chung, ST
   Lin, CK
   Tsai, WT
   Lin, HM
AF Lee, Pee-Yew
   Chung, Sung-Ting
   Lin, Chung-Kwei
   Tsai, Wen-Ta
   Lin, Hong-Ming
TI Preparation and characterization of dual-phase bulk metallic glasses
   through powder metallurgy route
SO MATERIALS TRANSACTIONS
CT 5th International Conference on Bulk Metallic Glasses
CY OCT 01-05, 2006
CL Osaka Univ, Awaji Isl, JAPAN
SP Minist Educ, Culture, Sports, Sci & Technol, Inst Mat Res, Tohoku Univ, Japan Soc Promot Sci, Natl Inst Mat Sci, Hyogo Int Assoc
HO Osaka Univ
AB In the present study. amorphous M49Y15Cu36 and Ti50Cu28Ni15Sn7 alloy powders were synthesized separately by using a mechanical alloying technique. The dual-phase (Mg49Y15Cu36)(100-x)(Ti50Cu28Ni15Sn7)(x) (x = 0, 10, 20, 30, 40, and 50 vol%) powders were prepared by mixing the corresponding amorphous powders. The amorphous dual-phase powders were then consolidated into bulk metallic glass (BMG) discs. The amorphization status of as-prepared powders and BMG discs was confirmed by X-ray diffraction and by using a differential scanning calorimeter. The microstructure of the BMG discs showed that the Ti50Cu28Ni15Sn7 phase is distributed homogeneously within the Mg49Y15Cu36 matrix. The (Mg49Y15Cu36)(50)(Ti50Cu28Ni15Sn7)(50) BMG disc exhibited a relative density of 97.2% and its Vickers microhardness was 526 +/- 20 kg/mm(2).
SN 1345-9678
EI 1347-5320
PD JUL
PY 2007
VL 48
IS 7
BP 1595
EP 1599
DI 10.2320/matertrans.MJ200791
UT WOS:000248743100005
ER

PT J
AU Takeuchi, A
   Murty, BS
   Hasegawa, M
   Ranganathan, S
   Inoue, A
AF Takeuchi, Akira
   Murty, Budaraju Srinivasa
   Hasegawa, Masashi
   Ranganathan, Srinivasa
   Inoue, Akihisa
TI Analysis of bulk metallic glass formation using a tetrahedron
   composition diagram that consists of constituent classes based on blocks
   of elements in the periodic table
SO MATERIALS TRANSACTIONS
AB The formation of bulk metallic glasses (BMGs) has been analyzed with a tetrahedron composition diagram, which is comprised of constituent classes from blocks of elements in the periodic table. When Al and Ga are involved in the BMG composition environment. they are assumed to correspond to either s- or p-block elements. The analysis under the assumption reveals the presence of a composition band that connects the composition regions over different classes of BMGs. The diagram has a topological simplicity, is applicable to any multicomponent alloy system, and can be analyzed from the bonding nature of the atomic pairs. Thus, this diagram is an important tool for analyzing and developing BMGs.
RI Ranganathan, Srinivasa/F-9187-2013; Takeuchi, Akira/A-3619-2010; Inoue,
   Akihisa/E-5271-2015
OI Takeuchi, Akira/0000-0002-7246-8644; Murty, BS/0000-0002-4399-8531
SN 1345-9678
EI 1347-5320
PD JUN
PY 2007
VL 48
IS 6
BP 1304
EP 1312
DI 10.2320/matertrans.MF200604
UT WOS:000247823300027
ER

PT J
AU Wilde, G
AF Wilde, G
TI Thermodynamics, viscous flow and relaxation dynamics of bulk
   glass-forming Pd alloys
SO ANNALES DE CHIMIE-SCIENCE DES MATERIAUX
AB Experimental results concerning the thermodynamic excess properties and the equilibrium shear viscosity of the undercooled liquid state am presented which were consistently obtained on Pd-rich bulk metallic glasses of different stability by applying the concept of the limiting fictive temperature These results are evaluated with respect to the stability criteria that are controlling vitrification. In addition, modulated-temperature calorimetry has been performed to obtain the average relaxation time of the cooperative liquid modes near the glass transition directly in dependence of temperature. The results are compared to data obtained from static non-equilibrium measurements and discussed with respect to the relation between fragility and thermal stability of Pd-rich bulk glass-forming alloys.
RI Wilde, Gerhard/C-7808-2013
OI Wilde, Gerhard/0000-0001-8001-5998
SN 0151-9107
PD SEP-OCT
PY 2002
VL 27
IS 5
BP 49
EP 54
DI 10.1016/S0151-9107(02)80044-7
UT WOS:000179384800007
ER

PT J
AU Qin, ZX
   Li, CH
   Zhang, HF
   Wang, ZG
   Hu, ZQ
   Liu, ZQ
AF Qin, Zuoxiang
   Li, Cuihong
   Zhang, Haifeng
   Wang, Zhongguang
   Hu, Zhuangqi
   Liu, Zhiqiang
TI Friction Stir Welding of Zr55Al10Ni5Cu30 Bulk Metallic Glass to
   Crystalline Aluminum
SO JOURNAL OF MATERIALS SCIENCE & TECHNOLOGY
AB The Zr55Al10Ni5Cu30 bulk metallic glass plate were successfully welded to crystalline aluminum plates by using a friction stir welding (FSW) method. The welded zone was examined. No defects, cracks or pores were observed and no other crystalline phases except for aluminum were found in the welded joint. The strength of the joint is higher than that of aluminum. The glassy phase in the stir zone keeps the amorphous state, showing a successful welding. The storage modulus softens over the glass transition. And the weldability was discussed according to this phenomena.
SN 1005-0302
PD NOV
PY 2009
VL 25
IS 6
BP 853
EP 856
UT WOS:000272784100026
ER

PT J
AU Haruyama, O
   Annoshita, N
   Kimura, H
   Nishiyama, N
   Inoue, A
AF Haruyama, O
   Annoshita, N
   Kimura, H
   Nishiyama, N
   Inoue, A
TI Anomalous behavior of electrical resistivity in glass transition region
   of a bulk Pd40Ni40P20 metallic glass
SO JOURNAL OF NON-CRYSTALLINE SOLIDS
CT 55th Yamada Conference/11th International Conference on Liquid and
   Amorphous Metals (LAM 11)
CY SEP 09-14, 2001
CL YOKOHAMA, JAPAN
SP Yamada Sci Fdn, Keio Univ, Fac Sci & Technol, Yokohama Convent & Visitors Bur
AB The electrical resistivity of a bulk Pd40Ni40P20 metallic glass was measured as a function of temperature. This glass showed a positive slope, drho/dT = (1.28 +/- 0.03) x 10(-4) muOmega m/K, with a residual resistivity rho(300) of 1.29 +/- 0.03 muOmega m at room temperature. Also, the change in the resistivity due to the glass transition was carefully investigated, but, no variation in the neighborhood of glass transition temperature was observed. Bulk glasses Pd40Cu3Ni37P20 and Pd43Ni37P20 with slightly different composition were also prepared for a comparison. In case of these glasses, the slope, drho/dT, varied at the glass transition, i.e. the resistivity decreased after the glass transition for the former and increased for the latter. The slope of the resistivity curve measured for the Pd40Ni40P20 melt was larger than that of the glass. Thus, the extrapolated resistivity to higher temperature did not connect smoothly with the resistivity of the liquid phase. (C) 2002 Elsevier Science B.V. All rights reserved.
RI Kimura, Hisamichi/D-5449-2012; Inoue, Akihisa/E-5271-2015; Nishiyama,
   Nobuyuki/C-8228-2015
SN 0022-3093
PY 2002
VL 312-14
BP 552
EP 556
AR PII S0022-3093(02)01769-6
DI 10.1016/S0022-3093(02)01769-6
UT WOS:000178809700108
ER

PT J
AU Wang, XY
   Yang, C
   Fan, CZ
   Liu, RP
   Sun, LL
   Zhang, J
   Zhan, ZJ
   Wang, WK
AF Wang, XY
   Yang, C
   Fan, CZ
   Liu, RP
   Sun, LL
   Zhang, J
   Zhan, ZJ
   Wang, WK
TI Effect of proton irradiation on structure relaxation of
   Zr41.5Ti14.9CU12.6Ni10.5Be20.4 bulk metallic glass
SO CHINESE SCIENCE BULLETIN
AB Effects of proton irradiation on structure relaxation of Zr41.5Ti14.9Cu12.6Ni10.5Be20.4 bulk metallic glass are investigated by means of X-ray diffraction, differential scanning calorimetric and electronic resistance measurements. The results show that, at 203 K, the structure of Zr41.5Ti14.9Cu12.6Ni10.5Be20.4 bulk metal glass is relaxed obviously by proton irradiation with the energy and the dose being of 160 keV and 1.65x10(16) ions/cm(2), respectively. However, Zr41.5Ti14.9Cu12.6Ni10.5Be20.4 bulk metallic glass becomes more steady amorphous state under two conditions, one is that the energy and the dose are 160 keV and 1.1x10(15) ions/cm(2), respectively. Another is that the energy and the dose are 120 keV and 6.5x10(15) ions/cm(2), respectively.
RI Fan, Changzeng/I-6837-2018; Yang, Chao/H-1905-2015
OI Fan, Changzeng/0000-0001-6727-5912;
SN 1001-6538
PD MAY
PY 2004
VL 49
IS 10
BP 999
EP 1001
DI 10.1360/03we0210
UT WOS:000223146800003
ER

PT J
AU Hu, Y
   Yan, HH
   Lin, T
   Li, JF
   Zhou, YH
AF Hu, Y.
   Yan, H. H.
   Lin, T.
   Li, J. F.
   Zhou, Y. H.
TI Effect of cooling rate on the bending plasticity of Zr55Al10Ni5Cu30 bulk
   metallic glass
SO JOURNAL OF ALLOYS AND COMPOUNDS
AB The effect of cooling rate on the bending plasticity of Zr55Al10Ni5Cu30 bulk metallic glass was investigated by varying the thickness of the as-cast sample. It turned out that as the thickness of the as-cast sample increases from 1 to 3 mm, short-range ordering in the microstructures is enhanced until noticeable nanocrystallization occurs in the thickest sample, but the bending plasticity measured under the same test specification first decreases and then increases. It is suggested that both free volume and structural heterogeneity dominate the plasticity of metallic glasses. (C) 2012 Elsevier B.V. All rights reserved.
SN 0925-8388
PD JUN 25
PY 2012
VL 527
BP 36
EP 39
DI 10.1016/j.jallcom.2012.02.126
UT WOS:000302776600007
ER

PT J
AU Feng, SD
   Li, G
   Yu, PF
   Zhang, SL
   Ma, MZ
   Qi, L
   Liu, RP
AF Feng, Shidong
   Li, Gong
   Yu, Pengfei
   Zhang, Shiliang
   Ma, Mingzhen
   Qi, Li
   Liu, Riping
TI Effects of Aspect Ratio on the Shear Band Arrangements of Zr-Based
   Metallic Glasses
SO METALLURGICAL AND MATERIALS TRANSACTIONS A-PHYSICAL METALLURGY AND
   MATERIALS SCIENCE
AB Effects of aspect ratios of Zr-based metallic glasses on the shear band arrangements are investigated through molecular dynamics simulations and experiments. It is found that as the aspect ratio decreases, the dense multiple shear bands form, effectively depressing the formation of the penetrating shear bands, which improves plasticity and strength of metallic glass. Simulation reproduces the images of the evolution of the shear bands in metallic glass, explaining the experimental observation. It is found that as the aspect ratio decreases, shear transformation zones disperse evenly in the entire model, restraining connecting into penetrating shear bands. (C) The Minerals, Metals & Materials Society and ASM International 2014
RI zhang, shiliang/F-9287-2011; Zhang, Shiliang/N-2348-2017
OI Zhang, Shiliang/0000-0001-7795-437X
SN 1073-5623
EI 1543-1940
PD MAR
PY 2015
VL 46A
IS 3
BP 1119
EP 1124
DI 10.1007/s11661-014-2666-6
UT WOS:000350300200013
ER

PT J
AU Liang, L
   Hui, X
   Chen, GL
AF Liang, L.
   Hui, X.
   Chen, G. L.
TI Thermal stability and magnetocaloric properties of GdDyAlCo bulk
   metallic glasses
SO MATERIALS SCIENCE AND ENGINEERING B-SOLID STATE MATERIALS FOR ADVANCED
   TECHNOLOGY
AB Gd56-xDyxAl24Co20 (x = 16, 20 and 22) bulk metallic glasses (BMGs) alloys with a diameter of 2, 3 and 3 mm, respectively, were prepared by using copper mold casting. These alloys exhibit higher values of the glass transition temperature, crystallization temperature, and activation energy of the glass transition and crystallization, compared with those of other known rare-earth-based BMGs. A maximum magnetic entropy changes of 15.78 J/(kg K) is obtained in Gd40Dy16Al24Co20, which is the maximal among all the bulk metallic glasses, and is much larger than those of the known crystalline magnetic refrigerant compound Gd5Si2Ge1.9Fe0.1 and pure Gd metal. All the three BMG alloys have a broader temperature range of the entropy change peak, resulting in larger refrigerate capacities (RC) than those of conventional crystalline materials. The excellent magnetocaloric properties combining with high thermal stability make them an attractive candidate for magnetic refrigerants in the temperature range of 20-100 K. (c) 2007 Elsevier B.V. All rights reserved.
RI Hui, Xidong/A-1741-2010
SN 0921-5107
PD JAN 25
PY 2008
VL 147
IS 1
BP 13
EP 18
DI 10.1016/j.mseb.2007.10.017
UT WOS:000253664600003
ER

PT J
AU Ferenc, J
   Kowalczyk, M
   Cieslak, G
   Kulik, T
AF Ferenc, Jaroslaw
   Kowalczyk, Maciej
   Cieslak, Grzegorz
   Kulik, Tadeusz
TI Magnetostrictive Iron-Based Bulk Metallic Glasses for Force Sensors
SO IEEE TRANSACTIONS ON MAGNETICS
CT 21st Conference on Soft Magnetic Materials (SMM)
CY SEP 01-04, 2013
CL Budapest, HUNGARY
SP Hungarian Acad Sci, Wignet Ctr Phys, Inst Solid State Phys, IEEE Magnet Soc
AB Iron-based bulk metallic glasses have been known for several attractive properties, such as high mechanical strength and magnetic softness. They also exhibit sensitivity of magnetic properties to stress, which is the result of magnetoelastic coupling. In this paper, these combined features were exploited to build a simple, but effective magnetoelastic force sensor. It was found that when rods made of glassy (Fe, Co, Ni)-Nb-Si-B alloys, used as cores in a transformer, are subjected to compressive stress, voltage on secondary winding changes with the application of force. When proper measurement conditions are met, the dependence of output voltage versus force is nearly linear. Furthermore, rods with 2 mm in diameter are able to withstand forces reaching 8 kN, which gives an opportunity to design small-sized, high-load magnetoelastic force sensors.
OI Kowalczyk, Maciej /0000-0002-5432-4073
SN 0018-9464
EI 1941-0069
PD APR
PY 2014
VL 50
IS 4
AR 2003203
DI 10.1109/TMAG.2013.2287240
PN 1
UT WOS:000343032900022
ER

PT J
AU Park, ES
   Kim, DH
AF Park, E. S.
   Kim, D. H.
TI Effect of manipulating atomic scale heterogeneity on plasticity in
   Mg-based bulk metallic glasses
SO INTERMETALLICS
CT 7th International Conference on Bulk-Metallic Glasses
CY NOV 01-05, 2009
CL Busan, SOUTH KOREA
SP Yonsei Univ, Ctr Noncrystalline Mat
AB This paper investigates the effect of manipulating atomic scale heterogeneity on plasticity in Mg-based bulk metallic glasses (BMGs). First, we show that tailoring combinations of bonding energy among constituent elements in Mg-based BMGs results in local structural and/or chemical heterogeneity, which can influence the formation and propagation of the shear bands during deformation. Secondly, we show that by controlling cooling rate during vitrification, it is possible to modulate heterogeneity and thereby influence the viscous flow in supercooled liquid region, which is closely related to mechanical properties in BMGs. These findings suggest that optimization of heterogeneity by controlling the cooling rate as well as tailoring combinations of constituent elements could enhance plasticity in various BMGs. (C) 2010 Elsevier Ltd. All rights reserved.
RI Kim, Do Hyang/J-6575-2012; Park, Eun Soo/A-4443-2008; bang,
   changwook/J-7922-2012; Park, Eun Soo/A-9860-2014
SN 0966-9795
PD OCT
PY 2010
VL 18
IS 10
SI SI
BP 1867
EP 1871
DI 10.1016/j.intermet.2010.01.025
UT WOS:000281420700019
ER

PT J
AU Makino, A
   Kubota, T
   Chang, C
   Makabe, M
   Inoue, A
AF Makino, Akihiro
   Kubota, Takeshi
   Chang, Chuntao
   Makabe, Masahiro
   Inoue, Akihisa
TI FeSiBP bulk metallic glasses with unusual combination of high
   magnetization and high glass-forming ability
SO MATERIALS TRANSACTIONS
AB Among many kinds of bulk metallic glasses (BMGs), Fe-based BMGs with good magnetic properties, high strength and low materials cost should have great potential for wide variety of applications. However, the glass-forming metal elements such as Al, Ga, Nb, Mo, Y and so forth in the Fe-based BMGs significantly decrease saturation magnetization (J(s)) which is a essential property as soft magnetic materials and also increase the material cost.
   The coexistence of high Fe content and high glass-forming ability (GFA) has been earnestly desired from academia to industry. We report a novel Fe76SigB(10)P(5) (at%) bulk metallic glass with unusual combination of high J(s) of 1.51 T due to high Fe content and high GFA leading to a glassy rod with a diameter of 2.5 mm despite not-containing any glass-forming metal elements. This alloy composed of familiar and low-priced elements, also exhibiting very excellent magnetic softness and rather high strength, has a great advantage for engineering and industry, and thus should make a contribution to conservation of earth resources and environment through energy saving.
RI MAKINO, AKIHIRO/B-2549-2009; Inoue, Akihisa/E-5271-2015
SN 1345-9678
EI 1347-5320
PD NOV
PY 2007
VL 48
IS 11
BP 3024
EP 3027
DI 10.2320/matertrans.MRP2007198
UT WOS:000251869800040
ER

PT J
AU Huang, YJ
   Xue, P
   Cheng, X
   Wang, YM
   Cao, FY
   Ning, ZL
   Sun, JF
AF Huang, Yong-jiang
   Xue, Peng
   Cheng, Xiang
   Wang, Ya-ming
   Cao, Fu-yang
   Ning, Zhi-liang
   Sun, Jian-fei
TI Plasticity improvement of a Zr-based bulk metallic glass by micro-arc
   oxidation
SO JOURNAL OF IRON AND STEEL RESEARCH INTERNATIONAL
CT 17th IUMRS International Conference in Asia
CY OCT 20-24, 2016
CL Qingdao, PEOPLES R CHINA
SP IUMRS
AB Mciro-arc oxidation (MAO) was used to coat porous films on the surface of a Zr-based hulk metallic glass sample. The compressive test results indicated that, compared with the as-cast sample, the MAO treated one exhibited higher deformation capacity, associated with multiple shear hands with higher density on the side surface and well-developed vein patterns with smaller size on the fractured surface. The pore in the MAOed film and the matrix/coating interface initiated the shear hands and impeded the rapid propagation of shear hands, thus favoring the enhanced plasticity of the MAO treated sample. The obtained results demonstrated that MAO can he considered as an effective method to finely tune the mechanical performance of monolithic hulk metallic glasses.
RI Wang, Yaming/D-3200-2013
OI Wang, Yaming/0000-0002-8208-8696
SN 1006-706X
EI 2210-3988
PD APR
PY 2017
VL 24
IS 4
BP 416
EP 420
DI 10.1016/S1006-706X(17)30062-6
UT WOS:000401088300011
ER

PT J
AU Xia, MX
   Meng, QG
   Zhang, SG
   Ma, CL
   Li, JG
AF Xia Ming-Xu
   Meng Qing-Ge
   Zhang Shu-Guang
   Ma Chao-Li
   Li Jian-Guo
TI Thermodynamic characteristics of metallic glass-forming liquids
SO ACTA PHYSICA SINICA
AB On the basis of analyzing the thermodynamic model of regular melt, the mixing enthalpy Delta H-mix and the mixing entropy Delta S-mix of typical metallic glass melts were calculated. The distribution of Delta H-mix vs. Delta S-mix for typical metallic glasses was generalized, and a strategy for pinpointing metallic glass formers has been proposed by combining the critical cooling rate R. based on the atomic intrinsic characteristics of the alloys including atom size, composition, and mixing enthalpy of binary systems among the components. On the condition of Delta S-mix greater than 0.6 J center dot K-1 mol(-1) and Delta H less than -15 kJ center dot mol(-1), the alloy tends to form a bulk metallic glass(BMG). It shows that R-c is intimately related with Delta S-mix, and can be expressed as R-c = 42.24 x 10(4) exp(-13.91 Delta S-mix) + 19.66. Two new glass formers, Zr40Al10Ni15Cu35, located far from the glass forming area of the existing Zr-Al-Ni-Cu BMGs with a content of 55at%-65at% zirconium, and Fe53Co5Nd12B30 of quaternary Fe-B-based BMG, were successfully prepared with this approach.
RI xia, mingxu/C-4070-2011
OI xia, mingxu/0000-0002-3113-4795
SN 1000-3290
PD DEC
PY 2006
VL 55
IS 12
BP 6543
EP 6549
UT WOS:000243900800060
ER

PT J
AU Wu, J
   Pan, Y
   Liu, H
   Pi, J
AF Wu, J.
   Pan, Y.
   Liu, H.
   Pi, J.
TI Activation energy, viscosity and fragility of Cu-Zr-Ti-In bulk metallic
   glasses
SO MATERIALS SCIENCE AND TECHNOLOGY
AB In the present paper, activation energy, viscosity and fragility of two In alloyed Cu-Zr-Ti-In bulk metallic glasses are evaluated by differential scanning calorimetry and thermomechanical analysis respectively. E-g of 60Cu-34Zr-5Ti-1In and 58Cu-34Zr-5Ti-3In is 175 +/- 9 and 182 +/- 5 kJ mol(-1) respectively. Ex of 60Cu-34Zr-5Ti-1In and 58Cu-34Zr-5Ti-3In is 369 +/- 24 and 317 +/- 10 kJ mol(-1) respectively, indicating that increasing the content of indium decreases the value of E-x of the as cast bulk metallic glasses. By means of comparison of activation energies referring to the crystallisation peak temperature, one can find that the second step crystallisation of both bulk metallic glasses is easier to occur. The possible phases of the annealed samples are determined. Thermomechanical analysis shows that viscosity for 58Cu-34Zr-5Ti-3In is larger than that for 60Cu-34Zr-5Ti-1In in the supercooled region, but Vogel-Fulcher-Tammann (VFT) temperature of 58Cu-34Zr-5Ti-3In is lower than that of 60Cu-34Zr-5Ti-1In. Both 60Cu-34Zr-5Ti-1In and 58Cu-34Zr-5Ti-3In bulk metallic glasses are stronger than o-terphenyl, but more fragile than SiO2. 58Cu-34Zr-5Ti-3In is stronger than 60Cu-34Zr-5Ti-1In. It indicates that increasing indium content can decrease the VFT temperature but make glass stronger.
SN 0267-0836
EI 1743-2847
PD JUN
PY 2014
VL 30
IS 6
BP 670
EP 675
DI 10.1179/1743284713Y.0000000394
UT WOS:000336737200007
ER

PT J
AU Xue, RJ
   Zhao, LZ
   Shi, CL
   Ma, T
   Xi, XK
   Gao, M
   Zhu, PW
   Wen, P
   Yu, XH
   Jin, CQ
   Pan, MX
   Wang, WH
   Bai, HY
AF Xue, R. J.
   Zhao, L. Z.
   Shi, C. L.
   Ma, T.
   Xi, X. K.
   Gao, M.
   Zhu, P. W.
   Wen, P.
   Yu, X. H.
   Jin, C. Q.
   Pan, M. X.
   Wang, W. H.
   Bai, H. Y.
TI Enhanced kinetic stability of a bulk metallic glass by high pressure
SO APPLIED PHYSICS LETTERS
AB The metastable nature of metallic glasses (MGs) limits their applications. We report the formation of a stable Pd40.16Ni9.64Cu30.12P20.08 MG with bulk size under high pressure at room temperature. The stable MG shows remarkably enhanced thermal and kinetic stability with substantially increased glass transition temperature T-g, crystallization temperature T-x, density and mechanical properties. The unique stability can be further reinforced by higher pressure and maintained even above T-g. This result can advance the glass design and the understanding of the fundamental issues in MGs. Published by AIP Publishing.
OI Xi, Xuekui/0000-0002-9783-9552
SN 0003-6951
EI 1077-3118
PD NOV 28
PY 2016
VL 109
IS 22
AR 221904
DI 10.1063/1.4968834
UT WOS:000390243100017
ER

PT J
AU Zhang, J
   Lim, KY
   Feng, YP
   Li, Y
AF Zhang, J.
   Lim, K. Y.
   Feng, Y. P.
   Li, Y.
TI Fe-Nd-B-based hard magnets from bulk amorphous precursor
SO SCRIPTA MATERIALIA
AB The glass forming ability of the (Nd,Fe,B)(96)Nb-4 system has been studied and the best glass former is Fe65.28B24Nd6.72Nb4, with a critical diameter of 4 mm. After subsequent crystallization, the Fe64.32B22.08Nd9.6Nb4 alloy with a uniformly distributed Nd2Fe14B phase shows typical characteristics of a hard magnet with a coercivity of 1100 kA m(-1) and an energy product of 33 kJ m(-3). The combination of hard magnetic properties and the large critical sample size may make Fe-Nd-B-Nb a commercially viable candidate for industrial applications. (C) 2007 Acta Materialia Inc. Published by Elsevier Ltd. All rights reserved.
RI Feng, Yuan Ping/A-4507-2012
OI Feng, Yuan Ping/0000-0003-2190-2284
SN 1359-6462
PD JUN
PY 2007
VL 56
IS 11
BP 943
EP 946
DI 10.1016/j.scriptamat.2007.02.016
UT WOS:000246270600007
ER

PT J
AU Liang, SX
   Yin, LX
   Ma, MZ
   Jing, R
   Yu, PF
   Zhang, YF
   Wang, BA
   Liu, RP
AF Liang, S. X.
   Yin, L. X.
   Ma, M. Z.
   Jing, R.
   Yu, P. F.
   Zhang, Y. F.
   Wang, B. A.
   Liu, R. P.
TI A multi-component Zr alloy with comparable strength and Higher
   plasticity than Zr-based bulk metallic glasses
SO MATERIALS SCIENCE AND ENGINEERING A-STRUCTURAL MATERIALS PROPERTIES
   MICROSTRUCTURE AND PROCESSING
AB Zirconium (Zr)-based bulk metallic glass possesses the highest potential as a structural material among metallic glasses. Although Zr-based bulk metallic glass exhibits extremely high strength, its potential application has been restricted by a number of issues, such as fragility, small size, difficult fabrication into different shapes and poisonous beryllium content, among others. In this paper, a Zr-based crystal alloy with comparable strength and higher plasticity than Zr-based bulk metallic glass is presented. The proposed Zr-based alloy has a tensile strength greater than 1600 MPa. That value is comparable to the 1500 MPa to 2000 MPa strength of Zr-based bulk metallic glasses (BMGs). The ductility in terms of elongation reached 6.2%; at the same time, the 1400 MPa tensile strength was retained. This phenomenon is not possible for Zr-based BMGs. XRD results show that the proposed ultrahigh-strength Zr-based crystal alloy has two-phase structures: an hcp-structured alpha phase and a bcc-structured beta phase. The forged specimen exhibits a typical basket-weave microstructure, which is characterised by the interlaced plate alpha phase separated from the beta phase matrix. Fine, short bar-shaped alpha phases precipitated along the original beta grain boundary together with ultrafine dot-shaped alpha phases that presented inside the original beta grain when the ageing temperature was between 500 degrees C and 525 degrees C. As the ageing temperature increased, the dot-shaped alpha phase grew into plate shapes, decreasing the material's strength and increasing its plasticity. The ultrafine dot-shaped and short bar-shaped alpha phases in the original beta phase matrix are the main strengthening mechanisms of the ultrahigh-strength Zr-based crystal alloy. Crown Copyright (C) 2012 Published by Elsevier B.V. All rights reserved.
SN 0921-5093
EI 1873-4936
PD JAN 20
PY 2013
VL 561
BP 13
EP 16
DI 10.1016/j.msea.2012.10.061
UT WOS:000325835700003
ER

PT J
AU Zhang, Y
   Wang, WH
   Greer, AL
AF Zhang, Y.
   Wang, W. H.
   Greer, A. L.
TI Making metallic glasses plastic by control of residual stress
SO NATURE MATERIALS
AB Metallic glasses, now that many compositions can be made in bulk(1-3), are of interest for structural applications exploiting their yield stress and yield strain, which are exceptionally high for metallic materials(4). Their applicability is limited by their near-zero tensile ductility resulting from work-softening and shear localization. Even though metallic glasses can show extensive local plasticity, macroscopically they can effectively be brittle, and much current research is directed at improving their general plasticity. In conventional engineering materials as diverse as silicate glasses and metallic alloys, we can improve mechanical properties by the controlled introduction of compressive surface stresses(5-7). Here we demonstrate that we can controllably induce such residual stresses in a bulk metallic glass, and that they improve the mechanical performance, in particular the plasticity, but that the mechanisms underlying the improvements are distinct from those operating in conventional materials.
RI Greer, Alan Lindsay/G-1977-2011; Greer, Lindsay/E-9433-2017
SN 1476-1122
PD NOV
PY 2006
VL 5
IS 11
BP 857
EP 860
DI 10.1038/nmat1758
UT WOS:000241732000017
PM 17041581
ER

PT J
AU Shamlaye, KF
   Laws, KJ
   Ferry, M
AF Shamlaye, Karl F.
   Laws, Kevin J.
   Ferry, Michael
TI Supercooled liquid fusion of carbon fibre-bulk metallic glass composites
   with superplastic forming properties
SO SCRIPTA MATERIALIA
AB Bulk metallic glasses (BMGs) are multicomponent metal alloys that form a glassy structure with relative ease upon cooling from the melt. Unique to glassy materials is a glass-transition (temperature), whereby the vitreous solid relaxes into a supercooled liquid state, leading to a dramatic decrease in viscosity. This softening behaviour allows novel thermoplastic forming and bonding processes to be carried out that are simply unachievable among conventional metal processing methods. The work presented herein utilises this supercooled liquid state by infiltrating carbon fibres within a Mg-based BMG to manufacture fully-dense carbon fibre reinforced BMGs which also exhibit unique secondary forming capabilities. (c) 2015 Acta Materialia Inc. Published by Elsevier Ltd. All rights reserved.
SN 1359-6462
PD JAN 15
PY 2016
VL 111
BP 127
EP 130
DI 10.1016/j.scriptamat.2015.08.001
UT WOS:000364253400028
ER

PT J
AU Greer, AL
   Castellero, A
   Madge, SV
   Walker, IT
   Wilde, JR
AF Greer, AL
   Castellero, A
   Madge, SV
   Walker, IT
   Wilde, JR
TI Nanoindentation studies of shear banding in fully amorphous and
   partially devitrified metallic alloys
SO MATERIALS SCIENCE AND ENGINEERING A-STRUCTURAL MATERIALS PROPERTIES
   MICROSTRUCTURE AND PROCESSING
CT 11th International Conference on Rapidly Quenched and Metastable
   Materials
CY AUG 25-30, 2002
CL Univ Oxford, Dept Mat, Oxford, ENGLAND
HO Univ Oxford, Dept Mat
AB Nanoindentation loading curves on metallic glasses can show sharp, constant-load steps. The relationship of these steps to shear-band initiation is explored for a wide range of metallic glasses, from thin films to bulk. On a given loading curve, there is a well defined strain for initiation of shear bands. The steps may be blurred by instrumental effects or partial devitrification. The nanoindentation results do not suggest a transition in deformation mode as a function of strain rate. (C) 2003 Elsevier B.V. All rights reserved.
RI Greer, Alan Lindsay/G-1977-2011; Greer, Lindsay/E-9433-2017
OI Castellero, Alberto/0000-0001-8290-7543; Madge,
   Shantanu/0000-0001-7996-8652
SN 0921-5093
PD JUL 15
PY 2004
VL 375
SI SI
BP 1182
EP 1185
DI 10.1016/j.msea.2003.10.032
UT WOS:000223329700235
ER

PT S
AU Miskuf, J
   Csach, K
   Jurikova, A
AF Miskuf, Jozef
   Csach, Kornel
   Jurikova, Alena
BE Longauerova, M
   Zubko, P
TI Fragmentation of Co-Fe-Ta-B Bulk Metallic Glass
SO METALLOGRAPHY XV
SE Materials Science Forum
CT 15th International Symposium on Metallography (Metallography 2013)
CY APR 24-26, 2013
CL Stara Lesna, SLOVAKIA
SP Tech Univ Kosice, Fac Met, Dept Mat Sci, Slovak Acad Sci Kosice, Inst Mat Res, Slovak Acad Sci, Met Sci Soc, Soc Profess Metallurgists, Slovak Acad Sci Kosice, Inst Expt Phys, US Steel Kosice, sro, SMZ, Slovak Magnet Works, TSI Syst sro, STRUERS GmbH, Org Div, MITAR sro, Carl ZEISS SLOVAKIA sro, NIKON spol sro, ZENA R Slovakia sro, JEOL (Europe) SAS, Org Div, Edlin sro, METROTEST sro
AB The main limitation of bulk metallic glasses for their application as structural materials is the large brittleness under the external loading. We analyzed the failure characteristics of Co43Fe20Ta5.5B31.5 (at.%) bulk metallic glass deformed in a compression at the room temperature and a low strain rate. Under loading the amorphous structure can store high elastic energy. During the failure this energy is released and the alloy breaks into small particles or powder exhibiting a fragmentation mode. The nanoscale fracture surface morphology respects the micromechanisms of failure of the amorphous structure. The fracture surface consists of a smooth mirror cleavage zone and a river pattern zone with the nanosized dimples arranged in lines respecting the periodic corrugation zones oriented perpendicular to the crack propagation direction.
SN 0255-5476
PY 2014
VL 782
BP 553
EP 556
DI 10.4028/www.scientific.net/MSF.782.553
UT WOS:000338978600104
ER

PT J
AU Narayan, RL
   Tandaiya, P
   Garrett, GR
   Demetriou, MD
   Ramamurty, U
AF Narayan, R. L.
   Tandaiya, Parag
   Garrett, G. R.
   Demetriou, M. D.
   Ramamurty, U.
TI On the variability in fracture toughness of 'ductile' bulk metallic
   glasses
SO SCRIPTA MATERIALIA
AB The mode I fracture toughness, K-Ic, of ductile bulk metallic glasses (BMGs) exhibits a high degree of specimen-to-specimen variability. By conducting fracture experiments in modes I and II, we demonstrate that the observed high variability in mode I, vis-a-vis mode II, is a result of highly variable propensity for the conversion of shear bands into cracks in mode I whereas in mode II, crack growth direction is fixed. Thus, the measured variability in K-Ic is intrinsic to the nature of BMGs. (C) 2015 Acta Materialia Inc. Published by Elsevier Ltd. All rights reserved.
OI Narayan, R. L./0000-0002-6178-8124
SN 1359-6462
PD JUN
PY 2015
VL 102
BP 75
EP 78
DI 10.1016/j.scriptamat.2015.02.017
UT WOS:000353090600019
ER

PT J
AU Kiprianov, AA
   Golubko, NV
   Karpukhina, NG
   Molodozhen, VA
AF Kiprianov, AA
   Golubko, NV
   Karpukhina, NG
   Molodozhen, VA
TI Thermal, electrical, and potentiometric characteristics of sodium
   aluminosilicate glasses with fluoride additives
SO GLASS PHYSICS AND CHEMISTRY
AB The bulk characteristics and electrode properties are experimentally studied for a number of sodium aluminosilicate glasses with fluoride additives. It is shown that the characteristic temperatures regularly decrease and the resistivity exhibits a nonlinear behavior with an increase in the fluorine content in glasses. The fluoride additives are found to produce a positive effect on the electrode properties of glasses with a low content of the second glass-former. These glasses possess a pronounced selectivity for potassium ions. It is assumed that the strongly acidic groups [AlO3/2F]Na--(+) favoring an increase in the electric conductivity and manifestation of metallic function are formed in the glass structure.
RI KIPRIANOV, ANDREY/I-8095-2013
OI KIPRIANOV, ANDREY/0000-0002-1872-4846
SN 1087-6596
PD JUL-AUG
PY 1998
VL 24
IS 4
BP 350
EP 354
UT WOS:000076163600012
ER

PT J
AU Liu, BY
   Ye, F
AF Liu, Bo-Yang
   Ye, Feng
TI Glass transition kinetics of La55Al25Ni10Cu10 bulk metallic glass by
   electrical resistivity measurement
SO RARE METALS
AB The glass transition behavior of La55Al25Ni10Cu10 bulk metallic glass was investigated using electrical resistivity and differential-scanning calorimetry measurements. There are good agreements of the onset temperature (T (g-onset)) and the end temperature (T (g-end)) of the glass transition between the DSC curve and temperature dependent electrical resistivity (TER) curve in the same heating rate. Kinetics of the glass transition is studied with the electrical resistivity data. The apparent activation energy of the glass transition (Delta E (g)) is found to be 306.4 kJ center dot mol(-1). Fragility parameter D (*) and VFT temperature T (0) are 9.8 and 324.9 K, respectively.
RI Ye, Feng/G-8236-2014
OI Ye, Feng/0000-0002-8808-9075
SN 1001-0521
PD AUG
PY 2013
VL 32
IS 4
BP 359
EP 362
DI 10.1007/s12598-013-0089-y
UT WOS:000323735900006
ER

PT J
AU Guan, H
   Kou, HC
   Wang, J
   Tang, B
   Zhong, H
   Li, JS
AF Guan Heng
   Kou Hongchao
   Wang Jun
   Tang Bin
   Zhong Hong
   Li Jinshan
TI Study on Structural Transformation Behavior of a Ti-Based Bulk Metallic
   Glass by Thermal Expansion Method
SO RARE METAL MATERIALS AND ENGINEERING
AB Ti40Zr25Ni8Cu9Be18 bulk metallic glass rod with diameter of 3 mm was prepared by the arc melting and injection casting method. The structural transformation behavior of the Ti-based bulk metallic glass has been studied by continuous heating thermal expansion method with a pusher thermal expansion instrument. It is found that there are five distinct stages during continuous heating according to the thermal expansion curve. The five distinct stages are the relaxation preparation stage, the structural relaxation stage, the first crystallization stage, the second crystallization stage and the stage of grain growth. Nanocrystals with size of 20 nm are found after thermal expansion. The study has also found that the characteristic temperatures of the Ti-based metallic glass in the thermal expansion curve have a good consistency with that in the DSC curve.
RI WANG, Jun/A-1526-2015; WANG, Jun/B-1229-2010; Tang, Bin/M-1302-2018
OI WANG, Jun/0000-0001-8101-2967; WANG, Jun/0000-0001-8101-2967; Tang,
   Bin/0000-0002-3355-5999; Li, Jinshan/0000-0002-6894-9760
SN 1002-185X
PD MAY
PY 2014
VL 43
IS 5
BP 1047
EP 1050
UT WOS:000336885400005
ER

PT J
AU Ma, MZ
   Zong, HT
   Wang, HY
   Zhang, WG
   Song, AJ
   Liang, SX
   Wang, Q
   Zhang, XY
   Jing, Q
   Li, G
   Liu, RP
AF Ma, M. Z.
   Zong, H. T.
   Wang, H. Y.
   Zhang, W. G.
   Song, A. J.
   Liang, S. X.
   Wang, Q.
   Zhang, X. Y.
   Jing, Q.
   Li, G.
   Liu, R. P.
TI Indentation and friction of Zr-based bulk metallic glasses on nano-scale
SO MATERIALS LETTERS
AB Indentation and sliding friction of the as-cast, the relaxed and the crystallized Zr(41)Ti(14)Cu(12.5)Ni(10)Be(22.5) bulk metallic glass (BMG) are investigated on nano - scale by nanoindentation. The depth, the width and the morphology of the scratches formed by the tip of the nanoindentor are different, which reflect the differences in structure, not only between the glassy and the crystallized states but also between the two glassy states, the as - cast and the relaxed. The coefficient of sliding friction of Zr - based BMG is increased with increasing the load, and it is maximal for annealed at 553K. Improved plastic deformability is found for the relaxed one. Crown Copyright (C) 2008 Published by Elsevier B.V. All rights reserved.
SN 0167-577X
PD NOV 15
PY 2008
VL 62
IS 28
BP 4348
EP 4350
DI 10.1016/j.matlet.2008.07.019
UT WOS:000259420300012
ER

PT J
AU Schuster, BE
   Wei, Q
   Ervin, MH
   Hruszkewycz, SO
   Miller, MK
   Hufnagel, TC
   Ramesh, KT
AF Schuster, B. E.
   Wei, Q.
   Ervin, M. H.
   Hruszkewycz, S. O.
   Miller, M. K.
   Hufnagel, T. C.
   Ramesh, K. T.
TI Bulk and microscale compressive properties of a Pd-based metallic glass
SO SCRIPTA MATERIALIA
AB We present quasi-static, room temperature compression data for Pd40Ni40P20 metallic glasses for both bulk and microscale specimens. At all sizes, plastic flow is localized in shear bands which are accompanied by sudden strain bursts in the microscale tests. We show a modest (similar to 9%) increase in the 0.2% offset yield strength in going from bulk specimens to the smallest measured (similar to 2 mu m), and attribute this increase to the effect of defects on shear band initiation, rather than to an intrinsic length scale dependence of plastic deformation. (c) 2007 Acta Materialia Inc. Published by Elsevier Ltd. All rights reserved.
RI Hufnagel, Todd/A-3309-2010; Wei, Qiuming/B-7579-2008
OI Hufnagel, Todd/0000-0002-6373-9377;
SN 1359-6462
PD SEP
PY 2007
VL 57
IS 6
BP 517
EP 520
DI 10.1016/j.scriptamat.2007.05.025
UT WOS:000248638600018
ER

PT J
AU Tam, CY
   Shek, CH
AF Tam, C. Y.
   Shek, C. H.
TI Improved oxidation resistance of Cu(60)Zr(30)Ti(10)BMG with plasma
   immersion ion implantation
SO JOURNAL OF NON-CRYSTALLINE SOLIDS
CT 12th International Conference on Liquid and Amorphous Metals (LAM12)
CY JUL 11-16, 2004
CL Metz, FRANCE
AB The influences of nitrogen ion implantation on the Cu60Zr30Ti10 bulk metallic glass were investigated. It was found that the surface composition and structure of the nitrogen-ion implanted metallic glasses were different from those of the as-cast one and the oxidation resistance was improved by the surface modification process. Grazing incidence X-ray diffraction (GIXRD) showed that the implanted samples remained amorphous but with some ZrO2 and copper oxides on the surface. X-ray photoelectron spectroscopy (XPS) results further revealed that a thin ZrN layer is formed while no Cu atom can be observed on the top surface of the implanted samples. Thermogravimetric analysis (TGA) showed an improved oxidation resistance of the implanted samples compared with the as-cast metallic glass. Both as-cast and implanted metallic glasses formed ZrO2 and copper oxides after oxidation. This was consistent with our observations in scanning electron microscopy (SEM). (c) 2007 Elsevier B.V. All rights reserved.
RI SHEK, Chan Hung/J-3857-2015
OI SHEK, Chan Hung/0000-0002-6870-523X
SN 0022-3093
PD OCT 15
PY 2007
VL 353
IS 32-40
BP 3590
EP 3595
DI 10.1016/j.jnoncrysol.2007.05.118
UT WOS:000250235200124
ER

PT J
AU Chen, QJ
   Wei, DD
   Zhou, XL
   Yan, JW
   Hua, XZ
   Ai, YL
AF Chen Qingjun
   Wei Dandan
   Zhou Xianliang
   Yan Jianwu
   Hua Xiaozhen
   Ai Yunlong
TI Non-Isothermal Crystallization Kinetics of Fe41Co7Cr15Mo14Y2C15B6 Bulk
   Amorphous Alloy
SO RARE METAL MATERIALS AND ENGINEERING
AB The non-isothermal crystallization kinetics of Fe41Co7Cr15Mo14Y2C15B6 bulk amorphous alloy with high glass forming ability was studied by the differential thermal analyzer (DTA). Glass transition temperature T-g, onset crystallization temperature T-x and peak crystallization temperature T-p1 and T-p2 were obtained from DTA curves. These characteristic temperatures were of obvious dynamic effect. The glass transition activation energy E-g, crystallization activation energy E-x, and activation energy E-p1, E-p2 corresponding to characteristic temperatures T-g, T-x, T-p1, T-p2 were calculated by Kissinger and Ozawa equations, respectively. The thermodynamics mechanism of Fe41Co7Cr15Mo14Y2C15B6 bulk amorphous alloy with high thermal stability was explained by Kissinger method and Ozawa method.
SN 1002-185X
PD AUG
PY 2009
VL 38
IS 8
BP 1343
EP 1347
UT WOS:000269849400007
ER

PT S
AU Yue, LJ
   Han, JS
   Xie, K
AF Yue Li Jie
   Han Jin Sheng
   Xie Kun
BE Wang, RM
   Wu, Y
   Wu, XF
TI The Microalloying Effects in Cu-based Bulk Metallic Glasses
SO NANO-SCALE AND AMOURPHOUS MATERIALS
SE Materials Science Forum
CT 11th International Conference in Asia of the
   International-Union-of-Materials-Research-Societies
CY SEP 25-28, 2010
CL Qingdao, PEOPLES R CHINA
SP Int Union Mat Res Soc (IUMRS), Chinese Mat Res Soc, Mat Res Soc Taiwan, Mat Res Soc Japan, Govt Qingdao City
AB The glassy alloy rods of Cu50Zr43Al7, (Cu50Zr43Al7)(100-x)Y-x(x=2,5) and (Cu50Zr43Al7)(100-x)Y-x(x=6,7) with diameters of 3.0 mm were prepared by copper mold suction casting method. The influence of adding Ag and Y to Cu50Zr43Al7 metallic glass on glass formation ability (GFA) and thermal stability was studied by means of X-ray diffraction analysis (XRD) and differential scanning calorimetry (DSC). The results show that Ag and Y appropriate micro-addition enhance the glass formation ability and thermal stability of the Cu-Zr-Al metallic glass. The effect of Ag is better than that of Y. The addition of Ag causes a increase of the reduced glass transition temperature (T-rg) of (Cu50Zr43Al7)(100-x)Y-x alloy from 0.618 at 0 at.% Ag to 0.628 at 7 at.% Ag. The width of the supercooled liquid region of Cu43Zr43Al7Ag7 glassy alloy increases about 25K compared with that of Cu50Zr43Al7, and the gamma value of Cu43Zr43Al7Ag7 reaches 0.433. The electrochemical corrosion behaviors of Cu50Zr43Al7, (Cu50Zr43Al7)(100-x)Y-x(x=2,5) and (Cu50Zr43Al7)(100-x)Y-x(x=6,7) metallic glasses in 3.5% NaCl solution were investigated by potentiodynamic polarization method. It is found that corrosion resistance of all amorphous alloys is better than that of the corresponding crystalline alloys. The Ag and Y micro-addition improve corrosion resistance of Cu50Zr43Al7 metallic glass. The corrosion current density of Cu43Zr43Al7Ag7 metallic glass decreases 1 similar to 2 orders of magnitude compared with that of Cu50Zr43Al7.
SN 0255-5476
PY 2011
VL 688
BP 407
EP +
DI 10.4028/www.scientific.net/MSF.688.407
UT WOS:000302644200072
ER

PT J
AU Zhang, T
   Zhang, XG
   Zhang, HY
   Jia, F
   Zhang, W
   Inoue, A
   Ma, YJ
AF Zhang Tao
   Zhang Xingguo
   Zhang Huanyue
   Jia Fei
   Zhang Wei
   Inoue, Akihisa
   Ma Yuejiao
TI Continuous Casting of Bulk Metallic Glass
SO RARE METAL MATERIALS AND ENGINEERING
AB A new continuous casting method for production of bulk metallic glass (BMG) was proposed. An intermittent withdrawal procedure, in which a withdrawal cycle was employed, was adopted for the continuous casting of BMG. A Zr-based bulk glassy alloy rod with a diameter of 10 mm and a length of 60 cm was prepared using this method. The glassy nature of the rod was identified by optical microscopy, X-ray diffraction and differential scanning calorimetry. The continuous cooling transformation curve of the glassy alloy was adopted for the discussion of cooling history of the continuous cast. The newly developed continuous casting method can be used for the massive production of bulk glassy alloys, and it can effectively reduce production costs and accelerate the development and application of metallic glasses.
RI Inoue, Akihisa/E-5271-2015
SN 1002-185X
PD DEC
PY 2012
VL 41
IS 12
BP 2205
EP 2209
UT WOS:000313859000029
ER

PT J
AU Zong, HT
   Bian, LY
   Cheng, JY
   Cao, GH
   Kang, CY
   Li, M
AF Zong, Haitao
   Bian, Linyan
   Cheng, Jingyun
   Cao, Guohua
   Kang, Chaoyang
   Li, Ming
TI Glass forming ability, thermal stability and elastic properties of
   Zr-Ti-Cu-Be-(Fe) bulk metallic glasses
SO Results in Physics
AB A series of Zr35-xTi30Cu7.5Be27.5Fex (x = 0-10) bulk metallic glasses are cast using water-cooled copper mold casting technique. The glass forming ability (GFA) and thermal stability of Zr35-xTi30Cu7.5Be27.5Fex alloys were studied by means of X-ray diffraction and differential scanning calorimetry. It was found that the Zr35-xTi30Cu7.5Be27.5Fex (x = 2, 3, 5 and 7, respectively) alloys could be cast into glassy cylindrical rods with a diameters up to 20 mm. However, higher Fe content (x = 10) deteriorates the GFA remarkably. The supercooled liquid region (Delta T-x), gamma parameter (defined as T-x/(T-g + T-l)) and the reduced glass transition temperature T-rg (defined as T-g/T-l) are employed to evaluate the GFA in Zr35-xTi30Cu7.5Be27.5Fex (x = 0-10) alloys. The results showed that Trg was more effective in gauging the GFA of the Zr35-xTi30Cu7.5Be27.5Fex alloy systems than Delta T-x and gamma parameter. In addition, elastic constant (Poisson's ratio) was also employed as a gauge to evaluate the glass forming ability in Zr35-xTi30Cu7.5Be27.5Fex alloys. (C) 2016 The Authors. Published by Elsevier B.V.
SN 2211-3797
PY 2016
VL 6
BP 1157
EP 1160
DI 10.1016/j.rinp.2016.09.015
UT WOS:000389770300203
ER

PT J
AU Zhuang, YX
   Chen, J
   Liu, WJ
   He, JC
AF Zhuang, Y. X.
   Chen, J.
   Liu, W. J.
   He, J. C.
TI Effect of high magnetic field on crystallization of
   Zr46.75Ti8.25Cu7.5Ni10Be27.5 bulk metallic glass
SO JOURNAL OF ALLOYS AND COMPOUNDS
CT 16th International Symposium on Metastable, Amorphous and Nanostructured
   Materials
CY JUL 05-09, 2009
CL Beijing, PEOPLES R CHINA
AB It has been demonstrated that high magnetic field is a very powerful tool in controlling microstructures of materials. In this work, the effect of high magnetic field on the crystallization of Zr46.75Ti8.25Cu7.5Ni10Be27.5 bulk metallic glass has been investigated. The isothermal crystallization of the bulk metallic glass was performed at the temperatures ranging from 643 to 713K with or without a high magnetic field. The direction of the magnetic field is parallel or perpendicular to the sample surfaces. The annealed samples were characterized by X-ray diffraction and differential scanning calorimeter. It has been found that the crystallization of the bulk metallic glasses depends not only on the annealing temperatures, but also on the direction and the intensity of the high magnetic field. The high magnetic field significantly retards the crystallization of the bulk metallic glass at certain annealing temperatures. The possible mechanisms of high magnetic field effect on the crystallization have been discussed. (C) 2010 Elsevier B.V. All rights reserved.
RI Zhuang, Yanxin/F-7199-2011
SN 0925-8388
PD AUG
PY 2010
VL 504
SU 1
BP S256
EP S259
DI 10.1016/j.jallcom.2010.02.166
UT WOS:000285252600065
ER

PT J
AU Choi-Yim, H
   Tokarz, M
   Bilello, JC
   Johnson, WL
AF Choi-Yim, H
   Tokarz, M
   Bilello, JC
   Johnson, WL
TI Structure and properties of Ni-60(Nb100-xTax)(34)Sn-6 bulk metallic
   glass alloys
SO JOURNAL OF NON-CRYSTALLINE SOLIDS
AB Refractory bulk metallic glasses and bulk metallic glass composites are formed in quaternary Ni-Nb-Ta-Sn alloy system. Alloys of composition Ni-60(Nb100-xTax)(34)Sn-6 (x = 20, 40, 60, 80) alloys were prepared by injection-casting the molten alloys into copper molds. Glassy alloys are formed in the thickness of half mm strips. With thicker strips (e.g., 1 mm), Nb2O5 and Ni3Sn phases and the amorphous phase form an in situ composite. Glass transition temperatures, crystallization temperatures, and Delta T-x, defined as T-x1 - T-g (T-x1: first crystallization temperature, T-g: glass transition temperature) of the alloys increase dramatically with increasing Ta contents. These refractory bulk amorphous alloys exhibit high Young's modulus (155-170 GPa), shear modulus (56-63 GPa), and estimated yield strength (3-3.6 GPa). (c) 2006 Elsevier B.V. All rights reserved.
SN 0022-3093
PD JUN 1
PY 2006
VL 352
IS 8
BP 747
EP 755
DI 10.1016/j.jnoncrysol.2006.02.003
UT WOS:000237751000003
ER

PT J
AU Zhang, Y
   Zhou, M
   Zhao, XY
   Ma, LQ
AF Zhang, Yu
   Zhou, Min
   Zhao, Xiangyu
   Ma, Liqun
TI Co substituted Zr-Cu-Al-Ni metallic glasses with enhanced glass-forming
   ability and high plasticity
SO JOURNAL OF NON-CRYSTALLINE SOLIDS
AB Zr-based bulk metallic glasses have been considered as promising engineering materials. However, their widespread application is limited by their contrasting properties of glass-forming ability (GFA) and plasticity. Herein, the effects of Ni substitution by Co on the GFA and plasticity of the representative Zr-based metallic glass of Zr65Cu17.5Al7.5Ni10 were investigated. A series of Zr65Cu17.5Al7.5Ni10 - Co-X(x) (x = 0, 2, 4, 6, 7, 8, 10) rod samples with 2 mm in diameter were prepared by injection copper mold casting method. Amorphous structure was successfully achieved when the Co content is less than 8 at.%. Importantly, the Co addition contributed to significant enhancements in,both GFA and plasticity of the amorphous Zr65Cu17.5Al7.5Ni10 - Co-x(x) (x = 0, 2, 4, 6, 7) alloys. The as-prepared Zr65Cu17.5Al7.5Ni8Co2 amorphous alloy shows the best and attractive performance with a supercooled liquid region width of 80 K and a compressive plasticity of 7.4%, which are much higher than 50 K and 0.2% of the pristine Zr65Cu17.5Al7.5Ni10 amorphous alloy, demonstrating that the Co substituted Zr-AlNi-Cu metallic glass has the potential to be used for engineering applications.
SN 0022-3093
EI 1873-4812
PD OCT 1
PY 2017
VL 473
BP 120
EP 124
DI 10.1016/j.jnoncrysol.2017.08.001
UT WOS:000412381400017
ER

PT J
AU Chen, YQ
   Huang, YJ
   Fan, HB
   Wang, DJ
   Shen, J
AF Chen, Yiqiang
   Huang, Yongjiang
   Fan, Hongbo
   Wang, Dongjun
   Shen, Jun
TI The local structure nature for a Ti-based bulk metallic glass
SO MATERIALS SCIENCE AND ENGINEERING B-ADVANCED FUNCTIONAL SOLID-STATE
   MATERIALS
AB In the present work, the local atomic structures of a Be-containing Ti-based bulk metallic glass (BMG) have been characterized using electron spectrum for chemical analysis and Raman scattering, including directional bonds and medium range order. It might suggest that a coefficient could be extracted from Raman scattering to characterize the glass forming ability (GFA), which could be employed to interpret the enhanced GFA by Be addition of Ti-based BMG. Additionally, compared with the crystallized sample, the glassy sample exhibits larger average bond length and larger content of local bond distortion using Raman scattering. (C) 2012 Elsevier B.V. All rights reserved.
RI Huang, Yongjiang/D-4809-2009
SN 0921-5107
PD JAN 1
PY 2013
VL 178
IS 1
BP 117
EP 121
DI 10.1016/j.mseb.2012.10.036
UT WOS:000313397200018
ER

PT J
AU Lu, ZP
   Liu, CT
AF Lu, ZP
   Liu, CT
TI Comments on "fabrication of ternary Mg-Cu-Gd bulk metallic glass with
   high glass-forming ability under air atmosphere" [H. Men and D H Kim, J.
   Mater. Res. 18, 1502 (2003)]
SO JOURNAL OF MATERIALS RESEARCH
AB A new Mg-based bulk amorphous alloy (i.e., Mg65Cu25Gd10) has successfully been developed by Men and Kim [H. Men and D.H. Kim, J. Mater. Res. 18, 1502 (2003)]. They showed that this alloy exhibits significantly improved glass-forming ability (GFA) in comparison with Mg65Cu25Y10 alloy. However, this improved GFA cannot be indicated by the supercooled liquid region AT and the reduced glass-transition temperature T-rg. As shown in the current comment, the new parameter gamma, T-x/(T-g + T-1) defined in our recent papers [Z.P. Lu and C.T. Liu, Acta Mater. 50, 3501 (2002); Z.P. Lu and C.T. Liu, Phys. Rev. Lett. 91, 115505 (2003)] can well gauge GFA for bulk metallic glasses, including the current Mg-based alloys.
RI Lu, Zhao-Ping/A-2718-2009
OI Lu, Zhao-Ping/0000-0003-1463-8948; Liu, Chain Tsuan/0000-0001-7888-9725
SN 0884-2914
PD FEB
PY 2004
VL 19
IS 2
BP 427
EP 428
DI 10.1557/jmr.2004.19.2.427
UT WOS:000222316200006
ER

PT J
AU Li, JY
   Gittleson, FS
   Liu, YH
   Liu, JB
   Loye, AM
   McMillon-Brown, L
   Kyriakides, TR
   Schroers, J
   Taylor, AD
AF Li, Jinyang
   Gittleson, Forrest S.
   Liu, Yanhui
   Liu, Jingbei
   Loye, Ayomiposi M.
   McMillon-Brown, Lyndsey
   Kyriakides, Themis R.
   Schroers, Jan
   Taylor, Andre D.
TI Exploring a wider range of Mg-Ca-Zn metallic glass as biocompatible
   alloys using combinatorial sputtering
SO CHEMICAL COMMUNICATIONS
AB In order to bypass the limitation of bulk metallic glasses fabrication, we synthesized thin film metallic glasses to study the corrosion characteristics of a wide atomic% composition range, Mg(35.9-63%)Ca(4.1-21%)Zn(17.9-58.3%), in simulated body fluid. We highlight a clear relationship between Zn content and corrosion current such that Zn-medium metallic glasses exhibit minimum corrosion. In addition, we found higher Zn content leads to a poor in vitro cell viability. These results showcase the benefit of evaluating a larger alloy compositional space to probe the limits of corrosion resistance and prescreen for biocompatible applications.
OI gittleson, forrest/0000-0003-0360-8348
SN 1359-7345
EI 1364-548X
PD JUL 28
PY 2017
VL 53
IS 59
BP 8288
EP 8291
DI 10.1039/c7cc02733h
UT WOS:000405907900009
PM 28665424
ER

PT J
AU Abrosimova, GE
   Aronin, AS
   Gurov, AF
   Kir'yanov, YV
   Molokanov, VV
AF Abrosimova, GE
   Aronin, AS
   Gurov, AF
   Kir'yanov, YV
   Molokanov, VV
TI Initial stages in the decay of the amorphous phase in the bulk metallic
   glass Zr-Cu-Ti
SO PHYSICS OF THE SOLID STATE
AB Using methods of x-ray diffraction analysis, differential scanning calorimetry, dilatometry, and transmission electron microscopy, we have investigated the initial stages of decay of the amorphous phase in a bulk metallic glass based on zirconium. We found that crystallization of the bulk metallic glass proceeds in several stages, where in the first stage the bulk conversion effect is equal to more than 1.6%, or about 80% of the total bulk crystallization effect. We showed that the first stage of decay of the amorphous phase in the bulk metallic glass Zr29Ti11Cu60 leads to the formation of a nanocrystalline structure with a grain size of 1-5 nm. We have analyzed the change in the shape of the diffraction maximum during the formation of the very fine nanocrystalline structure. (C) 1999 American Institute of Physics. [S1063-7834(99)00107-0].
SN 1063-7834
PD JUL
PY 1999
VL 41
IS 7
BP 1027
EP 1031
DI 10.1134/1.1130930
UT WOS:000081819400001
ER

PT J
AU Qi, XT
   Zou, YT
   Wang, XB
   Chen, T
   Welch, DO
   Jiang, JZ
   Li, BS
AF Qi, Xintong
   Zou, Yongtao
   Wang, Xuebing
   Chen, Ting
   Welch, David O.
   Jiang, Jianzhong
   Li, Baosheng
TI Elastic Anomaly and Polyamorphic Transition in (La, Ce)-based Bulk
   Metallic Glass under Pressure
SO SCIENTIFIC REPORTS
AB Pressure-induced polyamorphism in Ce-based metallic glass has attracted significant interest in condensed matter physics. In this paper, we discover that in association with the polyamorphism of La32Ce32Al16Ni5Cu15 bulk metallic glass, the acoustic velocities, measured up to 12.3 GPa using ultrasonic interferometry, exhibit velocity minima at 1.8 GPa for P wave and 3.2 GPa for S wave. The low and high density amorphous states are distinguished by their distinct pressure derivatives of the bulk and shear moduli. The elasticity, permanent densification, and polyamorphic transition are interpreted by the topological rearrangement of solute-centered clusters in medium-range order (MRO) mediated by the 4f electron delocalization of Ce under pressure. The precisely measured acoustic wave travel times which were used to derive the velocities and densities provided unprecedented data to document the evolution of the bulk and shear elastic moduli associated with a polyamorphic transition in La32Ce32Al16Ni5Cu15 bulk metallic glass and can shed new light on the mechanisms of polyamorphism and structural evolution in metallic glasses under pressure.
SN 2045-2322
PD APR 7
PY 2017
VL 7
AR 724
DI 10.1038/s41598-017-00737-0
UT WOS:000398548100006
PM 28389659
ER

PT J
AU Duan, G
   Xu, DH
   Johnson, WL
AF Duan, G
   Xu, DH
   Johnson, WL
TI High copper content bulk glass formation in bimetallic Cu-Hf system
SO METALLURGICAL AND MATERIALS TRANSACTIONS A-PHYSICAL METALLURGY AND
   MATERIALS SCIENCE
AB We report in this article that strip-shaped amorphous samples with thicknesses from 0.5 to 2 nun were successfully synthesized for binary Cu-Hf alloys containing 60 to 68 at. pct Cu by the traditional copper mold casting method. The best glass former Cu66Hf34 with casting thickness up to 2 turn has an undercooled liquid region (DeltaT = T-x - T-g, where T-g is the glass transition temperature and T-x is the onset temperature of the first crystallization event) of 51 K, which is somewhat narrow compared with other neighboring 2 alloys in the same system. The bulk glassy Cu66Hf34 alloy exhibits Vicker's Hardness (H-v) similar to 779 kg/mm(2), Young's modulus similar to108 GPa, fracture strength similar to2.1 GPa, and an almost constant elastic elongation similar to1.8 pct upon compression. The discovery of Cu66Hf34 as a bulk glass confirms the existence of rather simple bulk-glass-forming metallic systems. Moreover, the present Cu-Hf alloys may be the highest copper content bulk metallic glasses reported to date, to the best of our knowledge.
RI Duan, Gang/B-3188-2009; Xu, Donghua/A-5263-2008
OI Xu, Donghua/0000-0001-5018-5603
SN 1073-5623
PD FEB
PY 2005
VL 36A
IS 2
BP 455
EP 458
DI 10.1007/s11661-005-0316-8
UT WOS:000226679300016
ER

PT J
AU Wang, ZR
   Qiang, JB
   Wang, YM
   Wang, Q
   Dong, DD
   Dong, C
AF Wang, Z. R.
   Qiang, J. B.
   Wang, Y. M.
   Wang, Q.
   Dong, D. D.
   Dong, C.
TI Composition design procedures of Ti-based bulk metallic glasses using
   the cluster-plus-glue-atom model
SO ACTA MATERIALIA
AB It is known that bulk metallic glasses are interpreted by simple composition formulas [cluster](glue atom)(1 or 3), with the valence electron number per unit formula (e/u) being universally 24. In the present work, this cluster-plus-glue-atom approach was explored in great details for the objective of establishing the standard procedures towards designing bulk metallic glasses, via example of Ti-Cu-based alloys. The cluster selection from devitrification phases and the e/u calculation methods were first clarified. Then, step-by-step procedures toward developing alloys with high glass forming abilities were proposed. The cluster formula [Ti-Cu6Ti8]Cu-3, with e/u approximate to 23.6, was taken as the basic composition formula. Upon properly alloying with Zr and Sn, large glass forming abilities up to 5 mm in diameter were experimentally reached at Ti40Zr10Cu56.94Sn3.06 via copper mold suction casting. Another alloy Ti45.71Zr11.43Cu39.29Sn3.37 showed a slightly inferior glass forming ability of 4 mm but contained the highest Ti(Zr) content of all the Ti-based BMGs, Ni-, Pd- and Be-free. The thus-obtained bulk metallic glasses generally exhibited high compressive strengths of about 1.8 GPa and nearly zero ductilities, except a Co-alloyed one, [Ti-Cu5.3Sn0.2Co0.2Ti6.2Zr1.8]Cu-3, whose compressive ductility exceeded 6%. (C) 2016 Acta Materialia Inc. Published by Elsevier Ltd. All rights reserved.
SN 1359-6454
EI 1873-2453
PD JUN 1
PY 2016
VL 111
BP 366
EP 376
DI 10.1016/j.actamat.2016.03.072
UT WOS:000375812100038
ER

PT J
AU Plummer, JD
   Goodall, R
   Figueroa, IA
   Todd, I
AF Plummer, J. D.
   Goodall, R.
   Figueroa, I. A.
   Todd, I.
TI A study of mechanical homogeneity in as-cast bulk metallic glass by
   nanoindentation
SO JOURNAL OF NON-CRYSTALLINE SOLIDS
AB A surface softening effect induced during copper-mould suction casting of bulk metallic glass is investigated as a function of rod diameter and glass fragility index. m, by nanoindentation. A reduction in hardness and reduced modulus at the rod surface is found to be favoured in small diameter castings and in fragile systems, respectively resulting from limited in-situ annealing and from a greater diversity of metastable atomic environments in the potential energy landscape of fragile glasses. Enhanced propensity for shear transformation zone nucleation in the low moduli surface is explained in terms of reduced atomic connectivity arising from a reduction in local co-ordination number and a lowering of the shear modulus. Finally, the structure and mechanical diversity that is possible in as-cast bulk metallic glass rods is explored through a relative quantification of shear modulus and plastic zone size across the whole as-cast state and in a single rod. These findings illustrate the sensitivity of bulk metallic glass to preparation, especially in respect of thermal history, potentially making replication of mechanical data between researchers problematic. (C) 2010 Elsevier B.V. All rights reserved.
RI Figueroa, Ignacio/J-5914-2012
OI Todd, Iain/0000-0003-0217-1658; Plummer, John/0000-0003-4824-8497
SN 0022-3093
PD FEB 1
PY 2011
VL 357
IS 3
BP 814
EP 819
DI 10.1016/j.jnoncrysol.2010.11.002
UT WOS:000288232500005
ER

PT J
AU Guo, J
   Bian, XF
   Li, XL
   Zhang, CZ
AF Guo, Jing
   Bian, Xiufang
   Li, Xuelian
   Zhang, Chunzhi
TI Evaluation of liquid fragility for glass-forming alloys based on mixing
   enthalpy and mismatch entropy
SO INTERMETALLICS
AB A calculable parameter delta, defined as the absolute multiplication of the chemical mixing enthalpy and mismatch entropy, has been proposed to evaluate the liquid dynamic fragility of glass-forming alloys. The alloy with a larger delta exhibits a stronger liquid characteristic in an alloy system with the same base element. The proposal of the parameter delta gives a much easier and more available method in evaluating liquid fragility without any experiments. (C) 2010 Elsevier Ltd. All rights reserved.
SN 0966-9795
PD MAY
PY 2010
VL 18
IS 5
BP 933
EP 937
DI 10.1016/j.intermet.2010.01.004
UT WOS:000277494400028
ER

PT J
AU Bhatt, J
   Dey, GK
   Murty, BS
AF Bhatt, Jatin
   Dey, G. K.
   Murty, B. S.
TI Thermodynamic and topological modeling and synthesis of
   Cu-Zr-Ti-Ni-based bulk metallic glasses by mechanical alloying
SO METALLURGICAL AND MATERIALS TRANSACTIONS A-PHYSICAL METALLURGY AND
   MATERIALS SCIENCE
CT Symposium on Materials Behavior - Far from Equilibrium
CY DEC 15-16, 2006
CL Bombay, INDIA
SP Bhabha Atom Res Ctr
AB In the present investigation, thermodynamic and topological concepts have been used together to understand the formation of bulk metallic glasses. The article attempts to develop a parameter that combines chemical mixing enthalpy (Delta H(chem)), mismatch entropy normalized by Boltzmann constant (Delta S(sigma)/k(B)), and configurational entropy (Delta S(config)/R), which can be effectively used to identify the best bulk glass forming compositions in multicomponent systems. The Delta H(chem) for multicomponent systems has been calculated by extending the Miedema's macroscopic model, and Delta S(sigma) /k(B) was obtained from Mansoori's approach. The iso-Delta H(chem), iso-Delta S(sigma) /k(B), and iso-Delta S(config)/R contours have been developed. The product of Delta H(chem) and Delta S(sigma) /k(B) in the Delta S(config)/R range of 0.9 to 1.0 can be correlated strongly to glass forming ability. This has been demonstrated in the case of the quaternary Cu-Zr-Ti-Ni bulk glass forming system to determine the bulk metallic glass forming compositions. Mechanical alloying is used to prepare the bulk amorphous powder at the compositions predicted by the model in order to validate the model.
RI Murty, BS/P-3354-2015
OI Murty, BS/0000-0002-4399-8531
SN 1073-5623
PD JUL
PY 2008
VL 39A
IS 7
BP 1543
EP 1551
DI 10.1007/s11661-007-9354-8
UT WOS:000256081500010
ER

PT J
AU Sun, Y
   Huang, YJ
   Fan, HB
   Liu, FY
   Shen, J
   Sun, JF
   Chen, JJJ
AF Sun, Yu
   Huang, Yongjiang
   Fan, Hongbo
   Liu, Fangyu
   Shen, Jun
   Sun, Jianfei
   Chen, John J. J.
TI Comparison of mechanical behaviors of several bulk metallic glasses for
   biomedical application
SO JOURNAL OF NON-CRYSTALLINE SOLIDS
AB Bulk metallic glass (BMG), Zr46Cu37.6Ag8.4Al8 (ZrCuAlAg), has been systematically evaluated in terms of microstructure, compressive properties, and wear resistance, aimed at exploring its use as a novel biomedical material. For comparison, the above-mentioned tests were also performed on Zr51.9Cu23.3Ni10.5Al14.3, Zr51Ti5Ni10Cu25Al9, and Ti40Zr25Ni12Cu3Be20 BMGs, pure Ti and Ti-6Al-4V alloy. ZrCuAlAg BMG exhibits higher strength, higher hardness, and better wear resistance than other materials. All the results imply that the Ag-bearing ZrCuAlAg BMG with excellent mechanocompatibility will open up a new path for biomedical applications. (C) 2014 Elsevier B.V. All rights reserved.
RI Huang, Yongjiang/D-4809-2009
SN 0022-3093
EI 1873-4812
PD DEC 15
PY 2014
VL 406
BP 144
EP 150
DI 10.1016/j.jnoncrysol.2014.09.021
UT WOS:000347592100022
ER

PT J
AU Cao, JD
   Laws, KJ
   Birbilis, N
   Ferry, M
AF Cao, J. D.
   Laws, K. J.
   Birbilis, N.
   Ferry, M.
TI Potentiodynamic polarisation study of bulk metallic glasses based on the
   Mg-Zn-Ca ternary system
SO CORROSION ENGINEERING SCIENCE AND TECHNOLOGY
AB Bulk metallic glasses (BMGs) based on the Mg-Zn-Ca ternary system are considered as potential bioresorbable metals for orthopaedic applications as they are comprised solely of biocompatible elements. In the present study, a series of six Ca rich and ten Mg rich BMGs were synthesised and their corrosion behaviours characterised using potentiodynamic polarisation. A complex interaction between the elements in this compositional window was observed, whereby a significant increase in dissolution rate was accompanied by increasing Ca content or decreasing Mg content. Although the addition of Zn had a positive contribution in reducing the rate of corrosion, its effect was not dominant. In addition, a form of passivation was observed in the Mg rich BMGs, although it appears that such passive corrosion behaviour is not directly related to a high Zn content.
RI BAI, JIE/D-7448-2016
OI Birbilis, Nick/0000-0002-7910-7470
SN 1478-422X
PD AUG
PY 2012
VL 47
IS 5
BP 329
EP 334
DI 10.1179/1743278212Y.0000000016
UT WOS:000306234100003
ER

PT J
AU Chen, BQ
   Li, Y
   Yi, M
   Li, R
   Pang, SJ
   Wang, H
   Zhang, T
AF Chen, Bingqing
   Li, Yan
   Yi, Min
   Li, Ran
   Pang, Shujie
   Wang, Hui
   Zhang, Tao
TI Optimization of mechanical properties of bulk metallic glasses by
   residual stress adjustment using laser surface melting
SO SCRIPTA MATERIALIA
AB Residual stress adjustment was achieved in (Zr0.55Al0.1Ni0.05Cu0.3)(99)Y-1 bulk metallic glasses (BMGs) using laser surface melting. The residual stress distribution in the treated alloy was revealed based on the experimental measurement and finite element simulation. The surface layer exhibited tensile residual stress and the nether part of the alloy indicated compressive residual stress for balance. The mechanical properties in bending of the treated BMG were profoundly affected by the laser-introduced residual stress, and the strength and plasticity were enhanced. (C) 2012 Acta Materialia Inc. Published by Elsevier Ltd. All rights reserved.
RI Zhang, Tao/O-4911-2014; Li, Ran/B-4618-2010; wang, hui/H-4352-2016;
   Chen, Bingqing/R-6300-2016; Pang, Shujie/D-8305-2016; Yi,
   Min/A-1474-2012
OI wang, hui/0000-0003-3120-1828; Yi, Min/0000-0001-7672-3107
SN 1359-6462
PD JUN
PY 2012
VL 66
IS 12
BP 1057
EP 1060
DI 10.1016/j.scriptamat.2012.02.046
UT WOS:000304641500022
ER

EF
```
